# ﻿On the nomenclatural status of type genera in Coleoptera (Insecta)

**DOI:** 10.3897/zookeys.1194.106440

**Published:** 2024-01-13

**Authors:** Patrice Bouchard, Yves Bousquet, Anthony E. Davies, Chenyang Cai

**Affiliations:** 1 Canadian National Collection of Insects, Arachnids and Nematodes, Agriculture and Agri-Food Canada, 960 Carling Avenue, Ottawa, Ontario, K1A 0C6, Canada Agriculture and Agri-Food Canada Ottawa Canada; 2 Gatineau, Quebec, Canada Unaffiliated Quebec Canada; 3 State Key Laboratory of Palaeobiology and Stratigraphy, Nanjing Institute of Geology and Palaeontology, and Center for Excellence in Life and Paleoenvironment, Chinese Academy of Sciences, Nanjing 210008, China Nanjing Institute of Geology and Palaeontology, and Center for Excellence in Life and Paleoenvironment, Chinese Academy of Sciences Nanjing China

**Keywords:** Beetles, catalogue, classification, family-group name, nomenclature, publication dates, type genus, type species

## Abstract

More than 4700 nominal family-group names (including names for fossils and ichnotaxa) are nomenclaturally available in the order Coleoptera. Since each family-group name is based on the concept of its type genus, we argue that the stability of names used for the classification of beetles depends on accurate nomenclatural data for each type genus. Following a review of taxonomic literature, with a focus on works that potentially contain type species designations, we provide a synthesis of nomenclatural data associated with the type genus of each nomenclaturally available family-group name in Coleoptera. For each type genus the author(s), year of publication, and page number are given as well as its current status (i.e., whether treated as valid or not) and current classification.

Information about the type species of each type genus and the type species fixation (i.e., fixed originally or subsequently, and if subsequently, by whom) is also given. The original spelling of the family-group name that is based on each type genus is included, with its author(s), year, and stem. We append a list of nomenclaturally available family-group names presented in a classification scheme. Because of the importance of the Principle of Priority in zoological nomenclature, we provide information on the date of publication of the references cited in this work, when known. Several nomenclatural issues emerged during the course of this work. We therefore appeal to the community of coleopterists to submit applications to the International Commission on Zoological Nomenclature (henceforth “Commission”) in order to permanently resolve some of the problems outlined here.

The following changes of authorship for type genera are implemented here (these changes do not affect the concept of each type genus): CHRYSOMELIDAE: *Fulcidax* Crotch, 1870 (previously credited to “Clavareau, 1913”); CICINDELIDAE: *Euprosopus* W.S. MacLeay, 1825 (previously credited to “Dejean, 1825”); COCCINELLIDAE: *Alesia* Reiche, 1848 (previously credited to “Mulsant, 1850”); CURCULIONIDAE: *Arachnopus* Boisduval, 1835 (previously credited to “Guérin-Méneville, 1838”); ELATERIDAE: *Thylacosternus* Gemminger, 1869 (previously credited to “Bonvouloir, 1871”); EUCNEMIDAE: *Arrhipis* Gemminger, 1869 (previously credited to “Bonvouloir, 1871”), *Mesogenus* Gemminger, 1869 (previously credited to “Bonvouloir, 1871”); LUCANIDAE: *Sinodendron* Hellwig, 1791 (previously credited to “Hellwig, 1792”); PASSALIDAE: *Neleides* Harold, 1868 (previously credited to “Kaup, 1869”), *Neleus* Harold, 1868 (previously credited to “Kaup, 1869”), *Pertinax* Harold, 1868 (previously credited to “Kaup, 1869”), *Petrejus* Harold, 1868 (previously credited to “Kaup, 1869”), *Undulifer* Harold, 1868 (previously credited to “Kaup, 1869”), *Vatinius* Harold, 1868 (previously credited to “Kaup, 1869”); PTINIDAE: *Mezium* Leach, 1819 (previously credited to “Curtis, 1828”); PYROCHROIDAE: *Agnathus* Germar, 1818 (previously credited to “Germar, 1825”); SCARABAEIDAE: *Eucranium* Dejean, 1833 (previously “Brullé, 1838”).

The following changes of type species were implemented following the discovery of older type species fixations (these changes do not pose a threat to nomenclatural stability): BOLBOCERATIDAE: *Bolbocerusbocchus* Erichson, 1841 for *Bolbelasmus* Boucomont, 1911 (previously *Bolbocerasgallicum* Mulsant, 1842); BUPRESTIDAE: *Stigmoderaguerinii* Hope, 1843 for *Neocuris* Saunders, 1868 (previously *Anthaxiafortnumi* Hope, 1846), *Stigmoderaperoni* Laporte & Gory, 1837 for *Curis* Laporte & Gory, 1837 (previously *Buprestiscaloptera* Boisduval, 1835); CARABIDAE: *Carabuselatus* Fabricius, 1801 for *Molops* Bonelli, 1810 (previously *Carabusterricola* Herbst, 1784 sensu Fabricius, 1792); CERAMBYCIDAE: *Prionuspalmatus* Fabricius, 1792 for *Macrotoma* Audinet-Serville, 1832 (previously *Prionusserripes* Fabricius, 1781); CHRYSOMELIDAE: *Donaciaequiseti* Fabricius, 1798 for *Haemonia* Dejean, 1821 (previously *Donaciazosterae* Fabricius, 1801), *Eumolpusruber* Latreille, 1807 for *Euryope* Dalman, 1824 (previously *Cryptocephalusrubrifrons* Fabricius, 1787), *Galerucaaffinis* Paykull, 1799 for *Psylliodes* Latreille, 1829 (previously *Chrysomelachrysocephala* Linnaeus, 1758); COCCINELLIDAE: *Dermestesrufus* Herbst, 1783 for *Coccidula* Kugelann, 1798 (previously *Chrysomelascutellata* Herbst, 1783); CRYPTOPHAGIDAE: *Ipscaricis* G.-A. Olivier, 1790 for *Telmatophilus* Heer, 1841 (previously *Cryptophagustyphae* Fallén, 1802), *Silphaevanescens* Marsham, 1802 for *Atomaria* Stephens, 1829 (previously *Dermestesnigripennis* Paykull, 1798); CURCULIONIDAE: *Bostrichuscinereus* Herbst, 1794 for *Crypturgus* Erichson, 1836 (previously *Bostrichuspusillus* Gyllenhal, 1813); DERMESTIDAE: *Dermestestrifasciatus* Fabricius, 1787 for *Attagenus* Latreille, 1802 (previously *Dermestespellio* Linnaeus, 1758); ELATERIDAE: *Elatersulcatus* Fabricius, 1777 for *Chalcolepidius* Eschscholtz, 1829 (previously *Chalcolepidiuszonatus* Eschscholtz, 1829); ENDOMYCHIDAE: *Endomychusrufitarsis* Chevrolat, 1835 for *Epipocus* Chevrolat, 1836 (previously *Endomychustibialis* Guérin-Méneville, 1834); EROTYLIDAE: *Ipshumeralis* Fabricius, 1787 for *Dacne* Latreille, 1797 (previously *Dermestesbipustulatus* Thunberg, 1781); EUCNEMIDAE: *Fornaxaustrocaledonicus* Perroud & Montrouzier, 1865 for *Mesogenus* Gemminger, 1869 (previously *Mesogenusmellyi* Bonvouloir, 1871); GLAPHYRIDAE: *Melolonthaserratulae* Fabricius, 1792 for *Glaphyrus* Latreille, 1802 (previously *Scarabaeusmaurus* Linnaeus, 1758); HISTERIDAE: *Histerstriatus* Forster, 1771 for *Onthophilus* Leach, 1817 (previously *Histersulcatus* Moll, 1784); LAMPYRIDAE: *Ototretafornicata* E. Olivier, 1900 for *Ototreta* E. Olivier, 1900 (previously *Ototretaweyersi* E. Olivier, 1900); LUCANIDAE: *Lucanuscancroides* Fabricius, 1787 for *Lissotes* Westwood, 1855 (previously *Lissotesmenalcas* Westwood, 1855); MELANDRYIDAE: *Nothusclavipes* G.-A. Olivier, 1812 for *Nothus* G.-A. Olivier, 1812 (previously *Nothuspraeustus* G.-A. Olivier, 1812); MELYRIDAE: *Lagriaater* Fabricius, 1787 for *Enicopus* Stephens, 1830 (previously *Dermesteshirtus* Linnaeus, 1767); NITIDULIDAE: *Sphaeridiumluteum* Fabricius, 1787 for *Cychramus* Kugelann, 1794 (previously *Strongylusquadripunctatus* Herbst, 1792); OEDEMERIDAE: *Helopslaevis* Fabricius, 1787 for *Ditylus* Fischer, 1817 (previously *Ditylushelopioides* Fischer, 1817 [sic]); PHALACRIDAE: *Sphaeridiumaeneum* Fabricius, 1792 for *Olibrus* Erichson, 1845 (previously *Sphaeridiumbicolor* Fabricius, 1792); RHIPICERIDAE: *Sandalusniger* Knoch, 1801 for *Sandalus* Knoch, 1801 (previously *Sandaluspetrophya* Knoch, 1801); SCARABAEIDAE: *Cetoniaclathrata* G.-A. Olivier, 1792 for *Inca* Lepeletier & Audinet-Serville, 1828 (previously *Cetoniaynca* Weber, 1801); *Gnathoceravitticollis* W. Kirby, 1825 for *Gnathocera* W. Kirby, 1825 (previously *Gnathoceraimmaculata* W. Kirby, 1825); *Melolonthavillosula* Illiger, 1803 for *Chasmatopterus* Dejean, 1821 (previously *Melolonthahirtula* Illiger, 1803); STAPHYLINIDAE: *Staphylinuspolitus* Linnaeus, 1758 for *Philonthus* Stephens, 1829 (previously *Staphylinussplendens* Fabricius, 1792); ZOPHERIDAE: *Hispamutica* Linnaeus, 1767 for *Orthocerus* Latreille, 1797 (previously *Tenebriohirticornis* DeGeer, 1775).

The discovery of type species fixations that are older than those currently accepted pose a threat to nomenclatural stability (an application to the Commission is necessary to address each problem): CANTHARIDAE: *Malthinus* Latreille, 1805, *Malthodes* Kiesenwetter, 1852; CARABIDAE: *Bradycellus* Erichson, 1837, *Chlaenius* Bonelli, 1810, *Harpalus* Latreille, 1802, *Lebia* Latreille, 1802, *Pheropsophus* Solier, 1834, *Trechus* Clairville, 1806; CERAMBYCIDAE: *Callichroma* Latreille, 1816, *Callidium* Fabricius, 1775, *Cerasphorus* Audinet-Serville, 1834, *Dorcadion* Dalman, 1817, *Leptura* Linnaeus, 1758, *Mesosa* Latreille, 1829, *Plectromerus* Haldeman, 1847; CHRYSOMELIDAE: *Amblycerus* Thunberg, 1815, *Chaetocnema* Stephens, 1831, *Chlamys* Knoch, 1801, *Monomacra* Chevrolat, 1836, *Phratora* Chevrolat, 1836, *Stylosomus* Suffrian, 1847; COLONIDAE: *Colon* Herbst, 1797; CURCULIONIDAE: *Cryphalus* Erichson, 1836, *Lepyrus* Germar, 1817; ELATERIDAE: *Adelocera* Latreille, 1829, *Beliophorus* Eschscholtz, 1829; ENDOMYCHIDAE: *Amphisternus* Germar, 1843, *Dapsa* Latreille, 1829; GLAPHYRIDAE: *Anthypna* Eschscholtz, 1818; HISTERIDAE: *Hololepta* Paykull, 1811, *Trypanaeus* Eschscholtz, 1829; LEIODIDAE: *Anisotoma* Panzer, 1796, *Camiarus* Sharp, 1878, *Choleva* Latreille, 1797; LYCIDAE: *Calopteron* Laporte, 1838, *Dictyoptera* Latreille, 1829; MELOIDAE: *Epicauta* Dejean, 1834; NITIDULIDAE: *Strongylus* Herbst, 1792; SCARABAEIDAE: *Anisoplia* Schönherr, 1817, *Anticheira* Eschscholtz, 1818, *Cyclocephala* Dejean, 1821, *Glycyphana* Burmeister, 1842, *Omaloplia* Schönherr, 1817, *Oniticellus* Dejean, 1821, *Parachilia* Burmeister, 1842, *Xylotrupes* Hope, 1837; STAPHYLINIDAE: *Batrisus* Aubé, 1833, *Phloeonomus* Heer, 1840, *Silpha* Linnaeus, 1758; TENEBRIONIDAE: *Bolitophagus* Illiger, 1798, *Mycetochara* Guérin-Méneville, 1827.

Type species are fixed for the following nominal genera: ANTHRIBIDAE: *Decataphanesgracilis* Labram & Imhoff, 1840 for *Decataphanes* Labram & Imhoff, 1840; CARABIDAE: *Feroniaerratica* Dejean, 1828 for *Loxandrus* J.L. LeConte, 1853; CERAMBYCIDAE: *Tmesisternusoblongus* Boisduval, 1835 for *Icthyosoma* Boisduval, 1835; CHRYSOMELIDAE: *Brachydactylaannulipes* Pic, 1913 for *Pseudocrioceris* Pic, 1916, *Cassidaviridis* Linnaeus, 1758 for *Evaspistes* Gistel, 1856, *Ocnosceliscyanoptera* Erichson, 1847 for *Ocnoscelis* Erichson, 1847, *Promecothecapetelii* Guérin-Méneville, 1840 for *Promecotheca* Guérin- Méneville, 1840; CLERIDAE: *Attelabusmollis* Linnaeus, 1758 for *Dendroplanetes* Gistel, 1856; CORYLOPHIDAE: *Corylophusmarginicollis* J.L. LeConte, 1852 for *Corylophodes* A. Matthews, 1885; CURCULIONIDAE: *Hoplorhinusmelanocephalus* Chevrolat, 1878 for *Hoplorhinus* Chevrolat, 1878; *Sonnetiusbinarius* Casey, 1922 for *Sonnetius* Casey, 1922; ELATERIDAE: *Pyrophorusmelanoxanthus* Candèze, 1865 for *Alampes* Champion, 1896; PHYCOSECIDAE: *Phycosecislitoralis* Pascoe, 1875 for *Phycosecis* Pascoe, 1875; PTILODACTYLIDAE: *Aploglossasallei* Guérin-Méneville, 1849 for *Aploglossa* Guérin-Méneville, 1849, *Coloboderaovata* Klug, 1837 for *Colobodera* Klug, 1837; PTINIDAE: *Dryophilusanobioides* Chevrolat, 1832 for *Dryobia* Gistel, 1856; SCARABAEIDAE: *Achloahelvola* Erichson, 1840 for *Achloa* Erichson, 1840, *Camentaobesa* Burmeister, 1855 for *Camenta* Erichson, 1847, *Pinotustalaus* Erichson, 1847 for *Pinotus* Erichson, 1847, *Psilonychusecklonii* Burmeister, 1855 for *Psilonychus* Burmeister, 1855.

New replacement name: CERAMBYCIDAE: *Basorus* Bouchard & Bousquet, **nom. nov.** for *Sobarus* Harold, 1879.

New status: CARABIDAE: KRYZHANOVSKIANINI Deuve, 2020, **stat. nov.** is given the rank of tribe instead of subfamily since our classification uses the rank of subfamily for PAUSSINAE rather than family rank; CERAMBYCIDAE: *Amymoma* Pascoe, 1866, **stat. nov.** is used as valid over *Neoamymoma* Marinoni, 1977, *Holopterus* Blanchard, 1851, **stat. nov.** is used as valid over *Proholopterus* Monné, 2012; CURCULIONIDAE: *Phytophilus* Schönherr, 1835, **stat. nov.** is used as valid over the unnecessary new replacement name *Synophthalmus* Lacordaire, 1863; EUCNEMIDAE: *Nematodinus* Lea, 1919, **stat. nov.** is used as valid instead of *Arrhipis* Gemminger, 1869, which is a junior homonym.

Details regarding additional nomenclatural issues that still need to be resolved are included in the entry for each of these type genera: BOSTRICHIDAE: *Lyctus* Fabricius, 1792; BRENTIDAE: *Trachelizus* Dejean, 1834; BUPRESTIDAE: *Pristiptera* Dejean, 1833; CANTHARIDAE: *Chauliognathus* Hentz, 1830, *Telephorus* Schäffer, 1766; CARABIDAE: *Calathus* Bonelli, 1810, *Cosnania* Dejean, 1821, *Dicrochile* Guérin-Méneville, 1847, *Epactius* D.H. Schneider, 1791, *Merismoderus* Westwood, 1847, *Polyhirma* Chaudoir, 1850, *Solenogenys* Westwood, 1860, *Zabrus* Clairville, 1806; CERAMBYCIDAE: *Ancita* J. Thomson, 1864, *Compsocerus* Audinet-Serville, 1834, *Dorcadodium* Gistel, 1856, *Glenea* Newman, 1842; *Hesperophanes* Dejean, 1835, *Neoclytus* J. Thomson, 1860, *Phymasterna* Laporte, 1840, *Tetrops* Stephens, 1829, *Zygocera* Erichson, 1842; CHRYSOMELIDAE: *Acanthoscelides* Schilsky, 1905, *Corynodes* Hope, 1841, *Edusella* Chapuis, 1874; *Hemisphaerota* Chevrolat, 1836; *Physonota* Boheman, 1854, *Porphyraspis* Hope, 1841; CLERIDAE: *Dermestoides* Schäffer, 1777; COCCINELLIDAE: *Hippodamia* Chevrolat, 1836, *Myzia* Mulsant, 1846, *Platynaspis* L. Redtenbacher, 1843; CURCULIONIDAE: *Coeliodes* Schönherr, 1837, *Cryptoderma* Ritsema, 1885, *Deporaus* Leach, 1819, *Epistrophus* Kirsch, 1869, *Geonemus* Schönherr, 1833, *Hylastes* Erichson, 1836; DYTISCIDAE: *Deronectes* Sharp, 1882, *Platynectes* Régimbart, 1879; EUCNEMIDAE: *Dirhagus* Latreille, 1834; HYBOSORIDAE: *Ceratocanthus* A. White, 1842; HYDROPHILIDAE: *Cyclonotum* Erichson, 1837; LAMPYRIDAE: *Luciola* Laporte, 1833; LEIODIDAE: *Ptomaphagus* Hellwig, 1795; LUCANIDAE: *Leptinopterus* Hope, 1838; LYCIDAE: *Cladophorus* Guérin-Méneville, 1830, *Mimolibnetis* Kazantsev, 2000; MELOIDAE: *Mylabris* Fabricius, 1775; NITIDULIDAE: *Meligethes* Stephens, 1829; PTILODACTYLIDAE: *Daemon* Laporte, 1838; SCARABAEIDAE: *Allidiostoma* Arrow, 1940, *Heterochelus* Burmeister, 1844, *Liatongus* Reitter, 1892, *Lomaptera* Gory & Percheron, 1833, *Megaceras* Hope, 1837, *Stenotarsia* Burmeister, 1842; STAPHYLINIDAE: *Actocharis* Fauvel, 1871, *Aleochara* Gravenhorst, 1802; STENOTRACHELIDAE: *Stenotrachelus* Berthold, 1827; TENEBRIONIDAE: *Cryptochile* Latreille, 1828, *Heliopates* Dejean, 1834, *Helops* Fabricius, 1775.

First Reviser actions deciding the correct original spelling: CARABIDAE: *Aristochroodes* Marcilhac, 1993 (not *Aritochroodes*); CERAMBYCIDAE: *Dorcadodium* Gistel, 1856 (not *Dorcadodion*), EVODININI Zamoroka, 2022 (not EVODINIINI); CHRYSOMELIDAE: *Caryopemon* Jekel, 1855 (not *Carpopemon*), *Decarthrocera* Laboissière, 1937 (not *Decarthrocerina*); CICINDELIDAE: *Odontocheila* Laporte, 1834 (not *Odontacheila*); CLERIDAE: CORMODINA Bartlett, 2021 (not CORMODIINA), *Orthopleura* Spinola, 1845 (not *Orthoplevra*, not *Orthopleuva*); CURCULIONIDAE: *Arachnobas* Boisduval, 1835 (not *Arachnopus*), *Palaeocryptorhynchus* Poinar, 2009 (not *Palaeocryptorhynus*); DYTISCIDAE: *Ambarticus* Yang et al., 2019 and AMBARTICINI Yang et al., 2019 (not *Ambraticus*, not AMBRATICINI); LAMPYRIDAE: *Megalophthalmus* G.R. Gray, 1831 (not *Megolophthalmus*, not *Megalopthalmus*); SCARABAEIDAE: *Mentophilus* Laporte, 1840 (not *Mintophilus*, not *Minthophilus*), *Pseudadoretusdilutellus* Semenov, 1889 (not *P.ditutellus*).

While the correct identification of the type species is assumed, in some cases evidence suggests that species were misidentified when they were fixed as the type of a particular nominal genus. Following the requirements of Article 70.3.2 of the International Code of Zoological Nomenclature we hereby fix the following type species (which in each case is the taxonomic species actually involved in the misidentification): ATTELABIDAE: *Rhynchitescavifrons* Gyllenhal, 1833 for *Lasiorhynchites* Jekel, 1860; BOSTRICHIDAE: *Ligniperdaterebrans* Pallas, 1772 for *Apate* Fabricius, 1775; BRENTIDAE: *Ceocephalusappendiculatus* Boheman, 1833 for *Uroptera* Berthold, 1827; BUPRESTIDAE: *Buprestisundecimmaculata* Herbst, 1784 for *Ptosima* Dejean, 1833; CARABIDAE: *Amaralunicollis* Schiødte, 1837 for *Amara* Bonelli, 1810, *Buprestisconnexus* Geoffroy, 1785 for *Polistichus* Bonelli, 1810, *Carabusatrorufus* Strøm, 1768 for *Patrobus* Dejean, 1821, *Carabusgigas* Creutzer, 1799 for *Procerus* Dejean, 1821, *Carabusteutonus* Schrank, 1781 for *Stenolophus* Dejean, 1821, *Carenumbonellii* Westwood, 1842 for *Carenum* Bonelli, 1813, *Scaritespicipes* G.-A. Olivier, 1795 for *Acinopus* Dejean, 1821, *Trigonotomaindica* Brullé, 1834 for *Trigonotoma* Dejean, 1828; CERAMBYCIDAE: *Cerambyxlusitanus* Linnaeus, 1767 for *Exocentrus* Dejean, 1835, *Clytussupernotatus* Say, 1824 for *Psenocerus* J.L. LeConte, 1852; CICINDELIDAE: *Ctenostomajekelii* Chevrolat, 1858 for *Ctenostoma* Klug, 1821; CURCULIONIDAE: *Cnemogonuslecontei* Dietz, 1896 for *Cnemogonus* J.L. LeConte, 1876; *Phloeophagusturbatus* Schönherr, 1845 for *Phloeophagus* Schönherr, 1838; GEOTRUPIDAE: *Lucanusapterus* Laxmann, 1770 for *Lethrus* Scopoli, 1777; HISTERIDAE: *Histerrugiceps* Duftschmid, 1805 for *Hypocaccus* C.G. Thomson, 1867; HYBOSORIDAE: *Hybosorusilligeri* Reiche, 1853 for *Hybosorus* W.S. MacLeay, 1819; HYDROPHILIDAE: *Hydrophilusmelanocephalus* G.-A. Olivier, 1793 for *Enochrus* C.G. Thomson, 1859; MYCETAEIDAE: *Dermestessubterraneus* Fabricius, 1801 for *Mycetaea* Stephens, 1829; SCARABAEIDAE: *Aulaciumcarinatum* Reiche, 1841 for *Mentophilus* Laporte, 1840, *Phanaeusvindex* W.S. MacLeay, 1819 for *Phanaeus* W.S. MacLeay, 1819, *Ptinusgermanus* Linnaeus, 1767 for *Rhyssemus* Mulsant, 1842, *Scarabaeuslatipes* Guérin-Méneville, 1838 for *Cheiroplatys* Hope, 1837; STAPHYLINIDAE: *Scydmaenustarsatus* P.W.J. Müller & Kunze, 1822 for *Scydmaenus* Latreille, 1802.

New synonyms: CERAMBYCIDAE: CARILIINI Zamoroka, 2022, **syn. nov.** of ACMAEOPINI Della Beffa, 1915, DOLOCERINI Özdikmen, 2016, **syn. nov.** of BRACHYPTEROMINI Sama, 2008, PELOSSINI Tavakilian, 2013, **syn. nov.** of LYGRINI Sama, 2008, PROHOLOPTERINI Monné, 2012, **syn. nov.** of HOLOPTERINI Lacordaire, 1868.

## ﻿Introduction

Type genera are arguably the most important genera in any taxonomic group since authors have recognized the need to propose new suprageneric taxa to accommodate them in then-existing classifications. Information about the fixation of type species for genus-group names is often absent or incomplete in major works. For example, the influential and broadly used book series *Catalogue of Palaearctic Coleoptera* lists the type species of all genus-group names found in the Palaearctic Region but does not include information about the type species fixation (i.e., fixed originally or subsequently, and if subsequently then by whom; ICZN 1999a, Article 67.4). The lack of information about the type species fixation in these and other works makes it impossible for subsequent authors to clearly determine if a type species designation found in the literature is the oldest, and therefore the valid fixation of the type species (ICZN 1999a, Article 70.2), or not. We believe that the inclusion of type genera (and, if possible, of their type species) in phylogenetic studies is of utmost importance since decisions regarding classifications are based on the clades that emerge from such analyses. Using incorrect terminal taxa in phylogenetic analyses has the potential to lead to undesirable decisions about classifications.

Following a review of scientific literature with a focus on works that potentially include type species designations, as well as trying to determine the date of publication of major works as accurately as possible, we provide a synthesis of nomenclatural information on Coleoptera type genera.

## ﻿Methods

### ﻿Nomenclatural data

Type genera are listed alphabetically. For each nominal type genus, the name, author(s), year of publication, page number, current status and placement, type species, and typification, as well as information regarding the family-group name based on it, are given.

Type species are important nomenclaturally since they provide the objective standard of reference for the application of the genus-group name they represent (ICZN 1999a, Article 61.1). Species that can be fixed as type are nominal species originally included in the newly established genus or subgenus that are cited by an available name (excluded are nominal species that were doubtfully or conditionally included in the new taxon) (see ICZN 1999a, Article 67.2). Type species can either be fixed in the original publication (ICZN 1999a, Article 68), in a subsequent publication (ICZN 1999a, Article 69) or, when nomenclatural problems are resolved by the International Commission on Zoological Nomenclature (henceforth “Commission”), by a decision under its plenary power (ICZN 1999a, Article 81). In cases where several nominal species were originally included in a new genus or subgenus established before 1931, but none were fixed as the type species, the first author who subsequently designated one of the originally included nominal species as the type species fixed the type species and no later designation is valid (ICZN 1999a, Article 69.1). When an earlier type species fixation is discovered, it is to be accepted or a Case submitted to the Commission for a ruling if the overlooked fixation is considered to cause instability or confusion (ICZN 1999a, Article 70.2). When a type species is fixed, it is generally assumed that the identification of that species is correct (ICZN 1999a, Article 70.1); however, where evidence suggests that the type species was misidentified at the time it was fixed, we have selected the taxonomic species actually involved as type species accompanied with a reference to Article 70.3.2 (ICZN 1999a). We follow previous authors (e.g., Alonso-Zarazaga and Lyal 2009; Bousquet et al. 2015, 2018; Bouchard et al. 2021) and consider type-species designations in R. Lucas (1920) as potentially valid when a single species is listed under a particular genus-group name. Based on Recommendation 67B (ICZN 1999a), the name of each type species is cited by its original binomen and, when this is currently considered invalid, its valid synonym is given in parentheses (preceded by “=”). Also note that the type species (and fixation) for a new replacement name is the same as for the previously established name it replaces (ICZN 1999a, Article 67.8). Fossil taxa are indicated by the symbol “†”.

While the correct identification of a type genus is assumed when an author establishes a new nominal family-group taxon (ICZN 1999a, Articles 65.1), evidence suggests that in some cases the type genus was misidentified (i.e., interpreted in a sense other than that defined by its type species). The cases where these family-group names are not currently used as valid (summarized in Table 1) are likely to cause instability and confusion and therefore need to be referred to the Commission for a ruling (ICZN 1999a, Articles 65.2.1). For this reason, names in Table 1 are not included elsewhere in this work (for additional information see appendix 2 and appendix 3 in Bouchard et al. 2011, Bouchard and Bousquet 2020a). We follow Evenhuis (2023: 3) in using the Nomenclator Zoologicus (e.g., Neave 1939, 1940) as a potential source of First Reviser actions when more than one spelling was used to establish a new genus-group name.

**Table 1.** List of available family-group names with misidentified type genera. These cases are to be referred to the Commission for a ruling (ICZN 1999a, Article 65.2.1).

Family-group name	Misidentified type genus	Actual genus	Family
AGELASINI Leng, 1920	*Agelasa* Motschulsky sensu G.H. Horn (1893a: 131)	*Sermylassa* Reitter, 1912	CHRYSOMELIDAE
ANTHOBIINI Portevin, 1929	*Anthobium* Leach sensu Erichson (1840b: 890)	*Eusphalerum* Kraatz, 1857	STAPHYLINIDAE
CANTHARIDIAE Latreille, 1802	*Cantharis* Linnaeus sensu Latreille (1802b: 185)	*Lytta* Fabricius, 1775	MELOIDAE
CÉOCÉPHALIDES Lacordaire, 1865	*Ceocephalus* Schönherr sensu Lacordaire (1865: 451)	*Orphanobrentus* Damoiseau, 1962 or *Pseudoceocephalus* Kleine, 1920	BRENTIDAE
CNÉMACANTHIDES Lacordaire, 1854	*Cnemacanthus* G.R. Gray sensu Brullé (1834: 375)	*Cnemalobus* Guérin-Méneville, 1838	CARABIDAE
COPTORRHYNCHINA Voss, 1940	*Coptorhynchus* Guérin-Méneville sensu Faust (1898b: 236)	*Resites* Alonso-Zarazaga and Lyal, 1999	CURCULIONIDAE
DICRONYCHIDAE Schwarz, 1897	*Dicronychus* Brullé sensu Laporte (1840a: 251)	*Eudicronychus* Méquignon, 1931	ELATERIDAE
EUBRACHINI Jacoby, 1908	*Eubrachis* Dejean sensu Baly (1878a: 248)	*Pseudocolaspis* Laporte, 1833	CHRYSOMELIDAE
EUPSALINI Muizon, 1960	*Eupsalis* Lacordaire sensu Kleine (1927: 38)	*Orfilaia* Haedo Rossi, 1955	BRENTIDAE
GRANIGERINI Bedel, 1900	*Graniger* Motschulsky sensu Chaudoir (1876c: 118)	*Cymbionotum* Baudi, 1864	CARABIDAE
IPS Latreille, 1802	*Ips* DeGeer sensu Latreille (1802b: 132)	*Cryptophagus* Herbst, 1792	CRYPTOPHAGIDAE
ISCHNOPODINI Hatch, 1957	*Ischnopoda* Stephens sensu Westwood (1838c: 19)	*Acrotona* C. G. Thomson, 1859	STAPHYLINIDAE
LYTÉRIIDES Lacordaire, 1865	*Lyterius* Schönherr sensu Lacordaire (1865: 249, 250)	*Tonesia* Casey, 1922	CURCULIONIDAE
MYCHOCERINAE Sharp, 1895	*Mychocerus* Erichson sensu J.L. LeConte (1869b: 255)	*Mychocerinus* Ślipiński 1990	CERYLONIDAE
NÉMOCÉPHALIDES Lacordaire, 1865	*Nemocephalus* Guérin-Méneville sensu Lacordaire (1865: 462)	*Neacratus* Alonso-Zarazaga et al. in Alonso-Zarazaga and Lyal, 1999	BRENTIDAE
NOSODERMINI Casey, 1907	*Nosoderma* Guérin-Méneville sensu Solier (1841b: 31)	*Verodes* Casey, 1907	ZOPHERIDAE
NOTARINI Zumpt, 1929	*Notaris* Germar sensu Tournier (1874: 92)	*Tournotaris* Alonso-Zarazaga and Lyal, 1999	CURCULIONIDAE
NOTOXII Sturm, 1826	*Notoxus* Geoffroy sensu Fabricius (1775: 158)	*Opilo* Latreille, 1802	CLERIDAE
OXYOPISTHINAE Kolbe, 1899	*Oxyopisthen* J. Thomson sensu Lacordaire (1865: 279)	*Korotyaevius* Alonso-Zarazaga and Lyal, 1999	CURCULIONIDAE
PHLOEOBIINAE Fowler, 1888	*Phloeobium* Dejean sensu Erichson (1840b: 907)	*Metopsia* Wollaston, 1854	STAPHYLINIDAE
PLASTOCERINI J.L. LeConte, 1861	*Plastocerus* Schaum sensu J.L. LeConte (1853f: 502)	*Octinodes* Candèze, 1863	ELATERIDAE
PSECTROPINI Kaszab, 1940	*Psectropus* Gemminger sensu Kaszab (1940: 140)	*Schyzoschelus* Koch, 1954	TENEBRIONIDAE
PTEROTARSINI Cobos, 1965	*Pterotarsus* Guérin-Méneville sensu Guérin-Méneville (1838: 68)	*Galbites* Fleutiaux, 1918	EUCNEMIDAE
RHINOMACERIDES Schönherr, 1823	*Rhinomacer* Geoffroy sensu G.-A. Olivier (1807: 457)	*Cimberis* Gozis, 1881	NEMONYCHIDAE
RHYNCHAENIDES Latreille, 1828	*Rhynchaenus* Clairville sensu Latreille (1828b: 600)	*Notaris* Germar, 1817	CURCULIONIDAE
SCOLYTINI Jakobson, 1911	*Scolytus* Geoffroy sensu Jakobson (1911: 652)	*Onthophilus* Leach, 1817	HISTERIDAE
SPERMOPHAGIDAE Crotch, 1873	*Spermophagus* Schönherr sensu G.H. Horn (1873b: 311)	*Amblycerus* Thunberg, 1815	CHRYSOMELIDAE
STENOCORIDAE Hope, 1834	*Stenocorus* Geoffroy sensu Hope (1834a: 107)	*Phoracantha* Newman, 1840	CERAMBYCIDAE
SUNIINA Sharp, 1886	*Sunius* Stephens sensu Erichson (1839a: 523)	*Astenus* Dejean, 1833	STAPHYLINIDAE
TOMICIDAE Shuckard, 1839	*Tomicus* Latreille sensu Shuckard (1839: 64)	*Ips* DeGeer, 1775	CURCULIONIDAE

### ﻿Classification

The classification presented in Appendix 1 (down to the rank of subfamily) is based on the studies of Cai et al. (2022) unless stated otherwise. The synoptic classification in Bouchard et al. (2011) was used as the starting point for the classification of beetles below the rank of subfamily. While we tried to use the most up-to-date information to generate the classification of all beetles presented in Appendix 1, we realize that errors may remain and that there are often different points of view in the community regarding how taxa should be classified. We nevertheless hope that the information thus presented will adequately assist the retrieval of relevant information for the readers. We included type genera of all available family-group names known to us, up to the date of publication of this article; however, we also included the type genus of a small number of family-group names that were published after 2011 in an electronic-only work that failed to meet the criteria of availability as required in amendments of the International Code of Zoological Nomenclature (ICZN 2012f; henceforth “Code”). In these cases, the family-group name and its associated genus are included to highlight the generally unknown nomenclatural problems. The stem we include under each family-group name and use in Appendix 1 is the stem that we recommend to be used in order to provide greatest stability of the Coleoptera classification (see Bouchard et al. 2011, Bouchard and Bousquet 2020a). The spelling of an incorrectly formed stem is sometimes maintained following recommendations in Article 29 (ICZN 1999a).

It should be noted that a disproportionally large number of new family-group names have been proposed in recent years in the superfamily CURCULIONOIDEA Latreille, 1802 by a small number of individuals (e.g., Legalov, Poinar). While these taxa are included here for completeness, some were evidently proposed without adequate comparative morphological analysis (e.g., Oberprieler and Zimmerman 2020: 556–558; Wanat 2021: 54) and their validity remains to be tested in a phylogenetic framework. The recent proliferation of new family-group names, combined with the challenging diversity of the group, has unfortunately resulted in an unsatisfactory weevil classification that still requires much attention before a stable and globally accepted system can be agreed upon.

### ﻿Excluded family-group names and their associated type genera

COLEOCATINIIDAE Ponomarenko & Prokin (2016): as pointed out by Bouchard and Bousquet (2020a: 70), this family-group name is unavailable since it was not based on an available genus-group name (ICZN 1999a, Article 11.7.1.1).

DONACOCIADAEGistel (1856): this name, which was listed as available by Bouchard et al. (2011: 509), is based on *Donacocia* used by Gistel (1856: 377), which we consider to be an incorrect subsequent spelling of *Donacia* Fabricius, 1775. Consequently, the family-group name must be corrected (ICZN 1999a, Article 35.4.1) to DONACIADAE (see entry below under the type genus *Donacia* Fabricius, 1775).

PHILHYDRIDA J.E. LeConte (1849): the informal name Philhydrida, which was not based on an available type genus, was first coined by W.S. MacLeay (1825: 6). J.E. LeConte (1849: 27) was the first to include the genus-group name *Philydrus* Solier, 1834 under the name Philhydrida; however, we believe that he did not propose a new family-group name but rather used W.S. MacLeay’s informal name. Therefore, we do not treat Philhydrida, as used by J.E. LeConte (1849), as an available family- group name as previously done by Bouchard et al. (2011: 155).

RHYTIDORHINIDESWollaston (1865): this name was listed as available by Bouchard et al. (2011: 603); however, it is based on *Rhytidorhinus* Agassiz, 1846, an unjustified emendation of *Rhythirrinus* Schönherr, 1823, and as such must be corrected (ICZN 1999a, Article 35.4.1) to RHYTHIRRINIDAE (see entry below under the type genus *Rhythirrinus* Schönherr, 1823). *Rhytidorhinus* Wollaston, 1864, incorrectly given as the type genus of RHYTIDORHINIDES Wollaston by Bouchard et al. (2011: 603), is an unavailable genus-group name not homonymous with *Rhytidorhinus* Agassiz, 1846.

### ﻿Authorship

Initials for the following authors were used in the text to avoid confusion: A. Abdullah, M. Abdullah, D.M. Anderson, R.S. Anderson, W.H. Anderson, M. Bai, X.L. Bai, F. Balfour-Browne, J. Balfour- Browne, F. Bates, H.W. Bates, J.R. Bell, K.L. Bell, R.T. Bell, T. Bell, A. Bezděk, J. Bezděk, W.F.H. Blandford, W.T. Blandford, A.E. Brown, F.M. Brown, C.H. Chen, S.H. Chen, Z.-Y. Chen, A. Costa, C. Costa, A.G. Desmarest, E. Desmarest, E.S. Dillon, L.S. Dillon, H.B. Douglas, J.W. Douglas, F. Dupuis, P. Dupuis, M.C. Ferreira, V.S. Ferreira, H. Franz, N.M. Franz, C. Girard, M. Girard, G.R. Gray, J.E. Gray, G.H. Horn, W. Horn, D. Huang, F. Huang, H. Huang, A. Janssens, E. Janssens, A.J. Johnson, C. Johnson, P.J. Johnson, W. Kirby, W.F. Kirby, C. Koch, C.L. Koch, J.D.W. Koch, J.E. LeConte, J.L. LeConte, J. Li, S. Li, X.-Y. Li, Y.-D. Li, L.Y. Liu, M. Liu, P.H. Lucas, R. Lucas, W.J. MacLeay, W.S. MacLeay, G.A.K. Marshall, T.A. Marshall, A. Matthews, A.[H.] Matthews, E.G. Matthews, G.S. Medvedev, L.N. Medvedev, S.I .Medvedev, B. Meyer, H. Meyer, J. Meyer, K.B. Miller, L. Miller, N.C.E. Miller, A. Milne-Edwards, H. Milne-Edwards, B.P. Moore, F. Moore, C. Müller, J. Müller, O.F. Müller, P.W.J. Müller, E. Olivier, G.-A. Olivier, C. d’Orbigny, H. d’Orbigny, M.F. Pacheco, T.L. Pacheco, J. Parker, J.B. Parker, H. Pang, X.-F. Pang, G. Pelletier, J. Pelletier, F. Philippi, R.A. Philippi, J. Redtenbacher, L. Redtenbacher, W. Redtenbacher, H.G.L. Reichenbach, L. Reichenbach, D. Ren, G.-D. Ren, L. Ren, P.O. Richter, R. Richter, C.V. Riley, N.D. Riley, C.R. Sahlberg, J.R. Sahlberg, R.F. Sahlberg, C. Schaufuss, L.W. Schaufuss, A. Schmidt, F.J. Schmidt, J. Schmidt, W.L.E. Schmidt, D.H. Schneider, J.G. Schneider, E.A. Schwarz, O. Schwarz, H. Scott, J. Scott, C. Shin, S. Shin, A.B.T. Smith, A.D. Smith, F. Smith, A. Solari, F. Solari, G. Strohmeyer, H. Strohmeyer, D.B. Thomas, M.C. Thomas, C.G. Thomson, J. Thomson, J.A. Thomson, B.D. Valentine, J.M. Valentine, A. Villa, G.B. Villa, B. Wang, W.I. Wang, C.O. Waterhouse, G.R. Waterhouse, J.B. Waterhouse, G. Wheeler, Q.D. Wheeler, W.M. Wheeler, A. White, B.E. White, R.E. White, A. Winkler, J.R. Winkler, Q. Yang, X.-K. Yang, F.N. Young, R.M. Young, H. Zhang, P. Zhang, W. Zhang, A. Zimmermann, C. Zimmermann. We follow Bouchard et al. (2021: 8) and use the surname “Fischer” for G. Fischer’s works up to the end of 1832 and “Fischer von Waldheim” starting from 1833, which is the year he became a member of the Russian nobility. Similarly, we use “Semenov” for works published by this author up to the end of 1906 and “Semenov-Tjan-Shansky” for works published after 1906 in order to account for the honorary title “Tjan-Schansky” added by the Emperor of All Russia Nikolay II to the surname of A.P. Semenov’s father (and all his family members) in 1906 (e.g., see Chigray et al. 2022: 4).

### ﻿Publication dates

Because of the importance of the Principle of Priority in zoological nomenclature (ICZN 1999a, Article 23), we have tried to establish the date of publication of works listed herein as accurately as possible. In the References section, publication dates are listed after each reference in square brackets “ []” and preceded by DP (i.e., Date of Publication). These dates are either specific dates of publication (e.g., extracted from the works themselves or the journal wrappers, title pages, footers) or are the earliest known publication dates (e.g., extracted from recording journals or accounts of society meetings). In the latter case, the date is preceded by the word “by.”

For the Annali del Museo Civico di Storia Naturale di Genova, we originally compiled the dates on which the signatures were printed; however, following Poggi (2010: 48–49, 79–228), the publication date is that printed on the reprint, which is the date on which the compiled signatures were sent to the authors for distribution [although Poggi occasionally gave a reprint date earlier than the date of the last signature in the paper, in which case we cite the signature date]. The volume numbers listed in the articles published in this journal are for the entire sequence of volumes, ignoring the “Series” numbers.

Although we found it difficult to determine the date of publication for many Russian journals and books, we have often cited the date on which the article was “approved to print”, that is the date posted by the censor (“podpisano k pechati”), usually inside the front or back wrapper, or on the endleaf. With few exceptions, this date indicates the earliest possible date of publication, and the works were apparently usually published shortly thereafter (see Technical Translations 15 (12): Doklady Biological Sciences 154–156: B-5). All Julian dates for Russian publications have been converted to Gregorian dates.

Sources of information from recording journals (for further historical information see Bousquet 2016: 24–36), specific works and date stamps from various institutions are mentioned below.

### ﻿Journals recording notices and dates of publication

Allg Anz Deutsch: Allgemeiner Anzeiger der Deutschen (Gotha, Germany).

Allgem Bibl: Allgemeine Bibliographie. Monatliches Verzeichniss der wichtigern neuen Erscheinungen der Deutschen und Ausländischen Literatur (Leipzig, Germany).

Allg Bibl Deutsch: Allgemeine Bibliographie für Deutschland (Leipzig, Germany).

Allg Lit Ztg: Allgemeine Literatur-Zeitung (Jena/Halle, Germany).

Amer Nat: The American Naturalist (Philadelphia, U.S.A.).

Ann Soc Ent Fr: Annales de la Société Entomologique de France (Paris, France).

Arch Naturg: Archiv für Naturgeschichte (Berlin, Germany).

Athenaeum: The Athenaeum. Journal of Literature, Science, and the Fine Arts (London, U.K.).

Berl Ent Zeit: Berliner Entomologische Zeitschrift (Berlin, Germany).

Bibl Fr: Bibliographie de la France (Paris, France).

Bulletin du Nord: Bulletin du Nord, journal scientifique et littéraire, contenant: des mémoires et notices, des analyses et extraits d’ouvrages nouveaux; des variétés et mélanges, des annonces bibliographiques, etc., etc. (Moscow, Russia).

Bull Acad R Sci Belg: Bulletins de l’Académie Royale des Sciences, des Lettres et des Beaux-Arts de Belgique (Bruxelles, Belgium).

Bull Acad Sci St-Pétersb: Bulletin de l’Académie Impériale des Sciences de St.-Péterbourg (St. Petersburg, Russia).

Bull Soc Imp Nat Mosc: Bulletin de la Société Impériale des Naturalistes de Moscou (Moscow, Russia).

Bull Soc Ent Fr: Bulletin de la Société Entomologique de France (Paris, France).

Bull Soc Hist Nat Toulouse: Bulletin de la Société d’Histoire Naturelle de Toulouse (Toulouse, France).

Bull Soc Imp Nat Mosc: Bulletin de la Société Impériale des Naturalistes de Moscou (Moscow, Russia).

Bull Zool Nomencl: The Bulletin of Zoological Nomenclature (London, U.K.).

Compt Rend Acad Sci Fr: Comptes Rendus (hebdomadaire) des séances de l’Académie des Sciences de France (Paris, France).

Compt Rend Soc Ent Belg: Comptes Rendus des Séances de la Société Entomologique de Belgique (Bruxelles, Belgium).

Ent Bericht: Entomologische Berichten (Amsterdam, Netherlands).

Ent Nachr: Entomologische Nachrichten (Berlin, Germany).

Ent News: Entomological News (Academy of Natural Sciences, Philadelphia, U.S.A.).

Ent Ztg Stettin: Entomologische Zeitung (Stettin, Germany [now in Poland]).

Feuil J Lib: Feuilleton du Journal de la Librairie Nationale de la France (issued with “Bibliographie de la France”; Paris, France).

Intell Allg Lit Ztg: Intelligenzblatt der Allgemeinen Literatur-Zeitung (Jena/Halle, Germany; published in conjunction with “Allgemeine Literatur-Zeitung”).

Leipz Repert: Leipziger Repertorium der Deutschen und Ausländischen Literatur (Leipzig, Germany).

Lit Centr Deutsch: Literarisches Centralblatt für Deutschland (Leipzig, Germany).

Lit Ztg: Literarische Zeitung (Berlin, Germany).

Lond Lit Gaz: The London Literary Gazette, and Journal of the Belles Lettres, Arts, Sciences (London, U.K.).

Merc Fr: Mercure de France (Paris, France).

Nat Nov: Naturae Novitates (Berlin, Germany).

Nature: Nature, a Weekly Illustrated Journal of Science (London, U.K. and New York, U.S.A.).

Öfvers Kongl Vetens Forh: Öfversigt af Kongliga Vetenskaps-Akademiens Förhandlingar (Stockholm, Sweden).

Pan-Pacific Ent: The Pan-Pacific Entomologist (San Francisco, U.S.A.).

Proc Acad Nat Sci Philad: Proceedings of the Academy of Natural Sciences, Philadelphia (Philadelphia, U.S.A.).

Proc Amer Phil Soc: Proceedings of the American Philosophical Society, held at Philadelphia, for promoting useful knowledge (Philadelphia, U.S.A.).

Proc Bost Soc Nat Hist: Proceedings of the Boston Society of Natural History (Boston, U.S.A.).

Proc Ent Acad Nat Sci: Proceedings of the Monthly Meetings of the Entomological Section of the Academy of Natural Sciences of Philadelphia (Philadelphia, U.S.A.).

Proc Ent Soc Lond: The Proceedings of the Entomological Society of London [and Journal of Proceedings] (London, U.K.).

Proc Ent Soc NSW: The Proceedings of the Entomological Society of New South Wales (Sydney, Australia; published with the Transactions).

Proc Linn Soc NSW: The Proceedings of the Linnean Society of New South Wales (Sydney, Australia).

Proc-verb Acad Sci Fr: Procès-verbaux des Séances de l’Académie des Sciences de France (Paris, France).

Rev Mag Zool: Revue et Magasin de Zoologie (Paris, France).

Rev Zool: Revue Zoologique par la Société Cuvierienne, Journal Mensuel (Paris, France).

Sitz-Ber Zool-Bot Ges Wien: Sitzungsberichte der Kaiserlich-Königlichen Zoologisch-Botanischen Gesellschaft in Wien (Vienna, Austria).

Soc Ent Fr, Bull Bibliogr: Société Entomologique de France, Bulletin Bibliographique (Paris, France).

Soc Ent Fr, Bull Ent: Société Entomologique de France, Bulletin Entomologique (Paris, France).

Tijdschr Ent: Tijdschrift voor Entomologie (Nederlandsche Entomologische Vereniging, Leiden, Netherlands).

Trans Ent Soc Lond: Transactions of the Entomological Society of London (London, U.K.).

Trans Proc New Zealand Inst: Transactions and Proceedings of the New Zealand Institute (Wellington, New Zealand).

Verh Ver Naturw Unt Hamb: Verhandlungen des Vereins für Naturwissenschaftliche Unterhaltung zu Hamburg (Hamburg, Germany).

Verz Bücher Landkarten: Verzeichniss der Bücher, Landkarten &c (Leipzig, Germany).

Verz neuer Bücher: Verzeichniss neuer Bücher (Leipzig, Germany).

Zool Rec: Zoological Record (London, U.K.).

Zoologist: The Zoologist: A Popular Miscellany of Natural History (London, U.K.).

### ﻿Works reporting dates of publication

The following works included important information for dating the references: Alonso-Zarazaga and Lyal (1999), Bellamy (2003), Bousquet (1993, 2012, 2016, 2017a, 2018), Bousquet and Bouchard (2017), Bousquet and Davies (2021), F.M. Brown (1964a, 1964b), Cowan (1969), Dawson (1958), Duncan (1937), Evenhuis (1994, 1997a-c, 2002, 2003a-b, 2008, 2012, 2014, 2015a, 2015b, 2019), Fox (1913), Griffin (1932), von Hayek (1989), Horn and Schenkling (1928–1929), Houston and Weir (1992), ICZN (1958b, 1999a), Kappel (1896), Kerzhner (1984), Lefèvre (1885, 1895), Merkl et al. (2008), Nagel and Schmidlin (2014), Oshanin (1910), Peavot (1913), Poggi (2010), Raphael (1970), Sclater (1893), Sherborn (1922, 1934, 1937), Silfverberg (1971), G. Wheeler (1912), and Zerche (1987).

### ﻿Establishments recording receipt of publications (date stamps)

**Biblioth Nat Fr**: Bibliothèque Nationale de France (Paris, France)

**BMNH**: The Natural History Museum (London, U.K.)

**CNC**: Canadian National Collection of Insects, Arachnids and Nematodes (Ottawa, Canada)

**CUL**: Cornell University Library (Ithaca, New York, U.S.A.)

**Smithsonian**: The Smithsonian Institution (Washington, D.C., U.S.A.)

### ﻿Availability and date of publication of electronic works published after 2011

We established the availability of electronic works published after 2011 that contained new names or new nomenclatural acts following the recommendations outlined in amendments to the Code (ICZN 2012f). Based on these requirements, electronic works are available when: 1) simultaneously obtainable copies of the work with fixed content and layout are widely accessible (Art. 8.1), and are not disclaimed (Arts 8.2, 8.3); 2) the work itself contains a date of publication (Art. 8.5.2); 3) the work was registered in ZooBank before it was published and contains evidence of such registration (Art. 8.5.3); 4) the ZooBank record indicates an intended online archive (Art. 8.5.3.1); and 5) the ZooBank record includes an ISSN/ISBN (Art. 8.5.3.2).

In many cases, works published after 2011 were issued electronically first and then in print later. According to Article 21.9 (ICZN 2012f) the “name or nomenclatural act published in a work issued in both print and electronic editions takes its date of publication from the edition that first fulfilled the criteria of publication of Article 8 and is not excluded by Article 9.” As recommended by Krell (2015), we use the final page numbers, since they are better finding aids, when pagination in an available electronic work differs from the pagination of the same work subsequently assigned to a particular journal issue. We report the first date of publication we could find for cited works published after 2011 that do not contain new names or new nomenclatural acts.

The following works, which were issued and distributed electronically only, are unavailable because they failed to meet one or more of the criteria of availability published in ICZN (2012f): Legalov (2017a, 2018c, but see Legalov 2023c), Kirejtshuk (2020), Rosa et al. (2020a, but see Rosa et al. 2020b), Szawaryn et al. (2020) and Motyka et al. (2023a). The new names and nomenclatural acts included in those works are therefore nomenclaturally unavailable. In order to promote stability (since the majority of the zoological community may not be aware of these facts), such unavailable names and acts are included in this work but preceded by an asterisk “*”. The Commission is currently exploring potential solutions to address the growing problem with new names and acts proposed only in electronic works since 2012 and that are not Code compliant (T. Pape, pers. comm. to P.B., 2022).

### ﻿Dates of publication for Bonvouloir’s ‘Monographie de la famille des eucnémides’ (1871–1875)

Bousquet (2016: 87) gave the dates “1871–1875” for this publication since he could find no evidence that the plates were issued in 1870, in spite of the fact that they all have the inscription “Tome X. 2e Partie (1870)”. Bonvouloir submitted the 42 plates and a preliminary version of the text to the Société Entomologique de France on 23.III.1870 (Bull Soc Ent Fr 1870: xvii), with the hope of having them published in Tome X. On 13.IV.1870 the Commission de Publication agreed to print the plates “au nombre ordinaire de notre tirage des Annales” (Bull Soc Ent Fr 1870: xxv–xxvi), but this apparently did not happen, since no collection of the plates was ever recorded with the Annales for 1870 or anywhere else, in spite of the fact that they were to be distributed “gratuitement à nos membres ainsi qu’aux diverses sociétés savantes”. Bonvouloir was the ‘Archiviste-Bibliothécaire’ for the Société at the time, so one would expect that if they were available in 1870, they would have been entered into the Bibliothèque at least in 1871 (the first notice of copies being available, as cahiers). The dates recorded in the Bulletin Bibliographique (see References) were very specific as to the number of pages and plates in each cahier, as well as to the “dates de parution”. Likewise, the text was not published “en deux fascicules: l’un publié dans le courant de 1870 et l’autre au commencement de 1871” as originally agreed. In fact, Bonvouloir made additions and changes to the text long after the original was submitted, citing (p. 765) a paper published at the end of 1871. Evidently he sent Gemminger a preliminary list of the new names and plates in 1869 because they were listed (as “Monogr. Bonvouloir. *Ann. Fr. 1870*” or “Bonv. *Mon.*”) with their provenances, in the Catalogue by Gemminger and Harold (1869b), but the page numbers were never cited, only the prospective plate and figure numbers for those illustrated, and some of the figures from this list were changed in the actual publication (e.g., *Microrhaguspavidus* “Bonv. Mon. t. 27 f. 2” was replaced with *M.inconsultus*, pl. 27 fig. 2; *Nematodesbuqueti* (pl. 33 fig. 1) was not listed; *Cephalodendronmozambicanum* “Bonv. Mon. t. 36. f. 1” was changed to *C.indigaceum* var., etc.). Similarly, Gemminger included names published by Baudi the following year (1870), without page numbers. In the *Nécrologie* for Bonvouloir (Bull Soc Ent Fr 1914: 407) it was noted that the *Monographie* was issued “en 4 fascicules publiés de 1871 à 1875”, with no mention of a separate edition of the plates. We follow Muona (1987: 79–92) who, in his review of the generic names of Eucnemidae, did not include the plates as a separate publication, but cited species and genera from the plates as issued in the cahiers as 1871–1875.

## ﻿Results

### ﻿Alphabetical list of type genera in Coleoptera

***Abacetus* Dejean, 1828**: 195. Current status: valid genus in CARABIDAE: HARPALINAE: ABACETINI: ABACETINA.

Type species: *Abacetusgagates* Dejean, 1828, by monotypy.

Family-group name: ABACÉTIDES Chaudoir, 1873. Stem: *Abacet*-.

***Abacomorphus* Chaudoir, 1878a**: 14. Current status: valid genus in CARABIDAE: HARPALINAE: PTEROSTICHINI: ABACOMORPHINA.

Type species: *Abaxcaledonicus* Montrouzier, 1860, by monotypy.

Family-group name: ABACOMORPHINI Tschitschérine, 1902. Stem: *Abacomorph*-.

***Abax* Bonelli, 1810**: Tab. Syn. Current status: valid genus in CARABIDAE: HARPALINAE: PTEROSTICHINI: PTEROSTICHINA.

Type species: *Carabusstriola* Fabricius, 1792 (= *Carabusparallelepipedus* Piller & Mitterpacher, 1783), by subsequent designation (Westwood 1838c: 4). Note: no species were originally included in this genus by Bonelli (1810); the first ones were the two species listed by Panzer (1813: 68).

Family-group name: ABAXINI Schuler, 1970. Stem: *Abac*-.

***Ablabera* Dejean, 1833b**: 159. Current status: valid genus in SCARABAEIDAE: SERICINAE: ABLABERINI.

Type species: *Melolonthasplendida* Fabricius, 1781, by monotypy.

Family-group name: ABLABERITAE Blanchard, 1850. Stem: *Ablaber*-.

***Abraeus* Leach, 1817**: 76. Current status: valid genus in HISTERIDAE: ABRAEINAE: ABRAEINI.

Type species: *Histerglobosus* P.W.J. Müller, 1803 (= *Histerperpusillus* Marsham, 1802), by subsequent designation (Westwood 1838c: 22).

Family-group name: ABREIDAE W.S. MacLeay, 1819. Stem: *Abrae*-.

†***Abrocar* M. Liu & Ren, 2006**: 62. Current status: valid genus in CARIDAE: †ABROCARINI. Note: Lower Cretaceous (China).

Type species: †*Abrocarbrachyorhinos* M. Liu & Ren, 2006, by original designation.

Family-group name: †ABROCARINA Legalov, 2009. Stem: *Abrocar*-.

***Abroteles* Casey, 1890a**: 190. Current status: valid genus in STAPHYLINIDAE: ALEOCHARINAE: COROTOCINI: ABROTELINA.

Type species: *Abrotelesbeaumonti* Casey, 1890, by monotypy.

Family-group name: ABROTELINA Seevers, 1957. Stem: *Abrotel*-.

***Abryna* Newman, 1842a**: 289. Current status: valid genus in CERAMBYCIDAE: LAMIINAE: PTEROPLIINI.

Type species: *Abrynacoenosa* Newman, 1842, by subsequent designation (J. Thomson 1864: 44).

Family-group name: ABRYNITAE J. Thomson, 1864. Stem: *Abryn*-.

***Acalles* Schönherr, 1825**: 586. Current status: valid genus in CURCULIONIDAE: CRYPTORHYNCHINAE: CRYPTORHYNCHINI: TYLODINA.

Type species: *Curculiocamelus* Fabricius, 1792, by original designation.

Family-group name: ACALLINAE Joy, 1932. Stem: *Acall*-.

***Acallopistus* Schönherr, 1826**: 249. Current status: valid genus in CURCULIONIDAE: CURCULIONINAE: NERTHOPINI.

Type species: *Acallopistusvellicosus* Schönherr, 1826, by original designation.

Family-group name: ACALLOPISTIDES Lacordaire, 1865. Stem: *Acallopist*-.

***Acalyptomerus* Crowson, 1979**: 615. Current status: valid genus in CLAMBIDAE: ACALYPTOMERINAE.

Type species: *Acalyptomerusasiaticus* Crowson, 1979, by original designation.

Family-group name: ACALYPTOMERINAE Crowson, 1979. Stem: *Acalyptomer*-.

***Acalyptus* Schönherr, 1833**: 20. Current status: valid genus in CURCULIONIDAE: CURCULIONINAE: ACALYPTINI.

Type species: *Curculiocarpini* Fabricius, 1792, by original designation.

Family-group name: ACALYPTINA C.G. Thomson, 1859. Stem: *Acalypt*-. Note: Gapon et al. (2019: 175) submitted an application to the Commission to remove the homonymy between this family-group name and ACALYPTINI Blatchley, 1926 (type genus *Acalypta* Westwood, 1840) in Hemiptera; Gapon et al. (2019: 175) recommended a change to the family-group name in Hemiptera, leaving ACALYPTINI C.G. Thomson, 1859 in Coleoptera unchanged.

***Acamptus* J.L. LeConte in J.L. LeConte and G.H. Horn, 1876**: 238. Current status: valid genus in CURCULIONIDAE: COSSONINAE: ACAMPTINI.

Type species: *Acamptusrigidus* J.L. LeConte, 1876, by monotypy.

Family-group name: ACAMPTI J.L. LeConte, 1876. Stem: *Acampt*-.

***Acangassu* Galileo & Martins, 2001**: 97. Current status: valid genus in CERAMBYCIDAE: CERAMBYCINAE: ACANGASSUINI.

Type species: *Acangassudiminuta* Galileo & Martins, 2001, by original designation.

Family-group name: ACANGASSUINI Galileo & Martins, 2001. Stem: *Acangassu*-.

***Acanthinodera* Hope, 1834a**: 106. Current status: junior synonym of *Amallopodes* Lequien, 1833 in CERAMBYCIDAE: PRIONINAE: MACRODONTIINI.

Type species: *Prionuscumingii* Hope, 1834, by original designation.

Family-group name: ACANTHINODERITAE J. Thomson, 1864. Stem: *Acanthinoder*-.

***Acanthinomerus* Boheman, 1859**: 141. Current status: valid genus in CURCULIONIDAE: COSSONINAE: ACANTHINOMERINI.

Type species: *Acanthinomerusarmatus* Boheman, 1859, by monotypy.

Family-group name: ACANTHINOMERINI Voss, 1972. Stem: *Acanthinomer*-.

***Acanthocerus* W.S. MacLeay, 1819**: 136. Current status: invalid senior synonym of *Ceratocanthus* A. White, 1842 in HYBOSORIDAE: CERATOCANTHINAE: CERATOCANTHINI. Note: junior homonym of *Acanthocerus* Palisot de Beauvois, 1818 [Hemiptera].

Type species: *Acanthocerusaeneus* W.S. MacLeay, 1819, by subsequent designation (Duponchel 1839: 30).

Family-group name: ACANTHOCERIDAE Streubel, 1846. Stem: *Acanthocer*-. Note: family-group name permanently invalid (ICZN 1999a, Article 39).

***Acanthocinus* Dejean, 1821**: 106. Current status: valid genus in CERAMBYCIDAE: LAMIINAE: ACANTHOCININI.

Type species: *Cerambyxaedilis* Linnaeus, 1758, by subsequent designation (Blanchard in Audouin et al. 1841: pl. 67).

Family-group name: ACANTHOCINITES Blanchard, 1845. Stem: *Acanthocin*-.

***Acanthocnemus* Perris, 1866**: 187. Current status: valid genus in ACANTHOCNEMIDAE.

Type species: *Acanthocnemusciliatus* Perris, 1866 (= *Dasytesnigricans* Hope, 1843), by monotypy.

Family-group name: ACANTHOCNEMINAE Crowson, 1964. Stem: *Acanthocnem*-.

***Acanthoderes* Audinet-Serville, 1835a**: 29. Current status: valid genus in CERAMBYCIDAE: LAMIINAE: ACANTHODERINI.

Type species: *Cerambyxdaviesii* Swederus, 1787, by subsequent designation (Duponchel 1839: 32).

Family-group name: ACANTHODERITAE J. Thomson, 1860. Stem: *Acanthoder*-.

***Acanthoglossa* Kraatz, 1859**: 144. Current status: valid genus in STAPHYLINIDAE: PAEDERINAE: LATHROBIINI: MEDONINA.

Type species: *Acanthoglossahirta* Kraatz, 1859, by subsequent designation (R. Lucas 1920: 68).

Family-group name: ACANTHOGLOSSI Coiffait, 1982. Stem: *Acanthogloss*-.

***Acanthomerosternoplon* Tippmann, 1956**: 10. Current status: junior synonym of *Omosarotes* Pascoe, 1860 in CERAMBYCIDAE: LAMIINAE: CYRTININI.

Type species: *Acanthomerosternoplonparadoxum* Tippmann, 1956, by original designation.

Family-group name: ACANTHOMEROSTERNOPLONINI Tippmann, 1956. Stem: Acanthomerosternopl-.

***Acanthophorus* Audinet-Serville, 1832**: 152. Current status: valid genus in CERAMBYCIDAE: PRIONINAE: ACANTHOPHORINI.

Type species: *Prionusserraticornis* G.-A. Olivier, 1795, by subsequent designation (Duponchel 1839: 35).

Family-group name: ACANTHOPHORITAE J. Thomson, 1864. Stem: *Acanthophor*-.

***Acanthopygus* Montrouzier, 1861a**: 869. Current status: valid genus in ANTHRIBIDAE: ANTHRIBINAE: ACANTHOPYGINI.

Type species: *Acanthopygusmetallicus* Montrouzier, 1861, by subsequent designation (Kuschel 1998: 346).

Family-group name: ACANTHOPYGINI Legalov, 2023c: 22. Stem: *Acanthopyg*-. Note: ACANTHOPYGINILegalov (2018c: 787) was published in a work that is nomenclaturally unavailable (ICZN 2012f, Article 8.5.3).

***Acanthoscelides* Schilsky, 1905**: 95. Current status: valid genus in CHRYSOMELIDAE: BRUCHINAE: BRUCHINI: ACANTHOSCELIDINA. Note: two original spellings were used: *Acanthocelides* (pp. 41f, 41g, 41C) and *Acanthoscelides* (pp. 95, 96, 97, 98); Neave (1939: 11) acted as First Reviser (ICZN 1999a, Article 24.2.3) and selected *Acanthocelides*; an application to the Commission is necessary to retain the spelling *Acanthoscelides*, which is in prevailing usage.

Type species: *Bruchusirresectus* Fåhraeus, 1839 (= *Bruchusobtectus* Say, 1831), by subsequent designation (Bridwell 1929: 42).

Family-group name: ACANTHOSCELIDINI Bridwell, 1946. Stem: *Acanthoscelid*-.

***Acanthoscelis* Dejean, 1825**: 402. Current status: valid genus in CARABIDAE: SCARITINAE: SCARITINI: ACANTHOSCELINA.

Type species: *Scaritesruficornis* Fabricius, 1801, by monotypy.

Family-group name: ACANTHOSCELINA Csiki, 1927. Stem: *Acanthoscel*-.

***Acanthotrachelus* Schönherr, 1842b**: 236. Current status: valid genus in CURCULIONIDAE: ENTIMINAE: CYPHICERITAE: CYPHICERINI: ACANTHOTRACHELINA.

Type species: *Acanthotrachelusventricosus* Boheman, 1842, by original designation.

Family-group name: ACANTHOTRACHELINI G.A.K. Marshall, 1944. Stem: *Acanthotrachel*-.

***Acanthovalgus* Kraatz, 1895**: 444. Current status: valid genus in SCARABAEIDAE: CETONIINAE: VALGINI: VALGINA.

Type species: *Acanthovalgusmarquardi* Kraatz, 1895, by monotypy.

Family-group name: ACANTHOVALGINAE Kolbe, 1904. Stem: *Acanthovalg*-.

***Acentrus* Duponchel, 1839**: 61. Current status: valid genus in CURCULIONIDAE: CURCULIONINAE: ACENTRINI. Note: we consider that *Acentrus*, as used by Schönherr (1845: 57), is a subsequent use of *Acentrus* Duponchel, 1839 and not a new distinct taxon; the entry in the *Dictionnaire universel d’histoire naturelle* where the name *Acentrus* is made available is signed “(D.)” which corresponds to Duponchel (as indicated in the “Liste des auteurs par ordre de matières”), not to Desmarest as reported by some authors (e.g., Alonso-Zarazaga et al. 2017: 180).

Type species: *Cryptorhynchushistrio* Schönherr, 1837, by original designation.

Family-group name: ACENTRINA Seidlitz, 1890. Stem: *Acentr*-.

***Aceraius* Kaup, 1868a**: 26. Current status: valid genus in PASSALIDAE: PASSALINAE: MACROLININI.

Type species: *Passalusgrandis* Burmeister, 1847, by subsequent designation (Gravely 1918: 89).

Family-group name: ACERAIAE Kaup, 1871. Stem: *Acerai*-.

***Acetalius* Sharp, 1883b**: 322. Current status: valid genus in STAPHYLINIDAE: PSELAPHINAE: EUPLECTITAE: TRICHONYCHINI: PANAPHANTINA.

Type species: *Acetaliusdubius* Sharp, 1883, by monotypy.

Family-group name: ACETALIINI Jeannel, 1958. Stem: *Acetali*-.

***Achaenops* Suffrian, 1857**: 61, 234. Current status: valid genus in CHRYSOMELIDAE: CRYPTOCEPHALINAE: CRYPTOCEPHALINI: ACHAENOPINA.

Type species: *Achaenopsdorsalis* Suffrian, 1857, by monotypy.

Family-group name: ACHAENOPITES Chapuis, 1874. Stem: *Achaenop*-.

***Acherusia* Laporte & Gory, 1838**: 1. Current status: invalid senior synonym of *Mimicoclytrina* Bellamy, 2003 in BUPRESTIDAE: POLYCESTINAE: TYNDARIDINI: MIMICOCLYTRININA. Note: junior homonym of *Acherusia* O.G. Costa, 1834 [Crustacea].

Type species: *Acherusiachildreni* Laporte & Gory, 1838, by monotypy.

Family-group name: ACHERUSINI Cobos, 1955. Stem: *Acherusi*-. Note: family-group name permanently invalid (ICZN 1999a, Article 39).

***Achloa* Erichson, 1840a**: 41. Current status: valid genus in SCARABAEIDAE: MELOLONTHINAE: TANYPROCTINI: TANYPROCTINA.

Type species: *Achloahelvola* Erichson, 1840, by **present designation**.

Family-group name: ACHLOIDAE Burmeister, 1855. Stem: *Achlo*-.

***Achryson* Audinet-Serville, 1834a**: 572. Current status: valid genus in CERAMBYCIDAE: CERAMBYCINAE: ACHRYSONINI.

Type species: *Stenocoruscircumflexus* Fabricius, 1787, by monotypy.

Family-group name: ACHRYSONIDES Lacordaire, 1868. Stem: *Achryson*-.

***Acicnemis* Fairmaire, 1849**: 511. Current status: valid genus in CURCULIONIDAE: MOLYTINAE: TRACHODINI.

Type species: *Acicnemisvariegata* Fairmaire, 1849, by subsequent designation (Fairmaire 1881: 299).

Family-group name: ACICNÉMIDES Lacordaire, 1865. Stem: *Acicnemid*-.

***Acidocerus* Klug, 1855**: 649. Current status: valid genus in HYDROPHILIDAE: ACIDOCERINAE.

Type species: *Acidocerusaphodioides* Klug, 1855, by monotypy.

Family-group name: ACIDOCERINI Zaitzev, 1908. Stem: *Acidocer*-.

***Acilius* Leach, 1817**: 69, 72. Current status: valid genus in DYTISCIDAE: DYTISCINAE: ACILIINI. Note: the senior homonym *Acilius* Rafinesque, 1815, an unnecessary new replacement name for *Symethus* Rafinesque, 1814 in Crustacea, was suppressed for both the Principles of Homonymy and Priority and *Acilius* Leach, 1817 placed on the Official List of Generic Names in Zoology (ICZN 1961e).

Type species: *Dytiscussulcatus* Linnaeus, 1758, by monotypy (see ICZN 1961e). Note: name placed on the Official List of Specific Names in Zoology (ICZN 1961e).

Family-group name: ACILIIDES C.G. Thomson, 1867. Stem: *Acili*-.

***Acinopus* Dejean, 1821**: 13. Current status: valid genus in CARABIDAE: HARPALINAE: HARPALINI: HARPALINA.

Type species: **here fixed** (under ICZN 1999a, Article 70.3.2) as *Scaritespicipes* G.-A. Olivier, 1795, misidentified as *Carabusmegacephalus* Rossi, 1794 (sensu Illiger, 1802) in the original designation by monotypy in Dejean (1821: 13).

Family-group name: ACINOPIDAE Laporte, 1834. Stem: *Acinopod*-.

***Aclopus* Erichson, 1835**: 259. Current status: valid genus in SCARABAEIDAE: ACLOPINAE: ACLOPINI.

Type species: *Aclopusvittatus* Erichson, 1835, by subsequent designation (R. Lucas 1920: 70).

Family-group name: ACLOPITAE Blanchard, 1850. Stem: *Aclop*-.

***Acmaeodera* Eschscholtz, 1829b**: 9. Current status: valid genus in BUPRESTIDAE: POLYCESTINAE: ACMAEODERINI: ACMAEODERINA. Note: name placed on the Official List of Generic Names in Zoology (ICZN 2005b).

Type species: *Buprestiscylindrica* Fabricius, 1775, by subsequent designation (Volkovitsh 1979: 339; see ICZN 2005b). Note: name placed on the Official List of Specific Names in Zoology (ICZN 2005b).

Family-group name: ACMAEODERINI Kerremans, 1893. Stem: *Acmaeoder*-.

***Acmaeoderoides* Van Dyke, 1942**: 108. Current status: valid genus in BUPRESTIDAE: POLYCESTINAE: ACMAEODERINI: ACMAEODEROIDINA.

Type species: *Acmaeoderainsignis* G.H. Horn, 1894, by original designation.

Family-group name: ACMAEODEROIDINI Cobos, 1955. Stem: *Acmaeoderoid*-.

***Acmaeops* J.L. LeConte, 1850a**: 316, 321. Current status: valid genus in CERAMBYCIDAE: LEPTURINAE: ACMAEOPINI.

Type species: *Lepturaproteus* W. Kirby, 1837, by subsequent designation (Casey 1913: 219).

Family-group name: ACMAEOPSINI Della Beffa, 1915. Stem: *Acmaeop*-.

***Acmocera* Dejean, 1835**: 345. Current status: valid genus in CERAMBYCIDAE: LAMIINAE: ACMOCERINI.

Type species: *Lamiacompressa* Fabricius, 1801, by monotypy.

Family-group name: ACMOCERITAE J. Thomson, 1864. Stem: *Acmocer*-.

***Acoma* Casey, 1890a**: 165. Current status: valid genus in SCARABAEIDAE: MELOLONTHINAE: ACOMINI.

Type species: *Acomabrunnea* Casey, 1890, by monotypy.

Family-group name: ACOMINI Evans & A.B.T. Smith, 2020: 54. Stem: *Acom*-.

***Acorynus* Schönherr, 1833**: 3, 123. Current status: valid genus in ANTHRIBIDAE: ANTHRIBINAE: TROPIDERINI.

Type species: *Acorynussulcirostris* Boheman, 1833, by original designation.

Family-group name: ACORYNIDES Lacordaire, 1865. Stem: *Acoryn*-.

***Acratus* Lacordaire, 1865**: 463. Current status: valid genus in BRENTIDAE: BRENTINAE: BRENTINI: ITHYSTENINA.

Type species: *Brentussuturalis* Lund, 1802, by original designation.

Family-group name: ACRATINI Alonso-Zarazaga, Lyal, Bartolozzi & Sforzi, 1999. Stem: *Acrat*-.

***Acridocephala* Chevrolat, 1855**: 287. Current status: valid genus in CERAMBYCIDAE: LAMIINAE: ACRIDOCEPHALINI.

Type species: *Acridocephalabistriata* Chevrolat, 1855, by monotypy.

Family-group name: ACRIDOCEPHALIDI E.S. Dillon & L.S. Dillon, 1959. Stem: *Acridocephal*-.

***Acritomorphus* Wenzel, 1944**: 56. Current status: valid genus in HISTERIDAE: ABRAEINAE: ACRITOMORPHINI.

Type species: *Acritomorphuspraecursor* Wenzel, 1944, by original designation.

Family-group name: ACRITOMORPHINI Wenzel, 1944. Stem: *Acritomorph*-.

***Acritorrhynchites* Voss, 1941**: 119, 212. Current status: valid genus in ATTELABIDAE: RHYNCHITINAE: RHYNCHITITAE: EUGNAMPTINI.

Type species: *Rhynchitesaddendus* Sharp, 1889, by monotypy.

Family-group name: ACRITORRHYNCHITINA Legalov, 2007. Stem: *Acritorrhynchit*-.

***Acritosoma* Pakaluk & Ślipiński, 1995**: 330. Current status: valid genus in ANAMORPHIDAE.

Type species: *Acritosomaelongatum* Pakaluk & Ślipiński, 1995, by original designation.

Family-group name: ACRITOSOMATINAE Pakaluk & Ślipiński, 1995. Stem: *Acritosomat*-.

***Acritus* J.L. LeConte, 1853c**: 288. Current status: valid genus in HISTERIDAE: ABRAEINAE: ACRITINI.

Type species: *Histernigricornis* Hoffmann, 1803, by subsequent designation (C.G. Thomson 1859: 75).

Family-group name: ACRITINI Wenzel, 1944. Stem: *Acrit*-.

***Acrobolbia* Ohaus, 1912b**: 316. Current status: valid genus in SCARABAEIDAE: DYNASTINAE: CYCLOCEPHALINI.

Type species: *Acrobolbiamacrophylla* Ohaus, 1912, by monotypy.

Family-group name: ACROBOLBIINA Ohaus, 1918. Stem: *Acrobolbi*-.

***Acrocinus* Illiger, 1806**: 247. Current status: valid genus in CERAMBYCIDAE: LAMIINAE: ACROCININI.

Type species: *Cerambyxlongimanus* Linnaeus, 1758, by monotypy.

Family-group name: ACROCININAE Swainson, 1840. Stem: *Acrocin*-.

***Acrognathus* Erichson, 1839a**: 607. Current status: invalid senior synonym of *Manda* Blackwelder, 1952 in STAPHYLINIDAE: OXYTELINAE: PLANEUSTOMINI. Note: junior homonym of *Acrognathus* Agassiz, 1836 [Pisces].

Type species: *Omaliummandibulare* Gyllenhal, 1827, by subsequent designation (Duponchel 1841b: 57).

Family-group name: ACROGNATHINI Reitter, 1909. Stem: *Acrognath*-. Note: family-group name permanently invalid (ICZN 1999a, Article 39).

***Acroleptus* Bourgeois, 1886**: lxx. Current status: valid genus in LYCIDAE: LYCINAE: CALOPTERINI: ACROLEPTINA.

Type species: *Acroleptuschevrolati* Bourgeois, 1886, by monotypy.

Family-group name: ACROLEPTINA Bocáková, 2005. Stem: *Acrolept*-.

***Acropis* Burmeister, 1840a**: [Heft 5]. Current status: valid genus in ZOPHERIDAE: COLYDIINAE: ACROPINI.

Type species: *Acropistuberculiferus* Burmeister, 1840, by monotypy.

Family-group name: ACROPINAE Sharp, 1894. Stem: *Acrop*-.

***Acropteron* Perty, 1832**: 64. Current status: junior synonym of *Acropteryx* Gistel, 1831 in TENEBRIONIDAE: TENEBRIONINAE: ACROPTERONINI.

Type species: *Acropteronrufipes* Perty, 1832 (= *Acropteryxrufipes* Gistel, 1831), by subsequent designation (Hope 1841a: 133).

Family-group name: ACROPTERONINI Doyen, 1989. Stem: *Acropteron*-.

***Acrotona* C.G. Thomson, 1859**: 38. Current status: valid genus in STAPHYLINIDAE: ALEOCHARINAE: ATHETINI: ATHETINA. Note: name placed on the Official List of Generic Names in Zoology (ICZN 1961b).

Type species: *Aleocharaaterrima* Gravenhorst, 1802, by original designation (see ICZN 1961b). Note: name placed on the Official List of Specific Names in Zoology (ICZN 1961b).

Family-group name: ACROTONAE Seevers, 1978. Stem: *Acroton*-.

***Acrotrichis* Motschulsky, 1848**: 569, table. Current status: valid genus in PTILIIDAE: PTILIINAE: ACROTRICHINI.

Type species: *Latridiusfascicularis* Herbst, 1793, by subsequent designation (C. Johnson 1969: 229).

Family-group name: ACROTRICHINI Reitter, 1909. Stem: *Acrotrich*-.

***Actenodes* Dejean, 1833a**: 80. Current status: valid genus in BUPRESTIDAE: BUPRESTINAE: ACTENODINI. Note: name placed on the Official List of Generic Names in Zoology (ICZN 2002b).

Type species: *Buprestisnobilis* Linnaeus, 1758, by monotypy (see ICZN 2002b). Note: name placed on the Official List of Specific Names in Zoology (ICZN 2002b).

Family-group name: ACTENODEIDAE Gistel, 1848. Stem: *Actenod*-.

***Actenonyx* A. White, 1846**: 2. Current status: valid genus in CARABIDAE: HARPALINAE: ODACANTHINI: ACTENONYCINA.

Type species: *Actenonyxbembidioides* A. White, 1846, by monotypy.

Family-group name: ACTENONYCINAE H.W. Bates, 1871. Stem: *Actenonyc*-.

***Actidium* A. Matthews, 1868**: 12. Current status: valid genus in PTILIIDAE: PTILIINAE: PTILIINI.

Type species: *Ptiliumcoarctatum* Haliday, 1855, by original designation.

Family-group name: ACTIDIINI Portevin, 1929. Stem: *Actidi*-.

***Actinophorus* Creutzer, 1799**: 79. Current status: junior synonym of *Scarabaeus* Linnaeus, 1758 in SCARABAEIDAE: SCARABAEINAE: SCARABAEINI.

Type species: *Scarabaeussacer* Linnaeus, 1758, by subsequent designation (Ádám 2003: 130).

Family-group name: ACTINOPHORINI Ádám, 2003. Stem: *Actinophor*-.

***Actizeta* Pascoe, 1875**: 214. Current status: valid genus in TENEBRIONIDAE: PIMELIINAE: CNEMEPLATIINI: ACTIZETINA.

Type species: *Actizetaammobioides* Pascoe, 1875 (= *Actizetaalbata* Pascoe, 1875), by original designation.

Family-group name: ACTIZETINA Watt, 1992. Stem: *Actizet*-.

***Actobaena* Gistel, 1856**: 266, 355. Current status: junior synonym of *Hyphydrus* Illiger, 1802 in DYTISCIDAE: HYDROPORINAE: HYPHYDRINI.

Type species: *Hyphydrusfabricii* Cristofori & Jan, 1832 (= *Dytiscusovatus* Linnaeus, 1761), by monotypy.

Family-group name: ACTOBAENIDAE Gistel, 1856. Stem: *Actobaen*-.

***Actocharis* Fauvel, 1871**: 19. Current status: junior synonym of *Actocharis* Sharp, 1870 in STAPHYLINIDAE: ALEOCHARINAE: ACTOCHARINI. Note: junior homonym of *Actocharis* Sharp, 1870 [Coleoptera: STAPHYLINIDAE].

Type species: *Actocharismarina* Fauvel, 1871 (= *Actocharisreadingii* Sharp, 1870), by monotypy.

Family-group name: ACTOCHARI Bernhauer & Schubert, 1911. Stem: *Actochar*-. Note: this family-group name was based on *Actocharis* “Fauvel, 1869” by Bernhauer and Schubert (1911: 91), assuming that the name had been issued prior to *Actocharis* Sharp, 1870; however, Fauvel’s name was not issued until 1871 (see Rye 1872: 30, 31) and is currently treated as a junior homonym of Sharp’s name; therefore, ACTOCHARINI Bernhauer & Schubert, 1911 is permanently invalid (ICZN 1999a, Article 39); an application to the Commission is necessary to change the type genus of this family-group name to *Actocharis* Sharp, 1870 or a new family-group name should be proposed for this tribe.

***Aculagnathus* Oke, 1932**: 22. Current status: valid genus in CERYLONIDAE: CERYLONINAE.

Type species: *Aculagnathusmirabilis* Oke, 1932, by monotypy.

Family-group name: ACULAGNATHIDAE Oke, 1932. Stem: *Aculagnath*-.

***Acupalpus* Latreille, 1829a**: 391. Current status: valid genus in CARABIDAE: HARPALINAE: HARPALINI: STENOLOPHINA.

Type species: *Carabusmeridianus* Linnaeus, 1761, by subsequent designation (Westwood 1840b: 498).

Family-group name: ACUPALPINI Tschitschérine, 1900. Stem: *Acupalp*-.

***Acylophorus* Nordmann, 1837**: 5, 127. Current status: valid genus in STAPHYLINIDAE: STAPHYLININAE: STAPHYLININI: ACYLOPHORINA.

Type species: *Acylophorusahrensii* Nordmann, 1837 (= *Staphylinusglaberrimus* Herbst, 1784), by subsequent designation (Blackwelder 1943: 466).

Family-group name: ACYLOPHORINI Outerelo & Gamarra, 1986. Stem: *Acylophor*-.

***Adalia* Mulsant, 1846**: [2 (Addenda et errata)]. Current status: valid genus in COCCINELLIDAE: COCCINELLINAE: COCCINELLINI. Note: new replacement name for *Idalia* Mulsant, 1846.

Type species: *Coccinellabipunctata* Linnaeus, 1758, by subsequent designation (Crotch 1874a: 99).

Family-group name: ADALIINA Hatch, 1962: 173. Stem: *Adali*-. Note: this name was overlooked in Bouchard et al. (2011) and Bouchard and Bousquet (2020a).

***Adelaidia* Blackburn, 1891**: 130. Current status: valid genus in DERMESTIDAE: ATTAGENINAE: ADELAIDIINI.

Type species: *Adelaidiarigua* Blackburn, 1891, by monotypy.

Family-group name: ADELAIDIINA Háva, 2015: 1. Stem: *Adelaidi*-.

***Adelina* Dejean, 1835**: 315. Current status: valid genus in TENEBRIONIDAE: DIAPERINAE: DIAPERINI: ADELININA.

Type species: *Cucujusplanus* Fabricius, 1801, by monotypy.

Family-group name: ADELININI J.L. LeConte, 1862. Stem: *Adelin*-.

***Adelium* W. Kirby, 1819**: 420. Current status: valid genus in TENEBRIONIDAE: LAGRIINAE: ADELIINI.

Type species: *Adeliumcalosomoides* W. Kirby, 1819, by subsequent designation (J. Meyer 1844: 180).

Family-group name: ADELIADAE W. Kirby, 1828. Stem: *Adeli*-. Note: senior homonym of ADELIINI Viereck, 1918 (type genus *Adelius* Haliday, 1833) in Hymenoptera.

***Adelocera* Latreille, 1829a**: 451. Current status: valid genus in ELATERIDAE: AGRYPNINAE: AGRYPNINI.

Type species [suggested]: *Elaterovalis* Germar, 1823, by subsequent designation (Hyslop 1921: 623). Note: the valid type species fixation is that of Duponchel (1840: 120), who selected *Elaterfuscus* Fabricius, 1801, but that species is currently included in the genus *Agrypnus* Eschscholtz, 1829 (see Kundrata et al. 2019c: 88); an application to the Commission is necessary to fix *Elaterovalis* Germar, 1823 as the type species of *Adelocera* Latreille, 1829.

Family-group name: ADELOCERAEIDAE Gistel, 1848. Stem: *Adelocer*-. Note: nomen oblitum (see Bouchard et al. 2011: 307).

***Adelostoma* Duponchel, 1827**: 342. Current status: valid genus in TENEBRIONIDAE: PIMELIINAE: ADELOSTOMINI.

Type species: *Adelostomasulcatum* Duponchel, 1827, by monotypy.

Family-group name: ADÉLOSTOMITES Solier, 1834. Stem: *Adelostom*-.

†***Ademosyne* Handlirsch, 1906**: 402. Current status: valid genus in †ADEMOSYNIDAE. Note: Triassic (Argentina; Australia; Austria; Chile; South Africa).

Type species: †*Ademosynemajor* Handlirsch, 1906, by subsequent designation (Tillyard 1916: 17).

Family-group name: †ADEMOSYNIDAE Ponomarenko, 1968. Stem: *Ademosyn*-.

***Aderpas* J. Thomson, 1864**: 62. Current status: valid genus in CERAMBYCIDAE: LAMIINAE: ADERPASINI.

Type species: *Crossotusgriseus* J. Thomson, 1858, by original designation.

Family-group name: ADERPASINI Breuning & Téocchi, 1978. Stem: *Aderpas*-.

***Aderus* Stephens, 1829b**: 20. Current status: valid genus in ADERIDAE. Note: name placed on the Official List of Generic Names in Zoology (ICZN 1989d; original publication incorrectly given as Stephens 1829c: 255).

Type species: *Lyttaboleti* Marsham, 1802 (= *Notoxuspopulneus* Creutzer, 1796), by subsequent designation (Westwood 1830a: 58; see ICZN 1989d). Note: *Lyttaboleti* Marsham, 1802 was placed on the Official List of Specific Names in Zoology (ICZN 1989d).

Family-group name: ADERIDAE Csiki, 1909. Stem: *Ader*-.

***Adesmia* Fischer, 1822**: 153. Current status: valid genus in TENEBRIONIDAE: PIMELIINAE: ADESMIINI.

Type species: *Adesmialongipes* Fischer, 1822 (= *Pimeliaanomala* Fischer, 1820), by monotypy.

Family-group name: ADESMIIDES Lacordaire, 1859. Stem: *Adesmi*-. Note: nomen protectum (see Bouchard et al. 2007: 386, 2011: 861).

***Adetus* J.L. LeConte, 1852a**: 161. Current status: valid genus in CERAMBYCIDAE: LAMIINAE: APOMECYNINI.

Type species: *Polyopsiaanalis* Melsheimer, 1847, by monotypy.

Family-group name: ADÉTIDES Lacordaire, 1872. Stem: *Adet*-.

***Adimerus* Sharp, 1894**: 441. Current status: junior synonym of *Monoedus* G.H. Horn, 1882 in ZOPHERIDAE: COLYDIINAE: ADIMERINI.

Type species: *Adimeruscrispatus* Sharp, 1894, by subsequent designation (Ivie and Ślipiński 1990: 15).

Family-group name: ADIMERIDAE Sharp, 1894. Stem: *Adimer*-.

***Adinopsis* Cameron, 1919**: 242. Current status: valid genus in STAPHYLINIDAE: ALEOCHARINAE: GYMNUSINI.

Type species: *Adinopsisrufobrunnea* Cameron, 1919, by monotypy.

Family-group name: ADINOPSINI Cameron, 1919. Stem: *Adinopse*-.

***Adoretus* Dejean, 1833b**: 157. Current status: valid genus in SCARABAEIDAE: RUTELINAE: ADORETINI: ADORETINA.

Type species: *Melolonthanigrifrons* Steven, 1809, by subsequent designation (S.I. Medvedev 1949: 313).

Family-group name: ADORETIDAE Burmeister, 1844. Stem: *Adoret*-.

***Adorium* Fabricius, 1801a**: xx, 409. Current status: junior synonym of *Oides* Weber, 1801 in CHRYSOMELIDAE: GALERUCINAE: OIDINI.

Type species: *Chrysomelabipunctata* Fabricius, 1781 (= *Oidesandrewesi* Jacoby, 1900), by subsequent designation (Latreille 1810: 432).

Family-group name: ADORIITES Chapuis, 1875. Stem: *Adori*-.

***Adorodocia* Brenske, 1893**: 1. Current status: valid genus in SCARABAEIDAE: RUTELINAE: ADORETINI: ADORETINA.

Type species: *Adorodociamaxima* Brenske, 1893 (= *Adoretusvittaticollis* Fairmaire, 1883), by subsequent designation (Arrow 1901: 36).

Family-group name: ADORODOCIINA Ohaus, 1912. Stem: *Adorodoci*-.

***Adoroleptus* Brenske, 1893**: 1. Current status: valid genus in SCARABAEIDAE: RUTELINAE: ADORETINI: ADORETINA.

Type species: *Melolonthalanata* Fabricius, 1801, by monotypy.

Family-group name: ADOROLEPTINA Ohaus, 1912. Stem: *Adorolept*-.

***Adorrhinyptia* Arrow, 1917**: 273. Current status: valid genus in SCARABAEIDAE: RUTELINAE: ADORETINI: ADORRHINYPTIINA.

Type species: *Rhinyptiadorsalis* Burmeister, 1855, by original designation.

Family-group name: ADORRHINYPTIINI Arrow, 1917. Stem: *Adorrhinypti*-.

***Adoxus* W. Kirby, 1837**: 209. Current status: junior synonym of *Bromius* Chevrolat, 1836 in CHRYSOMELIDAE: EUMOLPINAE: BROMIINI.

Type species: *Cryptocephalusvitis* Fabricius, 1775 (= *Chrysomelaobscura* Linnaeus, 1758), by original designation.

Family-group name: ADOXINAE Baly, 1863. Stem: *Adox*-.

***Adranes* J.L. LeConte, 1849**: 83. Current status: valid genus in STAPHYLINIDAE: PSELAPHINAE: CLAVIGERITAE: CLAVIGERINI.

Type species: *Adranescoecus* J.L. LeConte, 1849, by monotypy.

Family-group name: ADRANITES E. Desmarest, 1852. Stem: *Adran*-. Note: senior homonym of ADRANIDAE Scarlato & Starobogatov, 1985 (type genus *Adrana* H. Adams & A. Adams, 1858) in Mollusca.

***Adrastus* Eschscholtz, 1829a**: 35. Current status: valid genus in ELATERIDAE: ELATERINAE: SYNAPTINI.

Type species: *Elaterlimbatus* Fabricius, 1777, by monotypy.

Family-group name: ADRASTITES Candèze, 1863. Stem: *Adrast*-.

***Adzusa* Kishii, 1957**: 10. Current status: valid genus in ELATERIDAE incertae sedis.

Type species: *Adzusainexpecta* Kishii, 1957, by monotypy.

Family-group name: ADZUSINI Kishii, 1989. Stem: *Adzus*-.

***Aedemonus* Schönherr, 1837**: 243. Current status: valid genus in CURCULIONIDAE: CRYPTORHYNCHINAE: AEDEMONINI.

Type species: *Aedemonuseminentepunctatus* Boheman, 1837, by original designation.

Family-group name: ACDEMONINI Faust, 1898. Stem: *Aedemon*-.

***Aedilis* Audinet-Serville, 1835a**: 32. Current status: junior synonym of *Acanthocinus* Dejean, 1821 in CERAMBYCIDAE: LAMIINAE: ACANTHOCININI.

Type species: *Aedilismontana* Audinet-Serville, 1835 (= *Cerambyxaedilis* Linneaus, 1758), by subsequent designation (Westwood 1838c: 40).

Family-group name: AEDILINAE Perrier, 1893. Stem: *Aedil*-.

***Aegialia* Latreille, 1806**: 96. Current status: valid genus in SCARABAEIDAE: AEGIALIINAE: AEGIALIINI.

Type species: *Scarabaeusglobosus* Kugelann, 1794 (= *Scarabaeusarenarius* Fabricius, 1787), by monotypy. Note: *Scarabaeusarenarius* Fabricius, 1787 was placed on the Official List of Specific Names in Zoology (ICZN 2006c).

Family-group name: AEGIALITES Laporte, 1840. Stem: *Aegiali*-.

***Aegialites* Mannerheim, 1853**: 178. Current status: valid genus in SALPINGIDAE: AEGIALITINAE.

Type species: *Aegialitesdebilis* Mannerheim, 1853, by monotypy.

Family-group name: AEGIALITIDAE J.L. LeConte, 1862. Stem: *Aegialit*-.

***Aegidinus* Arrow, 1904**: 739. Current status: valid genus in SCARABAEIDAE: ORPHNINAE: AEGIDIINI: AEGIDININA.

Type species: *Aegidiumguianense* Westwood, 1846, by subsequent designation (Paulian 1984: 87).

Family-group name: AEGIDININA Frolov, Akhmetova & Neita-Moreno, 2023: 37. Stem: *Aegidin*-.

***Aegidium* Westwood, 1845a**: 439, 440. Current status: valid genus in SCARABAEIDAE: ORPHNINAE: AEGIDIINI: AEGIDIINA.

Type species: *Aegidiumcolumbianum* Westwood, 1845, by subsequent designation (Paulian 1984: 70).

Family-group name: AEGIDIINAE Paulian, 1984. Stem: *Aegidi*-.

***Aegoprepes* Pascoe, 1871c**: 277. Current status: valid genus in CERAMBYCIDAE: LAMIINAE: AGAPANTHIINI.

Type species: *Aegoprepesantennator* Pascoe, 1871, by monotypy.

Family-group name: AEGOPREPINAE Pascoe, 1871. Stem: *Aegoprep*-.

***Aegosoma* Audinet-Serville, 1832**: 162. Current status: valid genus in CERAMBYCIDAE: PRIONINAE: AEGOSOMATINI.

Type species: *Cerambyxscabricornis* Scopoli, 1763, by monotypy.

Family-group name: AEGOSOMITAE J. Thomson, 1861. Stem: *Aegosomat*-.

***Aegostheta* Dejean, 1833b**: 159. Current status: valid genus in SCARABAEIDAE: MELOLONTHINAE: TANYPROCTINI: MACROPHYLLINA.

Type species: *Melolonthalongicornis* Fabricius, 1787, by monotypy.

Family-group name: AEGOSTHETINI Lacroix, 2007. Stem: *Aegosthet*-.

***Aegus* W.S. MacLeay, 1819**: 112. Current status: valid genus in LUCANIDAE: LUCANINAE: AEGINI.

Type species: *Aeguschelifer* W.S. MacLeay, 1819, by subsequent designation (Arrow 1950: 174).

Family-group name: AEGINI H. Huang & C.H. Chen, 2013: 68. Stem: *Aeg*-.

***Aenictobia* Seevers, 1953**: 127. Current status: valid genus in STAPHYLINIDAE: ALEOCHARINAE: AENICTOTERATINI.

Type species: *Aenictobialongicornis* Seevers, 1953, by original designation.

Family-group name: AENICTOBIINA Kistner, 1997. Stem: *Aenictobi*-.

***Aenictoteras* W.M. Wheeler, 1932**: 301. Current status: valid genus in STAPHYLINIDAE: ALEOCHARINAE: AENICTOTERATINI.

Type species: *Aenictoteraschapmani* W.M. Wheeler, 1932, by original designation.

Family-group name: AENICTOTERATINI Kistner, 1993. Stem: *Aenictoterat*-.

***Aenigmaticum* A. Matthews, 1888**: 104. Current status: valid genus in CORYLOPHIDAE: CORYLOPHINAE: AENIGMATICINI.

Type species: *Aenigmaticumptilioides* A. Matthews, 1888, by monotypy.

Family-group name: AENIGMATICINI Casey, 1900. Stem: *Aenigmatic*-.

***Aephnidius* W.S. MacLeay, 1825**: 23. Current status: valid subgenus of *Anaulacus* W.S. MacLeay, 1825 in CARABIDAE: HARPALINAE: MASOREINI.

Type species: *Anaulacusadelioides* W.S. MacLeay, 1825, by monotypy.

Family-group name: AEPHNIDIINA Jakobson, 1907. Stem: *Aephnidi*-.

***Aepus* Leach in Samouelle, 1819**: 149. Current status: valid genus in CARABIDAE: TRECHINAE: TRECHINI: TRECHINA. Note: this name is often attributed to Samouelle; however, in Opinion 1722 (ICZN 1993b) Leach was accepted as the author of the new genera attributed to him in Samouelle.

Type species: *Aepusfulvescens* Samouelle, 1819 (= *Cicindelamarina* Strøm, 1783), by monotypy.

Family-group name: AËPYES Fowler, 1887. Stem: *Aep*-.

***Aepyceratus* Poinar, A.E. Brown & Legalov, 2017**: 76. Current status: valid genus in MESOPHYLETIDAE: AEPYCERATINAE.

Type species: *Aepyceratushyperochus* Poinar, A.E. Brown & Legalov, 2017, by original designation.

Family-group name: AEPYCERATINAE Poinar, A.E. Bown & Legalov, 2017: 75. Stem: *Aepycerat*-.

***Aerenea* J. Thomson, 1857**: 298. Current status: valid genus in CERAMBYCIDAE: LAMIINAE: COMPSOSOMATINI. Note: the original spelling was *Aerenaea* but *Aerenea*, an incorrect subsequent spelling, is in prevailing usage, attributed to the publication of the original spelling, and therefore deemed to be the correct original spelling (ICZN 1999a, Article 33.3.1).

Type species: *Aereneaposticalis* J. Thomson, 1857, by monotypy.

Family-group name: AERENEITES J. Thomson, 1868. Stem: *Aerene*-.

***Aerenica* Dejean, 1835**: 352. Current status: valid genus in CERAMBYCIDAE: LAMIINAE: AERENICINI.

Type species: *Saperdacanescens* Klug, 1825, by monotypy.

Family-group name: AERÉNICIDES Lacordaire, 1872. Stem: *Aerenic*-.

***Aesalus* Fabricius, 1801a**: xii. Current status: valid genus in LUCANIDAE: AESALINAE: AESALINI.

Type species: *Lucanusscarabaeoides* Panzer, 1795, by subsequent monotypy (Fabricius 1801b: 254).

Family-group name: AESALIDAE W.S. MacLeay, 1819. Stem: *Aesal*-.

***Aferos* Kazantsev, 1992**: 44. Current status: valid genus in LYCIDAE: EROTINAE: SLIPINSKIINI.

Type species: *Stadenusaethiops* Kleine, 1933, by original designation.

Family-group name: AFEROTINI Kazantsev, 2004. Stem: *Aferot*-.

***Afremus* Levey, 1985**: 420. Current status: valid genus in TENEBRIONOIDEA incertae sedis.

Type species: *Afremuspickeri* Levey, 1985, by original designation.

Family-group name: AFREMINAE Levey, 1985. Stem: *Afrem*-.

***Afroapoderus* Legalov, 2003**: 483. Current status: valid genus in ATTELABIDAE: APODERINAE: HOPLAPODERINI: AFROAPODERINA.

Type species: *Apoderusseminiger* Faust, 1894, by original designation.

Family-group name: AFROAPODERINA Legalov, 2003. Stem: *Afroapoder*-.

***Afrocorynus* G.A.K. Marshall, 1955a**: 21. Current status: valid genus in BELIDAE: OXYCORYNINAE: OXYCORYNITAE: AFROCORYNINI.

Type species: *Afrocorynusturbatus* G.A.K. Marshall, 1955, by original designation.

Family-group name: AFROCORYNINI Voss, 1957. Stem: *Afrocoryn*-.

***Afroeubria* Villiers, 1961**: 439. Current status: valid genus in PSEPHENIDAE: AFROEUBRIINAE.

Type species: *Afroeubriahydropetrica* Villiers, 1961, by original designation.

Family-group name: AFROEUBRIINAE Lee, Satô, Shepard & Jäch, 2007. Stem: *Afroeubri*-.

***Afroquedius* Solodovnikov, 2006**: 1075. Current status: valid genus in STAPHYLINIDAE: STAPHYLININAE: STAPHYLININI: AFROQUEDIINA.

Type species: *Quediussexpunctatus* Bernhauer, 1917, by original designation.

Family-group name: AFROQUEDIINA Brunke, Żyła & Solodovnikov in Brunke et al., 2019: 179. Stem: *Afroquedi*-.

***Agabetes* Crotch, 1873d**: 398, 401. Current status: valid genus in DYTISCIDAE: LACCOPHILINAE: AGABETINI.

Type species: *Colymbetesacuductus* Harris, 1828, by original designation.

Family-group name: AGABETINI Branden, 1884. Stem: *Agabet*-.

***Agabinus* Crotch, 1873d**: 397. Current status: junior synonym of *Platambus* C.G. Thomson, 1859 in DYTISCIDAE: AGABINAE: AGABINI.

Type species: *Colymbetesglabrellus* Motschulsky, 1859, by original designation.

Family-group name: AGABININI Crotch, 1873. Stem: *Agabin*-.

***Agabus* Leach, 1817**: 72. Current status: valid genus in DYTISCIDAE: AGABINAE: AGABINI.

Type species: *Agabuspaykullii* Leach, 1817 (= *Dytiscusserricornis* Paykull, 1799), by monotypy.

Family-group name: AGABIDES C.G. Thomson, 1867. Stem: *Agab*-.

***Agaeocera* Saunders, 1871**: 50. Current status: valid genus in BUPRESTIDAE: BUPRESTINAE: BUPRESTINI: AGAEOCERINA.

Type species: *Anthaxiagigas* Gory & Laporte, 1839, by monotypy.

Family-group name: AGAEOCERINI Nelson, 1982. Stem: *Agaeocer*-.

***Agallissus* Dalman, 1823**: 66. Current status: valid genus in CERAMBYCIDAE: CERAMBYCINAE: AGALLISSINI.

Type species: *Agallissusmelaniodes* Dalman, 1823, by monotypy.

Family-group name: AGALLISSINI J.L. LeConte, 1873. Stem: *Agalliss*-.

***Agaocephala* Lepeletier & Audinet-Serville, 1828**: 370. Current status: valid genus in SCARABAEIDAE: DYNASTINAE: AGAOCEPHALINI. Note: the original spelling was *Agacephala*, but *Agaocephala*, an incorrect subsequent spelling, is in prevailing usage, attributed to the publication of the original spelling, and therefore deemed to be the correct original spelling (ICZN 1999a, Article 33.3.1).

Type species: *Agaocephalacornigera* Lepeletier & Audinet-Serville, 1828, by monotypy.

Family-group name: AGAOCEPHALIDAE Burmeister, 1847. Stem: *Agaocephal*-.

***Agapanthia* Audinet-Serville, 1835a**: 35. Current status: valid genus in CERAMBYCIDAE: LAMIINAE: AGAPANTHIINI.

Type species: *Cerambyxcardui* Linnaeus, 1767, by subsequent designation (Westwood 1838c: 41).

Family-group name: AGAPANTHAIRES Mulsant, 1839. Stem: *Agapanthi*-.

***Agapytho* Broun, 1921**: 542. Current status: valid genus in AGAPYTHIDAE.

Type species: *Agapythofoveicollis* Broun, 1921, by monotypy.

Family-group name: AGAPYTHINAE Sen Gupta & Crowson, 1969. Stem: *Agapyth*-.

***Agathidium* Panzer, 1796**: no 13. Current status: valid genus in LEIODIDAE: LEIODINAE: ANISOTOMINI. Note: nomen protectum (see Wheeler and Miller 2005: 34).

Type species: *Tetratomaglobosa* Herbst, 1791 (= *Silphaseminulum* Linnaeus, 1758), by monotypy.

Family-group name: AGATHIDIIDAE Westwood, 1838. Stem: *Agathidi*-.

***Agelaea* Gené, 1839**: 49. Current status: valid genus in CARABIDAE: HARPALINAE: PLATYNINI.

Type species: *Agelaeafulva* Gené, 1839, by monotypy.

Family-group name: AGELAEINA Jakobson, 1907. Stem: *Agelae*-.

***Agelastica* Chevrolat in Dejean, 1836a**: 381. Current status: valid genus in CHRYSOMELIDAE: GALERUCINAE: HYLASPINI.

Type species: *Chrysomelaalni* Linnaeus, 1758, by monotypy.

Family-group name: AGELASTICITES Chapuis, 1875. Stem: *Agelastic*-.

***Aglenus* Erichson, 1845a**: 285. Current status: valid genus in SALPINGIDAE: AGLENINAE.

Type species: *Hypophlaeusbrunneus* Gyllenhal, 1813, by monotypy.

Family-group name: AGLENI G.H. Horn, 1878. Stem: *Aglen*-.

***Aglycyderes* Westwood, 1864a**: 179. Current status: valid genus in BELIDAE: OXYCORYNINAE: AGLYCYDERITAE: AGLYCYDERINI.

Type species: *Aglycyderessetifer* Westwood, 1864, by monotypy.

Family-group name: AGLYCYDERIDAE Wollaston, 1864. Stem: *Aglycyder*-.

***Aglymbus* Sharp, 1880**: cxlix. Current status: valid genus in DYTISCIDAE: COPELATINAE.

Type species: *Agabusleprieurii* Aubé, 1838, by monotypy.

Family-group name: AGLYMBINI Branden, 1884. Stem: *Aglymb*-.

***Agnathus* Germar, 1818**: 232. Current status: valid genus in PYROCHROIDAE: AGNATHINAE. Note: this name, which is usually credited to “Germar, 1825”, was first published in an available work as a junior synonym and subsequently used as valid before 1961 by several authors, including in LaFerté-Sénectère (1849: 293), *Agnathus* was therefore made available from its first publication as a synonym (ICZN 1999a, Article 11.6.1).

Type species: *Notoxusdecoratus* Germar, 1818, by monotypy.

Family-group name: AGNATHIDES Lacordaire, 1859. Stem: *Agnath*-.

***Agnesiotis* Pascoe, 1870**: 474. Current status: valid genus in BELIDAE: BELINAE: PACHYURITAE: AGNESIOTIDINI.

Type species: *Agnesiotispilosula* Pascoe, 1870, by monotypy.

Family-group name: AGNESIOTIDINI Zimmerman, 1994. Stem: *Agnesiotid*-.

***Agnia* Newman, 1842a**: 291. Current status: valid genus in CERAMBYCIDAE: LAMIINAE: LAMIINI.

Type species: *Agniacasta* Newman, 1842, by subsequent designation (J. Thomson 1864: 86).

Family-group name: AGNITAE J. Thomson, 1864. Stem: *Agni*-.

***Agonica* Sloane, 1920**: 174. Current status: valid genus in CARABIDAE: HARPALINAE: PELECIINI: AGONICINA.

Type species: *Agonicasimsoni* Sloane, 1920, by subsequent designation (B.P. Moore 1963a: 22).

Family-group name: AGONICINI Sloane, 1920. Stem: *Agonic*-.

***Agonum* Bonelli, 1810**: Tab. Syn. Current status: valid genus in CARABIDAE: HARPALINAE: PLATYNINI. Note: name placed on the Official List of Generic Names in Zoology (ICZN 1996d).

Type species: *Carabusmarginatus* Linnaeus, 1758, by subsequent designation (Curtis 1827: pl. 183; see ICZN 1996d). Note: no species were originally included in this genus by Bonelli (1810); the first ones were the 12 species listed by Panzer (1813: 52–54); name placed on the Official List of Specific Names in Zoology (ICZN 1996d).

Family-group name: AGONIDAE W. Kirby, 1837. Stem: *Agonum*- (ICZN 1996d).

***Agra* Fabricius, 1801a**: xxiii, 224. Current status: valid genus in CARABIDAE: HARPALINAE: LEBIINI: AGRINA.

Type species: *Agraaenea* Fabricius, 1801 (= *Carabuscajennensis* G.-A. Olivier, 1795), by subsequent designation (Latreille 1810: 426).

Family-group name: AGRIDAE W. Kirby, 1837. Stem: *Agr*-.

***Agraphus* Say, 1832**: 13. Current status: valid genus in CURCULIONIDAE: ENTIMINAE: OTIORHYNCHITAE: AGRAPHINI. Note: see Prena (2018: 331) for notes on the authorship of this genus-group name.

Type species: *Peritelusbellicus* Say, 1832, by monotypy.

Family-group name: AGRAPHI G.H. Horn, 1876. Stem: *Agraph*-.

***Agrilus* Curtis, 1825**: pl. 67. Current status: valid genus in BUPRESTIDAE: AGRILINAE: AGRILINI: AGRILINA.

Type species: *Buprestisviridis* Linnaeus, 1758, by original designation.

Family-group name: AGRILIDAE Laporte, 1835. Stem: *Agril*-.

***Agriotes* Eschscholtz, 1829a**: 34. Current status: valid genus in ELATERIDAE: ELATERINAE: AGRIOTINI: AGRIOTINA.

Type species: *Elatersputator* Linnaeus, 1758, by subsequent designation (Curtis 1838a: pl. 694).

Family-group name: AGRIOTITES Laporte, 1840. Stem: *Agriot*-.

***Agrodes* Nordmann, 1837**: 7, 161. Current status: junior synonym of *Plochionocerus* Dejean, 1833 in STAPHYLINIDAE: STAPHYLININAE: XANTHOLININI. Note: name placed on the Official List of Generic Names in Zoology (ICZN 1996c).

Type species: *Agrodeselegans* Nordmann, 1837, by monotypy (see ICZN 1996c). Note: name placed on the Official List of Specific Names in Zoology (ICZN 1996c).

Family-group name: AGRAEFORMES Nordmann, 1837. Stem: *Agrod*-. Note: name placed on the Official List of Family-Group Names in Zoology (ICZN 1996c, as AGRODINI Nordmann, 1837); younger name XANTHOLININI Erichson, 1839 given precedence over this name (ICZN 1996c).

***Agronoma* Gistel, 1848a**: [2]. Current status: junior synonym of *Amara* Bonelli, 1810 in CARABIDAE: HARPALINAE: ZABRINI: AMARINA. Note: junior homonym of *Agronoma* Hübner, 1821 [Lepidoptera].

Type species: *Carabusfamiliaris* Duftschmid, 1812, by subsequent designation (Bouchard et al. 2011: 145).

Family-group name: AGRONOMAEIDAE Gistel, 1848. Stem: *Agronom*-. Note: family-group name permanently invalid (ICZN 1999a, Article 39).

***Agrypnus* Eschscholtz, 1829a**: 32. Current status: valid genus in ELATERIDAE: AGRYPNINAE: AGRYPNINI.

Type species: *Elatermurinus* Linnaeus, 1758, by subsequent designation (Westwood 1838c: 26).

Family-group name: AGRYPNIDES Candèze, 1857. Stem: *Agrypn*-. Note: nomen protectum (see Bouchard et al. 2011: 307).

***Agyrtes* Frölich, 1799**: 15. Current status: valid genus in AGYRTIDAE: AGYRTINAE.

Type species: *Mycetophaguscastaneus* Fabricius, 1792, by subsequent designation (Latreille 1810: 427).

Family-group name: AGYRTIDAE C.G. Thomson, 1859. Stem: *Agyrt*-.

***Agyrtodes* Portevin, 1907**: 75. Current status: valid genus in LEIODIDAE: CAMIARINAE: AGYRTODINI.

Type species: *Agyrtodesovatus* Portevin, 1907, by monotypy.

Family-group name: AGYRTODINI Jeannel, 1936. Stem: *Agyrtod*-.

***Akalyptoischion* Andrews, 1976**: 3. Current status: valid genus in AKALYPTOISCHIIDAE.

Type species: *Akalyptoischiontomeus* Andrews, 1976, by original designation.

Family-group name: AKALYPTOISCHIIDAE Lord, Hartley, Lawrence, McHugh, Whiting & K.B. Miller, 2010. Stem: *Akalyptoischi*-.

***Akatastopsis* Pace, 2000a**: 112. Current status: valid genus in STAPHYLINIDAE: ALEOCHARINAE: AKATASTOPSISINI.

Type species: *Akatastopsispapuana* Pace, 2000, by original designation.

Family-group name: AKATASTOPSISINI Pace, 2000. Stem: *Akatastopsis*- (ICZN 1999a, Article 29.4).

***Akis* Herbst, 1799**: 124. Current status: valid genus in TENEBRIONIDAE: PIMELIINAE: AKIDINI.

Type species: *Pimeliareflexa* Fabricius, 1775, by subsequent designation (Latreille 1810: 429).

Family-group name: ACIDES Billberg, 1820. Stem: *Akid*-.

***Alampes* Champion, 1896**: 463. Current status: invalid senior synonym of *Peralampes* P.J. Johnson, 2002 in ELATERIDAE: AGRYPNINAE: ANAISSINI. Note: this genus was first made available on page 463, issued on January 1896, by the association of two available species, *Pyrophorusmelanoxanthus* Candèze, 1865 and *Pyrophorusabnormis* Candèze, 1863; the genus was described on page 474, issued on March 1896, with two species described, including *Alampesvestitus* Champion, 1896; the type species must be one of the species mentioned in January 1896 section of the publication; junior homonym of *Alampes* Horvath, 1884 [Hemiptera].

Type species: *Pyrophorusmelanoxanthus* Candèze, 1865, by **present designation**. Note: the type species recognized in the literature, *Alampesvestitus* Champion, 1896, by subsequent designation (Hyslop 1921: 624), is not an originally available species as noted above.

Family-group name: ALAMPINA C. Costa, 1975. Stem: *Alamp*-. Note: family-group name permanently invalid (ICZN 1999a, Article 39).

***Alanizus* Di Iorio, 2003**: 1. Current status: valid genus in CERAMBYCIDAE: CERAMBYCINAE: ALANIZINI.

Type species: *Alanizustortuosus* Di Iorio, 2003, by original designation.

Family-group name: ALANIZINI Di Iorio, 2003. Stem: *Alaniz*-.

***Alaudes* G.H. Horn, 1870**: 361. Current status: valid genus in TENEBRIONIDAE: PIMELIINAE: CNEMEPLATIINI: ALAUDINA.

Type species: *Alaudessingularis* G.H. Horn, 1870, by monotypy.

Family-group name: ALAUDINA Aalbu, Catarino & A.D. Smith, 2018: 250. Stem: *Alaud*-. Note: junior homonym of ALAUDIDAE Vigors, 1825 (type genus *Alauda* Linnaeus, 1758) in Aves.

***Alaus* Eschscholtz, 1829a**: 33. Current status: valid genus in ELATERIDAE: AGRYPNINAE: HEMIRHIPINI: HEMIRHIPINA.

Type species: *Elateroculatus* Linnaeus, 1758, by subsequent designation (Duponchel 1840: 242).

Family-group name: ALAITES Candèze, 1874. Stem: *Ala*-.

***Alceis* Billberg, 1820**: 45. Current status: valid genus in CURCULIONIDAE: ENTIMINAE: POLYDRUSITAE: NAUPACTINI: NAUPACTINA.

Type species: *Curculiolongimanus* Fabricius, 1775 (= *Siderodactylusornatus* Pascoe, 1879), by monotypy.

Family-group name: ALCEIDINI Pierce, 1913. Stem: *Alceent*-.

***Alcides* C.R. Sahlberg, 1823b**: 47. Current status: invalid senior synonym of *Alcidodes* G.A.K. Marshall, 1939 in CURCULIONIDAE: MOLYTINAE: MECYSOLOBINI. Note: junior homonym of *Alcides* Hübner, 1822 [Lepidoptera].

Type species: *Alcidessenex* C.R. Sahlberg, 1823, by monotypy.

Family-group name: ALCIDIDES Jekel, 1865. Stem: *Alcid*-. Note: family-group name permanently invalid (ICZN 1999a, Article 39).

***Alcidodes* G.A.K. Marshall, 1939a**: 582. Current status: valid genus in CURCULIONIDAE: MOLYTINAE: MECYSOLOBINI. Note: new replacement name for *Alcides* Schönherr, 1823.

Type species: *Alcidessenex* C.R. Sahlberg, 1823, by monotypy (type fixation for *Alcides* Schönherr).

Family-group name: ALCIDODINAE G.A.K. Marshall, 1939. Stem: *Alcidod*-.

***Alegoria* Laporte, 1840b**: 221. Current status: valid genus in TENEBRIONIDAE: TENEBRIONINAE: ULOMINI.

Type species: *Alegoriadilatata* Laporte, 1840, by monotypy.

Family-group name: ALÉGORIIDES Lacordaire, 1859. Stem: *Alegori*-.

***Aleochara* Gravenhorst, 1802**: 67. Current status: valid genus in STAPHYLINIDAE: ALEOCHARINAE: ALEOCHARINI: ALEOCHARINA.

Type species: *Staphylinuscurtulus* Goeze, 1777, misidentified as *Staphylinusfuscipes* Linnaeus, 1758 (sensu Gravenhort, 1802) in the subsequent designation by Samouelle (1819: 177). Note: we follow Smetana (2004: 30) in using Article 69.2.4 (ICZN 1999a) to determine the identity of the type species for this genus; Goeze’s (1777) work is not consistently binominal (see ICZN 2021a) and therefore an application to the Commission is necessary to retain *Staphylinuscurtulus* Goeze, 1777 as the valid name for the type species.

Family-group name: ALEOCHARIDAE Fleming, 1821. Stem: *Aleochar*-.

***Alesia* Reiche, 1848**: pl. 26. Current status: junior synonym of *Micraspis* Chevrolat, 1836 in COCCINELLIDAE: COCCINELLINAE: COCCINELLINI. Note: this name is usually credited to Mulsant (1850: 343) in the literature but Reiche made it available earlier by the illustration of *annulata* on plate 26 of Ferret and Galinier’s ‘Voyage en Abyssinie’.

Type species: *Alesiaannulata* Reiche, 1848, by monotypy.

Family-group name: ALESIAIRES Mulsant, 1850. Stem: *Alesi*-.

***Alexia* Stephens, 1833a**: 23. Current status: junior synonym of *Sphaerosoma* Stephens, 1832 in ALEXIIDAE.

Type species: *Tritomapiliferum* P.W.J. Müller, 1821, by monotypy. Note: this species is usually listed as a junior synonym of *Sphaerosomaquercus* Samouelle, 1819 but *S.quercus* is a nomen nudum in Samouelle (1819: 394); the species was described by Stephens only in 1832.

Family-group name: ALEXIADAE Imhoff, 1856. Stem: *Alexi*-.

***Algon* Sharp, 1874a**: 22. Current status: valid genus in STAPHYLINIDAE: STAPHYLININAE: STAPHYLININI: ALGONINA.

Type species: *Algongrandicollis* Sharp, 1874, by monotypy.

Family-group name: ALGONINA Schillhammer & Brunke in Chani-Posse et al., 2018: 30. Stem: *Algon*- (ICZN 1999a, Article 29.4).

†***Alitrepanum* Peng, Jiang, Engel & Wang in Peng et al., 2022**: 2. Current status: valid genus in BOSTRICHIDAE: †ALITREPANINAE. Note: mid-Cretaceous (Myanmar).

Type species: †*Alitrepanumaladelicatum* Peng, Jiang, Engel & Wang, 2022, by original designation.

Family-group name: †ALITREPANINI Peng, Jiang, Engel & Wang in Peng et al., 2022: 2. Stem: Alitrepan-.

***Allandrus* J.L. LeConte in J.L. LeConte and G.H. Horn, 1876**: 396. Current status: valid genus in ANTHRIBIDAE: ANTHRIBINAE: ALLANDRINI.

Type species: *Allandrusbifasciatus* J.L. LeConte, 1876, by monotypy.

Family-group name: ALLANDRINI Pierce, 1930. Stem: *Allandr*-.

***Allapoderus* Voss, 1927**: 15. Current status: valid genus in ATTELABIDAE: APODERINAE: CLITOSTYLINI: ALLAPODERINA.

Type species: *Apoderusdentipes* Faust, 1883, by subsequent designation (Legalov 2003: 468).

Family-group name: ALLAPODERINA Legalov, 2003. Stem: *Allapoder*-.

***Allara*Britton, 1955**: 124. Current status: valid genus in SCARABAEIDAE: SERICOIDINAE: LIPARETRINI.

Type species: *Allarainsularis*Britton, 1955, by original designation.

Family-group name: ALLARINIBritton, 1955. Stem: *Allar*-.

***Allecula* Fabricius, 1801b**: 21. Current status: valid genus in TENEBRIONIDAE: ALLECULINAE: ALLECULINI: ALLECULINA.

Type species: *Cistelamorio* Fabricius, 1787, by subsequent designation (Duponchel 1840: 283).

Family-group name: ALLÉCULITES Laporte, 1840. Stem: *Allecul*-.

***Alleuscelus* Voss, 1937a**: 159. Current status: valid genus in ATTELABIDAE: ATTELABINAE: ATTELABINI: EUSCELINA.

Type species: *Alleuscelusviolaceipennis* Voss, 1937, by monotypy.

Family-group name: ALLEUSCELINA Legalov, 2003. Stem: *Alleuscel*-.

***Allidiostoma* Arrow, 1940**: 16. Current status: valid genus in SCARABAEIDAE: ALLIDIOSTOMATINAE. Note: new replacement name for *Idiostoma* Arrow, 1904; the older but unused name *Pytoderus* Berg, 1880 (type species *Orphnusstrobeli* Steinheil, 1873, currently placed in *Allidiostoma*) has priority over *Allidiostoma* Arrow, 1940; Reversal of Precedence (ICZN 1999a, Article 23.9) cannot be considered since *Pytoderus* has been used as valid at least once since 1900 (e.g., Ruiz 1924); an application to the Commission is necessary to conserve usage of the name *Allidiostoma*.

Type species: *Idiostomarufum* Arrow, 1904, by original designation (type fixation for *Idiostoma* Arrow).

Family-group name: ALLIDIOSTOMIDAE Arrow, 1940. Stem: *Allidiostomat*-.

***Allocorynus* Sharp, 1890b**: 46. Current status: junior synonym of *Rhopalotria* Chevrolat, 1878 in BELIDAE: OXYCORYNINAE: OXYCORYNITAE: ALLOCORYNINI.

Type species: *Allocorynusmollis* Sharp, 1890, by monotypy.

Family-group name: ALLOCORYNINAE Sharp, 1890. Stem: *Allocoryn*-.

†***Alloioscarabaeus* Bai, Ren & X.-K. Yang in Bai et al., 2012a**: 163. Current status: valid genus in †ALLOIOSCARABAEIDAE. Note: Middle Jurassic (China).

Type species: †*Alloioscarabaeuscheni* Bai, Ren & X.-K. Yang, 2012, by original designation.

Family-group name: †ALLOIOSCARABAEIDAE Bai, Ren & X.-K. Yang in Bai et al., 2012a: 163. Stem: *Alloioscarabae*-.

***Allomorphus* Folwaczny, 1968**: 41. Current status: valid genus in CURCULIONIDAE: COSSONINAE: ALLOMORPHINI.

Type species: *Allomorphusfranzi* Folwaczny, 1968, by monotypy.

Family-group name: ALLOMORPHINI Folwaczny, 1973. Stem: *Allomorph*-.

***Allopoda* J.L. LeConte, 1866a**: 144. Current status: valid genus in SCRAPTIIDAE: SCRAPTIINAE: ALLOPODINI.

Type species: *Scraptialutea* Haldeman, 1848, by monotypy.

Family-group name: ALLOPODINI Franciscolo, 1964. Stem: *Allopod*-.

***Allopogonia* Cockerell, 1906**: 241. Current status: valid genus in ARTEMATOPODIDAE: ALLOPOGONIINAE. Note: new replacement name for *Allopogon* G.H. Horn, 1880.

Type species: *Allopogonvillosus* G.H. Horn, 1880, by monotypy (type fixation for *Allopogon* G.H. Horn).

Family-group name: ALLOPOGONINI Crowson, 1973. Stem: *Allopogoni*-.

***Alloscelus* Boucomont, 1923**: 40. Current status: valid genus in SCARABAEIDAE: SCARABAEINAE: ONTHOPHAGINI.

Type species: *Alloscelusparadoxus* Boucomont, 1923, by monotypy.

Family-group name: ALLOSCELIDES A. Janssens, 1946. Stem: *Alloscel*-.

***Allotrius* Laporte, 1840a**: 231. Current status: junior synonym of *Senodonia* Laporte, 1838 in ELATERIDAE: LISSOMINAE: PROTELATERINI. Note: junior homonym of *Allotrius* Temminck, 1835 [Aves].

Type species: *Semiotusquadricollis* Laporte, 1838, by monotypy.

Family-group name: ALLOTRIITES Candèze, 1863. Stem: *Allotri*-. Note: senior homonym of ALLOTRIINAE Förster, 1869 (type genus *Allotria* Westwood, 1833) in Hymenoptera; family-group name permanently invalid (ICZN 1999a, Article 39).

***Alloxycorynus* Voss, 1957**: 101. Current status: valid genus in BELIDAE: OXYCORYNINAE: AGLYCYDERITAE: ALLOXYCORYNINI.

Type species: *Oxycorynusbruchi* Heller, 1911, by original designation.

Family-group name: ALLOXYCORYNINI Legalov, 2009. Stem: *Alloxycoryn*-.

***Alniphagus* Swaine, 1918**: 73. Current status: valid genus in CURCULIONIDAE: SCOLYTINAE: HYLESININI.

Type species: *Hylesinusaspericollis* J.L. LeConte, 1876, by original designation.

Family-group name: ALNIPHAGINI Murayama, 1963. Stem: *Alniphag*-.

***Alocorhinus* C.R. Sahlberg, 1823a**: 32. Current status: valid genus in CURCULIONIDAE: ENTIMINAE: ENTIMITAE: LORDOPINI.

Type species: *Alocorhinuscomprimatus* C.R. Sahlberg, 1823, by subsequent designation (Jekel 1854: [VI] 12).

Family-group name: ALOCORHINI Jekel, 1856. Stem: *Alocorhin*-.

***Alophus* Schönherr, 1826**: 166. Current status: junior synonym of *Graptus* Schönherr, 1823 in CURCULIONIDAE: ENTIMINAE: ENTIMITAE: BYRSOPAGINI: STRANGALIODINA.

Type species: *Curculiotriguttatus* Fabricius, 1775, by original designation.

Family-group name: ALOPHINI J.L. LeConte, 1874. Stem: *Aloph*-.

***Alphitobius* Stephens, 1829b**: 19. Current status: valid genus in TENEBRIONIDAE: TENEBRIONINAE: ALPHITOBIINI. Note: name placed on the Official List of Generic Names in Zoology (ICZN 1975).

Type species: *Helopspicipes* Panzer, 1794 (= *Opatrumlaevigatum* Fabricius, 1781), by monotypy (see ICZN 1975). Note: *Opatrumlaevigatum* Fabricius, 1781 was placed on the Official List of Specific Names in Zoology (ICZN 1975).

Family-group name: ALPHITOBIINI Reitter, 1917. Stem: *Alphitobi*-.

***Alphitophagus* Stephens, 1832b**: 12. Current status: valid genus in TENEBRIONIDAE: DIAPERINAE: DIAPERINI: ADELININA.

Type species: *Alphitophagusquadripustulatus* Stephens, 1832 (= *Diaperisbifasciatus* Say, 1824), by monotypy.

Family-group name: ALPHITOPHAGIDA Gistel, 1856. Stem: *Alphitophag*-.

***Althanus* Lewis, 1903**: 420. Current status: valid genus in HISTERIDAE: HISTERINAE: PLATYSOMATINI.

Type species: *Althanusteretrioides* Lewis, 1903, by monotypy.

Family-group name: ALTHANINI Cooman, 1939. Stem: *Althan*-.

***Altica* Geoffroy, 1762**: 244. Current status: valid genus in CHRYSOMELIDAE: GALERUCINAE: ALTICINI. Note: name placed on the Official List of Generic Names in Zoology (ICZN 1994a).

Type species: *Chrysomelaoleracea* Linnaeus, 1758, by subsequent designation (Latreille 1810: 432; see ICZN 1994a). Note: name placed on the Official List of Specific Names in Zoology (ICZN 1994a).

Family-group name: HALTICITES Newman, 1834. Stem: *Altic*-.

***Alurnus* Fabricius, 1775**: 94. Current status: valid genus in CHRYSOMELIDAE: CASSIDINAE: ALURNINI.

Type species: *Alurnusgrossus* Fabricius, 1775, by monotypy.

Family-group name: ALURNITES Chapuis, 1875. Stem: *Alurn*-.

***Alvarenganiella* Viana & Martínez, 1971**: 122. Current status: valid genus in PTINIDAE: ALVARENGANIELLINAE.

Type species: *Alvarenganiellaseabrai* Viana & Martínez, 1971 (= *Dasytanobiummonstrosum* Pic, 1910), by original designation.

Family-group name: ALVARENGANIELLINAE Viana & Martínez, 1971. Stem: *Alvarenganiell*-.

***Alvarengius* Frey, 1975**: 84. Current status: junior synonym of *Ottokelleria* d’Andretta & Martínez, 1957 in SCARABAEIDAE: RUTELINAE: ALVARENGIINI.

Type species: *Alvarengiussilphoides* Frey, 1975 (= *Pachylusdispar* Burmeister, 1847), by monotypy.

Family-group name: ALVARENGIINI Frey, 1975. Stem: *Alvarengi*-.

***Alyculus* Kazantsev, 1999**: 252. Current status: valid genus in LYCIDAE: LYROPAEINAE: ALYCULINI.

Type species: *Alyculuskurbatovi* Kazantsev, 1999, by original designation.

Family-group name: ALYCULINI Bocák & Bocáková, 2008. Stem: *Alycul*-.

***Alzadaesthetus* Kistner, 1961**: 217. Current status: valid genus in STAPHYLINIDAE: EUAESTHETINAE: ALZADAESTHETINI.

Type species: *Alzadaesthetuschilensis* Kistner, 1961, by original designation.

Family-group name: ALZADAESTHETINI Scheerpeltz, 1974. Stem: *Alzadaesthet*-.

***Amalactus* Schönherr, 1835**: 251. Current status: valid genus in CURCULIONIDAE: MOLYTINAE: AMALACTINI.

Type species: *Amalactusnigritus* Gyllenhal, 1835, by original designation.

Family-group name: AMALACTIDES Lacordaire, 1863. Stem: *Amalact*-.

***Amalthocus* Fairmaire, 1886**: 40. Current status: valid genus in MELYRIDAE: MALACHIINAE: AMALTHOCINI.

Type species: *Amalthocustetraspilus* Fairmaire, 1886, by original designation.

Family-group name: AMALTHOCINAE Majer, 2002. Stem: *Amalthoc*-.

***Amalus* Schönherr, 1825**: 583. Current status: valid genus in CURCULIONIDAE: CEUTORHYNCHINAE: AMALINI.

Type species: *Curculioscortillum* Herbst, 1795, by original designation.

Family-group name: AMALINA Wagner, 1936. Stem: *Amal*-.

***Amara* Bonelli, 1810**: Tab. Syn. Current status: valid genus in CARABIDAE: HARPALINAE: ZABRINI: AMARINA.

Type species: **here fixed** (under ICZN 1999a, Article 70.3.2) as *Amaralunicollis* Schiødte, 1837, misidentified as *Carabusvulgaris* Linnaeus, 1758 (sensu Panzer, 1796), in the subsequent designation by Westwood (1838c: 4). Note: no species were originally included in this genus by Bonelli (1810); the first ones were the eight species listed by Panzer (1813: 57–59).

Family-group name: AMAROIDEN C. Zimmermann, 1832. Stem: *Amar*-.

***Amarotypus* H.W. Bates, 1872**: 50. Current status: valid genus in CARABIDAE: MIGADOPINAE: AMAROTYPINI.

Type species: *Amarotypusedwardsii* H.W. Bates, 1872, by monotypy.

Family-group name: AMAROTYPINI Erwin, 1985. Stem: *Amarotyp*-.

***Amarygmus* Dalman, 1823**: 60. Current status: valid genus in TENEBRIONIDAE: TENEBRIONINAE: AMARYGMINI.

Type species: *Chrysomelamicans* Fabricius, 1794, by subsequent designation (Gebien 1921b: 410).

Family-group name: AMARYGMIIDAE Gistel, 1848. Stem: *Amarygm*-.

***Amauronioides* Champion, 1923a**: 13. Current status: junior synonym of *Pseudamauronia* Pic, 1915 in MELYRIDAE: DASYTINAE: DANACEINI.

Type species: *Amauronioidesschilskyi* Champion, 1923 (= *Dasytiscusdanacaeoides* Reitter, 1885), by monotypy. Note: combined description by indication of a new genus and a single new species (ICZN 1999a, Article 12.2.6).

Family-group name: AMAURONOININI Majer, 1987. Stem: *Amauronioid*-.

***Amaurops* Fairmaire, 1851**: 527. Current status: valid genus in STAPHYLINIDAE: PSELAPHINAE: BATRISITAE: AMAUROPINI.

Type species: *Amauropsaubei* Fairmaire, 1851, by monotypy.

Family-group name: AMAUROPSINI Jeannel, 1948. Stem: *Amaurop*-.

†***Ambarticus* Q. Yang, Z.-Y. Chen & Jia, 2019**: 2. Current status: valid genus in DYTISCIDAE: DYTISCINAE: †AMBARTICINI. Note: mid-Cretaceous (Myanmar). Note: two original spellings were used, including *Ambraticus* (pp. 3, 4); as far as we know, no one has acted as First Reviser (ICZN 1999a, Article 24.2.3) and we **here select***Ambarticus* as the correct original spelling.

Type species: †*Ambarticusmyanmaricus* Q. Yang, Chen & Jia, 2019, by original designation.

Family-group name: †AMBARTICINI Q. Yang, Z.-Y. Chen & Jia, 2019: 2. Stem: *Ambartic*-. Note: two spellings were originally used for the name of this new tribe, including AMBRATICINI (p. 1); as far as we know no one has acted as First Reviser (ICZN 1999a, Article 24.2.3) so we **here select**AMBARTICINI as the correct original spelling.

***Ambates* Schönherr, 1835**: 278. Current status: junior synonym of *Embates* Chevrolat, 1833 in CURCULIONIDAE: BARIDINAE: AMBATINI.

Type species: *Ambatespictus* Gyllenhal, 1835, by original designation.

Family-group name: AMBATIDES Lacordaire, 1863. Stem: *Ambat*-.

***Ambicocerus* Leleup, 1970**: 314. Current status: valid genus in STAPHYLINIDAE: PSELAPHINAE: BATRISITAE: BATRISINI: AMBICOCERINA.

Type species: *Ambicoceruscarinaticollis* Leleup, 1970, by original designation.

Family-group name: AMBICOCERINA Leleup, 1970. Stem: *Ambicocer*-.

***Amblycerus* Thunberg, 1815**: 109. Current status: valid genus in CHRYSOMELIDAE: BRUCHINAE: AMBLYCERINI: AMBLYCERINA.

Type species [suggested]: *Bruchusrobiniae* Fabricius, 1781, by subsequent designation (Bridwell in Pierce 1930: 29). Note: the valid type species fixation is that of Crotch (1870b: 227), who selected *Anthribusnebulosus* Forster, 1770 (see Bradley 1946: 97, 98), but that species is currently included in the genus *Anthribus* Geoffroy, 1762; Alonso-Zarazaga and Lyal (1999: 8, 23) mentioned that they would submit an application to the Commission to fix this problem but to our knowledge this has not been done, and an application is still necessary to fix *Bruchusrobiniae* Fabricius, 1781 as the type species of *Amblycerus* Thunberg, 1815.

Family-group name: AMBLYCERINAE Bridwell, 1932. Stem: *Amblycer*-.

***Amblycheila* Say, 1830a**: 67. Current status: valid genus in CICINDELIDAE: MANTICORINI.

Type species: *Manticoracylindriformis* Say, 1823, by original designation.

Family-group name: AMBLYCHILINAE Csiki, 1903. Stem: *Amblycheil*-.

***Amblyderus* LaFerté-Sénectère in P.H. Lucas, 1847b**: 368. Current status: valid genus in ANTHICIDAE: ANTHICINAE: ANTHICINI. Note: the original spelling was *Aneblyderes* but the spelling *Amblyderus*, changed by LaFerté-Sénectère in Guérin-Méneville (1849 [no 23]: 1), is in prevailing usage, attributed to the publication of the original spelling, and therefore deemed to be the correct original spelling (ICZN 1999a, Article 33.2.3.1).

Type species: *Anthicusscabricollis* LaFerté-Sénectère, 1847, by monotypy.

Family-group name: AMBLYDERINI Desbrochers des Loges, 1899. Stem: *Amblyder*-.

***Amblyopinus* Solsky, 1875**: 10. Current status: valid genus in STAPHYLINIDAE: STAPHYLININAE: STAPHYLININI: AMBLYOPININA.

Type species: *Amblyopinusielskii* Solsky, 1875, by subsequent designation (R. Lucas 1920: 88).

Family-group name: AMBLYOPININAE Seevers, 1944. Stem: *Amblyopin*-.

***Amblysterna* Saunders, 1871**: 2. Current status: valid genus in BUPRESTIDAE: JULODINAE.

Type species: *Julodisnatalensis* Fåhraeus, 1851, by subsequent designation (Rye 1873: 271).

Family-group name: AMBLYSTERNINI Cobos, 1955. Stem: *Amblystern*-.

***Amblystomus* Erichson, 1837**: 59. Current status: valid genus in CARABIDAE: HARPALINAE: HARPALINI: AMBLYSTOMINA.

Type species: *Acupalpusmauritanicus* Dejean, 1829, by subsequent designation (Andrewes 1938: 130).

Family-group name: AMBLYSTOMINI Fauvel, 1889. Stem: *Amblystom*-.

***Amblytelus* Erichson, 1842**: 129. Current status: valid genus in CARABIDAE: PSYDRINAE: MORIOMORPHINI: AMBLYTELINA.

Type species: *Carabuscurtus* Fabricius, 1801, by monotypy.

Family-group name: AMBLYTELIDES Blackburn, 1892. Stem: *Amblytel*-. Note: senior homonym of AMBLYTELINA Viereck, 1918 (type genus *Amblyteles* Wesmael, 1845) in Hymenoptera.

***Amborotubus* Leschen & Carlton, 2004**: 443. Current status: valid genus in NITIDULIDAE: NITIDULINAE: CYLLODINI.

Type species: *Amborotubusclarkei* Leschen & Carlton, 2004, by original designation.

Family-group name: AMBOROTUBINI Leschen & Carlton, 2004. Stem: *Amborotub*-.

***Ameris* Dejean, 1821**: 86. Current status: valid genus in CURCULIONIDAE: MOLYTINAE: CHOLINI: CHOLINA.

Type species: *Rhynchaenusdufresnii* W. Kirby, 1819, by monotypy.

Family-group name: AMERININAE Pierce, 1919. Stem: *Amerin*-.

***Ametalla* Hope, 1841a**: 179. Current status: valid genus in CHRYSOMELIDAE: SAGRINAE: MEGAMERINI.

Type species: *Ametallaspinolae* Hope, 1841, by original designation.

Family-group name: AMÉTALLITES Chapuis, 1874. Stem: *Ametall*-.

***Ametrocephala* Blanchard, 1851**: 480. Current status: junior synonym of *Pseudocephalus* Newman, 1842 in CERAMBYCIDAE: CERAMBYCINAE: PSEUDOCEPHALINI.

Type species: *Ametrocephalamonstrosa* Blanchard, 1851, by monotypy.

Family-group name: AMETROCEPHALITAE J. Thomson, 1861. Stem: *Ametrocephal*-.

***Aminyops* Voss, 1956**: 29. Current status: junior synonym of *Niphadonyx* Schenkling, 1932 in CURCULIONIDAE: MOLYTINAE: AMINYOPINI.

Type species: *Aminyopscastaneus* Voss, 1956, by monotypy.

Family-group name: AMINYOPINI Voss, 1956. Stem: *Aminyop*-.

***Ammobius* Guérin-Méneville, 1844a**: 121. Current status: valid genus in TENEBRIONIDAE: BLAPTINAE: OPATRINI: AMMOBIINA.

Type species: *Ammobiusrufus* Guérin-Méneville, 1844, by monotypy. Note: combined description of a new genus and a single new species (ICZN 1999a, Article 12.2.6).

Family-group name: AMMOBIINI Desbrochers des Loges, 1902. Stem: *Ammobi*-.

***Amomphus* Schönherr, 1848**: 359. Current status: junior synonym of *Aspidiotes* Schönherr, 1847 in CURCULIONIDAE: ENTIMINAE: POLYDRUSITAE: TANYMECINI: TAINOPHTHALMINA. Note: unnecessary new replacement name for *Aspidiotes* Schönherr, 1847.

Type species: *Aspidioteswestringii* Schönherr, 1847, by original designation (type fixation for *Aspidiotes* Schönherr).

Family-group name: AMOMPHI J.L. LeConte, 1874. Stem: *Amomph*-.

***Amorphocephalus* Schönherr, 1840a**: 485. Current status: invalid senior synonym of *Amorphocephala* Damoiseau, 1966 in BRENTIDAE: BRENTINAE: BRENTINI: EREMOXENINA. Note: junior homonym of *Amorphocephalus* Bowdich, 1825 [Pisces].

Type species: *Brentuscoronatus* Germar, 1817, by original designation.

Family-group name: AMORPHOCEPHALIDES Power, 1879. Stem: *Amorphocephal*-. Note: family- group name permanently invalid (ICZN 1999a, Article 39).

***Amorphocerus* Schönherr, 1826**: 329. Current status: valid genus in CURCULIONIDAE: MOLYTINAE: AMORPHOCERINI.

Type species: *Amorphocerustalpa* Schönherr, 1826, by original designation.

Family-group name: AMORPHOCERINI Voss, 1939. Stem: *Amorphocer*-.

***Amorphomerus* Sloane, 1923**: 249. Current status: valid genus in CARABIDAE: HARPALINAE: AMORPHOMERINI. Note: new replacement name for *Trimerus* Chaudoir, 1878.

Type species: *Trimerusraffrayi* Chaudoir, 1878, by monotypy (type fixation for *Trimerus* Chaudoir).

Family-group name: AMORPHOMERINI Sloane, 1923. Stem: *Amorphomer*-.

***Amorphosoma* Laporte, 1835**: 166. Current status: valid genus in BUPRESTIDAE: AGRILINAE: CORAEBINI: AMORPHOSOMINA.

Type species: *Buprestispenicillata* Klug, 1825, by subsequent designation (Descarpentries and Villiers 1967: 488).

Family-group name: AMORPHOSOMINA Majer, 2000. Stem: *Amorphosom*- (ICZN 1999a, Article 29.4).

***Amorphosternus* Deyrolle, 1865**: 114. Current status: valid genus in BUPRESTIDAE: AGRILINAE: AGRILINI: AMORPHOSTERNINA.

Type species: *Amorphosternuscucullatus* Deyrolle, 1865, by monotypy.

Family-group name: AMORPHOSTERNAE Cobos, 1974. Stem: *Amorphostern*-.

***Ampedus* Dejean, 1833a**: 92. Current status: valid genus in ELATERIDAE: ELATERINAE: AMPEDINI.

Type species: *Elatersanguineus* Linnaeus, 1758, by subsequent designation (Curtis 1838a: pl. 694).

Family-group name: AMPEDIDAE Gistel, 1848. Stem: *Amped*-.

***Amphicoma* Latreille, 1806**: 118. Current status: valid genus in GLAPHYRIDAE: AMPHICOMINAE.

Type species: *Melolonthaabdominalis* Fabricius, 1781, by subsequent designation (Latreille 1810: 428).

Family-group name: AMPHICOMITES Blanchard, 1845. Stem: *Amphicom*-.

***Amphicranus* Erichson, 1836**: 63. Current status: valid genus in CURCULIONIDAE: SCOLYTINAE: CORTHYLINI: CORTHYLINA.

Type species: *Amphicranusthoracicus* Erichson, 1836, by monotypy.

Family-group name: AMPHICRANIDAE Eichhoff, 1878. Stem: *Amphicran*-.

***Amphicrossus* Erichson, 1843a**: 346. Current status: valid genus in NITIDULIDAE: AMPHICROSSINAE.

Type species: *Nitidulaciliata* G.-A. Olivier, 1811, by subsequent designation (E. Desmarest 1851: 283).

Family-group name: AMPHICROSSINI Kirejtshuk, 1986. Stem: *Amphicross*-.

***Amphicyrta* Erichson in Steffahny, 1842**: 5, 39. Current status: valid genus in BYRRHIDAE: AMPHICYRTINAE.

Type species: *Amphicyrtadentipes* Erichson, 1842, by subsequent designation (Chevrolat 1843a: 657).

Family-group name: AMPHICYRTINI J.L. LeConte, 1861. Stem: *Amphicyrt*-.

***Amphidora* Eschscholtz, 1829d**: 9. Current status: valid genus in TENEBRIONIDAE: BLAPTINAE: AMPHIDORINI.

Type species: *Amphidoralittoralis* Eschscholtz, 1829, by monotypy.

Family-group name: AMPHIDORAE J.L. LeConte, 1862. Stem: *Amphidor*-.

***Amphilabris* Gistel, 1834**: 12. Current status: junior synonym of *Sericus* Eschscholtz, 1829 in ELATERIDAE: ELATERINAE: ELATERINI.

Type species: *Elaterbrunneus* Linnaeus, 1758, by subsequent designation (Sánchez-Ruiz 1996: 155).

Family-group name: AMPHILABRIDAE Gistel, 1856. Stem: *Amphilabr*-.

***Amphionycha* Dejean, 1835**: 352. Current status: junior synonym of *Adesmus* Lepeletier & Audinet- Serville, 1825 in CERAMBYCIDAE: LAMIINAE: HEMILOPHINI.

Type species: *Saperdahemispila* Germar, 1821, by subsequent designation (Marinoni 1977: 40).

Family-group name: AMPHIONYCHITAE J. Thomson, 1860. Stem: *Amphionych*-. Note: nomen oblitum (see Bousquet et al. 2009: 31).

***Amphiops* Erichson, 1843b**: 229. Current status: valid genus in HYDROPHILIDAE: HYDROPHILINAE: AMPHIOPINI.

Type species: *Hydrophilusgibbus* Illiger, 1801, by subsequent designation (Knisch 1924: 259).

Family-group name: AMPHIOPITAE Kuwert, 1890. Stem: *Amphiop*-.

***Amphiscolytus* Mandelshtam & Beaver, 2003**: 2. Current status: valid genus in CURCULIONIDAE: SCOLYTINAE: AMPHISCOLYTINI.

Type species: *Dacryophthoruscapensis* Schedl, 1971, by original designation.

Family-group name: AMPHISCOLYTINI Mandelshtam & Beaver, 2003. Stem: *Amphiscolyt*-.

***Amphisternus* Germar, 1843**: 85. Current status: valid genus in ENDOMYCHIDAE: LYCOPERDININAE.

Type species [suggested]: *Amphisternustuberculatus* Germar, 1843, by subsequent designation (R. Lucas 1920: 91). Note: the valid type species fixation is that of Guérin-Méneville (1857: 579), who selected *Amphisternusinaequalis* Germar, 1843, but that species is currently included in the genus *Cacodaemon* J. Thomson, 1857; an application to the Commission is necessary to fix *Amphisternustuberculatus* Germar, 1843 as the type species of *Amphisternus* Germar, 1843.

Family-group name: AMPHISTERNINI Strohecker, 1964. Stem: *Amphistern*-.

***Amphix* Laporte, 1840b**: 522. Current status: junior synonym of *Corynomalus* Chevrolat, 1836 in ENDOMYCHIDAE: LYCOPERDININAE.

Type species: *Eumorphusbinotatus* Laporte, 1840 (= *Erotylusmarginatus* Fabricius, 1798), by subsequent designation (Strohecker 1980: 14).

Family-group name: AMPHICINI Csiki, 1910. Stem: *Amphic*-.

***Amphizoa* J.L. LeConte, 1853b**: 227. Current status: valid genus in AMPHIZOIDAE.

Type species: *Amphizoainsolens* J.L. LeConte, 1853, by monotypy.

Family-group name: AMPHIZOIDAE J.L. LeConte, 1853. Stem: *Amphizo*-.

***Amphoecus* Montrouzier, 1861b**: 274. Current status: valid genus in CERAMBYCIDAE: LAMIINAE: AMPHOECINI.

Type species: *Amphoecusmetallicus* Montrouzier, 1861, by monotypy.

Family-group name: AMPHOECINI Breuning, 1951. Stem: *Amphoec*-.

***Amplipalpa* Harold, 1875**: 185. Current status: junior synonym of *Oediopalpa* Baly, 1858 in CHRYSOMELIDAE: CASSIDINAE: OEDIOPALPINI. Note: unnecessary new replacement name for *Oediopalpa* Baly, 1858.

Type species: *Hispacyanipennis* Fabricius, 1801, by subsequent designation (Monrós and Viana 1947: 151) (type fixation for *Oediopalpa* Baly).

Family-group name: AMPLIPALPINI Weise, 1910. Stem: *Amplipalp*-.

***Amycterus* Schönherr, 1823**: 1144. Current status: valid genus in CURCULIONIDAE: CYCLOMINAE: AMYCTERINI.

Type species: *Amycterustalpa* Schönherr, 1823, by original designation.

Family-group name: AMYCTERIDAE G.R. Waterhouse, 1854. Stem: *Amycter*-.

***Amydetes* Illiger, 1807b**: 342. Current status: valid genus in LAMPYRIDAE: AMYDETINAE.

Type species: *Amydetesfastigiata* Illiger, 1807, by monotypy.

Family-group name: AMYDETINI E. Olivier, 1907. Stem: *Amydet*-.

***Amyia* Saunders, 1871**: 111. Current status: valid genus in BUPRESTIDAE: AGRILINAE: AGRILINI: AMYIINA.

Type species: *Eumerusviolaceus* Gory & Laporte, 1839, by subsequent designation (Rye 1873: 271).

Family-group name: AMYIINA Holyński, 1993. Stem: *Amyi*-.

***Amymoma* Pascoe, 1866c**: 329, 332. Current status: valid genus **stat. nov.** in CERAMBYCIDAE: LAMIINAE: DESMIPHORINI. Note: this name has been considered a junior homonym of *Amymoma* Latreille, 1797 [Crustacea] but Latreille’s name is an incorrect subsequent spelling of *Amymone* O.F. Müller, 1776 as noted by Neave (1939: 168) not in prevailing usage, and therefore the name is not available; the new replacement name *Neoamymoma* Marinoni, 1977 is therefore unnecessary.

Type species: *Amymomapulchella* Pascoe, 1866, by monotypy.

Family-group name: AMYMOMIDES Lacordaire, 1872. Stem: *Amymom*-.

***Anacaena* C.G. Thomson, 1859**: 18. Current status: valid genus in HYDROPHILIDAE: CHAETARTHRIINAE: ANACAENINI. Note: name placed on the Official List of Generic Names in Zoology (ICZN 1960).

Type species: *Hydrophilusglobulus* Paykull, 1798, by original designation (see ICZN 1960, type fixation given as “by monotypy”). Note: name placed on the Official List of Specific Names in Zoology (ICZN 1960).

Family-group name: ANACAENINI Hansen, 1991. Stem: *Anacaen*-.

***Anacanthus* Audinet-Serville, 1832**: 165. Current status: invalid senior synonym of *Chorenta* Gistel, 1848 in CERAMBYCIDAE: PRIONINAE: CALLIPOGONINI. Note: junior homonym of *Anacanthus* J.E. Gray, 1831 [Pisces].

Type species: *Anacanthuscostatus* Audinet-Serville, 1832 (= *Prionusreticulatus* Dalman, 1817), by monotypy.

Family-group name: ANACANTHITAE J. Thomson, 1864. Stem: *Anacanth*-. Note: family-group name permanently invalid (ICZN 1999a, Article 39).

***Anacolus* Berthold, 1827**: 395. Current status: valid genus in CERAMBYCIDAE: PRIONINAE: ANACOLINI.

Type species: *Prionussanguineus* Lepeletier & Audinet-Serville, 1825, by subsequent designation (E. Desmarest 1860: 306). Note: no species were originally included in this genus by Berthold (1827); the first ones were the two species listed by Latreille (1829b: 108).

Family-group name: ANACOLITES J. Thomson, 1857. Stem: *Anacol*-.

***Anaesthetis* Dejean, 1835**: 348. Current status: valid genus in CERAMBYCIDAE: LAMIINAE: DESMIPHORINI.

Type species: *Saperdatestacea* Fabricius, 1781, by monotypy.

Family-group name: ANAESTHETINAE Perrier, 1893. Stem: *Anaesthet*-.

***Anaglyptes* Gistel, 1848b**: 128. Current status: junior synonym of *Chalcophora* Solier, 1833 in BUPRESTIDAE: CHRYSOCHROINAE: CHRYSOCHROINI: CHALCOPHORINA.

Type species: *Anaglyptesmerianus* Gistel, 1848 (= *Buprestismariana* Linnaeus, 1758), by monotypy.

Family-group name: ANAGLYPTISIDAE Gistel, 1848. Stem: *Anaglypt*-. Note: nomen oblitum (see Bousquet et al. 2009: 41); senior homonym of ANAGLYPTINI Lacordaire, 1868 (type genus *Anaglyptus* Mulsant, 1839) in Coleoptera: CERAMBYCIDAE.

***Anaglyptus* Mulsant, 1839**: 71, 91. Current status: valid genus in CERAMBYCIDAE: CERAMBYCINAE: ANAGLYPTINI. Note: two original spellings were used, including *Anaclyptus* (p. 71); subsequently, Mulsant (1863: 184, 185) used only *Anaglyptus* and therefore acted as First Reviser (ICZN 1999a, Article 24.2.4).

Type species: *Lepturamystica* Linnaeus, 1758, by subsequent designation (C.G. Thomson 1859: 150).

Family-group name: ANAGLYPTIDES Lacordaire, 1868. Stem: *Anaglypt*-. Note: nomen protectum (see Bousquet et al. 2009: 41); junior homonym of ANAGLYPTIDAE Gistel, 1848 (type genus *Anaglyptes* Gistel, 1848) in Coleoptera: BUPRESTIDAE.

***Anaides* Westwood, 1842a**: 457. Current status: valid genus in HYBOSORIDAE: ANAIDINAE.

Type species: *Anaidesfossulatus* Westwood, 1842, by monotypy.

Family-group name: ANAIDINI Nikolajev, 1996. Stem: *Anaid*-.

***Anaissus* Candèze, 1857**: 187. Current status: valid genus in ELATERIDAE: AGRYPNINAE: ANAISSINI.

Type species: *Anaissustarsalis* Candèze, 1857, by monotypy.

Family-group name: ANAISSINI Golbach, 1984. Stem: *Anaiss*-.

***Anamorphus* J.L. LeConte in E.A. Schwarz, 1878**: 445. Current status: valid genus in ANAMORPHIDAE.

Type species: *Anamorphuspusillus* J.L. LeConte, 1878, by monotypy.

Family-group name: ANAMORPHINI Strohecker, 1953. Stem: *Anamorph*-.

***Anapleus* G.H. Horn, 1873b**: 311. Current status: valid genus in HISTERIDAE: DENDROPHILINAE: ANAPLEINI.

Type species: *Bacaniusmarginatus* J.L. LeConte, 1853, by monotypy.

Family-group name: ANAPLEINI Olexa, 1982. Stem: *Anaple*-.

***Anaplopus* Blackburn, 1890**: 311. Current status: valid genus in PYTHIDAE.

Type species: *Anaplopustuberculatus* Blackburn, 1890, by monotypy.

Family-group name: ANAPLOPINAE M. Abdullah, 1966. Stem: *Anaplopod*-.

***Anaspimorda* Ermisch, 1950**: 89. Current status: valid genus in SCRAPTIIDAE: ANASPIDINAE: ANASPIMORDINI.

Type species: *Anaspimordaforticornis* Ermisch, 1950, by original designation.

Family-group name: ANASPIMORDINI Franciscolo, 1954. Stem: *Anaspimord*-.

***Anaspis* Geoffroy, 1762**: 315. Current status: valid genus in SCRAPTIIDAE: ANASPIDINAE: ANASPIDINI. Note: name attributed to Geoffroy, 1762 and placed on the Official List of Generic Names in Zoology (ICZN 1984a).

Type species: *Mordellafrontalis* Linnaeus, 1758, by plenary power (see ICZN 1984a). Note: name placed on the Official List of Specific Names in Zoology (ICZN 1984a).

Family-group name: ANASPIENS Mulsant, 1856. Stem: *Anaspid*-.

***Anatista* Brême, 1845**: 305. Current status: valid genus in SCARABAEIDAE: RUTELINAE: ANATISTINI.

Type species: *Anatistalafertei* Brême, 1845, by monotypy.

Family-group name: ANATISTIDES Lacordaire, 1855. Stem: *Anatist*-.

***Anaulacus* W.S. MacLeay, 1825**: 22. Current status: valid genus in CARABIDAE: HARPALINAE: MASOREINI.

Type species: *Anaulacussericipennis* W.S. MacLeay, 1825, by monotypy.

Family-group name: ANAULACINI Csiki, 1932. Stem: *Anaulac*-.

***Anauxesis* J. Thomson, 1857**: 191. Current status: valid genus in CERAMBYCIDAE: LAMIINAE: AGAPANTHIINI.

Type species: *Nemotraguscalabaricus* Chevrolat, 1855, by subsequent designation (J. Thomson 1864: 94).

Family-group name: ANAUXESITAE J. Thomson, 1864. Stem: *Anauxese*-.

†***Anchineus* Poinar & A.E. Brown, 2009a**: 264. Current status: valid genus in †MESOPHYLETIDAE: †MESOPHYLETINAE. Note: mid-Cretaceous (Myanmar).

Type species: †*Anchineusdolichobothris* Poinar & A.E. Brown, 2009, by original designation.

Family-group name: †ANCHINEINI Poinar & Legalov in Legalov and Poinar, 2014: 558. Stem: *Anchine*-.

***Anchisteus* Kolbe, 1883**: 185. Current status: valid genus in BRENTIDAE: BRENTINAE: TRACHELIZINI: HEPHEBOCERINA.

Type species: *Anchisteusperegrinus* Kolbe, 1883, by monotypy.

Family-group name: ANCHISTENINI Schedl, 1961. Stem: *Anchiste*-.

***Anchomenus* Bonelli, 1810**: Tab. Syn. Current status: valid genus in CARABIDAE: HARPALINAE: PLATYNINI.

Type species: *Carabusprasinus* Thunberg, 1784 (= *Carabusdorsalis* Pontoppidan, 1763), by subsequent designation (Westwood 1838c: 3). Note: no nominal species were originally included in this genus by Bonelli (1810); the first ones were the three species listed by Panzer (1813: 54, 55).

Family-group name: ANCHOMENII Bonelli, 1810. Stem: *Anchomen*-.

***Anchomma* J.L. LeConte, 1858a**: 63. Current status: valid genus in TENEBRIONIDAE: PIMELIINAE: STENOSINI: DICHILLINA.

Type species: *Anchommacostatum* J.L. LeConte, 1858, by monotypy.

Family-group name: ANCHOMMINI G.H. Horn, 1878. Stem: *Anchommat*-.

***Anchonoderus* Reiche, 1843a**: 38. Current status: valid genus in CARABIDAE: HARPALINAE: LACHNOPHORINI: LACHNOPHORINA.

Type species: *Platynuselegans* Brullé, 1837, by original designation.

Family-group name: ANCHONODÉRIDES Lacordaire, 1854. Stem: *Anchonoder*-.

***Anchonus* Schönherr, 1825**: 584. Current status: valid genus in CURCULIONIDAE: MOLYTINAE: ANCHONINI.

Type species: *Curculiosuillus* Fabricius, 1792, by original designation.

Family-group name: ANCHONIDAE Imhoff, 1856. Stem: *Anchon*-.

***Anchytarsus* Guérin-Méneville, 1843a**: 194. Current status: valid genus in PTILODACTYLIDAE: ANCHYTARSINAE.

Type species: *Anchytarsusater* Guérin-Méneville, 1849 (= *Atopabicolor* Mel­sheimer, 1845), by subsequent monotypy (Guérin-Méneville 1849 [no 15]: 1).

Family-group name: ANCHYTARSINI Champion, 1897. Stem: *Anchytars*-. Note: an application to the Commission is needed to conserve this family-group name as valid over COLOBODERINAE Erichson, 1847 (see Bouchard et al. 2011: 297).

***Ancistria* Erichson, 1845a**: 305. Current status: valid genus in PASSANDRIDAE: PASSANDRINAE.

Type species: *Colydiumretusum* Fabricius, 1801, by monotypy.

Family-group name: ANCISTRIINAE Sharp, 1899. Stem: *Ancistri*-.

***Ancistrotus* Audinet-Serville, 1832**: 135. Current status: valid genus in CERAMBYCIDAE: PRIONINAE: MACRODONTIINI.

Type species: *Ancistrotushamaticollis* Audinet-Serville, 1832 (= *Prionusuncinatus* Klug, 1825), by monotypy.

Family-group name: ANCISTROTIDES Lacordaire, 1868. Stem: *Ancistrot*-.

***Ancita* J. Thomson, 1864**: 63. Current status: junior synonym of *Hebecerus* Dejean, 1835 in CERAMBYCIDAE: LAMIINAE: ANCITINI. Note: *Hebecerus* Dejean, 1835 has priority over the name *Ancita* J. Thomson, 1864, which has been used as valid in recent publications; we believe that a request should be submitted to the Commission to retain *Ancita* (and the tribe ANCITINI, instead of HEBESECIDINI Pascoe, 1871) as valid (see Bousquet and Bouchard 2013a: 83; Bouchard and Bousquet 2020a: 107).

Type species: *Ancitacrossotoides* J. Thomson, 1864 (= *Hebecerusniphonoides* Pascoe, 1863), by original designation.

Family-group name: ANCITINI Aurivillius, 1917. Stem: *Ancit*-.

***Ancylocera* Audinet-Serville, 1834b**: 107. Current status: valid genus in CERAMBYCIDAE: CERAMBYCINAE: TRACHYDERINI: ANCYLOCERINA.

Type species: *Cerambyxcardinalis* Dalman, 1823, by monotypy.

Family-group name: ANCYLOCERITAE J. Thomson, 1864. Stem: *Ancylocer*-.

***Ancylocheira* Eschscholtz, 1829b**: 9. Current status: valid subgenus of *Buprestis* Linnaeus, 1758 in BUPRESTIDAE: BUPRESTINAE: BUPRESTINI: BUPRESTINA. Note: the original spelling was *Ancylochira* but *Ancylocheira*, an incorrect subsequent spelling, is in prevailing usage, attributed to the publication of the original spelling, and therefore deemed to be the correct original spelling (ICZN 1999a, Article 33.3.1).

Type species: *Buprestisrustica* Linnaeus, 1758, by subsequent designation (Westwood 1838c: 24).

Family-group name: ANCYLOCHIRINA Jakobson, 1913. Stem: *Ancylocheir*-.

***Ancylocnemis* G.A.K. Marshall, 1920**: 387. Current status: valid genus in CURCULIONIDAE: CURCULIONINAE: ANCYLOCNEMIDINI.

Type species: *Ancylocnemisfasciculata* G.A.K. Marshall, 1920, by original designation.

Family-group name: ANCYLOCNEMINI Voss, 1962. Stem: *Ancylocnemid*-.

***Ancylonotus* Dejean, 1835**: 335. Current status: valid genus in CERAMBYCIDAE: LAMIINAE: ANCYLONOTINI.

Type species: *Lamiatribulus* Fabricius, 1775, by monotypy.

Family-group name: ANCYLONOTIDES Lacordaire, 1869. Stem: *Ancylonot*-.

***Ancylopoma* Pascoe, 1871d**: 354. Current status: valid genus in TENEBRIONIDAE incertae sedis.

Type species: *Ancylopomapunctigerum* Pascoe, 1871, by monotypy.

Family-group name: ANCYLOPOMINAE Pascoe, 1871. Stem: *Ancylopomat*-.

***Ancyrona* Reitter, 1876c**: 51. Current status: valid genus in LOPHOCATERIDAE.

Type species: *Ancyronalewisi* Reitter, 1876, by subsequent designation (Kolibáč 1993: 16).

Family-group name: ANCYRONINI Kolibáč, 2006. Stem: *Ancyron*-.

***Ancyronyx* Erichson, 1847a**: 522. Current status: valid genus in ELMIDAE: ELMINAE: ANCYRONYCHINI.

Type species: *Macronychusvariegatus* Germar, 1823, by monotypy.

Family-group name: ANCYRONYCHINI Ganglbauer, 1904. Stem: *Ancyronych*-.

***Andotypus* Spangler, 1979**: 303. Current status: valid genus in HYDROPHILIDAE: CYLOMINAE.

Type species: *Andotypusashworthi* Spangler, 1979, by original designation.

Family-group name: ANDOTYPINI Hansen, 1991. Stem: *Andotyp*-.

***Androlyperus* Crotch, 1873b**: 55. Current status: valid genus in CHRYSOMELIDAE: GALERUCINAE: LUPERINI.

Type species: *Androlyperusfulvus* Crotch, 1873, by monotypy.

Family-group name: ANDROLYPERITES G.H. Horn, 1893. Stem: *Androlyper*-.

***Androzelma* Dostal, 1993**: 117. Current status: valid genus in CARABIDAE: SCARITINAE: SALCEDIINI: ANDROZELMINA.

Type species: *Androzelmagigas* Dostal, 1993, by original designation.

Family-group name: ANDROZELMINA R.T. Bell, 1998. Stem: *Androzelm*-.

***Anelastes* W. Kirby, 1819**: 384. Current status: valid genus in EUCNEMIDAE: PHYLLOCERINAE: ANELASTINI.

Type species: *Anelastesdrurii* W. Kirby, 1819, by monotypy.

Family-group name: ANELASTINI Reitter, 1911. Stem: *Anelast*-.

***Anelastidius* Jacquelin du Val, 1859c**: 117, 121. Current status: valid genus in EUCNEMIDAE: MACRAULACINAE: ANELASTIDINI.

Type species: *Anelastidiusineditus* Jacquelin du Val, 1859 (= *Eucnemisfeisthameli* Graells, 1847), by original designation.

Family-group name: ANELASTIDINI Muona, 1993. Stem: *Anelastid*-.

***Anemadus* Reitter, 1884b**: 38, 58. Current status: valid genus in LEIODIDAE: CHOLEVINAE: ANEMADINI: ANEMADINA.

Type species: *Catopsstrigosus* Kraatz, 1852, by subsequent designation (Jeannel 1922: 41).

Family-group name: ANEMADINA Hatch, 1927. Stem: *Anemad*-.

***Anepius* Blackburn, 1902**: 29. Current status: valid genus in STAPHYLINIDAE: PROTEININAE: ANEPIINI.

Type species: *Anepiuskoebelei* Blackburn, 1902, by subsequent designation (R. Lucas 1920: 96).

Family-group name: ANEPIINI Steel, 1966. Stem: *Anepi*-.

***Anepsius* J.L. LeConte, 1851a**: 147. Current status: valid genus in TENEBRIONIDAE: PIMELIINAE: ANEPSIINI.

Type species: *Anepsiusdelicatulus* J.L. LeConte, 1851, by monotypy.

Family-group name: ANEPSIINI J.L. LeConte, 1862. Stem: *Anepsi*-.

***Angianus* Sharp, 1919**: 151. Current status: junior synonym of *Vanapa* Pouillaude, 1915 in CURCULIONIDAE: MOLYTINAE: PISSODINI: ORTHORHININA.

Type species: *Angianuspratti* Sharp, 1919 (= *Vanapaoberthuri* Pouillaude, 1915), by monotypy.

Family-group name: ANGIANIDES Sharp, 1919. Stem: *Angian*-.

***Anilara* Saunders, 1868**: 19. Current status: valid genus in BUPRESTIDAE: BUPRESTINAE: CURIDINI: ANILARINA.

Type species: *Anthaxiaadelaidae* Hope, 1846, by monotypy.

Family-group name: ANILARINI Bílý, 2000. Stem: *Anilar*-.

***Anillus* Jacquelin du Val, 1851**: lxxiii. Current status: valid genus in CARABIDAE: TRECHINAE: ANILLINI.

Type species: *Anilluscoecus* Jacquelin du Val, 1851, by monotypy.

Family-group name: ANILLINI Jeannel, 1937. Stem: *Anill*-.

***Anisarthron* Dejean, 1835**: 331. Current status: valid genus in CERAMBYCIDAE: SPONDYLIDINAE: ANISARTHRINI.

Type species: *Cerambyxbarbipes* Schrank, 1781, by monotypy.

Family-group name: ANISARTHRONINI Mamaev & Danilevsky, 1973. Stem: *Anisarthr*-.

***Anischia* Fleutiaux, 1896**: 300. Current status: valid genus in EUCNEMIDAE: ANISCHIINAE.

Type species: *Anischiaboliviana* Fleutiaux, 1896, by subsequent designation (Fleutiaux 1936: 292).

Family-group name: ANISCHINAE Fleutiaux, 1936. Stem: *Anischi*-.

***Anisocerus* Faldermann, 1837**: 39. Current status: invalid senior synonym of *Ceratanisus* Gemminger, 1870 in TENEBRIONIDAE: PIMELIINAE: CERATANISINI. Note: junior homonym of *Anisocerus* Lacordaire, 1830 [Coleoptera: CERAMBYCIDAE].

Type species: *Anisocerustristis* Faldermann, 1837, by monotypy.

Family-group name: ANISOCERINI Reitter, 1906. Stem: *Anisocer*-. Note: junior homonym of ANISOCERINI J. Thomson, 1860 (type genus *Anisocerus* Lacordaire, 1830) in Coleoptera: CERAMBYCIDAE; family-group name permanently invalid (ICZN 1999a, Article 39).

***Anisocerus* Lacordaire, 1830b**: 181. Current status: valid genus in CERAMBYCIDAE: LAMIINAE: ANISOCERINI.

Type species: *Anisoceruspenicillatus* Lacordaire, 1830 (= *Lamiascopifera* Germar, 1823), by monotypy.

Family-group name: ANISOCERITAE J. Thomson, 1860. Stem: *Anisocer*-.

***Anisodactylus* Dejean, 1829**: 132. Current status: valid genus in CARABIDAE: HARPALINAE: HARPALINI: ANISODACTYLINA.

Type species: *Carabusbinotatus* Fabricius, 1787, by subsequent designation (Westwood 1838c: 4).

Family-group name: ANISODACTYLIDES Lacordaire, 1854. Stem: *Anisodactyl*-. Note: nomen protectum (see Bouchard et al. 2011: 128).

***Anisodera* Chevrolat in Dejean, 1836a**: 363. Current status: valid genus in CHRYSOMELIDAE: CASSIDINAE: ANISODERINI.

Type species: *Alurnusferrugineus* Fabricius, 1801, by monotypy.

Family-group name: ANISODÉRITES Chapuis, 1875. Stem: *Anisoder*-.

***Anisolemnia* Crotch, 1874a**: 146. Current status: valid genus in COCCINELLIDAE: COCCINELLINAE: COCCINELLINI.

Type species: *Anisolemniacomplicata* Crotch, 1874, by original designation.

Family-group name: ANISOLEMNIINA Mader, 1954. Stem: *Anisolemni*-.

***Anisolinus* Sharp, 1889a**: 113. Current status: valid genus in STAPHYLINIDAE: STAPHYLININAE: STAPHYLININI: ANISOLININA.

Type species: *Anisolinuspicticornis* Sharp, 1889, by subsequent designation (R. Lucas 1920: 98).

Family-group name: ANISOLININA Hayashi, 1993. Stem: *Anisolin*-.

***Anisomeria* Brinck, 1943**: 140. Current status: junior synonym of *Rhantus* Dejean, 1833 in DYTISCIDAE: COLYMBETINAE. Note: new replacement name for *Anisomera* Brullé, 1834.

Type species: *Anisomerabistriata* Brullé, 1834 (= *Rhantusselkirki* Jäch, Balke & Michat, 2014), by monotypy (type fixation for *Anisomera* Brullé).

Family-group name: ANISOMERIINI Brinck, 1948. Stem: *Anisomeri*-.

***Anisomerinus* Voss, 1933**: 53. Current status: valid genus in ATTELABIDAE: RHYNCHITINAE: RHYNCHITITAE: RHYNCHITINI: TEMNOCERINA.

Type species: *Anisomerinusproteae* Voss, 1933, by original designation.

Family-group name: ANISOMERININA Legalov, 2003. Stem: *Anisomerin*-.

***Anisomerus* O. Schwarz, 1897**: 13. Current status: valid genus in ELATERIDAE: ELATERINAE: ANISOMERINI.

Type species: *Dicronychusbipectinatus* O. Schwarz, 1896, by subsequent designation (Hyslop 1921: 627).

Family-group name: ANISOMERINI C. Girard, 2019: 413. Stem: *Anisomer*-. Note: junior homonym of ANISOMERINA Osten-Sacken, 1860 (type genus *Anisomera* Meigen, 1818) in Diptera.

***Anisonychus* Voss, 1927**: 41. Current status: valid genus in ATTELABIDAE: APODERINAE: APODERINI.

Type species: *Apoderusinterstitialis* Voss, 1922, by subsequent designation (Legalov 2003: 555).

Family-group name: ANISONYCHINA Legalov, 2003. Stem: *Anisonych*-. Note: junior homonym of ANISONYCHIDAE Burmeister, 1844 (type genus *Anisonyx* Latreille, 1806) in Coleoptera: SCARABAEIDAE; Burmeister’s name is currently considered a junior synonym.

***Anisonyx* Latreille, 1806**: 119. Current status: valid genus in SCARABAEIDAE: MELOLONTHINAE: HOPLIINI: LEPITHRICHINA.

Type species: *Melolonthacrinita* Fabricius, 1777 (= *Scarabaeuslongipes* Linnaeus, 1764), by subsequent designation (Latreille 1810: 428).

Family-group name: ANISONYCHIDAE Burmeister, 1844. Stem: *Anisonych*-. Note: senior homonym of ANISONYCHINI Legalov, 2003 (type genus *Anisonychus* Voss, 1927) in Coleoptera: ATTELABIDAE.

***Anisoplia* Schönherr, 1817**: 186. Current status: valid genus in SCARABAEIDAE: RUTELINAE: ANOMALINI: ANISOPLIINA.

Type species [suggested]: *Scarabaeusagricola* Poda von Neuhaus, 1761, by subsequent designation (S.I. Medvedev 1949: 239). Note: the valid type species fixation is that of Curtis (1834: pl. 526), who selected *Scarabaeushorticola* Linnaeus, 1758, but that is the type species of the genus *Phyllopertha* Stephens, 1830; a subsequent designation based on an originally included nominal species is that of Westwood (1838c: 24), who selected *Melolonthaaustriaca* Herbst, 1783, but that is the type species of the subgenus Autanisoplia S.I. Medvedev 1949, not the nominotypical subgenus; an application to the Commission is necessary to fix *Scarabaeusagricola* Poda von Neuhaus, 1761 as type species of *Anisoplia* Schönherr, 1817 (see Branco 2007: 5, 7).

Family-group name: ANISOPLIADAE Burmeister, 1844. Stem: *Anisopli*-.

***Anisosphaera* Tömösváry, 1883**: 128. Current status: junior synonym of *Cephennium* P.W.J. Müller, 1822 in STAPHYLINIDAE: SCYDMAENINAE: CEPHENNIITAE: CEPHENNIINI.

Type species: *Anisosphaeraproblematica* Tömösváry, 1883 (= *Cephenniummajus* Reitter, 1882), by monotypy. Note: Tömösváry’s species was described from larval stages as a Thysanura.

Family-group name: ANISOSPHAERIDAE Tömösváry, 1883. Stem: *Anisosphaer*-.

***Anisosticta* Chevrolat in Dejean, 1836a**: 432. Current status: valid genus in COCCINELLIDAE: COCCINELLINAE: COCCINELLINI.

Type species: *Coccinellanovemdecimpunctata* Linnaeus, 1758, by subsequent designation (Crotch 1874a: 93).

Family-group name: ANISOSTICTINA Jakobson, 1915. Stem: *Anisostict*-.

***Anisotarsus* Chaudoir, 1837**: 41. Current status: valid subgenus of *Notiobia* Perty, 1830 in CARABIDAE: HARPALINAE: HARPALINI: ANISODACTYLINA.

Type species: *Anisotarsusbrevicollis* Chaudoir, 1837, by subsequent designation (Casey 1914: 209).

Family-group name: ANISOTARSI Csiki, 1932. Stem: *Anisotars*-.

***Anisotoma* Panzer, 1796**: no 8. Current status: valid genus in LEIODIDAE: LEIODINAE: ANISOTOMINI.

Type species [suggested]: *Volvoxisglabra* Kugelann, 1794, by subsequent designation (Hatch 1929: 50). Note: see Peck et al. (2020: 69) for interpretation of the type species; the valid type species fixation is that of C.G. Thomson (1859: 58), who selected *Anisotomapicea* Panzer, 1796, but that species is currently included in the genus *Leiodes* Latreille, 1797; an application to the Commission is necessary to fix *Volvoxisglabra* Kugelann, 1794 as the type species of *Anisotoma* Panzer, 1796.

Family-group name: ANISOTOMIDAE Horaninow, 1834. Stem: *Anisotom*-.

***Anisoxiella* Nikitsky, 1989**: 42. Current status: valid genus in MELANDRYIDAE: MELANDRYINAE: ANISOXIELLINI.

Type species: *Anisoxyaocularis* Nomura & Katô, 1959, by original designation.

Family-group name: ANISOXIELLINI Nikitsky, 2007. Stem: *Anisoxiell*-.

***Anobium* Fabricius, 1775**: 62. Current status: valid genus in PTINIDAE: ANOBIINAE. Note: name placed on the Official List of Generic Names in Zoology (ICZN 1976).

Type species: *Ptinuspunctatus* DeGeer, 1774, by plenary power (see ICZN 1976). Note: name placed on the Official List of Specific Names in Zoology (ICZN 1976).

Family-group name: ANOBIUMEDAE Fleming, 1821. Stem: *Anobi*-.

***Anochilia* Burmeister, 1842**: 558. Current status: valid genus in SCARABAEIDAE: CETONIINAE: STENOTARSIINI: ANOCHILIINA.

Type species: *Cetonialaevigata* Gory & Percheron, 1835, by subsequent designation (Sakai and Nagai 1998: 175).

Family-group name: ANOCHILIINA Krikken, 1984. Stem: *Anochili*-.

***Anomala* Leach in Samouelle, 1819**: 191. Current status: valid genus in SCARABAEIDAE: RUTELINAE: ANOMALINI. Note: the senior homonym *Anomala* von Block, 1799 [Hymenoptera], and all other uses of the name *Anomala* prior to *Anomala* Leach in Samouelle, 1819, were suppressed for the purposes of the Principle of Priority and the Principle of Homonymy by the Commission and *Anomala* Leach in Samouelle, 1819 was placed on the Official List of Generic Names in Zoology (ICZN 1989c, as *Anomala* Samouelle, 1819).

Type species: *Melolonthafrischii* Fabricius, 1775 (= *Scarabaeusdubius* Scopoli, 1763), by monotypy (see ICZN 1989c). Note: *Melolonthafrischii* Fabricius, 1775 was placed on the Official List of Specific Names in Zoology (ICZN 1989c).

Family-group name: ANOMALIDAE Streubel, 1839. Stem: *Anomal*-. Note: nomen protectum (see A.B.T. Smith 2006: 173).

***Anomalipus* Guérin-Méneville, 1831b**: pl. 29. Current status: valid genus in TENEBRIONIDAE: BLAPTINAE: PLATYNOTINI: PLATYNOTINA. Note: nomen protectum (see Bouchard and Bousquet 2020a: 101); the original spelling was *Anomalipes* but *Anomalipus*, an unjustified emendation by Lacordaire (1859a: 257), is in prevailing usage, attributed to the publication of the original spelling, and therefore deemed to be a justified emendation and the correct original spelling (ICZN 1999a, Article 33.2.3.1).

Type species: *Blapsdentipes* Fabricius, 1794, by monotypy.

Family-group name: ANOMALIPINA C. Koch, 1954. Stem: *Anomalipod*-.

***Anommatus* Wesmael, 1835**: 338. Current status: valid genus in TEREDIDAE: ANOMMATINAE.

Type species: *Anommatusterricola* Wesmael, 1835 (= *Lyctusduodecimstriatus* P.W.J. Müller, 1821), by original designation.

Family-group name: ANOMMATINI Ganglbauer, 1899. Stem: *Anommat*-.

***Anomophthalmus* Fairmaire, 1884**: 498. Current status: valid genus in CURCULIONIDAE: ENTIMINAE: ENTIMITAE: ANOMOPHTHALMINI.

Type species: *Anomophthalmusinsolitus* Fairmaire, 1884, by monotypy.

Family-group name: ANOMOPHTHALMINA Morrone, 1998. Stem: *Anomophthalm*-.

***Anomotarus* Chaudoir, 1875**: 48. Current status: valid genus in CARABIDAE: HARPALINAE: LEBIINI: CALLEIDINA.

Type species: *Anomotarusolivaceus* Chaudoir, 1875 (= *Cymindisillawarrae* W.J. MacLeay, 1873), by monotypy.

Family-group name: ANOMOTARINA Habu, 1967. Stem: *Anomotar*-.

***Anopidium* Jeannel & Paulian, 1945**: 63. Current status: valid genus in TENEBRIONIDAE: DIAPERINAE: GNATHIDIINI: ANOPIDIINA.

Type species: *Anopidiumelgonicum* Jeannel & Paulian, 1945, by original designation.

Family-group name: ANOPIDIINI Jeannel & Paulian, 1945. Stem: *Anopidi*-.

***Anoploderma* Guérin-Méneville, 1840a**: 276. Current status: valid genus in VESPERIDAE: ANOPLODERMATINAE: ANOPLODERMATINI.

Type species: *Anoplodermabicolor* Guérin-Méneville, 1840, by monotypy.

Family-group name: ANOPLODERMIENS Guérin-Méneville, 1840. Stem: *Anoplodermat*-.

***Anoplogenius* Chaudoir, 1852**: 88. Current status: junior synonym of *Loxoncus* Schmidt-Göbel, 1846 in CARABIDAE: HARPALINAE: HARPALINI: STENOLOPHINA.

Type species: *Stenolophusalacer* Dejean, 1831, by subsequent designation (Lacordaire 1854: 304).

Family-group name: ANOPLOGENII Schauberger, 1937. Stem: *Anoplogeni*-.

***Anoplognathus* Leach, 1815b**: [Indexes]. Current status: valid genus in SCARABAEIDAE: RUTELINAE: ANOPLOGNATHINI: ANOPLOGNATHINA. Note: the name listed on page 43 is *Rutela* but it was changed in both the unpaginated Index Systematicus and the General Index to *Anoplognathus*.

Type species: *Melolonthaviridiaenea* Donovan, 1805, by subsequent designation (Carne 1957a: 93).

Family-group name: ANOPLOGNATHIDAE W.S. MacLeay, 1819. Stem: *Anoplognath*-.

***Anoplus* Germar, 1820**: 195. Current status: valid genus in CURCULIONIDAE: MOLYTINAE: ANOPLINI.

Type species: *Curculioplantaris* Naezén, 1794, by monotypy.

Family-group name: ANOPLINI Bedel, 1883. Stem: *Anopl*-.

***Anopsilus* Kirsch, 1870**: 218. Current status: valid genus in CURCULIONIDAE: BARIDINAE: BARIDINI: BARIDINA.

Type species: *Anopsilusbonvouloirii* Kirsch, 1870, by monotypy.

Family-group name: ANOPSILINI Bondar, 1942. Stem: *Anopsil*-.

***Antarctia* Dejean, 1828**: 525. Current status: invalid senior synonym of *Metius* Curtis, 1838 in CARABIDAE: HARPALINAE: PTEROSTICHINI: METIINA. Note: junior homonym of *Antarctia* Hübner, 1820 [Lepidoptera].

Type species: *Carabuscarnifex* Fabricius, 1775, by subsequent designation (Hope 1838a: 86).

Family-group name: ANTARCTIIDES Lacordaire, 1854. Stem: *Antarcti*-. Note: family-group name permanently invalid (ICZN 1999a, Article 39).

***Antennolycus* Bocáková & Bocák, 1999**: 105. Current status: valid genus in LYCIDAE: LYROPAEINAE: ANTENNOLYCINI.

Type species: *Antennolycusconstrictus* Bocáková & Bocák, 1999, by original designation.

Family-group name: ANTENNOLYCINI Bocák & Bocáková, 2008. Stem: *Antennolyc*-.

***Anthaxia* Eschscholtz, 1829b**: 9. Current status: valid genus in BUPRESTIDAE: BUPRESTINAE: ANTHAXIINI. Note: name placed on the Official List of Generic Names in Zoology (ICZN 2002c).

Type species: *Buprestisnitida* Rossi, 1792 (= *Buprestisfulgurans* Schrank, 1789), by plenary power (see ICZN 2002c). Note: *Buprestisfulgurans* Schrank, 1789 was placed on the Official List of Specific Names in Zoology (ICZN 2002c).

Family-group name: ANTHAXIDAE Gory & Laporte, 1839. Stem: *Anthaxi*-.

***Anthaxomorphus* Deyrolle, 1865**: 219. Current status: valid genus in BUPRESTIDAE: AGRILINAE: APHANISTICINI: ANTHAXOMORPHINA.

Type species: *Anthaxomorphuspapuanus* Deyrolle, 1865, by subsequent designation (Cobos 1979: 427).

Family-group name: ANTHAXOMORPHINA Holyński, 1993. Stem: *Anthaxomorph*-.

***Antherophagus* Dejean, 1821**: 45. Current status: valid genus in CRYPTOPHAGIDAE: CRYPTOPHAGINAE: CRYPTOPHAGINI.

Type species: *Tenebriopallens* Linnaeus, 1758, by subsequent designation (Curtis 1835: pl. 546). Note: as noted by C. Johnson et al. (2007: 514) the type species listed by Curtis (1835) was possibly misidentified for *Antherophagussimilis* Curtis, 1835; however, in the absence of clear evidence to this effect, we prefer to recognize the species actually cited by Curtis (1835: pl. 546); both species are currently placed in the genus *Antherophagus* Dejean.

Family-group name: ANTHEROPHAGI J.L. LeConte, 1861. Stem: *Antherophag*-.

***Anthia* Weber, 1801**: 17. Current status: valid genus in CARABIDAE: HARPALINAE: ANTHIINI.

Type species: *Carabussexguttatus* Fabricius, 1775, by subsequent designation (Latreille 1810: 426).

Family-group name: ANTHIES Bonelli, 1813. Stem: *Anthi*-.

***Anthicoclerus* Schenkling, 1906**: 264. Current status: valid genus in CLERIDAE: CLERINAE: CLERITAE: HYDNOCERINI: ANTHICOCLERINA.

Type species: *Clerusanthicoides* A. White, 1849, by monotypy.

Family-group name: ANTHICOCLERINAE Opitz, 2010. Stem: *Anthicocler*-.

***Anthicoxenus* Fairmaire & Germain, 1860a**: 2. Current status: valid genus in MELOIDAE: ELETICINAE: SPASTICINI: ANTHICOXENINA.

Type species: *Anthicoxenusnigroplagiatus* Fairmaire & Germain, 1860, by monotypy.

Family-group name: ANTHICOXENINA Selander, 1966. Stem: *Anthicoxen*-.

***Anthicus* Paykull, 1798**: 253. Current status: valid genus in ANTHICIDAE: ANTHICINAE: ANTHICINI. Note: name placed on the Official List of Generic Names in Zoology (ICZN 2016).

Type species: *Meloeantherinus* Linnaeus, 1761, by subsequent designation (Westwood 1830a: 59; see ICZN 2016, as *Meloeantherinus* Linnaeus, 1760). Note: name placed on the Official List of Specific Names in Zoology (ICZN 2016).

Family-group name: ANTHICITES Latreille, 1819. Stem: *Anthic*-.

***Anthomanes* Gistel, 1848b**: xi, xiv. Current status: junior synonym of *Pyrochroa* Geoffroy, 1762 in PYROCHROIDAE: PYROCHROINAE. Note: unnecessary new replacement name for *Pyrochroa* Geoffroy, 1762.

Type species: *Canthariscoccinea* Linnaeus, 1761, by subsequent designation (Westwood 1838c: 30; see ICZN 1994a) (type fixation for *Pyrochroa* Geoffroy).

Family-group name: ANTHOMANISIDAE Gistel, 1848. Stem: *Anthoman*-.

***Anthonomus* Germar, 1817b**: 340. Current status: valid genus in CURCULIONIDAE: CURCULIONINAE: ANTHONOMINI.

Type species: *Curculioavarus* Fabricius, 1798 (= *Curculiopedicularius* Linnaeus, 1758), by subsequent designation (Kojima and Morimoto 1994: 148).

Family-group name: ANTHONOMINA C.G. Thomson, 1859. Stem: *Anthonom*-.

***Anthophagus* Gravenhorst, 1802**: 120. Current status: valid genus in STAPHYLINIDAE: OMALIINAE: ANTHOPHAGINI. Note: name placed on the Official List of Generic Names in Zoology (ICZN 2004e).

Type species: *Staphylinusalpinus* Fabricius, 1792 (= *Staphylinusalpinus* Paykull, 1790), by subsequent designation (C.G. Thomson 1859: 48; see ICZN 2004e). Note: name placed on the Official List of Specific Names in Zoology (ICZN 2004e).

Family-group name: ANTHOPHAGIDES C.G. Thomson, 1859. Stem: *Anthophag*-.

***Anthotribus* Gistel, 1856**: 260, 375. Current status: junior synonym of *Platystomos* D.H. Schneider, 1791 in ANTHRIBIDAE: ANTHRIBINAE: PLATYSTOMINI. Note: junior homonym of *Anthotribus* Hoffmann, 1803 (unjustified emendation of *Anthribus* Geoffroy, 1862) [Coleoptera: ANTHRIBIDAE].

Type species: *Curculioalbinus* Linnaeus, 1758, by monotypy.

Family-group name: ANTHOTRIBIDAE Gistel, 1856. Stem: *Anthotrib*-. Note: family-group name permanently invalid (ICZN 1999a, Article 39).

***Anthracias* Dejean, 1834**: 205. Current status: junior synonym of *Cryphaeus* Klug, 1833 in TENEBRIONIDAE: TENEBRIONINAE: TOXICINI: TOXICINA.

Type species: *Ulomacornutum* Fischer, 1823, by monotypy.

Family-group name: ANTHRACIINI Houlbert, 1922. Stem: *Anthraci*-.

***Anthracus* Motschulsky, 1850**: vii. Current status: valid genus-group name in CARABIDAE: HARPALINAE: HARPALINI: STENOLOPHINA.

Type species: *Carabusconsputus* Duftschmid, 1812, by original designation.

Family-group name: ANTHRACINI Schuler, 1970. Stem: *Anthrac*-.

***Anthrenocerus* Arrow, 1915**: 443. Current status: junior synonym of *Eurhopalus* Solier, 1849 in DERMESTIDAE: MEGATOMINAE: MEGATOMINI: TROGODERMINA. Note: generic synonymy established by Zhou et al. (2022: 19).

Type species: *Anthrenusaustralis* Hope, 1843, by original designation.

Family-group name: ANTHRENOCERINA Háva, 2015: 1. Stem: *Anthrenocer*-.

***Anthrenus* Geoffroy, 1762**: 113. Current status: valid genus in DERMESTIDAE: MEGATOMINAE: ANTHRENINI. Note: name placed on the Official List of Generic Names in Zoology (ICZN 1994a).

Type species: *Dermestesscrophulariae* Linnaeus, 1758, by subsequent designation (Mroczkowski 1968: 139; see ICZN 1994a). Note: name placed on the Official List of Specific Names in Zoology (ICZN 1994a).

Family-group name: ANTHRENIDAE Gistel, 1848. Stem: *Anthren*-.

***Anthribus* Geoffroy, 1762**: 306. Current status: valid genus in ANTHRIBIDAE: ANTHRIBINAE: ANTHRIBINI. Note: name placed on the Official List of Generic Names in Zoology (ICZN 1994a).

Type species: *Anthribusfasciatus* Forster, 1770, by subsequent designation (Jordan 1931: 285; see ICZN 1994a). Note: name placed on the Official List of Specific Names in Zoology (ICZN 1994a).

Family-group name: ANTHRIBIDES Billberg, 1820. Stem: *Anthrib*-.

***Anthroherpon* Reitter, 1889e**: 294. Current status: valid genus in LEIODIDAE: CHOLEVINAE: LEPTODIRINI: ANTHROHERPONINA.

Type species: *Leptoderuscylindricollis* Apfelbeck, 1889, by subsequent designation (Jeannel 1910: 26).

Family-group name: ANTROHERPONA Jeannel, 1910. Stem: *Anthroherpon*-.

***Anthypna* Eschscholtz, 1818**: 472. Current status: valid genus in GLAPHYRIDAE: AMPHICOMINAE.

Type species [suggested]: *Melolonthacyanipennis* Fabricius, 1801 (= *Melolonthameles* Fabricius, 1792), by subsequent designation (Chapin 1938: 80). Note: the valid type species fixation is that of Crotch (1870b: 231), who selected *Melolonthaursus* Fabricius, 1775, but that species is currently in the genus *Anisonyx* Latreille, 1805 in the tribe HOPLIINI (family SCARABAEIDAE); an application to the Commission is necessary to fix *Melolonthacyanipennis* Fabricius, 1801 as the type species of *Anthypna* Eschscholtz, 1818 in order to conserve the current usage of this genus-group name.

Family-group name: ANTHYPNIDAE Chalande, 1884. Stem: *Anthypn*-.

***Anticheira* Eschscholtz, 1818**: 475. Current status: valid genus in SCARABAEIDAE: RUTELINAE: RUTELINI: RUTELINA.

Type species [suggested]: *Scarabaeusvirens* Drury, 1773, by subsequent designation (Ohaus 1934: 146). Note: the valid type species fixation is that of Duponchel (1841a: 610), who selected *Scarabaeustetradactylus* Linnaeus, 1771, but that species is currently placed in the genus *Macraspis* W.S. MacLeay, 1819; an application to the Commission is necessary to fix *Scarabaeusvirens* Drury, 1773 as the type species of *Anticheira* Eschscholtz, 1818.

Family-group name: ANTICHIRIDES Lacordaire, 1855. Stem: *Anticheir*-.

†***Antigracilus* Zhou, Caterino, Ren & Ślipiński, 2020**: 537. Current status: valid genus in HISTERIDAE: †ANTIGRACILINAE. Note: Lower Cretaceous (China).

Type species: †*Antigraciluscostatus* Zhou, Caterino, Ren & Ślipiński, 2020, by original designation.

Family-group name: †ANTIGRACILINAE Zhou, Caterino, Ren & Ślipiński, 2020: 537. Stem: *Antigracil*-.

***Antillusa* Pace, 2012**: 59. Current status: valid genus in STAPHYLINIDAE: ALEOCHARINAE: ANTILLUSINI.

Type species: *Antillusadominicana* Pace, 2012, by original designation.

Family-group name: ANTILLUSINI Pace, 2012: 59. Stem: *Antillus*-.

***Antimerus* Fauvel, 1878**: 549, 550. Current status: valid genus in STAPHYLINIDAE: STAPHYLININAE: STAPHYLININI: ANTIMERINA.

Type species: *Antimerussmaragdinus* Fauvel, 1878, by monotypy.

Family-group name: ANTIMERINA Brunke, Żyła & Solodovnikov in Brunke et al., 2019: 181. Stem: *Antimer*-.

***Antipha* Baly, 1865a**: 251. Current status: invalid senior synonym of *Dercetina* Gressitt & Kimoto, 1963 in CHRYSOMELIDAE: GALERUCINAE: HYLASPINI. Note: junior homonym of *Antipha* Walker, 1855 [Lepidoptera].

Type species: *Antiphapicipes* Baly, 1865, by original designation.

Family-group name: ANTIPHITES Chapuis, 1875. Stem: *Antiph*-. Note: family-group name permanently invalid (ICZN 1999a, Article 39).

†***Antirhelus* Kirejtshuk in Kirejtshuk et al., 2023a**: 22. Current status: valid genus in KATERETIDAE: †ANTIRHELINAE. Note: Eocene (Rovno amber).

Type species: †*Heterhelusbuzina* Kupryjanowicz, Lyubarsky & Perkovsky, 2021, by original designation.

Family-group name: †ANTIRHELINAE Kirejtshuk in Kirejtshuk et al., 2023a: 21. Stem: *Antirhel*-.

***Antliarhis* Billberg, 1820**: 40. Current status: valid genus in BRENTIDAE: APIONINAE: ANTLIARHINITAE.

Type species: *Curculiohaustellatus* G.-A. Olivier, 1791 (= *Curculiozamiae* Thunberg, 1784), by monotypy.

Family-group name: ANTLIARHINIDES Schönherr, 1823. Stem: *Antliarhin*-.

***Anypotactus* Schönherr, 1840b**: 299. Current status: valid genus in CURCULIONIDAE: ENTIMINAE: POLYDRUSITAE: ANYPOTACTINI.

Type species: *Anypotactusexilis* Boheman, 1840, by original designation.

Family-group name: ANYPOTACTINA Champion, 1911. Stem: *Anypotact*-.

***Aonychus* Schönherr, 1844**: 387. Current status: valid genus in CURCULIONIDAE: BRACHYCERINAE: ERIRHININI.

Type species: *Aonychushopei* Boheman, 1844, by original designation.

Family-group name: AONYCHINI Zimmerman, 1993. Stem: *Aonych*-. Note: junior homonym of AONYCHINI Sokolov, 1973 (type genus *Aonyx* Lesson, 1827) in Mammalia; because of the homonymy, the stem of the genus was changed to *Aonychus*- and the family group name to AONYCHUSINI by Zimmerman in Alonso-Zarazaga and Lyal (1999: 72) but cases involving homonymy of family-group names based on similar genus-group names are to be referred to the Commission for a ruling (ICZN 1985g, 1999a).

***Apalochrus* Erichson, 1840a**: 50. Current status: valid genus in MELYRIDAE: MALACHIINAE: APALOCHRINI.

Type species: *Apalochrusfemoralis* Erichson, 1840, by subsequent designation (C.G. Thomson 1859: 112).

Family-group name: APALOCHRAIRES Mulsant & Rey, 1867. Stem: *Apalochr*-.

***Apalus* Fabricius, 1775**: 127. Current status: valid genus in MELOIDAE: NEMOGNATHINAE: NEMOGNATHINI: SITARINA.

Type species: *Meloebimaculatus* Linnaeus, 1761, by monotypy.

Family-group name: APALIDES J.B. Parker & Böving, 1924. Stem: *Apal*-.

***Apate* Fabricius, 1775**: 54. Current status: valid genus in BOSTRICHIDAE: BOSTRICHINAE: APATINI. Note: nomen protectum (see Borowski and Węgrzynowicz 2009: 190).

Type species: **here fixed** (under ICZN 1999a, Article 70.3.2) as *Ligniperdaterebrans* Pallas, 1772, misidentified as *Dermestesmuricatus* Linnaeus, 1767 (sensu Fabricius, 1775), in the subsequent designation by Gorham (1883a: 212).

Family-group name: APATIDES Billberg, 1820. Stem: *Apat*-.

***Apatetica* Westwood, 1848a**: 86. Current status: valid genus in STAPHYLINIDAE: APATETICINAE.

Type species: *Apateticalebioides* Westwood, 1848, by monotypy.

Family-group name: APATETICAE Fauvel, 1895. Stem: *Apatetic*-.

***Apatides* Casey, 1898**: 66, 70. Current status: valid genus in BOSTRICHIDAE: BOSTRICHINAE: BOSTRICHINI.

Type species: *Amphicerusfortis* J.L. LeConte, 1866, by subsequent designation (Lesne 1900: 47).

Family-group name: APATIDINI Bradley, 1930. Stem: *Apatid*-.

***Apatophysis* Chevrolat, 1860a**: 95. Current status: valid genus in CERAMBYCIDAE: CERAMBYCINAE: DORCASOMINI.

Type species: *Apatophysistoxotoides* Chevrolat, 1860 (= *Polyarthronbarbarum* P.H. Lucas, 1858), by monotypy. Note: combined description of a new genus and a single new species (ICZN 1999a, Article 12.2.6).

Family-group name: APATOPHYSIDES Lacordaire, 1869. Stem: *Apatophyse*-.

***Apenes* J.L. LeConte, 1851a**: 174. Current status: valid genus in CARABIDAE: HARPALINAE: LEBIINI: APENINA. NOTE: nomen protectum (see Ball and Shpeley 2009: 95).

Type species: *Cymindislucidula* Dejean, 1831, by subsequent designation (Lacordaire 1854: 110).

Family-group name: APENINA Ball, 1983. Stem: *Apen*-.

***Aphaenostemmus* Peyerimhoff, 1914**: 245. Current status: valid genus in STAPHYLINIDAE: OMALIINAE: APHAENOSTEMMINI.

Type species: *Aphaenostemmusbordei* Peyerimhoff, 1914, by monotypy.

Family-group name: APHAENOSTEMMINI Peyerimhoff, 1914. Stem: *Aphaenostemm*-.

***Aphanasium* Dejean, 1835**: 322. Current status: valid genus in CERAMBYCIDAE: CERAMBYCINAE: APHANASIINI.

Type species: *Callidiumaustrale* Boisduval, 1835, by monotypy.

Family-group name: APHANASIIDES Lacordaire, 1868. Stem: *Aphanasi*-.

***Aphanisticus* Latreille, 1810**: 169. Current status: valid genus in BUPRESTIDAE: AGRILINAE: APHANISTICINI: APHANISTICINA.

Type species: *Buprestisemarginata* Fabricius, 1792 (= *Buprestisemarginata* G.-A. Olivier, 1790), by monotypy.

Family-group name: APHANISTICITES Jacquelin du Val, 1859. Stem: *Aphanistic*-.

***Aphanocephalus* Wollaston, 1873a**: 278. Current status: valid genus in DISCOLOMATIDAE: APHANOCEPHALINAE.

Type species: *Aphaenocephalushemisphaericus* Wollaston, 1873, by monotypy.

Family-group name: APHANOCEPHALIDAE Jakobson, 1904. Stem: *Aphanocephal*-.

***Aphela* Pascoe, 1865b**: 416. Current status: valid genus in CURCULIONIDAE: CYCLOMINAE: NOTIOMIMETINI.

Type species: *Aphelahelopoides* Pascoe, 1865, by monotypy.

Family-group name: APHELI Csiki, 1936. Stem: *Aphel*-.

***Aphneope* Pascoe, 1863b**: 567. Current status: valid genus in CERAMBYCIDAE: CERAMBYCINAE: APHNEOPINI.

Type species: *Aphneopesericata* Pascoe, 1863, by monotypy.

Family-group name: APHNÉOPIDES Lacordaire, 1868. Stem: *Aphneop*-.

***Aphodius* Hellwig, 1798**: 101. Current status: valid genus in SCARABAEIDAE: APHODIINAE: APHODIINI: APHODIINA. Note: see Alonso-Zarazaga and Krell (2011: 67) for comments on the authorship of this genus-group name.

Type species: *Scarabaeusfimetarius* Linnaeus, 1758, by subsequent designation (Latreille 1810: 428). Note: name placed on the Official List of Specific Names in Zoology (ICZN 2014b).

Family-group name: APHODIDA Leach, 1815. Stem: *Aphodi*-.

***Aphrastus* Schönherr in Say, 1832**: 9. Current status: valid genus in CURCULIONIDAE: ENTIMINAE: OTIORHYNCHITAE: PHYLLOBIINI. Note: see Prena (2018: 331) for notes on the authorship of this genus-group name.

Type species: *Aphrastustaeniatus* Say, 1832, by monotypy. Note: combined description of a new genus and a single new species (ICZN 1999a, Article 12.2.6).

Family-group name: APHRASTI J.L. LeConte, 1874. Stem: *Aphrast*-.

***Aphricus* J.L. LeConte, 1853f**: 501. Current status: valid genus in ELATERIDAE: CARDIOPHORINAE.

Type species: *Aphricuscalifornicus* J.L. LeConte, 1853, by monotypy.

Family-group name: APHRICI J.L. LeConte, 1861. Stem: *Aphric*-.

***Aphthona* Chevrolat in Dejean, 1836a**: 391. Current status: valid genus in CHRYSOMELIDAE: GALERUCINAE: ALTICINI.

Type species: *Halticacyparissiae* Knoch, 1803, by subsequent designation (Maulik 1926: 366).

Family-group name: APHTHONITES Chapuis, 1875. Stem: *Aphthon*-.

***Aphyllura* Reitter in Brenske and Reitter, 1884**: 97. Current status: valid genus in CURCULIONIDAE: COSSONINAE: RHYNCOLINI: PSEUDOMIMINA.

Type species: *Aphyllurabrenskei* Reitter, 1884, by monotypy.

Family-group name: APHYLLURINA Voss, 1955. Stem: *Aphyllur*-.

†***Aphytocerus* Zherichin, 1977**: 131. Current status: valid genus in CEROPHYTIDAE. Note: Upper Cretaceous (Siberia, Russia).

Type species: †*Aphytoceruscommunis* Zherichin, 1977, by original designation.

Family-group name: †APHYTOCERINAE Kirejtshuk in Kirejtshuk and Azar, 2013: 128. Stem: *Aphytocer*-.

***Aphytopus* Sharp, 1886**: 375. Current status: valid genus in STAPHYLINIDAE: ALEOCHARINAE: OXYPODINI: APHYTOPODINA.

Type species: *Aphytopusgracilis* Sharp, 1886, by monotypy.

Family-group name: APHYTOPI Bernhauer & Scheerpeltz, 1926. Stem: *Aphytopod*-.

***Apiomorphus* Wagner, 1912c**: 33. Current status: valid genus in BRENTIDAE: APIONINAE: TANAITAE: APIOMORPHINI.

Type species: *Apiomorphuscyaneus* Wagner, 1912 (= *Apioneximium* Beguin-Billecocq, 1910), by monotypy.

Family-group name: APIOMORPHINI Legalov, 2023c: 30. Stem: *Apiomorph*-. Note: APIOMORPHINILegalov (2018c: 810) was published in a work that is nomenclaturally unavailable (ICZN 2012f, Article 8.5.3).

***Apion* Herbst, 1797**: 100. Current status: valid genus in BRENTIDAE: APIONINAE: APIONITAE: APIONINI: APIONINA.

Type species: *Curculiofrumentarius* Linnaeus, 1758, by subsequent designation (Latreille 1810: 430).

Family-group name: APIONIDES Schönherr, 1823. Stem: *Apion*-.

***Apistus* Agassiz, 1846**: 29. Current status: invalid senior synonym of *Rhopalocerus* W. Redtenbacher, 1842 in ZOPHERIDAE: COLYDIINAE: RHOPALOCERINI. Note: unjustified emendation of *Apeistus* Motschulsky, 1840; junior homonym of *Apistus* Cuvier, 1829 [Pisces]; *Apistus* Agassiz, 1846 and *Apeistus* Motschulsky, 1840 were suppressed for the Principle of Priority and placed on the Official Index of Rejected and Invalid Names in Zoology (ICZN 1986b).

Type species: *Monotomarondanii* A. Villa & G.B. Villa, 1833, by monotypy (type fixation for *Spartycerus* Motschulsky, 1837). Note: name placed on the Official List of Specific Names in Zoology (ICZN 1986b).

Family-group name: APISTINI Ganglbauer, 1899. Stem: *Apist*-. Note: family-group name permanently invalid (ICZN 1999a, Article 39); name placed on the Official List of Rejected and Invalid Family-Group Names in Zoology (see ICZN 1986b).

***Aplastus* J.L. LeConte, 1859a**: 73. Current status: valid genus in ELATERIDAE: ELATERINAE: CEBRIONINI: APLASTINA. Note: nomen protectum (see Appendix 2).

Type species: *Aplastussperatus* J.L. LeConte, 1859, by monotypy.

Family-group name: APLASTINAE Stibick, 1979. Stem: *Aplast*-.

***Aplemonus* Schönherr, 1847**: 5. Current status: valid genus in BRENTIDAE: APIONINAE: APIONITAE: APLEMONINI.

Type species: *Aplemonusgibbipennis* Schönherr, 1847, by original designation. Note: combined description of a new genus-group taxon and a single new nominal species (ICZN 1999a, Article 12.2.6).

Family-group name: APLEMONINI Kissinger, 1968. Stem: *Aplemon*-.

***Aplocnemus* Stephens, 1830**: 311, 316. Current status: valid genus in RHADALIDAE: RHADALINAE.

Type species: *Criocerisimpressa* Marsham, 1802, by original designation.

Family-group name: HAPLOCNÉMATES Mulsant & Rey, 1868. Stem: *Aplocnem*-.

***Aploderus* Stephens, 1833b**: 273. Current status: valid genus in STAPHYLINIDAE: OXYTELINAE: THINOBIINI.

Type species: *Staphylinusbrachypterus* Marsham, 1802 (= *Oxyteluscaelatus* Gravenhorst, 1802), by subsequent monotypy (Stephens 1834: 321).

Family-group name: APLODERINI Ádám, 2001. Stem: *Aploder*-.

***Aploglossa* Guérin-Méneville, 1849** [no 33]: 1. Current status: valid genus in PTILODACTYLIDAE: APLOGLOSSINAE.

Type species: *Aploglossasallei* Guérin-Méneville, 1849, by **present designation**.

Family-group name: HAPLOGLOSSINI Champion, 1897. Stem: *Aplogloss*-.

***Aplonycha* Boisduval, 1835**: 193. Current status: valid genus in SCARABAEIDAE: SERICOIDINAE: LIPARETRINI. Note: originally proposed as a synonym of *Melolontha* Fabricius, 1775 and subsequently treated as valid before 1961 by several authors, including Duponchel and Chevrolat (1841: 16).

Type species: *Melolonthaobesa* Boisduval, 1835, by subsequent designation (Duponchel and Chevrolat 1841: 16). Note: Britton (1986: 4) mentioned that the name-bearing type of *Melolonthaobesa* has been lost and the species is unidentifiable based on the brief original description.

Family-group name: HAPLONYCHIDAE Burmeister, 1855. Stem: *Aplonych*-. Note: senior homonym of APLONYCHINI De Stefani, 1908 (type genus *Aplonyx* De Stefani, 1908) in Diptera.

***Aplothorax* G.R. Waterhouse, 1841a**: 145. Current status: valid genus in CARABIDAE: CARABINAE: CARABINI: CALOSOMATINA.

Type species: *Aplothoraxburchellii* G.R. Waterhouse, 1841, by monotypy. Note: combined description of a new genus-group taxon and a single new nominal species (ICZN 1999a, Article 12.2.6).

Family-group name: APLOTHORACINA Lapouge, 1927. Stem: *Aplothorac*-.

***Apocellus* Erichson, 1839b**: 30. Current status: valid genus in STAPHYLINIDAE: OXYTELINAE: OXYTELINI.

Type species: *Lathrobiumsphaericolle* Say, 1831, by subsequent designation (Duponchel and Chevrolat 1841: 22). Note: no species were originally included in this genus by Erichson (1839b); the first ones were the three species described by Erichson (1840b: 813–814).

Family-group name: APOCELLARIA Lynch Arribálzaga, 1884. Stem: *Apocell*-.

***Apocrypha* Eschscholtz, 1831**: 13. Current status: valid genus in TENEBRIONIDAE: TENEBRIONINAE: APOCRYPHINI.

Type species: *Apocryphaanthicoides* Eschscholtz, 1831, by monotypy.

Family-group name: APOCRYPHIDES Lacordaire, 1859. Stem: *Apocryph*-.

***Apodasya* Pascoe, 1863a**: 54. Current status: valid genus in CERAMBYCIDAE: LAMIINAE: DESMIPHORINI. Note: name placed on the Official List of Generic Names in Zoology (ICZN 2011d).

Type species: *Apodasyapilosa* Pascoe, 1863 (= *Chaetosomapilosum* Chevrolat, 1843), by original designation (see ICZN 2011d). Note: *Chaetosomapilosum* Chevrolat, 1843 was placed on the Official List of Specific Names in Zoology (ICZN 2011d).

Family-group name: APODASYIDES Lacordaire, 1872. Stem: *Apodasy*-.

***Apoderiger* Wasmann, 1897**: 263. Current status: valid genus in STAPHYLINIDAE: PSELAPHINAE: CLAVIGERITAE: CLAVIGERINI.

Type species: *Apoderigercervinus* Wasmann, 1897, by monotypy.

Family-group name: APODERIGERINI Jeannel, 1954. Stem: *Apoderiger*-.

***Apoderus* G.-A. Olivier, 1807**: 12. Current status: valid genus in ATTELABIDAE: APODERINAE: APODERINI.

Type species: *Attelabuscoryli* Linnaeus, 1758, by subsequent designation (Latreille 1810: 430).

Family-group name: APODERIDAE Jekel, 1860. Stem: *Apoder*-.

***Apodytapion* Wanat, 2021**: 23. Current status: valid genus in BRENTIDAE: APIONINAE incertae sedis.

Type species: *Apodytapionstepniewskii* Wanat, 2021, by original designation.

Family-group name: APODYTAPIINI Wanat, 2021: 56. Stem: *Apodytapi*-.

***Apogonia* W. Kirby, 1819**: 401. Current status: valid genus in SCARABAEIDAE: MELOLONTHINAE: DIPLOTAXINI.

Type species: *Apogoniagemellata* W. Kirby, 1819 (= *Melolontharauca* Fabricius, 1781), by monotypy.

Family-group name: APOGONIITAE Blanchard, 1851. Stem: *Apogoni*-.

***Apolecta* Pascoe, 1859c**: 431. Current status: valid genus in ANTHRIBIDAE: ANTHRIBINAE: APOLECTINI.

Type species: *Mecocerusparvulus* J. Thomson, 1858, by original designation.

Family-group name: APOLECTIDES Lacordaire, 1865. Stem: *Apolect*-. Note: senior homonym of APOLECTIDAE Jordan, 1923 (type genus *Apolectus* Cuvier, 1832) in Pisces; Jordan’s name is permanently invalid (based on a preoccupied type genus).

***Apoleon* Gorham, 1885a**: 51. Current status: valid genus in BOSTRICHIDAE: DYSIDINAE.

Type species: *Apoleonedax* Gorham, 1885, by monotypy.

Family-group name: APOLEONINAE Gardner, 1933. Stem: *Apoleont*-.

***Apolites* Jacquelin du Val, 1861**: 324. Current status: invalid senior synonym of *Ceratanisus* Gemminger, 1870 in TENEBRIONIDAE: PIMELIINAE: CERATANISINI. Note: junior homonym of *Apolites* Sundevall, 1835 [Aves].

Type species: *Helopsmucoreus* Waltl, 1838, by monotypy.

Family-group name: APOLITINA Seidlitz, 1895. Stem: *Apolit*-. Note: family-group name permanently invalid (ICZN 1999a, Article 39).

***Apomecyna* Dejean, 1821**: 108. Current status: valid genus in CERAMBYCIDAE: LAMIINAE: APOMECYNINI.

Type species: *Saperdaalboguttata* Megerle, 1802 (= *Lamiahistrio* Fabricius, 1792), by monotypy. Note: *Saperdaalboguttata* Megerle, 1802 was placed on the Official List of Specific Names in Zoology (ICZN 1993a, as *Saperdaalboguttata* Megerle, 1803).

Family-group name: APOMECYNITAE J. Thomson, 1860. Stem: *Apomecyn*-.

***Apophylia* Duponchel & Chevrolat, 1841**: 31. Current status: valid genus in CHRYSOMELIDAE: GALERUCINAE: GALERUCINI. Note: this genus is often credited to J. Thomson (1858b: 221) in the literature but it was first made available by Duponchel and Chevrolat (1841: 31) when they provided a description of this new taxon; the three species associated with the name *Apophylia* in Duponchel and Chevrolat (1841: 31) are nomina nuda and the first available species associated with the name was established by J. Thomson (1858b: 221).

Type species: *Apophyliachloroptera* J. Thomson, 1858, by subsequent monotypy (J. Thomson 1858b: 221).

Family-group name: APOPHYLIITES Chapuis, 1875. Stem: *Apophyli*-.

†***Apophisandra* Molino-Olmedo, 2017**: 170. Current status: valid genus in †APOPHISANDRIDAE. Note: mid-Cretaceous (Myanmar).

Type species: †*Apophisandraammytae* Molino-Olmedo, 2017, by original designation. Note: combined description of a new genus and a single new species (ICZN 1999a, Article 13.4).

Family-group name: †APOPHISANDRINI Molino-Olmedo, 2017: 173. Stem: *Apophisandr*-.

***Apostasimerus* Schönherr, 1844**: 101. Current status: valid genus in CURCULIONIDAE: BARIDINAE: APOSTASIMERINI: APOSTASIMERINA.

Type species: *Apotasimerusserrirostris* Boheman, 1844, by original designation.

Family-group name: APOSTASIMERIDES Schönherr, 1844. Stem: *Apostasimer*-.

†***Apotomoura* Bao, Walczyńska, Moody, B. Wang & Rust, 2018**: 16. Current status: valid genus in †APOTOMOURIDAE. Note: mid-Cretaceous (Myanmar).

Type species: †*Apotomourafortiscrura* Bao, Walczyńska, Moody, B. Wang & Rust, 2018, by monotypy.

Family-group name: †APOTOMOURIDAE Bao, Walczyńska, Moody, B. Wang & Rust, 2018: 16. Stem: *Apotomour*-.

***Apotomus* Illiger, 1807b**: 348. Current status: valid genus in CARABIDAE: APOTOMINAE.

Type species: *Scaritesrufus* Rossi, 1790, by monotypy.

Family-group name: APOTOMI J.L. LeConte, 1853. Stem: *Apotom*-.

***Apphianus* Beal, 2006**: 489. Current status: valid genus in DERMESTIDAE: ATTAGENINAE incertae sedis.

Type species: *Apphianusyuccae* Beal, 2006, by monotypy.

Family-group name: APPHIANINI Háva in Zahradnik and Háva, 2014: 306. Stem: *Apphian*-.

***Aproida* Pascoe, 1863a**: 55. Current status: valid genus in CHRYSOMELIDAE: CASSIDINAE: APROIDINI.

Type species: *Aproidabalyi* Pascoe, 1863, by monotypy.

Family-group name: APROIDINI Weise, 1911. Stem: *Aproid*-.

***Aprosopus* C. d’Orbigny, 1841**: 40. Current status: valid genus in CERAMBYCIDAE: LAMIINAE: AGAPANTHIINI.

Type species: *Aprosopusbuquetii* C. d’Orbigny, 1841, by original designation.

Family-group name: APROSOPITAE J. Thomson, 1864. Stem: *Aprosop*-.

***Apteroessa* Hope, 1838a**: 13, 159. Current status: valid genus in CICINDELIDAE: CICINDELINI: APTEROESSINA.

Type species: *Cicindelagrossa* Fabricius, 1781, by original designation.

Family-group name: APTEROESSINA Rivalier, 1971. Stem: *Apteroess*-.

***Apterospasta* J.L. LeConte, 1862**: 272. Current status: junior synonym of *Epicauta* Dejean, 1834 in MELOIDAE: MELOINAE: EPICAUTINI.

Type species: *Lyttasegmenta* Say, 1824, by subsequent designation (Wellman 1910a: 393).

Family-group name: APTEROSPASTIDES Wellman, 1910. Stem: *Apterospast*-.

***Aptila* Fåhraeus, 1870**: 258. Current status: valid genus in TENEBRIONIDAE: BLAPTINAE: PEDININI: HELOPININA.

Type species: *Aptilacostata* Fåhraeus, 1870, by subsequent designation (R. Lucas 1920: 115).

Family-group name: APTILINA C. Koch, 1958. Stem: *Aptil*-.

***Aptinus* Bonelli, 1810**: Tab. Syn. Current status: valid genus in CARABIDAE: BRACHININAE: BRACHININI: APTININA.

Type species: *Brachinusmutilatus* Fabricius, 1801 (= *Carabusbombarda* Illiger, 1800), by subsequent monotypy (Panzer 1813: 71).

Family-group name: APTINIDAE Gistel, 1848. Stem: *Aptin*-.

***Aptopus* Eschscholtz, 1829a**: 32. Current status: valid genus in ELATERIDAE: CARDIOPHORINAE.

Type species: *Aptopustibialis* Eschscholtz, 1829, by subsequent designation (Hyslop 1921: 629).

Family-group name: APTOPINA Jakobson, 1913. Stem: *Aptopod*-.

***Aquilex* Moret, 1990**: 246. Current status: valid genus in CARABIDAE: MIGADOPINAE: MIGADOPINI: AQUILICINA.

Type species: *Aquilexdiabolicola* Moret, 1990, by original designation.

Family-group name: AQUILICINA Moret, 2005. Stem: *Aquilic*-.

***Arachnopus* Boisduval, 1835**: 435. Current status: junior synonym of *Arachnobas* Boisduval, 1835 in CURCULIONIDAE: MOLYTINAE: ARACHNOPODINI. Note: this name has been credited to Guérin-Méneville (1838: 127) in the literature but it was first published in an available work by Boisduval (1835: 435) as a junior synonym of *Arachnobas* Boisduval, 1835; since the name has been treated as available before 1961 (e.g., Guérin-Méneville 1838: 127) it dates from its first publication as a synonym (ICZN 1999a, Article 11.6.1); we act as First Revisers (ICZN 1999a, Article 24.2.3) and select *Arachnobas* Boisduval, 1835 to have precedence over *Arachnopus* Boisduval, 1835.

Type species: *Cryptorhynchusstriga* Guérin-Méneville, 1831, by subsequent designation (Lacordaire 1865: 160).

Family-group name: ARACHNOPIDES Lacordaire, 1865. Stem: *Arachnopod*-.

***Araecerus* Schönherr, 1823**: 1135. Current status: valid genus in ANTHRIBIDAE: CHORAGINAE: ARAECERINI.

Type species: *Anthribuscoffeae* Fabricius, 1801 (= *Curculiofasciculatus* DeGeer, 1775), by original designation.

Family-group name: ARAEOCÉRIDES Lacordaire, 1865. Stem: *Araecer*-.

***Araeocnemus* Nordmann, 1837**: 7, 163. Current status: junior synonym of *Plochionocerus* Dejean, 1833 in STAPHYLINIDAE: STAPHYLININAE: XANTHOLININI.

Type species: *Staphylinusfulgens* Fabricius, 1792, by subsequent designation (Duponchel and Chevrolat 1841: 64).

Family-group name: ARAEOCNEMES Casey, 1906. Stem: *Araeocnem*-.

***Araeopidius* Cockerell, 1906**: 241. Current status: valid genus in PTILODACTYLIDAE: ARAEOPIDIINAE. Note: new replacement name for *Araeopus* J.L. LeConte, 1874.

Type species: *Araeopusmonachus* J.L. LeConte, 1874, by monotypy (type fixation for *Araeopus* J.L. LeConte).

Family-group name: ARAEOPIDIINAE Lawrence, 1991. Stem: *Araeopidi*-.

***Araeoschizus* J.L. LeConte, 1851a**: 138. Current status: valid genus in TENEBRIONIDAE: PIMELIINAE: STENOSINI: ARAEOSCHIZINA.

Type species: *Araeoschizuscostipennis* J.L. LeConte, 1851, by monotypy.

Family-group name: ARAEOSCHIZINI Casey, 1907. Stem: *Araeoschiz*-.

***Araptus* Eichhoff, 1872**: 136. Current status: valid genus in CURCULIONIDAE: SCOLYTINAE: CORTHYLINI: PITYOPHTHORINA.

Type species: *Araptusrufopalliatus* Eichhoff, 1872, by monotypy.

Family-group name: ARAPTIDAE Eichhoff, 1878. Stem: *Arapt*-.

***Aratea* Lacordaire, 1848**: 467. Current status: valid genus in CHRYSOMELIDAE: CRYPTOCEPHALINAE: CLYTRINI: ARATEINA.

Type species: *Arateacostata* Lacordaire, 1848, by monotypy.

Family-group name: ARATEINI Moldenke, 1981. Stem: *Arate*-.

***Araucarius* Kuschel, 1966**: 7. Current status: valid genus in CURCULIONIDAE: COSSONINAE: ARAUCARIINI.

Type species: *Araucariusmedius* Kuschel, 1966, by original designation.

Family-group name: ARAUCARIINI Kuschel, 1966. Stem: *Araucari*-.

***Archaeoglenes* Broun, 1893a**: 188. Current status: valid genus in TENEBRIONIDAE: PHRENAPATINAE: ARCHAEOGLENINI.

Type species: *Archaeoglenescostipennis* Broun, 1893, by monotypy.

Family-group name: ARCHAEOGLENINI Watt, 1975. Stem: *Archaeoglen*-.

†***Archaeoluprops* Nabozhenko, Perkovsky & Nazarenko, 2023**: 585. Current status: valid genus in TETRATOMIDAE: TETRATOMINAE: †ARCHAEOLUPROPINI. Note: combined description of a new family-group name and a single new genus (ICZN 1999a, Article 13.5). Note: Eocene (Baltic amber).

Type species: †*Archaeolupropsgroehni* Nabozhenko, Perkovsky & Nazarenko, 2023, by monotypy.

Family-group name: †ARCHAEOLUPROPINI Nabozhenko, Perkovsky & Nazarenko, 2023: 585. Stem: *Archaeoluprop*-.

***Archarias* A. Villa & G.B. Villa, 1833**: 23. Current status: invalid senior synonym of *Balanobius* Jekel, 1861 in CURCULIONIDAE: CURCULIONINAE: CURCULIONINI: BALANOBIINA. Note: junior homonym of *Archarias* Dejean, 1821 [Coleoptera: CURCULIONIDAE]; *Archarias* Dejean, 1821 was suppressed for the purposes of the Principle of Priority but not for those of the Principle of Homonymy (ICZN 1987d); see Alonso-Zarazaga et al. (2023: 8) for an alternative interpretation.

Type species: *Curculiosalicivorus* Paykull, 1792, by subsequent designation (Alonso-Zarazaga and Lyal 1999: 21, 73).

Family-group name: ARCHARIINA Pelsue & O’Brien, 2011: 30. Stem: *Archari*-. Note: this family-group name was based on “*Archarius* Gistel, 1856”, which is an incorrect spelling of *Archarias* A. Villa & G.B. Villa, 1833, introduced as a valid name erroneously by Alonso-Zarazaga and Lyal (1999: 73); family-group name permanently invalid (ICZN 1999a, Article 39).

***Archeocrypticus* Kaszab, 1964**: 360. Current status: valid genus in ARCHEOCRYPTICIDAE.

Type species: *Archeocrypticustopali* Kaszab, 1964, by original designation.

Family-group name: ARCHEOCRYPTICINI Kaszab, 1964. Stem: *Archeo­cryptic*-.

†***Archescarabaeus* Nikolajev, 2010**: 69. Current status: valid genus in PLEOCOMIDAE: †ARCHESCARABAEINAE. Note: Lower Cretaceous (Transbai­kalia).

Type species: †*Archescarabaeusmesozoicus* Nikolajev, 2010, by original designation.

Family-group name: †ARCHESCARABAEINAE Nikolajev, 2010. Stem: *Archescarabae*-.

***Archetypus* J. Thomson, 1861**: 292, 319. Current status: valid genus in CERAMBYCIDAE: PRIONINAE: MACROTOMINI: ARCHETYPINA.

Type species: *Archetypusparandroides* J. Thomson, 1861 (= *Mallodonfulvipenne* Pascoe, 1859), by subsequent designation (J. Thomson 1864: 307).

Family-group name: ARCHETYPI Lameere, 1912. Stem: *Archetyp*-.

***Archeuops* Legalov, 2003**: 360. Current status: valid genus in ATTELABIDAE: ATTELABINAE: EUOPINI.

Type species: *Euopsvossi* Heller, 1929, by original designation.

Family-group name: ARCHEUOPSINA Legalov, 2003. Stem: *Archeuops*- (ICZN 1999a, Article 29.4).

***Archicorynus* R.S. Anderson & Marvaldi, 2013**: 66. Current status: valid genus in BELIDAE: OXYCORYNINAE: OXYCORYNITAE: ARCHICORYNINI.

Type species: *Archicorynuskuscheli* R.S. Anderson & Marvaldi, 2013, by original designation.

Family-group name: ARCHICORYNINI R.S. Anderson & Marvaldi, 2013: 66. Stem: *Archicoryn*-.

***Archolabus* Voss, 1929b**: 200. Current status: junior synonym of *Microstylus* Schönherr, 1847 in CURCULIONIDAE: CURCULIONINAE: MICROSTYLINI.

Type species: *Archolabusmethneri* Voss, 1929, by original designation.

Family-group name: ARCHOLABINAE Voss, 1929. Stem: *Archolab*-.

***Ardella* Paulsen, 2021**: 2. Current status: valid genus in SCARABAEIDAE: MELOLONTHINAE: ARDELLINI.

Type species: *Ardellamagnaemirabilis* Paulsen, 2021, by original designation.

Family-group name: ARDELLINI Paulsen, 2021: 5. Stem: *Ardell*-.

***Ardistomis* Putzeys, 1846**: 2, 118. Current status: valid genus in CARABIDAE: SCARITINAE: CLIVININI: ARDISTOMINA.

Type species: *Ardistomisfasciolata* Putzeys, 1846, by subsequent designation (E. Desmarest 1851: 102).

Family-group name: ARDISTOMIDES Putzeys, 1867. Stem: *Ardistom*-.

***Areoda* W.S. MacLeay, 1819**: 158. Current status: valid genus in SCARABAEIDAE: RUTELINAE: RUTELINI: AREODINA.

Type species: *Areodaleachii* W.S. MacLeay, 1819, by monotypy.

Family-group name: AREODIDAE Burmeister, 1844. Stem: *Areod*-.

***Arescus* Perty, 1832**: 100. Current status: valid genus in CHRYSOMELIDAE: CASSIDINAE: ARESCINI.

Type species: *Arescuslabiatus* Perty, 1832, by monotypy.

Family-group name: ARESCITES Chapuis, 1875. Stem: *Aresc*-.

***Argentinomacer* Legalov, 2023c**: 21. Current status: valid genus in NEMONYCHIDAE: RHINORHYNCHINAE: ARGENTINOMACERINI. Note: *Argentinomacer*Legalov (2017a: 74) was published in a work that is nomenclaturally unavailable (ICZN 2012f, Article 8.5.3).

Type species: *Argentinomacerunicus* Legalov, 2023, by original designation. Note: *Argentinomacerunicus* Legalov, 2017 was published in a work that is nomenclaturally unavailable (ICZN 2012f, Article 8.5.3).

Family-group name: ARGENTINOMACERINI Legalov, 2023c: 21. Stem: *Argentinomacer*-. Note: ARGENTINOMACERINILegalov (2017a: 73) was published in a work that is nomenclaturally unavailable (ICZN 2012f, Article 8.5.3).

***Argentipilosa* Gordon & de Almeida, 1991**: 150. Current status: valid genus in COCCINELLIDAE: COCCINELLINAE: ARGENTIPILOSINI.

Type species: *Argentipilosanigra* Gordon & de Almeida, 1991, by original designation.

Family-group name: ARGENTIPILOSINI Gordon & de Almeida, 1991. Stem: *Argentipilos*-.

***Arhina* Murray, 1867a**: 178. Current status: invalid senior synonym of *Arhinops* Kirejtshuk & Bouchard, 2018 in NITIDULIDAE: CRYPTARCHINAE: ARHINOPINI. Note: junior homonym of *Arhina* Agassiz, 1846 [Diptera].

Type species: *Arhinastrongyloides* Murray, 1867, by monotypy.

Family-group name: ARHININI Kirejtshuk, 1987. Stem: *Arhin*-. Note: family-group name permanently invalid (ICZN 1999a, Article 39).

***Arhinops* Kirejtshuk & Bouchard, 2018**: 157. Current status: valid genus in NITIDULIDAE: CRYPTARCHINAE: ARHINOPINI. Note: new replacement name for *Arhina* Murray, 1867.

Type species: *Arhinastrongyloides* Murray, 1867, by monotypy (type fixation for *Arhina* Murray).

Family-group name: ARHINOPINI Kirejtshuk & Bouchard, 2018: 157. Stem: *Arhinop*-.

***Arhytodes* Reitter, 1882a**: 193, 209. Current status: junior synonym of *Rhytus* Westwood, 1870 in STAPHYLINIDAE: PSELAPHINAE: PSELAPHITAE: ARHYTODINI. Note: unnecessary new replacement name for *Rhytus* Westwood, 1870.

Type species: *Rhytusvestitus* Westwood, 1870, by monotypy (type fixation for *Rhytus* Westwood).

Family-group name: ARHYTODINI Raffray, 1890. Stem: *Arhytod*-.

***Aristochroodes* Marcilhac, 1993**: 273. Current status: valid genus in CARABIDAE: HARPALINAE: PTEROSTICHINI: PTEROSTICHINA. Note: Marcilhac used two different spellings for this taxon, *Aristochroodes* (p. 271, 273, 274) and *Aritochroodes* (p. 274); we are not aware of anyone who acted as First Reviser (ICZN 1999a, Article 24.2.3) and we **here select***Aristochroodes* as the correct original spelling.

Type species: *Pterostichusreginae* Marcilhac, 1993, by original designation.

Family-group name: ARISTOCHROODINI Sciaky, 1996. Stem: *Aristochrood*-.

***Arneus* Gistel, 1848b**: xi, xiv. Current status: junior synonym of *Sericus* Eschscholtz, 1829 in ELATERIDAE: ELATERINAE: ELATERINI. Note: unnecessary new replacement name for *Sericosomus* Dejean, 1833.

Type species: *Elaterbrunneus* Linnaeus, 1758, by subsequent designation (Westwood 1838c: 25) (type fixation for *Sericosomus* Dejean).

Family-group name: ARNEIDAE Gistel, 1848. Stem: *Arne*-.

***Arnyllium* Reitter, 1884a**: 391. Current status: valid genus in STAPHYLINIDAE: PSELAPHINAE: GONIACERITAE: ARNYLLIINI.

Type species: *Arnylliumpectinatum* Reitter, 1884, by subsequent designation (Jeannel 1952: 101).

Family-group name: ARNYLLIINI Jeannel, 1952. Stem: *Arnylli*-.

***Arpediomimus* Cameron, 1917b**: 277. Current status: junior synonym of *Crymus* Fauvel, 1904 in STAPHYLINIDAE: OMALIINAE: OMALIINI. Note: new replacement name for *Arpediopsis* Cameron, 1917.

Type species: *Arpediopsisfalklandica* Cameron, 1917 (= *Crymusantarcticus* Fauvel, 1904), by monotypy (type fixation for *Arpediopsis* Cameron).

Family-group name: ARPEDIOMIMI Cameron, 1917. Stem: *Arpediomim*-.

***Arpediopsis* Cameron, 1917a**: 124. Current status: junior synonym of *Crymus* Fauvel, 1904 in STAPHYLINIDAE: OMALIINAE: OMALIINI. Note: junior homonym of *Arpediopsis* Ganglbauer, 1895 [Coleoptera: STAPHYLINIDAE].

Type species: *Arpediopsisfalklandica* Cameron, 1917 (= *Crymusantarticus* Fauvel, 1904), by monotypy.

Family-group name: ARPEDIOPSINI Cameron, 1917. Stem: *Arpediopse*-. Note: family-group name permanently invalid (ICZN 1999a, Article 39).

***Arrenodes*Schönherr 1823**: 1137. Current status: valid genus in BRENTIDAE: BRENTINAE: BRENTINI: ARRENODINA.

Type species: *Brentusmaxillosus* G.-A. Olivier, 1807 (= *Curculiominutus* Drury, 1773), by original designation.

Family-group name: ARRHÉNODIDES Lacordaire, 1865. Stem: *Arrenod*-. Note: this family-group name is based on a misidentification of *Arrenodes* Schönherr, 1823 by Lacordaire (1865: 429), which corresponds to the genus *Estenorhinus* Lacordaire, 1865; Alonso- Zarazaga and Lyal (1999: 78) mentioned that they would apply to the Commission for the conservation of this tribe by designating *Arrenodes* Schönherr, 1823 as its type genus; meanwhile current usage is maintained.

***Arrhipis* Gemminger in Gemminger and Harold, 1869b**: 1477. Current status: invalid senior synonym of *Nematodinus* Lea, 1919 in EUCNEMIDAE: MELASINAE: DIRHAGINI. Note: junior homonym of *Arrhipis* Agassiz, 1846 [Pisces]; this genus-group name is usually credited to Bonvouloir, 1871 but it was first made available by Gemminger in 1869 when he associated two available species with the name; *Arrhipis* Gemminger is currently recognized as a valid genus but since it is a junior homonym, *Nematodinus* Lea, 1919, **stat. nov.** is used as valid instead.

Type species: *Hylocharessubacutus* Guérin-Méneville, 1843, by subsequent designation (Fleutiaux 1902: 659).

Family-group name: ARHIPINI Cobos, 1965. Stem: *Arrhipid*-. Note: family-group name permanently invalid (ICZN 1999a, Article 39).

***Arrowinus* Bernhauer, 1935**: 215. Current status: valid genus in STAPHYLINIDAE: STAPHYLININAE: ARROWININI.

Type species: *Arrowinusphaenomenalis* Bernhauer, 1935, by original designation.

Family-group name: ARROWININI Solodovnikov & Newton, 2005. Stem: *Arrowin*-.

***Arsipoda* Erichson, 1842**: 235. Current status: valid genus in CHRYSOMELIDAE: GALERUCINAE: ALTICINI.

Type species: *Arsipodabifrons* Erichson, 1842, by monotypy.

Family-group name: ARSIPODITES Chapuis, 1875. Stem: *Arsipod*-.

***Artaxus* Gistel, 1848b**: 216. Current status: junior synonym of *Mycterus* Clairville, 1798 in MYCTERIDAE: MYCTERINAE. Note: unnecessary new replacement name for *Mycterus* Clairville, 1798.

Type species: *Mycterusgriseus* Clairville, 1798 (= *Rhinomacercurculioides* Fabricius, 1781), by monotypy (type fixation for *Mycterus* Clairville).

Family-group name: ARTAXIDAE Gistel, 1848. Stem: *Artax*-.

***Artematopus* Perty, 1832**: 115. Current status: valid genus in ARTEMATOPODIDAE: ARTEMATOPODINAE: ARTEMATOPODINI.

Type species: *Artematopuslongicornis* Perty, 1832, by monotypy.

Family-group name: ARTÉMATOPIDES Lacordaire, 1857. Stem: *Artematopod*-.

***Arthrobrachus* Solier, 1849**: 414. Current status: valid genus in MELYRIDAE: MELYRINAE: ARTHROBRACHINI.

Type species: *Arthrobrachusvarians* Solier, 1849 (= *Zygiaflavipennis* Laporte, 1838), by subsequent designation (Majer 1987: 794).

Family-group name: ARTHROBRACHINI Majer, 1987. Stem: *Arthrobrach*-.

***Arthrolips* Wollaston, 1854**: 475. Current status: valid genus in CORYLOPHIDAE: CORYLOPHINAE: PARMULINI.

Type species: *Clypeasterpiceus* Comolli, 1837, by monotypy.

Family-group name: ARTHROLIPINAE Böving & Craighead, 1931. Stem: *Arthrolip*-.

†***Arthropterites* Wasmann, 1926**: 28. Current status: valid genus in CARABIDAE: PAUSSINAE: PAUSSINI: †ARTHROPTERITINA. Note: Eocene (Baltic amber).

Type species: †*Arthropteritesklebsi* Wasmann, 1926, by monotypy.

Family-group name: †ARTHROPTERITINA Luna de Carvalho, 1961. Stem: *Arthropterit*-.

***Arthropterus* W.S. MacLeay, 1838**: 75. Current status: valid genus in CARABIDAE: PAUSSINAE: PAUSSINI: CERAPTERINA.

Type species: *Cerapterusmacleaii* Donovan, 1805, by monotypy.

Family-group name: ARTHROPTERINI Wasmann, 1928. Stem: *Arthropter*-. Note: junior homonym of ARTHROPTERIDAE Jordan, 1923 (type genus *Arthropterus* Agassiz, 1843) in Pisces; Jordan’s name is permanently invalid (based on a preoccupied type genus).

***Arthrostenus* Schönherr, 1826**: 287. Current status: valid genus in CURCULIONIDAE: BRACHYCERINAE: ERIRHININI.

Type species: *Arthrostenusfullo* Boheman, 1836, by subsequent designation (E. Desmarest 1860: 242). Note: no available species was originally included for this genus in Schönherr (1826); the first ones are the three species listed in Schönherr (1836: 534, 535).

Family-group name: ARTHROSTENINI Reitter, 1913. Stem: *Arthrosten*-.

***Articerus* Dalman, 1826**: 394. Current status: valid genus in STAPHYLINIDAE: PSELAPHINAE: CLAVIGERITAE: CLAVIGERINI.

Type species: *Articerusarmatus* Dalman, 1826, by monotypy.

Family-group name: ARTICERIDES E. Desmarest, 1852. Stem: *Articer*-. Note: this family-group name has been credited to L.W. Schaufuss (1872: 245) by most authors (e.g., Newton and Chandler 1989: 5) and its type genus is considered to be misidentified in the sense of Hope (1845a: 106); however, the family-group name was first made available by E. Desmarest (1852: 145) in the proper sense since E. Desmarest referred to the insect described by Dalman in copal.

***Artipus* C.R. Sahlberg, 1823a**: 22. Current status: valid genus in CURCULIONIDAE: ENTIMINAE: POLYDRUSITAE: NAUPACTINI: NAUPACTINA. Note: we consider *Artipus* used by Schönherr (1823: 1141) a subsequent usage of *Artipus* C.R. Sahlberg, 1823.

Type species: *Artipuscorycaeus* C.R. Sahlberg, 1823, by monotypy. Note: combined description of a new nominal genus and a single new nominal species (ICZN 1999a, Article 12.2.6).

Family-group name: ARTIPI G.H. Horn, 1876. Stem: *Artipod*-.

***Asaphes* W. Kirby, 1837**: 146. Current status: invalid senior synonym of *Hemicrepidius* Germar, 1839 in ELATERIDAE: DENDROMETRINAE: DENDROMETRINI: HEMICREPIDIINA. Note: junior homonym of *Asaphes* Walker, 1834 [Hymenoptera].

Type species: *Pedetesruficornis* W. Kirby, 1837, by monotypy.

Family-group name: ASAPHITES Candèze, 1863. Stem: *Asaph*-. Note: family-group name permanently invalid (ICZN 1999a, Article 39).

***Asclera* Dejean, 1834**: 228. Current status: junior synonym of *Ischnomera* Stephens, 1832 in OEDEMERIDAE: OEDEMERINAE: ASCLERINI.

Type species: *Necydalissanguinicollis* Fabricius, 1787, by subsequent designation (Arnett 1950: 219).

Family-group name: ASCLERAEIDAE Gistel, 1848. Stem: *Ascler*-.

***Ascydmus* Casey, 1897**: 505. Current status: junior synonym of *Euthiconus* Reitter, 1882 in STAPHYLINIDAE: SCYDMAENINAE: CEPHENNIITAE: EUTHEIINI.

Type species: *Ascydmustener* Casey, 1897, by monotypy.

Family-group name: ASCYDMINI Casey, 1897. Stem: *Ascydm*-.

***Asemum* Eschscholtz, 1830a**: 66. Current status: valid genus in CERAMBYCIDAE: SPONDYLIDINAE: ASEMINI.

Type species: *Cerambyxstriatus* Linnaeus, 1758, by subsequent designation (Westwood 1838c: 40). Note: name placed on the Official List of Specific Names in Zoology (ICZN 2017a).

Family-group name: ASEMITAE J. Thomson, 1861. Stem: *Asem*-.

***Asida* Latreille, 1802b**: 167. Current status: valid genus in TENEBRIONIDAE: PIMELIINAE: ASIDINI.

Type species: *Opatrumgriseum* Fabricius, 1781, by monotypy.

Family-group name: ASIDADAE Fleming, 1821. Stem: *Asid*-.

†***Asiocoleus* Rohdendorf, 1961**: 396. Current status: valid genus in †ASIOCOLEIDAE. Note: lower Permian (Russia).

Type species: †*Asiocoleusnovojilovi* Rohdendorf, 1961, by original designation.

Family-group name: †ASIOCOLEIDAE Rohdendorf, 1961. Stem: *Asiocole*-.

***Asphalus* Pascoe, 1868**: xii. Current status: valid genus in TENEBRIONIDAE: TENEBRIONINAE: HELEINI: ASPHALINA.

Type species: *Asphalusebeninus* Pascoe, 1868, by monotypy.

Family-group name: ASPHALINA E.G. Matthews & Lawrence, 2005. Stem: *Asphal*-.

***Aspicela* Dejean, 1836a**: 387. Current status: valid genus in CHRYSOMELIDAE: GALERUCINAE: ALTICINI.

Type species: *Alticaunipunctata* Latreille, 1813, by subsequent designation (Monrós and Bechyné 1956: 1134).

Family-group name: ASPICÉLITES Chapuis, 1875. Stem: *Aspicel*-.

***Aspidapion* Schilsky, 1901**: 38G. Current status: valid genus in BRENTIDAE: APIONINAE: ASPIDAPIITAE: ASPIDAPIINI.

Type species: *Apionvalidum* Germar, 1817, by original designation.

Family-group name: ASPIDAPIINI Alonso-Zarazaga, 1990. Stem: *Aspidapi*-.

***Aspidimerus* Mulsant, 1850**: 944. Current status: valid genus in COCCINELLIDAE: COCCINELLINAE: ASPIDIMERINI.

Type species: *Aspidimerusspencii* Mulsant, 1850, by monotypy.

Family-group name: ASPIDIMÉRAIRES Mulsant, 1850. Stem: *Aspidimer*-.

***Aspidimorpha* Hope, 1841a**: 158, 159. Current status: valid genus in CHRYSOMELIDAE: CASSIDINAE: ASPIDIMORPHINI.

Type species: *Cassidamiliaris*Fabricius 1775, by original designation.

Family-group name: ASPIDIMORPHITES Chapuis, 1875. Stem: *Aspidimorph*-.

***Aspidiphorus* Dejean, 1821**: 47. Current status: valid genus in SPHINDIDAE: SPHINDINAE. Note: this genus was originally spelled *Arpidiphorus* but corrected to *Aspidiphorus* by the Commission and the name was placed (as *Aspidiphorus* Ziegler in Dejean, 1821) on the Official List of Generic Names in Zoology (ICZN 1997).

Type species: *Nitidulaorbiculata* Gyllenhal, 1808, by monotypy (see ICZN 1997). Note: name placed on the Official List of Specific Names in Zoology (ICZN 1997).

Family-group name: ASPIDIPHORIDAE Kiesenwetter, 1877. Stem: *Aspidiphor*-.

***Aspidytes* Ribera, Beutel, Balke & Vogler, 2002**: 2352. Current status: valid genus in ASPIDYTIDAE.

Type species: *Aspidytesniobe* Ribera, Beutel, Balke & Vogler, 2002, by original designation.

Family-group name: ASPIDYTIDAE Ribera, Beutel, Balke & Vogler, 2002. Stem: *Aspidyt*-.

***Aspilus* Schaum, 1848**: 61. Current status: valid genus in SCARABAEIDAE: CETONIINAE: CREMASTOCHEILINI: ASPILINA.

Type species: *Ptychophorusgambiensis* Burmeister, 1842, by monotypy.

Family-group name: ASPILINA Krikken, 1984. Stem: *Aspil*-.

***Astaena* Erichson, 1847c**: 101. Current status: valid genus in SCARABAEIDAE: SERICINAE: SERICINI: ASTAENINA.

Type species: *Astaenatridentata* Erichson, 1847, by monotypy.

Family-group name: ASTAENIDAE Burmeister, 1855. Stem: *Astaen*-.

***Astathes* Newman, 1842a**: 299. Current status: junior synonym of *Tetraophthalmus* Dejean, 1835 in CERAMBYCIDAE: LAMIINAE: ASTATHINI.

Type species: *Astathesperplexa* Newman, 1842, by subsequent designation (J. Thomson 1864: 118).

Family-group name: ASTATHITAE J. Thomson, 1864. Stem: *Astath*-.

***Astenus* Dejean, 1833a**: 65. Current status: valid genus in STAPHYLINIDAE: PAEDERINAE: LATHROBIINI: ASTENINA.

Type species: *Staphylinusangustatus* Paykull, 1789 (= *Staphylinusgracilis* Paykull, 1789), by subsequent designation (Westwood 1838c: 17).

Family-group name: ASTENINA Hatch, 1957. Stem: *Asten*-.

***Asteriza* Chevrolat in Dejean, 1836a**: 372. Current status: valid genus in CHRYSOMELIDAE: CASSIDINAE: ISCHYROSONYCHINI.

Type species: *Cassidaflavicornis* G.-A. Olivier, 1790, by monotypy.

Family-group name: ASTERIZINI Hincks, 1952. Stem: *Asteriz*-.

***Astraeus* Laporte & Gory, 1838**: 1. Current status: valid genus in BUPRESTIDAE: POLYCESTINAE: ASTRAEINI. Note: the original spelling *Asthraeus* was emended to *Astraeus* by the Commission and *Astraeus* Laporte & Gory, 1838 placed on the Official List of Generic Names in Zoology (ICZN 1966b, as *Astraeus* Laporte & Gory, 1837).

Type species: *Astraeusflavopictus* Laporte & Gory, 1838, by monotypy (see ICZN 1966b, as *Asthraeusflavopictus* Laporte & Gory, 1837).

Family-group name: ASTRAEUSINI Cobos, 1980. Stem: *Astrae*-.

***Astrotus* J.L. LeConte, 1858b**: 19. Current status: valid genus in TENEBRIONIDAE: PIMELIINAE: ASIDINI.

Type species: *Microschatiacontorta* J.L. LeConte, 1853, by original designation.

Family-group name: ASTROTI G.H. Horn, 1870. Stem: *Astrot*-.

***Astylus* Laporte, 1838**: 32. Current status: valid genus in MELYRIDAE: MELYRINAE: ASTYLINI.

Type species: *Anobiumlineatum* Fabricius, 1775, by subsequent designation (Constantin 2011: 39).

Family-group name: ASTYLINI Pic, 1929. Stem: *Astyl*-.

***Astynomus* Dejean, 1835**: 337. Current status: junior synonym of *Aedilis* Audinet-Serville, 1835 in CERAMBYCIDAE: LAMIINAE: ACANTHOCININI. Note: unnecessary new replacement name for *Aedilis* Audinet-Serville, 1835.

Type species: *Aedilismontana* Audinet-Serville, 1835 (= *Cerambyxaedilis* Linnaeus, 1758), by subsequent designation (Westwood 1838c: 40) (type fixation for *Aedilis* Audinet- Serville).

Family-group name: ASTYNOMINI Portevin, 1927. Stem: *Astynom*-.

***Ataenius* Harold, 1867a**: 82. Current status: valid genus in SCARABAEIDAE: APHODIINAE: EUPARIINI. Note: this genus was described as new, with a single other new species, by von Harold (1867b: 100); name given precedence over *Aphodinus* Motschulsky, 1862 whenever the two are considered to be synonyms and placed on the Official List of Generic Names in Zoology (ICZN 2010a).

Type species: *Ataeniusscutellaris* Harold, 1867, by subsequent designation (Chapin 1940: 12; see ICZN 2010a). Note: name placed on the Official List of Specific Names in Zoology (ICZN 2010a).

Family-group name: ATAENIDAE Harold, 1867. Stem: *Ataeni*-. Note: nomen oblitum (see A.B.T. Smith 2006: 158).

***Atanygnathus* Jakobson, 1909**: 521. Current status: valid genus in STAPHYLINIDAE: STAPHYLININAE: STAPHYLININI: TANYGNATHININA. Note: new replacement name for *Tanygnathus* Erichson, 1839.

Type species: *Tanygnathusterminalis* Erichson, 1839, by monotypy (type fixation for *Tanygnathus* Erichson).

Family-group name: ATANYGNATHINI Lohse, 1964. Stem: *Atanygnath*-.

***Ataxia* Haldeman, 1847**: 56. Current status: valid genus in CERAMBYCIDAE: LAMIINAE: PTEROPLIINI.

Type species: *Ataxiasordida* Haldeman, 1847 (= *Lamiacrypta* Say, 1832), by monotypy.

Family-group name: ATAXIIDES Lacordaire, 1872. Stem: *Ataxi*-.

***Ateledera* Lacordaire, 1845**: 607. Current status: valid genus in MEGALOPODIDAE: ATELEDERINAE.

Type species: *Atelederacygnoides* Lacordaire, 1845, by monotypy.

Family-group name: ATELEDERINAE Rodríguez-Mirón in Rodríguez-Mirón et al., 2021: 694. Stem: *Ateleder*-.

***Atelius* C.O. Waterhouse, 1878**: 104. Current status: valid genus in LYCIDAE: ATELIINAE: ATELIINI.

Type species: *Lycusexpansicornis* Walker, 1858, by original designation.

Family-group name: ATELINAE Kleine, 1929. Stem: *Ateli*-.

***Aterpus* Schönherr, 1826**: 156. Current status: junior synonym of *Aades* Schönherr, 1823 in CURCULIONIDAE: CYCLOMINAE: ATERPINI. Note: unnecessary new replacement name for *Aades* Schönherr, 1823.

Type species: *Curculiocultratus* Fabricius, 1775, by subsequent designation (Schönherr 1833: 13).

Family-group name: ATERPIDES Lacordaire, 1863. Stem: *Aterp*-. Note: nomen protectum (see Bouchard et al. 2011: 601).

***Ateuchus* Weber, 1801**: 10. Current status: valid genus in SCARABAEIDAE: SCARABAEINAE: ATEUCHINI: ATEUCHINA. Note: this genus was described twice in the same year by Weber (1801: 10) and Fabricius (1801a: 54); bibliographic research suggests that Weber’s book (issued by March 1801, see Evenhuis 1997b: 809) was published before the first volume of Fabricius’ ‘Systema Eleutheratorum’ (issued by 26 April 1801, see Bousquet 2017a: 108; see also Chapin 1946: 79).

Type species: *Ateuchushisteroides* Weber, 1801, by monotypy.

Family-group name: ATEUCHIDAE Perty, 1830. Stem: *Ateuch*-. Note: Perty (1830: 38) did not provide the author to the genus *Ateuchus*; it is assumed here that he referred to *Ateuchus* Weber, not *Ateuchus* Fabricius.

***Athertonium* Crowson, 1990**: 91, 92. Current status: valid genus in BOGANIIDAE: PARACUCUJINAE.

Type species: *Athertoniumparvum* Crowson, 1990, by original designation.

Family-group name: ATHERTONIINI Crowson, 1990. Stem: *Athertoni*-.

***Atheta* C.G. Thomson, 1858**: 36. Current status: valid genus in STAPHYLINIDAE: ALEOCHARINAE: ATHETINI: ATHETINA. Note: name placed on the Official List of Generic Names in Zoology (ICZN 1961b).

Type species: *Aleocharagraminicola* Gravenhorst, 1806, by subsequent monotypy (see ICZN 1961b). Note: although the Commission (ICZN 1961b: 241) mentioned that the type species was designated by monotypy, this is incorrect since there is no species associated with the name *Atheta* in C.G. Thomson (1858: 36); *Aleocharagraminicola* Gravenhorst was fixed as the type species by subsequent monotypy in C.G. Thomson (1859: 39); the Official List of Generic Names in Zoology needs to be updated to reflect the changes for the fixation of the type species mentioned above.

Family-group name: ATHETAE Casey, 1910. Stem: *Athet*-.

***Athexenia* Pace, 2000b**: 336. Current status: valid genus in STAPHYLINIDAE: ALEOCHARINAE: LOMECHUSINI: MYRMEDONIINA.

Type species: *Athexenianilgiriensis* Pace, 2000, by original designation.

Family-group name: ATHEXENINA Pace, 2000. Stem: *Athexeni*-.

***Athlia* Erichson, 1835**: 266. Current status: valid genus in SCARABAEIDAE: MELOLONTHINAE: ATHLIINI.

Type species: *Athliarustica* Erichson, 1835, by monotypy.

Family-group name: ATHLIINI A.B.T. Smith & Evans, 2018: 280. Stem: *Athli*-

***Athoomorphus* O. Schwarz, 1898**: 156. Current status: valid genus in ELATERIDAE incertae sedis.

Type species: *Athoomorphuscylindricus* O. Schwarz, 1898, by monotypy.

Family-group name: ATHOOMORPHINAE Laurent, 1966. Stem: *Athoomorph*-.

***Athous* Eschscholtz, 1829a**: 33. Current status: valid genus in ELATERIDAE: DENDROMETRINAE: DENDROMETRINI: DENDROMETRINA.

Type species: *Elatervittatus* Fabricius, 1792, by subsequent designation (Westwood 1838c: 26).

Family-group name: ATHOITES Candèze, 1859. Stem: *Atho*-.

***Athyreus* W.S. MacLeay, 1819**: 123. Current status: valid genus in BOLBOCERATIDAE: ATHYREINAE. Note: name placed on the Official List of Generic Names in Zoology (ICZN 1985a).

Type species: *Athyreusbifurcatus* W.S. MacLeay, 1819, by subsequent designation (Howden and Martínez 1963: 350; see ICZN 1985a). Note: name placed on the Official List of Specific Names in Zoology (ICZN 1985a).

Family-group name: ATHYREITAE Lynch Arribálzaga, 1878. Stem: *Athyre*-.

***Atimia* Haldeman, 1847**: 56. Current status: valid genus in CERAMBYCIDAE: SPONDYLIDINAE: ATIMIINI.

Type species: *Atimiatristis* Haldeman, 1847 (= *Clytusconfusus* Say, 1826), by monotypy.

Family-group name: ATIMIINI J.L. LeConte, 1873. Stem: *Atimi*-.

***Atomaria* Stephens, 1829b**: 8. Current status: valid genus in CRYPTOPHAGIDAE: ATOMARIINAE: ATOMARIINI.

Type species: *Silphaevanescens* Marsham, 1802 (= *Dermestespusillus* Paykull, 1798), by subsequent designation (Partington 1834: 244). Note: the earliest type species designation previously known for this genus was *Dermestesnigripennis* Paykull, 1798 (= *Latridiusnigripennis* Kugelann, 1794), as designated subsequently by Westwood (1838c: 14) (e.g., C. Johnson 2007a: 65, Otero and Pereira Martínez 2022: 3), but Partington’s fixation is older; *Silphaevanescens* Marsham, 1802, which was later nominally placed in the genus *Ptenidium* Erichson, 1845 in the family PTILIIDAE (e.g., Matthews 1872: 80) is actually a junior synonym of *Atomariapusilla* (Paykull, 1798) (see Gemminger and von Harold 1868c: 890; Ganglbauer 1899: 303); the synonymy of the type species has been confirmed by examination of type photographs (M.L. Gimmel, pers. comm. to P.B., 2023); this change of type species will result in the subgenus previously known under the name *Anchicera* C.G. Thomson, 1863 changing to Atomaria Stephens, 1829 and the subgenus previously known under the name *Atomaria* Stephens, 1829 changing to *Agathengis* Gozis, 1886 (M.L. Gimmel, pers. comm. to P.B., 2023).

Family-group name: ATOMARIINI J.L. LeConte, 1861. Stem: *Atomari*-.

***Atopa* Paykull, 1799**: 116. Current status: junior synonym of *Dascillus* Latreille, 1797 in DASCILLIDAE: DASCILLINAE: DASCILLINI.

Type species: *Chrysomelacervina* Linnaeus, 1758, by monotypy.

Family-group name: ATOPITES Laporte, 1834. Stem: *Atop*-.

***Atopida* A. White, 1846**: 8. Current status: valid genus in SCIRTIDAE: SCIRTINAE.

Type species: *Atopidacastanea* A. White, 1846, by monotypy.

Family-group name: ATOPIDINI Pic, 1914. Stem: *Atopid*-.

***Atopobrentus* Damoiseau, 1965**: 3. Current status: valid genus in BRENTIDAE: BRENTINAE: TRACHELIZINI: ATOPOBRENTINA.

Type species: *Atopobrentusallardi* Damoiseau, 1965, by original designation.

Family-group name: ATOPOBRENTINI Damoiseau, 1965. Stem: *Atopobrent*-.

***Atossa* J. Thomson, 1864**: 100. Current status: synonym of *Grammoechus* J. Thomson, 1864 in CERAMBYCIDAE: LAMIINAE: PTEROPLIINI. Note: the First Reviser (ICZN 1999a, Article 24.2.2) for *Grammoechus* J. Thomson, 1864 versus *Atossa* J. Thomson, 1864 is Breuning (1961: 232).

Type species: *Atossastrenua* J. Thomson, 1864, by original designation.

Family-group name: ATOSSIDES Lacordaire, 1872. Stem: *Atoss*-.

***Atractocerus* Palisot de Beauvois, 1801**: 516. Current status: valid genus in LYMEXYLIDAE: ATRACTOCERINAE. Note: this genus-group name was spelled *Atractourus* in the original article but corrected to *Atractocerus* on page 576 of the same publication.

Type species: *Atractocerusnecydaloides* Palisot de Beauvois, 1801 (= *Necydalisbrevicornis* Linnaeus, 1767), by monotypy.

Family-group name: ATRACTOCÉRITES Laporte, 1840. Stem: *Atractocer*-.

***Atranopsis* Baehr, 1982**: 261. Current status: valid genus in CARABIDAE: HARPALINAE: SPHODRINI: ATRANOPSINA.

Type species: *Atranopsisscheuerni* Baehr, 1982, by original designation.

Family-group name: ATRANOPSINA Baehr, 1982. Stem: *Atranops*-.

***Atranus* J.L. LeConte, 1847**: 438. Current status: valid genus in CARABIDAE: HARPALINAE: ATRANINI.

Type species: *Anchomenuspubescens* Dejean, 1828, by monotypy.

Family-group name: ATRANI G.H. Horn, 1881. Stem: *Atran*-.

***Atrecus* Jacquelin du Val, 1857b**: 31. Current status: valid genus in STAPHYLINIDAE: STAPHYLININAE: OTHIINI.

Type species: *Staphylinuspilicornis* Paykull, 1790, by original designation.

Family-group name: ATRECINI Hatch, 1957. Stem: *Atrec*-.

***Attagenus* Latreille, 1802b**: 121. Current status: valid genus in DERMESTIDAE: ATTAGENINAE: ATTAGENINI.

Type species: *Dermestestrifasciatus* Fabricius, 1787, by subsequent designation (des Gozis 1886: 10). Note: *Dermestespellio* Linnaeus, 1758 is usually listed as type species of the genus (e.g., Háva 2007: 307) but it is not an originally included species.

Family-group name: ATTAGÉNITES Laporte, 1840. Stem: *Attagen*-.

***Attalomimus* Wittmer, 1976a**: 288. Current status: valid genus in MELYRIDAE: MALACHIINAE: LEMPHINI.

Type species: *Attalomimuscephalotes* Wittmer, 1976, by original designation.

Family-group name: ATTALOMIMIDAE Majer, 1995. Stem: *Attalomim*-.

***Attalus* Erichson, 1840a**: 89. Current status: valid genus in MELYRIDAE: MALACHIINAE: MALACHIINI.

Type species: *Attaluslusitanicus* Erichson, 1840, by subsequent designation (Champion 1914a: 42).

Family-group name: ATTALAIRES Abeille de Perrin, 1890. Stem: *Attal*-.

***Attapsenius*Bruch, 1933a**: 27. Current status: valid genus in STAPHYLINIDAE: PSELAPHINAE: PSELAPHITAE: ATTAPSENIINI.

Type species: *Attapseniuschernosvitovi*Bruch, 1933, by original designation.

Family-group name: ATTAPSENIINIBruch, 1933. Stem: *Attapseni*-.

***Attavicinus* Philips & K.L. Bell, 2008**: 68. Current status: valid genus in SCARABAEIDAE: SCARABAEINAE: ONITICELLINI: ATTAVICINA.

Type species: *Oniticellusmonstrosus* H.W. Bates, 1887, by original designation.

Family-group name: ATTAVICINA Philips, 2016: 41. Stem: *Attavicin*-.

***Attelabus* Linnaeus, 1758**: 342, 387. Current status: valid genus in ATTELABIDAE: ATTELABINAE: ATTELABINI: ATTELABINA. Note: name placed on the Official List of Generic Names in Zoology (ICZN 1983a).

Type species: *Attelabuscurculionoides* Linnaeus, 1767 (= *Curculionitens* Scopoli, 1763), by plenary power (see ICZN 1983a). Note: *Curculionitens* Scopoli, 1763 was placed on the Official List of Specific Names in Zoology (ICZN 1983a).

Family-group name: ATTELABIDES Billberg, 1820. Stem: *Attelab*-.

***Atysa* Baly, 1864a**: 238. Current status: valid genus in CHRYSOMELIDAE: GALERUCINAE: GALERUCINI.

Type species: *Atysaterminalis* Baly, 1864, by original designation.

Family-group name: ATYSITES Chapuis, 1875. Stem: *Atys*-.

***Aubehydrus* Guignot, 1942**: 11. Current status: junior synonym of *Notaticus* A. Zimmermann, 1928 in DYTISCIDAE: DYTISCINAE: AUBEHYDRINI.

Type species: *Aubehydrusspeciosissimus* Guignot, 1942 (= *Notaticusfasciatus* A. Zimmermann, 1928), by original designation.

Family-group name: AUBEHYDRINAE Guignot, 1942. Stem: *Aubehydr*-.

***Auchmeresthes* Kraatz, 1862a**: 119. Current status: valid genus in CURCULIONIDAE: ENTIMINAE: CYPHICERITAE: METACINOPINI.

Type species: *Auchmerestheskiesenwetteri* Kraatz, 1862, by monotypy.

Family-group name: AUCHMERESTHINAE Reitter, 1913. Stem: *Auchmeresthet*-.

***Auchmobius* J.L. LeConte, 1851a**: 139. Current status: valid genus in TENEBRIONIDAE: PIMELIINAE: EDROTINI.

Type species: *Auchmobiussublaevis* J.L. LeConte, 1851, by monotypy.

Family-group name: AUCHMOBII J.L. LeConte & G.H. Horn, 1883. Stem: *Auchmobi*-.

***Augyles* Schiødte, 1866**: 159, 166. Current status: valid genus in HETEROCERIDAE: HETEROCERINAE: AUGYLINI.

Type species: *Heterocerushispidulus* Kiesenwetter, 1843, by monotypy.

Family-group name: AUGYLIINI M.F. Pacheco, 1964. Stem: *Augyl*-.

***Aulacocerus* A. White, 1853**: 13. Current status: junior synonym of *Braderochus* Buquet, 1852 in CERAMBYCIDAE: PRIONINAE: PRIONINI.

Type species: *Aulacocerusmundus* A. White, 1853, by monotypy.

Family-group name: AULACOCERITAE J. Thomson, 1864. Stem: *Aulacocer*-.

***Aulacocyclus* Kaup, 1868a**: 4. Current status: valid genus in PASSALIDAE: AULACOCYCLINAE: AULACOCYCLINI.

Type species: *Passalusedentulus* W.S. MacLeay, 1826, by subsequent designation (Gravely 1918: 17).

Family-group name: AULACOCYCLINAE Kaup, 1868. Stem: *Aulacocycl*-.

***Aulacophora* Chevrolat in Dejean, 1836a**: 378. Current status: valid genus in CHRYSOMELIDAE: GALERUCINAE: LUPERINI.

Type species: *Gallerucaquadraria* G.-A. Olivier, 1808, by subsequent designation (Duponchel and Chevrolat 1842a: 337).

Family-group name: AULACOPHORITES Chapuis, 1875. Stem: *Aulacophor*-.

***Aulacopus* Audinet-Serville, 1832**: 144. Current status: valid genus in CERAMBYCIDAE: PRIONINAE: MACROTOMINI: MACROTOMINA.

Type species: *Aulacopusreticulatus* Audinet-Serville, 1832, by monotypy.

Family-group name: AULACOPINAE Kolbe, 1897. Stem: *Aulacopod*-.

***Aulacoscelis* Duponchel & Chevrolat, 1842a**: 342. Current status: valid genus in ORSODACNIDAE: AULACOSCELIDINAE.

Type species: *Aulacoscelismelanocera* Duponchel & Chevrolat, 1842, by monotypy.

Family-group name: AULACOSCÉLITES Chapuis, 1874. Stem: *Aulacoscelid*-.

***Aulacotrachelus* Benick, 1921**: 1. Current status: junior synonym of *Megalopinus* Eichelbaum, 1915 in STAPHYLINIDAE: MEGALOPSIDIINAE. Note: new replacement name for *Megalops* Erichson, 1839.

Type species: *Oxyporuscaelatus* Gravenhorst, 1802, by subsequent designation (E. Desmarest 1852: 85) (type fixation for *Megalops* Erichson).

Family-group name: AULACOTRACHELINAE L. Benick, 1921. Stem: *Aulacotrachel*-.

***Auletanus* Voss, 1922a**: 17, 20. Current status: valid genus in ATTELABIDAE: RHYNCHITINAE: RHYNCHITITAE: AULETINI: AULETOBIINA.

Type species: *Auletobiusascendens* Heller, 1915, by subsequent designation (Voss 1932: 101).

Family-group name: AULETANINI Legalov, 2003. Stem: *Auletan*-.

***Auletes* Schönherr, 1826**: 46. Current status: valid genus in ATTELABIDAE: RHYNCHITINAE: RHYNCHITITAE: AULETINI: AULETINA.

Type species: *Auletestubicen* Schönherr, 1826, by original designation. Note: combined description of a new nominal genus and a single new nominal species (ICZN 1999a, Article 12.2.6).

Family-group name: AULETINIDAE Desbrochers des Loges, 1908. Stem: *Aulet*-.

***Auletobius* Desbrochers des Loges, 1869**: 396. Current status: valid genus in ATTELABIDAE: RHYNCHITINAE: RHYNCHITITAE: AULETINI: AULETOBIINA.

Type species: *Auletesbasilaris* Gyllenhal, 1839 (= *Involvulussanguisorbae* Schrank, 1798), by subsequent designation (Brauer 1870: 133).

Family-group name: AULETOBINI Voss, 1930. Stem: *Auletobi*-.

***Auletorhinus* Voss, 1935**: 509. Current status: valid genus in ATTELABIDAE: RHYNCHITINAE: RHYNCHITITAE: AULETINI: AULETOBIINA.

Type species: *Auletorhinushirtellus* Voss, 1935, by original designation.

Family-group name: AULETORHININI Voss, 1935. Stem: *Auletorhin*-.

***Aulonocnemis* Klug, 1837**: 53. Current status: valid genus in SCARABAEIDAE: AULONOCNEMINAE.

Type species: *Aulonocnemisopatrina* Klug, 1837, by subsequent designation (R. Lucas 1920: 129).

Family-group name: AULONOCNEMINAE A. Janssens, 1946. Stem: *Aulonocnem*-.

***Aulonogyrus* Motschulsky, 1853c**: 9. Current status: valid genus in GYRINIDAE: GYRININAE: DINEUTINI: ENHYDRUSINA.

Type species: *Gyrinusstrigipennis* Suffrian, 1842 (= *Gyrinusconcinnus* Klug, 1834), by subsequent designation (Ochs 1930: 3).

Family-group name: AULONOGYRINI Ochs, 1953. Stem: *Aulonogyr*-.

***Aurelius* Kuwert, 1891**: 168. Current status: valid genus in PASSALIDAE: PASSALINAE: MACROLININI.

Type species: *Aureliusdohrnii* Kuwert, 1891, by monotypy.

Family-group name: AURELIINAE Kuwert, 1896. Stem: *Aureli*-.

***Australestes* Pace, 2015**: 66. Current status: valid genus in STAPHYLINIDAE: ALEOCHARINAE: AUSTRALESTESINI.

Type species: *Australestescrassum* Pace, 2015, by original designation.

Family-group name: AUSTRALESTESINI Pace, 2015: 65. Stem: *Australestes*- (ICZN 1999a, Article 29.4).

***Australiotyphlus* Pace, 2013a**: 99. Current status: valid genus in STAPHYLINIDAE: LEPTOTYPHLINAE: AUSTRALIOTYPHLINI.

Type species: *Australiotyphluscabbagensis* Pace, 2013, by original designation.

Family-group name: AUSTRALIOTYPHLINI Pace, 2013a: 99. Stem: *Australiotyphl*-.

***Australobolbus* Howden & Cooper, 1977**: 19. Current status: valid genus in BOLBOCERATIDAE: BOLBOCERATINAE: BOLBOCERATINI.

Type species: *Bolbocerasobscurius* Blackburn, 1904, by original designation.

Family-group name: AUSTRALOBOLBINI Nikolajev, 1996. Stem: *Australobolb*-.

***Austroesthetus* Oke, 1933**: 112. Current status: valid genus in STAPHYLINIDAE: EUAESTHETINAE: AUSTROESTHETINI. Note: the original spelling was *Austroesthethus*, but *Austroesthetus*, an incorrect subsequent spelling, is in prevailing usage, attributed to the publication of the original spelling, and therefore deemed to be the correct original spelling (ICZN 1999a, Article 33.3.1).

Type species: *Austroesthetuspasserculus* Oke, 1933, by original designation.

Family-group name: AUSTROAESTHETINI Cameron, 1944. Stem: *Austroesthet*-.

***Austropassalus* Mjöberg, 1917**: 11. Current status: valid genus in PASSALIDAE: PASSALINAE: MACROLININI.

Type species: *Austropassalushultgreni* Mjöberg, 1917, by monotypy.

Family-group name: AUSTROPASSALINAE Mjöberg, 1917. Stem: *Austropassal*-.

***Austrorhysus* Steel, 1966**: 297. Current status: valid genus in STAPHYLINIDAE: PROTEININAE: AUSTRORHYSINI.

Type species: *Anepiusraucus* Blackburn, 1902, by original designation.

Family-group name: AUSTRORHYSINI Newton & Thayer, 1995. Stem: *Austrorhys*-.

***Autalia* Leach in Samouelle, 1819**: 177. Current status: valid genus in STAPHYLINIDAE: ALEOCHARINAE: AUTALIINI.

Type species: *Staphylinusimpressus* G.-A. Olivier, 1795, by subsequent designation (Westwood 1838c: 20).

Family-group name: AUTALIIDES C.G. Thomson, 1859. Stem: *Autali*-.

***Automolius*Britton, 1978**: 7. Current status: valid genus in SCARABAEIDAE: SERICOIDINAE: AUTOMOLIINI. Note: new replacement name for *Automolus* Burmeister, 1855.

Type species: *Automolusangustulus* Burmeister, 1855, by monotypy (type fixation for *Automolus* Burmeister).

Family-group name: AUTOMOLIINIBritton, 1978. Stem: *Automoli*-.

***Automolus* Burmeister, 1855**: 192. Current status: invalid senior synonym of *Automolius*Britton, 1978 in SCARABAEIDAE: SERICOIDINAE: AUTOMOLIINI. Note: junior homonym of *Automolus* L. Reichenbach, 1853 [Aves].

Type species: *Automolusangustulus* Burmeister, 1855, by monotypy.

Family-group name: AUTOMOLINIBritton, 1957. Stem: *Automol*-. Note: family-group name permanently invalid (ICZN 1999a, Article 39).

***Auxenocerus* Jeannel, 1962a**: 324. Current status: valid genus in STAPHYLINIDAE: PSELAPHINAE: EUPLECTITAE: JUBINI.

Type species: *Auxenocerusbispina* Jeannel, 1962, by original designation.

Family-group name: AUXENOCERINI Jeannel, 1962. Stem: *Auxenocer*-.

***Auxesis* J. Thomson, 1858b**: 196. Current status: valid genus in CERAMBYCIDAE: CERAMBYCINAE: AUXESINI.

Type species: *Auxesisgabonicus* J. Thomson, 1858, by monotypy.

Family-group name: AUXÉSIDES Lacordaire, 1872. Stem: *Auxes*-.

†***Avitortor* Ponomarenko, 1977**: 42. Current status: valid genus in CROWN COLEOPTERA incertae sedis. Note: Lower Cretaceous (Russia).

Type species: †*Avitortorprimitivus* Ponomarenko, 1977, by original designation.

Family-group name: †AVITORTORINAE Nikolajev, 2007. Stem: *Avitortor*-.

†***Axelrodiellus* Zherikhin & Gratshev, 2004**: 66. Current status: valid genus in BRENTIDAE: EURHYNCHINAE: †AXELRODIELLINI. Note: Lower Cretaceous (Brazil).

Type species: †*Axelrodiellusruptus* Zherikhin & Gratshev, 2004, by original designation.

Family-group name: †AXEIRODIELLINI Legalov, 2009. Stem: *Axelrodiell*-.

***Axinidium* Sturm, 1843**: 327. Current status: valid genus in CARABIDAE: SCARITINAE: PROMECOGNATHINI.

Type species: *Axinidiumafricanum* Sturm, 1843, by monotypy.

Family-group name: AXINIDIINI Basilewsky, 1963. Stem: *Axinidi*-.

***Axonya* Andrewes, 1923b**: 679. Current status: junior synonym of *Broscodes* Bolívar y Pieltain, 1914 in CARABIDAE: BROSCINAE: BROSCINI: AXONYINA.

Type species: *Axonyachampioni* Andrewes, 1923, by monotypy.

Family-group name: AXONYINA Roig-Juñent, 2000. Stem: *Axony*-.

***Azya* Mulsant, 1850**: 928. Current status: valid genus in COCCINELLIDAE: COCCINELLINAE: COCCIDULINI.

Type species: *Azyaluteipes* Mulsant, 1850, by subsequent designation (Crotch 1874a: 279).

Family-group name: AZYAIRES Mulsant, 1850. Stem: *Azy*-.

***Babia* Chevrolat in Dejean, 1836a**: 417. Current status: valid genus in CHRYSOMELIDAE: CRYPTOCEPHALINAE: CLYTRINI: BABIINA.

Type species: *Clytraquadriguttata* G.-A. Olivier, 1791, by subsequent designation (Monrós 1953: 46).

Family-group name: BABIDEAE Lacordaire, 1848. Stem: *Babi*-.

***Bacanius* J.L. LeConte, 1853c**: 291. Current status: valid genus in HISTERIDAE: DENDROPHILINAE: BACANIINI.

Type species: *Bacaniustantillus* J.L. LeConte, 1853, by subsequent designation (Bickhardt 1916: 71).

Family-group name: BACANIINI Kryzhanovskij, 1976. Stem: *Bacani*-.

***Badister* Clairville, 1806**: 90, 91. Current status: valid genus in CARABIDAE: HARPALINAE: LICININI: LICININA.

Type species: *Carabusbipustulatus* Fabricius, 1792 (= *Carabusbullatus* Schrank, 1798), by monotypy.

Family-group name: BADISTIDAE Gistel, 1856. Stem: *Badister*-.

***Baeocera* Erichson, 1845a**: 4. Current status: valid genus in STAPHYLINIDAE: SCAPHIDIINAE: SCAPHISOMATINI. Note: name placed on the Official List of Generic Names in Zoology (ICZN 1982).

Type species: *Baeocerafalsata* Achard, 1920, by plenary power (see ICZN 1982). Note: name placed on the Official List of Specific Names in Zoology (ICZN 1982).

Family-group name: BAEOCERITAE Achard, 1924. Stem: *Baeocer*-.

***Baeoceridium* Reitter, 1889a**: 6. Current status: valid genus in STAPHYLINIDAE: SCAPHIDIINAE: SCAPHISOMATINI.

Type species: *Baeoceridiumdepressipes* Reitter, 1889, by monotypy.

Family-group name: BAEOCERIDIITAE Achard, 1924. Stem: *Baeoceridi*-.

***Bagous* Germar, 1817b**: 340. Current status: valid genus in CURCULIONIDAE: BAGOINAE.

Type species: *Curculiobinodulus* Herbst, 1795, by subsequent designation (Schönherr 1826: 21).

Family-group name: BAGOINA C.G. Thomson, 1859. Stem: *Bago*-. Note: nomen protectum (see Bouchard et al. 2011: 586).

†***Baissorhynchus* Zherikhin, 1977a**: 176. Current status: valid genus in CARIDAE: †BAISSORHYNCHINAE: †BAISSORHYNCHINI. Note: Lower Cretaceous (Russia).

Type species: †*Baissorhynchustarsalis* Zherikhin, 1977, by original designation.

Family-group name: †BAISSORHYNCHINI Zherikhin, 1993. Stem: *Baissorhynch*-. Note: more research is needed to clarify the correct position of †BAISSORHYNCHINAE as well as the tribe therein before the priority problem with the older the name †NANOPHYDINI can be addressed.

***Balaninus* Germar, 1817b**: 340. Current status: junior synonym of *Curculio* Linnaeus, 1758 in CURCULIONIDAE: CURCULIONINAE: CURCULIONINI: CURCULIONINA.

Type species: *Curculionucum* Linnaeus, 1758, by subsequent designation (Partington 1834: 292).

Family-group name: BALANINIDAE Gistel, 1848. Stem: *Balanin*-.

***Balanobius* Jekel, 1861**: 267. Current status: valid genus in CURCULIONIDAE: CURCULIONINAE: CURCULIONINI: BALANOBIINA. Note: this genus was listed as a junior synonym of “*Archarius* Gistel, 1856” by Alonso-Zarazaga and Lyal (1999: 73); however, “*Archarius* Gistel, 1856” is an incorrect spelling of *Archarias* A. Villa & G.B. Villa, 1833 (a junior homonym of *Archarias* Dejean, 1821 [Coleoptera: CURCULIONIDAE]) and therefore cannot be used as valid.

Type species: *Curculiocrux* Fabricius, 1777, by subsequent designation (Voss 1958: 88).

Family-group name: BALANOBIINA Prena, 2021: 752. Stem: *Balanobi*-. Note: new replacement name for ARCHARIINA Pelsue & O’Brien, 2011.

***Balgus* Fleutiaux, 1920**: 95, 97. Current status: valid genus in ELATERIDAE: THYLACOSTERNINAE. Note: this name was proposed simultaneously as a new replacement name for *Galba* Latreille, 1829 in EUCNEMIDAE, a junior homonym of *Galba* Schrank, 1803 [Mollusca], and as a new replacement name for *Pterotarsus* sensu Latreille, 1834 in ELATERIDAE (non Guérin-Méneville, 1829); this taxon is considered as valid in the family ELATERIDAE (e.g., Kundrata et al. 2019d).

Type species: *Melasistuberculosa* Dalman, 1823, by original designation.

Family-group name: BALGINAE Fleutiaux, 1926. Stem: *Balg*-.

†***Baltognathus* Brunke, Żyła & Solodovnikov in Brunke et al., 2019**: 179. Current status: valid genus in STAPHYLINIDAE: STAPHYLININAE: STAPHYLININI: †BALTOGNATHINA. Note: mid- Eocene (Baltic amber).

Type species: †*Baltognathusaenigmaticus* Brunke, Żyła & Solodovnikov, 2019, by original designation.

Family-group name: †BALTOGNATHINA Brunke, Żyła & Solodovnikov in Brunke et al., 2019: 175. Stem: *Baltognath*-.

†***Baltostigus* Jałoszyński, 2016**: 1060. Current status: valid genus in STAPHYLINIDAE: SCYDMAENINAE: MASTIGITAE: †BALTOSTIGINI. Note: Eocene (Lithuania; Poland; Russia; Ukraine).

Type species: †*Baltostigusantennatus* Jałoszyński, 2016, by original designation.

Family-group name: †BALTOSTIGINI Jałoszyński & Brunke in Jałoszyński et al., 2018: 638. Stem: *Baltostig*-.

***Barada* Raffray, 1891**: 314. Current status: junior synonym of *Euphalepsus* Reitter, 1883 in STAPHYLINIDAE: PSELAPHINAE: GONIACERITAE: BRACHYGLUTINI: BARADINA.

Type species: *Baradamucronata* Raffray, 1891, by monotypy.

Family-group name: BARADIINI Park, 1951. Stem: *Barad*-.

***Baraeus* J. Thomson, 1858b**: 163. Current status: valid genus in CERAMBYCIDAE: LAMIINAE: PTEROPLIINI.

Type species: *Baraeusaurisecator* J. Thomson, 1858, by monotypy.

Family-group name: BAROEIDES Lacordaire, 1872. Stem: *Barae*-.

***Baripus* Dejean, 1828**: 24. Current status: valid genus in CARABIDAE: BROSCINAE: BROSCINI: BARIPODINA.

Type species: *Molopsrivalis* Germar, 1823, by monotypy.

Family-group name: BARYPITAE Jeannel, 1941. Stem: *Baripod*-.

***Baris* Germar, 1817b**: 340. Current status: valid genus in CURCULIONIDAE: BARIDINAE: BARIDINI: BARIDINA.

Type species: *Curculioartemisiae* Herbst, 1795 (= *Curculioartemisiae* Panzer, 1794), by subsequent designation (Curtis 1839: pl. 766).

Family-group name: BARIDIDES Schönherr, 1836. Stem: *Barid*-.

***Barrosellus* Jeannel, 1951a**: 107. Current status: valid genus in STAPHYLINIDAE: PSELAPHINAE: GONIACERITAE: BARROSELLINI.

Type species: *Barrosellussphaeroides* Jeannel, 1951, by original designation.

Family-group name: BARROSELLINI Leleup, 1973. Stem: *Barrosell*-.

***Barymela* Weise, 1910a**: 459. Current status: valid genus in CHRYSOMELIDAE: CHRYSOMELINAE: CHRYSOMELINI.

Type species: *Barymelascutellaris* Weise, 1910, by monotypy.

Family-group name: BARYMELINI Bechyné, 1948. Stem: *Barymel*-.

***Barymerus* Lacordaire, 1865**: 259. Current status: valid genus in CURCULIONIDAE: BARIDINAE: MADARINI: BARYMERINA. Note: new replacement name for *Physomerus* Schönherr, 1844.

Type species: *Physomeruscalandroides* Boheman, 1844, by original designation (type fixation for *Physomerus* Schönherr).

Family-group name: BARYMÉRIDES Lacordaire, 1865. Stem: *Barymer*-.

***Barynotus* Germar, 1817b**: 341. Current status: valid genus in CURCULIONIDAE: ENTIMINAE: POLYDRUSITAE: GEONEMINI.

Type species: *Curculioobscurus* Fabricius, 1775, by subsequent designation (Schönherr 1823: 1142). Note: nomen protectum (see Alonso-Zarazaga 2013a: 68).

Family-group name: BARYNOTIDES Lacordaire, 1863. Stem: *Barynot*-.

***Baryodirus* Perreau, 2000**: 19. Current status: valid genus in LEIODIDAE: CHOLEVINAE: PTOMAPHAGINI: BARYODIRINA.

Type species: *Baryodirushammondi* Perreau, 2000, by original designation.

Family-group name: BARYODIRINA Perreau, 2000. Stem: *Baryodir*-.

***Barypeithes* Jacquelin du Val, 1854**: 13. Current status: valid genus in CURCULIONIDAE: ENTIMINAE: CYPHICERITAE: OMIINI.

Type species: *Barypeithesrufipes* Jacquelin du Val, 1854 (= *Omiassulcifrons* Boheman, 1843), by subsequent designation (Lacordaire 1863: 59).

Family-group name: BARYPEITHINI Pic, 1897: 298. Stem: *Barypeith*-. Note: this name was noted by Alonso-Zarazaga (2013b: 71) but overlooked in Bouchard et al. (2011), Bouchard and Bousquet (2020a).

***Bascanus* Péringuey, 1896**: 539, 540. Current status: valid genus in CARABIDAE: HARPALINAE: BASCANINI.

Type species: *Bascanusgracilis* Péringuey, 1896, by subsequent designation (Basilewsky 1953: 47).

Family-group name: BASCANINI Basilewsky, 1953. Stem: *Bascan*-.

***Basilepta* Baly, 1860**: 23. Current status: valid genus in CHRYSOMELIDAE: EUMOLPINAE: TYPOPHORINI.

Type species: *Basileptalongipes* Baly, 1860, by original designation.

Family-group name: BASILEPTINI Chûjô, 1956. Stem: *Basilept*-.

***Basiprionota* Chevrolat in Dejean, 1836a**: 367. Current status: valid genus in CHRYSOMELIDAE: CASSIDINAE: BASIPRIONOTINI.

Type species: *Cassidaoctopunctata* Fabricius, 1787, by monotypy.

Family-group name: BASIPRIONOTINI Gressitt, 1952. Stem: *Basiprionot*-.

***Basipta* Chevrolat, 1842a**: 489. Current status: valid genus in CHRYSOMELIDAE: CASSIDINAE: CASSIDINI.

Type species: *Basiptaglauca* Chevrolat, 1842, by monotypy. Note: combined description of a new nominal genus and a single new nominal species (ICZN 1999a, Article 12.2.6).

Family-group name: BASIPTITES Chapuis, 1875. Stem: *Basipt*-.

***Basiptera* J. Thomson, 1864**: 204. Current status: valid genus in CERAMBYCIDAE: CERAMBYCINAE: BASIPTERINI.

Type species: *Basipteracastaneipennis* J. Thomson, 1864, by original designation.

Family-group name: BASIPTERINI Fragoso, Monné & Campos Seabra, 1987. Stem: *Basipter*-.

***Basitoxus* Audinet-Serville, 1832**: 174. Current status: valid genus in CERAMBYCIDAE: PRIONINAE: MACROTOMINI: BASITOXINA.

Type species: *Basitoxusarmatus* Audinet-Serville, 1832 (= *Prionusmegacephalus* Germar, 1823), by subsequent designation (E. Desmarest 1860: 307).

Family-group name: BASITOXI Lameere, 1912. Stem: *Basitox*-.

***Basitropis* Jekel, 1855**: 90. Current status: valid genus in ANTHRIBIDAE: ANTHRIBINAE: BASITROPINI.

Type species: *Basitropisnitidicutis* Jekel, 1855, by original designation. Note: the type species was listed as *nitidicollis* on page 91, which was corrected to *nitidicutis* on page 237 in the same work (Jekel 1855).

Family-group name: BASITROPIDES Lacordaire, 1865. Stem: *Basitrop*-.

***Bathyris* J.L. LeConte, 1874a**: 462. Current status: junior synonym of *Colecerus* Schönherr, 1840 in CURCULIONIDAE: ENTIMINAE: ENTIMITAE: EUDIAGOGINI.

Type species: *Bathyrisdispar* J.L. LeConte, 1874, by monotypy.

Family-group name: BATHYRINI J.L. LeConte, 1874. Stem: *Bathyrin*-.

***Bathyscia* Schiødte, 1848**: 79. Current status: valid genus in LEIODIDAE: CHOLEVINAE: LEPTODIRINI: BATHYSCIINA.

Type species: *Bathysciamontana* Schiødte, 1848, by subsequent designation (Jeannel 1910: 16).

Family-group name: BATHYSCIAE G.H. Horn, 1880. Stem: *Bathysci*-.

***Bathysciotes* Jeannel, 1910**: 15. Current status: valid genus in LEIODIDAE: CHOLEVINAE: LEPTODIRINI: BATHYSCIOTINA.

Type species: *Adelopskhevenhuelleri* L. Miller, 1852, by original designation.

Family-group name: BATHYSCIOTINA Guéorguiev, 1974. Stem: *Bathysciot*-.

***Batobius* Fairmaire & Germain, 1863**: 268. Current status: valid genus in MYCTERIDAE: EURYPINAE.

Type species: *Batobiusruficollis* Fairmaire & Germain, 1863, by subsequent designation (Spilman 1954: 87).

Family-group name: BATOBIINA Seidlitz, 1917. Stem: *Batobi*-.

***Batocera* Dejean, 1835**: 341. Current status: valid genus in CERAMBYCIDAE: LAMIINAE: BATOCERINI.

Type species: *Cerambyxrubus* Linnaeus, 1758, by subsequent designation (Blanchard 1845a: 175).

Family-group name: BATOCERITAE J. Thomson, 1864. Stem: *Batocer*-.

***Batonota* Hope, 1839**: 98. Current status: junior synonym of *Dorynota* Chevrolat, 1836 in CHRYSOMELIDAE: CASSIDINAE: DORYNOTINI.

Type species: *Cassidabidens* Fabricius, 1781, by original designation.

Family-group name: BATONOTITAE Spaeth, 1923. Stem: *Batonot*-.

***Batrisus* Aubé, 1833**: 12, 45. Current status: valid genus in STAPHYLINIDAE: PSELAPHINAE: BATRISITAE: BATRISINI: BATRISINA.

Type species [suggested]: *Batrisusformicarius* Aubé, 1833, by subsequent designation (Duponchel 1842a: 506). Note: the valid type species fixation is that of Westwood (1838c: 21), who selected *Bryaxisnigriventris* Denny, 1825 (= *Pselaphusvenustus* H.G.L. Reichenbach, 1816), a species currently placed in the genus *Batrisodes* Reitter, 1882 (e.g., see Newton and Chandler 1989: 8); an application to the Commission is necessary to fix *Batrisusformicarius* Aubé, 1833 as the type species of *Batrisus* Aubé, 1833.

Family-group name: BATRISINI Reitter, 1882. Stem: *Batris*-.

***Batulius* J.L. LeConte, 1851a**: 148. Current status: valid genus in TENEBRIONIDAE: PIMELIINAE: ANEPSIINI.

Type species: *Batuliussetosus* J.L. LeConte, 1851, by subsequent designation (Casey 1907: 497).

Family-group name: BATULIINI G.H. Horn, 1870. Stem: *Batuli*-.

***Beccaria* Gorham, 1885b**: 521. Current status: invalid senior synonym of *Beccariola* Arrow, 1943 in ENDOMYCHIDAE: LYCOPERDININAE. Note: junior homonym of *Beccaria* Trinchese, 1870 [Mollusca].

Type species: *Beccariapapuensis* Gorham, 1885, by monotypy.

Family-group name: BECCARIINI Arrow, 1925. Stem: *Beccari*-. Note: family-group name permanently invalid (ICZN 1999a, Article 39).

***Belionota* Eschscholtz, 1829b**: 8, 9. Current status: valid genus in BUPRESTIDAE: BUPRESTINAE: ACTENODINI.

Type species: *Belionotasagittaria* Eschscholtz, 1829, by monotypy.

Family-group name: BELIONOTINA Jakobson, 1913. Stem: *Belionot*-.

***Beliophorus* Eschscholtz, 1829a**: 34. Current status: valid genus in ELATERIDAE incertae sedis.

Type species [suggested]: *Beliophoruscebrionoides* Eschscholtz, 1829, by subsequent designation (Lacordaire 1857: 162). Note: the valid type species fixation is that of Duponchel (1842a: 532), who selected *Elatermucronatus* G.-A. Olivier, 1792, but that species is the type species of *Oxynopterus* Hope, 1842; an application to the Commission is necessary to fix *Beliophoruscebrionoides* Eschscholtz, 1829 as the type species of *Beliophorus* Eschscholtz, 1829.

Family-group name: BELIOPHORINA Jakobson, 1913. Stem: *Beliophor*-.

***Belohina* Paulian, 1959**: 42. Current status: valid genus in BELOHINIDAE.

Type species: *Belohinainexpectata* Paulian, 1959, by original designation.

Family-group name: BELOHININAE Paulian, 1959. Stem: *Belohin*-.

***Belopherus* Schönherr, 1833**: 6, 334. Current status: junior synonym of *Belorhynchus* Berthold, 1827 in BRENTIDAE: BRENTINAE: BRENTINI: ARRENODINA.

Type species: *Brentusmilitaris* G.-A. Olivier, 1807, by original designation.

Family-group name: BÉLOPHÉRIDES Lacordaire, 1865. Stem: *Belopher*-.

***Belopus* Gebien, 1911**: 459. Current status: valid genus in TENEBRIONIDAE: LAGRIINAE: BELOPINI. Note: nomen protectum (see Bouchard and Bousquet 2020b: 6); new replacement name for *Calcar* Dejean, 1821.

Type species: *Tenebrioelongatus* Herbst, 1797, by monotypy (type fixation for *Calcar* Dejean).

Family-group name: BELOPINAE Reitter, 1917. Stem: *Belop*-.

***Belorhynchus* Berthold, 1827**: 384. Current status: valid genus in BRENTIDAE: BRENTINAE: BRENTINI: ARRENODINA.

Type species: *Curculionasutus* Fabricius, 1781, by subsequent monotypy (Latreille 1828b: 595).

Family-group name: BÉLORHYNCHIDES Lacordaire, 1865. Stem: *Belorhynch*-.

***Belus* Schönherr, 1823**: 1137. Current status: junior synonym of *Rhinotia* W. Kirby, 1819 in BELIDAE: BELINAE: BELITAE: BELINI: BELINA.

Type species: *Curculiosemipunctatus* Fabricius, 1775, by original designation.

Family-group name: BELIDES Schönherr, 1826. Stem: *Bel*-.

***Bembidarenas* Erwin, 1972**: 8. Current status: valid genus in CARABIDAE: TRECHINAE: BEMBIDARENINI.

Type species: *Bembidionreicheellum* Csiki, 1929, by original designation.

Family-group name: BEMBIDARENINI Maddison & Erwin in Maddison et al., 2019: 173. Stem: *Bembidaren*-.

***Bembidion* Latreille, 1802b**: 82. Current status: valid genus in CARABIDAE: TRECHINAE: BEMBIDIINI.

Type species: *Carabusquadriguttatus* Fabricius, 1775 [p. 248] (= *Cicindelaquadrimaculata* Linnaeus, 1761), by subsequent designation (Andrewes 1935: 17). Note: Andrewes (1935: 17) designated *Cicindelaquadrimaculata* Linnaeus, 1761 as type species of *Bembidion* Latreille, 1802, a species not originally included, but since he listed that species in synonymy with *Carabusquadriguttatus* Fabricius, 1775, a species originally included in *Bembidion*, he is deemed to have designated the latter species as type species (ICZN 1999a, Article 69.2.2).

Family-group name: BEMBIDIIDAE Stephens, 1827. Stem: *Bembidi*-.

†***Berendtimirus* J.R. Winkler, 1987**: 52. Current status: valid genus in †BERENDTIMIRIDAE: †BERENDTIMIRINAE. Note: [geological time not mentioned] (Baltic amber).

Type species: †*Berendtimirusprogenitor* J.R. Winkler, 1987, by original designation.

Family-group name: †BERENDTIMIRIDAE J.R. Winkler, 1987. Stem: *Berendtimir*-.

***Berginus* Erichson, 1846**: 405. Current status: valid genus in MYCETOPHAGIDAE: BERGININAE.

Type species: *Berginustamarisci* Erichson, 1846, by monotypy. Note: this species is usually credited to Wollaston (1854: 195) in the literature but Erichson (1846), when describing the genus *Berginus* in his key, listed one species by name, providing therefore a combined description of a new nominal genus and a new nominal species (ICZN 1999a, Article 12.2.6).

Family-group name: BERGINI Leng, 1920. Stem: *Bergin*-.

***Berosus* Leach, 1817**: 92. Current status: valid genus in HYDROPHILIDAE: HYDROPHILINAE: BEROSINI. Note: name placed on the Official List of Generic Names in Zoology (ICZN 1990a).

Type species: *Dytiscusluridus* Linnaeus, 1761, by monotypy (see ICZN 1990a). Note: name placed on the Official List of Specific Names in Zoology (ICZN 1990a).

Family-group name: BÉROSAIRES Mulsant, 1844. Stem: *Beros*-.

***Betschia* Dajoz, 1980a**: 135. Current status: valid genus in TENEBRIONIDAE: DIAPERINAE: GNATHIDIINI: BETSCHIINA.

Type species: *Betschiaminuta* Dajoz, 1980, by original designation.

Family-group name: BETSCHIINI Dajoz, 1980. Stem: *Betschi*-.

***Bibloplectus* Reitter, 1882b**: 529. Current status: valid genus in STAPHYLINIDAE: PSELAPHINAE: EUPLECTITAE: TRICHONYCHINI: PANAPHANTINA.

Type species: *Pselaphusambiguus* H.G.L. Reichenbach, 1816, by subsequent designation (R. Lucas 1920: 137).

Family-group name: BIBLOPLECTINA Jeannel, 1959. Stem: *Bibloplect*-.

***Bibloporellus* Jeannel, 1949a**: 47. Current status: valid genus in STAPHYLINIDAE: PSELAPHINAE: EUPLECTITAE: TRICHONYCHINI: BIBLOPORINA.

Type species: *Bibloporellusmicrophthalmus* Jeannel, 1949, by original designation.

Family-group name: BIBLOPORELLINA Jeannel, 1952. Stem: *Bibloporell*-.

***Bibloporus* C.G. Thomson, 1859**: 53. Current status: valid genus in STAPHYLINIDAE: PSELAPHINAE: EUPLECTITAE: TRICHONYCHINI: BIBLOPORINA.

Type species: *Euplectusbicolor* Denny, 1825, by original designation.

Family-group name: BIBLOPORINI Park, 1951. Stem: *Biblopor*-.

***Bidessus* Sharp, 1880**: cxlviii. Current status: valid genus in DYTISCIDAE: HYDROPORINAE: BIDESSINI. Note: name placed on the Official List of Generic Names in Zoology (ICZN 2021a).

Type species: *Dytiscusunistriatus* Schrank, 1781, by plenary power (see ICZN 2021a). Note: name placed on the Official List of Specific Names in Zoology (ICZN 2021a).

Family-group name: BIDESSINI Sharp, 1880. Stem: *Bidess*-.

***Bimia* A. White, 1850**: 13. Current status: valid genus in CERAMBYCIDAE: CERAMBYCINAE: BIMIINI.

Type species: *Bimiabicolor* A. White, 1850, by monotypy.

Family-group name: BIMIIDES Lacordaire, 1868. Stem: *Bimi*-.

***Biophytus* Guillebeau, 1894**: 279, 295. Current status: junior synonym of *Pseudolibrus* Flach, 1889 in PHALACRIDAE: PHALACRINAE.

Type species: *Biophytusgrouvellei* Guillebeau, 1894, by original designation.

Family-group name: BIOPHYTINI Guillebeau, 1894. Stem: *Biophyt*-.

***Bioplanes* Mulsant, 1854**: 144. Current status: valid genus in TENEBRIONIDAE: BLAPTINAE: DENDARINI: DENDARINA.

Type species: *Bioplanesmeridionalis* Mulsant, 1854, by monotypy.

Family-group name: BIOPLANINA Reikhardt, 1936. Stem: *Bioplanet*-.

***Biphyllus* Dejean, 1821**: 102. Current status: valid genus in BIPHYLLIDAE.

Type species: *Dermesteslunatus* Fabricius, 1787, by monotypy.

Family-group name: DIPHYLLIDAE J.L. LeConte, 1861. Stem: *Biphyll*-.

***Bitoma* Herbst, 1793**: 25. Current status: valid genus in ZOPHERIDAE: COLYDIINAE: SYNCHITINI.

Type species: *Tritomacrenata* Fabricius, 1775, by subsequent designation (Latreille 1810: 431). Note: even if Latreille’s designation was for *Ditoma* (as “*Ditome*”), an unjustified emendation of *Bitoma* by Illiger (1807b: 320), both available genus-group names have the same type species (ICZN 1999a, Article 67.8).

Family-group name: DITOMIDAE Imhoff, 1856. Stem: *Bitom*-.

***Bius* Dejean, 1834**: 205. Current status: valid genus in TENEBRIONIDAE: TENEBRIONINAE: TENEBRIONINI.

Type species: *Trogositathoracicus* Fabricius, 1792, by monotypy.

Family-group name: BIUINI Skopin, 1978. Stem: *Bi*-.

***Blaesia* Burmeister, 1842**: 615. Current status: valid genus in SCARABAEIDAE: CETONIINAE: GYMNETINI: BLAESIINA.

Type species: *Blaesiaatra* Burmeister, 1842, by monotypy.

Family-group name: BLAESIAE Schoch, 1895. Stem: *Blaesi*-.

***Blaps* Fabricius, 1775**: 254. Current status: valid genus in TENEBRIONIDAE: BLAPTINAE: BLAPTINI: BLAPTINA.

Type species: *Tenebriomortisagus* Linnaeus, 1758, by subsequent designation (Latreille 1810: 429).

Family-group name: BLAPSIDA Leach, 1815. Stem: *Blapt*-.

***Blapstinus* Dejean, 1821**: 66. Current status: valid genus in TENEBRIONIDAE: BLAPTINAE: OPATRINI: BLAPSTININA.

Type species: *Blapspunctata* Fabricius, 1792, by monotypy.

Family-group name: BLAPSTINITES Mulsant & Rey, 1853. Stem: *Blapstin*-.

***Blastophila* Gistel, 1856**: 370. Current status: junior synonym of *Trachodes* Germar, 1823 in CURCULIONIDAE: MOLYTINAE: TRACHODINI.

Type species: *Curculiohispidus* Linnaeus, 1758, by monotypy.

Family-group name: BLASTOPHILADAE Gistel, 1856. Stem: *Blastophil*-.

***Bledius* Leach in Samouelle, 1819**: 174. Current status: valid genus in STAPHYLINIDAE: OXYTELINAE: BLEDIINI.

Type species: *Staphylinusarmatus* Panzer, 1799 (= *Staphylinustricornis* Herbst, 1784), by monotypy.

Family-group name: BLEDIINI Ádám, 2001. Stem: *Bledi*-.

***Blepharhymenus* Solier, 1849**: 339. Current status: valid genus in STAPHYLINIDAE: ALEOCHARINAE: OXYPODINI: DINARDINA.

Type species: *Blepharhymenussulcicollis* Solier, 1849, by monotypy.

Family-group name: BLEPHARRHYMENI Klimaszewski & Peck, 1986. Stem: *Blepharhymen*-.

***Blepharida* Chevrolat in Dejean, 1836a**: 394. Current status: valid genus in CHRYSOMELIDAE: GALERUCINAE: ALTICINI.

Type species: *Chrysomelameticulosus* G.-A. Olivier, 1808 (= *Chrysomelarhois* Forster, 1771 [as *rhoi*]), by subsequent designation (Chevrolat 1842a: 606).

Family-group name: BLÉPHARIDITES Chapuis, 1875. Stem: *Blepharid*-.

***Bleusea* Bedel, 1897**: 345. Current status: valid genus in CARABIDAE: HARPALINAE: HARPALINI: HARPALINA.

Type species: *Bleuseadeserticola* Bedel, 1897, by monotypy.

Family-group name: BLEUSEI Antoine, 1959. Stem: *Bleuse*-.

***Blosyrus* Schönherr, 1823**: 1139. Current status: valid genus in CURCULIONIDAE: ENTIMINAE: POLYDRUSITAE: BLOSYRINI.

Type species: *Curculiooniscus* G.-A. Olivier, 1807, by original designation.

Family-group name: BLOSYRIDES Lacordaire, 1863. Stem: *Blosyr*-.

***Boganium* Sen Gupta & Crowson, 1966**: 70. Current status: valid genus in BOGANIIDAE: BOGANIINAE.

Type species: *Boganiumarmstrongi* Sen Gupta & Crowson, 1966, by original designation.

Family-group name: BOGANIIDAE Sen Gupta & Crowson, 1966. Stem: *Bogani*-.

***Bolbelasmus* Boucomont, 1911**: 335. Current status: valid genus in BOLBOCERATIDAE: BOLBOCERATINAE: BOLBELASMINI.

Type species: *Bolbocerusbocchus* Erichson, 1841, by subsequent designation (Sharp 1912: 205). Note: the type species usually listed for this genus is *Bolbocerasgallicum* Mulsant, 1842 (e.g., Branco 2007: 8; Nikolajev et al. 2016: 33) by subsequent designation in Cartwright (1953: 97) but Sharp’s fixation is older; both nominal species are currently included in the nominotypical subgenus of *Bolbelasmus*.

Family-group name: BOLBELASMINI Iablokoff-Khnzorian, 1977. Stem: *Bolbelasm*-.

***Bolboceras* W. Kirby, 1819**: 459. Current status: valid genus in BOLBOCERATIDAE: BOLBOCERATINAE: BOLBOCERATINI. Note: name not given priority over *Odonteus* Samouelle, 1819 whenever the two are considered synonyms and *Bolboceras* W. Kirby, 1819 placed on the Official List of Generic Names in Zoology (ICZN 2006a).

Type species: *Scarabaeusquadridens* Fabricius, 1781, by plenary power (see ICZN 2006a). Note: name placed on the Official List of Specific Names in Zoology (ICZN 2006a).

Family-group name: BOLBOCÉRAIRES Mulsant, 1842. Stem: *Bolbocerat*-.

***Bolbochromus* Boucomont, 1909**: 117. Current status: valid genus in BOLBOCERATIDAE: BOLBOCERATINAE: BOLBOCHROMINI.

Type species: *Bolboceraslaetus* Westwood, 1852, by subsequent designation (Sharp 1911: 231).

Family-group name: BOLBOCHROMINI Nikolajev, 1970. Stem: *Bolbochrom*-.

†***Boleopsis* Kirejtshuk & Nel, 2013**: 189. Current status: valid genus in †BOLEOPSIDAE. Note: lower Eocene (France).

Type species: †*Boleopsispolinae* Kirejtshuk & Nel, 2013, by original designation.

Family-group name: †BOLEOPSIDAE Kirejtshuk & Nel, 2013: 185. Stem: *Boleops*-.

***Bolitobius* Leach in Samouelle, 1819**: 176. Current status: valid genus in STAPHYLINIDAE: MYCETOPORINAE.

Type species: fixed by Schülke (1999: 982) according to Article 70.3.2 (ICZN 1999a) as *Megacronuscastaneus* Stephens, 1832, the taxonomic species involved in the misidentification of *Staphylinusanalis* Fabricius, 1787 (sensu Paykull, 1789) in the original designation by Samouelle (1819: 176). Note: the fixation of the type species by Schülke (1999: 982), although published before the start of the fourth edition of the Code, is valid (see ICZN 1999a, Article 86.1.1).

Family-group name: BOLITOBII G.H. Horn, 1877. Stem: *Bolitobi*-.

***Bolitochara* Mannerheim, 1830**: 75. Current status: valid genus in STAPHYLINIDAE: ALEOCHARINAE: HOMALOTINI: BOLITOCHARINA. Note: name placed on the Official List of Generic Names in Zoology (ICZN 1961a).

Type species: *Aleocharapulchra* Gravenhorst, 1806, by plenary power (see ICZN 1961a). Note: name placed on the Official List of Specific Names in Zoology (ICZN 1961a).

Family-group name: BOLITOCHARIDES C.G. Thomson, 1859. Stem: *Bolitochar*-.

***Bolitophagus* Illiger, 1798**: 100. Current status: valid genus in TENEBRIONIDAE: TENEBRIONINAE: BOLITOPHAGINI.

Type species [suggested]: *Silphareticulata* Linnaeus, 1767, by subsequent designation (C.G. Thomson 1859: 115). Note: the valid type species fixation is that of Curtis (1836: pl. 586), who selected *Opatrumagricola* Herbst, 1783, which is the type species of the genus *Eledona* Latreille, 1797 (see Bouchard and Bousquet 2020b: 8); an application to the Commission is necessary to fix *Silphareticulata* Linnaeus, 1767 as the type species of *Bolitophagus* Illiger, 1798.

Family-group name: BOLITOPHAGIDAE W. Kirby, 1837. Stem: *Bolitophag*-. Note: nomen protectum (see Bouchard et al. 2011: 415).

***Bonesia* Baly, 1865b**: 100. Current status: valid genus in CHRYSOMELIDAE: GALERUCINAE: HYLASPINI.

Type species: *Bonesiaclarkii* Baly, 1865, by original designation.

Family-group name: BONESIITES Laboissière, 1926. Stem: *Bonesi*-.

***Borborophorus* Hansen, 1990**: 321, 326. Current status: valid genus in HYDROPHILIDAE: CYLOMINAE.

Type species: *Borborophoruspubescens* Hansen, 1990, by original designation.

Family-group name: BORBOROPHORINI Hansen, 1991. Stem: *Borborophor*-.

***Boreaphilus* C.R. Sahlberg, 1832**: 433. Current status: valid genus in STAPHYLINIDAE: OMALIINAE: CORYPHIINI: BOREAPHILINA.

Type species: *Boreaphilushenningianus* C.R. Sahlberg, 1832, by monotypy.

Family-group name: BOREAPHILINA Zerche, 1990. Stem: *Boreaphil*-.

***Boreocypha* Klimaszewski & Langor in Klimaszewski et al., 2011**: 79. Current status: valid genus in STAPHYLINIDAE: ALEOCHARINAE: BOREOCYPHINI.

Type species: *Boreocyphawebsteri* Klimaszewski & Langor, 2011, by original designation.

Family-group name: BOREOCYPHINI Klimaszewski & Langor in Klimaszewski et al., 2011: 79. Stem: *Boreocyph*-.

***Boromorphus* Wollaston, 1854**: 492. Current status: valid genus in TENEBRIONIDAE: PIMELIINAE: BOROMORPHINI.

Type species: *Boromorphusmaderae* Wollaston, 1854 (= *Borostagenioides* P.H. Lucas, 1846), by monotypy.

Family-group name: BOROMORPHINI Skopin, 1978. Stem: *Boromorph*-.

***Boros* Herbst, 1797**: 318. Current status: valid genus in BORIDAE: BORINAE.

Type species: *Boroselongatus* Herbst, 1797 (= *Helopsschneideri* Panzer, 1796), by monotypy.

Family-group name: BORIDAE C.G. Thomson, 1859. Stem: *Bor*-.

***Bostrichus* Geoffroy, 1762**: 301. Current status: valid genus in BOSTRICHIDAE: BOSTRICHINAE: BOSTRICHINI. Note: name placed on the Official List of Generic Names in Zoology (ICZN 1994a).

Type species: *Dermestescapucinus* Linnaeus, 1758, by subsequent designation (Latreille 1810: 431; see ICZN 1994a). Note: name placed on the Official List of Specific Names in Zoology (ICZN 1994a).

Family-group name: BOSTRICHINI Latreille, 1802. Stem: *Bostrich*-.

***Bostrychopsis* Lesne, 1899**: 444, 524. Current status: valid genus in BOSTRICHIDAE: BOSTRICHINAE: BOSTRICHINI.

Type species: fixed by Ivie (2010: 29) according to Article 70.3.2 (ICZN 1999a) as *Bostrychopsisvillosula* Lesne, 1905, the taxonomic species involved in the misidentification of *Bostrichuscephalotes* G.-A. Olivier, 1790 (sensu Lesne, 1899), as designated subsequently by Chûjô (1937: 52). Note: see Lesne (1905: 298) for a discussion about the misidentification of the type species.

Family-group name: BOSTRYCHOPSINI Lesne, 1921. Stem: *Bostrychopse*-.

***Bothrideres* Dejean, 1835**: 312. Current status: valid genus in BOTHRIDERIDAE.

Type species: *Dermestescontractus* Fourcroy, 1785, by monotypy. Note: the species listed by Dejean (1835: 312) is “contractus Fabr.”, which corresponds to *Lyctuscontractus* Fabricius, 1792 where there is a reference to *Ipscontracta* G.-A. Olivier, 1790, where, in turn, there is a reference to *Dermestescontractus* Fourcroy, 1785; therefore, Fourcroy’s species is the type species.

Family-group name: BOTHRIDERINI Erichson, 1845. Stem: *Bothrider*-.

***Bothriophorus* Mulsant & Rey, 1853a**: 19. Current status: valid genus in LIMNICHIDAE: LIMNICHINAE: LIMNICHINI.

Type species: *Bothriophorusatomus* Mulsant & Rey, 1853, by monotypy.

Family-group name: BOTRIOPHORATES Mulsant & Rey, 1869. Stem: *Bothriophor*-.

***Bothriospila* Aurivillius, 1924**: 1. Current status: valid genus in CERAMBYCIDAE: CERAMBYCINAE: BOTHRIOSPILINI.

Type species: *Bothriospilaelegans* Aurivillius, 1924, by monotypy.

Family-group name: BOTHRIOSPILINAE Lane, 1950. Stem: *Bothriospil*-.

***Bothrorrhina* Burmeister, 1842**: 200. Current status: junior synonym of *Plaesiorrhina* Westwood, 1842 in SCARABAEIDAE: CETONIINAE: GOLIATHINI: RHOMBORHININA.

Type species: *Cetoniareflexa* Gory & Percheron, 1835, by subsequent designation (Pouillaude 1916: 56).

Family-group name: BOTHRORRHINAE Schoch, 1894. Stem: *Bothrorrhin*-.

***Bothrosternus* Eichhoff, 1868a**: 150. Current status: valid genus in CURCULIONIDAE: SCOLYTINAE: BOTHROSTERNINI.

Type species: *Bothrosternustruncatus* Eichhoff, 1868, by monotypy.

Family-group name: BOTHROSTERNINI W.F.H. Blandford, 1896. Stem: *Bothrostern*-.

***Bothynus* Hope, 1837**: 95. Current status: valid genus in SCARABAEIDAE: DYNASTINAE: PENTODONTINI: PENTODONTINA. Note: name placed on the Official List of Generic Names in Zoology (ICZN 2008a).

Type species: *Scarabaeusascanius* W. Kirby, 1819, by subsequent designation (Lacordaire 1855: 413; see ICZN 2008a). Note: name placed on the Official List of Specific Names in Zoology (ICZN 2008a).

Family-group name: BOTHYNIDAE Burmeister, 1847. Stem: *Bothyn*-.

***Botryonopa* Guérin-Méneville, 1840b**: 332. Current status: valid genus in CHRYSOMELIDAE: CASSIDINAE: BOTRYONOPINI. Note: the original spelling was *Bothryonopa* but the incorrect subsequent spelling *Botryonopa* is in prevailing usage, attributed to the original publication, and therefore deemed to be the valid name (ICZN 1999a, Article 33.3.1).

Type species: *Botryonopasanguinea* Guérin-Méneville, 1840, by subsequent designation (Baly 1858: 92).

Family-group name: BOTRYONOPITES Chapuis, 1875. Stem: *Botryonop*-.

***Brachiacantha* Chevrolat in Dejean, 1836a**: 434. Current status: valid genus in COCCINELLIDAE: COCCINELLINAE: HYPERASPIDINI: BRACHIACANTHINA.

Type species: *Coccinelladentipes* Fabricius, 1801, by subsequent designation (Crotch 1873c: 377).

Family-group name: BRACHYACANTHAIRES Mulsant, 1850. Stem: *Brachiacanth*-.

***Brachinus* Weber, 1801**: 22. Current status: valid genus in CARABIDAE: BRACHININAE: BRACHININI: BRACHININA.

Type species: *Carabuscrepitans* Linnaeus, 1758, by subsequent designation (Latreille 1810: 426).

Family-group name: BRACHINII Bonelli, 1810. Stem: *Brachin*-.

***Brachonyx* Schönherr, 1825**: 583. Current status: valid genus in CURCULIONIDAE: CURCULIONINAE: ANTHONOMINI.

Type species: *Curculioindigena* Herbst, 1795 (= *Curculiopineti* Paykull, 1792), by original designation.

Family-group name: BRACHONYCHINAE Joy, 1932. Stem: *Brachonych*-.

†***Brachycamacus* Poinar, Legalov & A.E. Brown, 2013**: 2. Current status: valid genus in CURCULIONIDAE: ENTIMINAE: POLYDRUSITAE: EUSTYLINI: †BRACHYCAMACINA. Note: lower Miocene (Dominican Republic)

Type species: †*Brachycamacusgyrommatus* Poinar, Legalov & A.E. Brown, 2013, by original designation.

Family-group name: †BRACHYCAMACINA Poinar, Legalov & A.E. Brown, 2013: 2. Stem: *Brachycamac*-.

***Brachyceropsis* Aurivillius, 1886**: 24. Current status: valid genus in CURCULIONIDAE: MOLYTINAE: BRACHYCEROPSEINI.

Type species: *Brachycerustuberculosus* Gyllenhal, 1833, by original designation.

Family-group name: BRACHYCEROPSEINAE Aurivillius, 1926. Stem: *Brachyceropse*-.

***Brachycerus* G.-A. Olivier, 1789a**: 36. Current status: valid genus in CURCULIONIDAE: BRACHYCERINAE: BRACHYCERINI.

Type species: *Curculiobarbarus* Linnaeus, 1758, by subsequent designation (Latreille 1810: 430). Note: no species were originally included in this genus by G.-A. Olivier (1789a: 36); the first ones were the 20 species listed by G.-A. Olivier (1790b: 181, 182).

Family-group name: BRACHYCERIDES Billberg, 1820. Stem: *Brachycer*-.

***Brachyderes* Schönherr, 1823**: 1140. Current status: valid genus in CURCULIONIDAE: ENTIMINAE: POLYDRUSITAE: BRACHYDERINI. Note: name placed on the Official List of Generic Names in Zoology (ICZN 1987a).

Type species: *Curculioincanus* Linnaeus, 1758, by original designation (see ICZN 1987a). Note: name placed on the Official List of Specific Names in Zoology (ICZN 1987a).

Family-group name: BRACHYDERIDES Schönherr, 1826. Stem: *Brachyder*-.

***Brachygluta* C.G. Thomson, 1859**: 54. Current status: valid genus in STAPHYLINIDAE: GONIACERITAE: BRACHYGLUTINI: BRACHYGLUTINA.

Type species: *Pselaphusfossulatus* H.G.L. Reichenbach, 1816, by original designation.

Family-group name: BRACHYGLUTINI Raffray, 1904. Stem: *Brachyglut*-.

***Brachygnathus* Perty, 1830**: 6. Current status: valid genus in CARABIDAE: HARPALINAE: PANAGAEINI: BRACHYGNATHINA.

Type species: *Brachygnathusmuticus* Perty, 1830, by subsequent designation (Lorenz 1998: 55).

Family-group name: BRACHYGNATHINI Basilewsky, 1946. Stem: *Brachygnath*-.

***Brachylobus* Chaudoir, 1876a**: 287. Current status: valid subgenus of *Chlaenius* Bonelli, 1810 in CARABIDAE: HARPALINAE: CHLAENIINI: CHLAENIINA.

Type species: *Chlaeniuslithophilus* Say, 1823, by monotypy.

Family-group name: BRACHYLOBINI Basilewsky & Grundmann, 1955. Stem: *Brachylob*-.

***Brachypsectra* J.L. LeConte, 1874b**: 55. Current status: valid genus in BRACHYPSECTRIDAE.

Type species: *Brachypsectrafulva* J.L. LeConte, 1874, by monotypy.

Family-group name: BRACHYPSECTRINI G.H. Horn, 1881. Stem: *Brachypsectr*-.

***Brachypteroma* Heyden, 1863**: 128. Current status: valid genus in CERAMBYCIDAE: CERAMBYCINAE: BRACHYPTEROMINI.

Type species: *Brachypteromaottomanum* Heyden, 1863, by monotypy.

Family-group name: BRACHYPTEROMINI Sama, 2008. Stem: *Brachypterom*- (ICZN 1999a, Article 29.4).

***Brachypterus* Kugelann, 1794**: 560. Current status: valid genus in KATERETIDAE: KATERETINAE. Note: name placed on the Official List of Generic Names in Zoology (ICZN 1999b).

Type species: *Dermestesurticae* Fabricius, 1792, by subsequent designation (C.G. Thomson 1859: 67; see ICZN 1999b). Note: name placed on the Official List of Specific Names in Zoology (ICZN 1999b).

Family-group name: BRACHYPTERINAE Erichson, 1845. Stem: *Brachypter*-.

***Brachypus* Schönherr, 1826**: 217. Current status: invalid senior synonym of *Brachygyius* G.A.K. Marshall, 1939 in CURCULIONIDAE: BRACHYCERINAE: TANYSPHYRINI. Note: junior homonym of *Brachypus* B. Meyer, 1814 [Aves]; Schönherr’s name was placed on the Official Index of Rejected and Invalid Generic Names in Zoology (see ICZN 1958a).

Type species: *Brachypuslixoides* Schönherr, 1826, by original designation.

Family-group name: BRACHYPI J.L. LeConte, 1876. Stem: *Brachypod*-. Note: family-group name permanently invalid (ICZN 1999a, Article 39).

***Brachyrhinus* Latreille, 1802b**: 200. Current status: invalid senior synonym of *Cryphiphorus* Stierlin, 1883 in CURCULIONIDAE: ENTIMINAE: OTIORHYNCHITAE: OTIORHYNCHINI. Note: name suppressed for the Principle of Priority and placed on the Official Index of Rejected and Invalid Generic Names in Zoology (ICZN 1972).

Type species: *Curculioligustici* Linnaeus, 1758, by subsequent designation (Pierce 1913: 422).

Family-group name: BRACHYRRHINIDAE Bedel, 1883. Stem: *Brachyrhin*-. Note: family-group name permanently invalid (ICZN 1999a, Article 39); name placed on the Official Index of Rejected and Invalid Family-group Names in Zoology (see ICZN 1972).

***Brachys* Dejean, 1833a**: 83. Current status: valid genus in BUPRESTIDAE: AGRILINAE: TRACHEINI: BRACHINA.

Type species: *Trachystesselatus* Fabricius, 1801, by monotypy.

Family-group name: BRACHES J.L. LeConte, 1861. Stem: *Brach*-.

***Brachysternus* Guérin-Méneville, 1831a**: pl. 3. Current status: valid genus in SCARABAEIDAE: RUTELINAE: ANOPLOGNATHINI: BRACHYSTERNINA.

Type species: *Brachysternusprasinus* Guérin-Méneville, 1831, by monotypy.

Family-group name: BRACHYSTERNIDAE Burmeister, 1844. Stem: *Brachystern*-.

***Brachytarsus* Schönherr, 1823**: 1135. Current status: junior synonym of *Anthribus* Geoffroy, 1762 in ANTHRIBIDAE: ANTHRIBINAE: ANTHRIBINI.

Type species: *Bruchusvarius* Fabricius, 1787 (= *Anthribusnebulosus* Forster, 1770), by original designation.

Family-group name: BRACHYTARSINA C.G. Thomson, 1859. Stem: *Brachytars*-.

***Bradybaenus* Dejean, 1829**: 160. Current status: valid genus in CARABIDAE: HARPALINAE: HARPALINI: HARPALINA.

Type species: *Carabusscalaris* G.-A. Olivier, 1790, by subsequent designation (Hope 1838a: 84).

Family-group name: BRADYBAENI Csiki, 1932. Stem: *Bradybaen*-. Note: senior homonym of BRADYBAENIDAE Pilsbry, 1934 (type genus *Bradybaena* Beck, 1837) in Gastropoda.

***Bradybatus* Germar, 1823**: 305. Current status: valid genus in CURCULIONIDAE: CURCULIONINAE: ANTHONOMINI.

Type species: *Bradybatuscreutzeri* Germar, 1823, by monotypy.

Family-group name: BRADYBATINI Pierce, 1919. Stem: *Bradybat*-. Note: junior homonym of BRADYBATINA Bonaparte, 1850 (type genus *Bradybates* Tschudi, 1838) in Amphibia.

***Bradycellus* Erichson, 1837**: 64. Current status: valid genus in CARABIDAE: HARPALINAE: HARPALINI: STENOLOPHINA.

Type species [suggested]: *Carabuscollaris* Paykull, 1798 (= *Acupalpuscaucasicus* Chaudoir, 1846), by subsequent designation (Andrewes 1935: 20). Note: the valid type species fixation is that of Westwood (1838c: 6), who selected *Harpalusplacidus* Gyllenhal, 1827, a species currently included in the genus *Trichocellus* Ganglbauer, 1892; an application to the Commission is necessary to fix *Carabuscollaris* Paykull, 1798 as the type species of *Bradycellus* Erichson, 1837.

Family-group name: BRADYCELLINI Jeannel, 1942. Stem: *Bradycell*-.

***Branchus* J.L. LeConte, 1862**: 222. Current status: valid genus in TENEBRIONIDAE: PIMELIINAE: BRANCHINI.

Type species: *Branchusfloridanus* J.L. LeConte, 1862, by monotypy.

Family-group name: BRANCHINI J.L. LeConte, 1862. Stem: *Branch*-.

***Brarus* Kuschel in Kuschel and May, 1997**: 16. Current status: valid genus in NEMONYCHIDAE: RHINORHYNCHINAE: MECOMACERINI: BRARINA.

Type species: *Brarusmystes* Kuschel, 1997, by original designation.

Family-group name: BRARINA Legalov, 2009. Stem: *Brar*-.

***Brasilucanus* Vulcano & Pereira, 1961**: 473. Current status: valid genus in LUCANIDAE: LUCANINAE: LUCANINI.

Type species: *Brasilucanusalvarengai* Vulcano & Pereira, 1961, by original designation.

Family-group name: BRASILUCANINI Nikolajev, 1999. Stem: *Brasilucan*-.

***Brathinus* J.L. LeConte, 1852f**: 156. Current status: valid genus in STAPHYLINIDAE: OMALIINAE: ANTHOPHAGINI.

Type species: *Brathinusnitidus* J.L. LeConte, 1852, by subsequent designation (Blackwelder 1952: 411). Note: R. Lucas (1920: 149) listed two species as type species and this is considered as a typification made in an ambiguous manner and is not available under the Code (ICZN 1999a, Article 67.5.3).

Family-group name: BRATHINIDAE J.L. LeConte, 1861. Stem: *Brathin*-.

†***Brenthorrhinoides* Gratshev & Zherikhin, 1996**: 119. Current status: valid genus in NEMONYCHIDAE: †BRENTHORRHININAE: †BRENTHORRHININI: †BRENTHORRHINOIDINA. Note: Upper Jurassic (Kazakhstan).

Type species: †*Brenthorrhinoidesmandibulatus* Gratshev & Zherikhin, 1996, by original designation.

Family-group name: †BRENTHORRHINOIDINI Legalov, 2003. Stem: *Brenthorrhinoid*-.

†***Brenthorrhinus* Arnoldi, 1977**: 172. Current status: valid genus in NEMONYCHIDAE: †BRENTHORRHININAE: †BRENTHORRHININI: †BRENTHORRHININA. Note: Upper Jurassic (Kazakhstan).

Type species: †*Brenthorrhinusmirabilis* Arnoldi, 1977, by original designation.

Family-group name: †BRENTHORRHININAE Arnoldi, 1977. Stem: *Brenthorrhin*-.

***Brentus* Fabricius, 1787**: 95. Current status: valid genus in BRENTIDAE: BRENTINAE: BRENTINI: BRENTINA.

Type species: *Curculioanchorago* Linnaeus, 1758, by subsequent designation (Latreille 1810: 430).

Family-group name: BRENTHIDES Billberg, 1820. Stem: *Brent*-.

***Brises* Pascoe, 1869a**: 145. Current status: valid genus in TENEBRIONIDAE: TENEBRIONINAE: HELEINI: HELEINA.

Type species: *Brisestrachynotoides* Pascoe, 1869, by monotypy.

Family-group name: BRISEINAE Carter, 1924. Stem: *Bris*-.

***Brittona* G.S. Medvedev & Lawrence, 1986**: 574. Current status: valid genus in TENEBRIONIDAE: DIAPERINAE: HYOCIINI: BRITTONINA.

Type species: *Brittonaminuta* G.S. Medvedev & Lawrence, 1986, by original designation.

Family-group name: BRITTONINA G.S. Medvedev & Lawrence, 1986. Stem: *Britton*-.

†***Brochocoleus* Hong, 1982**: 103. Current status: valid genus in OMMATIDAE: †BROCHOCOLEINAE. Note: Lower Jurassic/Late Cretaceous (China; Kazakhstan; Mongolia; Myanmar; Russia; Spain; United Kingdom).

Type species: †*Brochocoleuspunctatus* Hong, 1982, by original designation.

Family-group name: †BROCHOCOLEIDAE Hong, 1982. Stem: *Brochocole*-.

***Bromius* Chevrolat in Dejean, 1836a**: 412. Current status: valid genus in CHRYSOMELIDAE: EUMOLPINAE: BROMIINI. Note: name placed on the Official List of Generic Names in Zoology (ICZN 2012b).

Type species: *Chrysomelaobscura* Linnaeus, 1758, by subsequent designation (Monrós and Bechyné 1956: 1127; see ICZN 2012b). Note: name hereby placed on the Official List of Specific Names in Zoology (ICZN 2012b).

Family-group name: BROMIINAE Baly, 1865. Stem: *Bromi*-.

***Brontes* Fabricius, 1801a**: xix. Current status: junior synonym of *Uleiota* Latreille, 1797 in SILVANIDAE: BRONTINAE: BRONTINI.

Type species: *Cucujusflavipes* Fabricius, 1779 (= *Cerambyxplanatus* Linnaeus, 1761), by subsequent designation (Partington 1835: 632). Note: no species were originally included in this genus by Fabricius (1801a); the first ones were the five species listed in Fabricius (1801b: 97, 98).

Family-group name: BRONTITES Blanchard, 1845. Stem: *Bront*-.

***Broscus* Panzer, 1813**: 62. Current status: valid genus in CARABIDAE: BROSCINAE: BROSCINI: BROSCINA. Note: new replacement name for *Cephalotes* Bonelli, 1810.

Type species: *Carabuscephalotes* Linnaeus, 1758, by monotypy.

Family-group name: BROSCHIDAE Hope, 1838. Stem: *Brosc*-.

***Brotheus* Stephens, 1829b**: 13. Current status: valid genus in CURCULIONIDAE: BRACHYCERINAE: BRACHYCERINI.

Type species: *Curculioporcatus* Marsham, 1802 (= *Brachyceruspraemorsus* Thunberg, 1799), by monotypy.

Family-group name: BROTHEINAE G.A.K. Marshall, 1907. Stem: *Brothe*-. Note: junior homonym of BROTHEINI Simon, 1879 (type genus *Brotheas* C.L. Koch, 1837) in Scorpiones; because of the homonymy, the stem of the genus was changed to *Brotheus*- and the family group name to BROTHEUSINI by Lyal and Alonso-Zarazaga (2006: 24); however, cases involving homonymy of family-group names based on similar genus- group names must be referred to the Commission unless the senior homonym is a nomen oblitum (ICZN 1999a, Article 55.3.1).

***Bruchela* Dejean, 1821**: 78. Current status: valid genus in ANTHRIBIDAE: URODONTINAE.

Type species: *Anthribussericeus* Fabricius, 1801 (= *Bruchusrufipes* G.-A. Olivier, 1790), by subsequent designation (Schönherr 1823: 1134) (type fixation for *Urodon* Schönherr, 1823, an unnecessary new replacement name for *Bruchela* Dejean).

Family-group name: BRUCHELIDAE Pierce, 1916. Stem: *Bruchel*-.

***Bruchidius* Schilsky, 1905**: 41B. Current status: valid genus in CHRYSOMELIDAE: BRUCHINAE: BRUCHINI: ACANTHOSCELIDINA.

Type species: *Bruchusquinqueguttatus* G.-A. Olivier, 1795, by subsequent designation (Bridwell 1932: 104).

Family-group name: BRUCHIDIINI Bridwell, 1946. Stem: *Bruchidi*-.

***Bruchus* Linnaeus, 1767**: 604. Current status: valid genus in CHRYSOMELIDAE: BRUCHINAE: BRUCHINI: BRUCHINA. Note: the senior homonym *Bruchus*Geoffroy (1762: 163) was suppressed for both Principles of Priority and Homonymy and placed on the Official Index of Generic Names in Zoology while *Bruchus* Linnaeus, 1767 was placed on the Official List of Generic Names in Zoology (ICZN 1995b).

Type species: *Dermestespisorum* Linnaeus, 1758, by subsequent designation (Latreille 1810: 430; see ICZN 1995b). Note: name placed on the Official List of Specific Names in Zoology (ICZN 1995b).

Family-group name: BRUCHELAE Latreille, 1802. Stem: *Bruch*-.

***Bryaxis* Kugelann, 1794**: 580. Current status: valid genus in STAPHYLINIDAE: PSELAPHINAE: GONIACERITAE: BYTHININI: BYTHININA. Note: name placed on the Official List of Generic Names in Zoology (ICZN 1969b).

Type species: *Pselaphusbulbifer* H.G.L. Reichenbach, 1816, by plenary power (see ICZN 1969b). Note: name placed on the Official List of Specific Names in Zoology (ICZN 1969b).

Family-group name: BRYAXINA Jakobson, 1910. Stem: *Bryaxe*-.

***Bryaxis* Leach, 1817**: 81. Current status: invalid senior synonym of *Rybaxis* Saulcy, 1876 in STAPHYLINIDAE: PSELAPHINAE: GONIACERITAE: BRACHYGLUTINI: BRACHYGLUTINA. Note: junior homonym of *Bryaxis* Kugelann, 1794; name placed on the Official Index of Rejected and Invalid Generic Names in Zoology (see ICZN 1969b).

Type species: *Staphylinussanguineus* Linnaeus, 1758, by subsequent designation (Curtis 1830: pl. 315).

Family-group name: BRYAXES J.L. LeConte, 1861. Stem: *Bryaxe*-. Note: family-group name permanently invalid (ICZN 1999a, Article 39).

***Brychius* C.G. Thomson, 1859**: 11. Current status: valid genus in HALIPLIDAE.

Type species: *Dytiscuselevatus* Panzer, 1793, by original designation.

Family-group name: BRYCHIINI Ádám, 1996. Stem: *Brychi*-.

***Bubastes* Laporte & Gory, 1838**: 1. Current status: valid genus in BUPRESTIDAE: BUPRESTINAE: BUBASTINI.

Type species: *Bubastessphaenoida* Laporte & Gory, 1838, by monotypy.

Family-group name: BUBASTINI Obenberger, 1920. Stem: *Bubast*-.

***Bulaea* Mulsant, 1850**: 36, 69. Current status: valid genus in COCCINELLIDAE: COCCINELLINAE: COCCINELLINI.

Type species: *Coccinellalichatschovii* Hummel, 1827, by subsequent designation (Crotch 1874a: 105).

Family-group name: BULAEINI Savoyskaya, 1969. Stem: *Bulae*-.

***Bulis* Laporte & Gory, 1838**: 1. Current status: valid genus in BUPRESTIDAE: POLYCESTINAE: BULINI.

Type species: *Buprestisbivittata* Fabricius, 1801, by subsequent designation (Lacordaire 1857: 42).

Family-group name: BULISINA Bellamy, 1995. Stem: *Bul*-.

***Bumetopia* Pascoe, 1858**: 252. Current status: valid genus in CERAMBYCIDAE: LAMIINAE: HOMONOEINI.

Type species: *Bumetopiaoscitans* Pascoe, 1858, by monotypy.

Family-group name: BUMÉTOPIDES Lacordaire, 1872. Stem: *Bumetopi*-.

***Bunyaeus* Kuschel, 1994**: 612. Current status: valid genus in NEMONYCHIDAE: RHINORHYNCHINAE: MECOMACERINI: BUNYAEINA.

Type species: *Bunyaeusmonteithi* Kuschel, 1994, by original designation.

Family-group name: BUNYAEINA Legalov, 2023c: 21. Stem: *Bunyae*-. Note: BUNYAEINALegalov (2017a: 69) was published in a work that is nomenclaturally unavailable (ICZN 2012f, Article 8.5.3).

***Buprestis* Linnaeus, 1758**: 342, 408. Current status: valid genus in BUPRESTIDAE: BUPRESTINAE: BUPRESTINI: BUPRESTINA. Note: name placed on the Official List of Generic Names in Zoology (ICZN 1994f).

Type species: *Buprestisoctoguttata* Linnaeus, 1758, by plenary power (see ICZN 1994f). Note: name placed on the Official List of Specific Names in Zoology (ICZN 1994f).

Family-group name: BUPRESTIDES Leach, 1815. Stem: *Buprest*-.

†***Burmocorynus* Legalov, 2020c**: 132. Current status: valid genus in †MESOPHYLETIDAE: †MESOPHYLETINAE. Note: mid-Cretaceous (Myanmar).

Type species: †*Burmocorynusjarzembowskii* Legalov, 2020, by original designation.

Family-group name: †BURMOCORYNINI Legalov, 2020c: 132. Stem: *Burmocoryn*-.

†***Burmolycus* Bocák, Li & Ellenberger, 2019**: 152. Current status: valid genus in LYCIDAE: DEXORINAE: DEXORINI: †BURMOLYCINA. Note: mid-Cretaceous (Myanmar).

Type species: †*Burmolycuscompactus* Bocák, Li & Ellenberger, 2019, by original designation.

Family-group name: †BURMOLYCINI Bocák, Li & Ellenberger, 2019: 150. Stem: *Burmolyc*-.

†***Burmomacer* Legalov, 2020c**: 129. Current status: valid genus in NEMONYCHIDAE: RHINORHYNCHINAE: †BURMOMACERATINI. Note: mid-Cretaceous (Myanmar).

Type species: †*Burmomacerkirejtshuki* Legalov, 2020, by original designation.

Family-group name: †BURMOMACERATINI Legalov, 2020c: 128. Stem: *Burmomacerat*-. Note: the names *Burmomacer* and BURMOMACERATINI were established in the printed version of this article (Legalov 2020c) and not in the electronic version that was made public in 2018 since it was not registered in Official Register of Zoological Nomenclature (ZooBank) before the electronic work was published (ICZN 2012f, Article 8.5.3); Clarke et al. (2018: 39) pre-emptively synonymized the unavailable BURMOMACERATINI Legalov, 2018 (as BURMOMACRINI) with MECOMACERINI; the correct spelling and status of this family-group name (which could be preserved as originally proposed if Article 29.4 is used (ICZN 1999a)) needs further attention.

***Byctiscus* C.G. Thomson, 1859**: 130. Current status: valid genus in ATTELABIDAE: RHYNCHITINAERHYNCHITITAE: BYCTISCINI: BYCTISCINA.

Type species: *Curculiopopuli* Linnaeus, 1758, by original designation.

Family-group name: BYCTISCINI Voss, 1923. Stem: *Byctisc*-.

***Byrrhidium* Harold, 1869a**: 96. Current status: treated as a valid genus in SCARABAEIDAE: SCARABAEINAE: BYRRHIDIINI [but see notes below]. Note: junior homonym of *Byrrhidium* Heer, 1864^1^ in Coleoptera: BYRRHIDAE; this name was first used by Harold in Gemminger and von Harold (1869a: 997) but it is not available from that publication since there is no description or indication, the sole species associated with the name being a nomen nudum; a substitute name will be proposed by the authors of BYRRHIDIINI in the near future (C. Deschodt, pers. comm. to P.B., 2022).

Type species: *Byrrhidiumovale* Harold, 1869, by monotypy.

Family-group name: BYRRHIDIINI Davis, Deschodt & Scholtz, 2019: 134. Stem: *Byrrhidi*-. Note: family-group name permanently invalid (ICZN 1999a, Article 39); a substitute name will be proposed by the authors of BYRRHIDIINI in the near future (C. Deschodt, pers. comm. to P.B., 2022).

***Byrrhus* Linnaeus, 1767**: 568. Current status: valid genus in BYRRHIDAE: BYRRHINAE: BYRRHINI. Note: the senior homonym *Byrrhus* Geoffroy, 1762 (and all other uses of the name prior to the publication of *Byrrhus* Linnaeus, 1767) was suppressed for both Principles of Priority and Homonymy and placed on the Official Index of Rejected and Invalid Generic Names in Zoology while *Byrrhus* Linnaeus, 1767 was placed on the Official List of Generic Names in Zoology (ICZN 1994a).

Type species: *Dermestespilula* Linnaeus, 1758, by subsequent designation (Latreille 1810: 428; see ICZN 1994a). Note: name placed on the Official List of Specific Names in Zoology (ICZN 1994a).

Family-group name: BYRRHII Latreille, 1804. Stem: *Byrrh*-.

***Byrsopages* Schönherr, 1842a**: 208. Current status: valid genus in CURCULIONIDAE: ENTIMINAE: ENTIMITAE: BYRSOPAGINI: BYRSOPAGINA.

Type species: *Byrsopagesvillosus* Bohemam, 1842, by original designation.

Family-group name: BYRSOPAGIDES Lacordaire, 1863. Stem: *Byrsopag*-.

***Byrsops* Germar, 1829**: 358. Current status: valid genus in CURCULIONIDAE: BRACHYCERINAE: BRACHYCERINI.

Type species: *Brachycerusquadratus* Wiedemann, 1821, by subsequent designation (Alonso- Zarazaga and Lyal 1999: 21, 62).

Family-group name: BYRSOPTIDES Germar, 1829. Stem: *Byrsop*-.

***Bythinoplectus* Reitter, 1882a**: 195. Current status: valid genus in STAPHYLINIDAE: PSELAPHINAE: EUPLECTITAE: BYTHINOPLECTINI: BYTHINOPLECTINA.

Type species: *Bythinoplectusfoveatus* Reitter, 1883, by subsequent monotypy (Reitter 1883a: 37).

Family-group name: BYTHINOPLECTINI L.W. Schaufuss, 1890. Stem: *Bythinoplect*-.

***Bythinus* Leach, 1817**: 82. Current status: valid genus in STAPHYLINIDAE: PSELAPHINAE: GONIACERITAE: BYTHININI: BYTHININA. Note: name placed on the Official List of Generic Names in Zoology (ICZN 1969b).

Type species: *Pselaphussecuriger* H.G.L. Reichenbach, 1816, by subsequent designation (Westwood 1838c: 21; see ICZN 1969b). Note: name placed on the Official List of Specific Names in Zoology (ICZN 1969b).

Family-group name: BYTHININI Raffray, 1890. Stem: *Bythin*-.

***Byturus* Latreille, 1797**: 69. Current status: valid genus in BYTURIDAE: BYTURINAE.

Type species: *Dermestestomentosus* DeGeer, 1774, by subsequent designation (Latreille 1810: 427). Note: no species were originally included in this genus by Latreille (1797); the first ones were the three species listed by Latreille (1802b: 134).

Family-group name: BYTURIDAE Gistel, 1848. Stem: *Bytur*-.

***Cachiporra* Chamorro & Konstantinov, 2011**: 50. Current status: valid genus in CHRYSOMELIDAE: LAMPROSOMATINAE: CACHIPORRINI.

Type species: *Cachiporraextremaglobosa* Chamorro & Konstantinov, 2011, by original designation.

Family-group name: CACHIPORRINI Chamorro & Konstantinov, 2011: 49. Stem: *Cachiporr*-.

†***Cacomorphocerus* C. Schaufuss, 1891a**: 57. Current status: valid genus in CANTHARIDAE: CANTHARINAE: †CACOMORPHOCERINI. Note: Eocene (Baltic and Rovno ambers).

Type species: †*Cacomorphoceruscerambyx* C. Schaufuss, 1891, by monotypy.

Family-group name: †CACOMORPHOCERINI Fanti & Kupryjanowicz, 2018: 150. Stem: *Cacomorphocer*-.

***Cacosceles* Newman, 1838**: 491. Current status: valid genus in CERAMBYCIDAE: PRIONINAE: CACOSCELINI.

Type species: *Cacoscelesoedipus* Newman, 1838, by monotypy.

Family-group name: CACOSCELITAE J. Thomson, 1861. Stem: *Cacoscel*-.

***Cacoscelis* Chevrolat in Dejean, 1836a**: 389. Current status: valid genus in CHRYSOMELIDAE: GALERUCINAE: ALTICINI.

Type species: *Chrysomelafamelica* Fabricius, 1787 (= *Alticamarginata* Fabricius, 1775), by subsequent designation (Monrós and Bechyné 1956: 1133).

Family-group name: CACOSCELINI Bechyné, 1968. Stem: *Cacoscelid*-.

***Cactopinus* O. Schwarz, 1899**: 11. Current status: valid genus in CURCULIONIDAE: SCOLYTINAE: CACTOPININI.

Type species: *Cactopinushubbardi* O. Schwarz, 1899, by monotypy.

Family-group name: CACTOPINAE Chamberlin, 1939. Stem: *Cactopin*-.

***Caecossonus* Gilbert, 1955**: 193. Current status: valid genus in CURCULIONIDAE: MOLYTINAE: LYMANTINI: CAECOSSONINA.

Type species: *Caecossonusdentipes* Gilbert, 1955, by monotypy.

Family-group name: CAECOSSONINA Osella, 1980. Stem: *Caecosson*-.

***Caelostomus* W.S. MacLeay, 1825**: 23. Current status: valid genus in CARABIDAE: HARPALINAE: DRIMOSTOMATINI.

Type species: *Anaulacuspicipes* W.S. MacLeay, 1825, by monotypy.

Family-group name: CAELOSTOMINA Burgeon, 1935. Stem: *Caelostom*-.

***Caenocara* C.G. Thomson, 1859**: 90. Current status: valid genus in PTINIDAE: DORCATOMINAE.

Type species: *Dorcatomabovistae* P.W.J. Müller, 1803, by original designation. Note: the authorship of the type species is credited to Hoffmann in the literature but Niehuis (2012: 2, 3, 42) showed that P.W.J. Müller is the author.

Family-group name: CAENOCARINI Böving, 1927. Stem: *Caenocar*-.

***Caenocrypticus* Gebien, 1920**: 139. Current status: valid genus in TENEBRIONIDAE: PIMELIINAE: CAENOCRYPTICINI.

Type species: *Caenocrypticusuncinatus* Gebien, 1920, by monotypy.

Family-group name: CAENOCRYPTICINI C. Koch, 1958. Stem: *Caenocryptic*-.

***Caenoscelis* C.G. Thomson, 1863**: 266. Current status: valid genus in CRYPTOPHAGIDAE: CRYPTOPHAGINAE: CAENOSCELINI.

Type species: *Cryptophagusferrugineus* C.R. Sahlberg, 1820, by monotypy.

Family-group name: CAENOSCELINI Casey, 1900. Stem: *Caenoscel*-.

***Calandra* Clairville, 1798**: 62, pl. 2. Current status: invalid senior synonym of *Sphenophorus* Schönherr, 1838 in CURCULIONIDAE: DRYOPHTHORINAE: SPHENOPHORINI: SPHENOPHORINA. Note: two original spellings were used, including *Calendra* (p. 62); Latreille (1806: 270) acted as First Reviser (ICZN 1999a, Article 24.2.3) and selected the spelling *Calandra*; the name *Calendra* is suppressed for the Principle of Priority and placed on the Official Index of Rejected and Invalid Generic Names in Zoology (ICZN 1959b, as *Calandra* Clairville & Schellenberg, 1798).

Type species: *Curculioabbreviatus* Fabricius, 1787, by subsequent designation (Latreille 1810: 431). Note: name validated by the Commission (i.e., not a primary homonym of *Curculioabbreviatus* Linnaeus, 1758) and placed on the Official List of Specific Names in Zoology (ICZN 1959b).

Family-group name: CALANDRAEDES Billberg, 1820. Stem: *Calandr*-. Note: family-group name permanently invalid (ICZN 1999a, Article 39).

***Calandra* Gistel, 1848b**: 136. Current status: junior synonym of *Sitophilus* Schönherr, 1838 in CURCULIONIDAE: DRYOPHTHORINAE: LITOSOMINI: SITOPHILINA. Note: unnecessary new replacement name for *Sitophilus* Schönherr, 1838; name placed on the Official Index of Rejected and Invalid Generic Names in Zoology (see ICZN 1959b).

Type species: *Curculiooryzae* Linnaeus, 1763, by original designation (type fixation for *Sitophilus* Schönherr).

Family-group name: CALANDRINA C.G. Thomson, 1859. Stem: *Calandr*-. Note: family-group name permanently invalid (ICZN 1999a, Article 39).

***Calathus* Bonelli, 1810**: Tab. Syn. Current status: valid genus in CARABIDAE: HARPALINAE: SPHODRINI: CALATHINA.

Type species: *Carabuscisteloides* Panzer, 1793 (= *Carabusfuscipes* Goeze, 1777), by subsequent designation (Curtis 1827: pl. 184). Note: no species were originally included in this genus by Bonelli (1810); the first ones were the two species listed by Panzer (1813: 60); Goeze’s (1777) work is not consistently binominal (see ICZN 2021a) and therefore an application to the Commission is necessary to retain *Carabusfuscipes* Goeze, 1777 as the valid name for the type species.

Family-group name: CALATHIDAE Laporte, 1834. Stem: *Calath*-.

***Calcar* Dejean, 1821**: 67. Current status: invalid senior synonym of *Belopus* Gebien, 1911 in TENEBRIONIDAE: LAGRIINAE: BELOPINI. Note: junior homonym of *Calcar* de Montfort, 1810 [Mollusca].

Type species: *Tenebrioelongatus* Herbst, 1797, by monotypy.

Family-group name: CALCARIENS Mulsant, 1854. Stem: *Calcar*-. Note: family-group name permanently invalid (ICZN 1999a, Article 39).

***Calcodes* Westwood, 1834**: 118. Current status: valid genus in LUCANIDAE: LUCANINAE: LUCANINI. Note: name also referred to as “*Chalcodes*”, an incorrect subsequent spelling of *Calcodes*, in the literature.

Type species: *Lucanusaeratus* Hope, 1833, by monotypy.

Family-group name: CHALCODINAE Didier & Séguy, 1953. Stem: *Calcod*-.

***Caledonica* Chaudoir, 1861**: 312. Current status: valid genus in CICINDELIDAE: CICINDELINI: DROMICINA.

Type species: *Distipsideramniszechii* J. Thomson, 1856, by subsequent designation (W. Horn 1915: 438).

Family-group name: CALEDONICINI W. Horn, 1893. Stem: *Caledonic*-.

***Calicnemis* Laporte, 1832a**: pl. 7. Current status: valid genus in SCARABAEIDAE: DYNASTINAE: PENTODONTINI: PENTODONTINA.

Type species: *Calicnemislatreillei* Laporte, 1832 [as *latreillii*], by monotypy.

Family-group name: CALICNÉMIENS Mulsant, 1842. Stem: *Calicnemid*-.

***Calitys* C.G. Thomson, 1859**: 71. Current status: valid genus in TROGOSSITIDAE: CALITYINAE.

Type species: *Silphadentata* Fabricius, 1787 (= *Hispascabra* Thunberg, 1784), by original designation.

Family-group name: CALITYNI Houlbert, 1922. Stem: *Cality*-.

***Calleida* Latreille in Latreille and Dejean, 1824**: 132. Current status: valid genus in CARABIDAE: HARPALINAE: LEBIINI: CALLEIDINA.

Type species: *Carabusdecorus* Fabricius, 1801, by subsequent designation (E. Desmarest 1851: 72).

Family-group name: CALLIDIDES Chaudoir, 1873. Stem: *Calleid*-.

***Callia* Audinet-Serville, 1835a**: 60. Current status: valid genus in CERAMBYCIDAE: LAMIINAE: CALLIINI.

Type species: *Calliaazurea* Audinet-Serville, 1835, by monotypy.

Family-group name: CALLITAE J. Thomson, 1864. Stem: *Calli*-.

***Callicerus* Gravenhorst, 1802**: 65. Current status: valid genus in STAPHYLINIDAE: ALEOCHARINAE: GEOSTIBINI. Note: name placed on the Official List of Generic Names in Zoology (ICZN 2012e).

Type species: *Callicerusobscurus* Gravenhorst, 1802, by monotypy (see ICZN 2012e). Note: name placed on the Official List of Specific Names in Zoology (see ICZN 2012e).

Family-group name: CALLICERINA Jakobson, 1908. Stem: *Callicer*-. Note: suppressed for both the Principle of Priority and Principle of Homonymy and placed on the Official Index of Rejected and Invalid Family-group Names in Zoology (ICZN 2012e).

***Callichroma* Latreille, 1816**: 341. Current status: valid genus in CERAMBYCIDAE: CERAMBYCINAE: CALLICHROMATINI.

Type species [suggested]: *Cerambyxsuturalis* Fabricius, 1781 (= *Cerambyxauricomus* Linnaeus, 1767), by subsequent designation (E. Desmarest 1860: 315). Note: the valid type species fixation is that of Audouin (1823: 52), who selected *Cerambyxalpinus* Linnaeus, 1758, but that species is the type species of the genus *Rosalia* Audinet- Serville, 1834; a subsequent designation based on an originally included nominal species is that of G.R. Waterhouse (1840: 228), who selected *Cerambyxmoschatus* Linnaeus, 1758, but that species is the type species of the genus *Aromia* Audinet- Serville, 1834; an application to the Commission is necessary to fix *Cerambyxsuturalis* Fabricius, 1781 as the type species of *Callichroma* Latreille, 1816 to conserve current usage (e.g., Monné and Nearns 2022: 16).

Family-group name: CALLICHROMINAE Swainson, 1840. Stem: *Callichromat*-.

***Callidiopis* A. White, 1855**: 328. Current status: valid genus in CERAMBYCIDAE: CERAMBYCINAE: CALLIDIOPINI.

Type species: *Callidiumscutellare* Fabricius, 1801, by subsequent designation (J. Thomson 1864: 237).

Family-group name: CALLIDIOPSIDES Lacordaire, 1868. Stem: *Callidiop*-.

***Callidium* Fabricius, 1775**: 187. Current status: valid genus in CERAMBYCIDAE: CERAMBYCINAE: CALLIDIINI.

Type species [suggested]: *Cerambyxviolaceus* Linnaeus, 1758, by subsequent designation (C.G. Thomson 1859: 150). Note: the valid type species fixation is that of Latreille (1810: 431), who selected *Cerambyxbajulus* Linnaeus, 1758, but that species is the type species of the genus *Hylotrupes* Audinet-Serville, 1834; a subsequent designation based on an originally included nominal species is that of Lefebvre (1838a: 46), who designated *Cerambyxvariabilis* Linnaeus, 1761, but that species is the type species of the genus *Phymatodes* Mulsant, 1839; yet another designation based on an originally included nominal species is that of Blanchard in Audouin et al. (1843: pl. 66), who selected *Lepturaarietis* Linnaeus, 1758, but that species is the type species of the genus *Clytus* Laicharting, 1784; an application to the Commission is necessary to fix *Cerambyxviolaceus* Linnaeus, 1758 as the type species of *Callidium* Fabricius, 1775.

Family-group name: CALLIDIADAE W. Kirby, 1837. Stem: *Callidi*-.

***Callimerus* Gorham, 1876**: 60, 64. Current status: valid genus in CLERIDAE: CLERINAE: CLERITAE: HYDNOCERINI: CALLIMERINA. Note: this name, published on 7 August 1876 (p. 57 footer of the journal), is a senior homonym of *Callimerus* Stebbing, 1876 [Crustacea], issued in volume 18 (fourth series) of the ‘Annals and Magazine of Natural History’ on 1 December 1876 (Evenhuis 2003a: 27).

Type species: *Clerusdulcis* Westwood, 1852, by original designation.

Family-group name: CALLIMERINI Kolibáč, 1998. Stem: *Callimer*-.

***Callipogon* Audinet-Serville, 1832**: 125, 140. Current status: valid genus in CERAMBYCIDAE: PRIONINAE: CALLIPOGONINI.

Type species: *Prionusbarbatus* Fabricius, 1781, by monotypy.

Family-group name: CALLIPOGONITAE J. Thomson, 1861. Stem: *Callipogon*-.

***Calliprason* A. White in A. White and Doubleday, 1843**: 277. Current status: valid genus in CERAMBYCIDAE: CERAMBYCINAE: RHAGIOMORPHINI.

Type species: *Callichromasinclairi* A. White, 1843, by monotypy.

Family-group name: CALLIPRASONINI McKeown, 1947. Stem: *Callipras*-.

***Callirhipis* Latreille, 1829a**: 459. Current status: valid genus in CALLIRHIPIDAE.

Type species: *Callirhipisdejeanii* Latreille, 1829, by original designation.

Family-group name: CALLIRHIPINI Emden, 1924. Stem: *Callirhip*-.

***Callirhopalus* Hochhuth, 1851**: 54. Current status: valid genus in CURCULIONIDAE: ENTIMINAE: POLYDRUSITAE: CNEORHININI.

Type species: *Callirhopalussedakowii* Hochhuth, 1851, by monotypy.

Family-group name: CALLIRHOPALINI Voss, 1956. Stem: *Callirhopal*-.

***Callisina* Baly, 1860**: 30. Current status: valid genus in CHRYSOMELIDAE: EUMOLPINAE: TYPOPHORINI.

Type species: *Callisinafasciata* Baly, 1860, by original designation.

Family-group name: CALLISINITES Chapuis, 1874. Stem: *Callisin*-.

***Callispa* Baly, 1858**: 4. Current status: valid genus in CHRYSOMELIDAE: CASSIDINAE: CALLISPINI.

Type species: *Callispafortunii* Baly, 1858, by original designation.

Family-group name: CALLISPITES Chapuis, 1875. Stem: *Callisp*-.

***Callisthenes* Fischer, 1820**: pl. 7. Current status: valid subgenus of *Calosoma* Weber, 1801 in CARABIDAE: CARABINAE: CARABINI: CALOSOMATINA.

Type species: *Callisthenespanderi* Fischer, 1820, by monotypy.

Family-group name: CALLISTHENISIDAE Gistel, 1848. Stem: *Callisthen*-.

***Callistoides* Motschulsky, 1865**: 334. Current status: valid genus in CARABIDAE: HARPALINAE: CHLAENIINI: CHLAENIINA.

Type species: *Callistoidesmalachinus* Motschulsky, 1865, by monotypy.

Family-group name: CALLISTOIDINI Basilewsky, 1950. Stem: *Callistoid*-.

***Callistus* Bonelli, 1810**: Tab. Syn. Current status: valid genus in CARABIDAE: HARPALINAE: CHLAENIINI: CALLISTINA.

Type species: *Carabuslunatus* Fabricius, 1775, by subsequent monotypy (Panzer 1813: 52).

Family-group name: CALLISTIDAE Laporte, 1834. Stem: *Callist*-.

***Callohispa* Uhmann, 1960**: 61. Current status: valid genus in CHRYSOMELIDAE: CASSIDINAE: CALLOHISPINI.

Type species: *Callohispamirifica* Uhmann, 1960, by original designation.

Family-group name: CALLOHISPINI Uhmann, 1960. Stem: *Callohisp*-.

***Callopistus* Deyrolle, 1865**: 9. Current status: invalid senior synonym of *Eucallopistus* Bellamy, 2003 in BUPRESTIDAE: CHRYSOCHROINAE: CHRYSOCHROINI: EUCALLOPISTINA. Note: junior homonym of *Callopistus* Schönherr, 1832 [Coleoptera: CURCULIONIDAE].

Type species: *Callopistuscastelnaudii* Deyrolle, 1865, by original designation.

Family-group name: CALLOPISTINA Kurosawa, 1990. Stem: *Callopist*-. Note: family-group name permanently invalid (ICZN 1999a, Article 39).

***Calocerus* Fauvel, 1891**: 88. Current status: junior synonym of *Glyptoma* Erichson, 1839 in STAPHYLINIDAE: OSORIINAE: THORACOPHORINI: GLYPTOMINA. Note: junior homonym of *Calocerus* J.L. LeConte, 1853 [Coleoptera: ELATERIDAE].

Type species: *Glyptomacrassicorne* Erichson, 1840, by subsequent designation (R. Lucas 1920: 159).

Family-group name: CALOCERI Blackwelder, 1942. Stem: *Calocer*-. Note: family-group name permanently invalid (ICZN 1999a, Article 39).

***Calochromus* Guérin-Méneville, 1833b**: 158. Current status: valid genus in LYCIDAE: CALOCHROMINAE.

Type species: *Calochromusglaucopterus* Guérin-Méneville, 1833, by monotypy.

Family-group name: CALOCHROMIDES Lacordaire, 1857. Stem: *Calochrom*-.

***Calocomus* Audinet-Serville, 1832**: 194. Current status: valid genus in CERAMBYCIDAE: PRIONINAE: CALOCOMINI.

Type species: *Calocomushamatiferus* Audinet-Serville, 1832 (= *Prionusdesmarestii* Guérin- Méneville, 1831), by monotypy.

Family-group name: CALOCOMINI Galileo & Martins, 1993. Stem: *Calocom*-.

***Calodera* Mannerheim, 1830**: 85. Current status: valid genus in STAPHYLINIDAE: ALEOCHARINAE: OXYPODINI: OXYPODINA.

Type species: *Caloderanigrita* Mannerheim, 1830, by subsequent designation (Westwood 1838c: 20).

Family-group name: CALODÉRATES Mulsant & Rey, 1874. Stem: *Caloder*-.

***Calodromus* Guérin-Méneville in Guérin-Méneville and Gory, 1832**: pl. 34. Current status: valid genus in BRENTIDAE: BRENTINAE: CYPHAGOGINI.

Type species: *Calodromusmellyi* Guérin-Méneville & Gory, 1832, by monotypy.

Family-group name: CALODROMINI Kleine, 1922. Stem: *Calodrom*-.

***Calognathus* Guérin-Méneville, 1837**: pl. 172. Current status: valid genus in TENEBRIONIDAE: PIMELIINAE: CRYPTOCHILINI: CALOGNATHINA.

Type species: *Calognathuschevrolatii* Guérin-Méneville, 1837, by monotypy.

Family-group name: CALOGNATHIDES Lacordaire, 1859. Stem: *Calognath*-.

***Calonecrus* J. Thomson, 1857**: 117. Current status: valid genus in NITIDULIDAE: CALONECRINAE.

Type species: *Calonecruswallacei* J. Thomson, 1857, by monotypy.

Family-group name: CALONECRINAE Kirejtshuk, 1982. Stem: *Calonecr*-.

***Calophaena* Klug, 1821**: 295. Current status: valid genus in CARABIDAE: HARPALINAE: CALOPHAENINI.

Type species: *Carabusacuminatus* G.-A. Olivier, 1790, by subsequent designation (Hope 1838a: 104).

Family-group name: CALOPHAENIDAE Jeannel, 1948. Stem: *Calophaen*-.

***Calopteron* Laporte, 1838**: 25. Current status: valid genus in LYCIDAE: LYCINAE: CALOPTERINI: CALOPTERINA.

Type species [suggested]: fixed by Bocáková (2003: 215) according to Article 70.3.2 (ICZN 1999a) as *Lycusapicalis* Guérin-Méneville, 1838, the taxonomic species involved in the misidentification of *Lycuslimbatus* Fabricius, 1801 (sensu Kleine, 1933), as designated subsequently by Bocák (1998: 249). Note: the valid type species fixation is that of Bescherelle (1845: 520), who selected *Cantharisbicolor* Linnaeus, 1763, but that species is currently placed in the genus *Thonalmus* Bourgeois, 1884; an application to the Commission is necessary to retain *Lycusapicalis* Guérin-Méneville, 1838 as the type species of *Calopteron* Laporte, 1838.

Family-group name: CALOPTERINI Green, 1949. Stem: *Calopter*-.

***Calopus* Fabricius, 1775**: 182. Current status: valid genus in OEDEMERIDAE: CALOPODINAE.

Type species: *Cerambyxserraticornis* Linnaeus, 1758, by subsequent designation (Latreille 1810: 430).

Family-group name: CALOPINI A. Costa, 1852. Stem: *Calopod*-. Note: nomen protectum (see Bouchard et al. 2011: 435).

***Calosoma* Weber, 1801**: 20. Current status: valid genus in CARABIDAE: CARABINAE: CARABINI: CALOSOMATINA. Note: this genus was described in 1801 by both Weber (p. 20) and Fabricius (p. 211); bibliographic research suggests that Weber’s book (issued by March 1801, see Evenhuis 1997b: 809) was published before Fabricius first volume of his Systema Eleutheratorum (issued by 26 April 1801, see Bousquet 2017a: 108; see also Chapin 1946: 79).

Type species: *Carabussycophanta* Linnaeus, 1758, by subsequent designation (Latreille 1810: 426).

Family-group name: CALOSOMII Bonelli, 1810. Stem: *Calosomat*-.

***Calospasta* J.L. LeConte, 1862**: 273. Current status: junior synonym of *Eupompha* J.L. LeConte, 1858 in MELOIDAE: MELOINAE: EUPOMPHINI.

Type species: *Epicautaelegans* J.L. LeConte, 1851, by monotypy.

Family-group name: CALOSPASTIDES Wellman, 1910. Stem: *Calospast*-.

***Calydus* Reitter, 1896**: 192. Current status: valid genus in MELOIDAE: MELOINAE: MYLABRINI. Note: new replacement name for *Caloenas* Reitter, 1889.

Type species: *Caloenaspulcher* Reitter, 1889, by monotypy (type fixation for *Caloenas* Reitter).

Family-group name: CALYDINA Kaszab, 1960. Stem: *Calyd*-.

***Calyptillus* G.H. Horn in J.L. LeConte and G.H. Horn, 1876**: 27. Current status: valid genus in CURCULIONIDAE: ENTIMINAE: POLYDRUSITAE: PSALLIDIINI.

Type species: *Calyptilluscryptops* G.H. Horn, 1876, by monotypy.

Family-group name: CALYPTILLI G.H. Horn, 1876. Stem: *Calyptill*-.

***Calyptocephalus* G.R. Gray in Griffith and Pidgeon, 1831**: 370, pls 39, 61. Current status: valid genus in LAMPYRIDAE: LAMPYRINAE: PLEOTOMINI.

Type species: *Calyptocephalusfasciatus* G.R. Gray, 1831, by monotypy.

Family-group name: CALYPTOCEPHALINA Jakobson, 1911. Stem: *Calyptocephal*-.

***Calyptocerus* Guérin-Méneville, 1843b**: 177. Current status: valid genus in EUCNEMIDAE: MELASINAE: CALYPTOCERINI.

Type species: *Calyptocerusleboucherii* Guérin-Méneville, 1843, by monotypy.

Family-group name: CALYPTOCERINI Muona, 1993. Stem: *Calyptocer*-.

***Calyptomerus* L. Redtenbacher, 1847a**: 159. Current status: valid genus in CLAMBIDAE: CALYPTOMERINAE.

Type species: *Calyptomerusalpestris* L. Redtenbacher, 1847, by monotypy.

Family-group name: CALYPTOMERIDAE Crowson, 1955. Stem: *Calyptomer*-.

***Camaragnathus* Bertrand-Bocandé, 1849**: 460. Current status: junior synonym of *Hiletus* Schiødte, 1847 in CARABIDAE: HILETINAE.

Type species: *Camaragnathusguerinii* Bertrand-Bocandé, 1849 (= *Hiletusversutus* Schiødte, 1847), by subsequent designation (E. Desmarest 1851: 158).

Family-group name: CAMARAGNATHINI Csiki, 1927. Stem: *Camaragnath*-.

***Camaria* Lepeletier & Audinet-Serville, 1828**: 454. Current status: valid genus in TENEBRIONIDAE: STENOCHIINAE: CNODALONINI.

Type species: *Camarianitida* Lepeletier & Audinet-Serville, 1828 (= *Tenebrionitens* G.-A. Olivier, 1795), by monotypy.

Family-group name: CAMARIINAE Anonymous, 1924. Stem: *Camari*-.

***Camarotus* Germar in Schönherr, 1833**: 185. Current status: valid genus in CURCULIONIDAE: CURCULIONINAE: CAMAROTINI: CAMAROTINA.

Type species: *Camarotuscoccinelloides* Germar, 1833, by original designation (p. 4).

Family-group name: CAMAROTIDES Schönherr, 1833. Stem: *Camarot*-.

***Cambaia* Lane, 1951**: 12. Current status: junior synonym of *Paraleptidea* Gounelle, 1913 in CERAMBYCIDAE: CERAMBYCINAE: PSEBIINI.

Type species: *Cambaialongitarsis* Lane, 1951, by original designation.

Family-group name: CAMBAIINAE Lane, 1951. Stem: *Cambai*-.

†***Camelomorpha* Kirejtshuk, Azar & Telnov in Kirejtshuk and Azar, 2008**: 41. Current status: valid genus in ANTHICIDAE: MACRATRIINAE: †CAMELOMORPHINI. Note: Lower Cretaceous (Lebanon).

Type species: †*Camelomorphalongicervix* Kirejtshuk, Azar & Telnov, 2008, by original designation.

Family-group name: †CAMELOMORPHINI Kirejtshuk, Azar & Telnov, 2008. Stem: *Camelomorph*-.

***Camenta* Erichson, 1847b**: 695. Current status: valid genus in SCARABAEIDAE: SERICINAE: ABLABERINI.

Type species: *Camentaobesa* Burmeister, 1855, by **present designation**. Note: no species were originally included in this genus by Erichson (1847b); the first ones were the five species described by Burmeister (1855: 144–146).

Family-group name: CAMENTINI Machatschke, 1959. Stem: *Cament*-.

***Camiarus* Sharp, 1878b**: 36. Current status: valid genus in LEIODIDAE: CAMIARINAE: CAMIARINI. Note: new replacement name for *Camirus* Sharp, 1876.

Type species [suggested]: *Camirusthoracicus* Sharp, 1876, by subsequent designation (Hatch 1928: 210). Note: the valid type species fixation is that of Bertkau (1879: 448), who selected *Camirusconvexus* Sharp, 1876, but that species is the type species of the genus *Camiarites* Jeannel, 1957; an application to the Commission is necessary to fix *Camirusthoracicus* Sharp, 1876 as the type species of *Camiarus* Sharp, 1878.

Family-group name: CAMIARINAE Jeannel, 1911. Stem: *Camiar*-.

***Campsosternus* Latreille, 1834**: 141. Current status: valid genus in ELATERIDAE: DENDROMETRINAE: OXYNOPTERINI.

Type species: *Elaterfulgens* G.-A. Olivier, 1790 (= *Elaterauratus* Drury, 1773), by monotypy.

Family-group name: CAMPSOSTERNINAE Fleutiaux, 1927. Stem: *Campsostern*-.

***Camptocerus* Dejean, 1821**: 100. Current status: valid genus in CURCULIONIDAE: SCOLYTINAE: SCOLYTINI.

Type species: *Hylesinusaeneipennis* Fabricius, 1801, by monotypy.

Family-group name: CAMPTOCÉRIDES Lacordaire, 1865. Stem: *Camptocer*-. Note: senior homonym of CAMPTOCERINI Wagner, 1961 (type genus *Camptocera* Jakovlev, 1877) in Hemiptera.

***Camptorhinus* Schönherr, 1825**: 585. Current status: valid genus in CURCULIONIDAE: CRYPTORHYNCHINAE: CAMPTORHININI.

Type species: *Curculiostatua* Fabricius, 1792 (= *Curculiostatua* Rossi, 1790), by original designation.

Family-group name: CAMPTORHINIDES Lacordaire, 1865. Stem: *Camptorhin*-.

***Campyloscelus* Schönherr, 1845**: 197. Current status: valid genus in CURCULIONIDAE: CONODERINAE: CAMPYLOSCELINI: CAMPYLOSCELINA.

Type species: *Campylosceluswestermanni* Boheman, 1845, by original designation.

Family-group name: CAMPYLOSCELIDES Schönherr, 1845. Stem: *Campyloscel*-.

***Campyloxenus* Fairmaire & Germain, 1860a**: 6. Current status: valid genus in ELATERIDAE: PITYOBIINAE: CAMPYLOXENINI.

Type species: *Campyloxenuspyrothorax* Fairmaire & Germain, 1860, by monotypy.

Family-group name: CAMPYLOXENINAE C. Costa, 1975. Stem: *Campyloxen*-.

***Campylus* Fischer, 1823a**: pl. 24. Current status: junior synonym of *Denticollis* Piller & Mitterpacher, 1783 in ELATERIDAE: DENDROMETRINAE: DENDROMETRINI: DENTICOLLINA.

Type species: *Elatermesomelus* Linnaeus, 1758 (= *Elaterlinearis* Linnaeus, 1758), by subsequent designation (Latreille 1830: 76). Note: Latreille (1830: 76) selected *Elaterlinearis* Linnaeus, 1758 as type species of *Campylus* Fischer, 1823, which is not an originally included species, but since he listed it in synonymy with *Elatermesomelus* Linnaeus, 1758, an originally included species, he is deemed to have designated the latter species as type species (ICZN 1999a, Article 69.2.2).

Family-group name: CAMPYLIDAE Gistel, 1848. Stem: *Campyl*-.

***Canephorotomus* Voss, 1954**: 212. Current status: junior synonym of *Amitrus* Schönherr, 1840 in CURCULIONIDAE: ENTIMINAE: POLYDRUSITAE: NAUPACTINI: NAUPACTINA.

Type species: *Canephorusjelskyi* Kirsch, 1890, by original designation.

Family-group name: CANEPHOROTOMINA Voss, 1954. Stem: *Canephorotom*-.

***Canonopsis* C.O. Waterhouse, 1875**: 54. Current status: valid genus in CURCULIONIDAE: ENTIMINAE: OTIORHYNCHITAE: ECTEMNORHININI.

Type species: *Canonopsissericea* C.O. Waterhouse, 1875, by monotypy.

Family-group name: CANONOPSINI Dreux & Voisin, 1989. Stem: *Canonopse*-.

***Cantharis* Linnaeus, 1758**: 342, 400. Current status: valid genus in CANTHARIDAE: CANTHARINAE: CANTHARINI.

Type species: *Cantharisfusca* Linnaeus, 1758, by subsequent designation (C.G. Thomson 1859: 110).

Family-group name: CANTHARIDAE Imhoff, 1856. Stem: *Canthar*-. Note: junior homonym of CANTHARINI Bonaparte, 1831 (type genus *Cantharus* Cuvier, 1816) in Pisces; Bonaparte’s name is permanently invalid (based on a preoccupied type genus).

***Cantharocnemis* Audinet-Serville, 1832**: 132. Current status: valid genus in CERAMBYCIDAE: PRIONINAE: CANTHAROCNEMINI.

Type species: *Cantharocnemisspondyloides* Audinet-Serville, 1832, by monotypy.

Family-group name: CANTHAROCNEMITAE J. Thomson, 1861. Stem: *Cantharocnem*-.

***Canthon* Hoffmannsegg, 1817**: 38. Current status: valid genus in SCARABAEIDAE: SCARABAEINAE: DELTOCHILINI.

Type species: *Scarabaeusvolvens* Fabricius, 1792 (= *Scarabaeuspilularius* Linnaeus, 1758), by subsequent designation (Duponchel 1842b: 131). Note: the type species has been cited in recent literature (e.g., Cupello and Vaz-de-Mello 2018) as *Scarabaeuspilularius* Linnaeus, 1758 by subsequent designation of Paulian (1939: 17); however, Duponchel’s fixation is older.

Family-group name: CANTHONIDES Van Lansberge, 1874. Stem: *Canthon*-.

***Capnisa* Dejean, 1836b**: 197. Current status: junior synonym of *Gnathosia* Fischer, 1821 in TENEBRIONIDAE: PIMELIINAE: TENTYRIINI.

Type species: *Bradyuskarelini* Faldermann, 1836, by monotypy. Note: see Bousquet and Bouchard (2013b: 231) regarding the date of publication of *Bradyuskarelini* Faldermann, 1836.

Family-group name: CAPNISINI Casey, 1907. Stem: *Capnis*-.

***Capnodis* Eschscholtz, 1829b**: 9. Current status: valid genus in BUPRESTIDAE: CHRYSOCHROINAE: DICERCINI: DICERCINA.

Type species: *Buprestistenebricosa* G.-A. Olivier, 1790, by subsequent designation (Duponchel 1842b: 136).

Family-group name: CAPNODINI Csiki, 1909. Stem: *Capnod*-.

***Cappadox* Alonso-Zarazaga & Lyal, 1999**: 29. Current status: junior synonym of *Phloeophilus* Schönherr, 1833 in ANTHRIBIDAE: ANTHRIBINAE: PHLOEOPHILINI.

Type species: *Phloeophilussulcifrons* Fåhraeus, 1839, by original designation.

Family-group name: CAPPADOCINI Alonso-Zarazaga & Lyal, 1999. Stem: *Cappadoc*-.

***Capreolucanus* Didier, 1929**: 65. Current status: valid genus in LUCANIDAE: LUCANINAE: CAPREOLUCANINI.

Type species: *Capreolucanussicardi* Didier, 1929, by original designation.

Family-group name: CAPREOLUCANINI H. Huang & C.H. Chen, 2013: 69. Stem: *Capreolucan*-.

***Capula* Jakobson, 1925**: 145. Current status: valid genus in CHRYSOMELIDAE: GALERUCINAE: HYLASPINI.

Type species: *Capulametallica* Jakobson, 1925, by original designation.

Family-group name: CAPULINI Ogloblin, 1936. Stem: *Capul*-. Note: junior homonym of CAPULIDAE Fleming, 1822 (type genus *Capulus* Montfort, 1810) in Mollusca.

***Car* Blackburn, 1897**: 35. Current status: valid genus in CARIDAE: CARINAE.

Type species: *Carcondensatus* Blackburn, 1897, by monotypy.

Family-group name: CARINAE Thompson, 1992. Stem: *Car*-.

***Carabdytes* Balke, Hendrich & Wewalka, 1992**: 94. Current status: valid genus in DYTISCIDAE: COLYMBETINAE.

Type species: *Carabdytesupin* Balke, Hendrich & Wewalka, 1992, by original designation.

Family-group name: CARABDYTINI Pederzani, 1995. Stem: *Carabdyt*-.

***Carabhydrus* Watts, 1978**: 26. Current status: valid genus in DYTISCIDAE: HYDROPORINAE: HYDROPORINI: STERNOPRISCINA.

Type species: *Carabhydrusmonteithi* Watts, 1978, by original designation.

Family-group name: CARABHYDRINI Watts, 1978. Stem: *Carabhydr*-.

***Carabidomemnus* Kolbe, 1924a**: 346. Current status: valid genus in CARABIDAE: PAUSSINAE: PAUSSINI: CARABIDOMEMNINA. Note: this name was also proposed later in the same year by Kolbe (1924b: 1).

Type species: *Arthropteruspallidus* Raffray, 1886, by subsequent designation (Wasmann 1928: 271).

Family-group name: CARABIDOMEMNINAE Wasmann, 1928. Stem: *Carabidomemn*-.

***Carabus* Linnaeus, 1758**: 342, 413. Current status: valid genus in CARABIDAE: CARABINAE: CARABINI: CARABINA. Note: name placed on the Official List of Generic Names in Zoology (ICZN 1954a).

Type species: *Carabusgranulatus* Linnaeus, 1758, by plenary power (see ICZN 1954a). Note: name placed on the Official List of Specific Names in Zoology (ICZN 1954a).

Family-group name: CARABICI Latreille, 1802. Stem: *Carab*-.

***Caraphia* Gahan, 1906**: 75. Current status: valid genus in CERAMBYCIDAE: LEPTURINAE: CARAPHIINI.

Type species: *Caraphiacribrata* Gahan, 1906, by original designation.

Family-group name: CARAPHIINI Ohbayashi, Lin & Yamasako, 2016: 189. Stem: *Caraphi*-.

***Carcilia* Roelofs, 1875**: 152. Current status: valid genus in CURCULIONIDAE: MESOPTILIINAE: CARCILIINI.

Type species: *Carciliastrigicollis* Roelofs, 1875, by monotypy.

Family-group name: CARCILIINAE Pierce, 1916. Stem: *Carcili*-.

***Cardiophorus* Eschscholtz, 1829a**: 34. Current status: valid genus in ELATERIDAE: CARDIOPHORINAE.

Type species: *Elaterthoracicus* Fabricius, 1775 (= *Elatergramineus* Scopoli, 1763), by subsequent designation (Curtis 1838a: pl. 694).

Family-group name: CARDIOPHORITES Candèze, 1859. Stem: *Cardiophor*-.

***Cardiorhinus* Eschscholtz, 1829a**: 34. Current status: valid genus in ELATERIDAE: ELATERINAE: AGRIOTINI: CARDIORHININA.

Type species: *Elaterplagiatus* Germar, 1823, by subsequent designation (Duponchel 1842b: 168).

Family-group name: CARDIORHINITES Candèze, 1863. Stem: *Cardiorhin*-.

***Carenum* Bonelli, 1813**: 479. Current status: valid genus in CARABIDAE: SCARITINAE: CARENINI.

Type species: **here fixed** (under ICZN 1999a, Article 70.3.2) as *Carenumbonellii* Westwood, 1842, misidentified as *Scaritescyaneus* Fabricius, 1775 (sensu Bonelli, 1813), in the original designation by Bonelli (1813: 479). Note: see Westwood (1842b: 83) regarding the misidentification of the type species.

Family-group name: CARENIDES W.J. MacLeay, 1887. Stem: *Caren*-.

**Carilia Mulsant, 1863**: 489. Current status: valid genus in CERAMBYCIDAE: LEPTURINAE: ACMAEOPINI.

Type species: *Lepturavirginea* Linnaeus, 1758, by monotypy.

Family-group name: CARILIINI Zamoroka, 2022: 308, **syn. nov.** Stem: *Carili*-. Note: this family-group name is unnecessary since the name ACMAEOPINI Della Beffa, 1915 is already available for this taxon.

***Carinodula* Gordon, Pakaluk & Ślipiński, 1989**: 360. Current status: valid genus in COCCINELLIDAE: MICROWEISEINAE: CARINODULINI.

Type species: *Carinodulacampbelli* Gordon, Pakaluk & Ślipiński, 1989, by original designation.

Family-group name: CARINODULINI Gordon, Pakaluk & Ślipiński, 1989. Stem: *Carinodul*-.

***Carodes* Zimmerman, 1994**: 511. Current status: valid genus in CARIDAE: CHILECARINAE: CARODESINI.

Type species: *Carodesrevelatus* Zimmerman, 1994, by original designation.

Family-group name: CARODESINA Legalov, 2009. Stem: *Carodes*- (ICZN 1999a, Article 29.4).

***Carpelimus* Leach in Samouelle, 1819**: 174. Current status: valid genus in STAPHYLINIDAE: OXYTELINAE: THINOBIINI.

Type species: *Oxytelusfuliginosus* Gravenhorst, 1802, by subsequent monotypy (Stephens 1829b: 24, as *Carpalimus*).

Family-group name: CARPELIMINI Hatch, 1957. Stem: *Carpelim*-.

***Carphoborus* Eichhoff, 1864**: 27, 44. Current status: valid genus in CURCULIONIDAE: SCOLYTINAE: POLYGRAPHINI.

Type species: *Hylesinusminimus* Fabricius, 1801, by subsequent designation (Lacordaire 1865: 362).

Family-group name: CARPHOBORINAE Nüsslin, 1911. Stem: *Carphobor*-.

***Carphodicticus* Wood, 1971**: 19. Current status: valid genus in CURCULIONIDAE: SCOLYTINAE: CARPHODICTICINI.

Type species: *Carphodicticuscristatus* Wood, 1971, by original designation.

Family-group name: CARPHODICTICINI Wood, 1971. Stem: *Carphodictic*-.

***Carphurus* Erichson, 1840a**: 132. Current status: valid genus in MELYRIDAE: MALACHIINAE: CARPHURINI.

Type species: *Carphurusdispar* Erichson, 1840, by subsequent designation (Champion 1923b: 9).

Family-group name: CARPHURINAE Champion, 1923. Stem: *Carphur*-.

***Carpophagus* W.S. MacLeay, 1826**: 447. Current status: valid genus in CHRYSOMELIDAE: SAGRINAE: CARPOPHAGINI.

Type species: *Carpophagusbanksiae* W.S. MacLeay, 1826, by monotypy.

Family-group name: CARPOPHAGITES Chapuis, 1874. Stem: *Carpophag*-. Note: junior homonym of CARPOPHAGINAE Selby, 1835 (type genus *Carpophaga* Selby, 1835) in Aves.

***Carpophilus* Stephens, 1829b**: 7. Current status: valid genus in NITIDULIDAE: CARPOPHILINAE.

Type species: *Nitidulaflexuosa* Herbst, 1790 (= *Dermesteshemipterus* Linnaeus, 1758), by monotypy.

Family-group name: CARPOPHILINAE Erichson, 1842. Stem: *Carpophil*-.

***Caryedon* Schönherr, 1823**: 1134. Current status: valid genus in CHRYSOMELIDAE: BRUCHINAE: PACHYMERINI: CARYEDONTINA.

Type species: *Bruchusserratus* G.-A. Olivier, 1790, by original designation.

Family-group name: CARYEDINI Bridwell, 1929. Stem: *Caryedont*-.

***Caryonoda* Bechyné, 1951**: 265. Current status: valid genus in CHRYSOMELIDAE: EUMOLPINAE: CARYONODINI.

Type species: *Caryonodakuscheli* Bechyné, 1951, by original designation.

Family-group name: CARYONODINI Bechyné, 1951. Stem: *Caryonod*-.

***Caryopemon* Jekel, 1855**: 25. Current status: valid genus in CHRYSOMELIDAE: BRUCHINAE: PACHYMERINI: CARYOPEMINA. Note: two original spellings were used, including *Carpopemon* (p. 26); as far as we know no one has acted as First Reviser (ICZN 1999a, Article 24.2.3) and we **here select***Caryopemon*, the spelling in prevailing usage.

Type species: *Caryopemonhieroglyphicus* Jekel, 1855, by original designation.

Family-group name: CARYOPEMINI Bridwell, 1929. Stem: *Caryopem*-.

***Casignetus* W.S. MacLeay, 1819**: 98. Current status: valid genus in LUCANIDAE: LUCANINAE: CASIGNETINI.

Type species: *Casignetusgeotrupoides* W.S. MacLeay, 1819 (= *Lamprimahumboldti* Gyllenhal, 1817), by monotypy.

Family-group name: CASIGNETINI Reid, 1999. Stem: *Casignet*-. Note: junior homonym of CASIGNETINI Falkovitsh, 1972 (type genus *Casigneta* Wallengren, 1881) in Lepidoptera; Falkovitsh’s name is permanently invalid (i.e., its type genus is preoccupied by *Casigneta* Brunner von Wattenwyl, 1878, in Orthoptera).

***Cassida* Linnaeus, 1758**: 342, 362. Current status: valid genus in CHRYSOMELIDAE: CASSIDINAE: CASSIDINI. Note: name placed on the Official List of Generic Names in Zoology (ICZN 1974b).

Type species: *Cassidanebulosa* Linnaeus, 1758, by subsequent designation (Spaeth 1914: 140; see ICZN 1974b). Note: name placed on the Official List of Specific Names in Zoology (ICZN 1974b).

Family-group name: CASSIDEAE Gyllenhal, 1813. Stem: *Cassid*-.

***Catachirotes* Gistel, 1856**: 384. Current status: junior synonym of *Calopus* Fabricius, 1775 in OEDEMERIDAE: CALOPODINAE.

Type species: *Cerambyxserraticornis* Linnaeus, 1758, by monotypy.

Family-group name: CATACHIROTIDAE Gistel, 1856. Stem: *Catachirotet*-.

***Catadromus* W.S. MacLeay, 1825**: 18. Current status: valid genus in CARABIDAE: HARPALINAE: PTEROSTICHINI: PTEROSTICHINA.

Type species: *Carabustenebrioides* G.-A. Olivier, 1790, by monotypy.

Family-group name: CATADROMIENS Brullé, 1834. Stem: *Catadrom*-.

***Cataphronetis* P.H. Lucas, 1846**: pl. 30. Current status: junior synonym of *Phtora* Germar, 1836 in TENEBRIONIDAE: DIAPERINAE: PHALERIINI.

Type species: *Cataphronetislevaillantii* P.H. Lucas, 1846 (= *Phtoracrenata* Germar, 1836), by monotypy.

Family-group name: CATAPHRONETINI Reitter, 1917. Stem: *Cataphronet*-.

***Catapiesis* Solier in Brullé, 1835**: 42. Current status: valid genus in CARABIDAE: HARPALINAE: CATAPIESEINI.

Type species: *Catapiesisnitida* Solier, 1835, by subsequent designation (Hope 1838a: 108).

Family-group name: CATAPIESINAE H.W. Bates, 1882. Stem: *Catapiese*-.

***Catapiestus* Perty, 1831**: xxxviii. Current status: valid genus in TENEBRIONIDAE: STENOCHIINAE: CNODALONINI.

Type species: *Catapiestuspiceus* Perty, 1831, by monotypy.

Family-group name: CATAPIESTIDES Lacordaire, 1859. Stem: *Catapiest*-.

***Catapion* Schilsky, 1906**: vi. Current status: valid genus in BRENTIDAE: APIONINAE: APIONITAE: CATAPIINI.

Type species: *Apionseniculus* W. Kirby, 1808, by original designation.

Family-group name: CATAPIINA Alonso-Zarazaga, 1990. Stem: *Catapi*-.

***Catascopus* W. Kirby, 1823a**: 94. Current status: valid genus in CARABIDAE: HARPALINAE: LEBIINI: PERICALINA.

Type species: *Catascopushardwickii* W. Kirby, 1823, by monotypy.

Family-group name: CATASCOPI Csiki, 1932. Stem: *Catascop*-.

***Cathormiocerus* Schönherr, 1842b**: 120. Current status: valid genus in CURCULIONIDAE: ENTIMINAE: CYPHICERITAE: TRACHYPHLOEINI: TRACHYPHLOEINA.

Type species: *Trachyphloeushorrens* Gyllenhal, 1834, by original designation.

Family-group name: CATHORMIOCERINI Reitter, 1913. Stem: *Cathormiocer*-.

†***Catinius* Ponomarenko, 1968**: 137. Current status: valid genus in †CATINIIDAE. Note: Jurassic and Early Cretaceous (Kazakhstan; China).

Type species: †*Catiniuspelta* Ponomarenko, 1968, by original designation.

Family-group name: †CATINIIDAE Ponomarenko, 1968. Stem: *Catini*-. Note: junior homonym of CATINIIDAE Bocquet & Stock, 1957 (type genus *Catinia* Bocquet & Stock, 1957) in Crustacea.

***Catogenus* Westwood, 1830b**: 221. Current status: valid genus in PASSANDRIDAE: PASSANDRINAE.

Type species: *Cucujusrufus* Fabricius, 1798, by original designation.

Family-group name: CATOGENINI Grouvelle, 1916. Stem: *Catogen*-.

***Catopocerus* Motschulsky, 1870**: 351. Current status: valid genus in LEIODIDAE: CATOPOCERINAE: CATOPOCERINI.

Type species: *Catopoceruspolitus* Motschulsky, 1870, by monotypy.

Family-group name: CATOPOCERINI Hatch, 1927. Stem: *Catopocer*-.

***Catopochrotus* Reitter, 1889d**: 290. Current status: valid genus in CRYPTOPHAGIDAE: CRYPTOPHAGINAE: CRYPTOPHAGINI.

Type species: *Catopochrotuscrematogastri* Reitter, 1889, by monotypy.

Family-group name: CATOPOCHROTIDAE Reitter, 1889. Stem: *Catopochrot*-.

***Catops* Paykull, 1798**: 342. Current status: valid genus in LEIODIDAE: CHOLEVINAE: CHOLEVINI: CATOPINA.

Type species: *Helopsfuscus* Panzer, 1794, misidentified as *Tritomasericea* Fabricius, 1787 (sensu Paykull, 1798) in the subsequent designation by Jeannel (1922: 44). Note: we follow Peck et al. (2020: 36) in using Article 69.2.4 (ICZN 1999a) to determine the identity of the type species for this genus.

Family-group name: CATOPIDES Chaudoir, 1845. Stem: *Catop*-.

***Catoxantha* Solier, 1833**: 261, 266. Current status: invalid senior synonym of *Xanthocata* Kubáň, 2016 in BUPRESTIDAE: CHRYSOCHROINAE: CHRYSOCHROINI: CHRYSOCHROINA. Note: junior homonym of *Catoxantha* Dejean, 1833 [Coleoptera: BUPRESTIDAE] (see Bousquet and Bouchard 2013a: 16, 17).

Type species: *Buprestisopulenta* Gory, 1832, by monotypy.

Family-group name: CATOXANTHINA Jakobson, 1913. Stem: *Catoxanth*-. Note: family-group name permanently invalid (ICZN 1999a, Article 39).

***Catypnes* Pascoe, 1864a**: 243. Current status: valid genus in CERAMBYCIDAE: PRIONINAE: CATYPNINI.

Type species: *Catypnesmacleayi* Pascoe, 1864, by monotypy.

Family-group name CATYPNIDES Lacordaire, 1868. Stem: *Catypn*-. Note: this name was treated as unavailable by Bouchard et al. (2011: 454) but was accepted as valid and attributed to Lacordaire, 1868 by Jin et al. (2023: 510) and is therefore available from that date.

***Caulobius* Le Guillou, 1844**: 224. Current status: invalid senior synonym of *Deuterocaulobius* Dalla Torre, 1912 in SCARABAEIDAE: SERICOIDINAE: AUTOMOLIINI. Note: junior homonym of *Caulobius* Duponchel, 1838 [Lepidoptera].

Type species: *Caulobiusvillosus* Le Guillou, 1844, by monotypy.

Family-group name: CAULOBIINA Burmeister, 1855. Stem: *Caulobi*-. Note: family-group name permanently invalid (ICZN 1999a, Article 39).

***Cautires* C.O. Waterhouse, 1880a**: 36. Current status: valid genus in LYCIDAE: METRIORRHYNCHINAE: METRIORRHYNCHINI: CAUTIRINA.

Type species: *Lycusexcellens* C.O. Waterhouse, 1878, by subsequent designation (Kleine 1936: 4).

Family-group name: CAUTIRINA Sklenarova, Kubecek & Bocák, 2014: 52. Stem: *Cautir*-.

***Cavicoxum* Pic, 1928a**: 21. Current status: junior synonym of *Agraeus* Candèze, 1857 in ELATERIDAE: AGRYPNINAE: AGRYPNINI.

Type species: *Cavicoxummonstrosum* Pic, 1928, by monotypy.

Family-group name: CAVICOXUMIDAE Pic, 1928. Stem: *Cavicox*-.

***Cavognatha* Crowson, 1965**: 241. Current status: junior synonym of *Taphropiestes* Reitter, 1875 in CAVOGNATHIDAE.

Type species: *Cavognathapullivora* Crowson, 1965, by original designation.

Family-group name: CAVOGNATHINAE Sen Gupta & Crowson, 1966. Stem: *Cavognath*-.

***Ceballosmelasis* Cobos, 1965**: 381. Current status: valid genus in EUCNEMIDAE: MELASINAE: CEBALLOSMELASINI.

Type species: *Ceballosmelasiselongatissimus* Cobos, 1965, by monotypy.

Family-group name: CEBALLOSMELASINI Muona, 1993. Stem: *Ceballosmelas*-.

***Cebrio* G.-A. Olivier, 1790a**: [30bis] 1, 4. Current status: valid genus in ELATERIDAE: ELATERINAE: CEBRIONINI: CEBRIONINA.

Type species: *Cistelagigas* Fabricius, 1787, by subsequent designation (Latreille 1810: 426).

Family-group name: CEBRIONATES Latreille, 1802. Stem: *Cebrion*-.

***Cebriognathus* Chobaut, 1899**: 22. Current status: junior synonym of *Analestesa* Leach, 1824 in ELATERIDAE: CARDIOPHORINAE.

Type species: *Cebriognathusdesertorum* Chobaut, 1899 (= *Analestesatestacea* Leach, 1824), by monotypy.

Family-group name: CEBRIOGNATHINAE Paulus, 1981. Stem: *Cebriognath*-.

***Cechenus* Fischer, 1822**: 110. Current status: invalid senior synonym of *Cechenochilus* Motschulsky, 1850 in CARABIDAE: CARABINAE: CARABINI: CARABINA. Note: junior homonym of *Cechenus* Illiger, 1807 [Hymenoptera].

Type species: *Carabusboeberi* Adams, 1817, by subsequent designation (Froriep 1825: 348).

Family-group name: CECHENOGENICI Csiki, 1927. Stem: *Cechen*-. Note: family-group name permanently invalid (ICZN 1999a, Article 39).

***Celaenephes* Schmidt-Göbel, 1846**: 77. Current status: valid genus in CARABIDAE: HARPALINAE: LEBIINI: CELAENEPHINA.

Type species: *Celaenephesparallelus* Schmidt-Göbel, 1846, by monotypy.

Family-group name: CELAENEPHINA Habu, 1982. Stem: *Celaeneph*-.

***Celetes* Schönherr, 1836**: 634. Current status: valid genus in CURCULIONIDAE: CURCULIONINAE: DERELOMINI: DERELOMINA.

Type species: *Celetesbinotatus* Gyllenhal, 1836, by original designation.

Family-group name: CELETINI Voss, 1954. Stem: *Celet*-.

***Celeuthetes* Schönherr, 1842b**: 250. Current status: valid genus in CURCULIONIDAE: ENTIMINAE: OTIORHYNCHITAE: CELEUTHETINI: CELEUTHETINA. Note: unnecessary new replacement name for *Sphaeromus* Schönherr, 1834.

Type species: *Sphaeromusaustralis* Gyllenhal, 1834 (= *Curculioechinatus* Fabricius, 1801), by original designation (type fixation for *Sphaeromus* Schönherr).

Family-group name: CÉLEUTHÉTIDES Lacordaire, 1863. Stem: *Celeuthet*-.

***Celina* Aubé, 1837**: 218, 219. Current status: valid genus in DYTISCIDAE: HYDROPORINAE: METHLINI.

Type species: *Hydroporuslatipes* Brullé, 1836, by monotypy.

Family-group name: CELININI Branden, 1884. Stem: *Celin*-.

***Celioschesis* Tschitschérine, 1898**: 93. Current status: junior synonym of *Aristopus* LaFerté-Sénectère, 1853 in CARABIDAE: HARPALINAE: ABACETINI: ABACETINA.

Type species: *Distrigusbipustulatus* Brullé, 1834, by subsequent designation (Jeannel 1948: 442).

Family-group name: CELIOSCHESINI Jeannel, 1948. Stem: *Celioschese*-.

***Cenocephalus* Chapuis, 1865**: 325. Current status: valid genus in CURCULIONIDAE: PLATYPODINAE: TESSEROCERINI: TESSEROCERINA.

Type species: *Cenocephalusthoracicus* Chapuis, 1865, by monotypy.

Family-group name: CENOCEPHALARIAE H. Strohmeyer, 1914. Stem: *Cenocephal*-.

***Centrinus* Schönherr, 1825**: 586. Current status: valid genus in CURCULIONIDAE: BARIDINAE: APOSTASIMERINI: DIORYMERINA.

Type species: *Barisbicuspis* Germar, 1823, by subsequent designation (Schönherr 1826: 22).

Family-group name: CENTRINIDES Jekel, 1865. Stem: *Centrin*-. Note: junior homonym of CENTRINIDAE Swainson, 1838 (type genus *Centrina* Cuvier, 1816) in Pisces.

***Centrioptera* Mannerheim, 1843**: 279. Current status: junior synonym of *Cryptoglossa* Solier, 1837 in TENEBRIONIDAE: PIMELIINAE: CRYPTOGLOSSINI.

Type species: *Centriopteracaraboides* Mannerheim, 1843, by monotypy.

Family-group name: CENTRIOPTÉRIDES Lacordaire, 1859. Stem: *Centriopter*-.

***Centrocorynus* Jekel, 1860**: 167. Current status: valid genus in ATTELABIDAE: APODERINAE: APODERINI.

Type species: *Apoderusscutellaris* Gyllenhal, 1833, by original designation.

Family-group name: CENTROCORYNINA Legalov, 2003. Stem: *Centrocoryn*-.

***Centronopus* Solier, 1848b**: 154, 258. Current status: valid genus in TENEBRIONIDAE: TENEBRIONINAE: CENTRONOPINI.

Type species: *Centronopusextensicollis* Solier, 1848 (= *Tenebriosuppressus* Say, 1835), by original designation.

Family-group name: CENTRONOPINI Doyen, 1989. Stem: *Centronop*-.

***Centrophthalmus* Schmidt-Göbel, 1838**: 7. Current status: valid genus in STAPHYLINIDAE: PSELAPHINAE: PSELAPHITAE: TYRINI: CENTROPHTHALMINA.

Type species: *Centrophthalmusparia* Schmidt-Göbel, 1838, by monotypy.

Family-group name: CENTROPHTHALMINA Jeannel, 1949. Stem: *Centrophthalm*-.

***Ceophyllus* J.L. LeConte, 1849**: 73. Current status: valid genus in STAPHYLINIDAE: PSELAPHINAE: PSELAPHITAE: TYRINI: TYRINA.

Type species: *Ceophyllusmonilis* J.L. LeConte, 1849, by monotypy.

Family-group name: CEOPHYLLINI Park, 1951. Stem: *Ceophyll*-.

***Cephacerus* Rafinesque, 1815**: 113. Current status: junior synonym of *Erodius* Fabricius, 1775 in TENEBRIONIDAE: PIMELIINAE: ERODIINI. Note: unnecessary new replacement name for *Erodius* Fabricius, 1775.

Type species: *Erodiusgibbus* Fabricius, 1775, by subsequent designation (Latreille 1810: 429) (type fixation for *Erodius* Fabricius).

Family-group name: CEPHACERIA Rafinesque, 1815. Stem: *Cephacer*-.

***Cephalobyrrhus* Pic, 1923**: 4. Current status: valid genus in LIMNICHIDAE: CEPHALOBYRRHINAE.

Type species: *Cephalobyrrhuslatus* Pic, 1923, by subsequent designation (Satô 1965: 123).

Family-group name: CEPHALOBYRRHINAE Champion, 1925. Stem: *Cephalobyrrh*-.

***Cephalodonta* Chevrolat, 1842b**: 272. Current status: junior synonym of *Sceloenopla* Chevrolat, 1836 in CHRYSOMELIDAE: CASSIDINAE: SCELOENOPLINI.

Type species: *Chalepusgoniapterus* Perty, 1832, by monotypy.

Family-group name: CEPHALODONTINAE Gestro, 1906. Stem: *Cephalodont*-.

***Cephaloleia* Chevrolat in Dejean, 1836a**: 366. Current status: valid genus in CHRYSOMELIDAE: CASSIDINAE: CEPHALOLEIINI.

Type species: *Hispanigricornis* Fabricius, 1792, by subsequent designation (Staines 1991: 247).

Family-group name: CÉPHALOLÉITES Chapuis, 1875. Stem: *Cephalolei*-.

***Cephaloon* Newman, 1838**: 376. Current status: valid genus in STENOTRACHELIDAE: CEPHALOINAE.

Type species: *Cephaloonlepturides* Newman, 1838, by monotypy.

Family-group name: CEPHALOIDAE J.L. LeConte, 1862. Stem: *Cephalo*-.

***Cephalophanus* John, 1940**: 82. Current status: valid genus in DISCOLOMATIDAE: CEPHALOPHANINAE.

Type species: *Cephalophanusclipeoexcisus* John, 1940, by original designation.

Family-group name: CEPHALOPHANINAE John, 1954. Stem: *Cephalophan*-.

***Cephaloplectus* Sharp, 1883a**: 295. Current status: valid genus in PTILIIDAE: PTILIINAE: CEPHALOPLECTINI.

Type species: *Cephaloplectusgodmani* Sharp, 1883, by monotypy.

Family-group name: CEPHALOPLECTINAE Sharp, 1883. Stem: *Cephaloplect*-.

***Cephaloscymnus* Crotch, 1873c**: 382. Current status: valid genus in COCCINELLIDAE: COCCINELLINAE: CEPHALOSCYMNINI.

Type species: *Cephaloscymnuszimmermanni* Crotch, 1873, by monotypy.

Family-group name: CEPHALOSCYMNINI Gordon, 1985. Stem: *Cephaloscymn*-.

***Cephalotes* Bonelli, 1810**: Tab Syn. Current status: invalid senior synonym of *Broscus* Panzer, 1813 in CARABIDAE: BROSCINAE: BROSCINI: BROSCINA. Note: junior homonym of *Cephalotes* Latreille, 1802 [Hymenoptera].

Type species: *Carabuscephalotes* Linnaeus, 1758, by subsequent monotypy in Panzer (1813: 62) (type fixation for *Broscus* Panzer).

Family-group name: CEPHALOTIDA Heer, 1838. Stem: *Cephalot*-. Note: family-group name permanently invalid (ICZN 1999a, Article 39).

***Cephalotoma* Lesne, 1911**: 207. Current status: valid genus in BOSTRICHIDAE: LYCTINAE: CEPHALOTOMINI.

Type species: *Cephalotomasingularis* Lesne, 1911, by monotypy.

Family-group name: CEPHALOTOMINI L.Y. Liu in L.Y. Liu and Schönitzer, 2011: 99, 120. Stem: *Cephalotom*-.

***Cephalotrichia* Hope, 1837**: 102. Current status: invalid senior synonym of *Sparrmannia* Laporte, 1840 (nomen protectum) in SCARABAEIDAE: MELOLONTHINAE: TANYPROCTINI: TANYPROCTINA. Note: nomen oblitum (see Bouchard et al. 2011: 255).

Type species: *Melolonthaalopex* Fabricius, 1787, by original designation.

Family-group name: CEPHALOTRICHIADAE Burmeister, 1855. Stem: *Cephalotrichi*-.

***Cephalotyphlus* Coiffait, 1955**: 68. Current status: valid genus in STAPHYLINIDAE: LEPTOTYPHLINAE: CEPHALOTYPHLINI.

Type species: *Leptotyphlusrevelierei* Saulcy, 1878, by original designation.

Family-group name: CEPHALOTYPHLINI Coiffait, 1963. Stem: *Cephalotyphl*-.

***Cephennium* P.W.J. Müller in P.W.J. Müller and Kunze, 1822**: 188. Current status: valid genus in STAPHYLINIDAE: SCYDMAENINAE: CEPHENNIITAE: CEPHENNIINI. Note: this name was first proposed by P.W.J. Müller in P.W.J. Müller and Kunze (1822: 188) in synonymy with *Scydmaenusthoracicus* P.W.J. Müller & Kunze, 1822; it is available from that date since it was treated as an available name before 1961 by several authors (e.g., Casey 1897: 500; Ganglbauer 1899: 11).

Type species: *Scydmaenusthoracicus* P.W.J. Müller & Kunze, 1822, by subsequent designation (E. Desmarest 1852: 148). Note: the type species usually listed for this genus is “*Cephenniumtruncorum* P.W.J. Müller, 1822 (= *Scydmaenusthoracicus* P.W.J. Müller & Kunze, 1822)”; however, *Cephenniumtruncorum* was first proposed by P.W.J. Müller in P.W.J. Müller and Kunze (1822: 188) in synonymy with *Scydmaenusthoracicus* P.W.J. Müller & Kunze, 1822 but, as far as we know, it is unavailable since it was not treated as an available name before 1961 and either adopted as the name of a taxon or treated as a senior homonym (ICZN 1999a, Article 11.6.1); the first available species associated with the name *Cephennium* are the three species, *Scydmaenusthoracicus* P.W.J. Müller & Kunze, 1822, *Scydmaenuslaticollis* Aubé, 1843, and *Scydmaenusminutissimus* Aubé, 1843, listed by Erichson (1845b: 99).

Family-group name: CEPHENNIINI Reitter, 1882. Stem: *Cephenni*-.

***Cepurus* Schönherr, 1834b**: 347. Current status: valid genus in CURCULIONIDAE: HYPERINAE: HYPERINI: CEPURINA.

Type species: *Curculiotorridus* G.-A. Olivier, 1807, by original designation.

Family-group name: CÉPURIDES Capiomont, 1867. Stem: *Cepur*-.

***Ceracupes* Kaup, 1871**: 16. Current status: valid genus in PASSALIDAE: AULACOCYCLINAE: CERACUPEDINI.

Type species: *Passalusfronticornis* Westwood, 1841, by monotypy.

Family-group name: CERACUPINI Boucher, 2006. Stem: *Ceracup*- (ICZN 1999a, Article 29.4).

†***Ceracyclus* Boucher & Bai in Boucher et al., 2017**: 7. Current status: valid genus in PASSALIDAE: AULACOCYCLINAE: CYLINDROCAULINI. Note: mid-Cretaceous (Myanmar).

Type species: †*Ceracycluslotus* Boucher, Bai & Montreuil, 2017, by original designation.

Family-group name: †CERACYCLINI Boucher & Bai in Boucher et al., 2017: 4. Stem: *Ceracycl*-.

***Cerallus* Jacquelin du Val, 1859a**: 43. Current status: valid genus in MELYRIDAE: MELYRINAE: CERALLINI.

Type species: *Dasytesrubidus* Gyllenhal, 1817, by original designation.

Family-group name: CERALLINI Pic, 1929. Stem: *Cerall*-.

***Cerambyciscapha* Pic, 1915a**: 30. Current status: valid genus in STAPHYLINIDAE: SCAPHIDIINAE: SCAPHIDIINI.

Type species: *Cerambyciscaphadohertyi* Pic, 1915, by monotypy.

Family-group name: CERAMBYCISCAPHINI Pic, 1915. Stem: *Cerambyciscaph*-.

***Cerambyx* Linnaeus, 1758**: 342, 388. Current status: valid genus in CERAMBYCIDAE: CERAMBYCINAE: CERAMBYCINI: CERAMBYCINA.

Type species: *Cerambyxcerdo* Linnaeus, 1758, by subsequent designation (Latreille 1810: 431).

Family-group name: CERAMBICINI Latreille, 1802. Stem: *Cerambyc*-.

***Cerapterus* Swederus, 1788**: 203. Current status: valid genus in CARABIDAE: PAUSSINAE: PAUSSINI: CERAPTERINA.

Type species: *Cerapteruslatipes* Swederus, 1788, by monotypy.

Family-group name: CERAPTERIDES Billberg, 1820. Stem: *Cerapter*-.

***Cerasommatidia* Brèthes, 1925**: 199. Current status: valid genus in CERASOMMATIDIIDAE.

Type species: *Cerasommatidiaarrowi* Brèthes, 1925, by monotypy.

Family-group name: CERASOMMATIDIIDAE Brèthes, 1925. Stem: *Cerasommatidi*-.

***Cerasphorus* Audinet-Serville, 1834b**: 10. Current status: valid genus in CERAMBYCIDAE: CERAMBYCINAE: HESPEROPHANINI: HESPEROPHANINA.

Type species [suggested]: *Cerasphorushirticornis* Audinet-Serville, 1834, by subsequent designation (J. Thomson 1861: 236). Note: the valid type species fixation is that of E. Desmarest (1860: 312), who selected *Stenocorusgarganicus* Fabricius, 1775, but that species is currently included as a junior synonym of *Cerambyxcinctus* Drury, 1773 in the genus *Chion* Newman, 1840 (see Bousquet 2008a: 619); an application to the Commission is necessary to fix *Cerasphorushirticornis* Audinet-Serville, 1834 as the type species of *Cerasphorus* Audinet-Serville, 1834.

Family-group name: CERASPHORITAE J. Thomson, 1861. Stem: *Cerasphor*-.

***Ceraspis* Lepeletier & Audinet-Serville, 1828**: 370. Current status: valid genus in SCARABAEIDAE: MELOLONTHINAE: MACRODACTYLINI.

Type species: *Ceraspispruinosa* Lepeletier & Audinet-Serville, 1828 (= *Melolonthabivulnerata* Germar, 1823), by subsequent designation (Duponchel 1842b: 287).

Family-group name: CERASPIDIDAE Burmeister, 1855. Stem: *Ceraspid*-.

***Ceratanisus* Gemminger in Gemminger and Harold, 1870**: 1818. Current status: valid genus in TENEBRIONIDAE: PIMELIINAE: CERATANISINI. Note: new replacement name for *Anisocerus* Faldermann, 1837.

Type species: *Anisocerustristis* Faldermann, 1837, by monotypy (type fixation for *Anisocerus* Faldermann).

Family-group name: CERATANISINI Gebien, 1937. Stem: *Ceratanis*-.

***Ceratapion* Schilsky, 1901**: 38F. Current status: valid genus in BRENTIDAE: APIONINAE: ASPIDAPIITAE: CERATAPIINI.

Type species: *Apioncarduorum* W. Kirby, 1808, by original designation.

Family-group name: CERATAPIINI Alonso-Zarazaga, 1990. Stem: *Ceratapi*-.

***Ceratocanthus* A. White, 1842**: 93. Current status: valid genus in HYBOSORIDAE: CERATOCANTHINAE: CERATOCANTHINI. Note: new replacement name for *Acanthocerus* W.S. MacLeay, 1819; the valid name for this genus should be *Sphaeromorphus* Dejean, 1833 (see Bousquet and Bouchard 2013a: 41); an application has been submitted to the Commission by A. Ballerio (Case 3766) to retain *Ceratocanthus* A. White, 1842 as valid.

Type species: *Acanthocerusaeneus* W.S. MacLeay, 1819, by subsequent designation (Duponchel 1839: 30) (type fixation for *Acanthocerus* W.S. MacLeay).

Family-group name: CERATOCANTHINI Martínez, 1968. Stem: *Ceratocanth*-.

***Ceratoderus* Westwood, 1841b**: 112. Current status: valid genus in CARABIDAE: PAUSSINAE: PAUSSINI: PAUSSINA.

Type species: *Paussusbifasciatus* Kollar, 1836, by monotypy.

Family-group name: CERATODERINA Darlington, 1950. Stem: *Ceratoder*-.

***Ceratognathus* Westwood, 1838a**: 260. Current status: valid genus in LUCANIDAE: AESALINAE: CERATOGNATHINI.

Type species: *Ceratognathusniger* Westwood, 1838, by original designation.

Family-group name: CERATOGNATHINI Sharp, 1899. Stem: *Ceratognath*-.

***Ceratopus* Schönherr, 1843**: 120. Current status: valid genus in CURCULIONIDAE: CURCULIONINAE: CERATOPODINI.

Type species: *Ceratopusbisignatus* Boheman, 1843, by original designation.

Family-group name: CÉRATOPIDES Lacordaire, 1863. Stem: *Ceratopod*-.

***Ceratorhina* Westwood, 1843a**: 170. Current status: junior synonym of *Eudicella* A. White, 1839 in SCARABAEIDAE: CETONIINAE: GOLIATHINI: RHOMBORHININA.

Type species: *Goliathusgrallii* Buquet, 1936, by subsequent designation (Marais and Holm 1992: 14).

Family-group name: CERATORRHINIDAE Kraatz, 1880. Stem: *Ceratorhin*-.

***Ceratotrupes* Jekel, 1866**: 540. Current status: valid genus in GEOTRUPIDAE: GEOTRUPINAE: CERATOTRUPINI.

Type species: *Geotrupesfronticornis* Erichson, 1847, by original designation.

Family-group name: CERATOTRUPINI Zunino, 1984. Stem: *Ceratotrup*-.

***Cercus* Latreille, 1797**: 68. Current status: junior synonym of *Kateretes* Herbst, 1793 in KATERETIDAE: KATERETINAE.

Type species: *Dermestespedicularius* Linnaeus, 1758, by subsequent monotypy (Latreille 1802b: 135).

Family-group name: CERCIDAE E. Desmarest, 1851. Stem: *Cerc*-.

***Cercyon* Leach, 1817**: 95. Current status: valid genus in HYDROPHILIDAE: SPHAERIDIINAE: MEGASTERNINI: MEGASTERNINA.

Type species: *Dermestesmelanocephalus* Linnaeus, 1758, by subsequent designation (C.G. Thomson 1859: 19).

Family-group name: CERCYONES G.H. Horn, 1890. Stem: *Cercyon*-.

***Cerenopus* J.L. LeConte, 1851a**: 143. Current status: valid genus in TENEBRIONIDAE: TENEBRIONINAE: CERENOPINI.

Type species: *Cerenopusconcolor* J.L. LeConte, 1851, by subsequent designation (R. Lucas 1920: 173).

Family-group name: CERENOPI G.H. Horn, 1870. Stem: *Cerenop*-.

***Cerocoma* Geoffroy, 1762**: 357. Current status: valid genus in MELOIDAE: MELOINAE: CEROCOMINI. Note: name placed on the Official List of Generic Names in Zoology (ICZN 1994a).

Type species: *Meloeschaefferi* Linnaeus, 1758, by subsequent monotypy (Fabricius 1775: 262; see ICZN 1994a). Note: name placed on the Official List of Specific Names in Zoology (ICZN 1994a).

Family-group name: CEROCOMATIDA Leach, 1815. Stem: *Cerocom*-.

***Cerocosmus* Gemminger, 1873**: 146. Current status: valid genus in PTINIDAE: ERNOBIINAE: ERNOBIINI. Note: new replacement name for *Cosmocerus* Solier, 1849.

Type species: *Cosmoceruscinereus* Solier, 1849, by monotypy (type fixation for *Cosmocerus* Solier).

Family-group name: CEROCOSMINAE Pic, 1912. Stem: *Cerocosm*-.

***Ceroglossus* Solier, 1848a**: 58. Current status: valid genus in CARABIDAE: CARABINAE: CEROGLOSSINI.

Type species: *Carabuschilensis* Eschscholtz, 1829, by original designation.

Family-group name: CEROGLOSSINA Lapouge, 1927. Stem: *Cerogloss*-.

***Cerophysa* Chevrolat in Dejean, 1836a**: 379. Current status: valid genus in CHRYSOMELIDAE: GALERUCINAE: LUPERINI.

Type species: *Gallerucanodicornis* Wiedemann, 1823, by monotypy.

Family-group name: CEROPHYSITES Chapuis, 1875. Stem: *Cerophys*-.

***Cerophytum* Latreille, 1809a**: 375. Current status: valid genus in CEROPHYTIDAE.

Type species: *Melasiselateroides* Latreille, 1804, by monotypy.

Family-group name: CEROPHYTIDES Latreille, 1834. Stem: *Cerophyt*-.

***Ceroplesis* Audinet-Serville, 1835a**: 93. Current status: valid genus in CERAMBYCIDAE: LAMIINAE: CEROPLESINI: CEROPLESINA. Note: name placed on the Official List of Generic Names in Zoology (ICZN 1986c).

Type species: *Lamiaaethiops* Fabricius, 1775, by plenary power (see ICZN 1986c). Note: name placed on the Official List of Specific Names in Zoology (ICZN 1986c).

Family-group name: CEROPLESITAE J. Thomson, 1860. Stem: *Ceroples*-.

***Cerotoma* Chevrolat in Dejean, 1836a**: 379. Current status: valid genus in CHRYSOMELIDAE: GALERUCINAE: LUPERINI.

Type species: *Crioceriscaminea* Fabricius, 1801 (= *Criocerisruficornis* G.-A. Olivier, 1791), by subsequent designation (Chapuis 1875: 230).

Family-group name: CÉROTOMITES Chapuis, 1875. Stem: *Cerotom*-.

***Certallum* Dejean, 1821**: 111. Current status: valid genus in CERAMBYCIDAE: CERAMBYCINAE: CERTALLINI.

Type species: *Saperdaruficollis* Fabricius, 1787 (= *Cerambyxebulinus* Linnaeus, 1767), by monotypy.

Family-group name: CARTALLITES Fairmaire, 1864. Stem: *Certall*-.

†***Ceruchites* Statz, 1952**: 5. Current status: valid genus in LUCANIDAE: †CERUCHITINAE. Note: Oligocene (Germany).

Type species: †*Ceruchiteshahnei* Statz, 1952, by monotypy.

Family-group name: †CERUCHITINAE Nikolajev, 2006. Stem: *Ceruchit*-.

***Ceruchus* W.S. MacLeay, 1819**: 96, 115. Current status: valid genus in LUCANIDAE: SYNDESINAE: CERUCHINI.

Type species: *Lucanustenebroides* Fabricius, 1787 (= *Lucanuschrysomelinus* Hochenwarth, 1785), by monotypy.

Family-group name: CERUCHITES Jacquelin du Val, 1859. Stem: *Ceruch*-.

***Cerylon* Latreille, 1802b**: 205. Current status: valid genus in CERYLONIDAE: CERYLONINAE. Note: name placed on the Official List of Generic Names in Zoology (ICZN 1995d).

Type species: *Lyctushisteroides* Fabricius, 1792, by plenary power (see ICZN 1995d). Note: name placed on the Official List of Specific Names in Zoology (ICZN 1995d).

Family-group name: CERYLONIDES Billberg, 1820. Stem: *Cerylon*-.

***Cesauletes* Hamilton, 1983**: 68. Current status: valid genus in ATTELABIDAE: RHYNCHITINAE: RHYNCHITITAE: CESAULETINI.

Type species: *Auletesnasalis* J.L. LeConte, 1876, by original designation.

Family-group name: CESAULETINI Legalov, 2003. Stem: *Cesaulet*-.

***Cetonia* Fabricius, 1775**: 42. Current status: valid genus in SCARABAEIDAE: CETONIINAE: CETONIINI: CETONIINA.

Type species: *Scarabaeusauratus* Linnaeus, 1758, by subsequent designation (Latreille 1810: 429).

Family-group name: CETONIDA Leach, 1815. Stem: *Cetoni*-.

***Ceutocerus* Germar, 1823**: 85. Current status: valid genus in MURMIDIIDAE.

Type species: *Ceutocerusadvena* Germar, 1823 (= *Histerovalis* Beck, 1817), by subsequent designation (Hope 1841a: 108).

Family-group name: CEUTHOCERA Mannerheim, 1852. Stem: *Ceutocer*-. Note: nomen oblitum (see Bouchard and Bousquet 2020a: 128).

***Ceutorhynchus* Germar, 1823**: 217. Current status: valid genus in CURCULIONIDAE: CEUTORHYNCHINAE: CEUTORHYNCHINI. Note: name placed on the Official List of Generic Names in Zoology (ICZN 1989b, as *Ceutorhynchus* Germar, 1824).

Type species: *Curculioassimilis* Paykull, 1792, by subsequent designation (C.G. Thomson 1859: 140; see ICZN 1989b). Note: the senior homonym *Curculioassimilis* Fabricius, 1775 and all other uses of that name prior to *Curculioassimilis* Paykull, 1792 were suppressed for both the Principles of Priority and Homonymy and *Curculioassimilis* Paykull, 1792 was placed on the Official List of Specific Names in Zoology (ICZN 1989b).

Family-group name: CENTORHYNCHIDAE Gistel, 1848. Stem: *Ceutorhynch*-.

***Chaeridiona* Baly, 1869**: 380. Current status: valid genus in CHRYSOMELIDAE: CASSIDINAE: ONCOCEPHALINI.

Type species: *Chaeridionametallica* Baly, 1869, by subsequent designation (Maulik 1919: 85).

Family-group name: CHOERIDIONINI Weise, 1911. Stem: *Chaeridion*-.

***Chaerodes* A. White, 1846**: 12. Current status: valid genus in TENEBRIONIDAE: LAGRIINAE: CHAERODINI.

Type species: *Chaerodestrachyscelides* A. White, 1846, by monotypy.

Family-group name: CHAERODINI Doyen, E.G. Matthews & Lawrence, 1990. Stem: *Chaerod*-.

***Chaetarthria* Stephens, 1833a**: 22. Current status: valid genus in HYDROPHILIDAE: CHAETARTHRIINAE: CHAETARTHRIINI.

Type species: *Hydrophilusseminulum* Paykull, 1798 (= *Hydrophilusseminulum* Herbst, 1797), by monotypy.

Family-group name: CHAETARTHRIINI Bedel, 1881. Stem: *Chaetarthri*-.

***Chaetocanthus* Péringuey, 1901**: 492, 495. Current status: valid genus in OCHODAEIDAE: CHAETOCANTHINAE: CHAETOCANTHINI.

Type species: *Chaetocanthusinsuetus* Péringuey, 1901, by monotypy.

Family-group name: CHAETOCANTHINAE Scholtz, 1988. Stem: *Chaetocanth*-.

***Chaetocnema* Stephens, 1831b**: 325. Current status: valid genus in CHRYSOMELIDAE: GALERUCINAE: ALTICINI.

Type species [suggested]: *Galerucaaridella* Paykull, 1799 (= *Alticahortensis* Geoffroy, 1785), by subsequent designation (C.G. Thomson 1859: 156). Note: the valid type species fixation is that of Westwood (1838c: 42), who selected *Chrysomelaconcinna* Marsham, 1802, but that species is currently included in the subgenus Tlanoma Motschulsky, 1845, not in the nominotypical subgenus; an application to the Commission is necessary to fix *Galerucaaridella* Paykull, 1799 as the type species of *Chaetocnema* Stephens, 1831.

Family-group name: CHAETOCNEMAE J.L. LeConte & G.H. Horn, 1883. Stem: *Chaetocnem*-.

***Chaetodactyla* Tschitschérine, 1897**: 271. Current status: valid genus in CARABIDAE: HARPALINAE: CHAETODACTYLINI.

Type species: *Chaetodactylamirabilis* Tschitschérine, 1897, by monotypy.

Family-group name: CHAETODACTYLINI Tschitschérine, 1903. Stem: *Chaetodactyl*-. Note: senior homonym of CHAETODACTYLIDAE Zachvatkin, 1941 (type genus *Chaetodactylus* Rondani, 1866) in Acari: Astigmata.

***Chaetogenys* Emden, 1958**: 19. Current status: valid genus in CARABIDAE: HARPALINAE: CHAETOGENYINI.

Type species: *Chaetogenysstraneoi* Emden, 1958, by original designation.

Family-group name: CHAETOGENYINA Emden, 1958. Stem: *Chaetogeny*-.

***Chaetomalachius* Kraatz, 1882b**: 96. Current status: valid genus in MELYRIDAE: DASYTINAE: CHAETOMALACHIINI.

Type species: *Chaetomalachiusdasytoides* Kraatz, 1882, by monotypy.

Family-group name: CHAETOMALACHIINI Majer, 1987. Stem: *Chaetomalachi*-.

***Chaetophloeus* J.L. LeConte in J.L. LeConte and G.H. Horn, 1876**: 382. Current status: valid genus in CURCULIONIDAE: SCOLYTINAE: CHAETOPHLOEINI.

Type species: *Hylesinushystrix* J.L. LeConte, 1858, by monotypy.

Family-group name: CHAETOPHLOEINI Jordal, 2021: 108. Stem: *Chaetophloe*-. Note: this family-group name was attributed to Schedl (1966: 361) in Bouchard et al. (2011: 635) but we agree with Alonso-Zarazaga and Lyal (2009: 74) and Jordal (2021: 108) that the family-group name is not available from Schedl (1966: 361) since a description was not provided.

***Chaetosoma* Westwood, 1851**: 171. Current status: valid genus in CHAETOSOMATIDAE. Note: the senior homonym *Chaetosoma* Chevrolat, 1843 [Coleoptera: CERAMBYCIDAE] was suppressed for the purposes of both the Principles of Priority and Homonymy and placed on the Official Index of Rejected and Invalid Generic Names in Zoology while *Chaetosoma* Westwood, 1851 was placed on the Official List of Generic Names in Zoology (ICZN 2011d).

Type species: *Chaetosomascaritides* Westwood, 1851, by monotypy (see ICZN 2011d). Note: name placed on the Official List of Specific Names in Zoology (ICZN 2011d).

Family-group name: CHAETOSOMATIDAE Crowson, 1952. Stem: *Chaetosomat*-. Note: the senior homonym CHAETOSOMATIDAE Claus, 1872 (type genus *Chaetosoma* Claparède, 1863) in Nematoda was suppressed for the purposes of both the Principles of Priority and Homonymy and placed on the Official Index of Rejected and Invalid Family-Group Names in Zoology (ICZN 2011d).

***Chalcodrya* L. Redtenbacher, 1868**: 138. Current status: valid genus in CHALCODRYIDAE.

Type species: *Chalcodryavariegata* L. Redtenbacher, 1868, by monotypy.

Family-group name: CHALCODRYIDAE Watt, 1974. Stem: *Chalcodry*-.

***Chalcolepidius* Eschscholtz, 1829a**: 32. Current status: valid genus in ELATERIDAE: AGRYPNINAE: HEMIRHIPINI: HEMIRHIPINA.

Type species: *Elatersulcatus* Fabricius, 1777, by subsequent designation (Duponchel 1843a: 371). Note: the type species often reported in the literature is *Chalcolepidiuszonatus* Eschscholtz, 1829 by subsequent designation of Hyslop (1921: 634) but Duponchel’s typification is earlier (see Kundrata et al. 2019c: 103); both species are currently included in the genus *Chalcolepidius*.

Family-group name: CHALCOLÉPIDIIDES Candèze, 1857. Stem: *Chalcolepidi*-.

***Chalcophana* Chevrolat in Dejean, 1836a**: 407. Current status: valid genus in CHRYSOMELIDAE: EUMOLPINAE: EUMOLPINI.

Type species: *Colaspishilaris* Germar, 1823, by subsequent designation (Monrós and Bechyné 1956: 1125).

Family-group name: CHALCOPHANITES Chapuis, 1874. Stem: *Chalcophan*-. Note: junior homonym of CHALCOPHANINAE Sundevall, 1872 (type genus *Chalcophanes* Wagler, 1827) in Aves.

***Chalcophora* Dejean, 1833a**: 77. Current status: valid genus in BUPRESTIDAE: CHRYSOCHROINAE: CHRYSOCHROINI: CHALCOPHORINA.

Type species: *Buprestismariana* Linnaeus, 1758, by subsequent designation (Duponchel 1843a: 372).

Family-group name: CHALCOPHORIDES Lacordaire, 1857. Stem: *Chalcophor*-.

***Chalcophorella* Kerremans, 1903a**: 79. Current status: valid genus in BUPRESTIDAE: CHRYSOCHROINAE: CHRYSOCHROINI: CHALCOPHORINA.

Type species: *Buprestisstigmatica* Dalman, 1817, by subsequent designation (Obenberger 1942: 4).

Family-group name: CHALCOPHORELLINI Tôyama, 1986. Stem: *Chalcophorell*-.

***Chalcoplectus* Oke, 1925**: 13. Current status: valid genus in STAPHYLINIDAE: PSELAPHINAE: PSELAPHITAE: TYRINI: TYRINA.

Type species: *Chalcoplectusdepressus* Oke, 1925, by monotypy.

Family-group name: CHALCOPLECTINI Oke, 1925. Stem: *Chalcoplect*-.

***Chalcosoma* Hope, 1837**: 86. Current status: valid genus in SCARABAEIDAE: DYNASTINAE: DYNASTINI: CHALCOSOMINA.

Type species: *Scarabaeusatlas* Linnaeus, 1758, by original designation.

Family-group name: CHALCOSOMINA Rowland & K.B. Miller, 2012: 11. Stem: *Chalcosom*- (ICZN 1999a, Article 29.4).

***Chalcothea* Burmeister, 1842**: 319. Current status: valid genus in SCARABAEIDAE: CETONIINAE: TAENIODERINI: CHALCOTHEINA.

Type species: *Macronotaresplendens* Gory & Percheron, 1834, by subsequent designation (Mikšić 1976: 379)

Family-group name: CHALCOTHEINA Mikšić, 1976. Stem: *Chalcothe*-.

***Chalepus* W.S. MacLeay, 1819**: 149. Current status: invalid senior synonym of *Dyscinetus* Harold, 1869 in SCARABAEIDAE: DYNASTINAE: CYCLOCEPHALINI. Note: junior homonym of *Chalepus* Thunberg, 1805 [Coleoptera: CHRYSOMELIDAE].

Type species: *Melolonthageminata* Fabricius, 1801 (= *Melolonthadubia* G.-A. Olivier, 1789), by original designation.

Family-group name: CHALEPIDAE Streubel, 1846. Stem: *Chalep*-. Note: senior homonym of CHALEPINI Weise, 1910 (type genus *Chalepus* Thunberg, 1805) in Coleoptera: CHRYSOMELIDAE; family-group name permanently invalid (ICZN 1999a, Article 39).

***Chalepus* Thunberg, 1805**: 282. Current status: valid genus in CHRYSOMELIDAE: CASSIDINAE: CHALEPINI.

Type species: *Hispasanguinicollis* Linnaeus, 1771, by subsequent designation (Crotch 1870b: 218).

Family-group name: CHALEPINI Weise, 1910. Stem: *Chalep*-. Note: nomen protectum (see Bouchard and Bousquet 2020a: 108); junior homonym of CHALEPIDAE Streubel, 1846 (type genus *Chalepus* W.S. MacLeay, 1819) in Coleoptera: SCARABAEIDAE; Streubel’s name is permanently invalid (based on a preoccupied type genus).

***Chaltenia* Roig-Juñent & Cicchino, 2001**: 663. Current status: valid genus in CARABIDAE: TRECHINAE: SINOZOLINI: CHALTENIINA.

Type species: *Chalteniapatagonia* Roig-Juñent & Cicchino, 2001, by original designation.

Family-group name: CHALTENIINA Roig-Juñent & Cicchino, 2001. Stem: *Chalteni*-.

***Chanopterus* Boheman, 1858**: 98. Current status: valid genus in PROMECHEILIDAE.

Type species: *Chanopterusparadoxus* Boheman, 1858, by monotypy.

Family-group name: CHANOPTERINAE Borchmann, 1915. Stem: *Chanopter*-.

***Chapuisia* Dugès, 1886**: 56. Current status: invalid senior synonym of *Schedlarius* Wood, 1957 in CURCULIONIDAE: PLATYPODINAE: SCHEDLARIINI. Note: junior homonym of *Chapuisia* Duvivier, 1885 [Coleoptera: CHRYSOMELIDAE].

Type species: *Chapuisiamexicana* Dugès, 1886, by monotypy.

Family-group name: CHAPUISIIDES W.F.H. Blandford, 1895. Stem: *Chapuisi*-. Note: family- group name permanently invalid (ICZN 1999a, Article 39).

***Charidotis* Boheman, 1855**: 7. Current status: valid genus in CHRYSOMELIDAE: CASSIDINAE: CASSIDINI.

Type species: *Charidotisminiata* Boheman, 1855, by subsequent designation (Hincks 1952: 343).

Family-group name: CHARIDOTITAE Spaeth, 1942. Stem: *Charidotid*-.

†***Charonoscapha* Ponomarenko, 1977**: 32. Current status: valid genus in †COPTOCLAVIDAE: †CHARONOSCAPHINAE. Note: Upper Jurassic (Kazakhstan).

Type species: †*Charonoscaphagrossa* Ponomarenko, 1977, by original designation.

Family-group name: †CHARONOSCAPHINAE Ponomarenko, 1977. Stem: *Charonoscaph*-.

***Chasmatopterus* Dejean, 1821**: 60. Current status: valid genus in SCARABAEIDAE: MELOLONTHINAE: CHASMATOPTERINI.

Type species: *Melolonthavillosula* Illiger, 1803, by subsequent designation (Blanchard in Audouin et al. 1843: pl. 44). Note: the type species usually listed for this genus is *Melolonthahirtula* Illiger, 1803 (e.g., Branco 2007: 9; Branco and A. Bezdĕk 2016: 211) by subsequent designation of Baraud (1965: 264) but Blanchard’s type fixation is earlier; both nominal species are currently placed in the genus *Chasmatopterus*.

Family-group name: CHASMATOPTÉRIDES Lacordaire, 1855. Stem: *Chasmatopter*-.

***Chasme* Lepeletier & Audinet-Serville, 1828**: 378. Current status: valid genus in SCARABAEIDAE: MELOLONTHINAE: HOPLIINI: HOPLIINA.

Type species: *Chasmedecora* Lepeletier & Audinet-Serville, 1828 (= *Melolonthadecora* Wiedemann, 1823), by monotypy.

Family-group name: CHASMIDAE Streubel, 1846. Stem: *Chasm*-.

***Chasmodia* W.S. MacLeay, 1819**: 155. Current status: junior synonym of *Lagochile* Hoffmannsegg, 1817 in SCARABAEIDAE: RUTELINAE: RUTELINI: RUTELINA.

Type species: *Chasmodiaviridis* W.S. MacLeay, 1819 (= *Rutelaemarginata* Gyllenhal, 1817), by subsequent designation (Duponchel 1843a: 405).

Family-group name: CHASMODIIDAE Burmeister, 1844. Stem: *Chasmodi*-.

***Chauliognathus* Hentz, 1830**: 460. Current status: valid genus in CANTHARIDAE: CHAULIOGNATHINAE: CHAULIOGNATHINI.

Type species: *Cantharisbimaculata* Fabricius, 1781 (= *Telephoruspensylvanicus* DeGeer, 1774), by subsequent designation (Calder 1998: 186). Note: Calder (1998: 186) selected *Telephoruspensylvanicus* DeGeer, 1774 as type species, which is not an originally included species, but since he listed it in synonymy with *Cantharisbimaculata* Fabricius, 1881, an originally included species, he is deemed to have designated the latter species as type species (ICZN 1999a, Article 69.2.2); Fanti (2019: 29) remarked that *Telephoruspensylvanicus* DeGeer, 1774 is a junior synonym of *Cantharisamericana* Forster, 1771, as noted already by some early authors, such as J.L. LeConte (1851b: 338); an application to the Commission is necessary to conserve usage of the well-known name *Chauliognathuspensylvanicus* (DeGeer, 1774).

Family-group name: CHAULIOGNATHINI J.L. LeConte, 1861. Stem: *Chauliognath*-.

***Cheguevaria* Kazantsev, 2007**: 370. Current status: valid genus in LAMPYRIDAE: CHEGUEVARIINAE.

Type species: *Cheguevariataino* Kazantsev, 2007, by original designation.

Family-group name: CHEGUEVARIINI Kazantsev, 2007. Stem: *Cheguevari*-.

***Cheilomenes* Chevrolat in Dejean, 1836a**: 435. Current status: valid genus in COCCINELLIDAE: COCCINELLINAE: COCCINELLINI.

Type species: *Coccinellalunata* Fabricius, 1775, by subsequent designation (Crotch 1874a: 179).

Family-group name: CHEILOMENINI F.A. Schilder & M. Schilder, 1928. Stem: *Cheilomen*-.

***Cheiridea* Baly, 1878a**: 253. Current status: valid genus in CHRYSOMELIDAE: EUMOLPINAE: TYPOPHORINI.

Type species: *Cheirideachapuisi* Baly, 1878, by original designation.

Family-group name: CHEIRIDEITAE Lefèvre, 1885. Stem: *Cheiride*-.

***Cheiroplatys* Hope, 1837**: 84. Current status: valid genus in SCARABAEIDAE: DYNASTINAE: PENTODONTINI: CHEIROPLATINA.

Type species: **here fixed** (under ICZN 1999a, Article 70.3.2) as *Scarabaeuslatipes* Guérin- Méneville, 1838, misidentified as *Scarabaeustruncatus* Fabricius, 1775, in the original designation by Hope (1837: 84). Note: see Carne (1957b: 83) regarding the misidentification of the type species.

Family-group name: CHEIROPLATINA Carne, 1957. Stem: *Cheiroplat*-.

***Chelonarium* Fabricius, 1801a**: xviii, 101. Current status: valid genus in CHELONARIIDAE.

Type species: *Chelonariumatrum* Fabricius, 1801, by subsequent designation (T. Bell 1830: 509).

Family-group name: CHELONARIITES Blanchard, 1845. Stem: *Chelonari*-.

***Chennium* Latreille, 1807**: 77. Current status: valid genus in STAPHYLINIDAE: PSELAPHINAE: PSELAPHITAE: CTENISTINI.

Type species: *Chenniumbituberculatum* Latreille, 1807, by monotypy.

Family-group name: CHENNIIDES Reitter, 1882. Stem: *Chenni*-.

***Cherrocrius* Berg, 1898**: 31. Current status: valid genus in VESPERIDAE: ANOPLODERMATINAE: ANOPLODERMATINI.

Type species: *Cherrocriusbruchi* Berg, 1898, by original designation.

Family-group name: CHERROCRIINAE Prosen, 1960. Stem: *Cherrocri*-.

***Chespirito* V.S. Ferreira, Keller & Branham, 2020**: 4. Current status: valid genus in LAMPYRIDAE: CHESPIRITOINAE.

Type species: *Chespiritozaragozai* V.S. Ferreira, Keller & Branham, 2020, by original designation.

Family-group name: CHESPIRITOINAE V.S. Ferreira, Keller & Branham, 2020: 4. Stem: *Chespirito*-.

***Chevrolatia* Jacquelin du Val, 1850**: 45. Current status: valid genus in STAPHYLINIDAE: SCYDMAENINAE: SCYDMAENITAE: CHEVROLATIINI.

Type species: *Chevrolatiainsignis* Jacquelin du Val, 1850, by monotypy.

Family-group name: CHEVROLATINI Reitter, 1882. Stem: *Chevrolati*-.

***Chiasognathus* Stephens, 1831a**: 5. Current status: valid genus in LUCANIDAE: LUCANINAE: CHIASOGNATHINI.

Type species: *Chiasognathusgrantii* Stephens, 1831, by original designation.

Family-group name: CHIASOGNATHIDAE Burmeister, 1847. Stem: *Chiasognath*-.

***Chilapion* Kissinger, 1968**: 19. Current status: valid genus in BRENTIDAE: APIONINAE: CHILAPIITAE.

Type species: *Apionmacilentum* Blanchard, 1851, by original designation.

Family-group name: CHILAPIITAE Wanat, 2001. Stem: *Chilapi*-.

***Chilecar* Kuschel, 1992**: 205. Current status: valid genus in CARIDAE: CHILECARINAE: CHILECARINI.

Type species: *Chilecarpilgerodendri* Kuschel, 1992, by original designation.

Family-group name: CHILECARINI Legalov, 2009. Stem: *Chilecar*-.

***Chilenius* Lesne, 1921**: 287. Current status: valid genus in BOSTRICHIDAE: PSOINAE: CHILENIINI.

Type species: *Exopioidesspinicollis* Fairmaire & Germain, 1861, by original designation.

Family-group name: CHILENIIDAE Lesne, 1921. Stem: *Chileni*-.

***Chilocorus* Leach, 1815a**: 116. Current status: valid genus in COCCINELLIDAE: COCCINELLINAE: CHILOCORINI.

Type species: *Coccinellacacti* Linnaeus, 1767, by monotypy.

Family-group name: CHILOCORIENS Mulsant, 1846. Stem: *Chilocor*-.

***Chiloea* Dajoz, 1973**: 173. Current status: junior synonym of *Sosteamorphus* Hinton, 1936 in DRYOPIDAE.

Type species: *Chiloeachilensis* Dajoz, 1973 (= *Sosteamorphusverrucatus* Hinton, 1936), by original designation.

Family-group name: CHILOEIDAE Dajoz, 1973. Stem: *Chiloe*-.

***Chiron* W.S. MacLeay, 1819**: 96, 107. Current status: valid genus in SCARABAEIDAE: CHIRONINAE.

Type species: *Sinodendrondigitatum* Fabricius, 1801 (= *Scaritescylindrus* Fabricius, 1798), by monotypy.

Family-group name: CHIRIDITES Blanchard, 1845. Stem: *Chiron*-.

***Chiroscelis* Lamarck, 1804**: 261. Current status: valid genus in TENEBRIONIDAE: LAGRIINAE: PYCNOCERINI.

Type species: *Chiroscelisbifenestra* Lamarck, 1804 (= *Tenebriodigitatus* Fabricius, 1801), by monotypy.

Family-group name: CHIROSCELIDAE Hope, 1841. Stem: *Chiroscelid*-.

***Chlaenioctenus* H.W. Bates, 1892**: 309. Current status: valid genus in CARABIDAE: HARPALINAE: CHLAENIINI: CHLAENIINA.

Type species: *Chlaeniuspectinipes* H.W. Bates, 1892, by subsequent designation (Basilewsky and Grundmann 1954: 243).

Family-group name: CHLAENIOCTENINI Basilewsky & Grundmann, 1955. Stem: *Chlaeniocten*-.

***Chlaeniodus* Jeannel, 1949b**: 778. Current status: valid subgenus of *Chlaenius* Bonelli, 1810 in CARABIDAE: HARPALINAE: CHLAENIINI: CHLAENIINA.

Type species: *Chlaeniusperrieri* Fairmaire, 1901 (= *Chlaeniusperrieranus* Lorenz, 1998), by original designation.

Family-group name: CHLAENIODINI Jeannel, 1949. Stem: *Chlaeniod*-.

***Chlaenionus* Kuntzen, 1913**: 33. Current status: valid subgenus of *Chlaenius* Bonelli, 1810 in CARABIDAE: HARPALINAE: CHLAENIINI: CHLAENIINA.

Type species: *Rembusdohrnii* Bertoloni, 1857, by subsequent designation (Basilewsky 1950: 44).

Family-group name: CHLAENIONINI Jeannel, 1949. Stem: *Chlaenion*-.

***Chlaenius* Bonelli, 1810**: Tab. Syn. Current status: valid genus in CARABIDAE: HARPALINAE: CHLAENIINI: CHLAENIINA.

Type species [suggested]: *Carabusfestivus* Panzer, 1796, by subsequent designation (Madge 1975: 581). Note: no species were originally included in this genus by Bonelli (1810); the first ones were the five species listed by Panzer (1813: 55, 56); the valid type species fixation is that of Hope (1838a: 75), who selected *Carabusspoliatus* Rossi, 1792, but that species is currently included in the subgenus Chlaenites Motschulsky, 1860, not in the nominotypical subgenus; an application to the Commission is necessary to fix *Carabusfestivus* Panzer, 1796 as the type species of *Chlaenius* Bonelli, 1810.

Family-group name: CHLAENIDES Brullé, 1834. Stem: *Chlaeni*-.

***Chlamisus* Rafinesque, 1815**: 116. Current status: valid genus in CHRYSOMELIDAE: CRYPTOCEPHALINAE: FULCIDACINI. Note: new replacement name for *Chlamys* Knoch, 1801.

Type species [suggested]: *Chlamysfoveolata* Knoch, 1801, by subsequent designation (Navajas 1946: 244) (type fixation for *Chlamys* Knoch). Note: see “Note” in the entry for *Chlamys* Knoch, 1801 below.

Family-group name: CHLAMISINAE Gressitt, 1946. Stem: *Chlamis*-.

***Chlamydopsis* Westwood, 1869a**: 317. Current status: valid genus in HISTERIDAE: CHLAMYDOPSINAE.

Type species: *Chlamydopsisstriatella* Westwood, 1869, by subsequent designation (Lewis 1903: 428).

Family-group name: CHLAMYDOPSINI Bickhardt, 1914. Stem: *Chlamydops*-.

***Chlamys* Knoch, 1801**: 122. Current status: invalid senior synonym of *Chlamisus* Rafinesque, 1815 in CHRYSOMELIDAE: CRYPTOCEPHALINAE: FULCIDACINI. Note: junior homonym of *Chlamys* Röding, 1798 [Mollusca].

Type species [suggested]: *Chlamysfoveolata* Knoch, 1801, by subsequent designation (Navajas 1946: 244). Note: the valid type species fixation is that of S.H. Chen (1940: 191), who selected *Chlamystuberosa* Knoch, 1801 (as “*Bruchusgibbosus* F. (= *Chlamystuberosa* Kn.)”), one of the two originally included species (see ICZN 1999a, Article 69.2.2); however, this type species is a junior synonym of *Bruchusgibbosus* Fabricius, 1777 (now in *Neochlamisus* Karren, 1972; see C. Shin et al. 2012: 120); an application to the Commission is necessary to fix *Chlamysfoveolata* Knoch, 1801 as the type species of *Chlamys* Knoch, 1801 in order to conserve the concept for this type genus and its new replacement name.

Family-group name: CHLAMYDEAE Lacordaire, 1848. Stem: *Chlamyd*-. Note: family-group name permanently invalid (ICZN 1999a, Article 39).

***Chlidones* C.O. Waterhouse, 1879a**: 320. Current status: valid genus in CERAMBYCIDAE: CERAMBYCINAE: CHLIDONINI.

Type species: *Chlidoneslineolatus* C.O. Waterhouse, 1879, by monotypy.

Family-group name: CHLIDONINAE C.O. Waterhouse, 1879. Stem: *Chlidon*-.

***Chloemela* Gistel, 1856**: 379. Current status: invalid senior synonym of *Chrysolina* Motschulsky, 1860 (nomen protectum) in CHRYSOMELIDAE: CHRYSOMELINAE: CHRYSOMELINI. Note: nomen oblitum (see J. Bezděk 2020: 180).

Type species: *Chrysomelastaphylaea* Linnaeus, 1758, by subsequent designation (J. Bezděk 2020: 180).

Family-group name: CHLOËMELADAE Gistel, 1856. Stem: *Chloemel*-.

***Chnaunanthus* Burmeister, 1844**: 6, 31. Current status: valid genus in SCARABAEIDAE: MELOLONTHINAE: CHNAUNANTHINI.

Type species: *Chnaunanthusdiscolor* Burmeister, 1844, by monotypy.

Family-group name: CHNAUNANTHINI Evans & A.B.T. Smith, 2020: 63. Stem: *Chnaunanth*-.

***Chnoodes* Chevrolat, 1843a**: 612. Current status: valid genus in COCCINELLIDAE: COCCINELLINAE: COCCIDULINI.

Type species: *Chnoodeschaudoiri* Mulsant, 1850, by subsequent designation (Gordon 2007: 1). Note: no species were originally included in this genus by Chevrolat (1843a); the first species included in the genus are the 13 species listed by Mulsant (1850: 908–915).

Family-group name: CHNOODIENS Mulsant, 1850. Stem: *Chnood*-.

***Choeridium* Lepeletier & Audinet-Serville, 1828**: 356. Current status: junior synonym of *Ateuchus* Weber, 1801 in SCARABAEIDAE: SCARABAEINAE: ATEUCHINI: ATEUCHINA. Note: see Cupello et al. (2021: 7) for details regarding the original spelling of this genus-group name.

Type species: *Choeridiumsimplex* Lepeletier & Audinet-Serville, 1828, by subsequent designation (Chevrolat 1843a: 613).

Family-group name: CHOERIDIDAE Harold, 1867. Stem: *Choeridi*-.

***Choerorhinus* Fairmaire, 1858**: 742. Current status: valid genus in CURCULIONIDAE: COSSONINAE: RHYNCOLINI: CHOERORHININA.

Type species: *Choerorhinussqualidus* Fairmaire, 1858, by monotypy.

Family-group name: CHOERORHINI Folwaczny, 1973. Stem: *Choerorhin*-.

***Choleva* Latreille, 1797**: 14. Current status: valid genus in LEIODIDAE: CHOLEVINAE: CHOLEVINI: CHOLEVINA.

Type species [suggested]: *Luperuscisteloides* Frölich, 1799, by subsequent designation of Jeannel (1922: 43). Note: no species were originally included in *Choleva*; the valid type species fixation is that of Latreille (1802b: 122), who first associated *Tritomasericea* Fabricius, 1787 (as “*Catopssericea*. F.”) with this genus-group name, but that species is the type species of *Ptomaphagus* Hellwig, 1795; an application to the Commission is needed to fix *Luperuscisteloides* Frölich, 1799 as the type species of *Choleva* Latreille, 1797.

Family-group name: CHOLEVIDAE W. Kirby, 1837. Stem: *Cholev*-.

***Cholomus* Roelofs, 1880**: xl. Current status: valid genus in CURCULIONIDAE: MOLYTINAE: CHOLINI: CHOLOMINA.

Type species: *Cholomusvillei* Roelofs, 1880, by monotypy.

Family-group name: CHOLOMINI Vaurie, 1974. Stem: *Cholom*-.

***Cholus* Germar, 1823**: 212. Current status: valid genus in CURCULIONIDAE: MOLYTINAE: CHOLINI: CHOLINA. Note: name placed on the Official List of Generic Names in Zoology (ICZN 1987d, as *Cholus* Germar, 1824).

Type species: *Cholusalbicinctus* Germar, 1823, by subsequent designation (Schönherr 1825: 584; see ICZN 1987d). Note: name placed on the Official List of Specific Names in Zoology (ICZN 1987d, as *Cholusalbicinctus* Germar, 1824).

Family-group name: CHOLIDES Schönherr, 1825. Stem: *Chol*-.

***Chonostropheus* Prell, 1924**: 162. Current status: valid genus in ATTELABIDAE: RHYNCHITINAE: RHYNCHITITAE: DEPORAINI.

Type species: *Attelabustristis* Fabricius, 1794, by monotypy.

Family-group name: CHONOSTROPHEINA Morimoto, 1962. Stem: *Chonostrophe*-.

***Choragus* W. Kirby, 1819**: 447. Current status: valid genus in ANTHRIBIDAE: CHORAGINAE: CHORAGINI. Note: name placed on the Official List of Generic Names in Zoology (ICZN 1994b).

Type species: *Choragussheppardi* W. Kirby, 1819, by monotypy (see ICZN 1994b). Note: name placed on the Official List of Specific Names in Zoology (ICZN 1994b).

Family-group name: CHORAGIDAE W. Kirby, 1819. Stem: *Chorag*-.

***Chorina* Baly, 1866**: 471. Current status: valid genus in CHRYSOMELIDAE: GALERUCINAE: GALERUCINI.

Type species: *Monocestacincta* Clark, 1865, by original designation.

Family-group name: CHORINI Weise, 1923. Stem: *Chorin*-. Note: junior homonym of CHORININAE Dana, 1851 (type genus *Chorinus* Latreille, 1825) in Crustacea.

***Chrestomera* Jeannel, 1962a**: 344. Current status: valid genus in STAPHYLINIDAE: PSELAPHINAE: EUPLECTITAE: TRICHONYCHINI: TRICHONYCHINA.

Type species: *Chrestomeralongicollis* Jeannel, 1962, by original designation.

Family-group name: CHRESTOMERINA Jeannel, 1962. Stem: *Chrestomer*-.

***Chromogeotrupes* Bovo & Zunino, 1983**: 404. Current status: valid subgenus of *Phelotrupes* Jekel, 1866 in GEOTRUPIDAE: GEOTRUPINAE: ENOPLOTRUPINI.

Type species: *Geotrupesauratus* Motschulsky, 1858, by original designation.

Family-group name: CHROMOGEOTRUPINI Zunino, 1984. Stem: *Chromogeotrup*-.

***Chromoptilia* Westwood, 1842b**: 128. Current status: valid genus in SCARABAEIDAE: CETONIINAE: STENOTARSIINI: CHROMOPTILIINA.

Type species: *Chromoptiliadiversipes* Westwood, 1842, by monotypy.

Family-group name: CHROMOPTILIINA Krikken, 1984. Stem: *Chromoptili*-.

***Chrysobothris* Eschscholtz, 1829b**: 9. Current status: valid genus in BUPRESTIDAE: BUPRESTINAE: CHRYSOBOTHRINI. Note: the original spelling was *Chrysobotris* but *Chrysobothris* was ruled the correct spelling by the Commission and the name was placed on the Official List of Generic Names in Zoology (ICZN 1994f).

Type species: *Buprestischrysostigma* Linnaeus, 1758, by subsequent designation (Westwood 1838c: 24; see ICZN 1994f). Note: name placed on the Official List of Specific Names in Zoology (ICZN 1994f).

Family-group name: CHRYSOBOTHRIDAE Gory & Laporte, 1836. Stem: *Chrysobothr*-.

***Chrysochroa* Dejean, 1833a**: 75. Current status: valid genus in BUPRESTIDAE: CHRYSOCHROINAE: CHRYSOCHROINI: CHRYSOCHROINA. Note: this name is a nomen protectum and its senior synonym *Euchloris* Billberg, 1820 is a nomen oblitum (Bílý 2001: 149).

Type species: *Buprestisfulminans* Fabricius, 1787, by subsequent designation (Kurosawa 1982: 185).

Family-group name: CHRYSOCHROIDAE Laporte, 1835. Stem: *Chrysochro*-.

***Chrysodema* Laporte & Gory, 1837**: 1. Current status: valid genus in BUPRESTIDAE: CHRYSOCHROINAE: CHRYSOCHROINI: CHALCOPHORINA. Note: name placed on the Official List of Generic Names in Zoology (ICZN 2004c, as *Chrysodema* Laporte & Gory, 1835).

Type species: *Chrysodemasonnerati* Laporte & Gory, 1837, by plenary power (see ICZN 2004c). Note: name placed on the Official List of Specific Names in Zoology (ICZN 2004c, as *Chrysodemasonnerati* Laporte & Gory, 1835).

Family-group name: CHRYSODÉMIDES Kerremans, 1892. Stem: *Chrysodem*-.

***Chrysodina* Baly, 1864b**: 221. Current status: junior synonym of *Spintherophyta* Dejean, 1836 in CHRYSOMELIDAE: EUMOLPINAE: EUMOLPINI.

Type species: *Chrysodinaigneicollis* Baly, 1864, by monotypy.

Family-group name: CHRYSODINITAE Lefèvre, 1885. Stem: *Chrysodin*-.

***Chrysolina* Motschulsky, 1860b**: 210. Current status: valid genus in CHRYSOMELIDAE: CHRYSOMELINAE: CHRYSOMELINI. Note: given precedence over *Atechna* Chevrolat, 1837 whenever the two names are considered synonyms and name placed on the Official List of Generic Names in Zoology (ICZN 1984c); nomen protectum (see J. Bezdĕk 2020: 180).

Type species: *Chrysomelastaphylaea* Linnaeus, 1758, by original designation (see ICZN 1984c). Note: name placed on the Official List of Specific Names in Zoology (ICZN 1984c).

Family-group name: CHRYSOLININA S.H. Chen, 1936. Stem: *Chrysolin*-.

***Chrysomela* Linnaeus, 1758**: 342, 368. Current status: valid genus in CHRYSOMELIDAE: CHRYSOMELINAE: CHRYSOMELINI. Note: name placed on the Official List of Generic Names in Zoology (ICZN 1984c).

Type species: *Chrysomelapopuli* Linnaeus, 1758, by subsequent designation (Latreille 1810: 432; see ICZN 1984c). Note: name placed on the Official List of Specific Names in Zoology (ICZN 1984c).

Family-group name: CHRYSOMELINAE Latreille, 1802. Stem: *Chrysomel*-.

***Chrysophora* Dejean, 1821**: 60. Current status: valid genus in SCARABAEIDAE: RUTELINAE: RUTELINI: RUTELINA.

Type species: *Melolonthachrysochlora* Latreille, 1809, by monotypy.

Family-group name: CHRYSOPHORIDAE Burmeister, 1844. Stem: *Chrysophor*-.

***Ciceronius* Kaup, 1871**: 29. Current status: valid genus in PASSALIDAE: PASSALINAE: SOLENOCYCLINI.

Type species: *Passalusmorbillosus* Klug, 1833, by monotypy.

Family-group name: CICERONINAE Kuwert, 1891. Stem: *Ciceroni*-.

***Cicindela* Linnaeus, 1758**: 342, 407. Current status: valid genus in CICINDELIDAE: CICINDELINI: CICINDELINA.

Type species: *Cicindelacampestris* Linnaeus, 1758, by subsequent designation (Latreille 1810: 425).

Family-group name: CICINDELETAE Latreille, 1802. Stem: *Cicindel*-.

***Cicindis*Bruch, 1908**: 497. Current status: valid genus in CARABIDAE: CICINDINAE.

Type species: *Cicindishorni*Bruch, 1908, by monotypy.

Family-group name: CICINDINI Csiki, 1927. Stem: *Cicind*-.

***Cillaeus* Laporte, 1834c**: 133. Current status: valid genus in NITIDULIDAE: CILLAEINAE: CILLAEINI.

Type species: *Cillaeuscastaneus* Laporte, 1834, by subsequent designation (E. Desmarest 1851: 290).

Family-group name: CILLAEINAE Kirejtshuk & Audisio, 1986. Stem: *Cillae*-.

***Cimberis* Gozis, 1881**: 150. Current status: valid genus in CIMBERIDIDAE: CIMBERIDINI. Note: name placed on the Official List of Generic Names in Zoology (ICZN 2005c).

Type species: *Rhinomacerattelaboides* Fabricius, 1787, by subsequent designation (Kuschel 1959: 234; see ICZN 2005c). Note: name placed on the Official List of Specific Names in Zoology (ICZN 2005c).

Family-group name: CIMBERIDAE Gozis, 1882. Stem: *Cimberid*-.

***Cinnabarium* Fairmaire, 1894**: ccvi. Current status: junior synonym of *Coptocera* Murray, 1868 in DASCILLIDAE: DASCILLINAE: CINNABARIINI.

Type species: *Cinnabariumseminigrum* Fairmaire, 1894, by monotypy.

Family-group name: CINNABARIINI Pic, 1914. Stem: *Cinnabari*-.

***Cinyra* Laporte & Gory, 1837**: 4. Current status: valid genus in BUPRESTIDAE: CHRYSOCHROINAE: PARALEPTODEMINI: PARALEPTODEMINA.

Type species: *Buprestiselongata* Laporte & Gory, 1837, by subsequent designation (Casey 1909: 53).

Family-group name: CINYRINI Cobos, 1979. Stem: *Cinyr*-.

***Cionus* Clairville, 1798**: 56, 64. Current status: valid genus in CURCULIONIDAE: CURCULIONINAE: CIONINI.

Type species: *Curculioblattariae* Fabricius, 1792 (= *Curculioalauda* Herbst, 1784), by subsequent designation (Crotch 1870a: 50).

Family-group name: CIONIDES Schönherr, 1825. Stem: *Cion*-.

†***Circaeus* Iablokoff-Khnzorian, 1961**: 209. Current status: valid genus in ADERIDAE. Note: Lower Tertiary (Baltic amber).

Type species: †*Circaeusborisjaki* Iablokoff-Khnzorian, 1961, by original designation.

Family-group name: †CIRCAEIDAE Iablokoff-Khnzorian, 1961. Stem: *Circae*-.

***Cis* Latreille, 1797**: 50. Current status: valid genus in CIIDAE: CIINAE: CIINI.

Type species: *Dermestesboleti* Scopoli, 1763, by subsequent monotypy (Latreille 1802b: 205).

Family-group name: CISIDAE Leach, 1819. Stem: *Ci*-.

***Cisanthribus* Zimmerman, 1938**: 247. Current status: valid genus in ANTHRIBIDAE: CHORAGINAE: CISANTHRIBINI.

Type species: *Cisanthribusconvexus* Zimmerman, 1938, by original designation.

Family-group name: CISANTHRIBINI Zimmerman, 1994. Stem: *Cisanthrib*-.

***Cisseis* Gory & Laporte, 1839**: 1. Current status: junior synonym of *Diphucrania* Dejean, 1833 in BUPRESTIDAE: AGRILINAE: CORAEBINI: CISSEINA.

Type species: *Buprestisduodecimguttata* Guérin-Méneville, 1838 (= *Buprestisduodecimmaculata* Fabricius, 1801), by subsequent designation (Duponchel 1843a: 719).

Family-group name: CISSEINA Majer, 2000. Stem: *Cisse*-.

***Cissites* Latreille, 1804a**: 154, 199. Current status: valid genus in MELOIDAE: NEMOGNATHINAE: HORIINI.

Type species: *Cucujusmaculatus* Swederus, 1787, by monotypy.

Family-group name: CISSITINI Kaszab, 1966. Stem: *Cissit*-.

***Cistela* Fabricius, 1775**: 116. Current status: invalid senior synonym of *Cteniopus* Solier, 1835 in TENEBRIONIDAE: ALLECULINAE: CTENIOPODINI. Note: junior homonym of *Cistela* Geoffroy, 1762 [Coleoptera: BYRRHIDAE], which was suppressed for the Principle of Priority but not for the Principle of Homonymy (ICZN 1994a).

Type species: *Chrysomelasulphurea* Linnaeus, 1758, by subsequent designation (Latreille 1810: 429).

Family-group name: CISTELENIAE Latreille, 1802. Stem: *Cistel*-. Note: family-group name permanently invalid (ICZN 1999a, Article 39).

***Cladodes* Solier, 1849**: 444. Current status: valid genus in LAMPYRIDAE: CLADODINAE.

Type species: *Cladodesflabellatus* Solier, 1849, by monotypy.

Family-group name: CLADODINAE Bocáková, Campello-Gonçalves & Silveira, 2022: 1186. Stem: *Cladod*-. Note: this name could be a junior homonym of CLADODIDAE (type genus *Cladodus* Egerton, 1841) in Pisces, although more research is needed to confirm this; if a family-group name based on *Cladodus* is indeed available in Pisces then the case will need to be referred to the Commission for a ruling to remove the homonymy ICZN (1999a, Article 55.3.1).

***Cladognathus* Burmeister, 1847**: 364. Current status: junior synonym of *Prosopocoilus* Hope, 1845 in LUCANIDAE: LUCANINAE: DORCINI. Note: new replacement name for *Macrognathus* Hope, 1845.

Type species: *Lucanusgirafa* G.-A. Olivier, 1789, by subsequent designation (Lacordaire 1855: 29) (type fixation for *Macrognathus* Hope).

Family-group name: CLADOGNATHIDAE Parry, 1870. Stem: *Cladognath*-.

***Cladophorus* Guérin-Méneville, 1830**: pl. 2. Current status: valid genus in LYCIDAE: METRIORRHYNCHINAE: METRIORRHYNCHINI: METRIORRHYNCHINA. Note: this genus-group name was first made available by Guérin-Méneville (1829a: pl. 14) when he illustrated the new species *Cladophorusruficollis*; however, that species is currently placed in the valid genus *Pseudodontocerus* Pic, 1921; in order to preserve nomenclatural stability, we recommend that an application be submitted to the Commission to suppress the name *Cladophorus* Guérin-Méneville, 1829 for both the Principles of Homonymy and Priority.

Type species: *Cladophorusdimidiatus* Guérin-Méneville, 1830, by subsequent designation (Bocák 1998: 247). Note: Bocák (1998: 247) credited the type species designation to C.O. Waterhouse (1878: 103); however, C.O. Waterhouse listed *Cladophorusformosus* Guérin-Méneville, 1838, which is not a species originally included in *Cladophorus*, as type species of this genus.

Family-group name: CLADOPHORINAE Kleine, 1929. Stem: *Cladophor*-.

***Cladotoma* Westwood, 1836**: 254. Current status: valid genus in PTILODACTYLIDAE: CLADOTOMINAE.

Type species: *Cladotomaovalis* Westwood, 1836, by monotypy.

Family-group name: CLADOTOMINI Pic, 1914. Stem: *Cladotom*-.

***Cladoxena* Motschulsky, 1866a**: 428. Current status: valid genus in EROTYLIDAE: LANGURIINAE: LANGURIINI.

Type species: *Cladoxenarufipes* Motschulsky, 1866, by subsequent designation (Crotch 1876: 396).

Family-group name: CLADOXENINAE Arrow, 1925. Stem: *Cladoxen*-.

***Clambus* Fischer, 1821b**: 52. Current status: valid genus in CLAMBIDAE: CLAMBINAE.

Type species: *Dermestesarmadillo* DeGeer, 1774 [as *armadillus*], by subsequent designation (Westwood 1838c: 10).

Family-group name: CLAMBINI Fischer, 1821. Stem: *Clamb*-.

***Clanis* Mulsant, 1850**: 999. Current status: invalid senior synonym of *Jauravia* Motschulsky, 1858 in COCCINELLIDAE: COCCINELLINAE: STICHOLOTIDINI. Note: junior homonym of *Clanis* Hübner, 1819 [Lepidoptera].

Type species: *Coccinellapubescens* Fabricius, 1798, by monotypy.

Family-group name: CLANINI Weise, 1901. Stem: *Clan*-. Note: family-group name permanently invalid (ICZN 1999a, Article 39).

***Claviger* Preyssler, 1790**: 68. Current status: valid genus in STAPHYLINIDAE: PSELAPHINAE: CLAVIGERITAE: CLAVIGERINI.

Type species: *Clavigertestaceus* Preyssler, 1790, by monotypy.

Family-group name: CLAVIGERIDES Leach, 1815. Stem: *Claviger*-. Note: senior homonym of CLAVIGERINAE J.B. Waterhouse, 1975 (type genus *Clavigera* J.A. Thomson, 1913) in Brachiopoda.

***Clavigerodes* Raffray, 1877**: 279. Current status: valid genus in STAPHYLINIDAE: PSELAPHINAE: CLAVIGERITAE: CLAVIGERINI.

Type species: *Clavigerodesabyssinicus* Raffray, 1877, by monotypy.

Family-group name: CLAVIGERODINI L.W. Schaufuss, 1882. Stem: *Clavigerod*-.

***Clavigeropsis* Raffray, 1882**: 3. Current status: valid genus in STAPHYLINIDAE: PSELAPHINAE: CLAVIGERITAE: CLAVIGERINI.

Type species: *Clavigeropsisformicarius* Raffray, 1882, by monotypy.

Family-group name: CLAVIGEROPSINI L.W. Schaufuss, 1890. Stem: *Clavigeropse*-.

***Clavilispinus* Bernhauer, 1926**: 255. Current status: valid genus in STAPHYLINIDAE: OSORIINAE: THORACOPHORINI: CLAVILISPININA.

Type species: *Paralispinussiargaoanus* Bernhauer, 1926, by monotypy.

Family-group name: CLAVILISPININA Newton & Thayer, 1992. Stem: *Clavilispin*-.

***Clavipalpus* Laporte, 1833b**: 406. Current status: valid genus in SCARABAEIDAE: MELOLONTHINAE: MACRODACTYLINI.

Type species: *Clavipalpusdejeanii* Laporte, 1833, by monotypy.

Family-group name: CLAVIPALPIDES Lacordaire, 1855. Stem: *Clavipalp*-.

***Cleidecosta* P.J. Johnson, 2002**: 16. Current status: valid genus in ELATERIDAE: AGRYPNINAE: EUPLINTHINI: CLEIDECOSTINA. Note: new replacement name for *Heligmus* Candèze, 1865.

Type species: *Heligmusglyphoderus* Candèze, 1865, by monotypy (type fixation for *Heligmus* Candèze).

Family-group name: CLEIDECOSTINI P.J. Johnson, 2002. Stem: *Cleidecost*-.

***Cleidostethus* Arrow, 1929a**: 464. Current status: valid genus in CORYLOPHIDAE: CORYLOPHINAE: CLEIDOSTETHINI.

Type species: *Cleidostethusmeliponae* Arrow, 1929, by monotypy.

Family-group name: CLEIDOSTETHINI Bowestead, Booth, Ślipiński & Lawrence, 2001. Stem: *Cleidosteth*-.

***Clema* Semenov, 1900**: 590. Current status: valid genus in BUPRESTIDAE: AGRILINAE: CORAEBINI: CLEMINA.

Type species: *Clemadeserti* Semenov, 1900, by original designation.

Family-group name: CLEMINA Majer, 2000. Stem: *Clem*- (ICZN 1999a, Article 29.4).

***Cleogonus* Schönherr, 1825**: 586. Current status: valid genus in CURCULIONIDAE: MOLYTINAE: CLEOGONINI.

Type species: *Curculiorubetra* Fabricius, 1787, by original designation.

Family-group name: CLEOGONIDAE Gistel, 1848. Stem: *Cleogon*-.

***Cleomenes* J. Thomson, 1864**: 161. Current status: valid genus in CERAMBYCIDAE: CERAMBYCINAE: CLEOMENINI.

Type species: *Cleomenesdihammaphoroides* J. Thomson, 1864, by original designation.

Family-group name: CLÉOMÉNIDES Lacordaire, 1868. Stem: *Cleomen*-.

***Cleonis* Dejean, 1821**: 96. Current status: valid genus in CURCULIONIDAE: LIXINAE: CLEONINI.

Type species: *Curculiosulcirostris* Linnaeus, 1767 (= *Curculiopiger* Scopoli, 1763), by subsequent designation (Chevrolat 1843b: 6).

Family-group name: CLEONIDAE W. Kirby, 1837. Stem: *Cleon*-. Note: junior homonym of CLEONIDES Schönherr, 1826 (type genus *Cleonus* Schönherr, 1826) in Coleoptera: CURCULIONIDAE.

***Cleonus* Schönherr, 1826**: 145. Current status: junior synonym of *Cleonis* Dejean, 1821 in CURCULIONIDAE: LIXINAE: CLEONINI. Note: unnecessary new replacement name for *Geomorus* Schönherr, 1823.

Type species: *Curculiosulcirostris* Linnaeus, 1767 (= *Curculiopiger* Scopoli, 1763), by original designation (type fixation for *Geomorus* Schönherr).

Family-group name: CLEONIDES Schönherr, 1826. Stem: *Cleon*-. Note: nomen protectum (see Bouchard et al. 2011: 620); senior homonym of CLEONIDAE W. Kirby, 1837 (type genus *Cleonis* Dejean, 1821) in Coleoptera: CURCULIONIDAE.

***Cleopterium* Gistel, 1856**: 360. Current status: junior synonym of *Acrotrichis* Motschulsky, 1848 in PTILIIDAE: PTILIINAE: ACROTRICHINI.

Type species: *Latridiusfascicularis* Herbst, 1793, by subsequent designation (Biström and Silfverberg 1979: 13).

Family-group name: CLEOPTERIIDAE Gistel, 1856. Stem: *Cleopteri*-.

***Cleropiestus* Fairmaire, 1889**: xcii. Current status: valid genus in CLERIDAE: CLERINAE: OPILONITAE: OPILONINI: CLEROPIESTINA.

Type species: *Cleropiestusoberthurii* Fairmaire, 1890, by monotypy.

Family-group name: CLEROPIESTINAE J.R. Winkler, 1980. Stem: *Cleropiest*-.

***Clerus* Geoffroy, 1762**: 303. Current status: valid genus in CLERIDAE: CLERINAE: CLERITAE: CLERINI. Note: name attributed to Geoffroy, 1762 and placed on the Official List of Generic Names in Zoology (ICZN 1984a).

Type species: *Clerusmutillarius* Fabricius, 1775, by plenary power (see ICZN 1984a). Note: name placed on the Official List of Specific Names in Zoology (ICZN 1984a).

Family-group name: CLERII Latreille, 1802. Stem: *Cler*-.

†***Clessidromma* Jarzembowski, B. Wang & Zheng, 2018**: 112. Current status: valid genus in OMMATIDAE incertae sedis. Note: mid-Cretaceous (Myanmar).

Type species: †*Clessidrommapalmeri* Jarzembowski, B. Wang & Zheng, 2018, by original designation.

Family-group name: †CLESSIDROMMATINI Jarzembowski, B. Wang & Zheng, 2018: 112. Stem: *Clessidrommat*-.

***Clidicus* Laporte, 1833b**: 396. Current status: valid genus in STAPHYLINIDAE: SCYDMAENINAE: MASTIGITAE: CLIDICINI.

Type species: *Clidicusgrandis* Laporte, 1833, by monotypy.

Family-group name: CLIDICINI Casey, 1897. Stem: *Clidic*-.

***Clinidium* W. Kirby, 1830**: 6. Current status: valid genus in CARABIDAE: RHYSODINAE: CLINIDIINI.

Type species: *Clinidiumguildingii* W. Kirby, 1830, by monotypy.

Family-group name: CLINIDIINA R.T. Bell & J.R. Bell, 1978. Stem: *Clinidi*-.

***Clinolabus* Jekel, 1860**: 196. Current status: valid genus in ATTELABIDAE: ATTELABINAE: ATTELABINI: EUSCELINA.

Type species: *Attelabusangulatus* Fabricius, 1787, by original designation. Note: Jekel (1860) designated two type species separately in his work, one (*Attelabusangulatus* Fabricius, 1787) on page 197, the other (*Attelabusmelancoryphus* Germar, 1823) in the table [unpaginated page]; Alonso-Zarazaga and Lyal (1999: 45) acted as First Revisers (ICZN 1999a, Article 24.2.2) for the type species selection.

Family-group name: CLINOLABINA Legalov, 2003. Stem: *Clinolab*-.

***Clinteria* Burmeister, 1842**: 299. Current status: valid genus in SCARABAEIDAE: CETONIINAE: GYMNETINI: GYMNETINA.

Type species: *Clinteriaguttifera* Burmeister, 1842 [by 6 December 1842] (= *Gymnetisauronotata* Blanchard, 1842 [November 1842]), by subsequent designation (Duponchel 1843b: 10).

Family-group name: CLINTERIIDAE Kraatz, 1882. Stem: *Clinteri*-.

***Clitostylus* Voss, 1929b**: 193. Current status: junior synonym of *Trachelismus* Motschulsky, 1870 in ATTELABIDAE: APODERINAE: CLITOSTYLINI: CLITOSTYLINA.

Type species: *Apoderusbadeni* Faust, 1883 (= *Apoderusmacrostylus* Motschulsky, 1861), by subsequent designation (Legalov 2003: 476). Note: Legalov (2003: 476) selected *Apoderusmacrostylus* Motschulsky, 1861 as type species of *Clitostylus* Voss, 1929, which is not an originally included species, but since he listed it in synonymy with *Apoderusbadeni* Faust, 1883, an originally included species, he is deemed to have designated the latter species as type species (ICZN 1999a, Article 69.2.2).

Family-group name: CLITOSTYLINI Voss, 1929. Stem: *Clitostyl*-.

***Clivina* Latreille, 1802b**: 96. Current status: valid genus in CARABIDAE: SCARITINAE: CLIVININI: CLIVININA.

Type species: *Scaritesarenarius* Fabricius, 1775 (= *Tenebriofossor* Linnaeus, 1758), by monotypy.

Family-group name: CLIVINIDIA Rafinesque, 1815. Stem: *Clivin*-.

***Cloniocerus* Dejean, 1835**: 340. Current status: valid genus in CERAMBYCIDAE: LAMIINAE: CLONIOCERINI.

Type species: *Lamiahystrix* Fabricius, 1781, by monotypy.

Family-group name: CLONIOCÉRIDES Lacordaire, 1872. Stem: *Cloniocer*-.

***Closterus* Audinet-Serville, 1832**: 193. Current status: valid genus in CERAMBYCIDAE: PRIONINAE: MEROSCELISINI.

Type species: *Closterusflabellicornis* Audinet-Serville, 1832, by monotypy.

Family-group name: CLOSTÉRIDES Lacordaire, 1868. Stem: *Closter*-.

***Clypeaster* Dejean, 1821**: 129. Current status: invalid senior synonym of *Clypastraea* Haldeman, 1842 in CORYLOPHIDAE: CORYLOPHINAE: PARMULINI. Note: junior homonym of *Clypeaster* Lamarck, 1801 [Echinodermata].

Type species: *Cossyphuspusillus* Gyllenhal, 1810, by monotypy.

Family-group name: CLYPEASTRINA Perty, 1840. Stem: *Clypeaster*-. Note: family-group name permanently invalid (ICZN 1999a, Article 39).

***Clytra* Laicharting, 1781**: 165. Current status: valid genus in CHRYSOMELIDAE: CRYPTOCEPHALINAE: CLYTRINI: CLYTRINA.

Type species: *Chrysomelaquadripunctata* Linnaeus, 1758, by subsequent designation (Latreille 1810: 432).

Family-group name: CLYTHRIDAE W. Kirby, 1837. Stem: *Clytr*-.

***Clytus* Laicharting, 1784**: 88. Current status: valid genus in CERAMBYCIDAE: CERAMBYCINAE: CLYTINI.

Type species: *Lepturaarietis* Linnaeus, 1758, by subsequent designation (Curtis 1828: pl. 199).

Family-group name: CLYTAIRES Mulsant, 1839. Stem: *Clyt*-.

***Cnemalobus* Guérin-Méneville, 1839a**: pl. 226 (p. 9). Current status: valid genus in CARABIDAE: HARPALINAE: CNEMALOBINI.

Type species: *Cnemalobusdesmarestii* Guérin-Méneville, 1839, by subsequent designation (Roig-Juñent 1993: 10).

Family-group name: CNEMALOBINI Germain, 1911. Stem: *Cnemalob*-.

***Cnemeplatia* A. Costa, 1847**: 146. Current status: valid genus in TENEBRIONIDAE: PIMELIINAE: CNEMEPLATIINI: CNEMEPLATIINA.

Type species: *Cnemeplatiaatropos* A. Costa, 1847, by monotypy.

Family-group name: CNÉMÉPLATIITES Jacquelin du Val, 1861. Stem: *Cnemeplati*-.

***Cnemidontus* Schenkling, 1935**: 29. Current status: valid genus in CURCULIONIDAE: MESOPTILIINAE: MESOPTILIINI. Note: new replacement name for *Cnemidophorus* Schönherr, 1835.

Type species: *Cnemidophorusfasciculatus* Boheman, 1835, by original designation (type fixation for *Cnemidophorus* Schönherr).

Family-group name: CNEMIDONTINI Kuschel, 1955. Stem: *Cnemidont*-.

***Cnemidophorus* Schönherr, 1835**: 276. Current status: invalid senior synonym of *Cnemidontus* Schenkling, 1935 in CURCULIONIDAE: MESOPTILIINAE: MESOPTILIINI. Note: junior homonym of *Cnemidophorus* Wagler, 1830 [Reptilia].

Type species: *Cnemidophorusfasciculatus* Boheman, 1835, by original designation.

Family-group name: CNEMIDOPHORINI Hustache, 1937. Stem: *Cnemidophor*-. Note: family- group name permanently invalid (ICZN 1999a, Article 39).

***Cnemodinus* Cockerell, 1906**: 242. Current status: valid genus in TENEBRIONIDAE: PIMELIINAE: CNEMODININI. Note: new replacement name for *Cnemodus* G.H. Horn, 1870.

Type species: *Cnemodustestaceus* G.H. Horn, 1870, by monotypy (type fixation for *Cnemodus* G.H. Horn).

Family-group name: CNEMODININAE Gebien, 1910. Stem: *Cnemodin*-.

***Cnemodus* G.H. Horn, 1870**: 266. Current status: invalid senior synonym of *Cnemodinus* Cockerell, 1906 in TENEBRIONIDAE: PIMELIINAE: CNEMODININI. Note: junior homonym of *Cnemodus* Herrich- Schäffer, 1850 [Hemiptera].

Type species: *Cnemodustestaceus* G.H. Horn, 1870, by monotypy.

Family-group name: CNEMODINI G.H. Horn, 1870. Stem: *Cnemod*-. Note: family-group name permanently invalid (ICZN 1999a, Article 39).

***Cnemogonus* J.L. LeConte in J.L. LeConte and G.H. Horn, 1876**: 269. Current status: valid genus in CURCULIONIDAE: CEUTORHYNCHINAE: CNEMOGONINI.

Type species: **here fixed** (under ICZN 1999a, Article 70.3.2) as *Cnemogonuslecontei* Dietz, 1896, misidentified as *Curculioepilobii* Paykull, 1800 (sensu J.L. LeConte, 1876) in the designation by monotypy by J.L. LeConte in J.L. LeConte and G.H. Horn (1876: 269).

Family-group name: CNEMOGONINI Colonnelli, 1979. Stem: *Cnemogon*-.

***Cnemoplites* Newman, 1842b**: 351. Current status: valid genus in CERAMBYCIDAE: PRIONINAE: MACROTOMINI: MACROTOMINA.

Type species: *Cnemoplitesedulis* Newman, 1842, by subsequent designation (J. Thomson 1864: 301).

Family-group name: CNEMOPLITINAE Schröder, 1905. Stem: *Cnemoplit*-.

***Cneoglossa* Guérin-Méneville, 1843a**: 194. Current status: valid genus in CNEOGLOSSIDAE.

Type species: *Cneoglossacollaris* Guérin-Méneville, 1849, by subsequent monotypy (Guérin- Méneville 1849: [no 36] 1).

Family-group name: CNEOGLOSSINI Champion, 1897. Stem: *Cneogloss*-.

***Cneorhinus* Schönherr, 1823**: 1139. Current status: valid genus in CURCULIONIDAE: ENTIMINAE: POLYDRUSITAE: CNEORHININI.

Type species: *Curculiobarcelonicus* Herbst, 1797, by original designation.

Family-group name: CNÉORHINIDES Lacordaire, 1863. Stem: *Cneorhin*-.

***Cnides* Motschulsky, 1862**: 38. Current status: valid genus in CARABIDAE: TRECHINAE: TRECHINI: TRECHODINA.

Type species: *Cnidesrostratus* Motschulsky, 1862, by monotypy.

Family-group name: CNIDINA Jeannel, 1958. Stem: *Cnid*-.

***Cnodalon* Latreille, 1797**: 23. Current status: valid genus in TENEBRIONIDAE: STENOCHIINAE: CNODALONINI. Note: name placed on the Official List of Generic Names in Zoology (ICZN 2023).

Type species: *Cnodalonviride* Latreille, 1804, by plenary power (see ICZN 2023). Note: no species were originally included in this genus by Latreille (1797); Bousquet and Bouchard (2019: 114, 116) submitted a request to the Commission to retain *Cnodalonviride* Latreille, 1804, the currently accepted type species of the genus, after the discovery that this was not an originally included species; name placed on the Official List of Specific Names in Zoology (ICZN 2023).

Family-group name: NODALIDEN Oken, 1843. Stem: *Cnodalon*-.

***Cnopus* Champion, 1893**: 460. Current status: valid genus in ADERIDAE.

Type species: *Cnopusflohri* Champion, 1893, by monotypy.

Family-group name: CNOPINA Báguena Corella, 1948. Stem: *Cnop*-.

***Coccidula* Kugelann in Illiger, 1798**: 421. Current status: valid genus in COCCINELLIDAE: COCCINELLINAE: COCCIDULINI.

Type species: *Dermestesrufus* Herbst, 1783, by subsequent designation (C.G. Thomson 1859: 161). Note: *Chrysomelascutellata* Herbst, 1783 is usually listed as the type species of this genus (e.g., Kovář 2007: 572; Szawaryn et al. 2021: 64) by subsequent designation of Crotch (1874a: 300) but C.G. Thomson’s designation is earlier; both species are currently included in the genus *Coccidula*.

Family-group name: COCCIDULIENS Mulsant, 1846. Stem: *Coccidul*-.

***Coccinella* Linnaeus, 1758**: 342, 364. Current status: valid genus in COCCINELLIDAE: COCCINELLINAE: COCCINELLINI.

Type species: *Coccinellaseptempunctata* Linnaeus, 1758, by subsequent designation (Latreille 1810: 432).

Family-group name: COCCINELLIDAE Latreille, 1807. Stem: *Coccinell*-.

***Coelaenomenodera* Blanchard, 1845a**: 181. Current status: valid genus in CHRYSOMELIDAE: CASSIDINAE: COELAENOMENODERINI.

Type species: *Acentropteracucullata* Guérin-Méneville, 1844, by subsequent designation (Baly 1858: 119). Note: no species were originally included in this genus by Blanchard (1845a); the first ones were the four species listed by Baly (1858: 119, 120).

Family-group name: COELAENOMENODERINI Weise, 1911. Stem: *Coelaenomenoder*-.

***Coeliodes* Schönherr, 1837**: 282. Current status: valid genus in CURCULIONIDAE: CEUTORHYNCHINAE: CEUTORHYNCHINI. Note: name placed on the Official List of Generic Names in Zoology (ICZN 1989b).

Type species: *Curculioquercus* Linnaeus, 1758 (sensu Fabricius, 1787) (= *Curculiorana* Fabricius, 1787), by original designation (see ICZN 1989b). Note: the Commission (ICZN 1989b) ruled that the type species of *Coeliodes* Schönherr, 1837 was “*Curculioquercus* Fabricius, 1787 (a junior primary homonym replaced by *Curculiodryados* Gmelin, 1790)” and placed *Curculiodryados* Gmelin, 1790 on the Official List of Specific Names in Zoology; however, *Curculioquercus* Fabricius, 1787 is not available as Fabricius misidentified *Curculioquercus* Linnaeus, 1758 (currently *Orchestesquercus* (Linnaeus, 1758)); the taxon *Curculioquercus* Linnaeus, 1758 (sensu Fabricius, 1787) is currently known under the valid name *Curculiorana* Fabricius, 1787 (see Alonso-Zarazaga et al. 2017: 141); an application to the Commission is necessary to change the entry for *Coeliodes* Schönherr, 1837 and its type species on the Official Lists.

Family-group name: COELIODES J.L. LeConte, 1876. Stem: *Coeliod*-.

***Coelomera* Chevrolat in Dejean, 1836a**: 375. Current status: valid genus in CHRYSOMELIDAE: GALERUCINAE: GALERUCINI.

Type species: *Chrysomelacajennensis* Fabricius, 1787, by subsequent designation (Weise 1924: 51).

Family-group name: COELOMÉRITES Chapuis, 1875. Stem: *Coelomer*-.

***Coelometopon* E. Janssens, 1972**: 386. Current status: valid genus in HYDRAENIDAE: PROSTHETOPINAE: COELOMETOPONINI.

Type species: *Coelometoponleleupi* E. Janssens, 1972, by original designation.

Family-group name: COELOMETOPONINI Perkins, 2005. Stem: *Coelometopon*-.

***Coelometopus* Solier, 1848b**: 154, 278. Current status: valid genus in TENEBRIONIDAE: STENOCHIINAE: CNODALONINI.

Type species: *Coelometopusclypeatus* Solier, 1848 (= *Blaps clypeata* Germar, 1813), by monotypy.

Family-group name: COELOMETOPIDAE Schaum, 1859. Stem: *Coelometop*-.

***Coelonertus* A. Solari & F. Solari, 1906**: 441. Current status: valid genus in CURCULIONIDAE: BARIDINAE: APOSTASIMERINI: COELONERTINA.

Type species: *Coelonertussquamulosus* A. Solari & F. Solari, 1906, by subsequent designation (Champion 1908: 335).

Family-group name: COELONERTINI Casey, 1922. Stem: *Coelonert*-.

***Coelophora* Mulsant, 1850**: 390. Current status: valid genus in COCCINELLIDAE: COCCINELLINAE: COCCINELLINI.

Type species: *Coccinellainaequalis* Fabricius, 1775, by subsequent designation (Crotch 1874a: 148).

Family-group name: COELOPHORAIRES Mulsant, 1850. Stem: *Coelophor*-.

***Coelopterus* Mulsant & Rey, 1853c**: 89. Current status: valid genus in COCCINELLIDAE: COCCINELLINAE: STICHOLOTIDINI. Note: this genus is often attributed to Mulsant & Rey, 1852 (p. 224); however, that version of the article (in the ‘Mémoires de l’Académie Nationale des Sciences…’) was evidently not published until after the version in the ‘Opuscules Entomologiques’, in 1853 (see Reference 1853c cited above).

Type species: *Coelopterussalinus* Mulsant & Rey, 1853, by monotypy.

Family-group name: COELOPTERINI Weise, 1906. Stem: *Coelopter*-.

***Coelorrhina* Hope, 1841b**: 302. Current status: valid genus in SCARABAEIDAE: CETONIINAE: GOLIATHINI: RHOMBORHININA. Note: the original spelling was *Caelorrhina* but *Coelorrhina*, an incorrect subsequent spelling, is in prevailing usage, attributed to the publication of the original spelling, and therefore deemed to be the correct original spelling (ICZN 1999a, Article 33.3.1).

Type species: *Coelorrhinaconcolor* Hope, 1841, by monotypy.

Family-group name: COELORRHINAE Schoch, 1895. Stem: *Coelorrhin*-.

***Coelostoma* Brullé, 1835**: 293. Current status: valid genus in HYDROPHILIDAE: SPHAERIDIINAE: COELOSTOMATINI.

Type species: *Hydrophilusorbicularis* Fabricius, 1775, by monotypy.

Family-group name: COELOSTOMITAE Heyden, 1891. Stem: *Coelostomat*-.

***Coelus* Eschscholtz, 1829d**: 5. Current status: valid genus in TENEBRIONIDAE: PIMELIINAE: CONIONTINI.

Type species: *Coelusciliatus* Eschscholtz, 1829, by monotypy.

Family-group name: COELINI Casey, 1907. Stem: *Coel*-.

***Coenochilus* Schaum in Burmeister and Schaum, 1841**: 250, 268. Current status: valid genus in SCARABAEIDAE: CETONIINAE: CREMASTOCHEILINI: COENOCHILINA.

Type species: *Cremastocheiluspaulus* Gory & Percheron, 1833 (= *Cetoniamaura* Fabricius, 1801), by subsequent designation (Duponchel 1843b: 78).

Family-group name: COENOCHILIDAE Burmeister, 1842. Stem: *Coenochil*-.

***Colaphus* Dejean, 1836a**: 411. Current status: junior synonym of *Colaspidema* Laporte, 1833 in CHRYSOMELIDAE: CHRYSOMELINAE: CHRYSOMELINI. Note: unnecessary new replacement name for *Colaspidema* Laporte, 1833; *Colaphus* has been attributed to L. Redtenbacher (1845: 116) in the literature and considered a junior homonym of *Colaphus* Dejean, 1836; however, both names apply to the same taxon; the earlier usage of the name *Colaphus* by Dahl (1823: 46) was suppressed for the purposes of zoological nomenclature (see ICZN 1964).

Type species: *Colaspisbarbara* Fabricius, 1801, by monotypy (type fixation for *Colaspidema* Laporte).

Family-group name: COLAPHIDAE Siegel, 1866. Stem: *Colaph*-.

***Colaspis* Fabricius, 1801a**: xxi, 411. Current status: valid genus in CHRYSOMELIDAE: EUMOLPINAE: EUMOLPINI.

Type species: *Chrysomelaflavicornis* Fabricius, 1787 (= *Chrysomelaoccidentalis* Linnaeus, 1758), by subsequent designation (Latreille 1810: 432).

Family-group name: COLASPIDAE Hope, 1841. Stem: *Colaspid*-.

***Colaspoides* Laporte, 1833a**: 20. Current status: valid genus in CHRYSOMELIDAE: EUMOLPINAE: EUMOLPINI.

Type species: *Colaspislimbata* G.-A. Olivier, 1808, by subsequent designation (Baly 1864c: 134). Note: Moseyko and Sprecher-Uebersax (2010b: 632) listed *Cryptocephaluslimbatus* Fabricius, 1781 as type species of this genus; however, Baly (1864c: 134) designated “*Colaspoideslimbata*, Oliv.” as type species, an originally included species, and there is no reference to *Cryptocephaluslimbatus* Fabricius, 1781 in G.-A. Olivier’s text; the species described by G.-A. Olivier and Fabricius are both from Cayenne and whether or not they are synonymous has yet to be documented.

Family-group name: COLASPOIDINI S.H. Chen, 1940. Stem: *Colaspoid*-.

***Colasposoma* Laporte, 1833a**: 22. Current status: valid genus in CHRYSOMELIDAE: EUMOLPINAE: EURYOPINI.

Type species: *Colasposomasenegalensis* Laporte, 1833, by monotypy.

Family-group name: COLASPOSOMINI Špringlová de Bechyné, 1960. Stem: *Colasposomat*-.

***Colecerus* Schönherr, 1840a**: 927. Current status: valid genus in CURCULIONIDAE: ENTIMINAE: ENTIMITAE: EUDIAGOGINI.

Type species: *Colecerussetosus* Boheman, 1840, by original designation.

Family-group name: COLEOCERINI Jekel, 1875. Stem: *Colecer*-.

***Coleomerus* Schönherr, 1836**: 817. Current status: valid genus in CURCULIONIDAE: BARIDINAE: BARIDINI: COLEOMERINA.

Type species: *Coleomerusebeninus* Gyllenhal, 1836, by original designation.

Family-group name: COLEOMERINI Casey, 1922. Stem: *Coleomer*-.

†***Coleopsis* Kirejtshuk, Poschmann & Nel in Kirejtshuk et al., 2013**: 579. Current status: valid genus in †COLEOPSIDAE. Note: lower Permian (Germany).

Type species: †*Coleopsisarchaica* Kirejtshuk, Poschmann & Nel, 2013, by original designation.

Family-group name: †COLEOPSIDAE Kirejtshuk & Nel, 2016a: 71. Stem: *Coleops*- (ICZN 1999a, Article 29.4). Note: Kirejtshuk (2020: 10) proposed the emendation COLEOPSEIDAE for this family-group name but the work is nomenclaturally unavailable (ICZN 2012f, Article 8.5.3.1) and, consequently, the emendation is unavailable as well.

***Colilodion* Besuchet, 1991**: 500. Current status: valid genus in STAPHYLINIDAE: PSELAPHINAE: PSELAPHITAE: COLILODIONINI.

Type species: *Colilodioninopinatus* Besuchet, 1991, by original designation.

Family-group name: COLILODIONINI Besuchet, 1991. Stem: *Colilodion*-.

***Colliuris* DeGeer, 1774**: 79. Current status: valid genus in CARABIDAE: HARPALINAE: ODACANTHINI: ODACANTHINA.

Type species: *Attelabussurinamensis* Linnaeus, 1758, by monotypy.

Family-group name: COLLIURINI Bedel, 1910. Stem: *Colliur*-.

***Collops* Erichson, 1840a**: 54. Current status: valid genus in MELYRIDAE: MALACHIINAE: MALACHIINI.

Type species: *Malachiusbipunctatus* Say, 1823, by subsequent designation (Mayor 2007a: 60).

Family-group name: COLLOPINA Hatch, 1962. Stem: *Collop*-.

***Collyris* Fabricius, 1801a**: xxiii, 226. Current status: valid genus in CICINDELIDAE: COLLYRIDINI: COLLYRIDINA.

Type species: *Cicindelalongicollis* Fabricius, 1787, by subsequent designation (Latreille 1810: 425).

Family-group name: COLLYRIENS Brullé, 1834. Stem: *Collyrid*-.

***Colobodera* Klug, 1837**: 53. Current status: senior synonym of *Daemon* Laporte, 1838 in PTILODACTYLIDAE: ANCHYTARSINAE. Note: this name is considered a junior synonym of *Daemon* Laporte, 1838 (a name usually incorrectly listed as published in 1836); an application to the Commission is needed to retain *Daemon* Laporte, 1838 as the valid name for this genus.

Type species: *Coloboderaovata* Klug, 1837, by **present designation**.

Family-group name: COLOBODERIDES Erichson, 1847. Stem: *Coloboder*-.

***Colobodes* Schönherr, 1837**: 465. Current status: valid genus in CURCULIONIDAE: MOLYTINAE: ITHYPORINI: COLOBODINA.

Type species: *Colobodesbillbergii* Boheman, 1837, by original designation.

Family-group name: COLOBODINI Voss, 1958. Stem: *Colobod*-.

***Colobothea* Lepeletier & Audinet-Serville, 1825**: 336. Current status: valid genus in CERAMBYCIDAE: LAMIINAE: COLOBOTHEINI. Note: the original spelling was *Colobotea* but *Colobothea*, an incorrect subsequent spelling, is in prevailing usage, attributed to the publication of the original spelling, and therefore deemed to be the correct original spelling (ICZN 1999a, Article 33.3.1).

Type species: *Saperdacassandra* Dalman, 1823, by subsequent designation (Duponchel 1843b: 120).

Family-group name: COLOBOTHEITAE J. Thomson, 1860. Stem: *Colobothe*-.

***Colon* Herbst, 1797**: 224. Current status: valid genus in COLONIDAE. Note: the original spelling *Kolon* was changed to *Colon* and *Colon* Herbst, 1797 placed on the Official List of Generic Names in Zoology (ICZN 1995c).

Type species [suggested]: *Colonviennense* Herbst, 1797, by subsequent designation (C.G. Thomson 1859: 60; see ICZN 1995c, as *Kolonviennensis*). Note: name placed on the Official List of Specific Names in Zoology (ICZN 1995c); an earlier type species designation is that of *Colonclavigerum* Herbst, 1797 (as “*Colonclaviger* Herbst”) by Duponchel (1843b: 124); since the ruling by the ICZN (1995c) did not include a statement such as “all type species designations for prior to that of C.G. Thomson (1859) are hereby set aside”, Duponchel’s designation should be considered the valid type species fixation; however, *Colonclavigerum* is not currently classified in the nominotypical subgenus and using it would cause instability; an application should be submitted to the Commission to maintain *Colonviennense* Herbst, 1797 as the type species and dismiss Duponchel’s and any other type species designations before that of C.G. Thomson (1859: 60).

Family-group name: COLONES G.H. Horn, 1880. Stem: *Colon*-.

***Colotes* Erichson, 1840a**: 129. Current status: valid genus in MELYRIDAE: MALACHIINAE: MALACHIINI.

Type species: *Colotestrinotatus* Erichson, 1840 (= *Malachiusmaculatus* Laporte, 1838), by subsequent designation (Jacquelin du Val 1859a: 47).

Family-group name: CAULAUTAIRES Abeille de Perrin, 1890. Stem: *Colot*-.

***Colpochila* Erichson, 1843c**: 195. Current status: valid genus in SCARABAEIDAE: SERICOIDINAE: LIPARETRINI.

Type species: *Sericesthisgouldii* Hope, 1842, by monotypy.

Family-group name: COLPOCHILINIBritton, 1957. Stem: *Colpochil*-.

***Colpoderus* Audinet-Serville, 1832**: 178. Current status: junior synonym of *Notophysis* Audinet- Serville, 1832 in CERAMBYCIDAE: PRIONINAE: CACOSCELINI. Note: First Reviser (ICZN 1999a, Article 24.2.2) for *Notophysis* Audinet-Serville, 1832 versus *Colpoderus* Audinet-Serville, 1832 is Lameere (1903: 20).

Type species: *Colpoderuscaffer* Audinet-Serville, 1832, by monotypy.

Family-group name: COLPODÉRIDES Lacordaire, 1868. Stem: *Colpoder*-.

***Colpodes* W.S. MacLeay, 1825**: 17. Current status: valid genus in CARABIDAE: HARPALINAE: PLATYNINI.

Type species: *Rembusbrunneus* W.S. MacLeay, 1825, by monotypy.

Family-group name: COLPODIDAS Chaudoir, 1872. Stem: *Colpod*-. Note: senior homonym of COLPODIDAE Poche, 1913 (type genus *Colpoda* O.F. Müller, 1773) in Protista.

***Colpothis* Gistel, 1848b**: 216. Current status: junior synonym of *Dasytes* Paykull, 1798 in MELYRIDAE: DASYTINAE: DASYTINI. Note: unnecessary new replacement name for *Dasytes* Paykull, 1798.

Type species: *Hispacoerulea* Fabricius, 1775 (= *Telephoruscaeruleus* DeGeer, 1774), by subsequent designation (Westwood 1838c: 28) (type fixation for *Dasytes* Paykull).

Family-group name: COLPOTHISIDAE Gistel, 1848. Stem: *Colpoth*-.

***Colydiopeltis* Ślipiński, 1992**: 444. Current status: valid genus in LOPHOCATERIDAE.

Type species: *Colydiopeltisburckhardti* Ślipiński, 1992, by original designation.

Family-group name: COLYDIOPELTINI Kolibáč, 2006. Stem: *Colydiopelt*-.

***Colydium* Fabricius, 1792b**: 495. Current status: valid genus in ZOPHERIDAE: COLYDIINAE: COLYDIINI. Note: name placed on the Official List of Generic Names in Zoology (ICZN 1995d).

Type species: *Bostrichuselongatus* Fabricius, 1787, by subsequent designation (Latreille 1810: 431; see ICZN 1995d). Note: name placed on the Official List of Specific Names in Zoology (ICZN 1995d).

Family-group name: COLYDIIDES Billberg, 1820. Stem: *Colydi*-.

***Colymbetes* Clairville, 1806**: 188. Current status: valid genus in DYTISCIDAE: COLYMBETINAE.

Type species: *Dytiscusstriatus* Linnaeus, 1758, by subsequent designation (Curtis 1828: pl. 207).

Family-group name: COLYMBETINI Erichson, 1837. Stem: *Colymbet*-.

***Colymbomorpha* Blanchard in H. Milne-Edwards et al., 1850**: 98. Current status: valid genus in SCARABAEIDAE: MELOLONTHINAE: COLYMBOMORPHINI.

Type species: *Colymbomorphalineata* Blanchard, 1850, by monotypy.

Family-group name: COLYMBOMORPHITAE Blanchard, 1850. Stem: *Colymbomorph*-.

†***Colymbotethis* Ponomarenko, 1994**: 189. Current status: valid genus in †COLYMBOTETHIDAE. Note: Upper Triassic (Kazakhstan).

Type species: †*Colymbotethisantecessor* Ponomarenko, 1994, by original designation.

Family-group name: †COLYMBOTETHIDAE Ponomarenko, 1994. Stem: *Colymboteth*-.

***Commatocerus* Raffray, 1882**: 1. Current status: valid genus in STAPHYLINIDAE: PSELAPHINAE: CLAVIGERITAE: CLAVIGERINI.

Type species: *Commatoceruselegantulus* Raffray, 1882, by monotypy.

Family-group name: COMMATOCERINI L.W. Schaufuss, 1882. Stem: *Commatocer*-.

***Comophorina* Strand, 1928**: 3. Current status: valid genus in SCARABAEIDAE: MELOLONTHINAE: COMOPHINI. Note: new replacement name for *Comophorus* Blanchard, 1850.

Type species: *Comophorustestaceipennis* Blanchard, 1850, by monotypy (type fixation for *Comophorus* Blanchard).

Family-group name: COMOPHORININIBritton, 1987. Stem: *Comophorin*-.

***Comophorus* Blanchard in H. Milne-Edwards et al., 1850**: 106. Current status: invalid senior synonym of *Comophorina* Strand, 1928 in SCARABAEIDAE: MELOLONTHINAE: COMOPHINI. Note: junior homonym of *Comophorus* Agassiz, 1846 [Pisces].

Type species: *Comophorustestaceipennis* Blanchard, 1850, by monotypy.

Family-group name: COMOPHORINIBritton, 1957. Stem: *Comophor*-. Note: family-group name permanently invalid (ICZN 1999a, Article 39).

***Comophus*Britton, 1978**: 7. Current status: junior synonym of *Comophorina* Strand, 1928 in SCARABAEIDAE: MELOLONTHINAE: COMOPHINI. Note: new replacement name for *Comophorus* Blanchard, 1850.

Type species: *Comophorustestaceipennis* Blanchard, 1850, by monotypy (type fixation for *Comophorus* Blanchard).

Family-group name: COMOPHINIBritton, 1978. Stem: *Comoph*-.

***Compactopedia* Kistner, 1970c**: 18. Current status: valid genus in STAPHYLINIDAE: ALEOCHARINAE: ALEOCHARINI: COMPACTOPEDIINA.

Type species: *Compactopediacompactus* Kistner, 1970, by original designation.

Family-group name: COMPACTOPEDINA Kistner, 1970. Stem: *Compactopedi*-.

***Compsa* Perty, 1832**: 92. Current status: valid genus in CERAMBYCIDAE: CERAMBYCINAE: TROPIDINI: COMPSINA.

Type species: *Compsaalbopicta* Perty, 1832, by subsequent designation (R. Lucas 1920: 198).

Family-group name: COMPSINA Martins & Galileo, 2007. Stem: *Comps*-. Note: junior homonym of COMPSINI Pierce, 1913 (type genus *Compsus* Schönherr, 1823) in Coleoptera: CURCULIONIDAE.

***Compsocerus* Audinet-Serville, 1834b**: 62. Current status: valid genus in CERAMBYCIDAE: CERAMBYCINAE: COMPSOCERINI. Note: this genus-group name was first made available by Lacordaire (1830b: 175) when he listed *Saperdabarbicornis* Fabricius, 1792 (as “*C.barbicornis* (*Saperdaplumigera*, Oliv.)”) within it; however, that species is the type species of *Paromoeocerus* Gounelle, 1910, and a request should be submitted to the Commission to suppress *Compsocerus* Lacordaire, 1830 for the purposes of the Principles of Priority and Homonymy (see Bousquet et al. 2009: 44).

Type species: *Compsocerusbarbicornis* Audinet-Serville, 1834, by monotypy.

Family-group name: COMPSOCERITAE J. Thomson, 1864. Stem: *Compsocer*-.

***Compsocnemis* Bonvouloir, 1871**: 72 [in key]. Current status: valid genus in EUCNEMIDAE: MELASINAE: MELASINI: COMPSOCNEMINA. Note: this name was first used by Gemminger in Gemminger and von Harold (1869b: 1479) but is unavailable from that date as the three species listed under the name were nomina nuda; the relevant plate of Bonvouloir’s work on EUCNEMIDAE referred to by Gemminger was not issued until 1872 (1872b).

Type species: *Compsocnemismaculata* Bonvouloir, 1872, by subsequent designation (Muona 1987: 82). Note: no species were originally included in this genus by Bonvouloir (1871); the first ones were the species illustrated by Bonvouloir (1872b: plate 33).

Family-group name: COMPSOCNEMINA Muona, 1993. Stem: *Compsocnem*-.

***Compsoplinthus* C. Costa, 1975**: 71. Current status: valid genus in ELATERIDAE: AGRYPNINAE: EUPLINTHINI: COMPSOPLINTHINA.

Type species: *Pyrophorusruber* Candèze, 1878, by original designation.

Family-group name: COMPSOPLINTHINA C. Costa, 1975. Stem: *Compsoplinth*-.

***Compsosoma* Lacordaire, 1830b**: 183. Current status: valid genus in CERAMBYCIDAE: LAMIINAE: COMPSOSOMATINI.

Type species: *Compsosomaniveosignata* Audinet-Serville, 1835, by subsequent designation (E. Desmarest 1860: 325). Note: no available species was originally included for this genus in Lacordaire (1830b); the first ones are the three species listed by Audinet-Serville (1835a: 56, 57).

Family-group name: COMPSOSOMITES J. Thomson, 1857. Stem: *Compsosomat*-.

***Compsus* Schönherr, 1823**: 1140. Current status: valid genus in CURCULIONIDAE: ENTIMINAE: POLYDRUSITAE: EUSTYLINI: EUSTYLINA.

Type species: *Curculioelegans* G.-A. Olivier, 1807 (= *Curculioargyreus* Linnaeus, 1758), by monotypy.

Family-group name: COMPSI Pierce, 1913. Stem: *Comps*-. Note: senior homonym of COMPSINA Martins & Galileo, 2007 (type genus *Compsa* Perty, 1832) in Coleoptera: CERAMBYCIDAE.

***Conalia* Mulsant & Rey, 1858**: 313. Current status: valid genus in MORDELLIDAE: MORDELLINAE: CONALIINI.

Type species: *Conaliabaudii* Mulsant & Rey, 1858, by monotypy.

Family-group name: CONALIINI Ermisch, 1956. Stem: *Conali*-.

***Conderis* C.O. Waterhouse, 1880a**: 59. Current status: valid genus in LYCIDAE: LYCINAE: CONDERINI.

Type species: *Calopteronsignicolle* Kirsch, 1875, by original designation.

Family-group name: CONDERINI Bocák & Bocáková, 1990. Stem: *Conder*-.

***Coniatus* Germar, 1817b**: 340. Current status: valid genus in CURCULIONIDAE: HYPERINAE: HYPERINI: CONIATINA.

Type species: *Curculiotamarisci* Fabricius, 1787, by subsequent designation (Schönherr 1823: 1143).

Family-group name: CONIATINA Legalov, 2007. Stem: *Coniat*-.

***Coniontis* Eschscholtz, 1829d**: 7. Current status: valid genus in TENEBRIONIDAE: PIMELIINAE: CONIONTINI.

Type species: *Coniontisviatica* Eschscholtz, 1829, by subsequent designation (Casey 1908: 57).

Family-group name: CONIONTIDAE G.R. Waterhouse, 1858. Stem: *Coniont*-.

***Coniporus* C.G. Thomson, 1859**: 90. Current status: junior synonym of *Aspidiphorus* Dejean, 1821 in SPHINDIDAE: SPHINDINAE. Note: name placed on the Official Index of Rejected and Invalid Generic Names in Zoology (see ICZN 1997).

Type species: *Nitidulaorbiculata* Gyllenhal, 1808, by original designation.

Family-group name: CONIPORINA C.G. Thomson, 1859. Stem: *Conipor*-. Note: family-group name permanently invalid (ICZN 1999a, Article 39).

†***Conjunctia* Ponomarenko, 1977**: 87. Current status: valid genus in CARABIDAE: †CONJUNCTIINAE. Note: Lower Cretaceous (Russia; China).

Type species: †*Conjunctiaprodroma* Ponomarenko, 1977, by original designation.

Family-group name: †CONJUNCTIINI Ponomarenko, 1977. Stem: *Conjuncti*-.

***Conoderes* Schönherr, 1833**: 26. Current status: valid genus in CURCULIONIDAE: CONODERINAE: CONODERINI.

Type species: *Conoderesalbidus* Schönherr, 1833, by original designation.

Family-group name: CONODERIDES Schönherr, 1833. Stem: *Conoder*-. Note: senior homonym of CONODERINAE Fleutiaux, 1919 (type genus *Conoderus* Eschscholtz, 1829) in Coleoptera: ELATERIDAE.

***Conoderus* Eschscholtz, 1829a**: 31. Current status: junior synonym of *Monocrepidius* Eschscholtz, 1829 in ELATERIDAE: AGRYPNINAE: OOPHORINI. Note: the First Reviser (ICZN 1999a, Article 24.2.2) for *Conoderus* Eschscholtz, 1829 versus *Monocrepidius* Eschscholtz, 1829 is Candèze (1859: 187).

Type species: *Conoderusfuscofasciatus* Eschscholtz, 1829, by subsequent designation (Duponchel 1843b: 188).

Family-group name: CONODERINAE Fleutiaux, 1919. Stem: *Conoder*-. Note: junior homonym of CONODERINAE Schönherr, 1833 (type genus *Conoderes* Schönherr, 1833) in Coleoptera: CURCULIONIDAE.

***Cononotus* J.L. LeConte, 1851a**: 137. Current status: valid genus in PYROCHROIDAE: AGNATHINAE.

Type species: *Cononotussericans* J.L. LeConte, 1851, by subsequent designation (Spilman 1954: 88).

Family-group name: CONONOTINI J.L. LeConte, 1862. Stem: *Cononot*-.

***Conopalpus* Gyllenhal, 1810**: 547. Current status: valid genus in MELANDRYIDAE: OSPHYINAE.

Type species: *Conopalpusflavicollis* Gyllenhal, 1810 (= *Melyristestacea* G.-A. Olivier, 1790), by monotypy.

Family-group name: CONOPALPIENS Mulsant, 1856. Stem: *Conopalp*-.

***Conophorus* Schönherr, 1838**: 719. Current status: junior synonym of *Conoderes* Schönherr, 1833 in CURCULIONIDAE: CONODERINAE: CONODERINI. Note: junior homonym of *Conophorus* Meigen, 1803 [Diptera]; unnecessary new replacement name for *Conoderes* Schönherr, 1833.

Type species: *Conoderesalbidus* Schönherr, 1833, by original designation (type fixation for *Conoderes* Schönherr).

Family-group name: CONOPHORIDES Schönherr, 1838. Stem: *Conophor*-. Note: family-group name permanently invalid (ICZN 1999a, Article 39).

***Conosoma* Kraatz, 1857b**: 431. Current status: junior synonym of *Tachinus* Gravenhorst, 1802 in STAPHYLINIDAE: TACHYPORINAE: TACHYPORINI: EUCONOSOMATINA. Note: junior homonym of *Conosoma* Lenz, 1802 [Diptera]; new replacement name for *Conurus* Stephens, 1829.

Type species: *Oxyporusbipustulatus* Fabricius, 1792, by subsequent designation (Westwood 1838c: 19) (type fixation for *Conurus* Stephens).

Family-group name: CONOSOMINI Jeannel & Jarrige, 1949. Stem: *Conosomat*-. Note: family- group name permanently invalid (ICZN 1999a, Article 39).

***Conotelus* Erichson, 1843a**: 249. Current status: valid genus in NITIDULIDAE: CILLAEINAE: CONOTELINI.

Type species: *Stenusconicus* Fabricius, 1801, by subsequent designation (E. Desmarest 1851: 291).

Family-group name: CONOTELINI Kirejtshuk & Kovalev, 2022: 12. Stem: *Conotel*-.

***Conotrachelus* Dejean, 1835**: 296. Current status: valid genus in CURCULIONIDAE: MOLYTINAE: CLEOGONINI.

Type species: *Balaninusdiaconitus* Klug, 1829, by subsequent designation (Schönherr 1837: 392).

Family-group name: CONOTRACHELIDES Jekel, 1865. Stem: *Conotrachel*-.

***Coomania* Cameron, 1939**: 22. Current status: valid genus in STAPHYLINIDAE: STAPHYLININAE: COOMANIINI.

Type species: *Coomaniatonkinensis* Cameron, 1939, by monotypy.

Family-group name: COOMANIINAE Żyła & Solodovnikov, 2019: 124. Stem: *Coomani*-.

***Coomaniella* Bourgoin, 1924**: 178. Current status: valid genus in BUPRESTIDAE: BUPRESTINAE: COOMANIELLINI.

Type species: *Coomaniellamodesta* Bourgoin, 1924, by subsequent designation (Bílý 1974: 30).

Family-group name: COOMANIELLINI Bílý, 1974. Stem: *Coomaniell*-.

***Copelatus* Erichson, 1832**: 18, 38. Current status: valid genus in DYTISCIDAE: COPELATINAE.

Type species: *Dytiscusposticatus* Fabricius, 1801, by monotypy.

Family-group name: COPELATINI Branden, 1884. Stem: *Copelat*-.

***Copobaenus* Fairmaire & Germain, 1863**: 236. Current status: valid genus in ANTHICIDAE: COPOBAENINAE.

Type species: *Copobaenustristis* Fairmaire & Germain, 1863, by subsequent designation (R. Lucas 1920: 201).

Family-group name: COPOBAENINAE M. Abdullah, 1969. Stem: *Copobaen*-.

†***Coprinisphaera* Sauer, 1955**: 129. Current status: valid genus in †COPRINISPHAERIDAE. Note: Ichnotaxon; Upper Cretaceous to Pleistocene (Antarctica, Argentina, Ecuador, Kenya, Pakistan, Somalia, Tanzania, Uruguay, and USA); this name was given precedence over *Fontanai* Roselli, 1939, a rarely used senior subjective synonym whenever the two are considered to be synonyms, and *Coprinisphaera* Sauer, 1955 was placed on the Official List of Generic Names in Zoology (ICZN 2008b).

Type species: †*Coprinisphaeraecuadoriensis* Sauer, 1955, by monotypy (see ICZN 2008b). Note: name placed on the Official List of Specific Names in Zoology ICZN 2008b).

Family-group name: †COPRINISPHAERIDAE Genise, 2004. Stem: *Coprinisphaer*-.

***Copris* Geoffroy, 1762**: 87. Current status: valid genus in SCARABAEIDAE: SCARABAEINAE: COPRINI. Note: name placed on the Official List of Generic Names in Zoology (ICZN 1994a).

Type species: *Scarabaeuslunaris* Linnaeus, 1758, by subsequent designation (Latreille 1810: 428; see ICZN 1994a). Note: name placed on the Official List of Specific Names in Zoology (ICZN 1994a).

Family-group name: COPRIDES Leach, 1815. Stem: *Copr*-.

***Coprobius* Latreille, 1829a**: 535. Current status: junior synonym of *Canthon* Hoffmansegg, 1817 in SCARABAEIDAE: SCARABAEINAE: DELTOCHILINI.

Type species: *Scarabaeusvolvens* Fabricius, 1792 (= *Scarabaeuspilularius* Linnaeus, 1758), by subsequent designation (Reiche 1841: 213).

Family-group name: COPROBIADAE Burmeister, 1873. Stem: *Coprobi*-.

***Coprophilus* Latreille, 1829a**: 439. Current status: valid genus in STAPHYLINIDAE: OXYTELINAE: COPROPHILINI. Note: name placed on the Official List of Generic Names in Zoology (ICZN 1993b).

Type species: *Staphylinusstriatulus* Fabricius, 1792, by plenary power (see ICZN 1993b). Note: name placed on the Official List of Specific Names in Zoology (ICZN 1993b).

Family-group name: COPROPHILINA Heer, 1839. Stem: *Coprophil*-.

†***Coptoclava* Ping, 1928**: 39. Current status: valid genus in †COPTOCLAVIDAE: †COPTOCLAVINAE. Note: Lower Cretaceous (China; Mongolia; Russia; South Korea).

Type species: †*Coptoclavalongipoda* Ping, 1928, by original designation.

Family-group name: †COPTOCLAVIDAE Ponomarenko, 1961. Stem: *Coptoclav*-.

†***Coptoclavisca* Ponomarenko, 1987**: 84. Current status: valid genus in †COPTOCLAVIDAE: †COPTOCLAVISCINAE. Note: Lower Cretaceous (China; Mongolia).

Type species: †*Coptoclaviscanigricollinus* Ponomarenko, 1987, by original designation.

Family-group name: †COPTOCLAVISCINAE Soriano, Ponomarenko & Delclòs, 2007. Stem: *Coptoclavisc*-.

***Coptocycla* Chevrolat in Dejean, 1836a**: 372. Current status: valid genus in CHRYSOMELIDAE: CASSIDINAE: CASSIDINI.

Type species: *Cassidaundecimpunctata* Fabricius, 1781, by subsequent designation (Duponchel and Chevrolat 1842b: 211).

Family-group name: COPTOCYCLITAE Spaeth & Reitter, 1926. Stem: *Coptocycl*-.

***Coptodactyla* Burmeister, 1846**: [*Copris* 5. Subgen., in key]. Current status: valid genus in SCARABAEIDAE: SCARABAEINAE: COPRINI.

Type species: *Coptodactylaemana* Burmeister, 1846, by monotypy. Note: combined description of a new genus-group taxon and a single new nominal species (ICZN 1999a, Article 12.2.6).

Family-group name: COPTODACTYLINI Paulian, 1933. Stem: *Coptodactyl*-.

***Coptodera* Dejean, 1825**: 169, 273. Current status: valid genus in CARABIDAE: HARPALINAE: LEBIINI: PERICALINA.

Type species: *Coptoderafestiva* Dejean, 1825, by subsequent designation (Hope 1838a: 85).

Family-group name: COSTODÉRIDES Chaudoir, 1848. Stem: *Coptoder*-.

***Coptomia* Burmeister, 1842**: 549. Current status: valid genus in SCARABAEIDAE: CETONIINAE: STENOTARSIINI: COPTOMIINA.

Type species: *Cetoniamauritania* Gory & Percheron, 1833, by subsequent designation (Duponchel 1843b: 202).

Family-group name: COPTOMIINI Schenkling, 1921. Stem: *Coptomi*-.

***Coptomma* Newman, 1840a**: 18. Current status: valid genus in CERAMBYCIDAE: CERAMBYCINAE: COPTOMMATINI.

Type species: *Coptommavirgatum* Newman, 1840 (= *Callidiumvariegatum* Fabricius, 1775), by subsequent designation (Lacordaire 1869: 223). Note: Lacordaire (1869) selected *Callidiumvariegatum* Fabricius, 1775, not an originally included nominal species, as type species but since he listed it in synonymy with *Coptommavirgatum* Newman, 1840, an originally included species, he is deemed to have designated the latter species as type species (ICZN 1999a, Article 69.2.2).

Family-group name: COPTOMMIDES Lacordaire, 1869. Stem: *Coptommat*-.

***Coptonotus* Chapuis, 1869**: 11. Current status: valid genus in CURCULIONIDAE: SCOLYTINAE: COPTONOTINI.

Type species: *Coptonotuscyclopus* Chapuis, 1869, by monotypy.

Family-group name: COPTONOTIDAE Chapuis, 1869. Stem: *Coptonot*-.

***Coptotermoecia* Oke, 1933**: 135. Current status: valid genus in STAPHYLINIDAE: ALEOCHARINAE: ATHETINI: COPTOTERMOECIINA.

Type species: *Coptotermoeciaalutacia* Oken, 1933, by original designation.

Family-group name: COPTOTERMOECIINA Kistner & Pasteels, 1970. Stem: *Coptotermoeci*-.

***Coptotomus* Say, 1830b**: 29. Current status: valid genus in DYTISCIDAE: COPTOTOMINAE.

Type species: *Coptotomusserripalpus* Say, 1830, by monotypy.

Family-group name: COPTOTOMINI Branden, 1884. Stem: *Coptotom*-.

***Copturus* Schönherr, 1825**: 586. Current status: valid genus in CURCULIONIDAE: CONODERINAE: LECHRIOPINI.

Type species: *Poecilmapapaveratum* Germar, 1823, by original designation.

Family-group name: COPTURIDAE Desbrochers des Loges, 1892. Stem: *Coptur*-.

***Coraebus* Gory & Laporte, 1839**: 1. Current status: valid genus in BUPRESTIDAE: AGRILINAE: CORAEBINI: CORAEBINA.

Type species: *Buprestisundatus* Fabricius, 1787, by subsequent designation (Descarpentries and Villiers 1967: 471).

Family-group name: CORAEBINI Bedel, 1921. Stem: *Coraeb*-.

***Cordobanus* Bernhauer, 1910**: 386. Current status: valid genus in STAPHYLINIDAE: ALEOCHARINAE: CORDOBANINI.

Type species: *Cordobanusmirabilis* Bernhauer, 1910, by monotypy.

Family-group name: CORDOBANINI Bernhauer, 1910. Stem: *Cordoban*-.

***Cordylospasta* G.H. Horn, 1876**: 152. Current status: valid genus in MELOIDAE: MELOINAE: EUPOMPHINI.

Type species: *Cordylospastafulleri* G.H. Horn, 1876, by monotypy.

Family-group name: CORDYLOSPASTIDES Wellman, 1910. Stem: *Cordylospast*-.

***Coriacephilus* Schedl, 1939**: 339. Current status: valid genus in CURCULIONIDAE: SCOLYTINAE: CORIACEPHILINI.

Type species: *Stephanoderescoriaceus* Eichhoff, 1878, by original designation.

Family-group name: CORIACEPHILINI A.J. Johnson in A.J. Johnson et al., 2020: 12. Stem: *Coriacephil*-.

***Corigetus* Desbrochers des Loges, 1872**: 662. Current status: valid genus in CURCULIONIDAE: ENTIMINAE: CYPHICERITAE: CYPHICERINI: CYPHICERINA.

Type species: *Corigetusmarmoratus* Desbrochers des Loges, 1873, by subsequent monotypy (Desbrochers des Loges 1873a: 746).

Family-group name: CORIGETINI Faust, 1885. Stem: *Coriget*-.

***Corimalia* Gozis, 1885**: 129. Current status: valid genus in BRENTIDAE: NANOPHYINAE: CORIMALIINI.

Type species: *Nanophyestamarisci* Gyllenhal, 1838, by subsequent designation (Pajni and Bhateja 1982: 433).

Family-group name: CORIMALIINI Alonso-Zarazaga, 1989. Stem: *Corimali*-.

***Corintascaris* Basilewsky, 1952a**: 227. Current status: valid genus in CARABIDAE: SCARITINAE: SCARITINI: CORINTASCARINA.

Type species: *Corintascarisferreirae* Basilewsky, 1952, by original designation.

Family-group name: CORINTASCARINI Basilewsky, 1973. Stem: *Corintascar*-.

***Cormodes* Pascoe, 1860b**: 46. Current status: valid genus in CLERIDAE: CLERINAE: NATALIINI: CORMODINA.

Type species: *Cormodesdarwini* Pascoe, 1860, by monotypy.

Family-group name: CORMODINA Bartlett, 2021: 750. Stem: *Cormod*-. Note: two spellings were originally used for the name of this new subtribe, including CORMODIINA (p. 764); as far as we know no one has acted as First Reviser (ICZN 1999a, Article 24.2.3) and we **here select**CORMODINA as the correct spelling.

***Corneolabium* Steel, 1950**: 54. Current status: valid genus in STAPHYLINIDAE: OMALIINAE: CORNEOLABIINI.

Type species: *Corneolabiummandibularis* Steel, 1950, by original designation.

Family-group name: CORNEOLABIINI Steel, 1950. Stem: *Corneolabi*-.

***Corotoca* Schiødte, 1854**: 102. Current status: valid genus in STAPHYLINIDAE: ALEOCHARINAE: COROTOCINI: COROTOCINA.

Type species: *Corotocamelantho* Schiødte, 1854, by subsequent designation (Fenyes 1919: 21).

Family-group name: COROTOCINI Fenyes, 1919. Stem: *Corotoc*-.

***Corrhecerus* Schönherr, 1826**: 40. Current status: valid genus in ANTHRIBIDAE: ANTHRIBINAE: CORRHECERINI.

Type species: *Anthribusmixtus* Germar, 1823, by original designation.

Family-group name: CORRHÉCÉRIDES Lacordaire, 1865. Stem: *Corrhecer*-.

***Corsyra* Dejean, 1825**: 168, 326. Current status: valid genus in CARABIDAE: HARPALINAE: CORSYRINI.

Type species: *Cymindisfusula* Fischer, 1820, by monotypy.

Family-group name: CORSYRINI Ganglbauer, 1891. Stem: *Corsyr*-.

***Corthylus* Erichson, 1836**: 64. Current status: valid genus in CURCULIONIDAE: SCOLYTINAE: CORTHYLINI: CORTHYLINA.

Type species: *Bostrichuscompressicornis* Fabricius, 1801, by subsequent designation (E. Desmarest 1860: 275).

Family-group name: CORTHYLI J.L. LeConte, 1876. Stem: *Corthyl*-.

***Corticaria* Marsham, 1802**: 106. Current status: valid genus in LATRIDIIDAE: CORTICARIINAE. Note: name placed on the Official List of Generic Names in Zoology (ICZN 2011e).

Type species: *Corticariaferruginea* Marsham, 1802, by subsequent designation (Dajoz 1975: 205; see ICZN 2011e). Note: name placed on the Official List of Specific Names in Zoology (ICZN 2011e).

Family-group name: CORTICARIDAE Curtis, 1829. Stem: *Corticari*-.

***Corticeus* Piller & Mitterpacher, 1783**: 87. Current status: valid genus in TENEBRIONIDAE: DIAPERINAE: HYPOPHLAEINI.

Type species: *Corticeusunicolor* Piller & Mitterpacher, 1783, by monotypy.

Family-group name: CORTICEINI Boddy, 1965. Stem: *Cortice*-.

***Corticus* Germar, 1823**: 146. Current status: valid genus in ZOPHERIDAE: COLYDIINAE: SYNCHITINI. Note: name first published as a synonym of *Sarrotrium* Illiger, 1798 and subsequently used as valid before 1961 by several authors, including Latreille (1829b: 24); see ICZN (1999a, Article 11.6.1).

Type species: *Sarrotriumceltis* Germar, 1823, by monotypy.

Family-group name: CORTICIDES Reitter, 1882. Stem: *Cortic*-.

***Corylophodes* A. Matthews, 1885**: 160. Current status: junior synonym of *Holopsis* Broun, 1883 in CORYLOPHIDAE: CORYLOPHINAE: PELTINODINI.

Type species: *Corylophusmarginicollis* J.L. LeConte, 1852, by **present designation**. Note: *Corylophodespunctipennis* A. Matthews, 1899 is often listed as the type species of this genus (e.g., Bowestead 1999: 23) by subsequent designation of Paulian (1950: 22) but that species is not an originally included species.

Family-group name: CORYLOPHODINI Paulian, 1950. Stem: *Corylophod*-.

***Corylophus* Stephens, 1833a**: 26. Current status: valid genus in CORYLOPHIDAE: CORYLOPHINAE: CORYLOPHINI.

Type species: *Dermestescassidoides* Marsham, 1802, by monotypy.

Family-group name: CORYLOPHI J.L. LeConte, 1852. Stem: *Coryloph*-.

***Corymbites* Latreille, 1834**: 150. Current status: junior synonym of *Ctenicera* Latreille, 1829 in ELATERIDAE: DENDROMETRINAE: PROSTERNINI.

Type species: *Elaterpectinicornis* Linnaeus, 1758, by subsequent designation (C.G. Thomson 1859: 102).

Family-group name: CORYMBITINI J.L. LeConte, 1861. Stem: *Corymbit*-.

***Corynemerus* Fåhraeus, 1871**: 281. Current status: valid genus in CURCULIONIDAE: CONODERINAE: CORYNEMERINI.

Type species: *Corynemerusfemoralis* Fåhraeus, 1871, by subsequent designation (Faust 1898b: 78).

Family-group name: CORYNEMERINAE Hustache, 1929. Stem: *Corynemer*-.

***Corynodes* Hope, 1841a**: 162. Current status: junior synonym of *Platycorynus* Chevrolat, 1836 in CHRYSOMELIDAE: EUMOLPINAE: EUMOLPINI.

Type species: *Chrysomelacyanea* Fabricius, 1792, by original designation. Note: the type species listed by Hope (1841a: 162) is “E[umolpus]. Cyaneus, Fabricius” and Fabricius (1801a: 419) referred this name to his *Chrysomelacyanea* Fabricius, 1792 described from “America meridionali”; it seems that the locality listed by Fabricius (1792a: 324; 1801a: 419) could be erroneous as G.-A. Olivier (1808: 900) recorded the species from “Bengale”, more research is needed to ascertain all the facts; the type species usually listed for this genus is *Eumolpuscompressicornis* Fabricius, 1801 described from Guinea (e.g., Jacoby 1908: 492; Moseyko and Sprecher-Uebersax 2010b: 633).

Family-group name: CORYNODINA T.A. Marshall, 1865. Stem: *Corynod*-.

***Corynofrea* Aurivillius, 1911a**: 37. Current status: valid genus in CERAMBYCIDAE: LAMIINAE: CEROPLESINI: CROSSOTINA.

Type species: *Corynofreamirabilis* Aurivillius, 1911, by monotypy.

Family-group name: CORYNOFREINAE Aurivillius, 1911. Stem: *Corynofre*-.

***Corynomalus* Chevrolat in Dejean, 1836a**: 439. Current status: valid genus in ENDOMYCHIDAE: LYCOPERDININAE. Note: this name is often credited to Erichson (1847c: 181) (e.g., Strohecker 1980: 14; Tomaszewska 2005: 17) but it was first made available by Chevrolat in 1836.

Type species: *Eumolpuslimbatus* G.-A. Olivier, 1808 (= *Erotylusmarginatus* Fabricius, 1798), by subsequent designation (Bousquet and Bouchard 2013a: 139).

Family-group name: CORYNOMALIDAE Gorham, 1873. Stem: *Corynomal*-.

***Coryphium* Stephens, 1834**: 344. Current status: valid genus in STAPHYLINIDAE: OMALIINAE: CORYPHIINI: CORYPHIINA. Note: name placed on the Official List of Generic Names in Zoology (ICZN 1990b).

Type species: *Coryphiumangusticolle* Stephens, 1834, by monotypy (see ICZN 1990b). Note: name placed on the Official List of Specific Names in Zoology (ICZN 1990b).

Family-group name: CORYPHIINA Jakobson, 1908. Stem: *Coryphi*-.

***Coryphocera* Burmeister, 1842**: 220. Current status: valid subgenus of *Dicheros* Gory & Percheron, 1833 in SCARABAEIDAE: CETONIINAE: GOLIATHINI: RHOMBORHININA.

Type species: *Dicheroscuvera* Newman, 1838, by subsequent designation (Mikšić 1974: 753).

Family-group name: CORYPHOCERIDAE Burmeister, 1842. Stem: *Coryphocer*-.

***Corypticus* Sturm, 1843**: 347. Current status: junior synonym of *Xiphodontus* Westwood, 1838 in LUCANIDAE: LUCANINAE: LUCANINI. Note: two original spellings were used, including *Coryptius* (p. 136); Bouchard et al. (2011: 233) acted as First Revisers (ICZN 1999a, Article 24.2.3) and selected *Corypticus*.

Type species: *Corypticuscapensis* Sturm, 1843 (= *Xiphodontusantilope* Westwood, 1838), by monotypy.

Family-group name: CORYPTICIDAE Gistel, 1848. Stem: *Coryptic*-.

***Coryssomerus* Schönherr, 1825**: 583. Current status: junior synonym of *Poecilma* Germar, 1821 in CURCULIONIDAE: CONODERINAE: CORYSSOMERINI. Note: see Anzaldo (2022: 339) regarding the synonymy of the type genus and usage of *Poecilma* as valid, however, Alonso-Zarazaga et al. (2023: 8) advocate for usage of *Coryssomerus* as valid while an application to the Commission is being prepared.

Type species: *Rhynchaenuscapucinus* Beck, 1817, by original designation.

Family-group name: CORYSSOMERINA C.G. Thomson, 1859. Stem: *Coryssomer*-.

***Coryssopus* Schönherr, 1826**: 303. Current status: valid genus in CURCULIONIDAE: CONODERINAE: CORYSSOPODINI.

Type species: *Zygopshexastichus* Schönherr, 1826, by original designation.

Family-group name: CORYSSOPIDES Lacordaire, 1865. Stem: *Coryssopod*-.

***Corythoderus* Klug, 1845**: [Decas 5], sign. b. Current status: valid genus in SCARABAEIDAE: APHODIINAE: CORYTHODERINI.

Type species: *Corythoderusloripes* Klug, 1845, by monotypy.

Family-group name: CORYTHODERINA A. Schmidt, 1910. Stem: *Corythoder*-.

***Coscinia* Dejean, 1831**: 478. Current status: invalid senior synonym of *Cymbionotum* Baudi di Selve, 1864 in CARABIDAE: MELAENINAE. Note: junior homonym of *Coscinia* Hübner, 1819 [Lepidoptera].

Type species: *Siagonaschuppelii* Dejean, 1825, by subsequent designation (Hope 1838a: 81).

Family-group name: COSCINIDES Chaudoir, 1876. Stem: *Coscini*-. Note: family-group name permanently invalid (ICZN 1999a, Article 39).

***Cosmovalgus* Kolbe, 1897a**: 190, 204. Current status: valid genus in SCARABAEIDAE: CETONIINAE: VALGINI: VALGINA.

Type species: *Cosmovalgusconradti* Kolbe, 1897, by original designation.

Family-group name: COSMOVALGINAE Kolbe, 1904. Stem: *Cosmovalg*-.

***Cosnania* Dejean, 1821**: 2. Current status: valid subgenus of *Colliuris* DeGeer, 1774 in CARABIDAE: HARPALINAE: ODACANTHINI. Note: this name was used in the literature for a long time under the spelling *Casnonia* and attributed to Latreille in Latreille and Dejean (1824: 130) or Dejean (1825: 170) but the name was first made available by Dejean (1821) under the spelling *Cosnania*, which is currently in prevailing usage; this name is a junior objective synonym of *Macrotrachelus* Latreille, 1818 (see Bousquet 2002: 30), which was not used as valid after 1899 to our knowledge; however, we are unable to provide 25 references to conserve usage of *Cosnania* (or the variant spelling *Casnonia*) as the valid name (ICZN 1999a, Article 23.9.1.2); nevertheless we believe that *Cosnania* Dejean, 1821 should be retained as the valid name and the case submitted to the Commission for a ruling.

Type species: *Attelabuspensylvanicus* Linnaeus, 1758, by monotypy.

Family-group name: CASNONIAE J.L. LeConte, 1861. Stem: *Cosnani*-.

***Cossonus* Clairville, 1798**: 57, 58. Current status: valid genus in CURCULIONIDAE: COSSONINAE: COSSONINI.

Type species: *Curculiolinearis* Fabricius, 1775, by subsequent designation (Latreille 1810: 431).

Family-group name: COSSONIDES Schönherr, 1825. Stem: *Cosson*-.

***Cossyphodes* Westwood, 1851**: 168. Current status: valid genus in TENEBRIONIDAE: PIMELIINAE: COSSYPHODINI: COSSYPHODINA.

Type species: *Cossyphodeswollastonii* Westwood, 1851, by monotypy.

Family-group name: COSSYPHODIDAE Wasmann, 1899. Stem: *Cossyphod*-.

***Cossyphodites* Brauns, 1901**: 91. Current status: valid genus in TENEBRIONIDAE: PIMELIINAE: COSSYPHODINI: COSSYPHODITINA.

Type species: *Cossyphodeswoodrooffei* Péringuey, 1885, by monotypy.

Family-group name: COSSYPHODITINAE Basilewsky, 1950. Stem: *Cossyphodit*-.

***Cossyphus* G.-A. Olivier, 1791**: 121. Current status: valid genus in TENEBRIONIDAE: LAGRIINAE: COSSYPHINI.

Type species: *Lampyrisdepressa* Fabricius, 1781, by monotypy.

Family-group name: COSSYPHORES Latreille, 1802. Stem: *Cossyph*-. Note: this family-group name is a junior homonym of COSSYPHINA Vigors, 1825 (type genus *Cossypha* Vigors, 1825) in Aves.

***Cotaster* Motschulsky, 1851**: 425. Current status: valid genus in CURCULIONIDAE: COSSONINAE: DRYOTRIBINI.

Type species: *Phloeophagusuncipes* Boheman, 1838, by subsequent designation (Lacordaire 1865: 330).

Family-group name: COTASTERINEN Faust, 1886. Stem: *Cotaster*-.

***Cotasteromimus* Chûjô & Voss, 1960**: 10. Current status: valid genus in CURCULIONIDAE: MOLYTINAE: PISSODINI: COTASTEROMIMINA.

Type species: *Cotasteromimusmorimotoi* Chûjô & Voss, 1960, by original designation.

Family-group name: COTASTEROMIMINI Morimoto, 1962. Stem: *Cotasteromim*-.

***Cotasterosoma* Konishi, 1962**: 3. Current status: valid genus in CURCULIONIDAE: COSSONINAE: RHYNCOLINI: RHYNCOLINA.

Type species: *Cotasterosomaomogoense* Konishi, 1962, by original designation.

Family-group name: COTASTEROSOMINI Konishi, 1962. Stem: *Cotasterosomat*-.

***Coxelus* Dejean, 1821**: 67. Current status: valid genus in ZOPHERIDAE: COLYDIINAE: SYNCHITINI.

Type species: *Bolitophaguspictus* Sturm, 1807, by monotypy.

Family-group name: COXELINI Seidlitz, 1872. Stem: *Coxel*-.

***Craniotus* J.L. LeConte, 1851a**: 142. Current status: valid genus in TENEBRIONIDAE: PIMELIINAE: ASIDINI.

Type species: *Craniotuspubescens* J.L. LeConte, 1851, by monotypy.

Family-group name: CRANIOTINI J.L. LeConte & G.H. Horn, 1883. Stem: *Craniot*-.

***Cranophorus* Mulsant, 1850**: 940. Current status: valid genus in COCCINELLIDAE: COCCINELLINAE: COCCIDULINI.

Type species: *Cranophorusquadrinotatus* Mulsant, 1850, by subsequent designation (Crotch 1874a: 293).

Family-group name: CRANOPHORAIRES Mulsant, 1850. Stem: *Cranophor*-.

***Cranopoeus* G.A.K. Marshall, 1931**: 274. Current status: valid genus in CURCULIONIDAE: CURCULIONINAE: CRANOPOEINI.

Type species: *Cranopoeusturritus* G.A.K. Marshall, 1931, by original designation.

Family-group name: CRANOPOEINI Kuschel, 2009. Stem: *Cranopoe*-.

***Craspedomerus* Bernhauer, 1911**: 88. Current status: valid genus in STAPHYLINIDAE: STAPHYLININAE: STAPHYLININI: PHILONTHINA.

Type species: *Philonthusglenoides* Schubert, 1908, by monotypy.

Family-group name: CRASPEDOMERI Bernhauer, 1911. Stem: *Craspedomer*-.

***Cratacanthus* Dejean, 1829**: 4, 40. Current status: valid genus in CARABIDAE: HARPALINAE: HARPALINI: HARPALINA.

Type species: *Cratacanthuspensylvanicus* Dejean, 1829 (= *Harpalusdubius* Palisot de Beauvois, 1812), by monotypy.

Family-group name: CRATACANTHI Lindroth, 1968. Stem: *Cratacanth*-.

***Cratocara* J.L. LeConte, 1863a**: 11. Current status: junior synonym of *Polpochila* Solier, 1849 in CARABIDAE: HARPALINAE: HARPALINI: STENOLOPHINA. Note: new replacement name for *Melanotus* Dejean, 1831.

Type species: *Melanotusflavipes* Dejean, 1831, by subsequent designation (Hope 1838a: 111) (type fixation for *Melanotus* Dejean).

Family-group name: CRATOCARINI Casey, 1914. Stem: *Cratocar*-.

***Cratocerus* Dejean, 1829**: 3, 12. Current status: valid genus in CARABIDAE: HARPALINAE: CRATOCERINI.

Type species: *Cratocerusmonilicornis* Dejean, 1829, by monotypy.

Family-group name: CRATOCÉRIDES Lacordaire, 1854. Stem: *Cratocer*-.

***Cratogaster* Blanchard, 1843**: pl. 2. Current status: valid genus in CARABIDAE: HARPALINAE: PTEROSTICHINI: PTEROSTICHINA.

Type species: *Cratogastersulcatus* Blanchard, 1843, by monotypy.

Family-group name: CRATOGASTRI Csiki, 1929. Stem: *Cratogastr*-.

***Cratognathus* Dejean, 1829**: 4, 46. Current status: valid genus in CARABIDAE: HARPALINAE: HARPALINI: HARPALINA.

Type species: *Cratognathusmandibularis* Dejean, 1829, by monotypy.

Family-group name: CRATOGNATHIDAE Laporte, 1834. Stem: *Cratognath*-.

***Cratomorphus* Motschulsky, 1853a**: 35. Current status: valid genus in LAMPYRIDAE: LAMPYRINAE: CRATOMORPHINI.

Type species: *Photinusfabricii* Laporte, 1840, by original designation.

Family-group name: CRATOMORPHINAE E. Olivier, 1911. Stem: *Cratomorph*-.

***Cratonychus* Dejean, 1833a**: 87. Current status: junior synonym of *Melanotus* Eschscholtz, 1829 in ELATERIDAE: ELATERINAE: MELANOTINI. Note: unnecessary new replacement name for *Melanotus* Eschscholtz, 1829.

Type species: *Elaterfulvipes* Herbst, 1806 (= *Elatervillosus* Geoffroy, 1785), by subsequent designation (Westwood 1838c: 26) (type fixation for *Melanotus* Eschscholtz).

Family-group name: CRATONYCHIDAE Gistel, 1848. Stem: *Cratonych*-.

***Cratoparis* Dejean, 1834**: 235. Current status: junior synonym of *Euparius* Schönherr, 1823 in ANTHRIBIDAE: ANTHRIBINAE: CRATOPARINI. Note: unnecessary new replacement name for *Euparius* Schönherr, 1823.

Type species: *Anthribuslunatus* Fabricius, 1801 (= *Macrocephalusmarmoreus* G.-A. Olivier, 1800), by monotypy (type fixation for *Euparius* Schönherr).

Family-group name: CRATOPARES J.L. LeConte, 1876. Stem: *Cratopar*-.

***Cratopus* Schönherr, 1823**: 1140. Current status: valid genus in CURCULIONIDAE: ENTIMINAE: POLYDRUSITAE: CRATOPODINI.

Type species: *Curculiomelanocephalus* Fabricius, 1798, by original designation.

Family-group name: CRATOPINI Hustache, 1919. Stem: *Cratopod*-.

***Cremastocheilus* Knoch, 1801**: 115. Current status: valid genus in SCARABAEIDAE: CETONIINAE: CREMASTOCHEILINI: CREMASTOCHEILINA.

Type species: *Cremastocheiluscastaneae* Knoch, 1801, by monotypy.

Family-group name: CREMASTOCHILIDAE Burmeister & Schaum, 1841. Stem: *Cremastocheil*-.

***Crematoxenus* Mann, 1921**: 547. Current status: valid genus in STAPHYLINIDAE: ALEOCHARINAE: CREMATOXENINI.

Type species: *Crematoxenusaenigma* Mann, 1921, by original designation.

Family-group name: CREMATOXENINI Mann, 1921. Stem: *Crematoxen*-.

***Creobius* Guérin-Méneville, 1839a**: pl. 225 (p. 4). Current status: valid genus in CARABIDAE: BROSCINAE: BROSCINI: CREOBIINA.

Type species: *Feroniaeydouxii* Guérin-Méneville, 1839, by monotypy.

Family-group name: CREOBITAE Jeannel, 1941. Stem: *Creobi*-.

***Creophilus* Leach in Samouelle, 1819**: 172. Current status: valid genus in STAPHYLINIDAE: STAPHYLININAE: STAPHYLININI: STAPHYLININA. Note: name placed on the Official List of Generic Names in Zoology (ICZN 1959a).

Type species: *Staphylinusmaxillosus* Linnaeus, 1758, by original designation (see ICZN 1959a). Note: name placed on the Official List of Specific Names in Zoology (ICZN 1959a).

Family-group name: CREOPHILIDAE W. Kirby, 1837. Stem: *Creophil*-.

***Crepidodera* Chevrolat in Dejean, 1836a**: 391. Current status: valid genus in CHRYSOMELIDAE: GALERUCINAE: ALTICINI.

Type species: *Chrysomelanitidula* Linnaeus, 1758, by subsequent designation (Maulik 1926: 234).

Family-group name: CRÉPIDODÉRITES Chapuis, 1875. Stem: *Crepidoder*-.

***Crepidogaster* Boheman, 1848**: 68. Current status: valid genus in CARABIDAE: BRACHININAE: CREPIDOGASTRINI.

Type species: *Crepidogasterbimaculatus* Boheman, 1848, by monotypy.

Family-group name: CREPIDOGASTRITAE Jeannel, 1949. Stem: *Crepidogastr*-.

***Crepidomenus* Erichson, 1842**: 140. Current status: valid genus in ELATERIDAE: DENDROMETRINAE: CREPIDOMENINI.

Type species: *Crepidomenusfulgidus* Erichson, 1842, by subsequent designation (Hyslop 1921: 637).

Family-group name: CRÉPIDOMÉNITES Candèze, 1863. Stem: *Crepidomen*-.

***Crepidotus* Schönherr, 1838**: 859. Current status: valid genus in CURCULIONIDAE: DRYOPHTHORINAE: LITOSOMINI: CREPIDOTINA.

Type species: *Crepidotusaudouini* Gyllenhal, 1838 (= *Calandravariolosa* Klug, 1833), by original designation.

Family-group name: CREPIDOTINA Legalov, 2020a: 323. Stem: *Crepidot*-.

†***Cretakarenni* Peris & Delclòs, 2015**: 335. Current status: valid genus in MONOTOMIDAE: MONOTOMINAE: †CRETAKARENNIINI. Note: Cretaceous (Myanmar; Spain).

Type species: †*Cretakarennibirmanicus* Peris & Delclòs, 2015, by original designation.

Family-group name: †CRETAKARENNIINI Peris & Delclòs, 2015: 335. Stem: *Cretakarenni*-.

†***Cretanthribus* Legalov, 2009a**: 292. Current status: valid genus in ANTHRIBIDAE: ANTHRIBINAE: †CRETANTHRIBINI. Note: Lower Cretaceous (Russia).

Type species: †*Anthribitescretaceus* Zherikhin, 1993, by original designation.

Family-group name: †CRETANTHRIBINI Legalov, 2009. Stem: *Cretanthrib*-.

†***Cretochodaeus* Nikolajev, 1995a**: 79. Current status: valid genus in OCHODAEIDAE: †CRETOCHODAEINAE. Note: Lower Cretaceous (Mongolia).

Type species: †*Cretochodaeusmongolicus* Nikolajev, 1995, by original designation.

Family-group name: †CRETOCHODAEINI Nikolajev, 1995. Stem: *Cretochodae*-.

†***Cretochoragus* Soriano, Gratshev & Delclòs, 2006**: 557. Current status: valid genus in ANTHRIBIDAE: CHORAGINAE: †CRETOCHORAGINI. Note: Lower Cretaceous (Spain).

Type species: †*Cretochoraguspygmaeus* Soriano, Gratshev & Delclòs, 2006, by original designation.

Family-group name: †CRETOCHORAGINI Legalov, 2016: 1460. Stem: *Cretochorag*-.

†***Cretocoma* Nikolajev, 2002**: 54. Current status: valid genus in PLEOCOMIDAE: †CRETOCOMINAE. Note: Lower Cretaceous (Mongolia).

Type species: †*Cretocomatologoica* Nikolajev, 2002, by original designation.

Family-group name: †CRETOCOMINI Nikolajev, 2002. Stem: *Cretocom*-.

†***Cretodermestes* Deng, Ślipiński, Ren & H. Pang, 2017a**: 3. Current status: valid genus in DERMESTIDAE: ATTAGENINAE: †CRETODERMESTINI. Note: mid-Cretaceous (Myanmar).

Type species: †*Cretodermestespalpalis* Deng, Ślipiński, Ren & H. Pang, 2017, by original designation.

Family-group name: †CRETODERMESTINI Deng, Ślipiński, Ren & H. Pang, 2017: 3. Stem: *Cretodermest*-.

†***Cretogeotrupes* Nikolajev, 1992**: 80. Current status: valid genus in GEOTRUPIDAE: GEOTRUPINAE: †CRETOGEOTRUPINI. Note: Lower Cretaceous (China; Russia).

Type species: †*Cretogeotrupesconvexus* Nikolajev, 1992, by original designation.

Family-group name: †CRETOGEOTRUPINI Nikolajev, 1996. Stem: *Cretogeotrup*-.

†***Cretoglaphyrus* Nikolajev, 2005a**: 70. Current status: valid genus in GLAPHYRIDAE: †CRETOGLAPHYRINAE. Note: Lower Cretaceous (Transbaikalia).

Type species: †*Cretoglaphyrusrohdendorfi* Nikolajev, 2005, by original designation.

Family-group name: †CRETOGLAPHYRINI Nikolajev, 2005. Stem: *Cretoglaphyr*-.

†***Cretohister* Zhou, Caterino, Ślipiński & Cai, 2018**: 722. Current status: valid genus in †CRETOHISTERIDAE. Note: Lower Cretaceous (China).

Type species: †*Cretohistersinensis* Zhou, Caterino, Ślipiński & Cai, 2018, by original designation.

Family-group name: †CRETOHISTERIDAE Zhou, Caterino, Ślipiński & Cai, 2018: 721. Stem: *Cretohister*-.

†***Cretolycus* Tihelka, D. Huang & Cai, 2019a**: 263. Current status: valid genus in LYCIDAE: DEXORINAE: †CRETOLYCINI. Note: mid-Cretaceous (Myanmar).

Type species: †*Cretolycuspraecursor* Tihelka, D. Huang & Cai, 2019, by original designation.

Family-group name: †CRETOLYCINI Tihelka, D. Huang & Cai, 2019: 263. Stem: *Cretolyc*-.

†***Cretomelolontha* Nikolajev, 1998**: 81. Current status: valid genus in SCARABAEIDAE: MELOLONTHINAE: †CRETOMELOLONTHINI. Note: Lower Cretaceous (Russia).

Type species: †*Cretomelolonthatransbaikalica* Nikolajev, 1998, by original designation.

Family-group name: †CRETOMELOLONTHINI Nikolajev, 1998. Stem: *Cretomelolonth*-.

†***Cretonemonyx* Gratshev & Legalov, 2009**: 412. Current status: valid genus in NEMONYCHIDAE: †CRETONEMONYCHINAE: †CRETONEMONYCHINI. Note: Lower Cretaceous (Russia).

Type species: †*Cretonemonyxprofligatus* Gratshev & Legalov, 2009, by original designation.

Family-group name: †CRETONEMONYCHINAE Gratshev & Legalov, 2009. Stem: *Cretonemonych*-.

†***Cretonodes* Kirejtshuk & Azar in Kirejtshuk et al., 2009a**: 121. Current status: valid genus in DERMESTIDAE: TRINODINAE: †CRETONODINI. Note: Lower Cretaceous (Lebanon).

Type species: †*Cretonodesantounazari* Kirejtshuk & Azar, 2009, by original designation.

Family-group name: †CRETONODINI Kirejtshuk & Azar, 2009. Stem: *Cretonod*-.

†***Cretoparacucujus* Cai & Escalona in Cai et al., 2018**: 2806. Current status: valid genus in †PARANDREXIDAE: †CRETOPARACUCUJINAE. Note: mid-Cretaceous (Myanmar).

Type species: †*Cretoparacucujuscycadophilus* Cai & Escalona, 2018, by original designation. Note: combined description of a new genus and a single new species (ICZN 1999a, Article 13.4).

Family-group name: †CRETOPARACUCUJINAE Kirejtshuk in Kirejtshuk et al., 2023a: 24. Stem: *Cretoparacucu*-.

†***Cretophengodes* Y.-D. Li, Kundrata, Tihelka & Cai in Y.-D. Li et al., 2021**: 2. Current status: valid genus in †CRETOPHENGODIDAE. Note: mid-Cretaceous (Myanmar).

Type species: †*Cretophengodesazari* Y.-D. Li, Kundrata, Tihelka & Cai, 2021, by original designation.

Family-group name: †CRETOPHENGODIDAE Y.-D. Li, Kundrata, Tihelka & Cai in Y.-D. Li et al., 2021: 2. Stem: *Cretophengod*-.

†***Cretoscarabaeus* Nikolajev, 1995b**: 147. Current status: valid genus in SCARABAEIDAE: †CRETOSCARABAEINAE. Note: Lower Cretaceous (Russia).

Type species: †*Cretoscarabaeusgibbosus* Nikolajev, 1995, by original designation.

Family-group name: †CRETOSCARABAEINAE Nikolajev, 1995. Stem: *Cretoscarabae*-.

†***Cretulio* Zherikhin, 1993**: 33. Current status: valid genus in CARIDAE: CHILECARINAE: †CRETULIINI. Note: Lower Cretaceous (Russia).

Type species: †*Cretulionucula* Zherikhin, 1993, by original designation.

Family-group name: †CRETULIINI Legalov, 2009. Stem: *Cretuli*-.

***Crinotarsus* Blanchard, 1846**: pl. 16. Current status: valid genus in CERAMBYCIDAE: LAMIINAE: DESMIPHORINI.

Type species: *Crinotarsusplagiatus* Blanchard, 1846, by monotypy.

Family-group name: CRINOTARSIDES Lacordaire, 1872. Stem: *Crinotars*-.

***Criocephalum* Dejean, 1835**: 328. Current status: junior synonym of *Arhopalus* Audinet-Serville, 1834 in CERAMBYCIDAE: SPONDYLIDINAE: ASEMINI.

Type species: *Cerambyxrusticus* Linnaeus, 1758, by monotypy.

Family-group name: CRIOCEPHALINAE Perrier, 1893. Stem: *Criocephal*-.

***Crioceris* Geoffroy, 1762**: 237. Current status: valid genus in CHRYSOMELIDAE: CRIOCERINAE: CRIOCERINI. Note: name placed on the Official List of Generic Names in Zoology as *Crioceris* O.F. Müller, 1764 (ICZN 1970a); however, the author of this name was subsequently changed to Geoffroy, 1762 (ICZN 1994a).

Type species: *Chrysomelaasparagi* Linnaeus, 1758, by plenary power (see ICZN 1970a). Note: name placed on the Official List of Specific Names in Zoology (ICZN 1970a, 1994a).

Family-group name: CRIOCERIDES Latreille, 1804. Stem: *Criocer*-.

***Criomorphus* Mulsant, 1839**: 58. Current status: junior synonym of *Tetropium* W. Kirby, 1837 in CERAMBYCIDAE: SPONDYLIDINAE: TETROPIINI. Note: junior homonym of *Criomorphus* Curtis, 1831 [Hemiptera].

Type species: *Callidiumaulicum* Fabricius, 1775 (= *Cerambyxcastaneus* Linnaeus, 1758), by monotypy.

Family-group name: CRIOMORPHINI Portevin, 1927. Stem: *Criomorph*-. Note: family-group name permanently invalid (ICZN 1999a, Article 39).

***Crossotarsus* Chapuis, 1865**: 22, 44. Current status: valid genus in CURCULIONIDAE: PLATYPODINAE: PLATYPODINI.

Type species: *Platypuswallacei* J. Thomson, 1858, by subsequent designation (Hopkins 1914: 119).

Family-group name: CROSSOTARSARIAE H. Strohmeyer, 1914. Stem: *Crossotars*-.

***Crossotus* Audinet-Serville, 1835a**: 52. Current status: valid genus in CERAMBYCIDAE: LAMIINAE: CEROPLESINI: CROSSOTINA.

Type species: *Crossotusplumicornis* Audinet-Serville, 1835, by monotypy.

Family-group name: CROSSOTITAE J. Thomson, 1864. Stem: *Crossot*-.

***Crowsoniella* Pace, 1975**: 446. Current status: valid genus in CROWSONIELLIDAE.

Type species: *Crowsoniellarelicta* Pace, 1975, by original designation.

Family-group name: CROWSONIELLIDAE Iablokoff-Khnzorian, 1983. Stem: *Crowsoniell*-.

***Cryphalus* Erichson, 1836**: 61. Current status: valid genus in CURCULIONIDAE: SCOLYTINAE: CRYPHALINI.

Type species [suggested]: *Bostrichusasperatus* Gyllenhal, 1813, by subsequent designation (C.G. Thomson 1859: 146). Note: the valid type species fixation is that of Laporte (1840b: 370), who selected *Apatetiliae* Panzer, 1793, but that species is the type species of the genus *Ernoporus* C.G. Thomson, 1859; an application to the Commission is necessary to fix *Bostrichusasperatus* Gyllenhal, 1813 as the type species of *Cryphalus* Erichson, 1836.

Family-group name: CRYPHALOIDEAE Lindemann, 1876. Stem: *Cryphal*-.

***Cryptafricus* Leschen, 1996**: 624. Current status: valid genus in CRYPTOPHAGIDAE: ATOMARIINAE: CRYPTAFRICINI.

Type species: *Cryptafricusleleupi* Leschen, 1996, by original designation.

Family-group name: CRYPTAFRICINI Leschen, 1996. Stem: *Cryptafric*-.

***Cryptamorpha* Wollaston, 1854**: 156. Current status: valid genus in SILVANIDAE: BRONTINAE: TELEPHANINI.

Type species: *Cryptomorphamusae* Wollaston, 1854 (= *Psammoecusdesjardinsii* Guérin- Méneville, 1844), by monotypy.

Family-group name: CRYPTAMORPHINI Casey, 1884. Stem: *Cryptamorph*-.

***Cryptarcha* Shuckard, 1839**: 165. Current status: valid genus in NITIDULIDAE: CRYPTARCHINAE: CRYPTARCHINI.

Type species: *Nitidulastrigata* Fabricius, 1787, by subsequent designation (Hope 1841a: 154).

Family-group name: CRYPTARCHINA C.G. Thomson, 1859. Stem: *Cryptarch*-.

***Crypticus* Latreille, 1816**: 298. Current status: valid genus in TENEBRIONIDAE: DIAPERINAE: CRYPTICINI.

Type species: *Helopsglaber* Fabricius, 1775 (= *Tenebrioquisquilius* Linnaeus, 1761), by monotypy.

Family-group name: CRYPTICITES Brullé, 1832. Stem: *Cryptic*-.

***Cryptobium* Mannerheim, 1830**: 38. Current status: junior synonym of *Ochthephilum* Stephens, 1829 in STAPHYLINIDAE: PAEDERINAE: PAEDERINI: CRYPTOBIINA.

Type species: *Paederusfracticornis* Paykull, 1800, by monotypy.

Family-group name: CRYPTOBIA Casey, 1905. Stem: *Cryptobi*-. Note: senior homonym of CRYPTOBIIDAE Poche, 1913 (type genus *Cryptobia* Leidy, 1846) in Kinetoplastida.

†***Cryptocardius* Dolin, 1980**: 74. Current status: valid genus in ELATERIDAE: AGRYPNINAE: †CRYPTOCARDIINI. Note: Jurassic (Kazakhstan).

Type species: †*Cryptocardiusmirabilis* Dolin, 1980, by original designation.

Family-group name: †CRYPTOCARDIINI Dolin, 1980. Stem: *Cryptocardi*-.

***Cryptocephalus* Geoffroy, 1762**: 231. Current status: valid genus in CHRYSOMELIDAE: CRYPTOCEPHALINAE: CRYPTOCEPHALINI: CRYPTOCEPHALINA. Note: name placed on the Official List of Generic Names in Zoology (ICZN 1994a).

Type species: *Chrysomelasericea* Linnaeus, 1758, by subsequent designation (Latreille 1810: 432; see ICZN 1994a). Note: name placed on the Official List of Specific Names in Zoology (ICZN 1994a).

Family-group name: CRYPTOCEPHALOIDEAE Gyllenhal, 1813. Stem: *Cryptocephal*-.

***Cryptochile* Latreille, 1828a**: 576. Current status: valid genus in TENEBRIONIDAE: PIMELIINAE: CRYPTOCHILINI: CRYPTOCHILINA. Note: the older but unusued name *Phymatium* Billberg, 1820 has priority over *Cryptochile* Latreille, 1828; an application to the Commission is needed to conserve usage of the well-established name *Cryptochile* (see Bouchard et al. 2021: 152).

Type species: *Pimeliamaculata* Fabricius, 1781, by monotypy.

Family-group name: CRYPTOCHILITES Solier, 1841. Stem: *Cryptochil*-. Note: senior homonym of CRYPTOCHILIDAE Berger, 1979 (type genus *Cryptochilum* Maupas, 1883) in Ciliophora.

***Cryptodacne* Sharp, 1878c**: 82. Current status: valid genus in EROTYLIDAE: EROTYLINAE: DACNINI.

Type species: *Cryptodacnesynthetica* Sharp, 1878, by monotypy.

Family-group name: CRYPTODACNINI Sen Gupta, 1970. Stem: *Cryptodacn*-.

***Cryptoderma* Ritsema, 1885**: 54. Current status: valid genus in CURCULIONIDAE: DRYOPHTHORINAE: CRYPTODERMATINI. Note: new replacement name for *Oxyrhynchus* Schönherr, 1826; *Nosoxylon* Gistel, 1848 is an older name for *Cryptoderma* Ritsema, 1885 and an application to the Commission or usage of Reversal of Precedence (ICZN 1999a, Article 23.9) is necessary to retain Ritsema’s name as valid; Alonso-Zarazaga and Lyal (1999: 63) mentioned that they would submit an application to the Commission to suppress the older name *Nosoxylon* Gistel, 1848 but this has not taken place to our knowledge.

Type species: *Calandradiscors* Fabricius, 1801, by original designation (type fixation for *Oxyrhynchus* Schönherr).

Family-group name: CRYPTODERMINAE Bovie, 1908. Stem: *Cryptodermat*-.

***Cryptodontes* Burmeister, 1847**: 292. Current status: valid genus in SCARABAEIDAE: CETONIINAE: CRYPTODONTINI.

Type species: *Leptognathuslatreillianus* Westwood, 1841, by monotypy.

Family-group name: CRYPTODONTIDES Lacordaire, 1855. Stem: *Cryptodont*-. Note: senior homonym of CRYPTODONTIDAE Dall, 1895 (type genus *Cryptodon* Turton, 1822) in Mollusca: Bivalvia.

***Cryptodus* W.S. MacLeay, 1819**: 138. Current status: valid genus in SCARABAEIDAE: DYNASTINAE: PHILEURINI: CRYPTODINA.

Type species: *Cryptodusparadoxus* W.S. MacLeay, 1819, by monotypy.

Family-group name: CRYPTODINAE Burmeister & Schaum, 1840. Stem: *Cryptod*-.

***Cryptogenius* Westwood, 1842a**: 457. Current status: valid genus in HYBOSORIDAE: ANAIDINAE. Note: see additional information on the date of publication and page number for the establishment of this genus-group name in the entry for Westwood (1842a) in the References section, which differs slightly from data recently provided in Costa-Silva and Vaz-de-Mello (2023: 178).

Type species: *Cryptogeniusmiersianus* Westwood, 1842, by monotypy.

Family-group name: CRYPTOGENIINI Howden, 2001. Stem: *Cryptogeni*-.

***Cryptoglossa* Solier, 1837**: 638, 680. Current status: valid genus in TENEBRIONIDAE: PIMELIINAE: CRYPTOGLOSSINI.

Type species: *Cryptoglossabicostata* Solier, 1837, by monotypy.

Family-group name: CRYPTOGLOSSINI J.L. LeConte, 1862. Stem: *Cryptogloss*-.

***Cryptognatha* Mulsant, 1850**: 497. Current status: valid genus in COCCINELLIDAE: COCCINELLINAE: CRYPTOGNATHINI.

Type species: *Cryptognathaauriculata* Mulsant, 1850, by subsequent designation (Crotch 1874a: 206).

Family-group name: CRYPTOGNATHAIRES Mulsant, 1850. Stem: *Cryptognath*-. Note: senior homonym of CRYPTOGNATHIDAE Oudemans, 1902 (type genus *Cryptognathus* Kramer, 1879) in Acari: Trombidiformes.

***Cryptohypnus* Eschscholtz, 1830b**: 17. Current status: junior synonym of *Hypnoidus* Dillwyn, 1829 in ELATERIDAE: HYPNOIDINAE.

Type species: *Elaterriparius* Fabricius, 1792, by subsequent designation (Duponchel 1844a: 432).

Family-group name: CRYPTOHYPNITES Candèze, 1860. Stem: *Cryptohypn*-.

***Cryptolarynx* Schalkwyk, 1966**: 745. Current status: valid genus in CURCULIONIDAE: BRACHYCERINAE: CRYPTOLARYNGINI. Note: new replacement name for *Cryptopharynx* G.A.K. Marshall, 1957.

Type species: *Cryptopharynxvitis* G.A.K. Marshall, 1957, by original designation (type fixation for *Cryptopharynx* G.A.K. Marshall).

Family-group name: CRYPTOLARYNGINAE Schalkwyk, 1966. Stem: *Cryptolaryng*-.

***Cryptomera* Broun, 1893b**: 1358. Current status: junior synonym of *Eucinetus* Germar, 1818 in EUCINETIDAE. Note: junior homonym of *Cryptomera* Rafinesque 1820 [Chilopoda].

Type species: *Cryptomeranigra* Broun, 1893, by monotypy.

Family-group name: CRYPTOMERIDAE Broun, 1893. Stem: *Cryptomer*-. Note: family-group name permanently invalid (ICZN 1999a, Article 39).

***Cryptommata* Wollaston, 1877**: 156. Current status: valid genus in CURCULIONIDAE: COSSONINAE: CRYPTOMMATINI.

Type species: *Cryptommatacucullata* Wollaston, 1877, by monotypy.

Family-group name: CRYPTOMMATINI Voss, 1972. Stem: *Cryptommat*-.

***Cryptonotopsis* Pace, 2003**: 39. Current status: valid genus in STAPHYLINIDAE: ALEOCHARINAE: CRYPTONOTOPSISINI.

Type species: *Cryptonotopsisrougemonti* Pace, 2003, by original designation.

Family-group name: CRYPTONOTOPSISINI Pace, 2003. Stem: *Cryptonotopsis*- (ICZN 1999a, Article 29.4).

***Cryptonychus* Gyllenhal in Schönherr, 1817**: 7. Current status: valid genus in CHRYSOMELIDAE: CASSIDINAE: CRYPTONYCHINI.

Type species: *Hispaporrecta* Gyllenhal, 1817, by monotypy.

Family-group name: CRYPTONYCHITES Chapuis, 1875. Stem: *Cryptonych*-.

***Cryptophagus* Herbst, 1792**: 172. Current status: valid genus in CRYPTOPHAGIDAE: CRYPTOPHAGINAE: CRYPTOPHAGINI. Note: the original spelling *Kryptophagus* was changed to *Cryptophagus* and *Cryptophagus* Herbst, 1792 placed on the Official List of Generic Names in Zoology (ICZN 1995c).

Type species: *Dermestescellaris* Scopoli, 1763, by subsequent designation (Westwood 1838c: 13; see ICZN 1995c). Note: name placed on the Official List of Specific Names in Zoology (ICZN 1995c).

Family-group name: CRYPTOPHAGIDAE W. Kirby, 1826. Stem: *Cryptophag*-.

***Cryptopharynx* G.A.K. Marshall, 1957**: 17. Current status: invalid senior synonym of *Cryptolarynx* Schalkwyk, 1966 in CURCULIONIDAE: BRACHYCERINAE: CRYPTOLARYNGINI. Note: junior homonym of *Cryptopharynx* Kahl, 1928 [Protozoa].

Type species: *Cryptopharynxvitis* G.A.K. Marshall, 1957, by original designation.

Family-group name: CRYPTOPHARYNGINAE G.A.K. Marshall, 1957. Stem: *Cryptopharyng*-. Note: family-group name permanently invalid (ICZN 1999a, Article 39).

***Cryptophilus* Reitter, 1875a**: 381. Current status: valid genus in EROTYLIDAE: CRYPTOPHILINAE: CRYPTOPHILINI.

Type species: fixed by Gimmel et al. (2019a: 529) according to Article 70.3.2 (ICZN 1999a) as *Typhaeaangusta* Rosenhauer, 1856, the taxonomic species involved in the misidentification of *Cryptophagusinteger* Heer, 1841 (sensu Reitter, 1875), as subsequently designated by Chûjô (1969: 277).

Family-group name: CRYPTOPHILINI Casey, 1900. Stem: *Cryptophil*-.

***Cryptoplus* Erichson, 1842**: 198. Current status: valid genus in CURCULIONIDAE: CURCULIONINAE: CRYPTOPLINI.

Type species: *Cryptoplusperdix* Erichson, 1842, by monotypy.

Family-group name: CRYPTOPLIDES Lacordaire, 1863. Stem: *Cryptopl*-.

***Cryptops* Wiedemann, 1823b**: 164. Current status: invalid senior synonym of *Byrsops* Germar, 1829 in CURCULIONIDAE: BRACHYCERINAE: BRACHYCERINI. Note: junior homonym of *Cryptops* Leach, 1814 [Chilopoda]; Alonso-Zarazaga and Lyall (1999: 62) consider the type genus to have been proposed independently also by Schönherr (1823: 1138) but we consider that the two names apply to the same taxon.

Type species: *Brachycerusquadratus* Wiedemann, 1821, by subsequent designation (Schönherr 1823: 1138).

Family-group name: CRYPTOPSIDES Schönherr, 1826. Stem: *Cryptop*-. Note: family-group name permanently invalid (ICZN 1999a, Article 39).

***Cryptorhynchidius* Pierce, 1919**: 25. Current status: junior synonym of *Cryptorhynchus* Illiger, 1807 in CURCULIONIDAE: CRYPTORHYNCHINAE: CRYPTORHYNCHINI: CRYPTORHYNCHINA.

Type species: *Curculiolapathi* Linnaeus, 1758, by monotypy.

Family-group name: CRYPTORHYNCHIDIINI Bradley, 1930. Stem: *Cryptorhynchidi*-.

***Cryptorhynchus* Illiger, 1807b**: 330. Current status: valid genus in CURCULIONIDAE: CRYPTORHYNCHINAE: CRYPTORHYNCHINI: CRYPTORHYNCHINA. Note: name placed on the Official List of Generic Names in Zoology (ICZN 1967b).

Type species: *Curculiolapathi* Linnaeus, 1758, by plenary power (see ICZN 1967b). Note: name placed on the Official List of Specific Names in Zoology (ICZN 1967b).

Family-group name: CRYPTORHYNCHIDES Schönherr, 1825. Stem: *Cryptorhynch*-.

***Cryptosomatula* Bruce, 1940**: 683. Current status: valid genus in CRYPTOPHAGIDAE: CRYPTOPHAGINAE: PICROTINI.

Type species: *Cryptosomatulalongicornis* Bruce, 1940, by monotypy.

Family-group name: CRYPTOSOMATULINI Leschen, 1996. Stem: *Cryptosomatul*-.

***Cryptostoma* Dejean, 1821**: 34. Current status: invalid senior synonym of *Ceratogonys* Perty, 1830 in EUCNEMIDAE: MACRAULACINAE: ORODOTINI. Note: junior homonym of *Cryptostoma* de Blainville, 1818 [Mollusca].

Type species: *Elaterspinicornis* Fabricius, 1801, by monotypy.

Family-group name: CRYPTOSTOMIDAE Laporte, 1835. Stem: *Cryptostomat*-. Note: family-group name permanently invalid (ICZN 1999a, Article 39).

***Crypturgus* Erichson, 1836**: 60. Current status: valid genus in CURCULIONIDAE: SCOLYTINAE: CRYPTURGINI.

Type species: *Bostrichuscinereus* Herbst, 1794, by subsequent designation (Laporte 1840b: 370). Note: the type species usually listed for this genus is *Bostrichuspusillus* Gyllenhal, 1813 (e.g., Alonso-Zarazaga and Lyal 2009: 48; Knížek 2011: 227) by subsequent designation of C.G. Thomson (1859: 147) but Laporte’s designation is earlier; both species are currently included in the genus *Crypturgus* Erichson, 1836.

Family-group name: CRYPTURGI J.L. LeConte, 1876. Stem: *Crypturg*-.

***Ctenicera* Latreille, 1829a**: 454. Current status: valid genus in ELATERIDAE: DENDROMETRINAE: PROSTERNINI.

Type species: *Elaterpectinicornis* Linnaeus, 1758, by subsequent designation (Westwood 1838c: 26, as *Ctenicerus*).

Family-group name: CTENICERINA Jakobson, 1913. Stem: *Ctenicer*-.

***Ctenidia* Laporte, 1840b**: 264. Current status: valid genus in MORDELLIDAE: CTENIDIINAE.

Type species: *Ctenidiamordelloides* Laporte, 1840, by monotypy.

Family-group name: CTENIDIINAE Franciscolo, 1951. Stem: *Ctenidi*-.

***Cteniopus* Solier, 1835b**: 245, 246. Current status: valid genus in TENEBRIONIDAE: ALLECULINAE: CTENIOPODINI.

Type species: *Chrysomelasulphurea* Linnaeus, 1758, by subsequent designation (Westwood 1838c: 32).

Family-group name: CTÉNIOPITES Solier, 1835. Stem: *Cteniopod*-.

***Ctenistes* H.G.L. Reichenbach, 1816**: 75. Current status: valid genus in STAPHYLINIDAE: PSELAPHINAE: PSELAPHITAE: CTENISTINI.

Type species: *Ctenistespalpalis* H.G.L. Reichenbach, 1816, by monotypy.

Family-group name: CTÉNISTITES Blanchard, 1845. Stem: *Ctenist*-.

***Ctenodactyla* Dejean, 1825**: 169, 226. Current status: valid genus in CARABIDAE: HARPALINAE: CTENODACTYLINI.

Type species: *Ctenodactylachevrolatii* Dejean, 1825, by monotypy.

Family-group name: CTENODACTYLIDAE Laporte, 1834. Stem: *Ctenodactyl*-. Note: senior homonym of CTENODACTYLINA C. Girard, 1851 (type genus *Ctenodactylus* J.E. Gray, 1830) in Mammalia.

***Ctenophorus* Chapuis, 1869**: 49. Current status: junior synonym of *Scolytodes* Ferrari, 1867 in CURCULIONIDAE: SCOLYTINAE: HEXACOLINI. Note: junior homonym of *Ctenophorus* Fitzinger, 1843 [Reptilia].

Type species: *Ctenophoruslaevigatus* Chapuis, 1869 (= *Scolytodeschapuisi* Wood, 1977), by monotypy.

Family-group name: CTENOPHORIDAE Chapuis, 1869. Stem: *Ctenophor*-. Note: family-group name permanently invalid (ICZN 1999a, Article 39).

***Ctenoscelis* Audinet-Serville, 1832**: 134. Current status: valid genus in CERAMBYCIDAE: PRIONINAE: CALLIPOGONINI.

Type species: *Prionusater* G.-A. Olivier, 1795, by subsequent designation (J. Thomson 1864: 297).

Family-group name: CTENOSCELITAE J. Thomson, 1864. Stem: *Ctenoscelid*-.

***Ctenostoma* Klug, 1821**: 302. Current status: valid genus in CICINDELIDAE: CTENOSTOMATINI.

Type species: **here fixed** (under ICZN 1999a, Article 70.3.2) as *Ctenostomajekelii* Chevrolat, 1858, misidentified as *Collyrisformicaria* Fabricius, 1801 (sensu Klug, 1821) in the original designation by monotypy in Klug (1821: 302).

Family-group name: CTENOSTOMIDAE Laporte, 1834. Stem: *Ctenostomat*-.

***Ctesias* Stephens, 1830**: 124. Current status: valid genus in DERMESTIDAE: MEGATOMINAE: CTESIINI.

Type species: *Dermestesserra* Fabricius, 1792, by monotypy.

Family-group name: CTESIINI Rees, 1943. Stem: *Ctesi*-.

***Ctesibius* Champion, 1897**: 592. Current status: valid genus in ARTEMATOPODIDAE: ARTEMATOPODINAE: CTESIBIINI.

Type species: *Ctesibiuseumolpoides* Champion, 1897, by monotypy.

Family-group name: CTESIBIINAE Crowson, 1973. Stem: *Ctesibi*-.

***Cubispa* Barber, 1946**: 21. Current status: valid genus in CHRYSOMELIDAE: EUMOLPINAE: CUBISPINI.

Type species: *Cubispaturquino* Barber, 1946, by original designation.

Family-group name: CUBISPINI Monrós, 1954. Stem: *Cubisp*-.

***Cucujus* Fabricius, 1775**: 204. Current status: valid genus in CUCUJIDAE: CUCUJINAE. Note: the senior homonym *Cucujus* Geoffroy, 1762 (and all other uses of the name prior to the publication of *Cucujus* Fabricius, 1775) was suppressed for both Principles of Priority and Homonymy and placed on the Official Index of Rejected and Invalid Generic Names in Zoology while *Cucujus* Fabricius, 1775 was placed on the Official List of Generic Names in Zoology (ICZN 1994a).

Type species: *Cucujusdepressus* Fabricius, 1775 (= *Meloecinnaberinus* Scopoli, 1763), by monotypy (see ICZN 1994a). Note: *Meloecinnaberinus* Scopoli, 1763 was placed on the Official List of Specific Names in Zoology (ICZN 1994a).

Family-group name: CUCUJIPES Latreille, 1802. Stem: *Cucuj*-.

***Cuneipectus* Sloane, 1907**: 358. Current status: valid genus in CARABIDAE: HARPALINAE: CUNEIPECTINI.

Type species: *Cuneipectusfrenchi* Sloane, 1907, by monotypy.

Family-group name: CUNEIPECTINI Sloane, 1907. Stem: *Cuneipect*-.

***Cupes* Fabricius, 1801a**: xvii. Current status: valid genus in CUPEDIDAE: CUPEDINAE.

Type species: *Cupescapitata* Fabricius, 1801, by subsequent monotypy (Fabricius 1801b: 60).

Family-group name: CUPESIDAE Laporte, 1838. Stem: *Cuped*-.

***Curculio* Linnaeus, 1758**: 342, 377. Current status: valid genus in CURCULIONIDAE: CURCULIONINAE: CURCULIONINI: CURCULIONINA.

Type species: *Curculionucum* Linnaeus, 1758, by subsequent designation (Latreille 1810: 430).

Family-group name: CURCULIONITES Latreille, 1802. Stem: *Curculion*-.

†***Curculiopsis* Martynov, 1937**: 39. Current status: invalid senior synonym of *Rossocoleus* Rohdendorf, 1961 in †SCHIZOCOLEIDAE. Note: junior homonym of *Curculiopsis* Handlirsch, 1907 [Coleoptera: CURCULIONOIDEA]; Permian (Russia).

Type species: †*Curculiopsisellipticus* Martynov, 1937, by original designation.

Family-group name: †CURCULIOPSIDAE Martynov, 1937. Stem: *Curculiopse*-. Note: family- group name permanently invalid (ICZN 1999a, Article 39).

***Curis* Laporte & Gory, 1837**: 47. Current status: junior synonym of *Selagis* Dejean, 1836 in BUPRESTIDAE: BUPRESTINAE: CURIDINI: CURIDINA.

Type species: *Stigmoderaperoni* Laporte & Gory, 1837, by subsequent designation (Rye 1873: 271). Note: the type species usually recognized for this genus is *Buprestiscaloptera* Boisduval, 1835, by subsequent designation of Bellamy (1997a: 376) but Rye’s designation is earlier; both species are currently included in the genus *Curis*.

Family-group name: CURIDINA Holyński, 1988. Stem: *Curid*-.

***Curius* Newman, 1840d**: 17. Current status: valid genus in CERAMBYCIDAE: CERAMBYCINAE: CURIINI.

Type species: *Curiusdentatus* Newman, 1840, by monotypy.

Family-group name: CURII J.L. LeConte, 1873. Stem: *Curi*-.

***Curtimorda* Méquignon, 1946**: 58. Current status: valid genus in MORDELLIDAE: MORDELLINAE: CURTIMORDINI.

Type species: *Mordellabisignata* L. Redtenbacher, 1849, by original designation.

Family-group name: CURTIMORDINI Odnosum, 2010. Stem: *Curtimord*-.

***Curtos* Motschulsky, 1845b**: 36. Current status: valid genus in LAMPYRIDAE: LUCIOLINAE: CURTOSINI.

Type species: *Curtosmongolicus* Motschulsky, 1845, by monotypy.

Family-group name: CURTOSINI McDermott, 1964. Stem: *Curtos*-.

***Cussolenis* Fleutiaux, 1918a**: 59. Current status: valid genus in ELATERIDAE: THYLACOSTERNINAE. Note: new replacement name for *Soleniscus* Bonvouloir, 1871.

Type species: *Soleniscusmutabilis* Bonvouloir, 1875, by subsequent monotypy (de Bonvouloir 1875: 828; type fixation for *Soleniscus* Bonvouloir).

Family-group name: CUSSOLENITAE Cobos, 1961. Stem: *Cussolen*-.

***Cyathiger* King, 1865**: 174. Current status: junior synonym of *Plagiophorus* Motschulsky, 1851 in STAPHYLINIDAE: PSELAPHINAE: GONIACERITAE: CYATHIGERINI.

Type species: *Cyathigerpunctatus* King, 1865, by monotypy.

Family-group name: CYATHIGERINI L.W. Schaufuss, 1872. Stem: *Cyathiger*-.

***Cyathocerus* Sharp, 1882c**: 142. Current status: junior synonym of *Lepicerus* Motschulsky, 1855 in LEPICERIDAE.

Type species: *Cyathocerushorni* Sharp, 1882 (= *Lepicerusinaequalis* Motschulsky, 1855), by monotypy.

Family-group name: CYATHOCERIDAE Sharp, 1882. Stem: *Cyathocer*-.

***Cybebus* Schönherr, 1839**: 447. Current status: valid genus in BRENTIDAE: APIONINAE: CYBEBITAE.

Type species: *Cybebusrufipennis* Boheman, 1839 (= *Curculiodimidiatus* G.-A. Olivier, 1791), by original designation.

Family-group name: CYBÉBIDES Lacordaire, 1863. Stem: *Cybeb*-.

***Cybister* Curtis, 1827**: pl. 151. Current status: valid genus in DYTISCIDAE: CYBISTERINAE. Note: new replacement name for *Trogus* Leach, 1817.

Type species: *Dytiscuslateralis* Fabricius, 1798 (= *Dytiscustripunctatus* G.-A. Olivier, 1795), by monotypy (type fixation for *Trogus* Leach).

Family-group name: CYBISTRINI Sharp, 1880. Stem: *Cybister*-.

***Cybocephalus* Erichson, 1844**: 441. Current status: valid genus in NITIDULIDAE: CYBOCEPHALINAE.

Type species: *Anisotomaexigua* C.R. Sahlberg, 1833 (= *Nitidulapolita* Gyllenhal, 1813), by subsequent designation (E. Desmarest 1851: 278).

Family-group name: CYBOCÉPHALITES Jacquelin du Val, 1858. Stem: *Cybocephal*-.

***Cychramptodes* Reitter, 1878b**: 383. Current status: valid genus in NITIDULIDAE: NITIDULINAE: CYCHRAMPTODINI.

Type species: *Cychramptodesmurrayi* Reitter, 1878, by monotypy.

Family-group name: CYCHRAMPTODINI Kirejtshuk & Lawrence, 1992. Stem: *Cychramptod*-.

***Cychramus* Kugelann, 1794**: 543. Current status: valid genus in NITIDULIDAE: NITIDULINAE: CYCHRAMINI.

Type species: *Sphaeridiumluteum* Fabricius, 1787, by subsequent designation (Duponchel 1844a: 486). Note: the type species usually recognized for *Cychramus* Kugelann, 1794 is *Strongylusquadripunctatus* Herbst, 1792 (e.g., Jelínek and Audisio 2003: 161; Jelínek and Audisio 2007: 471) by subsequent designation of C.G. Thomson (1859: 69) but Duponchel’s designation is earlier; both *luteus* (Fabricius) and *quadripunctatus* (Herbst) are currently included in the genus *Cychramus*.

Family-group name: CYCHRAMIDAE Gistel, 1848. Stem: *Cychram*-.

***Cychrus* Fabricius, 1794**: 440. Current status: valid genus in CARABIDAE: CARABINAE: CYCHRINI. Note: this name was proposed by Fabricius in 1794 under two spellings in two different publications, as *Cychrus* in Tome 4 of his ‘Entomologia systematica’, issued by 5 October 1794 (see Bousquet 2017a: 107), and as *Cychrys* in Volume 3 of the journal ‘Skrivter af Naturhistorie-Selskabet’, for which only the year of publication is currently known; therefore, *Cychrus* is the correct original spelling and the one in use today.

Type species: *Tenebriorostratus* Linnaeus, 1761 (= *Tenebriocaraboides* Linnaeus, 1758), by subsequent designation (Latreille 1810: 426).

Family-group name: CYCHRII Perty, 1830. Stem: *Cychr*-.

***Cyclaxyra* Broun, 1893b**: 1076. Current status: valid genus in CYCLAXYRIDAE. Note: new replacement name for *Cyclomorpha* Broun, 1881; name placed on the Official List of Generic Names in Zoology (ICZN 1988a).

Type species: *Cyclomorphapolitula* Broun, 1881, by monotypy (see ICZN 1988a) (type fixation for *Cyclomorpha* Broun). Note: name placed on the Official List of Specific Names in Zoology (ICZN 1988a).

Family-group name: CYCLAXYRIDAE Gimmel, Leschen & Ślipiński, 2009. Stem: *Cyclaxyr*-.

***Cyclocephala* Dejean, 1821**: 57. Current status: valid genus in SCARABAEIDAE: DYNASTINAE: CYCLOCEPHALINI.

Type species [suggested]: *Melolonthasignata* Fabricius, 1781, by subsequent designation (Casey 1915: 112). Note: the valid type species fixation is that of Blanchard in Audouin et al. (1843: pl. 41), who selected *Melolonthageminata* Fabricius, 1801, but that nominal species is currently placed in the genus *Dyscinetus* Harold, 1869; an application to the Commission is necessary to fix *Melolonthasignata* Fabricius, 1781 as the type species of *Cyclocephala* Dejean, 1821.

Family-group name: CYCLOCEPHALITES Laporte, 1840. Stem: *Cyclocephal*-.

***Cycloderes* C.R. Sahlberg, 1823a**: 21. Current status: valid genus in CURCULIONIDAE: ENTIMINAE: POLYDRUSITAE: TANYMECINI: TANYMECINA. Note: name placed on the Official List of Generic Names in Zoology (ICZN 1987a).

Type species: *Cycloderescatarractus* C.R. Sahlberg, 1823 (= *Curculiomus* Herbst, 1797), by monotypy (see ICZN 1987a). Note: combined description of a new genus-group taxon and a single new species (ICZN 1999a, Article 12.2.6); *Cycloderescatarractus* C.R. Sahlberg, 1823 was placed on the Official List of Specific Names in Zoology (ICZN 1987a).

Family-group name: CYCLODERINI Hoffmann, 1950. Stem: *Cycloder*-.

***Cyclommatus* Parry, 1863**: 449. Current status: valid genus in LUCANIDAE: LUCANINAE: CYCLOMMATINI. Note: new replacement name for *Megaloprepes* J. Thomson, 1862.

Type species: *Lucanustarandus* Thunberg, 1806, by original designation (type fixation for *Megaloprepes* J. Thomson).

Family-group name: CYCLOMMATINI H. Huang & C.H. Chen, 2013: 70. Stem: *Cyclommat*-.

***Cyclomus* Schönherr, 1826**: 198. Current status: junior synonym of *Epicthonius* Schönherr, 1823 in CURCULIONIDAE: CYCLOMINAE: CYCLOMINI.

Type species: *Curculiosimus* Wiedemann, 1823, by monotypy.

Family-group name: CYCLOMIDES Schönherr, 1826. Stem: *Cyclom*-.

***Cyclonotum* Erichson, 1837**: 212, 214. Current status: junior synonym of *Coelostoma* Brullé, 1835 in HYDROPHILIDAE: SPHAERIDIINAE: COELOSTOMATINI. Note: this genus-group name was first made available by Dejean (1833b: 134) with *Sphaeridiumabdominale* Fabricius, 1792 as type species by monotypy, a species currently placed in the genus *Dactylosternum* Wollaston, 1854; a request should be submitted to the Commission to suppress Dejean’s name for the purposes of both the Principles of Priority and Homonymy (see Bousquet and Bouchard 2013a: 30) in order to conserve stability.

Type species: *Hydrophilusorbicularis* Fabricius, 1775, by subsequent designation (Westwood 1838c: 10).

Family-group name: CYCLONOTIDES G.H. Horn, 1890. Stem: *Cyclonot*-.

***Cyclopterus* Marseul, 1872**: 30. Current status: invalid senior synonym of *Nucterocephalus* Desbrochers des Loges, 1897 in CURCULIONIDAE: ENTIMINAE: OTIORHYNCHITAE: HOLCORHININI. Note: junior homonym of *Cyclopterus* Linnaeus, 1758 [Pisces].

Type species: *Cyclopterusspinifer* Marseul, 1872, by monotypy.

Family-group name: CYCLOPTERINI Reitter, 1913. Stem: *Cyclopter*-. Note: family-group name permanently invalid (ICZN 1999a, Article 39).

***Cyclosomus* Latreille, 1829a**: 394. Current status: valid genus in CARABIDAE: HARPALINAE: CYCLOSOMINI.

Type species: *Carabusflexuosus* Fabricius, 1775, by monotypy.

Family-group name: CYCLOSOMIDAE Laporte, 1834. Stem: *Cyclosom*-.

***Cycloteres* Schönherr, 1843**: 383. Current status: valid genus in CURCULIONIDAE: MOLYTINAE: CYCLOTERINI: CYCLOTERINA.

Type species: *Cycloteresaudouini* Boheman, 1843, by original designation.

Family-group name: CYCLOTÉRIDES Lacordaire, 1863. Stem: *Cycloter*-.

***Cyclotoma* Mulsant, 1851a**: 71. Current status: valid genus in ENDOMYCHIDAE: CYCLOTOMINAE.

Type species: *Cyclotomatestudinaria* Mulsant, 1851, by monotypy.

Family-group name: CYCLOTOMIDAE Imhoff, 1856. Stem: *Cyclotom*-.

***Cycloxenus* Arrow, 1925**: 152. Current status: valid genus in EUXESTIDAE.

Type species: *Cycloxenushispidus* Arrow, 1925, by original designation.

Family-group name: CYCLOXENINI Jeannel & Paulian, 1945. Stem: *Cyclo­xen*-.

***Cycnotrachelus* Jekel, 1860**: 160. Current status: valid genus in ATTELABIDAE: APODERINAE: APODERINI.

Type species: *Apoderusflavotuberosus* Jekel, 1860, by subsequent designation (Voss 1925: 5).

Family-group name: CYCNOTRACHELINA Legalov, 2003. Stem: *Cycnotrachel*-.

***Cydistus* Bourgeois, 1885**: xxxvii. Current status: valid genus in PHENGODIDAE: CYDISTINAE.

Type species: *Cydistusreitteri* Bourgeois, 1885, by monotypy.

Family-group name: CYDISTINAE Paulus, 1972. Stem: *Cydist*-.

***Cydonia* Mulsant, 1850**: 429, 430. Current status: junior synonym of *Cheilomenes* Chevrolat, 1836 in COCCINELLIDAE: COCCINELLINAE: COCCINELLINI.

Type species: *Cydoniaquadrilineata* Mulsant, 1850 (= *Cydoniapropinqua* Mulsant, 1850), by subsequent designation (Casey 1899: 84).

Family-group name: CYDONIAIRES Mulsant, 1850. Stem: *Cydoni*-.

***Cylas* Latreille, 1802b**: 196. Current status: valid genus in BRENTIDAE: BRENTINAE: CYLADINI: CYLADINA.

Type species: *Brentusbrunneus* G.-A. Olivier, 1790, by monotypy.

Family-group name: CYLADES Schönherr, 1823. Stem: *Cylad*-.

***Cylidrus* Latreille, 1817a**: 43. Current status: valid genus in CLERIDAE: TILLINAE. Note: this name is usually credited to Latreille (1829a: 476) in the literature but it was first made available by Latreille in 1817 (see Bouchard and Bousquet 2020a: 92).

Type species: *Cleruscyaneus* Fabricius, 1787, by monotypy.

Family-group name: CYLIDRINA Reitter, 1894. Stem: *Cylidr*-.

***Cylindrinotus* Faldermann, 1837**: 73. Current status: valid genus in TENEBRIONIDAE: TENEBRIONINAE: HELOPINI: CYLINDRINOTINA.

Type species: *Cylindrinotuslugubris* Faldermann, 1837 (= *Helopsfemoratus* Faldermann, 1837), by subsequent designation (Gebien 1943: 425). Note: Gebien (1943: 425) selected *Helopsfemoratus* Faldermann, 1837 as type species, a species not originally included, but since he listed it in synonymy with *Cylindrinotuslugubris* Faldermann, 1837, a species originally included, he is deemed to have designated the latter species as type (ICZN 1999a, Article 69.2.2).

Family-group name: CYLINDRONOTINI Español, 1956. Stem: *Cylindrinot*-.

†***Cylindrobrotus* Kirejtshuk, Azar, Beaver, Mandelshtam & Nel, 2009**: 105. Current status: valid genus in CURCULIONIDAE: SCOLYTINAE: †CYLINDROBROTINI. Note: Lower Cretaceous (Lebanese amber).

Type species: †*Cylindrobrotuspectinatus* Kirejtshuk, Azar, Beaver, Mandelshtam & Nel, 2009, by original designation.

Family-group name: †CYLINDROBROTINI Kirejtshuk, Azar, Beaver, Mandelshtam & Nel, 2009. Stem: *Cylindrobrot*-.

***Cylindrocaulus* Fairmaire, 1880**: 164. Current status: valid genus in PASSALIDAE: AULACOCYCLINAE: CYLINDROCAULINI.

Type species: *Cylindrocaulusbucerus* Fairmaire, 1880, by monotypy.

Family-group name: CYLINDROCAULINAE Zang, 1905. Stem: *Cylindrocaul*-.

***Cylindrocopturus* Heller, 1895**: 4, 56. Current status: valid genus in CURCULIONIDAE: CONODERINAE: ZYGOPINI.

Type species: *Zygopsquercus* Say, 1832, by subsequent designation (Sleeper 1963: 217).

Family-group name: CYLINDROCOPTURINI Böving, 1927. Stem: *Cylindrocoptur*-.

***Cylindrocorynus* Schönherr, 1837**: 231. Current status: valid genus in CURCULIONIDAE: CRYPTORHYNCHINAE: CRYPTORHYNCHINI: MECISTOSTYLINA.

Type species: *Cylindrocorynusimaginarius* Boheman, 1837, by original designation.

Family-group name: CYLINDROCORYNINAE Pascoe, 1875. Stem: *Cylindrocoryn*-.

***Cylindromorphoides* Kerremans, 1903c**: 301. Current status: valid genus in BUPRESTIDAE: AGRILINAE: APHANISTICINI: CYLINDROMORPHOIDINA.

Type species: *Taphrocerusagriliformis* Kerremans, 1897, by monotypy.

Family-group name: CYLINDROMORPHOIDINI Cobos, 1979. Stem: *Cylindromorphoid*-.

***Cylindromorphus* Kiesenwetter, 1856**: 159. Current status: valid genus in BUPRESTIDAE: AGRILINAE: APHANISTICINI: CYLINDROMORPHINA.

Type species: *Buprestisfilum* Gyllenhal, 1817, by subsequent designation (E. Desmarest 1860: 42).

Family-group name: CYLINDROMORPHINI Portevin, 1931. Stem: *Cylindromorph*-.

***Cylindroxystus* Bierig, 1943**: 158. Current status: valid genus in STAPHYLINIDAE: PAEDERINAE: LATHROBIINI: CYLINDROXYSTINA.

Type species: *Cylindroxystuslongulus* Bierig, 1943, by original designation.

Family-group name: CYLINDROXYSTINI Bierig, 1943. Stem: *Cylindroxyst*-.

***Cyllene* Newman, 1840c**: 7. Current status: invalid senior synonym of *Megacyllene* Casey, 1912 in CERAMBYCIDAE: CERAMBYCINAE: CLYTINI. Note: junior homonym of *Cyllene* J.E. Gray, 1834 [Mollusca].

Type species: *Cyllenespinifera* Newman, 1840 (= *Clytusnebulosus* Laporte & Gory, 1838), by monotypy.

Family-group name: CYLLENITAE J. Thomson, 1864. Stem: *Cyllen*-. Note: family-group name permanently invalid (ICZN 1999a, Article 39).

***Cyllodes* Erichson, 1843a**: 342. Current status: valid genus in NITIDULIDAE: NITIDULINAE: CYLLODINI.

Type species: *Strongylusater* Herbst, 1792, by subsequent designation (Duponchel 1844a: 515).

Family-group name: CYLLODINI Everts, 1898. Stem: *Cyllod*-.

***Cyloma* Sharp, 1872**: 152. Current status: valid genus in HYDROPHILIDAE: CYLOMINAE.

Type species: *Cylomalawsonus* Sharp, 1872, by monotypy.

Family-group name: CYLLOMINA Zaitzev, 1908. Stem: *Cylom*-.

***Cylydrorhinus* Guérin-Méneville, 1838**: 119. Current status: valid genus in CURCULIONIDAE: ENTIMINAE: CYLYDRORHINITAE: CYLYDRORHININI.

Type species: *Curculiolemniscatus* Quoy & Gaimard, 1825, by monotypy.

Family-group name: CYLINDRORHINIDES Lacordaire, 1863. Stem: *Cylydrorhin*-.

***Cymatopterus* Dejean, 1833a**: 54. Current status: junior synonym of *Colymbetes* Clairville, 1806 in DYTISCIDAE: COLYMBETINAE.

Type species: *Dytiscusfuscus* Linnaeus, 1758, by subsequent designation (C.G. Thomson 1859: 13).

Family-group name: CYMATOPTERINI Portevin, 1929. Stem: *Cymatopter*-.

***Cymbionotum* Baudi di Selve, 1864**: 211. Current status: valid genus in CARABIDAE: MELAENINAE.

Type species: *Cymbionotumcollare* Baudi di Selve, 1864 (= *Cosciniasemelederi* Chaudoir, 1861), by monotypy.

Family-group name: CYMBIONOTINI Andrewes, 1933. Stem: *Cymbionot*-.

***Cymindis* Latreille, 1805**: 190, 228. Current status: valid genus in CARABIDAE: HARPALINAE: LEBIINI: CYMINDIDINA.

Type species: *Buprestishumeralis* Geoffroy, 1785, by monotypy.

Family-group name: CYMINDIDAE Laporte, 1834. Stem: *Cymindid*-.

***Cymophorus* W. Kirby, 1827**: 153. Current status: valid genus in SCARABAEIDAE: CETONIINAE: CREMASTOCHEILINI: CYMOPHORINA.

Type species: *Cymophorusundatus* W. Kirby, 1827, by monotypy.

Family-group name: CYMOPHORINA Krikken, 1984. Stem: *Cymophor*-.

***Cynegetis* Chevrolat in Dejean, 1836a**: 437. Current status: valid genus in COCCINELLIDAE: COCCINELLINAE: EPILACHNINI.

Type species: *Coccinellaimpunctata* Linnaeus, 1767, by subsequent designation (C.G. Thomson 1859: 160).

Family-group name: CYNEGETIDES C.G. Thomson, 1866. Stem: *Cyneget*-.

***Cyno* T.A. Marshall, 1865**: 350. Current status: valid genus in CHRYSOMELIDAE: EUMOLPINAE: BROMIINI.

Type species: *Cynomordicans* T.A. Marshall, 1865, by monotypy.

Family-group name: CYNOINI Clavareau, 1914. Stem: *Cynoi*-.

***Cyparella* Achard, 1924**: 28. Current status: junior synonym of *Baeocera* Erichson, 1845 in STAPHYLINIDAE: SCAPHIDIINAE: SCAPHISOMATINI.

Type species: *Scaphisomarufoguttatum* Fairmaire, 1898, by original designation.

Family-group name: CYPARELLINI Achard, 1924. Stem: *Cyparell*-.

***Cyparium* Erichson, 1845a**: 3. Current status: valid genus in STAPHYLINIDAE: SCAPHIDIINAE: CYPARIINI.

Type species: *Cypariumpalliatum* Erichson, 1845, by monotypy.

Family-group name: CYPARIINI Achard, 1924. Stem: *Cypari*-.

***Cyphagogus* Parry, 1849**: 182. Current status: valid genus in BRENTIDAE: BRENTINAE: CYPHAGOGINI.

Type species: *Cyphagoguswestwoodii* Parry, 1849, by subsequent designation (R. Lucas 1920: 219).

Family-group name: CYPHAGOGINAE Kolbe, 1892. Stem: *Cyphagog*-.

***Cyphaleus* Westwood, 1841a**: 43. Current status: valid genus in TENEBRIONIDAE: TENEBRIONINAE: HELEINI: CYPHALEINA.

Type species: *Helopsrugosus* J.E. Gray, 1831, by subsequent designation (Duponchel 1844a: 547).

Family-group name: CYPHALÉIDES Lacordaire, 1859. Stem: *Cyphale*-.

***Cyphicerus* Schönherr, 1823**: 1144. Current status: valid genus in CURCULIONIDAE: ENTIMINAE: CYPHICERITAE: CYPHICERINI: CYPHICERINA.

Type species: *Curculioorbitalis* Wiedemann, 1819 (= *Curculionovemlineatus* G.-A. Olivier, 1807), by original designation.

Family-group name: CYPHICÉRIDES Lacordaire, 1863. Stem: *Cyphicer*-.

***Cypholoba* Chaudoir, 1850**: 43. Current status: valid genus in CARABIDAE: HARPALINAE: ANTHIINI.

Type species: *Anthiaalveolata* Brême, 1845, by monotypy.

Family-group name: CYPHOLOBINI G. Strohmeyer, 1928. Stem: *Cypholob*-.

***Cyphon* Paykull, 1799**: 117. Current status: junior synonym of *Elodes* Latreille, 1797 in SCIRTIDAE: SCIRTINAE.

Type species: *Cistelapallida* Fabricius, 1775 (= *Lampyrisminuta* Linnaeus, 1767), by subsequent designation (Westwood 1838c: 27).

Family-group name: CYPHONIDAE Stephens, 1829. Stem: *Cyphon*-.

***Cyphonocerus* Kiesenwetter, 1879**: 311. Current status: valid genus in LAMPYRIDAE: CYPHONOCERINAE.

Type species: *Cyphonocerusruficollis* Kiesenwetter, 1879, by monotypy.

Family-group name: CYPHONOCERINAE Crowson, 1972. Stem: *Cyphonocer*-.

***Cyphosoma* Hope, 1842a**: 426. Current status: invalid senior synonym of *Cratogaster* Blanchard, 1853 in CARABIDAE: HARPALINAE: PTEROSTICHINI: PTEROSTICHINA. Note: junior homonym of *Cyphosoma* Mannerheim, 1837 [Coleoptera: BUPRESTIDAE].

Type species: *Cyphosomaunicolor* Hope, 1842, by monotypy.

Family-group name: CYPHOSOMATINI Tschitschérine, 1902. Stem: *Cyphosomat*-. Note: family- group name permanently invalid (ICZN 1999a, Article 39).

***Cyphus* Germar, 1823**: 427. Current status: junior synonym of *Cyrtomon* Schönherr, 1823 in CURCULIONIDAE: ENTIMINAE: POLYDRUSITAE: NAUPACTINI: NAUPACTINA.

Type species: *Curculiogibber* Pallas, 1781, by subsequent designation (Schönherr 1826: 11).

Family-group name: CYPHIDES Lacordaire, 1863. Stem: *Cyph*-.

***Cyrionyx* Faust, 1896**: 97. Current status: valid genus in CURCULIONIDAE: BARIDINAE: OPTATINI.

Type species: *Cyrionyxbiplagiatus* Faust, 1896, by subsequent designation (Casey 1922: 10).

Family-group name: CYRIONICHINI Casey, 1922. Stem: *Cyrionych*-.

***Cyrtinus* J.L. LeConte, 1852a**: 166. Current status: valid genus in CERAMBYCIDAE: LAMIINAE: CYRTININI.

Type species: *Clytuspygmaeus* Haldeman, 1847, by monotypy.

Family-group name: CYRTINITAE J. Thomson, 1864. Stem: *Cyrtin*-. Note: senior homonym of CYRTININAE Fredericks, 1912 (type genus *Cyrtina* Davidson, 1858) in Brachiopoda.

***Cyrtoderus* Hope, 1842a**: 427. Current status: valid genus in CARABIDAE: HARPALINAE: PTEROSTICHINI: PTEROSTICHINA.

Type species: *Cyrtoderusaustralasiae* Hope, 1842, by monotypy.

Family-group name: CYRTODERINI Tschitschérine, 1902. Stem: *Cyrtoder*-.

***Cyrtognathus* Dejean, 1835**: 316. Current status: valid genus in CERAMBYCIDAE: PRIONINAE: PRIONINI.

Type species: *Prionusparadoxus* Faldermann, 1833, by monotypy.

Family-group name: CYRTHOGNATHITAE J. Thomson, 1861. Stem: *Cyrtognath*-.

***Cyrtolaus* H.W. Bates, 1882**: 99. Current status: valid genus in CARABIDAE: HARPALINAE: DRIMOSTOMATINI.

Type species: *Cyrtolausfurculifer* H.W. Bates, 1882, by subsequent designation (K.W. Kirby 1884: 32).

Family-group name: CYRTOLAINA Whitehead & Ball, 1975. Stem: *Cyrtola*-.

***Cyrtonops* A. White, 1853**: 32. Current status: valid genus in DISTENIIDAE: CYRTONOPINI.

Type species: *Cyrtonopspunctipennis* A. White, 1853, by monotypy.

Family-group name: CYRTONOPINI Gressitt, 1940. Stem: *Cyrtonop*-.

***Cyrtoquedius* Bernhauer, 1917**: 92. Current status: valid genus in STAPHYLINIDAE: STAPHYLININAE: STAPHYLININI: CYRTOQUEDIINA.

Type species: *Quediusbasiventris* Sharp, 1884, by subsequent designation (Blackwelder 1952: 115).

Family-group name: CYRTOQUEDIINA Brunke & Solodovnikov in Brunke et al., 2016: 445. Stem: *Cyrtoquedi*-.

***Cyrtoscydmus* Motschulsky, 1869b**: 260. Current status: junior synonym of *Stenichnus* C.G. Thomson, 1859 in STAPHYLINIDAE: SCYDMAENINAE: SCYDMAENITAE: STENICHNINI.

Type species: *Scydmaenusgodarti* Latreille, 1805, by subsequent designation (Atkinson 1891: 183).

Family-group name: CYRTOSCYDMINI L.W. Schaufuss, 1889. Stem: *Cyrtoscydm*-.

***Cyrtotriplax* Crotch, 1873a**: 189. Current status: junior synonym of *Tritoma* Fabricius, 1775 in EROTYLIDAE: EROTYLINAE: TRITOMINI. Note: new replacement name for *Tritoma* Fabricius, 1775.

Type species: *Tritomabipustulata* Fabricius, 1775, by subsequent designation (Latreille 1810: 432; see ICZN 1994a) (type fixation for *Tritoma* Fabricius).

Family-group name: CYRTOTRIPLACINA Chûjô, 1969. Stem: *Cyrtotriplac*-.

***Cysteodemus* J.L. LeConte, 1851a**: 158. Current status: valid genus in MELOIDAE: MELOINAE: EUPOMPHINI.

Type species: *Cysteodemusarmatus* J.L. LeConte, 1851, by subsequent designation (Wellman 1910a: 391).

Family-group name: CYSTEODEMIDES Wellman, 1910. Stem: *Cysteodem*-.

***Dacne* Latreille, 1797**: 12. Current status: valid genus in EROTYLIDAE: EROTYLINAE: DACNINI. Note: this genus was first described without nominal species by Latreille (1797: 12).

Type species: *Ipshumeralis* Fabricius, 1787 (= *Dermestesbipustulatus* Thunberg, 1781), by subsequent monotypy (Latreille 1802b: 132). Note: the type species used in recent literature (e.g., Skelley 2023: 2) is *Dermestesbipustulatus* Thunberg, 1781 by subsequent designation in Latreille (1810: 427).

Family-group name: DACNEIDAE Gistel, 1848. Stem: *Dacn*-.

***Dacoderus* J.L. LeConte, 1858a**: 74. Current status: valid genus in SALPINGIDAE: DACODERINAE.

Type species: *Dacoderusstriaticeps* J.L. LeConte, 1858, by monotypy.

Family-group name: DACODERINI J.L. LeConte, 1862. Stem: *Dacoder*-.

***Dactylipalpus* Chapuis, 1869**: 12. Current status: valid genus in CURCULIONIDAE: SCOLYTINAE: HYLESININI.

Type species: *Dactylipalpustransversus* Chapuis, 1869, by subsequent designation (Hopkins 1914: 120).

Family-group name: DACTYLIPALPI W.F.H. Blandford, 1893. Stem: *Dactylipalp*-.

***Dactylocalcar* Gebien, 1938a**: 46. Current status: valid subgenus of *Zophosis* Latreille, 1802 in TENEBRIONIDAE: PIMELIINAE: ZOPHOSINI.

Type species: *Dactylocalcarcaecus* Gebien, 1938, by monotypy.

Family-group name: DACTYLOCALCARINI Gebien, 1938. Stem: *Dactylocalcar*-.

***Dadophora* Duponchel, 1844a**: 574. Current status: valid genus in LAMPYRIDAE: LAMPYRINAE: LUCIDOTINI: DADOPHORINA. Note: the genus *Dadophora* is credited to E. Olivier (1907: 27) in the literature but the name was first made available by Duponchel (1844a: 574) when he described the species *Dadophorahyalina* (see Bouchard and Bousquet 2020a: 89); combined description of a new genus-group taxon and a single new species (ICZN 1999a, Article 12.2.6).

Type species: *Dadophorahyalina* Duponchel, 1844, by monotypy.

Family-group name: DADOPHORINI E. Olivier, 1907. Stem: *Dadophor*-.

***Daira* Gistel, 1848a**: [5]. Current status: junior synonym of *Synaptus* Eschscholtz, 1829 in ELATERIDAE: ELATERINAE: SYNAPTINI. Note: junior homonym of *Daira* H. Milne- Edwards, 1830 [Crustacea].

Type species: *Elaterfiliformis* Fabricius, 1781, by monotypy.

Family-group name: DAIRAEIDAE Gistel, 1848. Stem: *Dair*-. Note: family-group name permanently invalid (ICZN 1999a, Article 39).

***Dalmodes* Reitter, 1882a**: 197. Current status: valid genus in STAPHYLINIDAE: PSELAPHINAE: GONIACERITAE: INIOCYPHINI: INIOCYPHINA.

Type species: *Dalmodesrybaxides* Reitter, 1883, by subsequent designation (Newton and Chandler 1989: 50). Note: no species were originally included in this genus by Reitter (1882a); the first ones were the two species listed by Reitter (1883c: 382, 383); R. Lucas (1920: 225) mentioned two species as type species of *Dalmodes*, but this is considered as an ambiguous typification and is rejected (ICZN 1999a, Article 67.5.3).

Family-group name: DALMODIINI Park, 1951. Stem: *Dalmod*-.

***Dalyat* Mateu, 2002**: 67. Current status: valid genus in CARABIDAE: SCARITINAE: DALYATINI.

Type species: *Dalyatmirabilis* Mateu, 2002, by original designation.

Family-group name: DALYATINAE Mateu, 2002. Stem: *Dalyat*-.

***Danacaeomimus* Champion, 1922b**: 130. Current status: valid genus in MELYRIDAE: DASYTINAE: DANACEINI.

Type species: *Danacaeomimusnigropectus* Champion, 1922, by original designation.

Family-group name: DANACAEOMIMINI Majer, 1987. Stem: *Danacaeomim*-.

***Danacea* Laporte, 1838**: 31. Current status: valid genus in MELYRIDAE: DASYTINAE: DANACEINI.

Type species: *Lagriapallipes* Panzer, 1793, by monotypy.

Family-group name: DANACAEINA C.G. Thomson, 1859. Stem: *Danace*-.

***Danascelis* Tomaszewska, 1999**: 279. Current status: valid genus in ENDOMYCHIDAE: DANASCELINAE.

Type species: *Danasceliselongata* Tomaszewska, 1999, by original designation.

Family-group name: DANASCELINAE Tomaszewska, 2000. Stem: *Danascel*-.

***Dapsa* Latreille, 1829b**: 159. Current status: valid genus in ENDOMYCHIDAE: LYCOPERDININAE.

Type species [suggested]: *Endomychusdenticollis* Germar & Kaulfuss, 1816, by subsequent designation (R. Lucas 1920: 225). Note: the valid type species fixation is that of Latreille (1829b: 159), who included a single species in his new genus, *Eumorphuskirbyanus* Latreille, 1807, but that is the type species of the genus *Indalmus* Gerstaecker, 1858; an application to the Commission is necessary to fix *Endomychusdenticollis* Germar & Kaulfuss, 1816 as the type species of *Dapsa* Latreille, 1829 as is currently accepted in the literature.

Family-group name: DAPSINI Gerstaecker, 1858. Stem: *Daps*-.

***Daptus* Fischer, 1823a**: pl. 46. Current status: valid genus in CARABIDAE: HARPALINAE: HARPALINI: HARPALINA.

Type species: *Daptusvittatus* Fischer, 1823, by subsequent designation (Hope 1838a: 88).

Family-group name: DAPTINI J.L. LeConte, 1847. Stem: *Dapt*-.

***Daramus* Fairmaire, 1892**: 121. Current status: valid genus in CERAMBYCIDAE: CERAMBYCINAE: HESPEROPHANINI: DARAMINA.

Type species: *Daramusserricornis* Fairmaire, 1892, by monotypy.

Family-group name: DARAMINA Sama, 2008. Stem: *Daram*-.

***Darodilia* Laporte, 1867**: 71. Current status: valid genus in CARABIDAE: HARPALINAE: PTEROSTICHINI: PTEROSTICHINA.

Type species: *Darodiliamandibularis* Laporte, 1867, by monotypy.

Family-group name: DARODILIINI Tschitschérine, 1902. Stem: *Darodili*-.

***Dascillus* Latreille, 1797**: 43. Current status: valid genus in DASCILLIDAE: DASCILLINAE: DASCILLINI.

Type species: *Chrysomelacervina* Linnaeus, 1758, by subsequent monotypy (Latreille 1802b: 98).

Family-group name: DASCILLIDAE Guérin-Méneville, 1843. Stem: *Dascill*-.

***Dastarcus* Walker, 1858**: 209. Current status: valid genus in BOTHRIDERIDAE.

Type species: *Dastarcusporosus* Walker, 1858, by monotypy.

Family-group name: DASTARCINI Reitter, 1922. Stem: *Dastarc*-.

***Dasycerus* Brongniart, 1800**: 115. Current status: valid genus in STAPHYLINIDAE: DASYCERINAE.

Type species: *Dasycerussulcatus* Brongniart, 1800, by monotypy.

Family-group name: DASYCERINI Reitter, 1887. Stem: *Dasycer*-.

***Dasytes* Paykull, 1799**: 156. Current status: valid genus in MELYRIDAE: DASYTINAE: DASYTINI.

Type species: *Hispacoerulea* Fabricius, 1775 (= *Telephoruscaeruleus* DeGeer, 1774), by subsequent designation (Westwood 1838c: 28).

Family-group name: DASYDITES Laporte, 1840. Stem: *Dasyt*-.

***Dasyvalgus* Kolbe, 1904**: 25, 34. Current status: valid genus in SCARABAEIDAE: CETONIINAE: VALGINI: VALGINA.

Type species: *Valgusvethi* Ritsema, 1879, by subsequent designation (Arrow 1910: 233).

Family-group name: DASYVALGINAE Kolbe, 1904. Stem: *Dasyvalg*-.

†***Davidibelus* Zherikhin & Gratshev, 2004**: 63. Current status: valid genus in BELIDAE: BELINAE: BELITAE: †DAVIDIBELINI. Note: Lower Cretaceous (Brazil).

Type species: †*Davidibeluscearensis* Zherikhin & Gratshev, 2004, by original designation.

Family-group name: †DAVIDIBELINI Legalov, 2016: 1475. Stem: *Davidibel*-.

***Decamerus* Solier, 1849**: 369. Current status: valid genus in THYMALIDAE: DECAMERINAE.

Type species: *Decamerushaemorrhoidalis* Solier, 1849, by monotypy.

Family-group name: DECAMERINAE Crowson, 1964. Stem: *Decamer*-. Note: senior homonym of DECAMERIDAE Rasmussen, 1978 (type genus *Decameros* C. d’Orbigny, 1850) in Echinodermata.

***Decarthrocera* Laboissière, 1937**: 27. Current status: valid genus in CHRYSOMELIDAE: GALERUCINAE: DECARTHROCERINI. Note: two original spellings were used, including *Decarthrocerina* (p. 29); as far as we know, no one has acted as First Reviser (ICZN 1999a, Article 24.2.3) and we **here select***Decarthrocera* as the correct spelling.

Type species: *Decarthroceraviberti* Laboissière, 1937, by original designation.

Family-group name: DECARTHROCERINA Laboissière, 1937. Stem: *Decarthrocer*-.

***Decarthron* Brendel, 1865**: 30. Current status: valid genus in STAPHYLINIDAE: PSELAPHINAE: GONIACERITAE: BRACHYGLUTINI: DECARTHRINA.

Type species: *Bryaxisformiceti* J.L. LeConte, 1850, by subsequent designation (Bowman 1934: 144).

Family-group name: DECARTHRONINI Park, 1951. Stem: *Decarthr*-.

***Decataphanes* Labram & Imhoff, 1840**: [Heft 7]. Current status: valid genus in ANTHRIBIDAE: ANTHRIBINAE: DECATAPHANINI.

Type species: *Decataphanesgracilis* Labram & Imhoff, 1840, by **present designation**.

Family-group name: DÉCATAPHANIDES Lacordaire, 1865. Stem: *Decataphan*-.

***Declinia* Nikitsky, Lawrence, Kirejshuk & Gratshev, 1994**: 4. Current status: valid genus in DECLINIIDAE.

Type species: *Decliniarelicta* Nikitsky, Lawrence, Kirejshuk & Gratshev, 1994, by monotypy.

Family-group name: DECLINIIDAE Nikitsky, Lawrence, Kirejtshuk & Gratshev, 1994. Stem: *Declini*-.

***Dectes* J.L. LeConte, 1852a**: 144. Current status: valid genus in CERAMBYCIDAE: LAMIINAE: ACANTHOCININI.

Type species: *Lamiaspinosa* Say, 1826 (= *Dectessayi* L.S. Dillon & E.S. Dillon, 1953), by monotypy.

Family-group name: DECTITAE J. Thomson, 1860. Stem: *Dect*-.

***Decusa* Casey, 1900**: 54. Current status: valid genus in STAPHYLINIDAE: ALEOCHARINAE: OXYPODINI: DINARDINA.

Type species: *Homoeusaexpansa* J.L. LeConte, 1866, by original designation.

Family-group name: DECUSINI Fenyes, 1919. Stem: *Decus*-.

***Deilus* Audinet-Serville, 1834b**: 73. Current status: valid genus in CERAMBYCIDAE: CERAMBYCINAE: DEILINI.

Type species: *Callidiumfugax* G.-A. Olivier, 1790, by monotypy.

Family-group name: DÉILATES Fairmaire, 1864. Stem: *Deil*-.

***Deinopsis* A.[H.] Matthews, 1838**: 193. Current status: valid genus in STAPHYLINIDAE: ALEOCHARINAE: GYMNUSINI.

Type species: *Deinopsisfuscatus* A. Matthews, 1838 (= *Aleocharaerosa* Stephens, 1832), by monotypy.

Family-group name: DEINOPSINI Sharp, 1883. Stem: *Deinops*-.

***Dejanira* J. Thomson, 1864**: 134. Current status: valid genus in CERAMBYCIDAE: CERAMBYCINAE: DEJANIRINI.

Type species: *Dejaniraquadripunctata* J. Thomson, 1864, by original designation.

Family-group name: DÉJANIRIDES Lacordaire, 1868. Stem: *Dejanir*-.

***Deleaster* Erichson, 1839a**: 610. Current status: valid genus in STAPHYLINIDAE: OXYTELINAE: DELEASTERINI.

Type species: *Anthophagusdichrous* Gravenhorst, 1802, by monotypy.

Family-group name: DELEASTERINI Reitter, 1909. Stem: *Deleaster*-.

***Delevea* Reichardt, 1976**: 209. Current status: valid genus in TORRIDINCOLIDAE: DELEVEINAE.

Type species: *Deleveabertrandi* Reichardt, 1976, by original designation.

Family-group name: DELEVEINAE Endrödy-Younga, 1997. Stem: *Deleve*-.

***Delinius* Westwood, 1864b**: 3. Current status: valid genus in CARABIDAE: HARPALINAE: PTEROSTICHINI: PTEROSTICHINA.

Type species: *Deliniusessingtonii* Westwood, 1864, by monotypy.

Family-group name: DELINIINI Tschitschérine, 1902. Stem: *Delini*-.

***Delocheilus* J. Thomson, 1861**: 309. Current status: valid genus in CERAMBYCIDAE: PRIONINAE: ANACOLINI.

Type species: *Delocheilusprionoides* J. Thomson, 1861, by monotypy.

Family-group name: DELOCHILI Lameere, 1913. Stem: *Delocheil*-.

***Delocrania* Guérin-Méneville, 1844b**: pl. 131. Current status: valid genus in CHRYSOMELIDAE: CASSIDINAE: DELOCRANIINI.

Type species: *Delocraniacossyphoides* Guérin-Méneville, 1844, by monotypy.

Family-group name: DELOCRANIITAE Spaeth, 1929. Stem: *Delocrani*-.

***Deltochilum* Eschscholtz, 1822**: 37. Current status: valid genus in SCARABAEIDAE: SCARABAEINAE: DELTOCHILINI.

Type species: *Deltochilumdentipes* Eschscholtz, 1822, by subsequent designation (Thon 1832a: 424).

Family-group name: DELTOCHILIDES Lacordaire, 1855. Stem: *Deltochil*-.

***Deltomerodes* Deuve, 1992**: 82. Current status: valid genus in CARABIDAE: PATROBINAE: PATROBINI: DELTOMERODINA.

Type species: *Deltomerodesmemorabilis* Deuve, 1992, by original designation.

Family-group name: DELTOMERODINA Zamotajlov, 2002. Stem: *Deltomerod*-.

***Deltomerus* Motschulsky, 1850**: 71. Current status: valid genus in CARABIDAE: TRECHINAE: PATROBINI: DELTOMERINA.

Type species: *Platynusfulvipes* Motschulsky, 1839, by subsequent designation (Enderlein 1909: 487).

Family-group name: DELTOMERIDAE Chaudoir, 1871. Stem: *Deltomer*-.

***Demarziella* Balthasar, 1961**: 178. Current status: valid genus in SCARABAEIDAE: SCARABAEINAE: ATEUCHINI incertae sedis.

Type species: *Demarziellamirifica* Balthasar, 1961, by original designation.

Family-group name: DEMARZIELLINI Balthasar, 1961. Stem: *Demarziell*-.

***Demetrias* Bonelli, 1810**: Tab. Syn. Current status: valid genus in CARABIDAE: HARPALINAE: LEBIINI: DEMETRIADINA.

Type species: *Carabusatricapillus* Linnaeus, 1758, by subsequent designation (Latreille 1817a: 246). Note: no species were originally included in this genus by Bonelli (1810); the first ones were the three species listed by Panzer (1813: 73).

Family-group name: DEMETRIINAE H.W. Bates, 1886. Stem: *Demetriad*-.

***Demimaea* Pascoe, 1870**: 440. Current status: valid genus in CURCULIONIDAE: CURCULIONINAE: TYCHIINI: DEMIMAEINA.

Type species: *Demimaealuctuosa* Pascoe, 1870, by monotypy.

Family-group name: DEMIMAEINI Voss, 1937. Stem: *Demimae*-.

***Dendarus* Dejean, 1821**: 65. Current status: valid genus in TENEBRIONIDAE: BLAPTINAE: DENDARINI: DENDARINA.

Type species: *Helopstristis* Rossi, 1790, by subsequent designation (Blanchard in Audouin et al. 1844: pl. 48). Note: see Bouchard et al. (2021: 159) and Kamiński et al. (2021: 576) for comments on the type species of this genus.

Family-group name: PANDARITES Mulsant & Rey, 1854. Stem: *Dendar*-.

***Dendezia* Basilewsky, 1952b**: 36. Current status: valid genus in LUCANIDAE: LUCANINAE: LUCANINI.

Type species: *Dendeziarenieri* Basilewsky, 1952, by original designation.

Family-group name: DENDEZIINI Benesh, 1955. Stem: *Dendezi*-.

***Dendrocharis* Guérin-Méneville, 1843b**: 193. Current status: valid genus in EUCNEMIDAE: EUCNEMINAE: DENDROCHARINI.

Type species: *Galbaflavicornis* Guérin-Méneville, 1843, by monotypy.

Family-group name: DENDROCHARINI Fleutiaux, 1920. Stem: *Dendrochar*-.

***Dendroctonus* Erichson, 1836**: 52. Current status: valid genus in CURCULIONIDAE: SCOLYTINAE: HYLURGINI. Note: name placed on the Official List of Generic Names in Zoology (ICZN 1963a).

Type species: *Bostrichusmicans* Kugelann, 1794, by plenary power (see ICZN 1963a). Note: name placed on the Official List of Specific Names in Zoology (ICZN 1963a).

Family-group name: DENDROCTONIDES Nüsslin, 1912. Stem: *Dendrocton*-.

***Dendrometrus* Gistel, 1848b**: xi. Current status: junior synonym of *Limonius* Eschscholtz, 1829 in ELATERIDAE: DENDROMETRINAE: DENDROMETRINI: DENDROMETRINA. Note: unnecessary new replacement name for *Limonius* Eschscholtz, 1829.

Type species: *Elaterminutus* Linnaeus, 1758, by subsequent designation (Curtis 1838a: pl. 694) (type fixation for *Limonius* Eschscholtz).

Family-group name: DENDROMETRIDAE Gistel, 1848. Stem: *Dendrometr*-.

***Dendrophagus* Schönherr, 1809**: 50. Current status: valid genus in SILVANIDAE: BRONTINAE: BRONTINI.

Type species: *Cucujuscrenatus* Paykull, 1799, by monotypy.

Family-group name: DENDROPHAGIDAE Gistel, 1848. Stem: *Dendrophag*-.

***Dendrophilus* Leach, 1817**: 77. Current status: valid genus in HISTERIDAE: DENDROPHILINAE: DENDROPHILINI.

Type species: *Histerpunctatus* Herbst, 1791, by monotypy.

Family-group name: DENDROPHILINI Reitter, 1909. Stem: *Dendrophil*-.

***Dendroplanetes* Gistel, 1856**: 368. Current status: junior synonym of *Opilo* Latreille, 1802 in CLERIDAE: CLERINAE: OPILONITAE: OPILONINI: OPILONINA.

Type species: *Attelabusmollis* Linnaeus, 1758, by **present designation**.

Family-group name: DENDROPLANETEIDAE Gistel, 1856. Stem: *Dendroplanet*-.

***Denticollis* Piller & Mitterpacher, 1783**: 86. Current status: valid genus in ELATERIDAE: DENDROMETRINAE: DENDROMETRINI: DENTICOLLINA.

Type species: *Denticollisrubens* Piller & Mitterpacher, 1783, by subsequent designation (Hyslop 1921: 639).

Family-group name: DENTICOLLINI Stein & Weise, 1877. Stem: *Denticoll*-.

***Depasophilus* Voss, 1922b**: 410. Current status: valid genus in ATTELABIDAE: RHYNCHITINAE: RHYNCHITITAE: DEPORAINI.

Type species: *Depasophilusbakeri* Voss, 1922, by monotypy.

Family-group name: DEPASOPHILINA Legalov, 2003. Stem: *Depasophil*-.

***Deporaus* Leach in Samouelle, 1819**: 201. Current status: valid genus in ATTELABIDAE: RHYNCHITINAE: RHYNCHITITAE: DEPORAINI. Note: this name is currently considered valid but it is a junior synonym of *Platyrynchus* Thunberg, 1815; Alonso-Zarazaga and Lyal (1999: 43) mentioned that they would submit an application to the Commission to suppress the older name *Platyrynchus* Thunberg, 1815, but this has not taken place; *Deporaus* is listed as a nomen protectum in more recent literature (e.g., Alonso-Zarazaga 2011: 117) but the 25 references needed to establish its status as a nomen protectum (see ICZN 1999a, Article 23.9.1.2) have not been published to our knowledge; an application to the Commission (or publication of evidence to support the nomen protectum status) is still required to conserve the status of *Deporaus* Leach, 1819 as valid.

Type species: *Attelabusbetulae* Linnaeus, 1758, by monotypy.

Family-group name: DEPORAINI Voss, 1929. Stem: *Depora*-.

***Deracanthus* Schönherr, 1823**: 1139. Current status: valid genus in CURCULIONIDAE: ENTIMINAE: ENTIMITAE: OPHRYASTINI: DERACANTHINA.

Type species: *Curculiospinifex* Fabricius, 1787 (= *Curculioinderiensis* Pallas, 1771), by original designation.

Family-group name: DERACANTHINA Legalov, 2020b: 339. Stem: *Deracanth*-. Note: junior homonym of DERACANTHIDAE Jacobson, 1905 (type genus *Deracantha* Fischer von Waldheim, 1833) in Orthoptera.

***Derallus* Sharp, 1882c**: 77. Current status: valid genus in HYDROPHILIDAE: HYDROPHILINAE: BEROSINI.

Type species: *Derallusangustus* Sharp, 1882, by monotypy.

Family-group name: DERALLINI Bertrand, 1967. Stem: *Derall*-.

***Derancistrus* Audinet-Serville, 1832**: 181. Current status: valid genus in CERAMBYCIDAE: PRIONINAE: SOLENOPTERINI.

Type species: *Prionuselegans* Palisot de Beauvois, 1819, by monotypy.

Family-group name: DERANCISTRINI Lameere, 1912. Stem: *Derancistr*-.

***Derbyiella* Lea, 1907**: 430. Current status: valid genus in CURCULIONIDAE: CRYPTORHYNCHINAE: AEDEMONINI. Note: new replacement name for *Derbyia* Lea, 1900.

Type species: *Derbyialaminatus* Lea, 1900, by monotypy (type fixation for *Derbyia* Lea).

Family-group name: DERBYIELLINA Zimmerman, 1994. Stem: *Derbyiell*-.

***Dercylus* Laporte, 1833b**: 392. Current status: valid genus in CARABIDAE: HARPALINAE: DERCYLINI.

Type species: *Dercylusater* Laporte, 1833, by monotypy.

Family-group name: DERCYLINI Sloane, 1923. Stem: *Dercyl*-.

***Derelomus* Schönherr, 1825**: 583. Current status: valid genus in CURCULIONIDAE: CURCULIONINAE: DERELOMINI: DERELOMINA.

Type species: *Curculiochamaeropis* Fabricius, 1798, by original designation.

Family-group name: DÉRÉLOMIDES Lacordaire, 1865. Stem: *Derelom*-.

***Derema* Fauvel, 1899**: 41. Current status: valid genus in STAPHYLINIDAE: ALEOCHARINAE: MIMANOMMATINI: DORYLOPHILINA.

Type species: *Deremafoveicollis* Fauvel, 1899, by subsequent designation (Fenyes 1912: 24).

Family-group name: DEREMINI Seevers, 1965. Stem: *Derem*-.

***Deretaphrus* Newman, 1842c**: 403. Current status: valid genus in BOTHRIDERIDAE.

Type species: *Deretaphrusfossus* Newman, 1842, by subsequent designation (Wollaston in Wollaston and Newman 1855: ccviii).

Family-group name: DERETAPHRINI G.H. Horn, 1878. Stem: *Deretaphr*-.

***Deridea* Westwood, 1875**: 226. Current status: valid genus in MELOIDAE: ELETICINAE: DERIDEINI.

Type species: *Derideacurculionides* Westwood, 1875, by monotypy.

Family-group name: DERIDEIDES Wellman, 1910. Stem: *Deride*-.

***Dermatodes* Schönherr, 1840a**: 895. Current status: valid genus in CURCULIONIDAE: ENTIMINAE: POLYDRUSITAE: DERMATODINI. Note: new replacement name for *Lagostomus* Schönherr, 1833.

Type species: *Lagostomuspaganus* Gyllenhal, 1833, by original designation (type fixation for *Lagostomus* Schönherr).

Family-group name: DERMATODINI Emden, 1936. Stem: *Dermatod*-.

***Dermatohomoeus* Hlisnikovský, 1963**: 302. Current status: valid genus in LEIODIDAE: LEIODINAE: PSEUDOLIODINI.

Type species: *Dermatohomoeusguineensis* Hlisnikovský, 1963, by original designation.

Family-group name: DERMATOHOMOEINI Hlisnikovský, 1963. Stem: *Dermatohomoe*-.

***Dermeanthrenus* Háva, 2008**: 62. Current status: valid genus in DERMESTIDAE: MEGATOMINAE: ANTHRENINI.

Type species: *Dermeanthrenuspretiosus* Háva, 2008, by original designation.

Family-group name: DERMEANTHRENINA Háva, 2011: 6. Stem: *Dermeanthren*-.

***Dermestes* Linnaeus, 1758**: 342, 354. Current status: valid genus in DERMESTIDAE: DERMESTINAE: DERMESTINI.

Type species: *Dermesteslardarius* Linnaeus, 1758, by subsequent designation (Latreille 1810: 428).

Family-group name: DERMESTINI Latreille, 1804. Stem: *Dermest*-.

***Dermestoides* Schäffer, 1777**: pl. cxxxviii. Current status: valid genus in CLERIDAE: KORYNETINAE.

Type species [suggested]: *Dermestessanguinicollis* Fabricius, 1787, by subsequent designation (J.R. Winkler 1979: 311). Note: no species were originally included in this genus by Schäffer (1777); the first ones were the two species listed by Herbst (1783: 40), *Dermestoidesunipunctatus* Herbst, 1783 (= *Dermesteslinearis* Goeze, 1777) and *Dermestoidesbipunctatus* Herbst, 1783 (possibly a senior synonym of *Rhizophagusbipunctatus* Herbst, 1793); acceptance of either of these two as type species of *Dermestoides* will threaten the status of this type genus since the former is now included in *Lyctus* Fabricius, 1792 (BOSTRICHIDAE) and the latter possibly in MONOTOMIDAE (more research is needed to ascertain the identity of *Dermestoidesbipunctatus* Herbst, 1783); an application to the Commission is necessary to fix *Dermestessanguinicollis* Fabricius, 1787 as the type species of *Dermestoides* Schäffer, 1777 in order to conserve its currently accepted concept.

Family-group name: DERMESTOIDINI Jakobson, 1911. Stem: *Dermestoid*-.

***Derobrachus* Audinet-Serville, 1832**: 154. Current status: valid genus in CERAMBYCIDAE: PRIONINAE: PRIONINI.

Type species: *Derobrachusbrevicollis* Audinet-Serville, 1832, by monotypy.

Family-group name: DEROBRACHITAE J. Thomson, 1864. Stem: *Derobrach*-.

***Derodontus* J.L. LeConte, 1861**: 100. Current status: valid genus in DERODONTIDAE: DERODONTINAE.

Type species: *Cryptophagusmaculatus* Melsheimer, 1844, by subsequent designation (R. Lucas 1920: 232).

Family-group name: DERODONTIDAE J.L. LeConte, 1861. Stem: *Derodont*-.

***Derolathrus* Sharp in Sharp and H. Scott, 1908**: 430. Current status: valid genus in JACOBSONIIDAE.

Type species: *Derolathrusatomus* Sharp, 1908, by monotypy.

Family-group name: DEROLATHRIINAE Sen Gupta, 1979. Stem: *Derolathr*-.

***Deronectes* Sharp, 1882b**: 390, 418. Current status: valid genus in DYTISCIDAE: HYDROPORINAE: HYDROPORINI: DERONECTINA. Note: this genus was first made available by Sharp (1880: cxlviii) when he listed two available species, *Dytiscusduodecimpustulatus* Fabricius, 1792 and *Dytiscuselegans* Panzer, 1794, but the first species is the type species of *Stictotarsus* A. Zimmermann, 1919 and the second is currently included in the genus *Nebrioporus* Régimbart, 1906; an application to the Commission is necessary to suppress the older but unused name *Deronectes* Sharp, 1880 in order to conserve the current usage of *Deronectes* Sharp, 1882.

Type species: *Hydroporuslatus* Stephens, 1829, by subsequent designation (F. Balfour-Browne 1934: 179).

Family-group name: DERONECTINI Galewski, 1994. Stem: *Deronect*-.

***Derops* Sharp, 1889a**: 418. Current status: valid genus in STAPHYLINIDAE: TACHYPORINAE: DEROPINI.

Type species: *Deropslongicornis* Sharp, 1889, by monotypy.

Family-group name: DEROPSINI Smetana, 1983. Stem: *Derop*-.

†***Desmatus* Dolin, 1975**: 60. Current status: valid genus in ELATERIDAE: †PROTAGRYPNINAE: †DESMATINI. Note: Jurassic (Kazakhstan).

Type species: †*Desmatuslapidarius* Dolin, 1975, by original designation.

Family-group name: †DESMATINI Dolin, 1975. Stem: *Desmat*-.

***Desmidophorus* Dejean, 1835**: 296. Current status: valid genus in CURCULIONIDAE: BRACHYCERINAE: ERIRHININI.

Type species: *Curculiohebes* Fabricius, 1781, by monotypy.

Family-group name: DESMIDOPHORINAE Morimoto, 1962. Stem: *Desmidophor*-.

***Desmiphora* Audinet-Serville, 1835a**: 62. Current status: valid genus in CERAMBYCIDAE: LAMIINAE: DESMIPHORINI.

Type species: *Lamiafasciculata* G.-A. Olivier, 1792, by subsequent designation (Drapiez 1838: 456).

Family-group name: DESMIPHORITAE J. Thomson, 1860. Stem: *Desmiphor*-.

***Desmocerus* Dejean, 1821**: 111. Current status: valid genus in CERAMBYCIDAE: LEPTURINAE: DESMOCERINI.

Type species: *Stenocoruscyaneus* Fabricius, 1775 (= *Cerambyxpalliatus* Forster, 1771), by monotypy.

Family-group name: DESMOCÉRITES Blanchard, 1845. Stem: *Desmocer*-.

***Desmonyx* Arrow, 1907**: 355. Current status: valid genus in SCARABAEIDAE: RUTELINAE: RUTELINI: DESMONYCHINA.

Type species: *Desmonyxhumeralis* Arrow, 1907, by monotypy.

Family-group name: DESMONYCHINAE Arrow, 1917. Stem: *Desmonych*-.

***Desmoris* J.L. LeConte in J.L. LeConte and G.H. Horn, 1876**: 167. Current status: junior synonym of *Smicronyx* Schönherr, 1843 in CURCULIONIDAE: CURCULIONINAE: SMICRONYCHINI.

Type species: *Rhynchaenusconstrictus* Say, 1824, by subsequent designation (D.M. Anderson 1962: 322).

Family-group name: DESMORHINES J.L. LeConte, 1876. Stem: *Desmorin*-. Note: nomen oblitum (see Bouchard et al. 2011: 584).

***Dexoris* C.O. Waterhouse, 1878**: 105. Current status: valid genus in LYCIDAE: DEXORINAE: DEXORINI: DEXORINA.

Type species: *Dexorisinsignis* C.O. Waterhouse, 1878, by original designation.

Family-group name: DEXORINAE Bocák & Bocáková, 1989. Stem: *Dexor*-.

***Dhysores* Grouvelle, 1903**: 90, 92. Current status: valid genus in CARABIDAE: RHYSODINAE: DHYSORINI.

Type species: *Rhysodesthoreyi* Grouvelle, 1903, by subsequent designation (Hincks 1950a: 4).

Family-group name: DHYSORINA R.T. Bell & J.R. Bell, 1978. Stem: *Dhysor*-.

***Diabathrarius* Schönherr, 1840b**: 464. Current status: valid genus in CURCULIONIDAE: MOLYTINAE: LITHININI: LITHININA.

Type species: *Diabathrariusvariegatus* Boheman, 1840, by original designation.

Family-group name: DIABATHRARIIDES Lacordaire, 1863. Stem: *Diabathrari*-.

***Diabrotica* Chevrolat in Dejean, 1836a**: 380. Current status: valid genus in CHRYSOMELIDAE: GALERUCINAE: LUPERINI.

Type species: *Criocerisfucata* Fabricius, 1787, by subsequent designation (Barber 1947a: 151).

Family-group name: DIABROTICITES Chapuis, 1875. Stem: *Diabrotic*-.

***Diacantha* Chevrolat in Dejean, 1836a**: 378. Current status: valid genus in CHRYSOMELIDAE: GALERUCINAE: LUPERINI.

Type species: *Gallerucaunifasciata* G.-A. Olivier, 1808, by subsequent designation (Hincks 1949: 613).

Family-group name: DIACANTHINI Weise, 1902a: 273. Stem: *Diacanth*-. Note: this name was overlooked in Bouchard et al. (2011) and Bouchard and Bousquet (2020a); junior homonym of the nomen oblitum DIACANTHIDAE Gistel, 1848 (type genus *Diacanthus* Latreille, 1834) in Coleoptera: ELATERIDAE.

***Diacanthus* Latreille, 1834**: 151. Current status: junior synonym of *Selatosomus* Stephens, 1830 in ELATERIDAE: DENDROMETRINAE: PROSTERNINI.

Type species: *Elateraeneus* Linnaeus, 1758, by subsequent designation (C.G. Thomson 1859: 102).

Family-group name: DIACANTHIDAE Gistel, 1848. Stem: *Diacanth*-. Note: nomen oblitum (see Bouchard et al. 2011: 315); senior homonym of DIACANTHINI Weise, 1902 (type genus *Diacantha* Chevrolat, 1836) in Coleoptera: CHRYSOMELIDAE.

***Diamerus* Erichson, 1836**: 57. Current status: valid genus in CURCULIONIDAE: SCOLYTINAE: DIAMERINI.

Type species: *Hylesinushispidus* Klug, 1833, by monotypy.

Family-group name: DIAMERINAE Hagedorn, 1909. Stem: *Diamer*-.

***Diaperis* Geoffroy, 1762**: 337. Current status: valid genus in TENEBRIONIDAE: DIAPERINAE: DIAPERINI: DIAPERINA. Note: name placed on the Official List of Generic Names in Zoology (ICZN 1994a).

Type species: *Chrysomelaboleti* Linnaeus, 1758, by subsequent monotypy (O.F. Müller 1776: 74; see ICZN 1994a). Note: name placed on the Official List of Specific Names in Zoology (ICZN 1994a).

Family-group name: DIAPERIALAE Latreille, 1802. Stem: *Diaper*-.

***Diaphanops* Schönherr, 1845**: 342. Current status: valid genus in CHRYSOMELIDAE: SAGRINAE: DIAPHANOPSIDINI.

Type species: *Diaphanopswestermanni* Boheman, 1845, by original designation.

Family-group name: DIAPHANOPSIDINI Monrós, 1958. Stem: *Diaphanopsid*-.

***Diaphonia* Newman, 1840b**: 366. Current status: valid genus in SCARABAEIDAE: CETONIINAE: SCHIZORHININI: SCHIZORHININA.

Type species: *Diaphoniadispar* Newman, 1840, by monotypy.

Family-group name: DIAPHONIADAE Kraatz, 1880. Stem: *Diaphoni*-.

***Diapus* Chapuis, 1865**: 43, 329. Current status: valid genus in CURCULIONIDAE: PLATYPODINAE: TESSEROCERINI: DIAPODINA.

Type species: *Diapusquadrispinatus* Chapuis, 1865, by subsequent designation (Hopkins 1914: 121).

Family-group name: DIAPODARIAE H. Strohmeyer, 1914. Stem: *Diapod*-.

***Diatelium* Pascoe, 1863a**: 27. Current status: valid genus in STAPHYLINIDAE: SCAPHIDIINAE: SCAPHIDIINI.

Type species: *Diateliumwallacei* Pascoe, 1863, by monotypy.

Family-group name: DIATELIITAE Achard, 1924. Stem: *Diateli*-.

***Dibolia* Latreille, 1829b**: 155. Current status: valid genus in CHRYSOMELIDAE: GALERUCINAE: ALTICINI.

Type species: *Halticaoccultans* J.D.W. Koch, 1803, by subsequent designation (Curtis 1833a: pl. 435 [p. 2]).

Family-group name: DIBOLIITES Chapuis, 1875. Stem: *Diboli*-.

***Dicaelus* Bonelli, 1813**: 446. Current status: valid genus in CARABIDAE: HARPALINAE: LICININI: DICAELINA.

Type species: *Dicaelusviolaceus* Bonelli, 1813 (= *Dicaeluspurpuratus* Bonelli, 1813), by subsequent designation (Hope 1838a: 82).

Family-group name: DICOELIDAE Laporte, 1834. Stem: *Dicael*-.

***Dicax* Fauvel, 1878**: 512, 518. Current status: valid genus in STAPHYLINIDAE: PAEDERINAE: PAEDERINI: DICAXINA.

Type species: *Lathrobiumlongiceps* Fauvel, 1877, by subsequent designation (Bertkau 1879: 441).

Family-group name: DICAXINA Schomann & Solodovnikov, 2017: 10. Stem: *Dicax*-.

***Dicerca* Eschscholtz, 1829b**: 9. Current status: valid genus in BUPRESTIDAE: CHRYSOCHROINAE: DICERCINI: DICERCINA. Note: the original spelling was *Dicerea* but *Dicerca* was ruled by the Commission to be the correct original spelling and the name was placed on the Official List of Generic Names in Zoology (ICZN 1994f).

Type species: *Buprestisaenea* Linnaeus, 1761, by subsequent designation (Westwood 1838c: 24; see ICZN 1994f). Note: name placed on the Official List of Specific Names in Zoology (ICZN 1994f).

Family-group name: DICERCAEIDAE Gistel, 1848. Stem: *Dicerc*-.

***Dichelonyx* Harris, 1827**: 7. Current status: valid genus in SCARABAEIDAE: MELOLONTHINAE: DICHELONYCHINI.

Type species: *Melolonthalinearis* Gyllenhal, 1817 (= *Melolonthaelongatula* Schönherr, 1817), by monotypy.

Family-group name: DICHELONYCHIDAE Burmeister, 1855. Stem: *Dichelonych*-.

***Dichelus* Lepeletier & Audinet-Serville, 1828**: 373. Current status: valid genus in SCARABAEIDAE: MELOLONTHINAE: HOPLIINI: HOPLIINA.

Type species: *Melolonthadentipes* Fabricius, 1781, by monotypy.

Family-group name: DICHELIDEN Oken, 1843. Stem: *Dichel*-.

***Dichillus* Jacquelin du Val, 1860b**: 253. Current status: valid genus in TENEBRIONIDAE: PIMELIINAE: STENOSINI: DICHILLINA.

Type species: *Tageniaminuta* Solier, 1838, by original designation.

Family-group name: DICHILLINA Reitter, 1916. Stem: *Dichill*-.

***Dichophyia* Gistel, 1848b**: xi, 131. Current status: junior synonym of *Mallosoma* Audinet-Serville, 1834 in CERAMBYCIDAE: CERAMBYCINAE: DICHOPHYIINI. Note: unnecessary new replacement name for *Mallosoma* Audinet-Serville, 1834.

Type species: *Mallosomaelegans* Audinet-Serville, 1834 (= *Cerambyxzonatus* C.R. Sahlberg, 1823), by monotypy (type fixation for *Mallosoma* Audinet-Serville).

Family-group name: DICHOPHYIAEIDAE Gistel, 1848. Stem: *Dichophyi*-.

***Dichotomius* Hope, 1838b**: 321. Current status: valid genus in SCARABAEIDAE: SCARABAEINAE: DICHOTOMIINI.

Type species: *Scarabaeusboreus* G.-A. Olivier, 1789, by original designation.

Family-group name: DICHOTOMIINI Pereira, 1954. Stem: *Dichotomi*-.

***Dichotrachelus* Stierlin, 1853**: 171. Current status: valid genus in CURCULIONIDAE: CYCLOMINAE: DICHOTRACHELINI.

Type species: *Dichotrachelussulcipennis* Stierlin, 1853, by monotypy.

Family-group name: DICHOTRACHELINI Hoffmann, 1957. Stem: *Dichotrachel*-.

***Dicrania* Lepeletier & Audinet-Serville, 1828**: 371. Current status: valid genus in SCARABAEIDAE: MELOLONTHINAE: MACRODACTYLINI.

Type species: *Dicraniarubricollis* Lepeletier & Audinet-Serville, 1828, by subsequent designation (Blanchard in Audouin et al. 1843: pl. 44).

Family-group name: DICRANIADAE Burmeister, 1855. Stem: *Dicrani*-.

***Dicranosterna* Motschulsky, 1860b**: 193. Current status: valid genus in CHRYSOMELIDAE: CHRYSOMELINAE: CHRYSOMELINI.

Type species: *Paropsispicea* G.-A. Olivier, 1807, by original designation.

Family-group name: DICRANOSTERNINI Weise, 1915. Stem: *Dicranostern*-.

***Dicrepidius* Eschscholtz, 1829a**: 31. Current status: valid genus in ELATERIDAE: ELATERINAE: DICREPIDIINI.

Type species: *Dicrepidiuspectinicornis* Eschscholtz, 1829 (= *Elaterramicornis* Palisot de Beauvois, 1805), by subsequent designation (Hyslop 1921: 640).

Family-group name: DICREPIDIITAE J. Thomson, 1858. Stem: *Dicrepidi*-.

***Dicrochile* Guérin-Méneville, 1847**: ciii. Current status: valid genus in CARABIDAE: HARPALINAE: LICININI: DICROCHILINA. Note: this genus was first described without nominal species by Guérin-Méneville (1846: 428) under the spelling *Dicronochilus*; the spelling *Dicrochile*, which is in prevailing usage, is an incorrect subsequent spelling of *Dicronochilus* but since *Dicrochile* is not attributed to the publication of the original spelling, Article 33.3.1 (ICZN 1999a) cannot be used; an application to the Commission is necessary to conserve the currently used spelling *Dicrochile*.

Type species: *Dicrochileanchomenoides* Guérin-Méneville, 1847, by subsequent designation (Larochelle and Larivière 2001: 114).

Family-group name: DICROCHILINA Ball, 1992. Stem: *Dicrochil*-.

***Dicrodontus* Chaudoir, 1872a**: 139. Current status: valid genus in CARABIDAE: HARPALINAE: ZUPHIINI: DICRODONTINA.

Type species: *Polistichusbrunneus* Dejean, 1831, by monotypy.

Family-group name: DICRODONTINI Machado, 1992. Stem: *Dicrodont*-.

***Dicronocephalus* Hope, 1831**: 24. Current status: valid genus in SCARABAEIDAE: CETONIINAE: GOLIATHINI: DICRONOCEPHALINA.

Type species: *Dicronocephaluswallichii* Hope, 1831, by monotypy.

Family-group name: DICRONOCEPHALINA Krikken, 1984. Stem: *Dicronocephal*-.

***Dictyoptera* Latreille, 1829a**: 464. Current status: valid genus in LYCIDAE: EROTINAE: DICTYOPTERINI.

Type species [suggested]: *Pyrochroaaurora* Herbst, 1784, misidentified as *Cantharissanguineus* Linnaeus, 1761 (sensu Latreille, 1829) in the subsequent designation by Blanchard in Audouin et al. (1842: pl. 32). Note: the valid type species fixation is that of Westwood (1838c: 27), who selected *Pyrochroaminuta* Fabricius, 1787, but that species is the type species of *Platycis* C.G. Thomson, 1859; an application to the Commission is necessary to fix *Pyrochroaaurora* Herbst, 1784 as the type species of *Dictyoptera* Latreille, 1829.

Family-group name: DICTYOPTERINI Houlbert, 1922. Stem: *Dictyopter*-.

***Didactylia* H. d’Orbigny, 1896**: 247. Current status: valid genus in SCARABAEIDAE: APHODIINAE: APHODIINI: DIDACTYLIINA.

Type species: *Aphodiuspallicolor* Fairmaire, 1886, by monotypy. Note: H. d’Orbigny (1896: 248) proposed the unjustified emendation *pallidicolor* when he fixed the type species of his new genus.

Family-group name: DIDACTYLIINA Pittino, 1985. Stem: *Didactyli*-.

***Didrepanephorus* Wood-Mason, 1878**: 423. Current status: valid genus in SCARABAEIDAE: RUTELINAE: RUTELINI: DIDREPANEPHORINA.

Type species: *Didrepanephorusbifalcifer* Wood-Mason, 1878, by original designation.

Family-group name: DIDREPANOPHORINA Ohaus, 1918. Stem: *Didrepanephor*-.

***Didymonycha* Aurivillius, 1922a**: 31. Current status: junior synonym of *Amillarus* J. Thomson, 1857 in CERAMBYCIDAE: LAMIINAE: AGAPANTHIINI.

Type species: *Didymonychasingularis* Aurivillius, 1922, by monotypy.

Family-group name: DIDYMONYCHINI Aurivillius, 1922. Stem: *Didymonych*-.

***Dieropsis* Gahan, 1908**: 95. Current status: valid genus in CLERIDAE: CLERINAE: OPILONITAE: DIEROPSEINI.

Type species: *Dieropsisquadriplagiata* Gahan, 1908, by monotypy.

Family-group name: DIEROPSINAE J.R. Winkler, 1964. Stem: *Dieropse*-. Note: see Bouchard et al. (2011: 347) regarding the recommended stem for this family-group name.

***Diestota* Mulsant & Rey, 1870**: 194. Current status: valid genus in STAPHYLINIDAE: ALEOCHARINAE: DIESTOTINI.

Type species: *Diestotamayeti* Mulsant & Rey, 1870 (= *Bolitocharatestacea* Kraatz, 1859), by monotypy.

Family-group name: DIESTOTATES Mulsant & Rey, 1871. Stem: *Diestot*-.

***Diglossa* Haliday, 1837**: 252. Current status: invalid senior synonym of *Diglotta* Champion, 1899 in STAPHYLINIDAE: ALEOCHARINAE: DIGLOTTINI. Note: junior homonym of *Diglossa* Wagler, 1832 [Aves].

Type species: *Diglossamersa* Haliday, 1837, by monotypy.

Family-group name: DIGLOSSAIRES Mulsant & Rey, 1873. Stem: *Digloss*-. Note: family-group name permanently invalid (ICZN 1999a, Article 39).

***Diglotta* Champion, 1899**: 265. Current status: valid genus in STAPHYLINIDAE: ALEOCHARINAE: DIGLOTTINI. Note: new replacement name for *Diglossa* Haliday, 1837.

Type species: *Diglossamersa* Haliday, 1837, by monotypy (type fixation for *Diglossa* Haliday).

Family-group name: DIGLOTTINA Jakobson, 1909. Stem: *Diglott*-.

***Dignamptus* J.L. LeConte, 1878a**: 421. Current status: junior synonym of *Talanus* Jacquelin du Val, 1857 in TENEBRIONIDAE: STENOCHIINAE: TALANINI.

Type species: *Dignamptusstenochinus* J.L. LeConte, 1878, by subsequent designation (Bousquet et al. 2018: 325).

Family-group name: DIGNAMPTINI J.L. LeConte & G.H. Horn, 1883. Stem: *Dignampt*-.

***Digrammus* Fauvel, 1900a**: 123. Current status: valid genus in STAPHYLINIDAE: ALEOCHARINAE: DIGRAMMINI.

Type species: *Digrammusmiricollis* Fauvel, 1900, by monotypy.

Family-group name: DIGRAMMINI Fauvel, 1900. Stem: *Digramm*-.

***Dihammatus* C.O. Waterhouse, 1880a**: 29. Current status: valid genus in LYCIDAE: METRIORRHYNCHINAE: DIHAMMATINI.

Type species: *Dihammatuscribripennis* C.O. Waterhouse, 1880, by subsequent designation (Bourgeois 1891: 349).

Family-group name: DIHAMMATINI Bocák & Bocáková, 2008. Stem: *Dihammat*-.

***Dila* Fischer von Waldheim, 1844**: 111. Current status: valid genus in TENEBRIONIDAE: BLAPTINAE: BLAPTINI: BLAPTINA.

Type species: *Blaps laevicollis* Gebler, 1841, by subsequent designation (Motschulsky 1860: 530).

Family-group name: DILINA Ren in Ren et al., 2016: 85, 431. Stem: *Dil*-.

***Dilolycus* Kleine, 1926**: 186. Current status: junior synonym of *Metriorrhynchus* Gemminger, 1869 in LYCIDAE: METRIORRHYNCHINAE: METRIORRHYNCHINI: METRIORRHYNCHINA.

Type species: *Dilolycuslamellatus* Kleine, 1926, by original designation.

Family-group name: DILOLYCINAE Kleine, 1926. Stem: *Dilolyc*-.

***Dilophochila* H.W. Bates, 1888**: 261. Current status: valid genus in SCARABAEIDAE: RUTELINAE: ANOMALINI: ANOMALINA.

Type species: *Dilophochilabolacoides* H.W. Bates, 1888, by monotypy.

Family-group name: DILOPHOCHILINA Ohaus, 1918. Stem: *Dilophochil*-.

***Dilophotes* C.O. Waterhouse, 1880a**: 75. Current status: valid genus in LYCIDAE: METRIORRHYNCHINAE: DILOPHOTINI.

Type species: *Lycusexilis* C.O. Waterhouse, 1878, by original designation.

Family-group name: DILOPHOTINAE Kleine, 1929. Stem: *Dilophot*-.

***Dima* Charpentier, 1825**: 191. Current status: valid genus in ELATERIDAE: DENDROMETRINAE: DIMINI.

Type species: *Dimaelateroides* Charpentier, 1825, by monotypy.

Family-group name: DIMITES Candèze, 1863. Stem: *Dim*-.

***Dimerometopus* Célis, 1970**: 245. Current status: valid genus in STAPHYLINIDAE: PSELAPHINAE: CLAVIGERITAE: CLAVIGERINI.

Type species: *Hadrophorusbicephalus* Jeannel, 1954, by original designation.

Family-group name: DIMEROMETOPINI Célis, 1970. Stem: *Dimerometop*-.

***Dimerus* Fiori, 1900**: 103. Current status: junior synonym of *Octomicrus* L.W. Schaufuss, 1877 in STAPHYLINIDAE: PSELAPHINAE: EUPLECTITAE: DIMERINI.

Type species: *Dimerusstaphylinoides* Fiori, 1900, by monotypy.

Family-group name: DIMERINI Raffray, 1908. Stem: *Dimer*-.

***Dimetrota* Mulsant & Rey, 1873**: 165. Current status: valid subgenus of *Atheta* C.G. Thomson, 1858 in STAPHYLINIDAE: ALEOCHARINAE: ATHETINI: ATHETINA.

Type species: *Homalotatristicula* Mulsant & Rey, 1873 (= *Homalotacadaverina* Brisout de Barneville, 1860), by subsequent designation (Blackwelder 1952: 125).

Family-group name: DIMETROTAE Seevers, 1978. Stem: *Dimetrot*-.

***Dimonomera* Cameron, 1933**: 103. Current status: valid genus in STAPHYLINIDAE: ALEOCHARINAE: MYLLAENINI.

Type species: *Dimonomeraindica* Cameron, 1933, by monotypy.

Family-group name: DIMONOMERINI Cameron, 1933. Stem: *Dimonomer*-.

***Dinapate* G.H. Horn, 1886**: 1. Current status: valid genus in BOSTRICHIDAE: BOSTRICHINAE: APATINI.

Type species: *Dinapatewrightii* G.H. Horn, 1886, by monotypy.

Family-group name: DINAPATINAE Lesne, 1910. Stem: *Dinapat*-.

***Dinarda* Leach in Samouelle, 1819**: 177. Current status: valid genus in STAPHYLINIDAE: ALEOCHARINAE: OXYPODINI: DINARDINA.

Type species: *Lomechusadentata* Gravenhorst, 1806, by original designation.

Family-group name: DINARDAIRES Mulsant & Rey, 1873. Stem: *Dinard*-.

***Dinardopsis*Bruch, 1917**: 257. Current status: valid genus in STAPHYLINIDAE: ALEOCHARINAE: HOMALOTINI: DINARDOPSINA.

Type species: *Dinardopsissolenopsidicola*Bruch, 1917, by original designation.

Family-group name: DINARDOPSES Bernhauer & Scheerpeltz, 1926. Stem: *Dinardops*-.

***Dineutus* W.S. MacLeay, 1825**: 30. Current status: valid genus in GYRINIDAE: GYRININAE: DINEUTINI: DINEUTINA.

Type species: *Dineutuspolitus* W.S. MacLeay, 1825, by monotypy.

Family-group name: DINEUTIDES E. Desmarest, 1851. Stem: *Dineut*-.

***Dinoderus* Stephens, 1830**: 349, 352. Current status: valid genus in BOSTRICHIDAE: DINODERINAE.

Type species: *Dinoderusocellaris* Stephens, 1830, by monotypy. Note: two species were originally listed by Stephens (1830) but one of them is conditionally included and therefore cannot be considered as an originally included species (ICZN 1999a, Article 67.2.5).

Family-group name: DINODERINA C.G. Thomson, 1863. Stem: *Dinoder*-.

***Dinomorphus* Perty, 1832**: 71. Current status: valid genus in CURCULIONIDAE: MOLYTINAE: DINOMORPHINI.

Type species: *Dinomorphuspimelioides* Perty, 1832, by monotypy.

Family-group name: DINOMORPHIDES Lacordaire, 1863. Stem: *Dinomorph*-.

***Dinorhopala* Pascoe, 1860b**: 61. Current status: valid genus in CURCULIONIDAE: CURCULIONINAE: RHAMPHINI: DINORHOPALINA.

Type species: *Dinorhopalaspinosa* Pascoe, 1860, by monotypy.

Family-group name: DINORRHOPALINA Voss, 1936. Stem: *Dinorhopal*-.

***Diocalandra* Faust, 1894a**: 353. Current status: valid genus in CURCULIONIDAE: DRYOPHTHORINAE: LITOSOMINI: DIOCALANDRINA.

Type species: *Calandrafrumenti* Fabricius, 1801, by monotypy.

Family-group name: DIOCALANDRINI Zimmerman, 1993. Stem: *Diocalandr*-.

***Diochus* Erichson, 1839b**: 300. Current status: valid genus in STAPHYLINIDAE: STAPHYLININAE: DIOCHINI.

Type species: *Diochusnanus* Erichson, 1839, by monotypy.

Family-group name: DIOCHI Casey, 1906. Stem: *Dioch*-.

***Diodesma* Latreille, 1829b**: 97. Current status: valid genus in ZOPHERIDAE: COLYDIINAE: SYNCHITINI.

Type species: *Diodesmasubterranea* Latreille, 1829, by monotypy.

Family-group name: DIODESMINI Reitter, 1911. Stem: *Diodesmat*-.

***Diomus* Mulsant, 1850**: 951. Current status: valid genus in COCCINELLIDAE: COCCINELLINAE: DIOMINI.

Type species: *Coccinellathoracica* Fabricius, 1801, by subsequent designation (Korschefsky 1931: 116).

Family-group name: DIOMINI Gordon, 1999. Stem: *Diom*-.

***Diorus* A. White, 1853**: 135. Current status: valid genus in CERAMBYCIDAE: CERAMBYCINAE: DIORINI.

Type species: *Diorusbiapiculatus* A. White, 1853, by monotypy.

Family-group name: DIORINAE Lane, 1950. Stem: *Dior*-.

***Dioryche* W.S. MacLeay, 1825**: 21. Current status: valid genus in CARABIDAE: HARPALINAE: HARPALINI: HARPALINA.

Type species: *Harpalustorta* W.S. MacLeay, 1825, by monotypy.

Family-group name: DIORYCHI Csiki, 1932. Stem: *Diorych*-.

***Diorymerus* Schönherr, 1825**: 586. Current status: valid genus in CURCULIONIDAE: BARIDINAE: APOSTASIMERINI: DIORYMERINA.

Type species: *Orobitisaltus* Germar, 1823, by original designation.

Family-group name: DIORYMERIDES Jekel, 1865. Stem: *Diorymer*-.

***Dipelicus* Hope, 1843a**: 62. Current status: valid genus in SCARABAEIDAE: DYNASTINAE: PENTODONTINI: DIPELICINA.

Type species: *Dipelicuscantori* Hope, 1843, by monotypy.

Family-group name: DIPELICINA Carne, 1957. Stem: *Dipelic*-.

***Diphaulaca* Chevrolat in Dejean, 1836a**: 388. Current status: valid genus in CHRYSOMELIDAE: GALERUCINAE: ALTICINI.

Type species: *Alticaaulica* G.-A. Olivier, 1808, by subsequent designation (Chevrolat 1844: 46).

Family-group name: DIPHAULACINI Bechyné & Špringlová de Bechyné, 1975. Stem: *Diphaulac*-.

***Diphucephala* Dejean, 1821**: 58. Current status: valid genus in SCARABAEIDAE: SERICINAE: DIPHUCEPHALINI.

Type species: *Melolonthasericea* W. Kirby, 1818, by monotypy.

Family-group name: DIPHUCÉPHALITES Laporte, 1840. Stem: *Diphucephal*-.

***Diphycerus* Deyrolle & Fairmaire, 1878**: 100. Current status: valid genus in SCARABAEIDAE: MELOLONTHINAE: DIPHYCERINI.

Type species: *Diphycerusdavidis* Fairmaire, 1878, by monotypy.

Family-group name: DIPHYCERINI S.I. Medvedev, 1952. Stem: *Diphycer*-.

***Diphyllostoma* Fall, 1901**: 324. Current status: valid genus in DIPHYLLOSTOMATIDAE. Note: new replacement name for *Phyllostoma* Fall, 1901.

Type species: *Phyllostomafimbriatum* Fall, 1901, by monotypy (type fixation for *Phyllostoma* Fall).

Family-group name: DIPHYLLOSTOMATIDAE Holloway, 1972. Stem: *Diphyllostomat*-.

***Diplocyrtus* Quedenfeldt, 1887**: 257. Current status: valid genus in TENEBRIONIDAE: TENEBRIONINAE: APOCRYPHINI.

Type species: *Diplocyrtusfloccosus* Quedenfeldt, 1887, by monotypy.

Family-group name: DIPLOCYRTHINI Escalera, 1914. Stem: *Diplocyrt*-.

***Diplognatha* Gory & Percheron, 1833**: 19, 31, 51. Current status: valid genus in SCARABAEIDAE: CETONIINAE: DIPLOGNATHINI. Note: the spelling *Diplognata* used on page 51 was corrected to *Diplognatha* in the “Errata” of the same work.

Type species: *Scarabaeusgagates* Forster, 1771, by subsequent designation (Duponchel 1843a: 360).

Family-group name: DIPLOGNATHIDAE Burmeister, 1842. Stem: *Diplognath*-.

***Diplotaxis* W. Kirby, 1837**: 129. Current status: valid genus in SCARABAEIDAE: MELOLONTHINAE: DIPLOTAXINI.

Type species: *Diplotaxistristis* W. Kirby, 1837, by monotypy.

Family-group name: DIPLOTAXIDAE W. Kirby, 1837. Stem: *Diplotax*-.

***Dircaea* Fabricius, 1798**: 6. Current status: valid genus in MELANDRYIDAE: MELANDRYINAE: DIRCAEINI.

Type species: *Dircaeaquadriguttata* Fabricius, 1798 (= *Hypulusquadriguttatus* Paykull, 1798), by subsequent designation (Blanchard in Audouin et al. 1844: pl. 52). Note: based on currently available dates of publication, it is possible that Fabricius’ species, issued by 29 April 1798 (Bousquet 2017a: 107), may be older than Paykull’s species, issued by 20 June 1798 (Bousquet 2016: 409) although the date of the introduction in Paykull’s work predates that of Fabricius’ work by about two weeks; at this time we prefer to maintain the status quo.

Family-group name: DIRCAEIDAE W. Kirby, 1837. Stem: *Dircae*-.

***Dirhagus* Latreille, 1834**: 130. Current status: junior synonym of *Microrhagus* Dejean, 1833 in EUCNEMIDAE: MELASINAE: DIRHAGINI.

Type species [suggested]: *Elaterpygmaeus* Fabricius, 1792, by subsequent designation (Muona 1987: 82). Note: the type species for this genus is usually given as “*Elaterpygmaeus* Fabricius, 1792, by monotypy”; however, contrary to comments in Muona (1987: 82), the species *Dirhaguslongulus* was made available by Latreille (1834: 131) and unambiguously combined with *Dirhagus*, as such, it should be treated as a second species originally included in *Dirhagus*; additionally, *D.longulus*, the identity of which needs further investigation, was selected as type species of *Dirhagus* by Guérin- Méneville (1843b: 181).

Family-group name: DIRRHAGINI Reitter, 1911. Stem: *Dirhag*-.

***Dirotognathus* G.H. Horn in J.L. LeConte and G.H. Horn, 1876**: 79. Current status: valid genus in CURCULIONIDAE: ENTIMINAE: ENTIMITAE: BYRSOPAGINI: BYRSOPAGINA.

Type species: *Dirotognathussordidus* G.H. Horn, 1876, by monotypy.

Family-group name: DIROTOGNATHINI G.H. Horn, 1876. Stem: *Dirotognath*-.

***Disarthricerus* Raffray, 1895**: 78. Current status: valid genus in STAPHYLINIDAE: PSELAPHINAE: CLAVIGERITAE: DISARTHRICERINI.

Type species: *Disarthricerusinteger* Raffray, 1895, by monotypy.

Family-group name: DIARTHRICERINI Jeannel, 1949. Stem: *Disarthricer*-.

***Discheramocephalus* C. Johnson, 2007b**: 223. Current status: valid genus in PTILIIDAE: PTILIINAE: DISCHERAMOCEPHALINI.

Type species: *Discheramocephalussemisulcatus* C. Johnson, 2007, by original designation.

Family-group name: DISCHERAMOCEPHALINI Grebennikov, 2009. Stem: *Discheramocephal*-.

***Discoloma* Erichson, 1845a**: 292. Current status: valid genus in DISCOLOMATIDAE: DISCOLOMATINAE.

Type species: *Discolomaparmula* Erichson, 1845, by monotypy.

Family-group name: DISCOLOMIDAE G.H. Horn, 1878. Stem: *Discolomat*-.

***Discoptera* Semenov, 1889a**: 396. Current status: valid genus in CARABIDAE: HARPALINAE: CORSYRINI.

Type species: *Discopterakomarowi* Semenov, 1889, by subsequent designation (Kryzhanovskij et al. 1995: 160).

Family-group name: DISCOPTERINI Jedlička, 1941. Stem: *Discopter*-.

***Discotenes* Labram & Imhoff, 1841**: [Heft 8]. Current status: valid genus in ANTHRIBIDAE: ANTHRIBINAE: DISCOTENINI.

Type species: *Discotenescoelebs* Labram & Imhoff, 1841, by monotypy.

Family-group name: DISCOTÉNIDES Lacordaire, 1865. Stem: *Discoten*-.

***Discotoma* Mulsant, 1850**: 215. Current status: valid genus in COCCINELLIDAE: COCCINELLINAE: COCCINELLINI.

Type species: *Discotomaornata* Mulsant, 1850, by monotypy.

Family-group name: DISCOTOMAIRES Mulsant, 1850. Stem: *Discotom*-.

***Disker* Dreux & Voisin, 1987**: 314. Current status: valid genus in CURCULIONIDAE: ENTIMINAE: OTIORHYNCHITAE: ECTEMNORHININI.

Type species: *Antarctonesiodestenuicornis* Jeannel, 1940, by original designation.

Family-group name: DISKERINI Voisin, Chapelin-Viscardi, Ponel & Rapp, 2017: 134. Stem: *Disker*-.

***Dismorpha* Gistel, 1848b**: x. Current status: valid genus in BUPRESTIDAE: AGRILINAE: CORAEBINI: DISMORPHINA. Note: new replacement name for *Stenogaster* Solier, 1833.

Type species: *Buprestisatomaria* Fabricius, 1792, by monotypy (type fixation for *Stenogaster* Solier).

Family-group name: DISMORPHINA Cobos, 1990. Stem: *Dismorph*-.

***Disonycha* Chevrolat in Dejean, 1836a**: 390. Current status: valid genus in CHRYSOMELIDAE: GALERUCINAE: ALTICINI.

Type species: *Crioceriscollata* Fabricius, 1801, by subsequent designation (Blake 1933: 1).

Family-group name: DISONYCHAE J.L. LeConte & G.H. Horn, 1883. Stem: *Disonych*-.

***Disphericus* G.R. Waterhouse, 1841b**: 298. Current status: valid genus in CARABIDAE: HARPALINAE: PELECIINI: PELECIINA.

Type species: *Disphericusgambianus* G.R. Waterhouse, 1841, by monotypy.

Family-group name: DISPHAERICINI Sloane, 1923. Stem: *Dispheric*-.

***Dissonomus* Jacquelin du Val, 1861**: 280. Current status: valid genus in TENEBRIONIDAE: TENEBRIONINAE: DISSONOMINI. Note: new replacement name for *Heterophylus* Mulsant & Rey, 1859.

Type species: *Heliopatespicipes* Faldermann, 1837, by original designation. Note: the type fixation for for this genus was erroneously given as “by subsequent designation (Gebien 1938b: 397)” in Bouchard et al. (2021: 166).

Family-group name: DISSONOMINI G.S. Medvedev, 1968. Stem: *Dissonom*-.

***Distenia* Lepeletier & Audinet-Serville, 1828**: 485. Current status: valid genus in DISTENIIDAE: DISTENIINI.

Type species: *Disteniacolumbina* Lepeletier & Audinet-Serville, 1828, by monotypy.

Family-group name: DISTENITAE J. Thomson, 1861. Stem: *Disteni*-.

†***Distenorrhinoides* Gratshev & Zherikhin, 2000a**: 39. Current status: valid genus in NEMONYCHIDAE: †EOBELINAE: †DISTENORRHINOIDINI. Note: Lower Cretaceous (Spain).

Type species: †*Distenorrhinoidessimulator* Gratshev & Zherikhin, 2000, by original designation.

Family-group name: †DISTENORRHINOIDINI Legalov, 2009. Stem: *Distenorrhinoid*-.

†***Distenorrhinus* Arnoldi, 1977**: 170. Current status: valid genus in NEMONYCHIDAE: †BRENTHORRHININAE: †DISTENORRHININI. Note: Upper Jurassic (Kazakhstan).

Type species: †*Distenorrhinusangulatus* Arnoldi, 1977, by original designation.

Family-group name: †DISTENORRHININI Arnoldi, 1977. Stem: *Distenorrhin*-.

***Disterna* J. Thomson, 1864**: 88. Current status: valid genus in CERAMBYCIDAE: LAMIINAE: ZYGOCERINI.

Type species: *Zygocerabifasciata* Pascoe, 1859, by monotypy.

Family-group name: DISTERNINAE Pascoe, 1871. Stem: *Distern*-.

***Distichocera* W. Kirby, 1819**: 471. Current status: valid genus in CERAMBYCIDAE: CERAMBYCINAE: DISTICHOCERINI.

Type species: *Distichoceramaculicollis* W. Kirby, 1819, by monotypy.

Family-group name: DISTICHOCERINAE Pascoe, 1867. Stem: *Distichocer*-.

***Distipsidera* Westwood, 1836**: 251. Current status: valid genus in CICINDELIDAE: CICINDELINI: IRESIINA.

Type species: *Distipsideraundulata* Westwood, 1836, by monotypy.

Family-group name: DISTYPSIDERINI W. Horn, 1893. Stem: *Distipsider*-.

***Ditomus* Bonelli, 1810**: Tab. Syn. Current status: valid genus in CARABIDAE: HARPALINAE: HARPALINI: DITOMINA.

Type species: *Scaritescalydonius* Rossi, 1790, by subsequent designation (Hope 1838a: 81). Note: no species were originally included in this genus by Bonelli (1810); the first ones were the five species listed by Latreille (1816: 190) without question marks under the new replacement name *Aristus* Latreille, 1816 (see Madge 1975: 582).

Family-group name: DITOMICI Bonelli, 1810. Stem: *Ditom*-.

***Ditropalia* Casey, 1906**: 263. Current status: junior synonym of *Bolitochara* Mannerheim, 1830 in STAPHYLINIDAE: ALEOCHARINAE: HOMALOTINI: BOLITOCHARINA.

Type species: *Bolitocharabella* Märkel, 1844, by subsequent designation (Fenyes 1919: 22).

Family-group name: DITROPALIINI Hatch, 1957. Stem: *Ditropali*-.

***Ditylus* Fischer, 1817**: 469. Current status: valid genus in OEDEMERIDAE: OEDEMERINAE: DITYLINI.

Type species: *Helopslaevis* Fabricius, 1787, by subsequent monotypy (Dejean 1821: 72). Note: *Ditylushelopioides* Fischer is often listed as type species of this genus (e.g., Švihla 2008: 361; Kubisz and Iwan 2020: 487) but that species was not described until 1821 [November] (not in 1817 as noted, but after Dejean (1821 [May])) and therefore is not an originally included species.

Family-group name: DITYLATES Mulsant, 1858. Stem: *Dityl*-.

***Docohammus* Aurivillius, 1908**: 217. Current status: valid genus in CERAMBYCIDAE: LAMIINAE: LAMIINI.

Type species: *Docohammusbennigseni* Aurivillius, 1908, by monotypy.

Family-group name: DOCOHAMMIDI E.S. Dillon & L.S. Dillon, 1959. Stem: *Docohamm*-.

***Dodecosis* H.W. Bates, 1867**: 27. Current status: valid genus in CERAMBYCIDAE: CERAMBYCINAE: DODECOSINI.

Type species: *Dodecosissaperdina* H.W. Bates, 1867, by monotypy.

Family-group name: DODECOSINI Aurivillius, 1912. Stem: *Dodecos*-.

***Dolicaon* Laporte, 1834c**: 119. Current status: valid genus in STAPHYLINIDAE: PAEDERINAE: PAEDERINI: DOLICAONINA.

Type species: *Dolicaonlathrobioides* Laporte, 1834, by monotypy.

Family-group name: DOLICAONES Casey, 1905. Stem: *Dolicaon*-.

***Dolichus* Bonelli, 1810**: Tab. Syn. Current status: valid genus in CARABIDAE: HARPALINAE: SPHODRINI: DOLICHINA.

Type species: *Carabusflavicornis* Fabricius, 1787 (= *Carabushalensis* Schaller, 1783), by subsequent designation (Latreille 1817a: 532). Note: no species were originally included in this genus by Bonelli (1810); the first ones were the two species listed in Latreille (1816: 194).

Family-group name: DOLICHIENS Brullé, 1834. Stem: *Dolich*-.

***Doliema* Pascoe, 1860b**: 50. Current status: junior synonym of *Adelina* Dejean, 1835 in TENEBRIONIDAE: DIAPERINAE: DIAPERINI: ADELININA.

Type species: *Doliemaplatisoides* Pascoe, 1860, by monotypy.

Family-group name: DOLIEMINI Reitter, 1917. Stem: *Doliem*-.

***Dolocerus* Mulsant, 1863**: 223, 230. Current status: junior synonym of *Brachypteroma* Heyden, 1863 in CERAMBYCIDAE: CERAMBYCINAE: BRACHYPTEROMINI.

Type species: *Dolocerusreichii* Mulsant, 1863, by monotypy.

Family-group name: DOLOCERINI Özdikmen in Albayati et al., 2016: 665. Stem: *Dolocer*-. Note: Özdikmen in Albayati et al. (2016: 665) proposed DOLOCERINI as a replacement for BRACHYPTEROMINI Sama, 2008 because the type genus of the latter name, *Brachypteroma* Heyden, 1863, was considered a junior synonym of *Dolocerus* Mulsant, 1863; however, Heyden’s *Brachypteroma* was published in June 1863 while Mulsant’s name was issued late in 1863 as it was recorded on 4 January 1864 by the ‘Bibliographie de la France’ (Bousquet 2016: 377); also, such nomenclatural action is contrary to Article 40.1 (ICZN 1999a), which states that when the name of a type genus of a nominal family-group taxon is considered to be a junior synonym of the name of another nominal genus, the family-group name is not to be replaced on that account alone.

***Dolosus* Dajoz, 1963**: 91. Current status: junior synonym of *Thyroderus* Sharp, 1885 in CERYLONIDAE: CERYLONINAE.

Type species: *Dolosusleleupi* Dajoz, 1963, by original designation.

Family-group name: DOLOSIDAE Dajoz, 1963. Stem: *Dolos*-.

†***Dominibrentus* Poinar, 2009a**: 51. Current status: valid genus in BRENTIDAE: BRENTINAE: TAPHRODERINI. Note: Miocene (Dominican amber).

Type species: †*Dominibrentusleptus* Poinar, 2009, by original designation.

Family-group name: †DOMINIBRENTINI Poinar, 2009. Stem: *Dominibrent*-.

***Dominopteron* Kazantsev, 2013a**: 15. Current status: valid genus in LYCIDAE: LYCINAE: LEPTOLYCINI: DOMINOPTERINA.

Type species: *Dominopteronhispaniolum* Kazantsev, 2013, by original designation.

Family-group name: DOMINOPTERINI Kazantsev, 2013: 28. Stem: *Dominopter*-.

***Donacia* Fabricius, 1775**: 195. Current status: valid genus in CHRYSOMELIDAE: DONACIINAE: DONACIINI.

Type species: *Donaciacrassipes* Fabricius, 1775, by subsequent designation (Curtis 1834: pl. 494).

Family-group name: DONACIADAE W. Kirby, 1837. Stem: *Donaci*-.

***Dorcacerus* Dejean, 1821**: 105. Current status: valid genus in CERAMBYCIDAE: CERAMBYCINAE: TRACHYDERINI: TRACHYDERINA.

Type species: *Cerambyxbarbatus* Fabricius, 1775, by monotypy.

Family-group name: DORCACERINAE H.W. Bates, 1870. Stem: *Dorcacer*-.

***Dorcadion* Dalman in Schönherr, 1817**: 397. Current status: valid genus in CERAMBYCIDAE: LAMIINAE: DORCADIONINI.

Type species [suggested]: *Cerambyxglicyrrhizae* Pallas, 1773, by subsequent designation (J. Thomson 1864: 43). Note: as pointed out by Bousquet (2008a: 619), the valid type species fixation is that of Blanchard in Audouin et al. (1841: pl. 68), who selected *Cerambyxfuliginator* Linnaeus, 1758, but that species is the type species of the genus *Iberodorcadion* Breuning, 1943; an application to the Commission is necessary to fix *Cerambyxglicyrrhizae* Pallas, 1773 as the type species of *Dorcadion* Dalman, 1817 to conserve current usage (e.g., Danilevsky 2020: 337).

Family-group name: DORCADIONINAE Swainson, 1840. Stem: *Dorcadion*-.

***Dorcadodium* Gistel, 1856**: 263, 406. Status: senior synonym of the subgenus Carinatodorcadion Breuning, 1943 of the genus *Dorcadion* Dalman, 1817 in CERAMBYCIDAE: LAMIINAE: DORCADIONINI. Note: two spellings were originally used for this new taxon, including *Dorcadodion* (pp. 293, 296, 470); we act as First Revisers and selected *Dorcadodium* as the correct original spelling; some authors (e.g., Sama 2002: 88, Özdikmen 2012: 56) treat *Dorcadodium* Gistel, 1856 as a nomen oblitum, however, they did not specifically give evidence that the conditions of Article 23.9.1.2 were met (see such requirement in Article 23.9.2, ICZN 1999a); an application to the Commission is necessary to maintain *Carinatodorcadion* Breuning, 1943 as valid.

Type species: *Lamiamorio* Fabricius, 1787 (= *Cerambyxaethiops* Scopoli, 1763), by subsequent designation (Vives and Alonso-Zarazaga 2000: 659).

Family-group name: DORCADODIIDAE Gistel, 1856. Stem: *Dorcadodi*-.

***Dorcaschema* Chevrolat, 1844**: 110. Current status: valid genus in CERAMBYCIDAE: LAMIINAE: DORCASCHEMATINI.

Type species: *Dorcaschemaleptocera* Chevrolat, 1844 (= *Saperdaalternata* Say, 1824), by monotypy.

Family-group name: DORCASCHEMITAE J. Thomson, 1860. Stem: *Dorcaschemat*-.

***Dorcasomus* Audinet-Serville, 1834b**: 12. Current status: valid genus in CERAMBYCIDAE: CERAMBYCINAE: DORCASOMINI.

Type species: *Cerambyxebulinus* Fabricius, 1787, by monotypy.

Family-group name: DORCASOMIDES Lacordaire, 1868. Stem: *Dorcasom*-.

***Dorcatoma* Herbst, 1791**: 103. Current status: valid genus in PTINIDAE: DORCATOMINAE. Note: the original spelling *Dorkatoma* was changed to *Dorcatoma* and *Dorcatoma* Herbst, 1791 was placed on the Official List of Generic Names in Zoology (ICZN 1995c, as *Dorcatoma* Herbst, 1792).

Type species: *Dorcatomadresdensis* Herbst, 1791, by monotypy (see ICZN 1995c, as *Dorkatomadresdensis* Herbst, 1792). Note: name placed on the Official List of Specific Names in Zoology (ICZN 1995c).

Family-group name: DORCATOMINA C.G. Thomson, 1859. Stem: *Dorcatom*-.

***Dorcus* W.S. MacLeay, 1819**: 111. Current status: valid genus in LUCANIDAE: LUCANINAE: DORCINI.

Type species: *Scarabaeusparallelipipedus* Linnaeus, 1758, by subsequent designation (Thon 1836: 87).

Family-group name: DORCIDAE Parry, 1864. Stem: *Dorc*-.

***Dorylogaster* Wasmann, 1904**: 625. Current status: valid genus in STAPHYLINIDAE: ALEOCHARINAE: DORYLOGASTRINI.

Type species: *Dorylogasterlongipes* Wasmanm, 1904, by monotypy.

Family-group name: DORYLOGASTRINI Wasmann, 1916. Stem: *Dorylogastr*-.

***Dorylomimus* Wasmann, 1904**: 620. Current status: valid genus in STAPHYLINIDAE: ALEOCHARINAE: DORYLOMIMINI.

Type species: *Dorylomimuskohli* Wasmann, 1904, by monotypy.

Family-group name: DORYLOMIMINI Wasmann, 1916. Stem: *Dorylomim*-.

***Dorylophila* Wasmann, 1904**: 632. Current status: valid genus in STAPHYLINIDAE: ALEOCHARINAE: MIMANOMMATINI: DORYLOPHILINA.

Type species: *Dorylophilarotundicollis* Wasmann, 1904, by monotypy.

Family-group name: DORYLOPHILINI Fenyes, 1921. Stem: *Dorylophil*-.

***Dorynota* Chevrolat in Dejean, 1836a**: 370. Current status: valid genus in CHRYSOMELIDAE: CASSIDINAE: DORYNOTINI.

Type species: *Cassidabidens* Fabricius, 1781, by subsequent designation (Duponchel and Chevrolat 1842b: 211).

Family-group name: DORYNOTINI Monrós & Viana, 1949. Stem: *Dorynot*-.

***Doryphora* Illiger, 1807b**: 331. Current status: valid genus in CHRYSOMELIDAE: CHRYSOMELINAE: CHRYSOMELINI.

Type species: *Chrysomelapunctatissima* G.-A. Olivier, 1791, by subsequent designation (Latreille 1810: 432).

Family-group name: DORYPHORES Motschulsky, 1860. Stem: *Doryphor*-. Note: junior homonym of DORYPHORI Fitzinger, 1843 (type genus *Doryphorus* Cuvier, 1829) in Reptilia.

***Doryscelis* Dejean, 1836b**: 189. Current status: valid genus in SCARABAEIDAE: CETONIINAE: STENOTARSIINI: DORYSCELINA.

Type species: *Cetoniacalcarata* Klug, 1833, by monotypy.

Family-group name: DORYSCELINA Schenkling, 1921. Stem: *Doryscel*-.

***Dorytomus* Germar, 1817b**: 340. Current status: valid genus in CURCULIONIDAE: CURCULIONINAE: ELLESCINI.

Type species: *Curculiovorax* Fabricius, 1792 (= *Curculiolongimanus* Forster, 1771), by subsequent designation (Westwood 1838c: 37).

Family-group name: DORYTOMINI Bedel, 1886. Stem: *Dorytom*-.

***Doydirhynchus* Dejean, 1821**: 79. Current status: valid genus in CIMBERIDIDAE: DOYDIRHYNCHINI.

Type species: *Rhynchitesaustriacus* G.-A. Olivier, 1807, by monotypy.

Family-group name: DOYDIRHYNCHOIDEA Pierce, 1916. Stem: *Doydirhynch*-.

***Drapetes* Dejean, 1821**: 34. Current status: valid genus in ELATERIDAE: LISSOMINAE: LISSOMINI.

Type species: *Elaterequestris* Fabricius, 1798 (= *Elatermordelloides* Host, 1789), by monotypy.

Family-group name: DRAPETINI J.L. LeConte, 1863. Stem: *Drapet*-. Note: the name DRAPETINI Collin, 1961 (type genus *Drapetis* Meigen, 1822) in Diptera has been used with the correct stem (as DRAPETIDINI) in recent literature (e.g., Sinclair et al. 2022).

***Drasterius* Eschscholtz, 1829a**: 33. Current status: valid genus in ELATERIDAE: AGRYPNINAE: OOPHORINI. Note: name placed on the Official List of Generic Names in Zoology (ICZN 1987b).

Type species: *Elaterbimaculatus* Rossi, 1790, by subsequent designation (Curtis 1838a: 694, see Sánchez-Ruiz 1996: 48). Note: name placed on the Official List of Specific Names in Zoology (ICZN 1987b); the subsequent designation was attributed to Westwood (1838c: 26) in ICZN (1987b); however, it was published later than the fixation by (Curtis 1838a: 694) and as a result, an application to the Commission is necessary to change the entry on the Official List of Generic Names in Zoology.

Family-group name: DRASTERIINI Houlbert, 1912. Stem: *Drasteri*-. Note: senior homonym of DRASTERIINI Wiltshire, 1976 (type genus *Drasteria* Hübner, 1818) in Lepidoptera.

***Drepanocerus* W. Kirby, 1828**: 521. Current status: valid genus in SCARABAEIDAE: SCARABAEINAE: ONITICELLINI: DREPANOCERINA.

Type species: *Drepanoceruskirbyi* Kirby, 1828 [as *kirbii*], by monotypy.

Family-group name: DRÈPANOCÉRIDES Van Lansberge, 1875. Stem: *Drepanocer*-.

***Drepanoxenus* Kistner & Watson, 1972**: 2. Current status: valid genus in STAPHYLINIDAE: ALEOCHARINAE: DREPANOXENINI.

Type species: *Drepanoxenusphysogastra* Kistner & Watson, 1972, by original designation.

Family-group name: DREPANOXENINI Kistner & Watson, 1972. Stem: *Drepanoxen*-.

***Drilonius* Kiesenwetter, 1874**: 282. Current status: valid genus in OMETHIDAE: DRILONIINAE.

Type species: *Driloniusstriatulus* Kiesenwetter, 1874, by monotypy.

Family-group name: DRILONIINAE Crowson, 1972. Stem: *Driloni*-.

***Drilus* G.-A. Olivier, 1790a**: [23] 1. Current status: valid genus in ELATERIDAE: AGRYPNINAE: DRILINI.

Type species: *Ptilinusflavescens* Geoffroy, 1785, by subsequent designation (Latreille 1810: 426).

Family-group name: DRILITES Blanchard, 1845. Stem: *Dril*-.

***Drimostoma* Dejean, 1831**: 694, 745. Current status: junior synonym of *Caelostomus* W.S. MacLeay, 1825 in CARABIDAE: HARPALINAE: DRIMOSTOMATINI.

Type species: *Drimostomastriatocolle* Dejean, 1831, by subsequent designation (Drapiez 1838: 579).

Family-group name: DRIMOSTOMIDAS Chaudoir, 1872. Stem: *Drimostomat*-.

***Dromaeolus* Kiesenwetter, 1858**: 197. Current status: valid genus in EUCNEMIDAE: MACRAULACINAE: MACRAULACINI.

Type species: *Eucnemisbarnabita* Comolli, 1837, by monotypy. Note: the author of the type species is usually given as “A. Villa & G.B. Villa” in the literature (e.g., Muona 2007: 86) because the name “Villa” is attached to the species’ name in Comolli (1837: 15); however, the contents of Comolli’s paper do not reveal that the brothers Villa were responsible for satisfying the criteria of availability (ICZN 1999a, Article 50.1.1).

Family-group name: DROMAEOLINI Beaulieu, 1919. Stem: *Dromaeol*-. Note: as pointed out by Bouchard and Bousquet (2020a: 87) the names FORNACINI Beaulieu, 1919 and DROMAEOLINI Beaulieu, 1919 take precedence over MACRAULACINAE/-INI Fleutiaux, 1923 and an application to the Commission is necessary to preserve usage of MACRAULACINAE/-INI as valid.

***Dromica* Dejean, 1826**: 434. Current status: valid genus in CICINDELIDAE: CICINDELINI: DROMICINA.

Type species: *Cicindelacoarctata* Dejean, 1822, by monotypy.

Family-group name: DROMICITAE J. Thomson, 1859. Stem: *Dromic*-. Note: senior homonym of DROMICINAE Cope, 1896 (type genus *Dromicus* Cocteau & Bibron, 1843) in Reptilia.

***Dromius* Bonelli, 1810**: Tab. Syn. Current status: valid genus in CARABIDAE: HARPALINAE: LEBIINI: DROMIUSINA. Note: name placed on the Official List of Generic Names in Zoology (ICZN 2006b).

Type species: *Carabusquadrimaculatus* Linnaeus, 1758, by subsequent designation (Hope 1838a: 63; see ICZN 2006b). Note: name placed on the Official List of Specific Names in Zoology (ICZN 2006b).

Family-group name: DROMIEI Bonelli, 1810. Stem: *Dromius*- (ICZN 2006b).

***Drosochrus* Erichson, 1843b**: 243. Current status: valid genus in TENEBRIONIDAE: BLAPTINAE: PEDININI: HELOPININA.

Type species: *Drosochrusbrunnipes* Erichson, 1843, by subsequent designation (Gebien 1943: 910).

Family-group name: DROSOCHRINI C. Koch, 1958. Stem: *Drosochr*-.

***Dryobia* Gistel, 1856**: 368. Current status: junior synonym of *Dryophilus* Chevrolat, 1832 in PTINIDAE: DRYOPHILINAE: DRYOPHILINI.

Type species: *Dryophilusanobioides* Chevrolat, 1832, by **present designation**.

Family-group name: DRYOBIADAE Gistel, 1856. Stem: *Dryobi*-. Note: nomen oblitum (see Bousquet et al. 2009: 45); senior homonym of DRYOBIINI Arnett, 1962 (type genus *Dryobius* J.L. LeConte, 1850) in Coleoptera: CERAMBYCIDAE.

***Dryobius* J.L. LeConte, 1850c**: 23. Current status: valid genus in CERAMBYCIDAE: CERAMBYCINAE: DRYOBIINI.

Type species: *Callidiumsexfasciatum* Say, 1824 (= *Dryobiussexnotatus* Linsley, 1957), by monotypy.

Family-group name: DRYOBIINI Arnett, 1962. Stem: *Dryobi*-. Note: junior homonym of DRYOBIIDAE Gistel, 1856 (type genus *Dryobia* Gistel, 1856) in Coleoptera: PTINIDAE.

***Dryocoetes* Eichhoff, 1864**: 38, 45. Current status: valid genus in CURCULIONIDAE: SCOLYTINAE: DRYOCOETINI. Note: name placed on the Official List of Generic Names in Zoology (ICZN 1979b).

Type species: *Bostrichusautographus* Ratzeburg, 1837, by subsequent designation (Hopkins 1914: 121; see ICZN 1979b). Note: name placed on the Official List of Specific Names in Zoology (ICZN 1979b).

Family-group name: DRYOCOETOIDEAE Lindemann, 1877. Stem: *Dryocoet*-.

***Dryoctenes* Audinet-Serville, 1835a**: 27. Current status: valid genus in CERAMBYCIDAE: LAMIINAE: ACANTHODERINI.

Type species: *Dryoctenescaliginosus* Audinet-Serville, 1835 (= *Lamiascrupulosa* Germar, 1823), by monotypy.

Family-group name: DRYOCTENITAE J. Thomson, 1860. Stem: *Dryocten*-.

***Dryophilus* Chevrolat, 1832a**: pl. 3. Current status: valid genus in PTINIDAE: DRYOPHILINAE: DRYOPHILINI.

Type species: *Dryophilusanobioides* Chevrolat, 1832, by monotypy.

Family-group name: DRYOPHILIDAE Gistel, 1848. Stem: *Dryophil*-.

***Dryophthorus* Germar, 1823**: 302. Current status: valid genus in CURCULIONIDAE: DRYOPHTHORINAE: DRYOPHTHORINI. Note: name placed on the Official List of Generic Names in Zoology (ICZN 1987c).

Type species: *Curculiolymexylon* Fabricius, 1792 (= *Curculiocorticalis* Paykull, 1792), by monotypy (see ICZN 1987c). Note: *Curculiocorticalis* Paykull, 1792 was placed on the Official List of Specific Names in Zoology (ICZN 1987c).

Family-group name: DRYOPHTHORIDES Schönherr, 1825. Stem: *Dryophthor*-.

***Dryops* G.-A. Olivier, 1791**: 297. Current status: valid genus in DRYOPIDAE.

Type species: *Dermestesauriculatus* Geoffroy, 1785, by subsequent designation (Froriep 1827: 483).

Family-group name: DRYOPIDES Billberg, 1820. Stem: *Dryop*-.

***Dryotribus* G.H. Horn, 1873c**: 432. Current status: valid genus in CURCULIONIDAE: COSSONINAE: DRYOTRIBINI.

Type species: *Dryotribusmimeticus* G.H. Horn, 1873, by monotypy.

Family-group name: DRYOTRIBI J.L. LeConte, 1876. Stem: *Dryotrib*-.

***Drypta* Latreille, 1797**: 75. Current status: valid genus in CARABIDAE: HARPALINAE: DRYPTINI.

Type species: *Carabusemarginatus* Gmelin, 1790 (= *Carabusdentatus* Rossi, 1790), by subsequent designation (Latreille 1810: 426). Note: no species were originally included in this genus by Latreille (1797); the first ones were the two species listed by Fabricius (1801a: 230, 231).

Family-group name: DRYPTINAE Bonelli, 1810. Stem: *Drypt*-.

***Dynamopus* Semenov, 1896**: 336. Current status: junior synonym of *Orubesa* Reitter, 1895 in SCARABAEIDAE: DYNAMOPODINAE: DYNAMOPODINI.

Type species: *Dynamopusathleta* Semenov, 1896, by monotypy.

Family-group name: DYNAMOPINAE Arrow, 1911. Stem: *Dynamopod*-.

***Dynamostes* Pascoe, 1857**: 90. Current status: valid genus in DISTENIIDAE: DYNAMOSTINI.

Type species: *Dynamostesaudax* Pascoe, 1857, by monotypy.

Family-group name: DYNAMOSTIDES Lacordaire, 1868. Stem: *Dynamost*-.

***Dynastes* W.S. MacLeay, 1819**: 66. Current status: valid genus in SCARABAEIDAE: DYNASTINAE: DYNASTINI: DYNASTINA. Note: name placed on the Official List of Generic Names in Zoology (ICZN 2014a).

Type species: *Scarabaeushercules* Linnaeus, 1758, by subsequent designation (K.W. Kirby 1825: 567; see ICZN 2014a). Note: name placed on the Official List of Specific Names in Zoology (ICZN 2014a).

Family-group name: DYNASTIDAE W.S. MacLeay, 1819. Stem: *Dynast*-.

***Dysantes* Pascoe, 1869a**: 31. Current status: valid genus in TENEBRIONIDAE: TENEBRIONINAE: TOXICINI: DYSANTINA. Note: *Dysantes* Forster, 1869 [published in May] in Hymenoptera is a junior homonym of *Dysantes* Pascoe, 1869 [published 1 January] and not the opposite as noted in the literature (see Bouchard and Bousquet 2020a: 101).

Type species: *Dicerodereselongatus* L. Redtenbacher, 1868, by monotypy.

Family-group name: DYSANTINAE Gebien, 1922. Stem: *Dysant*-.

***Dyscharachthis* Blackburn, 1900**: 216, 217. Current status: valid genus in EUCNEMIDAE: EUCNEMINAE: DYSCHARACHTHINI.

Type species: *Dyscharachthisbrevipennis* Blackburn, 1900, by monotypy.

Family-group name: DYSCHARACHTHINI Muona, 1993. Stem: *Dyscharachth*-.

***Dyscherus* Chaudoir, 1855**: 4, 21. Current status: valid genus in CARABIDAE: SCARITINAE: SCARITINI: DYSCHERINA.

Type species: *Scaritescostatus* Klug, 1833, by monotypy.

Family-group name: DYSCHERINA Basilewsky, 1973. Stem: *Dyscher*-.

***Dyschirius* Bonelli, 1810**: Tab. Syn. Current status: valid genus in CARABIDAE: SCARITINAE: DYSCHIRIINI.

Type species: *Scaritesthoracicus* Rossi, 1790, by subsequent designation (Curtis 1831: pl. 354). Note: no species were originally included in this genus by Bonelli (1810); the first ones were the two species listed by Panzer (1813: 67).

Family-group name: DYSCHIRIINI Kolbe, 1880. Stem: *Dyschiri*-.

***Dysides* Perty, 1832**: 113. Current status: valid genus in BOSTRICHIDAE: DYSIDINAE.

Type species: *Dysidesobscurus* Perty, 1832, by monotypy.

Family-group name: DYSIDINI Lesne, 1894. Stem: *Dysid*-.

***Dyslobus* J.L. LeConte, 1869a**: 380. Current status: valid genus in CURCULIONIDAE: ENTIMINAE: ENTIMITAE: BYRSOPAGINI: STRANGALIODINA.

Type species: *Otiorhynchussegnis* J.L. LeConte, 1857, by subsequent designation (G.H. Horn in J.L. LeConte and G.H. Horn 1876: 41).

Family-group name: DYSLOBINI J.L. LeConte, 1874. Stem: *Dyslob*-.

***Dysmorphocerus* Solier, 1849**: 451. Current status: valid genus in CANTHARIDAE: DYSMORPHOCERINAE.

Type species: *Dysmorphocerusblanchardii* Solier, 1849, by monotypy.

Family-group name: DYSMORPHOCERINAE Brancucci, 1980. Stem: *Dysmorphocer*-.

***Dystaxia* J.L. LeConte, 1867**: 385. Current status: valid genus in SCHIZOPODIDAE: DYSTAXIINI.

Type species: *Dystaxiamurrayi* J.L. LeConte, 1867, by monotypy.

Family-group name: DYSTAXINI Théry, 1929. Stem: *Dystaxi*-.

***Dystheamon* Grouvelle, 1927**: 141. Current status: valid genus in DISCOLOMATIDAE: NOTIOPHYGINAE: DYSTHEAMONINI.

Type species: *Dystheamonaeneum* Grouvelle, 1927, by monotypy.

Family-group name: DYSTHEAMONINI John, 1954. Stem: *Dystheamon*-.

***Dytiscus* Linnaeus, 1758**: 342, 411. Current status: valid genus in DYTISCIDAE: DYTISCINAE: DYTISCINI. Note: name placed on the Official List of Generic Names in Zoology (ICZN 1961e).

Type species: *Dytiscusmarginalis* Linnaeus, 1758, by subsequent designation (Latreille 1810: 426; see ICZN 1961e). Note: name placed on the Official List of Specific Names in Zoology (ICZN 1961e).

Family-group name: DYTICIDES Leach, 1815. Stem: *Dytisc*-.

***Earophilus* Gistel, 1856**: 375. Current status: junior synonym of *Cucujus* Fabricius, 1775 in CUCUJIDAE: CUCUJINAE.

Type species: *Cantharissanguinolenta* Linnaeus, 1767 (= *Meloecinnaberinus* Scopoli, 1763), by monotypy.

Family-group name: EAROPHILIDAE Gistel, 1856. Stem: *Earophil*-.

***Ebaeus* Erichson, 1840b**: 113. Current status: valid genus in MELYRIDAE: MALACHIINAE: MALACHIINI.

Type species: *Cantharispedicularia* Linnaeus, 1758, by subsequent designation (C.G. Thomson 1859: 112).

Family-group name: EBAEINI Portevin, 1931. Stem: *Ebae*-.

***Eboo* Reid, 1993**: 62. Current status: valid genus in CHRYSOMELIDAE: EUMOLPINAE: BROMIINI. Note: new replacement name for *Odontionopa* Erichson, 1842.

Type species: *Colaspisviridula* Erichson, 1842, by subsequent designation (Reid 1993: 62) (type fixation for *Odontionopa* Erichson).

Family-group name: EBOOINA Reid, 1993. Stem: *Eboo*-.

***Eburia* Lacordaire, 1830b**: 177. Current status: valid genus in CERAMBYCIDAE: CERAMBYCINAE: EBURIINI.

Type species: *Cerambyxquadrimaculatus* Linnaeus, 1767, by subsequent designation (Hope 1844: 189).

Family-group name: ÉBURIITES Blanchard, 1845. Stem: *Eburi*-.

***Eburniogaster* Seevers, 1938**: 424. Current status: valid genus in STAPHYLINIDAE: ALEOCHARINAE: COROTOCINI: EBURNIOGASTRINA.

Type species: *Eburniogastertermitocolus* Seevers, 1938, by original designation.

Family-group name: EBURNIOGASTRINA Jacobson, Kistner & Pasteels, 1986. Stem: *Eburniogastr*-.

†***Eccoptarthrus* Arnoldi, 1977**: 169. Current status: valid genus in NEMONYCHIDAE: †ECCOPTARTHRINAE. Note: Upper Jurassic (Kazakhstan).

Type species: †*Eccoptarthruscrassipes* Arnoldi, 1977, by original designation.

Family-group name: †ECCOPTARTHRINI Arnoldi, 1977. Stem: *Eccoptarthr*-.

***Eccoptomenus* Chaudoir, 1850**: 409. Current status: valid genus in CARABIDAE: HARPALINAE: CHLAENIINI: CHLAENIINA.

Type species: *Chlaeniuseximius* Dejean, 1831, by subsequent designation (Basilewsky and Grundmann 1954: 247).

Family-group name: ECCOPTOMENINI Jeannel, 1949. Stem: *Eccoptomen*-.

***Eccoptopterus* Motschulsky, 1863**: 515. Current status: valid genus in CURCULIONIDAE: SCOLYTINAE: XYLEBORINI.

Type species: *Eccoptopterussexspinosus* Motschulsky, 1863 (= *Scolytusspinosus* G.-A. Olivier, 1795), by monotypy.

Family-group name: ECCOPTOPTERINI Kalshoven, 1959. Stem: *Eccoptopter*-.

†***Eccoptothorax* Arnoldi, 1977**: 158. Current status: junior synonym of *Procurculio* Arnoldi, 1977 in NEMONYCHIDAE: †ECCOPTARTHRINAE. Note: Upper Jurassic (Kazakhstan).

Type species: †*Eccoptothoraxlatipennis* Arnoldi, 1977 (= *Procurculiofortipes* Arnoldi, 1977), by original designation.

Family-group name: †ECCOPTOTHORACINI Arnoldi, 1977. Stem: *Eccoptothorac*-.

***Eccoptus* Dejean, 1821**: 86. Current status: invalid senior synonym of *Zygops* Schönherr, 1825 in CURCULIONIDAE: CONODERINAE: ZYGOPINI. Note: name suppressed for the Principle of Priority and placed on the Official Index of Rejected and Invalid Generic Names in Zoology (see ICZN 1987e).

Type species: *Curculiostrix* G.-A. Olivier, 1791, by monotypy.

Family-group name: ECCOPTINAE Pierce, 1919. Stem: *Eccopt*-. Note: family-group name permanently invalid (ICZN 1999a, Article 39).

***Ecelonerus* Schönherr, 1839**: 163. Current status: junior synonym of *Dendropemon* Schönherr, 1839 in ANTHRIBIDAE: ANTHRIBINAE: ECELONERINI. Note: First Reviser (ICZN 1999a, Article 24.2.2) for *Dendropemon* Schönherr, 1839 versus *Ecelonerus* Schönherr, 1839 is Zimmerman (1994: 83).

Type species: *Ecelonerussubfasciatus* Fåhraeus, 1839, by original designation.

Family-group name: ECÉLONÉRIDES Lacordaire, 1865. Stem: *Eceloner*-.

***Echiaster* Erichson, 1839b**: 29. Current status: valid genus in STAPHYLINIDAE: PAEDERINAE: LATHROBIINI: ECHIASTERINA.

Type species: *Echiasterlongicollis* Erichson, 1840, by subsequent designation (Duponchel 1841b: 57). Note: no species were originally included in this genus by Erichson (1839b); the first ones were the two species described by Erichson (1840a: 637).

Family-group name: ECHIASTERES Casey, 1905. Stem: *Echiaster*-.

***Echinaspis* Blatchley, 1922**: 122. Current status: junior synonym of *Microhyus* J.L. LeConte, 1876 in CURCULIONIDAE: MOLYTINAE: CLEOGONINI. Note: junior homonym of *Echinaspis* Haeckel, 1881 [Protista].

Type species: *Echinaspiswolcotti* Blatchley, 1922 (= *Microhyussetiger* J.L. LeConte, 1876), by monotypy.

Family-group name: ECHINASPINI Blatchley, 1922. Stem: *Echinaspid*-. Note: family-group name permanently invalid (ICZN 1999a, Article 39).

***Echinocnemus* Schönherr, 1843**: 315. Current status: valid genus in CURCULIONIDAE: BRACHYCERINAE: ERIRHININI.

Type species: *Erirhinussquameus* Boheman, 1836, by original designation.

Family-group name: ECHINOCNEMINA Legalov, 2020a: 321. Stem: *Echinocnem*-.

***Echthrogaster* Blackburn, 1900**: 213. Current status: valid genus in EUCNEMIDAE: MACRAULACINAE: ECHTHROGASTERINI.

Type species: *Echthrogasterlugubris* Blackburn, 1900, by monotypy.

Family-group name: ECHTHROGASTERINI Cobos, 1965. Stem: *Echthro­gaster*-.

***Ecitochara* Wasmann, 1887**: 404. Current status: valid genus in STAPHYLINIDAE: ALEOCHARINAE: ATHETINI: ECITOCHARINA.

Type species: *Ecitocharafusicornis* Wasmann, 1887, by monotypy.

Family-group name: ECITOCHARINI Seevers, 1965. Stem: *Ecitochar*-.

***Ecitoclimax* Borgmeier, 1934**: 452. Current status: valid genus in STAPHYLINIDAE: OXYTELINAE: OXYTELINI.

Type species: *Ecitoclimaxtarsalis* Borgmeier, 1934, by original designation.

Family-group name: ECITOCLIMACINI Borgmeier, 1934. Stem: *Ecitoclimac*-.

***Ecitogaster* Wasmann, 1900a**: 404. Current status: valid genus in STAPHYLINIDAE: ALEOCHARINAE: ECITOGASTRINI.

Type species: *Ecitogasterschmalzi* Wasmann, 1900, by subsequent monotypy (Wasmann 1900b: 224).

Family-group name: ECITOGASTRINI Fenyes, 1919. Stem: *Ecitogastr*-.

†***Eckfeldattagenus* Háva & Wappler, 2014**: 68. Current status: valid genus in DERMESTIDAE: ATTAGENINAE: †ECKFELDATTAGENINI. Note: Middle Eocene (Germany).

Type species: †*Eckfeldattagenuseocenicus* Háva & Wappler, 2014, by original designation.

Family-group name: †ECKFELDATTAGENINI Háva, 2015: 1. Stem: *Eckfeldattagen*-.

***Ectatosia* Pascoe, 1857**: 109. Current status: valid genus in CERAMBYCIDAE: LAMIINAE: APOMECYNINI.

Type species: *Ectatosiamoorei* Pascoe, 1857, by monotypy.

Family-group name: ECTATOSIIDES Lacordaire, 1872. Stem: *Ectatosi*-.

***Ectemnorhinus* G.R. Waterhouse, 1853**: 184. Current status: valid genus in CURCULIONIDAE: ENTIMINAE: OTIORHYNCHITAE: ECTEMNORHININI.

Type species: *Ectemnorhinusviridis* G.R. Waterhouse, 1853, by monotypy.

Family-group name: ECTEMNORHINIDES Lacordaire, 1863. Stem: *Ectemnorhin*-.

***Ectenessa* H.W. Bates, 1885**: 257. Current status: valid genus in CERAMBYCIDAE: CERAMBYCINAE: ECTENESSINI.

Type species: *Ectenessanitida* H.W. Bates, 1885, by subsequent designation (Napp and Martins 1982: 371).

Family-group name: ECTENESSINI Martins, 1998. Stem: *Ecteness*-.

***Ectrephes* Pascoe, 1866d**: xvi. Current status: valid genus in PTINIDAE: PTININAE: ECTREPHINI.

Type species: *Ectrephesformicarum* Pascoe, 1866, by monotypy.

Family-group name: ECTREPHIDAE Wasmann, 1894. Stem: *Ectreph*-.

***Ectyche* Pascoe, 1869a**: 143. Current status: valid genus in TENEBRIONIDAE: DIAPERINAE: ECTYCHINI.

Type species: *Ectycheerebea* Pascoe, 1869, by monotypy.

Family-group name: ECTYCHINI Doyen, E.G. Matthews & Lawrence, 1990. Stem: *Ectych*-.

***Ecyroschema* J. Thomson, 1864**: 48. Current status: valid genus in CERAMBYCIDAE: LAMIINAE: CEROPLESINI: CROSSOTINA.

Type species: *Ecyroschemafavosa* J. Thomson, 1864, by original designation.

Family-group name: ÉCYROSCHÉMIDES Lacordaire, 1872. Stem: *Ecyroschemat*-.

***Edrotes* J.L. LeConte, 1851a**: 140. Current status: valid genus in TENEBRIONIDAE: PIMELIINAE: EDROTINI.

Type species: *Edrotesventricosus* J.L. LeConte, 1851, by monotypy.

Family-group name: ÉDROTIDES Lacordaire, 1859. Stem: *Edrot*-.

***Edusa* Chevrolat in Dejean, 1836a**: 408. Current status: junior synonym of *Edusella* Chapuis, 1874 in CHRYSOMELIDAE: EUMOLPINAE: EUMOLPINI. Note: this name has precedence over *Edusella* Chapuis, which is currently used as valid (see Bousquet and Bouchard 2013a: 111); an application to the Commission is needed to conserve *Edusella* Chapuis, 1874 as valid.

Type species: *Colaspisvaripes* Boisduval, 1835, by monotypy.

Family-group name: EDUSITES Chapuis, 1874. Stem: *Edus*-.

***Edusella* Chapuis, 1874**: 309. Current status: valid genus in CHRYSOMELIDAE: EUMOLPINAE: EUMOLPINI.

Type species: *Edusasuturalis* Chapuis, 1874, by monotypy.

Family-group name: EDUSELLINI Clavareau, 1914. Stem: *Edusell*-.

***Ega* Laporte, 1834b**: 93. Current status: valid genus in CARABIDAE: HARPALINAE: LACHNOPHORINI: LACHNOPHORINA.

Type species: *Egaformicaria* Laporte, 1834, by monotypy.

Family-group name: EGINI G.H. Horn, 1881. Stem: *Eg*-.

***Egidyella* Reitter, 1899**: 284. Current status: valid genus in DERMESTIDAE: ATTAGENINAE incertae sedis.

Type species: *Egidyellaprophetea* Reitter, 1899, by monotypy.

Family-group name: EPIDYELLINI Semenov-Tjan-Shansky, 1914. Stem: *Egidyell*-.

***Egolia* Erichson, 1842**: 150. Current status: valid genus in TROGOSSITIDAE: EGOLIINAE.

Type species: *Egoliavariegata* Erichson, 1842, by monotypy.

Family-group name: ÉGOLIIDES Lacordaire, 1854. Stem: *Egoli*-.

***Egrius* Pascoe, 1865b**: 428. Current status: valid genus in CURCULIONIDAE: CEUTORHYNCHINAE: EGRIINI.

Type species: *Egriuscamelus* Pascoe, 1865, by monotypy.

Family-group name: EGRIINI Pajni & Kohli, 1982. Stem: *Egri*-.

***Eicolyctus* J.R. Sahlberg, 1919**: 4. Current status: junior synonym of *Zavaljus* Reitter, 1880 in EROTYLIDAE: XENOSCELINAE.

Type species: *Cryptophagusbrunneus* Gyllenhal, 1808, by monotypy.

Family-group name: EICOLYCTINI Vogt, 1967. Stem: *Eicolyct*-.

***Eidoreus* Sharp in Blackburn and Sharp, 1885**: 146. Current status: valid genus in EUPSILOBIIDAE.

Type species: *Eidoreusminutus* Sharp, 1885, by monotypy.

Family-group name: EIDOREINAE Sasaji, 1986. Stem: *Eidore*-.

***Ekkoptogaster* Herbst, 1793**: 124. Current status: junior synonym of *Scolytus* Geoffroy, 1762 in CURCULIONIDAE: SCOLYTINAE: SCOLYTINI. Note: this name, as well as its unjustified emendation *Eccoptogaster* Erichson, 1836, were placed on the Official Index of Rejected and Invalid Generic Names in Zoology (ICZN 1963c).

Type species: *Bostrichusscolytus* Fabricius, 1775, by subsequent designation (Hopkins 1914: 121).

Family-group name: ECCOPTOGASTRIDAE Gistel, 1848. Stem: *Ekkoptogastr*-. Note: family- group name permanently invalid (ICZN 1999a, Article 39).

***Elacatis* Pascoe, 1860b**: 52. Current status: valid genus in SALPINGIDAE: OTHNIINAE.

Type species: *Elacatisdelusa* Pascoe, 1860, by monotypy.

Family-group name: ELACATIDAE Reitter, 1879. Stem: *Elacat*-. Note: junior homonym of ELACATINAE Gill, 1861 (type genus *Elacate* Cuvier, 1832) in Pisces.

***Elachistarthron* Notman, 1920**: 715. Current status: junior synonym of *Apheloglossa* Casey, 1894 in STAPHYLINIDAE: ALEOCHARINAE: DIESTOTINI.

Type species: *Elachistarthronambiguum* Notman, 1920, by monotypy.

Family-group name: ELACHISTARTHRONINI Notman, 1920. Stem: *Elachistarthr*-.

***Elaphidion* Audinet-Serville, 1834b**: 66. Current status: valid genus in CERAMBYCIDAE: CERAMBYCINAE: ELAPHIDIINI.

Type species: *Cerambyxspinicornis* Drury, 1773, by subsequent designation (Newman 1840c: 6).

Family-group name: ELAPHIDIONITAE J. Thomson, 1864. Stem: *Elaphidi*-.

***Elaphinis* Burmeister, 1842**: 595. Current status: valid genus in SCARABAEIDAE: CETONIINAE: CETONIINI: CETONIINA.

Type species: *Cetoniacinerascens* Fabricius, 1798 (= *Scarabaeuscinereonebulosus* DeGeer, 1778), by subsequent designation (Duponchel 1844b: 227).

Family-group name: ELAPHINI Schoch, 1894. Stem: *Elaphin*-.

***Elaphocera* Gené, 1836a**: iii. Current status: valid genus in SCARABAEIDAE: MELOLONTHINAE: TANYPROCTINI: TANYPROCTINA. Note: this name was also introduced in the same year by Gené (1836b: 188) in volume 39 of ‘Memorie della Reale Accademia delle Scienze di Torino’; volume 5 (p. iii) of the ‘Annales de la Société Entomologique de France’ was published earlier.

Type species: *Elaphoceraobscura* Gené, 1836 (= *Melolonthaemarginata* Gyllenhal, 1817), by monotypy.

Family-group name: ELAPHOCERITAE Blanchard, 1851. Stem: *Elaphocer*-. Note: nomen oblitum (see A.B.T. Smith 2006: 168).

***Elaphrus* Fabricius, 1775**: 227. Current status: valid genus in CARABIDAE: ELAPHRINAE.

Type species: *Cicindelariparia* Linnaeus, 1758, by subsequent designation (Latreille 1810: 425).

Family-group name: ELAPHRII Latreille, 1802. Stem: *Elaphr*-.

***Elater* Linnaeus, 1758**: 342, 404. Current status: valid genus in ELATERIDAE: ELATERINAE: ELATERINI.

Type species: *Elaterferrugineus* Linnaeus, 1758, by subsequent designation (Latreille 1810: 426).

Family-group name: ELATERIDES Leach, 1815. Stem: *Elater*-.

†***Electrapate* Iablokoff-Khnzorian, 1962**: 87. Current status: valid genus in SCHIZOPODIDAE: †ELECTRAPATINI. Note: Eocene (Lithuania).

Type species: †*Electrapatemartynovi* Iablokoff-Khnzorian, 1962, by original designation.

Family-group name: †ELECTRAPATIDAE Iablokoff-Khnzorian, 1962. Stem: *Electrapat*-.

***Electribius* Crowson, 1973**: 231. Current status: valid genus in ARTEMATOPODIDAE: ELECTRIBIINAE.

Type species: *Electribiusoligocenicus* Crowson, 1973, by original designation.

Family-group name: ELECTROPOGONINI Crowson, 1975. Stem: *Electribi*-. Note: ELECTROPOGONINI used by Crowson (1975: 77) is a *lapsus calami* for ELECTRIBIIDAE (see Lawrence and Newton 1995: 850).

†***Electrocoryssopus* Legalov, 2023a**: 50. Current status: valid genus in CURCULIONIDAE: CONODERINAE: †ELECTROCORYSSOPINI. Note: upper Eocene (Baltic amber).

Type species: †*Electrocoryssopusandrushchenkoi* Legalov, 2023, by original designation.

Family-group name: †ELECTROCORYSSOPINI Legalov, 2023a: 49. Stem: *Electrocoryssop*-.

†***Electropteron* Kazantsev, 2012a**: 1. Current status: valid genus in LYCIDAE: LYCINAE: LEPTOLYCINI: †ELECTROPTERINA. Note: Miocene (Dominican amber).

Type species: †*Electropteronavus* Kazantsev, 2012, by original designation.

Family-group name: †ELECTROPTERINI Kazantsev, 2013a: 28. Stem: *Electropter*-.

†***Electrorubesopsis* Bai & B. Wang in Li et al., 2018**: 348. Current status: valid genus in SCARABAEIDAE: †ELECTRORUBESOPSINAE. Note: mid-Cretaceous (Myanmar).

Type species: †*Electrorubesopsisbeuteli* Bai & B. Wang, 2018, by original designation.

Family-group name: †ELECTRORUBESOPSINAE Bai & B. Wang in Li et al., 2018: 348. Stem: *Electrorubesops*-.

***Eledona* Latreille, 1797**: 19. Current status: valid genus in TENEBRIONIDAE: TENEBRIONINAE: BOLITOPHAGINI.

Type species: *Opatrumagricola* Herbst, 1783, by subsequent monotypy (Latreille 1802b: 162).

Family-group name: ELEDONAEDES Billberg, 1820. Stem: *Eledon*-. Note: nomen oblitum (see Bouchard et al. 2011: 415); senior homonym of ELEDONINAE Grimpe, 1921 (type genus *Eledone* Leach, 1817) in Mollusca.

***Eleia* Gistel, 1848b**: viii, xiv. Current status: junior synonym of *Chrysomela* Linnaeus, 1758 in CHRYSOMELIDAE: CHRYSOMELINAE: CHRYSOMELINI. Note: unnecessary new replacement name for *Chrysomela* Linnaeus, 1758.

Type species: *Chrysomelapopuli* Linnaeus, 1758, by subsequent designation (Latreille 1810: 432; see ICZN 1984c) (type fixation for *Chrysomela* Linnaeus).

Family-group name: ELEIAEIDAE Gistel, 1848. Stem: *Elei*-.

***Elenophorus* Dejean, 1821**: 64. Current status: junior synonym of *Leptoderis* Billberg, 1820 in TENEBRIONIDAE: PIMELIINAE: ELENOPHORINI.

Type species: *Tenebriocollaris* Linnaeus, 1767, by monotypy.

Family-group name: ELÉNOPHORITES Solier, 1837. Stem: *Elenophor*-.

***Eleodes* Eschscholtz, 1829d**: 8. Current status: valid genus in TENEBRIONIDAE: BLAPTINAE: AMPHIDORINI.

Type species: *Eleodesdentipes* Eschscholtz, 1829, by subsequent designation (Hope 1841a: 124).

Family-group name: ELEODIINI Blaisdell, 1909. Stem: *Eleod*-.

***Eleodopsis* Blaisdell, 1939**: 52. Current status: junior synonym of *Blapylis* G.H. Horn, 1870 in TENEBRIONIDAE: BLAPTINAE: AMPHIDORINI.

Type species: *Eleodopsissubvestita* Blaisdell, 1939, by original designation.

Family-group name: ELEODOPSINAE Blaisdell, 1939. Stem: *Eleodopse*-.

***Eletica* Dejean, 1834**: 224. Current status: valid genus in MELOIDAE: ELETICINAE: ELETICINI: ELETICINA.

Type species: *Lyttarufa* Fabricius, 1801 (= *Lyttabipustulata* Fabricius, 1801), by monotypy.

Family-group name: ELETICIDES Wellman, 1910. Stem: *Eletic*-.

***Eleusis* Laporte, 1834c**: 131. Current status: valid genus in STAPHYLINIDAE: OSORIINAE: ELEUSININI.

Type species: *Eleusistibialis* Laporte, 1834, by monotypy.

Family-group name: ELEUSININA Sharp, 1887. Stem: *Eleusin*-.

***Eligmoderma* J. Thomson, 1864**: 246. Current status: valid genus in CERAMBYCIDAE: CERAMBYCINAE: ELIGMODERMINI.

Type species: *Eligmodermaibidionoides* J. Thomson, 1864, by original designation.

Family-group name: ÉLIGMODERMIDES Lacordaire, 1868. Stem: *Eligmoderm*-.

***Ellescus* Dejean, 1821**: 87. Current status: valid genus in CURCULIONIDAE: CURCULIONINAE: ELLESCINI.

Type species: *Curculioscanicus* Paykull, 1792, by subsequent designation (Schönherr 1835: 321).

Family-group name: ELLESCHINA C.G. Thomson, 1859. Stem: *Ellesc*-.

***Elmis* Latreille, 1802a**: 398. Current status: valid genus in ELMIDAE: ELMINAE: ELMINI: ELMINA. Note: name placed on the Official List of Generic Names in Zoology (ICZN 1995e).

Type species: *Elmismaugetii* Latreille, 1802, by monotypy (see ICZN 1995e). Note: name placed on the Official List of Specific Names in Zoology (1995e).

Family-group name: ELMIDAE Curtis, 1830. Stem: *Elm*- (see ICZN 1995e).

***Elodes* Latreille, 1797**: 44. Current status: valid genus in SCIRTIDAE: SCIRTINAE.

Type species: *Cistelapallida* Fabricius, 1775 (= *Lampyrisminuta* Linnaeus, 1767), by subsequent designation (Latreille 1810: 426). Note: no species were originally included in this genus by Latreille (1797); the first ones were the two species listed by Latreille (1802b: 98).

Family-group name: ELODIIDAE Shuckard, 1839. Stem: *Elod*-.

†***Elodophthalmus* Kirejtshuk & Azar, 2008**: 25. Current status: valid genus in †ELODOPHTHALMIDAE. Note: Lower Cretaceous (Lebanese amber).

Type species: †*Elodophthalmusharmonicus* Kirejtshuk & Azar, 2008, by original designation.

Family-group name: †ELODOPHTHALMIDAE Kirejtshuk & Azar, 2008. Stem: *Elodophthalm*-.

***Elosoma* Motschulsky, 1845b**: 33. Current status: valid genus in MELYRIDAE: MALACHIINAE incertae sedis.

Type species: *Elosomapersica* Motschulsky, 1845, by monotypy.

Family-group name: ELOSOMATIDAE Jakobson, 1915. Stem: *Elosomat*-.

***Elpidus* Péringuey, 1907**: 314, 318. Current status: valid genus in SCARABAEIDAE: CETONIINAE: TRICHIINI.

Type species: *Xiphoscelishopei* Burmeister, 1842, by monotypy.

Family-group name: ELPIDIDES Péringuey, 1907. Stem: *Elpid*-.

***Elythomerus* C.O. Waterhouse, 1874**: 535. Current status: valid genus in HETEROCERIDAE: ELYTHOMERINAE.

Type species: *Elythomeruselongatulus* C.O. Waterhouse, 1874, by monotypy.

Family-group name: ELYTHOMERINI M.F. Pacheco, 1964. Stem: *Elythomer*-.

***Elytracantha* Lane, 1955**: 281. Current status: invalid senior synonym of *Elytracanthina* Monné, 2005 in CERAMBYCIDAE: LAMIINAE: ELYTRACANTHININI. Note: junior homonym of *Elytracantha* Kleine, 1915 [Coleoptera: BRENTIDAE].

Type species: *Elytracanthapugionata* Lane, 1955, by monotypy.

Family-group name: ELYTRACANTHINAE Lane, 1955. Stem: *Elytracanth*-. Note: family-group name permanently invalid (ICZN 1999a, Article 39).

***Elytracanthina* Monné, 2005**: 428. Current status: valid genus in CERAMBYCIDAE: LAMIINAE: ELYTRACANTHININI. Note: new replacement name for *Elytracantha* Lane, 1955.

Type species: *Elytracanthapugionata* Lane, 1955, by monotypy (type fixation for *Elytracantha* Lane).

Family-group name: ELYTRACANTHININI Bousquet, 2009. Stem: *Elytracanthin*-.

***Elytroxys* Jekel, 1856**: 9bis. Current status: valid genus in CURCULIONIDAE: ENTIMINAE: ENTIMITAE: LORDOPINI.

Type species: *Elytroxysinflatus* Lacordaire, 1863, by subsequent monotypy (Lacordaire 1863: 275). Note: no species were originally included in this genus by Jekel (1856); the first one was included by Lacordaire (1863: 275).

Family-group name: ELYTROXYSI Jekel, 1856. Stem: *Elytroxe*-.

***Elytrurus* Boisduval, 1835**: 400. Current status: valid genus in CURCULIONIDAE: ENTIMINAE: OTIORHYNCHITAE: ELYTRURINI.

Type species: *Elytruruslapeyrousei* Boisduval, 1835, by subsequent designation (Schönherr 1842b: 239).

Family-group name: ELYTRURINI G.A.K. Marshall, 1956. Stem: *Elytrur*-.

***Embrithes* Schönherr, 1842b**: 392. Current status: valid genus in CURCULIONIDAE: ENTIMINAE: CYPHICERITAE: EMBRITHINI.

Type species: *Embrithesagnatus* Boheman, 1842, by original designation.

Family-group name: EMBRITHINI G.A.K. Marshall, 1942. Stem: *Embrith*-.

***Emelinus* Casey, 1895**: 773, 777. Current status: valid genus in ADERIDAE.

Type species: *Xylophilusmelsheimeri* J.L. LeConte, 1855, by subsequent designation (Werner 1990: 212).

Family-group name: EMELININI Báguena Corella, 1948. Stem: *Emelin*-.

***Emma* Escalera, 1913**: 318. Current status: invalid senior synonym of *Emmita* Escalera, 1914 in DASCILLIDAE: KARUMIINAE: EMMITINI. Note: junior homonym of *Emma* J.E. Gray, 1843 [Bryozoa].

Type species: *Emmaserricornis* Escalera, 1913, by monotypy.

Family-group name: EMMINAE Escalera, 1913. Stem: *Emm*-. Note: family-group name permanently invalid (ICZN 1999a, Article 39).

***Emmalus* Erichson, 1843b**: 251. Current status: valid genus in TENEBRIONIDAE: BLAPTINAE: OPATRINI: AMMOBIINA.

Type species: *Emmaluspilosus* Erichson, 1843, by monotypy.

Family-group name: EMMALLINA C. Koch, 1956. Stem: *Emmal*-.

***Emmita* Escalera, 1914**: 226. Current status: valid genus in DASCILLIDAE: KARUMIINAE: EMMITINI. Note: new replacement name for *Emma*Escalera 1913.

Type species: *Emmaserricornis* Escalera, 1913, by monotypy (type fixation for *Emma* Escalera).

Family-group name: EMMITINAE Escalera, 1914. Stem: *Emmit*-.

***Empelus* J.L. LeConte, 1861**: 52. Current status: valid genus in STAPHYLINIDAE: EMPELINAE.

Type species: *Litochrusbrunnipennis* Mannerheim, 1852, by original designation.

Family-group name: EMPELINAE Newton & Thayer, 1992. Stem: *Empel*-.

***Emphyastes* Mannerheim, 1852**: 353. Current status: valid genus in CURCULIONIDAE: MOLYTINAE: EMPHYASTINI.

Type species: *Emphyastesfucicola* Mannerheim, 1852, by monotypy.

Family-group name: EMPHIASTIDES Lacordaire, 1863. Stem: *Emphyast*-.

***Emphylus* Erichson, 1846**: 346. Current status: junior synonym of *Spavius* Motschulsky, 1844 in CRYPTOPHAGIDAE: CRYPTOPHAGINAE: CRYPTOPHAGINI.

Type species: *Cryptophagusglaber* Gyllenhal, 1808, by monotypy.

Family-group name: EMPHYLI Casey, 1900. Stem: *Emphyl*-.

***Emphytoecia* Fairmaire & Germain, 1860b**: 529. Current status: valid genus in CERAMBYCIDAE: LAMIINAE: PTEROPLIINI.

Type species: *Agapanthiasuturella* Blanchard, 1851, by subsequent designation (J. Thomson 1864: 115).

Family-group name: EMPHYTOECIIDES Lacordaire, 1872. Stem: *Emphyto­eci*-.

***Empocryptus* Sharp, 1900**: 593. Current status: valid genus in EROTYLIDAE: CRYPTOPHILINAE: EMPOCRYPTINI.

Type species: *Empocryptusovalis* Sharp, 1900, by monotypy.

Family-group name: EMPOCRYPTINI Leschen, 2003. Stem: *Empocrypt*-.

***Enaria* Erichson, 1847b**: 657. Current status: valid genus in SCARABAEIDAE: MELOLONTHINAE: MELOLONTHINI: ENARIINA.

Type species: *Melolonthamelanictera* Klug, 1833, by subsequent monotypy (Blanchard in H. Milne-Edwards et al. 1851: 152).

Family-group name: ENARINA Dewailly, 1950. Stem: *Enari*-.

***Encaustes* Lacordaire, 1842a**: 30, 33. Current status: valid genus in EROTYLIDAE: EROTYLINAE: ENCAUSTINI.

Type species: *Engisverticalis* W.S. MacLeay, 1825, by subsequent designation (Arrow 1925: 69).

Family-group name: ENCAUSTINI Crotch, 1876. Stem: *Encaust*-.

***Enceladus* Bonelli, 1813**: 460. Current status: valid genus in CARABIDAE: SIAGONINAE: ENCELADINI.

Type species: *Enceladusgigas* Bonelli, 1813 [as *gygas*], by monotypy.

Family-group name: ENCELADINI G.H. Horn, 1881. Stem: *Encelad*-.

***Enchoptera* Saunders, 1850**: 76. Current status: valid genus in CERAMBYCIDAE: CERAMBYCINAE: MACRONINI.

Type species: *Enchopteraapicalis* Saunders, 1850, by subsequent designation (J. Thomson 1864: 139).

Family-group name: ENCHAPTERITAE J. Thomson, 1861. Stem: *Enchopter*-.

***Encrates* Gistel, 1848b**: ix, 127. Current status: junior synonym of *Lebia* Latreille, 1802 in CARABIDAE: HARPALINAE: LEBIINI: LEBIINA. Note: unnecessary new replacement name for *Lebia* Latreille, 1802.

Type species: *Carabushaemorrhoidalis* Fabricius, 1787 (= *Buprestismarginatus* Geoffroy, 1785), by subsequent designation (E. Desmarest 1851: 76) (type fixation for *Lebia* Latreille).

Family-group name: ENCRATIDAE Gistel, 1856. Stem: *Encrat*-.

***Encyclops* Newman, 1838**: 392. Current status: valid genus in CERAMBYCIDAE: LEPTURINAE: ENCYCLOPINI.

Type species: *Encyclopspallipes* Newman, 1838 (= *Lepturacaerulea* Say, 1826), by monotypy.

Family-group name: ENCYCLOPINI J.L. LeConte, 1873. Stem: *Encyclop*-.

***Endaeus* Schönherr, 1826**: 250. Current status: valid genus in CURCULIONIDAE: CURCULIONINAE: OCHYROMERINI.

Type species: *Endaeuscastus* Schönherr, 1826, by original designation.

Family-group name: ENDAEINI Voss, 1958. Stem: *Endae*-.

***Endecatomus* Mellié, 1847**: 108. Current status: valid genus in BOSTRICHIDAE: ENDECATOMINAE.

Type species: *Anobiumreticulatum* Herbst, 1793, by monotypy.

Family-group name: ENDECATOMINI J.L. LeConte, 1861. Stem: *Endecatom*-.

***Endocephalus* Chevrolat in Dejean, 1836a**: 412. Current status: valid genus in CHRYSOMELIDAE: EUMOLPINAE: EUMOLPINI.

Type species: *Eumolpusbigatus* Germar, 1823 (= *Cryptocephaluslineatus* Fabricius, 1775), by subsequent designation (Monrós and Bechyné 1956: 1127).

Family-group name: ENDOCÉPHALITES Chapuis, 1874. Stem: *Endocephal*-.

***Endomia* Laporte, 1840b**: 259. Current status: valid genus in ANTHICIDAE: ANTHICINAE: ENDOMIINI.

Type species: *Anthicuselongatissimus* Laporte, 1840 (= *Notoxustenuicollis* Rossi, 1792), by monotypy.

Family-group name: ENDOMIINI Kaszab, 1956. Stem: *Endomi*-.

***Endomychus* Panzer, 1795**: 175. Current status: valid genus in ENDOMYCHIDAE: ENDOMYCHINAE.

Type species: *Chrysomelacoccinea* Linnaeus, 1758, by subsequent designation (Latreille 1810: 432).

Family-group name: ENDOMYCHIDES Leach, 1815. Stem: *Endomych*-.

***Endophloeus* Dejean, 1834**: 195. Current status: valid genus in ZOPHERIDAE: COLYDIINAE: SYNCHITINI.

Type species: *Eledonaspinosula* Latreille, 1807 (= *Silphamarkovichiana* Piller & Mitterpacher, 1783), by monotypy.

Family-group name: ENDOPHLOEIDAE Wollaston, 1868. Stem: *Endophloe*-.

***Endroedyolus* Scholtz & Howden, 1987**: 92. Current status: valid genus in SCARABAEIDAE: SCARABAEINAE: ENDROEDYOLINI.

Type species: *Endroedyolusparadoxus* Scholtz & Howden, 1987, by original designation.

Family-group name: ENDROEDYOLINI Davis, Deschodt & Scholtz, 2019: 138. Stem: *Endroedyol*-.

***Engis* W.S. MacLeay, 1825**: 41. Current status: invalid senior synonym of *Encaustes* Lacordaire, 1842 in EROTYLIDAE: EROTYLINAE: ENCAUSTINI. Note: junior homonym of *Engis* Paykull, 1800 [Coleoptera: EROTYLIDAE]; W.S. MacLeay (1825: 41) added “Nobis” after the name *Engis* indicating that he was creating a new genus despite the fact that he was aware of *Engis* Paykull, 1800; therefore, we consider *Engis* W.S. MacLeay, 1825 as a junior homonym of *Engis* Paykull, 1800 as done by Węgrzynowicz (2007: 539), and not as a misidentification of Paykull’s genus as done by Bouchard et al. (2011: 358).

Type species: *Engisverticalis* W.S. MacLeay, 1825, by subsequent designation (Węgrzynowicz 2007: 539).

Family-group name: ENGIDAE W.S. MacLeay, 1825. Stem: *Engid*-. Note: family-group name permanently invalid (ICZN 1999a, Article 39).

***Enhydrus* Laporte, 1834c**: 110. Current status: valid genus in GYRINIDAE: GYRININAE: DINEUTINI: ENHYDRUSINA. Note: the older genus-group name *Enhydrus* W.S. MacLeay, 1825 was suppressed for the Principles of Priority and Homonymy and placed on the Official Index of Rejected and Invalid Generic Names in Zoology while *Enhydrus* Laporte, 1834 was placed on the Official List of Generic Names in Zoology (ICZN 1964).

Type species: *Gyrinussulcatus* Wiedemann, 1821, by monotypy (see ICZN 1964). Note: name placed on the Official List of Specific Names in Zoology (ICZN 1964).

Family-group name: ENHYDRINI Branden, 1882. Stem: *Enhydrus*- (ICZN 2012a).

***Enicocerus* Stephens, 1829a**: 196. Current status: valid subgenus of *Ochthebius* Leach, 1815 in HYDRAENIDAE: OCHTHEBIINAE: OCHTHEBIINI.

Type species: *Enicocerusviridiaeneus* Stephens, 1829 (= *Ochthebiusexsculptus* Germar, 1823), by monotypy.

Family-group name: ENICOCERINA Perkins, 1997. Stem: *Enicocer*-.

***Enicodes* G.R. Gray in Griffith and Pidgeon, 1831**: pls 65, 73. Current status: valid genus in CERAMBYCIDAE: LAMIINAE: ENICODINI.

Type species: *Cerambyxfichtelii* Schreber, 1802, by monotypy.

Family-group name: ENICODITAE J. Thomson, 1864. Stem: *Enicod*-.

***Enicopus* Stephens, 1830**: 318. Current status: valid genus in MELYRIDAE: DASYTINAE: ENICOPINI.

Type species: *Lagriaatra* Fabricius, 1787, by monotypy. Note: the type species often reported for this genus (e.g., Mayor 2007b: 407) is *Dermesteshirtus* Linnaeus, 1767 as selected by Westwood (1838c: 28), but that species is not an originally included species; *Enicopusater* (Fabricius, 1787) is currently included in the nominotypical subgenus of *Enicopus* Stephens, 1830.

Family-group name: HENICOPINI Escalera, 1927. Stem: *Enicop*- (ICZN 1999a, Article 29.5).

***Enneapaussus* Jeannel, 1946**: 62. Current status: junior synonym of *Anapaussus* Wasmann, 1929 in CARABIDAE: PAUSSINAE: PAUSSINI: PAUSSINA.

Type species: *Paussushowa* Dohrn, 1881, by original designation.

Family-group name: ENNEAPAUSSINI Jeannel, 1946. Stem: *Enneapauss*-.

***Ennearabdus* Van Lansberge, 1874**: 20. Current status: valid genus in SCARABAEIDAE: SCARABAEINAE: EUCRANIINI.

Type species: *Onthophaguslobocephalus* Harold, 1868, by monotypy.

Family-group name: ENNEARABDINI Pereira & Martínez, 1956. Stem: *Ennearabd*-.

***Ennearthron* Mellié, 1847**: 110. Current status: valid genus in CIIDAE: CIINAE: CIINI.

Type species: *Ciscornutus* Gyllenhal, 1827, by subsequent designation (E. Desmarest 1860: 261). Note: no available species was originally included in this genus by Mellié (1847); the first ones are the species listed by Mellié (1849: 361, 362).

Family-group name: ENNEARTHRONINAE Chûjô, 1939. Stem: *Ennearthr*-.

***Enochrus* C.G. Thomson, 1859**: 18. Current status: valid genus in HYDROPHILIDAE: ENOCHRINAE.

Type species: **here fixed** (under ICZN 1999a, Article 70.3.2) as *Hydrophilusmelanocephalus* G.-A. Olivier, 1793, misidentified as *Hydrophilusbicolor* Fabricius, 1792 (sensu Gyllenhal, 1808) in the original designation by C.G. Thomson (1859: 18).

Family-group name: ENOCHRINAE Short & Fikáček, 2013: 741. Stem: *Enochr*-.

***Enodognathus* Benderitter, 1920**: 112. Current status: valid genus in OCHODAEIDAE: OCHODAEINAE: ENODOGNATHINI.

Type species: *Enodognathusgilleti* Benderitter, 1920, by monotypy.

Family-group name: ENDOGNATHINI Scholtz, 1988. Stem: *Enodognath*-.

***Enoicus* Péringuey, 1896**: 568, 581. Current status: valid genus in CARABIDAE: HARPALINAE: ENOICINI.

Type species: *Euleptusfallax* Péringuey, 1896, by monotypy.

Family-group name: ENOICINI Basilewsky, 1985. Stem: *Enoic*-.

***Enoplium* Latreille, 1802b**: 111. Current status: valid genus in CLERIDAE: KORYNETINAE.

Type species: *Tillusserraticornis* G.-A. Olivier, 1790, by monotypy.

Family-group name: ENOPLIIDAE Gistel, 1848. Stem: *Enopli*-.

***Enoplocerus* Audinet-Serville, 1832**: 146. Current status: valid genus in CERAMBYCIDAE: PRIONINAE: CALLIPOGONINI.

Type species: *Cerambyxarmillatus* Linnaeus, 1767, by monotypy.

Family-group name: ENOPLOCERITAE J. Thomson, 1864. Stem: *Enoplocer*-.

***Enoplopus* Solier, 1848b**: 151, 158. Current status: junior synonym of *Accanthopus* Dejean, 1821 in TENEBRIONIDAE: TENEBRIONINAE: HELOPINI: ENOPLOPODINA. Note: unnecessary new replacement name for *Accanthopus* Dejean, 1821.

Type species: fixed by Bouchard et al. (2021: 174) according to Article 70.3.2 (ICZN 1999a) as *Tenebriovelikensis* Piller & Mitterpacher, 1783, the taxonomic species involved in the misidentification of *Tenebriocaraboides* Linnaeus, 1758 (sensu Germar, 1817), as designated by monotypy by Dejean (1821: 71) (type fixation for *Accanthopus* Dejean).

Family-group name: ENOPLOPINI Reitter, 1917. Stem: *Enoplopod*-.

***Enoplotrupes* P.H. Lucas, 1869**: xiii. Current status: valid genus in GEOTRUPIDAE: GEOTRUPINAE: ENOPLOTRUPINI.

Type species: *Enoplotrupessinensis* P.H. Lucas, 1869, by monotypy.

Family-group name: ENOPLOTRUPINI Paulian, 1945. Stem: *Enoplotrup*-.

***Enoptes* Gistel, 1848b**: x. Current status: junior synonym of *Pytho* Latreille, 1797 in PYTHIDAE. Note: unnecessary new replacement name for *Pytho* Latreille, 1797.

Type species: *Cucujuscoeruleus* Fabricius, 1787 (= *Tenebriodepressus* Linnaeus, 1767), by subsequent designation (Latreille 1810: 429) (type fixation for *Pytho* Latreille).

Family-group name: ENOPTISIDAE Gistel, 1848. Stem: *Enopt*-.

***Entimus* Germar, 1817b**: 341. Current status: valid genus in CURCULIONIDAE: ENTIMINAE: ENTIMITAE: ENTIMINI: ENTIMINA.

Type species: *Curculioimperialis* Forster, 1771, by subsequent designation (Schönherr 1823: 1138).

Family-group name: ENTIMIDES Schönherr, 1823. Stem: *Entim*-.

***Entomoculia* Croissandeau, 1891**: 150. Current status: valid genus in STAPHYLINIDAE: LEPTOTYPHLINAE: ENTOMOCULIINI.

Type species: fixed by Herman (2001: 6) according to Article 70.3.2 (ICZN 1999a) as *Entomoculiajeanneli* Coiffait, 1955, the taxonomic species involved in the misidentification of *Leptotyphlusgrouvellei* Fauvel, 1890 (sensu Croissandeau, 1891), as designated subsequently by Blackwelder (1952: 149).

Family-group name: ENTOMOCULINI Coiffait, 1957. Stem: *Entomoculi*-.

***Entomosatopus* Bonvouloir, 1871**: 67, 279. Current status: valid genus in EUCNEMIDAE: EUCNEMINAE: ENTOMOSATOPINI.

Type species: *Entomosatopuscurtus* Bonvouloir, 1871, by monotypy.

Family-group name: ENTOMOSATOPINI Muona, 1993. Stem: *Entomosatop*-.

***Entomoscelis* Chevrolat in Dejean, 1836a**: 402. Current status: valid genus in CHRYSOMELIDAE: CHRYSOMELINAE: CHRYSOMELINI.

Type species: *Chrysomelaadonidis* Pallas, 1771, by subsequent designation (Chevrolat 1843a: 654).

Family-group name: ENTOMOSCÉLITES Chapuis, 1874. Stem: *Entomoscelid*-.

†***Eobelus* Arnoldi, 1977**: 147. Current status: valid genus in NEMONYCHIDAE: †EOBELINAE: †EOBELINI. Note: Upper Jurassic (Kazakhstan).

Type species: †*Eobeluslongipes* Arnoldi, 1977, by original designation.

Family-group name: †EOBELIDAE Arnoldi, 1977. Stem: *Eobel*-.

†***Eocaenonemonyx* Legalov, 2013a**: 58. Current status: valid genus in NEMONYCHIDAE: †CRETONEMONYCHINAE: †EOCAENONEMONYCHINI. Note: middle Eocene (USA).

Type species: †*Eocaenonemonyxkuscheli* Legalov, 2013, by original designation.

Family-group name: †EOCAENONEMONYCHINI Legalov, 2013a: 58. Stem: *Eocaenonemonych*-.

***Eocatops* Peyerimhoff, 1924**: 63. Current status: valid genus in LEIODIDAE: CHOLEVINAE: ANEMADINI: EOCATOPINA.

Type species: *Eocatopsambiguus* Peyerimhoff, 1924, by original designation.

Family-group name: EOCATOPINA Jeannel, 1936. Stem: *Eocatop*-.

†***Eocenesibinia* Legalov, 2017b**: 984. Current status: valid genus in CURCULIONIDAE: CURCULIONINAE: TYCHIINI: †EOCENESIBINIINA. Note: upper Eocene (Baltic amber).

Type species: †*Eocenesibiniaprussica* Legalov, 2017, by original designation.

Family-group name: †EOCENESIBINIINA Legalov, 2017b: 984. Stem: *Eocenesibini*-.

***Eoclytra* Monrós, 1958**: 36. Current status: junior synonym of *Diapericera* Lacordaire, 1848 in CHRYSOMELIDAE: CRYPTOCEPHALINAE: CLYTRINI: EOCLYTRINA.

Type species: *Eoclytrafreudei* Monrós, 1958, by original designation.

Family-group name: EOCLYTRINI Monrós, 1958. Stem: *Eoclytr*-.

†***Eodromeus* Ponomarenko, 1977**: 66. Current status: valid genus in TRACHYPACHIDAE: †EODROMEINAE. Note: Middle-Upper Jurassic/Lower Cretaceous (China; Kazakhstan; Mongolia; Russia).

Type species: †*Eodromeusdissectus* Ponomarenko, 1977 [as *Eodromeusfasciatus*], by original designation. Note: see Bouchard et al. (2011: 101) for a discussion regarding the type species.

Family-group name: †EODROMEINAE Ponomarenko, 1977. Stem: *Eodrome*-.

***Eohomopterus* Wasmann, 1920**: 110. Current status: valid genus in CARABIDAE: PAUSSINAE: PAUSSINI: CARABIDOMEMNINA.

Type species: *Homopterusaequatoriensis* Wasmann, 1899, by monotypy.

Family-group name: EOHOMOPTERINAE Wasmann, 1929. Stem: *Eohomopter*-.

†***Eopaussus* Wasmann, 1926**: 29. Current status: valid genus in CARABIDAE: PAUSSINAE: PAUSSINI: †EOPAUSSINA. Note: Eocene (Russia).

Type species: †*Eopaussusbalticus* Wasmann, 1926, by monotypy.

Family-group name: †EOPAUSSINAE Luna de Carvalho, 1951. Stem: *Eopauss*-.

***Eorhipidius* Iablokoff-Khnzorian, 1986**: 89. Current status: junior synonym of *Pterydrias* Reitter, 1895 in RIPIPHORIDAE: RIPIDIINAE: EORHIPIDIINI.

Type species: *Eorhipidiusjanuschevi* Iablokoff-Khnzorian, 1986, by monotypy.

Family-group name: EORHIPIDIINI Iablokoff-Khnzorian, 1986. Stem: *Eorhipidi*-.

***Eosalacus* Legalov, 2007**: 30. Current status: junior synonym of *Pseudominurus* Voss, 1956 in ATTELABIDAE: RHYNCHITINAE: RHYNCHITITAE: AULETINI: MANDELSCHTAMIINA.

Type species: *Eosalacusreunionensis* Legalov, 2007, by original designation.

Family-group name: EOSALACINA Legalov, 2007. Stem: *Eosalac*-.

***Eospasta* Selander, 1966**: 474. Current status: valid genus in MELOIDAE: ELETICINAE: ELETICINI: EOSPASTINA.

Type species: *Spasticainconstans* Fairmaire & Germain, 1863, by original designation.

Family-group name: EOSPASTINA Selander, 1966. Stem: *Eospast*-.

***Epactius* D.H. Schneider in Fabricius, 1791**: 23. Current status: senior synonym of *Omophron* Latreille, 1802 in CARABIDAE: OMOPHRONINAE. Note: new replacement name for *Scolytus* Fabricius, 1791; this name should have priority over *Omophron* Latreille, 1802 and an application to the Commission is needed to retain Latreille’s well-established name; Reversal of Precedence (ICZN 1999a, Article 23.9) cannot be considered since *Epactius* has been used as valid at least once since 1900 (e.g., Semenov 1903a).

Type species: *Carabuslimbatus* Fabricius, 1777, by subsequent designation (Latreille 1810: 426) (type fixation for *Omophron* Latreille).

Family-group name: EPACTIINI Fauvel, 1888. Stem: *Epacti*-.

***Epactoides* Olsouffief, 1947**: 171. Current status: valid genus in SCARABAEIDAE: SCARABAEINAE: EPACTOIDINI.

Type species: *Phacosomamadecassus* Paulian, 1936, by monotypy.

Family-group name: EPACTOIDINI Rossini, Grebennikov, Merrien, Miraldo, Viljanen & Tarasov 2022: 661. Stem: *Epactoid*-.

***Eparchium* Bernhauer, 1934**: 481. Current status: junior synonym of *Apterodasytes* Champion, 1923 in MELYRIDAE: DASYTINAE: DASYTINI.

Type species: *Eparchiumparadoxum* Bernhauer, 1934, by original designation.

Family-group name: EPARCHIINI Bernhauer, 1934: 481. Stem: *Eparchi*-. Note: this name was overlooked in Bouchard et al. (2011), Bouchard and Bousquet (2020a).

***Ephistemus* Stephens, 1829a**: 167. Current status: valid genus in CRYPTOPHAGIDAE: ATOMARIINAE: ATOMARIINI.

Type species: *Dermestesgyrinoides* Marsham, 1802 (= *Dermestesglobulus* Paykull, 1798), by subsequent designation (Westwood 1838c: 10).

Family-group name: EPHISTEMINI Casey, 1900. Stem: *Ephistem*-.

***Epicaerus* Schönherr, 1834a**: 323. Current status: valid genus in CURCULIONIDAE: ENTIMINAE: POLYDRUSITAE: GEONEMINI.

Type species: *Epicaerusmexicanus* Boheman, 1834, by original designation.

Family-group name: EPICAERI G.H. Horn, 1876. Stem: *Epicaer*-.

***Epicasta* J. Thomson, 1864**: 90. Current status: valid genus in CERAMBYCIDAE: LAMIINAE: DESMIPHORINI.

Type species: *Epicastaocellata* J. Thomson, 1864, by original designation.

Family-group name: ÉPICASTIDES Lacordaire, 1872. Stem: *Epicast*-.

***Epicauta* Dejean, 1834**: 224. Current status: valid genus in MELOIDAE: MELOINAE: EPICAUTINI.

Type species [suggested]: *Meloeerythrocephalus* Pallas, 1771, by subsequent designation (Werner 1945: 425). Note: the valid type species fixation is that of Duponchel (1844b: 356), who selected *Lyttagigas* Fabricius, 1792 (= *Cantharisgigas* G.-A. Olivier, 1790), but that species is currently included in the genus *Cyaneolytta* Péringuey, 1909; an application to the Commission is necessary to fix *Meloeerythrocephalus* Pallas, 1771 as the type species of *Epicauta* Dejean, 1834.

Family-group name: EPICAUTINI J.B. Parker & Böving, 1924. Stem: *Epicaut*-.

***Epiclines* Chevrolat in Guérin-Méneville, 1839a**: 40. Current status: valid genus in CLERIDAE: EPICLININAE.

Type species: *Epiclinesgayi* Chevrolat, 1839, by monotypy.

Family-group name: EPICLININAE Gunter, Leavengood & Bartlett in Gunter et al., 2013: 633. Stem: *Epiclin*-.

***Epilachna* Chevrolat in Dejean, 1836a**: 436. Current status: valid genus in COCCINELLIDAE: COCCINELLINAE: EPILACHNINI.

Type species: *Coccinellaborealis* Fabricius, 1775, by subsequent designation (Hope 1841a: 157).

Family-group name: EPILACHNIENS Mulsant, 1846. Stem: *Epilachn*-.

***Epilissus* Dejean, 1836b**: 151. Current status: valid genus in SCARABAEIDAE: SCARABAEINAE: EPILISSINI.

Type species: *Canthonprasinus* Klug, 1833, by subsequent designation (Reiche 1841: 212).

Family-group name: EPILISSIDES Van Lansberge, 1874. Stem: *Epiliss*-.

***Epimetopus* Lacordaire, 1854**: 465, 467. Current status: valid genus in EPIMETOPIDAE. Note: new replacement name for *Ceratoderus* Mulsant, 1851.

Type species: *Ceratoderusgraniger* Mulsant, 1851, by monotypy (type fixation for *Ceratoderus* Mulsant).

Family-group name: EPIMETOPINA Zaitzev, 1908. Stem: *Epimetop*-.

***Epipedocera* Chevrolat, 1863**: 339. Current status: valid genus in CERAMBYCIDAE: CERAMBYCINAE: TILLOMORPHINI.

Type species: *Epipedocerazona* Chevrolat, 1863, by subsequent designation (Pascoe 1869b: 640).

Family-group name: EPIPEDOCERINI Gahan, 1906. Stem: *Epipedocer*-.

***Epipedophyes* G.A.K. Marshall, 1946**: 97. Current status: valid genus in CURCULIONIDAE: MOLYTINAE: PETALOCHILINI. Note: new replacement name for *Epipedus* Schönherr, 1842.

Type species: *Epipedussquamifer* Boheman, 1842, by original designation (type fixation for *Epipedus* Schönherr).

Family-group name: EPIPEDOPHYINAE Kuschel, 1955. Stem: *Epipedophy*-.

***Epipedus* Schönherr, 1842a**: 462. Current status: invalid senior synonym of *Epipedophyes* G.A.K. Marshall, 1946 in CURCULIONIDAE: MOLYTINAE: PETALOCHILINI. Note: junior homonym of *Epipedus* Spinola, 1837 [Hemiptera].

Type species: *Epipedussquamifer* Boheman, 1842, by original designation.

Family-group name: ÉPIPÉDIDES Lacordaire, 1865. Stem: *Epiped*-. Note: family-group name permanently invalid (ICZN 1999a, Article 39).

***Epiphanis* Eschscholtz, 1829a**: 35. Current status: valid genus in EUCNEMIDAE: MELASINAE: EPIPHANINI.

Type species: *Epiphaniscornutus* Eschscholtz, 1829, by monotypy.

Family-group name: EPIPHANINI Muona, 1993. Stem: *Epiphan*-. Note: junior homonym of EPIPHANIDAE Harring, 1913 (type genus *Epiphanes* Ehrenberg, 1832) in Rotifera.

***Epiphloeus* Spinola, 1841**: 75. Current status: valid genus in CLERIDAE: KORYNETINAE.

Type species: *Epiphloeuspantherinus* Spinola, 1845, by subsequent designation (E. Desmarest 1852: 265). Note: no species were originally included in this genus by Spinola (1841); the first ones were the six species listed by Spinola (1845b: 8, as *Epiphlaeus*).

Family-group name: EPIPHLÖINEN Kuwert, 1893. Stem: *Epiphloe*-.

***Epiphysa* Dejean, 1834**: 178. Current status: valid genus in TENEBRIONIDAE: PIMELIINAE: ADESMIINI.

Type species: *Pimeliaflavicollis* Fabricius, 1794, by monotypy.

Family-group name: ÉPIPHYSIDES Lacordaire, 1859. Stem: *Epiphys*-.

***Epipocus* Chevrolat in Dejean, 1836a**: 439. Current status: valid genus in ENDOMYCHIDAE: EPIPOCINAE.

Type species: *Endomychusrufitarsis* Chevrolat, 1835, by subsequent designation (R. Lucas 1920: 270). Note: the type species often reported for *Epipocus* is *Endomychustibialis* Guérin-Méneville, 1834 by subsequent designation of Strohecker (1953: 66) but R. Lucas’ typification is earlier; both species are currently placed in the genus *Epipocus*.

Family-group name: EPIPOCIDAE Gorham, 1873. Stem: *Epipoc*-.

***Epirinus* Dejean, 1833b**: 137. Current status: valid genus in SCARABAEIDAE: SCARABAEINAE: EPIRININI.

Type species: *Scarabaeusgranulatus* G.-A. Olivier, 1789 (= *Scarabaeusflagellatus* Fabricius, 1775), by subsequent designation (M.C. Ferreira 1972: 136). Note: M.C. Ferreira (1972: 136) designated *Scarabaeusflagellatus* Fabricius, 1775 as type species, not an originally included species, but since she listed it in synonymy with *Scarabaeusgranulatus* G.-A. Olivier, 1789, a species originally included, she is deemed to have designated the latter species as type species (ICZN 1999a, Article 69.2.2; see Daniel 2019: 64).

Family-group name: EPIRINIDES Van Lansberge, 1874. Stem: *Epirin*-.

***Epirrhynchus* Schönherr, 1823**: 1139. Current status: valid genus in CURCULIONIDAE: LIXINAE: CLEONINI.

Type species: *Curculioargus* Sparrman, 1785, by original designation.

Family-group name: EPIRHYNCHINI Petri, 1914. Stem: *Epirrhynch*-.

***Episomus* Schönherr, 1823**: 1143. Current status: valid genus in CURCULIONIDAE: ENTIMINAE: OTIORHYNCHITAE: EPISOMINI.

Type species: *Curculiolacerta* Fabricius, 1781, by original designation.

Family-group name: EPISOMIDES Lacordaire, 1863. Stem: *Episom*-.

***Epistictina* Hincks, 1950b**: 509. Current status: valid genus in CHRYSOMELIDAE: CASSIDINAE: BASIPRIONOTINI. Note: new replacement name for *Epistictia* Boheman, 1850.

Type species: *Epistictiaviridimaculata* Boheman, 1850, by subsequent designation (Maulik 1919: 318) (type fixation for *Epistictia* Boheman).

Family-group name: EPISTICTININI Hincks, 1952. Stem: *Epistictin*-.

***Epistomentis* Solier, 1849**: 479. Current status: valid genus in BUPRESTIDAE: BUPRESTINAE: EPISTOMENTINI.

Type species: *Chrysochroapicta* Gory, 1841, by monotypy.

Family-group name: EPISTOMENTINI Levey, 1978. Stem: *Epistoment*-.

***Epistrophus* Kirsch, 1869**: 200. Current status: valid genus in CURCULIONIDAE: MOLYTINAE: HYLOBIINI: EPISTROPHINA. Note: this taxon is a junior homonym of *Epistrophus* Gistel, 1834, which is a junior objective synonym of *Drasterius* Eschscholtz, 1829 [Coleoptera: ELATERIDAE]; Alonso-Zarazaga and Lyal (1999: 202) mentioned that an application would be submitted to the Commission for the suppression of Gistel’s name and recommended that current usage be maintained; an application to the Commission has yet to be submitted to fix this problem.

Type species: *Epistrophustumidus* Kirsch, 1869, by monotypy.

Family-group name: EPISTROPHINA G.A.K. Marshall, 1932. Stem: *Epistroph*-.

***Epitragus* Latreille, 1802b**: 165. Current status: valid genus in TENEBRIONIDAE: PIMELIINAE: EPITRAGINI.

Type species: *Epitragusfuscus* Latreille, 1804, by subsequent monotypy (Latreille 1804b: 322). Note: Latreille (1802b) originally included “*Helopsvariegatus*? Fabricius” (= *Tenebriovariegatus* Fabricius, 1781) in his genus but since the species was cited as a species inquirenda, it is deemed not to be originally included (ICZN 1999a, Article 67.2.5).

Family-group name: ÉPITRAGITES Blanchard, 1845. Stem: *Epitrag*-.

***Epiverta* Dieke, 1947**: 169. Current status: valid genus in COCCINELLIDAE: COCCINELLINAE: EPILACHNINI.

Type species: *Solanophilachelonia* Mader, 1933, by original designation.

Family-group name: EPIVERTINI X.-F. Pang & Mao, 1979. Stem: *Epivert*-.

***Epuraea* Erichson, 1843a**: 267. Current status: valid genus in NITIDULIDAE: EPURAEINAE: EPURAEINI.

Type species: *Nitidulasilacea* Herbst, 1783, by subsequent designation (C.G. Thomson 1859: 68).

Family-group name: EPURAEINI Kirejtshuk, 1986. Stem: *Epurae*-.

***Eremazus* Mulsant, 1851b**: 139. Current status: valid genus in SCARABAEIDAE: EREMAZINAE: EREMAZINI.

Type species: *Eremazusunistriatus* Mulsant, 1851, by monotypy.

Family-group name: EREMAZINA Stebnicka, 1977. Stem: *Eremaz*-.

***Eremnus* Schönherr, 1823**: 1145. Current status: valid genus in CURCULIONIDAE: ENTIMINAE: ENTIMITAE: TANYRHYNCHINI.

Type species: *Eremnusexaratus* Schönherr, 1823, by original designation.

Family-group name: ÉREMNIDES Lacordaire, 1863. Stem: *Eremn*-.

***Eremochilus* Weise, 1913**: 117. Current status: valid genus in COCCINELLIDAE: COCCINELLINAE: EPILACHNINI.

Type species: *Eremochilusperegrinus* Weise, 1913, by monotypy.

Family-group name: EREMOCHILINI Gordon & Vanderberg, 1987. Stem: *Eremochil*-.

***Eremosaprinus* Ross, 1939**: 39. Current status: valid genus in HISTERIDAE: SAPRININAE: EREMOSAPRININI.

Type species: *Saprinusunguiculatus* Ross, 1939, by original designation.

Family-group name: EREMOSAPRININI Lackner in Lackner et al. 2024: 59. Stem: *Eremosaprin*-.

***Eremotes* Wollaston, 1861c**: 364. Current status: junior synonym of *Rhyncolus* Germar, 1817 in CURCULIONIDAE: COSSONINAE: RHYNCOLINI: RHYNCOLINA.

Type species: *Hylurguscrassicornis* Brullé, 1839, by monotypy.

Family-group name: EREMOTINI Voss, 1939. Stem: *Eremot*-.

***Eremoxenus* Semenov, 1892**: 438. Current status: valid genus in BRENTIDAE: BRENTINAE: BRENTINI: EREMOXENINA.

Type species: *Eremoxenuschan* Semenov, 1892, by monotypy.

Family-group name: EREMOXENIDAE Semenov, 1892. Stem: *Eremoxen*-.

***Eretes* Laporte, 1833b**: 397. Current status: valid genus in DYTISCIDAE: DYTISCINAE: ERETINI.

Type species: *Dytiscusgriseus* Fabricius, 1781, by original designation.

Family-group name: ERETINI Crotch, 1873. Stem: *Eret*-.

***Ergania* Pascoe, 1882**: 445. Current status: valid genus in CURCULIONIDAE: CURCULIONINAE: CURCULIONINI: ERGANIINA.

Type species: *Erganiagibba* Pascoe, 1882, by monotypy.

Family-group name: ERGANIINA Pelsue & O’Brien, 2011: 29. Stem: *Ergani*-.

***Ergates* Audinet-Serville, 1832**: 143. Current status: valid genus in CERAMBYCIDAE: PRIONINAE: ERGATINI.

Type species: *Prionusserrarius* Panzer, 1793 (= *Cerambyxfaber* Linnaeus, 1761), by monotypy.

Family-group name: ERGATITES Fairmaire, 1864. Stem: *Ergat*-.

***Erichsonia* Westwood, 1849**: 210. Current status: valid genus in CERAMBYCIDAE: PRIONINAE: PARANDRINI: ERICHSONIINA. Note: *Erichsonia* Dana, 1849 [Crustacea] was published in November 1849 while *Erichsonia* Westwood, 1849 was published on 5 November 1849 (G. Wheeler 1912: 754), therefore, Westwood’s name has priority.

Type species: *Erichsoniadentifrons* Westwood, 1849, by monotypy.

Family-group name: ERICHSONITAE J. Thomson, 1861. Stem: *Erichsoni*-. Note: senior homonym of ERICHSONIINA Brunke & Solodovnikov, 2016 (type genus *Erichsonius* Fauvel, 1874) in Coleoptera: STAPHYLINIDAE.

***Erichsonius* Fauvel, 1874**: 394, 427. Current status: valid genus in STAPHYLINIDAE: STAPHYLININAE: STAPHYLININI: ERICHSONIINA.

Type species: *Staphylinuscinerascens* Gravenhorst, 1802, by subsequent designation (R. Lucas 1920: 73) (type fixation for *Actobius* Fauvel, 1875, an unnecessary new replacement name for *Erichsonius* Fauvel).

Family-group name: ERICHSONIINA Brunke & Solodovnikov in Brunke et al., 2016: 447. Stem: *Erichsoni*-. Note: junior homonym of ERICHSONITAE J. Thomson, 1861 (type genus *Erichsonia* Westwood, 1849) in Coleoptera: CERAMBYCIDAE.

***Erineophilus* Hopkins, 1902**: 34. Current status: junior synonym of *Scolytodes* Ferrari, 1867 in CURCULIONIDAE: SCOLYTINAE: HEXACOLINI.

Type species: *Erineophilusschwarzi* Hopkins, 1902, by original designation.

Family-group name: ERINEOPHILIDES Hopkins, 1902. Stem: *Erineophil*-.

***Eriocnemis* Kaup, 1868a**: 21. Current status: invalid senior synonym of *Pelopides* Kuwert, 1896 in PASSALIDAE: PASSALINAE: MACROLININI. Note: junior homonym of *Eriocnemis* L. Reichenbach, 1849 [Aves].

Type species: *Passalustridens* Wiedemann, 1823, by subsequent designation (Hincks and Dibb 1935: 85).

Family-group name: ERIOCNEMIAE Kaup, 1871. Stem: *Eriocnemid*-. Note: family-group name permanently invalid (ICZN 1999a, Article 39).

***Erionomus* Kaup, 1868a**: 16. Current status: valid genus in PASSALIDAE: PASSALINAE: SOLENOCYCLINI.

Type species: *Passalusplaniceps* Eschscholtz, 1828, by monotypy.

Family-group name: ERIONOMINAE Kuwert, 1891. Stem: *Erionom*-.

***Eriotomus* Piochard de la Brûlerie, 1873**: 11, 72. Current status: junior synonym of *Oedesis* Motschulsky, 1850 in CARABIDAE: HARPALINAE: HARPALINI: DITOMINA.

Type species: *Ditomustomentosus* Dejean, 1831, by subsequent designation (Tschitschérine 1901: 225).

Family-group name: ERIOTOMI Antoine, 1959. Stem: *Eriotom*-.

***Eriphus* Audinet-Serville, 1834b**: 88. Current status: valid genus in CERAMBYCIDAE: CERAMBYCINAE: TRACHYDERINI: TRACHYDERINA. Note: Santos-Silva et al. (2023: 3) treated this name as a nomen protectum over *Eryphus* Klug, 1829; however, they did not specifically give evidence that the conditions of Article 23.9.1.2 were met (see such requirement in Article 23.9.2, ICZN 1999a); Santos-Silva et al. (2023: 3) also mention that the name *Eriphus* was made available earlier by Audinet-Serville (1834a: 541); however, the name in the key used by Audinet-Serville (1834a: 541) was a French vernacular name and as such is not a scientific name in the sense of Article 1 (ICZN 1999a, also see Glossary therein).

Type species: *Callidiumbisignatum* Germar, 1823, by subsequent designation (Chevrolat 1844: 404).

Family-group name: ERIPHITAE J. Thomson, 1864. Stem: *Eriph*-.

***Erirhinus* Schönherr, 1825**: 582. Current status: junior synonym of *Notaris* Germar, 1817 in CURCULIONIDAE: BRACHYCERINAE: ERIRHININI.

Type species: *Curculioaethiops* Fabricius, 1792, by subsequent designation (Schönherr 1826: 229).

Family-group name: ERIRHINIDES Schönherr, 1825. Stem: *Erirhin*-.

***Erlandia* Aurivillius, 1904**: 205. Current status: valid genus in CERAMBYCIDAE: CERAMBYCINAE: ERLANDIINI.

Type species: *Erlandiainopinata* Aurivillius, 1904, by monotypy.

Family-group name: ERLANDIINI Aurivillius, 1912. Stem: *Erlandi*-.

***Ernobius* C.G. Thomson, 1859**: 88. Current status: valid genus in PTINIDAE: ERNOBIINAE: ERNOBIINI.

Type species: *Dermestesmollis* Linnaeus, 1758, by original designation.

Family-group name: ERNOBIINAE Pic, 1912. Stem: *Ernobi*-.

***Ernoporus* C.G. Thomson, 1859**: 147. Current status: valid genus in CURCULIONIDAE: SCOLYTINAE: ERNOPORINI.

Type species: *Apatetiliae* Panzer, 1793, by original designation.

Family-group name: ERNOPORINAE Nüsslin, 1911. Stem: *Ernopor*-.

***Erodiscus* Schönherr, 1825**: 583. Current status: valid genus in CURCULIONIDAE: CURCULIONINAE: ERODISCINI.

Type species: *Lixusattenuatus* Fabricius, 1801, by original designation.

Family-group name: ERODISCIDES Lacordaire, 1863. Stem: *Erodisc*-. Note: this name is based on a misidentification of *Erodiscus* Schönherr, 1825 by Lacordaire (1863: 567) since Lacordaire excluded the type species of *Erodiscus*, placing it in *Toxeutes* Schönherr, 1843; Alonso-Zarazaga and Lyal (1999: 78) mentioned that they will apply to the Commission for the conservation of this tribe by designating *Erodiscus* Schönherr, 1825 as its type genus; meanwhile current usage is maintained.

***Erodius* Fabricius, 1775**: 258. Current status: valid genus in TENEBRIONIDAE: PIMELIINAE: ERODIINI.

Type species: *Erodiusgibbus* Fabricius, 1775, by subsequent designation (Latreille 1810: 429).

Family-group name: ERODIIDES Billberg, 1820. Stem: *Erodi*-.

***Eros* Newman, 1838**: 382. Current status: valid genus in LYCIDAE: EROTINAE: EROTINI.

Type species: *Lycushumeralis* Fabricius, 1801, by original designation.

Family-group name: EROTES J.L. LeConte, 1881. Stem: *Erot*-.

***Eroschema* Pascoe, 1859a**: 17. Current status: valid genus in CERAMBYCIDAE: CERAMBYCINAE: EROSCHEMINI.

Type species: *Eroschemapoweri* Pascoe, 1859, by monotypy.

Family-group name: ÉROSCHÉMIDES Lacordaire, 1868. Stem: *Eroschem*-.

***Erotylus* Fabricius, 1775**: 123. Current status: valid genus in EROTYLIDAE: EROTYLINAE: EROTYLINI.

Type species: *Coccinellagigantea* Linnaeus, 1758, by subsequent designation (J.E. Gray 1831a: 611).

Family-group name: EROTILENAE Latreille, 1802. Stem: *Erotyl*-.

***Ertlia* Borchmann, 1942**: 702. Current status: junior synonym of *Morphozonitis* Pic, 1922 in MELOIDAE: ELETICINAE: ERTLIANINI. Note: junior homonym of *Ertlia* Aurivillius, 1907 [Coleoptera: CERAMBYCIDAE].

Type species: *Ertliafasciata* Borchmann, 1942, by monotypy.

Family-group name: ERTLIINI Kaszab, 1959. Stem: *Ertli*-. Note: family-group name permanently invalid (ICZN 1999a, Article 39).

***Ertliana* Selander, 1964**: 11. Current status: junior synonym of *Morphozonitis* Pic, 1922 in MELOIDAE: ELETICINAE: ERTLIANINI. Note: new replacement name for *Ertlia* Borchmann, 1942.

Type species: *Ertliafasciata* Borchmann, 1942, by monotypy (type fixation for *Ertlia* Borchmann).

Family-group name: ERTLIANINA Selander, 1966. Stem: *Ertlian*-.

***Erythraenus* H.W. Bates, 1875**: 52. Current status: valid genus in CERAMBYCIDAE: PRIONINAE: ANACOLINI.

Type species: *Erythraenusborneensis* H.W. Bates, 1875, by monotypy.

Family-group name: ERYTHRAENINAE H.W. Bates, 1875. Stem: *Erythraen*-.

***Erythrus* A. White, 1853**: 142. Current status: valid genus in CERAMBYCIDAE: CERAMBYCINAE: PSEUDOLEPTURINI. Note: the older name *Erythrus* Walker in Curtis, 1829 [Hymenoptera] is a nomen nudum (see Heffern 2013: [76]).

Type species: *Erythruschampioni* A. White, 1853, by subsequent designation (J. Thomson 1864: 158) (designation for *Pseudoleptura* J. Thomson, 1861).

Family-group name: ERYTHRINAE Pascoe, 1866. Stem: *Erythr*-.

***Esarcus* Reiche, 1864**: 238. Current status: valid genus in MYCETOPHAGIDAE: ESARCINAE.

Type species: *Esarcusleprieurii* Reiche, 1864, by monotypy.

Family-group name: ESARCINI Reitter, 1882. Stem: *Esarc*-.

***Escalerina* Bolívar y Pieltain, 1926**: 196, 199. Current status: junior synonym of *Karumia* Escalera, 1913 in DASCILLIDAE: KARUMIINAE: ESCALERININI.

Type species: *Karumiamicrocephala* Escalera, 1913, by original designation.

Family-group name: ESCALERINI Paulus, 1972. Stem: *Escalerin*-.

***Eschatoporis* Blaisdell, 1906**: 76. Current status: valid genus in TENEBRIONIDAE: LAGRIINAE: ESCHATOPORIINI.

Type species: *Eschatoporisnunenmacheri* Blaisdell, 1906, by monotypy.

Family-group name: ESCHATOPORINI Blaisdell, 1906. Stem: *Eschatopori*-.

†***Escheria* Heer, 1847**: 57. Current status: valid genus in HYDROPHILIDAE incertae sedis. Note: late Miocene (Germany).

Type species: †*Escheriaovalis* Heer, 1847, by monotypy.

Family-group name: †ESCHERIIDAE Kolbe, 1880. Stem: *Escheri*-.

***Esemephe* Steiner, 1980**: 385. Current status: valid genus in TENEBRIONIDAE: PIMELIINAE: COSSYPHODINI: ESEMEPHINA.

Type species: *Esemephetumi* Steiner, 1980, by original designation.

Family-group name: ESEMEPHINI Steiner, 1980. Stem: *Esemeph*-.

***Essisus* Pascoe, 1866a**: 90. Current status: valid genus in CERAMBYCIDAE: LAMIINAE: DESMIPHORINI.

Type species: *Essisusdispar* Pascoe, 1866, by monotypy.

Family-group name: ESSISINI Aurivillius, 1917. Stem: *Essis*-.

***Estadia* Fairmaire, 1903**: 183. Current status: junior synonym of *Dietta* Sharp, 1876 in LEIODIDAE: LEIODINAE: ESTADIINI.

Type species: *Estadiacapito* Fairmaire, 1903, by original designation.

Family-group name: ESTADIINI Portevin, 1914. Stem: *Estadi*-.

***Esthesopus* Eschscholtz, 1829a**: 32. Current status: valid genus in ELATERIDAE: CARDIOPHORINAE.

Type species: *Esthesopuscastaneus* Eschscholtz, 1829, by monotypy.

Family-group name: ESTHESOPINAE Fleutiaux, 1919. Stem: *Esthesopod*-.

***Estola* Fairmaire & Germain, 1860b**: 524. Current status: valid genus in CERAMBYCIDAE: LAMIINAE: DESMIPHORINI.

Type species: *Estolahirsuta* Fairmaire & Germain, 1860 (= *Estolahirsutella* Aurivillius, 1922), by subsequent designation (J. Thomson 1861: 348).

Family-group name: ESTOLIDES Lacordaire, 1872. Stem: *Estol*-.

***Ethonion* Kubáň in Kubáň et al. 2001**: 202. Current status: valid genus in BUPRESTIDAE: AGRILINAE: CORAEBINI: ETHONIINA.

Type species: *Buprestisfissiceps* W. Kirby, 1818, by original designation.

Family-group name: ETHONIINA Majer, 2001. Stem: *Ethoni*-.

***Euaesthetus* Gravenhorst, 1806**: 201. Current status: valid genus in STAPHYLINIDAE: EUAESTHETINAE: EUAESTHETINI.

Type species: *Euaesthetusscaber* Gravenhorst, 1806 (= *Stenusbipunctatus* Ljungh, 1804), by monotypy.

Family-group name: EUAESTHETINA C.G. Thomson, 1859. Stem: *Euaesthet*-.

***Euanoma* Reitter, 1889b**: 98. Current status: valid genus in ELATERIDAE: OMALISINAE.

Type species: *Euanomastarcki* Reitter, 1889, by monotypy.

Family-group name: EUANOMINI Kazantsev, 2010. Stem: *Euanom*-.

***Eubaptus* Lacordaire, 1845**: 226, 605. Current status: valid genus in CHRYSOMELIDAE: BRUCHINAE: EUBAPTINI.

Type species: *Eubaptuspalliatus* Lacordaire, 1845, by monotypy.

Family-group name: EUBAPTINAE Bridwell, 1932. Stem: *Eubapt*-.

***Eubolbitus* Reitter, 1892**: 125. Current status: valid genus in BOLBOCERATIDAE: BOLBOCERATINAE: EUBOLBITINI.

Type species: *Bolbocerasradoszkovskii* Solsky, 1876, by monotypy.

Family-group name: EUBOLBITINI Nikolajev, 1970. Stem: *Eubolbit*-.

***Eubria* Germar, 1818**: 239. Current status: valid genus in PSEPHENIDAE: EUBRIINAE. Note: notwithstanding Löbl’s (2016: 32) statement, this name was first proposed as a junior synonym of *Cyphon* Paykull, 1799 and subsequently treated as the name of a valid taxon before 1961 by several authors, including Latreille (1829a: 462); therefore, it dates from its first publication as a synonym (ICZN 1999a, Article 11.6.1).

Type species: *Cyphonpalustris* Germar, 1818, by monotypy. Note: name placed on the Official List of Specific Names in Zoology (ICZN 2011b).

Family-group name: EUBRIADES Lacordaire, 1857. Stem: *Eubri*-.

***Eubrianax* Kiesenwetter, 1874**: 246. Current status: valid genus in PSEPHENIDAE: EUBRIANACINAE.

Type species: *Eubrianaxramicornis* Kiesenwetter, 1874, by monotypy.

Family-group name: EUBRIANACINI Jakobson, 1913. Stem: *Eubrianac*-.

***Eucaerus* J.L. LeConte, 1853e**: 384, 386. Current status: valid genus in CARABIDAE: HARPALINAE: LACHNOPHORINI: LACHNOPHORINA.

Type species: *Eucaerusvaricornis* J.L. LeConte, 1853, by monotypy.

Family-group name: EUCAERI J.L. LeConte, 1861. Stem: *Eucaer*-.

***Eucallia* Guérin-Méneville, 1844c**: pl. 144. Current status: junior synonym of *Callidema* Guérin- Méneville, 1843 in CICINDELIDAE: CICINDELINI: IRESIINA.

Type species: *Callidemaboussingaultii* Guérin-Méneville, 1843, by monotypy.

Family-group name: EUCALLIINI W. Horn, 1893. Stem: *Eucalli*-.

***Eucallopistus* Bellamy, 2003**: 32. Current status: junior synonym of *Pseudocallopistus* Obenberger, 1942 in BUPRESTIDAE: CHRYSOCHROINAE: CHRYSOCHROINI: EUCALLOPISTINA. Note: new replacement name for *Callopistus* Deyrolle, 1865.

Type species: *Callopistuscastelnaudii* Deyrolle, 1865, by original designation (type fixation for *Callopistus* Deyrolle).

Family-group name: EUCALLOPISTINA Bellamy, 2003. Stem: *Eucallopist*-.

***Eucalosphaera* Jelínek, 1978**: 217. Current status: valid genus in NITIDULIDAE: CRYPTARCHINAE: EUCALOSPHAERINI. Note: new replacement name for *Calosphaera* Jelínek, 1974.

Type species: *Cryptarchaocularis* Reitter, 1875, by original designation (type fixation for *Calosphaera* Jelínek).

Family-group name: EUCALOSPHAERINI Kirejtshuk, 1987. Stem: *Eucalosphaer*-.

***Eucanthus* Westwood, 1848c**: 387. Current status: valid genus in BOLBOCERATIDAE: BOLBOCERATINAE: EUCANTHINI.

Type species: *Scarabaeusmeliboeus* Fabricius, 1792 (= *Scarabaeuslazarus* Fabricius, 1775), by monotypy.

Family-group name: EUCANTHINI Nikolajev, 2003. Stem: *Eucanth*-.

***Eucatops* Portevin, 1903**: 157, 162. Current status: valid genus in LEIODIDAE: CHOLEVINAE: EUCATOPINI.

Type species: *Eucatopscurvipes* Portevin, 1903, by subsequent designation (Jeannel 1922: 39).

Family-group name: EUCATOPINI Jeannel, 1921. Stem: *Eucatop*-.

***Eucheila* Dejean in Dejean and Boisduval, 1829**: 60. Current status: valid genus in CARABIDAE: HARPALINAE: LEBIINI: PERICALINA. Note: the original spelling was *Eucheyla* but *Eucheila*, an incorrect subsequent spelling, is in prevailing usage, attributed to the publication of the original spelling, and therefore deemed to be the correct original spelling (ICZN 1999a, Article 33.3.1).

Type species: *Eucheilaflavilabris* Dejean, 1829, by subsequent monotypy (Dejean and Boisduval, 1830: 178).

Family-group name: EUCHEILINAE H.W. Bates, 1883. Stem: *Eucheil*-.

***Euchirus* W. Kirby in W. Kirby and Spence 1828b**: 636. Current status: valid genus in SCARABAEIDAE: MELOLONTHINAE: EUCHIRINI.

Type species: *Scarabaeuslongimanus* Linnaeus, 1758, by monotypy.

Family-group name: EUCHEIRIDAE Hope, 1841. Stem: *Euchir*-.

***Euchlora* W.S. MacLeay, 1819**: 147. Current status: junior synonym of *Anomala* Leach, 1819 in SCARABAEIDAE: RUTELINAE: ANOMALINI: ANOMALINA.

Type species: *Melolonthaviridis* Fabricius, 1775, by subsequent designation (Hope 1837: 71).

Family-group name: EUCHLORIDAE Hope, 1839. Stem: *Euchlor*-. Note: nomen oblitum (see A.B.T. Smith 2006: 173).

***Euchroa* Brullé, 1834**: 330, 335. Current status: valid genus in CARABIDAE: HARPALINAE: PTEROSTICHINI: EUCHROINA.

Type species: *Euchroanitidicollis* Brullé, 1834, by monotypy.

Family-group name: EUCHROIDES Chaudoir, 1874. Stem: *Euchro*-.

***Euchroea* Burmeister, 1842**: 571. Current status: valid genus in SCARABAEIDAE: CETONIINAE: STENOTARSIINI: EUCHROEINA.

Type species: *Euchroeaaurora* Burmeister, 1842, by subsequent designation (Pouillaude 1916: 66).

Family-group name: EUCHROEINA Paulian & Descarpentries, 1982. Stem: *Euchroe*-. Note: junior homonym of EUCHROEIDAE Dahlbom, 1854 (type genus *Euchroeus* Latreille, 1809) in Hymenoptera.

***Euchroma* Dejean, 1833a**: 76. Current status: valid genus in BUPRESTIDAE: CHRYSOCHROINAE: PARALEPTODEMINI: EUCHROMATINA.

Type species: *Buprestisgigantea* Linnaeus, 1758, by monotypy.

Family-group name: EUCHROMATINA Holyński, 1993. Stem: *Euchromat*-.

***Eucibdelus* Kraatz, 1859**: 70. Current status: valid genus in STAPHYLINIDAE: STAPHYLININAE: STAPHYLININI: STAPHYLININA.

Type species: *Eucibdelusgracilis* Kraatz, 1859, by monotypy.

Family-group name: EUCIBDELINI Sharp, 1889. Stem: *Eucibdel*-.

***Eucinetus* Germar, 1818**: 255. Current status: valid genus in EUCINETIDAE.

Type species: *Scaphidiumhaemorrhoidale* Germar, 1818, by monotypy.

Family-group name: EUCINÉTIDES Lacordaire, 1857. Stem: *Eucinet*-.

***Eucnemis* Ahrens, 1812**: 38. Current status: valid genus in EUCNEMIDAE: EUCNEMINAE: EUCNEMINI.

Type species: *Eucnemiscapucinus* Ahrens, 1812, by monotypy.

Family-group name: EUCNEMIDES Eschscholtz, 1829. Stem: *Eucnem*-.

***Euconnus* C.G. Thomson, 1859**: 61. Current status: valid genus in STAPHYLINIDAE: SCYDMAENINAE: SCYDMAENITAE: STENICHNINI.

Type species: *Pselaphushirticollis* Illiger, 1798, by original designation.

Family-group name: EUCONNINI Casey, 1897. Stem: *Euconn*-.

***Euconosoma* Cameron, 1918**: 215, 216. Current status: valid genus in STAPHYLINIDAE: TACHYPORINAE: TACHYPORINI: EUCONOSOMATINA. Note: two original spellings were used, including *Eucononosoma* (p. 215) but this spelling was changed in the errata (p. xix) to *Euconosoma*.

Type species: *Euconosomaelegans* Cameron, 1918 (= *Conosomapictum* Bernhauer, 1903), by original designation.

Family-group name: EUCONOSOMINI Cameron, 1918. Stem: *Euconosomat*-.

***Eucrada* J.L. LeConte, 1861**: 202. Current status: valid genus in PTINIDAE: EUCRADINAE: EUCRADINI.

Type species: *Hedobiahumeralis* Melsheimer, 1845, by monotypy.

Family-group name: EUCRADINI J.L. LeConte, 1861. Stem: *Eucrad*-.

***Eucranium* Dejean, 1833**: 135. Current status: valid genus in SCARABAEIDAE: SCARABAEINAE: EUCRANIINI. Note: name previously credited to Brullé, 1838 with *Eucraniumarachnoides* Reiche, 1842 as its type species (by subsequent designation in Reiche 1842: 65); however, see below regarding the earlier establishment of the name of the type species.

Type species: *Ateuchusarachnoides* Lacordaire, 1830, by monotypy. Note: this species name was first established by Lacordaire (1830a: 261).

Family-group name: EUCRANIADAE Burmeister, 1873. Stem: *Eucrani*-.

***Eudactylus* Sallé, 1855**: 266. Current status: invalid senior synonym of *Platycrepidius* Candèze, 1859 in ELATERIDAE: AGRYPNINAE: PLATYCREPIDIINI. Note: junior homonym of *Eudactylus* Fitzinger, 1843 [Reptilia].

Type species: *Eudactyluswapleri* Sallé, 1855, by monotypy.

Family-group name: EUDACTYLITES Candèze, 1859. Stem: *Eudactyl*-. Note: family-group name permanently invalid (ICZN 1999a, Article 39).

***Euderes* Schönherr, 1825**: 582. Current status: valid genus in CURCULIONIDAE: MOLYTINAE: EUDERINI.

Type species: *Rhynchaenuslineicollis* Wiedemann, 1823, by original designation.

Family-group name: EUDÉRIDES Lacordaire, 1865. Stem: *Euder*-. Note: senior homonym of EUDERINI Erdös, 1956 (type genus *Euderus* Haliday, 1844) in Hymenoptera.

***Euderia* Broun, 1880**: 344. Current status: valid genus in BOSTRICHIDAE: EUDERIINAE.

Type species: *Euderiasquamosa* Broun, 1880, by monotypy.

Family-group name: EUDERIITAE Lesne, 1934. Stem: *Euderi*-.

***Eudiagogus* Schönherr, 1840b**: 307. Current status: valid genus in CURCULIONIDAE: ENTIMINAE: ENTIMITAE: EUDIAGOGINI.

Type species: *Promecopsepiscopalis* Gyllenhal, 1834, by original designation.

Family-group name: EUDIAGOGINI J.L. LeConte, 1874. Stem: *Eudiagog*-.

***Eudicronychus* Méquignon, 1931**: 207. Current status: valid genus in ELATERIDAE: ELATERINAE: EUDICRONYCHINI.

Type species: *Dicronychusserraticornis* Laporte, 1840, by original designation.

Family-group name: EUDICRONYCHINAE C. Girard, 1971. Stem: *Eudicronych*-.

***Eudyasmus* Pascoe, 1885**: 275. Current status: valid genus in CURCULIONIDAE: MOLYTINAE: EUDYASMINI.

Type species: *Eudyasmusalbertisii* Pascoe, 1885, by monotypy.

Family-group name: EUDYASMINI Legalov, 2018d: 347. Stem: *Eudyasm*-.

***Eudysantes* Bouchard, Lawrence, Davies & Newton, 2005**: 508. Current status: junior synonym of *Dysantes* Pascoe, 1869 in TENEBRIONIDAE: TENEBRIONINAE: TOXICINI: DYSANTINA. Note: unnecessary new replacement name for *Dysantes* Pascoe, 1869.

Type species: *Dicerodereselongatus* L. Redtenbacher, 1868, by monotypy (type fixation for *Dysantes* Pascoe).

Family-group name: EUDYSANTINA Bouchard, Lawrence, Davies & Newton, 2005. Stem: *Eudysant*-.

***Eugenysa* Chevrolat in Dejean, 1836a**: 368. Current status: valid genus in CHRYSOMELIDAE: CASSIDINAE: MESOMPHALIINI.

Type species: *Cassidagrossa* Linnaeus, 1758, by monotypy.

Family-group name: EUGENYSINI Hincks, 1952. Stem: *Eugenys*-.

***Euglenes* Westwood, 1830a**: 59. Current status: valid genus in ADERIDAE. Note: name placed on the Official List of Generic Names in Zoology (ICZN 1989d).

Type species: *Anthicusoculatus* Paykull, 1798, by subsequent designation (Pic 1900: 3; see ICZN 1989d). Note: name placed on the Official List of Specific Names in Zoology (ICZN 1989d).

Family-group name: EUGLENINI Seidlitz, 1875. Stem: *Euglenes*- (ICZN 1989d).

***Euglochis* Gistel, 1856**: 382. Current status: junior synonym of *Orthocerus* Latreille, 1797 in ZOPHERIDAE: COLYDIINAE: ORTHOCERINI.

Type species: *Dermestesclavicornis* Linnaeus, 1758, by monotypy.

Family-group name: EUGLOCHIDAE Gistel, 1856. Stem: *Euglochin*-.

***Eugnamptus* Schönherr, 1839**: 339. Current status: valid genus in ATTELABIDAE: RHYNCHITINAE: RHYNCHITITAE: EUGNAMPTINI.

Type species: *Anthribuscollaris* Fabricius, 1801 (= *Rhynchitesangustatus* Herbst, 1797), by original designation.

Family-group name: EUGNAMPTINA Voss, 1930. Stem: *Eugnampt*-.

***Eugnomus* Schönherr, 1847**: 45. Current status: valid genus in CURCULIONIDAE: CURCULIONINAE: EUGNOMINI: EUGNOMINA.

Type species: *Eugnomusdurvillei* Schönherr, 1847, by original designation.

Family-group name: EUGNOMIDES Lacordaire, 1863. Stem: *Eugnom*-.

***Eugonus* Schönherr, 1833**: 144. Current status: valid genus in ANTHRIBIDAE: ANTHRIBINAE: EUGONINI.

Type species: *Eugonusvirgatus* Gyllenhal, 1833, by original designation.

Family-group name: EUGONIDES Lacordaire, 1865. Stem: *Eugon*-.

***Eulabis* Eschscholtz, 1829d**: 14. Current status: valid genus in TENEBRIONIDAE: TENEBRIONINAE: EULABINI.

Type species: *Eulabisbicarinata* Eschscholtz, 1829, by subsequent designation (Blaisdell 1932: 44).

Family-group name: EULABES G.H. Horn, 1870. Stem: *Eulab*-.

***Eulichas* Jakobson, 1913**: 727. Current status: valid genus in EULICHADIDAE. Note: new replacement name for *Lichas* Westwood, 1853.

Type species: *Lichasfunebris* Westwood, 1853, by monotypy (type fixation for *Lichas* Westwood).

Family-group name: EULICHADIDAE Crowson, 1973. Stem: *Eulichad*-.

***Eumichthus* J.L. LeConte, 1873**: 190. Current status: valid genus in CERAMBYCIDAE: CERAMBYCINAE: EUMICHTHINI.

Type species: *Eumichthusoedipus* J.L. LeConte, 1873, by monotypy.

Family-group name: EUMICHTHINI Linsley, 1940. Stem: *Eumichth*-.

***Eumicrus* Laporte, 1833b**: 395. Current status: junior synonym of *Scydmaenus* Latreille, 1802 in STAPHYLINIDAE: SCYDMAENINAE: SCYDMAENITAE: SCYDMAENINI.

Type species: *Scydmaenustarsatus* P.W.J. Müller & Kunze, 1822, by subsequent designation (Duponchel 1844b: 496).

Family-group name: EUMICRINI Reitter, 1882. Stem: *Eumicr*-.

***Eumolpus* Weber, 1801**: 28, 52. Current status: valid genus in CHRYSOMELIDAE: EUMOLPINAE: EUMOLPINI. Note: the senior homonym *Eumolpus* Illiger, 1798 was suppressed for the purposes of both Principles of Priority and Homonymy and placed on the Official Index of Rejected and Invalid Names in Zoology while *Eumolpus* Weber, 1801 was placed on the Official List of Generic Names in Zoology (ICZN 2012b).

Type species: *Chrysomelaignita* Fabricius, 1787, by subsequent designation (Hope 1841a: 162; see ICZN 2012b). Note: name placed on the Official List of Specific Names in Zoology (ICZN 2012b).

Family-group name: EUMOLPIDAE Hope, 1841. Stem: *Eumolp*-.

***Eumorphus* Weber, 1801**: 31. Current status: valid genus in ENDOMYCHIDAE: LYCOPERDININAE.

Type species: *Eumorphussumatrae* Weber, 1801 (= *Erotylusquadriguttatus* Illiger, 1800), by monotypy.

Family-group name: EUMORPHIDAE Gistel, 1848. Stem: *Eumorph*-. Note: senior homonym of EUMORPHIDAE Tutt, 1904 (type genus *Eumorpha* Hübner, 1807) in Lepidoptera.

***Eunectes* Erichson, 1832**: 17, 23. Current status: invalid senior synonym of *Eretes* Laporte, 1833 in DYTISCIDAE: DYTISCINAE: ERETINI. Note: junior homonym of *Eunectes* Wagler, 1830 [Reptilia].

Type species: *Dytiscussticticus* Linnaeus, 1767, by monotypy.

Family-group name: EUNECTINI Portevin, 1929. Stem: *Eunect*-. Note: family-group name permanently invalid (ICZN 1999a, Article 39).

***Eunemadus* Portevin, 1914**: 192. Current status: valid genus in LEIODIDAE: CHOLEVINAE: ANEMADINI: EUNEMADINA.

Type species: *Eunemaduschilensis* Portevin, 1914, by subsequent designation (Hatch 1928: 208).

Family-group name: EUNEMADINA Newton, 1998. Stem: *Eunemad*-.

***Eunidia* Erichson, 1843b**: 261. Current status: valid genus in CERAMBYCIDAE: LAMIINAE: EUNIDIINI.

Type species: *Eunidianebulosa* Erichson, 1843, by monotypy.

Family-group name: EUNIDIINI Téocchi, Sudre & Jiroux, 2010. Stem: *Eunidi*-.

***Euomus* Schönherr, 1847**: 52. Current status: valid genus in CURCULIONIDAE: CYCLOMINAE: AMYCTERINI.

Type species: *Amycterusinsculptus* Boheman, 1843, by original designation.

Family-group name: EUOMIDES Lacordaire, 1863. Stem: *Euom*-.

***Euops* Schönherr, 1839**: 318. Current status: valid genus in ATTELABIDAE: ATTELABINAE: EUOPINI.

Type species: *Euopsaustralasiae* Fåhraeus, 1839 (= *Attelabusfalcatus* Guérin-Méneville, 1833), by original designation.

Family-group name: EUOPSINI Voss, 1925. Stem: *Euop*-.

***Eupages* Schönherr, 1834b**: 413. Current status: valid genus in CURCULIONIDAE: CYCLOMINAE: RHYTHIRRININI.

Type species: *Eupagestuberculosus* Gyllenhal, 1834, by original designation.

Family-group name: EUPAGIDESp>Family-group name: EUPAGIDES Lacordaire, 1863. Stem: *Eupag*-.

***Euparia* Lepeletier & Audinet-Serville, 1828**: 357. Current status: valid genus in SCARABAEIDAE: APHODIINAE: EUPARIINI.

Type species: *Eupariacastanea* Lepeletier & Audinet-Serville, 1828, by monotypy.

Family-group name: EUPARIINA A. Schmidt, 1910. Stem: *Eupari*-. Note: nomen protectum (see A.B.T. Smith 2006: 158); senior homonym of EUPARIINI B.D. Valentine, 1960 (type genus *Euparius* Schönherr, 1823) in Coleoptera: ANTHRIBIDAE.

***Euparius* Schönherr, 1823**: 1135. Current status: valid genus in ANTHRIBIDAE: ANTHRIBINAE: CRATOPARINI.

Type species: *Anthribuslunatus* Fabricius, 1801 (= *Macrocephalusmarmoreus* G.-A. Olivier, 1800), by monotypy.

Family-group name: EUPARIINI B.D. Valentine, 1960. Stem: *Eupari*-. Note: junior homonym of EUPARIINI A. Schmidt, 1910 (type genus *Euparia* Lepeletier & Serville, 1828) in Coleoptera: SCARABAEIDAE.

***Euphanias* Fairmaire & Laboulbène, 1856**: 657. Current status: valid genus in STAPHYLINIDAE: OXYTELINAE: EUPHANIINI.

Type species: *Euphaniasinsignicornis* Fairmaire & Laboulbène, 1856 (= *Pholidusinsignis* Mulsant & Rey, 1856), by monotypy.

Family-group name: EUPHANIAE Reitter, 1909. Stem: *Euphani*-.

***Eupholus* Boisduval, 1835**: 363. Current status: valid genus in CURCULIONIDAE: ENTIMINAE: POLYDRUSITAE: EUPHOLINI. Note: we consider *Eupholus* used by Guérin-Méneville (1838: 114) a subsequent usage of *Eupholus* Boisduval, 1835.

Type species: *Geonemusgeoffroyii* Guérin-Méneville, 1831, by subsequent designation (Porion 1993: 9).

Family-group name: EUPHOLINI Günther, 1943. Stem: *Euphol*-.

***Euphoria* Burmeister, 1842**: 370. Current status: valid genus in SCARABAEIDAE: CETONIINAE: CETONIINI: EUPHORIINA.

Type species: *Cetoniasepulcralis* Fabricius, 1801, by subsequent designation (Casey 1915: 298).

Family-group name: EUPHORIAE G.H. Horn, 1880. Stem: *Euphori*-.

***Euplectalecia* Obenberger, 1924**: 10. Current status: valid genus in BUPRESTIDAE: CHRYSOCHROINAE: PARALEPTODEMINI: EUPLECTALECIINA.

Type species: *Haleciapyropus* Kerremans, 1893 (= *Buprestiserythropus* Gory, 1841), by original designation.

Family-group name: EUPLECTALECIINA Holyński, 1993. Stem: *Euplectaleci*-.

***Euplectroscelis* Crotch, 1873b**: 75. Current status: valid genus in CHRYSOMELIDAE: GALERUCINAE: ALTICINI.

Type species: *Euplectroscelisxanti* Crotch, 1873, by monotypy.

Family-group name: EUPLECTROSCELES G.H. Horn, 1889. Stem: *Euplectroscelid*-.

***Euplectus* Leach, 1817**: 80, 82. Current status: valid genus in STAPHYLINIDAE: PSELAPHINAE: EUPLECTITAE: EUPLECTINI.

Type species: *Euplectusreichenbachii* Leach, 1817 (= *Pselaphusnanus* H.G.L. Reichenbach, 1816), by monotypy.

Family-group name: EUPLECTIDAE Streubel, 1839. Stem: *Euplect*-.

***Euplinthus* C. Costa, 1975**: 67. Current status: valid genus in ELATERIDAE: AGRYPNINAE: EUPLINTHINI: EUPLINTHINA.

Type species: *Elaterophthalmicus* Perty, 1830, by original designation.

Family-group name: EUPLINTHINA C. Costa, 1975. Stem: *Euplinth*-.

***Eupoecila* Burmeister, 1842**: 538. Current status: valid genus in SCARABAEIDAE: CETONIINAE: SCHIZORHININI: SCHIZORHININA.

Type species: *Cetoniaaustralasiae* Donovan, 1805, by subsequent designation (Kraatz 1880: 190).

Family-group name: EUPOECILIDAE Kraatz, 1880. Stem: *Eupoecil*-.

***Eupogonius* J.L. LeConte, 1852a**: 159. Current status: valid genus in CERAMBYCIDAE: LAMIINAE: DESMIPHORINI.

Type species: *Desmiphoratomentosa* Haldeman, 1847, by subsequent designation (J. Thomson 1861: 346).

Family-group name: EUPOGONII J.L. LeConte, 1873. Stem: *Eupogoni*-.

***Eupompha* J.L. LeConte, 1858b**: 21. Current status: valid genus in MELOIDAE: MELOINAE: EUPOMPHINI.

Type species: *Eupomphafissiceps* J.L. LeConte, 1858, by monotypy.

Family-group name: EUPOMPHAE J.L. LeConte, 1862. Stem: *Eupomph*-.

***Eupromera* Westwood, 1845b**: 209. Current status: valid genus in CERAMBYCIDAE: LAMIINAE: EUPROMERINI.

Type species: *Eupromeraspryana* Westwood, 1845, by monotypy.

Family-group name: EUPROMERINI Galileo & Martins, 1995. Stem: *Eupromer*-.

***Euprosopus* W.S. MacLeay, 1825**: 9. Current status: valid genus in CICINDELIDAE: CICINDELINI: IRESIINA. Note: this name is attributed to Dejean (1825: 4, 150 [5 September]) in the literature but it was first made available by W.S. MacLeay (1825 [1 June]) when he associated *Cicindelaquadrinotata* Latreille & Dejean, 1822 (as *Euprosopusquadrinotatus* Lat.) with it.

Type species: *Cicindelaquadrinotata* Latreille & Dejean, 1822, by monotypy.

Family-group name: EUPROSOPINI W. Horn, 1893. Stem: *Euprosop*-.

***Eupsenius* J.L. LeConte, 1849**: 90. Current status: valid genus in STAPHYLINIDAE: PSELAPHINAE: GONIACERITAE: BRACHYGLUTINI: EUPSENIINA.

Type species: *Eupseniusglaber* J.L. LeConte, 1849, by monotypy.

Family-group name: EUPSENIINI Park, 1951. Stem: *Eupseni*-.

***Eupsilobius* Casey, 1895**: 454. Current status: junior synonym of *Eidoreus* Sharp, 1885 in EUPSILOBIIDAE.

Type species: *Eupsilobiuspolitus* Casey, 1895, by monotypy.

Family-group name: EUPSILOBIINI Casey, 1895. Stem: *Eupsilobi*-.

***Eurhinus* W. Kirby, 1819**: 427. Current status: invalid senior synonym of *Eurhynchus* W. Kirby, 1828 in BRENTIDAE: EURHYNCHINAE: EURHYNCHINI. Note: two original spellings were used, including *Eurhynus* (p. 428); Zimmerman (1994: 255) acted as First Reviser (ICZN 1999a, Article 24.2.3) and selected the spelling *Eurhinus*; junior homonym of *Eurhinus* Illiger, 1807 [Coleoptera: CURCULIONIDAE].

Type species: *Eurhinusscabrior* W. Kirby, 1819, by subsequent designation (Schönherr 1833: 248; see ICZN 1985e).

Family-group name: EURHININI Kissinger, 1968. Stem: *Eurhin*-. Note: junior homonym of EURHININA Lacordaire, 1865 (based on *Eurhinus* Illiger, 1807) in Coleoptera: CURCULIONIDAE; family-group name permanently invalid (ICZN 1999a, Article 39).

***Eurhinus* Illiger, 1807a**: 309. Current status: valid genus in CURCULIONIDAE: BARIDINAE: BARIDINI: EURHININA. Note: this name was originally proposed under the spelling *Eurhin* but this spelling was placed on the Official Index of Rejected and Invalid Generic Names in Zoology, *Eurhinus* was considered as the correct original spelling and *Eurhinus* Illiger, 1807 placed on the Official List of Generic Names in Zoology (ICZN 1985e).

Type species: *Eurhinuscupratus* Illiger, 1807, by monotypy (see ICZN 1985e). Note: name placed on the Official List of Specific Names in Zoology (ICZN 1985e).

Family-group name: EURHINIDES Lacordaire, 1865. Stem: *Eurhin*-. Note: senior homonym of EURHININI Kissinger, 1968 (type genus *Eurhinus* W. Kirby, 1819) in Coleoptera: BRENTIDAE; Kissinger’s taxon is permanently invalid (based on a preoccupied type genus).

***Eurhynchus* W. Kirby in W. Kirby and Spence, 1828a**: 324. Current status: valid genus in BRENTIDAE: EURHYNCHINAE: EURHYNCHINI. Note: new replacement name for *Eurhinus* W. Kirby, 1819; the senior homonym *Eurhynchus* Berthold, 1827 was suppressed for the purposes of both the Principle of Priority and Homonymy and placed on the Official Index of Rejected and Invalid Generic Names in Zoology (see ICZN 1985e).

Type species: *Eurhinusscabrior* W. Kirby, 1819, by subsequent designation (Schönherr 1833: 248; see ICZN 1985e).

Family-group name: EURHYNCHIDES Lacordaire, 1863. Stem: *Eurhynch*-.

***Eurispa* Baly, 1858**: 85. Current status: valid genus in CHRYSOMELIDAE: CASSIDINAE: EURISPINI.

Type species: *Eurispavittata* Baly, 1858, by original designation.

Family-group name: EURISPITES Chapuis, 1875. Stem: *Eurisp*-.

***Europs* Wollaston, 1854**: 149. Current status: valid genus in MONOTOMIDAE: MONOTOMINAE: EUROPINI.

Type species: *Europsimpressicollis* Wollaston, 1854, by monotypy.

Family-group name: EUROPINI Sen Gupta, 1988. Stem: *Europ*-.

***Eurrhacus* C.O. Waterhouse, 1880a**: 24. Current status: valid genus in LYCIDAE: LYCINAE: EURRHACINI.

Type species: *Eurrhacustristis* C.O. Waterhouse, 1880, by monotypy.

Family-group name: EURRHACINA Bocáková, 2005. Stem: *Eurrhac*-.

***Eurychora* Thunberg, 1789**: 9. Current status: valid genus in TENEBRIONIDAE: PIMELIINAE: ADELOSTOMINI. Note: the original spelling was *Evrychora* but *Eurychora*, an incorrect subsequent spelling, is in prevailing usage and attributed to the original publication, and therefore deemed to be the correct original spelling (ICZN 1999a, Article 33.3.1).

Type species: *Pimeliaciliata* Fabricius, 1781, by monotypy.

Family-group name: EURYCHORIDAE Hope, 1841. Stem: *Eurychor*-.

***Eurygenius* LaFerté-Sénectère in Guérin-Méneville, 1849**: [No 17] 1. Current status: valid genus in ANTHICIDAE: EURYGENIINAE: EURYGENIINI.

Type species: *Eurygeniusreichei* LaFerté-Sénectère, 1849, by monotypy.

Family-group name: EURYGENII J.L. LeConte, 1862. Stem: *Eurygeni*-.

***Eurylobus* Schönherr, 1826**: 152. Current status: valid genus in CURCULIONIDAE: ENTIMINAE: ENTIMITAE: EURYLOBINI.

Type species: *Hypsonotuscingulatus* Germar, 1823, by original designation (p. 13).

Family-group name: EURYLOBI Jekel, 1856. Stem: *Eurylob*-.

***Eurymetopon* Eschscholtz, 1831**: 5, 8. Current status: valid genus in TENEBRIONIDAE: PIMELIINAE: EDROTINI.

Type species: *Eurymetoponrufipes* Eschscholtz, 1831, by subsequent designation (Casey 1907: 288).

Family-group name: EURYMETOPONINI Casey, 1907. Stem: *Eurymetop*-.

***Eurymycter* J.L. LeConte in J.L. LeConte and G.H. Horn, 1876**: 394. Current status: valid genus in ANTHRIBIDAE: ANTHRIBINAE: TROPIDERINI.

Type species: *Macrocephalusfasciatus* A.-G. Olivier, 1800, by monotypy.

Family-group name: EURYMYCTERINI Pierce, 1930. Stem: *Eurymycter*-.

***Eurynotus* W. Kirby, 1819**: 418. Current status: valid genus in TENEBRIONIDAE: BLAPTINAE: PLATYNOTINI: EURYNOTINA.

Type species: *Eurynotusmuricatus* W. Kirby, 1819 (= *Helopscapensis* Fabricius, 1794), by monotypy.

Family-group name: EURYNOTAIRES Mulsant & Rey, 1854. Stem: *Eurynot*-.

***Euryoda* Lacordaire, 1842b**: 12, 27. Current status: junior synonym of *Heptodonta* Hope, 1838 in CICINDELIDAE: CICINDELINI: CICINDELINA. Note: unnecessary new replacement name for *Heptodonta* Hope, 1838.

Type species: *Cicindelaanalis* Fabricius, 1801, by original designation (type fixation for *Heptodonta* Hope).

Family-group name: EURYODINI W. Horn, 1899. Stem: *Euryod*-.

***Euryope* Dalman, 1824**: 17. Current status: valid genus in CHRYSOMELIDAE: EUMOLPINAE: EURYOPINI.

Type species: *Eumolpusruber* Latreille, 1807 (= *Cryptocephalussubserricornis* Latreille, 1805), by subsequent designation (Blanchard in Audouin et al. 1841: pl. 72). Note: the type species usually recognized is *Cryptocephalusrubrifrons* Fabricius, 1787 by subsequent designation of Moseyko and Sprecher-Uebersax (2010a: 83) but Blanchard’s typification is earlier; both species are currently included in the genus *Euryope*.

Family-group name: EURYOPITES Chapuis, 1874. Stem: *Euryop*-.

***Eurypalpus* J.L. LeConte, 1850b**: 241. Current status: invalid senior synonym of *Psephenus* Haldeman, 1853 in PSEPHENIDAE: PSEPHENINAE. Note: junior homonym of *Eurypalpus* Macquart, 1835 [Diptera].

Type species: *Eurypalpuslecontei* J.L. LeConte, 1850, by monotypy.

Family-group name: EURYPALPINI J.L. LeConte, 1852. Stem: *Eurypalp*-. Note: family-group name permanently invalid (ICZN 1999a, Article 39).

***Eurypoda* Saunders, 1853**: 109. Current status: valid genus in CERAMBYCIDAE: PRIONINAE: EURYPODINI.

Type species: *Eurypodaantennata* Saunders, 1853, by monotypy.

Family-group name: EURYPODINI Gahan, 1906. Stem: *Eurypod*-.

***Eurypogon* Motschulsky, 1860a**: 358, 363. Current status: valid genus in ARTEMATOPODIDAE: ARTEMATOPODINAE: MACROPOGONINI.

Type species: *Ochinanigra* Melsheimer, 1845, by monotypy.

Family-group name: EURYPOGONIDAE Böving & Craighead, 1931. Stem: *Eurypogon*-.

***Euryptychus* J.L. LeConte, 1852c**: 45, 46. Current status: valid genus in EUCNEMIDAE: MACRAULACINAE: EURYPTYCHINI.

Type species: *Eucnemisheterocerus* Say, 1839, by monotypy.

Family-group name: EURYPTYCHINI Mamaev, 1976. Stem: *Euryptych*-.

***Eurypus* W. Kirby, 1819**: 389. Current status: valid genus in MYCTERIDAE: EURYPINAE.

Type species: *Eurypusrubens* W. Kirby, 1819, by monotypy.

Family-group name: EURYPITAE J. Thomson, 1860. Stem: *Euryp*-.

***Eurysphindus* J.L. LeConte, 1878b**: 602. Current status: valid genus in SPHINDIDAE: SPHINDINAE.

Type species: *Eurysphindushirtus* J.L. LeConte, 1878, by monotypy.

Family-group name: EURYSPHINDINAE Sen Gupta & Crowson, 1979. Stem: *Eurysphind*-.

***Eurysternus* Dalman, 1824**: 8. Current status: valid genus in SCARABAEIDAE: SCARABAEINAE: EURYSTERNINI.

Type species: *Eurysternusplanus* Dalman, 1824 (= *Scarabaeuscaribaeus* Herbst, 1789), by subsequent designation (Jessop 1985: 1089).

Family-group name: EURYSTERNINI Vulcano, Martínez & Pereira, 1961. Stem: *Eurystern*-. Note: junior homonym of EURYSTERNIDAE Dollo, 1886 (type genus *Eurysternum* J. Meyer, 1839) in Reptilia.

***Eurystethes* Seidlitz, 1916**: 127. Current status: junior synonym of *Aegialites* Mannerheim, 1853 in SALPINGIDAE: AEGIALITINAE. Note: unnecessary new replacement name for *Aegialites* Mannerheim, 1853.

Type species: *Aegialitesdebilis* Mannerheim, 1853, by monotypy (type fixation for *Aegialites* Mannerheim).

Family-group name: EURYSTETHIDAE Seidlitz, 1916. Stem: *Eurysteth*-.

***Eurytrichus* J.L. LeConte, 1847**: 387. Current status: junior synonym of *Anisotarsus* Chaudoir, 1837 in CARABIDAE: HARPALINAE: HARPALINI: ANISODACTYLINA.

Type species: *Feroniaterminata* Say, 1823, by subsequent designation (Emden 1953: 525).

Family-group name: EURYTRICHINI J.L. LeConte, 1847. Stem: *Eurytrich*-. Note: nomen oblitum (see Bouchard et al. 2011: 128).

***Euryusa* Erichson, 1837**: 371. Current status: valid genus in STAPHYLINIDAE: ALEOCHARINAE: HOMALOTINI: BOLITOCHARINA.

Type species: *Euryusasinuata* Erichson, 1837, by monotypy.

Family-group name: EURYUSIDES C.G. Thomson, 1859. Stem: *Euryus*-.

***Eusattus* J.L. LeConte, 1851a**: 131. Current status: valid genus in TENEBRIONIDAE: PIMELIINAE: CONIONTINI.

Type species: *Eusattusdifficilis* J.L. LeConte, 1851, by subsequent designation (Casey 1908: 56).

Family-group name: EUSATTI Casey, 1908. Stem: *Eusatt*-.

***Euscelophilus* Voss, 1925**: 30. Current status: valid genus in ATTELABIDAE: ATTELABINAE: ATTELABINI: EUSCELOPHILINA.

Type species: *Eusceluschinensis* Schilsky, 1906, by subsequent designation (Ter-Minasian 1950: 162).

Family-group name: EUSCELOPHILINA Voss, 1925. Stem: *Euscelophil*-.

***Euscelus* Schönherr, 1833**: 4, 205. Current status: valid genus in ATTELABIDAE: ATTELABINAE: ATTELABINI: EUSCELINA.

Type species: *Attelabusscutellatus* Gyllenhal, 1833 (= *Attelabusscutellatus* Klug, 1829), by original designation.

Family-group name: EUSCELINA Voss, 1925. Stem: *Euscel*-.

***Eusphalerum* Kraatz, 1857c**: 1003. Current status: valid genus in STAPHYLINIDAE: OMALIINAE: EUSPHALERINI.

Type species: *Anthobiumtriviale* Erichson, 1839 (= *Omaliumprimulae* Stephens, 1834), by subsequent designation (Blackwelder 1952: 161).

Family-group name: EUSPHALERINI Hatch, 1957. Stem: *Eusphaler*-.

***Euspilotus* Lewis, 1907**: 320. Current status: valid genus in HISTERIDAE: SAPRININAE: EUSPILOTINI.

Type species: *Euspilotuszonalis* Lewis, 1907, by original designation.

Family-group name: EUSPILOTINI Lackner in Lackner et al. 2024: 56. Stem: *Euspilot*-.

***Eusteniamorpha* Cameron, 1920**: 253. Current status: valid genus in STAPHYLINIDAE: ALEOCHARINAE: EUSTENIAMORPHINI.

Type species: *Eusteniamorpharufa* Cameron, 1920, by monotypy.

Family-group name: EUSTENIAMORPHINI Bernhauer & Scheerpeltz, 1926. Stem: *Eusteniamorph*-.

***Eustilbus* Sharp, 1888**: 253. Current status: junior synonym of *Stilbus* Seidlitz, 1872 in PHALACRIDAE: PHALACRINAE. Note: unnecessary new replacement name for *Stilbus* Seidlitz, 1872.

Type species: *Anisotomatestacea* Panzer, 1797, by subsequent designation (Guillebeau 1894: 283).

Family-group name: EUSTILBINI Guillebeau, 1892. Stem: *Eustilb*-.

***Eustra* Schmidt-Göbel, 1846**: 65. Current status: valid genus in CARABIDAE: PAUSSINAE: OZAENINI.

Type species: *Eustraplagiata* Schmidt-Göbel, 1846, by monotypy.

Family-group name: EUSTRINI Jeannel, 1946. Stem: *Eustr*-.

***Eustrophus* Illiger, 1802**: 301. Current status: valid genus in TETRATOMIDAE: EUSTROPHINAE: EUSTROPHINI.

Type species: *Mycetophagusdermestoides* Fabricius, 1792, by monotypy.

Family-group name: EUSTROPHIDAE Gistel, 1848. Stem: *Eustroph*-.

***Eustylus* Schönherr, 1842b**: 40. Current status: valid genus in CURCULIONIDAE: ENTIMINAE: POLYDRUSITAE: EUSTYLINI: EUSTYLINA.

Type species: *Curculiopuber* G.-A. Olivier, 1807, by original designation.

Family-group name: EUSTYLIDES Lacordaire, 1863. Stem: *Eustyl*-.

***Eusyneta* Gistel, 1856**: 356. Current status: junior synonym of *Callistus* Bonelli, 1810 in CARABIDAE: HARPALINAE: CHLAENIINI: CALLISTINA.

Type species: *Carabuslunatus* Fabricius, 1775, by monotypy.

Family-group name: EUSYNETADAE Gistel, 1856. Stem: *Eusynet*-.

***Eutelus* Solier, 1843**: 56. Current status: invalid senior synonym of *Eutelonotus* Alluaud, 1902 in TENEBRIONIDAE: STENOCHIINAE: CNODALONINI. Note: two original spellings were used, including *Lutelus* (p. 4); Bouchard et al. (2021: 186) acted as First Revisers (ICZN 1999a, Article 24.2.3) and chose *Eutelus* as the correct original spelling; junior homonym of *Eutelus* Walker, 1834 [Hymenoptera].

Type species: *Eutelusrequieni* Solier, 1843, by subsequent designation (R. Lucas 1920: 291).

Family-group name: EUTÉLIDES Lacordaire, 1859. Stem: *Eutel*-. Note: family-group name permanently invalid (ICZN 1999a, Article 39).

***Eutheia* Stephens, 1830**: 59. Current status: valid genus in STAPHYLINIDAE: SCYDMAENINAE: CEPHENNIITAE: EUTHEIINI.

Type species: *Eutheiascydmaenoides* Stephens, 1830, by subsequent monotypy (Stephens 1830: 115).

Family-group name: EUTHEIINI Casey, 1897. Stem: *Euthei*-.

***Eutomus* Lacordaire, 1865**: 369. Current status: junior synonym of *Rhipidandrus* J.L. LeConte, 1862 in TENEBRIONIDAE: TENEBRIONINAE: BOLITOPHAGINI.

Type species: *Eutomusmicrographus* Lacordaire, 1865, by subsequent designation (Barber 1914: 191).

Family-group name: EUTOMIDES Lacordaire, 1866. Stem: *Eutom*-.

***Eutoxus* Schönherr, 1844**: 103. Current status: valid genus in CURCULIONIDAE: BARIDINAE: EUTOXINI: EUTOXINA.

Type species: *Eutoxusreflexus* Boheman, 1844, by original designation.

Family-group name: EUTOXIDES Champion, 1908. Stem: *Eutox*-.

***Eutrachelus* Berthold, 1827**: 383. Current status: valid genus in BRENTIDAE: BRENTINAE: BRENTINI: ARRENODINA. Note: the original spelling *Entrachelus* (p. 383) was corrected to *Eutrachelus* (p. 575).

Type species: *Brentustemminckii* Latreille, 1825, by monotypy.

Family-group name: EUTRACHÉLIDES Lacordaire, 1865. Stem: *Eutrachel*-.

***Eutryptes* Gistel, 1856**: 375. Current status: valid subgenus of *Mycterus* Clairville, 1798 in MYCTERIDAE: MYCTERINAE.

Type species: *Bruchusumbellatarum* Fabricius, 1787, by subsequent designation (Löbl 2008b: 412).

Family-group name: FUTRYPTEIDAE Gistel, 1856. Stem: *Eutrypt*-.

***Euxestus* Wollaston, 1858**: 411. Current status: valid genus in EUXESTIDAE.

Type species: *Euxestusparkii* Wollaston, 1858, by monotypy.

Family-group name: EUXESTINAE Grouvelle, 1908. Stem: *Euxest*-. Note: senior homonym of EUXESTINAE Hennig, 1940 (type genus *Euxesta* Loew, 1868) in Diptera.

***Evambates* Gistel, 1856**: 365. Current status: junior synonym of *Trichius* Fabricius, 1775 in SCARABAEIDAE: CETONIINAE: TRICHIINI.

Type species: *Scarabaeusfasciatus* Linnaeus, 1758, by subsequent designation (Bouchard and Bousquet 2020a: 84). Note: name placed on the Official List of Specific Names in Zoology (ICZN 2004d).

Family-group name: EVAMBATEIDAE Gistel, 1856. Stem: *Evambat*-.

***Evaniocera* Guérin-Méneville in Guérin-Méneville and Percheron, 1835**: pl. 2. Current status: junior synonym of *Ptilophorus* Dejean, 1834 in RIPIPHORIDAE: PTILOPHORINAE. Note: two original spellings were used, including *Evanicocera* (p. [4]); Mannerheim (1838: 76) acted as First Reviser (ICZN 1999a, Article 24.2.3) and selected *Evaniocera*.

Type species: *Pelecotomadufourii* Latreille, 1817, by original designation.

Family-group name: ÉVANIOCÉRIDES Lacordaire, 1859. Stem: *Evaniocer*-.

***Evaniosomus* Guérin-Méneville, 1834b**: pl. 109bis (p. 14). Current status: valid genus in TENEBRIONIDAE: PIMELIINAE: EVANIOSOMINI.

Type species: *Evaniosomusorbignianus* Guérin-Méneville, 1834, by monotypy.

Family-group name: ÉVANIOSOMIDES Lacordaire, 1859. Stem: *Evaniosom*-.

***Evaspistes* Gistel, 1856**: 381. Current status: junior synonym of *Cassida* Linnaeus, 1758 in CHRYSOMELIDAE: CASSIDINAE: CASSIDINI.

Type species: *Cassidaviridis* Linnaeus, 1758, by **present designation**.

Family-group name: EVASPIDOTIDAE Gistel, 1856. Stem: *Evaspist*-.

***Evides* Dejean, 1833a**: 77. Current status: valid genus in BUPRESTIDAE: CHRYSOCHROINAE: EVIDINI.

Type species: *Buprestiselegans* Fabricius, 1781, by subsequent designation (Bellamy 1997a: 370).

Family-group name: EVIDINI Tôyama, 1987. Stem: *Evid*-.

***Evodinus* LeConte, 1850a**: 325. Current status: valid genus in CERAMBYCIDAE: LEPTURINAE: EVODININI.

Type species: *Lepturamonticola* Randall, 1838, by monotypy.

Family-group name: EVODININI Zamoroka, 2022: 303. Stem: *Evodin*-. Note: two original spellings were used, EVODININI (pp. 297, 301) and EVODINIINI (pp. 302, 303); we **here select**EVODININI as the correct original spelling.

***Evotus* J.L. LeConte, 1874a**: 459. Current status: valid genus in CURCULIONIDAE: ENTIMINAE: POLYDRUSITAE: EVOTINI.

Type species: *Otiorhynchusnaso* J.L. LeConte, 1857, by monotypy.

Family-group name: EVOTINI J.L. LeConte, 1874. Stem: *Evot*-.

***Exagistus* Deyrolle, 1865**: 65. Current status: valid genus in BUPRESTIDAE: BUPRESTINAE: EXAGISTINI.

Type species: *Exagistusigniceps* Deyrolle, 1865, by monotypy.

Family-group name: EXAGISTINI Tôyama, 1987. Stem: *Exagist*-.

***Exapion* Bedel, 1887**: 360. Current status: valid genus in BRENTIDAE: APIONINAE: APIONITAE: EXAPIINI.

Type species: *Curculiofuscirostris* Fabricius, 1775, by subsequent designation (Schilsky 1901: 38F).

Family-group name: EXAPIINI Alonso-Zarazaga, 1990. Stem: *Exapi*-.

***Exocentrus* Dejean, 1835**: 339. Current status: valid genus in CERAMBYCIDAE: LAMIINAE: EXOCENTRINI.

Type species: **here fixed** (under ICZN 1999a, Article 70.3.2) as *Cerambyxlusitanus* Linnaeus, 1767, misidentified as *Cerambyxbalteus* Linnaeus, 1767 (sensu Dejean, 1835), in the original designation by monotypy in Dejean (1835: 339). Note: the nominal species *Cerambyxbalteus* Linnaeus, 1767 is a valid species in the genus *Parmena* Dejean, 1821 of the tribe PARMENINI Mulsant, 1839 (see Bousquet and Bouchard 2013a: 82).

Family-group name: EXOCENTRINAE Pascoe, 1864. Stem: *Exocentr*-.

***Exomella* Casey, 1914**: 378. Current status: valid genus in BYRRHIDAE: BYRRHINAE: EXOMELLINI. Note: new replacement name for *Exoma* Casey, 1908.

Type species: *Exomapleuralis* Casey, 1908, by original designation (type fixation for *Exoma* Casey).

Family-group name: EXOMELLINI Casey, 1914. Stem: *Exomell*-.

***Exophthalmodes* Pierce, 1916**: 464. Current status: junior synonym of *Exophthalmus* Schönherr, 1823 in CURCULIONIDAE: ENTIMINAE: POLYDRUSITAE: EUSTYLINI: EUSTYLINA. Note: unnecessary new replacement name for *Exophthalmus* Schönherr, 1823.

Type species: *Curculioquadrivittatus* G.-A. Olivier, 1807, by original designation (type fixation for *Exophthalmus* Schönherr).

Family-group name: EXOPHTHALMODINA Voss, 1954. Stem: *Exophthalmod*-.

***Exophthalmus* Schönherr, 1823**: 1140. Current status: valid genus in CURCULIONIDAE: ENTIMINAE: EUSTYLINI: EUSTYLINA.

Type species: *Curculioquadrivittatus* G.-A. Olivier, 1807, by original designation.

Family-group name: EXOPHTHALMINI G.H. Horn, 1876. Stem: *Exophthalm*-.

***Exoplectra* Chevrolat in Dejean, 1836a**: 437. Current status: valid genus in COCCINELLIDAE: COCCINELLINAE: COCCIDULINI.

Type species: *Coccinellacoccinea* Fabricius, 1801, by subsequent designation (Korschefsky 1932: 227).

Family-group name: EXOPLECTRIDES Crotch, 1874. Stem: *Exoplectr*-.

***Exosternus* Lewis, 1902**: 233. Current status: valid genus in HISTERIDAE: HISTERINAE: EXOSTERNINI.

Type species: *Exosternusmanicatus* Lewis, 1902, by subsequent designation (Bickhardt 1919: 206).

Family-group name: EXOSTERNINI Bickhardt, 1914. Stem: *Exostern*-.

***Exothispa* Kolbe, 1897a**: 354. Current status: valid genus in CHRYSOMELIDAE: CASSIDINAE: EXOTHISPINI.

Type species: *Exothispareimeri* Kolbe, 1897, by monotypy.

Family-group name: EXOTHISPINI Weise, 1911. Stem: *Exothisp*-.

***Fabia* Martínez & Viana, 1964**: 8. Current status: invalid senior synonym of *Fabrasia* Martínez & Viana, 1964 in PTINIDAE incertae sedis. Note: junior homonym of *Fabia* Dana, 1851 [Crustacea].

Type species: *Fabiaalvarengai* Martínez & Viana, 1964, by original designation.

Family-group name: FABIINAE Martínez & Viana, 1964. Stem: *Fabi*-. Note: family-group name permanently invalid (ICZN 1999a, Article 39).

***Fabrasia* Martínez & Viana, 1965**: 18. Current status: valid genus in PTINIDAE incertae sedis. Note: new replacement name for *Fabia* Martínez & Viana, 1964.

Type species: *Fabiaalvarengai* Martínez & Viana, 1964, by original designation (type fixation for *Fabia* Martínez & Viana).

Family-group name: FABRASIINAE Lawrence & Reichardt, 1966. Stem: *Fabrasi*-.

***Falagria* Leach in Samouelle, 1819**: 177. Current status: valid genus in STAPHYLINIDAE: ALEOCHARINAE: FALAGRIINI.

Type species: *Staphylinussulcatus* Paykull, 1789 (= *Falagriacaesa* Erichson, 1837), by original designation.

Family-group name: FALAGRIATES Mulsant & Rey, 1873. Stem: *Falagri*-.

***Falklandius* Enderlein, 1907**: 65. Current status: valid genus in CURCULIONIDAE: CYCLOMINAE: LISTRODERINI: FALKLANDIINA.

Type species: *Falklandiusbrachyomma* Enderlein, 1907 (= *Otiorhynchusantarcticus* Stierlin, 1903), by original designation.

Family-group name: FALKLANDIINA Morrone, 2013: 42. Stem: *Falklandi*-.

***Falsamblesthis* Breuning, 1959**: 85. Current status: valid genus in CERAMBYCIDAE: LAMIINAE: FORSTERIINI.

Type species: *Amblesthisseriepilosa* Kirsch, 1890, by original designation.

Family-group name: FALSAMBLESTHIINI Gilmour, 1961. Stem: *Falsamblesth*-.

***Falsocossyphus* Pic, 1916a**: 4. Current status: valid genus in TENEBRIONIDAE: TENEBRIONINAE: FALSOCOSSYPHINI.

Type species: *Falsocossyphuspilosus* Pic, 1916, by monotypy.

Family-group name: FALSOCOSSYPHINI Ferrer, 2006. Stem: *Falsocossyph*-.

***Falsomycterus* Pic, 1907**: 127. Current status: valid genus in TENEBRIONIDAE: PIMELIINAE: FALSOMYCTERINI.

Type species: *Falsomycterusdiversipes* Pic, 1907, by monotypy.

Family-group name: FALSOMYCTERINAE Gebien, 1910. Stem: *Falsomycter*-.

***Faronus* Aubé, 1844**: 157. Current status: valid genus in STAPHYLINIDAE: PSELAPHINAE: FARONITAE.

Type species: *Faronuslafertei* Aubé, 1844, by monotypy.

Family-group name: FARONIDES Reitter, 1882. Stem: *Faron*-.

***Febra* Clark, 1864**: 261. Current status: valid genus in CHRYSOMELIDAE: GALERUCINAE: ALTICINI.

Type species: *Febravenusta* Clark, 1864, by original designation.

Family-group name: FEBRAINA Nadein, 2013: 499. Stem: *Febra*- (ICZN 1999a, Article 29.4).

***Felda* Blackwelder, 1952**: 165. Current status: junior synonym of *Termitobiella* Wasmann, 1916 in STAPHYLINIDAE: ALEOCHARINAE: FELDINI. Note: new replacement name for *Asticta* Wasmann, 1916.

Type species: *Astictabutteli* Wasmann, 1916, by monotypy (type fixation for *Asticta* Wasmann).

Family-group name: FELDINI Kistner, 1972. Stem: *Feld*-.

***Fenderia* Hatch, 1957**: 245. Current status: valid genus in STAPHYLINIDAE: EUAESTHETINAE: STICTOCRANIINI.

Type species: *Fenderiacapizzii* Hatch, 1957, by original designation.

Family-group name: FENDERIINI Scheerpeltz, 1974. Stem: *Fenderi*-.

***Feronia* Latreille, 1816**: 191. Current status: junior synonym of *Poecilus* Bonelli, 1810 in CARABIDAE: HARPALINAE: PTEROSTICHINI: PTEROSTICHINA.

Type species: *Carabuscupreus* Linnaeus, 1758, by subsequent designation (Blanchard in Audouin et al. 1842: pl. 22).

Family-group name: FÉRONIENS Dejean, 1825. Stem: *Feroni*-.

***Figulus* W.S. MacLeay, 1819**: 96, 109. Current status: valid genus in LUCANIDAE: LUCANINAE: FIGULINI.

Type species: *Lucanusstriatus* G.-A. Olivier, 1789, by monotypy.

Family-group name: FIGULIDAE Burmeister, 1847. Stem: *Figul*-.

***Flagrax* Kazantsev, 1992**: 38. Current status: valid genus in LYCIDAE: EROTINAE: SLIPINSKIINI.

Type species: *Stadenusauberti* Bourgeois, 1881, by original designation.

Family-group name: FLAGRAXINA Kazantsev, 2004. Stem: *Flagrax*-.

***Flaminius* Kuwert, 1891**: 185. Current status: valid genus in PASSALIDAE: PASSALINAE: SOLENOCYCLINI.

Type species: *Flaminiusnonfriedi* Kuwert, 1891, by monotypy.

Family-group name: FLAMININAE Kuwert, 1891. Stem: *Flamini*-.

***Foadia* Pakaluk, 1985**: 406. Current status: valid genus in CORYLOPHIDAE: CORYLOPHINAE: FOADIINI.

Type species: *Foadiamaculata* Pakaluk, 1985, by original designation.

Family-group name: FOADIINI Ślipiński, Tomaszewska & Lawrence, 2009. Stem: *Foadi*-.

***Foranotum* Nabozhenko & Sadeghi, 2017**: 165. Current status: valid genus in TENEBRIONIDAE: KUHITANGIINAE: FORANOTINI.

Type species: *Foranotumperforatum* Nabozhenko & Sadeghi, 2017, by original designation.

Family-group name: FORANOTINI Nabozhenko & Sadeghi, 2017: 165. Stem: *Foranot*-.

***Forcipator* Maindron, 1904**: 265. Current status: valid genus in CARABIDAE: SCARITINAE: FORCIPATORINI. Note: new replacement name for *Oxystomus* Dejean, 1825.

Type species: *Oxystomuscylindricus* Dejean, 1825, by monotypy (type fixation for *Oxystomus* Dejean).

Family-group name: FORCIPATORINA Bänninger, 1938. Stem: *Forcipator*-.

***Formicomus* LaFerté-Sénectère in Guérin-Méneville, 1849** [No 25]: 1. Current status: junior synonym of *Anthelephila* Hope, 1833 in ANTHICIDAE: ANTHICINAE: FORMICOMINI. Note: unjustified emendation of *Formicoma* Motschulsky, 1845 (see Kejval 2003: 382).

Type species: *Carabuspedestris* Rossi, 1790, by original designation (type fixation for *Formicoma* Motschulsky).

Family-group name: FORMICOMINI Bonadona, 1974. Stem: *Formicom*-.

***Fornax* Laporte, 1835**: 167, 172. Current status: valid genus in EUCNEMIDAE: MACRAULACINAE: MACRAULACINI.

Type species: *Fornaxruficollis* Laporte, 1835, by monotypy.

Family-group name: FORNACINI Beaulieu, 1919. Stem: *Fornac*-. Note: as pointed out by Bouchard and Bousquet (2020a: 87) the names FORNACINI Beaulieu, 1919 and DROMAEOLINI Beaulieu, 1919 take precedence over MACRAULACINAE/-INI Fleutiaux, 1923 and an application to the Commission is necessary to preserve usage of MACRAULACINAE/-INI as valid.

***Forsteria* Tippmann, 1960**: 211. Current status: junior synonym of *Falsamblesthis* Breuning, 1955 in CERAMBYCIDAE: LAMIINAE: FORSTERIINI.

Type species: *Forsteriaunguicularis* Tippmann, 1960, by original designation.

Family-group name: FORSTERIINI Tippmann, 1960. Stem: *Forsteri*-.

***Fruhstorferia* Kolbe, 1894**: 3. Current status: valid genus in SCARABAEIDAE: RUTELINAE: RUTELINI: RUTELINA.

Type species: *Fruhstorferiajavana* Kolbe, 1894, by monotypy.

Family-group name: FRUHSTORFERIINA Ohaus, 1918. Stem: *Fruhstorferi*-.

***Fulcidax* Crotch, 1870b**: 214. Current status: junior synonym of *Poropleura* Lacordaire, 1848 in CHRYSOMELIDAE: CRYPTOCEPHALINAE: FULCIDACINI. Note: this name was first proposed by Voet (1806: 33) but it is not available because Voet’s work is not consistently binominal (ICZN 1999a, Article 11.4); the name was subsequently listed in synonymy by Crotch (1870b: 214) and considered as valid by Clavareau (1913: 222), therefore, the name *Fulcidax* is available and dates from its first publication as a synonym (ICZN 1999a, Article 11.6.1).

Type species: *Clytramonstrosa* Fabricius, 1798, by subsequent designation (Chamorro-Lacayo and Konstantinov 2009: 76). Note: no species were originally included in this genus by Crotch (1870b); the first ones were the six species listed by Clavareau (1913: 223).

Family-group name: FULCIDACINA Jakobson, 1924. Stem: *Fulcidac*-.

***Fustiger* J.L. LeConte, 1866c**: 108, 109. Current status: valid genus in STAPHYLINIDAE: PSELAPHINAE: CLAVIGERITAE: CLAVIGERINI.

Type species: *Articerusbrasiliensis* Westwood, 1856, by subsequent designation (Newton and Chandler 1989: 64).

Family-group name: FUSTIGERINI Jeannel, 1949. Stem: *Fustiger*-.

***Gahania* Distant, 1907**: 423. Current status: valid genus in CERAMBYCIDAE: CERAMBYCINAE: GAHANIINI.

Type species: *Gahaniasimmondsi* Distant, 1907, by monotypy.

Family-group name: GAHANIINI Quentin & Villiers, 1969. Stem: *Gahani*-.

***Galba* Latreille, 1829a**: 451. Current status: invalid senior synonym of *Galbites* Fleutiaux, 1918 in EUCNEMIDAE: EUCNEMINAE: GALBITINI. Note: junior homonym of *Galba* Schrank, 1803 [Mollusca].

Type species: *Galbamarmorata* Guérin-Méneville, 1829, by subsequent monotypy (Guérin- Méneville 1829a: pl. 12).

Family-group name: GABINI Beaulieu, 1919. Stem: *Galb*-. Note: family-group name permanently invalid (ICZN 1999a, Article 39).

***Galbella* Westwood, 1848a**: 83. Current status: valid genus in BUPRESTIDAE: GALBELLINAE.

Type species: *Galbellaviolacea* Westwood, 1848, by monotypy.

Family-group name: GALBELLINAE Reitter, 1911. Stem: *Galbell*-.

***Galbites* Fleutiaux, 1918a**: 59. Current status: valid genus in EUCNEMIDAE: EUCNEMINAE: GALBITINI. Note: new replacement name for *Galba* Latreille, 1829.

Type species: *Galbamarmorata* Guérin-Méneville, 1829, by subsequent monotypy (Guérin- Méneville 1829a: pl. 12) (type fixation for *Galba* Latreille).

Family-group name: GALBITINI Muona, 1991. Stem: *Galbit*-.

***Galerita* Fabricius, 1801a**: xxiii, 214. Current status: valid genus in CARABIDAE: HARPALINAE: GALERITINI: GALERITINA. Note: the senior homonym *Galerita* Gouan, 1770 [Aves] was suppressed both for the Principles of Priority and Homonymy and placed on the Official Index of Rejected and Invalid Names in Zoology while *Galerita* Fabricius, 1801 was placed on the Official List of Generic Names in Zoology (ICZN 1968b).

Type species: *Carabusamericanus* Linnaeus, 1758, by subsequent designation (Latreille 1810: 426; see ICZN 1968b). Note: name placed on the Official List of Specific Names in Zoology (ICZN 1968b).

Family-group name: GALERITIDAE W. Kirby, 1825. Stem: *Galerit*-.

***Galeritina* Jeannel, 1949b**: 1058. Current status: junior synonym of *Galerita* Fabricius, 1801 in CARABIDAE: HARPALINAE: GALERITINI: GALERITINA. Note: new replacement name for *Galerita* Fabricius, 1801.

Type species: *Carabusamericanus* Linnaeus, 1758, by subsequent designation (Latreille 1810: 426) (type fixation for *Galerita* Fabricius).

Family-group name: GALERITININI Jeannel, 1949. Stem: *Galeritin*-. Note: name placed on the Official List of Rejected and Invalid Family-Group Names in Zoology (ICZN 1968b).

***Galeritula* Strand, 1936**: 168. Current status: junior synonym of *Galerita* Fabricius, 1801 in CARABIDAE: HARPALINAE: GALERITINI: GALERITINA. Note: new replacement name for *Galerita* Fabricius, 1801.

Type species: *Carabusamericanus* Linnaeus, 1758, by subsequent designation (Latreille 1810: 426) (type fixation for *Galerita* Fabricius).

Family-group name: GALERITULINI Jedlička, 1963. Stem: *Galeritul*-. Note: name placed on the Official List of Rejected and Invalid Family-Group Names in Zoology (ICZN 1968b).

***Galeruca* Geoffroy, 1762**: 251. Current status: valid genus in CHRYSOMELIDAE: GALERUCINAE: GALERUCINI. Note: name placed on the Official List of Generic Names in Zoology (ICZN 1994a).

Type species: *Chrysomelatanaceti* Linnaeus, 1758, by subsequent designation (Latreille 1810: 432; see ICZN 1994a). Note: name placed on the Official List of Specific Names in Zoology (ICZN 1994a).

Family-group name: GALERUCAE Latreille, 1802. Stem: *Galeruc*-.

***Gallerucida* Motschulsky, 1861**: 24. Current status: valid genus in CHRYSOMELIDAE: GALERUCINAE: HYLASPINI.

Type species: *Gallerucidabifasciata* Motschulsky, 1861, by monotypy.

Family-group name: GALLERUCIDINI Gressitt & Kimoto, 1963. Stem: *Gallerucid*-.

***Gallerucidia* Chaudoir, 1872b**: 415, 417. Current status: valid genus in CARABIDAE: HARPALINAE: LEBIINI: GALLERUCIDIINA.

Type species: *Gallerucidiaoctonotata* Chaudoir, 1872, by subsequent designation (Lorenz 1998: 89).

Family-group name: GALLERUCIDIAE Chaudoir, 1872. Stem: *Gallerucidi*-.

***Galloisia* Hustache, 1920a**: 493. Current status: valid genus in CURCULIONIDAE: CURCULIONINAE: MICROSTYLINI.

Type species: *Galloisiainflata* Hustache, 1920, by original designation.

Family-group name: GALLOISIINAE Morimoto, 1962. Stem: *Galloisi*-.

***Ganglbaueria* Semenov, 1891**: 378. Current status: valid genus in OEDEMERIDAE: OEDEMERINAE: ASCLERINI.

Type species: *Ganglbaueriacollaris* Semenov, 1891, by monotypy.

Family-group name: GANGLBAUERIIDAE Semenov, 1894. Stem: *Ganglbaueri*-.

***Gasterocercus* Laporte & Brullé, 1828**: 198. Current status: valid genus in CURCULIONIDAE: CRYPTORHYNCHINAE: GASTEROCERCINI.

Type species: *Gasterocercusdumerilii* Laporte & Brullé, 1828 (= *Curculiodepressirostris* Fabricius, 1792), by subsequent designation (Chevrolat 1845: 30). Note: Chevrolat (1845) selected “*G.depressicornis* Fab.” (a lapsus for *G.depressirostris* Fab.) as type species, a species not originally included, but since he listed it as a synonym of *Gasterocercusdumerilii* Laporte & Brullé, 1828, a species originally included, he is deemed to have designated the latter species as the type (ICZN 1999a, Article 69.2.2).

Family-group name: GASTEROCERCINI Zherikhin, 1991. Stem: *Gasterocerc*-.

***Gastraulacus* Guérin-Méneville, 1843b**: 188. Current status: valid genus in EUCNEMIDAE: EUCNEMINAE: EUCNEMINI.

Type species: *Galbabisulcatus* Latreille, 1834, by original designation.

Family-group name: GASTRAULACI Fleutiaux, 1902. Stem: *Gastraulac*-.

***Gastrophysa* Chevrolat in Dejean, 1836a**: 405. Current status: valid genus in CHRYSOMELIDAE: CHRYSOMELINAE: CHRYSOMELINI.

Type species: *Chrysomelapolygoni* Linnaeus, 1758, by subsequent designation (Chevrolat 1845: 34).

Family-group name: GASTROPHYSINA Kippenberg, 2010. Stem: *Gastrophys*-. Note: junior homonym of GASTROPHYSINI Harting, 1864 (type genus *Gastrophysus* J. Müller, 1843) in Pisces.

***Gehringia* Darlington, 1933**: 110. Current status: valid genus in CARABIDAE: GEHRINGIINAE: GEHRINGIINI: GEHRINGIINA.

Type species: *Gehringiaolympica* Darlington, 1933, by original designation.

Family-group name: GEHRINGIINI Darlington, 1933. Stem: *Gehringi*-.

***Gempylodes* Pascoe, 1863c**: 123, 132. Current status: valid genus in ZOPHERIDAE: COLYDIINAE: GEMPYLODINI.

Type species: *Gempylodesmacer* Pascoe, 1863, by monotypy.

Family-group name: GEMPYLODINI Sharp, 1893. Stem: *Gempylod*-.

***Genecerus* Walker, 1871**: 13. Current status: valid genus in DASCILLIDAE: KARUMIINAE: GENECERINI.

Type species: *Geneceruscervinus* Walker, 1871, by monotypy.

Family-group name: GENECERINI Pic, 1914. Stem: *Genecer*-.

***Geniates* W. Kirby, 1819**: 401. Current status: valid genus in SCARABAEIDAE: RUTELINAE: GENIATINI.

Type species: *Geniatesbarbatus* W. Kirby, 1819, by monotypy.

Family-group name: GENIATIDAE Burmeister, 1844. Stem: *Geniat*-.

***Genuchus* W. Kirby, 1825**: 569. Current status: valid genus in SCARABAEIDAE: CETONIINAE: CREMASTOCHEILINI: GENUCHINA.

Type species: *Cetoniacruenta* Fabricius, 1787 (= *Cetoniahottentotta* Fabricius, 1775), by original designation.

Family-group name: GENUCHINA Krikken, 1984. Stem: *Genuch*-.

***Genyocerus* Motschulsky, 1858a**: 68. Current status: valid genus in CURCULIONIDAE: PLATYPODINAE: TESSEROCERINI: DIAPODINA.

Type species: *Genyocerusalbipennis* Motschulsky, 1858, by monotypy.

Family-group name: GENYOCERINAE Hopkins, 1915. Stem: *Genyocer*-.

***Geobaenus* Dejean, 1829**: 5, 402. Current status: valid genus in CARABIDAE: HARPALINAE: GEOBAENINI.

Type species: *Geobaenuslateralis* Dejean, 1829, by monotypy.

Family-group name: GEOBAENIDES Péringuey, 1896. Stem: *Geobaen*-.

***Geochus* Broun, 1882a**: 409. Current status: valid genus in CURCULIONIDAE: MOLYTINAE: PHRYNIXINI: GEOCHINA. Note: new replacement name for *Geophilus* Broun, 1880; the same name was also proposed subsequently by Broun (1882b: 128) on [31] May, as opposed to 1 May in Broun (1882a).

Type species: *Geophilusinaequalis* Broun, 1880, by monotypy (type fixation for *Geophilus* Broun).

Family-group name: GEOCHINA Legalov, 2020a: 328. Stem: *Geoch*-.

***Geomorus* Schönherr, 1823**: 1141. Current status: junior synonym of *Cleonis* Dejean, 1821 in CURCULIONIDAE: LIXINAE: CLEONINI.

Type species: *Curculiosulcirostris* Linnaeus, 1767, by original designation.

Family-group name: GEOMORIDES Schönherr, 1823. Stem: *Geomor*-. Note: nomen oblitum (see Bouchard et al. 2011: 620).

***Geonemus* Schönherr, 1833**: 13. Current status: valid genus in CURCULIONIDAE: ENTIMINAE: POLYDRUSITAE: GEONEMINI. Note: this genus-group name is a junior homonym of *Geonemus* Guérin-Méneville, 1831 [Coleoptera: CURCULIONIDAE]; Alonso Zarazaga and Lyal (1999: 159) mentioned that an application will be submitted to the Commission to suppress the senior homonym and that meanwhile current usage should be maintained; name placed on the Official List of Generic Names in Zoology (ICZN 1988d).

Type species: *Curculioflabellipes* G.-A. Olivier, 1807, by plenary power (see ICZN 1988d). Note: name placed on the Official List of Specific Names in Zoology (ICZN 1988d).

Family-group name: GEONEMIDAE Gistel, 1848. Stem: *Geonem*-.

***Geopaederus* Gistel, 1848b**: x, xi. Current status: junior synonym of *Paederus* Fabricius, 1775 in STAPHYLINIDAE: PAEDERINAE: PAEDERINI: PAEDERINA. Note: unnecessary new replacement name for *Paederus* Fabricius, 1775.

Type species: *Staphylinusriparius* Linnaeus, 1758, by subsequent designation (Latreille 1810: 427) (type fixation for *Paederus* Fabricius).

Family-group name: GEOPAEDERIDAE Gistel, 1848. Stem: *Geopaeder*-.

***Georissus* Latreille, 1809a**: 377. Current status: valid genus in GEORISSIDAE. Note: name placed on the Official List of Generic Names in Zoology (ICZN 1998).

Type species: *Pimeliapygmaea* Fabricius, 1798 (= *Byrrhuscrenulatus* Rossi, 1794), by monotypy (see ICZN 1998). Note: *Byrrhuscrenulatus* Rossi, 1794 placed on the Official List of Specific Names in Zoology (ICZN 1998).

Family-group name: GÉORISSITES Laporte, 1840. Stem: *Georiss*-. Note: senior homonym of GEORISSINAE W.T. Blandford, 1864 (type genus *Georissa* W.T. Blandford, 1864) in Mollusca.

***Geostiba* C.G. Thomson, 1858**: 33. Current status: valid genus in STAPHYLINIDAE: ALEOCHARINAE: GEOSTIBINI. Note: *Evanystes* Gistel, 1856, a senior synonym of *Geostiba*, has been suppressed for the purposes of the Principle of Priority but not for the Principle of Homonymy and *Geostiba* C.G. Thomson, 1858 placed on the Official List of Generic Names in Zoo1ogy (ICZN 2005a).

Type species: *Aleocharacircellaris* Gravenhorst, 1806, by monotypy (see ICZN 2005a). Note: name placed on the Officia1 List of Specific Names in Zoo1ogy (ICZN 2005a).

Family-group name: GEOSTIBAE Seevers, 1978. Stem: *Geostib*-.

***Geotrupes* Latreille, 1797**: 6. Current status: valid genus in GEOTRUPIDAE: GEOTRUPINAE: GEOTRUPINI. Note: name placed on the Official List of Generic Names in Zoology (ICZN 1955b).

Type species: *Scarabaeusstercorarius* Linnaeus, 1758, by plenary power (see ICZN 1955b). Note: name placed on the Official List of Specific Names in Zoology (ICZN 1955b).

Family-group name: GEOTRUPINI Latreille, 1802. Stem: *Geotrup*-.

***Geralius* Harold, 1869b**: 123. Current status: valid genus in BUPRESTIDAE: AGRILINAE: CORAEBINI: GERALIINA. Note: new replacement name for *Acanthopygus* Deyrolle, 1864.

Type species: *Stenogasterfurciventris* Chevrolat, 1838, by monotypy (type fixation for *Acanthopygus* Deyrolle).

Family-group name: GERALIINA Cobos, 1988. Stem: *Gerali*-.

***Gerania* Audinet-Serville, 1835a**: 70. Current status: valid genus in CERAMBYCIDAE: LAMIINAE: LAMIINI.

Type species: *Saperdaboscii* Fabricius, 1801, by monotypy.

Family-group name: GERANITAE J. Thomson, 1864. Stem: *Gerani*-.

***Germarica* Blackburn, 1888**: 257. Current status: valid genus in BUPRESTIDAE: AGRILINAE: APHANISTICINI: GERMARICINA.

Type species: *Germaricacasuarinae* Blackburn, 1888, by monotypy.

Family-group name: GERMARICINI Cobos, 1979. Stem: *Germaric*-.

***Ghanius* Ahmad, 1973**: 449. Current status: valid genus in COCCINELLIDAE: COCCINELLINAE: SHIROZUELLINI.

Type species: *Ghaniuskarachiensis* Ahmad, 1973, by monotypy.

Family-group name: GHANIINI Ahmad, 1973. Stem: *Ghani*-.

***Ghidinia* Pavan, 1939**: 106. Current status: junior synonym of *Boldoria* Jeannel, 1924 in LEIODIDAE: CHOLEVINAE: LEPTODIRINI: PHOLEUINA.

Type species: *Ghidiniamorettii* Pavan, 1939, by monotypy.

Family-group name: GHIDINIINA Guéorguiev, 1974. Stem: *Ghidini*-.

***Gibbium* Scopoli, 1777**: 505. Current status: valid genus in PTINIDAE: PTININAE: GIBBIINI.

Type species: *Ptinusscotias* Fabricius, 1781 (= *Scotiaspsylloides* Czenpinski, 1778), by subsequent monotypy (Latreille 1804d: 37).

Family-group name: GIBBIITES Jacquelin du Val, 1860. Stem: *Gibbi*-.

***Gietella* Constantin & Menier, 1987**: 53. Current status: valid genus in RHADALIDAE: GIETELLINAE.

Type species: *Gietellafortunata* Constantin & Menier, 1987, by original designation.

Family-group name: GIETELLINAE Constantin & Menier, 1987. Stem: *Gietell*-.

***Gilletinus* Boucomont, 1932**: 264. Current status: valid genus in BOLBOCERATIDAE: BOLBOCERATINAE: GILLETININI.

Type species: *Bolbocerasmulticostatus* Van Lansberge, 1885, by original designation.

Family-group name: GILLETININI Nikolajev, 1990. Stem: *Gilletin*-.

***Ginema* Ball & Shpeley, 2002**: 83. Current status: valid genus in CARABIDAE: HARPALINAE: GINEMINI.

Type species: *Ginemathomasi* Ball & Shpeley, 2002, by original designation.

Family-group name: GINEMINI Ball & Shpeley, 2002. Stem: *Ginem*-.

***Glacicavicola* Westcott, 1968**: 3. Current status: valid genus in LEIODIDAE: CATOPOCERINAE: GLACICAVICOLINI.

Type species: *Glacicavicolabathyscioides* Westcott, 1968, by original designation.

Family-group name: GLACICAVICOLINAE Westcott, 1968. Stem: *Glacicavicol*-.

***Glandularia* L.W. Schaufuss, 1889**: 3. Current status: junior synonym of *Napochus* C.G. Thomson, 1859 in STAPHYLINIDAE: SCYDMAENINAE: SCYDMAENITAE: STENICHNINI.

Type species: *Glandulariafricatoris* L.W. Schaufuss, 1889, by original designation.

Family-group name: GLANDULARIIDAE L.W. Schaufuss, 1889. Stem: *Glandulari*-.

***Glaphyrometopus* Pierce, 1913**: 406. Current status: valid genus in CURCULIONIDAE: ENTIMINAE: POLYDRUSITAE: NAUPACTINI: NAUPACTINA.

Type species: *Glaphyrometopusornithodorus* Pierce, 1913, by monotypy.

Family-group name: GLAPHYROMETOPI Pierce, 1913. Stem: *Glaphyro­metop*-.

†***Glaphyroptera* Heer, 1852**: 13. Current status: valid genus in BUPRESTIDAE: BUPRESTINAE: †GLAPHYROPTERINI. Note: Lower Jurassic (Switzerland).

Type species: †*Glaphyropterainsignis* Heer, 1852, by subsequent designation (Handlirsch 1906: 452).

Family-group name: †GLAPHYROPTERIDAE Pongrácz, 1935. Stem: *Glaphyropter*-.

***Glaphyrus* Latreille, 1802b**: 150. Current status: valid genus in GLAPHYRIDAE: GLAPHYRINAE.

Type species: *Melolonthaserratulae* Fabricius, 1792, by monotypy. Note: the type species often listed for this genus is *Scarabaeusmaurus* Linnaeus, 1758 (e.g., Nikodým and A. Bezděk 2016: 95) but Latreille (1802b: 151) listed a single species, *serratulae* Fabricius in his new genus; both species are currently included in the nominotypical subgenus of *Glaphyrus*.

Family-group name: GLAPHYRIDAE W.S. MacLeay, 1819. Stem: *Glaphyr*-.

***Glaresis* Erichson, 1848**: 925. Current status: valid genus in GLARESIDAE.

Type species: *Glaresisrufa* Erichson, 1848, by monotypy.

Family-group name: GLARESINI Prudhomme de Borre, 1886. Stem: *Glares*-.

***Glaucytes* J. Thomson, 1858a**: 423. Current status: valid genus in CERAMBYCIDAE: CERAMBYCINAE: GLAUCYTINI. Note: new replacement name for *Leptocera* Dejean, 1821.

Type species: *Cerambyxscriptus* Fabricius, 1798 (= *Cerambyxinterruptus* G.-A. Olivier, 1790), by monotypy (type fixation for *Leptocera* Dejean).

Family-group name: GLAUCYTIDES Lacordaire, 1868. Stem: *Glaucyt*-.

***Glenea* Newman, 1842a**: 301. Current status: valid genus in CERAMBYCIDAE: LAMIINAE: SAPERDINI. Note: new replacement name for *Sphenura* Dejean, 1835.

Type species: *Saperdanovemguttata* Guérin-Méneville, 1825, by subsequent designation (Breuning 1956: 2). Note: this species is not a nominal species originally included in the senior synonym *Sphenura* Dejean (see Article 67.8.1, ICZN 1999a); however, the three originally included nominal species are currently placed in other genera; a request should be submitted to the Commission to retain Breuning’s type species fixation (see Bousquet et al. 2009: 37).

Family-group name: GLENEÏTAE J. Thomson, 1864. Stem: *Glene*-.

***Glischrochilus* Reitter, 1873**: 162. Current status: valid genus in NITIDULIDAE: CRYPTARCHINAE: CRYPTARCHINI.

Type species: *Silphaquadripustulata* Linnaeus, 1761 (= *Silphaquadripunctata* Linnaeus, 1758), by subsequent designation (Parsons 1943: 264).

Family-group name: GLISCHROCHILINI Chagnon, 1934. Stem: *Glischrochil*-.

***Globa* Raffray, 1887**: 37. Current status: valid genus in STAPHYLINIDAE: PSELAPHINAE: GONIACERITAE: BRACHYGLUTINI: BRACHYGLUTINA.

Type species: *Globalongipes* Raffray, 1887, by monotypy.

Family-group name: GLOBINA Jeannel, 1959. Stem: *Glob*-.

***Globulosis* García, 2001**: 153. Current status: valid genus in HYDROPHILIDAE: ACIDOCERINAE.

Type species: *Globulosishemisphericus* García, 2001, by original designation.

Family-group name: GLOBULINA García, 2001. Stem: *Globulos*-.

***Gloeosoma* Wollaston, 1854**: 480. Current status: valid genus in CORYLOPHIDAE: CORYLOPHINAE: RYPOBIINI.

Type species: *Gloeosomavelox* Wollaston, 1854, by monotypy.

Family-group name: GLOEOSOMATINI Bowestead, 1999. Stem: *Gloeosomat*-.

***Glostatus* Schedl, 1939**: 386. Current status: valid genus in CURCULIONIDAE: SCOLYTINAE: XYLOCTONINI: GLOSTATINA.

Type species: *Glostatusdeclividepressus* Schedl, 1939, by monotypy.

Family-group name: GLOSTATINA Jordal, 2023: 203. Stem: *Glostat*-.

***Glycyphana* Burmeister, 1842**: 345. Current status: valid genus in SCARABAEIDAE: CETONIINAE: CETONIINI: CETONIINA.

Type species [suggested]: *Cetoniahorsfieldii* Hope, 1831, by subsequent designation (Arrow 1910: 120). Note: the valid type species fixation is that of Duponchel (1867: 535), who selected *Cetoniatricolor* G.-A. Olivier, 1789, but that nominal species is the type species of the genus *Glycosia* Schoch, 1896; an application to the Commission is necessary to fix *Cetoniahorsfieldii* Hope, 1831 as the type species of *Glycyphana* Burmeister, 1842 to conserve the current usage of the genus-group name.

Family-group name: GLYCYPHANAE Schoch, 1894. Stem: *Glycyphan*-.

***Glypholoma* Jeannel, 1962b**: 482. Current status: valid genus in STAPHYLINIDAE: GLYPHOLOMATINAE.

Type species: *Glypholomapustuliferum* Jeannel, 1962, by original designation.

Family-group name: GLYPHOLOMINI Jeannel, 1962. Stem: *Glypholomat*-.

***Glyptoma* Erichson, 1839b**: 32. Current status: valid genus in STAPHYLINIDAE: OSORIINAE: THORACOPHORINI: GLYPTOMINA.

Type species: *Glyptomacrassicorne* Erichson, 1840, by subsequent designation (Duponchel 1841b: 57). Note: no species were originally included in this genus by Erichson (1839a); the first ones were the six species listed by Erichson (1840a: 908–910).

Family-group name: GLYPTOMINA Newton & Thayer, 1992. Stem: *Glyptom*-.

***Glyptoscelimorpha* G.H. Horn, 1893b**: 137. Current status: valid genus in SCHIZOPODIDAE: DYSTAXIINI.

Type species: *Glyptoscelimorphamarmorata* G.H. Horn, 1893, by monotypy.

Family-group name: GLYPTOSCELIMORPHINI Cobos, 1963. Stem: *Glyptoscelimorph*-.

***Glyptus* Brullé, 1835**: 83. Current status: valid genus in CARABIDAE: HARPALINAE: GLYPTINI. Note: the senior homonym *Glyptus* Hoffmannsegg, 1818 (and all uses of that name prior to the publication of *Glyptus* Brullé, 1835) was suppressed for the purposes both the Principle of Priority and Homonymy and placed on the Official Index of Rejected and Invalid Names in Zoology while *Glyptus* Brullé, 1835 was placed on the Official List of Generic Names in Zoology (ICZN 1985a).

Type species: *Glyptussculptilis* Brullé, 1835, by monotypy (see ICZN 1985a). Note: name placed on the Official List of Specific Names in Zoology (ICZN 1985a).

Family-group name: GLYPTI G.H. Horn, 1881. Stem: *Glypt*-. Note: senior homonym of GLYPTINI Cushman & Rohwer, 1920 (type genus *Glypta* Gravenhorst, 1829) in Hymenoptera.

***Gnaphalocnemis* Heller, 1901**: 10. Current status: junior synonym of *Pelopides* Kuwert, 1896 in PASSALIDAE: PASSALINAE: MACROLININI. Note: new replacement name for *Eriocnemis* Kaup, 1868.

Type species: *Passalustridens* Wiedemann, 1823, by subsequent designation (Hincks and Dibb 1935: 85).

Family-group name: GNAPHALOCNEMINAE Heller, 1901. Stem: *Gnaphalocnemid*-.

***Gnaptor* Brullé, 1831**: 254. Current status: valid genus in TENEBRIONIDAE: BLAPTINAE: BLAPTINI: GNAPTORINA.

Type species: *Tenebriospinimanus* Pallas, 1781, by monotypy.

Family-group name: GNAPTORINA G.S. Medvedev, 2001. Stem: *Gnaptor*-.

***Gnaptorina* Reitter, 1887**: 364. Current status: valid genus in TENEBRIONIDAE: BLAPTINAE: BLAPTINI: GNAPTORININA.

Type species: *Gnaptorinafelicitana* Reitter, 1887, by monotypy.

Family-group name: GNAPTORININA G.S. Medvedev, 2001. Stem: *Gnaptorin*-.

***Gnathidium* Gebien, 1921a**: 41. Current status: valid genus in TENEBRIONIDAE: DIAPERINAE: GNATHIDIINI: GNATHIDIINA.

Type species: *Gnathidiumcephalotes* Gebien, 1921, by monotypy.

Family-group name: GNATHIDIINAE Gebien, 1921. Stem: *Gnathidi*-.

***Gnathocera* W. Kirby, 1825**: 571. Current status: valid genus in SCARABAEIDAE: CETONIINAE: GOLIATHINI: RHOMBORHININA.

Type species: *Gnathoceravitticollis* W. Kirby, 1825 (= *Scarabaeustrivittatus* Swederus, 1787), by original designation. Note: *Gnathoceraimmaculata* W. Kirby, 1825 is usually listed as type species of this genus (e.g., J. Bezděk 2016: 394) but W. Kirby (1825: 572) unambiguously designated *Gnathoceravitticollis* as type species; both nominal species are currently included in *Gnathocera* W. Kirby, 1825.

Family-group name: GNATHOCERIDAE Schoch, 1894. Stem: *Gnathocer*-.

***Gnathosia* Fischer, 1821a**: 13. Current status: valid genus in TENEBRIONIDAE: PIMELIINAE: TENTYRIINI.

Type species: *Gnathosiaglabra* Fischer, 1821, by monotypy.

Family-group name: GNATHOSIIDES Lacordaire, 1859. Stem: *Gnathosi*-.

***Gnathotrichus* Eichhoff, 1869**: 275. Current status: valid genus in CURCULIONIDAE: SCOLYTINAE: CORTHYLINI: CORTHYLINA.

Type species: *Gnathotrichuscorthyloides* Eichhoff, 1869 (= *Tomicusmateriarius* Fitch, 1858), by monotypy.

Family-group name: GNATHOTRICHINA Balachowsky, 1949. Stem: *Gnathotrich*-.

***Gnathymenus* Solier, 1849**: 326. Current status: valid genus in STAPHYLINIDAE: PAEDERINAE: PAEDERINI: DOLICAONINA.

Type species: *Gnathymenusapterus* Solier, 1849, by monotypy.

Family-group name: GNATIMENITOS Solier, 1849. Stem: *Gnathymen*-.

***Gnatocerus* Thunberg, 1814**: 47. Current status: valid genus in TENEBRIONIDAE: DIAPERINAE: DIAPERINI: ADELININA. Note: name placed on the Official List of Generic Names in Zoology (ICZN 1975).

Type species: *Gnatocerusruber* Thunberg, 1814 (= *Trogossitacornuta* Fabricius, 1798), by monotypy (see ICZN 1975). Note: *Trogossitacornuta* Fabricius, 1798 was placed on the Official List of Specific Names in Zoology (ICZN 1975).

Family-group name: GNATHOCERINI Skopin, 1978. Stem: *Gnatocer*-.

***Gnoma* Fabricius, 1801a**: xxii. Current status: valid genus in CERAMBYCIDAE: LAMIINAE: LAMIINI.

Type species: *Cerambyxlongicollis* Fabricius, 1787, by subsequent designation (J. Thomson 1864: 82). Note: no species were originally included in this genus by Fabricius (1801a); the first ones were the four species listed by Fabricius (1801b: 315–317).

Family-group name: GNOMITAE J. Thomson, 1860. Stem: *Gnom*-.

***Gnostus* Westwood, 1855a**: 90. Current status: valid genus in PTINIDAE: PTININAE: GNOSTINI.

Type species: *Gnostusformicicola* Westwood, 1855, by monotypy.

Family-group name: GNOSTIDAE King, 1866. Stem: *Gnost*-.

†***Gobicar* Gratshev & Zherikhin, 1999**: 40. Current status: valid genus in CARIDAE: †BAISSORHYNCHINAE: †GOBICARINI. Note: Upper Jurassic (Mongolia).

Type species: †*Gobicarponomarenkoi* Gratshev & Zherikhin, 1999, by original designation.

Family-group name: †GOBICARINI Legalov, 2009. Stem: *Gobicar*-.

***Goes* J.L. LeConte, 1852a**: 150. Current status: valid genus in CERAMBYCIDAE: LAMIINAE: LAMIINI.

Type species: *Cerambyxtigrinus* DeGeer, 1775, by subsequent designation (J. Thomson 1864: 76).

Family-group name: GOES J.L. LeConte, 1873. Stem: *Goet*-.

***Goliathopsis* Janson, 1881**: 609. Current status: valid genus in SCARABAEIDAE: CETONIINAE: CREMASTOCHEILINI: GOLIATHOPSIDINA.

Type species: *Goliathopsiscervus* Janson, 1881, by monotypy. Note: *Goliathopsiscervus* is often listed as a junior synonym of *Pilinurgusdespectus* Westwood, 1874, but Xu and Qiu (2020: 95) consider Janson’s name as valid.

Family-group name: GOLIATHOPSIDINA Krikken, 1984. Stem: *Goliathopsid*-.

***Goliathus* Lamarck, 1801**: 209. Current status: valid genus in SCARABAEIDAE: CETONIINAE: GOLIATHINI: GOLIATHINA.

Type species: *Scarabaeusgoliatus* Linnaeus, 1771, by subsequent designation (Latreille 1810: 429).

Family-group name: GOLIATHIDES Latreille, 1829. Stem: *Goliath*-.

***Gompelia* Alonso-Zarazaga, 2010**: 299. Current status: valid genus in ADERIDAE. Note: new replacement name for *Olotelus* Mulsant & Rey, 1866.

Type species: *Xylophilusneglectus* Jacquelin du Val, 1863, by subsequent designation (Alonso- Zarazaga 2010: 299) (type fixation for *Olotelus* Mulsant & Rey).

Family-group name: GOMPELIINA Bouchard, 2011. Stem: *Gompeli*-.

***Gonatas* Kaup, 1871**: 50. Current status: valid genus in PASSALIDAE: PASSALINAE: MACROLININI.

Type species: *Passalusnaviculator* Percheron, 1844, by subsequent designation (Gravely 1918: 108).

Family-group name: GONATINAE Kuwert, 1891. Stem: *Gonat*-. Note: junior homonym of GONATIDAE Hoyle, 1886 (type genus *Gonatus* J.E. Gray, 1849) in Cephalopoda.

***Goniacerus* Motschulsky, 1855a**: 17. Current status: valid genus in STAPHYLINIDAE: PSELAPHINAE: GONIACERITAE: GONIACERINI.

Type species: *Goniacerusgibbus* Motschulsky, 1855, by monotypy.

Family-group name: GONIACERIDES Reitter, 1882. Stem: *Goniacer*-.

***Goniadera* Perty, 1832**: 62. Current status: valid genus in TENEBRIONIDAE: LAGRIINAE: GONIADERINI. Note: two original spellings were used, including *Gonyodera* (p. 63); subsequently, Perty (1833: 14) used *Goniadera* and therefore acted as First Reviser (ICZN 1999a, Article 24.2.4).

Type species: *Goniaderacrenata* Perty, 1832, by monotypy.

Family-group name: GONIADÉRIDES Lacordaire, 1859. Stem: *Goniader*-.

†***Gonialaena* Nabozhenko, Bukejs & Telnov, 2019**: 254. Current status: valid genus in TENEBRIONIDAE: LAGRIINAE: †GONIALAENINI. Note: Eocene (Kaliningrad, Russia).

Type species: †*Gonialaenagroehni* Nabozhenko, Bukejs & Telnov, 2019, by original designation.

Family-group name: †GONIALAENINI Nabozhenko, Bukejs & Telnov, 2019: 254. Stem: *Gonialaen*-.

***Goniastes* Westwood, 1870**: 125. Current status: valid genus in STAPHYLINIDAE: PSELAPHINAE: GONIACERITAE: GONIACERINI.

Type species: *Goniastessulcifrons* Westwood, 1870, by monotypy.

Family-group name: GONIASTINI L.W. Schaufuss, 1872. Stem: *Goniast*-.

***Goniochenia* Weise, 1896**: 10. Current status: valid genus in CHRYSOMELIDAE: CASSIDINAE: GONIOCHENIINI.

Type species: *Mesomphaliaquadraticollis* Boheman, 1850, by subsequent designation (Spaeth 1914: 29).

Family-group name: GONIOCHENIITAE Spaeth, 1942. Stem: *Goniocheni*-.

***Gonioctena* Chevrolat in Dejean, 1836a**: 403. Current status: valid genus in CHRYSOMELIDAE: CHRYSOMELINAE: CHRYSOMELINI.

Type species: *Chrysomelaviminalis* Linnaeus, 1758, by subsequent designation (C.G. Thomson 1859: 158).

Family-group name: GONIOCTÈNES Motschulsky, 1860. Stem: *Goniocten*-.

***Gonipterus* Schönherr, 1833**: 8, 456. Current status: valid genus in CURCULIONIDAE: HYPERINAE: GONIPTERINI.

Type species: *Gonipteruslepidotus* Gyllenhal, 1833, by original designation.

Family-group name: GONIPTÉRIDES Lacordaire, 1863. Stem: *Gonipter*-.

***Gonocephalum* Solier, 1834bc**: 498. Current status: valid genus in TENEBRIONIDAE: BLAPTINAE: OPATRINI: OPATRINA.

Type species: *Opatrumfuscum* Herbst, 1793 (= *Opatrumrusticum* G.-A. Olivier, 1812), by subsequent designation (Gebien 1939: 443).

Family-group name: GONOCÉPHALATES Mulsant & Revelière, 1861. Stem: *Gonocephal*-.

***Gonodera* Mulsant, 1856a**: 41. Current status: valid genus in TENEBRIONIDAE: ALLECULINAE: ALLECULINI: GONODERINA.

Type species: *Cistelafulvipes* Fabricius, 1792 (= *Cistelaluperus* Herbst, 1783), by monotypy.

Family-group name: GONODERINA Seidlitz, 1896. Stem: *Gonoder*-.

***Gonophora* Chevrolat in Dejean, 1836a**: 366. Current status: valid genus in CHRYSOMELIDAE: CASSIDINAE: GONOPHORINI.

Type species: *Hispahaemorrhoidalis* Weber, 1801, by monotypy.

Family-group name: GONOPHORITES Chapuis, 1875. Stem: *Gonophor*-.

***Gonopus* Latreille, 1828a**: 580. Current status: valid genus in TENEBRIONIDAE: BLAPTINAE: PLATYNOTINI: PLATYNOTINA.

Type species: *Blapstibialis* Fabricius, 1798, by monotypy.

Family-group name: GONOPIDES Lacordaire, 1859. Stem: *Gonopod*-.

***Gracilia* Audinet-Serville, 1834b**: 81. Current status: valid genus in CERAMBYCIDAE: CERAMBYCINAE: GRACILIINI.

Type species: *Callidiumpygmaeum* Fabricius, 1792 (= *Saperdaminuta* Fabricius, 1781), by monotypy.

Family-group name: GRACILIAIRES Mulsant, 1839. Stem: *Gracili*-.

***Grammoptera* Audinet-Serville, 1835b**: 215. Current status: valid genus in CERAMBYCIDAE: LEPTURINAE: LEPTURINI.

Type species: *Lepturapraeusta* Fabricius, 1787 (= *Lepturaustulata* Schaller, 1783), by subsequent designation (Westwood 1838c: 41).

Family-group name: GRAMMOPTERINI Della Beffa, 1915. Stem: *Grammopter*-.

***Graniger* Motschulsky, 1864**: 197. Current status: valid genus in CARABIDAE: HARPALINAE: HARPALINI: HARPALINA.

Type species: *Granigeralgirinus* Motschulsky, 1864 (= *Carterusfemoralis* Coquerel, 1858), by monotypy.

Family-group name: GRANIGERI Antoine, 1959. Stem: *Graniger*-.

***Graphipterus* Latreille, 1802b**: 83. Current status: valid genus in CARABIDAE: HARPALINAE: GRAPHIPTERINI.

Type species: *Carabusvariegatus* Fabricius, 1781 (= *Carabusserrator* Forsskål, 1775), by subsequent designation (Latreille 1810: 426).

Family-group name: GRAPHIPTERIDES Latreille, 1802. Stem: *Graphipter*-.

***Graphisurus* W. Kirby, 1837**: 169. Current status: valid genus in CERAMBYCIDAE: LAMIINAE: ACANTHOCININI.

Type species: *Cerambyxfasciatus* DeGeer, 1775, by original designation.

Family-group name: GRAPHISURINI Leng, 1920. Stem: *Graphisur*-.

***Graphoderus* Dejean, 1833a**: 54. Current status: valid genus in DYTISCIDAE: DYTISCINAE: ACILIINI. Note: name placed on the Official List of Generic Names in Zoology (ICZN 1961d).

Type species: *Dytiscuscinereus* Linnaeus, 1758, by subsequent designation (Westwood 1838c: 8; see ICZN 1961d). Note: name placed on the Official List of Specific Names in Zoology (ICZN 1961d).

Family-group name: GRAPHODERINI Houlbert, 1934. Stem: *Graphoder*-.

†***Gratshevbelus* Soriano, 2009**: 100. Current status: valid genus in BELIDAE: †MONTSECBELINAE: †GRATSHEVBELINI. Note: Lower Cretaceous (France).

Type species: †*Gratshevbeluserici* Soriano, 2009, by original designation.

Family-group name: †GRATSHEVIBELINI Legalov, 2016: 1474. Stem: *Gratshevbel*-. Note: Legalov (2016: 1474) used the incorrect subsequent spelling *Gratshevibelus* Soriano to form the stem of his new family-group name.

***Gromphas* Dejean, 1836**: 143. Current status: valid genus in SCARABAEIDAE: SCARABAEINAE: PHANAEINI: GROMPHADINA.

Type species: *Coprobiuslacordairii* Oken, 1834, by monotypy. Note: see Cupello (2024: 32) for information on the type species.

Family-group name: GROMPHINA Zunino, 1985. Stem: *Gromphad*-.

***Gronops* Schönherr, 1823**: 1142. Current status: valid genus in CURCULIONIDAE: CYCLOMINAE: HIPPORHININI.

Type species: *Curculiolunatus* Fabricius, 1775, by subsequent designation (Schönherr 1826: 13).

Family-group name: GRONOPINI Bedel, 1884. Stem: *Gronop*-.

***Gryllica* J. Thomson, 1860a**: 120. Current status: valid genus in CERAMBYCIDAE: LAMIINAE: CALLIINI.

Type species: *Onocephalapicta* Pascoe, 1858, by subsequent designation (L.S. Dillon and E.S. Dillon 1946: 156).

Family-group name: GRYLLICIDES Lacordaire, 1872. Stem: *Gryllic*-.

***Guineauletes* Legalov, 2003**: 110. Current status: valid genus in ATTELABIDAE: RHYNCHITINAE: RHYNCHITITAE: AULETINI: AULETOBIINA.

Type species: *Guineauletesmirabilis* Legalov, 2003, by original designation.

Family-group name: GUINEAULETINA Legalov, 2003. Stem: *Guineaulet*-.

***Guioperus* Perty, 1832**: 78. Current status: valid genus in CURCULIONIDAE: MOLYTINAE: GUIOPERINI.

Type species: *Guioperusgriseus* Perty, 1832, by monotypy.

Family-group name: GUIOPÉRIDES Lacordaire, 1865. Stem: *Guioper*-.

***Gyaritus* Pascoe, 1858**: 244. Current status: valid genus in CERAMBYCIDAE: LAMIINAE: GYARITINI.

Type species: *Gyaritushamatus* Pascoe, 1858, by monotypy.

Family-group name: GYARITINI Breuning, 1950. Stem: *Gyarit*-.

***Gymnetis* W.S. MacLeay, 1819**: 152. Current status: valid genus in SCARABAEIDAE: CETONIINAE: GYMNETINI: GYMNETINA. Note: name placed on the Official List of Generic Names in Zoology (ICZN 1967a).

Type species: *Scarabaeuslanius* Linnaeus, 1758, by plenary power (see ICZN 1967a). Note: name placed on the Official List of Specific Names in Zoology (ICZN 1967a).

Family-group name: GYMNETIDAE W. Kirby, 1827. Stem: *Gymnet*-.

***Gymnetron* Schönherr, 1825**: 587. Current status: valid genus in CURCULIONIDAE: CURCULIONINAE: MECININI.

Type species: *Curculiobeccabungae* Linnaeus, 1761, by subsequent designation (Schönherr 1826: 23).

Family-group name: GYMNETRINA C.G. Thomson, 1859. Stem: *Gymnetr*-. Note: junior homonym of GYMNETRINAE Swainson, 1839 (type genus *Gymnetrus* Bloch, 1795) in Pisces.

***Gymnochila* Erichson, 1844**: 454. Current status: junior synonym of *Gymnocheilis* Dejean, 1835 in TROGOSSITIDAE: TROGOSSITINAE: TROGOSSITINI.

Type species: *Trogositavestita* Griffith, 1832 (= *Trogositavaria* Fabricius, 1801), by monotypy.

Family-group name: GYMNOCHILIDES Lacordaire, 1854. Stem: *Gymnochil*-.

***Gymnognathus* Schönherr, 1826**: 37. Current status: valid genus in ANTHRIBIDAE: ANTHRIBINAE: GYMNOGNATHINI.

Type species: *Anthribusancora* Germar, 1823, by original designation.

Family-group name: GYMNOGNATHINI B.D. Valentine, 1960. Stem: *Gymnognath*-.

***Gymnoloma* Dejean, 1833b**: 167. Current status: valid genus in SCARABAEIDAE: MELOLONTHINAE: HOPLIINI: HOPLIINA.

Type species: *Melolonthaatomaria* Fabricius, 1781, by monotypy.

Family-group name: GYMNOLOMIDAE Burmeister, 1844. Stem: *Gymnolomat*-.

***Gymnopleurus* Illiger, 1803**: 199. Current status: valid genus in SCARABAEIDAE: SCARABAEINAE: GYMNOPLEURINI.

Type species: *Scarabaeusgeoffroyi* Fuesslin, 1775 (cited as “*Ateuchuspilularius* of Fabricius”), by subsequent designation (Partington 1836a: 693). Note: we follow Branco (2007: 12) in using Article 69.2.4 (ICZN 1999a) to determine the identity of the type species for this genus, although our data associated with the fixation of the type species (i.e., Partington 1836a: 693) predates the designation given by Reiche (1841: 212), which is typically reported in the literature.

Family-group name: GYMNOPLEURIDAE Streubel, 1846. Stem: *Gymnopleur*-.

***Gymnusa* Gravenhorst, 1806**: 173. Current status: valid genus in STAPHYLINIDAE: ALEOCHARINAE: GYMNUSINI.

Type species: *Gymnusasinuata* Gravenhorst, 1806 (= *Staphylinusbrevicollis* Paykull, 1800), by monotypy.

Family-group name: GYMNUSIDA Heer, 1839. Stem: *Gymnus*-.

***Gynaecomeloe* Wellman, 1910b**: 217. Current status: junior synonym of *Cordylospasta* G.H. Horn, 1876 in MELOIDAE: MELOINAE: EUPOMPHINI.

Type species: *Megetraopaca* G.H. Horn, 1868, by original designation.

Family-group name: GYNAECOMELODIDES Wellman, 1910. Stem: *Gynaecomelo*-.

***Gyrinus* Geoffroy, 1762**: 193. Current status: valid genus in GYRINIDAE: GYRININAE: GYRININI. Note: name placed on the Official List of Generic Names in Zoology (ICZN 1994a).

Type species: *Dytiscusnatator* Linnaeus, 1758, by subsequent designation (Latreille 1810: 426; see ICZN 1994a). Note: name placed on the Official List of Specific Names in Zoology (ICZN 1994a).

Family-group name: GYRINITES Latreille, 1810. Stem: *Gyrin*-.

***Gyrohypnus* Leach in Samouelle, 1819**: 172. Current status: valid genus in STAPHYLINIDAE: STAPHYLININAE: XANTHOLININI. Note: name placed on the Official List of Generic Names in Zoology (ICZN 1983c, as *Gyrohypnus* Samouelle, 1819).

Type species: *Staphylinusfracticornis* O.F. Müller, 1776, by plenary power (see ICZN 1983c). Note: name placed on the Official List of Specific Names in Zoology (ICZN 1983c).

Family-group name: GYROHYPNIDAE W. Kirby, 1837. Stem: *Gyrohypn*-. Note: placed on the Official List of Family-Group Names in Zoology (ICZN 1996c, as GYROHYPNINI Kirby, 1837); younger name XANTHOLININI Erichson, 1839 given precedence over this name (ICZN 1996c).

***Gyrophaena* Mannerheim, 1830**: 74. Current status: valid genus in STAPHYLINIDAE: ALEOCHARINAE: HOMALOTINI: GYROPHAENINA.

Type species: *Staphylinusnanus* Paykull, 1800, by subsequent designation (Westwood 1838c: 20).

Family-group name: GYROPHAENINI Kraatz, 1856. Stem: *Gyrophaen*-.

***Habrobates* Semenov, 1903b**: 11. Current status: valid genus in TENEBRIONIDAE: PIMELIINAE: PIMELIINI: HABROBATINA.

Type species: *Habrobatesvernalis* Semenov, 1903, by monotypy.

Family-group name: HABROBATINA Nabozhenko & Chigray, 2022 in Chigray et al. 2022: 5. Stem: *Habrobat*-.

***Habrocerus* Erichson, 1839a**: 400. Current status: valid genus in STAPHYLINIDAE: HABROCERINAE.

Type species: *Tachyporuscapillaricornis* Gravenhorst, 1806, by monotypy.

Family-group name: HABROCÉRIENS Mulsant & Rey, 1876. Stem: *Habrocer*-.

***Habrophora* Erichson, 1847c**: 163. Current status: valid genus in CHRYSOMELIDAE: EUMOLPINAE: HABROPHORINI.

Type species: *Habrophoralateralis* Erichson, 1847, by subsequent designation (Baly 1863: 156).

Family-group name: HABROPHORINI Bechyné & Špringlová de Bechyné, 1969. Stem: *Habrophor*-.

***Hadesia* J. Müller, 1911**: 175. Current status: valid genus in LEIODIDAE: CHOLEVINAE: LEPTODIRINI: ANTHROHERPONINA.

Type species: *Hadesiavasiceki* J. Müller, 1911, by monotypy.

Family-group name: HADESIINI Absolon, 1913. Stem: *Hadesi*-.

***Hadrognathus* Schaum, 1852**: 31. Current status: valid genus in STAPHYLINIDAE: OMALIINAE: HADROGNATHINI. Note: new replacement name for *Eugnathus* Mulsant & Rey, 1851.

Type species: *Eugnathuslongipalpis* Mulsant & Rey, 1851, by monotypy (type fixation for *Eugnathus* Mulsant & Rey).

Family-group name: HADROGNATHINI Portevin, 1929. Stem: *Hadrognath*-.

***Hadromerus* Schönherr, 1834**: 127. Current status: invalid senior synonym of *Hadromeropsis* Pierce, 1913 in CURCULIONIDAE: ENTIMINAE: POLYDRUSITAE: TANYMECINI: TANYMECINA. Note: junior homonym of *Hadromerus* Schönherr, 1823 [Coleoptera: CURCULIONIDAE].

Type species: *Hadromerusnobilitatus* Gyllenhal, 1834, by monotypy.

Family-group name: HADROMERIDES Jekel, 1865. Stem: *Hadromer*-. Note: family-group name permanently invalid (ICZN 1999a, Article 39).

***Haemonia* Dejean, 1821**: 114. Current status: junior synonym of *Macroplea* Samouelle, 1819 in CHRYSOMELIDAE: DONACIINAE: HAEMONIINI.

Type species: *Donaciaequiseti* Fabricius, 1798 (= *Donaciaappendiculata* Panzer, 1794), by monotypy. Note: the type species often reported for *Haemonia* Dejean is *Donaciazosterae* Fabricius, 1801 (= *Rhagiummuticum* Fabricius, 1792) (e.g., Silfverberg 2010: 357) but *zosterae* is listed as a junior synonym of *equiseti*, the sole valid species listed by Dejean (1821: 114) in the genus *Haemonia*; Article 68.3 (ICZN 1999a) denotes that when an author establishes a new nominal genus-group taxon for a single taxonomic species, that species is the type species by monotypy regardless of any cited synonyms.

Family-group name: HAEMONIINI S.H. Chen, 1941. Stem: *Haemoni*-.

***Haeterius* Dejean, 1833b**: 128. Current status: valid genus in HISTERIDAE: HAETERIINAE: HAETERIINI.

Type species: *Histerquadratus* Kugelann, 1794 (= *Histerferrugineus* G.-A. Olivier, 1789), by monotypy.

Family-group name: HÉTÉRIENS Marseul, 1857. Stem: *Haeteri*-.

***Haliplus* Latreille, 1802b**: 77. Current status: valid genus in HALIPLIDAE.

Type species: *Dytiscusimpressus* Fabricius, 1787 (= *Dytiscusruficollis* DeGeer, 1774), by subsequent designation (Latreille 1810: 426).

Family-group name: HALIPLIDES Aubé, 1836. Stem: *Halipl*-.

***Hallomenus* Panzer, 1793**: no 17. Current status: valid genus in TETRATOMIDAE: HALLOMENINAE.

Type species: *Hallomenushumeralis* Panzer, 1793 (= *Chrysomelabinotata* Quensel, 1790), by subsequent designation (Latreille 1810: 429).

Family-group name: HALLOMENIDAE Gistel, 1848. Stem: *Hallomen*-.

***Halorabyxis* Jeannel, 1954a**: 338. Current status: junior synonym of *Physoplectus* Reitter, 1882 in STAPHYLINIDAE: PSELAPHINAE: GONIACERITAE: BRACHYGLUTINI: BRACHYGLUTINA.

Type species: *Halorabyxisvinsoni* Jeannel, 1954, by original designation.

Family-group name: HALORABYXINA Leleup, 1969. Stem: *Halorabyx*-.

***Halyzia* Mulsant, 1846**: 148. Current status: valid genus in COCCINELLIDAE: COCCINELLINAE: COCCINELLINI.

Type species: *Coccinellasedecimguttata* Linnaeus, 1758 [as *sexdecimguttata*], by monotypy.

Family-group name: HALYZIAIRES Mulsant, 1846. Stem: *Halyzi*-.

***Hameedia* M. Abdullah & Qadri, 1968**: 311. Current status: valid genus in STAPHYLINOIDEA incertae sedis.

Type species: *Hameediabatoolae* M. Abdullah & Qadri, 1968, by original designation.

Family-group name: HAMEEDINI M. Abdullah & Qadri, 1968. Stem: *Hameedi*-.

***Hamotus* Aubé, 1844**: 91. Current status: valid genus in STAPHYLINIDAE: PSELAPHINAE: PSELAPHITAE: TYRINI: SOMATIPIONINA.

Type species: *Hamotuslatericius* Aubé, 1844, by subsequent designation (R. Lucas 1920: 314).

Family-group name: HAMOTINI Park, 1951. Stem: *Hamot*-.

***Hapalips* Reitter, 1877**: 122. Current status: valid genus in EROTYLIDAE: LANGURIINAE: HAPALIPINI.

Type species: *Hapalipsmexicanus* Reitter, 1877, by subsequent designation (Sen Gupta 1968: 6).

Family-group name: HAPALIPINI Leschen, 2003. Stem: *Hapalip*-.

***Hapatesus* Candèze, 1863**: 76, 188. Current status: valid genus in ELATERIDAE: HAPATESINAE.

Type species: *Hapatesushirtus* Candèze, 1863, by monotypy.

Family-group name: HAPATESINAE Kusy, Motyka & Bocák, 2021: 8. Stem: *Hapates*-.

†***Haplochelus* Kirejtshuk & Poinar, 2006**: 157. Current status: junior synonym of *Lepicerus* Motschulsky, 1855 in LEPICERIDAE. Note: mid-Cretaceous (Myanmar).

Type species: †*Haplochelusgeorissoides* Kirejtshuk & Poinar, 2006, by original designation.

Family-group name: †HAPLOCHELIDAE Kirejtshuk & Poinar, 2006. Stem: *Haplochel*-.

***Haplonyx* Schönherr, 1836**: 606. Current status: valid genus in CURCULIONIDAE: CURCULIONINAE: CRYPTOPLINI.

Type species: *Haplonyxspencei* Gyllenhal, 1836, by original designation.

Family-group name: HAPLONYCIDES Lacordaire, 1865. Stem: *Haplonych*-.

***Haplostethus* J.L. LeConte, 1860c**: 253. Current status: junior synonym of *Mastogenius* Solier, 1849 in BUPRESTIDAE: POLYCESTINAE: HAPLOSTETHINI.

Type species: *Haplostethussubcyaneus* J.L. LeConte, 1860, by monotypy.

Family-group name: HAPLOSTETHINI J.L. LeConte, 1861. Stem: *Haplosteth*-.

***Haplotrinchus* Kerremans, 1903b**: 126. Current status: valid genus in BUPRESTIDAE: CHRYSOCHROINAE: DICERCINI: HAPLOTRINCHINA.

Type species: *Buprestisviridula* G.-A. Olivier, 1790, by subsequent designation (Bellamy 1997b: 76).

Family-group name: HAPLOTRINCHINA Holyński, 1993. Stem: *Haplotrinch*-.

***Hapsodrilus* C. Costa, 1975**: 90. Current status: valid genus in ELATERIDAE: AGRYPNINAE: PYROPHORINI: HAPSODRILINA.

Type species: *Pyrophorusignifer* Germar, 1841, by original designation.

Family-group name: HAPSODRILINA C. Costa, 1975. Stem: *Hapsodril*-.

†***Hapsomela* Poinar & A.E. Brown, 2004**: 790. Current status: valid genus in STAPHYLINIDAE: SCYDMAENINAE: †HAPSOMELITAE. Note: Cretaceous (Burmese amber).

Type species: †*Hapsomelaburmitis* Poinar & A.E. Brown, 2004, by original designation.

Family-group name: †HAPSOMELINAE Poinar & A.E. Brown, 2004. Stem: *Hapsomel*-.

***Haraia* García & Jiménez-Ramos, 2020**: 461. Current status: valid genus in HETEROCERIDAE: HETEROCERINAE: HARAIAINI.

Type species: *Haraiacerromachensis* García & Jiménez-Ramos, 2020, by original designation.

Family-group name: Tribe HARAIAINI García & Jiménez-Ramos, 2020: 460. Stem: *Haraia*- (ICZN 1999a, Article 29.4).

***Harpaglossus* Motschulsky, 1858b**: 173. Current status: valid genus in CARABIDAE: HARPALINAE: CHLAENIINI: CHLAENIINA. Note: new replacement name for *Ceroglossus* Chaudoir, 1856.

Type species: *Feronialaevigata* Dejean, 1828, by subsequent designation (Andrewes 1938: 135).

Family-group name: HARPAGLOSSINI Basilewsky, 1950. Stem: *Harpagloss*-.

***Harpalus* Latreille, 1802b**: 92. Current status: valid genus in CARABIDAE: HARPALINAE: HARPALINI: HARPALINA.

Type species [suggested]: *Carabusproteus* Paykull, 1790 (= *Carabusaffinis* Schrank, 1781), by subsequent designation (Andrewes 1935: 19). Note: the valid type species fixation is that of Latreille (1810: 426), who selected *Carabusruficornis* Fabricius, 1775 (= *Carabusrufipes* DeGeer, 1774), but that species is currently included in the subgenus PseudoophonusMotschulsky, 1844, not the nominotypicalsubgenusofHarpalus Latreille; an application to the Commission is necessary to fix *Carabusproteus* Paykull, 1790 as the type species of *Harpalus* Latreille, 1802; a first request was postponed (see ICZN 1950); Andrewes (1935: 19) designated *Carabusaffinis* Schrank, 1781 as type species of *Harpalus*, a species not originally included, but since he listed it at the same time as a synonym of *Carabusproteus* Paykull, 1790, a species originally included, he is deemed to have designated the latter species as type species (ICZN, 1999a, Article 69.2.2).

Family-group name: HARPALII Bonelli, 1810. Stem: *Harpal*-.

***Haruspex* J. Thomson, 1864**: 221. Current status: valid genus in CERAMBYCIDAE: CERAMBYCINAE: PIEZOCERINI: HARUSPICINA.

Type species: *Ozodesbrevipes* A. White, 1855, by original designation.

Family-group name: HARUSPICINA Martins, 1976. Stem: *Haruspic*-.

***Harvengia* Ferrer, 2004**: 367. Current status: valid genus in TENEBRIONIDAE: PIMELIINAE: STENOSINI: HARVENGIINA.

Type species: *Harvengiavietnamita* Ferrer, 2004, by original designation.

Family-group name: HARVENGINA Ferrer, 2004. Stem: *Harvengi*-.

***Hebesecis* Pascoe, 1865a**: 353. Current status: junior synonym of *Hebecerus* Dejean, 1835 in CERAMBYCIDAE: LAMIINAE: ANCITINI. Note: unnecessary new replacement name for *Hebecerus* Dejean, 1835.

Type species: *Acanthocinusmarginicollis* Boisduval, 1835, by subsequent designation (Bousquet and Bouchard 2013a: 83) (type fixation for *Hebecerus* Dejean).

Family-group name: HEBESECINAE Pascoe, 1871. Stem: *Hebesecid*-.

***Hebestola* Blanchard, 1851**: 513. Current status: invalid senior synonym of *Neohebestola* Marinoni, 1977 in CERAMBYCIDAE: LAMIINAE: FORSTERIINI. Note: junior homonym of *Hebestola* Haldeman, 1847 [Coleoptera: CERAMBYCIDAE].

Type species: *Hebestolaparvula* Blanchard, 1851, by subsequent designation (J. Thomson 1864: 108).

Family-group name: HEBESTOLITAE J. Thomson, 1864. Stem: *Hebestol*-. Note: family-group name permanently invalid (ICZN 1999a, Article 39).

***Hecyrida* J. Thomson, 1860a**: 39. Current status: junior synonym of *Hecyra* J. Thomson, 1857 in CERAMBYCIDAE: LAMIINAE: CEROPLESINI: CROSSOTINA. Note: unnecessary new replacement name for *Hecyra* J. Thomson, 1857.

Type species: *Hecyraimproba* J. Thomson, 1857, by monotypy (type fixation for *Hecyra* J. Thomson).

Family-group name: HÉCYRIDIDES Lacordaire, 1872. Stem: *Hecyrid*-.

***Hedobia* Dejean, 1821**: 41. Current status: valid genus in PTINIDAE: EUCRADINAE: HEDOBIINI.

Type species: *Ptinuspubescens* G.-A. Olivier, 1790, by monotypy.

Family-group name: HÉDOBIAIRES Mulsant & Rey, 1868. Stem: *Hedobi*-.

***Hedyphanes* Fischer, 1820**: pl. 15. Current status: valid genus in TENEBRIONIDAE: TENEBRIONINAE: HELOPINI: HELOPINA.

Type species: *Hedyphanescoerulescens* Fischer, 1820, by monotypy.

Family-group name: HEDYPHANINA Reitter, 1922. Stem: *Hedyphan*-.

***Hegemona* Laporte, 1840b**: 230. Current status: valid genus in TENEBRIONIDAE: STENOCHIINAE: CNODALONINI.

Type species: *Hegemonaresplendens* Laporte, 1840, by monotypy.

Family-group name: HEGEMONINI Reitter, 1922. Stem: *Hegemon*-.

***Heilipus* Germar, 1823**: 399. Current status: valid genus in CURCULIONIDAE: MOLYTINAE: HYLOBIINI: HYLOBIINA.

Type species: *Heilipuslactarius* Germar, 1823, by subsequent designation (Schönherr 1825: 582).

Family-group name: HEILIPINAE Faust, 1892. Stem: *Heilipod*-.

***Helea* Latreille, 1804b**: 326. Current status: valid genus in TENEBRIONIDAE: TENEBRIONINAE: HELEINI: HELEINA. Note: the older name *Helea* Meigen, 1800 [Diptera] was published in a work that has been suppressed for the purposes of zoological nomenclature (see ICZN 1963b); the name *Helea* Osten Sacken, 1882 is available in Diptera (see Evenhuis and Pape 2017: 37).

Type species: *Heleaperforatus* Latreille, 1817, by subsequent monotypy (Latreille 1817b: 261).

Family-group name: HELEADAE Fleming, 1821. Stem: *Hele*-.

***Helenaea* Schatzmayr & Koch, 1934**: 23, 24. Current status: valid genus in CARABIDAE: GEHRINGIINAE: GEHRINGIINI: HELENAEINA.

Type species: *Helenaeatorretassoi* Schatzmayr & C. Koch, 1934, by monotypy.

Family-group name: HELENAEINA Deuve, 2007. Stem: *Helenae*-.

***Helictopleurus* H. d’Orbigny, 1915**: 402. Current status: valid genus in SCARABAEIDAE: SCARABAEINAE: ONITICELLINI: HELICTOPLEURINA.

Type species: *Scarabaeusmarsyas* G.-A. Olivier, 1789, by subsequent designation (Paulian 1937: 132).

Family-group name: HELICTOPLEURIDES A. Janssens, 1946. Stem: *Helictopleur*-.

***Heligmus* Candèze, 1865**: 52. Current status: invalid senior synonym of *Cleidecosta* P.J. Johnson, 2002 in ELATERIDAE: AGRYPNINAE: EUPLINTHINI: CLEIDECOSTINA. Note: junior homonym of *Heligmus* Dujardin, 1844 [Nematoda].

Type species: *Heligmusglyphoderus* Candèze, 1865, by monotypy.

Family-group name: HELIGMINI C. Costa, 1975. Stem: *Heligm*-. Note: family-group name permanently invalid (ICZN 1999a, Article 39).

***Heliolus* Fauvel, 1907**: 70. Current status: valid genus in CERAMBYCIDAE: LAMIINAE: HELIOLINI. Note: new replacement name for *Helius* Fauvel, 1906.

Type species: *Heliusbrevicornis* Fauvel, 1906, by monotypy (type fixation for *Helius* Fauvel).

Family-group name: HELIOLINI Breuning, 1951. Stem: *Heliol*-.

***Heliomene* Gistel, 1848b**: xi, 134. Current status: junior synonym of *Chrysolopus* Germar, 1817 in CURCULIONIDAE: CYCLOMINAE: ATERPINI: ATERPINA. Note: unnecessary new replacement name for *Chrysolopus* Germar, 1817.

Type species: *Curculiospectabilis* Fabricius, 1775, by subsequent designation (Schönherr 1823: 1142) (type fixation for *Chrysolopus* Germar).

Family-group name: HELIOMENEIDAE Gistel, 1848. Stem: *Heliomen*-. Note: nomen oblitum (see Bouchard et al. 2011: 601).

***Heliopates* Dejean, 1834**: 191. Current status: valid genus in TENEBRIONIDAE: BLAPTINAE: DENDARINI: DENDARINA.

Type species [suggested]: *Tenebriolusitanicus* Herbst, 1797, by subsequent designation (Gebien 1938b: 304). Note: as noted by Bousquet and Bouchard (2013a: 46), *Heliopates* is a new replacement name for *Heliophilus* Dejean, 1821 and as such should have the same type species, *Pedinushybridus* Latreille, 1804 (= *Tenebrioabbreviatus* G.-A. Olivier, 1795) by subsequent designation of Duponchel (1845: 517), which is currently included in the genus *Phylan* Dejean, 1821; however, currently *Heliopates* Dejean is recognized as a valid genus and subgenus (Iwan and Löbl 2020: 302, Bouchard et al. 2021: 202); an application to the Commission is needed to resolve this nomenclatural issue.

Family-group name: HÉLIOPATHAIRES Mulsant & Rey, 1854. Stem: *Heliopat*-.

***Helluo* Bonelli, 1813**: 450, 453. Current status: valid genus in CARABIDAE: HARPALINAE: HELLUONINI: HELLUONINA.

Type species: *Helluocostatus* Bonelli, 1813, by original designation.

Family-group name: HELLUONIDAE Hope, 1838. Stem: *Helluon*-.

***Helluodes* Westwood, 1846**: 354. Current status: valid genus in CARABIDAE: HARPALINAE: PHYSOCROTAPHINI.

Type species: *Helluodestaprobanae* Westwood, 1846, by monotypy.

Family-group name: HELLUODINI Csiki, 1932. Stem: *Helluod*-.

***Helluomorpha* Laporte, 1834b**: 52. Current status: valid genus in CARABIDAE: HARPALINAE: HELLUONINI: OMPHRINA.

Type species: *Helluoheros* Gory, 1833, by subsequent designation (Hope 1838a: 110).

Family-group name: HELLUOMORPHINA Reichardt, 1974. Stem: *Helluomorph*-.

***Helochares* Mulsant, 1844**: [197]. Current status: valid genus in HYDROPHILIDAE: ACIDOCERINAE. Note: name placed on the Official List of Generic Names in Zoology (ICZN 1964).

Type species: *Dytiscuslividus* Forster, 1771, by subsequent designation (C.G. Thomson 1859: 18; see ICZN 1964). Note: name placed on the Official List of Specific Names in Zoology (ICZN 1964).

Family-group name: HELOCHARAE Orchymont, 1919. Stem: *Helochar*-.

***Helopeltis* G.H. Horn, 1873a**: 118, 137. Current status: invalid senior synonym of *Helobata* Bergroth, 1888 in HYDROPHILIDAE: ACIDOCERINAE. Note: junior homonym of *Helopeltis* Signoret, 1858 [Hemiptera].

Type species: *Helopeltislarvalis* G.H. Horn, 1873, by monotypy.

Family-group name: HELOPELTINI G.H. Horn, 1873: 118. Stem: *Helopelt*-. Note: family-group name permanently invalid (ICZN 1999a, Article 39).

***Helophorus* Fabricius, 1775**: 66. Current status: valid genus in HELOPHORIDAE. Note: the original spelling *Elophorus* was emended to *Helophorus* by the Commission and *Helophorus* Fabricius, 1775 was placed on the Official List of Generic Names in Zoology (ICZN 1993c).

Type species: *Silphaaquatica* Linnaeus, 1758, by subsequent designation (Latreille 1810: 428; see ICZN 1993c). Note: name placed on the Official List of Specific Names in Zoology (ICZN 1993c).

Family-group name: HELOPHERIDA Leach, 1815. Stem: *Helophor*-.

***Helopinus* Solier, 1848b**: 152, 197. Current status: valid genus in TENEBRIONIDAE: BLAPTINAE: PEDININI: HELOPININA.

Type species: *Helopinuscostatus* Solier, 1848, by monotypy.

Family-group name: HÉLOPINIDES Lacordaire, 1859. Stem: *Helopin*-.

***Helops* Fabricius, 1775**: 257. Current status: valid genus in TENEBRIONIDAE: TENEBRIONINAE: HELOPINI: HELOPINA. Note: name placed on the Official List of Generic Names in Zoology ICZN 2009c).

Type species: *Tenebriocaeruleus* Linnaeus, 1758, by plenary power (see ICZN 2009c). Note: as mentioned in Bousquet et al. (2018: 183) and Vela et al. (2020: 63) *Tenebriocaeruleus* Linnaeus, 1758, the type species recognized by the Commission for the genus *Helops* (ICZN 2009c), is in fact a leaf beetle species which belongs to the genus *Timarcha* Samouelle, 1819 [Coleoptera: CHRYSOMELIDAE]; obviously, Fabricius (1775: 257) misidentified Linnaeus’s *Tenebriocaeruleus*; a request to the Commission is necessary to change the type species of *Helops* to *Tenebriocaeruleus* Linnaeus, 1758 (sensu Fabricius, 1775) (= *Helopschalibaeus* Rossi, 1790).

Family-group name: HELOPII Latreille, 1802. Stem: *Helop*-.

***Helota* W.S. MacLeay, 1825**: 42. Current status: valid genus in HELOTIDAE.

Type species: *Helotavigorsii* W.S. MacLeay, 1825, by monotypy.

Family-group name: HÉLOTIDES Chapuis, 1876. Stem: *Helot*-. Note: junior homonym of HELOTIDAE Adams et al., 1854 (type genus *Helotes* Cuvier, 1829) in Pisces.

***Hemiconderis* Kleine, 1926**: 162. Current status: junior synonym of *Falsolucidota* Pic, 1921 in LYCIDAE: METRIORRHYNCHINAE: METRIORRHYNCHINI: METRIORRHYNCHINA.

Type species: *Hemiconderisexplicatus* Kleine, 1926, by monotypy.

Family-group name: HEMICONDERININA Bocák & Bocáková, 1990. Stem: *Hemiconderin*-.

***Hemicrepidius* Germar, 1839**: 206, 212. Current status: valid genus in ELATERIDAE: DENDROMETRINAE: DENDROMETRINI: HEMICREPIDIINA.

Type species: *Hemicrepidiusthomasi* Germar, 1839 (= *Elatermemnonius* Herbst, 1806), by monotypy. Note: the original spelling of the valid name of the type species was *nemnonius*; however, the incorrect subsequent spelling *memnonius* is in prevailing usage, attributed to the publication of the original spelling, and therefore deemed to be the correct original spelling (ICZN 1999a, Article 33.3.1).

Family-group name: HEMICREPIDIINI Champion, 1896. Stem: *Hemicrepidi*-.

***Hemilophus* Audinet-Serville, 1835a**: 49. Current status: valid genus in CERAMBYCIDAE: LAMIINAE: HEMILOPHINI.

Type species: *Hemilophusdimidiaticornis* Audinet-Serville, 1835, by monotypy.

Family-group name: HEMILOPHITAE J. Thomson, 1868. Stem: *Hemiloph*-. Note: nomen protectum (see Bousquet et al. 2009: 31).

***Hemiops* Eschscholtz in Laporte, 1838**: table. Current status: valid genus in ELATERIDAE: HEMIOPINAE: HEMIOPINI.

Type species: *Hemiopsflava* Laporte, 1838, by subsequent designation (Hyslop 1921: 648).

Family-group name: HEMIOPINAE Fleutiaux, 1941. Stem: *Hemiop*-.

***Hemipeplus* Latreille, 1829b**: 53. Current status: valid genus in MYCTERIDAE: HEMIPEPLINAE. Note: this name is sometimes credited to Berthold (1827: 394) (e.g., Löbl et al. 2020: 563) but the genus is not made available by a description or an indication in Berthold’s work.

Type species: *Hemipeplushemipterus* Lacordaire, 1854, by subsequent monotypy (Lacordaire 1854: 454).

Family-group name: HÉMIPÉPLIDES Lacordaire, 1854. Stem: *Hemipepl*-.

***Hemipharis* Burmeister, 1842**: 531. Current status: valid genus in SCARABAEIDAE: CETONIINAE: SCHIZORHININI: SCHIZORHININA.

Type species: *Schyzorhinainsularis* Gory & Percheron, 1833, by subsequent designation (Kraatz 1880: 183).

Family-group name: HEMIPHARIDAE Kraatz, 1880. Stem: *Hemiphar*-.

***Hemirhipidius* Heller, 1920**: 168. Current status: valid genus in RIPIPHORIDAE: HEMIRHIPIDIINAE.

Type species: *Hemirhipidiusnigroapicalis* Heller, 1920, by original designation.

Family-group name: HEMIRHIPIDIINI Heller, 1920. Stem: *Hemirhipidi*-.

***Hemirhipus* Berthold, 1827**: 336. Current status: valid genus in ELATERIDAE: AGRYPNINAE: HEMIRHIPINI: HEMIRHIPINA.

Type species: *Elaterlineatus* G.-A. Olivier, 1790, by monotypy.

Family-group name: HÉMIRHIPIDES Candèze, 1857. Stem: *Hemirhip*-.

***Hemisphaerota* Chevrolat in Dejean, 1836a**: 367. Current status: valid genus in CHRYSOMELIDAE: CASSIDINAE: HEMISPHAEROTINI.

Type species: *Cassidaerythrocera* Germar, 1823, by monotypy. Note: this species is generally listed as a junior synonym of *Imatidiumcyaneum* Say, 1824, but Germar’s name is older and should be used as the valid name for the species; an application to the Commission is necessary to conserve usage of *Hemisphaerotacyanea* (Say, 1824) as valid.

Family-group name: HEMISPHAEROTINI Monrós & Viana, 1951. Stem: *Hemisphaerot*-.

***Hemydacne* Jacoby, 1897**: 244. Current status: valid genus in CHRYSOMELIDAE: EUMOLPINAE: HEMYDACNINI.

Type species: *Hemydacnemaculicollis* Jacoby, 1897, by monotypy.

Family-group name: HEMYDACNINI Bechyné, 1951. Stem: *Hemydacn*-.

***Henicolabus* Voss, 1925**: 224. Current status: valid genus in ATTELABIDAE: ATTELABINAE: ATTELABINI: HENICOLABINA.

Type species: *Attelabusgiganteus* Faust, 1882, by original designation.

Family-group name: HENICOLABINA Legalov, 2007. Stem: *Henicolab*-.

***Hephebocerus* Schönherr, 1840a**: 501. Current status: valid genus in BRENTIDAE: BRENTINAE: TRACHELIZINI: HEPHEBOCERINA.

Type species: *Brenthusnanus* Boheman, 1833, by original designation.

Family-group name: HÉPHÉBOCÉRIDES Lacordaire, 1865. Stem: *Hephebocer*-.

***Heptaphylla* Friedenreich, 1883**: 375. Current status: junior synonym of *Rhipidandrus* J.L. LeConte, 1862 in TENEBRIONIDAE: TENEBRIONINAE: BOLITOPHAGINI.

Type species: *Heptaphyllafungicola* Friedenreich, 1883, by monotypy.

Family-group name: HEPTAPHYLLINI Prudhomme de Borre, 1886. Stem: *Heptaphyll*-.

***Heptophylla* Motschulsky, 1857a**: 32. Current status: valid genus in SCARABAEIDAE: MELOLONTHINAE: HEPTOPHYLLINI.

Type species: *Heptophyllapicea* Motschulsky, 1857, by monotypy.

Family-group name: HEPTOPHYLLINI S.I. Medvedev, 1951. Stem: *Heptophyll*-.

***Hermaeophaga* Foudras, 1860**: 35, 299. Current status: valid genus in CHRYSOMELIDAE: GALERUCINAE: ALTICINI.

Type species: *Halticacicatrix* Illiger, 1807, by subsequent designation (Maulik 1926: 411).

Family-group name: HERMAEOPHAGINA Bechyné, 1997. Stem: *Hermaeophag*-.

***Hesperophanes* Dejean, 1835**: 328. Current status: valid genus in CERAMBYCIDAE: CERAMBYCINAE: HESPEROPHANINI: HESPEROPHANINA.

Type species [suggested]: *Callidiumsericeum* Fabricius, 1787, by subsequent designation (J. Thomson 1864: 253). Note: this species is a species inquirenda in Dejean (1835: 328) and therefore is not an originally included species (ICZN 1999a, Article 67.2.5) but all originally included species belong to other cerambycid genera (see Bousquet and Bouchard 2013a: 83); we believe that a request should be submitted to the Commission to fix *Callidiumsericeum* Fabricius, 1787 as the type species of *Hesperophanes* Dejean, 1835.

Family-group name: HESPÉROPHANAIRES Mulsant, 1839. Stem: *Hesperophan*-.

***Hesthesis* Newman, 1840a**: 17. Current status: valid genus in CERAMBYCIDAE: CERAMBYCINAE: HESTHESINI.

Type species: *Lepturavariegata* Fabricius, 1775, by subsequent designation (J. Thomson 1864: 162).

Family-group name: HESTHESINAE Pascoe, 1867. Stem: *Hesthes*-.

***Hetairotermes* Cameron, 1920**: 223. Current status: valid genus in STAPHYLINIDAE: ALEOCHARINAE: TERMITOHOSPITINI: HETAIROTERMITINA. Note: new replacement name for *Termophila* Lea, 1910.

Type species: *Termophilalatebricola* Cameron, 1920, by subsequent designation (Fenyes 1919: 25) (type fixation for *Termophila* Lea).

Family-group name: HETAIROTERMITINA Seevers, 1957. Stem: *Hetairotermit*-.

***Heteraspis* Chevrolat in Dejean, 1836a**: 413. Current status: valid genus in CHRYSOMELIDAE: EUMOLPINAE: BROMIINI.

Type species: *Eumolpusvittatus* G.-A. Olivier, 1808, by monotypy.

Family-group name: HETERASPINAE Baly, 1863. Stem: *Heteraspid*-.

***Heterocerus* Fabricius, 1792a**: 262. Current status: valid genus in HETEROCERIDAE: HETEROCERINAE: HETEROCERINI.

Type species: *Apatemarginatus* Fabricius, 1787, by subsequent designation (Latreille 1810: 428).

Family-group name: HETEROCERIDAE W.S. MacLeay, 1825. Stem: *Heterocer*-.

***Heterocheira* Dejean, 1836b**: 220. Current status: valid genus in TENEBRIONIDAE: BLAPTINAE: OPATRINI: HETEROTARSINA.

Type species: *Ulomaaustrale* Boisduval, 1835, by monotypy.

Family-group name: HETEROCHEIRINI C. Koch, 1956. Stem: *Heterocheir*-.

***Heterochelus* Burmeister, 1844**: 87. Current status: valid genus in SCARABAEIDAE: MELOLONTHINAE: HOPLIINI: HOPLIINA.

Type species: to be determined. Note: Burmeister (1844) placed the 54 species of the genus *Heterochelus* in several informal groups and for one of the groups he designated *Melolonthadentipes* Fabricius, 1781 as the type on page 108; however, we do not consider that this represents a valid type species fixation for *Heterochelus* (also, *Melolonthadentipes* Fabricius, 1781 is the type species of *Dichelus* Lepeletier & Audinet-Serville, 1828); as far as we know, no type species has been proposed for *Heterochelus* and we refrain from suggesting one since the taxonomy of the African Hopliini is in need of a thorough revision (H. Dombrow, pers. comm. to P.B., 2021).

Family-group name: HETEROCHELIDAE Burmeister, 1844. Stem: *Heterochel*-.

***Heterogenius* Moser, 1911**: 143. Current status: valid genus in SCARABAEIDAE: CETONIINAE: CREMASTOCHEILINI: HETEROGENIINA.

Type species: *Heterogeniusalbomaculatus* Moser, 1911, by monotypy.

Family-group name: HETEROGENIINA Krikken, 1984. Stem: *Heterogeni*-.

***Heterogyrus* Legros, 1953**: 63. Current status: valid genus in GYRINIDAE: HETEROGYRINAE.

Type species: *Heterogyrusmilloti* Legros, 1953, by original designation.

Family-group name: HETEROGYRINI Brinck, 1956. Stem: *Heterogyr*-.

***Heteromastix* Boheman, 1858**: 81. Current status: valid genus in CANTHARIDAE: HETEROMASTIGINAE.

Type species: *Heteromastixbicolor* Boheman, 1858, by monotypy.

Family-group name: HETEROMASTIGINAE Motyka, Biffi & Bocák in Motyka et al., 2023b: 559. Stem: *Heteromastig*-.

***Heteronyx* Guérin-Méneville, 1831a**: pl. 3. Current status: valid genus in SCARABAEIDAE: SERICOIDINAE: HETERONYCHINI.

Type species: *Heteronyxaustralis* Guérin-Méneville, 1831, by monotypy.

Family-group name: HÉTÉRONYCIDES Lacordaire, 1855. Stem: *Heteronych*-. Note: senior homonym of HETERONYCHIDAE André, 1891 (type genus *Heteronyx* Saussure, 1887) in Hymenoptera; André’s name is permanently invalid (based on a preoccupied type genus).

***Heteropalpus* Buquet, 1843**: pl. 118. Current status: valid genus in DISTENIIDAE: HETEROPALPINI.

Type species: *Heteropalpuspretiosus* Buquet,1843, by monotypy.

Family-group name: HETEROPALPINI Villiers, 1961. Stem: *Heteropalp*-.

***Heteropaussus* J. Thomson, 1860c**: 68, 70. Current status: valid genus in CARABIDAE: PAUSSINAE: PAUSSINI: HETEROPAUSSINA.

Type species: *Cerapterusalternans* Westwood, 1849, by monotypy.

Family-group name: HETEROPAUSSINES E. Janssens, 1950. Stem: *Heteropauss*-.

***Heterophana* Burmeister, 1842**: 602. Current status: valid genus in SCARABAEIDAE: CETONIINAE: STENOTARSIINI: HETEROPHANINA.

Type species: *Cetoniacanaliculata* Gory & Percheron, 1833, by subsequent designation (Sakai and Nagai 1998: 182).

Family-group name: HETEROPHANAE Schoch, 1894. Stem: *Heterophan*-.

***Heterops* Blanchard, 1842a**: 51. Current status: valid genus in CERAMBYCIDAE: CERAMBYCINAE: EBURIINI.

Type species: *Purpuricenusloreyi* Duponchel, 1837, by subsequent designation (J. Thomson 1864: 203).

Family-group name: HÉTÉROPSIDES Lacordaire, 1868. Stem: *Heterops*-. Note: nomen protectum (see Bousquet et al. 2009: 48).

***Heterorhina* Westwood, 1842b**: 132. Current status: valid genus in SCARABAEIDAE: CETONIINAE: GOLIATHINI: RHOMBORHININA. Note: two original spellings were used, including *Hetororhina* (p. 134); Neave (1940: 644) acted as First Reviser (ICZN 1999a, Article 24.2.3) and selected the spelling *Heterorhina*, the spelling currently in prevailing usage in publications dealing with CETONIINAE or SCARABAEIDAE classification and nomenclature.

Type species: *Cetonianigritarsis* Hope, 1831, by subsequent designation (Duponchel 1845: 603).

Family-group name: HETERORRHINIDAE Kraatz, 1880. Stem: *Heterorhin*-.

***Heteroscapha* Achard, 1914a**: 394. Current status: junior synonym of *Bironium* Csiki, 1909 in STAPHYLINIDAE: SCAPHIDIINAE: SCAPHISOMATINI.

Type species: *Heteroscaphafeai* Achard, 1914, by original designation.

Family-group name: HETEROSCAPHINI Achard, 1914. Stem: *Heteroscaph*-.

***Heterosoma* Schaum, 1845**: 390, 426. Current status: valid genus in SCARABAEIDAE: CETONIINAE: STENOTARSIINI: HETEROSOMATINA.

Type species: *Cetoniacollata* Gory & Percheron, 1835, by monotypy.

Family-group name: HETEROSOMATINA Krikken, 1984. Stem: *Heterosomat*-.

***Heterosternus* Dupont, 1832**: pl. 10. Current status: valid genus in SCARABAEIDAE: RUTELINAE: RUTELINI: HETEROSTERNINA.

Type species: *Heterosternusbuprestoides* Dupont, 1832, by monotypy.

Family-group name: HETEROSTERNINAE H.W. Bates, 1888. Stem: *Heterostern*-. Note: nomen protectum (see A.B.T. Smith 2006: 171).

***Heterota* Mulsant & Rey, 1873**: 162. Current status: valid genus in STAPHYLINIDAE: ALEOCHARINAE: HOMALOTINI: BOLITOCHARINA.

Type species: *Homalotaplumbea* G.R. Waterhouse, 1858, by monotypy.

Family-group name: HETEROTAE Fenyes, 1919. Stem: *Heterot*-.

***Heterotarsus* Latreille, 1829b**: 26. Current status: valid genus in TENEBRIONIDAE: BLAPTINAE: OPATRINI: HETEROTARSINA.

Type species: *Heterotarsustenebrioides* Guérin-Méneville, 1831, by subsequent monotypy (Guérin-Méneville 1831b: pl. 30).

Family-group name: HÉTÉROTARSITES Blanchard, 1845. Stem: *Heterotars*-.

***Heterotaxus* Bernhauer, 1915a**: 313. Current status: valid genus in STAPHYLINIDAE: ALEOCHARINAE: OXYPODININI.

Type species: *Heterotaxusbihastatus* Bernhauer, 1915, by monotypy.

Family-group name: HETEROTAXINI Fenyes, 1921. Stem: *Heterotax*-.

***Heterothops* Stephens, 1829b**: 23. Current status: valid genus in STAPHYLINIDAE: STAPHYLININAE: STAPHYLININI: AMBLYOPININA.

Type species: *Staphylinusbinotatus* Gravenhorst, 1802, by monotypy. Note: name placed on the Official List of Specific Names in Zoology (ICZN 2003b).

Family-group name: HETEROTHOPSI Coiffait, 1978. Stem: *Heterothop*-.

***Hexacolus* Eichhoff, 1868b**: 399. Current status: junior synonym of *Scolytodes* Ferrari, 1867 in CURCULIONIDAE: SCOLYTINAE: HEXACOLINI.

Type species: *Hexacolusglaber* Eichhoff, 1868, by monotypy.

Family-group name: HEXACOLIDAE Eichhoff, 1878. Stem: *Hexacol*-.

***Hexagonia* W. Kirby, 1825**: 563. Current status: valid genus in CARABIDAE: HARPALINAE: HEXAGONIINI.

Type species: *Hexagoniaterminata* W. Kirby, 1825, by monotypy.

Family-group name: HEXAGONIAE G.H. Horn, 1881. Stem: *Hexagoni*-.

***Hexaplatarthrus* Jeannel, 1955**: 181. Current status: valid genus in CARABIDAE: PAUSSINAE: PAUSSINI: PENTAPLATARTHRINA.

Type species: *Hexaplatarthrusvadoni* Jeannel, 1955, by original designation.

Family-group name: HEXAPLATARTHRINA Luna de Carvalho, 1961. Stem: *Hexaplatarthr*-.

***Hexatricha* A. White, 1846**: 21. Current status: valid genus in CERAMBYCIDAE: LAMIINAE: PARMENINI.

Type species: *Lamiapulverulenta* Westwood, 1843, by monotypy.

Family-group name: HEXATHRICITAE J. Thomson, 1864. Stem: *Hexatrich*-.

***Hexodon* G.-A. Olivier, 1789b**: [07] 3. Current status: valid genus in SCARABAEIDAE: DYNASTINAE: HEXODONTINI.

Type species: *Hexodonreticulatum* G.-A. Olivier, 1789, by subsequent designation (Latreille 1810: 428).

Family-group name: HEXODONTIDES Lacordaire, 1855. Stem: *Hexodont*-.

***Hexoplon* J. Thomson, 1864**: 219. Current status: valid genus in CERAMBYCIDAE: CERAMBYCINAE: HEXOPLONINI.

Type species: *Hexoplonvenus* J. Thomson, 1864, by original designation.

Family-group name: HEXOPLONINI Martins, 2006. Stem: *Hexoplon*- (ICZN 1999a, Article 29.4).

***Hiletus* Schiødte, 1847**: 346. Current status: valid genus in CARABIDAE: HILETINAE.

Type species: *Hiletusversutus* Schiødte, 1847, by monotypy.

Family-group name: HILETINI Schiødte, 1847. Stem: *Hilet*-.

***Himalusa* Pace, 2006**: 357. Current status: valid genus in STAPHYLINIDAE: ALEOCHARINAE: HIMALUSINI.

Type species: *Himalusaannapurnensis* Pace, 2006, by original designation.

Family-group name: HIMALUSINI Klimaszewski, Pace & Center, 2010. Stem: *Himalus*-.

***Himasthlophallus* Egorov & Zherikhin in Zherikhin and Egorov, 1991**: 32. Current status: valid genus in CURCULIONIDAE: BRACHYCERINAE: HIMASTHLOPHALLINI.

Type species: *Himasthlophallusflagellifer* Egorov & Zherikhin, 1991, by original designation.

Family-group name: HIMASTHLOPHALLINI Zherikhin, 1991. Stem: *Himasthlophall*-.

***Himatinum* Cockerell, 1906**: 243. Current status: junior synonym of *Himatium* Wollaston, 1873 in CURCULIONIDAE: COSSONINAE: HIMATININI. Note: unnecessary new replacement name for *Himatium* Wollaston, 1873.

Type species: *Himatiumpubescens* Wollaston, 1873, by monotypy (type fixation for *Himatium* Wollaston).

Family-group name: HIMATININI Konishi, 1962. Stem: *Himatin*-.

***Himatismus* Erichson, 1843b**: 253. Current status: valid genus in TENEBRIONIDAE: PIMELIINAE: TENTYRIINI.

Type species: *Himatismusmandibularis* Erichson, 1843, by subsequent designation (Gebien 1937: 574).

Family-group name: HIMATISMINA Skopin, 1979. Stem: *Himatism*-.

***Himatolabus* Jekel, 1860**: 189. Current status: valid genus in ATTELABIDAE: ATTELABINAE: ATTELABINI: HIMATOLABINA.

Type species: *Attelabusvestitus* Gyllenhal, 1839, by original designation.

Family-group name: HIMATOLABINA Legalov, 2003. Stem: *Himatolab*-.

***Hippodamia* Chevrolat in Dejean, 1836a**: 432. Current status: valid genus in COCCINELLIDAE: COCCINELLINAE: COCCINELLINI.

Type species: *Coccinellamutabilis* Scriba, 1791 (= *Coccinellavariegata* Goeze, 1777), by subsequent designation (Chevrolat 1845: 623). Note: Goeze’s (1777) work is not consistently binominal (see ICZN 2021a) and therefore an application to the Commission is necessary to retain *Coccinellavariegata* Goeze, 1777 as the valid name for the type species.

Family-group name: HIPPODAMIINI Weise, 1885. Stem: *Hippodami*-.

***Hippoloetis* Laporte, 1834c**: 152. Current status: valid genus in CARABIDAE: HARPALINAE: HARPALINI: STENOLOPHINA. Note: Laporte used two original spellings, including *Hipollaetis* (p. 153); Laporte (1840a: 95) subsequently used only *Hippoloetis* and therefore acted as First Reviser (ICZN 1999a, Article 24.2.4).

Type species: *Hippoloetisrufa* Laporte, 1834, by monotypy.

Family-group name: HIPPOLAETINA Basilewsky, 1951. Stem: *Hippoloet*-.

***Hippomelas* Laporte & Gory, 1837**: 3. Current status: valid genus in BUPRESTIDAE: CHRYSOCHROINAE: DICERCINI: HIPPOMELANINA.

Type species: *Buprestismexicana* Laporte & Gory, 1837, by monotypy.

Family-group name: HIPPOMELANINA Holyński, 1993. Stem: *Hippomelan*-.

***Hippopsicon* J. Thomson, 1858b**: 195. Current status: valid genus in CERAMBYCIDAE: LAMIINAE: AGAPANTHIINI.

Type species: *Hippopsiconlacteolum* J. Thomson, 1858, by monotypy.

Family-group name: HIPPOPSICONINI L.S. Dillon & E.S. Dillon, 1945. Stem: *Hippopsicon*-.

***Hippopsis* Lepeletier & Audinet-Serville, 1825**: 336. Current status: valid genus in CERAMBYCIDAE: LAMIINAE: AGAPANTHIINI.

Type species: *Hippopsislineolatus* Lepeletier & Audinet-Serville, 1825, by subsequent designation (J. Thomson 1864: 97). Note: although Thomson (1864: 97) listed *Saperdalemniscata* Fabricius, 1801, not an originally included nominal species, as type species of *Hippopsis*, he listed “*Hippopsislineolata* [sic] Lepeletier & Audinet-Serville”, one of the two originally included species in *Hippopsis*, at the same time in synonymy with *Saperdalemniscata*, he is therefore deemed to have designed *Hippopsislineolata* Lepeletier & Audinet-Serville as type species (ICZN 1999a, Article 69.2.2).

Family-group name: HIPPOPSITAE J. Thomson, 1860. Stem: *Hippopse*-.

***Hipporhis* Billberg, 1820**: 45. Current status: junior synonym of *Bronchus* Germar, 1817 in CURCULIONIDAE: CYCLOMINAE: HIPPORHININI. Note: unnecessary new replacement name for *Bronchus* Germar, 1817.

Type species: *Curculiocapensis* Linnaeus, 1764, by subsequent designation (Alonso-Zarazaga and Lyal 1999: 21, 138) (type fixation for *Bronchus* Germar).

Family-group name: HIPPORHINIDES Lacordaire, 1863. Stem: *Hipporhin*-.

***Hispa* Linnaeus, 1767**: 603. Current status: valid genus in CHRYSOMELIDAE: CASSIDINAE: HISPINI.

Type species: *Hispaatra* Linnaeus, 1767, by subsequent designation (Latreille 1810: 432).

Family-group name: HISPOIDEAE Gyllenhal, 1813. Stem: *Hisp*-.

†***Hispanoclavina* Soriano, Ponomarenko & Delclòs, 2007**: 527. Current status: valid genus in †COPTOCLAVIDAE: †HISPANOCLAVINAE. Note: Lower Cretaceous (Spain).

Type species: †*Hispanoclavinadiazromerali* Soriano, Ponomarenko & Delclòs, 2007, by original designation.

Family-group name: †HISPANOCLAVINAE Soriano, Ponomarenko & Delclòs, 2007. Stem: *Hispanoclav*- (ICZN 1999a, Article 29.4).

***Hispodes* G.A.K. Marshall, 1955a**: 23. Current status: valid genus in BELIDAE: OXYCORYNINAE: OXYCORYNITAE: HISPODINI.

Type species: *Hispodesspicatus* G.A.K. Marshall, 1955, by original designation.

Family-group name: HISPODINI Voss, 1957. Stem: *Hispod*-.

***Hispoleptis* Baly, 1864d**: 262. Current status: valid genus in CHRYSOMELIDAE: CASSIDINAE: HISPOLEPTINI.

Type species: *Promecothecadiluta* Guérin-Méneville, 1840, by original designation.

Family-group name: HISPOLEPTITES Chapuis, 1875. Stem: *Hispolept*-.

***Hispostoma* Weise, 1907**: 134. Current status: valid genus in CHRYSOMELIDAE: CHRYSOMELINAE: CHRYSOMELINI.

Type species: *Hispostomamarginatum* Weise, 1907, by monotypy.

Family-group name: HISPOSTOMINI Daccordi, 1981. Stem: *Hispostomat*-.

***Hister* Linnaeus, 1758**: 342, 358. Current status: valid genus in HISTERIDAE: HISTERINAE: HISTERINI.

Type species: *Histerunicolor* Linnaeus, 1758, by subsequent designation (Latreille 1810: 428).

Family-group name: HISTEROIDES Gyllenhal, 1808. Stem: *Hister*-.

***Hobartius* Sen Gupta & Crowson, 1966**: 74. Current status: valid genus in HOBARTIIDAE.

Type species: *Hobartiustasmanicus* Sen Gupta & Crowson, 1966 (= *Atomariaeucalypti* Blackburn, 1892), by original designation.

Family-group name: HOBARTIINI Sen Gupta & Crowson, 1966. Stem: *Hobarti*-.

***Hodoxenus* Kistner, 1970b**: 12. Current status: valid genus in STAPHYLINIDAE: ALEOCHARINAE: ALEOCHARINI: HODOXENINA.

Type species: *Hodoxenussheasbyi* Kistner, 1970, by original designation.

Family-group name: HODOXENINA Kistner, 1970. Stem: *Hodoxen*-.

***Holcorhinus* Schönherr, 1826**: 194. Current status: valid genus in CURCULIONIDAE: ENTIMINAE: OTIORHYNCHITAE: HOLCORHININI.

Type species: *Holcorhinushispidulus* Thon, 1833, by subsequent monotypy (Thon 1833: 13).

Family-group name: HOLCORHINIDAE Desbrochers des Loges, 1898. Stem: *Holcorhin*-.

†***Holcorobeus* Nikritin, 1977**: 127. Current status: valid genus in SCARABAEIDAE: ACLOPINAE: †HOLCOROBEINI. Note: Upper Jurassic/Lower Cretaceous (China; Kazakhstan; Mongolia; Russia); two original spellings were used, including *Holcoribeus* (p. 127); Krell (2000: 889) acted as First Reviser (ICZN 1999a, Article 24.2.3) and selected *Holcorobeus*.

Type species: †*Holcorobeusvittatus* Nikritin, 1977, by original designation.

Family-group name: †HOLCOROBEINI Nikolajev, 1992. Stem: *Holcorobe*-.

***Holisus* Erichson, 1839b**: 298. Current status: valid genus in STAPHYLINIDAE: STAPHYLININAE: STAPHYLININI: HYPTIOMINA.

Type species: *Holisusanalis* Erichson, 1839, by subsequent designation (Duponchel 1841b: 57).

Family-group name: HOLISINA Newton, 1988. Stem: *Holis*-.

***Hololepta* Paykull, 1811**: 101. Current status: valid genus in HISTERIDAE: HISTERINAE: HOLOLEPTINI.

Type species [suggested]: *Histerplanus* Sulzer, 1776, by subsequent designation (Hope 1841a: 105). Note: the valid type species fixation is that of J.E. Gray (1831b: 323), who selected *Histerdepressus* Fabricius, 1787, but that species is currently a junior synonym of *Histercompressus* Herbst, 1783 in the genus *Platysoma* Leach, 1817; an application to the Commission is necessary to fix *Histerplanus* Sulzer, 1776 as the type species of *Hololepta* Paykull, 1811.

Family-group name: HOLOLEPTIDAE Hope, 1841. Stem: *Hololept*-.

***Holoparamecus* Curtis, 1833b**: 186. Current status: valid genus in ENDOMYCHIDAE: MEROPHYSIINAE.

Type species: *Holoparamecusdepressus* Curtis, 1833, by monotypy.

Family-group name: HOLOPARAMECINI Seidlitz, 1888. Stem: *Holoparamec*-.

***Holopleura* J.L. LeConte, 1873**: 193. Current status: valid genus in CERAMBYCIDAE: CERAMBYCINAE: HOLOPLEURINI.

Type species: *Holopleuramarginata* J.L. LeConte, 1873, by subsequent designation (Linsley 1962: 181).

Family-group name: HOLOPLEURINI Chemsak & Linsley, 1974. Stem: *Holopleur*-.

***Holopterus* Blanchard, 1851**: 475. Current status: valid genus **stat. nov.** in CERAMBYCIDAE: CERAMBYCINAE: HOLOPTERINI.

Type species: *Holopteruschilensis* Blanchard, 1851, by monotypy.

Family-group name: HOLOPTÉRIDES Lacordaire, 1868. Stem: *Holopter*-.

***Holostrophus* G.H. Horn, 1888**: 32, 36. Current status: valid genus in TETRATOMIDAE: EUSTROPHINAE: HOLOSTROPHINI.

Type species: *Eustrophusbifasciatus* Say, 1824, by subsequent designation (Nikitsky 1998: 48).

Family-group name: HOLOSTROPHINI Nikitsky, 1998. Stem: *Holostroph*-.

***Holozodus* Fairmaire, 1898**: 346. Current status: valid genus in STAPHYLINIDAE: PSELAPHINAE: PSELAPHITAE: ARHYTODINI. Note: new replacement name for *Hologlyptus* Fairmaire, 1898.

Type species: *Hologlyptusraffrayi* Fairmaire, 1898, by monotypy (type fixation for *Hologlyptus* Fairmaire).

Family-group name: HOLOZODINI Raffray, 1900. Stem: *Holozod*-.

***Homalocerus* Schönherr, 1839**: 358. Current status: valid genus in BELIDAE: BELINAE: BELITAE: BELINI: HOMALOCERINA.

Type species: *Rhinotialyciformis* Germar, 1833, by original designation.

Family-group name: HOMALOCERINA Legalov, 2009. Stem: *Homalocer*-.

***Homalota* Mannerheim, 1830**: 73. Current status: valid genus in STAPHYLINIDAE: ALEOCHARINAE: HOMALOTINI.

Type species: *Aleocharaplana* Gyllenhal, 1810, by monotypy.

Family-group name: HOMALOTIDA Heer, 1839. Stem: *Homalot*-.

***Homebius* Endrödy-Younga, 1989**: 124. Current status: valid genus in TENEBRIONIDAE: PIMELIINAE: CRYPTOCHILINI: HOMEBIINA.

Type species: *Homebiuskaszabi* Endrödy-Younga, 1989, by original designation.

Family-group name: HOMEBIINA Endrödy-Younga, 1989. Stem: *Homebi*-.

***Homethes* Newman, 1842c**: 402. Current status: valid genus in CARABIDAE: HARPALINAE: ODACANTHINI: HOMETHINA.

Type species: *Hometheselegans* Newman, 1842, by monotypy.

Family-group name: HOMETHINA Liebherr, 2016: 223. Stem: *Hometh*-.

***Homoeodera* Wollaston, 1870**: 23. Current status: valid genus in ANTHRIBIDAE: CHORAGINAE: HOMOEODERINI.

Type species: *Homoeoderarotundipennis* Wollaston, 1870, by subsequent designation (Basilewsky 1972: 274).

Family-group name: HOMOEODERIDES Wollaston, 1870. Stem: *Homoeoder*-.

***Homoeoplastus* Gistel, 1856**: 360. Current status: junior synonym *Byturus* Latreille, 1797 in BYTURIDAE: BYTURINAE.

Type species: *Dermestestomentosus* DeGeer, 1774, by subsequent designation (Bouchard and Bousquet 2020a: 93).

Family-group name: HOMOEOPLASTIDAE Gistel, 1856. Stem: *Homoeoplast*-.

***Homonoea* Newman, 1842a**: 319. Current status: valid genus in CERAMBYCIDAE: LAMIINAE: HOMONOEINI.

Type species: *Homonoeapatrona* Newman, 1842, by subsequent designation (J. Thomson 1864: 35).

Family-group name: HOMONAEITAE J. Thomson, 1864. Stem: *Homonoe*-.

***Homopterus* Westwood, 1840a**: 75. Current status: valid genus in CARABIDAE: PAUSSINAE: PAUSSINI: HOMOPTERINA.

Type species: *Cerapterusbrasiliensis* Westwood, 1840, by original designation.

Family-group name: HOMOPTERINAE Wasmann, 1920. Stem: *Homopter*-. Note: junior homonym of HOMOPTERINAE Boisduval, 1852 (type genus *Homoptera* Boisduval, 1852) in Lepidoptera.

***Homorhythmus* Bedel, 1883**: 38. Current status: junior synonym of *Simo* Dejean, 1821 in CURCULIONIDAE: ENTIMINAE: CYPHICERITAE: PERITELINI: SIMONINA.

Type species: *Curculiohirticornis* Herbst, 1795, by monotypy.

Family-group name: HOMORYTHMINI Hoffmann, 1950. Stem: *Homorhythm*-.

***Hoplandria* Kraatz, 1857a**: 4. Current status: valid genus in STAPHYLINIDAE: ALEOCHARINAE: HOPLANDRIINI: HOPLANDRIINA.

Type species: *Hoplandriaochracea* Kraatz, 1857 (= *Gyrophaenalateralis* Melsheimer, 1844), by subsequent designation (Casey 1910: 170).

Family-group name: HOPLANDRIAE Casey, 1910. Stem: *Hoplandri*-.

***Hoplapoderus* Jekel, 1860**: 171. Current status: valid genus in ATTELABIDAE: APODERINAE: HOPLAPODERINI: HOPLAPODERINA.

Type species: *Attelabusgemmatus* Thunberg, 1784, by subsequent designation (Voss 1925: 5).

Family-group name: HOPLAPODERINI Voss, 1926. Stem: *Hoplapoder*-.

***Hoplia* Illiger, 1803**: 226. Current status: valid genus in SCARABAEIDAE: MELOLONTHINAE: HOPLIINI: HOPLIINA.

Type species: *Scarabaeusfarinosus* Linnaeus, 1761 (= *Scarabaeusargenteus* Poda von Neuhaus, 1761), by subsequent designation (Latreille 1810: 428).

Family-group name: HOPLIDES Latreille, 1829. Stem: *Hopli*-.

***Hoplideres* Audinet-Serville, 1832**: 147. Current status: valid genus in CERAMBYCIDAE: PRIONINAE: HOPLIDERINI.

Type species: *Hoplideresspinipennis* Audinet-Serville, 1832, by monotypy.

Family-group name: HOPLIDERITAE J. Thomson, 1864. Stem: *Hoplider*-.

***Hoplionota* Hope, 1841a**: 153. Current status: junior synonym of *Notosacantha* Chevrolat, 1836 in CHRYSOMELIDAE: CASSIDINAE: NOTOSACANTHINI.

Type species: *Cassidaechinata* Fabricius, 1801, by original designation.

Family-group name: HOPLIONOTITAE Spaeth, 1929. Stem: *Hoplionot*-.

***Hoplitoxenus* Jeannel, 1960**: 73, 173. Current status: valid genus in STAPHYLINIDAE: PSELAPHINAE: CLAVIGERITAE: CLAVIGERINI.

Type species: *Hoplitoxenusjoannae* Jeannel, 1960, by original designation.

Family-group name: HOPLITOXENINI Célis, 1969. Stem: *Hoplitoxen*-.

***Hoplopisthius* Senna, 1892**: 451. Current status: valid genus in BRENTIDAE: BRENTINAE: TRACHELIZINI: HOPLOPISTHIINA.

Type species: *Hoplopisthiustrichemerus* Senna, 1892, by monotypy.

Family-group name: HOPLOPISTHI Senna & Calabresi, 1919. Stem: *Hoplopisthi*-.

***Hoplorhinus* Chevrolat, 1878a**: 163. Current status: junior synonym of *Celetes* Schönherr, 1836 in CURCULIONIDAE: CURCULIONINAE: DERELOMINI: DERELOMINA.

Type species: *Hoplorhinusmelanocephalus* Chevrolat, 1878, by **present designation**.

Family-group name: HOPLORRHININA Champion, 1903. Stem: *Hoplorhin*-.

***Horatoma* Solier, 1841a**: 210, 264. Current status: valid genus in TENEBRIONIDAE: PIMELIINAE: CRYPTOCHILINI: CRYPTOCHILINA.

Type species: *Horatomaparvula* Solier, 1841, by original designation.

Family-group name: HORATOMINA C. Koch, 1955. Stem: *Horatom*-.

***Horelophopsis* Hansen, 1997**: 109. Current status: valid genus in HYDROPHILIDAE: ACIDOCERINAE.

Type species: *Horelophopsisavita* Hansen, 1997, by original designation.

Family-group name: HORELOPHOPSINAE Hansen, 1997. Stem: *Horelophops*-.

***Horelophus* Orchymont, 1913a**: 94, 99. Current status: valid genus in HYDROPHILIDAE: CHAETARTHRIINAE: ANACAENINI.

Type species: *Horelophuswalkeri* Orchymont, 1913, by monotypy.

Family-group name: HORELOPHINAE Hansen, 1991. Stem: *Horeloph*-.

***Horia* Fabricius, 1787**: 164. Current status: valid genus in MELOIDAE: NEMOGNATHINAE: HORIINI. Note: name placed on the Official List of Generic Names in Zoology (ICZN 1999c).

Type species: *Lymexylontestaceum* Fabricius, 1781 (= *Horiafabriciana* Betrem, 1929), by subsequent designation (Betrem 1929: xxvii; see ICZN 1999c, as *Horiafabriciana* Betrem, 1929). Note: *Horiafabriciana* Betrem, 1929 placed on the Official List of Specific Names in Zoology (ICZN 1999c).

Family-group name: HORIALES Latreille, 1802. Stem: *Hori*-.

***Hormops* J.L. LeConte in J.L. LeConte and G.H. Horn, 1876**: 321. Current status: valid genus in CURCULIONIDAE: MOLYTINAE: PETALOCHILINI.

Type species: *Hormopsabducens* J.L. LeConte, 1876, by monotypy.

Family-group name: HORMOPINI J.L. LeConte, 1876. Stem: *Hormop*-.

***Hormorus* G.H. Horn in J.L. LeConte and G.H. Horn, 1876**: 23. Current status: valid genus in CURCULIONIDAE: ENTIMINAE: OTIORHYNCHITAE: HORMORINI.

Type species: *Chlorophanusundulatus* Uhler, 1855, by monotypy.

Family-group name: HORMORI G.H. Horn, 1876. Stem: *Hormor*-.

***Hornia* C.V. Riley, 1877**: 564, 565. Current status: valid genus in MELOIDAE: NEMOGNATHINAE: NEMOGNATHINI: NEMOGNATHINA.

Type species: *Horniaminutipennis* C.V. Riley, 1877, by monotypy.

Family-group name: HORNII Dugès, 1889. Stem: *Horni*-.

***Hornibius* Fairmaire, 1888a**: 60, 61. Current status: junior synonym of *Hornius* Fairmaire, 1885 in CHRYSOMELIDAE: SPILOPYRINAE. Note: unnecessary new replacement name for *Hornius* Fairmaire, 1885.

Type species: *Horniussulcifrons* Fairmaire, 1885, by monotypy (type fixation for *Hornius* Fairmaire).

Family-group name: HORNIBIINI Crowson, 1946. Stem: *Hornibi*-.

***Hornietus* Stebnicka, 2000**: 287. Current status: valid genus in SCARABAEIDAE: APHODIINAE: HORNIETIINI.

Type species: *Pleurophorusventralis* G.H. Horn, 1887, by original designation.

Family-group name: HORNIETIINI Minkina, 2020: 444. Stem: *Hornieti*- (ICZN 1999a, Article 29.3.3).

***Horologion* J.M. Valentine, 1932**: 2. Current status: valid genus in CARABIDAE: TRECHINAE: HOROLOGIONINI.

Type species: *Horologionspeokoites* J.M. Valentine, 1932, by monotypy.

Family-group name: HOROLOGIONIDAE Jeannel, 1949. Stem: *Horologion*-.

***Hybalus* Dejean, 1833b**: 149. Current status: valid genus in SCARABAEIDAE: ORPHNINAE: ORPHNINI.

Type species: *Geobiuscornifrons* Brullé, 1832, by monotypy.

Family-group name: HYBALIDAE Marseul, 1857. Stem: *Hybal*-.

***Hybocephalus* Motschulsky, 1852**: 482. Current status: valid genus in STAPHYLINIDAE: PSELAPHINAE: PSELAPHITAE: HYBOCEPHALINI.

Type species: *Hybocephalusminimus* Motschulsky, 1852, by subsequent designation (R. Lucas 1920: 339).

Family-group name: HYBOCEPHALINI Raffray, 1890. Stem: *Hybocephal*-.

***Hybodera* J.L. LeConte, 1873**: 191. Current status: valid genus in CERAMBYCIDAE: CERAMBYCINAE: HYBODERINI.

Type species: *Hyboderatuberculata* J.L. LeConte, 1873, by monotypy.

Family-group name: HYBODERINI Linsley, 1940. Stem: *Hyboder*-.

***Hybolabus* Jekel, 1860**: 195. Current status: valid genus in ATTELABIDAE: ATTELABINAE: ATTELABINI: HYBOLABINA.

Type species: *Attelabusvariabilis* Gyllenhal, 1833 (= *Attelabusater* G.-A. Olivier, 1789), by original designation.

Family-group name: HYBOLABINA Voss, 1925. Stem: *Hybolab*-.

***Hybomorphus* Saunders & Jekel, 1855**: 301. Current status: valid genus in CURCULIONIDAE: CURCULIONINAE: STOREINI.

Type species: *Hybomorphusmelanosomus* Saunders & Jekel, 1855, by original designation.

Family-group name: HYBOMORPHIDES Lacordaire, 1865. Stem: *Hybomorph*-.

***Hyborhabdus* Aurivillius, 1911b**: 22. Current status: valid genus in CERAMBYCIDAE: LAMIINAE: HYBORHABDINI.

Type species: *Hyborhabdussingularis* Aurivillius, 1911, by monotypy.

Family-group name: HYBORHABDINAE Aurivillius, 1911. Stem: *Hyborhabd*-.

***Hybosispa* Weise, 1910b**: 96. Current status: valid genus in CHRYSOMELIDAE: CASSIDINAE: HYBOSISPINI.

Type species: *Hybosispamelanura* Weise, 1910, by monotypy.

Family-group name: HYBOSITES Weise, 1910. Stem: *Hybosisp*-.

***Hybosorus* W.S. MacLeay, 1819**: 120. Current status: valid genus in HYBOSORIDAE: HYBOSORINAE.

Type species: **here fixed** (under ICZN 1999a, Article 70.3.2) as *Hybosorusilligeri* Reiche, 1853, misidentified as *Scarabaeusarator* Fabricius, 1775 (sensu W.S. MacLeay, 1819) in the original designation by monotypy in W.S. MacLeay (1819: 120). Note: see Branco (2007: 13) for interpretation of the type species; *Hybosorusilligeri* Reiche, 1853 was placed on the Official List of Specific Names in Zoology (ICZN 2021c).

Family-group name: HYBOSORIDAE Erichson, 1847. Stem: *Hybosor*-.

***Hydaticus* Leach, 1817**: 69, 72. Current status: valid genus in DYTISCIDAE: DYTISCINAE: HYDATICINI.

Type species: *Dytiscustransversalis* Pontoppidan, 1763, by subsequent designation (Curtis 1825: pl. 95).

Family-group name: HYDATICINI Sharp, 1880. Stem: *Hydatic*-.

***Hydatophilus* Gistel, 1856**: 353. Current status: junior synonym of *Hydrophilus* Geoffroy, 1762 in HYDROPHILIDAE: HYDROPHILINAE: HYDROPHILINI.

Type species: *Dytiscuspiceus* Linnaeus, 1758, by monotypy.

Family-group name: HYDATOPHILIDAE Gistel, 1856. Stem: *Hydatophil*-.

***Hyderodes* Hope, 1838a**: 131, 166. Current status: valid genus in DYTISCIDAE: DYTISCINAE: DYTISCINI.

Type species: *Hyderodesshuckardi* Hope, 1838, by original designation.

Family-group name: HYDERODINI K.B. Miller, 2000. Stem: *Hyderod*-.

***Hydnobius* W.L.E. Schmidt, 1841**: 193. Current status: valid genus in LEIODIDAE: LEIODINAE: SOGDINI.

Type species: *Anisotomapunctatum* Sturm, 1807, by subsequent designation (C.G. Thomson 1859: 58).

Family-group name: HYDNOBIINI Jeannel, 1962. Stem: *Hydnobi*-.

***Hydnocera* Newman, 1838**: 379. Current status: junior synonym of *Phyllobaenus* Dejean, 1833 in CLERIDAE: CLERINAE: CLERITAE: HYDNOCERINI: HYDNOCERINA.

Type species: *Hydnoceraserrata* Newman, 1838 (= *Cleruspallipennis* Say, 1825), by monotypy.

Family-group name: HYDNOCÉROÏDES Spinola, 1844. Stem: *Hydnocer*-.

***Hydraena* Kugelann, 1794**: 578. Current status: valid genus in HYDRAENIDAE: HYDRAENINAE: HYDRAENINI.

Type species: *Hydraenariparia* Kugelann, 1794, by monotypy.

Family-group name: HYDRAENAIRES Mulsant, 1844. Stem: *Hydraen*-.

***Hydraenida* Germain, 1901**: 531, 537. Current status: valid genus in HYDRAENIDAE: HYDRAENINAE: HYDRAENIDINI. Note: the original spelling was *Hydroenida*, but *Hydraenida*, an incorrect subsequent spelling introduced by d’ Orchymont (1913b: 103), is in prevailing usage, attributed to the publication of the original spelling, and therefore deemed to be the correct original spelling (ICZN 1999a, Article 33.3.1).

Type species: *Hydraenidaocellata* Germain, 1901, by monotypy.

Family-group name: HYDRAENIDINI Perkins, 1980. Stem: *Hydraenid*-.

***Hydrobius* Leach, 1815a**: 96. Current status: valid genus in HYDROPHILIDAE: HYDROPHILINAE: HYDROBIUSINI. Note: name placed on the Official List of Generic Names in Zoology (ICZN 1990a).

Type species: *Dytiscusfuscipes* Linnaeus, 1758, by subsequent designation (Hope 1838a: 125 [December]; see ICZN 1990a). Note: name placed on the Official List of Specific Names in Zoology (ICZN 1990a); Westwood (1838c: 10 [May]) is the first author who selected that species as type species of *Hydrobius* Leach, 1815; however, the Commission (ICZN 1990a) set aside all fixations of type species for the nominal genus *Hydrobius* Leach, 1815 before the designation of Hope (1838a).

Family-group name: HYDROBIAIRES Mulsant, 1844. Stem: *Hydrobius*- (ICZN 2003a).

***Hydrocanthus* Say, 1823**: 105. Current status: valid genus in NOTERIDAE: NOTERINAE: NOTERINI.

Type species: *Hydrocanthusiricolor* Say, 1823, by monotypy.

Family-group name: HYDROCANTHINI Sharp, 1880. Stem: *Hydrocanth*-.

***Hydrochus* Leach, 1817**: 90. Current status: valid genus in HYDROCHIDAE.

Type species: *Silphaelongata* Schaller, 1783, by subsequent designation (Curtis 1831: pl. 359).

Family-group name: HYDROCHIDAE C.G. Thomson, 1859. Stem: *Hydroch*-.

***Hydrocoptus* Motschulsky, 1853c**: 5. Current status: junior synonym of *Hydroporus* Clairville, 1806 in DYTISCIDAE: HYDROPORINAE: HYDROPORINI: HYDROPORINA.

Type species: *Dytiscustristis* Paykull, 1798, by subsequent designation (Nilsson et al. 1989: 297).

Family-group name: HYDROCOPTINI Kolbe, 1883. Stem: *Hydrocopt*-.

***Hydrodytes* K.B. Miller, 2001**: 77. Current status: valid genus in DYTISCIDAE: HYDRODYTINAE.

Type species: *Agaporomorphusopalinus* A. Zimmermann, 1921, by original designation.

Family-group name: HYDRODYTINAE K.B. Miller, 2001. Stem: *Hydrodyt*-.

***Hydronebrius* Jakowlew, 1897**: 37. Current status: valid genus in DYTISCIDAE: AGABINAE: AGABINI.

Type species: *Agabuscordaticollis* Reitter, 1896, by original designation.

Family-group name: HYDRONEBRIINI Guignot, 1948. Stem: *Hydronebri*-.

***Hydronomus* Schönherr, 1825**: 583. Current status: junior synonym of *Bagous* Germar, 1817 in CURCULIONIDAE: BAGOINAE.

Type species: *Curculioalismatis* Marsham, 1802, by original designation.

Family-group name: HYDRONOMIDES Lacordaire, 1863. Stem: *Hydronom*-.

***Hydrophilus* Geoffroy, 1762**: 180. Current status: valid genus in HYDROPHILIDAE: HYDROPHILINAE: HYDROPHILINI. Note: name placed on the Official List of Generic Names in Zoology (ICZN 1994a).

Type species: *Dytiscuspiceus* Linnaeus, 1758, by subsequent designation (Latreille 1810: 428; see ICZN 1994a). Note: name placed on the Official List of Specific Names in Zoology (ICZN 1994a).

Family-group name: HYDROPHILII Latreille, 1802. Stem: *Hydrophil*-.

***Hydroporus* Clairville, 1806**: 182. Current status: valid genus in DYTISCIDAE: HYDROPORINAE: HYDROPORINI: HYDROPORINA.

Type species: *Dytiscusfusculus* Schrank, 1781 (= *Dytiscusplanus* Fabricius, 1781), by subsequent designation (Ádám 1996: 20). Note: *Dytiscusplanus* Fabricius, 1781 was placed on the Official List of Specific Names in Zoology (ICZN 1989e).

Family-group name: HYDROPORIDES Aubé, 1836. Stem: *Hydropor*-.

***Hydroscapha* J.L. LeConte, 1874b**: 45. Current status: valid genus in HYDROSCAPHIDAE: HYDROSCAPHINAE.

Type species: *Hydroscaphanatans* J.L. LeConte, 1874, by monotypy.

Family-group name: HYDROSCAPHIDAE J.L. LeConte, 1874. Stem: *Hydroscaph*-.

***Hydrotrupes* Sharp, 1882b**: 491, 492. Current status: valid genus in DYTISCIDAE: AGABINAE: HYDROTRUPINI.

Type species: *Hydrotrupespalpalis* Sharp, 1882, by monotypy.

Family-group name: HYDROTRUPINAE Roughley, 2000. Stem: *Hydrotrup*-.

***Hydrovatus* Motschulsky, 1853c**: 4. Current status: valid genus in DYTISCIDAE: HYDROPORINAE: HYDROVATINI.

Type species: *Hyphydruscuspidatus* Kunze, 1818, by monotypy.

Family-group name: HYDROVATINI Sharp, 1880. Stem: *Hydrovat*-.

***Hygrobia* Latreille, 1804a**: 139. Current status: valid genus in HYGROBIIDAE. Note: new replacement name for *Hydrachna* Fabricius, 1801; this name was originally spelled *Hygriobia* but subsequently emended to *Hygrobia* by the Commission and placed on the Official List of Generic Names in Zoology (ICZN 1954b).

Type species: *Dytiscushermanni* Fabricius, 1775 [as *herrmanni*], by monotypy (see ICZN 1954b). Note: name placed on the Official List of Specific Names in Zoology (ICZN 1954b).

Family-group name: HYGROBIINAE Régimbart, 1879. Stem: *Hygrobi*-.

***Hygronoma* Erichson, 1837**: 312. Current status: valid genus in STAPHYLINIDAE: ALEOCHARINAE: HYGRONOMINI: HYGRONOMINA.

Type species: *Aleocharadimidiata* Gravenhorst, 1806, by monotypy.

Family-group name: HYGRONOMIDES C.G. Thomson, 1859. Stem: *Hygronom*-.

***Hygrotus* Stephens, 1828**: 38, 46. Current status: valid genus in DYTISCIDAE: HYDROPORINAE: HYGROTINI.

Type species: *Dytiscusinaequalis* Fabricius, 1777, by subsequent designation (Curtis 1835: pl. 531).

Family-group name: HYGROTINI Portevin, 1929. Stem: *Hygrot*-.

***Hylaspes* Baly, 1865c**: 436. Current status: junior synonym of *Gallerucida* Motschulsky, 1861 in CHRYSOMELIDAE: GALERUCINAE: HYLASPINI.

Type species: *Hylaspeslongicornis* Baly, 1865, by original designation.

Family-group name: HYLASPITES Chapuis, 1875. Stem: *Hylasp*-.

***Hylastes* Erichson, 1836**: 47. Current status: valid genus in CURCULIONIDAE: SCOLYTINAE: HYLASTINI.

Type species [suggested]: *Bostrichusater* Paykull, 1800, by subsequent designation (Westwood 1838c: 39). Note: as pointed out by Alonso-Zarazaga et al. (2016: 5), “*Bostrichusater* Paykull, 1800” is a misidentification of *Bostrichusater* Fabricius, 1792 (= *Bostrichusbidentatus* Herbst, 1783, currently in the genus *Pityogenes* Bedel, 1888) and is therefore an unavailable name; however, because “*Bostrichusater* Paykull, 1800” is the accepted name of a major pest, we believe that an application be submitted to the Commission to treat Paykull’s name as available and the type species of *Hylastes* Erichson, 1836.

Family-group name: HYLASTES J.L. LeConte, 1876. Stem: *Hylast*-.

***Hylecoetus* Latreille, 1805**: 266. Current status: junior synonym of *Elateroides* Schäffer, 1777 in LYMEXYLIDAE: HYLECOETINAE.

Type species: *Cantharisdermestoides* Linnaeus, 1761, by monotypy.

Family-group name: HYLECOETI Germar, 1818. Stem: *Hylecoet*-.

***Hylepnigalio* Gistel, 1856**: 384. Current status: junior synonym of *Melandrya* Fabricius, 1801 in MELANDRYIDAE: MELANDRYINAE: MELANDRYINI.

Type species: *Chrysomelacaraboides* Linnaeus, 1761, by subsequent designation (Bouchard et al. 2011: 388).

Family-group name: HYLEPNIGALIONIDAE Gistel, 1856. Stem: *Hylepnigalion*-.

***Hylesinus* Fabricius, 1801a**: xii. Current status: valid genus in CURCULIONIDAE: SCOLYTINAE: HYLESININI.

Type species: *Hylesinuscrenatus* Fabricius, 1801, by subsequent designation (Latreille 1810: 431). Note: no species were originally included in this genus by Fabricius (1801a); the first ones were the 30 species listed by Fabricius (1801b: 390–395).

Family-group name: HYLESINEN Erichson, 1836. Stem: *Hylesin*-.

***Hylobius* Germar, 1817b**: 340. Current status: valid genus in CURCULIONIDAE: MOLYTINAE: HYLOBIINI: HYLOBIINA.

Type species: *Curculiopineti* Fabricius, 1792 (= *Curculioexcavatus* Laicharting, 1781), by subsequent designation (Schönherr 1823: 1143).

Family-group name: HYLOBIDAE W. Kirby, 1837. Stem: *Hylobi*-.

***Hylochares* Latreille, 1834**: 127. Current status: valid genus in EUCNEMIDAE: MELASINAE: HYLOCHARINI.

Type species: *Elatercruentatus* Gyllenhal, 1808, by subsequent designation (Lacordaire 1857: 114).

Family-group name: HYLOCHARITES Jacquelin du Val, 1859. Stem: *Hylochar*-.

***Hylocurus* Eichhoff, 1872**: 133. Current status: valid genus in CURCULIONIDAE: SCOLYTINAE: MICRACIDINI.

Type species: *Hylocuruselegans* Eichhoff, 1872, by monotypy.

Family-group name: HYLOCURIDAE Eichhoff, 1878. Stem: *Hylocur*-.

***Hylophilus* Berthold, 1827**: 375. Current status: invalid senior synonym of *Aderus* Stephens, 1829 in ADERIDAE. Note: junior homonym of *Hylophilus* Temminck, 1822 [Aves].

Type species: *Notoxuspopulneus* Panzer, 1796, by original designation.

Family-group name: HYLOPHILIDAE Streubel, 1846. Stem: *Hylophil*-. Note: family-group name permanently invalid (ICZN 1999a, Article 39).

***Hylotorus* Dalman, 1823**: 103. Current status: valid genus in CARABIDAE: PAUSSINAE: PAUSSINI: PAUSSINA.

Type species: *Paussusbucephalus* Gyllenhal, 1817, by monotypy.

Family-group name: HYLOTORINI Luna de Carvalho, 1951. Stem: *Hylotor*-.

***Hylotrupes* Audinet-Serville, 1834b**: 77. Current status: valid genus in CERAMBYCIDAE: CERAMBYCINAE: HYLOTRUPINI.

Type species: *Cerambyxbajulus* Linnaeus, 1758, by monotypy.

Family-group name: HYLOTRUPINI Rose, 1983. Stem: *Hylotrup*-.

***Hylurgops* J.L. LeConte in J.L. LeConte and G.H. Horn, 1876**: 389. Current status: valid genus in CURCULIONIDAE: SCOLYTINAE: HYLASTINI.

Type species: *Hylastespinifex* Fitch, 1858 (= *Hylurgusrugipennis* Mannerheim, 1843), by subsequent designation (Hopkins 1914: 123).

Family-group name: HYLURGOPINA Balachowsky, 1949. Stem: *Hylurgop*-.

***Hylurgus* Latreille, 1806**: 274. Current status: valid genus in CURCULIONIDAE: SCOLYTINAE: HYLURGINI.

Type species: *Bostrichusligniperda* Fabricius, 1787 [as *Bostricilusligniperda*], by monotypy.

Family-group name: HYLURGIDAE Gistel, 1848. Stem: *Hylurg*-.

***Hymaea* Pascoe, 1869a**: 35. Current status: valid genus in PHLOEOSTICHIDAE.

Type species: *Hymaeasuccinifera* Pascoe, 1869, by monotypy.

Family-group name: HYMAEINAE Sen Gupta & Crowson, 1966. Stem: *Hymae*-.

***Hyocis* Pascoe, 1866b**: 457. Current status: valid genus in TENEBRIONIDAE: DIAPERINAE: HYOCIINI: HYOCIINA.

Type species: *Hyocisbakewellii* Pascoe, 1866, by original designation.

Family-group name: HYOCINI G.S. Medvedev & Lawrence, 1982. Stem: *Hyoci*-.

***Hyorrhynchus* W.F.H. Blandford, 1894**: 58. Current status: valid genus in CURCULIONIDAE: SCOLYTINAE: HYORRHYNCHINI.

Type species: *Hyorrhynchuslewisi* W.F.H. Blandford, 1894, by monotypy.

Family-group name: HYORRHYNCHINAE Hopkins, 1915. Stem: *Hyorrhynch*-.

***Hypasclera* Kirsch, 1866**: 210. Current status: valid genus in OEDEMERIDAE: OEDEMERINAE: ASCLERINI.

Type species: *Hypascleraschistacea* Kirsch, 1866, by subsequent designation (Arnett 1950: 221).

Family-group name: HYPASCLERINI Arnett, 1984. Stem: *Hypascler*-.

***Hypera* Germar, 1817b**: 340. Current status: valid genus in CURCULIONIDAE: HYPERINAE: HYPERINI: HYPERINA.

Type species: *Curculionigrirostris* Fabricius, 1775, by subsequent designation (Samouelle 1819: 205).

Family-group name: HYPÉRIDES Lacordaire, 1863. Stem: *Hyper*-.

***Hyperacantha* Chapuis, 1879**: 18. Current status: valid genus in CHRYSOMELIDAE: GALERUCINAE: LUPERINI.

Type species: *Hyperacanthafenestrata* Chapuis, 1879, by subsequent designation (Hincks 1949: 612).

Family-group name: HYPERACANTHITES Laboissière, 1924. Stem: *Hyperacanth*-.

***Hyperaspis* Chevrolat in Dejean, 1836a**: 435. Current status: valid genus in COCCINELLIDAE: COCCINELLINAE: HYPERASPIDINI: HYPERASPIDINA.

Type species: *Coccinellareppensis* Herbst, 1783, by subsequent designation (C.G. Thomson 1859: 161).

Family-group name: HYPÉRASPIENS Mulsant, 1846. Stem: *Hyperaspid*-.

***Hyphalus*Britton, 1971**: 88. Current status: valid genus in LIMNICHIDAE: HYPHALINAE.

Type species: *Hyphalusinsularis*Britton, 1971, by original designation.

Family-group name: HYPHALINAEBritton, 1971. Stem: *Hyphal*-.

***Hyphydrus* Illiger, 1802**: 299. Current status: valid genus in DYTISCIDAE: HYDROPORINAE: HYPHYDRINI.

Type species: *Dytiscusgibbus* Fabricius, 1777 (= *Dytiscusovatus* Linnaeus, 1761), by subsequent designation (Latreille 1810: 426).

Family-group name: HYPHYDRIIDAE Gistel, 1848. Stem: *Hyphydr*-.

***Hypnoidus* Dillwyn, 1829**: 32. Current status: valid genus in ELATERIDAE: HYPNOIDINAE.

Type species: *Elaterriparius* Fabricius, 1792, by subsequent designation (Westwood 1838c: 26).

Family-group name: HYPNOIDINI O. Schwarz, 1906. Stem: *Hypnoid*-.

†***Hypnomorphus* Dolin, 1975**: 54. Current status: valid genus in ELATERIDAE: †PROTAGRYPNINAE: †HYPNOMORPHINI. Note: Jurassic (Kazakhstan).

Type species: †*Hypnomorphusrohdendorfi* Dolin, 1975, by original designation.

Family-group name: †HYPNOMORPHINI Dolin, 1975. Stem: *Hypnomorph*-.

***Hypoborus* Erichson, 1836**: 62. Current status: valid genus in CURCULIONIDAE: SCOLYTINAE: HYPOBORINI: HYPOBORINA.

Type species: *Hypoborusficus* Erichson, 1836, by monotypy.

Family-group name: HYPOBORINAE Nüsslin, 1911. Stem: *Hypobor*-.

***Hypocaccus* C.G. Thomson, 1867**: 400. Current status: valid genus in HISTERIDAE: SAPRININAE: HYPOCACCINI.

Type species: **here fixed** (under ICZN 1999a, Article 70.3.2) as *Histerrugiceps* Duftschmid, 1805, misidentified as *Histerquadristriatus* Thunberg, 1794 (sensu Hoffmann, 1803) in the subsequent designation by Lewis (1899: 3).

Family-group name: HYPOCACCINI Lackner in Lackner et al. 2024: 60. Stem: *Hypocacc*-.

***Hypocephalus* A.G. Desmarest, 1832**: pl. 24. Current status: valid genus in VESPERIDAE: ANOPLODERMATINAE: HYPOCEPHALINI.

Type species: *Hypocephalusarmatus* A.G. Desmarest, 1832, by monotypy.

Family-group name: HYPOCÉPHALIENS Blanchard, 1845. Stem: *Hypocephal*-.

***Hypocoprus* Motschulsky, 1839**: 72. Current status: valid genus in CRYPTOPHAGIDAE: ATOMARIINAE: HYPOCOPRINI.

Type species: *Hypocopruslatridioides* Motschulsky, 1839, by monotypy.

Family-group name: HYPOCOPRINI Reitter, 1879. Stem: *Hypocopr*-.

***Hypocyphtus* Gyllenhal, 1827**: 294. Current status: junior synonym of *Cypha* Leach, 1819 in STAPHYLINIDAE: ALEOCHARINAE: HYPOCYPHTINI.

Type species: *Scaphidiumlongicorne* Paykull, 1800, by monotypy.

Family-group name: HYPOCYPHTIDAE Laporte, 1835. Stem: *Hypocypht*-.

***Hypodacne* J.L. LeConte, 1876**: 170. Current status: valid genus in EUXESTIDAE.

Type species: *Hypodacnepunctata* J.L. LeConte, 1876, by monotypy.

Family-group name: HYPODACNINAE Dajoz, 1976. Stem: *Hypodacn*-.

***Hypodesis* Latreille, 1834**: 156. Current status: valid genus in ELATERIDAE: ELATERINAE: ELATERINI.

Type species: *Hypodesissericea* Latreille, 1834, by monotypy.

Family-group name: HYPODÉSITES Candèze, 1863. Stem: *Hypodese*-.

***Hypohypurus* Hustache, 1920b**: 334. Current status: valid genus in CURCULIONIDAE: CEUTORHYNCHINAE: HYPOHYPURINI.

Type species: *Hypohypurusperrieri* Hustache, 1920, by subsequent designation (Colonelli 1997: 387).

Family-group name: HYPOHYPURINI Colonnelli, 2004. Stem: *Hypohypur*-.

***Hypolithus* Eschscholtz, 1829a**: 33. Current status: valid genus in ELATERIDAE: HYPNOIDINAE. Note: this name, issued by 10 May 1829, is a senior homonym of *Hypolithus* Dejean, 1829 [Coleoptera: CARABIDAE], published by 23 November, 1829.

Type species: *Hypolithuslittoralis* Eschscholtz, 1829, by subsequent designation (Hyslop 1921: 650).

Family-group name: HYPOLITHINAE Fleutiaux, 1928. Stem: *Hypolith*-.

***Hypomelus* Solier, 1843**: 4, 93. Current status: valid genus in TENEBRIONIDAE: PIMELIINAE: SEPIDIINI: HYPOMELINA.

Type species: *Hypomelusbicolor* Solier, 1843 (= *Helopsperonatus* Germar, 1823), by original designation.

Family-group name: HYPOMELINA C. Koch, 1955. Stem: *Hypomel*-.

***Hypophlaeus* Fabricius, 1790**: 222. Current status: junior synonym of *Corticeus* Piller & Mitterpacher, 1783 in TENEBRIONIDAE: DIAPERINAE: HYPOPHLAEINI.

Type species: *Hypophlaeuscastaneus* Fabricius, 1790 (= *Corticeusunicolor* Piller & Mitterpacher, 1783), by subsequent designation (Curtis 1832: pl. 430).

Family-group name: HYPOPHLAEIDES Billberg, 1820. Stem: *Hypophlae*-.

***Hypoprasis* Fairmaire & Germain, 1864**: 260. Current status: valid genus in BUPRESTIDAE: CHRYSOCHROINAE: PARALEPTODEMINI: HYPOPRASINA.

Type species: *Hypoprasisharpagon* Fairmaire & Germain, 1864, by monotypy.

Family-group name: HYPOPRASINA Holyński, 1993. Stem: *Hypopras*-.

***Hypselogenia* Burmeister, 1840b**: [Heft 6]. Current status: valid genus in SCARABAEIDAE: CETONIINAE: GOLIATHINI: GOLIATHINA.

Type species: *Diplognathaconcava* Gory & Percheron, 1833, by subsequent designation (Krikken 1979: 114).

Family-group name: HYPSELOGENIAE Schoch, 1894. Stem: *Hypselogeni*-.

***Hypselomus* Perty, 1832**: 95. Current status: valid genus in CERAMBYCIDAE: LAMIINAE: ONCIDERINI.

Type species: *Hypselomuscristatus* Perty, 1832, by monotypy.

Family-group name: HYPSELOMINAE Pascoe, 1864. Stem: *Hypselom*-.

***Hypsioma* Audinet-Serville, 1835a**: 38. Current status: valid genus in CERAMBYCIDAE: LAMIINAE: ONCIDERINI.

Type species: *Hypsiomagibberum* Audinet-Serville, 1835, by monotypy.

Family-group name: HYPSIOMITAE J. Thomson, 1860. Stem: *Hypsiomat*-.

***Hypsonotus* Germar, 1823**: 367. Current status: valid genus in CURCULIONIDAE: ENTIMINAE: ENTIMITAE: LORDOPINI.

Type species: *Hypsonotusclavulus* Germar, 1823, by subsequent designation (Schönherr 1826: 13).

Family-group name: HYPSONOTIDAE Jekel, 1853. Stem: *Hypsonot*-.

***Hyptioma* Casey, 1906**: 362. Current status: junior synonym of *Holisus* Erichson, 1839 in STAPHYLINIDAE: STAPHYLININAE: STAPHYLININI: HYPTIOMINA.

Type species: *Hyptiomacubensis* Casey, 1906, by monotypy.

Family-group name: HYPTIOMAE Casey, 1906. Stem: *Hyptiom*-.

***Hypulus* Paykull, 1798**: 251. Current status: valid genus in MELANDRYIDAE: MELANDRYINAE: HYPULINI.

Type species: *Elaterquercinus* Quensel, 1790, by subsequent designation (Curtis 1829: pl. 255).

Family-group name: HYPULIDAE Gistel, 1848. Stem: *Hypul*-. Note: junior homonym of HYPULIA Rafinesque, 1815 (type genus *Hypulus* Rafinesque, 1815) in Coleoptera: TENEBRIONIDAE; Rafinesque’s name is permanently invalid (based on a preoccupied type genus).

***Hypulus* Rafinesque, 1815**: 114. Current status: junior synonym of *Helops* Fabricius, 1775 in TENEBRIONIDAE: TENEBRIONINAE: HELOPINI: HELOPINA. Note: unnecessary new replacement name for *Helops* Fabricius, 1775; junior homonym of *Hypulus* Paykull, 1798 [Coleoptera: MELANDRYIDAE].

Type species: *Tenebriocaeruleus* Linnaeus, 1758, by subsequent designation (Hope 1841a: 133) (type fixation for *Helops* Fabricius).

Family-group name: HYPULIA Rafinesque, 1815. Stem: *Hypul*-. Note: family-group name permanently invalid (ICZN 1999a, Article 39).

***Hypurus* Rey, 1882**: 187, 188. Current status: valid genus in CURCULIONIDAE: CEUTORHYNCHINAE: HYPURINI.

Type species: *Ceutorhynchusbertrandi* Perris, 1852, by subsequent designation (K.W. Kirby 1884: 105).

Family-group name: HYPURIDAE Schultze, 1902. Stem: *Hypur*-.

***Iberobaenia* Bocák, Kundrata, Andújar & Vogler, 2016**: 4. Current status: valid genus in IBEROBAENIIDAE. Note: combined description of a new family-group name and a single new genus (ICZN 1999a, Article 13.5).

Type species: *Iberobaeniaminuta* Bocák, Kundrata, Andújar & Vogler, 2016, by original designation.

Family-group name: IBEROBAENIIDAE Bocák, Kundrata, Andújar & Vogler, 2016: 5. Stem: *Iberobaeni*-.

***Ibidion* Audinet-Serville, 1834b**: 103. Current status: invalid senior synonym of *Neoibidion* Monné, 2012 in CERAMBYCIDAE: CERAMBYCINAE: TROPIDINI: NEOIBIDIONINA. Note: junior homonym of *Ibidion* Gory, 1833 [Coleoptera: CERAMBYCIDAE].

Type species: *Ibidioncomatum* Audinet-Serville, 1834, by subsequent designation (J. Thomson 1864: 215).

Family-group name: IBIDIONITAE J. Thomson, 1861. Stem: *Ibidion*-. Note: family-group name permanently invalid (ICZN 1999a, Article 39).

***Ichnea* Laporte, 1838**: 55. Current status: valid genus in CLERIDAE: KORYNETINAE.

Type species: *Ichnealycoides* Laporte, 1838, by monotypy.

Family-group name: ICHNOÏDES Spinola, 1841. Stem: *Ichne*-.

***Ichnestoma* Gory & Percheron, 1833**: 19, 41. Current status: valid genus in SCARABAEIDAE: CETONIINAE: GOLIATHINI: ICHNESTOMATINA.

Type species: *Ichnestomaheteroclyta* Gory & Percheron, 1833, by monotypy. Note: this species is made available by the combined description of a new genus and a single new species (ICZN 1999a, Article 12.2.6); the other species in the genus were described by Gory & Percheron in another livraison of the work issued in 1834.

Family-group name: ISCHNOSTOMIDAE Burmeister, 1842. Stem: *Ichnestomat*-. Note: see Perissinotto (2022: 279) regarding the recommendation to use the incorrectly formed ICHNESTOMINA.

***Ichthyurus* Westwood, 1848a**: 83. Current status: valid genus in CANTHARIDAE: CHAULIOGNATHINAE: ICHTHYURINI.

Type species: *Ichthyuruslateralis* Westwood, 1848, by subsequent designation (Delkeskamp 1977: 468).

Family-group name: ICHTHYURINI Champion, 1915. Stem: *Ichthyur*-. Note: junior homonym of ICHTHYURINAE Packard, 1895 (type genus *Ichthyura* Hübner, 1819) in Lepidoptera.

***Icthyosoma* Boisduval, 1835**: 468. Current status: junior synonym of *Tmesisternus* Latreille, 1829 in CERAMBYCIDAE: LAMIINAE: TMESISTERNINI. Note: this taxon was originally proposed as a synonym of *Tmesisternus* Latreille, 1829 and subsequently treated as valid before 1961 by Montrouzier (1855: 58, 59).

Type species: *Tmesisternusoblongus* Boisduval, 1835, by **present designation**. Note: Bousquet et al. (2009: 39) designated *Icthyosomaarmatum* Montrouzier, 1855 as type species of *Icthyosoma* but that species is not an originally included species.

Family-group name: ICHTHYOSOMITAE J. Thomson, 1864. Stem: *Icthyosomat*-.

***Ictistygna* Pascoe, 1866b**: 491. Current status: valid genus in ANTHICIDAE: EURYGENIINAE: ICTISTYGNINI.

Type species: *Ictistygnaadusta* Pascoe, 1866, by subsequent designation (Borchmann 1936: 537).

Family-group name: ICTISTYGNINAE Borchmann, 1936. Stem: *Ictistygn*-.

***Idacantha* Fairmaire, 1869**: 252. Current status: junior synonym of *Diacantha* Chevrolat, 1836 in CHRYSOMELIDAE: GALERUCINAE: LUPERINI. Note: unnecessary new replacement name for *Diacantha* Chevrolat, 1836.

Type species: *Gallerucaunifasciata* G.-A. Olivier, 1808, by subsequent designation (Brauer 1870: 141).

Family-group name: IDACANTHITES Laboissière, 1921. Stem: *Idacanth*-.

***Ideratus* J. Thomson, 1864**: 183. Current status: valid genus in CERAMBYCIDAE: CERAMBYCINAE: IDERATINI.

Type species: *Ideratuscyanipennis* J. Thomson, 1864, by monotypy.

Family-group name: IDERATINI Martins & Napp, 2009. Stem: *Iderat*-.

***Idiobius* Gistel, 1856**: 383. Current status: junior synonym of *Olibrus* Erichson, 1845 in PHALACRIDAE: PHALACRINAE.

Type species: *Phalacrusflavicornis* Sturm, 1807, by subsequent designation (Pakaluk et al. 1994: 229).

Family-group name: IDIOBIIDAE Gistel, 1856. Stem: *Idiobi*-.

***Idiomacer* Kuschel in Kuschel and Leschen, 2011**: 592. Current status: valid genus in NEMONYCHIDAE: RHINORHYNCHINAE: IDIOMACERINI.

Type species: *Idiomacerbasicornis* Kuschel, 2011, by original designation.

Family-group name: IDIOMACERINAE Legalov, 2011a: 163. Stem: *Idiomacer*-.

***Idiomorphus* Chaudoir, 1847**: 515. Current status: valid genus in CARABIDAE: HARPALINAE: IDIOMORPHINI.

Type species: *Idiomorphusguerinii* Chaudoir, 1847, by monotypy.

Family-group name: IDIOMORPHINAE H.W. Bates, 1891. Stem: *Idiomorph*-.

***Idiostoma* Arrow, 1904**: 740. Current status: invalid senior synonym of *Allidiostoma* Arrow, 1940 in SCARABAEIDAE: ALLIDIOSTOMATINAE. Note: junior homonym of *Idiostoma* Walsingham, 1882 [Lepidoptera].

Type species: *Idiostomarufum* Arrow, 1904, by original designation.

Family-group name: IDIOSTOMINAE Arrow, 1904. Stem: *Idiostomat*-. Note: family-group name permanently invalid (ICZN 1999a, Article 39).

***Idisia* Pascoe, 1866b**: 452. Current status: valid genus in TENEBRIONIDAE: PIMELIINAE: IDISIINI.

Type species: *Idisiaornata* Pascoe, 1866, by monotypy.

Family-group name: IDISIINI G.S. Medvedev, 1973. Stem: *Idisi*-.

***Illops* Erichson, 1840b**: 87. Current status: valid genus in MELYRIDAE: MALACHIINAE: MALACHIINI.

Type species: *Illopscorniculatus* Erichson, 1840, by monotypy.

Family-group name: ILLOPINA Jakobson, 1911. Stem: *Illop*-.

***Ilybius* Erichson, 1832**: 18, 34. Current status: valid genus in DYTISCIDAE: AGABINAE: AGABINI.

Type species: *Dytiscusfenestratus* Fabricius, 1781, by subsequent designation (Westwood 1838c: 8).

Family-group name: ILYBII Acloque, 1895. Stem: *Ilybi*-.

***Imatidium* Fabricius, 1801a**: xvii, 345. Current status: valid genus in CHRYSOMELIDAE: CASSIDINAE: IMATIDIINI.

Type species: *Imatidiumthoracicum* Fabricius, 1801, by subsequent designation (Latreille 1810: 432).

Family-group name: IMATIDIIDAE Hope, 1841. Stem: *Imatidi*-.

***Imirus* Reitter, 1885**: 228. Current status: valid genus in STAPHYLINIDAE: PSELAPHINAE: GONIACERITAE: IMIRINI. Note: new replacement name for *Mirus* Saulcy, 1877.

Type species: *Miruspermirus* Saulcy, 1877, by monotypy (type fixation for *Mirus* Saulcy).

Family-group name: IMIRINI Jeannel, 1949. Stem: *Imir*-.

***Inca* Lepeletier & Audinet-Serville, 1828**: 380. Current status: valid genus in SCARABAEIDAE: CETONIINAE: INCINI.

Type species: *Cetoniaclathrata* G.-A. Olivier, 1792, by subsequent designation (E. Desmarest 1860: 59). Note: the type species usually listed for this genus is *Cetoniaynca* Weber, 1801 (e.g., Siedel et al. 2018: 396) by subsequent designation in Howden (1968: 12), but Demarest’s fixation is older.

Family-group name: INCADAE Burmeister, 1842. Stem: *Inc*-.

***Indoquedius* Blackwelder, 1952**: 199. Current status: valid genus in STAPHYLINIDAE: STAPHYLININAE: STAPHYLININI: INDOQUEDIINA. Note: this name was originally proposed by Cameron (1932: 281) who failed to fix a type species as required for all genus-group names published after 1930 (ICZN 1999a, Article 13.3); Blackwelder (1952: 1999) was the first author to do so.

Type species: *Quediusoculatus* Fauvel, 1895, by original designation.

Family-group name: INDOQUEDIINA Brunke & Solodovnikov in Brunke et al., 2016: 447. Stem: *Indoquedi*-.

***Iniocyphus* Raffray, 1912**: 435. Current status: valid genus in STAPHYLINIDAE: PSELAPHINAE: GONIACERITAE: INIOCYPHINI: INIOCYPHINA.

Type species: *Iniocyphusiheringi* Raffray, 1912, by monotypy.

Family-group name: INIOCYPHINI Park, 1951. Stem: *Iniocyph*-.

***Inopeplus* F. Smith, 1851**: 4. Current status: valid genus in SALPINGIDAE: INOPEPLINAE. Note: new replacement name for *Ino* Laporte, 1835.

Type species: *Inopicta* Laporte, 1835, by monotypy (type fixation for *Ino* Laporte).

Family-group name: INOPEPLINI Grouvelle, 1908. Stem: *Inopepl*-.

***Iphimeis* Baly, 1864c**: 133. Current status: valid genus in CHRYSOMELIDAE: EUMOLPINAE: EUMOLPINI.

Type species: *Iphimeisfulvipes* Baly, 1864, by original designation.

Family-group name: IPHIMÉITES Chapuis, 1874. Stem: *Iphime*-.

***Iphius* Schönherr, 1823**: 1140. Current status: junior synonym of *Alceis* Billberg, 1820 in CURCULIONIDAE: ENTIMINAE: POLYDRUSITAE: NAUPACTINI: NAUPACTINA. Note: unnecessary new replacement name for *Alceis* Billberg, 1820.

Type species: *Curculiolongimanus* Fabricius, 1775 (= *Siderodactylusornatus* Pascoe, 1879), by monotypy (type fixation for *Alceis* Billberg).

Family-group name: IPHIIDES Schönherr, 1823. Stem: *Iphi*-. Note: nomen oblitum (see Bouchard et al. 2011: 611).

***Ips* DeGeer, 1775**: 190, 193. Current status: valid genus in CURCULIONIDAE: SCOLYTINAE: IPINI: IPINA.

Type species: *Dermestestypographus* Linnaeus, 1758, by subsequent designation (Crotch 1870a: 44).

Family-group name: IPINI Bedel, 1888. Stem: *Ip*-.

***Iresia* Dejean in Dejean and Boisduval, 1829**: 5, 8. Current status: valid genus in CICINDELIDAE: CICINDELINI: IRESIINA.

Type species: *Iresialacordairei* Dejean, 1829, by monotypy.

Family-group name: IRESINA Rivalier, 1971. Stem: *Iresi*-.

***Iridotaenia* Deyrolle, 1865**: 25. Current status: valid genus in BUPRESTIDAE: CHRYSOCHROINAE: CHRYSOCHROINI: CHALCOPHORINA. Note: the original spelling was *Iridotoenia* but *Iridotaenia*, an incorrect subsequent spelling, is in prevailing usage, attributed to the publication of the original spelling, and therefore deemed to be the correct original spelling (ICZN 1999a, Article 33.3.1); name placed on the Official List of Generic Names in Zoology (ICZN 2004c).

Type species: *Chrysodemasumptuosa* Laporte & Gory, 1837, by subsequent designation (Kurosawa 1982: 192; see ICZN 2004c). Note: name placed on the Official List of Specific Names in Zoology (ICZN 2004c, *Chrysodemasumptuosa* Laporte & Gory, 1835).

Family-group name: IRIDOTAENINI Tôyama, 1987. Stem: *Iridotaeni*-.

***Isaniris* J. Thomson, 1858b**: 129. Current status: valid genus in CURCULIONIDAE: ENTIMINAE: OTIORHYNCHITAE: ISANIRINI.

Type species: *Isanirisviridimicans* J. Thomson, 1858, by subsequent designation (G.A.K. Marshall 1944: 337).

Family-group name: ISANIRINI Legalov, 2020b: 340. Stem: *Isanir*-.

***Ischalia* Pascoe, 1860b**: 54. Current status: valid genus in ISCHALIIDAE.

Type species: *Ischaliaindigacea* Pascoe, 1860, by monotypy.

Family-group name: ISCHALIINAE Blair, 1920. Stem: *Ischali*-.

***Ischioloncha* J. Thomson, 1860a**: 122. Current status: valid genus in CERAMBYCIDAE: LAMIINAE: APOMECYNINI.

Type species: *Ischiolonchawollastonii* J. Thomson, 1860, by monotypy.

Family-group name: ISCHIOLONCHIDES Lacordaire, 1872. Stem: *Ischiolonch*-.

***Ischiopachys* Chevrolat in Dejean, 1836a**: 416. Current status: valid genus in CHRYSOMELIDAE: CRYPTOCEPHALINAE: CLYTRINI: BABIINA.

Type species: *Clytrabicolor* G.-A. Olivier, 1791, by monotypy.

Family-group name: ISCHIOPACHITES Chapuis, 1874. Stem: *Ischiopache*-.

***Ischnocerus* Schönherr, 1839**: 191. Current status: junior synonym of *Meconemus* Labram & Imhoff, 1838 in ANTHRIBIDAE: ANTHRIBINAE: MECOCERINI.

Type species: *Ischnocerusinfuscatus* Fåhraeus, 1839 (= *Meconemustuberculatus* Labram & Imhoff, 1838), by original designation.

Family-group name: ISCHNOCÉRIDES Lacordaire, 1865. Stem: *Ischnocer*-.

***Ischnomerus* Labram & Imhoff, 1838**: [Heft 2]. Current status: valid genus in BRENTIDAE: BRENTINAE: BRENTINI: ITHYSTENINA.

Type species: *Ischnomeruserythroderes* Labram & Imhoff, 1838, by monotypy.

Family-group name: ISCHNOMERI Kolbe, 1883. Stem: *Ischnomer*-. Note: junior homonym of ISCHNOMÉRIDES Lacordaire, 1865 (type genus *Ischnomerus* Schönherr, 1840) in Coleoptera: BRENTIDAE; Lacordaire’s name is permanently invalid (based on a preoccupied type genus).

***Ischnomerus* Schönherr, 1840a**: 571. Current status: junior synonym of *Aulacoderes* Chevrolat, 1839 in BRENTIDAE: BRENTINAE: TAPHRODERINI. Note: two original spellings were used, including *Ischnocerus* (p. 572); subsequently, Schönherr (1842a: 492) used *Ischnomerus* and therefore acted as First Reviser (ICZN 1999a, Article 24.2.4); junior homonym of *Ischnomerus* Labram & Imhoff, 1838 [Coleoptera: BRENTIDAE].

Type species: *Ischnomeruslinearis* Boheman, 1840 (= *Aulacoderesimmotus* Chevrolat, 1839), by original designation.

Family-group name: ISCHNOMÉRIDES Lacordaire, 1865. Stem: *Ischnomer*-. Note: senior homonym of ISCHNOMERI Kolbe, 1883 (type genus *Ischnomerus* Labram & Imhoff, 1838) in Coleoptera: BRENTIDAE; family-group name permanently invalid (ICZN 1999a, Article 39).

***Ischnoscelis* Burmeister, 1842**: 179. Current status: valid genus in SCARABAEIDAE: CETONIINAE: GOLIATHINI: RHOMBORHININA.

Type species: *Goliathushoepfneri* Gory & Percheron, 1833, by monotypy. Note: the original spelling of the type species was *hoepfner*; however, the incorrect subsequent spelling *hoepfneri* is in prevailing usage, attributed to the publication of the original spelling, and therefore deemed to be the correct original spelling (ICZN 1999a, Article 33.3.1).

Family-group name: ISCHNOSCELI Schoch, 1894. Stem: *Ischnoscelid*-.

***Ischnovalgus* Kolbe, 1897b**: 185, 190. Current status: valid genus in SCARABAEIDAE: CETONIINAE: VALGINI: VALGINA.

Type species: *Ischnovalgusgracilis* Kolbe, 1897, by subsequent designation (Krikken 1978: 158).

Family-group name: ISCHNOVALGINAE Kolbe, 1904. Stem: *Ischnovalg*-.

***Ischyomius* Chevrolat, 1878b**: 98. Current status: valid genus in PYTHIDAE.

Type species: *Ischyomiussingularis* Chevrolat, 1878, by monotypy.

Family-group name: ISCHYOMIIDES Champion, 1886. Stem: *Ischyomi*-.

***Ischyrosonyx* Sturm, 1843**: 273. Current status: junior synonym of *Eurypedus* Gistel, 1834 in CHRYSOMELIDAE: CASSIDINAE: ISCHYROSONYCHINI.

Type species: *Cassidaoblonga* Thon, 1826 (= *Ischyrosonyxthoni* Barber, 1946), by monotypy.

Family-group name: ISCHYROSONYCHITES Chapuis, 1875. Stem: *Ischyrosonych*-.

***Isoclerus* Lewis, 1892**: 191. Current status: valid genus in THANEROCLERIDAE: THANEROCLERINAE: ISOCLERINI.

Type species: *Isocleruspictus* Lewis, 1892, by monotypy.

Family-group name: ISOCLERINA Kolibáč, 1992. Stem: *Isocler*-.

***Isolabus* Voss, 1925**: 214. Current status: valid genus in ATTELABIDAE: ATTELABINAE: ATTELABINI: ISOLABINA.

Type species: *Attelabuscaeruleus* Jekel, 1860 (= *Isolabusjekeli* Legalov, 2002), by original designation.

Family-group name: ISOLABINA Legalov, 2007. Stem: *Isolab*-.

***Isonychus* Mannerheim, 1828**: 69. Current status: valid genus in SCARABAEIDAE: MELOLONTHINAE: MACRODACTYLINI.

Type species: *Isonychussulphureus* Mannerheim, 1828, by original designation.

Family-group name: ISONYCHIDAE Burmeister, 1855. Stem: *Isonych*-.

***Isopleurus* W. Kirby, 1837**: 49. Current status: junior synonym of *Celia* C. Zimmermann, 1832 in CARABIDAE: HARPALINAE: ZABRINI: AMARINA.

Type species: *Isopleurusnitidus* W. Kirby, 1837 (= *Celiasinuosa* Casey, 1918), by monotypy.

Family-group name: ISOPLEURIDAE W. Kirby, 1837. Stem: *Isopleur*-.

***Isoplia* Burmeister, 1855**: 487. Current status: valid genus in SCARABAEIDAE: RUTELINAE: ANOMALINI: ISOPLIINA.

Type species: *Isoplialasiosoma* Burmeister, 1855, by monotypy.

Family-group name: ISOPLIINI Péringuey, 1902. Stem: *Isopli*-.

***Isopterus* Faust, 1895**: 4. Current status: valid genus in CURCULIONIDAE: ENTIMINAE: OTIORHYNCHITAE: CELEUTHETINI: ISOPTERINA.

Type species: *Isopterussignatus* Faust, 1895, by subsequent designation (Yunakov 2013: 65).

Family-group name: ISOPTERINA Morimoto & Kojima, 2001. Stem: *Isopter*-.

***Isorhynchus* Schönherr, 1833**: 22. Current status: valid genus in CURCULIONIDAE: CEUTORHYNCHINAE: CEUTORHYNCHINI.

Type species: *Curculiopudicus* Sparrman, 1785, by original designation.

Family-group name: ISORHYNCHIDES Lacordaire, 1865. Stem: *Isorhynch*-.

†***Isothea* Scudder, 1893**: 20. Current status: valid genus in ATTELABIDAE: RHYNCHITINAE: RHYNCHITITAE: RHYNCHITINI: RHYNCHITINA. Note: Oligocene (USA).

Type species: †*Isotheaalleni* Scudder,1893, by monotypy.

Family-group name: †ISOTHEINAE Scudder, 1893. Stem: *Isothe*-.

***Istrisia* Lewis, 1895a**: 254. Current status: valid genus in SALPINGIDAE: SALPINGINAE.

Type species: *Istrisiarufobrunnea* Lewis, 1895, by monotypy.

Family-group name: ISTRISIINI Nikitsky, 1992. Stem: *Istrisi*-.

***Ita* Tournier, 1878**: xviii. Current status: valid genus in CURCULIONIDAE: MOLYTINAE: ITINI.

Type species: *Itacrassirostris* Tournier, 1878, by subsequent designation (Alonso-Zarazaga and Lyal 1999: 21, 79).

Family-group name: ITINI Reitter, 1913. Stem: *It*-. Note: senior homonym of ITINI Lepesme, 1943 (type genus *Ites* C.O. Waterhouse, 1880) in Coleoptera: CERAMBYCIDAE.

***Italodytes* G. Müller, 1938**: 136, 139. Current status: valid genus in CARABIDAE: SCARITINAE: CLIVININI: CLIVININA.

Type species: *Italodytesstammeri* G. Müller, 1938, by original designation.

Family-group name: ITALODYTINA Jeannel, 1957. Stem: *Italodyt*-.

***Ites* C.O. Waterhouse, 1880b**: 297. Current status: valid genus in CERAMBYCIDAE: LAMIINAE: HEMILOPHINI.

Type species: *Itesplagiatus* C.O. Waterhouse, 1880, by monotypy.

Family-group name: ITESINI Lepesme, 1943. Stem: *It*-. Note: junior homonym of ITINI Reitter, 1913 (type genus *Ita* Tournier, 1878) in Coleoptera: CURCULIONIDAE.

***Ithaura* Pascoe, 1871b**: 215. Current status: valid genus in CURCULIONIDAE: MOLYTINAE: LYMANTINI: LYMANTINA.

Type species: *Ithaurastrangulata* Pacoe, 1871, by monotypy.

Family-group name: ITHAURINAE Kuschel, 1959. Stem: *Ithaur*-.

***Ithycerus* Schönherr, 1823**: 1136. Current status: valid genus in BRENTIDAE: ITHYCERINAE: ITHYCERINI.

Type species: *Rhynchitescurculionoides* Herbst, 1797 (= *Curculionoveboracensis* Forster, 1771), by original designation.

Family-group name: ITHYCERIDES Schönherr, 1823. Stem: *Ithycer*-.

***Ithyporus* Schönherr, 1836**: 550. Current status: valid genus in CURCULIONIDAE: MOLYTINAE: ITHYPORINI: ITHYPORINA.

Type species: *Ithyporuscapensis* Boheman, 1836 (= *Curculiostolidus* Fabricius, 1781), by original designation.

Family-group name: ITHYPORIDES Lacordaire, 1865. Stem: *Ithypor*-.

***Ithystenus* Pascoe, 1862**: 390. Current status: valid genus in BRENTIDAE: BRENTINAE: BRENTINI: ITHYSTENINA. Note: new replacement name for *Leptorhynchus* Boisduval, 1835.

Type species: *Brenthusangustatus* Guérin-Méneville, 1831, by monotypy (type fixation for *Leptorhynchus* Boisduval).

Family-group name: ITHYSTÉNIDES Lacordaire, 1865. Stem: *Ithysten*-.

***Ivieolus* Howden & Gill, 1988**: 2077. Current status: valid genus in HYBOSORIDAE: CERATOCANTHINAE: SCARABATERMITINI.

Type species: *Ivieoluspseudoscutellatus* Howden & Gill, 1988, by original designation.

Family-group name: IVIEOLINI Howden & Gill, 2000. Stem: *Ivieol*-.

***Ixalma* Pascoe, 1871b**: 214. Current status: valid genus in CURCULIONIDAE: CURCULIONINAE: RHAMPHINI: IXALMINA.

Type species: *Ixalmarufescens* Pascoe, 1871, by monotypy.

Family-group name: IXALMINA Voss, 1936. Stem: *Ixalm*-.

***Ixapion* Roudier & Tempère, 1973**: 80. Current status: valid genus in BRENTIDAE: APIONINAE: APIONITAE: IXAPIINI. Note: new replacement name for *Ixias* Sainte Claire-Deville, 1924.

Type species: *Apionvariegatum* Wencker, 1864, by monotypy (type fixation for *Ixias* Sainte Claire-Deville).

Family-group name: IXAPIINI Alonso-Zarazaga, 1990. Stem: *Ixapi*-.

***Jacobsonium* Heller, 1926**: 127. Current status: junior synonym of *Sarothrias* Grouvelle, 1918 in JACOBSONIIDAE.

Type species: *Jacobsoniumdimerum* Heller, 1926, by original designation.

Family-group name: JACOBSONIIDAE Heller, 1926. Stem: *Jacobsoni*-.

***Jamwonus* Harold, 1879**: 158. Current status: valid genus in CERAMBYCIDAE: PRIONINAE: AEGOSOMATINI.

Type species: *Jamwonussubcostatus* Harold, 1879, by monotypy.

Family-group name: JAMWONINAE Kolbe, 1897. Stem: *Jamwon*-.

***Janssenius* Luna de Carvalho, 1951**: 19. Current status: junior synonym of *Heteropaussus* J. Thomson, 1860 in CARABIDAE: PAUSSINAE: PAUSSINI: HETEROPAUSSINA. Note: new replacement name for *Pleuropterus* Westwood, 1841.

Type species: *Cerapteruswestermanni* Westwood, 1840, by monotypy (type fixation for *Pleuropterus* Westwood).

Family-group name: JANSSENINI Luna de Carvalho, 1951. Stem: *Jansseni*-.

***Janusculus* Cerruti, 1970**: 117. Current status: valid genus in STAPHYLINIDAE: PSELAPHINAE: PSELAPHITAE: TYRINI: JANUSCULINA.

Type species: *Janusculusleleupi* Cerruti, 1970, by monotypy.

Family-group name: JANUSCULINA Cerruti, 1970. Stem: *Januscul*-.

***Jenibuntor* Muona, 1993**: 21. Current status: valid genus in EUCNEMIDAE: MACRAULACINAE: JENIBUNTORINI.

Type species: *Jenibuntortabang* Muona, 1993, by original designation.

Family-group name: JENIBUNTORINI Muona, 1993. Stem: *Jenibuntor*-.

***Jordanthribus* Zimmerman, 1938**: 236. Current status: valid genus in ANTHRIBIDAE: ANTHRIBINAE: PROSCOPORHININI.

Type species: *Jordanthribusplanifacietus* Zimmerman, 1938, by original designation.

Family-group name: JORDANTHRIBINI Morimoto, 1980. Stem: *Jordanthrib*-.

***Juanorhinus* Aurivillius, 1926**: 466. Current status: valid genus in CURCULIONIDAE: MOLYTINAE: JUANORHININI.

Type species: *Juanorhinusrobinsoni* Aurivillius, 1926, by monotypy.

Family-group name: JUANORHININI Aurivillius, 1931. Stem: *Juanorhin*-.

***Jubus* L.W. Schaufuss, 1872**: 247. Current status: valid genus in STAPHYLINIDAE: PSELAPHINAE: EUPLECTITAE: JUBINI.

Type species: *Jubusspinicollis* L.W. Schaufuss, 1877, by subsequent designation (R. Lucas 1920: 355). Note: no species were originally included in this genus by L.W. Schaufuss (1872); the first ones were the four species listed by L.W. Schaufuss (1877b: 455–456).

Family-group name: JUBINI Raffray, 1898. Stem: *Jub*-.

***Julodimorpha* Harold in Gemminger and Harold, 1869b**: 1397. Current status: valid genus in BUPRESTIDAE: BUPRESTINAE: JULODIMORPHINI.

Type species: *Stigmoderabakewellii* A. White, 1859, by monotypy.

Family-group name: JULODIMORPHITES Kerremans, 1903. Stem: *Julodimorph*-.

***Julodis* Eschscholtz, 1829b**: 9. Current status: valid genus in BUPRESTIDAE: JULODINAE. Note: the original spelling was *Jalodis* but *Julodis*, an unjustified emendation by von Mannerheim (1837: 11), is in prevailing usage, attributed to the original author and date, and therefore deemed to be a justified emendation and the correct original spelling (ICZN 1999a, Article 33.2.3.1).

Type species: *Buprestisfascicularis* Linnaeus, 1758, by subsequent designation (Gussmann 1997: 338).

Family-group name: JULODIDES Lacordaire, 1857. Stem: *Julod*-.

***Jumnos* Saunders, 1839**: 176. Current status: valid genus in SCARABAEIDAE: CETONIINAE: GOLIATHINI: RHOMBORHININA.

Type species: *Jumnosruckeri* Saunders, 1839, by monotypy.

Family-group name: JUMNIDAE Burmeister, 1842. Stem: *Jumn*-.

†***Juranthribus* Legalov, 2011b**: 35. Current status: valid genus in NEMONYCHIDAE: †BRENTHORRHININAE: †JURANTHRIBINI. Note: Upper Jurassic (Karatau).

Type species: †*Juranthribusthompsoni* Legalov, 2011, by original designation.

Family-group name: †JURANTHRIBINAE Legalov, 2011b: 34. Stem: *Juranthrib*-.

***Jurasai* Rosa, C. Costa, Kramp & Kundrata, 2020b**: [1]. Current status: valid genus in JURASAIDAE. Note: this genus-group name was first proposed by Rosa et al. (2020a: [4]) but the work was not available nomenclaturally because the electronic-only version was not registered in ZooBank before it was published (ICZN 2012f, Article 8.5.3).

Type species: *Jurasaiitajubense* Rosa, C. Costa, Kramp & Kundrata, 2020, by original designation.

Family-group name: JURASAIDAE Rosa, C. Costa, Kramp & Kundrata, 2020b: [3]. Stem: *Jurasa*-. Note: this family-group name was first proposed by Rosa et al. (2020a: [7]) but the work was not available nomenclaturally because the electronic-only version was not registered in ZooBank before it was published (ICZN 2012f, Article 8.5.3).

†***Jurodes* Ponomarenko, 1985**: 54. Current status: valid genus in JURODIDAE. Note: Middle/Upper Jurassic (Russia; China; Mongolia).

Type species: †*Jurodesignoramus* Ponomarenko, 1985, by monotypy.

Family-group name: †JURODIDAE Ponomarenko, 1985. Stem: *Jurod*-.

***Kalcapion* Schilsky, 1906**: iv. Current status: valid genus in BRENTIDAE: APIONINAE: ASPIDAPIITAE: KALCAPIINI.

Type species: *Apionpallipes* W. Kirby, 1808, by original designation.

Family-group name: KALCAPIINI Alonso-Zarazaga, 1990. Stem: *Kalcapi*-.

†***Kaltanocoleus* Rohdendorf, 1961**: 397. Current status: valid genus in †PERMOCUPEDIDAE: †PERMOCUPEDINAE. Note: lower Permian (Russia).

Type species: †*Kaltanocoleuspospelovi* Rohdendorf, 1961, by original designation.

Family-group name: †KALTANOCOLEIDAE Rohdendorf, 1961. Stem: *Kaltanocole*-.

†***Kararhynchus* Zherikhin & Gratshev, 1994**: 60. Current status: valid genus in †OBRIENIIDAE: KARARHYNCHINAE: †KARARHYNCHINI. Note: Upper Jurassic (Kazakhstan).

Type species: †*Kararhynchusocciduus* Zherikhin & Gratshev, 1994, by original designation.

Family-group name: †KARARHYNCHINAE Zherikhin & Gratshev, 1994. Stem: *Kararhynch*-.

†***Karataucar* Legalov, 2009a**: 286, 288. Current status: junior synonym of *Ampliceps* Arnoldi, 1977 in NEMONYCHIDAE: †EOBELINAE: †KARATAUCARINI. Note: Upper Jurassic (Kazakhstan).

Type species: †*Oxycorynoidesprogressivus* Arnoldi, 1977, by original designation.

Family-group name: †KARATAUCARINI Legalov, 2009. Stem: *Karataucar*-.

***Karumia* Escalera, 1913**: 320. Current status: valid genus in DASCILLIDAE: KARUMIINAE: KARUMIINI.

Type species: *Karumiaestafilinoides* Escalera, 1913, by subsequent designation (R. Lucas 1920: 356).

Family-group name: KARUMINAE Escalera, 1913. Stem: *Karumi*-.

***Kateretes* Herbst, 1793**: 11. Current status: valid genus in KATERETIDAE: KATERETINAE. Note: name placed on the Official List of Generic Names in Zoology (ICZN 1999b).

Type species: *Dermestespedicularius* Linnaeus, 1758, by subsequent designation (Hope 1841a: 155; see ICZN 1999b). Note: name placed on the Official List of Specific Names in Zoology (ICZN 1999b).

Family-group name: CATHERETIDAE W. Kirby, 1837. Stem: *Kateret*-.

***Kaupiolus* Zang, 1903**: 418. Current status: junior synonym of *Labienus* Kaup, 1871 in PASSALIDAE: PASSALINAE: MACROLININI. Note: new replacement name for *Vellejus* Kaup, 1871.

Type species: *Passaluscompergus* Boisduval, 1835, by subsequent designation (Hincks and Dibb 1935: 93).

Family-group name: KAUPIOLINAE Zang, 1905. Stem: *Kaupiol*-.

†***Kenderlyka* Zherikhin & Gratshev, 1994**: 59. Current status: valid genus in †OBRIENIIDAE: †KARARHYNCHINAE: †KENDERLYKANINI. Note: Upper Triassic (Kazakhstan).

Type species: †*Kenderlykaconsobrina* Zherikhin & Gratshev, 1994, by original designation.

Family-group name: †KENDERLYKANINI Legalov, 2009. Stem: *Kenderlykan*- (ICZN 1999a, Article 29.4).

***Kisanthobia* Marseul, 1865**: 195, 200. Current status: valid genus in BUPRESTIDAE: BUPRESTINAE: KISANTHOBIINI.

Type species: *Anthaxiaariasi* Robert, 1858, by monotypy.

Family-group name: KISANTHOBIINI Richter, 1949. Stem: *Kisanthobi*-.

***Klewaria* Reitter, 1910**: 20. Current status: valid genus in TENEBRIONIDAE: PIMELIINAE: KLEWARIINI.

Type species: *Klewariacolydiiformis* Reitter, 1910, by monotypy.

Family-group name: KLEWARIINAE Gebien, 1910. Stem: *Klewari*-.

***Korynetes* Herbst, 1792**: 148. Current status: valid genus in CLERIDAE: KORYNETINAE. Note: name placed on the Official List of Generic Names in Zoology (ICZN 1961c).

Type species: *Cleruscaeruleus* DeGeer, 1775, by subsequent designation (Lacordaire 1857: 489; see ICZN 1961c). Note: name placed on the Official List of Specific Names in Zoology (ICZN 1961c).

Family-group name: CORYNETIDAE Laporte, 1838. Stem: *Korynet*-.

†***Kresnikus* Tihelka, D. Huang & Cai, 2019b**: 507. Current status: valid genus in TROGIDAE: †KRESNIKINAE. Note: mid-Cretaceous (Myanmar).

Type species: †*Kresnikusbeynoni* Tihelka, D. Huang & Cai, 2019, by original designation.

Family-group name: †KRESNIKINAE Tihelka, D. Huang & Cai, 2019: 507. Stem: *Kresnik*-.

†***Kryzhanovskiana* Kataev & Kirejtshuk in Kataev et al. 2019**: 2. Current status: valid genus in CARABIDAE: PAUSSINAE: KRYZHANOVSKIANINI. Note: mid-Cretaceous (Myanmar).

Type species: †*Kryzhanovskianaolegi* Kataev & Kirejtshuk, 2019, by original designation.

Family-group name: †KRYZHANOVSKIANINAE Deuve, 2020: 203. Stem: *Kryzhanovskian*-.

***Kuhitangia* G.S. Medvedev, 1962**: 1184. Current status: valid genus in TENEBRIONIDAE: KUHITANGIINAE: KUHITANGIINI.

Type species: *Kuhitangiakryzhanovskii* G.S. Medvedev, 1962, by original designation.

Family-group name: KUHITANGIINAE G.S. Medvedev, 1962. Stem: *Kuhitangi*-.

***Kuschelinus* Straneo, 1963**: 124. Current status: valid genus in CARABIDAE: HARPALINAE: PTEROSTICHINI: METIINA.

Type species: *Kuschelinusinsularis* Straneo, 1963, by original designation.

Family-group name: KUSCHELININI Jeannel, 1967. Stem: *Kuschelin*-.

†***Kuschelomacer* Riedel, 2010**: 31. Current status: valid genus in CIMBERIDIDAE: †KUSCHELOMACERINI. Note: Eocene (Baltic amber).

Type species: †*Kuschelomacerkerneggeri* Riedel, 2010, by original designation.

Family-group name: †KUSCHELOMACERINI Riedel, 2010. Stem: *Kuschelomacer*- (ICZN 1999a, Article 29.4).

***Kytorhinus* Fischer, 1809**: 298, 300. Current status: valid genus in CHRYSOMELIDAE: BRUCHINAE: KYTORHININI. Note: two original spellings were used, including *Kytorhynus* (p. 298); subsequently, Fischer (1821b: 73) used only *Kytorhinus* and therefore acted as First Reviser (ICZN 1999a, Article 24.2.4).

Type species: *Kytorhinuskarasini* Fischer, 1809, by subsequent designation (Crotch 1870b: 222).

Family-group name: KYTORHININAE Bridwell, 1932. Stem: *Kytorhin*-.

***Labaninus* Morimoto, 1981**: 110. Current status: valid genus in CURCULIONIDAE: CURCULIONINAE: CURCULIONINI: LABANININA.

Type species: *Curponinusplicatulus* Heller, 1925, by original designation.

Family-group name: LABANININA Pelsue & O’Brien, 2011: 30. Stem: *Labanin*-.

***Labidopullus* Borgmeier, 1958**: 240. Current status: valid genus in STAPHYLINIDAE: ALEOCHARINAE: MIMECITINI: LABIDOPULLINA.

Type species: *Labidopullusappendiculatus* Borgmeier, 1958, by original designation.

Family-group name: LABIDOPULLINA Jacobson & Kistner, 1991. Stem: *Labidopull*-.

†***Labradorocoleus* Ponomarenko, 1969b**: 309. Current status: valid genus in †LABRADOROCOLEIDAE. Note: Upper Cretaceous (Labrador, Canada).

Type species: †*Labradorocoleuscarpenteri* Ponomarenko, 1969, by original designation.

Family-group name: †LABRADOROCOLEIDAE Ponomarenko, 1969. Stem: *Labradorocole*-.

***Laccobius* Erichson, 1837**: 202. Current status: valid genus in HYDROPHILIDAE: HYDROPHILINAE: LACCOBIINI.

Type species: *Chrysomelaminuta* Linnaeus, 1758, by subsequent designation (Drapiez 1839: 222).

Family-group name: LACCOBIINI Houlbert, 1922. Stem: *Laccobi*-.

***Lacconectus* Motschulsky, 1855b**: 83. Current status: valid genus in DYTISCIDAE: COPELATINAE.

Type species: *Lacconectusfulvescens* Motschulsky, 1855, by monotypy.

Family-group name: LACCONECTINI Branden, 1884. Stem: *Lacconect*-.

***Lacconotus* J.L. LeConte, 1862**: 255. Current status: valid genus in MYCTERIDAE: EURYPINAE.

Type species: *Lacconotuspunctatus* J.L. LeConte, 1862, by monotypy.

Family-group name: LACCONOTINI J.L. LeConte, 1862. Stem: *Lacconot*-.

***Laccophilus* Leach, 1817**: 69, 72. Current status: valid genus in DYTISCIDAE: LACCOPHILINAE: LACCOPHILINI.

Type species: *Dytiscusminutus* Linnaeus, 1758, by monotypy.

Family-group name: LACCOPHILIDAE Gistel, 1848. Stem: *Laccophil*-.

***Laccornellus* Roughley and Wolfe, 1987**: 1347. Current status: valid genus in DYTISCIDAE: HYDROPORINAE: LACCORNELLINI.

Type species: *Hydroporuslugubri*s Aubé, 1838, by original designation.

Family-group name: LACCORNELLINI K.B. Miller & Bergsten, 2014: 139. Stem: *Laccornell*-.

***Laccornis* Gozis, 1914**: 111. Current status: valid genus in DYTISCIDAE: HYDROPORINAE: LACCORNINI.

Type species: *Hydroporusoblongus* Stephens, 1835, by monotypy.

Family-group name: LACCORNINI Wolfe & Roughley, 1990. Stem: *Laccorn*-.

***Laches* Kaup, 1871**: 48. Current status: junior synonym of *Episphenus* Kaup, 1871 in PASSALIDAE: PASSALINAE: MACROLININI. Note: junior homonym of *Laches* Gistel, 1848 [Hymenoptera] and *Laches* Thorell, 1869 [Arachnida]; the First Reviser (ICZN 1999a, Article 24.2.2) for *Laches* Kaup, 1871 versus *Episphenus* Kaup, 1871 is Gravely (1914: 281).

Type species: *Aceraiuscomptoni* Kaup, 1868, by subsequent designation (Hincks and Dibb 1935: 77).

Family-group name: LACHINAE Kuwert, 1896. Stem: *Lach*-. Note: family-group name permanently invalid (ICZN 1999a, Article 39).

***Lachna* Billberg, 1820**: 35. Current status: junior synonym of *Lagria* Fabricius, 1775 in TENEBRIONIDAE: LAGRIINAE: LAGRIINI: LAGRIINA.

Type species: *Chrysomelahirta* Linnaeus, 1758, by subsequent designation (Merkl 2006: 223).

Family-group name: LACHNAEDES Billberg, 1820. Stem: *Lachn*-. Note: nomen oblitum (see Favret and Bouchard 2016: 647); senior homonym of LACHNIDAE Herrich- Schäffer, 1854 (type genus *Lachnus* Burmeister 1835) in Hemiptera.

***Lachnodactylus* Seidlitz, 1898**: 837, 838. Current status: valid genus in TENEBRIONIDAE: PIMELIINAE: LACHNOGYINI: LACHNODACTYLINA. Note: new replacement name for *Lachnopus* Seidlitz, 1894.

Type species: *Lachnopusdigitalis* Seidlitz, 1894, by monotypy (type fixation for *Lachnopus* Seidlitz).

Family-group name: LACHNODACTYLINA Reitter, 1904. Stem: *Lachnodactyl*-.

***Lachnogya* Ménétriés, 1849b**: 228. Current status: valid genus in TENEBRIONIDAE: PIMELIINAE: LACHNOGYINI: LACHNOGYINA.

Type species: *Lachnogyasquamosa* Ménétriés, 1849, by monotypy.

Family-group name: LACHNOGYINI Seidlitz, 1894. Stem: *Lachnogy*-.

***Lachnophorus* Dejean, 1831**: 2, 28. Current status: valid genus in CARABIDAE: HARPALINAE: LACHNOPHORINI.

Type species: *Lachnophoruspilosus* Dejean, 1831, by subsequent designation (E. Desmarest 1851: 189).

Family-group name: LACHNOPHORI J.L. LeConte, 1853. Stem: *Lachnophor*-.

***Lacon* Laporte, 1838**: 11. Current status: valid genus in ELATERIDAE: AGRYPNINAE: AGRYPNINI.

Type species: *Elateratomarius* Fabricius, 1798 (= *Elaterpunctatus* Herbst, 1779), by subsequent designation (Hyslop 1921: 652).

Family-group name: LACONINI Dajoz, 1964. Stem: *Lacon*-.

***Lactica* Erichson, 1847c**: 173. Current status: junior synonym of *Monomacra* Chevrolat, 1836 in CHRYSOMELIDAE: GALERUCINAE: ALTICINI.

Type species: *Lacticamelaleuca* Erichson, 1847, by monotypy.

Family-group name: LACTICITES Chapuis, 1875. Stem: *Lactic*-.

***Laemophloeus* Dejean, 1835**: 315. Current status: valid genus in LAEMOPHLOEIDAE.

Type species: *Cucujusmuticus* Fabricius, 1781, by subsequent designation (C.G. Thomson 1859: 83).

Family-group name: LAEMOPHLOEINI Ganglbauer, 1899. Stem: *Laemophloe*-.

***Laemosaccus* Schönherr, 1823**: 1136. Current status: valid genus in CURCULIONIDAE: MESOPTILIINAE: LAEMOSACCINI.

Type species: *Curculioplagiatus* Fabricius, 1798 (= *Curculionephele* Herbst, 1797), by original designation.

Family-group name: LÉMOSACIDES Lacordaire, 1865. Stem: *Laemosacc*-.

***Laemotmetus* Gerstaecker, 1871**: 45. Current status: junior synonym of *Aulonosoma* Motschulsky, 1858 in PASSANDRIDAE: PASSANDRINAE.

Type species: *Laemotmetusferrugineus* Gerstaecker, 1871 (= *Aulonosomatenebrioides* Motschulsky, 1858), by monotypy.

Family-group name: LAEMOTMETINI Kessel, 1921. Stem: *Laemotmet*-.

***Laena* Dejean, 1821**: 64. Current status: valid genus in TENEBRIONIDAE: LAGRIINAE: LAENINI.

Type species: fixed by Bouchard et al. (2021: 225) according to Article 70.3.2 (ICZN 1999a) as *Scaurusviennensis* Sturm, 1807, the taxonomic species involved in the misidentification of *Helopspimelia* Fabricius, 1787 (sensu Dejean, 1821), as originally designated by monotypy by Dejean (1821: 64). Note: see Bouchard and Bousquet (2020b: 7) for comments regarding the identity of the type species.

Family-group name: LAENINA Seidlitz, 1895. Stem: *Laen*-.

***Lagenoderus* A. White, 1841**: 181. Current status: valid genus in ATTELABIDAE: ATTELABINAE: ATTELABINI: LAGENODERINA.

Type species: *Attelabusgnomoides* A. White, 1841, by monotypy.

Family-group name: LAGENODERINA Voss, 1925. Stem: *Lagenoder*-.

***Lagocheirus* Dejean, 1835**: 336. Current status: valid genus in CERAMBYCIDAE: LAMIINAE: ACANTHOCININI.

Type species: *Cerambyxaraneiformis* Linnaeus, 1767, by monotypy.

Family-group name: LAGOCHEIRINAE H.W. Bates, 1863. Stem: *Lagocheir*-.

***Lagria* Fabricius, 1775**: 124. Current status: valid genus in TENEBRIONIDAE: LAGRIINAE: LAGRIINI: LAGRIINA.

Type species: *Chrysomelahirta* Linnaeus, 1758, by subsequent designation (Latreille 1810: 429).

Family-group name: LAGRIARIAE Latreille, 1825. Stem: *Lagri*-.

***Lagrioida* Fairmaire & Germain, 1860a**: 3. Current status: valid genus in LAGRIOIDIDAE.

Type species: *Lagrioidarufula* Fairmaire & Germain, 1860, by subsequent designation (Spilman 1954: 89).

Family-group name: LAGRIOIDINI M. Abdullah & A. Abdullah, 1968. Stem: *Lagrioid*-.

***Laius* Guérin-Méneville, 1830**: pl. 2. Current status: valid genus in MELYRIDAE: MALACHIINAE: MALACHIINI.

Type species: *Laiuscyaneus* Guérin-Méneville, 1830, by monotypy.

Family-group name: LAIINA Jakobson, 1911. Stem: *Lai*-.

***Lamia* Fabricius, 1775**: 170. Current status: valid genus in CERAMBYCIDAE: LAMIINAE: LAMIINI.

Type species: *Cerambyxtextor* Linnaeus, 1758, by subsequent designation (Latreille 1810: 431).

Family-group name: LAMIARIAE Latreille, 1825. Stem: *Lami*-.

***Lamingtonium* Sen Gupta & Crowson, 1969**: 125. Current status: valid genus in LAMINGTONIIDAE.

Type species: *Lamingtoniumbinnaburrense* Sen Gupta & Crowson, 1969, by original designation.

Family-group name: LAMINGTONIIDAE Sen Gupta & Crowson, 1969. Stem: *Lamingtoni*-.

***Lamprias* Bonelli, 1810**: Tab. Syn. Current status: valid subgenus of *Lebia* Latreille, 1802 in CARABIDAE: HARPALINAE: LEBIINI: LEBIINA.

Type species: *Carabuscyanocephalus* Linnaeus, 1758, by subsequent monotypy (Panzer 1813: 71).

Family-group name: LAMPRIADAE Chaudoir, 1871. Stem: *Lampri*-.

***Lamprigera* Motschulsky, 1853a**: 47. Current status: valid genus in LAMPYRIDAE incertae sedis.

Type species: *Lamprigeraboyei* Motschulsky, 1853, by original designation.

Family-group name: LAMPRIGERINA McDermott, 1964. Stem: *Lampriger*-.

***Lamprima* Latreille, 1804a**: 150. Current status: valid genus in LUCANIDAE: LAMPRIMINAE: LAMPRIMINI.

Type species: *Lethrusaeneus* Fabricius, 1792, by monotypy.

Family-group name: LAMPRIMIDAE W.S. MacLeay, 1819. Stem: *Lamprim*-.

***Lamprocera* Laporte, 1833c**: 129. Current status: valid genus in LAMPYRIDAE: LAMPYRINAE: LAMPROCERINI.

Type species: *Homalisusgrandis* Sturm, 1826, by monotypy.

Family-group name: LAMPROCERINI E. Olivier, 1907. Stem: *Lamprocer*-.

***Lamprocheila* Saunders, 1871**: 15. Current status: valid genus in BUPRESTIDAE: BUPRESTINAE: BUPRESTINI: LAMPROCHEILINA.

Type species: *Chrysodemamaillei* Laporte & Gory, 1837, by monotypy.

Family-group name: LAMPROCHEILINA Holyński, 1993. Stem: *Lamprocheil*-.

***Lamprohiza* Motschulsky, 1853a**: 47. Current status: valid genus in LAMPYRIDAE: LAMPROHIZINAE.

Type species: *Lampyrissplendidula* Linnaeus, 1767, by original designation.

Family-group name: LAMPROHIZINI Kazantsev, 2010. Stem: *Lamprohiz*-.

***Lamprolabus* Jekel, 1860**: 189. Current status: valid genus in ATTELABIDAE: ATTELABINAE: ATTELABINI: LAMPROLABINA.

Type species: *Attelabusbispinosus* Gyllenhal, 1833, by original designation.

Family-group name: LAMPROLABINA Voss, 1925. Stem: *Lamprolab*-.

***Lampropepla* Fairmaire, 1904a**: 231. Current status: junior synonym of *Madecassia* Kerremans, 1903 in BUPRESTIDAE: CHRYSOCHROINAE: CHRYSOCHROINI: CHALCOPHORINA. Note: unnecessary new replacement name for *Madecassia* Kerremans, 1903.

Type species: *Chalcophoropsisrothschildi* Gahan, 1893, by monotypy (type fixation for *Madecassia* Kerremans).

Family-group name: LAMPROPEPLINA Holyński, 1993. Stem: *Lampropepl*-.

***Lamprosoma* W. Kirby, 1819**: 445. Current status: valid genus in CHRYSOMELIDAE: LAMPROSOMATINAE: LAMPROSOMATINI.

Type species: *Lamprosomabicolor* W. Kirby, 1819, by subsequent designation (Hope 1841a: 162).

Family-group name: LAMPROSOMIDEAE Lacordaire, 1848. Stem: *Lamprosomat*-.

***Lampyris* Geoffroy, 1762**: 165. Current status: valid genus in LAMPYRIDAE: LAMPYRINAE: LAMPYRINI. Note: name attributed to Geoffroy, 1762 and placed on the Official List of Generic Names in Zoology (ICZN 1984a).

Type species: *Cantharisnoctiluca* Linnaeus, 1758, by plenary power (see ICZN 1984a). Note: name placed on the Official List of Specific Names in Zoology (ICZN 1984a).

Family-group name: LAMPYRIA Rafinesque, 1815. Stem: *Lampyr*-.

***Lampyrolycus* Burgeon, 1937**: 152. Current status: valid genus in LYCIDAE: DEXORINAE: MIMOLIBNETINI: LAMPYROLYCINA.

Type species: *Lampyrolycushulstaerti* Burgeon, 1937, by monotypy.

Family-group name: LAMPYROLYCINI Kazantsev, 2018: 149. Stem: *Lampyrolyc*-.

***Lancetes* Sharp, 1882b**: 562, 602. Current status: valid genus in DYTISCIDAE: LANCETINAE.

Type species: *Dytiscusvarius* Fabricius, 1775, by subsequent designation (Guignot 1946: 117).

Family-group name: LANCETINI Branden, 1884. Stem: *Lancet*-.

***Langbianella* Prokofiev, 2015**: 21. Current status: valid genus in SCARABAEIDAE: MELOLONTHINAE: LANGBIANELLINI.

Type species: *Langbianellinaauricomes* Prokofiev, 2015, by original designation.

Family-group name: LANGBIANELLINI Prokofiev, 2015: 20. Stem: *Langbianell*-.

***Langelandia* Aubé, 1843**: 227. Current status: valid genus in ZOPHERIDAE: COLYDIINAE: SYNCHITINI.

Type species: *Langelandiaanophthalma* Aubé, 1843, by monotypy.

Family-group name: LANGELANDIINA Fowler, 1889. Stem: *Langelandi*-.

***Languria* Latreille, 1802b**: 209. Current status: valid genus in EROTYLIDAE: LANGURIINAE: LANGURIINI.

Type species: *Languriaruficollis* Latreille, 1802 (= *Trogositabicolor* Fabricius, 1798), by monotypy.

Family-group name: LANGUIRIDAE Hope, 1841. Stem: *Languri*-.

***Laogenia* Pascoe, 1874**: 75. Current status: valid genus in CURCULIONIDAE: DRYOPHTHORINAE: LITOSOMINI: LAOGENINA.

Type species: *Laogeniasorex* Pascoe, 1874, by subsequent designation (Setliff 2007: 73).

Family-group name: LAOGENINA Legalov, 2020a: 324. Stem: *Laogen*-.

***Laparocerus* Schönherr, 1834b**: 530. Current status: valid genus in CURCULIONIDAE: ENTIMINAE: OTIORHYNCHITAE: LAPAROCERINI.

Type species: *Laparocerusmorio* Boheman, 1834, by original designation.

Family-group name: LAPAROCÉRIDES Lacordaire, 1863. Stem: *Laparocer*-.

***Lapethus* Casey, 1890b**: 317, 321. Current status: junior synonym of *Mychocerus* Erichson, 1845 in CERYLONIDAE: CERYLONINAE.

Type species: *Lapethusdiscretus* Casey, 1890, by monotypy.

Family-group name: LAPETHINAE Sharp, 1894. Stem: *Lapeth*-.

***Lara* J.L. LeConte, 1852b**: 42. Current status: valid genus in ELMIDAE: LARAINAE: LARAINI. Note: *Lara* used by Drapiez (1819: 54) is an incorrect subsequent spelling of *Larra* Fabricius, 1792 in Hymenoptera (see Jäch et al. 2016: 104) not in prevailing usage; name placed on the Official List of Generic Names in Zoology (ICZN 1988g).

Type species: *Laraavara* J.L. LeConte, 1852, by monotypy (see ICZN 1988g). Note: name placed on the Official List of Specific Names in Zoology (ICZN 1988g).

Family-group name: LARINI J.L. LeConte, 1861. Stem: *Lara*- (ICZN 1988g).

***Laria* Scopoli, 1763**: 21. Current status: invalid senior synonym of *Bruchus* Linnaeus, 1767 in CHRYSOMELIDAE: BRUCHINAE: BRUCHINI: BRUCHINA. Note: name suppressed for the Principle of Priority and placed on the Official Index of Rejected and Invalid Generic Names in Zoology (ICZN 1995b).

Type species: *Lariasalicis* Scopoli, 1763, by subsequent designation (Pierce 1916: 463).

Family-group name: LARIIDAE Bedel, 1901. Stem: *Lari*-. Note: family-group name permanently invalid (ICZN 1999a, Article 39).

***Laricobius* Rosenhauer, 1846**: 5. Current status: valid genus in DERODONTIDAE: LARICOBIINAE.

Type species: *Laricobiuserichsonii* Rosenhauer, 1846, by monotypy.

Family-group name: LARICOBIENS Mulsant & Rey, 1864. Stem: *Laricobi*-.

***Larinotus* Carter & Zeck, 1937**: 182, 186. Current status: valid genus in TROGOSSITIDAE: LARINOTINAE.

Type species: *Larinotusumbilicatus* Carter & Zeck, 1937, by monotypy.

Family-group name: LARINOTINAE Ślipiński, 1992. Stem: *Larinot*-.

***Larinus* Dejean, 1821**: 97. Current status: valid genus in CURCULIONIDAE: LIXINAE: LIXINI. Note: name first proposed as a junior synonym of *Rhinobatus* Germar, 1817 and subsequently used as valid before 1961 (e.g., Schönherr 1823: 1146; Germar 1823: 379), it is available and dates from its first publication as a synonym (ICZN 1999a, Article 11.6.1).

Type species: *Curculiocynarae* Fabricius, 1787, by subsequent designation (Schönherr 1823: 1146). Note: nomen protectum (see Gültekin 2013: 108).

Family-group name: LARINIDAE Gistel, 1848. Stem: *Larin*-. Note: this name was listed as permanently invalid by Bouchard et al. (2011: 621) on the incorrect belief that it was proposed for *Larinus* Germar, 1823, a junior homonym of *Larinus* Dejean, 1821; we believe that Germar’s (1823: 379) name is simply a subsequent use of Dejean’s name.

***Lariversius* Blaisdell, 1947**: 59. Current status: valid genus in TENEBRIONIDAE: BLAPTINAE: AMPHIDORINI.

Type species: *Lariversiustibialis* Blaisdell, 1947, by original designation.

Family-group name: LARIVERSIINA La Rivers, 1948. Stem: *Lariversi*-.

***Lasiocala* Blanchard in H. Milne-Edwards et al., 1851**: 220. Current status: valid genus in SCARABAEIDAE: RUTELINAE: RUTELINI: LASIOCALINA.

Type species: *Lasiocalafulvohirta* Blanchard, 1851, by monotypy.

Family-group name: LASIOCALINA Ohaus, 1918. Stem: *Lasiocal*-.

***Lasiocera* Dejean, 1831**: 278, 283. Current status: valid genus in CARABIDAE: HARPALINAE: ODACANTHINI: ODACANTHINA.

Type species: *Lasioceranitidula* Dejean, 1831, by monotypy.

Family-group name: LASIOCERINI Jeannel, 1948. Stem: *Lasiocer*-.

***Lasioderma* Stephens, 1835a**: 417. Current status: valid genus in PTINIDAE: XYLETININAE: LASIODERMINI.

Type species: *Lasiodermatestaceum* Stephens, 1835 (= *Ptinusserricornis* Fabricius, 1792), by monotypy.

Family-group name: LASIODERMINI Böving, 1927. Stem: *Lasioderm*-.

***Lasiopus* J.L. LeConte, 1856b**: 282. Current status: invalid senior synonym of *Podolasia* Harold, 1869 in SCARABAEIDAE: PODOLASIINAE. Note: junior homonym of *Lasiopus* Schönherr, 1823 [Coleoptera: CURCULIONIDAE].

Type species: *Lasiopusferrugineus* J.L. LeConte, 1856, by monotypy.

Family-group name: LASIOPODES J.L. LeConte, 1856. Stem: *Lasiopod*-. Note: family-group name permanently invalid (ICZN 1999a, Article 39).

***Lasiorhynchites* Jekel, 1860**: 227. Current status: valid genus in ATTELABIDAE: RHYNCHITINAE: RHYNCHITITAE: RHYNCHITINI: LASIORHYNCHITINA.

Type species: **here fixed** (under ICZN 1999a, Article 70.3.2) as *Rhynchitescavifrons* Gyllenhal, 1833, misidentified as *Curculiopubescens* Fabricius, 1775 (sensu Herbst, 1797) by original designation in Jekel (1860: 227).

Family-group name: LASIORHYNCHITINA Legalov, 2003. Stem: *Lasiorhynchit*-.

†***Lasiosyne* Tan, Ren & Shih, 2007**: 237. Current status: valid genus in †LASIOSYNIDAE. Note: Middle/Upper Jurassic and Lower Cretaceous (Russia, Mongolia, China).

Type species: †*Lasiosyneeuglyphea* Tan, Ren & Shih, 2007, by original designation.

Family-group name: †LASIOSYNIDAE Kirejtshuk, Chang, Ren & Kun, 2010. Stem: *Lasiosyn*-.

***Lathrobium* Gravenhorst, 1802**: 51. Current status: valid genus in STAPHYLINIDAE: PAEDERINAE: LATHROBIINI: LATHROBIINA.

Type species: *Staphylinuselongatus* Linnaeus, 1767, by subsequent designation (Latreille 1810: 427).

Family-group name: LATHROBIDAE Laporte, 1835. Stem: *Lathrobi*-.

***Lathropus* Erichson, 1845a**: 307. Current status: valid genus in LAEMOPHLOEIDAE.

Type species: *Trogossitasepicola* P.W.J. Müller, 1821, by subsequent monotypy (Erichson 1846: 328).

Family-group name: LATHROPINI Casey, 1916: 117. Stem: *Lathrop*-. Note: this name was overlooked in Bouchard et al. (2011) and Bouchard and Bousquet (2020a).

***Laticranium* Lane, 1959**: 312. Current status: valid genus in CERAMBYCIDAE: LAMIINAE: LATICRANIINI.

Type species: *Laticraniummandibulare* Lane, 1959, by original designation.

Family-group name: LATICRANIINAE Lane, 1959. Stem: *Laticrani*-.

***Latometus* Erichson, 1842**: 213. Current status: valid genus in ZOPHERIDAE: ZOPHERINAE: LATOMETINI.

Type species: *Latometuspubescens* Erichson, 1842, by monotypy.

Family-group name: LATOMETINI Ślipiński & Lawrence, 1999. Stem: *Latomet*-.

***Latridius* Herbst, 1793**: 3. Current status: valid genus in LATRIDIIDAE: LATRIDIINAE. Note: name placed on the Official List of Generic Names in Zoology (ICZN 2011e).

Type species: *Latridiusporcatus* Herbst, 1793, by subsequent designation (Latreille 1810: 431; see ICZN 2011e).

Family-group name: LATHRIDIEN Erichson, 1842. Stem: *Latridi*-.

***Lawrencerosus* Kirejtshuk, 1991**: 866. Current status: valid genus in NITIDULIDAE: NITIDULINAE: LAWRENCEROSINI.

Type species: *Lawrencerosusmirator* Kirejtshuk, 1991, by original designation.

Family-group name: LAWRENCEROSINI Kirejtshuk, 1991. Stem: *Lawrenceros*-.

†***Lebanophytum*** Kirejtshuk & Azar, 2008: 31. Current status: valid genus in CRYPTOPHAGIDAE. Note: Lower Cretaceous (Lebanon).

Type species: †*Lebanophytumexcellens* Kirejtshuk & Azar, 2008, by original designation.

Family-group name: †LEBANOPHYTIDAE Kirejtshuk in Kirejtshuk and Azar, 2013: 114. Stem: *Lebanophyt*-.

***Lebia* Latreille, 1802b**: 85. Current status: valid genus in CARABIDAE: HARPALINAE: LEBIINI: LEBIINA.

Type species [suggested]: *Carabushaemorrhoidalis* Fabricius, 1787 (= *Buprestismarginatus* Geoffroy, 1785), by subsequent designation (E. Desmarest 1851: 76). Note: the valid type species fixation is that of Latreille (1810: 426), who selected *Carabusquadrimaculatus* Linnaeus, 1758, but that species is the type species of *Dromius* Bonelli, 1810; a subsequent designation is that of Drapiez (1839: 303), who selected *Carabuscyanocephalus* Linnaeus, 1758, but that species is currently included in the subgenus Lamprias Bonelli, 1810, not in the nominotypical subgenus; a new application to the Commission is necessary to fix *Carabushaemorrhoidalis* Fabricius, 1787 as the type species of *Lebia* Latreille, 1802 since a previous request was postponed (ICZN 1950: 447).

Family-group name: LEBIOTAE Bonelli, 1810. Stem: *Lebi*-.

***Lebidia* Morawitz, 1862**: 322. Current status: valid genus in CARABIDAE: HARPALINAE: LEBIINI: GALLERUCIDIINA.

Type species: *Lebidiaoctoguttata* Morawitz, 1862, by monotypy.

Family-group name: LEBIDIINA Jakobson, 1907. Stem: *Lebidi*-.

***Lechriops* Schönherr, 1825**: 586. Current status: valid genus in CURCULIONIDAE: CONODERINAE: LECHRIOPINI.

Type species: *Rhynchaenussciurus* Fabricius, 1801, by original designation.

Family-group name: LÉCHRIOPIDES Lacordaire, 1865. Stem: *Lechriop*-.

†***Leehermania* Chatzimanolis, Grimaldi & Engel in Chatzimanolis et al., 2012**: 6. Current status: valid genus in HYDROSCAPHIDAE: †LEEHERMANIINAE. Note: Upper Triassic (USA).

Type species: †*Leehermaniaprorova* Chatzimanolis et al., 2012, by original designation.

Family-group name: †LEEHERMANIINAE Kirejtshuk, Prokin & Ponomarenko, 2023b: 7. Stem: *Leehermani*-.

***Leichenum* Dejean, 1834**: 194. Current status: valid genus in TENEBRIONIDAE: BLAPTINAE: PEDININI: LEICHENINA.

Type species: *Opatrumpictum* Fabricius, 1801, by monotypy.

Family-group name: LEICHENAIRES Mulsant, 1854. Stem: *Leichen*-.

***Leiestes* Chevrolat in Dejean, 1836a**: 440. Current status: valid genus in ENDOMYCHIDAE: LEIESTINAE.

Type species: *Cryptophagusseminiger* Gyllenhal, 1808, by monotypy.

Family-group name: LEIESTINA C.G. Thomson, 1863. Stem: *Leiest*-.

***Leiochrinus* Westwood, 1883**: 68. Current status: valid genus in TENEBRIONIDAE: DIAPERINAE: LEIOCHRININI.

Type species: *Leiochrinusfulvicollis* Westwood, 1883, by subsequent designation (K.W. Kirby 1885: 89).

Family-group name: LEIOCHRININAE Lewis, 1894. Stem: *Leiochrin*-.

***Leiodes* Latreille, 1797**: 22. Current status: valid genus in LEIODIDAE: LEIODINAE: LEIODINI.

Type species: *Sphaeridiumferrugineum* Fabricius, 1787, by subsequent monotypy (Latreille 1802b: 163).

Family-group name: LEIODESIDAE Fleming, 1821. Stem: *Leiod*-.

***Leiopleura* Deyrolle, 1865**: 219. Current status: valid genus in BUPRESTIDAE: AGRILINAE: TRACHEINI: LEIOPLEURINA.

Type species: *Brachysconcinna* Gory, 1841, by original designation.

Family-group name: LEIOPLEURINA Holyński, 1993. Stem: *Leiopleur*-.

***Leiopus* Audinet-Serville, 1835a**: 86. Current status: valid genus in CERAMBYCIDAE: LAMIINAE: ACANTHOCININI.

Type species: *Cerambyxnebulosus* Linnaeus, 1758, by subsequent designation (Westwood 1838c: 41).

Family-group name: LIOPI J.L. LeConte, 1873. Stem: *Leiopod*-. Note: senior homonym of LEIOPIDAE [should be LEIOPODIDAE] Lang, 1970 (type genus *Leiopus* Beddard, 1886) in Crustacea; Lang’s name is permanently invalid (based on a preoccupied type genus).

***Leiosoma* Stephens, 1829b**: 14. Current status: valid genus in CURCULIONIDAE: MOLYTINAE: MOLYTINI: LEIOSOMATINA.

Type species: *Curculiopunctatus* Marsham, 1802 (= *Curculiodeflexus* Panzer, 1795), by monotypy.

Family-group name: LIOSOMINA Reitter, 1913. Stem: *Leiosomat*-.

***Leleupaussus* Luna de Carvalho, 1962**: 66, 68. Current status: valid genus in CARABIDAE: PAUSSINAE: PAUSSINI: PAUSSINA.

Type species: *Leleupaussustetramerus* Luna de Carvalho, 1962, by original designation.

Family-group name: LELEUPAUSSINA Luna de Carvalho, 1989. Stem: *Leleupauss*-.

***Leleupidia* Basilewsky, 1951**: 176. Current status: valid genus in CARABIDAE: HARPALINAE: ZUPHIINI: LELEUPIDIINA.

Type species: *Leleupidialuvubuana* Basilewsky, 1951, by original designation.

Family-group name: LELEUPIDIINI Basilewsky, 1951. Stem: *Leleupidi*-.

***Lema* Fabricius, 1798**: 4. Current status: valid genus in CHRYSOMELIDAE: CRIOCERINAE: LEMINI. Note: name placed on the Official List of Generic Names in Zoology (ICZN 1970a).

Type species: *Lemacyanea* Fabricius, 1798, by plenary power (see ICZN 1970a). Note: name placed on the Official List of Specific Names in Zoology (ICZN 1970a).

Family-group name: LEMOIDEAE Gyllenhal, 1813. Stem: *Lem*-.

***Lemidia* Spinola, 1841**: 75. Current status: valid genus in CLERIDAE: CLERINAE: CLERITAE: HYDNOCERINI: LEMIDIINA.

Type species: *Hydnoceranitens* Newman, 1841, by subsequent monotypy (Erichson 1843c: 179).

Family-group name: LEMIDIINI Kolibáč, 1998. Stem: *Lemidi*-.

***Lemodes* Boheman, 1858**: 103. Current status: valid genus in ANTHICIDAE: LEMODINAE.

Type species: *Lemodescoccinea* Boheman, 1858, by monotypy.

Family-group name: LEMODINAE E.G. Matthews, 1987. Stem: *Lemod*-.

***Lemphus* Erichson, 1840b**: 131. Current status: valid genus in MELYRIDAE: MALACHIINAE: LEMPHINI.

Type species: *Lemphusmancus* Erichson, 1840, by monotypy.

Family-group name: LEMPHINI Wittmer, 1976. Stem: *Lemph*-.

***Lenax* Sharp, 1877b**: 265, 269. Current status: valid genus in MONOTOMIDAE: MONOTOMINAE: LENACINI.

Type species: *Lenaxmirandus* Sharp, 1877, by monotypy.

Family-group name: LENACINAE Crowson, 1952. Stem: *Lenac*-.

***Lendomus* Casey, 1924**: 185. Current status: junior synonym of *Agyrtecanus* Reitter, 1901 in AGYRTIDAE: AGYRTINAE.

Type species: *Lendomuspolitus* Casey, 1924 (= *Necrophiluslongulus* J.L. LeConte, 1859), by original designation.

Family-group name: LENDOMINI Bradley, 1930. Stem: *Lendom*-.

***Leoglymmius* R.T. Bell & J.R. Bell, 1978**: 53. Current status: valid genus in CARABIDAE: RHYSODINAE: LEOGLYMMIINI.

Type species: *Rhysodeslignarius* Olliff, 1886, by original designation.

Family-group name: LEOGLYMMIINA R.T. Bell & J.R. Bell, 1978. Stem: *Leoglymmi*-.

***Lepanomus* J. Balfour-Browne, 1945**: 59. Current status: valid genus in BRENTIDAE: APIONINAE incertae sedis.

Type species: *Lepanomuscrinalis* J. Balfour-Browne, 1945, by original designation.

Family-group name: LEPANOMINI Wanat, 2021: 56. Stem: *Lepanom*-.

***Leperina* Erichson, 1844**: 453. Current status: valid genus in TROGOSSITIDAE: TROGOSSITINAE: TROGOSSITINI.

Type species: *Trogossitadecorata* Erichson, 1842, by subsequent designation (Kolibáč 2005: 65).

Family-group name: LEPERINI Reitter, 1876. Stem: *Leperin*-.

***Lepicerus* Motschulsky, 1855a**: 14. Current status: valid genus in LEPICERIDAE.

Type species: *Lepicerusinaequalis* Motschulsky, 1855, by monotypy.

Family-group name: LEPICERIDAE Hinton, 1936. Stem: *Lepicer*-.

***Lepisia* Lepeletier & Audinet-Serville, 1828**: 374. Current status: valid genus in SCARABAEIDAE: MELOLONTHINAE: HOPLIINI: HOPLIINA.

Type species: *Melolontharupicola* Fabricius, 1775, by subsequent designation (Blanchard in Audouin et al. 1843: pl. 43).

Family-group name: LEPISIIDAE Burmeister, 1844. Stem: *Lepisi*-.

***Lepithrix* Lepeletier & Audinet-Serville, 1828**: 381. Current status: valid genus in SCARABAEIDAE: MELOLONTHINAE: HOPLIINI: LEPITHRICHINA. Note: although originally established as *Lepitrix*, the spelling *Lepithrix*, an unjustified emendation by von Dalla Torre (1913: 342), is in prevailing usage, attributed to the original publication (see Cupello and Ribeiro-Costa 2019: 140, 141), and therefore deemed to be a justified emendation and the correct original spelling (ICZN 1999a, Article 33.2.3.1).

Type species: *Trichiuslineatus* Fabricius, 1775, by subsequent designation (Blanchard in Audouin et al. 1843: pl. 44).

Family-group name: LEPITRICHINA Perty, 1840. Stem: *Lepithrich*-.

***Leprotes* Baly, 1863**: 148, 158. Current status: junior synonym of *Fidia* Motschulsky, 1860 in CHRYSOMELIDAE: EUMOLPINAE: BROMIINI.

Type species: *Endoxusgracilicornis* Baly, 1861, by original designation.

Family-group name: LEPROTITES Chapuis, 1874. Stem: *Leprotet*-.

***Leptanillophilus* Holmgren, 1908**: 340. Current status: valid genus in STAPHYLINIDAE: ALEOCHARINAE: MIMECITINI: LEPTANILLOPHILINA.

Type species: *Leptanillophilussimilis* Holmgren, 1908, by monotypy.

Family-group name: LEPTANILLOPHILINI Fenyes, 1919. Stem: *Leptanillophil*-.

***Leptaulax* Kaup, 1868a**: 11. Current status: valid genus in PASSALIDAE: PASSALINAE: LEPTAULACINI.

Type species: *Passalusdentatus* Fabricius, 1792, by subsequent designation (Gravely 1918: 112).

Family-group name: LEPTAULACEAE Kaup, 1871. Stem: *Leptaulac*-.

***Leptidea* Mulsant, 1839**: 105. Current status: invalid senior synonym of *Nathrius* Brèthes, 1916 in CERAMBYCIDAE: CERAMBYCINAE: PSEBIINI. Note: junior homonym of *Leptidea* Billberg, 1820 [Lepidoptera].

Type species: *Leptideabrevipennis* Mulsant, 1839, by monotypy.

Family-group name: LEPTIDEINAE Perrier, 1893. Stem: *Leptide*-. Note: family-group name permanently invalid (ICZN 1999a, Article 39).

***Leptinopterus* Hope, 1838b**: 316. Current status: valid genus in LUCANIDAE: LUCANINAE: LUCANINI. Note: the original spelling was *Leptynopterus* but *Leptinopterus*, an unjustified emendation by Hope (1845b: 5), is in prevailing usage, attributed to the original author and date, and therefore deemed to be a justified emendation (ICZN 1999a, Article 33.2.3.1); *Psalicerus* Dejean, 1833 is a senior objective synonym of this genus (see Bousquet and Bouchard 2013a: 40) and an application to the Commission or Reversal of Precedence (ICZN 1999a, Article 23.9) is needed to conserve *Leptinopterus* Hope as a valid name.

Type species: *Lucanusfemoratus* G.-A. Olivier, 1789, by subsequent designation (R. Lucas 1920: 369).

Family-group name: LEPTINOPTERINI Jakobson, 1911. Stem: *Leptinopter*-.

***Leptinus* P.W.J. Müller, 1817**: 266. Current status: valid genus in LEIODIDAE: PLATYPSYLLINAE.

Type species: *Leptinustestaceus* P.W.J. Müller, 1817, by monotypy.

Family-group name: LEPTINIDAE J.L. LeConte, 1872. Stem: *Leptin*-.

***Leptispa* Baly, 1858**: 1. Current status: valid genus in CHRYSOMELIDAE: CASSIDINAE: LEPTISPINI. Note: new replacement name for *Leptomorpha* Germar, 1842.

Type species: *Leptomorphafiliformis* Germar, 1842, by monotypy (type fixation for *Leptomorpha* Germar).

Family-group name: LEPTISPITES Fairmaire, 1868. Stem: *Leptisp*-.

***Leptobium* Casey, 1905**: 57. Current status: valid genus in STAPHYLINIDAE: PAEDERINAE: PAEDERINI: DOLICAONINA.

Type species: *Lathrobiumbiguttulum* Lacordaire, 1835 (= *Lathrobiumgracile* Gravenhorst, 1802), by monotypy.

Family-group name: LEPTOBII Bordoni, 1980. Stem: *Leptobi*-.

***Leptochirus* Germar, 1823**: 35. Current status: valid genus in STAPHYLINIDAE: OSORIINAE: LEPTOCHIRINI.

Type species: *Leptochirusscoriaceus* Germar, 1823, by monotypy.

Family-group name: LEPTOCHIRINA Sharp, 1887. Stem: *Leptochir*-.

***Leptochromus* Motschulsky, 1855a**: 12. Current status: valid genus in STAPHYLINIDAE: SCYDMAENINAE: MASTIGITAE: LEPTOCHROMINI.

Type species: *Leptochromusfulvescens* Motschulsky, 1855, by monotypy.

Family-group name: LEPTOCHROMINI Jałoszyński & Brunke in Jałoszyński et al., 2018: 635. Stem: *Leptochrom*-.

***Leptodes* Dejean, 1834**: 181. Current status: valid genus in TENEBRIONIDAE: PIMELIINAE: LEPTODINI.

Type species: *Sepidium boisduvalii* Zoubkoff, 1833, by monotypy.

Family-group name: LEPTODIDES Lacordaire, 1859. Stem: *Leptod*-. Note: senior homonym of LEPTODINAE Schuchert, 1913 (type genus *Leptodus* Kayser, 1883) in Brachiopoda.

***Leptodinodes* Jeannel, 1949b**: 786. Current status: valid genus in CARABIDAE: HARPALINAE: CHLAENIINI: CHLAENIINA.

Type species: *Chlaeniusparallelus* Dejean, 1831, by original designation.

Family-group name: LEPTODINODINI Basilewsky & Grundmann, 1955. Stem: *Leptodinod*-.

***Leptodirus* F.J. Schmidt, 1832a**: 9. Current status: valid genus in LEIODIDAE: CHOLEVINAE: LEPTODIRINI: LEPTODIRINA. Note: the name was also proposed by F.J. Schmidt subsequently the same year (1832b: 83).

Type species: *Leptodirushochenwartii* F.J. Schmidt, 1832, by monotypy.

Family-group name: LEPTODÉRIDES Lacordaire, 1854. Stem: *Leptodir*-.

***Leptohoplia* Saylor, 1935**: 132. Current status: valid genus in SCARABAEIDAE: RUTELINAE: ANOMALINI: LEPTOHOPLIINA.

Type species: *Leptohopliatestaceipennis* Saylor, 1935, by original designation.

Family-group name: LEPOTHOPLIINI Potts, 1974. Stem: *Leptohopli*-.

***Leptolycus* Leng & Mutchler, 1922**: 430. Current status: valid genus in LYCIDAE: LYCINAE: LEPTOLYCINI: LEPTOLYCINA.

Type species: *Leptolycusheterocornis* Leng & Mutchler, 1922, by monotypy.

Family-group name: LEPTOLYCINI Leng & Mutchler, 1922. Stem: *Leptolyc*-.

***Leptomastax* Pirazzoli, 1855**: 3. Current status: valid genus in STAPHYLINIDAE: SCYDMAENINAE: MASTIGITAE: LEPTOMASTACINI.

Type species: *Leptomastaxhypogeum* Pirazzoli, 1855, by monotypy.

Family-group name: LEPTOMASTACINI Casey, 1897. Stem: *Leptomastac*-.

***Leptonota* J. Thomson, 1861**: 353. Current status: valid genus in CERAMBYCIDAE: LAMIINAE: ENICODINI.

Type species: *Enicodescomitessa* A. White, 1855, by monotypy.

Family-group name: LEPTONOTITAE J. Thomson, 1864. Stem: *Leptonot*-.

***Leptopalpus* Guérin-Méneville, 1834a**: pl. 35. Current status: valid genus in MELOIDAE: NEMOGNATHINAE: NEMOGNATHINI: ZONITIDINA.

Type species: *Leptopalpuschevrolatii* Guérin-Méneville, 1834 (= *Zonitisrostrata* Fabricius, 1792), by monotypy.

Family-group name: LEPTOPALPINA Kaszab, 1963. Stem: *Leptopalp*-.

***Leptopius* Oke, 1951**: 24. Current status: valid genus in CURCULIONIDAE: ENTIMINAE: ENTIMITAE: EUDIAGOGINI. Note: new replacement name for *Leptops* Schönherr, 1834.

Type species: *Curculiorobustus* G.-A. Olivier, 1807, by original designation (type fixation for *Leptops* Schönherr).

Family-group name: LEPTOPIINAE Oke, 1951. Stem: *Leptopi*-.

†***Leptopodocoleus* Hong, 1982**: 119. Current status: valid genus in TRACHYPACHIDAE: †EODROMEINAE. Note: Lower Cretaceous (China).

Type species: †*Leptopodocoleussarmentosus* Hong, 1982, by original designation.

Family-group name: †LEPTOPODOCOLEIDAE Hong, 1982. Stem: *Leptopodocole*-.

***Leptops* Schönherr, 1834a**: 297. Current status: invalid senior synonym of *Leptopius* Oke, 1951 in CURCULIONIDAE: ENTIMINAE: ENTIMITAE: EUDIAGOGINI. Note: junior homonym of *Leptops* Rafinesque, 1820 [Pisces].

Type species: *Curculiorobustus* G.-A. Olivier, 1807, by original designation.

Family-group name: LEPTOPSIDES Lacordaire, 1863. Stem: *Leptop*-. Note: family-group name permanently invalid (ICZN 1999a, Article 39).

***Leptorhynchus* Boisduval, 1835**: 318. Current status: invalid senior synonym of *Ithystenus* Pascoe, 1862 in BRENTIDAE: BRENTINAE: BRENTINI: ITHYSTENINA. Note: junior homonym of *Leptorhynchus* Clift, 1828 [Reptilia].

Type species: *Brenthusangustatus* Guérin-Méneville, 1831, by monotypy.

Family-group name: LEPTORRHYNCHIDAE Schoenfeldt, 1908. Stem: *Leptorhynch*-. Note: family-group name permanently invalid (ICZN 1999a, Article 39).

***Leptoschoinus* Dejean, 1835**: 291. Current status: valid genus in CURCULIONIDAE: BARIDINAE: MADARINI: MADARINA.

Type species: *Barisfucata* Klug, 1829, by monotypy.

Family-group name: LEPTOSCHOÏNIDES Lacordaire, 1865. Stem: *Leptoschoin*-.

***Leptoscydmus* Casey, 1897**: 518. Current status: valid genus in STAPHYLINIDAE: SCYDMAENINAE: SCYDMAENITAE: LEPTOSCYDMINI.

Type species: *Leptoscydmuscavifrons* Casey, 1897, by subsequent designation (H. Franz 1985: 177).

Family-group name: LEPTOSCYDMINI Casey, 1897: 518. Stem: *Leptoscydm*-.

***Leptosonyx* Weise, 1885b**: 315. Current status: junior synonym of *Theone* Gistel, 1857 in CHRYSOMELIDAE: GALERUCINAE: GALERUCINI.

Type species: *Gallerucasilphoides* Dalman, 1823, by original designation.

Family-group name: LEPTOSONYCHINI Weise, 1924. Stem: *Leptosonych*-.

***Leptostethus* G.R. Waterhouse, 1853**: 175. Current status: valid genus in CURCULIONIDAE: ENTIMINAE: ENTIMITAE: LEPTOSTETHINI.

Type species: *Leptostethusmarginatus* G.R. Waterhouse, 1853, by original designation.

Family-group name: LEPTOSTÉTHIDES Lacordaire, 1863. Stem: *Leptosteth*-.

***Leptosus* G.A.K. Marshall, 1952**: 265. Current status: junior synonym of *Leptopius* Oke, 1951 in CURCULIONIDAE: ENTIMINAE: ENTIMITAE: BYRSOPAGINI: STRANGALIODINA. Note: unnecessary new replacement name for *Leptops* Schönherr, 1833.

Type species: *Curculiorobustus* G.-A. Olivier, 1807, by original designation (type fixation for *Leptops* Schönherr).

Family-group name: LEPTOSINAE G.A.K. Marshall, 1952. Stem: *Leptos*-.

***Leptotyphlus* Fauvel, 1874**: [2^e^ supplément] 36. Current status: valid genus in STAPHYLINIDAE: LEPTOTYPHLINAE: LEPTOTYPHLINI.

Type species: *Leptotyphlussublaevis* Fauvel, 1874, by monotypy.

Family-group name: LEPTOTYPHLI Fauvel, 1874. Stem: *Leptotyphl*-.

***Leptura* Linnaeus, 1758**: 342, 397. Current status: valid genus in CERAMBYCIDAE: LEPTURINAE: LEPTURINI.

Type species [suggested]: *Lepturaquadrifasciata* Linnaeus, 1758, by subsequent designation (Westwood 1838c: 41). Note: the valid type species fixation is that of Partington (1836b: 45), who selected *Lepturamelanura* Linnaeus, 1758, but that species is the type species of the genus *Stenurella* Villiers, 1974; an application to the Commission is necessary to fix *Lepturaquadrifasciata* Linnaeus, 1758 as the type species of *Leptura* Linnaeus, 1758.

Family-group name: LEPTURETAE Latreille, 1802. Stem: *Leptur*-. Note: senior homonym of LEPTURINAE Gill, 1860 (type genus *Lepturus* Günther, 1854) in Pisces.

***Lepturoides* Herbst, 1784**: 103. Current status: junior synonym of *Denticollis* Piller & Mitterpacher, 1783 in ELATERIDAE: DENDROMETRINAE: DENDROMETRINI: DENTICOLLINA.

Type species: *Lepturoideslinearis* Herbst, 1784 (= *Elaterlinearis* Linnaeus, 1758), by monotypy.

Family-group name: LEPTUROIDINI O. Schwarz, 1906. Stem: *Lepturoid*-.

***Leptusa* Kraatz, 1856**: 60. Current status: valid genus in STAPHYLINIDAE: ALEOCHARINAE: HOMALOTINI: BOLITOCHARINA. Note: name placed on the Official List of Generic Names in Zoology (ICZN 2005d).

Type species: *Bolitocharapulchella* Mannerheim, 1830, by subsequent designation (Gusarov and Herman 2003: 115; see ICZN 2005d). Note: name placed on the Official List of Specific Names in Zoology (ICZN 2005d).

Family-group name: LEPTUSAE Fenyes, 1919. Stem: *Leptus*-.

***Lepyrus* Germar, 1817b**: 340. Current status: valid genus in CURCULIONIDAE: MOLYTINAE: LEPYRINI.

Type species [suggested]: *Curculiocolon* Linnaeus, 1771 (= *Curculiopalustris* Scopoli, 1763), by subsequent designation (Schönherr 1823: 1143). Note: the valid type species fixation is that of Samouelle (1819: 204), who selected *Curculiotriguttatus* Fabricius, 1775, but that species is the type species of the genus *Graptus* Schönherr, 1823; Alonso-Zarazaga and Lyal (1999: 204) mentioned that they would submit an application to the Commission to fix *Curculiocolon* Linnaeus, 1771 as the type species of this type genus but this has not taken place; an application to the Commission is still necessary to fix this problem.

Family-group name: LEPYRIDAE W. Kirby, 1837. Stem: *Lepyr*-.

***Lesteva* Latreille, 1797**: 75. Current status: valid genus in STAPHYLINIDAE: OMALIINAE: ANTHOPHAGINI. Note: name placed on the Official List of Generic Names in Zoology (ICZN 2004e).

Type species: *Lestevapunctulata* Latreille, 1804 (= *Staphylinuslongoelytratus* Goeze, 1777), by plenary power (see ICZN 2004e). Note: *Lestevapunctulata* Latreille, 1804 was placed on the Official List of Specific Names in Zoology (ICZN 2004e); Goeze’s (1777) work is not consistently binominal (see ICZN 2021a) and therefore an application to the Commission is necessary to retain *Staphylinuslongoelytratus* Goeze, 1777 as the valid name for the type species.

Family-group name: LESTEVINA Jakobson, 1908. Stem: *Lestev*-.

***Lestignathus* Erichson, 1842**: 132. Current status: valid genus in CARABIDAE: HARPALINAE: LICININI: LESTIGNATHINA.

Type species: *Lestignathuscursor* Erichson, 1842, by monotypy.

Family-group name: LESTIGNATHINA Ball, 1992. Stem: *Lestignath*-.

***Lethrus* Scopoli, 1777**: 439. Current status: valid genus in GEOTRUPIDAE: GEOTRUPINAE: LETHRINI.

Type species: **here fixed** (under ICZN 1999a, Article 70.3.2) as *Lucanusapterus* Laxmann, 1770, misidentified as *Scarabaeuscephalotes* Pallas, 1771 (sensu Acharius, 1781), by subsequent monotypy in Fabricius (1787: 2). Note: the nominal species *Scarabaeuscephalotes* Pallas, 1771 is currently placed in the subgenus Ceratodirus Fischer von Waldheim, 1845.

Family-group name: LETHREN Oken, 1843. Stem: *Lethr*-.

***Leucastea* Stål, 1855**: 344. Current status: valid genus in MEGALOPODIDAE: MEGALOPODINAE: LEUCASTEINI. Note: the original spelling was *Lucastea* but *Leucastea*, an incorrect subsequent spelling, is in prevailing usage, attributed to the publication of the original spelling, and therefore deemed to be the correct original spelling (ICZN 1999a, Article 33.3.1).

Type species: *Leucasteadohrni* Stål, 1855, by subsequent designation (Jacoby and Clavareau 1905: 15).

Family-group name: LEUCASTEINI Rodríguez-Mirón in Rodríguez-Mirón et al., 2021: 694. Stem: *Leucaste*-.

***Leucocelis* Burmeister, 1842**: 421. Current status: valid genus in SCARABAEIDAE: CETONIINAE: CETONIINI: LEUCOCELINA.

Type species: *Cetoniahaemorrhoidalis* Fabricius, 1775, by subsequent designation (Arrow 1910: 175).

Family-group name: LEUCOCELIDEN Kraatz, 1882. Stem: *Leucocel*-.

***Leucocraspedum* Kraatz, 1859**: 51. Current status: valid genus in STAPHYLINIDAE: ALEOCHARINAE: LEUCOCRASPEDINI.

Type species: *Leucocraspedumpulchellum* Kraatz, 1859, by monotypy.

Family-group name: LEUCOCRASPEDINI Fenyes, 1921. Stem: *Leucocrasped*-.

***Leucolaephus* P.H. Lucas, 1859**: xxii. Current status: valid genus in TENEBRIONIDAE: PIMELIINAE: PIMELIINI: PIMELIINA. Note: the original spelling was *Leucoloephus* but *Leucolaephus*, an incorrect subsequent spelling, is in prevailing usage, attributed to the publication of the original spelling, and therefore deemed to be the correct original spelling (ICZN 1999a, Article 33.3.1).

Type species: *Leucolaephusperrisii* P.H. Lucas, 1859 (= *Pimelialiliputana* P.H. Lucas, 1857), by subsequent designation (Gebien 1937: 803).

Family-group name: LEUCOLAEPHUSINI Pierre, 1961. Stem: *Leucolaeph*-.

***Leucopholis* Dejean, 1833b**: 160. Current status: valid genus in SCARABAEIDAE: MELOLONTHINAE: LEUCOPHOLINI.

Type species: *Melolontharorida* Fabricius, 1801, by subsequent designation (J. Bezděk 2006: 33).

Family-group name: LEUCOPHOLIDAE Burmeister, 1855. Stem: *Leucophol*-.

***Leucothyreus* W.S. MacLeay, 1819**: 145. Current status: valid genus in SCARABAEIDAE: RUTELINAE: GENIATINI.

Type species: *Leucothyreuskirbyanus* W.S. MacLeay, 1819, by monotypy.

Family-group name: LEUCOTHYREIDAE Burmeister, 1844. Stem: *Leucothyre*-.

***Leupelia* Jeannel, 1954b**: 106. Current status: valid genus in STAPHYLINIDAE: PSELAPHINAE: BATRISITAE: BATRISINI: LEUPELIINA.

Type species: *Leupelialongicornis* Jeannel, 1954, by original designation.

Family-group name: LEUPELIINA Jeannel, 1954. Stem: *Leupeli*-.

***Lexiphanes* Gistel, 1848b**: 123. Current status: valid genus in CHRYSOMELIDAE: CRYPTOCEPHALINAE: CRYPTOCEPHALINI: MONACHULINA. Note: new replacement name for *Monachus* Chevrolat, 1836.

Type species: *Cryptocephalussaponatus* Fabricius, 1801, by monotypy (type fixation for *Monachus* Chevrolat).

Family-group name: LEXIPHANINI Wilcox, 1954. Stem: *Lexiphan*-.

†***Liadytes* Ponomarenko, 1963**: 129. Current status: valid genus in †LIADYTIDAE. Note: Jurassic (Asia).

Type species: †*Liadytesavus* Ponomarenko, 1963, by original designation.

Family-group name: †LIADYTIDAE Ponomarenko, 1977. Stem: *Liadyt*-.

†***Liadytiscus* Prokin & Ren, 2010**: 48. Current status: valid genus in DYTISCIDAE: †LIADYTISCINAE: †LIADYTISCINI. Note: Lower Cretaceous (China).

Type species: †*Liadytiscuscretaceus* Prokin & Ren, 2010, by original designation.

Family-group name: †LIADYTISCINAE Prokin & Ren, 2010. Stem: *Liadytisc*-.

†***Liaoximordella* W.I. Wang, 1993**: 87. Current status: valid genus in †LIAOXIMORDELLIDAE. Note: Lower Cretaceous (China).

Type species: †*Liaoximordellahongi* W.I. Wang, 1993, by original designation.

Family-group name: †LIAOXIMORDELLIDAE W.I. Wang, 1993. Stem: *Liaoximordell*-.

***Liatongus* Reitter, 1892**: 167. Current status: valid genus in SCARABAEIDAE: SCARABAEINAE: ONITICELLINI: LIATONGINA. Note: this name is a junior synonym of *Onthosphaenus* Motschulsky, 1860 but *Liatongus* is well established and should be maintained until a case is submitted to the Commission (see J. Bezděk 2016: 13).

Type species: *Onthophagusphanaeoides* Westwood, 1839, by subsequent designation (Arrow 1931: 362).

Family-group name: LIATONGINA Philips, 2016: 41. Stem: *Liatong*-.

†***Libanopsis* Kirejtshuk in Kirejtshuk et al., 2015**: 466. Current status: valid genus in SPHINDIDAE: †LIBANOPSINAE. Note: Lower Cretaceous (Lebanon).

Type species: †*Libanopsispoinari* Kirejtshuk, 2015, by original designation.

Family-group name: †LIBANOPSINAE Kirejtshuk in Kirejtshuk et al., 2015: 462. Stem: *Libanops*-.

***Libnetis* C.O. Waterhouse, 1878**: 104. Current status: valid genus in LYCIDAE: METRIORRHYNCHINAE: LIBNETINI. Note: the original spelling was *Libnetus* but *Libnetis*, an unjustified emendation by Bourgeois (1883: 647), is in prevailing usage, attributed to the original author and date, and therefore deemed to be a justified emendation (ICZN 1999a, Article 33.2.3.1).

Type species: *Libnetispumilio* C.O. Waterhouse, 1878, by original designation.

Family-group name: LIBNETININA Bocák & Bocáková, 1990. Stem: *Libnet*-.

***Lichas* Westwood, 1853**: 237. Current status: invalid senior synonym of *Eulichas* Jakobson, 1913 in EULICHADIDAE. Note: junior homonym of *Lichas* Dalman, 1827 [Trilobita].

Type species: *Lichasfunebris* Westwood, 1853, by monotypy.

Family-group name: LICHADIDAE Csiki, 1902. Stem: *Lichad*-. Note: family-group name permanently invalid (ICZN 1999a, Article 39).

***Lichenophanes* Lesne, 1899**: 443, 457. Current status: valid genus in BOSTRICHIDAE: BOSTRICHINAE: BOSTRICHINI.

Type species: *Bostrichustristis* Fåhraeus, 1871, by subsequent designation (Chûjô 1937: 44).

Family-group name: LICHENOPHANINI Portevin, 1931. Stem: *Lichenophan*-.

***Lichnasthenus* J. Thomson, 1858b**: 35. Current status: valid genus in CARABIDAE: HARPALINAE: LEBIINI: DROMIUSINA. Note: Thomson originally used the spelling *Lichnastenus* (p. 35, 37, pl. 1), which he intentionnally changed to *Lichnasthenus* in the errata (p. [471]).

Type species: *Lichnasthenusarmiventris* J. Thomson, 1858, by monotypy.

Family-group name: LICHNASTHENITAE J. Thomson, 1858. Stem: *Lichnasthen*-.

***Lichnia* Erichson, 1835**: 269. Current status: valid genus in SCARABAEIDAE: MELOLONTHINAE: LICHNIINI.

Type species: *Lichnialimbata* Erichson, 1835, by monotypy.

Family-group name: LICHNIADAE Burmeister, 1844. Stem: *Lichni*-.

***Licinus* Latreille, 1802b**: 92. Current status: valid genus in CARABIDAE: HARPALINAE: LICININI: LICININA.

Type species: *Carabuscassideus* Fabricius, 1792, by subsequent designation (Latreille 1810: 426).

Family-group name: LICINII Bonelli, 1810. Stem: *Licin*-. Note: senior homonym of LICININA Edward, 1857 (type genus *Licina* J.E. Gray, 1850) in Mollusca.

†***Lidryops* Kirejtshuk & Nel, 2016b**: 142. Current status: valid genus in BYRRHIDAE: †LIDRYOPINAE. Note: Lower Cretaceous (France).

Type species: †*Lidryopsoccultus* Kirejtshuk & Nel, 2016, by original designation.

Family-group name: †LIDRYOPINAE Kirejtshuk & Nel, 2016b: 141. Stem: *Lidryop*-.

***Ligniperda* Pallas, 1772**: 6. Current status: invalid senior synonym of *Apate* Fabricius, 1775 (nomen protectum) in BOSTRICHIDAE: BOSTRICHINAE: APATINI. Note: nomen oblitum (see Borowski and Węgrzynowicz 2009: 190).

Type species: *Ligniperdaterebrans* Pallas, 1772, by subsequent designation (Borowski and Węgrzynowicz 2007: 19, 155).

Family-group name: LIGNIPERDIDAE Jacobi, 1906. Stem: *Ligniperd*-.

***Lignyodes* Dejean, 1835**: 278. Current status: valid genus in CURCULIONIDAE: CURCULIONINAE: TYCHIINI: LIGNYODINA.

Type species: *Curculioenucleator* Panzer, 1798, by monotypy.

Family-group name: LIGNYODINI Bedel, 1883. Stem: *Lignyod*-.

***Limnebius* Leach, 1815a**: 96. Current status: valid genus in HYDRAENIDAE: HYDRAENINAE: LIMNEBIINI.

Type species: *Hydrophiluspicinus* Marsham, 1802 (= *Hydrophilusnitidus* Marsham, 1802), by monotypy.

Family-group name: LIMNÉBIAIRES Mulsant, 1844. Stem: *Limnebi*-.

***Limnichopharus* Miyatake, 1994**: 269. Current status: valid genus in COCCINELLIDAE: COCCINELLINAE: LIMNICHOPHARINI.

Type species: *Limnichopharushainanensis* Miyatake, 1994, by original designation.

Family-group name: LIMNICHOPHARINI Miyatake, 1994. Stem: *Limnichophar*-.

***Limnichus* Dejean, 1821**: 49. Current status: valid genus in LIMNICHIDAE: LIMNICHINAE: LIMNICHINI.

Type species: *Byrrhuspygmaeus* Sturm, 1807, by monotypy.

Family-group name: LIMNICHINI Erichson, 1846. Stem: *Limnich*-.

***Limnius* Illiger, 1802**: 297. Current status: valid genus in ELMIDAE: ELMINAE: ELMINI: ELMINA.

Type species: *Dytiscusvolckmari* Panzer, 1793, by monotypy.

Family-group name: LIMNIIDAE C.G. Thomson, 1859. Stem: *Limni*-.

***Limnobaris* Bedel, 1885**: 183. Current status: valid genus in CURCULIONIDAE: BARIDINAE: APOSTASIMERINI: DIORYMERINA.

Type species: *Curculiot-album* Linnaeus, 1758, by original designation.

Family-group name: LIMNOBARINI Casey, 1922. Stem: *Limnobarid*-.

***Limonius* Eschscholtz, 1829a**: 33. Current status: valid genus in ELATERIDAE: DENDROMETRINAE: DENDROMETRINI: DENDROMETRINA.

Type species: *Elaterminutus* Linnaeus, 1758, by subsequent designation (Curtis 1838a: pl. 694). Note: Curtis’ typification, issued 1 June 1838 (see Evenhuis 1997a: 166), is earlier than Westwood’s typification, issued July 1838 (ICZN 1957a), of *Elaterbructeri* Panzer, 1795 (= *Elateraeneoniger* DeGeer, 1774), currently the type species of *Pheletes* Kiesenwetter, 1858.

Family-group name: LIMONIINA Jakobson, 1913. Stem: *Limoni*-. Note: junior homonym of LIMONIIDAE Speiser, 1909 (type genus *Limonia* Meigen, 1803) in Diptera.

***Limulodes* A. Matthews, 1867**: 409. Current status: valid genus in PTILIIDAE: PTILIINAE: CEPHALOPLECTINI.

Type species: *Limulodesparadoxus* A. Matthews, 1867, by monotypy.

Family-group name: LIMULODINAE Ganglbauer, 1898. Stem: *Limulod*-.

***Lina* Latreille, 1829b**: 151. Current status: junior synonym of *Chrysomela* Linnaeus, 1758 in CHRYSOMELIDAE: CHRYSOMELINAE: CHRYSOMELINI.

Type species: *Chrysomelapopuli* Linnaeus, 1758, by monotypy.

Family-group name: LINAEINES Motschulsky, 1860. Stem: *Lin*-.

***Liogenys* Guérin-Méneville, 1831a**: pl. 3. Current status: valid genus in SCARABAEIDAE: MELOLONTHINAE: DIPLOTAXINI.

Type species: *Liogenyscastaneus* Guérin-Méneville, 1831 (= *Melolonthapalpalis* Eschscholtz, 1822), by monotypy.

Family-group name: LIOGENITAE Blanchard, 1851. Stem: *Liogeny*-.

***Lionychus* Wissmann, 1846**: 25. Current status: valid genus in CARABIDAE: HARPALINAE: LEBIINI: DROMIUSINA.

Type species: *Lebiaquadrillum* Duftschmid, 1812, by monotypy.

Family-group name: LIONYCHIDAE Jeannel, 1948. Stem: *Lionych*-.

***Lioon* Casey, 1912**: 67. Current status: valid genus in BYRRHIDAE: BYRRHINAE: SIMPLOCARIINI.

Type species: *Lioonspeculare* Casey, 1912 (= *Amphicyrtasimplicipes* Mannerheim, 1852), by subsequent designation (R. Lucas 1920: 377).

Family-group name: LIOONINI Casey, 1912. Stem: *Lioon*-.

***Liophloeus* Germar, 1817b**: 341. Current status: valid genus in CURCULIONIDAE: ENTIMINAE: POLYDRUSITAE: POLYDRUSINI.

Type species: *Curculionubilus* Fabricius, 1777 (= *Curculiotessulatus* O.F. Müller, 1776), by monotypy.

Family-group name: LIOPHLOEIDAE Gistel, 1848. Stem: *Liophloe*-.

***Lioxyonyx* Hustache, 1933**: 100. Current status: valid genus in CURCULIONIDAE: CEUTORHYNCHINAE: LIOXYONYCHINI.

Type species: *Lioxyonyxuniseriatus* Hustache, 1933, by original designation.

Family-group name: LIOXYONYXINI Colonnelli, 1984. Stem: *Lioxyonych*-.

***Liparetrus* Guérin-Méneville, 1831a**: pl. 3. Current status: valid genus in SCARABAEIDAE: SERICOIDINAE: LIPARETRINI.

Type species: *Liparetrusdiscipennis* Guérin-Méneville, 1831, by monotypy.

Family-group name: LIPARETRIDAE Burmeister, 1855. Stem: *Liparetr*-.

***Liparocephalus* Mäklin in Mannerheim, 1853**: 191. Current status: valid genus in STAPHYLINIDAE: ALEOCHARINAE: LIPAROCEPHALINI.

Type species: *Liparocephalusbrevipennis* Mäklin, 1853, by monotypy.

Family-group name: LIPAROCEPHALI Fenyes, 1919. Stem: *Liparocephal*-.

***Liparochrus* Erichson, 1848**: 925. Current status: valid genus in HYBOSORIDAE: LIPAROCHRINAE.

Type species: *Liparochrusgeminatus* Westwood, 1852, by subsequent designation (Blackburn 1912: 41). Note: no species were originally included in this genus by Erichson (1848); the first included species were the three species described by Westwood (1852: 69, 70).

Family-group name: LIPAROCHRINAE Ocampo, 2006. Stem: *Liparochr*-.

***Liparoderus* LaFerté-Sénectère in Guérin-Méneville, 1849** [No 29]: 85. Current status: valid genus in ANTHICIDAE: ANTHICINAE: MICROHORIINI.

Type species: *Anthicusinsignis* P.H. Lucas, 1843, by original designation.

Family-group name: LIPARODERINI Bonadona, 1990. Stem: *Liparoder*-.

***Liparus* G.-A. Olivier, 1807**: [83] 283. Current status: valid genus in CURCULIONIDAE: MOLYTINAE: MOLYTINI: MOLYTINA.

Type species: *Curculiogermanus* Linnaeus, 1758, by subsequent designation (Latreille 1810: 430).

Family-group name: LIPARIDES Latreille, 1828. Stem: *Lipar*-. Note: senior homonym of LIPARIDAE Gill, 1861 (type genus *Liparis* Scopoli, 1777) in Pisces.

***Lispinus* Erichson, 1839b**: 31. Current status: valid genus in STAPHYLINIDAE: OSORIINAE: THORACOPHORINI: LISPININA.

Type species: *Lispinusattenuatus* Erichson, 1840, by subsequent designation (Duponchel 1841b: 57). Note: no species were originally included in this genus by Erichson (1839b); the first ones were the seven species listed by Erichson (1840a: 828–830).

Family-group name: LISPINI Bernhauer & Schubert, 1910. Stem: *Lispin*-.

***Lispotherium* Faust, 1891**: 194. Current status: valid genus in BRENTIDAE: APIONINAE: MYRMACICELITAE: LISPOTHERIINI.

Type species: *Lispotheriumhildebrandti* Faust, 1891, by monotypy.

Family-group name: LISPOTHERIINI Wanat, 2001. Stem: *Lispotheri*-.

***Lissauchenius* W.S. MacLeay, 1825**: 13. Current status: valid subgenus of *Chlaenius* Bonelli, 1810 in CARABIDAE: HARPALINAE: CHLAENIINI: CHLAENIINA.

Type species: *Lissaucheniusrufifemoratus* W.S. MacLeay, 1825, by monotypy.

Family-group name: LISSAUCHENIIDAE Gistel, 1848. Stem: *Lissaucheni*-.

***Lissodema* Curtis, 1833b**: 187. Current status: valid genus in SALPINGIDAE: SALPINGINAE.

Type species: *Lissodemaheyana* Curtis, 1833 (= *Salpinguscursor* Gyllenhal, 1813), by monotypy.

Family-group name: LISSODEMINA Seidlitz, 1916. Stem: *Lissodem*-.

***Lissogenius* Schaum, 1845**: 420. Current status: valid genus in SCARABAEIDAE: CETONIINAE: CREMASTOCHEILINI: LISSOGENIINA.

Type species: *Lissogeniusplanicollis* Schaum, 1845, by monotypy.

Family-group name: LISSOGENIINA Krikken, 1984. Stem: *Lissogeni*-.

***Lissomus* Dalman, 1824**: 13. Current status: valid genus in ELATERIDAE: LISSOMINAE: LISSOMINI.

Type species: *Lissomuspunctulatus* Dalman, 1824, by subsequent designation (Fleutiaux 1947: 138).

Family-group name: LISSOMIDAE Laporte, 1835. Stem: *Lissom*-.

***Lissonotus* Dalman in Schönherr, 1817**: 159. Current status: valid genus in CERAMBYCIDAE: CERAMBYCINAE: LISSONOTINI.

Type species: *Cerambyxbiguttatus* Dalman, 1817, by monotypy.

Family-group name: LISSONOTINAE Swainson, 1840. Stem: *Lissonot*-. Note: senior homonym of LISSONOTINI Förster, 1869 (type genus *Lissonota* Gravenhorst, 1829) in Hymenoptera.

***Lissopogonus* Andrewes, 1923a**: 213. Current status: valid genus in CARABIDAE: PATROBINAE: LISSOPOGONINI.

Type species: *Lissopogonusglabellus* Andrewes, 1923, by monotypy.

Family-group name: LISSOPOGONINI Zamotajlov, 2000. Stem: *Lissopogon*-.

***Lissorhoptrus* J.L. LeConte in J.L. LeConte and G.H. Horn, 1876**: 183. Current status: valid genus in CURCULIONIDAE: BRACHYCERINAE: TANYSPHYRINI.

Type species: *Bagoussimplex* Say, 1832, by subsequent designation (Kuschel 1952: 28).

Family-group name: LISSORHOPTRINAE Böving & Craighead, 1931. Stem: *Lissorhoptr*-.

***Lissotes* Westwood, 1855b**: 213. Current status: valid genus in LUCANIDAE: LUCANINAE: SCLEROSTOMINI.

Type species: *Lucanuscancroides* Fabricius, 1787, by subsequent designation (R. Lucas 1920: 378). Note: the type species usually listed in the literature is *Lissotesmenalcas* Westwood, 1855, by subsequent designation of Didier and Séguy (1953: 172) but R. Lucas’ designation is earlier; both nominal species are currently placed in the genus *Lissotes* Westwood, 1855.

Family-group name: LISSOTINI Benesh, 1955. Stem: *Lissotet*-.

***Listriophorus* L.W. Schaufuss, 1872**: 245. Current status: valid genus in STAPHYLINIDAE: PSELAPHINAE: GONIACERITAE: GONIACERINI.

Type species: *Listriophorusfelix* L.W. Schaufuss, 1874, by subsequent monotypy (L.W. Schaufuss 1874: 289).

Family-group name: LISTRIOPHORINI L.W. Schaufuss, 1890. Stem: *Listriophor*-.

***Listrobyctiscus* Voss, 1923**: 511. Current status: valid genus in ATTELABIDAE: RHYNCHITINAE: RHYNCHITITAE: BYCTISCINI: LISTROBYCTISCINA.

Type species: *Rhynchitescorvinus* Pascoe, 1885, by original designation.

Family-group name: LISTROBYCTISCINA Legalov, 2003. Stem: *Listrobyctisc*-.

***Listroderes* Schönherr, 1826**: 158. Current status: valid genus in CURCULIONIDAE: CYCLOMINAE: LISTRODERINI: LISTRODERINA.

Type species: *Listroderescostirostris* Schönherr, 1826, by original designation.

Family-group name: LISTRODERI J.L. LeConte, 1876. Stem: *Listroder*-.

***Listrus* Motschulsky, 1860a**: 389. Current status: valid genus in MELYRIDAE: DASYTINAE: LISTRINI.

Type species: *Listruspunctatus* Motschulsky, 1860, by subsequent designation (Blaisdell 1938: 27).

Family-group name: LISTRINI Majer, 1990. Stem: *Listr*-.

***Lithinus* Klug, 1833**: 108. Current status: valid genus in CURCULIONIDAE: MOLYTINAE: LITHININI: LITHININA.

Type species: *Lithinussuperciliosus* Klug, 1833, by monotypy. Note: combined description of a new genus-group taxon and a single new species (ICZN 1999a, Article 12.2.6).

Family-group name: LITHINIDES Lacordaire, 1863. Stem: *Lithin*-. Note: senior homonym of LITHININI Forbes, 1948 (type genus *Lithina* Hübner, 1825) in Lepidoptera.

***Lithocharis* Dejean, 1833a**: 65. Current status: valid genus in STAPHYLINIDAE: PAEDERINAE: LATHROBIINI: MEDONINA.

Type species: *Paederus ochraceus* Gravenhorst, 1802, by subsequent designation (C.G. Thomson 1859: 28).

Family-group name: LITHOCHARES Casey, 1905. Stem: *Lithochar*-.

†***Lithocupes* Ponomarenko, 1966**: 65. Current status: valid genus in ARCHOSTEMATA incertae sedis. Note: Upper Triassic (Kyrgystan).

Type species: †*Lithocupesincertus* Ponomarenko, 1966, by original designation.

Family-group name: †LITHOCUPEDINI Ponomarenko, 1969. Stem: *Lithocuped*-.

†***Litholamprima* Nikolajev & Ren, 2015**: 16. Current status: valid genus in LUCANIDAE: †LITHOLAMPRIMINAE. Note: Lower Cretaceous (China).

Type species: †*Litholamprimalongimana* Nikolajev & Ren, 2015, by original designation.

Family-group name: †LITHOLAMPRIMINAE Nikolajev in Nikolajev and Ren, 2015: 16. Stem: *Litholamprim*-.

***Lithophilus* Frölich, 1799**: 11. Current status: invalid senior synonym of *Tetrabrachys* Kapur, 1948 in COCCINELLIDAE: COCCINELLINAE: COCCIDULINI. Note: junior homonym of *Lithophilus* D.H. Schneider, 1791 [Coleoptera: CARABIDAE].

Type species: *Lithophilusruficollis* Frölich, 1799 (= *Tritomaconnata* Panzer, 1796), by monotypy.

Family-group name: LITHOPHILIDAE Imhoff, 1856. Stem: *Lithophil*-. Note: family-group name permanently invalid (ICZN 1999a, Article 39).

†***Lithoscarabaeus* Nikolajev, 1992**: 78. Current status: valid genus in SCARABAEIDAE: †LITHOSCARABAEINAE. Note: Lower Cretaceous (Russia).

Type species: †*Proteroscarabaeusbaissensis* Nikritin,1977, by original designation.

Family-group name: †LITHOSCARABAEINAE Nikolajev, 1992. Stem: *Lithoscarabae*-.

†***Lithostoma* Martynov, 1926**: 13. Current status: valid genus in TROGOSSITIDAE: TROGOSSITINAE: †LITHOSTOMINI. Note: Upper Jurassic (Kazakhstan).

Type species: †*Lithostomaexpansum* Martynov, 1926, by monotypy.

Family-group name: †LITHOSTOMINI Kolibáč & D.-Y. Huang, 2008. Stem: *Lithostom*- (ICZN 1999a, Article 29.4).

***Litoborus* Mulsant & Rey, 1854**: 124, 126. Current status: valid genus in TENEBRIONIDAE: BLAPTINAE: DENDARINI: MELAMBIINA.

Type species: *Philaxmoreletii* P.H. Lucas, 1846, by subsequent designation (Antoine 1931: 181).

Family-group name: LITOBORINAE Antoine, 1941. Stem: *Litobor*-.

***Litosomus* Lacordaire, 1865**: 305. Current status: synonym of *Toxorhinus* Lacordaire, 1865 in CURCULIONIDAE: DRYOPHTHORINAE: LITOSOMINI: LITOSOMINA. Note: First Reviser (ICZN 1999a, Article 24.2.2) for *Toxorhinus* Lacordaire, 1865 versus *Litosomus* Lacordaire, 1865 is Wibmer and O’Brien (1986: 364).

Type species: *Litosomusgrallarius* Lacordaire, 1865, by original designation.

Family-group name: LITOSOMIDES Lacordaire, 1865. Stem: *Litosom*-. Note: junior homonym of LITOSOMIDAE J.W. Douglas & J. Scott, 1865 (type genus *Litosoma* J.W. Douglas & J. Scott, 1865) in Hemiptera.

***Lixus* Fabricius, 1801a**: xi. Current status: valid genus in CURCULIONIDAE: LIXINAE: LIXINI.

Type species: *Curculioparaplecticus* Linnaeus, 1758, by subsequent designation (Latreille 1810: 430). Note: no species were originally included in this genus by Fabricius (1801a); the first ones were the 43 species listed by Fabricius (1801b: 498–507).

Family-group name: LIXIDES Schönherr, 1823. Stem: *Lix*-.

***Ljudmilinius* Legalov, 2003**: 362. Current status: valid genus in ATTELABIDAE: ATTELABINAE: EUOPINI.

Type species: *Euopsfemoralis* Voss, 1924, by original designation.

Family-group name: LJUDMILININA Legalov, 2007. Stem: *Ljudmilin*- (ICZN 1999a, Article 29.4).

†***Lobanoviella* Kirejtshuk & Reid, 2021**: 1895. Current status: valid genus in MEGALOPODIDAE: PALOPHAGINAE: †LOBANOVIELLINI. Note: Eocene (Baltic amber).

Type species: †*Lobanoviellaandreyi* Kirejtshuk & Reid, 2021, by original designation.

Family-group name: †LOBANOVIELLINI Kirejtshuk & Reid, 2021: 1895. Stem: *Lobanoviell*-.

***Loberonotha* Sen Gupta & Crowson, 1969**: 127. Current status: valid genus in EROTYLIDAE: XENOSCELINAE.

Type species: *Telmatophilusolivascens* Broun, 1893, by original designation.

Family-group name: LOBERONOTHINI Sen Gupta & Crowson, 1969. Stem: *Loberonoth*-.

***Loberus* J.L. LeConte, 1861**: 98. Current status: valid genus in EROTYLIDAE: LOBERINAE.

Type species: *Loberusimpressus* J.L. LeConte, 1863, by subsequent monotypy (J.L. LeConte 1863b: 70).

Family-group name: LOBERINAE Bruce, 1951. Stem: *Lober*-.

***Lobodontus* Chaudoir, 1842**: 841. Current status: valid genus in CARABIDAE: HARPALINAE: LEBIINI: PERICALINA.

Type species: *Lobodontustrisignatus* Chaudoir, 1842, by monotypy.

Family-group name: LOBODONTINI Jeannel, 1949. Stem: *Lobodont*-.

***Lobonyx* Jacquelin du Val, 1859a**: 42. Current status: valid genus in PRIONOCERIDAE.

Type species: *Lagriaaenea* Fabricius, 1798, by original designation. Note: this species, described from “Tanger”, is not the same as *Lagriaaenea* Fabricius, 1787 (= *Lagriaaenea* Fabricius, 1775) described from “insulis Americae.”

Family-group name: LOBONYCHINI Majer, 1987. Stem: *Lobonych*-.

***Loborhynchus* Dejean, 1821**: 90. Current status: invalid senior synonym of *Otiorhynchus* Germar, 1822 in CURCULIONIDAE: ENTIMINAE: OTIORHYNCHITAE: OTIORHYNCHINI. Note: name first published in an available work as a junior synonym and subsequently used as valid before 1961 (e.g., Schönherr 1823: 1145); name suppressed for the Principle of Priority and placed on the Official Index of Rejected and Invalid Generic Names in Zoology (as *Loborhynchus* Schönherr, 1823) in Opinion 982 (ICZN 1972) but the original work, author, and date of publication for this name were corrected in Opinion 2299 (ICZN 2012c).

Type species: to be determined (see ICZN 2012c).

Family-group name: LOBORHYNCHIDES Schönherr, 1823. Stem: *Loborhynch*-. Note: name placed on the Official Index of Rejected and Invalid Family-group Names in Zoology (see ICZN 1972, 2012c); family-group name permanently invalid (ICZN 1999a, Article 39).

***Lobotrachelus* Schönherr, 1838**: 711. Current status: valid genus in CURCULIONIDAE: CONODERINAE: LOBOTRACHELINI.

Type species: *Lobotrachelusvestitus* Rosenschoeld, 1838, by original designation.

Family-group name: LOBOTRACHÉLIDES Lacordaire, 1865. Stem: *Lobotrachel*-.

***Loebliorylon* Ślipiński, 1990**: 81. Current status: valid genus in CERYLONIDAE: LOEBLIORYLONINAE.

Type species: *Loeblioryloncarinatus* Ślipiński, 1990, by original designation.

Family-group name: LOEBLIORYLININAE Ślipiński, 1990. Stem: *Loebliorylon*-.

***Loebliquasis* Dolin, 1997**: 845. Current status: valid genus in ELATERIDAE: NEGASTRIINAE: QUASIMUSINI: LOEBLIQUASINA.

Type species: *Loebliquasisburkhardti* Dolin, 1997, by original designation.

Family-group name: LOEBLIQUASINA Schimmel & Tarnawski, 2009. Stem: *Loebliquas*-.

***Loensus* R. Lucas, 1920**: 380, 489. Current status: valid genus in TENEBRIONIDAE: BLAPTINAE: PEDININI: PEDININA. Note: new replacement name for *Pedinopsis* Gebien, 1910.

Type species: *Pedinopsispilipes* Gebien, 1910, by monotypy (type fixation for *Pedinopsis* Gebien).

Family-group name: LOENSINI C. Koch, 1956. Stem: *Loens*-.

***Lomanoxia* Martínez, 1951**: 29. Current status: valid genus in SCARABAEIDAE: APHODIINAE: EUPARIINI.

Type species: *Eupariacostulata* Harold, 1867, by original designation.

Family-group name: LOMANOXIINI Stebnicka, 1999. Stem: *Lomanoxi*-.

***Lomaptera* Gory & Percheron, 1833**: 19, 43. Current status: valid genus in SCARABAEIDAE: CETONIINAE: SCHIZORHININI: LOMAPTERINA.

Type species [suggested]: *Cetoniapapua* Guérin-Méneville, 1831, by subsequent designation (Valck Lucassen 1961: 1). Note: Gory and Percheron’s work on CETONIINAE was issued in livraisons published on different dates and the only species of *Lomaptera* mentioned in the first livraison (1833), where the genus is described, is *latreillei* [as *latreille*] and that species is the type species by monotypy since it is a combined description of a new genus and a single new species (ICZN 1999a, Article 12.2.6); that species; however, is currently included in the genus *Ischiopsopha* Gestro, 1874; Chevrolat (1846a: 428) selected *Lomapterafasciata* Burmeister, 1842 as the type species, but it is not an originally included nominal species; the current concept of the genus is based on the type species *Cetoniapapua* Guérin-Méneville, 1831, which is not an originally included nominal species, as designated by Valck Lucassen (1961: 1); an application to the Commission is necessary to fix *Cetoniapapua* Guérin-Méneville, 1831 as the type species of *Lomaptera* Gory & Percheron, 1833 in order to promote stability.

Family-group name: LOMAPTERIDAE Burmeister, 1842. Stem: *Lomapter*-.

***Lomechusa* Gravenhorst, 1806**: 178. Current status: valid genus in STAPHYLINIDAE: ALEOCHARINAE: LOMECHUSINI: LOMECHUSINA.

Type species: *Staphylinusemarginatus* Paykull, 1789, by subsequent designation (Latreille 1810: 427).

Family-group name: LOMECHUSIDAE Fleming, 1821. Stem: *Lomechus*-.

***Loncophorus* Chevrolat, 1832c**: 215. Current status: valid genus in CURCULIONIDAE: CURCULIONINAE: ANTHONOMINI.

Type species: *Loncophorusobliquus* Chevrolat, 1832, by subsequent designation (Pierce 1916: 467).

Family-group name: LONCOPHORINI Pierce, 1916. Stem: *Loncophor*-.

***Longitarsus* Latreille, 1829b**: 155. Current status: valid genus in CHRYSOMELIDAE: GALERUCINAE: ALTICINI.

Type species: *Chrysomelaatricilla* Linnaeus, 1761, by subsequent designation (Maulik 1926: 333).

Family-group name: LONGITARSES Maehler, 1850. Stem: *Longitars*-.

***Lopheros* J.L. LeConte, 1881b**: 23. Current status: valid genus in LYCIDAE: EROTINAE: EROTINI.

Type species: *Omalisusfraternus* Randall, 1838, by monotypy.

Family-group name: LOPHEROTINI Kazantsev, 2012b: 399. Stem: *Lopherot*-.

***Lophioderus* Casey, 1897**: 356. Current status: valid genus in STAPHYLINIDAE: SCYDMAENINAE: SCYDMAENITAE: STENICHNINI.

Type species: *Lophioderusarcifer* Casey, 1897, by subsequent designation (H. Franz 1985: 150).

Family-group name: LOPHIODERINI Casey, 1897. Stem: *Lophioder*-.

***Lophocateres* Olliff, 1883**: 180. Current status: valid genus in LOPHOCATERIDAE.

Type species: *Lophocateresnanus* Olliff, 1883 (= *Peltispusilla* Klug, 1833), by monotypy.

Family-group name: LOPHOCATERINAE Crowson, 1964. Stem: *Lophocater*-. Note: nomen protectum (see Bouchard et al. 2011: 343).

***Lophotus* Schönherr, 1834a**: 314. Current status: junior synonym of *Psuchocephalus* Latreille, 1828 in CURCULIONIDAE: CYCLOMINAE: ATERPINI: ATERPINA. Note: junior homonym of *Lophotus* Giorna, 1809 [Pisces].

Type species: *Lophotuseschscholtzi* Boheman, 1834, by original designation.

Family-group name: LOPHOTIDES Jekel, 1865. Stem: *Lophot*-. Note: family-group name permanently invalid (ICZN 1999a, Article 39).

***Lordops* Schönherr, 1823**: 1142. Current status: valid genus in CURCULIONIDAE: ENTIMINAE: ENTIMITAE: LORDOPINI.

Type species: *Curculioschoenherri* Dalman, 1823, by original designation.

Family-group name: LORDOPIDES Schönherr, 1823. Stem: *Lordop*-.

***Loricera* Latreille, 1802b**: 88. Current status: valid genus in CARABIDAE: LORICERINAE.

Type species: *Carabuspilicornis* Fabricius, 1775, by monotypy.

Family-group name: LORICERIDES Bonelli, 1810. Stem: *Loricer*-.

***Loubacantus* Bonadona, 1959**: 1033. Current status: junior synonym of *Entypodera* Gerstaecker, 1871 in TENEBRIONIDAE: LAGRIINAE: LAGRIINI: STATIRINA.

Type species: *Loubacantusgiganteus* Bonadona, 1959, by original designation.

Family-group name: LOUBACANTINI Bonadona, 1959. Stem: *Loubacant*-.

***Lovricia* Pretner, 1979**: 383. Current status: valid genus in CARABIDAE: TRECHINAE: LOVRICIINI.

Type species: *Lovriciajalzini* Pretner, 1979, by monotypy.

Family-group name: LOVRICIINA Giachino, Guéorguiev & Vailati, 2011: 68. Stem: *Lovrici*-.

***Loxandrus* J.L. LeConte, 1853a**: 226, 250. Current status: valid genus in CARABIDAE: HARPALINAE: ABACETINI: LOXANDRINA. Note: new replacement name for *Megalostylus* Chaudoir, 1842.

Type species: *Feroniaerratica* Dejean, 1828, by **present designation**. Note: the type species currently recognized for this genus, *Feroniarecta* Say, 1823 by subsequent designation of Casey (1918: 325), is not an originally included species in *Megalostylus* Chaudoir, 1842 and thus cannot be the type species of *Loxandrus* since this name is a new replacement name for *Megalostylus*.

Family-group name: LOXANDRINA Erwin & Sims, 1984. Stem: *Loxandr*-.

***Loxomerus* Chaudoir, 1842**: 851. Current status: valid genus in CARABIDAE: MIGADOPINAE: MIGADOPINI: MIGADOPINA.

Type species: *Loxomerusnebrioides* Chaudoir, 1842, by monotypy.

Family-group name: LOXOMERIFORMES Erwin, 1985. Stem: *Loxomer*-.

***Lucanus* Scopoli, 1763**: 1. Current status: valid genus in LUCANIDAE: LUCANINAE: LUCANINI.

Type species: *Scarabaeuscervus* Linnaeus, 1758, by subsequent designation (Latreille 1810: 429).

Family-group name: LUCANIDES Latreille, 1804. Stem: *Lucan*-.

***Lucidota* Laporte, 1833c**: 136. Current status: valid genus in LAMPYRIDAE: LAMPYRINAE: LUCIDOTINI: LUCIDOTINA.

Type species: *Lucidotabanoni* Laporte, 1833, by subsequent designation (de Motschulsky 1853a: 41).

Family-group name: LUCIDOTIDES Lacordaire, 1857. Stem: *Lucidot*-.

***Luciola* Laporte, 1833c**: 146. Current status: valid genus in LAMPYRIDAE: LUCIOLINAE: LUCIOLINI.

Type species: to be determined. Note: the type species frequently listed for this genus (e.g., McDermott 1964: 43, Calder 1998: 178, Ballantyne et al. 2019: 87) is “*Luciolapedemontana* Bonelli” as designated subsequently by de Motschulsky (1853a: 53); however, “*Luciolapedemontana* Bonelli” (= *Luciolapedemontana* Curtis, 1846) was not an available name when originally included in *Luciola* by Laporte and cannot be selected as the type species (ICZN 1999a, Article 67.2.1); other authors (e.g., Fanti 2022a: 169) have used *Cantharisitalica* Linnaeus, 1758 as the type species of this genus, as designated by de Motschulsky (1853a: 53); however, Motschulsky clearly selected the unavailable name “*Luciolapedemontana* Bonelli” as the type of *Luciola*; E. Desmarest (1860: 14) subsequently designated “*Luciolaitalica* Fab.” as the type species for *Luciola*; however, the species selected by E. Desmarest is a misidentification (*Cantharisitalica* Linnaeus, 1758 sensu Fabricius, 1775 = *Lampyrislusitanica* Charpentier, 1825); accordingly, following the recommendations in Article 70.3 (ICZN 1999a), authors working on this issue will determine if the nominal species previously cited as the type by E. Desmarest or the taxonomic species actually involved should be fixed as the type species of *Luciola* Laporte (L. Ballantyne, pers. comm. to P.B., 2023).

Family-group name: LUCIOLIDES Lacordaire, 1857. Stem: *Luciol*-.

***Ludioctenus* Fairmaire, 1893**: lxviii. Current status: junior synonym of *Tetrigus* Candèze, 1857 in ELATERIDAE: AGRYPNINAE: HEMIRHIPINI: LUDIOCTENINA.

Type species: *Ludioctenusakbesianus* Fairmaire, 1893 (= *Tetriguscyprius* Baudi di Selve, 1871), by monotypy.

Family-group name: LUDIOCTENINA Jakobson, 1913. Stem: *Ludiocten*-.

***Ludius* Berthold, 1827**: 336. Current status: junior synonym of *Elater* Linnaeus, 1758 in ELATERIDAE: ELATERINAE: ELATERINI.

Type species: *Elaterferrugineus* Linnaeus, 1758, by monotypy.

Family-group name: LUDIIDES Lacordaire, 1857. Stem: *Ludi*-. Note: senior homonym of LUDIINI Aurivillius, 1904 (type genus *Ludia* Wallengren, 1865) in Lepidoptera.

***Lulua* Burgeon, 1931**: 92. Current status: valid genus in CERAMBYCIDAE: PRIONINAE: MEROSCELISINI.

Type species: *Luluasquamosa* Burgeon, 1931, by monotypy.

Family-group name: LULUINA Gilmour, 1956. Stem: *Lulu*-.

***Lunilla* Jeannel, 1957**: 110. Current status: valid genus in STAPHYLINIDAE: PSELAPHINAE: CLAVIGERITAE: LUNILLINI.

Type species: *Lunillasaprinoides* Jeannel, 1957, by original designation.

Family-group name: LUNILLINI Célis, 1969. Stem: *Lunill*-.

***Luperaltica* Crotch, 1873b**: 70. Current status: valid genus in CHRYSOMELIDAE: GALERUCINAE: ALTICINI.

Type species: *Alticasenilis* Say, 1824, by subsequent designation (Wilcox 1953: 56).

Family-group name: LUPERALTICINI Leng, 1920. Stem: *Luperaltic*-.

***Luperca* Laporte, 1840a**: 63. Current status: valid genus in CARABIDAE: SIAGONINAE: LUPERCINI.

Type species: *Siagonaherculeana* Laporte, 1835 (= *Carabuslaevigatus* Fabricius, 1781), by monotypy.

Family-group name: LUPERCINI Lecordier, 1977. Stem: *Luperc*-.

***Luperus* Geoffroy, 1762**: 230. Current status: valid genus in CHRYSOMELIDAE: GALERUCINAE: LUPERINI. Note: name attributed to Geoffroy, 1762 and placed on the Official List of Generic Names in Zoology (ICZN 1984a).

Type species: *Chrysomelaflavipes* Linnaeus, 1767, by plenary power (see ICZN 1984a). Note: name placed on the Official List of Specific Names in Zoology (ICZN 1984a).

Family-group name: LUPERIIDAE Gistel, 1848. Stem: *Luper*-.

***Luprops* Hope, 1833**: 63. Current status: valid genus in TENEBRIONIDAE: LAGRIINAE: LUPROPINI.

Type species: *Lupropschrysophthalmus* Hope, 1833 (= *Tageniaindica* Wiedemann, 1823), by monotypy.

Family-group name: LUPROPINI Lesne, 1926. Stem: *Luprop*-.

***Luscosmodicum* Martins, 1970**: 47. Current status: valid genus in CERAMBYCIDAE: CERAMBYCINAE: LUSCOSMODICINI.

Type species: *Luscosmodicumbeaveri* Martins, 1970, by original designation.

Family-group name: LUSCOSMODICINI Martins, 2003. Stem: *Luscosmodic*-.

***Lutrochus* Erichson, 1847a**: 509. Current status: valid genus in LUTROCHIDAE.

Type species: *Lutrochuspilula* Erichson, 1847, by monotypy.

Family-group name: LUTROCHIDAE Kasap & Crowson, 1975. Stem: *Lutroch*-.

***Lycoperdina* Latreille, 1807**: 73. Current status: valid genus in ENDOMYCHIDAE: LYCOPERDININAE.

Type species: *Gallerucabovistae* Fabricius, 1792, by subsequent designation (Latreille 1810: 432).

Family-group name: LYCOPERDINADAE Bromhead, 1838. Stem: *Lycoperdin*-.

***Lycoprogenthes* Pic, 1915b**: 6. Current status: valid genus in LYCIDAE: METRIORRHYNCHINAE: LYCOPROGENTHINI. Note: the original spelling was *Lycoprogentes* but the incorrect subsequent spelling *Lycoprogenthes* is in prevailing usage, attributed to the original publication, and therefore is to be treated as the correct original spelling (ICZN 1999a, Article 33.3.1).

Type species: *Lycoprogenthespouilloni* Pic, 1915, by monotypy.

Family-group name: LYCOPROGENTHINI Bocák & Bocáková, 2008. Stem: *Lycoprogenth*-.

***Lycoptis* Casey, 1890b**: 311. Current status: valid genus in LOPHOCATERIDAE.

Type species: *Lycoptisvillosa* Casey, 1890 (= *Peltisamericana* Motschulsky, 1863), by monotypy.

Family-group name: LYCOPTINI Casey, 1890. Stem: *Lycopt*-. Note: nomen oblitum (see Bouchard et al. 2011: 343).

***Lyctus* Fabricius, 1792b**: 502. Current status: valid genus in BOSTRICHIDAE: LYCTINAE: LYCTINI.

Type species: *Lyctuscanaliculatus* Fabricius, 1792 (= *Dermesteslinearis* Goeze, 1777), by subsequent designation (Latreille 1810: 431). Note: Goeze’s (1777) work is not consistently binominal (see ICZN 2021a) and therefore an application to the Commission is necessary to retain *Dermesteslinearis* Goeze, 1777 as the valid name for the type species.

Family-group name: LYCTIDES Billberg, 1820. Stem: *Lyct*-.

***Lycus* Fabricius, 1787**: 163. Current status: valid genus in LYCIDAE: LYCINAE: LYCINI.

Type species: *Lampyrislatissima* Linnaeus, 1767, by subsequent designation (Blanchard in Audouin et al. 1842: pl. 32).

Family-group name: LYCUSIDAE Laporte, 1838. Stem: *Lyc*-.

***Lydus* Dejean, 1821**: 75. Current status: valid genus in MELOIDAE: MELOINAE: LYTTINI.

Type species: *Meloealgiricus* Linnaeus, 1758, by subsequent designation (Blanchard in Audouin et al. 1841: pl. 54).

Family-group name: LYDINA Kaszab, 1959. Stem: *Lyd*-. Note: junior homonym of LYDIDAE Newman, 1834 (type genus *Lyda* Fabricius, 1805) in Hymenoptera.

***Lygistopterus* Dejean, 1833b**: 98. Current status: valid genus in LYCIDAE: CALOCHROMINAE. Note: this name is often credited to Mulsant (1838: 79) in the literature (e.g., Bocáková and Bocák 2007: 224) but it was made available earlier by Dejean (1833b) (see Bousquet and Bouchard 2013a: 26).

Type species: *Cantharissanguineus* Linnaeus, 1758, by subsequent designation (Chevrolat 1846a: 515).

Family-group name: LYGISTOPTERI J.L. LeConte, 1881. Stem: *Lygistopter*-.

***Lygophilus* Rafinesque, 1815**: 114. Current status: junior synonym of *Epitragus* Latreille, 1802 in TENEBRIONIDAE: PIMELIINAE: EPITRAGINI. Note: unnecessary new replacement name for *Epitragus* Latreille, 1802.

Type species: *Epitragusfuscus* Latreille, 1804, by subsequent monotypy (Latreille 1804b: 322) (type fixation for *Epitragus* Latreille).

Family-group name: LYGOPHILIA Rafinesque, 1815. Stem: *Lygophil*-.

***Lygrus* Fåhraeus, 1872**: 55. Current status: junior synonym of *Pelossus* J. Thomson, 1864 in CERAMBYCIDAE: CERAMBYCINAE: LYGRINI.

Type species: *Lygrusapicalis* Fåhraeus, 1872, by monotypy.

Family-group name: LYGRINI Sama, 2008. Stem: *Lygr*-.

***Lymantes* Schönherr, 1838**: 1085. Current status: valid genus in CURCULIONIDAE: MOLYTINAE: LYMANTINI: LYMANTINA.

Type species: *Lymantesscrobicollis* Gyllenhal, 1838, by original designation.

Family-group name: LYMANTIDES Lacordaire, 1865. Stem: *Lymant*-.

***Lymexylon* Fabricius, 1775**: 204. Current status: valid genus in LYMEXYLIDAE: LYMEXYLINAE.

Type species: *Cantharisnavalis* Linnaeus, 1758, by subsequent designation (Latreille 1810: 427).

Family-group name: LYMOXYLONIDAE Fleming, 1821. Stem: *Lymexyl*-.

***Lymnastis* Motschulsky, 1862**: 27. Current status: valid genus in CARABIDAE: TRECHINAE: TACHYINI: TACHYINA. Note: Motschulsky used two original spellings, including *Lymastis* (p. 31); subsequently, de Motschulsky (1868c: 9) used only the spelling *Lymnastis* and therefore acted as First Reviser (ICZN 1999a, Article 24.2.4).

Type species: *Lymnaeumindicum* Motschulsky, 1851, by subsequent designation (Casey 1918: 3).

Family-group name: LIMNASTINI Jeannel, 1937. Stem: *Lymnast*-.

***Lypesthes* Baly, 1863**: 152. Current status: junior synonym of *Fidia* Motschulsky, 1860 in CHRYSOMELIDAE: EUMOLPINAE: BROMIINI.

Type species: *Fidiaatra* Motschulsky, 1861, by original designation.

Family-group name: LYPESTHINI Chûjô, 1956. Stem: *Lypesthet*-.

***Lypnea* Baly, 1876**: 446. Current status: valid genus in CHRYSOMELIDAE: GALERUCINAE: ALTICINI.

Type species: *Lypneaflava* Baly, 1876, by monotypy.

Family-group name: LYPNEINA Bechyné, 1968. Stem: *Lypne*-.

***Lyponia* C.O. Waterhouse, 1878**: 99. Current status: valid genus in LYCIDAE: ATELIINAE: LYPONIINI.

Type species: *Lyponiadebilis* C.O. Waterhouse, 1878, by original designation.

Family-group name: LYPONIININA Bocák & Bocáková, 1990. Stem: *Lyponi*-.

***Lyprus* Schönherr, 1826**: 21, 288. Current status: junior synonym of *Bagous* Germar, 1817 in CURCULIONIDAE: BAGOINAE.

Type species: *Curculiocylindrus* Paykull, 1800 (= *Bagoustubulus* Caldara & O’Brien, 1994), by original designation.

Family-group name: LYPRIIDAE Gistel, 1848. Stem: *Lypr*-. Note: nomen oblitum (see Bouchard et al. 2011: 586).

***Lyropaeus* C.O. Waterhouse, 1878**: 104. Current status: valid genus in LYCIDAE: LYROPAEINAE: LYROPAEINI.

Type species: *Lycusfallax* Walker, 1858, by original designation.

Family-group name: LYROPAEINI Bocák & Bocáková, 1989. Stem: *Lyropae*-.

***Lyrosoma* Mannerheim, 1853**: 174. Current status: valid genus in AGYRTIDAE: AGYRTINAE.

Type species: *Pterolomapallidum* Eschscholtz, 1829, by subsequent designation (Hatch 1928: 71).

Family-group name: LYROSOMINI G.H. Horn, 1880. Stem: *Lyrosomat*-.

***Lystronychus* Latreille, 1829b**: 41. Current status: valid genus in TENEBRIONIDAE: ALLECULINAE: ALLECULINI: XYSTROPODINA. Note: the original spelling was *Lystronichus* but the incorrect subsequent spelling *Lystronychus* is in prevailing usage, attributed to the publication of the original spelling, and therefore deemed to be the correct original spelling (ICZN 1999a, Article 33.3.1).

Type species: *Helopsequestris* Fabricius, 1775, by subsequent designation (Saunders 1837: 153).

Family-group name: LYSTRONYCHIDES Lacordaire, 1859. Stem: *Lystronych*-.

***Lytta* Fabricius, 1775**: 260. Current status: valid genus in MELOIDAE: MELOINAE: LYTTINI.

Type species: *Meloevesicatorius* Linnaeus, 1758, by subsequent designation (C.G. Thomson 1859: 124).

Family-group name: LYTTIDAE Streubel, 1846. Stem: *Lytt*-.

***Machadous* Jeannel, 1951a**: 105. Current status: valid genus in STAPHYLINIDAE: PSELAPHINAE: GONIACERITAE: MACHADOINI.

Type species: *Machadouspunctatus* Jeannel, 1951, by original designation.

Family-group name: MACHADOINI Jeannel, 1951. Stem: *Machado*-.

***Machaerites* L. Miller, 1855**: 509. Current status: valid genus in STAPHYLINIDAE: PSELAPHINAE: GONIACERITAE: BYTHININI: MACHAERITINA.

Type species: *Machaeritesspelaeus* L. Miller, 1855, by monotypy.

Family-group name: MACHAERITINA Jeannel, 1950. Stem: *Machaerit*-.

***Machla* Herbst, 1799**: 152. Current status: valid genus in TENEBRIONIDAE: PIMELIINAE: ASIDINI. Note: the senior homonym *Machla* Lichtenstein, 1796 was published in a work that was suppressed for nomenclatural purposes (ICZN 1995f).

Type species: *Opatrumvillosum* G.-A. Olivier, 1795, by subsequent designation (R. Lucas 1920: 386).

Family-group name: MACHLINI Chatanay, 1914. Stem: *Machl*-.

***Machozetus* Chaudoir, 1850**: 448. Current status: valid genus in CARABIDAE: HARPALINAE: HARPALINI: DITOMINA. Note: new replacement name for *Harpactes* Ménétriés, 1848.

Type species: *Harpacteslehmanni* Ménétriés, 1848, by monotypy (type fixation for *Harpactes* Ménétriés).

Family-group name: MACHOZETI Schauberger, 1934. Stem: *Machozet*-.

***Macraspis* W.S. MacLeay, 1819**: 156. Current status: valid genus in SCARABAEIDAE: RUTELINAE: RUTELINI: RUTELINA.

Type species: *Cetoniaquadrivittata* G.-A. Olivier, 1789 (= *Scarabaeuscincta* Drury, 1782), by subsequent designation (Crotch 1870b: 235).

Family-group name: MACRASPIDA Perty, 1840. Stem: *Macraspid*-.

***Macratria* Newman, 1838**: 377. Current status: valid genus in ANTHICIDAE: MACRATRIINAE: MACRATRIINI.

Type species: *Macratrialinearis* Newman, 1838 (= *Dircaeamurina* Fabricius, 1801), by monotypy.

Family-group name: MACRATRIINI J.L. LeConte, 1862. Stem: *Macratri*-.

***Macraulacus* Bonvouloir, 1871**: 71. Current status: valid genus in EUCNEMIDAE: MACRAULACINAE: MACRAULACINI. Note: this name was first used by Gemminger in Gemminger and von Harold (1869b: 1474) but is unavailable from that date as the species listed under the name is a nomen nudum; Bonvouloir’s plate 25 referred to by Gemminger was not issued until 1872 (1872a), but before the species description (1872b: 509).

Type species: *Macraulacusexcavatus* Bonvouloir, 1872, by subsequent monotypy (Bonvouloir 1872b: 509).

Family-group name: MACRAULACINAE Fleutiaux, 1923. Stem: *Macraulac*-.

***Macrobasis* J.L. LeConte, 1862**: 272. Current status: valid subgenus of *Epicauta* Dejean, 1834 in MELOIDAE: MELOINAE: EPICAUTINI.

Type species: *Lyttaalbida* Say, 1824, by subsequent designation (Wellman 1910a: 396). Note: no species were originally included in this genus by J.L. LeConte (1862); the first ones were the species listed by J.L. LeConte (1866b: 68).

Family-group name: MACROBASES J.L. LeConte, 1862. Stem: *Macrobase*-.

***Macrodactylus* Dejean, 1821**: 58. Current status: valid genus in SCARABAEIDAE: MELOLONTHINAE: MACRODACTYLINI.

Type species: *Melolonthasubspinosa* Fabricius, 1775, by monotypy.

Family-group name: MACRODACTYLIDAE W. Kirby, 1837. Stem: *Macrodactyl*-.

***Macrodontia* Lacordaire, 1830b**: 171. Current status: valid genus in CERAMBYCIDAE: PRIONINAE: MACRODONTIINI.

Type species: *Cerambyxcervicornis* Linnaeus, 1758, by monotypy.

Family-group name: MACRODONTITAE J. Thomson, 1861. Stem: *Macrodonti*-.

***Macrolinus* Kaup, 1868a**: 18. Current status: valid genus in PASSALIDAE: PASSALINAE: MACROLININI.

Type species: *Passaluslatipennis* Percheron, 1841, by subsequent designation (Gravely 1918: 80).

Family-group name: MACROLINEAE Kaup, 1871. Stem: *Macrolin*-.

***Macrolopha* Weise, 1902b**: 119. Current status: valid genus in MEGALOPODIDAE: MEGALOPODINAE: MEGALOPODINI: MACROLOPHINA.

Type species: *Macrolopharustica* Weise, 1902, by subsequent designation (Rodríguez-Mirón, 2018: 277).

Family-group name: MACROLOPHINA Rodríguez-Mirón in Rodríguez-Mirón et al., 2021: 695. Stem: *Macroloph*-.

***Macrolycinella* Pic, 1922**: 21. Current status: valid genus in LYCIDAE incertae sedis.

Type species: *Macrolycinellabicolor* Pic, 1922, by monotypy.

Family-group name: MACROLYCINELLINI Kazantsev, 2012b: 399. Stem: *Macrolycinell*-.

***Macrolycus* C.O. Waterhouse, 1878**: 96. Current status: valid genus in LYCIDAE: ATELIINAE: MACROLYCINI.

Type species: *Macrolycusbowringii* C.O. Waterhouse, 1878, by monotypy. Note: two original spellings were used by C.O. Waterhouse (1878) for this species, *M.coccineus* (p. 96) and *M.bowringii* (p. 105); subsequently, C.O. Waterhouse (1880a: pl. I, fig. 1) used only *M.bowringii* and therefore acted as First Reviser (ICZN 1999a, Article 24.2.4).

Family-group name: MACROLYCINAE Kleine, 1929. Stem: *Macrolyc*-.

***Macroma* Gory & Percheron, 1833**: 19, 35, 53. Current status: junior synonym of *Campsiura* Hope, 1831 in SCARABAEIDAE: CETONIINAE: CREMASTOCHEILINI: MACROMINA.

Type species: *Cetoniascutellata* Fabricius, 1801, by subsequent designation (Hope 1837: 63).

Family-group name: MACROMINAE Burmeister & Schaum, 1840. Stem: *Macrom*-.

***Macrones* Newman, 1841**: 33. Current status: valid genus in CERAMBYCIDAE: CERAMBYCINAE: MACRONINI.

Type species: *Macronesexilis* Newman, 1841, by monotypy.

Family-group name: MACRONIDES Lacordaire, 1868. Stem: *Macron*-.

***Macronota* Hoffmannsegg, 1817**: 15. Current status: valid genus in SCARABAEIDAE: CETONIINAE: SCHIZORHININI: LOMAPTERINA.

Type species: *Macronotaanthracina* Wiedemann, 1823 (= *Cetonianigrita* Fabricius, 1775), by subsequent designation (Krikken 2018: 21, valid name of type species given as “*Cetonianigrita* Fröhlich, 1792”). Note: no species were originally included in this genus by Hoffmannsegg (1817); the first ones were the three species listed by Wiedemann (1823a: 83, 84).

Family-group name: MACRONOTIDAE Burmeister, 1842. Stem: *Macronot*-.

***Macronychus* P.W.J. Müller, 1806**: 209. Current status: valid genus in ELMIDAE: ELMINAE: MACRONYCHINI.

Type species: *Macronychusquadrituberculatus* P.W.J. Müller, 1806, by monotypy.

Family-group name: MACRONYCHIDAE Gistel, 1848. Stem: *Macronych*-.

***Macrophylla* Hope, 1837**: 103. Current status: junior synonym of *Aegostheta* Dejean, 1833 in SCARABAEIDAE: MELOLONTHINAE: TANYPROCTINI: MACROPHYLLINA.

Type species: *Melolonthalongicornis* Fabricius, 1787, by original designation.

Family-group name: MACROPHYLLIDAE Burmeister, 1855. Stem: *Macrophyll*-. Note: senior homonym of MACROPHYLLINA J.E. Gray, 1866 (type genus *Macrophyllum* J.E. Gray, 1838) in Mammalia.

***Macroplea* Samouelle, 1819**: 211. Current status: valid genus in CHRYSOMELIDAE: DONACIINAE: HAEMONIINI.

Type species: *Donaciazosterae* Fabricius, 1801 (= *Rhagiummuticum* Fabricius, 1792), by subsequent designation (Curtis 1830: pl. 318).

Family-group name: MACROPLEINI Lopatin, 1977. Stem: *Macrople*-.

***Macropnus* G.H. Horn, 1867**: 397. Current status: junior synonym of *Macropoides* Guérin-Méneville, 1844 in SCARABAEIDAE: RUTELINAE: RUTELINI: HETEROSTERNINA.

Type species: *Macropnuscrassipes* G.H. Horn, 1867, by monotypy.

Family-group name: MACROPNI G.H. Horn, 1867. Stem: *Macropn*-. Note: nomen oblitum (see A.B.T. Smith 2006: 171).

***Macropoda* Solier, 1835d**: 512, 515. Current status: valid genus in TENEBRIONIDAE: PIMELIINAE: ADESMIINI.

Type species: *Pimeliavariolaris* G.-A. Olivier, 1795, by subsequent designation (Hope 1841a: 118).

Family-group name: MACROPODOIDAE Erichson, 1846. Stem: *Macropod*-. Note: the authorship of this family-group name was credited to “Agassiz” in Bouchard et al. (2011: 401) but here we follow Bouchard et al. (2021: 9) in crediting new nomenclatural acts in the fascicle on Coleoptera of Agassiz’ ‘Nomenclator Zoologicus’ to the beetle specialist W.F. Erichson, whose contribution to the publication was acknowledged on the fascicle’s title page; nomen oblitum (see Bouchard et al. 2011: 401); junior homonym of MACROPODIDAE J.E. Gray, 1821 (type genus *Macropus* Shaw, 1790) in Mammalia.

***Macropogon* Motschulsky, 1845b**: 38. Current status: valid genus in ARTEMATOPODIDAE: ARTEMATOPODINAE: MACROPOGONINI.

Type species: *Macropogonsibiricum* Motschulsky, 1845, by original designation.

Family-group name: MACROPOGONINI J.L. LeConte, 1861. Stem: *Macropogon*-.

***Macrosiagon* Hentz, 1830**: 462. Current status: valid genus in RIPIPHORIDAE: RIPIPHORINAE: MACROSIAGONINI.

Type species: *Mordelladimidiata* Fabricius, 1781, by monotypy.

Family-group name: MACROSIAGONINI Heyden, 1908. Stem: *Macrosiagon*-.

***Macrostylus* Boheman in Schönherr, 1840a**: 921. Current status: valid genus in CURCULIONIDAE: ENTIMINAE: POLYDRUSITAE: NAUPACTINI: NAUPACTINA.

Type species: *Macrostyluscrinitus* Boheman, 1840, by original designation.

Family-group name: MACROSTYLES J.L. LeConte, 1874. Stem: *Macrostyl*-.

***Macrostyphlus* Kirsch, 1890**: 25. Current status: valid genus in CURCULIONIDAE: CYCLOMINAE: LISTRODERINI: MACROSTYPHLINA.

Type species: *Macrostyphlusgualcalae* Kirsch, 1890, by monotypy.

Family-group name: MACROSTYPHLINA Morrone, 2013: 27. Stem: *Macrostyphl*-.

***Macrotarrhus* Bedel, 1906**: 93. Current status: valid genus in CURCULIONIDAE: HYPERINAE: HYPERINI: MACROTARRHUSINA. Note: new replacement name for *Macrotarsus* Schönherr, 1842.

Type species: *Macrotarsusbartelsii* Schönherr, 1842, by original designation (type fixation for *Macrotarsus* Schönherr).

Family-group name: MACROTARRHUSINA Legalov, 2007. Stem: *Macrotarrhus*- (ICZN 1999a, Article 29.4).

***Macrotoma* Audinet-Serville, 1832**: 137. Current status: valid genus in CERAMBYCIDAE: PRIONINAE: MACROTOMINI: MACROTOMINA. Note: nomen protectum (see Heffern et al. 2006).

Type species: *Prionuspalmatus* Fabricius, 1792, by subsequent designation (E. Desmarest 1860: 307). Note: the type species often listed in the literature is *Prionusserripes* Fabricius, 1781 (e.g., Drumont and Komiya 2010: 90, 2020: 111) by subsequent designation of J. Thomson (1864: 299) but E. Desmarest’s designation is earlier; both species are currently included in the genus *Macrotoma* Audinet-Serville, 1832.

Family-group name: MACROTOMITAE J. Thomson, 1861. Stem: *Macrotom*-.

***Mada* Mulsant, 1850**: 1095. Current status: valid genus in COCCINELLIDAE: COCCINELLINAE: EPILACHNINI.

Type species: *Epilachnafraterna* Mulsant, 1850, by subsequent designation (Korschefsky 1931: 68).

Family-group name: MADAINI Gordon, 1975. Stem: *Mad*-.

***Madagaster* Perkins, 1997**: 179. Current status: valid genus in HYDRAENIDAE: HYDRAENINAE: MADAGASTRINI.

Type species: *Madagastersteineri* Perkins, 1997, by original designation.

Family-group name: MADAGASTRINI Perkins, 1997. Stem: *Madagastr*-.

***Madarus* Schönherr, 1825**: 584. Current status: valid genus in CURCULIONIDAE: BARIDINAE: MADARINI: MADARINA.

Type species: *Calandracorvina* Fabricius, 1801, by original designation.

Family-group name: MADARIDES Jekel, 1865. Stem: *Madar*-.

*Madeirodula Szawaryn, Větrovec & Tomaszewska, 2020: 7. Current status: valid genus in COCCINELLIDAE: MICROWEISEINAE: *MADEIRODULINI. Note: published in a work that is nomenclaturally unavailable (ICZN 2012f, Article 8.5.3).

Type species: *Madeirodulaatlantica Szawaryn, Větrovec & Tomaszewska, 2020, by original designation. Note: published in a work that is nomenclaturally unavailable (ICZN 2012f, Article 8.5.3).

Family-group name: *MADEIRODULINI Szawaryn, Větrovec & Tomaszewska, 2020: 5. Stem: *Madeirodul*-. Note: published in a work that is nomenclaturally unavailable (ICZN 2012f, Article 8.5.3).

***Madopterus* Schönherr, 1836**: 734. Current status: valid genus in CURCULIONIDAE: BARIDINAE: BARIDINI: MADOPTERINA.

Type species: *Madapterustalpa* Gyllenhal, 1836, by original designation.

Family-group name: MADOPTÉRIDES Lacordaire, 1865. Stem: *Madopter*-.

***Maechidius* W.S. MacLeay, 1819**: 140. Current status: valid genus in SCARABAEIDAE: SERICOIDINAE: MAECHIDIINI.

Type species: *Troxspurius* W. Kirby, 1819, by monotypy.

Family-group name: MAECHIDIINA Burmeister, 1855. Stem: *Maechidi*-.

***Magdalinus* Germar in Schönherr, 1843**: 135. Current status: junior synonym of *Magdalis* Germar, 1817 in CURCULIONIDAE: MESOPTILIINAE: MAGDALIDINI. Note: unnecessary new replacement name for *Thamnophilus* Schönherr, 1823 (an unnecessary replacement name for *Magdalis* Germar, 1817); name placed on the Official Index of Rejected and Invalid Names in Zoology (see ICZN 1955a).

Type species: *Curculioviolaceus* Linnaeus, 1758, by plenary power (see ICZN 1955a) (type fixation for *Magdalis* Germar).

Family-group name: MAGDALINIDAE Gistel, 1856. Stem: *Magdalin*-. Note: family-group name permanently invalid (ICZN 1999a, Article 39).

***Magdalis* Germar, 1817b**: 340. Current status: valid genus in CURCULIONIDAE: MESOPTILIINAE: MAGDALIDINI. Note: name placed on the Official List of Generic Names in Zoology (ICZN 1955a).

Type species: *Curculioviolaceus* Linnaeus, 1758, by plenary power (see ICZN 1955a). Note: name placed on the Official List of Specific Names in Zoology (ICZN 1955a).

Family-group name: MAGDALINAE Pascoe, 1870. Stem: *Magdalid*-. Note: nomen protectum (see Bouchard et al. 2011: 622).

†***Magnocoleus* Hong, 1998**: 41. Current status: valid genus in †MAGNOCOLEIDAE. Note: Lower Cretaceous (China); type genus placed as incertae sedis within CUPEDOIDEA by Cai et al. (2022).

Type species: †*Magnocoleushuangjiapuensis* Hong, 1998, by original designation.

Family-group name: †MAGNOCOLEIDAE Hong, 1998. Stem: *Magnocole*-.

***Malachius* Fabricius, 1775**: 207. Current status: valid genus in MELYRIDAE: MALACHIINAE: MALACHIINI.

Type species: *Cantharisaenea* Linnaeus, 1758, by subsequent designation (Latreille 1810: 426).

Family-group name: MALACHIUSIDAE Fleming, 1821. Stem: *Malachi*-.

***Malchinus* Kiesenwetter, 1863**: 726. Current status: junior synonym of *Macrocerus* Motschulsky, 1845 in CANTHARIDAE: MALTHININAE: MALCHININI.

Type species: *Malthodessinuatocollis* Kiesenwetter, 1852, by subsequent designation (Delkeskamp 1977: 356).

Family-group name: MALCHININI Brancucci, 1980. Stem: *Malchin*-.

***Mallaspis* Audinet-Serville, 1832**: 188. Current status: valid genus in CERAMBYCIDAE: PRIONINAE: MALLASPINI.

Type species: *Prionusscutellaris* G.-A. Olivier, 1795, by monotypy.

Family-group name: MALLASPITAE J. Thomson, 1861. Stem: *Mallasp*-.

***Mallodon* Lacordaire, 1830b**: 171. Current status: valid genus in CERAMBYCIDAE: PRIONINAE: MACROTOMINI: MALLODONINA.

Type species: *Cerambyxspinibarbis* Linnaeus, 1758, by monotypy.

Family-group name: MALLODONITAE J. Thomson, 1861. Stem: *Mallodon*-.

***Mallodrya* G.H. Horn, 1888**: 42. Current status: valid genus in SYNCHROIDAE.

Type species: *Mallodryasubaenea* G.H. Horn, 1888, by monotypy.

Family-group name: MALLODRYINI G.H. Horn, 1888. Stem: *Mallodry*-.

***Malthinus* Latreille, 1805**: 261. Current status: valid genus in CANTHARIDAE: MALTHININAE: MALTHININI.

Type species [suggested]: *Malthinusflavus* Latreille, 1805 (= *Cantharisflaveola* Herbst, 1786), by subsequent designation (Hope 1841a: 141). Note: the valid type species fixation is that of J.E. Gray (1832: 723), who selected *Malthinusmarginatus* Latreille, 1805, but that species is currently placed in the genus *Malthodes* Kiesenwetter, 1852; an application to the Commission is necessary to fix *Malthinusflavus* Latreille, 1805 as the type species of *Malthinus* Latreille, 1805.

Family-group name: MALTHINEN Kiesenwetter, 1852. Stem: *Malthin*-.

***Malthodes* Kiesenwetter, 1852**: 242, 265. Current status: valid genus in CANTHARIDAE: MALTHININAE: MALTHODINI.

Type species [suggested]: *Malthinusmarginatus* Latreille, 1805, by subsequent designation (Brancucci 1980: 307). Note: the valid type species fixation is that of E. Desmarest (1860: 5), who selected *Cantharissanguinolenta* Linnaeus, 1767 (= *Meloecinnaberinus* Scopoli, 1763), but that species is currently placed in the genus *Cucujus* Fabricius, 1775; presumably E. Desmarest’s type species was based on a misidentification but the taxonomic species actually involved is unknown; an application to the Commission is necessary to fix *Malthinusmarginatus* Latreille, 1805 as the type species of *Malthodes* Kiesenwetter, 1852.

Family-group name: MALTHODINAE Böving & Craighead, 1931. Stem: *Malthod*-.

***Malvapion* Hoffmann, 1958**: 1471, 1503. Current status: valid genus in BRENTIDAE: APIONINAE: ASPIDAPIITAE: MALVAPIINI.

Type species: *Curculiomalvae* Fabricius, 1775, by monotypy.

Family-group name: MALVAPIINI Alonso-Zarazaga, 1990. Stem: *Malvapi*-.

***Manda* Blackwelder, 1952**: 230. Current status: valid genus in STAPHYLINIDAE: OXYTELINAE: PLANEUSTOMINI. Note: new replacement name for *Acrognathus* Erichson, 1839.

Type species: *Omaliummandibulare* Gyllenhal, 1827, by subsequent designation (Duponchel 1841b: 57) (type fixation for *Acrognathus* Erichson).

Family-group name: MANDINI Gildenkov, 2003. Stem: *Mand*-.

***Mandelschtamius* Legalov, 2003**: 107. Current status: valid genus in ATTELABIDAE: RHYNCHITINAE: RHYNCHITITAE: AULETINI: MANDELSCHTAMIINA.

Type species: *Mandelschtamiusturneroides* Legalov, 2003, by original designation.

Family-group name: MANDELSCHTAMIINA Legalov, 2003. Stem: *Mandelschtami*-.

***Manobia* Jacoby, 1885**: 73. Current status: valid genus in CHRYSOMELIDAE: GALERUCINAE: ALTICINI.

Type species: *Manobianigripennis* Jacoby, 1885, by subsequent designation (Maulik 1926: 407).

Family-group name: MANOBIINI Bechyné & Špringlová de Bechyné, 1975. Stem: *Manobi*-.

***Manticora* Fabricius, 1781**: 320. Current status: valid genus in CICINDELIDAE: MANTICORINI.

Type species: *Manticoramaxillosa* Fabricius, 1781 (= *Carabustuberculatus* DeGeer, 1778), by monotypy.

Family-group name: MANTICORIDAE Laporte, 1834. Stem: *Manticor*-.

***Maoraxia* Obenberger, 1937**: 1449. Current status: valid genus in BUPRESTIDAE: BUPRESTINAE: MAORAXIINI. Note: new replacement name for *Maoriella* Obenberger, 1924.

Type species: *Maoriellanovaezeelandiae* Obenberger, 1924, by original designation (type fixation for *Maoriella* Obenberger).

Family-group name: MAORAXIINI Holyński, 1984. Stem: *Maoraxi*-.

***Maorothius* Assing, 2000**: 22. Current status: valid genus in STAPHYLINIDAE: STAPHYLININAE: MAOROTHIINI.

Type species: *Othiusadustus* Broun, 1880, by original designation.

Family-group name: MAOROTHIINI Assing, 2000. Stem: *Maorothi*-.

***Marcapatia* Bechyné, 1958**: 653. Current status: valid genus in CHRYSOMELIDAE: GALERUCINAE: ALTICINI.

Type species: *Crepidoderalongicornis* Jacoby, 1904, by original designation.

Family-group name: MARCAPATIINI Bechyné & Špringlová de Bechyné, 1975. Stem: *Marcapati*-.

***Marcepania* Jałoszyński, 2013**: 177. Current status: valid genus in STAPHYLINIDAE: SCYDMAENINAE: CEPHENNIITAE: MARCEPANIINI.

Type species: *Marcepaniasemengohensis* Jałoszyński, 2013, by original designation.

Family-group name: MARCEPANIINI Jałoszyński, 2013: 177. Stem: *Marcepani*-.

***Mariouta* Pic, 1898**: 73. Current status: valid genus in DERMESTIDAE: DERMESTINAE: MARIOUTINI.

Type species: *Marioutaletourneuxi* Pic, 1898, by monotypy.

Family-group name: MARIOUTINI Jakobson, 1913. Stem: *Mariout*-.

***Marolia* Mulsant, 1856b**: 84, 92. Current status: valid genus in MELANDRYIDAE: MELANDRYINAE: HYPULINI.

Type species: *Serropalpusvariegatus* Bosc, 1791, by monotypy.

Family-group name: MAROLIINI Portevin, 1934. Stem: *Maroli*-.

***Masoreus* Dejean, 1821**: 15. Current status: valid genus in CARABIDAE: HARPALINAE: MASOREINI.

Type species: *Harpalusluxatus* Audinet-Serville, 1821 (= *Harpaluswetterhallii* Gyllenhal, 1813), by monotypy.

Family-group name: MAZORÉIDES Chaudoir, 1871. Stem: *Masore*-.

***Mastachilus* Kaup, 1868a**: 19. Current status: valid genus in PASSALIDAE: PASSALINAE: MACROLININI. Note: the original spelling *Mastachilus* was changed to *Mastochilus* in the next issue of the journal ‘Coleopterologische Hefte’ (vol. 4, p. 31); therefore, Article 32.5.1.1 (ICZN 1999a) does not apply and the original is to be considered the correct spelling unless *Mastochilus* is in prevailing usage, which is not the case; *Mastochilus* is an unjustified emendation of *Mastachilus*.

Type species: *Passaluspolyphyllus* W.S. MacLeay, 1826, by subsequent designation (Gravely 1918: 97).

Family-group name: MASTACHILINAE Kuwert, 1891. Stem: *Mastachil*-.

***Mastax* Fischer, 1828**: 111. Current status: valid genus in CARABIDAE: BRACHININAE: BRACHININI: MASTACINA.

Type species: *Brachinusthermarum* Steven, 1806, by monotypy.

Family-group name: MASTACINA Erwin, 1970. Stem: *Mastac*-.

***Mastiger* Motschulsky, 1852**: 501. Current status: valid genus in STAPHYLINIDAE: PSELAPHINAE: CLAVIGERITAE: MASTIGERINI.

Type species: *Mastigerabruptus* Motschulsky, 1852, by monotypy.

Family-group name: MASTIGERINI Jeannel, 1954. Stem: *Mastiger*-.

***Mastigocoleus*** Tihelka & Cai in Tihelka et al., 2022: 6. Current status: valid genus in MASTIGOCOLEIDAE.

Type species: *Mastigocoleusresinicola* Tihelka & Cai, 2022, by original designation.

Family-group name: MASTIGOCOLEIDAE Tihelka, Jäch, Kundrata & Cai in Tihelka et al., 2022: 5. Stem: *Mastigocole*-.

***Mastigus* Latreille, 1802b**: 115. Current status: valid genus in STAPHYLINIDAE: SCYDMAENINAE: MASTIGITAE: MASTIGINI.

Type species: *Ptinusspinicornis* Fabricius, 1787, by monotypy.

Family-group name: MASTIGOIDAE Fleming, 1821. Stem: *Mastig*-.

***Mastinocerus* Solier, 1849**: 440. Current status: valid genus in PHENGODIDAE: MASTINOCERINAE.

Type species: *Mastinocerusbrevipennis* Solier, 1849, by monotypy.

Family-group name: MASTINOCERINI J.L. LeConte, 1881. Stem: *Mastinocer*-.

***Mastogenius* Solier, 1849**: 507. Current status: valid genus in BUPRESTIDAE: POLYCESTINAE: HAPLOSTETHINI.

Type species: *Mastogeniusparallelus* Solier, 1849, by monotypy.

Family-group name: MASTOGENINI J.L. LeConte & G. H. Horn, 1883. Stem: *Mastogeni*-.

***Masuria* Cameron, 1928**: 51. Current status: valid genus in STAPHYLINIDAE: ALEOCHARINAE: MASURIINI.

Type species: *Masuriaplumbea* Cameron, 1928, by original designation.

Family-group name: MASURIINI Cameron, 1939. Stem: *Masuri*-.

***Matheteus* J.L. LeConte, 1874b**: 58. Current status: valid genus in OMETHIDAE: MATHETEINAE.

Type species: *Matheteustheveneti* J.L. LeConte, 1874, by monotypy.

Family-group name: MATHETEI J.L. LeConte, 1881. Stem: *Mathete*-.

***Matus* Aubé, 1836**: 42. Current status: valid genus in DYTISCIDAE: MATINAE.

Type species: *Colymbetesbicarinatus* Say, 1823, by subsequent monotypy (Aubé 1837: 189).

Family-group name: MATINI Branden, 1884. Stem: *Mat*-.

***Mauesia* Lane, 1956**: 19. Current status: valid genus in CERAMBYCIDAE: LAMIINAE: MAUESIINI.

Type species: *Mauesiacornuta* Lane, 1956, by monotypy.

Family-group name: MAUESINAE Lane, 1956. Stem: *Mauesi*-.

***Mauia* Blackburn in Blackburn and Sharp, 1885**: 194. Current status: valid genus in ANTHRIBIDAE: ANTHRIBINAE: MAUIINI.

Type species: *Mauiasatelles* Blackburn, 1885 (= *Araeocerussubnotatus* Boheman, 1859), by monotypy.

Family-group name: MAUIINI B.D. Valentine, 1990. Stem: *Maui*-.

***Mauroniscus* Bourgeois, 1911**: 211. Current status: valid genus in MAURONISCIDAE.

Type species: *Mauroniscusroberti* Bourgeois, 1911, by monotypy.

Family-group name: MAURONISCIDAE Majer, 1995. Stem: *Mauronisc*-.

***Mayetia* Mulsant & Rey, 1875**: 87. Current status: valid genus in STAPHYLINIDAE: PSELAPHINAE: EUPLECTITAE: MAYETIINI.

Type species: *Mayetiasphaerifer* Mulsant & Rey, 1875, by monotypy.

Family-group name: MAYETIINI Scheerpeltz, 1925. Stem: *Mayeti*-.

***Maynipeplus* Kirejtshuk, 1998**: 551. Current status: valid genus in NITIDULIDAE: MAYNIPEPLINAE.

Type species: *Maynipepluslomechusoides* Kirejtshuk, 1998, by original designation.

Family-group name: MAYNIPEPLINAE Kirejtshuk, 1998. Stem: *Maynipepl*-.

***Mechistocerus* Fauvel, 1863**: 159. Current status: valid genus in CURCULIONIDAE: CRYPTORHYNCHINAE: AEDEMONINI.

Type species: *Coelosternusimpressus* Montrouzier, 1860, by monotypy.

Family-group name: MECISTOCERINI Morimoto, 1978. Stem: *Mechistocer*-.

***Mecinus* Germar, 1821**: 315. Current status: valid genus in CURCULIONIDAE: CURCULIONINAE: MECININI. Note: nomen protectum (see Caldara 2013: 53); see Alonso-Zarazaga et al. (2023: 8) for an alternative interpretation regarding the author and type species of *Mecinus*, an application to the Commission may be necessary to unambiguously determine the correct author and type species for this genus-group name.

Type species: *Curculiopyraster* Herbst, 1795, by subsequent designation (Schönherr 1825: 587).

Family-group name: MECINIDAE Gistel, 1848. Stem: *Mecin*-.

***Mecistostylus* Lacordaire, 1865**: 136. Current status: valid genus in CURCULIONIDAE: CRYPTORHYNCHINAE: CRYPTORHYNCHINI: MECISTOSTYLINA.

Type species: *Mecistostylusdouei* Lacordaire, 1865, by original designation.

Family-group name: MÉCISTOSTYLIDES Lacordaire, 1865. Stem: *Mecistostyl*-.

***Mecocerus* Schönherr, 1833**: 115. Current status: junior synonym of *Acanthothorax* Gaede, 1832 in ANTHRIBIDAE: ANTHRIBINAE: MECOCERINI. Note: the senior homonym *Mecocerus* Billberg, 1820 [Coleoptera: CURCULIONIDAE] was suppressed for the purposes of both the Principles of Priority and Homonymy and *Mecocerus* Schönherr, 1833 was placed on the Official List of Generic Names in Zoology (ICZN 1972).

Type species: *Mecocerusgazella* Gyllenhal, 1833 (= *Acanthothoraxlongicornis* Gaede, 1832), by original designation (see ICZN 1972). Note: name placed on the Official List of Specific Names in Zoology (ICZN 1972).

Family-group name: MÉCOCÉRIDES Lacordaire, 1865. Stem: *Mecocer*-.

***Mecolenus* Schönherr, 1847**: 7. Current status: valid genus in BRENTIDAE: APIONINAE: MECOLENITAE.

Type species: *Mecolenuswahlbergi* Schönherr, 1847, by original designation.

Family-group name: MECOLENINAE Wanat, 2001. Stem: *Mecolen*-.

***Mecomacer* Kuschel, 1954a**: 108. Current status: valid genus in NEMONYCHIDAE: RHINORHYNCHINAE: MECOMACERINI: MECOMACERINA.

Type species: *Mecomacerscambus* Kuschel, 1954, by original designation.

Family-group name: MECOMACERINI Kuschel, 1994. Stem: *Mecomacer*-.

***Meconemus* Labram & Imhoff, 1839**: [Heft 4]. Current status: valid genus in ANTHRIBIDAE: ANTHRIBINAE: MECOCERINI.

Type species: *Meconemustuberculatus* Labram & Imhoff, 1839, by monotypy.

Family-group name: MECONEMINI Pierce, 1930. Stem: *Meconem*-. Note: junior homonym of MECONEMINI Burmeister, 1838 (type genus *Meconema* Audinet-Serville, 1831) in Orthoptera.

***Mecopelmus* Blackman, 1944**: 78. Current status: valid genus in CURCULIONIDAE: PLATYPODINAE: MECOPELMINI.

Type species: *Mecopelmuszeteki* Blackman, 1944, by original designation.

Family-group name: MECOPELMINAE Thompson, 1992. Stem: *Mecopelm*-.

***Mecopus* Schönherr, 1825**: 586. Current status: valid genus in CURCULIONIDAE: CONODERINAE: MECOPINI.

Type species: *Rhynchaenusbispinosus* Weber, 1801, by original designation.

Family-group name: MÉCOPIDES Lacordaire, 1865. Stem: *Mecop*-.

***Mecosarthron* Buquet, 1840a**: 172. Current status: valid genus in CERAMBYCIDAE: PRIONINAE: MACROTOMINI: BASITOXINA.

Type species: *Mecosarthronbuphagus* Buquet, 1840, by monotypy.

Family-group name: MECOSARTHRINI Melzer, 1919. Stem: *Mecosarthr*-.

***Mecyclothorax* Sharp, 1903**: 243. Current status: valid genus in CARABIDAE: PSYDRINAE: MORIOMORPHINI: AMBLYTELINA.

Type species: *Cyclothoraxmontivagus* Blackburn, 1878, by subsequent designation (Andrewes 1938: 135).

Family-group name: MECYCLOTHORACITAE Jeannel, 1940. Stem: *Mecyclothorac*-.

***Mecynodera* Hope, 1841a**: 181. Current status: valid genus in CHRYSOMELIDAE: SAGRINAE: MEGAMERINI.

Type species: *Mecynoderapicta* Hope, 1841 (= *Lemacoxalgica* Boisduval, 1835), by original designation.

Family-group name: MÉCYNODÉRITES Chapuis, 1874. Stem: *Mecynoder*-.

***Mecynorhina* Hope, 1837**: 60, 119. Current status: valid genus in SCARABAEIDAE: CETONIINAE: GOLIATHINI: RHOMBORHININA.

Type species: *Scarabaeuspolyphemus* Fabricius, 1781, by original designation.

Family-group name: MECYNORRHININA Schenkling, 1921. Stem: *Mecynorhin*-.

***Mecysmoderes* Schönherr, 1837**: 596. Current status: valid genus in CURCULIONIDAE: CEUTORHYNCHINAE: MECYSMODERINI.

Type species: *Mecysmodereseuglyptus* Gyllenhal, 1837, by original designation.

Family-group name: MECYSMODERINA Wagner, 1938. Stem: *Mecysmoder*-.

***Mecysolobus* Reitter, 1905**: 248. Current status: junior synonym of *Merus* Gistel, 1857 in CURCULIONIDAE: MOLYTINAE: MECYSOLOBINI.

Type species: *Myecysolobuslixoides* Reitter, 1905 (= *Alcidesflavosignatus* Roelofs, 1875), by monotypy.

Family-group name: MECYSLOBINI Reitter, 1913. Stem: *Mecysolob*-.

***Medisores* R.T. Bell & J.R. Bell, 1987**: 288. Current status: valid genus in CARABIDAE: RHYSODINAE: MEDISORINI.

Type species: *Medisoresabditus* R.T. Bell & J.R. Bell, 1987, by original designation.

Family-group name: MEDISORINA R.T. Bell & J.R. Bell, 1987. Stem: *Medisor*-.

†***Mediumiuga* Peris & Ruzzier, 2013**: 2. Current status: valid genus in MORDELLIDAE: MORDELLINAE: †MEDIUMIUGINI. Note: Lower Cretaceous (Spain).

Type species: †*Mediumiugasinespinis* Peris & Ruzzier, 2013, by original designation.

Family-group name: †MEDIUMIUGAMIINI Peris & Ruzzier, 2013: 2. Stem: *Mediumiug*- (stem corrected to *Mediumiug*- by Peris and Ruzzier (2015: 178)).

†***Medmetrioxenoides* Gratshev & Legalov, 2009**: 414. Current status: junior synonym of *Ampliceps* Arnoldi, 1977 in NEMONYCHIDAE: †EOBELINAE: †KARATAUCARINI. Note: Jurassic (Kazakhstan).

Type species: †*Medmetrioxenoidesprodromus* Gratshev & Legalov, 2009 (= *Amplicepsfurcitibia* Arnoldi, 1977), by original designation.

Family-group name: †MEDMETRIOXENOIDESINI Legalov, 2010. Stem: *Medmetrioxenoides*- (ICZN 1999a, Article 29.4).

***Medon* Stephens, 1833b**: 272, 273. Current status: valid genus in STAPHYLINIDAE: PAEDERINAE: LATHROBIINI: MEDONINA.

Type species: *Medonruddii* Stephens, 1833 (= *Paederuscastaneus* Gravenhorst, 1802), by monotypy.

Family-group name: MEDONES Casey, 1905. Stem: *Medon*-.

***Megabasis* Audinet-Serville, 1835a**: 53. Current status: valid genus in CERAMBYCIDAE: LAMIINAE: MEGABASINI.

Type species: *Megabasisspeculifer* Audinet-Serville, 1835 (= *Lamiaspeculifera* W. Kirby, 1818), by monotypy.

Family-group name: MEGABASITAE J. Thomson, 1860. Stem: *Megabas*-.

***Megacantha* Westwood, 1843c**: 121. Current status: valid genus in TENEBRIONIDAE: TENEBRIONINAE: AMARYGMINI.

Type species: *Megacanthatenebrosa* Westwood, 1843 (= *Helopsdentatus* Fabricius, 1801), by monotypy.

Family-group name: MÉGACANTHIDES Lacordaire, 1859. Stem: *Megacanth*-.

***Megacephala* Latreille, 1802b**: 79. Current status: valid genus in CICINDELIDAE: MEGACEPHALINI.

Type species: *Cicindelamegacephala* G.-A. Olivier, 1790, by absolute tautonymy.

Family-group name: MEGACEPHALIDAE Laporte, 1834. Stem: *Megacephal*-.

***Megaceras* Hope, 1837**: 29, 82. Current status: valid genus in SCARABAEIDAE: DYNASTINAE: ORYCTINI.

Type species: to be determined. Note: Hope (1837: 82) originally fixed “*Geotrupeschorinaeus*, Fab.” (= *Scarabaeuschorinaeus* Fabricius, 1775) as the type species of *Megaceras* but that species is currently considered a junior synonym of *Scarabaeuspan* Fabricius, 1775 in the genus *Enema* Hope, 1837 (Ratcliffe 2003: 300); *Scarabaeuschorinaeus* G.-A. Olivier, 1789, considered as a member of *Megaceras* and sometimes its type species, for which F. Dupuis and Mantilleri (2012: 176) designated a lectotype, is not an available name since G.-A. Olivier (1789b: [3] 15) referred to the same species described by Fabricius (1775: 5); an application to the Commission is necessary to fix one of the approximately twenty nominal species currently included in *Megaceras* Hope (e.g., *Scarabaeusjason* Fabricius, 1775) as type species.

Family-group name: MEGACERIDAE Burmeister, 1847. Stem: *Megacerat*-.

***Megacerus* Fåhraeus in Schönherr, 1839**: 34. Current status: valid genus in CHRYSOMELIDAE: BRUCHINAE: BRUCHINI: MEGACERINA. Note: this name was first proposed as a synonym of *Bruchus* Linnaeus, 1767, and subsequently used as valid before 1961 by Drapiez (1841: 203) and others, it is available and dates from its first publication as a synonym (ICZN 1999a, Article 11.6.1).

Type species: *Bruchuspescaprae* Fåhraeus, 1839, by monotypy.

Family-group name: MEGACERINI Bridwell, 1946. Stem: *Megacer*-. Note: senior homonym of MEGACERINI Viret, 1961 (type genus *Megaceros* Owen, 1844) in Mammalia.

***Megacoelus* Lacordaire, 1868**: 458. Current status: valid genus in CERAMBYCIDAE: CERAMBYCINAE: MEGACOELINI. Note: new replacement name for *Megaproctus* Chevrolat, 1840.

Type species: *Megaproctusdidelphis* Chevrolat, 1840, by monotypy (type fixation for *Megaproctus* Chevrolat).

Family-group name: MEGACOELINI Quentin & Villiers, 1969. Stem: *Megacoel*-.

***Megaderus* Dejean, 1821**: 106. Current status: invalid senior synonym of *Neomegaderus* Monné, 2006 in CERAMBYCIDAE: CERAMBYCINAE: TRACHYDERINI: TRACHYDERINA. Note: we consider the name *Megaderus* Dejean, 1821 as available and a junior homonym of *Megaderus* Rafinesque, 1815 [Pisces], notwithstanding the status recently given by Monné and Monné (2013: 360), who treat the cerambycid name as valid (as “*Megaderus* Germar, 1824”) as opposed to a junior homonym (see Monné 2006: 4, 179).

Type species: *Cerambyxstigma* Linnaeus, 1758, by monotypy.

Family-group name: MEGADERITAE J. Thomson, 1861. Stem: *Megader*-. Note: family-group name permanently invalid (ICZN 1999a, Article 39).

***Megagenius* Solier, 1835d**: 512, 513. Current status: valid genus in TENEBRIONIDAE: PIMELIINAE: TENTYRIINI.

Type species: *Megageniusfrioli* Solier, 1835, by monotypy.

Family-group name: MEGAGENIANOS Solier, 1851. Stem: *Megageni*-. Note: nomen oblitum (see Bouchard et al. 2011: 401).

***Megalodacne* Crotch, 1873c**: 352. Current status: valid genus in EROTYLIDAE: EROTYLINAE: MEGALODACNINI.

Type species: *Ipsfasciata* Fabricius, 1777, by original designation.

Family-group name: MEGALODACNINI Sen Gupta, 1970. Stem: *Megalodacn*-.

***Megalognatha* Baly, 1878b**: 416. Current status: valid genus in CHRYSOMELIDAE: GALERUCINAE: LUPERINI.

Type species: *Megalognathaelegans* Baly, 1878, by original designation.

Family-group name: MEGALOGNATHITES Laboissière, 1926. Stem: *Megalognath*-.

***Megalopaussus* Lea, 1906**: 217. Current status: valid genus in CARABIDAE: PAUSSINAE: PAUSSINI: CERAPTERINA.

Type species: *Megalopaussusamplipennis* Lea, 1906, by monotypy.

Family-group name: MEGALOPAUSSINAE Wasmann, 1920. Stem: *Megalopauss*-.

***Megalophthalmus* G.R. Gray in Griffith and Pidgeon, 1831**: 371, pls 39, 61. Current status: invalid senior synonym of *Magnoculus* McDermott, 1964 in LAMPYRIDAE: AMYDETINAE. Note: three spellings were used in the original work, *Megalophthalmus* (pl. 61), *Megolophthalmus* (pl. 39) and *Megalopthalmus* (p. 371); we act as First Revisers (ICZN 1999a, Article 24.2.3) and select *Megalophthalmus* as the correct original spelling; junior homonym of *Megalophthalmus* Leach, 1830 [Crustacea].

Type species: *Megalophthalmusbennettii* G.R. Gray, 1831, by monotypy.

Family-group name: MEGALOPHTHALMINI E. Olivier, 1907. Stem: *Megalophthalm*-. Note: family-group name permanently invalid (ICZN 1999a, Article 39).

***Megalopinus* Eichelbaum, 1915**: 104. Current status: valid genus in STAPHYLINIDAE: MEGALOPSIDIINAE. Note: new replacement name for *Megalops* Erichson, 1839.

Type species: *Oxyporuscaelatus* Gravenhorst, 1802, by subsequent designation (E. Desmarest 1852: 85) (type fixation for *Megalops* Erichson).

Family-group name: MEGALOPININAE Naomi, 1986. Stem: *Megalopin*-.

***Megalops* Erichson, 1839b**: 30. Current status: invalid senior synonym of *Megalopinus* Eichelbaum, 1915 in STAPHYLINIDAE: MEGALOPSIDIINAE. Note: junior homonym of *Megalops* Lacepède, 1803 [Pisces].

Type species: *Oxyporuscaelatus* Gravenhorst, 1802, by subsequent designation (E. Desmarest 1852: 85). Note: no species were originally included in this genus by Erichson (1839b); the first ones were the three species listed by Erichson (1840a: 751, 752).

Family-group name: MEGALOPINI Erichson, 1839. Stem: *Megalop*-. Note: family-group name permanently invalid (ICZN 1999a, Article 39).

***Megalopsidia* Leng, 1918**: 204. Current status: junior synonym of *Megalopinus* Eichelbaum, 1915 in STAPHYLINIDAE: MEGALOPSIDIINAE. Note: new replacement name for *Megalops* Erichson, 1839.

Type species: *Oxyporuscaelatus* Gravenhorst, 1802, by subsequent designation (E. Desmarest 1852: 85) (type fixation for *Megalops* Erichson).

Family-group name: MEGALOPSIDIINI Leng, 1920. Stem: *Megalopsidi*-.

***Megalopus* Fabricius, 1801a**: xvi. Current status: valid genus in MEGALOPODIDAE: MEGALOPODINAE: MEGALOPODINI: MEGALOPODINA.

Type species: *Megalopusnigricornis* Fabricius, 1801, by subsequent designation (Latreille 1810: 431). Note: no species were originally included in this genus by Fabricius (1801a); the first ones were the two species listed by Fabricius (1801b: 367, 368).

Family-group name: MEGALOPIDES Latreille, 1802. Stem: *Megalopod*-.

***Megalostomis* Chevrolat in Dejean, 1836a**: 416. Current status: valid genus in CHRYSOMELIDAE: CRYPTOCEPHALINAE: CLYTRINI: MEGALOSTOMIDINA.

Type species: *Clytraboopis* Germar, 1823 (= *Clytragrossa* Forsberg, 1821), by subsequent designation (Monrós 1953: 46).

Family-group name: MEGALOSTOMIDEAE Lacordaire, 1848. Stem: *Megalostomid*-.

***Megamerus* W.S. MacLeay, 1826**: 448. Current status: valid genus in CHRYSOMELIDAE: SAGRINAE: MEGAMERINI.

Type species: *Megameruskingii* W.S. MacLeay, 1826, by monotypy.

Family-group name: MÉGAMÉRITES Chapuis, 1874. Stem: *Megamer*-.

†***Megametrioxenoides* Gratshev & Legalov, 2009**: 413. Current status: valid genus in NEMONYCHIDAE: †METRIOXENOIDINAE. Note: Early Cretaceous (Mongolia).

Type species: †*Megametrioxenoideslongus* Gratshev & Legalov, 2009, by original designation.

Family-group name: †MEGAMETRIOXENOIDESINI Legalov, 2010. Stem: *Megametrioxenoides*- (ICZN 1999a, Article 29.4).

***Megapalpus* Guillebeau, 1893**: 297. Current status: invalid senior synonym of *Megistopalpus* Guillebeau, 1895 in PHALACRIDAE: PHALACRINAE. Note: junior homonym of *Megapalpus* Macquart, 1834 [Diptera].

Type species: *Megapalpussimoni* Guillebeau, 1893, by monotypy.

Family-group name: MEGAPALPINI Guillebeau, 1894. Stem: *Megapalp*-. Note: family-group name permanently invalid (ICZN 1999a, Article 39).

***Megapenthes* Kiesenwetter, 1858**: 353. Current status: valid genus in ELATERIDAE: ELATERINAE: MEGAPENTHINI.

Type species: *Elaterlugens* W. Redtenbacher, 1842, by subsequent designation (Hyslop 1921: 655).

Family-group name: MEGAPENTHINI Gurjeva, 1973. Stem: *Megapenth*-.

***Megarhinus* Schönherr, 1835**: 397. Current status: invalid senior synonym of *Trigonocolus* Lacordaire, 1863 in CURCULIONIDAE: MOLYTINAE: TRIGONOCOLINI. Note: junior homonym of *Megarhinus* Rafinesque, 1820 [Pisces].

Type species: *Megarhinusfirmus* Gyllenhal, 1835, by original designation.

Family-group name: MEGARHININAE Faust, 1888. Stem: *Megarhin*-. Note: family-group name permanently invalid (ICZN 1999a, Article 39).

***Megarthropsis* Cameron, 1919**: 231. Current status: valid genus in STAPHYLINIDAE: TACHYPORINAE: TACHINUSINI.

Type species: *Megarthropsisdecorata* Cameron, 1919, by monotypy.

Family-group name: MEGARTHROPSINI Cameron, 1919. Stem: *Megarthrops*-.

***Megarthrus* Stephens, 1829b**: 24. Current status: valid genus in STAPHYLINIDAE: PROTEININAE: PROTEININI.

Type species: *Staphylinusdepressus* Paykull, 1789, by subsequent designation (Westwood 1838c: 18).

Family-group name: MEGARTHRINI Joy, 1932. Stem: *Megarthr*-.

***Megascelis* Sturm, 1826**: 80. Current status: valid genus in CHRYSOMELIDAE: EUMOLPINAE: MEGASCELIDINI.

Type species: *Megascelisaenea* Sturm, 1826, by monotypy.

Family-group name: MÉGASCÉLIDES Chapuis, 1874. Stem: *Megascelid*-.

***Megasoma* W. Kirby, 1825**: 566. Current status: valid genus in SCARABAEIDAE: DYNASTINAE: DYNASTINI: DYNASTINA.

Type species: *Scarabaeusactaeon* Linnaeus, 1758, by original designation.

Family-group name: MEGASOMINAE Swainson, 1840. Stem: *Megasomat*-.

***Megasternum* Mulsant, 1844**: 187. Current status: valid genus in HYDROPHILIDAE: SPHAERIDIINAE: MEGASTERNINI: MEGASTERNINA. Note: name placed on the Official List of Generic Names in Zoology (ICZN 1981b).

Type species: *Dermestesobscurus* Marsham, 1802 (= *Dermestesconcinnus* Marsham, 1802), by plenary power (see ICZN 1981b). Note: *Dermestesobscurus* Marsham, 1802 was placed on the Official List of Specific Names in Zoology (ICZN 1981b).

Family-group name: MÉGASTERNAIRES Mulsant, 1844. Stem: *Megastern*-.

***Megataphrus* Casey, 1890b**: 309. Current status: valid genus in ZOPHERIDAE: COLYDIINAE: SYNCHITINI.

Type species: *Megataphrustenuicornis* Casey, 1890, by monotypy.

Family-group name: MEGATAPHRINI Casey, 1890. Stem: *Megataphr*-.

***Megatoma* Herbst, 1791**: 92. Current status: valid genus in DERMESTIDAE: MEGATOMINAE: MEGATOMINI: MEGATOMINA.

Type species: *Dermestesundatus* Linnaeus, 1758, by subsequent designation (Curtis 1829: pl. 244).

Family-group name: MEGATOMIDA Leach, 1815. Stem: *Megatom*-.

***Megelenophorus* Gebien, 1910**: 121. Current status: valid genus in TENEBRIONIDAE: PIMELIINAE: MEGELENOPHORINI. Note: new replacement name for *Cacicus* Dejean, 1834.

Type species: *Elenophorusamericanus* Lacordaire, 1830, by monotypy (type fixation for *Cacicus* Dejean).

Family-group name: MEGALENOPHORINA Ferrer, 2015: 24. Stem: *Megelenophor*-.

***Megetra* J.L. LeConte, 1859c**: 127. Current status: valid genus in MELOIDAE: MELOINAE: EUPOMPHINI.

Type species: *Meloecancellatus* Brandt & Erichson, 1832, by subsequent designation (Wellman 1910a: 392).

Family-group name: MEGETRINA Kaszab, 1959. Stem: *Megetr*-.

***Megodontus* Solier, 1848a**: 58. Current status: valid subgenus of *Carabus* Linnaeus, 1758 in CARABIDAE: CARABINAE: CARABINI: CARABINA.

Type species: *Carabuscaelatus* Fabricius, 1801, by original designation.

Family-group name: MEGADONTICI Csiki, 1927. Stem: *Megodont*-.

***Megopis* Audinet-Serville, 1832**: 161. Current status: valid genus in CERAMBYCIDAE: PRIONINAE: AEGOSOMATINI.

Type species: *Megopismutica* Audinet-Serville, 1832, by monotypy.

Family-group name: MEGOPIDES Lameere, 1912. Stem: *Megopid*-.

†***Mekorhamphus* Poinar, A.E. Brown & Legalov, 2016**: 158. Current status: valid genus in †MESOPHYLETIDAE: †MESOPHYLETINAE. Note: Upper Cretaceous (Myanmar).

Type species: †*Mekorhamphusgyralommus* Poinar, A.E. Brown & Legalov, 2016, by original designation.

Family-group name: †MEKORHAMPHINI Poinar, A.E. Brown & Legalov, 2016: 158. Stem: *Mekorhamph*-.

***Melaenus* Dejean, 1831**: 471, 481. Current status: valid genus in CARABIDAE: MELAENINAE.

Type species: *Melaenuselegans* Dejean, 1831, by monotypy.

Family-group name: MELAENINAE Csiki, 1933. Stem: *Melaen*-.

***Melambius* Mulsant & Rey, 1854**: 124. Current status: valid genus in TENEBRIONIDAE: BLAPTINAE: DENDARINI: MELAMBIINA.

Type species: *Opatrumbarbarum* Erichson, 1841, by monotypy.

Family-group name: MELAMBIATES Mulsant & Rey, 1854. Stem: *Melambi*-.

***Melanactes* J.L. LeConte, 1853f**: 493. Current status: valid genus in ELATERIDAE: DENDROMETRINAE: OXYNOPTERINI.

Type species: *Melanactesdensus* J.L. LeConte, 1853, by subsequent designation (Hyslop 1921: 656).

Family-group name: MÉLANACTIDES Candèze, 1857. Stem: *Melanact*-.

***Melanchiton* Andrewes, 1940**: 536. Current status: valid genus in CARABIDAE: HARPALINAE: MELANCHITONINI. Note: new replacement name for *Melanodes* Chaudoir, 1876.

Type species: *Chlaeniusaterrimus* LaFerté-Sénectère, 1853, by subsequent designation (Alluaud 1916: 227) (type fixation for *Melanodes* Chaudoir).

Family-group name: MELANCHITONITAE Jeannel, 1948. Stem: *Melanchiton*-.

***Melandrya* Fabricius, 1801a**: xxi, 163. Current status: valid genus in MELANDRYIDAE: MELANDRYINAE: MELANDRYINI.

Type species: *Helopsserratus* Fabricius, 1775 (= *Chrysomelacaraboides* Linnaeus, 1761), by subsequent designation (Latreille 1810: 429).

Family-group name: MELYANDRIDA Leach, 1815. Stem: *Melandry*-.

***Melaneros* Fairmaire, 1877**: 173. Current status: valid genus in LYCIDAE incertae sedis.

Type species: *Melanerosacuticollis* Fairmaire, 1877, by subsequent designation (Bourgeois 1891: 349). Note: the type species sometimes listed for this genus is *Melanerosatroviolaceus* Fairmaire, 1877 by subsequent designation of Blair (1928: 99) (e.g., Calder 1998: 149; Bocák and Bocáková 1992: 255) but, as pointed out by Bocáková (2001: 77), Bourgeois’ designation is older.

Family-group name: MELANEROTINI Kazantsev, 2010. Stem: *Melanerot*-.

***Melanimon* Steven, 1828**: 98. Current status: valid genus in TENEBRIONIDAE: TENEBRIONINAE: MELANIMONINI.

Type species: *Opatrumtibiale* Fabricius, 1781, by monotypy.

Family-group name: MELANIMONINA Seidlitz, 1894. Stem: *Melanimon*-.

***Melanodes* Chaudoir, 1876b**: 360. Current status: invalid senior synonym of *Melanchiton* Andrewes, 1940 in CARABIDAE: HARPALINAE: MELANCHITONINI. Note: junior homonym of *Melanodes* Guenée, 1857 [Lepidoptera].

Type species: *Chlaeniusaterrimus* LaFerté-Sénectère, 1853, by subsequent designation (Alluaud 1916: 227).

Family-group name: MELANODINI Alluaud, 1916. Stem: *Melanod*-. Note: family-group name permanently invalid (ICZN 1999a, Article 39).

***Melanophila* Eschscholtz, 1829b**: 9. Current status: valid genus in BUPRESTIDAE: BUPRESTINAE: MELANOPHILINI. Note: name placed on the Official List of Generic Names in Zoology (ICZN 1996b).

Type species: *Buprestisacuminata* DeGeer, 1774, by plenary power (see ICZN 1996b). Note: name placed on the Official List of Specific Names in Zoology (ICZN 1996b).

Family-group name: MELANOPHILINI Bedel, 1921. Stem: *Melanophil*-.

***Melanophthalma* Motschulsky, 1866b**: 269. Current status: valid genus in LATRIDIIDAE: CORTICARIINAE.

Type species: *Latridiustransversalis* Gyllenhal, 1827, by subsequent designation (Dajoz 1967: 606).

Family-group name: MELANOPHTHALMIDAE Stickney, 1923. Stem: *Melanophthalm*-.

***Melanotus* Eschscholtz, 1829a**: 32. Current status: valid genus in ELATERIDAE: ELATERINAE: MELANOTINI.

Type species: *Elaterfulvipes* Herbst, 1806 (= *Elatervillosus* Geoffroy, 1785), by subsequent designation (Curtis 1838a: pl. 694).

Family-group name: MÉLANOTITES Candèze, 1859. Stem: *Melanot*-.

***Melasis* G.-A. Olivier, 1790a**: [30] 1, 3. Current status: valid genus in EUCNEMIDAE: MELASINAE: MELASINI: MELASINA.

Type species: *Elaterbuprestoides* Linnaeus, 1761, by monotypy.

Family-group name: MELASIDAE Fleming, 1821. Stem: *Melas*-.

***Meleagros* Kirschenhofer, 1999**: 68, 72. Current status: valid genus in CARABIDAE: HARPALINAE: PLATYNINI.

Type species: *Meleagroscoeruleus* Kirschenhofer, 1999, by original designation.

Family-group name: MELEAGROSINI Morvan, 2004. Stem: *Meleagros*- (ICZN 1999a, Article 29.4).

***Meliboeus* Deyrolle, 1865**: 132. Current status: valid genus in BUPRESTIDAE: AGRILINAE: CORAEBINI: MELIBOEINA.

Type species: *Buprestisaeneicollis* Villers, 1789 (= *Coraebusfulgidicollis* P.H. Lucas, 1846), by subsequent designation (Bedel 1921: 193).

Family-group name: MELIBOEINA Majer, 2000. Stem: *Meliboe*-.

***Meligethes* Stephens, 1829b**: 7. Current status: valid genus in NITIDULIDAE: MELIGETHINAE. Note: this genus-group name is sometimes attributed to Stephens (1830: 30) in the literature (e.g., Jelínek and Audisio 2007: 478, Audisio et al. 2009: 456) but it was made available one year earlier by Stephens (1829b: 7).

Type species: to be determined. Note: *Nitidulaatrata* G.-A. Olivier, 1790 is sometimes listed as the type species of this genus (e.g., Jelínek and Audisio 2007: 478, Audisio et al. 2014: 19), but it is not an originally included species; Audisio et al. (2009: 456) used the following information for the type species of *Meligethes* “*Nitidularufipes* Marsham, 1802 (= *Nitidulaatrata* A. G. Olivier, 1790), by subsequent designation (C.G. Thomson 1859: 67)” but Marsham (1802: 130) did not propose a new species; instead he referred to *Nitidularufipes* Fabricius, 1775, which is considered a senior invalid synonym of *Brassicogethesviridescens* (Fabricius, 1787); the valid type species fixation for *Meligethes* is that of Westwood (1838c: 12), who selected *Nitidulaviridescens* Fabricius, 1787, but that species is currently included in the genus *Brassicogethes* Audisio & Cline, 2009; Hope (1841a: 154) subsequently selected *Nitidularufipes* Fabricius, 1775 as the type species of *Meligethes*; more research is needed to establish the necessary facts and determine the best possible solution, which may be to ask the Commission to fix *Nitidulaatrata* G.-A. Olivier, 1790 as the type species of *Meligethes* to conserve the current usage of the name.

Family-group name: MELIGETHINA C.G. Thomson, 1859. Stem: *Meligeth*-.

†***Meligethiella* L.N. Medvedev, 1969**: 119. Current status: valid genus in NITIDULOIDEA incertae sedis. Note: Upper Jurassic/Lower Cretaceous (Kazakhstan; eastern Siberia).

Type species: †*Meligethiellasoroniiformis* L.N. Medvedev, 1969, by original designation.

Family-group name: †MELIGETHIELLINAE Kirejtshuk & Ponomarenko, 1990. Stem: *Meligethiell*-.

***Melisodera* Westwood, 1835**: pl. 132. Current status: valid genus in CARABIDAE: PSYDRINAE: MORIOMORPHINI: MORIOMORPHINA.

Type species: *Melisoderapiceipennis* Westwood, 1835, by monotypy.

Family-group name: MELISODERIDES Sloane, 1898. Stem: *Melisoder*-.

***Melittomma* Murray, 1867b**: 314. Current status: valid genus in LYMEXYLIDAE: MELITTOMMATINAE.

Type species: *Hylecoetusbrasiliensis* Laporte, 1833, by original designation.

Family-group name: MELITTOMMINAE Q.D. Wheeler, 1986. Stem: *Melittommat*-.

***Melobasis* Laporte & Gory, 1837**: 3. Current status: valid genus in BUPRESTIDAE: BUPRESTINAE: MELOBASINI.

Type species: *Buprestiscupriceps* W. Kirby, 1819, by subsequent designation (Bellamy 1998: 11).

Family-group name: MELOBASINI Bílý, 2000. Stem: *Melobas*- (ICZN 1999a, Article 29.4).

***Meloe* Linnaeus, 1758**: 342, 419. Current status: valid genus in MELOIDAE: MELOINAE: MELOINI. Note: name placed on the Official List of Generic Names in Zoology (ICZN 1999c).

Type species: *Meloeproscarabaeus* Linnaeus, 1758, by subsequent designation (Latreille, 1810: 430; see ICZN 1999c). Note: name placed on the Official List of Specific Names in Zoology (ICZN 1999c).

Family-group name: MELOOIDES Gyllenhal, 1810. Stem: *Melo*-. Note: family-group name given precedence over HORIIDAE Latreille, 1802 (ICZN 1999c).

***Meloetyphlus* C.O. Waterhouse, 1872**: 31. Current status: valid genus in MELOIDAE: TETRAONYCINAE.

Type species: *Meloetyphlusfuscatus* C.O. Waterhouse, 1872, by monotypy.

Family-group name: MELOETYPHLINI Borgmeier, 1937. Stem: *Meloetyphl*-.

***Melolontha* Fabricius, 1775**: 31. Current status: valid genus in SCARABAEIDAE: MELOLONTHINAE: MELOLONTHINI: MELOLONTHINA. Note: the senior homonym *Melolontha* Geoffroy, 1762 (and all other uses of the name prior to the publication of *Melolontha* Fabricius, 1775) was suppressed for both Principles of Priority and Homonymy and placed on the Official Index of Rejected and Invalid Generic Names in Zoology while *Melolontha* Fabricius, 1775 was placed on the Official List of Generic Names in Zoology (ICZN 1994a).

Type species: *Scarabaeusmelolontha* Linnaeus, 1758, by absolute tautonymy (see ICZN 1994a). Note: name placed on the Official List of Specific Names in Zoology (ICZN 1994a).

Family-group name: MELOLONTHIDAE Leach, 1819. Stem: *Melolonth*-. Note: senior homonym of MELOLONTHINI Bedel, 1891 (type genus *Melolontha* Geoffroy, 1762) in Coleoptera: CHRYSOMELIDAE; Bedel’s name is permanently invalid (based on a suppressed type genus).

***Melolontha* Geoffroy, 1762**: 195. Current status: suppressed name in CHRYSOMELIDAE: CRYPTOCEPHALINAE: CLYTRINI: CLYTRINA. Note: usually listed as a synonym of *Clytra* Laicharting, 1781; name suppressed for both the Principles of Priority and Homonymy and placed on the Official Index of Rejected and Invalid Names in Zoology (see ICZN 1994a).

Type species: to be determined.

Family-group name: MELOLONTHINI Bedel, 1891. Stem: *Melolonth*-. Note: family-group name permanently invalid (ICZN 1999a, Article 39).

***Melyris* Fabricius, 1775**: 58. Current status: valid genus in MELYRIDAE: MELYRINAE: MELYRINI.

Type species: *Melyrisviridis* Fabricius, 1775, by monotypy.

Family-group name: MELYRIDES Leach, 1815. Stem: *Melyr*-.

***Mendizabalia* Cobos, 1957**: 233. Current status: valid genus in BUPRESTIDAE: BUPRESTINAE: MENDIZABALIINI.

Type species: *Agrilusgermaini* Germain & Kerremans, 1906, by original designation.

Family-group name: MENDIZABALIINI Cobos, 1968. Stem: *Mendizabali*-.

***Menemachus* Schönherr, 1843**: 266. Current status: valid genus in CURCULIONIDAE: CONODERINAE: MENEMACHINI.

Type species: *Menemachusnaevus* Boheman, 1843, by original designation.

Family-group name: MÉNÉMACHIDES Lacordaire, 1865. Stem: *Menemach*-.

***Menephilus* Mulsant, 1854**: 291. Current status: valid genus in TENEBRIONIDAE: STENOCHIINAE: CNODALONINI.

Type species: *Tenebriocurvipes* Fabricius, 1792 (= *Tenebriocylindricus* Herbst, 1784), by monotypy.

Family-group name: MENEPHILINI Reitter, 1920. Stem: *Menephil*-.

***Menoetius* Dejean, 1821**: 94. Current status: invalid senior synonym of *Lachnopus* Schönherr, 1840 in CURCULIONIDAE: ENTIMINAE: POLYDRUSITAE: EUSTYLINI: EUSTYLINA. Note: name suppressed for the Principle of Priority and placed on the Official Index of Rejected and Invalid Generic Names in Zoology (see ICZN 1987f).

Type species: *Curculiovalgus* Fabricius, 1775, by subsequent designation (O’Brien and Wibmer 1982: 8).

Family-group name: MENOETIINI Pierce, 1913. Stem: *Menoeti*-. Note: family-group name permanently invalid (ICZN 1999a, Article 39).

***Mentophilus* Laporte, 1840b**: 74. Current status: valid genus in SCARABAEIDAE: SCARABAEINAE: DELTOCHILINI. Note: three original spellings were used, including *Mintophilus* (pl. 23) and *Minthophilus* (p. 63); as far as we know, no one has acted as First Reviser (ICZN 1999a, Article 24.2.3) and we **here select***Mentophilus*, the spelling currently in prevailing usage, as the correct original spelling; this genus is a junior synonym of *Aulacium* Dejean, 1833 (see Bousquet and Bouchard 2013a: 33), and an application to the Commission is necessary to conserve *Mentophilus* as a valid name.

Type species: **here fixed** (under ICZN 1999a, Article 70.3.2) as *Aulaciumcarinatum* Reiche, 1841, misidentified as *Scarabaeusnovaehollandiae* Fabricius, 1775 (sensu Dejean, 1833), in the original designation by monotypy in Laporte (1840b: 74). Note: Laporte (1840b: 74) listed the species as “*hollandiae*” but from the references given, it is clear that he was referring to *Scarabaeusnovaehollandiae* Fabricius, 1775; the nominal species *Scarabaeusnovaehollandiae* Fabricius, 1775 is the type species of the genus *Tesserodon* Hope, 1837; see Duponchel and Chevrolat (1842a: 334, 335) regarding the misidentification of the type species of *Mentophilus*.

Family-group name: MINTHOPHILIDES Lacordaire, 1855. Stem: *Mentophil*-.

***Menuthianaspis* Franciscolo, 1972**: 124. Current status: valid genus in SCRAPTIIDAE: ANASPIDINAE: MENUTHIANASPIDINI.

Type species: *Anaspissicardi* Pic, 1942, by original designation.

Family-group name: MENUTHIANASPIDINI Franciscolo, 1972. Stem: *Menuthianaspid*-.

***Meonis* Laporte, 1867**: 69. Current status: valid genus in CARABIDAE: PSYDRINAE: MORIOMORPHINI: AMBLYTELINA.

Type species: *Meonisniger* Laporte, 1867, by subsequent designation (B.P. Moore 1963b: 288).

Family-group name: MEONIDES Sloane, 1898. Stem: *Meon*-.

***Meotica* Mulsant & Rey, 1873**: 174. Current status: valid genus in STAPHYLINIDAE: ALEOCHARINAE: OXYPODINI: MEOTICINA.

Type species: *Meoticaparasita* Mulsant & Rey, 1873, by subsequent designation (Blackwelder 1952: 237).

Family-group name: MEOTICAE Seevers, 1978. Stem: *Meotic*-.

***Meracantha* W. Kirby, 1837**: 237. Current status: valid genus in TENEBRIONIDAE: TENEBRIONINAE: AMARYGMINI.

Type species: *Meracanthacanadensis* W. Kirby, 1837 (= *Helopscontractus* Palisot de Beauvois, 1812), by monotypy.

Family-group name: MÉRACANTHIDES Lacordaire, 1859. Stem: *Meracanth*-.

***Meriphus* Erichson, 1842**: 199. Current status: valid genus in CURCULIONIDAE: CURCULIONINAE: EUGNOMINI: MERIPHINA.

Type species: *Meriphusfullo* Erichson, 1842, by monotypy.

Family-group name: MERIPHINAE G.A.K. Marshall, 1937. Stem: *Meriph*-.

***Merismoderus* Westwood, 1847c**: 130. Current status: junior synonym of *Melanospilus* Westwood, 1847 in CARABIDAE: PAUSSINAE: PAUSSINI: PAUSSINA. Note: *Merismoderus* was originally described as a subgenus of the new genus *Melanospilus*, but it is also an objective synonym of *Dimeroderus*Westwood (1847b: 469), which was also published as a subgenus of *Melanospilus*; see Nagel et al. (2017a: 12) regarding the need to submit an application to the Commission in order to maintain usage of the valid genus name *Melanospilus* Westwood, 1847 over the unused name *Leiomatocerus* Benson, 1846.

Type species: *Melanospilusbensoni* Westwood, 1847, by monotypy.

Family-group name: MERISMODERINI Wasmann, 1929. Stem: *Merismoder*-.

***Merizodus* Solier, 1849**: 185. Current status: valid genus in CARABIDAE: TRECHINAE: ZOLINI.

Type species: *Merizodusangusticollis* Solier, 1849, by monotypy.

Family-group name: MERIZODINI Sloane, 1920. Stem: *Merizodont*-.

***Meroda* Baly, 1860**: 29. Current status: valid genus in CHRYSOMELIDAE: EUMOLPINAE: MERODINI.

Type species: *Merodacostata* Baly, 1860, by original designation.

Family-group name: MERODITES Chapuis, 1874. Stem: *Merod*-.

***Merodontus* Jekel, 1856**: 9bis. Current status: valid genus in CURCULIONIDAE: ENTIMINAE: ENTIMITAE: LORDOPINI.

Type species: *Merodontusderosus* Jekel, 1856, by monotypy.

Family-group name: MERODONTI Jekel, 1856. Stem: *Merodont*-.

***Meropathus* Enderlein, 1901**: 121. Current status: valid genus in HYDRAENIDAE: OCHTHEBIINAE: OCHTHEBIINI.

Type species: *Meropathuschuni* Enderlein, 1901, by monotypy.

Family-group name: MEROPATHINA Perkins, 1997. Stem: *Meropath*-.

***Merophysia* P.H. Lucas, 1852**: xxix. Current status: valid genus in ENDOMYCHIDAE: MEROPHYSIINAE.

Type species: *Merophysiaformicaria* P.H. Lucas, 1852, by monotypy.

Family-group name: MEROPHYSIINI Seidlitz, 1872. Stem: *Merophysi*-.

***Meroscelisus* Audinet-Serville, 1832**: 157. Current status: valid genus in CERAMBYCIDAE: PRIONINAE: MEROSCELISINI.

Type species: *Meroscelisusviolaceus* Audinet-Serville, 1832, by monotypy.

Family-group name: MEROSCELISITAE J. Thomson, 1861. Stem: *Meroscelis*-.

***Meru* Spangler & Steiner, 2005**: 340. Current status: valid genus in MERUIDAE.

Type species: *Meruphyllisae* Spangler & Steiner, 2005, by original designation.

Family-group name: MERUIDAE Spangler & Steiner, 2005. Stem: *Meru*-.

***Meryx* Latreille, 1802b**: 208. Current status: valid genus in ULODIDAE.

Type species: *Meryxrugosa* Latreille, 1802, by monotypy.

Family-group name: MERYCIDAE Crowson, 1953. Stem: *Meryc*-.

***Mesagroicus* Schönherr, 1840b**: 281. Current status: valid genus in CURCULIONIDAE: ENTIMINAE: POLYDRUSITAE: NAUPACTINI: MESAGROICINA.

Type species: *Thylacitespiliferus* Boheman, 1833, by original designation.

Family-group name: MESAGROICINA Legalov, 2020b: 341. Stem: *Mesagroic*-.

†***Mesernobius* Engel, 2010**: 32. Current status: valid genus in SCIRTIDAE: †MESERNOBIINAE. Note: mid-Cretaceous (Burmese amber).

Type species: †*Mesernobiusanawrahtai* Engel, 2010, by original designation.

Family-group name: †MESERNOBIINAE Engel, 2010. Stem: *Mesernobi*-. Note: combined description of a new family-group taxon and a single new nominal genus (ICZN 1999a, Article 13.5).

†***Mesocinetus* Ponomarenko, 1986**: 96. Current status: valid genus in †MESOCINETIDAE. Note: Lower Cretaceous (Mongolia).

Type species: †*Mesocinetusmongolicus* Ponomarenko, 1986, by original designation.

Family-group name: †MESOCINETIDAE Kirejtshuk & Ponomarenko, 2010. Stem: *Mesocinet*-.

***Mesocoelopus* Jacquelin du Val, 1860a**: 143. Current status: valid genus in PTINIDAE: MESOCOELOPODINAE: MESOCOELOPODINI.

Type species: *Ptilinusniger* P.W.J. Müller, 1821, by original designation.

Family-group name: MÉSOCOELOPAIRES Mulsant & Rey, 1864. Stem: *Mesocoelopod*-.

†***Mesocupes* Martynov, 1926**: 6. Current status: valid genus in CUPEDIDAE: †MESOCUPEDINAE. Note: Middle/Upper Jurassic (Kazakhstan; China).

Type species: †*Mesocupesprimitivus* Martynov, 1926, by monotypy.

Family-group name: †MESOCUPEDINI Ponomarenko, 1969. Stem: *Mesocuped*-.

†***Mesoderus* Prokin & Ren, 2010**: 50. Current status: valid genus in DYTISCIDAE: †LIADYTISCINAE: †MESODERINI. Note: Mesozoic (China).

Type species: †*Mesoderusmagnus* Prokin & Ren, 2010, by original designation.

Family-group name: †MESODERINI Prokin, Petrov, B. Wang & Ponomarenko, 2013: 145. Stem: *Mesoder*-.

***Mesogenus* Gemminger in Gemminger and Harold, 1869b**: 1485. Current status: valid genus in EUCNEMIDAE: EUCNEMINAE: MESOGENINI.

Type species: *Fornaxaustrocaledonicus* Perroud & Montrouzier, 1865, by monotypy. Note: Since *Mesogenus* is credited to Bonvouloir’s work (1871–1875) on EUCNEMIDAE in the literature, the type species was considered to be *Mesogenusmellyi* Bonvouloir, 1871, by subsequent designation (Fleutiaux 1902: 651) (e.g., Calder 1998: 16); however, this genus-group name is available from Gemminger and Harold’s catalogue, with only one available species listed under it, *F.austroacledonicus*, which is therefore the type species by monotypy.

Family-group name: MESOGENINI Muona, 1993. Stem: *Mesogen*-.

†***Mesolpinus* Kirejtshuk, Moseyko & Ren, 2016**: 1426. Current status: valid genus in CHRYSOMELIDAE: CHRYSOMELINAE: †MESOLPININI. Note: Lower Cretaceous (China).

Type species: †*Mesolpinusantennatus* Kirejtshuk, Moseyko & Ren, 2016, by original designation.

Family-group name: †MESOLPININI Kirejtshuk, Moseyko & Ren, 2016: 1425. Stem: *Mesolpin*-.

***Mesomphalia* Hope, 1839**: 94. Current status: valid genus in CHRYSOMELIDAE: CASSIDINAE: MESOMPHALIINI.

Type species: *Cassidagibbosa* Fabricius, 1781, by original designation.

Family-group name: MESOMPHALIDAE Hope, 1841. Stem: *Mesomphali*-.

†***Mesopassandra* Jin, Ślipiński, Zhou & H. Pang, 2019**: 1951. Current status: valid genus in PASSANDRIDAE: †MESOPASSANDRINAE. Note: Late Cretaceous (Myanmar).

Type species: †*Mesopassandrakeyao* Jin, Ślipiński, Zhou & H. Pang, 2019, by original designation.

Family-group name: †MESOPASSANDRINAE Jin, Ślipiński, Zhou & H. Pang, 2019: 1951. Stem: *Mesopassandr*-.

†***Mesophyletis* Poinar, 2006**: 879. Current status: valid genus in †MESOPHYLETIDAE: †MESOPHYLETINAE. Note: mid-Cretaceous (Burmese amber).

Type species: †*Mesophyletiscalhouni* Poinar, 2006, by original designation.

Family-group name: †MESOPHYLETINAE Poinar, 2006. Stem: *Mesophylet*-.

***Mesoporus* Cameron, 1959**: 120. Current status: valid genus in STAPHYLINIDAE: ALEOCHARINAE: MESOPORINI.

Type species: *Mesoporusrufoflavus* Cameron, 1959, by monotypy.

Family-group name: MESOPORINAE Cameron, 1959. Stem: *Mesopor*-.

***Mesoptilius* Labram & Imhoff, 1845**: [Heft 13] 24. Current status: valid genus in CURCULIONIDAE: MESOPTILIINAE: MESOPTILIINI.

Type species: *Mesoptiliusapicalis* Labram & Imhoff, 1845, by monotypy.

Family-group name: MÉSOPTILIDES Lacordaire, 1863. Stem: *Mesoptili*-.

***Mesosa* Latreille, 1829b**: 124. Current status: valid genus in CERAMBYCIDAE: LAMIINAE: MESOSINI.

Type species [suggested]: *Cerambyxcurculionoides* Linnaeus, 1761, by subsequent designation (C.G. Thomson 1859: 153). Note: the valid type species fixation is that of Westwood (1838c: 41), who selected *Cerambyxnubilus* Gmelin, 1789 (= *Lamianebulosa* Fabricius, 1781), but that species is currently in the subgenus Aplocnemia Stephens, 1831, not the nominotypical subgenus; an application to the Commission is necessary to fix *Cerambyxcurculionoides* Linnaeus, 1761 as the type species of *Mesosa* Latreille, 1829.

Family-group name: MÉSOSAIRES Mulsant, 1839. Stem: *Mesos*-.

***Mesostyloides* Bouchard & Bousquet, 2020a**: 114. Current status: valid genus in CURCULIONIDAE: ENTIMINAE: OTIORHYNCHITAE: MESOSTYLOIDINI. Note: new replacement name for *Mesostylus* Faust, 1894.

Type species: *Mesostylushauseri* Faust, 1894, by subsequent designation (Faust 1898b: 231) (type fixation for *Mesostylus* Faust).

Family-group name: MESOSTYLOIDINI Bouchard & Bousquet, 2020a: 114. Stem: *Mesostyloid*-.

***Mesostylus* Faust in Hauser, 1894**: 57. Current status: invalid senior synonym of *Mesostyloides* Bouchard & Bousquet, 2020 in CURCULIONIDAE: ENTIMINAE: OTIORHYNCHITAE: MESOSTYLOIDINI. Note: junior homonym of *Mesostylus* Bronn & Roemer, 1852 [Crustacea].

Type species: *Mesostylushauseri* Faust, 1894, by subsequent designation (Faust 1898b: 231).

Family-group name: MESOSTYLINI Reitter, 1913. Stem: *Mesostyl*-. Note: family-group name permanently invalid (ICZN 1999a, Article 39).

***Mesothes* Mulsant & Rey, 1864b**: 311. Current status: valid genus in PTINIDAE: MESOCOELOPODINAE: MESOCOELOPODINI.

Type species: *Xyletinusferrugineus* Mulsant & Rey, 1861, by monotypy.

Family-group name: MESOTHINI Portevin, 1931. Stem: *Mesothet*-.

***Mestogaster* Schmidt-Göbel, 1838**: 9. Current status: valid genus in STAPHYLINIDAE: PSELAPHINAE: PSELAPHITAE: HYBOCEPHALINI.

Type species: *Mestogastercrassicornis* Schmidt-Göbel, 1838, by monotypy.

Family-group name: MESTOGASTRINA Jakobson, 1910. Stem: *Mestogastr*-.

***Metacerylon* Grouvelle, 1906**: 110. Current status: valid genus in EUXESTIDAE.

Type species: *Metacerylonparallelum* Grouvelle, 1906, by monotypy.

Family-group name: METACERYLINI Heinze, 1944. Stem: *Metacerylon*-.

***Metachroma* Chevrolat in Dejean, 1836a**: 412. Current status: valid genus in CHRYSOMELIDAE: EUMOLPINAE: BROMIINI: TYPOPHORINI.

Type species: *Colaspisquercata* Fabricius, 1801, by subsequent designation (Crotch 1873b: 41).

Family-group name: MÉTACHROMITES Chapuis, 1874. Stem: *Metachromat*-.

***Metacinops* Kraatz, 1862a**: 117. Current status: valid genus in CURCULIONIDAE: ENTIMINAE: CYPHICERITAE: METACINOPINI.

Type species: *Metacinopsrhinomacer* Kraatz, 1862, by monotypy.

Family-group name: METACINOPINAE Reitter, 1913. Stem: *Metacinop*-.

***Metaclisa* Jacquelin du Val, 1861**: 296. Current status: valid genus in TENEBRIONIDAE: TENEBRIONINAE: METACLISINI. Note: nomen protectum (see Bouchard et al. 2007: 393).

Type species: *Platydemaparallela* Fairmaire, 1855 (= *Diaperisazurea* Waltl, 1838), by original designation.

Family-group name: METACLISINI Steiner, 2016: 542. Stem: *Metaclis*-.

***Metacycla* Baly, 1861**: 206. Current status: valid genus in CHRYSOMELIDAE: GALERUCINAE: METACYCLINI.

Type species: *Metacyclasallei* Baly, 1861, by original designation.

Family-group name: MÉTACYCLITES Chapuis, 1875. Stem: *Metacycl*-.

***Metadromius* Bedel, 1907**: 279. Current status: valid genus in CARABIDAE: HARPALINAE: LEBIINI: DROMIUSINA.

Type species: *Dromiusmyrmidon* Fairmaire, 1859, by subsequent designation (Jeannel 1942: 1078).

Family-group name: METADROMIINA Basilewsky, 1984. Stem: *Metadromi*-.

***Metagnoma* Aurivillius, 1925**: 13. Current status: valid genus in CERAMBYCIDAE: LAMIINAE: PTEROPLIINI.

Type species: *Metagnomasingularis* Aurivillus, 1925, by monotypy.

Family-group name: METAGNOMINI Aurivillius, 1925. Stem: *Metagnom*-.

***Metallica* Chaudoir, 1873**: 175. Current status: valid genus in CARABIDAE: HARPALINAE: LEBIINI: METALLICINA.

Type species: *Metallicaviridipennis* Chaudoir, 1873, by subsequent designation (Shpeley 1986: 273).

Family-group name: METALLICINI Basilewsky, 1984. Stem: *Metallic*-.

***Metanastes* Arrow, 1911**: 166. Current status: valid genus in SCARABAEIDAE: DYNASTINAE: PENTODONTINI: PENTODONTINA.

Type species: *Heteronychusaustralis* Fauvel, 1863, by original designation.

Family-group name: METANASTINA Carne, 1957. Stem: *Metanast*-.

***Metanoeus* C.O. Waterhouse, 1880a**: 73. Current status: valid genus in LYCIDAE: METRIORRHYNCHINAE: METRIORRHYNCHINI: METANOEINA.

Type species: *Lycusconformis* C.O. Waterhouse, 1878, by subsequent designation (Bourgeois 1891: 347).

Family-group name: METANOEINA Sklenarova, Kubecek & Bocák, 2014: 49. Stem: *Metanoe*-.

***Metapion* Schilsky, 1906**: v. Current status: valid genus in BRENTIDAE: APIONINAE: ASPIDAPIITAE: METAPIINI.

Type species: *Apioncandidum* Wencker, 1864, by original designation.

Family-group name: METAPIINI Alonso-Zarazaga, 1990. Stem: *Metapi*-.

***Metatyges* Pascoe, 1865b**: 424. Current status: junior synonym of *Omophorus* Schönherr, 1835 in CURCULIONIDAE: MOLYTINAE: METATYGINI.

Type species: *Metatygesturritus* Pascoe, 1865 (= *Omophorusstomachosus* Boheman, 1835), by monotypy.

Family-group name: METATYGINAE Pascoe, 1888. Stem: *Metatyg*-.

***Metaxina* Broun, 1909**: 407. Current status: valid genus in CHAETOSOMATIDAE.

Type species: *Metaxinaornata* Broun, 1909, by monotypy.

Family-group name: METAXINIDAE Kolibáč, 2004. Stem: *Metaxin*-.

***Metaxymorphus* Chaudoir, 1850**: 370. Current status: valid genus in CARABIDAE: HARPALINAE: LEBIINI: DROMIUSINA.

Type species: *Dromiusfrenatus* Dejean, 1831, by original designation.

Family-group name: METAXYMORPHINA Basilewsky, 1984. Stem: *Metaxymorph*-.

***Metazuphium* Mateu, 1992**: 198. Current status: valid genus in CARABIDAE: HARPALINAE: ZUPHIINI: METAZUPHIINA.

Type species: *Metazuphiumspinangulis* Mateu, 1992, by original designation.

Family-group name: METAZUPHIINA Mateu, 1992. Stem: *Metazuphi*-.

***Methia* Newman, 1842d**: 418. Current status: valid genus in CERAMBYCIDAE: CERAMBYCINAE: METHIINI. Note: new replacement name for *Thia* Newman, 1842.

Type species: *Thiapusilla* Newman, 1840 (= *Saperdanecydalea* Fabricius, 1798), by monotypy (type fixation for *Thia* Newman).

Family-group name: METHIITAE J. Thomson, 1860. Stem: *Methi*-.

***Methioides* Chemsak & Linsley, 1967**: 29, 31. Current status: valid genus in CERAMBYCIDAE: CERAMBYCINAE: OEMINI: METHIOIDINA.

Type species: *Methioidescicatricosa* Chemsak & Linsley, 1967, by original designation.

Family-group name: METHIOIDINA Martins, 1997. Stem: *Methioid*-.

***Methles* Sharp, 1882b**: 317, 489. Current status: valid genus in DYTISCIDAE: HYDROPORINAE: METHLINI.

Type species: *Methlespunctipennis* Sharp, 1882 (= *Hydroporuscribratellus* Fairmaire, 1880), by subsequent designation (Guignot 1946: 113).

Family-group name: METHLIDAE Branden, 1884. Stem: *Methl*-.

***Metholcus* Jacquelin du Val, 1860a**: 142. Current status: valid genus in PTINIDAE: XYLETININAE: METHOLCINI.

Type species: *Ptilinuscylindricus* Germar, 1817, by original designation. Note: *Trypopitysphoenicis* Fairmaire, 1859 is often listed as the valid name for *Ptilinuscylindricus* Germar, 1817 (e.g., Zahradník 2007: 361) based on the belief that Germar’s name is a primary homonym of *Ptilinuscylindricus* O.F. Müller, 1776; however, O.F. Müller’s name was suppressed for both the Principles of Priority and Homonymy (ICZN 1994a) and therefore *Ptilinuscylindricus* Germar, 1817 can be used as the valid name for the type species.

Family-group name: METHOLCINI Zahradník, 2009. Stem: *Metholc*-.

***Metialma* Pascoe, 1871a**: 217. Current status: valid genus in CURCULIONIDAE: CONODERINAE: CORYSSOMERINI.

Type species: *Metialmascenica* Pascoe, 1871, by subsequent designation (G.A.K. Marshall 1939b: 10).

Family-group name: METIALMINI Hustache, 1932. Stem: *Metialm*-.

***Metius* Curtis, 1838b**: 329. Current status: valid genus in CARABIDAE: HARPALINAE: PTEROSTICHINI: METIINA.

Type species: *Metiusharpalioides* Curtis, 1838, by monotypy.

Family-group name: METIINI Straneo, 1951. Stem: *Meti*-.

***Metocalolabus* Legalov, 2003**: 433. Current status: valid genus in ATTELABIDAE: ATTELABINAE: ATTELABINI: METOCALOLABINA.

Type species: *Metocalolabusorientalis* Legalov, 2003, by original designation.

Family-group name: METOCALOLABINA Legalov, 2003. Stem: *Metocalolab*-.

***Meton* Pascoe, 1859a**: 42. Current status: valid genus in CERAMBYCIDAE: LAMIINAE: DESMIPHORINI.

Type species: *Metongranulicollis* Pascoe, 1859, by monotypy.

Family-group name: MÉTONIDES Lacordaire, 1869. Stem: *Metont*-.

***Metopias* Gory, 1832**: pl. 42. Current status: valid genus in STAPHYLINIDAE: PSELAPHINAE: EUPLECTITAE: METOPIASINI: METOPIASINA.

Type species: *Metopiascurculionoides* Gory, 1832, by monotypy (see ICZN 1994e).

Family-group name: METOPIINI Raffray, 1904. Stem: *Metopias*- (ICZN 1994e).

***Metopocoilus* Audinet-Serville, 1832**: 170. Current status: valid genus in CERAMBYCIDAE: CERAMBYCINAE: TRACHYDERINI: TRACHYDERINA.

Type species: *Metopocoilusmaculicollis* Audinet-Serville, 1832, by monotypy.

Family-group name: METOPOCOÏLITAE J. Thomson, 1864. Stem: *Metopocoil*-.

***Metoponcus* Kraatz, 1857b**: 651. Current status: junior synonym of *Zeteotomus* Jacquelin du Val, 1856 in STAPHYLINIDAE: STAPHYLININAE: XANTHOLININI.

Type species: *Leptacinusbrevicornis* Erichson, 1839, by subsequent designation (R. Lucas 1920: 410). Note: two species were originally included in this genus, including *Leptacinusfilarius* Erichson, 1839.

Family-group name: METOPONCI Casey, 1906. Stem: *Metoponc*-.

***Metopsia* Wollaston, 1854**: 616. Current status: valid genus in STAPHYLINIDAE: PROTEININAE: PROTEININI.

Type species: *Metopsiaampliata* Wollaston, 1854, by monotypy.

Family-group name: METOPSIINAE Tottenham, 1954. Stem: *Metopsi*-.

***Metriorrhynchus* Gemminger in Gemminger and Harold, 1869c**: 1629. Current status: valid genus in LYCIDAE: METRIORRHYNCHINAE: METRIORRHYNCHINI: METRIORRHYNCHINA. Note: this name is an unjustified emendation of *Metriorhynchus* Guérin-Méneville, 1838 (a junior homonym of *Metriorhynchus* H. Meyer, 1830 [Reptilia]) since two or more names in Gemminger in Gemminger and Harold (1869c) are treated in the same way (ICZN 1999a, Article 33.2.1) by the inclusion of an additional “r” to the original spelling to form “*rrhynchus*” (rostrum) instead of “*rhynchus*,” e.g., *Psilorrhynchus* (p. 1657) and *Metriorrhynchus* (see Biffi 2017: 132).

Type species: *Lycusparallelus* Guérin-Méneville, 1838, by subsequent designation (C.O. Waterhouse 1878: 101).

Family-group name: METRIORRHYNCHINAE Kleine, 1926. Stem: *Metriorrhynch*-.

***Metrioxena* Pascoe, 1870**: 442. Current status: valid genus in BELIDAE: OXYCORYNINAE: OXYCORYNITAE: METRIOXENINI: METRIOXENINA.

Type species: *Metrioxenaserricollis* Pascoe, 1870, by monotypy.

Family-group name: METRIOXENINI Voss, 1953. Stem: *Metrioxen*-.

†***Metrioxenoides* Gratshev, Zherikhin & Jarzembowski, 1998**: 323. Current status: valid genus in NEMONYCHIDAE: †METRIOXENOIDINAE. Note: Lower Cretaceous (England).

Type species: †*Metrioxenoidespusillus* Gratshev, Zherikhin & Jarzembowski 1998, by original designation.

Family-group name: †METRIOXENOIDINAE Legalov, 2009. Stem: *Metrioxenoid*-.

***Metrius* Eschscholtz, 1829b**: 8. Current status: valid genus in CARABIDAE: PAUSSINAE: METRIINI.

Type species: *Metriuscontractus* Eschscholtz, 1829, by monotypy.

Family-group name: METRII J.L. LeConte, 1853. Stem: *Metri*-.

***Metrotyphlus* Coiffait, 1959**: 328. Current status: valid genus in STAPHYLINIDAE: LEPTOTYPHLINAE: METROTYPHLINI.

Type species: *Megatyphlushervei* Coiffait, 1957, by original designation.

Family-group name: METROTYPHLINI Coiffait, 1963. Stem: *Metrotyphl*-.

***Mezium* Leach in Samouelle, 1819**: 180. Current status: valid genus in PTINIDAE: PTININAE: MEZIINI. Note: this name is credited to Curtis (1828: pl. 232) in the literature but it was first made available by Leach in 1819.

Type species: *Ptinussulcatus* Fabricius, 1781, by original designation.

Family-group name: MEZIINI Bellés, 1985. Stem: *Mezi*-.

***Miarus* Schönherr, 1826**: 320. Current status: valid genus in CURCULIONIDAE: CURCULIONINAE: MECININI.

Type species: *Curculiocampanulae* Linnaeus, 1767, by original designation.

Family-group name: MIARIDES Tournier, 1874. Stem: *Miar*-.

***Miccotrogus* Schönherr, 1825**: 583. Current status: junior synonym of *Tychius* Germar, 1817 in CURCULIONIDAE: CURCULIONINAE: TYCHIINI: TYCHIINA.

Type species: *Curculiocuprifer* Panzer, 1799, by original designation.

Family-group name: MICCOTROGIDAE Gistel, 1856. Stem: *Miccotrog*-.

***Micholaemus* Viana, 1971**: 70. Current status: valid genus in RIPIPHORIDAE: PELECOTOMINAE.

Type species: *Micholaemusgerstaeckeri* Viana, 1971, by original designation.

Family-group name: MICHOLAEMINAE Viana, 1971. Stem: *Micholaem*-.

***Michthisoma* J.L. LeConte, 1850c**: 30. Current status: valid genus in CERAMBYCIDAE: SPONDYLIDINAE: SAPHANINI.

Type species: *Michthisomaheterodoxus* J.L. LeConte, 1850, by monotypy.

Family-group name: MICHTHYSOMINI J.L. LeConte, 1873. Stem: *Michthisomat*-.

***Micilus* Mulsant & Rey, 1872**: 15. Current status: valid genus in HETEROCERIDAE: HETEROCERINAE: MICILINI. Note: two original spellings were used, including *Mirulus* (p. 40); Neave (1940: 145) acted as First Reviser (ICZN 1999a, Article 24.2.3) and selected the spelling *Micilus*, the spelling currently in prevailing usage.

Type species: *Heterocerusmurinus* Kiensewetter, 1843, by monotypy.

Family-group name: MICILINI M.F. Pacheco, 1964. Stem: *Micil*-.

***Micracis* J.L. LeConte in C. Zimmermann, 1869**: 164. Current status: valid genus in CURCULIONIDAE: SCOLYTINAE: MICRACIDINI.

Type species: *Micracissuturalis* J.L. LeConte, 1869, by subsequent designation (Hopkins 1914: 125).

Family-group name: MICRACIDES J.L. LeConte, 1876. Stem: *Micracid*-.

***Micragyrtes* Champion, 1918**: 46. Current status: junior synonym of *Microsilpha* Broun, 1886 in STAPHYLINIDAE: MICROSILPHINAE.

Type species: *Micragyrtesocelligerus* Champion, 1918, by original designation.

Family-group name: MICRAGYRTINI Jeannel, 1962. Stem: *Micragyrt*-.

***Micralymma* Westwood, 1837**: 129. Current status: valid genus in STAPHYLINIDAE: OMALIINAE: OMALIINI.

Type species: *Micralymmajohnstonis* Westwood, 1837 (= *Staphylinusmarinus* Strøm, 1783), by monotypy.

Family-group name: MICRALYMMATES Mulsant & Rey, 1880. Stem: *Micralymmat*-.

***Micrantereus* Solier, 1848b**: 151, 175. Current status: valid genus in TENEBRIONIDAE: BLAPTINAE: PEDININI: HELOPININA.

Type species: *Acanthomerusanomalus* Guérin-Méneville, 1834, by original designation.

Family-group name: MICRANTEREINI Reitter, 1917. Stem: *Micrantere*-.

***Micratopus* Casey, 1914**: 42. Current status: valid genus in CARABIDAE: TRECHINAE: TACHYINI: TACHYINA.

Type species: *Micratopusfusciceps* Casey, 1914 (= *Blemusaenescens* J.L. LeConte, 1848), by monotypy.

Family-group name: MICRATOPINI Casey, 1914. Stem: *Micratop*-.

***Microcephalus* Dejean, 1828**: 4, 198. Current status: valid genus in CARABIDAE: HARPALINAE: PTEROSTICHINI: MICROCEPHALINA.

Type species: *Microcephalusdepressicollis* Dejean, 1828, by monotypy.

Family-group name: MICROCEPHALIDES Tschitschérine, 1898. Stem: *Microcephal*-.

***Microceroxenus* Kistner, 1970a**: 10. Current status: valid genus in STAPHYLINIDAE: ALEOCHARINAE: ATHETINI: MICROCEROXENINA.

Type species: *Microceroxenusalzadae* Kistner, 1970, by original designation.

Family-group name: MICROCEROXENINA Kistner, 1970. Stem: *Microceroxen*-.

***Microcerus* Schönherr, 1833**: 8. Current status: valid genus in BRENTIDAE: MICROCERINAE.

Type species: *Curculioretusus* Fabricius, 1781, by original designation.

Family-group name: MICROCÉRIDES Lacordaire, 1863. Stem: *Microcer*-.

***Microchaetes* Hope, 1834b**: 12. Current status: valid genus in BYRRHIDAE: SYNCALYPTINAE: MICROCHAETINI.

Type species: *Microchaetessphaericus* Hope, 1834, by monotypy.

Family-group name: MICROCHAETINI Paulus, 1973. Stem: *Microchaet*-. Note: junior homonym of MICROCHAETINI Beddard, 1895 (type genus *Microchaetus* Rapp, 1849) in Oligochaeta.

***Microcheila* Brullé, 1834**: 330, 336. Current status: valid genus in CARABIDAE: HARPALINAE: MICROCHEILINI.

Type species: *Microcheilapicea* Brullé, 1834, by monotypy.

Family-group name: MICROCHILITAE Jeannel, 1948. Stem: *Microcheil*-.

***Microcrania* Burmeister, 1855**: 11, 75. Current status: junior synonym of *Barybas* Blanchard, 1850 in SCARABAEIDAE: MELOLONTHINAE: MACRODACTYLINI.

Type species: *Philochloeniacompacta* Erichson, 1847, by subsequent designation (Evans 2003: 230).

Family-group name: MICROCRANIADAE Burmeister, 1855. Stem: *Microcrani*-.

***Microcymatura* Breuning, 1951**: 386. Current status: valid genus in CERAMBYCIDAE: LAMIINAE: MICROCYMATURINI.

Type species: *Microcymaturaantennalis* Breuning, 1951, by original designation.

Family-group name: MICROCYMATURINI Breuning & Téocchi, 1985. Stem: *Microcymatur*-.

***Microglotta* Kraatz, 1862b**: 300. Current status: junior synonym of *Haploglossa* Kraatz, 1856 in STAPHYLINIDAE: ALEOCHARINAE: OXYPODINI: MICROGLOTTINA. Note: unnecessary new replacement name for *Haploglossa* Kraatz, 1856.

Type species: fixed by Smetana (2004: 31) according to Article 70.3.2 (ICZN 1999a) as *Aleocharavillosula* Stephens, 1832, misidentified as *Aleocharapulla* Gravenhorst, 1802 (sensu Gyllenhal, 1827), in the subsequent designation by C.G. Thomson (1859: 33) (type fixation for *Haploglossa* Kraatz).

Family-group name: MICROGLOTTAE Fenyes, 1919. Stem: *Microglott*-.

***Microhoria* Chevrolat, 1877**: 168. Current status: valid genus in ANTHICIDAE: ANTHICINAE: MICROHORIINI. Note: two original spellings were used, including *Michrohoria* (p. 168); *Microhoria* was placed on the Official List of Generic Names in Zoology (ICZN 2016).

Type species: *Anthicusoedipus* Chevrolat, 1860, by subsequent designation (Bonadona 1952: 234; see ICZN 2016). Note: name placed on the Official List of Specific Names in Zoology (ICZN 2016).

Family-group name: MICROHORINI Bonadona, 1974. Stem: *Microhori*-.

***Microjulistus* Reitter, 1889c**: 111. Current status: valid genus in RHADALIDAE: RHADALINAE.

Type species: *Microjulistusfulvus* Reitter, 1889, by monotypy.

Family-group name: MICROJULISTINI Majer, 1987. Stem: *Microjulist*-.

***Micromalthus* J.L. LeConte, 1878b**: 613. Current status: valid genus in MICROMALTHIDAE.

Type species: *Micromalthusdebilis* J.L. LeConte, 1878, by monotypy.

Family-group name: MICROMALTHIDAE Barber, 1913. Stem: *Micromalth*-.

***Micropeplus* Latreille, 1809a**: 377. Current status: valid genus in STAPHYLINIDAE: MICROPEPLINAE.

Type species: *Staphylinusporcatus* Paykull, 1789, by monotypy.

Family-group name: MICROPEPLIDA Leach, 1815. Stem: *Micropepl*-.

***Micropsalis* Burmeister, 1861**: 314. Current status: invalid senior synonym of *Apterocaulus* Fairmaire, 1864 in CERAMBYCIDAE: PRIONINAE: PRIONINI. Note: junior homonym of *Micropsalis* H. Meyer 1859 [Crustacea].

Type species: *Micropsalisheterogama* Burmeister, 1861, by monotypy.

Family-group name: MICROPSALINI Gounelle, 1911. Stem: *Micropsalid*-. Note: family-group name permanently invalid (ICZN 1999a, Article 39).

***Microrhagus* Dejean, 1833a**: 85. Current status: valid genus in EUCNEMIDAE: MELASINAE: DIRHAGINI.

Type species: *Elaterpygmaeus* Fabricius, 1792, by subsequent designation (Westwood 1838c: 25).

Family-group name: MICRORHAGINAE Fleutiaux, 1919. Stem: *Microrhag*-.

***Microrhopala* Chevrolat in Dejean, 1836a**: 365. Current status: valid genus in CHRYSOMELIDAE: CASSIDINAE: CHALEPINI.

Type species: *Hispavittata* Fabricius, 1798, by subsequent designation (Baly 1864d: 268).

Family-group name: MICRORHOPALIDES G.H. Horn, 1883. Stem: *Microrhopal*-. Note: nomen oblitum (see Bouchard and Bousquet 2020a: 108).

***Microsilpha* Broun, 1886**: 889. Current status: valid genus in STAPHYLINIDAE: MICROSILPHINAE.

Type species: *Microsilphalitorea* Broun, 1886, by monotypy.

Family-group name: MICROSILPHINAE Crowson, 1950. Stem: *Microsilph*-.

***Micrositus* Mulsant & Rey, 1854**: 131, 148. Current status: valid genus in TENEBRIONIDAE: BLAPTINAE: DENDARINI: DENDARINA.

Type species: *Micrositusorbicularis* Mulsant & Rey, 1854, by subsequent designation (Gebien 1938b: 415).

Family-group name: MICROSITATES Mulsant & Rey, 1854. Stem: *Microsit*-.

***Microsporus* Kolenati, 1846**: 64. Current status: junior synonym of *Sphaerius* Waltl, 1838 in SPHAERIUSIDAE. Note: name placed on the Official List of Generic Names in Zoology (ICZN 1985d).

Type species: *Microsporusobsidianus* Kolenati, 1846 (= *Sphaeriusacaroides* Waltl, 1838), by monotypy (see ICZN 1985d). Note: *Microsporusobsidianus* Kolenati, 1846 was placed on the Official List of Specific Names in Zoology (ICZN 1985d).

Family-group name: MICROSPORIDAE Crotch, 1873. Stem: *Microspor*-.

***Microstylus* Schönherr, 1847**: 15. Current status: valid genus in CURCULIONIDAE: CURCULIONINAE: MICROSTYLINI.

Type species: *Microstylusrufus* Schönherr, 1847, by original designation. Note: combined description of a new nominal genus and a single new nominal species (ICZN 1999a, Article 12.2.6).

Family-group name: MICROSTYLIDES Lacordaire, 1865. Stem: *Microstyl*-. Note: this family- group name was treated as unavailable by Bouchard et al. (2011: 582) but has since been latinized, accepted as valid, and as dating from its first publication in vernacular form (e.g., Caldara et al. 2014: 611, Alonso-Zarazaga et al. 2017: 204), MICROSTYLINI Lacordaire, 1865 is therefore treated as available (ICZN 1999a, Article 11.7.2).

***Microtrachelizus* Senna, 1893**: 315. Current status: valid genus in BRENTIDAE: BRENTINAE: TRACHELIZINI: MICROTRACHELIZINA.

Type species: *Trachelizuslyratus* Perroud & Montrouzier, 1865, by subsequent designation (R. Lucas 1920: 417).

Family-group name: MICROTRACHELIZINA Zimmerman, 1994. Stem: *Microtracheliz*-.

***Microvalgus* Kraatz, 1883**: 374. Current status: valid genus in SCARABAEIDAE: CETONIINAE: VALGINI: MICROVALGINA.

Type species: *Valguslapeyrouse* Gory & Percheron, 1833, by subsequent designation (W.F. Kirby 1885: 71).

Family-group name: MICROVALGINAE Kolbe, 1904. Stem: *Microvalg*-.

***Microweisea* Cockerell, 1903**: 38. Current status: valid genus in COCCINELLIDAE: MICROWEISEINAE: MICROWEISEINI. Note: new replacement name for *Smilia* Weise, 1892.

Type species: *Smiliafelschei* Weise, 1892, by monotypy (type fixation for *Smilia* Weise).

Family-group name: MICROWEISEINI Leng, 1920. Stem: *Microweise*-.

***Microxylobius* Chevrolat, 1835a**: 98. Current status: valid genus in CURCULIONIDAE: COSSONINAE: MICROXYLOBIINI.

Type species: *Microxylobiuswestwoodii* Chevrolat, 1835, by monotypy.

Family-group name: MICROXYLOBIINI Voss, 1972. Stem: *Microxylobi*-.

***Microzaena* Fairmaire, 1901**: 106. Current status: valid genus in CARABIDAE: PAUSSINAE: MICROZAENINI.

Type species: *Microzaenamadecassa* Fairmaire, 1901, by monotypy.

Family-group name: MICROZAENINI Deuve, 2020: 203. Stem: *Microzaen*-.

***Microzoum* Dejean, 1834**: 193. Current status: junior synonym of *Melanimon* Steven, 1828 in TENEBRIONIDAE: TENEBRIONINAE: MELANIMONINI.

Type species: *Opatrumtibiale* Fabricius, 1781, by monotypy.

Family-group name: MICROZOUMATES Mulsant, 1854. Stem: *Microzo*-.

***Migadops* G.R. Waterhouse, 1842a**: 136. Current status: valid genus in CARABIDAE: MIGADOPINAE: MIGADOPINI: MIGADOPINA.

Type species: *Migadopsvirescens* G.R. Waterhouse, 1842 (= *Antarctialata* Guérin-Méneville, 1841), by subsequent designation (Jeannel 1938: 27).

Family-group name: MIGADOPIDAE Chaudoir, 1861. Stem: *Migadop*-.

***Mimanomma* Wasmann, 1912**: 478. Current status: valid genus in STAPHYLINIDAE: ALEOCHARINAE: MIMANOMMATINI: MIMANOMMATINA.

Type species: *Mimanommaspectrum* Wasmann, 1912, by monotypy.

Family-group name: MIMANOMMATINAE Wasmann, 1912. Stem: *Mimanommat*-.

†***Mimaphodius* Nikolajev, 2007a**: 47. Current status: valid genus in HYBOSORIDAE: †MIMAPHODIINAE. Note: Lower Cretaceous (Russia).

Type species: †*Mimaphodiuspusillus* Nikolajev, 2007, by original designation.

Family-group name: †MIMAPHODIINAE Nikolajev, 2007. Stem: *Mimaphodi*-.

***Mimastra* Baly, 1865a**: 253. Current status: valid genus in CHRYSOMELIDAE: GALERUCINAE: LUPERINI.

Type species: *Mimastraarcuata* Baly, 1865, by original designation.

Family-group name: MIMASTRITES Chapuis, 1875. Stem: *Mimastr*-.

***Mimeciton* Wasmann, 1893a**: 97. Current status: valid genus in STAPHYLINIDAE: ALEOCHARINAE: MIMECITINI: MIMECITINA.

Type species: *Mimecitonpulex* Wasmann, 1893, by monotypy.

Family-group name: MIMECITONINI Wasmann, 1909. Stem: *Mimecit*-.

***Mimela* W. Kirby, 1823a**: 101. Current status: valid genus in SCARABAEIDAE: RUTELINAE: ANOMALINI: ANOMALINA.

Type species: *Mimelachinensis* W. Kirby, 1823, by monotypy.

Family-group name: MIMELIDAE Theobald, 1882. Stem: *Mimel*-.

***Mimicoclytrina* Bellamy, 2003**: 26. Current status: valid genus in BUPRESTIDAE: POLYCESTINAE: TYNDARIDINI: MIMICOCLYTRININA. Note: new replacement name for *Acherusia* Laporte & Gory, 1838.

Type species: *Acherusiachildreni* Laporte & Gory, 1838, by monotypy (type fixation for *Acherusia* Laporte & Gory).

Family-group name: MIMICOCLYTRININA Bellamy, 2003. Stem: *Mimicoclytrin*-.

***Mimolibnetis* Pic, 1936**: 33 [suggested]. Current status: valid genus in LYCIDAE: DEXORINAE: MIMOLIBNETINI: MIMOLIBNETINA. Note: this name was first proposed by Pic (1936: 33) without a type species designation, a mandatory requirement for genus-group names proposed after 1930 (ICZN 1999a, Article 13.3) and it is therefore unavailable from that date; Kazantsev (2000: 12) designated *Mimolibnetisapicalis* Pic, 1936 as type species of the genus but since he did not indicate he was proposing a new taxon, a mandatory requirement for names published after 1999 (ICZN 1999a, Article 16.1), *Mimolibnetis* is also unavailable from that date; in order to promote stability, we believe that a request should be submitted to the Commission to accept *Mimolibnetis* Pic, 1936 as available, as well as the family-group name formed from it.

Type species [suggested]: *Mimolibnetisapicalis* Pic, 1936, by original designation.

Family-group name: MIMOLIBNETINAE Kazantsev, 2013a: 28. Stem: *Mimolibnet*-. Note: family- group name used as valid in recent classifications but it is not an available name since it was not formed from an available genus-group name (ICZN 1999a, Article 13.2.1; see comments above in the entry for *Mimolibnetis* Kazantsev, 2000).

***Mimonilla* Wasmann, 1913**: 380. Current status: valid genus in STAPHYLINIDAE: ALEOCHARINAE: MIMECITINI: MIMONILLINA.

Type species: *Mimonillaecitonis* Wasmann, 1913, by monotypy.

Family-group name: MIMONILLAE Bernhauer & Scheerpeltz, 1926. Stem: *Mimonill*-.

†***Mimoplatycis* Kazantsev, 2013b**: 288. Current status: valid genus in CANTHARIDAE: MALTHININAE: †MIMOPLATYCINI. Note: Eocene (Baltic amber)

Type species: †*Mimoplatycisnotha* Kazantsev, 2013, by original designation.

Family-group name: †MIMOPLATYCINI Kazantsev, 2013b: 288. Stem: *Mimoplatyc*-.

***Mimoscymnus* Gordon, 1994**: 692, 712. Current status: valid genus in COCCINELLIDAE: COCCINELLINAE: HYPERASPIDINI: MIMOSCYMNINA.

Type species: *Mimoscymnusaquilerai* Gordon, 1994, by original designation.

Family-group name: MIMOSCYMNINA Vandenberg & Hanson, 2019: 262. Stem: *Mimoscymn*-.

***Miniduliticola* Kazantsev, 2003**: 16. Current status: valid genus in LYCIDAE: LYROPAEINAE: MINIDULITICOLINI.

Type species: *Miniduliticolanelsoni* Kazantsev, 2003, by original designation.

Family-group name: MINIDULITICOLINI Kazantsev, 2003. Stem: *Miniduliticol*-.

***Minulus* Eggers, 1912**: 206. Current status: junior synonym of *Cnemonyx* Eichhoff, 1868 in CURCULIONIDAE: SCOLYTINAE: SCOLYTINI. Note: this name is not a junior homonym of *Minulus* Rafinesque, 1820 [Pisces] since Neave (1940: 183) rejected the spelling of Rafinesque’s name in favor of the alternative original spelling *Minnilus* (see Alonso- Zarazaga and Lyal 2009: 24).

Type species: *Minulusbarbatus* Eggers, 1912 (= *Cnemonyxgaleritus* Eichhoff, 1868), by monotypy.

Family-group name: MINULINI Reitter, 1913. Stem: *Minul*-.

***Minurus* G.R. Waterhouse, 1842b**: 68. Current status: valid genus in ATTELABIDAE: SAYREVILLEINAE: MINURINI.

Type species: *Minurustestaceus* G.R. Waterhouse, 1842, by monotypy.

Family-group name: MINURINI Legalov, 2003. Stem: *Minur*-.

***Minyomerus* G.H. Horn in J.L. LeConte and G.H. Horn, 1876**: 17. Current status: valid genus in CURCULIONIDAE: ENTIMINAE: POLYDRUSITAE: TANYMECINI: TANYMECINA.

Type species: *Minyomerusinnocuus* G.H. Horn, 1876, by subsequent designation (Pierce 1913: 400).

Family-group name: MINYOMERI G.H. Horn, 1876. Stem: *Minyomer*-.

***Minyops* Schönherr, 1823**: 1143. Current status: valid genus in CURCULIONIDAE: MOLYTINAE: MOLYTINI: PLINTHINA.

Type species: *Curculiocarinatus* Linnaeus, 1767, by original designation.

Family-group name: MINYOPIDAE Marseul, 1863. Stem: *Minyop*-.

†***Miocenocylas* Legalov, 2018a**: 32. Current status: valid genus in BRENTIDAE: BRENTINAE: CYLADINI: †MIOCENOCYLADINA. Note: mid-Miocene (Germany).

Type species: †*Miocenocylasheeri* Legalov, 2018, by original designation.

Family-group name: †MIOCENOCYLADINI Legalov, 2018a: 35. Stem: *Miocenocylad*-.

***Miroclaviger* Wasmann, 1893a**: 108, 111. Current status: valid genus in STAPHYLINIDAE: PSELAPHINAE: CLAVIGERITAE: CLAVIGERINI.

Type species: *Miroclavigercervicornis* Wasmann, 1893, by monotypy.

Family-group name: MIROCLAVIGERINI Jeannel, 1949. Stem: *Miroclaviger*-.

***Mirus* Saulcy, 1877**: 169. Current status: invalid senior synonym of *Imirus* Reitter, 1885 in STAPHYLINIDAE: PSELAPHINAE: GONIACERITAE: IMIRINI. Note: junior homonym of *Mirus* Albers, 1850 [Mollusca].

Type species: *Miruspermirus* Saulcy, 1877, by monotypy.

Family-group name: MIRINI Raffray, 1917. Stem: *Mir*-. Note: junior homonym of MIRIDAE Hahn, 1833 (type genus *Miris* Fabricius, 1794) in Hemiptera; family-group name permanently invalid (ICZN 1999a, Article 39).

***Miscelus* Klug, 1834**: 82. Current status: valid genus in CARABIDAE: HARPALINAE: LEBIINI: PERICALINA.

Type species: *Miscelusjavanus* Klug, 1834, by monotypy.

Family-group name: MISCELINI Sloane, 1907. Stem: *Miscel*-.

***Mischocephalus* Chaudoir, 1863a**: 318. Current status: valid genus in CARABIDAE: HARPALINAE: ZUPHIINI: MISCHOCEPHALINA.

Type species: *Mischocephalusspinicollis* Chaudoir, 1863, by monotypy.

Family-group name: MISCHOCEPHALINA Mateu, 1992. Stem: *Mischocephal*-.

***Misolampidius* Solsky, 1876**: 292. Current status: valid genus in TENEBRIONIDAE: STENOCHIINAE: CNODALONINI.

Type species: *Misolampidiustentyrioides* Solskly, 1876, by monotypy.

Family-group name: MISOLAMPIDIINI Reitter, 1917. Stem: *Misolampidi*-.

***Misolampus* Latreille, 1806**: 160. Current status: valid genus in TENEBRIONIDAE: STENOCHIINAE: CNODALONINI.

Type species: *Misolampushoffmanssegii* Latreille, 1806 (= *Pimeliagibbula* Herbst, 1799), by monotypy.

Family-group name: MISOLAMPIDES Lacordaire, 1859. Stem: *Misolamp*-.

***Mitracephala* Raffray, 1890a**: 104, 107. Current status: invalid senior synonym of *Mitrametopus* Raffray, 1911 in STAPHYLINIDAE: PSELAPHINAE: EUPLECTITAE: EUPLECTINI. Note: junior homonym of *Mitracephala* J. Thomson, 1859 [Coleoptera: SCARABAEIDAE].

Type species: *Mitracephalalongipennis* Raffray, 1890, by monotypy. Note: combined description of a new nominal genus and a single new nominal species (ICZN 1999a, Article 12.2.6).

Family-group name: MITRACEPHALINI Park, 1951. Stem: *Mitracephal*-. Note: family-group name permanently invalid (ICZN 1999a, Article 39).

***Mitraelabrus* Solier, 1851**: 259. Current status: valid genus in ANTHICIDAE: EURYGENIINAE: MITRAELABRINI.

Type species: *Mitraelabrusobscurus* Solier, 1851, by subsequent designation (M. Abdullah 1969: 351).

Family-group name: MITRAELABRINI M. Abdullah & A. Abdullah, 1968. Stem: *Mitraelabr*-.

***Mitrametopus* Raffray, 1911**: 50. Current status: valid genus in STAPHYLINIDAE: PSELAPHINAE: EUPLECTITAE: EUPLECTINI. Note: new replacement name for *Mitracephala* Raffray, 1890.

Type species: *Mitracephalalongipennis* Raffray, 1890, by monotypy (type fixation for *Mitracephala* Raffray).

Family-group name: MITRAMETOPINA Park, 1952. Stem: *Mitrametop*-.

***Mitrorhinus* Kaup, 1871**: 79. Current status: valid genus in PASSALIDAE: PASSALINAE: PASSALINI.

Type species: *Mitrorhinuspunctifrons* Kaup, 1871 (= *Passaluscayor* Percheron, 1834), by monotypy.

Family-group name: MITRORHINAE Kuwert, 1891. Stem: *Mitrorhin*-.

***Mniophila* Stephens, 1831b**: 330. Current status: valid genus in CHRYSOMELIDAE: GALERUCINAE: ALTICINI.

Type species: *Alticamuscorum* J.D.W. Koch, 1803, by monotypy.

Family-group name: MNIOPHILITES Chapuis, 1875. Stem: *Mniophil*-.

***Molops* Bonelli, 1810**: Tab. Syn. Current status: valid genus in CARABIDAE: HARPALINAE: PTEROSTICHINI: PTEROSTICHINA.

Type species: *Carabuselatus* Fabricius, 1801, by subsequent designation (Stephens 1832c: 248). Note: no species were originally included in this genus by Bonelli (1810); the first ones were the three species listed by Panzer (1813: 69); the species often listed as type species of this genus is *Carabusterricola* Herbst, 1784 sensu Fabricius, 1792 (= *Scaritespiceus* Panzer, 1793) (e.g., Bousquet 2017b: 680) as designated by Hope (1838a: 71) but Stephens’ designation is earlier; both nominal species are currently included in the nominotypical subgenus of *Molops*.

Family-group name: MOLOPIDES Bonelli, 1810. Stem: *Molop*-.

***Molorchus* Fabricius, 1792b**: 356. Current status: valid genus in CERAMBYCIDAE: CERAMBYCINAE: MOLORCHINI.

Type species: *Necydalisumbellatarum* Schreber, 1759, by subsequent designation (Curtis 1824: pl. 11).

Family-group name: MOLORCHIDAE Gistel, 1848. Stem: *Molorch*-.

***Moluris* Latreille, 1802b**: 169. Current status: valid genus in TENEBRIONIDAE: PIMELIINAE: SEPIDIINI: MOLURINA.

Type species: *Pimeliagibba* Fabricius, 1787 (= *Tenebriogibbus* Pallas, 1781), by monotypy.

Family-group name: MOLURITES Solier, 1834. Stem: *Molur*-.

***Molytes* Schönherr, 1823**: 1143. Current status: junior synonym of *Liparus* G.-A. Olivier, 1807 in CURCULIONIDAE: MOLYTINAE: MOLYTINI: MOLYTINA.

Type species: *Curculiogermanus* Linnaeus, 1758, by original designation.

Family-group name: MOLYTIDES Schönherr, 1823. Stem: *Molyt*-.

***Mombasica* Fairmaire, 1888b**: 363. Current status: valid genus in CHRYSOMELIDAE: GALERUCINAE: GALERUCINI. Note: unnecessary new replacement name for *Mombasa* Fairmaire, 1884.

Type species: *Mombasaarmicollis* Fairmaire, 1884, by monotypy (type fixation for *Mombasa* Fairmaire).

Family-group name: MOMBASICITES Laboissière, 1922. Stem: *Mombasic*-.

***Monachulus* Leng, 1918**: 208. Current status: junior synonym of *Lexiphanes* Gistel, 1848 in CHRYSOMELIDAE: CRYPTOCEPHALINAE: CRYPTOCEPHALINI: MONACHULINA. Note: new replacement name for *Monachus* Chevrolat, 1836.

Type species: *Cryptocephalussaponatus* Fabricius, 1801, by monotypy (type fixation for *Monachus* Chevrolat).

Family-group name: MONACHULINI Leng, 1920. Stem: *Monachul*-.

***Monachus* Chevrolat in Dejean, 1836a**: 425. Current status: invalid senior synonym of *Lexiphanes* Gistel, 1848 in CHRYSOMELIDAE: CRYPTOCEPHALINAE: CRYPTOCEPHALINI: MONACHULINA. Note: junior homonym of *Monachus* Fleming, 1822 [Mammalia].

Type species: *Cryptocephalussaponatus* Fabricius, 1801, by monotypy.

Family-group name: MONACHITES Chapuis, 1874. Stem: *Monach*-. Note: senior homonym of MONACHINA J.E. Gray, 1869 (type genus *Monachus* Fleming, 1822) in Mammalia; the Coleoptera family-group name permanently invalid (ICZN 1999a, Article 39).

***Monardita* Bechyné, 1948**: 534. Current status: valid genus in CHRYSOMELIDAE: CHRYSOMELINAE: CHRYSOMELINI.

Type species: *Chrysomelaopulenta* Reiche, 1848 (= *Sphaeratrixlatifrons* Gistel, 1848), by original designation.

Family-group name: MONARDITINI Bechyné, 1953. Stem: *Monardit*-.

***Moneilema* Say, 1824**: 403. Current status: valid genus in CERAMBYCIDAE: LAMIINAE: ACANTHOCININI.

Type species: *Moneilemaannulata* Say, 1824, by monotypy.

Family-group name: MONEILEMITAE J. Thomson, 1864. Stem: *Moneilem*-.

†***Mongolobrenthorrhinus* Gratshev & Legalov in Legalov, 2011c**: 64. Current status: valid genus in CARIDAE: †BAISSORHYNCHINAE: †MONGOLOBRENTHORRHININI. Note: Lower Cretaceous (Mongolia).

Type species: †*Mongolobrenthorrhinuspusillus* Gratshev & Legalov, 2011, by original designation.

Family-group name: †MONGOLOBRENTHORRHININI Gratshev & Legalov in Legalov, 2011a: 64. Stem: *Mongolobrenthorrhin*-.

†***Mongolocar* Gratshev & Legalov, 2011**: 69. Current status: valid genus in BRENTIDAE: ITHYCERINAE: †MONGOLOCARINI. Note: Lower Cretaceous (Mongolia).

Type species: †*Mongolocarorcinus* Gratshev & Legalov, 2011, by original designation.

Family-group name: †MONGOLOCARINAE Gratshev & Legalov, 2011: 69. Stem: *Mongolocar*-.

***Monochamus* Dejean, 1821**: 106. Current status: valid genus in CERAMBYCIDAE: LAMIINAE: LAMIINI.

Type species: *Cerambyxsutor* Linnaeus, 1758, by subsequent designation (Curtis 1828: pl. 219).

Family-group name: MONOHAMMIDAE Gistel, 1848. Stem: *Monocham*-.

***Monochirus* Chapuis, 1875**: 330. Current status: invalid senior synonym of *Hispellinus* Weise, 1898 in CHRYSOMELIDAE: CASSIDINAE: HISPINI. Note: junior homonym of *Monochirus* Rafinesque, 1814 [Pisces].

Type species: *Hispacallicantha* H.W. Bates, 1866, by subsequent monotypy (Chapuis 1876: xxv).

Family-group name: MONOCHIRINI Weise, 1905. Stem: *Monochir*-. Note: family-group name permanently invalid (ICZN 1999a, Article 39).

***Monocoryna* Gorham, 1885b**: 527. Current status: valid genus in COCCINELLIDAE: MONOCORYNINAE.

Type species: *Monocorynadecempunctata* Gorham, 1885, by monotypy.

Family-group name: MONOCORYNINI Miyatake, 1988. Stem: *Monocoryn*-. Note: junior homonym of MONOCORYNINAE Rees, 1956 (type genus *Monocoryne* Broch, 1910) in Cnidaria.

***Monocrepidius* Eschscholtz, 1829a**: 31. Current status: valid genus in ELATERIDAE: AGRYPNINAE: OOPHORINI. Note: the First Reviser (ICZN 1999a, Article 24.2.2) for *Monocrepidius* Eschscholtz, 1829 versus *Conoderus* Eschscholtz, 1829 is Candèze (1859: 187).

Type species: *Monocrepidiuspallipes* Eschscholtz, 1829, by subsequent designation (Hyslop 1921: 657).

Family-group name: MONOCRÉPIDIITES Candèze, 1859. Stem: *Monocrepidi*-.

***Monodesmus* Audinet-Serville, 1832**: 160. Current status: valid genus in CERAMBYCIDAE: PRIONINAE: MEROSCELISINI.

Type species: *Monodesmuscallidioides* Audinet-Serville, 1832, by monotypy.

Family-group name: MONODESMINAE Gahan, 1890. Stem: *Monodesm*-.

***Monoedus* G.H. Horn, 1882**: 116. Current status: valid genus in ZOPHERIDAE: COLYDIINAE: ADIMERINI.

Type species: *Monoedusguttatus* G.H. Horn, 1882, by monotypy.

Family-group name: MONOEDIDAE Schaeffer, 1911. Stem: *Monoed*-.

***Monolepta* Chevrolat in Dejean, 1836a**: 383. Current status: valid genus in CHRYSOMELIDAE: GALERUCINAE: LUPERINI.

Type species: *Criocerisbioculata* Fabricius, 1781, by subsequent designation (Chevrolat 1845: 5).

Family-group name: MONOLEPTITES Chapuis, 1875. Stem: *Monolept*-.

***Monolobus* Solier, 1849**: 187. Current status: valid genus in CARABIDAE: MIGADOPINAE: MIGADOPINI: MIGADOPINA.

Type species: *Monolobustestaceus* Solier, 1849, by monotypy.

Family-group name: MONOLOBINAE Jeannel, 1938. Stem: *Monolob*-.

***Monomacra* Chevrolat in Dejean, 1836a**: 389. Current status: valid genus in CHRYSOMELIDAE: GALERUCINAE: ALTICINI.

Type species [suggested]: *Alticainermis* Klug, 1829, by subsequent designation (Monrós and Bechyné 1956: 1133). Note: the valid type species fixation is that of Chevrolat (1845: 6), who selected *Alticatibialis* G.-A. Olivier, 1808, but that species is currently included in the genus *Parchicola* Bechyné & Springlovà de Bechyné, 1975; an application to the Commission is necessary to fix *Alticainermis* Klug, 1829 as the type species of *Monomacra* Chevrolat, 1836.

Family-group name: MONOMACRINA Bechyné & Špringlová de Bechyné, 1975. Stem: *Monomacr*-.

***Monomma* Klug, 1833**: 94. Current status: valid genus in ZOPHERIDAE: ZOPHERINAE: MONOMMATINI.

Type species: *Monommairroratum* Klug, 1833, by monotypy. Note: combined description of a new genus-group taxon and a single new species (ICZN 1999a, Article 12.2.6).

Family-group name: MONOMMITES Blanchard, 1845. Stem: *Monommat*-.

***Mononychus* Germar, 1823**: 241. Current status: valid genus in CURCULIONIDAE: CEUTORHYNCHINAE: MONONYCHINI. Note: name placed on the Official List of Generic Names in Zoology (ICZN 1989b).

Type species: *Curculiopseudacori* Fabricius, 1792 (= *Curculiopunctumalbum* Herbst, 1784), by subsequent designation (Schönherr 1825: 586; see ICZN 1989b). Note: *Curculiopunctumalbum* Herbst, 1784 placed on the Official List of Specific Names in Zoology (ICZN 1989b).

Family-group name: MONONYCHI J.L. LeConte, 1876. Stem: *Mononych*-. Note: junior homonym of MONONYCHIDAE Fieber, 1851 (type genus *Mononyx* Laporte, 1832) in Hemiptera.

***Monoplatus* Chevrolat, 1846b**: 333. Current status: junior synonym of *Sphaeronychus* Dejean, 1836 in CHRYSOMELIDAE: GALERUCINAE: ALTICINI.

Type species: *Monoplatusnigripes* Clark, 1860, by subsequent designation (Scherer 1962: 501). Note: no species were originally included in this genus by Chevrolat (1846b); the first ones were the 20 species listed by Clark (1860: 5–21, 278–280).

Family-group name: MONOPLATITES Chapuis, 1875. Stem: *Monoplat*-.

***Monotoma* Herbst, 1793**: 22. Current status: valid genus in MONOTOMIDAE: MONOTOMINAE: MONOTOMINI. Note: nomen protectum (see Bouchard et al. 2011: 359).

Type species: *Monotomapicipes* Herbst, 1793, by subsequent designation (Stephens 1832c: 264).

Family-group name: MONOTOMITES Laporte, 1840. Stem: *Monotom*-.

†***Montsecanomalus* Soriano, Gratshev & Delclòs, 2006**: 558. Current status: valid genus in BRENTIDAE: ITHYCERINAE: †MONTSECANOMALINI. Note: Lower Cretaceous (Spain).

Type species: †*Montsecanomaluszherikhini* Soriano, Gratshev & Delclòs, 2006, by original designation.

Family-group name: †MONTSECANOMALINI Legalov, 2016: 1466. Stem: *Montsecanomal*-.

†***Montsecbelus* Zherikhin & Gratshev, 1997**: 626. Current status: valid genus in BELIDAE: †MONTSECBELINAE: †MONTSECBELINI. Note: Lower Cretaceous (Spain).

Type species: †*Eobelussolutus* Whalley & Jarzembowski, 1985, by original designation.

Family-group name: †MONTSECBELINAE Legalov, 2016: 1474. Stem: *Montsecbeli*-.

†***Moravocoleus* Kukalová, 1969**: 141. Current status: valid genus in †TSHEKARDOCOLEIDAE. Note: lower Permian (Czech Republic).

Type species: †*Moravocoleuspermianus* Kukalová, 1969, by original designation.

Family-group name: †MORAVOCOLEIDAE Kukalová-Peck & Beutel, 2012: 642. Stem: *Moravocole*-.

***Mordella* Linnaeus, 1758**: 342, 420. Current status: valid genus in MORDELLIDAE: MORDELLINAE: MORDELLINI.

Type species: *Mordellaaculeata* Linnaeus, 1758, by subsequent designation (Latreille 1810: 430).

Family-group name: MORDELLONAE Latreille, 1802. Stem: *Mordell*-.

***Mordellistena* A. Costa, 1854**: 16. Current status: valid genus in MORDELLIDAE: MORDELLINAE: MORDELLISTENINI.

Type species: *Mordellistenaconfinis* A. Costa, 1854, by subsequent designation (Chûjô 1935: 80).

Family-group name: MORDELLISTENINI Ermisch, 1941. Stem: *Mordellisten*-.

***Mordellochroa* Emery, 1876**: 80. Current status: valid genus in MORDELLIDAE: MORDELLINAE: MORDELLOCHROINI.

Type species: *Mordellaabdominalis* Fabricius, 1775, by subsequent designation (Ermisch 1954: 133).

Family-group name: MORDELLOCHROINI Odnosum, 2010. Stem: *Mordellochro*-.

***Morimonella* Podaný, 1979**: 43. Current status: valid genus in CERAMBYCIDAE: LAMIINAE: MORIMONELLINI.

Type species: *Morimonellabednariki* Podaný, 1979, by original designation.

Family-group name: MORIMONELLINI Lobanov, Danilevsky & Murzin, 1981. Stem: *Morimonell*-.

***Morimopsis* J. Thomson, 1857**: 182. Current status: valid genus in CERAMBYCIDAE: LAMIINAE: MORIMOPSINI.

Type species: *Morimopsislacrymans* J. Thomson, 1857, by monotypy.

Family-group name: MORIMOPSIDES Lacordaire, 1869. Stem: *Morimops*-.

***Morimus* Brullé, 1832**: 258. Current status: valid genus in CERAMBYCIDAE: LAMIINAE: LAMIINI. Note: name proposed as *Morinus*, but the spelling was corrected to *Morimus* in the Errata issued in 1833 in a subsequent part of the same volume.

Type species: *Lamialugubris* Fabricius, 1792 (= *Cerambyxasper* Sulzer, 1776), by subsequent designation (J. Thomson 1864: 77).

Family-group name: MORIMITAE J. Thomson, 1864. Stem: *Morim*-.

***Moriomorpha* Laporte, 1867**: 37. Current status: valid genus in CARABIDAE: PSYDRINAE: MORIOMORPHINI: MORIOMORPHINA.

Type species: *Moriomorphavictoriae* Laporte, 1867, by subsequent designation (B.P. Moore 1963b: 281).

Family-group name: MORIOMORPHINI Sloane, 1890. Stem: *Moriomorph*-.

***Morion* Latreille, 1810**: 159. Current status: valid genus in CARABIDAE: HARPALINAE: MORIONINI.

Type species: *Harpalusmonilicornis* Latreille, 1805, by monotypy.

Family-group name: MORIONIENS Brullé, 1835. Stem: *Morion*-.

***Mormolyce* Hagenbach, 1825**: 3. Current status: valid genus in CARABIDAE: HARPALINAE: LEBIINI: PERICALINA.

Type species: *Mormolycephyllodes* Hagenbach, 1825, by monotypy.

Family-group name: MORMOLYCIDAE E. Desmarest, 1851. Stem: *Mormolyc*-.

***Morostoma* Candèze, 1879**: 485. Current status: valid genus in ELATERIDAE: MOROSTOMINAE.

Type species: *Morostomapalpale* Candèze, 1879, by monotypy.

Family-group name: MOROSTOMINAE Dolin, 2000. Stem: *Morostom*- (ICZN 1999a, Article 29.4).

***Morphozonitis* Pic, 1922**: 16. Current status: valid genus in MELOIDAE: ELETICINAE: ERTLIANINI.

Type species: *Morphozonitisatripennis* Pic, 1922, by monotypy.

Family-group name: MORPHOZONITINI Kaszab, 1969. Stem: *Morphozonitid*-.

***Morychus* Erichson, 1846**: 468. Current status: valid genus in BYRRHIDAE: BYRRHINAE: MORYCHINI.

Type species: *Byrrhusaeneus* Fabricius, 1775, by subsequent designation (C.G. Thomson 1859: 74). Note: no species were originally included in this genus by Erichson (1846); the first ones were the three species listed by Erichson (1847a: 492, 493).

Family-group name: MORYCHINI El Moursy, 1961. Stem: *Morych*-.

***Mosotalesus* Kishii, 1977**: 31, 32. Current status: valid genus in ELATERIDAE: DENDROMETRINAE: SELATOSOMINI: MOSOTALESINA.

Type species: *Elaterimpressus* Fabricius, 1792, by original designation.

Family-group name: MOSOTALESINA Schimmel, Tarnawski, Han & Platia, 2015: 31. Stem: *Mosotales*-.

***Muonaja* Özdikmen, 2008**: 675. Current status: valid genus in EUCNEMIDAE: EUCNEMINAE: MUONAJINI. Note: new replacement name for *Yanga* Muona, 1993.

Type species: *Yangayonde* Muona, 1993, by original designation (type fixation for *Yanga* Muona).

Family-group name: MUONAJINI Özdikmen, 2008. Stem: *Muonaj*-.

***Murmidius* Leach, 1821**: 41. Current status: valid genus in MURMIDIIDAE.

Type species: *Murmidiusferrugineus* Leach, 1821 (= *Histerovalis* Beck, 1817), by monotypy.

Family-group name: MURMIDIIDES Jacquelin du Val, 1858. Stem: *Murmidi*-. Note: nomen protectum (see Bouchard and Bousquet 2020a: 128).

†***Myanmarops* Legalov & Kirejtshuk in Legalov et al., 2020a**: 2. Current status: valid genus in CHRYSOMELIDAE: BRUCHINAE: †MYANMAROPINI. Note: mid-Cretaceous (Myanmar).

Type species: †*Myanmaropsdamgaardi* Legalov & Kirejtshuk, 2020, by original designation.

Family-group name: †MYANMAROPINI Legalov & Kirejtshuk in Legalov et al., 2020a: 2. Stem: *Myanmarop*-.

***Myas* Sturm, 1826**: 6, 171. Current status: valid genus in CARABIDAE: HARPALINAE: PTEROSTICHINI: PTEROSTICHINA.

Type species: *Abaxchalybaeus* Palliardi, 1825, by monotypy.

Family-group name: MYADINA Jakobson, 1907. Stem: *Myad*-.

***Mycetaea* Stephens, 1829b**: 8. Current status: valid genus in MYCETAEIDAE.

Type species: **here fixed** (under ICZN 1999a, Article 70.3.2) as *Dermestessubterraneus* Fabricius, 1801, misidentified as *Dermestesfumatus* Linnaeus, 1767 (sensu Marsham, 1802), in the original designation by monotypy in Stephens (1829b: 8).

Family-group name: MYCETÉIDES Jacquelin du Val, 1857. Stem: *Mycetae*-.

***Mycetina* Mulsant, 1846**: 15. Current status: valid genus in ENDOMYCHIDAE: LYCOPERDININAE.

Type species: *Chrysomelacruciata* Schaller, 1783, by monotypy.

Family-group name: MYCETININA Lafer, 1992: 330. Stem: *Mycetin*-. Note: this name was overlooked in Bouchard et al. (2011) and Bouchard and Bousquet (2020a).

***Mycetochara* Guérin-Méneville, 1827**: 346. Current status: valid genus in TENEBRIONIDAE: ALLECULINAE: ALLECULINI: MYCETOCHARINA. Note: new replacement name for *Mycetophila* Gyllenhal, 1810.

Type species [suggested]: *Cistelaflavipes* Fabricius, 1792, by subsequent designation (C.G. Thomson 1859: 118; typification for *Mycetophila* Gyllenhal). Note: there are three earlier type species designations based on an originally included nominal species that threaten the stability of this genus: Guérin-Méneville (1827: 346), who selected *Cistelahumeralis* Fabricius, 1787, Stephens (1832c: 375), who selected *Cistelalinearis* Illiger, 1794 (= *Cistelamaura* Fabricius, 1792) for *Mycetocharis* Gyllenhal, 1827 (another new replacement name for *Mycetophila* Gyllenhal, 1810), and Westwood (1838c: 32), who selected *Cistelascapularis* Illiger, 1805 (= *Cistelahumeralis* Fabricius, 1787) for *Mycetochara* (as *Mycetocharus*); all these species are currently placed in the subgenus Ernocharis C.G. Thomson, 1859, not the nominotypical subgenus; an application to the Commission is necessary to fix *Cistelaflavipes* Fabricius, 1792 as the type species of *Mycetophila* Gyllenhal, 1810, for which *Mycetochara* Guérin-Méneville, 1827 is a new replacement name.

Family-group name: MYCETOCHARISIDAE Gistel, 1848. Stem: *Mycetochar*-.

***Mycetoma* Dejean, 1834**: 201. Current status: valid genus in TETRATOMIDAE: HALLOMENINAE.

Type species: *Dryopssuturalis* Panzer, 1797, by monotypy.

Family-group name: MYCÉTOMIENS Mulsant, 1856. Stem: *Mycetomat*-.

***Mycetophagus* Fabricius, 1792b**: 497. Current status: valid genus in MYCETOPHAGIDAE: MYCETOPHAGINAE: MYCETOPHAGINI.

Type species: *Silphaquadrimaculata* Schaller, 1783, by subsequent designation (Latreille 1810: 431).

Family-group name: MYCETOPHAGIDA Leach, 1815. Stem: *Mycetophag*-.

***Mycetoporus* Mannerheim, 1830**: 62. Current status: valid genus in STAPHYLINIDAE: MYCETOPORINAE. Note: name placed on the Official List of Generic Names in Zoology (ICZN 1993d, as *Mycetoporus* Mannerheim, 1831).

Type species: *Tachinuspunctus* Gravenhorst, 1806, by subsequent designation (C.G. Thomson 1859: 47; see ICZN 1993d). Note: name placed on the Official List of Specific Names in Zoology (ICZN 1993d).

Family-group name: MYCETOPORIDES C.G. Thomson, 1859. Stem: *Mycetopor*-.

***Mychothenus* Strohecker, 1953**: 24. Current status: valid genus in ANAMORPHIDAE. Note: new replacement name for *Mychophilus* Frivaldsky, 1877.

Type species: *Mychophilusminutus* Frivaldsky, 1877, by monotypy (type fixation for *Mychophilus* Frivaldsky).

Family-group name: MYCHOTHENINAE Sasaji, 1978. Stem: *Mychothen*-.

***Mycteis* Pascoe, 1860a**: 44. Current status: valid genus in ANTHRIBIDAE: ANTHRIBINAE: MYCTEINI.

Type species: *Mycteismarginicollis* Pascoe, 1860, by subsequent designation (Trýzna and Valentine 2011: 94).

Family-group name: MYCTEINI Morimoto, 1972. Stem: *Mycte*-.

***Mycterus* Clairville, 1798**: 56, 124. Current status: valid genus in MYCTERIDAE: MYCTERINAE.

Type species: *Mycterusgriseus* Clairville, 1798 (= *Rhinomacercurculioides* Fabricius, 1781), by monotypy.

Family-group name: MYCTERINA Perty, 1840. Stem: *Mycter*-.

***Mylabris* Fabricius, 1775**: 261. Current status: valid genus in MELOIDAE: MELOINAE: MYLABRINI. Note: the senior homonym *Mylabris*Geoffroy (1762: 163) was suppressed for both Principles of Priority and Homonymy and placed on the Official Index of Generic Names in Zoology while *Mylabris* Fabricius, 1775 was placed on the Official List of Generic Names in Zoology (ICZN 1995b).

Type species: *Meloecichorii* Linnaeus, 1758, by subsequent designation (Latreille 1810: 430; see ICZN 1995b). Note: *Meloecichorii* Linnaeus, 1758 was placed on the Official List of Specific Names in Zoology (ICZN 1995b); currently, that species is placed in the genus *Hycleus* Latreille, 1817 (Bologna 2020: 523); the Linnean syntypes consist of two species, one corresponding to *Hycleuschicorii* (Linnaeus, 1758) and the second to *Mylabrisvariabilis* (Pallas, 1781); a lectotype needs to be designated in order to clarify the application of the name *Meloecichorii* Linnaeus, 1758 (see Löbl 2008a: 45).

Family-group name: MYLABRIA Rafinesque, 1815. Stem: *Mylabr*-.

***Mylacorrhina* Reitter, 1913a**: 13. Current status: junior synonym of *Altonomus* Desbrochers des Loges, 1907 in CURCULIONIDAE: ENTIMINAE: CYPHICERITAE: CYPHICERINI: MYLACORRHININA.

Type species: *Mylacusvillosus* Reitter, 1906, by subsequent designation (Yunakov 2013: 65).

Family-group name: MYLACORRHYNCHINA Reitter, 1913. Stem: *Mylacorrhin*-.

***Mylacus* Boheman in Schönherr, 1842b**: 144. Current status: junior synonym of *Omias* Germar, 1817 in CURCULIONIDAE: ENTIMINAE: CYPHICERITAE: OMIINI.

Type species: *Mylacusmurinus* Boheman, 1842, by original designation.

Family-group name: MYLACINI Reitter, 1913. Stem: *Mylac*-.

***Mylassa* Stål, 1857**: 60. Current status: valid genus in CHRYSOMELIDAE: CRYPTOCEPHALINAE: MYLASSINI.

Type species: *Mylassafasciatipennis* Stål, 1857 (= *Pachybrachiscrassicollis* Blanchard, 1851), by subsequent designation (Jakobson 1924: 258). Note: Jakobson (1924: 257) selected *Pachybrachiscrassicollis* Blanchard, 1851 as type species, which was not an originally included nominal species, but since he listed it in synonymy with *Mylassafasciatipennis* Stål, 1857, one of the two nominal species originally included in *Mylassa*, he is deemed to have designated the latter species as type species (ICZN 1999a, Article 69.2.2).

Family-group name: MYLASSINI Gómez-Zurita & Cardoso, 2021: 605. Stem: *Mylass*-.

***Myllaena* Erichson, 1837**: 382. Current status: valid genus in STAPHYLINIDAE: ALEOCHARINAE: MYLLAENINI.

Type species: *Aleocharadubia* Gravenhorst, 1806, by subsequent designation (Duponchel 1841b: 57, as *Myllana*).

Family-group name: MYLLAENINI Ganglbauer, 1895. Stem: *Myllaen*-.

***Myllocerus* Schönherr, 1823**: 1144. Current status: valid genus in CURCULIONIDAE: ENTIMINAE: CYPHICERITAE: CYPHICERINI: MYLLOCERINA.

Type species: *Curculiocurvicornis* Fabricius, 1792, by original designation.

Family-group name: MYLLOCERINI Pierce, 1913. Stem: *Myllocer*-.

***Myloechus* Latreille, 1806**: 30. Current status: valid subgenus of *Colon* Herbst, 1797 in COLONIDAE.

Type species: *Myloechusbrunneus* Latreille, 1806, by monotypy.

Family-group name: MYLOECHINA C.G. Thomson, 1859. Stem: *Myloech*-.

***Myochrous* Erichson, 1847c**: 164. Current status: valid genus in CHRYSOMELIDAE: EUMOLPINAE: BROMIINI.

Type species: *Myochrousimmundus* Erichson, 1847, by monotypy.

Family-group name: MYOCHROINAE Baly, 1865. Stem: *Myochro*-.

***Myoderma* Dejean, 1833b**: 168. Current status: valid genus in SCARABAEIDAE: CETONIINAE: TRICHIINI. Note: this genus-group name is often listed as “*Myodermum* Burmeister & Schaum, 1840” in the literature but Dejean’s name has precedence (see Bousquet and Bouchard 2013a: 38).

Type species: *Stripsyphersordidus* Gory & Percheron, 1833 (= *Trichiusalutaceum* Afzelius, 1817), by monotypy.

Family-group name: MYODERMINI Péringuey, 1907. Stem: *Myodermat*-.

***Myodites* Latreille, 1819b**: 303. Current status: junior synonym of *Ripiphorus* Bosc, 1791 in RIPIPHORIDAE: RIPIPHORINAE: RIPIPHORINI. Note: new replacement name for *Myodes* Latreille, 1818; the original spelling was *Myodite* but *Myodites*, an incorrect subsequent spelling, is in prevailing usage, attributed to the publication of the original spelling, and therefore deemed to be the correct original spelling (ICZN 1999a, Article 33.3.1).

Type species: *Myodesdorthesii* Latreille, 1818 (= *Ripiphorussubdipterus* Fabricius, 1792), by monotypy (type fixation for *Myodes* Latreille).

Family-group name: MYODITINI Gerstaecker, 1855. Stem: *Myodit*-.

***Myorhinus* Schönherr, 1826**: 213. Current status: junior synonym of *Apsis* Germar, 1820 in CURCULIONIDAE: ENTIMINAE: OTIORHYNCHITAE: MYORHININI.

Type species: *Myorhinusalbolineatus* Guérin-Méneville, 1833, by subsequent monotypy (Guérin-Méneville 1833a: pl. 38).

Family-group name: MYORHINIDAE Marseul, 1863. Stem: *Myorhin*-. Note: senior homonym of MYORHININI Baranov, 1942 (type genus *Myorhina* Robineau-Desvoidy, 1830) in Diptera.

***Myrabolia* Reitter, 1876b**: 55. Current status: valid genus in MYRABOLIIDAE.

Type species: *Myraboliaharoldiana* Reitter, 1876 (= *Silvanusbrevicornis* Erichson, 1842), by monotypy.

Family-group name: MYRABOLIINAE Lawrence & Britton, 1991. Stem: *Myraboli*-.

***Myrmacicelus* Chevrolat, 1833**: 358. Current status: junior synonym of *Rhynolaccus* Guérin- Méneville, 1831 in BRENTIDAE: APIONINAE: MYRMACICELITAE: MYRMACICELINI.

Type species: *Myrmacicelusbistriatus* Chevrolat, 1833 (= *Rhynolaccusformicarius* Guérin- Méneville, 1831), by monotypy. Note: the type species was first listed as *Myrmacicelusformicarius* Guérin-Méneville but it was changed in the errata and addenda [p. xcix] to *Myrmacicelusbistriatus*.

Family-group name: MYRMACICELINAE Zimmerman, 1994. Stem: *Myrmacicel*-.

***Myrmechixenus* Chevrolat, 1835b**: 267. Current status: valid genus in TENEBRIONIDAE: DIAPERINAE: MYRMECHIXENINI.

Type species: *Myrmechixenussubterraneus* Chevrolat, 1835, by monotypy.

Family-group name: MYRMÉCHIXÉNITES Jacquelin du Val, 1858. Stem: *Myrmechixen*-.

***Myrmedonia* Erichson, 1837**: 286. Current status: junior synonym of *Drusilla* Leach, 1819 in STAPHYLINIDAE: ALEOCHARINAE: LOMECHUSINI: MYRMEDONIINA.

Type species: *Staphylinuscanaliculatus* Fabricius, 1787, by subsequent designation (Duponchel 1841b: 57).

Family-group name: MYRMEDONIIDES C.G. Thomson, 1867. Stem: *Myrmedoni*-.

***Myrmetes* Marseul, 1862**: 499, 511. Current status: valid genus in HISTERIDAE: SAPRININAE: MYRMETINI.

Type species: *Histerpiceus* Paykull, 1809 (= *Myrmetespaykulli* Kanaar, 1979), by monotypy.

Family-group name: MYRMETINI Portevin, 1929. Stem: *Myrmet*-.

***Myrmex* Sturm, 1826**: 32, 172. Current status: valid genus in CURCULIONIDAE: CURCULIONINAE: OTIDOCEPHALINI.

Type species: *Curculiomyrmex* Herbst, 1797, by monotypy.

Family-group name: MYRMECINAE Tanner, 1966. Stem: *Myrmec*-.

***Myrtonymus* Kuschel, 1990**: 80. Current status: valid genus in CURCULIONIDAE: BRACHYCERINAE: MYRTONYMINI.

Type species: *Myrtonymuszelandicus* Kuschel, 1990, by original designation.

Family-group name: MYRTONYMINA Kuschel, 1990. Stem: *Myrtonym*-.

***Mysteria* J. Thomson, 1861**: 278. Current status: valid genus in VESPERIDAE: ANOPLODERMATINAE: MYSTERIINI.

Type species: *Mysteriacylindripennis* J. Thomson, 1861, by monotypy.

Family-group name: MYSTERINAE Prosen, 1960. Stem: *Mysteri*-.

†***Mysteriomorphus* Alekseev & Ellenberger, 2019**: 485. Current status: valid genus in †MYSTERIOMORPHIDAE. Note: Cretaceous (Myanmar).

Type species: †*Mysteriomorphuspelevini* Alekseev & Ellenberger, 2019, by original designation.

Family-group name: †MYSTERIOMORPHIDAE Alekseev & Ellenberger, 2019: 484. Stem: *Mysteriomorph*-.

***Mystropomus* Chaudoir, 1848**: 107. Current status: valid genus in CARABIDAE: PAUSSINAE: MYSTROPOMINI.

Type species: *Mystropomussubcostatus* Chaudoir, 1848, by monotypy.

Family-group name: MYSTROPOMINI G.H. Horn, 1881. Stem: *Mystropom*-.

***Mystrops* Erichson, 1843a**: 234. Current status: valid genus in NITIDULIDAE: NITIDULINAE: MYSTROPINI.

Type species: *Mystropsdebilis* Erichson, 1843, by subsequent designation (Kirejtshuk and Couturier 2011: 372).

Family-group name: MYSTROPIDAE Murray, 1864. Stem: *Mystrop*-.

***Mythodes* J. Thomson, 1864**: 139. Current status: valid genus in CERAMBYCIDAE: CERAMBYCINAE: MYTHODINI.

Type species: *Mythodesplumosa* J. Thomson, 1864, by original designation.

Family-group name: MYTHODIDES Lacordaire, 1868. Stem: *Mythod*-.

***Myzia* Mulsant, 1846**: 129. Current status: valid genus in COCCINELLIDAE: COCCINELLINAE: COCCINELLINI. Note: as pointed out by Bouchard and Bousquet (2020a: 95–96) two original spellings were used, *Mysia* (p. 129) and *Myzia* (p. 277); subsequently, Mulsant (1850: 137) used the spelling *Mysia* and therefore acted as First Reviser (Article 24.2.4). *Mysia* is therefore the correct original spelling but this name is a junior homonym of *Mysia* Lamarck, 1818 [Mollusca]; *Myzia*, an incorrect original spelling, has been used as valid in the literature and is in prevailing usage; an application to the Commission is necessary to retain *Myzia* Mulsant, 1846 as a valid name.

Type species: *Coccinellaoblongoguttata* Linnaeus, 1758, by monotypy.

Family-group name: MYSIINA Hatch, 1962: 175. Stem: *Myzi*-. Note: a family-group name based on this type genus was attributed to Mulsant, 1846 (as MYSIATES) and treated as unavailable by Bouchard et al. (2011: 376).

***Nacerdes* Dejean, 1834**: 228. Current status: valid genus in OEDEMERIDAE: OEDEMERINAE: NACERDINI.

Type species: *Necydalisnotata* Fabricius, 1792 (= *Cantharismelanura* Linnaeus, 1758), by subsequent designation (Arnett 1950: 222).

Family-group name: NACERDATES Mulsant, 1858. Stem: *Nacerd*-.

***Nama* Borovec & Meregalli, 2013**: 502. Current status: valid genus in CURCULIONIDAE: ENTIMINAE incertae sedis.

Type species: *Namarichtersveldiana* Borovec & Meregalli, 2013, by original designation.

Family-group name: NAMAINI Borovec & Meregalli in Meregalli et al., 2021: 102. Stem: *Nama*-.

***Nanoglossa* Fauvel, 1868**: 379. Current status: valid genus in STAPHYLINIDAE: ALEOCHARINAE: HOMALOTINI: BOLITOCHARINA. Note: new replacement name for *Microglossa* Fauvel, 1866.

Type species: *Microglossachilensis* Fauvel, 1866, by subsequent designation (Fenyes 1919: 23) (type fixation for *Microglossa* Fauvel).

Family-group name: NANOGLOSSAE Fenyes, 1919. Stem: *Nanogloss*-.

†***Nanophydes* Arnoldi, 1977**: 173. Current status: valid genus in CARIDAE: †BAISSORHYNCHINAE: †NANOPHYDINI. Note: Upper Jurassic (Kazakhstan).

Type species: †*Nanophydesovatus* Arnoldi, 1977, by original designation.

Family-group name: †NANOPHYDINAE Arnoldi, 1977. Stem: *Nanophyd*-. Note: the name †NANOPHYDINI, as currently classified, is older than the subfamily †BAISSORHYNCHINAE; however, more research is needed to clarify the correct position of †BAISSORHYNCHINAE as well as the tribe therein before the priority problem can be addressed.

***Nanophyes* Schönherr, 1838**: 780. Current status: valid genus in BRENTIDAE: NANOPHYINAE: NANOPHYINI. Note: new replacement name for *Nanodes* Schönherr, 1825, which was suppressed for the Principle of Priority and placed on the Official Index of Rejected and Invalid Names in Zoology while *Nanophyes* Schönherr, 1838 was placed on the Official List of Generic Names in Zoology (ICZN 1989a).

Type species: *Curculiolythri* Fabricius, 1787 (= *Curculiomarmoratus* Goeze, 1777), by original designation (see ICZN 1989a) (type fixation for *Nanodes* Schönherr). Note: *Curculiomarmoratus* Goeze, 1777, which has been published in a work that is not consistently binominal, was placed on the Official List of Specific Names in Zoology (ICZN 1989a).

Family-group name: NANOPHYEIDAE Gistel, 1848. Stem: *Nanophy*-.

***Nanosella* Motschulsky, 1869a**: 172, 187. Current status: valid genus in PTILIIDAE: PTILIINAE: NANOSELLINI. Note: two original spellings were used, including *Nonosella* (p. 187); Neave (1940: 265, 345) acted as First Reviser (ICZN 1999a, Article 24.2.3) and selected *Nanosella*, the spelling currently in prevailing usage.

Type species: *Nanosellafungi* Motschulsky, 1869 (= *Ptiliumfungi* J.L. LeConte, 1863), by monotypy.

Family-group name: NANOSELLINAE Barber, 1924. Stem: *Nanosell*-.

***Narthecius* J.L. LeConte, 1861**: 95. Current status: valid genus in LAEMOPHLOEIDAE.

Type species: *Nartheciusgrandiceps* J.L. LeConte, 1863, by subsequent monotypy (J.L. LeConte 1863b: 70).

Family-group name: NARTHECIINI Casey, 1890. Stem: *Nartheci*-.

***Nascio* Laporte & Gory, 1838**: 1. Current status: valid genus in BUPRESTIDAE: BUPRESTINAE: NASCIONINI. Note: name placed on the Official List of Generic Names in Zoology (ICZN 2002b).

Type species: *Buprestisvetusta* Boisduval, 1835, by monotypy (see ICZN 2002b). Note: name placed on the Official List of Specific Names in Zoology (ICZN 2002b).

Family-group name: NASCIONINA Holyński, 1988. Stem: *Nascion*-.

***Nastus* Schönherr, 1842b**: 405. Current status: valid genus in CURCULIONIDAE: ENTIMINAE: OTIORHYNCHITAE: NASTINI.

Type species: *Nastusgoryi* Boheman, 1842, by original designation.

Family-group name: NASTINI Reitter, 1913. Stem: *Nast*-.

***Nasutiphilus* Kistner, 1970d**: 500. Current status: valid genus in STAPHYLINIDAE: ALEOCHARINAE: ATHETINI: NASUTIPHILINA.

Type species: *Nasutiphilusniger* Kistner, 1970, by original designation.

Family-group name: NASUTIPHILINA Kistner, 1970. Stem: *Nasutiphil*-.

***Nasutitella* Pasteels, 1967**: 54. Current status: valid genus in STAPHYLINIDAE: ALEOCHARINAE: COROTOCINI: NASUTITELLINA.

Type species: *Nasutitellapuncticollis* Pasteels, 1967, by original designation.

Family-group name: NASUTITELLINA Jacobson, Kistner & Pasteels, 1986. Stem: *Nasutitell*-.

***Natalis* Laporte, 1838**: 40 Current status: valid genus in CLERIDAE: CLERINAE: NATALIINI: NATALIINA.

Type species: *Natalislaplacei* Laporte, 1838, by monotypy.

Family-group name: NATALIINI Bartlett, 2021: 750. Stem: *Natali*-. Note: the correct stem based on *Natalis* Laporte, a Latin adjective, is *Natal*- (M.A. Alonso-Zarazaga, pers. comm. to P.B., 2022); however, since using the correct stem would have led to the creation of a homonym with NATALIDAE Gray, 1866 (type genus *Natalus* Gray, 1838) in Chiroptera, we assume that Bartlett (2021: 750) used the stem *Natalii*- in order to avoid creating a homonymous family-group name (ICZN 1999a, Article 29.6).

***Nathrius* Brèthes, 1916**: 76. Current status: valid genus in CERAMBYCIDAE: CERAMBYCINAE: PSEBIINI.

Type species: *Nathriusporteri* Brèthes, 1916 (= *Leptideabrevipennis* Mulsant, 1839), by monotypy.

Family-group name: NATHRIINI Arnett, 1962. Stem: *Nathri*-.

***Natypleurus* Newton & Thayer, 1992**: 20. Current status: valid genus in STAPHYLINIDAE: PSELAPHINAE: GONIACERITAE: INIOCYPHINI: NATYPLEURINA. Note: new replacement name for *Tanypleurus* Raffray, 1890.

Type species: *Tanypleurusmalaianus* Raffray, 1890, by monotypy (type fixation for *Tanypleurus* Raffray).

Family-group name: NATYPLEURINA Newton & Thayer, 1992. Stem: *Natypleur*-.

***Naupactus* Dejean, 1821**: 94. Current status: valid genus in CURCULIONIDAE: ENTIMINAE: POLYDRUSITAE: NAUPACTINI: NAUPACTINA.

Type species: *Curculiorivulosus* G.-A. Olivier, 1790, by subsequent designation (Champion 1911: 232).

Family-group name: NAUPACTIDAE Gistel, 1848. Stem: *Naupact*-. Note: nomen protectum (see Bouchard et al. 2011: 611).

***Navomorpha* A. White, 1855**: 334. Current status: valid genus in CERAMBYCIDAE: CERAMBYCINAE: COPTOMMATINI.

Type species: *Callidiumlineatum* Fabricius, 1775, by subsequent designation (J. Thomson 1864: 38).

Family-group name: NAVOMORPHIDES Lacordaire, 1869. Stem: *Navomorph*-.

†***Nebrenthorrhinus* Legalov, 2003**: 89. Current status: valid genus in NEMONYCHIDAE: †PALEOCARTINAE: †PALEOCARTINI: †NEBRENTHORRHININA. Note: Lower Cretaceous (Spain).

Type species: †*Brenthorrhinoideslacasei* Gratshev & Zherikhin, 2000, by original designation.

Family-group name: †NEBRENTHORRHININA Legalov, 2007. Stem: *Nebrenthorrhin*-.

***Nebria* Latreille, 1802b**: 89. Current status: valid genus in CARABIDAE: NEBRIINAE: NEBRIINI.

Type species: *Carabusbrevicollis* Fabricius, 1792, by subsequent designation (Latreille 1810: 426).

Family-group name: NEBRIIDAE Laporte, 1834. Stem: *Nebri*-.

***Necrobia* Latreille, 1797**: 35. Current status: valid genus in CLERIDAE: KORYNETINAE. Note: this name is usually credited to “G.-A. Olivier, 1795” in the literature but it was first made available by Latreille in 1797 (see Bouchard and Bousquet 2020a: 93); sections 67 (part)–80 in G.-A. Olivier’s work where *Necrobia* appears (i.e., in section 76: 4) were not issued until 1800 (see Bousquet 2018: 140); name placed on the Official List of Generic Names in Zoology (ICZN 1961c, as *Necrobia* G.-A. Olivier, 1795).

Type species: *Dermestesviolaceus* Linnaeus, 1758, by subsequent designation (Latreille 1810: 356; see ICZN 1961c). Note: no species were originally included in this genus by Latreille (1797); the first ones were the three species listed by G.-A. Olivier (1800: [76bis] 5, 6); name placed on the Official List of Specific Names in Zoology (ICZN 1961c).

Family-group name: NECROBIAEIDAE Gistel, 1848. Stem: *Necrobi*-.

***Necrodes* Leach, 1815a**: 88. Current status: valid genus in STAPHYLINIDAE: SILPHINAE: SILPHINI.

Type species: *Silphalittoralis* Linnaeus, 1758, by subsequent designation (Curtis 1830: pl. 334).

Family-group name: NECRODISIDAE Gistel, 1848. Stem: *Necrod*-.

†***Necronectes* Ponomarenko, 1977**: 22. Current status: junior synonym of *Timarchopsis* Brauer, J. Redtenbacher & Ganglbauer, 1889 in †COPTOCLAVIDAE: †TIMARCHOPSINAE. Note: junior homonym of *Necronectes* A. Milne-Edwards, 1881 [Crustacea].

Type species: †*Necronectesaquaticus* Ponomarenko, 1977, by original designation.

Family-group name: †NECRONECTINAE Ponomarenko, 1977. Stem: *Necronect*-. Note: family- group name permanently invalid (ICZN 1999a, Article 39).

***Necrophilus* Latreille, 1829a**: 500. Current status: valid genus in AGYRTIDAE: NECROPHILINAE.

Type species: *Silphasubterranea* Dahl, 1807, by monotypy.

Family-group name: NECROPHILINAE Newton, 1997. Stem: *Necrophil*-.

***Necydalis* Linnaeus, 1758**: 342, 421. Current status: valid genus in CERAMBYCIDAE: LEPTURINAE: NECYDALINI.

Type species: *Necydalismajor* Linnaeus, 1758, by subsequent designation (Latreille 1829b: 120).

Family-group name: NECYDALIDES Latreille, 1825. Stem: *Necydal*-.

***Necydalopsis* Blanchard, 1851**: 473. Current status: valid genus in CERAMBYCIDAE: CERAMBYCINAE: NECYDALOPSINI.

Type species: *Necydalopsistrizonatus* Blanchard, 1851, by monotypy.

Family-group name: NÉCYDALOPSIDES Lacordaire, 1868. Stem: *Necydalops*-.

***Nedine* J. Thomson, 1864**: 27. Current status: valid genus in CERAMBYCIDAE: LAMIINAE: DESMIPHORINI.

Type species: *Nedinelongipes* J. Thomson, 1864, by original designation.

Family-group name: NÉDINIDES Lacordaire, 1872. Stem: *Nedin*-.

***Negastrius* C.G. Thomson, 1859**: 106. Current status: valid genus in ELATERIDAE: NEGASTRIINAE: NEGASTRIINI.

Type species: *Elaterpulchellus* Linnaeus, 1761, by original designation.

Family-group name: NEGASTRIINAE Nakane & Kishii, 1956. Stem: *Negastri*-.

***Neleides* Harold in Gemminger and Harold, 1868c**: 978. Current status: valid genus in PASSALIDAE: PASSALINAE: PASSALINI. Note: this name is attributed to Kaup (1869: 33) in the literature; however, it was first made available by Harold in 1868 when he included four nominal species under it.

Type species: *Passalusaffinis* Percheron, 1834, by subsequent designation (Brauer 1870: 127).

Family-group name: NELIDINAE Kuwert, 1896. Stem: *Neleid*-.

***Neleus* Harold in Gemminger and Harold, 1868c**: 977. Current status: junior synonym of *Passalus* Fabricius, 1792 in PASSALIDAE: PASSALINAE: PASSALINI. Note: junior homonym of *Neleus* Desbonne & Schramm, 1867 [Crustacea]; this name is attributed to Kaup (1869: 30) in the literature; however, it was first made available by Harold in 1868 when he included several nominal species under this genus.

Type species: *Lucanusinterruptus* Linnaeus, 1758, by subsequent designation (Hincks and Dibb 1935: 38).

Family-group name: NELEINAE Kaup, 1869. Stem: *Nele*-. Note: family-group name permanently invalid (ICZN 1999a, Article 39).

***Nemadus* C.G. Thomson, 1867**: 351. Current status: valid genus in LEIODIDAE: CHOLEVINAE: ANEMADINI: NEMADINA.

Type species: *Catopscolonoides* Kraatz, 1851, by monotypy.

Family-group name: NEMADINAE Jeannel, 1936. Stem: *Nemad*-.

***Nemaschema* J. Thomson, 1861**: 354. Current status: valid genus in CERAMBYCIDAE: LAMIINAE: ENICODINI.

Type species: *Navomorphasanguinicollis* Chevrolat, 1857, by monotypy.

Family-group name: NEMASCHEMITAE J. Thomson, 1864. Stem: *Nemaschemat*-.

***Nematidium* Erichson, 1845a**: 275. Current status: valid genus in ZOPHERIDAE: COLYDIINAE: NEMATIDIINI.

Type species: *Colydiumcylindricum* Fabricius, 1801, by monotypy.

Family-group name: NEMATIDII G.H. Horn, 1878. Stem: *Nematidi*-.

***Nematodes* Guérin-Méneville, 1827**: 498. Current status: valid genus in EUCNEMIDAE: MACRAULACINAE: NEMATODINI. Note: this genus is often credited to Berthold (1827: 335) (e.g., Muona 2007: 87) but it was first made available by Guérin-Méneville (1827: 498) (see Bouchard and Bousquet 2020a: 87).

Type species: *Elaterfilum* Fabricius, 1801, by original designation.

Family-group name: NEMATODINI Leiler, 1976. Stem: *Nematod*-.

***Nematoplus* J.L. LeConte, 1855**: 270, 275. Current status: valid genus in STENOTRACHELIDAE: NEMATOPLINAE.

Type species: *Nematopluscollaris* J.L. LeConte, 1855, by monotypy.

Family-group name: NEMATOPLI J.L. LeConte, 1862. Stem: *Nematopl*-.

***Nematoscelis* Wollaston, 1868**: 231. Current status: valid genus in STAPHYLINIDAE: ALEOCHARINAE: HYPOCYPHTINI.

Type species: *Nematoscelisfilipes* Wollaston, 1868, by monotypy.

Family-group name: NEMATOSCELINI Fenyes, 1921. Stem: *Nematoscelid*-.

***Nemognatha* Illiger, 1807b**: 333. Current status: valid genus in MELOIDAE: NEMOGNATHINAE: NEMOGNATHINI: NEMOGNATHINA. Note: name placed on the Official List of Generic Names in Zoology (ICZN 1999c).

Type species: *Zonitisvittata* Fabricius, 1801 (= *Zonitispiazata* Fabricius, 1798), by monotypy (see ICZN 1999c). Note: *Zonitisvittata* Fabricius, 1801 placed on the Official List of Specific Names in Zoology (ICZN 1999c).

Family-group name: NEMOGNATHITES Laporte, 1840. Stem: *Nemognath*-. Note: family-group name given precedence over HORIIDAE Latreille, 1802 (ICZN 1999c).

***Nemonyx* L. Redtenbacher, 1845**: 96, 152. Current status: valid genus in NEMONYCHIDAE: NEMONYCHINAE. Note: name placed on the Official List of Generic Names in Zoology (ICZN 2005c).

Type species: *Rhinomacerlepturoides* Fabricius, 1801, by monotypy (see ICZN 2005c). Note: name placed on the Official List of Specific Names in Zoology (ICZN 2005c).

Family-group name: NEMONYCHIDAE Bedel, 1882. Stem: *Nemonych*-.

***Nemotarsus* J.L. LeConte, 1853e**: 377. Current status: valid genus in CARABIDAE: HARPALINAE: LEBIINI: NEMOTARSINA.

Type species: *Nemotarsuselegans* J.L. LeConte, 1853, by monotypy.

Family-group name: NEMOTARSINAE H.W. Bates, 1883. Stem: *Nemotars*-.

***Nemotragus* Westwood, 1843b**: 57. Current status: valid genus in CERAMBYCIDAE: LAMIINAE: AGAPANTHIINI.

Type species: *Nemotragushelvolus* Westwood, 1843, by monotypy.

Family-group name: NEMOTRAGITAE J. Thomson, 1864. Stem: *Nemotrag*-.

***Nemozoma* Latreille, 1804a**: 157. Current status: valid genus in TROGOSSITIDAE: TROGOSSITINAE: TROGOSSITINI.

Type species: *Dermesteselongatus* Linnaeus, 1761, by subsequent monotypy (Latreille 1804c: 239).

Family-group name: NEMOSOMIDA Leach, 1815. Stem: *Nemozomat*-.

***Neoceratopsis* Jeannel, 1956**: 50. Current status: valid genus in STAPHYLINIDAE: PSELAPHINAE: CLAVIGERITAE: CLAVIGERINI.

Type species: *Neoceratopsispauliani* Jeannel, 1956, by original designation.

Family-group name: NEOCERATOPSINI Célis, 1970. Stem: *Neoceratopse*-.

***Neocerus* Wasmann, 1893a**: 105, 111. Current status: valid genus in STAPHYLINIDAE: PSELAPHINAE: CLAVIGERITAE: CLAVIGERINI.

Type species: *Neoceruscompressicornis* Wasmann, 1893, by monotypy.

Family-group name: NEOCERINI Jeannel, 1954. Stem: *Neocer*-. Note: junior homonym of NEOCERINI Salmon, 1941 (type genus *Neocerus* Salmon, 1941) in Collembola: TOMOCERIDAE; Salmon’s name is permanently invalid (based on a preoccupied type genus).

***Neocharis* Sharp, 1877c**: 485. Current status: valid genus in EUCNEMIDAE: MELASINAE: NEOCHARINI.

Type species: *Neocharisvaria* Sharp, 1877, by subsequent designation (Fleutiaux 1921: 294).

Family-group name: NEOCHARINI Muona, 1993. Stem: *Neochar*-.

***Neochlamys* Jacoby, 1882**: 51. Current status: valid genus in CHRYSOMELIDAE: LAMPROSOMATINAE: NEOCHLAMYSINI.

Type species: *Neochlamysstrigicollis* Jacoby, 1882, by original designation.

Family-group name: NEOCHLAMYSINI Monrós, 1959. Stem: *Neochlamys*-.

***Neochthebius* Orchymont, 1932**: 43. Current status: junior synonym of *Ochthebius* Leach, 1815 in HYDRAENIDAE: OCHTHEBIINAE: OCHTHEBIINI.

Type species: *Ochthebiusvandykei* Knisch, 1924, by monotypy.

Family-group name: NEOCHTHEBINA Perkins, 1997. Stem: *Neochthebi*-.

***Neoclytus* J. Thomson, 1860b**: 67. Current status: valid genus in CERAMBYCIDAE: CERAMBYCINAE: CLYTINI. Note: new replacement name for *Rhopalomerus* Chevrolat, 1860; as pointed out by Bousquet et al. (2017: 26) the genus-group name *Rhopalopachys*Chevrolat (1860b: 457; type species *Clytusmorosus* Chevrolat, 1860 (= *Clytusirroratus* LeConte, 1858) by original designation) is older than *Neoclytus* J. Thomson, 1860 and should be used as valid; an application to the Commission is necessary to retain *Neoclytus* J. Thomson, 1860 as the valid name.

Type species: *Callidiumerythrocephalum* Fabricius, 1787 (= *Callidiumacuminatum* Fabricius, 1775), by original designation.

Family-group name: NEOCLYTITAE J. Thomson, 1861. Stem: *Neoclyt*-.

***Neocorus* J. Thomson, 1864**: 220. Current status: valid genus in CERAMBYCIDAE: CERAMBYCINAE: NEOCORINI.

Type species: *Stenygraibidionoides* Audinet-Serville, 1834, by original designation.

Family-group name: NEOCORINI Martins, 2005. Stem: *Neocor*-.

***Neocuris* Saunders, 1868**: 19. Current status: valid genus in BUPRESTIDAE: BUPRESTINAE: CURIDINI: NEOCURIDINA.

Type species: *Stigmoderaguerinii* Hope, 1843, by subsequent designation (Rye 1873: 271). Note: the type species usually recognized for this genus is *Anthaxiafortnumi* Hope, 1846, by subsequent designation of Bellamy (1997c: 221) but Rye’s designation is earlier; both species are currently included in the genus *Neocuris*.

Family-group name: NEOCURIDINA Holyński, 1988. Stem: *Neocurid*-.

***Neocyphus* Bedel, 1883**: 23. Current status: junior synonym of *Cyrtomon* Schönherr, 1823 in CURCULIONIDAE: ENTIMINAE: POLYDRUSITAE: NAUPACTINI: NAUPACTINA. Note unnecessary new replacement name for *Cyphus* Germar, 1823.

Type species: *Curculiogibber* Pallas, 1781, by subsequent designation (Schönherr 1826: 11) (type fixation for *Cyphus* Germar).

Family-group name: NEOCYPHINI Hustache, 1919. Stem: *Neocyph*-.

***Neohydrocoptus* Satô, 1972**: 144. Current status: valid genus in NOTERIDAE: NOTERINAE: NOTERINI.

Type species: *Hydrocoptusbivittis* Motschulsky, 1859, by original designation.

Family-group name: NEOHYDROCOPTINI Zalat, Saleh, Angus & Kaschef, 2000. Stem: *Neohydrocopt*-.

***Neoibidion* Monné, 2012**: 36. Current status: valid genus in CERAMBYCIDAE: CERAMBYCINAE: TROPIDINI: NEOIBIDIONINA. Note: new replacement name for *Ibidion* Audinet-Serville, 1834.

Type species: *Ibidioncomatum* Audinet-Serville, 1834, by subsequent designation (J. Thomson 1864: 215) (type fixation for *Ibidion* Audinet-Serville).

Family-group name: NEOIBIDIONINI Monné, 2012: 35. Stem: *Neoibidion*-.

***Neopachypterus* Bouchard, Löbl & Merkl, 2007**: 386. Current status: valid genus in TENEBRIONIDAE: BLAPTINAE: OPATRINI: NEOPACHYPTERINA. Note: new replacement name for *Pachypterus* P.H. Lucas, 1847.

Type species: *Pachypterusmauritanicus* P.H. Lucas, 1847, by monotypy (type fixation for *Pachypterus* P.H. Lucas).

Family-group name: NEOPACHYPTERINA Bouchard, Löbl & Merkl, 2007. Stem: *Neopachypter*-.

***Neopedilus* M. Abdullah, 1969**: 358. Current status: junior synonym of *Pedilus* Fischer, 1820 in PYROCHROIDAE: PEDILINAE.

Type species: *Pedilusparvicollis* Fall, 1919, by original designation.

Family-group name: NEOPEDILINI M. Abdullah, 1969. Stem: *Neopedil*-.

***Neopelatops* Jeannel, 1936**: 12. Current status: valid genus in LEIODIDAE: CAMIARINAE: NEOPELATOPINI.

Type species: *Neopelatopsedwardsi* Jeannel, 1936, by monotypy.

Family-group name: NEOPELATOPINI Jeannel, 1962. Stem: *Neopelatop*-.

***Neophonus* Fauvel, 1905**: 99. Current status: valid genus in STAPHYLINIDAE: NEOPHONINAE.

Type species: *Neophonusbruchi* Fauvel, 1905, by monotypy.

Family-group name: NEOPHONI Fauvel, 1905. Stem: *Neophon*-.

***Neopsectropus* Kaszab, 1941**: 30. Current status: valid genus in TENEBRIONIDAE: TENEBRIONINAE: ULOMINI.

Type species: *Neopsectropusgebieni* Kaszab, 1941, by original designation.

Family-group name: NEOPSECTROPINAE Kaszab, 1941. Stem: *Neopsectropod*-.

***Neorthopleura* Barr, 1976**: 2. Current status: valid genus in CLERIDAE: KORYNETINAE.

Type species: *Orthopleuratexana* Bland, 1863, by original designation.

Family-group name: NEORTHOPLEURINAE Opitz, 2009. Stem: *Neorthopleur*-.

***Neosharpia* Hoffmann, 1956**: 247. Current status: valid genus in CURCULIONIDAE: BARIDINAE: MADARINI: NEOSHARPIINA.

Type species: *Neosharpiaartemisiae* Hoffmann, 1956, by original designation.

Family-group name: NEOSHARPINI Hoffmann, 1956. Stem: *Neosharpi*-.

***Neostenus* Pascoe, 1857**: 91. Current status: valid genus in CERAMBYCIDAE: CERAMBYCINAE: URACANTHINI.

Type species: *Neostenussaundersii* Pascoe, 1857, by monotypy.

Family-group name: NÉOSTÉNIDES Lacordaire, 1868. Stem: *Neosten*-.

***Neotropospeonella* Pace, 1985**: 542. Current status: junior synonym of *Oryotus* L. Miller, 1856 in LEIODIDAE: CHOLEVINAE: LEPTODIRINI: BATHYSCIINA.

Type species: *Neotropospeonelladecui* Pace, 1985 (= *Oryotusravasinii* J. Müller, 1922), by original designation.

Family-group name: NEOTROPOSPEONELLINA Perkovsky, 1997. Stem: *Neotropospeonell*-.

***Neotyphlus* Coiffait, 1959**: 387. Current status: valid genus in STAPHYLINIDAE: LEPTOTYPHLINAE: NEOTYPHLINI.

Type species: *Neotyphluscalifornicus* Coiffait, 1959, by original designation.

Family-group name: NEOTYPHLINI Coiffait, 1963. Stem: *Neotyphl*-.

***Nephanes* C.G. Thomson, 1859**: 62. Current status: valid genus in PTILIIDAE: PTILIINAE: ACROTRICHINI. Note: name placed on the Official List of Generic Names in Zoology (ICZN 1985b).

Type species: *Trichopteryxabbreviatella* Heer, 1841 (= *Trichopteryxtitan* Newman, 1834), by original designation (see ICZN 1985b). Note: *Trichopteryxtitan* Newman, 1834 was placed on the Official List of Specific Names in Zoology (ICZN 1985b).

Family-group name: NEPHANINI Portevin, 1929. Stem: *Nephan*-.

***Nephodes* Blanchard, 1845a**: 34. Current status: invalid senior synonym of *Nephodinus* Gebien, 1943 in TENEBRIONIDAE: TENEBRIONINAE: HELOPINI: HELOPINA. Note: junior homonym of *Nephodes* Schönherr, 1840 [Coleoptera: CURCULIONIDAE].

Type species: *Nephodesvilliger* Rosenhauer, 1856, by subsequent monotypy (Rosenhauer 1856: 218).

Family-group name: NEPHODINI Reitter, 1917. Stem: *Nephod*-. Note: family-group name permanently invalid (ICZN 1999a, Article 39).

***Nephrites* Shuckard, 1838**: 512. Current status: valid genus in RIPIPHORIDAE: HEMIRHIPIDIINAE.

Type species: *Nephritesnitidus* Shuckard, 1838, by monotypy.

Family-group name: NEPHRITINAE Selander, 1957. Stem: *Nephrit*-.

***Nerissus* Chapuis, 1874**: 286. Current status: valid genus in CHRYSOMELIDAE: EUMOLPINAE: BROMIINI.

Type species: *Nerissusstrigosus* Chapuis, 1874, by monotypy.

Family-group name: NERISSINI Kuntzen, 1912. Stem: *Neriss*-.

***Nerthops* Schönherr, 1826**: 60. Current status: valid genus in CURCULIONIDAE: CURCULIONINAE: NERTHOPINI.

Type species: *Nerthopsmultiguttatus* Schönherr, 1826 (= *Bruchuspunctatus* Fabricius, 1798), by original designation.

Family-group name: NERTHOPIDES Lacordaire, 1865. Stem: *Nerthop*-.

***Nertinus* G.A.K. Marshall, 1943**: 118. Current status: valid genus in CURCULIONIDAE: BARIDINAE: APOSTASIMERINI: NERTININA. Note: new replacement name for *Nertus* Schönherr, 1844.

Type species: *Nertusmannerheimi* Boheman, 1844, by original designation (type fixation for *Nertus* Schönherr).

Family-group name: NERTININA Voss, 1954. Stem: *Nertin*-.

***Nertus* Schönherr, 1844**: 76. Current status: invalid senior synonym of *Nertinus* G.A.K. Marshall, 1943 in CURCULIONIDAE: BARIDINAE: APOSTASIMERINI: NERTININA. Note: junior homonym of *Nertus* Boie, 1828 [Aves].

Type species: *Nertusmannerheimi* Boheman, 1844, by original designation.

Family-group name: NERTINI Bondar, 1949. Stem: *Nert*-. Note: family-group name permanently invalid (ICZN 1999a, Article 39).

***Nesiobius* G.A.K. Marshall, 1943**: 118. Current status: valid genus in CURCULIONIDAE: COSSONINAE: NESIOBIINI. Note: new replacement name for *Nesiotes* Wollaston, 1861.

Type species: *Nesiotessquamosus* Wollaston, 1861, by monotypy (type fixation for *Nesiotes* Wollaston).

Family-group name: NESIOBIINI Alonso-Zarazaga & Lyal, 1999. Stem: *Nesiobi*-.

***Nesiotes* Wollaston, 1861d**: 211. Current status: invalid senior synonym of *Nesiobius* G.A.K. Marshall, 1943 in CURCULIONIDAE: COSSONINAE: NESIOBIINI. Note: junior homonym of *Nesiotes* Martens, 1860 [Mollusca].

Type species: *Nesiotessquamosus* Wollaston, 1861, by monotypy.

Family-group name: NESIOTINAE Voss, 1972. Stem: *Nesiot*-. Note: family-group name permanently invalid (ICZN 1999a, Article 39).

***Nesoneus* Bernhauer, 1939**: 205. Current status: valid genus in STAPHYLINIDAE: PROTEININAE: NESONEINI.

Type species: *Nesoneusacuticeps* Bernhauer, 1939, by monotypy.

Family-group name: NESONEINI Steel, 1966. Stem: *Nesone*-.

***Nesotrinchus* Obenberger, 1924**: 12. Current status: valid genus in BUPRESTIDAE: CHRYSOCHROINAE: POECILONOTINI: NESOTRINCHINA.

Type species: *Nesotrinchussimondsi* Obenberger, 1924 (= *Haplotrinchusaustralicus* Kerremans, 1903), by original designation.

Family-group name: NESOTRINCHINA Bílý, Kubáň & Volkovitsh, 2009. Stem: *Nesotrinch*-.

***Nessiara* Pascoe, 1860b**: 60. Current status: valid genus in ANTHRIBIDAE: ANTHRIBINAE: ZYGAENODINI. Note: new replacement name for *Nessia* Pascoe, 1859.

Type species: *Nessiadidyma* Pascoe, 1859, by subsequent designation (Jordan 1894b: 630).

Family-group name: NESSIARINI Morimoto, 1972. Stem: *Nessiar*-.

***Nettarhinus* Schönherr, 1826**: 269. Current status: valid genus in CURCULIONIDAE: MOLYTINAE: NETTARHININI.

Type species: *Nettarhinusanthribiformis* Schönherr, 1826, by original designation.

Family-group name: NETTARHINIDES Lacordaire, 1865. Stem: *Nettarhin*-.

***Netuschilia* Reitter, 1904**: 34, 35. Current status: valid genus in TENEBRIONIDAE: PIMELIINAE: LACHNOGYINI: NETUSCHILIINA.

Type species: *Lachnopushauseri* Reitter, 1897, by monotypy.

Family-group name: NETUSCHILIINA Ferrer & Yvinec, 2004. Stem: *Netuschili*-.

***Neuglenes* C.G. Thomson, 1859**: 63. Current status: junior synonym of *Ptinella* Motschulsky, 1844 in PTILIIDAE: PTILIINAE: PTINELLINI.

Type species: *Ptiliumapterum* Guérin-Méneville, 1839, by original designation.

Family-group name: NEUGLENINI Reitter, 1891. Stem: *Neuglen*-.

***Neumatora* Normand, 1920**: 24. Current status: valid genus in CURCULIONIDAE: COSSONINAE: NEUMATORINI.

Type species: *Neumatorascillae* Normand, 1920, by subsequent designation (Hlaváč and Maughan 2013: 59).

Family-group name: NEUMATORINI Folwaczny, 1973. Stem: *Neumator*-.

***Neuraphes* C.G. Thomson, 1859**: 61. Current status: valid genus in STAPHYLINIDAE: SCYDMAENINAE: SCYDMAENITAE: STENICHNINI. Note: the original spelling was *Nevraphes* but *Neuraphes*, an incorrect subsequent spelling, is in prevailing usage, attributed to the publication of the original spelling, and therefore deemed to be the correct original spelling (ICZN 1999a, Article 33.3.1).

Type species: *Scydmaenusangulatus* P.W.J. Müller & Kunze, 1822, by original designation.

Family-group name: NEURAPHINI Csiki, 1909. Stem: *Neuraph*-.

***Nicagus* J.L. LeConte, 1861**: 130. Current status: valid genus in LUCANIDAE: AESALINAE: NICAGINI.

Type species: *Ochodaeusobscurus* J.L. LeConte, 1847, by monotypy.

Family-group name: NICAGINI J.L. LeConte, 1861. Stem: *Nicag*-.

***Nicrophorus* Fabricius, 1775**: 71. Current status: valid genus in STAPHYLINIDAE: SILPHINAE: NICROPHORINI.

Type species: *Silphavespillo* Linnaeus, 1758, by subsequent designation (Latreille 1810: 427).

Family-group name: NECROPHORIDAE W. Kirby, 1837. Stem: *Nicrophor*-.

***Nigidius* W.S. MacLeay, 1819**: 96, 108. Current status: valid genus in LUCANIDAE: LUCANINAE: FIGULINI.

Type species: *Nigidiuscornutus* W.S. MacLeay, 1819, by monotypy.

Family-group name: NIGIDIINI Jakobson, 1911. Stem: *Nigidi*-.

***Nilio* Latreille, 1802b**: 179. Current status: valid genus in TENEBRIONIDAE: NILIONINAE. Note: the original spelling of the genus is *Nilion* but *Nilio*, an incorrect subsequent spelling, is in prevailing usage, attributed to the publication of the original spelling, and therefore deemed to be the correct original spelling (ICZN 1999a, Article 33.3.1).

Type species: *Coccinellavillosa* Fabricius, 1787, by monotypy.

Family-group name: NILIONIDEN Oken, 1843. Stem: *Nilion*-.

***Niphades* Pascoe, 1871b**: 174. Current status: valid genus in CURCULIONIDAE: MOLYTINAE: AMINYOPINI.

Type species: *Niphadespardalotus* Pascoe, 1871, by subsequent designation (Voss 1958: 47).

Family-group name: NIPHADINI Voss, 1963. Stem: *Niphad*-.

***Niphadonothus* Voss, 1965**: 340. Current status: valid genus in CURCULIONIDAE: MOLYTINAE: AMINYOPINI.

Type species: *Niphadonothusgentilis* Voss, 1965, by original designation.

Family-group name: NIPHADONOTHINA Voss, 1965. Stem: *Niphadonoth*-.

***Niphona* Mulsant, 1839**: 169. Current status: valid genus in CERAMBYCIDAE: LAMIINAE: PTEROPLIINI.

Type species: *Niphonapicticornis* Mulsant, 1839, by monotypy.

Family-group name: NIPHONINAE Pascoe, 1864. Stem: *Niphon*-.

***Niponius* Lewis, 1885**: 333. Current status: valid genus in HISTERIDAE: NIPONIINAE.

Type species: *Niponiusimpressicollis* Lewis, 1885, by subsequent designation (Gardner 1935: 4).

Family-group name: NIPONIIDAE Fowler, 1912. Stem: *Niponi*-.

***Nipponocyphon* Lawrence & Yoshitomi, 2007**: 509. Current status: valid genus in SCIRTIDAE: NIPPONOCYPHONINAE.

Type species: *Nipponocyphonnakanei* Lawrence & Yoshitomi, 2007, by original designation.

Family-group name: NIPPONOCYPHONINAE Lawrence & Yoshitomi, 2007. Stem: *Nipponocyphon*-.

***Nitidula* Fabricius, 1775**: 77. Current status: valid genus in NITIDULIDAE: NITIDULINAE: NITIDULINI.

Type species: *Silphabipustulata* Linnaeus, 1761 (= *Silphabipunctata* Linnaeus, 1758), by subsequent designation (Latreille 1810: 427).

Family-group name: NITIDULARIAE Latreille, 1802. Stem: *Nitidul*-.

***Noda* Chevrolat in Dejean, 1836a**: 410. Current status: invalid senior synonym of *Brachypnoea* Gistel, 1848 in CHRYSOMELIDAE: EUMOLPINAE: TYPOPHORINI. Note: junior homonym of *Noda* Schellenberg, 1803 [Diptera].

Type species: *Colaspistristis* G.-A. Olivier, 1808, by subsequent designation (Monrós and Bechyné 1956: 1124).

Family-group name: NODINI Selman, 1965. Stem: *Nod*-. Note: family-group name permanently invalid (ICZN 1999a, Article 39).

***Nodina* Motschulsky, 1858a**: 108. Current status: valid genus in CHRYSOMELIDAE: EUMOLPINAE: TYPOPHORINI.

Type species: *Nodinapusilla* Motschulsky, 1858, by subsequent designation (R. Lucas 1920: 441).

Family-group name: NODININI S.H. Chen, 1940. Stem: *Nodin*-.

***Nodostoma* Motschulsky, 1860b**: 176. Current status: junior synonym of *Basilepta* Baly, 1860 in CHRYSOMELIDAE: EUMOLPINAE: TYPOPHORINI.

Type species: *Nodostomafulvipes* Motschulsky, 1860, by subsequent designation (Jacoby 1908: 301).

Family-group name: NODOSTOMITES Chapuis, 1874. Stem: *Nodostomat*-.

***Nodotelus* C. Koch, 1950a**: 67. Current status: junior synonym of *Eutelonotus* Alluaud, 1902 in TENEBRIONIDAE: STENOCHIINAE: CNODALONINI. Note: new replacement name for *Eutelus* Solier, 1843.

Type species: *Eutelusrequieni* Solier, 1843, by subsequent designation (R. Lucas 1920: 291) (type fixation for *Eutelus* Solier).

Family-group name: NODOTELINI C. Koch, 1950. Stem: *Nodotel*-.

***Nomius* Laporte, 1834c**: 144. Current status: valid genus in CARABIDAE: PSYDRINAE: PSYDRINI. Note: *Moriopygmaeus* Dejean, 1831 was placed on the Official List of Generic Names in Zoology (ICZN 2011a).

Type species: *Nomiusgraecus* Laporte, 1834 (= *Moriopygmaeus* Dejean, 1831), by monotypy (see ICZN 2011a). Note: name placed on the Official List of Specific Names in Zoology (ICZN 2011a).

Family-group name: NOMIIDAE Gozis, 1875. Stem: *Nomius*- (ICZN 2011a).

***Nordenskioldia* J.R. Sahlberg, 1880**: 96. Current status: valid genus in STAPHYLINIDAE: EUAESTHETINAE: NORDENSKIOLDIINI.

Type species: *Nordenskioldiaglacialis* J.R. Sahlberg, 1880, by monotypy.

Family-group name: NORDENSKIOELDIINI Bernhauer & Schubert, 1911. Stem: *Nordenskioldi*-.

***Noseolucanus* Araya & Tanaka, 1998**: 334. Current status: valid genus in LUCANIDAE: LUCANINAE: NOSEOLUCANINI.

Type species: *Noseolucanusrugosus* Araya & Tanaka, 1998 (= *Pseudolucanusdenticulus* Boucher, 1996), by original designation.

Family-group name: NOSEOLUCANINI H. Huang & C.H. Chen, 2013: 67. Stem: *Noseolucan*-.

***Nosodendron* Latreille, 1804a**: 146. Current status: valid genus in NOSODENDRIDAE.

Type species: *Byrrhusfascicularis* G.-A. Olivier, 1790, by monotypy.

Family-group name: NOSODENDRINI Erichson, 1846. Stem: *Nosodendr*-.

***Nossidium* Erichson, 1845a**: 17. Current status: valid genus in PTILIIDAE: NOSSIDIINAE.

Type species: *Dermestespilosellus* Marsham, 1802, by subsequent designation (E. Desmarest 1852: 199).

Family-group name: NOSSIDIINAE Sörensson & Delgado, 2019: 791. Stem: *Nossidi*-.

***Notapion* Zimmerman, 1994**: 330. Current status: valid genus in BRENTIDAE: APIONINAE: RHADINOCYBITAE: NOTAPIONINI.

Type species: *Apiontrilobicolle* Lea, 1926, by original designation.

Family-group name: NOTAPIONINI Zimmerman, 1994. Stem: *Notapion*-.

***Noterapion* Kissinger, 2002**: 315. Current status: valid genus in BRENTIDAE: APIONINAE: NOTERAPIONITAE.

Type species: *Apionmeorrhynchum* R.A. Philippi & F. Philippi, 1864, by original designation.

Family-group name: NOTERAPIONINI Kissinger, 2004. Stem: *Noterapion*- (ICZN 1999a, Article 29.4).

***Noterus* Clairville, 1806**: 222. Current status: valid genus in NOTERIDAE: NOTERINAE: NOTERINI.

Type species: *Dytiscuscrassicornis* O.F. Müller, 1776, by monotypy.

Family-group name: NOTERIDES C.G. Thomson, 1860. Stem: *Noter*-.

***Nothobroscus* Roig-Juñent & Ball, 1995**: 303, 306. Current status: valid genus in CARABIDAE: BROSCINAE: BROSCINI: NOTHOBROSCINA.

Type species: *Nothobroscuschilensis* Roig-Juñent & Ball, 1995, by original designation.

Family-group name: NOTHOBROSCINA Roig-Juñent, 2000. Stem: *Nothobrosc*-.

***Nothochodaeus* Nikolajev, 2005b**: 219. Current status: valid genus in OCHODAEIDAE: OCHODAEINAE: NOTHOCHODAEINI. Note: two original spellings were used, including *Notochodaeus* (p. 219); Masumoto et al. (2013: 107) acted as First Revisers (ICZN 1999a, Article 24.2.3) and selected *Nothochodaeus*.

Type species: *Ochodaeusmaculatus* C.O. Waterhouse, 1875, by original designation.

Family-group name: NOTHOCHODAEINI Nikolajev, 2015a: 25. Stem: *Nothochodae*-.

***Nothognathus* G.A.K. Marshall, 1916**: 250. Current status: valid genus in CURCULIONIDAE: ENTIMINAE: OTIORHYNCHITAE: NOTHOGNATHINI.

Type species: *Nothognathusschoutedeni* G.A.K. Marshall, 1916, by original designation.

Family-group name: NOTHOGNATHIDES G.A.K. Marshall, 1916. Stem: *Nothognath*-.

***Nothomorpha* Saunders, 1871**: 78. Current status: valid genus in BUPRESTIDAE: POLYCESTINAE: ACMAEODERINI: NOTHOMORPHINA.

Type species: *Amorphosomaverrucosum* Gory & Laporte, 1839, by subsequent designation (Rye 1873: 271).

Family-group name: NOTOMORPHINI Cobos, 1955. Stem: *Nothomorph*-.

***Nothorhina* L. Redtenbacher, 1845**: 109, 153. Current status: valid genus in CERAMBYCIDAE: SPONDYLIDINAE: NOTHORHININI.

Type species: *Callidiummuricatum* Dalman, 1817, by subsequent designation (J. Thomson 1864: 267).

Family-group name: NOTHORHININI Zagajkevich, 1991. Stem: *Nothorhin*-.

†***Nothotytthonyx* Y.-D. Li, Biffi, Kundrata & Cai, 2022**: 264. Current status: valid genus in CANTHARIDAE: MALTHININAE: †NOTHOTYTTHONYCHINI. Note: mid-Cretaceous (Myanmar).

Type species: †*Nothotytthonyxserratus* Y.-D. Li, Biffi, Kundrata & Cai, 2022, by original designation.

Family-group name: †NOTHOTYTTHONYCHINI Fanti, 2022: 321. Stem: *Nothotytthonych*-.

***Nothus* G.-A. Olivier, 1812**: 383, 384. Current status: junior synonym of *Osphya* Illiger, 1807 in MELANDRYIDAE: OSPHYINAE.

Type species: *Nothusclavipes* G.-A. Olivier, 1812 (= *Cantharisbipunctata* Fabricius, 1775), by subsequent designation (Westwood 1838c: 31). Note: the type species currently recognized for this genus is *Nothuspraeustus* G.-A. Olivier, 1812 (e.g., Nikitsky 2020: 58), which is also a junior synonym of *Cantharisbipunctata* Fabricius, 1775, therefore there is no change in generic concept with the change of type species.

Family-group name: NOTHIDAE Shuckard, 1839. Stem: *Noth*-.

***Notiokasis* Kavanaugh & Nègre, 1983**: 551. Current status: valid genus in CARABIDAE: NEBRIINAE: NOTIOKASIINI.

Type species: *Notiokasischaudoiri* Kavanaugh & Nègre, 1983, by original designation.

Family-group name: NOTIOKASIINI Kavanaugh & Nègre, 1983. Stem: *Notiokasi*-.

***Notiomimetes* Wollaston, 1873b**: 440, 504. Current status: valid genus in CURCULIONIDAE: CYCLOMINAE: NOTIOMIMETINI.

Type species: *Notiomimetespascoei* Wollaston, 1873, by monotypy.

Family-group name: NOTIOMIMETIDES Wollaston, 1873. Stem: *Notiomimet*-.

***Notiophilus* Duméril, 1805**: 194. Current status: valid genus in CARABIDAE: NEBRIINAE: NOTIOPHILINI.

Type species: *Cicindelaaquatica* Linnaeus, 1758, by subsequent designation (Curtis 1829: pl. 254).

Family-group name: NOTIOPHILI Motschulsky, 1850. Stem: *Notiophil*-.

***Notiophygus* Gory, 1834**: 453. Current status: valid genus in DISCOLOMATIDAE: NOTIOPHYGINAE: NOTIOPHYGINI.

Type species: *Notiophygusnigropunctatus* Gory, 1834, by subsequent designation (John 1959: 20).

Family-group name: NOTIOPHYGIDAE Jakobson, 1915. Stem: *Notiophyg*-.

***Notioxenus* Wollaston, 1861d**: 212. Current status: invalid senior synonym of *Valenfriesia* Alonso-Zarazaga & Lyal, 1999 in ANTHRIBIDAE: CHORAGINAE: TYPOPHORINI. Note: junior homonym of *Notioxenus* Motschulsky, 1858 [Coleoptera: CARABIDAE].

Type species: *Notioxenusbewickii* Wollaston, 1861, by subsequent designation (Jordan 1907: 380).

Family-group name: NOTIOXÉNIDES Lacordaire, 1865. Stem: *Notioxen*-. Note: family-group name permanently invalid (ICZN 1999a, Article 39).

†***Notocupes* Ponomarenko, 1964**: 61. Current status: valid genus in ARCHOSTEMATA incertae sedis. Note: Triassic/Late Cretaceous (China; Kazakhstan; Mongolia; Russia).

Type species: †*Notocupespicturatus* Ponomarenko, 1964, by original designation.

Family-group name: †NOTOCUPEDINI Ponomarenko, 1966. Stem: *Notocuped*-.

***Notodermus* Desbrochers des Loges, 1875**: 19. Current status: junior synonym of *Procas* Stephens, 1831 in CURCULIONIDAE: BRACHYCERINAE: ERIRHININI.

Type species: *Notodermusbruleriei* Desbrochers des Loges, 1875, by monotypy.

Family-group name: NOTODERMINA Voss, 1952. Stem: *Notoderm*-.

***Notolomus* J.L. LeConte in J.L. LeConte and G.H. Horn, 1876**: 222. Current status: valid genus in CURCULIONIDAE: CURCULIONINAE: DERELOMINI: NOTOLOMINA.

Type species: *Notolomusbasalis* J.L. LeConte, 1876, by subsequent designation (Lepesme et al. 1947: 587).

Family-group name: NOTOLOMINA N.M. Franz, 2006. Stem: *Notolom*-.

***Notomicrus* Sharp, 1882b**: 260. Current status: valid genus in NOTERIDAE: NOTOMICRINAE: NOTOMICRINI.

Type species: *Notomicrusbrevicornis* Sharp, 1882, by subsequent designation (Guignot 1946: 115).

Family-group name: NOTOMICRINI Branden, 1884. Stem: *Notomicr*-.

***Notophysis* Audinet-Serville, 1832**: 158. Current status: valid genus in CERAMBYCIDAE: PRIONINAE: CACOSCELINI.

Type species: *Notophysislucanoides* Audinet-Serville, 1832, by monotypy.

Family-group name: NOTHOPHYSINI Lameere, 1903. Stem: *Notophyse*-.

***Notosacantha* Chevrolat in Dejean, 1836a**: 367. Current status: valid genus in CHRYSOMELIDAE: CASSIDINAE: NOTOSACANTHINI.

Type species: *Cassidaechinata* Fabricius, 1801, by monotypy.

Family-group name: NOTOSACANTHINA Gressitt, 1952. Stem: *Notosacanth*-.

***Nototylus* Gemminger & Harold, 1868a**: 161. Current status: valid genus in CARABIDAE: NOTOTYLINAE. Note: new replacement name for *Tylonotus* Schaum, 1863.

Type species: *Tylonotusfryi* Schaum, 1863, by monotypy (type fixation for *Tylonotus* Schaum).

Family-group name: NOTOTYLINI Bänninger, 1927. Stem: *Nototyl*-.

***Notoxus* Geoffroy, 1762**: 356. Current status: valid genus in ANTHICIDAE: NOTOXINAE. Note: name placed on the Official List of Generic Names in Zoology (ICZN 1994a).

Type species: *Attelabusmonoceros* Linnaeus, 1761, by subsequent designation (Latreille 1810: 430; see ICZN 1994a). Note: name placed on the Official List of Specific Names in Zoology (ICZN 1994a).

Family-group name: NOTOXIDAE Stephens, 1829. Stem: *Notox*-.

***Novius* Mulsant, 1846**: [4]. Current status: valid genus in COCCINELLIDAE: COCCINELLINAE: NOVIINI. Note: the spelling *Nomius* (p. 213) was changed to *Novius* in the Addenda and Errata (p. [4]).

Type species: *Noviuscruentatus* Mulsant, 1846, by monotypy.

Family-group name: NOVIAIRES Mulsant, 1846. Stem: *Novi*-.

***Nucleotops* Perkins & J. Balfour-Browne, 1994**: 13. Current status: valid genus in HYDRAENIDAE: PROSTHETOPINAE: NUCLEOTOPINI.

Type species: *Nucleotopsnimbaceps* Perkins & J. Balfour-Browne, 1994, by original designation.

Family-group name: NUCLEOTOPINI Perkins, 1994. Stem: *Nucleotop*-.

***Nyassinus* Westwood, 1879**: 199. Current status: valid genus in SCARABAEIDAE: CETONIINAE: CREMASTOCHEILINI: NYASSININA.

Type species: *Nyassinusmaculipes* Westwood, 1879, by subsequent designation (Marais and Holm 1992: 53).

Family-group name: NYASSININA Krikken, 1984. Stem: *Nyassin*-.

***Nyctelia* Berthold, 1827**: 367. Current status: valid genus in TENEBRIONIDAE: PIMELIINAE: NYCTELIINI. Note: nomen protectum (see Silvestro and Flores 2016: 658).

Type species: *Zophosisnodosa* Germar, 1823 (= *Zophosispicipes* Billberg, 1815), by monotypy.

Family-group name: NYCTÉLITES Solier, 1834. Stem: *Nycteli*-.

***Nycteropus* Klug, 1833**: 89. Current status: valid genus in TENEBRIONIDAE: TENEBRIONINAE: TOXICINI: NYCTEROPINA.

Type species: *Nycteropusebeninus* Klug, 1833, by subsequent designation (Hope 1841a: 124).

Family-group name: NYCTÉROPIDES Lacordaire, 1859. Stem: *Nycterop*-.

***Nyctimene* J. Thomson, 1857**: 314. Current status: invalid senior synonym of *Nyctimenius* Gressitt, 1951 in CERAMBYCIDAE: LAMIINAE: NYCTIMENIINI. Note: junior homonym of *Nyctimene* Borkenhausen, 1797 [Mammalia].

Type species: *Nyctimeneagriloides* J. Thomson, 1857 (= *Saperdavaricornis* Fabricius, 1801), by monotypy.

Family-group name: NYCTIMENITAE J. Thomson, 1864. Stem: *Nyctimen*-. Note: family-group name permanently invalid (ICZN 1999a, Article 39).

***Nyctimenius* Gressitt, 1951**: 629. Current status: valid genus in CERAMBYCIDAE: LAMIINAE: NYCTIMENIINI. Note: new replacement name for *Nyctimene* J. Thomson, 1857.

Type species: *Nyctimeneagriloides* J. Thomson, 1857 (= *Saperdavaricornis* Fabricius, 1801), by monotypy (type fixation for *Nyctimene* J. Thomson).

Family-group name: NYCTIMENIINI Gressitt, 1951. Stem: *Nyctimeni*-.

***Nyctophyxis* C. Costa, 1975**: 86. Current status: valid genus in ELATERIDAE: AGRYPNINAE: PYROPHORINI: NYCTOPHYXINA. Note: two original spellings were used, including *Nyctophixis* (p. 86); Sáiz et al. (1993: 70) acted as First Revisers (ICZN 1999a, Article 24.2.3) and selected *Nyctophyxis*, the spelling currently in prevailing usage.

Type species: *Pyrophorusocellatus* Germar, 1841, by original designation.

Family-group name: NYCTOPHYXINA C. Costa, 1975. Stem: *Nyctophyx*-.

***Nyctoporis* Eschscholtz, 1831**: 10, 11. Current status: valid genus in TENEBRIONIDAE: PIMELIINAE: NYCTOPORINI.

Type species: *Nyctoporiscristata* Eschscholtz, 1831, by subsequent designation (Hope 1841a: 124).

Family-group name: NYCTOPORIDES Lacordaire, 1859. Stem: *Nyctopor*-. Note: senior homonym of NYCTOPORINAE Hill, 1951 (type genus *Nyctopora* Nicholson, 1879) in Coelenterata.

***Nyctor* Semenov-Tjan-Shansky & Pjatakova, 1936**: 102. Current status: valid genus in ELATERIDAE: CARDIOPHORINAE.

Type species: *Nyctorexpallidus* Semenov-Tjan-Shansky & Pjatakova, 1936, by original designation.

Family-group name: NYCTORINI Semenov-Tjan-Shansky & Pjatakova, 1936. Stem: *Nyctor*-.

***Nyctozoilus* Guérin-Méneville, 1831a**: pl. 4. Current status: valid genus in TENEBRIONIDAE: TENEBRIONINAE: HELEINI: CYPHALEINA.

Type species: *Nyctozoilusobesus* Guérin-Méneville, 1831, by monotypy.

Family-group name: NYCTOZOÏLIDES Lacordaire, 1859. Stem: *Nyctozoil*-.

***Nymphister* Reichensperger, 1933**: 188. Current status: valid genus in HISTERIDAE: HAETERIINAE: NYMPHISTERINI.

Type species: *Nymphistersimplicissimus* Reichensperger, 1933, by original designation.

Family-group name: NYMPHISTERINI Tishechkin, 2007. Stem: *Nymphister*- (ICZN 1999a, Article 29.4).

***Oberea* Dejean, 1835**: 351. Current status: valid genus in CERAMBYCIDAE: LAMIINAE: SAPERDINI.

Type species: *Cerambyxlinearis* Linnaeus, 1761, by subsequent designation (C.G. Thomson 1859: 153).

Family-group name: OBEREITAE J. Thomson, 1864. Stem: *Obere*-.

†***Oborocoleus* Kukalová, 1969**: 155. Current status: valid genus in †OBOROCOLEIDAE. Note: Permian (Czech Republic).

Type species: †*Oborocoleusrohdendorfi* Kukalová, 1969, by original designation.

Family-group name: †OBOROCOLEIDAE Kukalová, 1969. Stem: *Oborocole*-.

†***Obrienia* Zherikhin & Gratshev, 1994**: 52. Current status: valid genus in †OBRIENIIDAE: †OBRIENIINAE. Note: Middle/Upper Triassic (Kyrgyzstan).

Type species: †*Obrieniakuscheli* Zherikhin & Gratshev, 1994, by original designation.

Family-group name: †OBRIENIIDAE Zherikhin & Gratshev, 1994. Stem: *Obrieni*-.

***Obrium* Dejean, 1821**: 110. Current status: valid genus in CERAMBYCIDAE: CERAMBYCINAE: OBRIINI.

Type species: *Cerambyxcantharinus* Linnaeus, 1767, by subsequent designation (Curtis 1825: pl. 91).

Family-group name: OBRIAIRES Mulsant, 1839. Stem: *Obri*-.

***Ocalea* Erichson, 1837**: 298. Current status: valid genus in STAPHYLINIDAE: ALEOCHARINAE: OXYPODINI: OXYPODINA.

Type species: *Ocaleacastanea* Erichson, 1837 (= *Aleocharapicata* Stephens, 1832), by subsequent designation (Weimar 1839: 506).

Family-group name: OCALEIDES C.G. Thomson, 1859. Stem: *Ocale*-.

***Ochina* Dejean, 1821**: 40. Current status: valid genus in PTINIDAE: ERNOBIINAE: OCHININI.

Type species: *Ptilinushederae* P.W.J. Müller, 1821 (= *Criocerisptinoides* Marsham, 1802), by subsequent designation (Blanchard 1845a: 91).

Family-group name: OCHININI Zahradník in Zahradník and Háva, 2014: 307. Stem: *Ochin*-.

***Ochodaeus* Dejean, 1821**: 56. Current status: valid genus in OCHODAEIDAE: OCHODAEINAE: OCHODAEINI.

Type species: *Melolonthachrysomelina* Fabricius, 1792 (= *Scarabaeuschrysomeloides* Schrank, 1781), by monotypy.

Family-group name: OCHODAEIDAE Streubel, 1846. Stem: *Ochodae*-.

***Ochrolitus* Sharp, 1889b**: 264. Current status: valid genus in PHALACRIDAE: PHALACRINAE.

Type species: *Ochrolitusoptatus* Sharp, 1889, by monotypy.

Family-group name: OCHROLITINI Guillebeau, 1894. Stem: *Ochrolit*-.

***Ochthebius* Leach, 1815a**: 95. Current status: valid genus in HYDRAENIDAE: OCHTHEBIINAE: OCHTHEBIINI. Note: name placed on the Official List of Generic Names in Zoology (ICZN 1991a).

Type species: *Helophorusmarinus* Paykull, 1798, by subsequent designation (d’ Orchymont 1942: 2; see ICZN 1991a). Note: name placed on the Official List of Specific Names in Zoology ICZN 1991a).

Family-group name: OCHTEBIIDAE C.G. Thomson, 1859. Stem: *Ochthebi*-.

***Ochtheosus* Perkins, 1997**: 124. Current status: valid genus in HYDRAENIDAE: OCHTHEBIINAE: OCHTHEOSINI.

Type species: *Ochtheosusfungicolus* Perkins, 1997, by original designation.

Family-group name: OCHTHEOSINI Perkins, 1997. Stem: *Ochtheos*-.

***Ochyra* Pascoe, 1871c**: 273. Current status: valid genus in CERAMBYCIDAE: CERAMBYCINAE: OCHYRINI.

Type species: *Ochyracoarctata* Pascoe, 1871, by monotypy.

Family-group name: OCHYRINAE Pascoe, 1871. Stem: *Ochyr*-.

***Ochyromera* Pascoe, 1874**: 31, 33. Current status: valid genus in CURCULIONIDAE: CURCULIONINAE: OCHYROMERINI.

Type species: *Ochyromerarufescens* Pascoe, 1874, by subsequent designation (G.A.K. Marshall 1926: 361).

Family-group name: OCHYROMERINA Voss, 1935. Stem: *Ochyromer*-.

***Ochyropus* Schiødte, 1847**: 350. Current status: valid genus in CARABIDAE: SCARITINAE: SCARITINI: OCHYROPINA.

Type species: *Ochyropusgigas* Schiødte, 1847, by monotypy.

Family-group name: OCHYROPINI Basilewsky, 1973. Stem: *Ochyrop*-.

***Ocladius* Schönherr, 1825**: 587. Current status: valid genus in CURCULIONIDAE: BRACHYCERINAE: ERIRHININI.

Type species: *Rhynchaenussalicorniae* G.-A. Olivier, 1807, by original designation.

Family-group name: OCLADIIDES Lacordaire, 1865. Stem: *Ocladi*-.

***Ocnoscelis* Erichson, 1847c**: 174. Current status: valid genus in CHRYSOMELIDAE: GALERUCINAE: ALTICINI.

Type species: *Ocnosceliscyanoptera* Erichson, 1847, by **present designation**.

Family-group name: OCNOSCELINA Bechyné, 1997. Stem: *Ocnoscelid*-.

***Ochthephilus* Nietner, 1857**: 136. Current status: invalid senior synonym of *Perileptus* Schaum, 1860 in CARABIDAE: TRECHINAE: TRECHINI: TRECHODINA. Note: junior homonym of *Ochthephilus* Mulsant & Rey, 1856 [Coleoptera: STAPHYLINIDAE].

Type species: *Ochthephilusceylanicus* Nietner, 1857, by monotypy.

Family-group name: OCHTHEPHILINI Jeannel, 1922. Stem: *Ochthephil*-. Note: family-group name permanently invalid (ICZN 1999a, Article 39).

***Octocryptus* Candèze, 1892**: 486. Current status: valid genus in ELATERIDAE: AGRYPNINAE: AGRYPNINI.

Type species: *Octocryptuscardoni* Candèze, 1892, by monotypy.

Family-group name: OCTOCRYPTINI O. Schwarz, 1906. Stem: *Octocrypt*-.

***Octogonotes* Drapiez, 1820**: 191. Current status: valid genus in CHRYSOMELIDAE: GALERUCINAE: ALTICINI.

Type species: *Octogonotesbanoni* Drapiez, 1820, by monotypy.

Family-group name: OCTOGONOTINI Weise, 1921. Stem: *Octogonot*-.

***Octomicrus* L.W. Schaufuss, 1877a**: 14. Current status: valid genus in STAPHYLINIDAE: PSELAPHINAE: EUPLECTITAE: DIMERINI.

Type species: *Octomicruslongulus* L.W. Schaufuss, 1877, by monotypy.

Family-group name: OCTOMICRINI Jeannel, 1952. Stem: *Octomicr*-.

***Octotemnus* Mellié, 1847**: 110. Current status: valid genus in CIIDAE: CIINAE: OROPHIINI.

Type species: *Cisglabriculus* Gyllenhal, 1827, by subsequent designation (Jacquelin du Val 1860b: 239).

Family-group name: OCTOTEMNIDAE Reitter, 1878. Stem: *Octotemn*-.

***Octotoma* Dejean, 1836a**: 366. Current status: valid genus in CHRYSOMELIDAE: CASSIDINAE: UROPLATINI.

Type species: *Hispaplicatula* Fabricius, 1801, by monotypy.

Family-group name: OCTOTOMIDES G.H. Horn, 1884. Stem: *Octotom*-.

***Ocularia* Jordan, 1894a**: 202. Current status: valid genus in CERAMBYCIDAE: LAMIINAE: OCULARIINI.

Type species: *Oculariaapicalis* Jordan, 1894, by original designation.

Family-group name: OCULARIINI Breuning, 1950. Stem: *Oculari*-.

***Ocypus* Leach in Samouelle, 1819**: 172. Current status: valid genus in STAPHYLINIDAE: STAPHYLININAE: STAPHYLININI: STAPHYLININA.

Type species: *Staphylinuscyaneus* Paykull, 1789 (= *Staphylinusophthalmicus* Scopoli, 1763), by original designation.

Family-group name: OCYPINA Hatch, 1957. Stem: *Ocypod*-. Note: junior homonym of OCYPODIDAE Rafinesque, 1815 (type genus *Ocypode* Weber, 1795) in Crustacea.

***Ocyusa* Kraatz, 1856**: 156. Current status: valid genus in STAPHYLINIDAE: ALEOCHARINAE: OXYPODINI: OXYPODINA.

Type species: *Oxypodamaura* Erichson, 1837, by subsequent designation (C.G. Thomson 1859: 36).

Family-group name: OCYUSATES Mulsant & Rey, 1874. Stem: *Ocyus*-.

***Odacantha* Paykull, 1798**: 169. Current status: valid genus in CARABIDAE: HARPALINAE: ODACANTHINI.

Type species: *Attelabusmelanurus* Linnaeus, 1767, by monotypy.

Family-group name: ODACANTHIDAE Laporte, 1834. Stem: *Odacanth*-.

***Odochilus* Harold, 1877**: 97. Current status: valid genus in SCARABAEIDAE: APHODIINAE: ODOCHILINI.

Type species: *Odochilussyntheticus* Harold, 1877, by monotypy.

Family-group name: ODOCHILINI Rakovič, 1987. Stem: *Odochil*-.

***Odontalgus* Raffray, 1877**: 286. Current status: valid genus in STAPHYLINIDAE: PSELAPHINAE: PSELAPHITAE: ODONTALGINI.

Type species: *Odontalgustuberculatus* Raffray, 1877, by subsequent designation (Jeannel 1949a: 178).

Family-group name: ODONTALGINI Jeannel, 1949. Stem: *Odontalg*-.

***Odonteus* Samouelle, 1819**: 189. Current status: valid genus in BOLBOCERATIDAE: BOLBOCERATINAE: ODONTEINI. Note: name given priority over *Bolboceras* W. Kirby, 1819 whenever the two are considered synonyms and *Odonteus* Samouelle, 1819 placed on the Official List of Generic Names in Zoology (ICZN 2006a).

Type species: *Scarabaeusmobilicornis* Fabricius, 1775 (= *Scarabaeusarmiger* Scopoli, 1772), by monotypy (see ICZN 2006a).

Family-group name: ODONTAEIDAE Streubel, 1846. Stem: *Odonte*-.

***Odontionopa* Chevrolat in Dejean, 1836a**: 408. Current status: valid genus in CHRYSOMELIDAE: EUMOLPINAE: EURYOPINI.

Type species: *Colaspissericea* Gyllenhal, 1808, by monotypy.

Family-group name: ODONTIONOPITES Lefèvre, 1876. Stem: *Odontionop*-.

***Odontionopa* Erichson, 1842**: 232. Current status: invalid senior synonym of *Eboo* Reid, 1993 in CHRYSOMELIDAE: EUMOLPINAE: BROMIINI. Note: junior homonym of *Odontionopa* Chevrolat, 1836 in Coleoptera: CHRYSOMELIDAE.

Type species: *Colaspisviridula* Erichson, 1842, by subsequent designation (Reid 1993: 62).

Family-group name: ODONTIONOPINI Clavareau, 1914. Stem: *Odontionop*-. Note: family-group name permanently invalid (ICZN 1999a, Article 39).

***Odontocheila* Laporte, 1834a**: 34. Current status: valid genus in CICINDELIDAE: CICINDELINI: DROMICINA. Note: Laporte used two original spellings, including *Odontacheila* (p. 35); Laporte (1840a: 20) subsequently used only *Odontocheila* and therefore acted as First Reviser (ICZN 1999a, Article 24.2.4).

Type species [suggested]: *Odontocheiladesmarestii* Laporte, 1834 (= *Cicindelafulgens* Klug, 1834), by subsequent designation (Bousquet 2002: 35). Note: the valid type species fixation is that of Hope (1838a: 15), who selected *Cicindelalacordairei* Guérin- Méneville, 1833, but that species is currently included in the subgenus Poecilochila Rivalier, 1969 of the genus *Pentacomia* H.W. Bates, 1872; an application to the Commission is necessary to fix *Odontocheiladesmarestii* Laporte, 1834 as the type species of *Odontocheila* Laporte, 1834.

Family-group name: ODONTOCHILINI W. Horn, 1899. Stem: *Odontocheil*-.

***Odontolabis* Hope, 1842b**: 127. Current status: valid genus in LUCANIDAE: LUCANINAE: ODONTOLABIDINI.

Type species: *Odontolabiscuvera* Hope, 1842, by subsequent designation (Benesh 1960: 112).

Family-group name: ODONTOLABIDAE Parry, 1870. Stem: *Odontolabid*-.

***Odontolochus* A. Schmidt, 1917**: 99. Current status: valid genus in SCARABAEIDAE: APHODIINAE: ODONTOLOCHINI. Note: new replacement name for *Odontoderus* Clouët des Pesruches, 1900.

Type species: *Ataeniusspinicollis* Harold, 1871, by subsequent designation (R. Lucas 1920: 450) (type fixation for *Odontoderus* Clouët des Pesruches).

Family-group name: ODONTOLOCHINI Stebnicka & Howden, 1996. Stem: *Odontoloch*-.

***Odontoloma* Boheman, 1857**: 202. Current status: valid genus in SCARABAEIDAE: SCARABAEINAE: ODONTOLOMINI.

Type species: *Odontolomapauxillum* Boheman, 1857, by monotypy.

Family-group name: ODONTOLOMINI Davis, Deschodt & Scholtz, 2019: 136. Stem: *Odontolom*-

***Odontonychus* Candèze, 1897**: 26. Current status: valid genus in ELATERIDAE: ELATERINAE: ODONTONYCHINI.

Type species: *Odontonychusgranulatus* Candèze, 1897, by monotypy.

Family-group name: ODONTONYCHINI C. Girard, 1973. Stem: *Odontonych*-.

***Odontosphindus* J.L. LeConte, 1878b**: 601. Current status: valid genus in SPHINDIDAE: ODONTOSPHINDINAE.

Type species: *Odontosphindusdenticollis* J.L. LeConte, 1878, by monotypy.

Family-group name: ODONTOSPHINDINI Sen Gupta & Crowson, 1979. Stem: *Odontosphind*-.

***Oedemera* G.-A. Olivier, 1789a**: 31. Current status: valid genus in OEDEMERIDAE: OEDEMERINAE: OEDEMERINI.

Type species: *Necydaliscaerulea* Linnaeus, 1767 (= *Cantharisnobilis* Scopoli, 1763), by subsequent designation (Latreille 1810: 430). Note: no species were originally included in this genus by G.-A. Olivier (1789a); the first ones were the 20 species listed by G.-A. Olivier (1795: [50] 4–15).

Family-group name: OEDEMERITES Latreille, 1810. Stem: *Oedemer*-.

***Oedenoderus* Chevrolat, 1858**: 245. Current status: valid genus in CERAMBYCIDAE: CERAMBYCINAE: OEDENODERINI.

Type species: *Oedenoderuspupa* Chevrolat, 1858, by subsequent designation (J. Thomson 1861: 251).

Family-group name: OEDENODERINI Aurivillius, 1912. Stem: *Oedenoder*-.

***Oedionychis* Latreille, 1829b**: 154. Current status: valid genus in CHRYSOMELIDAE: GALERUCINAE: ALTICINI.

Type species: *Galerucamarginella* Fabricius, 1801 (= *Chrysomelacincta* Fabricius, 1787), by subsequent designation (Heikertinger 1922: 46).

Family-group name: OEDIONYCHITES Chapuis, 1875. Stem: *Oedionych*-.

***Oediopalpa* Baly, 1858**: 16. Current status: valid genus in CHRYSOMELIDAE: CASSIDINAE: OEDIOPALPINI.

Type species: *Hispacyanipennis* Fabricius, 1801, by subsequent designation (Monrós and Viana 1947: 151).

Family-group name: OEDIOPALPINI Monrós & Viana, 1947. Stem: *Oediopalp*-.

***Oeme* Newman, 1840c**: 8. Current status: valid genus in CERAMBYCIDAE: CERAMBYCINAE: OEMINI: OEMINA.

Type species: *Oemeindecora* Newman, 1840 (= *Stenocorusrigidus* Say, 1826), by monotypy.

Family-group name: OEMIDES Lacordaire, 1868. Stem: *Oem*-.

***Oeneis* Mulsant, 1850**: 500. Current status: invalid senior synonym of *Calloeneis* Grote, 1873 in COCCINELLIDAE: COCCINELLINAE: CRYPTOGNATHINI. Note: junior homonym of *Oeneis* Hübner, 1819 [Lepidoptera].

Type species: *Oeneisnigrans* Mulsant, 1850, by subsequent designation (Crotch 1874a: 206).

Family-group name: OENEINI Casey, 1899. Stem: *Oene*-. Note: family-group name permanently invalid (ICZN 1999a, Article 39).

***Oestodes* J.L. LeConte, 1853f**: 422, 424. Current status: valid genus in ELATERIDAE: OESTODINAE.

Type species: *Elatertenuicollis* Randall, 1838, by subsequent designation (Hyslop 1921: 659).

Family-group name: OESTODINI Hyslop, 1917. Stem: *Oestod*-.

***Oides* Weber, 1801**: 26, 53. Current status: valid genus in CHRYSOMELIDAE: GALERUCINAE: OIDINI.

Type species: *Chrysomelabipunctata* Fabricius, 1781 (= *Oidesandrewesi* Jacoby, 1900), by subsequent designation (Laboissière 1922: 195).

Family-group name: OÏDITES Laboissière, 1921. Stem: *Oid*-.

***Oileus* Kaup, 1868b**: 19. Current status: valid genus in PASSALIDAE: PASSALINAE: PROCULINI.

Type species: *Passalusrimator* Truqui, 1857, by monotypy. Note: Kaup (1868b: 19) listed four species-group names in this genus but only one was available at the time.

Family-group name: OILEINAE Kuwert, 1891. Stem: *Oile*-.

†***Oiserhinus* Legalov, Kirejtshuk & Nel, 2020b**: 730. Current status: valid genus in ANTHRIBIDAE: ANTHRIBINAE: †OISERHININI. Note: lower Eocene (France).

Type species: †*Oiserhinusinsolitus* Legalov, Kirejtshuk & Nel, 2020, by original designation.

Family-group name: †OISERHININI Legalov, Kirejtshuk & Nel, 2020b: 730. Stem: *Oiserhin*-.

***Oisocerus* Bonvouloir in Murray, 1868**: 103. Current status: valid genus in EUCNEMIDAE: MACRAULACINAE: OISOCERINI.

Type species: *Oisocerusmurrayi* Bonvouloir, 1868, by monotypy.

Family-group name: OISOCERINI Muona, 1993. Stem: *Oisocer*-.

***Olexandrella* Zajciw, 1960**: 606. Current status: valid genus in CERAMBYCIDAE: CERAMBYCINAE: DODECOSINI.

Type species: *Olexandrellaserotina* Zajciw, 1960, by original designation.

Family-group name: OLEXANDRELLAEINI Zajciw, 1960. Stem: *Olexandrell*-.

***Olibrus* Erichson, 1845a**: 108, 113. Current status: valid genus in PHALACRIDAE: PHALACRINAE.

Type species: *Sphaeridiumaeneum* Fabricius, 1792, by subsequent designation (C.G. Thomson 1859: 66). Note: the type species usually recognized is *Sphaeridiumbicolor* Fabricius, 1792 (e.g., Švec 2007: 508) by subsequent designation of Guillebeau (1892: 147) but C.G. Thomson’s typification is earlier; both species are currently included in the genus *Olibrus*.

Family-group name: OLIBRINI Guillebeau, 1892. Stem: *Olibr*-.

***Oligocys* Gistel, 1856**: 373. Current status: junior synonym of *Liophloeus* Germar, 1817 in CURCULIONIDAE: ENTIMINAE: POLYDRUSITAE: POLYDRUSINI.

Type species: *Curculionubilus* Fabricius, 1777 (= *Curculiotessulatus* O.F. Müller, 1776), by monotypy.

Family-group name: OLIGOCYIDAE Gistel, 1856. Stem: *Oligoce*-.

***Oligota* Mannerheim, 1830**: 72. Current status: valid genus in STAPHYLINIDAE: ALEOCHARINAE: HYPOCYPHTINI.

Type species: *Aleocharapusillima* Gravenhorst, 1806, by monotypy.

Family-group name: OLIGOTIDES C.G. Thomson, 1859. Stem: *Oligot*-.

***Olisthaerus* Dejean, 1833a**: 69. Current status: valid genus in STAPHYLINIDAE: OLISTHAERINAE.

Type species: *Staphylinussubstriatus* Paykull, 1790, by monotypy.

Family-group name: OLISTHAERINI C.G. Thomson, 1858. Stem: *Olisthaer*-.

***Olocrates* Mulsant, 1854**: 147, 150, 383. Current status: junior synonym of *Phylan* Sturm, 1826 in TENEBRIONIDAE: BLAPTINAE: DENDARINI: DENDARINA. Note: this name was spelled *Omocrates* (pp. 147, 150) but changed to *Olocrates* in the errata of the same work (p. 383).

Type species: *Opatrumgibbum* Fabricius, 1775, by subsequent designation (C.G. Thomson 1859: 115, as *Omocrates*).

Family-group name: OLOCRATARII Baudi di Selve, 1875. Stem: *Olocrat*-.

***Olotelus* Mulsant & Rey, 1866**: 22. Current status: invalid senior synonym of *Gompelia* Alonso-Zarazaga, 2010 in ADERIDAE. Note: junior homonym of *Olotelus* Solier, 1851 [Coleoptera: ELATERIDAE].

Type species: *Xylophilusneglectus* Jacquelin du Val, 1863, by subsequent designation (Alonso- Zarazaga 2010: 299).

Family-group name: OLOTELINA Báguena Corella, 1948. Stem: *Olotel*-. Note: family-group name permanently invalid (ICZN 1999a, Article 39).

***Omalisus* Geoffroy, 1762**: 179. Current status: valid genus in ELATERIDAE: OMALISINAE. Note: name placed on the Official List of Generic Names in Zoology (ICZN 1994a).

Type species: *Omalisusfontisbellaquaei* Geoffroy, 1785, by subsequent monotypy (de Fourcroy 1785: 64; see ICZN 1994a). Note: name placed on the Official List of Specific Names in Zoology (ICZN 1994a).

Family-group name: HOMALISIDES Lacordaire, 1857. Stem: *Omalis*-.

***Omalium* Gravenhorst, 1802**: 111. Current status: valid genus in STAPHYLINIDAE: OMALIINAE: OMALIINI. Note: name placed on the Official List of Generic Names in Zoology (ICZN 2015b).

Type species: *Staphylinusrivularis* Paykull, 1789, by subsequent designation (Latreille 1810: 427; see ICZN 2015b).

Family-group name: OMALIDAE W.S. MacLeay, 1825. Stem: *Omali*-. Note: the spelling of the family-group name OMALIIDAE Handlirsch, 1904 [Archaeorthoptera] was ruled to be OMALIAIDAE by the Commission (see ICZN 2015b) to remove the homonymy with W.S. MacLeay’s name.

***Omalodera* Blanchard, 1842b**: pl. 1. Current status: valid genus in CARABIDAE: TRECHINAE: TRECHINI: TRECHINA.

Type species: *Omaloderalimbata* Blanchard, 1842, by subsequent designation (Bousquet 2002: 36).

Family-group name: HOMALODERINI Jeannel, 1926. Stem: *Omaloder*-.

***Omalodes* Dejean, 1833b**: 128. Current status: valid genus in HISTERIDAE: HISTERINAE: OMALODINI.

Type species: *Histeromega* W. Kirby, 1818, by subsequent designation (Hope 1841a: 105).

Family-group name: OMALODINI Kryzhanovskij, 1972. Stem: *Omalod*-.

***Omaloplia* Schönherr, 1817**: 178. Current status: valid genus in SCARABAEIDAE: SERICINAE: SERICINI: SERICINA.

Type species [suggested]: *Melolontharuricola* Fabricius, 1775, by subsequent designation (Stephens 1832c: 628). Note: the valid type species fixation is that of Thon (1832b: 347), who selected *Scarabaeusbrunneus* Linnaeus, 1758, but that nominal species is currently the type species of the genus *Serica* W.S. MacLeay, 1819; an application to the Commission is necessary to fix *Melolontharuricola* Fabricius, 1775 as the type species of *Omaloplia* Schönherr, 1817.

Family-group name: OMALOPLIITES Blanchard, 1845. Stem: *Omalopli*-.

***Omethes* J.L. LeConte, 1861**: 187. Current status: valid genus in OMETHIDAE: OMETHINAE.

Type species: *Omethesmarginatus* J.L. LeConte, 1861, by monotypy.

Family-group name: OMETHES J.L. LeConte, 1861. Stem: *Ometh*-.

***Ometis* Latreille, 1829a**: 554. Current status: junior synonym of *Lagochile* Hoffmannsegg, 1817 in SCARABAEIDAE: RUTELINAE: RUTELINI: RUTELINA.

Type species: *Rutelacetonioides* Lepeletier & Audinet-Serville, 1825, by subsequent designation (Brullé 1838: 349).

Family-group name: OMETIDAE Streubel, 1846. Stem: *Omet*-.

***Omias* Germar, 1817b**: 341. Current status: valid genus in CURCULIONIDAE: ENTIMINAE: CYPHICERITAE: OMIINI.

Type species: *Curculiorotundatus* Fabricius, 1792 (= *Omiaspuberulus* Boheman, 1834), by subsequent designation (Schönherr 1823: 1144).

Family-group name: OMIADAE Shuckard, 1839. Stem: *Omi*-. Note: senior homonym of OMIINI Beck, 1996 (type genus *Omia* Hübner, 1821) in Lepidoptera.

***Omicrus* Sharp, 1879**: 81. Current status: valid genus in HYDROPHILIDAE: SPHAERIDIINAE: OMICRINI.

Type species: *Omicrusbrevipes* Sharp, 1879, by monotypy.

Family-group name: OMICRINI Smetana, 1975. Stem: *Omicr*-.

***Omileus* G.H. Horn in J.L. LeConte and G.H. Horn, 1876**: 102. Current status: valid genus in CURCULIONIDAE: ENTIMINAE: POLYDRUSITAE: EUSTYLINI: EUSTYLINA.

Type species: *Omileusepicaeroides* G.H. Horn, 1876, by monotypy.

Family-group name: OMILEI G.H. Horn, 1876. Stem: *Omile*-.

***Omma* Newman, 1839**: 303. Current status: valid genus in OMMATIDAE: OMMATINAE.

Type species: *Ommastanleyi* Newman, 1839, by monotypy.

Family-group name: OMMADIDAE Sharp & Muir, 1912. Stem: *Ommat*-.

***Ommatolampes* Schönherr, 1838**: 837. Current status: valid genus in CURCULIONIDAE: DRYOPHTHORINAE: OMMATOLAMPINI.

Type species: *Calandrahaemorrhoidalis* Wiedemann, 1819, by original designation.

Family-group name: OMMATOLAMPIDES Lacordaire, 1865. Stem: *Ommatolamp*-.

***Omocerus* Chevrolat, 1835c**: no 119. Current status: valid genus in CHRYSOMELIDAE: CASSIDINAE: OMOCERINI. Note: the spelling *Omocerus* was changed to *Omocera* in the corrections ([p. 7] at the end of the work) issued in the same volume; therefore, the correct original spelling is *Omocera* but *Omocerus*, an incorrect subsequent spelling used subsequently by several authors, is in prevailing usage, attributed to the publication of the original spelling, and therefore deemed to be the correct original spelling (ICZN 1999a, Article 33.3.1).

Type species: *Cassidabicornis* Linnaeus, 1763, by subsequent designation (Duponchel and Chevrolat 1842b: 210).

Family-group name: OMOCERINI Hincks, 1952. Stem: *Omocer*-.

***Omoglymmius* Ganglbauer, 1891**: 533, 534. Current status: valid genus in CARABIDAE: RHYSODINAE: OMOGLYMMIINI.

Type species: *Rhysodesgermari* Ganglbauer, 1891, by monotypy.

Family-group name: OMOGLYMMIINA R.T. Bell & J.R. Bell, 1978. Stem: *Omoglymmi*-.

***Omolabus* Jekel, 1860**: 191. Current status: valid genus in ATTELABIDAE: ATTELABINAE: ATTELABINI: OMOLABINA.

Type species: *Attelabusbifoveatus* Jekel, 1860, by original designation.

Family-group name: OMOLABINA Legalov, 2003. Stem: *Omolab*-.

***Omophlus* Dejean, 1834**: 213. Current status: valid genus in TENEBRIONIDAE: ALLECULINAE: CTENIOPODINI. Note: the earlier usage of the name *Omophlus* by Dahl (1823: 46) was suppressed for the purposes of zoological nomenclature (see ICZN 1964).

Type species: *Cistelalepturoides* Fabricius, 1787, by subsequent designation (Solier 1835b: 246).

Family-group name: OMOPHLIENS Mulsant, 1856. Stem: *Omophl*-.

***Omophorus* Schönherr, 1835**: 479. Current status: valid genus in CURCULIONIDAE: MOLYTINAE: METATYGINI.

Type species: *Omophorusstomachosus* Boheman, 1835, by original designation.

Family-group name: OMOPHORINAE G.A.K. Marshall, 1917. Stem: *Omophor*-.

***Omophron* Latreille, 1802b**: 89. Current status: valid genus in CARABIDAE: OMOPHRONINAE. Note: new replacement name for *Scolytus* Fabricius, 1790.

Type species: *Carabuslimbatus* Fabricius, 1777, by subsequent designation (Latreille 1810: 426).

Family-group name: OMOPHRONII Bonelli, 1810. Stem: *Omophron*-.

***Omorgus* Erichson, 1847c**: 111. Current status: valid genus in TROGIDAE: OMORGINAE.

Type species: *Troxsuberosus* Fabricius, 1775, by subsequent designation (Lacordaire 1855: 151).

Family-group name: OMORGINI Nikolajev, 2005. Stem: *Omorg*-.

***Omphra* Dejean, 1825**: 285. Current status: valid genus in CARABIDAE: HARPALINAE: HELLUONINI: OMPHRINA. Note: this name was first published as a junior synonym of *Helluo* Bonelli, 1813 and, because it was treated as an available and valid name prior to 1961 (e.g., Reiche 1843b: 330), it is available and dates from its first publication as a synonym (ICZN 1999a, Article 11.6.1).

Type species: *Galeritahirta* Fabricius, 1801, by monotypy.

Family-group name: OMPHRINI Jedlička, 1941. Stem: *Omphr*-.

***Omphreus* Dejean, 1828**: 4, 93. Current status: valid genus in CARABIDAE: HARPALINAE: OMPHREINI.

Type species: *Omphreusmorio* Dejean, 1828, by monotypy.

Family-group name: OMPHREINI Ganglbauer, 1891. Stem: *Omphre*-.

***Omus* Eschscholtz, 1829b**: 3, 4. Current status: valid genus in CICINDELIDAE: MANTICORINI.

Type species: *Omuscalifornicus* Eschscholtz, 1829, by monotypy.

Family-group name: OMITES W. Horn, 1907. Stem: *Om*-.

***Oncerus* J.L. LeConte, 1856b**: 283. Current status: valid genus in SCARABAEIDAE: ONCERINAE.

Type species: *Oncerusfloralis* J.L. LeConte, 1856, by monotypy.

Family-group name: ONCERINI J.L. LeConte, 1861. Stem: *Oncer*-.

***Oncideres* Lacordaire, 1830b**: 183. Current status: valid genus in CERAMBYCIDAE: LAMIINAE: ONCIDERINI. Note: the original spelling was *Oncyderes* but *Oncideres*, an incorrect subsequent spelling, is in prevailing usage, attributed to the publication of the original spelling, and therefore deemed to be the correct original spelling (ICZN 1999a, Article 33.3.1).

Type species: *Lamiavomicosa* Germar, 1823 (= *Lamiasaga* Dalman, 1823), by subsequent designation (J. Thomson 1864: 104). Note: no species were originally included in this genus by Lacordaire (1830b); the first ones were the two species listed by Audinet- Serville (1835a: 68).

Family-group name: ONCIDERITAE J. Thomson, 1860. Stem: *Oncider*-.

***Oncideropsis* Aurivillius, 1922b**: 165. Current status: junior synonym of *Phelipara* Pascoe, 1866 in CERAMBYCIDAE: LAMIINAE: ONCIDEROPSIDINI.

Type species: *Oncideropsisnebulosa* Aurivillius, 1922, by monotypy.

Family-group name: ONCIDEROPSIDINI Aurivillius, 1922. Stem: *Oncideropsid*-.

***Oncocephala* Agassiz, 1846**: 259. Current status: junior synonym of *Onchocephala* Guérin-Méneville, 1844 in CHRYSOMELIDAE: CASSIDINAE: ONCOCEPHALINI. Note: unjustified emendation of *Onchocephala* Guérin-Méneville, 1844; Bouchard and Bousquet (2020a: 109) erroneously treated *Onchocephala* Guérin-Méneville, 1844 as a junior homonym of *Onchocephala* de Blainville, 1828 [Annelides], the name in Annelides is a suprageneric name and does not enter into homonymy at the genus-group level.

Type species: *Oncocephalaquadrilobata* Guérin-Méneville, 1844, by subsequent designation (Gressitt 1957: 265).

Family-group name: ONCOCÉPHALITES Chapuis, 1875. Stem: *Oncocephal*-.

***Oncomera* Stephens, 1829b**: 20. Current status: valid subgenus of *Oedemera* G.-A. Olivier, 1789 in OEDEMERIDAE: OEDEMERINAE: OEDEMERINI. Note: name placed on the Official List of Generic Names in Zoology (ICZN 1988f).

Type species: *Dryopsfemorata* Fabricius, 1792 (= *Oedemerafemoralis* G.-A. Olivier, 1803), by plenary power (see ICZN 1988f). Note: *Dryopsfemorata* Fabricius, 1792 was placed on the Official List of Specific Names in Zoology (ICZN 1988f).

Family-group name: ONCOMERADAE Gistel, 1856. Stem: *Oncomer*-.

***Oncosoma* Westwood, 1843c**: 121. Current status: junior synonym of *Amatodes* Dejean, 1834 in TENEBRIONIDAE: BLAPTINAE: PEDININI: HELOPININA. Note: the original spelling was *Ogcosoma* but the unjustified emendation *Oncosoma*, introduced by Agassiz (1846: 257, 259), is in prevailing usage, attributed to the original publication, and therefore deemed to be a justified emendation and the correct spelling (ICZN 1999a, Article 33.2.3.1).

Type species: *Oncosomagranulare* Westwood, 1843 (= *Pimeliagemmata* Fabricius, 1801), by monotypy.

Family-group name: ONCOSOMINA C. Koch, 1958. Stem: *Oncosom*-.

***Oncotus* Blanchard, 1845a**: 13, 24. Current status: valid genus in TENEBRIONIDAE: BLAPTINAE: PLATYNOTINI: EURYNOTINA. Note: this genus was described under the French vernacular name Oncote (p. 13) but listed in the same work under the scientific name *Oncotus* (p. 24).

Type species: *Oncotusfarctus* Solier, 1848, by subsequent designation (Gebien 1938b: 72). Note: no species were originally included in this genus by Blanchard (1845a); the first ones were the five species described by Solier (1848b: 216–222).

Family-group name: ONCOTINI C. Koch, 1953. Stem: *Oncot*-.

***Oniticellus* Dejean, 1821**: 53. Current status: valid genus in SCARABAEIDAE: SCARABAEINAE: ONITICELLINI: ONITICELLINA.

Type species [suggested]: *Scarabaeuscinctus* Fabricius, 1775, by subsequent designation (Arrow 1931: 375). Note: the valid type species fixation is that of Thon (1832b: 466), who selected *Scarabaeusflavipes* Fabricius, 1792, but that species is currently placed in the genus *Euoniticellus* A. Janssens, 1953; an application to the Commission is necessary to fix *Scarabaeuscinctus* Fabricius, 1775 as the type species of *Oniticellus* Dejean, 1821.

Family-group name: ONITICELLINI Kolbe, 1905. Stem: *Oniticell*-.

***Onitis* Fabricius, 1798**: 2. Current status: valid genus in SCARABAEIDAE: SCARABAEINAE: ONITINI.

Type species: *Scarabaeussphinx* Fabricius, 1775 [p. 25] (= *Onitisbelial* Fabricius, 1798), by subsequent designation (Latreille 1810: 428). Note: Fabricius (1798: 25–27) listed eight species in his new genus *Onitis* including *O.sphinx* (p. 26), which he referred to the *Scarabaeussphinx* of his work of 1792 (p. 53); in 1792, Fabricius referred his *S.sphinx* to the *Scarabaeussphinx* of G.-A. Olivier (1789b: [3] 135) who in turn referred the species to Fabricius (1775: 25); Fabricius (1775) described two species under the name *Scarabaeussphinx*, one on page 14 (from “Sierra Leon Africae”), the other on page 25 (from “America”), and the type species of *Onitis* is, following the sequence described above, the *Scarabaeussphinx* described by Fabricius (1775: 25) from “America,” which is, according to recent literature (e.g., Branco 2007: 16), a senior synonym of *Onitisbelial* Fabricius, 1798 described from “Cajennae”; the genus *Onitis*, except for adventive species in the Americas and Australia, is restricted to the southern Palaearctic, Afrotropical and Oriental Regions (Palestrini et al. 2002: 97) suggesting that the provenance given by Fabricius (1775 and 1798) for *Scarabaeussphinx* (p. 25) and *Onitisbelial* (p. 27) is incorrect.

Family-group name: ONITIDES Laporte, 1840. Stem: *Onit*-.

***Onocephala* Sturm, 1843**: 262. Current status: valid genus in CERAMBYCIDAE: LAMIINAE: ONCIDERINI.

Type species: *Saperdadiophthalma* Perty, 1830, by monotypy.

Family-group name: ONOCEPHALITAE J. Thomson, 1860. Stem: *Onocephal*-.

***Onthophagus* Latreille, 1802b**: 141. Current status: valid genus in SCARABAEIDAE: SCARABAEINAE: ONTHOPHAGINI.

Type species: *Scarabaeustaurus* Schreber, 1759, by monotypy.

Family-group name: ONTHOPHAGIDAE Streubel, 1846. Stem: *Onthophag*-.

***Onthophilus* Leach, 1817**: 76. Current status: valid genus in HISTERIDAE: ONTHOPHILINAE.

Type species: *Histerstriatus* Forster, 1771, by subsequent designation (Curtis 1828: pl. 220). Note: the type species often listed for this genus is *Histersulcatus* Moll, 1784 (= *Scolytuspunctatus* O.F. Müller, 1776) by subsequent designation of Westwood (1838c: 22) (e.g., Mazur 1997: 7; Bousquet and Laplante 2006: 423; Lackner et al. 2015: 110) but Curtis’ designation is older; both species are currently included in the genus *Onthophilus* Leach, 1817.

Family-group name: ONTHOPHILIDAE W.S. MacLeay, 1819. Stem: *Onthophil*-.

***Onychius* Chapuis, 1869**: 48. Current status: valid genus in CURCULIONIDAE: COSSONINAE: ONYCHIINI.

Type species: *Onychiusnitidus* Chapuis, 1869, by monotypy.

Family-group name: ONYCHIIDAE Chapuis, 1869. Stem: *Onychi*-. Note: junior homonym of ONYCHIINAE Forster, 1869 (type genus *Onychia* Haliday, 1830) in Hymenoptera.

***Onychocerus* Lacordaire, 1830b**: 181. Current status: valid genus in CERAMBYCIDAE: LAMIINAE: ANISOCERINI.

Type species: *Cerambyxscorpio* Fabricius, 1781, by monotypy.

Family-group name: ONYCHOCERITAE J. Thomson, 1864. Stem: *Onychocer*-.

***Onychoglenea* Aurivillius, 1922a**: 28. Current status: valid genus in CERAMBYCIDAE: LAMIINAE: ONYCHOGLENEINI.

Type species: *Onychogleneabrunnea* Aurivillius, 1922, by monotypy.

Family-group name: ONYCHOGLENEINI Aurivillius, 1923. Stem: *Onychoglene*-.

***Onycholips* Wollaston, 1861c**: 389. Current status: valid genus in CURCULIONIDAE: COSSONINAE: ONYCHOLIPINI.

Type species: *Onycholipsbifurcatus* Wollaston, 1861, by monotypy.

Family-group name: ONYCHOLIPIDES Wollaston, 1873. Stem: *Onycholip*-.

***Oochrotus* P.H. Lucas, 1852**: xxix. Current status: junior synonym of *Phtora* Germar, 1836 in TENEBRIONIDAE: DIAPERINAE: CRYPTICINI.

Type species: *Oochrotusunicolor* P.H. Lucas, 1852, by monotypy.

Family-group name: OOCHROTINI Desbrochers des Loges, 1901. Stem: *Oochrot*-.

***Oocyclus* Sharp, 1882c**: 61. Current status: valid genus in HYDROPHILIDAE: HYDROPHILINAE: LACCOBIINI.

Type species: *Oocyclusbrevicornis* Sharp, 1882, by subsequent designation (Knisch 1924: 181).

Family-group name: OOCYCLINI Hansen, 1991. Stem: *Oocycl*-.

***Oodes* Bonelli, 1810**: Tab. Syn. Current status: valid genus in CARABIDAE: HARPALINAE: OODINI.

Type species: *Carabushelopioides* Fabricius, 1792, by subsequent monotypy (Panzer 1813: 51).

Family-group name: OODITES LaFerté-Sénectère, 1851. Stem: *Ood*-.

***Oophorus* Dejean, 1833a**: 93. Current status: junior synonym of *Aeolus* Eschscholtz, 1829 in ELATERIDAE: AGRYPNINAE: OOPHORINI.

Type species: *Elaterelegans* Fabricius, 1792, by subsequent designation (Hyslop 1921: 659).

Family-group name: OOPHORIDAE Gistel, 1848. Stem: *Oophor*-.

***Oopterus* Guérin-Méneville, 1841**: 123. Current status: valid genus in CARABIDAE: TRECHINAE: ZOLINI.

Type species: *Oopterusclivinoides* Guérin-Méneville, 1841, by monotypy.

Family-group name: OOPTERINI Jeannel, 1940. Stem: *Oopter*-.

***Oosomus* Schönherr, 1826**: 195. Current status: valid genus in CURCULIONIDAE: ENTIMINAE: CYPHICERITAE: OOSOMINI.

Type species: *Peritelushariolus* Boheman, 1834, by subsequent designation (Alonso-Zarazaga and Lyal 1999: 22, 167).

Family-group name: OOSOMIDES Lacordaire, 1863. Stem: *Oosom*-.

***Oosternum* Sharp, 1882c**: 112. Current status: valid genus in HYDROPHILIDAE: SPHAERIDIINAE: MEGASTERNINI: OOSTERNINA. Note: name placed on the Official List of Generic Names in Zoology (ICZN 1998).

Type species: *Oosternumcostatum* Sharp, 1882, by monotypy. Note: name placed on the Official List of Specific Names in Zoology (ICZN 1998).

Family-group name: OOSTERNINA Arriaga-Varela & Fikáček in Arriaga-Varela et al. 2021: 577. Stem: *Oostern*-.

***Opanassenkovius* Legalov, 2003**: 555. Current status: valid genus in ATTELABIDAE: APODERINAE: APODERINI.

Type species: *Apoderusverrucosus* Pascoe, 1881, by original designation.

Family-group name: OPANASSENKOVIINA Legalov, 2003. Stem: *Opanassenkovi*-.

***Opatrinus* Dejean, 1821**: 66. Current status: valid genus in TENEBRIONIDAE: BLAPTINAE: PLATYNOTINI: PLATYNOTINA.

Type species: *Opatrumclathratum* Fabricius, 1787, by monotypy.

Family-group name: OPATRINAIRES Mulsant & Rey, 1853. Stem: *Opatrin*-.

***Opatrum* Fabricius, 1775**: 76. Current status: valid genus in TENEBRIONIDAE: BLAPTINAE: OPATRINI: OPATRINA.

Type species: *Silphasabulosa* Linnaeus, 1758, by subsequent designation (Latreille 1810: 429).

Family-group name: OPATRITES Brullé, 1832. Stem: *Opatr*-.

***Ophioglossa* Fauvel, 1866**: 255, 259. Current status: valid genus in STAPHYLINIDAE: ALEOCHARINAE: AUTALIINI.

Type species: *Ophioglossaaraucana* Fauvel, 1866, by monotypy.

Family-group name: OPHIOGLOSSAE Fenyes, 1919. Stem: *Ophiogloss*-.

***Ophonus* Dejean, 1821**: 13. Current status: valid genus in CARABIDAE: HARPALINAE: HARPALINI: HARPALINA. Note: name placed on the Official List of Generic Names in Zoology (ICZN 1990c).

Type species: *Carabussabulicola* Panzer, 1796, by plenary power (see ICZN 1990c). Note: name placed on the Official List of Specific Names in Zoology (ICZN 1990c).

Family-group name: OPHONIDAE Laporte, 1834. Stem: *Ophon*-.

***Ophryastes* Germar, 1829**: 358. Current status: valid genus in CURCULIONIDAE: ENTIMINAE: ENTIMITAE: OPHRYASTINI: OPHRYASTINA.

Type species: *Liparusvittatus* Say, 1824, by monotypy.

Family-group name: OPHRYASTIDES Lacordaire, 1863. Stem: *Ophryast*-.

***Ophtalmorrhynchus* Hoffmann, 1965**: 1408. Current status: valid genus in CURCULIONIDAE: ENTIMINAE: POLYDRUSITAE: DERMATODINI.

Type species: *Ophtalmorrhynchusdescarpentriesi* Hoffmann, 1965, by original designation.

Family-group name: OPHTALMORYNCHINI Hoffmann, 1965. Stem: *Ophtalmorrhynch*-.

***Opilo* Latreille, 1802b**: 111. Current status: valid genus in CLERIDAE: CLERINAE: OPILONITAE: OPILONINI: OPILONINA.

Type species: *Attelabusmollis* Linnaeus, 1758, by monotypy.

Family-group name: OPILONIDAE Gistel, 1848. Stem: *Opilon*-.

***Opisthius* W. Kirby, 1837**: 60. Current status: valid genus in CARABIDAE: NEBRIINAE: OPISTHIINI.

Type species: *Opisthiusrichardsoni* W. Kirby, 1837, by monotypy.

Family-group name: OPISTHIINAE P. Dupuis, 1912. Stem: *Opisthi*-.

***Oplosia* Mulsant, 1863**: 300. Current status: valid genus in CERAMBYCIDAE: LAMIINAE: ACANTHODERINI.

Type species: *Cerambyxfennicus* Paykull, 1800 (= *Exocentruscinereus* Mulsant, 1839), by monotypy.

Family-group name: HOPLOSIAE J.L. LeConte & G.H. Horn, 1883. Stem: *Oplosi*-.

***Oplostomus* W.S. MacLeay, 1838**: 20. Current status: valid genus in SCARABAEIDAE: CETONIINAE: CREMASTOCHEILINI: OPLOSTOMINA.

Type species: *Cetoniafuliginea* G.-A. Olivier, 1789, by monotypy.

Family-group name: OPLOSTOMINA Krikken, 1984. Stem: *Oplostom*-.

***Opresus* Casey, 1897**: 493, 494. Current status: junior synonym of *Microscydmus* Saulcy & Croissandeau, 1893 in STAPHYLINIDAE: SCYDMAENINAE: SCYDMAENITAE: STENICHNINI.

Type species: *Scydmaenusmisellus* J.L. LeConte, 1852, by subsequent designation (H. Franz 1985: 175).

Family-group name: OPRESINI Casey, 1897. Stem: *Opres*-.

***Opsimus* Mannerheim, 1843**: 305. Current status: valid genus in CERAMBYCIDAE: CERAMBYCINAE: OPSIMINI.

Type species: *Opsimusquadrilineatus* Mannerheim, 1843, by monotypy.

Family-group name: OPSIMI J.L. LeConte, 1873. Stem: *Opsim*-.

***Optatus* Pascoe, 1889**: 322, 327. Current status: valid genus in CURCULIONIDAE: BARIDINAE: OPTATINI.

Type species: *Centrinuspalmaris* Pascoe, 1889, by original designation.

Family-group name: OPTATIDES Champion, 1907. Stem: *Optat*-.

***Orchesia* Latreille, 1806**: 194. Current status: valid genus in MELANDRYIDAE: MELANDRYINAE: ORCHESIINI.

Type species: *Hallomenusmicans* Panzer, 1793, by monotypy.

Family-group name: ORCHÉSIENS Mulsant, 1856. Stem: *Orchesi*-.

***Orchestes* Illiger, 1798**: 498. Current status: valid genus in CURCULIONIDAE: CURCULIONINAE: RHAMPHINI: RHAMPHINA.

Type species: *Orchestessignifer* Creutzer, 1799 (= *Curculiohortorum* Fabricius, 1792), by subsequent monotypy (Creutzer 1799: 125).

Family-group name: ORCHESTIDES Latreille, 1828. Stem: *Orchest*-.

***Orchymontia* Broun, 1919**: 108. Current status: valid genus in HYDRAENIDAE: ORCHYMONTIINAE.

Type species: *Orchymontiaspinipennis* Broun, 1919, by monotypy.

Family-group name: ORCHYMONTINAE Perkins, 1997. Stem: *Orchymonti*-.

***Orectochilus* Dejean, 1833a**: 59. Current status: valid genus in GYRINIDAE: GYRININAE: ORECTOCHILINI.

Type species: *Gyrinusvillosus* O.F. Müller, 1776, by subsequent designation (Westwood 1838c: 8).

Family-group name: ORECTOCHILINI Kolbe, 1880. Stem: *Orectochil*-.

***Oreina* Chevrolat in Dejean, 1836a**: 402. Current status: valid genus in CHRYSOMELIDAE: CHRYSOMELINAE: CHRYSOMELINI.

Type species: *Chrysomelaspeciosa* Linnaeus, 1767, by subsequent designation (Chevrolat 1843a: 656).

Family-group name: OREININI Bechyné, 1958. Stem: *Orein*-. Note: junior homonym of OREININI Bleeker, 1863 (type genus *Oreinus* McClelland, 1839) in Pisces.

***Oreodera* Audinet-Serville, 1835a**: 19. Current status: valid genus in CERAMBYCIDAE: LAMIINAE: ACANTHODERINI.

Type species: *Cerambyxglaucus* Linnaeus, 1758, by subsequent designation (E. Desmarest 1860: 321).

Family-group name: OREODERITAE J. Thomson, 1860. Stem: *Oreoder*-.

***Oritocatops* Jeannel, 1921**: 235, 236. Current status: valid genus in LEIODIDAE: CHOLEVINAE: ORITOCATOPINI.

Type species: *Oritocatopskenyensis* Jeannel, 1921, by original designation.

Family-group name: ORITOCATOPINI Jeannel, 1936. Stem: *Oritocatop*-.

***Ormiscus* G.R. Waterhouse, 1845**: 37. Current status: valid genus in ANTHRIBIDAE: ANTHRIBINAE: ZYGAENODINI.

Type species: *Ormiscusvariegatus* G.R. Waterhouse, 1845, by monotypy.

Family-group name: HORMISCI J.L. LeConte, 1876. Stem: *Ormisc*-.

***Ornithognathus* J. Thomson, 1858b**: 215. Current status: valid genus in CHRYSOMELIDAE: GALERUCINAE: LUPERINI.

Type species: *Ornithognathusgenerosus* J. Thomson, 1858, by monotypy.

Family-group name: ORNITHOGNATHITES Chapuis, 1875. Stem: *Ornithognath*-.

***Orobitis* Germar, 1817b**: 340. Current status: valid genus in CURCULIONIDAE: OROBITIDINAE.

Type species: *Attelabusglobosus* Fabricius, 1792 (= *Curculiocyaneus* Linnaeus, 1758), by monotypy.

Family-group name: OROBITINA C.G. Thomson, 1859. Stem: *Orobitid*-.

***Orodotes* Bonvouloir, 1871**: 74. Current status: valid genus in EUCNEMIDAE: MACRAULACINAE: ORODOTINI. Note: this name was first used by Gemminger in Gemminger and von Harold (1869b: 1481) but is unavailable from that date since it was not accompanied by a description or indication (ICZN 1999a, Article 12.1).

Type species: *Orodotesjansoni* Bonvouloir, 1875, by subsequent monotypy (de Bonvouloir 1875: 717).

Family-group name: ORODOTINI Muona, 1993. Stem: *Orodot*-.

***Orophius* L. Redtenbacher, 1847c**: 350. Current status: junior synonym of *Octotemnus* Mellié, 1847 in CIIDAE: CIINAE: OROPHIINI.

Type species: *Cismandibularis* Gyllenhal, 1813, by monotypy.

Family-group name: OROPHIINA C.G. Thomson, 1863. Stem: *Orophi*-. Note: senior homonym of OROPHIINI Lvovsky, 1974 (type genus *Orophia* Hübner, 1825) in Lepidoptera; the lepidopteran name was recently replaced with EUTORNINI Lvovsky, 2019 but such action is contrary to Article 55.3.1 (ICZN 1999a), which requires that the case be referred to the Commission for a ruling to remove the homonymy.

†***Oropsis* Legalov & Kirejtshuk in Legalov et al., 2017**: 112. Current status: valid genus in NEMONYCHIDAE: †PALEOCARTINAE: †OROPSINI. Note: Lower Cretaceous (Lebanon).

Type species: †*Oropsismarinae* Legalov & Kirejtshuk, 2017, by original designation.

Family-group name: †OROPSINI Legalov & Kirejtshuk in Legalov et al., 2017: 112. Stem: *Orops*-.

***Orphilus* Erichson, 1846**: 424, 461. Current status: valid genus in DERMESTIDAE: ORPHILINAE.

Type species: *Anthrenusglabratus* Fabricius, 1801 (= *Byrrhusniger* Rossi, 1790), by subsequent designation (Lacordaire 1854: 473).

Family-group name: ORPHILI J.L. LeConte, 1861. Stem: *Orphil*-.

***Orphinus* Motschulsky, 1858a**: 48. Current status: valid genus in DERMESTIDAE: MEGATOMINAE: MEGATOMINI: ORPHININA.

Type species: *Orphinushaemorrhoidalis* Motschulsky, 1858, by subsequent designation (Arrow 1915: 437). Note: the spelling *haemorrhoidalis*, which is an incorrect subsequent spelling of the original spelling *haemorhoidalis*, is in prevailing usage, attributed to the publication of the original spelling, and therefore deemed to be the correct original spelling (ICZN 1999a, Article 33.3.1).

Family-group name: ORPHININA Zhou, Nicholls, Liu, Hartley, Szito, Ślipiński & Zwick, 2022: 18. Stem: *Orphin*-.

***Orphnus* W.S. MacLeay, 1819**: 119. Current status: valid genus in SCARABAEIDAE: ORPHNINAE: ORPHNINI.

Type species: *Geotrupesbicolor* Fabricius, 1801, by monotypy.

Family-group name: ORPHNIDAE Erichson, 1847. Stem: *Orphn*-.

***Orropygia* Raffray, 1910**: 419. Current status: valid genus in STAPHYLINIDAE: PSELAPHINAE: BATRISITAE: BATRISINI: BATRISINA.

Type species: *Orropygiamyrmecophila* Raffray, 1910, by monotypy.

Family-group name: OROPYGIINA Jeannel, 1949. Stem: *Orropygi*-.

***Orsodacne* Latreille, 1802b**: 223. Current status: valid genus in ORSODACNIDAE: ORSODACNINAE. Note: name placed on the Official List of Generic Names in Zoology (ICZN 2002a).

Type species: *Chrysomelacerasi* Linnaeus, 1758, by plenary power (see ICZN 2002a). Note: name placed on the Official List of Specific Names in Zoology (ICZN 2002a).

Family-group name: ORSODACHNIDAE C.G. Thomson, 1859. Stem: *Orsodacn*-.

***Ortalia* Mulsant, 1850**: 893. Current status: valid genus in COCCINELLIDAE: COCCINELLINAE: ORTALIINI.

Type species: *Ortaliavariata* Mulsant, 1850, by subsequent designation (Crotch 1874a: 275).

Family-group name: ORTALIENS Mulsant, 1850. Stem: *Ortali*-.

***Orthocerus* Latreille, 1797**: 16. Current status: valid genus in ZOPHERIDAE: COLYDIINAE: ORTHOCERINI. Note: name placed on the Official List of Generic Names in Zoology (ICZN 1995d, as *Orthocerus* Latreille, 1796).

Type species: *Hispamutica* Linnaeus, 1767 (= *Dermestesclavicornis* Linnaeus, 1758), by subsequent monotypy (Latreille 1802b: 173, as *Sarrotriummuticum* F.). Note: in Opinion 1811 (ICZN 1995d), the Commission listed the type species of *Orthocerus* as *Tenebriohirticornis* DeGeer, 1775 (= *Dermestesclavicornis* Linnaeus, 1758) by subsequent monotypy in Latreille (1807: 172); however, Latreille (1802b: 173) listed a different nominal species by name (also considered a junior synonym of *Dermestesclavicornis* Linnaeus, 1758) under *Orthocerus* in an earlier publication, hence the change of type species as reported here; the original spelling *Hispamuticus* Linnaeus, 1797 was corrected to *H.mutica* in the “Errata” section of the same work on page [37]; the valid name of the type species, *Dermestesclavicornis* Linnaeus, 1758, was already placed on the Official List of Specific Names in Zoology (ICZN 1995d).

Family-group name: ORTHOCÉRITES Blanchard, 1845. Stem: *Orthocer*-.

***Orthochaetes* Germar, 1823**: 302. Current status: valid genus in CURCULIONIDAE: CURCULIONINAE: STYPHLINI.

Type species: *Rhynchaenussetiger* Beck, 1817, by monotypy.

Family-group name: ORTHOCHAETINAE Joy, 1932. Stem: *Orthochaet*-.

***Orthognathus* Schönherr, 1838**: 812. Current status: valid genus in CURCULIONIDAE: DRYOPHTHORINAE: ORTHOGNATHINI.

Type species: *Orthognathuslividus* Gyllenhal, 1838, by original designation.

Family-group name: ORTHOGNATHIDES Lacordaire, 1865. Stem: *Orthognath*-.

***Orthogonius* W.S. MacLeay, 1825**: 26. Current status: valid genus in CARABIDAE: HARPALINAE: ORTHOGONIINI. Note: this name was also proposed in the same year by Dejean (1825: 169, 279 [by 5 September]) subsequently to W.S. MacLeay’s publication dated 1 June.

Type species: *Carabusduplicatus* Wiedemann, 1819, by subsequent designation (Hope 1838a: 85).

Family-group name: ORTHOGONIDEN Schaum, 1857. Stem: *Orthogoni*-.

***Orthomegas* Audinet-Serville, 1832**: 149. Current status: valid genus in CERAMBYCIDAE: PRIONINAE: CALLIPOGONINI.

Type species: *Cerambyxcorticinus* G.-A. Olivier, 1790 (= *Cerambyxcinnamomeus* Linnaeus, 1758), by subsequent designation (E. Desmarest 1860: 307).

Family-group name: ORTHOMEGITAE J. Thomson, 1864. Stem: *Orthomegal*-.

***Orthoperus* Stephens, 1829a**: 158. Current status: valid genus in CORYLOPHIDAE: CORYLOPHINAE: ORTHOPERINI.

Type species: *Dermestespunctum* Marsham, 1802, by subsequent designation (Westwood 1838c: 11). Note: no species were originally included in this genus by Stephens (1829a); the first ones were the six species listed by Stephens (1829a: 186, 187); Bowestead (2007: 634) listed the type species as a synonym of *Cryptophagusatomus* Gyllenhal, 1808 but mentioned that the assignment is doubtful.

Family-group name: ORTHOPÉRITES Jacquelin du Val, 1857. Stem: *Orthoper*-.

***Orthopleura* Spinola, 1845b**: 80. Current status: junior synonym of *Dermestoides* Schäffer, 1777 in CLERIDAE: KORYNETINAE. Note: three original spellings were used, *Orthoplevra* (pp. 80–83, 151, 167), *Orthopleura* (pl. xxxii), and *Orthopleuva* (pl. xlii); as far as we know, no one has acted as First Reviser (ICZN 1999a, Article 24.2.3) and therefore we **here select***Orthopleura* as the correct original spelling, i.e., the spelling in prevailing usage.

Type species: *Dermestessanguinicollis* Fabricius, 1787, by subsequent designation (Audinet- Serville 1845: 430).

Family-group name: ORTHOPLEURINAE Böving & Craighead, 1931. Stem: *Orthopleur*-.

***Orthorhinus* Schönherr, 1825**: 582. Current status: valid genus in CURCULIONIDAE: MOLYTINAE: PISSODINI: ORTHORHININA.

Type species: *Curculiocylindrirostris* Fabricius, 1775, by original designation.

Family-group name: ORTHORHINIDES Jekel, 1865. Stem: *Orthorhin*-.

***Orthosoma* Audinet-Serville, 1832**: 155. Current status: valid genus in CERAMBYCIDAE: PRIONINAE: PRIONINI.

Type species: *Prionuscylindricus* Fabricius, 1775 (= *Cerambyxbrunneus* Forster, 1771 [as *brunnus*]), by monotypy.

Family-group name: ORTHOSOMITAE J. Thomson, 1864. Stem: *Orthosomat*-.

***Orvoenia* Dajoz, 1980b**: 190. Current status: junior synonym of *Megauchenia* W.S. MacLeay, 1825 in NITIDULIDAE: NITIDULINAE: NITIDULINI.

Type species: *Orvoeniaborneensis* Dajoz, 1980, by original designation.

Family-group name: ORVOENINI Dajoz, 1980. Stem: *Orvoeni*-.

***Orychodes* Pascoe, 1862**: 389. Current status: valid genus in BRENTIDAE: BRENTINAE: BRENTINI: ARRENODINA.

Type species: *Brentusserrirostris* Lund, 1802, by subsequent designation (Lacordaire 1865: 433).

Family-group name: ORYCHODI Senna, 1895. Stem: *Orychod*-.

***Oryctes* Hellwig, 1798**: 100. Current status: valid genus in SCARABAEIDAE: DYNASTINAE: ORYCTINI. Note: see Alonso-Zarazaga and Krell (2011: 68) for comment regarding the authorship of this name.

Type species: *Scarabaeusnasicornis* Linnaeus, 1758, by subsequent designation (Latreille 1810: 428).

Family-group name: ORYCTÉSAIRES Mulsant, 1842. Stem: *Oryct*-.

***Oryctoderus* Boisduval, 1835**: 160. Current status: valid genus in SCARABAEIDAE: DYNASTINAE: ORYCTODERINI.

Type species: *Oryctoderuslatitarsis* Boisduval, 1835, by monotypy.

Family-group name: ORYCTODERINI Endrödi, 1966. Stem: *Oryctoder*-.

***Oryctomorphus* Guérin-Méneville, 1831a**: pl. 3. Current status: valid genus in SCARABAEIDAE: RUTELINAE: RUTELINI: ORYCTOMORPHINA.

Type species: *Oryctomorphusbimaculatus* Guérin-Méneville, 1831, by monotypy.

Family-group name: ORYCTOMORPHIDAE Burmeister, 1847. Stem: *Oryctomorph*-.

***Oryotus* L. Miller, 1856**: 627. Current status: valid genus in LEIODIDAE: CHOLEVINAE: LEPTODIRINI: BATHYSCIINA.

Type species: *Oryotusschmidtii* L. Miller, 1856, by monotypy.

Family-group name: ORIOTINI Reitter, 1889. Stem: *Oryot*-.

***Oryssomus* Mulsant, 1850**: 939. Current status: valid genus in COCCINELLIDAE: COCCINELLINAE: COCCIDULINI.

Type species: *Oryssomussubterminatus* Mulsant, 1850, by monotypy.

Family-group name: ORYSSOMINI Gordon, 1974. Stem: *Oryssom*-.

***Osmoderma* Lepeletier & Audinet-Serville, 1828**: 702. Current status: valid genus in SCARABAEIDAE: CETONIINAE: OSMODERMATINI. Note: name placed on the Official List of Generic Names in Zoology (ICZN 2007).

Type species: *Scarabaeuseremita* Scopoli, 1763, by subsequent designation (W.S. MacLeay 1838: 16; see ICZN 2007). Note: name placed on the Official List of Specific Names in Zoology (ICZN 2007).

Family-group name: OSMODERMINI Schenkling, 1922. Stem: *Osmodermat*-.

***Osorius* Latreille, 1829a**: 438. Current status: valid genus in STAPHYLINIDAE: OSORIINAE: OSORIINI: OSORIINA.

Type species: *Osoriusbrasiliensis* Guérin-Méneville, 1829, by subsequent monotypy (Guérin- Méneville 1829a: pl. 9).

Family-group name: OSORINI Erichson, 1839. Stem: *Osori*-.

***Osphryon* Pascoe, 1969b**: 662. Current status: valid genus in CERAMBYCIDAE: PRIONINAE: OSPHRYONINI.

Type species: *Osphryonadustus* Pascoe, 1869, by monotypy.

Family-group name: OSPHRYONINI Jin, De Keyzer, Ashman, Zwick & Ślipiński, 2023: 507. Stem: *Osphryon*-.

***Osphya* Illiger, 1807b**: 370. Current status: valid genus in MELANDRYIDAE: OSPHYINAE. Note: in Volume 6 of ‘Magazin für Insektenkunde’, the name *Pelecina* was used by Illiger (1807a: 300) and Illiger (1807b: 333) but the unnecessary new replacement name *Osphya* was proposed instead in the ‘Verbesserung’ on page 370 by Illiger (1807c); both *Pelecina* and *Osphya* have the same publication date; Mulsant (1856b: 108–109) acted as First Reviser (ICZN 1999a, Article 24.2.2) when he used *Osphya* as valid.

Type species: *Cantharisbipunctata* Fabricius, 1775, by monotypy.

Family-group name: OSPHYENS Mulsant, 1856. Stem: *Osphy*-.

***Osphyoplesius* A. Winkler, 1915**: 331. Current status: valid genus in PYTHIDAE.

Type species: *Osphyoplesiusanophthalmus* A. Winkler, 1915, by monotypy.

Family-group name: OSPHYOPLESIINI A. Winkler, 1915. Stem: *Osphyoplesi*-.

***Ostoma* Laicharting, 1781**: 102. Current status: valid genus in PELTIDAE. Note: this name became a senior synonym of *Peltis*, which has incorrectly been credited to O.F. Müller (1764: 13) in the literature (e.g., Kolibáč 2007: 366; Kolibáč et al. 2016: 683) since the Commission (ICZN 1994a) ruled that *Peltis* Geoffroy, 1762 and all other uses of the name prior to the publication of *Peltis* Kugelann, 1792 are suppressed for the purposes of both the Principle of Priority and the Principle of Homonymy.

Type species: *Silphaferruginea* Linnaeus, 1758, by subsequent designation (Stephens 1830: 368).

Family-group name: OSTOMINI Harold, 1876. Stem: *Ostomat*-.

***Ostomopsis* H. Scott, 1922**: 250. Current status: valid genus in CERYLONIDAE: OSTOMOPSINAE.

Type species: *Ostomopsissolitaria* H. Scott, 1922, by monotypy.

Family-group name: OSTOMOPSINI Sen Gupta & Crowson, 1973. Stem: *Ostomops*-.

***Othippia* Pascoe, 1874**: 49. Current status: valid genus in CURCULIONIDAE: CONODERINAE: OTHIPPIINI.

Type species: *Othippiadistigma* Pascoe, 1874, by subsequent designation (Setliff 2007: 94).

Family-group name: OTHIPPIINI Morimoto, 1962. Stem: *Othippi*-.

***Othius* Stephens, 1829b**: 23. Current status: valid genus in STAPHYLINIDAE: STAPHYLININAE: OTHIINI. Note: name placed on the Official List of Generic Names in Zoology (ICZN 1983c).

Type species: *Staphylinuspunctulatus* Goeze, 1777, by plenary power (see ICZN 1983c). Note: name placed on the Official List of Specific Names in Zoology (ICZN 1983c); this species name was published in a work that is not consistently binominal (see ICZN 2021a).

Family-group name: OTHIIDES C.G. Thomson, 1859. Stem: *Othi*-.

***Othnius* J.L. LeConte, 1861**: 103. Current status: junior synonym of *Elacatis* Pascoe, 1860 in SALPINGIDAE: OTHNIINAE.

Type species: *Othniusumbrosus* J.L. LeConte, 1861, by subsequent designation (Packard 1869: 447).

Family-group name: OTHNIIDAE J.L. LeConte, 1861. Stem: *Othni*-.

***Otidocephalus* Chevrolat, 1832b**: 100. Current status: junior synonym of *Myrmex* Sturm, 1826 in CURCULIONIDAE: CURCULIONINAE: OTIDOCEPHALINI. Note: the original spelling *Otiocephalus* was intentionally changed to *Otidocephalus* (p. 442) in a subsequent part (issued in 1833) of the same volume of the journal and therefore the original spelling is to be corrected (ICZN 1999a, Article 32.5.1.1).

Type species: *Otidocephalusmexicanus* Chevrolat, 1832, by subsequent designation (Schönherr 1835: 364).

Family-group name: OTIDOCÉPHALIDES Lacordaire, 1863. Stem: *Otidocephal*-.

***Otiorhynchus* Germar, 1822b**: no 12. Current status: valid genus in CURCULIONIDAE: ENTIMINAE: OTIORHYNCHITAE: OTIORHYNCHINI. Note: this name was credited to “Germar, 1824” and placed on the Official List of Generic Names in Zoology in Opinion 982 (ICZN 1972) but the original work and date of publication for this name were corrected in Opinion 2299 (ICZN 2012c).

Type species: *Curculiorhacusensis* Germar, 1822, by monotypy (see ICZN 2012c, type fixation previously recorded as *Curculioclavipes* Bonsdorff, 1785, by subsequent designation in Schönherr 1826 in Opinion 982 (ICZN 1972)). Note: name placed on the Official List of Specific Names in Zoology (ICZN 2012c).

Family-group name: OTIORHYNCHIDES Schönherr, 1826. Stem: *Otiorhynch*-.

***Ototreta* E. Olivier, 1900**: 237. Current status: junior synonym of *Drilaster* Kiesenwetter, 1879 in LAMPYRIDAE: OTOTRETINAE.

Type species: *Ototretafornicata* E. Olivier, 1900, by subsequent designation (R. Lucas 1920: 468). Note: the type species often listed for this genus-group name is *Ototretaweyersi* E. Olivier, 1900 (e.g., Geisthardt and Satô 2007: 233) by subsequent designation of McDermott (1964: 47) but R. Lucas’ designation is earlier; both species are currently included in the genus *Drilaster* Kiesenwetter, 1879.

Family-group name: OTOTRETINAE McDermott, 1964. Stem: *Ototret*-.

***Ototretadrilus* Pic, 1921**: 13. Current status: valid genus in LAMPYRIDAE: OTOTRETINAE.

Type species: *Ototretadrilusatritarsis* Pic, 1921, by subsequent designation (N.D. Riley 1923: 124). Note: the same type species was designated again by Janisova and Bocakova (2013: 6).

Family-group name: OTOTRETADRILINAE Crowson, 1972. Stem: *Ototretadril*-.

***Ottistira* Pascoe, 1872**: 440. Current status: valid genus in CURCULIONIDAE: ENTIMINAE: POLYDRUSITAE: OTTISTIRINI.

Type species: *Ottistirabispinosa* Pascoe, 1872, by subsequent designation (Heller 1925a: 55).

Family-group name: OTTISTIRINI Heller, 1925. Stem: *Ottistir*-.

***Ovosoma* Motschulsky, 1860b**: 214. Current status: valid genus in CHRYSOMELIDAE: CHRYSOMELINAE: CHRYSOMELINI.

Type species: *Chrysomelavernalis* Brullé, 1832, by original designation.

Family-group name: OVOSOMES Motschulsky, 1860. Stem: *Ovosomat*-.

***Oxacis* J.L. LeConte, 1866a**: 164, 165. Current status: valid genus in OEDEMERIDAE: OEDEMERINAE: ASCLERINI.

Type species: *Ascleracana* J.L. LeConte, 1854, by subsequent designation (Arnett 1950: 223).

Family-group name: OXACIINI Arnett, 1984. Stem: *Oxacid*-.

***Oxeostomum* Gistel, 1856**: 374. Current status: junior synonym of *Apion* Herbst, 1797 in BRENTIDAE: APIONINAE: APIONITAE: APIONINI: APIONINA.

Type species: *Curculiofrumentarius* Linnaeus, 1758, by subsequent designation (Alonso- Zarazaga and Lyal 1999: 21, 57).

Family-group name: OXEOSTOMATIDAE Gistel, 1856. Stem: *Oxeostom*-.

***Oxura* W. Kirby, 1819**: 413. Current status: valid genus in TENEBRIONIDAE: PIMELIINAE: SEPIDIINI: OXURINA.

Type species: *Oxurasetosa* W. Kirby, 1819, by monotypy.

Family-group name: OXURINA C. Koch, 1955. Stem: *Oxur*-.

***Oxycheila* Dejean, 1825**: 4, 15. Current status: valid genus in CICINDELIDAE: OXYCHEILINI.

Type species: *Cicindelatristis* Fabricius, 1775, by subsequent designation (Hope 1838a: 15).

Family-group name: OXYCHEILITES J. Thomson, 1857. Stem: *Oxycheil*-.

***Oxycoleus* Lacordaire, 1868**: 484. Current status: valid genus in CERAMBYCIDAE: CERAMBYCINAE: OXYCOLEINI.

Type species: *Oxycoleusclavipes* Lacordaire, 1868, by monotypy.

Family-group name: OXYCOLEINI Martins & Galileo, 2003. Stem: *Oxycole*-.

***Oxycopis* Arnett, 1951**: 305. Current status: valid genus in OEDEMERIDAE: OEDEMERINAE: ASCLERINI.

Type species: *Necydalisnotoxoides* Fabricius, 1801, by original designation.

Family-group name: OXYCOPIINI Arnett, 1984. Stem: *Oxycopid*-.

†***Oxycorynoides* Arnoldi, 1977**: 159. Current status: valid genus in NEMONYCHIDAE: †EOBELINAE: †OXYCORYNOIDINI. Note: Upper Jurassic (Kazakhstan).

Type species: †*Oxycorynoidessimilis* Arnoldi, 1977, by original designation.

Family-group name: †OXYCORYNOIDINAE Arnoldi, 1977. Stem: *Oxycorynoid*-.

***Oxycorynus* Chevrolat, 1832c**: 212. Current status: valid genus in BELIDAE: OXYCORYNINAE: OXYCORYNITAE: OXYCORYNINI.

Type species: *Oxycorynusmelanocerus* Chevrolat, 1832, by subsequent designation (Schönherr 1840a: 582).

Family-group name: OXYCORYNIDES Schönherr, 1840. Stem: *Oxycoryn*-.

***Oxycraspedus* Kuschel, 1955**: 309. Current status: valid genus in BELIDAE: OXYCORYNINAE: OXYCORYNITAE: OXYCRASPEDINI.

Type species: *Oxycorynusminutus* R.A. Philippi & F. Philippi, 1864, by original designation.

Family-group name: OXYCRASPEDINA Marvaldi & Oberprieler, 2006. Stem: *Oxycrasped*-.

***Oxygona* Chevrolat, 1847a**: 368. Current status: junior synonym of *Platiprosopus* Chevrolat, 1834 in CHRYSOMELIDAE: GALERUCINAE: ALTICINI.

Type species: *Platiprosopusacutangulus* Chevrolat, 1834, by monotypy.

Family-group name: OXYGONITES Chapuis, 1875. Stem: *Oxygon*-.

***Oxylobus* Chaudoir, 1855**: 4, 5. Current status: valid genus in CARABIDAE: SCARITINAE: SCARITINI: OXYLOBINA.

Type species: *Scariteslateralis* Dejean, 1825, by subsequent designation (Motschulsky 1857b: 96).

Family-group name: OXYLOBIDES Andrewes, 1929. Stem: *Oxylob*-.

***Oxymirus* Mulsant, 1863**: 464. Current status: valid genus in CERAMBYCIDAE: LEPTURINAE: OXYMIRINI.

Type species: *Cerambyxcursor* Linnaeus, 1758, by monotypy.

Family-group name: OXYMIRINI Danilevsky, 1997. Stem: *Oxymir*-.

***Oxynopterus* Hope, 1842c**: 77. Current status: valid genus in ELATERIDAE: DENDROMETRINAE: OXYNOPTERINI.

Type species: *Elatermucronatus* G.-A. Olivier, 1792, by subsequent designation (Lacordaire 1857: 160).

Family-group name: OXYNOPTÉRIDES Candèze, 1857. Stem: *Oxynopter*-.

***Oxyonyx* Faust, 1885**: 192. Current status: valid genus in CURCULIONIDAE: CEUTORHYNCHINAE: CEUTORHYNCHINI.

Type species: *Coeliodesbrisouti* Faust, 1885, by subsequent designation (Hustache 1931: 246).

Family-group name: OXYONYXINA Hoffmann, 1957. Stem: *Oxyonych*-.

***Oxypeltus* Blanchard, 1851**: 459. Current status: valid genus in OXYPELTIDAE.

Type species: *Oxypeltusquadrispinosus* Blanchard, 1851, by monotypy.

Family-group name: OXYPELTIDES Lacordaire, 1868. Stem: *Oxypelt*-.

***Oxypoda* Mannerheim, 1830**: 69. Current status: valid genus in STAPHYLINIDAE: ALEOCHARINAE: OXYPODINI: OXYPODINA. Note: name placed on the Official List of Generic Names in Zoology (ICZN 1957b, as *Oxypoda* Mannerheim, 1831).

Type species: *Oxypodaspectabilis* Märkel, 1844, by plenary power (see ICZN 1957b). Note: name placed on the Official List of Specific Names in Zoology (ICZN 1957b).

Family-group name: OXYPODIDES C.G. Thomson, 1859. Stem: *Oxypod*-.

***Oxypodinus* Bernhauer, 1902**: 174. Current status: valid genus in STAPHYLINIDAE: ALEOCHARINAE: OXYPODININI.

Type species: *Oxypodinusanxius* Bernhauer, 1902, by subsequent designation (Fenyes 1919: 24).

Family-group name: OXYPODININI Fenyes, 1919. Stem: *Oxypodin*-.

***Oxyporus* Fabricius, 1775**: 267. Current status: valid genus in STAPHYLINIDAE: OXYPORINAE.

Type species: *Staphylinusrufus* Linnaeus, 1758, by subsequent designation (Latreille 1810: 427).

Family-group name: OXYPORIDAE Fleming, 1821. Stem: *Oxypor*-.

***Oxyrhynchus* Schönherr, 1823**: 1137. Current status: invalid senior synonym of *Cryptoderma* Ritsema, 1885 in CURCULIONIDAE: DRYOPHTHORINAE: CRYPTODERMATINI. Note: junior homonym of *Oxyrhynchus* Leach, 1818 [Pisces].

Type species: *Calandradiscors* Fabricius, 1801, by original designation.

Family-group name: OXYRHYNCHIDES Schönherr, 1823. Stem: *Oxyrhynch*-. Note: family-group name permanently invalid (ICZN 1999a, Article 39).

***Oxystoma* Duméril, 1805**: 226. Current status: valid genus in BRENTIDAE: APIONINAE: APIONITAE: APIONINI: TOXORHYNCHINA.

Type species: *Attelabuspomonae* Fabricius, 1798, by subsequent designation (Schilsky 1901: 38G).

Family-group name: OXYSTOMATINI Alonso-Zarazaga, 1990. Stem: *Oxystomat*-.

***Oxystomus* Dejean, 1825**: 410. Current status: invalid senior synonym of *Forcipator* Maindron, 1904 in CARABIDAE: SCARITINAE: FORCIPATORINI. Note: junior homonym of *Oxystomus* Fischer, 1803 [Mammalia].

Type species: *Oxystomuscylindricus* Dejean, 1825, by monotypy.

Family-group name: OXYSTOMINA Csiki, 1927. Stem: *Oxystom*-. Note: family-group name permanently invalid (ICZN 1999a, Article 39).

***Oxytelus* Gravenhorst, 1802**: 101. Current status: valid genus in STAPHYLINIDAE: OXYTELINAE: OXYTELINI.

Type species: *Staphylinuspiceus* Linnaeus, 1767, by subsequent designation (Latreille 1810: 427).

Family-group name: OXYTELIDAE Fleming, 1821. Stem: *Oxytel*-.

***Ozaena* G.-A. Olivier, 1812**: 619. Current status: valid genus in CARABIDAE: PAUSSINAE: OZAENINI.

Type species: *Ozaenadentipes* G.-A. Olivier, 1812, by monotypy.

Family-group name: OZAENIDAE Hope, 1838. Stem: *Ozaen*-.

***Ozodecerus* Chevrolat, 1839**: 175. Current status: valid genus in BRENTIDAE: BRENTINAE: BRENTINI: ITHYSTENINA.

Type species: *Ozodecerusforficulatus* Chevrolat, 1839, by subsequent designation (Kleine 1939: 130).

Family-group name: OZODOCERINI Jakobson, 1911. Stem: *Ozodecer*-.

***Ozotomerus* Perroud, 1853**: 406. Current status: valid genus in ANTHRIBIDAE: ANTHRIBINAE: OZOTOMERINI.

Type species: *Ozotomerusmaculosus* Perroud, 1853, by monotypy.

Family-group name: OZOTOMERINI Morimoto, 1972. Stem: *Ozotomer*-.

***Pachnaeus* Schönherr, 1826**: 121. Current status: valid genus in CURCULIONIDAE: ENTIMINAE: POLYDRUSITAE: TANYMECINI: TANYMECINA. Note: unnecessary new replacement name for *Docorhinus* Schönherr, 1823; Reily and N.M. Franz (2019) submitted an application to the Commission to suppress the older name *Docorhinus* Schönherr, 1823 and to place *Pachnaeus* Schönherr, 1826 on the Official List of Generic Names in Zoology.

Type species: *Curculioopalus* G.-A. Olivier, 1807, by original designation (type fixation for *Docorhinus* Schönherr).

Family-group name: PACHNAEI J.L. LeConte, 1874. Stem: *Pachnae*-.

***Pachnoda* Burmeister, 1842**: 511. Current status: valid genus in SCARABAEIDAE: CETONIINAE: CETONIINI: CETONIINA.

Type species: *Scarabaeusmarginatus* Drury, 1773, by subsequent designation (Rigout 1989: 15).

Family-group name: PACHNODII Péringuey, 1907. Stem: *Pachnod*-. Note: senior homonym of PACHNODIDAE Steenberg, 1925 (type genus *Pachnodus* Martens, 1860) in Mollusca.

***Pacholenus* Schönherr, 1826**: 216. Current status: valid genus in CURCULIONIDAE: MOLYTINAE: PACHOLENINI.

Type species: *Pacholenuspelliceus* Boheman, 1835, by subsequent designation (Vanin and Reichardt 1976: 164). Note: no available species was originally included; the first ones are the two species listed by Schönherr (1835: 101, 102).

Family-group name: PACHOLÉNIDES Lacordaire, 1863. Stem: *Pacholen*-.

***Pachybrachis* Chevrolat in Dejean, 1836a**: 420. Current status: valid genus in CHRYSOMELIDAE: CRYPTOCEPHALINAE: PACHYBRACHINI.

Type species: *Cryptocephalushieroglyphicus* Laicharting, 1781, by subsequent designation (Jacoby 1908: 265).

Family-group name: PACHYBRACHITES Chapuis, 1874. Stem: *Pachybrach*-.

***Pachycarus* Solier, 1835a**: 660, 666. Current status: valid genus in CARABIDAE: HARPALINAE: HARPALINI: DITOMINA.

Type species: *Pachycaruslatreillei* Solier, 1835, by subsequent designation (E. Desmarest 1851: 106).

Family-group name: PACHYCARINA Stichel, 1923. Stem: *Pachycar*-.

***Pachycera* Eschscholtz, 1831**: 5, 7. Current status: junior synonym of *Hyperops* Eschscholtz, 1831 in TENEBRIONIDAE: PIMELIINAE: TENTYRIINI. Note: junior homonym of *Pachycera* Billberg, 1820 [Hemiptera].

Type species: fixed by Bouchard et al. (2021: 282) according to Article 70.3.2 (ICZN 1999a) as *Tenebriobuprestoides* Fabricius, 1781, the taxonomic species involved in the misidentifation of *Akislaevigata* Fabricius, 1801 (sensu Eschscholtz, 1831), as designated originally by monotypy by Eschscholtz (1831).

Family-group name: PACHYCERINA Skopin, 1979. Stem: *Pachycer*-. Note: family-group name permanently invalid (ICZN 1999a, Article 39).

***Pachycnema* Lepeletier & Audinet-Serville, 1828**: 375. Current status: valid genus in SCARABAEIDAE: MELOLONTHINAE: HOPLIINI: LEPITRICHINA. Note: the older name *Pachypus* Billberg, 1820, a senior objective synonym of this genus-group name, is a nomen oblitum (see Bouchard et al. 2011: 858, Cupello and Ribeiro-Costa 2019: 140).

Type species: *Melolonthacrassipes* Fabricius, 1775, by subsequent designation (Blanchard in Audouin et al. 1843: pl. 44).

Family-group name: PACHYCNÉMIDES Laporte, 1840. Stem: *Pachycnem*-.

***Pachydema* Laporte, 1832b**: pl. 37. Current status: valid genus in SCARABAEIDAE: MELOLONTHINAE: TANYPROCTINI: TANYPROCTINA.

Type species: *Pachydemanigricans* Laporte, 1832 (= *Melolonthahirticollis* Fabricius, 1787), by monotypy.

Family-group name: PACHYDEMIDAE Burmeister, 1855. Stem: *Pachydem*-. Note: nomen protectum (see A.B.T. Smith 2006: 168).

***Pachyderes* Guérin-Méneville, 1829a**: pl. 12. Current status: valid genus in ELATERIDAE: AGRYPNINAE: OOPHORINI.

Type species: *Pachyderesruficollis* Guérin-Méneville, 1829, by monotypy.

Family-group name: PACHYDERINAE Fleutiaux, 1919. Stem: *Pachyder*-.

***Pachydrus* Sharp, 1882b**: 336, 338. Current status: valid genus in DYTISCIDAE: HYDROPORINAE: PACHYDRINI.

Type species: *Pachydrusobesus* Sharp, 1882, by subsequent designation (Guignot 1946: 113).

Family-group name: PACHYDRINI F.N. Young, 1980. Stem: *Pachydr*-.

***Pachygastrodes* Leleup, 1969**: 284. Current status: valid genus in STAPHYLINIDAE: PSELAPHINAE: PSELAPHITAE: PACHYGASTRODINI.

Type species: *Pachygastrodesminimipennis* Leleup, 1969, by monotypy.

Family-group name: PACHYGASTRODINI Leleup, 1969. Stem: *Pachygastrod*-.

***Pachyglossa* Fauvel, 1868**: 379. Current status: invalid senior synonym of *Pagla* Blackwelder, 1952 in STAPHYLINIDAE: ALEOCHARINAE: PAGLINI. Note: new replacement name for *Euryglossa* Fauvel, 1866; junior homonym of *Pachyglossa* Hodgson, 1843 [Aves].

Type species: *Hoplandriaanthracina* Fairmaire & Germain, 1861, by subsequent designation (Fenyes 1919: 24) (type fixation for *Euryglossa* Fauvel).

Family-group name: PACHYGLOSSINI Fenyes, 1919. Stem: *Pachygloss*-. Note: family-group name permanently invalid (ICZN 1999a, Article 39).

***Pachymerus* Thunberg, 1805**: 282. Current status: valid genus in CHRYSOMELIDAE: BRUCHINAE: PACHYMERINI: PACHYMERINA.

Type species: *Dermestesbactris* Linnaeus, 1763, by monotypy.

Family-group name: PACHYMERINAE Bridwell, 1929. Stem: *Pachymer*-.

***Pachyochthes* Reitter, 1897**: 248. Current status: junior synonym of *Hypodacne* J.L. LeConte, 1876 in EUXESTIDAE.

Type species: *Pachyochthesedithae* Reitter, 1897, by monotypy.

Family-group name: PACHYOCHTHESINAE Reitter, 1911. Stem: *Pachyochth*-.

***Pachypeza* Audinet-Serville, 1835a**: 42. Current status: valid genus in CERAMBYCIDAE: LAMIINAE: AGAPANTHIINI.

Type species: *Saperdapennicornis* Germar, 1823, by monotypy.

Family-group name: PACHYPÉZIDES Lacordaire, 1872. Stem: *Pachypez*-.

***Pachyplacus* John, 1935**: 26. Current status: valid genus in DISCOLOMATIDAE: NOTIOPHYGINAE: PACHYPLACINI.

Type species: *Pachyplacuspostpressus* John, 1935, by monotypy.

Family-group name: PACHYPLACINI John, 1954. Stem: *Pachyplac*-.

***Pachyplectrus* J.L. LeConte, 1874b**: 53. Current status: valid genus in HYBOSORIDAE: PACHYPLECTRINAE.

Type species: *Pachyplectruslaevis* J.L. LeConte, 1874, by monotypy.

Family-group name: PACHYPLECTRINAE Ocampo, 2006. Stem: *Pachyplectr*-.

***Pachypterus* P.H. Lucas, 1847a**: pl. 29. Current status: invalid senior synonym of *Neopachypterus* Bouchard, Löbl & Merkl, 2007 in TENEBRIONIDAE: BLAPTINAE: OPATRINI: NEOPACHYPTERINA. Note: junior homonym of *Pachypterus* Swainson, 1839 [Pisces].

Type species: *Pachypterusmauritanicus* P.H. Lucas, 1847, by monotypy.

Family-group name: PACHYPTÉRATES Mulsant & Rey, 1859. Stem: *Pachypter*-. Note: family- group name permanently invalid (ICZN 1999a, Article 39).

***Pachypus* Dejean, 1821**: 57. Current status: valid genus in SCARABAEIDAE: MELOLONTHINAE: PACHYPODINI. Note: nomen protectum (see Bouchard et al. 2011: 253).

Type species: *Scarabaeusexcavatus* Fabricius, 1792, by monotypy. Note: the type species has recently been treated as valid (i.e., not as a junior synonym of *Scarabaeuscandidae* Petagna, 1787) by Eberle et al. (2019: 455).

Family-group name: PACHYPODEN Erichson, 1840. Stem: *Pachypod*-.

***Pachyrhinadoretus* Ohaus, 1912a**: 509. Current status: valid genus in SCARABAEIDAE: RUTELINAE: ADORETINI: PACHYRHINADORETINA.

Type species: *Pachyrhinadoretusrugipennis* Ohaus, 1912, by subsequent designation (Arrow 1917: 285).

Family-group name: PACHYRHINADORETINA Ohaus, 1912. Stem: *Pachyrhinadoret*-.

***Pachyrhynchus* Germar, 1823**: 336. Current status: valid genus in CURCULIONIDAE: ENTIMINAE: POLYDRUSITAE: PACHYRHYNCHINI. Note: the senior homonym *Pachyrhynchus* Wagler, 1822 [Aves] was suppressed for purposes of both the Principle of Priority and Homonymy and *Pachyrhynchus* Germar, 1823 placed on the Official List of Generic Names in Zoology (ICZN 1970b, as *Pachyrhynchus* Germar, 1824).

Type species: *Pachyrhynchusmoniliferus* Germar, 1823, by monotypy (see ICZN 1970b). Note: name placed on the Official List of Specific Names in Zoology (ICZN 1970b, as *Pachyrhynchusmoniliferus* Germar, 1824).

Family-group name: PACHYRHYNCHIDES Schönherr, 1826. Stem: *Pachyrhynch*-.

***Pachyrhynchus* W. Kirby, 1837**: 203. Current status: junior synonym of *Ithycerus* Schönherr, 1823 in BRENTIDAE: ITHYCERINAE: ITHYCERINI. Note: junior homonym of *Pachyrhynchus* Germar, 1823 [Coleoptera: CURCULIONIDAE].

Type species: *Pachyrhynchusschoenherri* W. Kirby, 1837 (= *Curculionoveboracensis* Forster, 1771), by monotypy.

Family-group name: PACHYRHINCHIDAE W. Kirby, 1837. Stem: *Pachyrhynch*-. Note: family- group name permanently invalid (ICZN 1999a, Article 39).

***Pachyschelus* Solier, 1833**: 313. Current status: valid genus in BUPRESTIDAE: AGRILINAE: TRACHEINI: PACHYSCHELINA.

Type species: *Pachyschelusscutellatus* Solier, 1833 (= *Trachysterminans* Fabricius, 1801), by monotypy.

Family-group name: PACHYSCHELINAE Böving & Craighead, 1931. Stem: *Pachyschel*-.

***Pachysoma* W.S. MacLeay, 1821**: 507. Current status: valid genus in SCARABAEIDAE: SCARABAEINAE: SCARABAEINI.

Type species: *Coprisaesculapius* G.-A. Olivier, 1790, by subsequent designation (Reiche 1841: 212).

Family-group name: PACHYSOMIDES M.C. Ferreira, 1953. Stem: *Pachysomat*-.

***Pachystola* Dejean, 1835**: 342. Current status: junior synonym of *Lamia* Fabricius, 1775 in CERAMBYCIDAE: LAMIINAE: LAMIINI.

Type species: *Cerambyxtextor* Linnaeus, 1758, by monotypy.

Family-group name: PACHYSTOLAEIDAE Gistel, 1848. Stem: *Pachystol*-.

***Pachyta* Dejean, 1821**: 112. Current status: valid genus in CERAMBYCIDAE: LEPTURINAE: RHAGIINI.

Type species: *Lepturaoctomaculata* Fabricius, 1792 (= *Lepturaquadrimaculata* Linnaeus, 1758), by subsequent designation (Westwood 1838c: 41).

Family-group name: PACHYTINI Portevin, 1934. Stem: *Pachyt*-.

***Pachyteles* Perty, 1830**: 3. Current status: valid genus in CARABIDAE: PAUSSINAE: OZAENINI.

Type species: *Pachytelesstriola* Perty, 1830, by subsequent designation (Hope 1838a: 99).

Family-group name: PACHYTELINI Jeannel, 1946. Stem: *Pachytel*-.

***Pachytrachelus* Chaudoir, 1852**: 85. Current status: valid genus in CARABIDAE: HARPALINAE: HARPALINI: STENOLOPHINA.

Type species: *Pachytracheluscribriceps* Chaudoir, 1852, by monotypy.

Family-group name: PACHYTRACHELI Csiki, 1932. Stem: *Pachytrachel*-.

***Pachytricha* Hope, 1841b**: 303. Current status: valid genus in SCARABAEIDAE: MELOLONTHINAE: PACHYTRICHINI.

Type species: *Pachytrichacastanea* Hope, 1841, by monotypy.

Family-group name: PACHYTRICHIADAE Burmeister, 1855. Stem: *Pachytrich*-.

***Pachyura* Hope, 1834a**: 102. Current status: valid genus in BELIDAE: BELINAE: PACHYURITAE: PACHYURINI.

Type species: *Pachyuraaustralis* Hope, 1834, by monotypy.

Family-group name: PACHYURINI Kuschel, 1959. Stem: *Pachyur*-.

***Pactola* Pascoe, 1876**: 57. Current status: valid genus in CURCULIONIDAE: CURCULIONINAE: EUGNOMINI: EUGNOMINA.

Type species: *Pactolavariabilis* Pascoe, 1876, by monotypy.

Family-group name: PACTOLINA Voss, 1936. Stem: *Pactol*-. Note: junior homonym of PACTOLIENS^2^ H. Milne-Edwards, 1837 (type genus *Pactolus* Leach, 1815) in Crustacea; H. Milne-Edwards’ family-group name is permanently invalid (ICZN 1999a, Article 39) since *Pactolus* Leach was suppressed under the plenary power for the purposes of the Principle of Priority (ICZN 1966a).

***Paederus* Fabricius, 1775**: 268. Current status: valid genus in STAPHYLINIDAE: PAEDERINAE: PAEDERINI: PAEDERINA.

Type species: *Staphylinusriparius* Linnaeus, 1758, by subsequent designation (Latreille 1810: 427).

Family-group name: POEDERIDAE Fleming, 1821. Stem: *Paeder*-.

***Paelobius* Schönherr, 1808**: 27. Current status: junior synonym of *Hygrobia* Latreille, 1804 in HYGROBIIDAE.

Type species: *Dytiscustardus* Herbst, 1779 (= *Dytiscushermanni* Fabricius, 1775), by monotypy.

Family-group name: PELOBIINAE Erichson, 1837. Stem: *Paelobi*-.

***Pagla* Blackwelder, 1952**: 287. Current status: valid genus in STAPHYLINIDAE: ALEOCHARINAE: PAGLINI. Note: new replacement name for *Pachyglossa* Fauvel, 1868.

Type species: *Hoplandriaanthracina* Fairmaire & Germain, 1861, by subsequent designation (Fenyes 1919: 22) (type fixation for *Euryglossa* Fauvel for which *Pachyglossa* Fauvel was a new replacement name).

Family-group name: PAGLINI Newton & Thayer, 1992. Stem: *Pagl*-.

***Pagria* Lefèvre, 1884**: 92. Current status: valid genus in CHRYSOMELIDAE: EUMOLPINAE: TYPOPHORINI.

Type species: *Pagriasuturalis* Lefèvre, 1884, by subsequent designation (Jacoby 1908: 356).

Family-group name: PAGRIITES Lefèvre, 1884. Stem: *Pagri*-.

***Pagurodactylus* Gorham, 1900**: 78. Current status: valid genus in MELYRIDAE: MALACHIINAE: PAGURODACTYLINI.

Type species: *Pagurodactylusvitticeps* Gorham, 1900, by monotypy.

Family-group name: PAGURODACTYLINAE Constantin, 2001. Stem: *Pagurodactyl*-.

***Paipalesomus* Schönherr, 1847**: 69. Current status: junior synonym of *Peribleptus* Schönherr, 1843 in CURCULIONIDAE: MOLYTINAE: PAIPALESOMINI.

Type species: *Paipalesomuspistriarius* Schönherr, 1847 (= *Alcidesdealbatus* Boisduval, 1835), by original designation.

Family-group name: PAIPALESOMINI G.A.K. Marshall, 1932. Stem: *Paipalesom*-.

***Palaechthus* C.O. Waterhouse, 1884**: 277. Current status: valid genus in CURCULIONIDAE: CYCLOMINAE: LISTRODERINI: PALAECHTHINA.

Type species: *Palaechthusglabratus* C.O. Waterhouse, 1884, by subsequent designation (Brinck 1948: 51).

Family-group name: PALAECHTINI Brinck, 1948. Stem: *Palaechth*-.

†***Palaeoanoplus* Legalov, 2021**: 66. Current status: valid genus in CURCULIONIDAE: CURCULIONINAE: †PALAEOANOPLINI. Note: upper Eocene (Baltic amber); combined description of a new family-group taxon and a single new nominal genus (ICZN 1999a, Article 13.5).

Type species: †*Palaeoanoplushorridus* Legalov, 2021, by original designation.

Family-group name: †PALAEOANOPLINI Legalov, 2021: 65. Stem: *Palaeoanopl*-.

†***Palaeoaxinidium* McKay, 1991**: 3. Current status: valid genus in CARABIDAE: SCARITINAE: †PALAEOAXINIDIINI. Note: Cretaceous (Botswana).

Type species: †*Palaeoaxinidiumorapensis* MacKay, 1991, by original designation.

Family-group name: †PALAEOAXINIDINI McKay, 1991. Stem: *Palaeoaxinidi*-.

†***Palaeocryptorhynchus* Poinar, 2009b**: 587. Current status: valid genus in CURCULIONIDAE: MOLYTINAE: †PALAEOCRYPTORHYNCHINI. Note: mid-Cretaceous (Myanmar); Poinar (2009b) used two different spellings, including *Palaeocryptorhynus* (p. 587); as far as we know no one has acted as First Reviser (ICZN 1999a, Article 24.2.3) and we **here select***Palaeocryptorhynchus*, the spelling in prevailing usage.

Type species: †*Palaeocryptorhynchusburmanus* Poinar, 2009, by original designation.

Family-group name: †PALEOCRYPTORHYNCHINI Poinar & Legalov in Legalov and Poinar, 2014: 562. Stem: *Palaeocryptorhynch*-.

†***Palaeogyrinus* Schlechtendal, 1894**: 201. Current status: valid genus in DYTISCIDAE: †PALAEOGYRININAE. Note: upper Oligocene (Germany).

Type species: †*Palaeogyrinusstrigatus* Schlechtendal, 1894, by monotypy.

Family-group name: †PALAEOGYRINIDAE Schlechtendal, 1894. Stem: *Palaeogyrin*-.

†***Palaeomallerus* Legalov, 2018b**: 75. Current status: valid genus in CURCULIONIDAE: CONODERINAE: †PALAEOMALLERINI. Note: lower Eocene (USA).

Type species: †*Palaeomalleruslongirostris* Legalov, 2018, by original designation.

Family-group name: †PALAEOMALLERINI Legalov, 2018b: 75. Stem: *Palaeomaller*-.

†***Palaeorhamphus* Legalov, 2017b**: 982. Current status: valid genus in CURCULIONIDAE: CURCULIONINAE: RHAMPHINI: †PALAEORHAMPHINA. Note: upper Eocene (Baltic amber).

Type species: †*Palaeorhamphusprimitivus* Legalov, 2017, by original designation.

Family-group name: †PALAEORHAMPHINA Legalov, 2017b: 981. Stem: *Palaeorhamph*-.

†***Palaeorhopalotria* Legalov, 2013b**: 64. Current status: valid genus in BELIDAE: OXYCORYNINAE: OXYCORYNITAE: †PALAEORHOPALOTRIINI. Note: upper Eocene (France).

Type species: †*Palaeorhopalotrianeli* Legalov, 2013, by original designation.

Family-group name: †PALAEORHOPALOTRIINI Legalov, 2013b: 63. Stem: *Palaeorhopalotri*-.

†***Palaeotanaos* Kirejtshuk, Legalov & Nel, 2016**: 1437. Current status: valid genus in BRENTIDAE: APIONINAE: TANAITAE: †PALAEOTANAINI. Note: lower Eocene (France).

Type species: †*Palaeotanaosoisensis* Kirejtshuk, Legalov & Nel, 2016, by original designation.

Family-group name: †PALAEOTANAINI Legalov, Kirejtshuk & Nel, 2020b: 734. Stem: *Palaeotana*-.

†***Palaeothroscus* Iablokoff-Khnzorian, 1962**: 83. Current status: junior synonym of *Trixagus* Kugelann, 1794 in THROSCIDAE. Note: upper Eocene (Baltic amber).

Type species: †*Palaeothroscussosnovskyi* Iablokoff-Khnzorian, 1962, by original designation.

Family-group name: †PALAEOTHROSCINI Iablokoff-Khnzorian, 1962. Stem: *Palaeothrosc*-.

†***Palaeotylus* Poinar, Vega & Legalov, 2018**: 138. Current status: valid genus in POLYPHAGA incertae sedis. Note: mid-Cretaceous (Myanmar).

Type species: †*Palaeotylusfemoralis* Poinar, Vega & Legalov, 2018, by original designation.

Family-group name: †PALAEOTYLINAE Poinar, Vega & Legalov, 2018: 2. Stem: *Palaeotyl*-.

***Palaeoxenus* G.H. Horn, 1891**: 40. Current status: valid genus in EUCNEMIDAE: PALAEOXENINAE.

Type species: *Cryptostomadohrni* G.H. Horn, 1878, by monotypy.

Family-group name: PALAEOXENINAE Muona, 1993. Stem: *Palaeoxen*-.

***Palaestra* Laporte, 1840b**: 251. Current status: valid genus in MELOIDAE: NEMOGNATHINAE: PALAESTRINI.

Type species: *Palaestrarubripennis* Laporte, 1840, by monotypy.

Family-group name: PALAESTRINI Bologna, Turco & Pinto, 2013: 394. Stem: *Palaestr*-.

†***Paleocartus* Legalov, 2003**: 78. Current status: valid genus in NEMONYCHIDAE: †PALEOCARTINAE: †PALEOCARTINI: †PALEOCARTINA. Note: Upper Jurassic (Kazakhstan).

Type species: †*Brenthorrhinoidespubescens* Gratshev & Zherikhin, 1996, by original designation.

Family-group name: †PALEOCARTINI Legalov, 2003. Stem: *Paleocart*-.

†***Pallichnus* Retallack, 1984**: 580. Current status: valid genus in †PALLICHNIDAE. Note: Ichnotaxon, Upper Cretaceous/Oligocene (Argentina; South Dakota, USA).

Type species: †*Pallichnusdakotensis* Retallack, 1984, by original designation.

Family-group name: †PALLICHNIDAE Genise, 2004. Stem: *Pallichn*-.

***Pallodes* Erichson, 1843a**: 348. Current status: valid genus in NITIDULIDAE: NITIDULINAE: NITIDULINI.

Type species: *Strongylusannulifer* Laporte, 1840, by subsequent designation (E. Desmarest 1851: 283).

Family-group name: PALLODINI Reitter, 1873: 98. Stem: *Pallod*-. Note: this name was overlooked in Bouchard et al. (2011), Bouchard and Bousquet (2020a).

***Palophagus* Kuschel in Kuschel and May, 1990**: 704. Current status: valid genus in MEGALOPODIDAE: PALOPHAGINAE: PALOPHAGINI.

Type species: *Palophagusbunyae* Kuschel, 1990, by original designation.

Family-group name: PALOPHAGINAE Kuschel & May, 1990. Stem: *Palophag*-.

***Palorus* Mulsant, 1854**: 250. Current status: valid genus in TENEBRIONIDAE: TENEBRIONINAE: PALORINI.

Type species: *Hypophlaeusdepressus* Fabricius, 1790, by monotypy.

Family-group name: PALORINAE E.G. Matthews, 2003. Stem: *Palor*-.

†***Palpattalus* Tshernyshev, 2017**: 955. Current status: valid genus in MELYRIDAE: MALACHIINAE: †PALPATTALINI. Note: Eocene (Kaliningrad region, Russia).

Type species: †*Palpattaluseocenicus* Tshernyshev, 2017, by original designation.

Family-group name: †PALPATTALINI Tshernyshev, 2020: 69. Stem: *Palpattal*-.

***Palpomodes* Koch, 1952**: 223. Current status: valid genus in TENEBRIONIDAE: PIMELIINAE: SEPIDIINI: PALPOMODINA.

Type species: *Psammodesphysopterus* Gebien, 1920, by monotypy.

Family-group name: PALPOMODINA Kamiński & Gearner in Kamiński et al., 2022: 645. Stem: *Palpomod*-.

***Pamborus* Latreille in G.-A. Olivier, 1812**: 677. Current status: valid genus in CARABIDAE: CARABINAE: PAMBORINI.

Type species: *Pamborusalternans* Latreille, 1812, by monotypy.

Family-group name: PAMBORIDAE Hope, 1838. Stem: *Pambor*-.

***Panagaeus* Latreille, 1802b**: 91. Current status: valid genus in CARABIDAE: HARPALINAE: PANAGAEINI: PANAGAEINA.

Type species: *Carabuscruxmajor* Linnaeus, 1758, by monotypy.

Family-group name: PANAGAEIDES Bonelli, 1810. Stem: *Panagae*-.

***Panaphantus* Kiesenwetter in Kraatz, 1858a**: 48. Current status: valid genus in STAPHYLINIDAE: PSELAPHINAE: EUPLECTITAE: TRICHONYCHINI: PANAPHANTINA.

Type species: *Panaphantusatomus* Kiesenwetter, 1858, by monotypy.

Family-group name: PANAPHANTINA Jeannel, 1950. Stem: *Panaphant*-.

***Pandeleteius* Schönherr, 1834a**: 129. Current status: valid genus in CURCULIONIDAE: ENTIMINAE: POLYDRUSITAE: TANYMECINI: PANDELETEIINA.

Type species: *Pandeleteiuspauperculus* Gyllenhal, 1834 (= *Curculiohilaris* Herbst, 1797), by original designation.

Family-group name: PANDELETEINI Pierce, 1913. Stem: *Pandeletei*-.

***Panelus* Lewis, 1895b**: 375. Current status: valid genus in SCARABAEIDAE: SCARABAEINAE: DELTOCHILINI.

Type species: *Temnoplectronparvulum* C.O. Waterhouse, 1874, by original designation.

Family-group name: PANELINI Arrow, 1931. Stem: *Panel*-.

***Pangaura* Gistel, 1856**: 366. Current status: junior synonym of *Lacon* Laporte, 1838 in ELATERIDAE: AGRYPNINAE: AGRYPNINI.

Type species: *Elatervarius* G.-A. Olivier, 1790 (= *Elaterquerceus* Herbst, 1784), by subsequent designation (Sánchez-Ruiz 1996: 44).

Family-group name: PANGAURADAE Gistel, 1856. Stem: *Pangaur*-. Note: nomen oblitum (see Bouchard et al. 2011: 307).

***Pangetes* Gistel, 1856**: 358. Current status: junior synonym of *Amara* Bonelli, 1810 in CARABIDAE: HARPALINAE: ZABRINI: AMARINA.

Type species: *Carabusovatus* Fabricius, 1792, by subsequent designation (Bousquet 2002: 37).

Family-group name: PANGETEIDAE Gistel, 1856. Stem: *Panget*-.

***Paniscus* Gistel, 1848a**: [5]. Current status: junior synonym of *Trichius* Fabricius, 1775 in SCARABAEIDAE: CETONIINAE: TRICHIINI. Note: junior homonym of *Paniscus* Schrank, 1802 [Hymenoptera].

Type species: *Scarabaeusfasciatus* Linnaeus, 1758, by subsequent designation (Bouchard et al. 2011: 271).

Family-group name: PANISCIDAE Gistel, 1848. Stem: *Panisc*-. Note: family-group name permanently invalid (ICZN 1999a, Article 39).

***Pantolia* Burmeister, 1842**: 567. Current status: valid genus in SCARABAEIDAE: CETONIINAE: STENOTARSIINI: PANTOLIINA.

Type species: *Cetoniaflavomarginata* Gory & Percheron, 1833, by subsequent designation (Sakai and Nagai 1998: 178).

Family-group name: PANTOLIINA Krikken, 1984. Stem: *Pantoli*-.

***Pantomorus* Schönherr, 1840a**: 942. Current status: valid genus in CURCULIONIDAE: ENTIMINAE: POLYDRUSITAE: NAUPACTINI: NAUPACTINA.

Type species: *Pantomorusalbosignatus* Schönherr, 1840, by original designation.

Family-group name: PANTOMORINA Voss, 1954. Stem: *Pantomor*-.

***Pantopoeus* Schönherr, 1842b**: 252. Current status: junior synonym of *Perperus* Schönherr, 1842 in CURCULIONIDAE: ENTIMINAE: ENTIMITAE: BYRSOPAGINI: STRANGALIODINA. Note: First Reviser (ICZN 1999a, Article 24.2.2) for *Perperus* Schönherr, 1842 versus *Pantopoeus* Schönherr, 1842 is Lea (1911: 76).

Type species: *Pantopoeuscervinus* Boheman, 1842, by original designation.

Family-group name: PANTOPÉIDES Lacordaire, 1863. Stem: *Pantopoe*-.

†***Pantostictus* Poinar & A.E. Brown, 2009b**: 39. Current status: valid genus in HISTERIDAE: ABRAEINAE: †PANTOSTICTINI. Note: mid-Cretaceous (Myanmar).

Type species: †*Pantostictusburmanicus* Poinar & A.E. Brown, 2009, by original designation.

Family-group name: †PANTOSTICTINI Zhou, Caterino & Ślipiński, 2020: 530. Stem: *Pantostict*-.

***Pantoteles* Schönherr, 1845**: 59. Current status: valid genus in CURCULIONIDAE: BARIDINAE: PANTOTELINI.

Type species: *Pantotelestenuirostris* Boheman, 1845, by original designation.

Family-group name: PANTOTÉLIDES Lacordaire, 1865. Stem: *Pantotel*-.

***Paoligena* Pic, 1928b**: 42. Current status: valid genus in TENEBRIONIDAE: TENEBRIONINAE: PAOLIGENINI.

Type species: *Praogenainhumeralis* Pic, 1928, by monotypy.

Family-group name: PAOLIGENINI Ferrer, 2013: 108. Stem: *Paoligen*-.

***Papusus* Casey, 1897**: 542. Current status: valid genus in STAPHYLINIDAE: SCYDMAENINAE: MASTIGITAE: PAPUSINI.

Type species: *Papususmacer* Casey, 1897, by monotypy.

Family-group name: PAPUSINI Jałoszyński & Brunke in Jałoszyński et al., 2018: 635. Stem: *Papus*-.

***Parablax* O. Schwarz, 1906**: 368. Current status: valid genus in ELATERIDAE: PITYOBIINAE: PARABLACINI.

Type species: *Metablaxtrisulcatus* O. Schwarz, 1903 (= *Parasaphesquinquesulcatus* Blackburn, 1900), by subsequent designation (Hyslop 1921: 661).

Family-group name: PARABLACINAE Kundrata, Gunter, H.B. Douglas & Bocák, 2016: 299. Stem: *Parablac*-.

***Paracatops* Portevin, 1907**: 69. Current status: valid genus in LEIODIDAE: CHOLEVINAE: ANEMADINI: PARACATOPINA.

Type species: *Cholevaantipoda* Kirsch, 1877, by monotypy.

Family-group name: PARACATOPINI Jeannel, 1936. Stem: *Paracatop*-.

***Parachilia* Burmeister, 1842**: 556. Current status: valid genus in SCARABAEIDAE: CETONIINAE: STENOTARSIINI: PARACHILIINA.

Type species [suggested]: *Parachiliamelanocala* Burmeister, 1842, by subsequent designation (Kraatz 1881: 72). Note: the valid type species fixation is that of Lacordaire (1855: 514), who selected *Cetoniabufo* Gory & Percheron, 1835, but that nominal species is the type species of the subgenus Chilamblys Kraatz, 1880, not of the nominotypical subgenus; an application to the Commission is necessary to fix *Parachiliamelanocala* Burmeister, 1842 as the type species of *Parachilia* Burmeister, 1842.

Family-group name: PARACHILIINA Krikken, 1984. Stem: *Parachili*-.

***Parachorius* Harold, 1873**: 103. Current status: valid genus in SCARABAEIDAE: SCARABAEINAE: PARACHORIINI.

Type species: *Parachoriusthomsoni* Harold, 1873, by monotypy.

Family-group name: PARACHORIINI Tarasov, 2017: 111. Stem: *Parachori*-.

***Paracucujus* Sen Gupta & Crowson, 1966**: 67. Current status: valid genus in BOGANIIDAE: PARACUCUJINAE.

Type species: *Paracucujusrostratus* Sen Gupta & Crowson, 1966, by original designation.

Family-group name: PARACUCUJINAE Endrödy-Younga & Crowson, 1986. Stem: *Paracucuj*-.

†***Paradermestes* Deng, Ślipiński, Ren & H. Pang, 2017b**: 110. Current status: valid genus in DERMESTIDAE: DERMESTINAE: †PARADERMESTINI. Note: Middle/Upper Jurassic (China).

Type species: †*Paradermestesjurassicus* Deng, Ślipiński, Ren & H. Pang, 2017, by original designation.

Family-group name: †PARADERMESTINI Deng, Ślipiński, Ren & H. Pang, 2017b: 110. Stem: *Paradermest*-.

***Paradoxenusa* Bruch, 1937**: 354. Current status: valid genus in STAPHYLINIDAE: ALEOCHARINAE: PARADOXENUSINI.

Type species: *Paradoxenusasilvestrii* Bruch, 1937, by original designation.

Family-group name: PARADOXENUSINI Bruch, 1937. Stem: *Paradoxenus*-.

***Paradrilus* Kiesenwetter, 1866**: 369. Current status: valid genus in ELATERIDAE: OMALISINAE.

Type species: *Paradrilusopacus* Kiesenwetter, 1866, by monotypy. Note: combined description of a new genus and a single new species (ICZN 1999a, Article 12.2.6).

Family-group name: PARADRILINAE Kundrata, Baena & Bocák, 2015: 417. Stem: *Paradril*-.

***Paraholopterus* Cerda & Cekalović, 1987**: 189. Current status: valid genus in CERAMBYCIDAE: CERAMBYCINAE: PARAHOLOPTERINI.

Type species: *Paraholopterusnahuelbutensis* Cerda & Cekalović, 1987, by original designation.

Family-group name: PARAHOLOPTERINI Martins, 1997. Stem: *Paraholopter*-.

†***Parahygrobia* Ponomarenko, 1977**: 20. Current status: valid genus in †PARAHYGROBIIDAE. Note: Upper Jurassic (Russia).

Type species: †*Parahygrobianatans* Ponomarenko, 1977, by original designation.

Family-group name: †PARAHYGROBIIDAE Ponomarenko, 1977. Stem: *Parahygrobi*-.

***Paraleptodema* Obenberger, 1936**: 106. Current status: junior synonym of *Cinyra* Laporte & Gory, 1837 in BUPRESTIDAE: CHRYSOCHROINAE: PARALEPTODEMINI: PARALEPTODEMINA.

Type species: *Paraleptodemastrandi* Obenberger, 1936, by monotypy.

Family-group name: PARALEPTODEMINI Cobos, 1975. Stem: *Paraleptodem*-.

***Paralispinus* Bernhauer, 1921**: 67. Current status: invalid senior synonym of *Clavilispinus* Bernhauer, 1926 in STAPHYLINIDAE: OSORIINAE: THORACOPHORINI: CLAVILISPININA. Note: replacement name for *Ancaeus* Fauvel, 1865; junior homonym of *Paralispinus* Eichelbaum, 1913 [Coleoptera: STAPHYLINIDAE].

Type species: *Ancaeusmegacephalus* Fauvel, 1865, by monotypy (type fixation for *Ancaeus* Fauvel).

Family-group name: PARALISPINI Blackwelder, 1942. Stem: *Paralispin*-. Note: family-group name permanently invalid (ICZN 1999a, Article 39).

***Parallelodemas* Faust, 1894a**: 306. Current status: valid genus in CURCULIONIDAE: BARIDINAE: BARIDINI: PARALLELODEMINA.

Type species: *Parallelodemasperfectum* Faust, 1894, by subsequent designation (Morimoto and Yoshihara 1996: 17).

Family-group name: PARALLELODEMINA Legalov, 2018e: 497. Stem: *Parallelodem*-.

†***Paralucanus* Nikolajev, 2000b**: S329. Current status: valid genus in LUCANIDAE: †PARALUCANINAE. Note: Upper Jurassic (Mongolia).

Type species: †*Paralucanusmesozoicus* Nikolajev, 2000, by original designation.

Family-group name: †PARALUCANINAE Nikolajev, 2000. Stem: *Paralucan*-.

***Paralycus* L.N. Medvedev & Kazantsev, 1992**: 55. Current status: junior synonym of *Lyropaeus* C.O. Waterhouse, 1878 in LYCIDAE: LYROPAEINAE: LYROPAEINI. Note: junior homonym of *Paralycus* Womersley, 1944 [Acarina].

Type species: *Paralycushelvius* L.N. Medvedev & Kazantsev, 1992, by monotypy.

Family-group name: PARALYCINAE L.N. Medvedev & Kazantsev, 1992. Stem: *Paralyc*-. Note: family-group name permanently invalid (ICZN 1999a, Article 39).

***Paramecolabus* Jekel, 1860**: 190. Current status: valid genus in ATTELABIDAE: ATTELABINAE: ATTELABINI: PARAMECOLABINA.

Type species: *Attelabusdiscolor* Fåhraeus, 1839, by original designation.

Family-group name: PARAMECOLABINA Legalov, 2003. Stem: *Paramecolab*-.

***Paramecosoma* Curtis, 1833b**: 186. Current status: valid genus in CRYPTOPHAGIDAE: CRYPTOPHAGINAE: CRYPTOPHAGINI.

Type species: *Paramecosomabicolor* Curtis, 1833 (= *Latridiusmelanocephalus* Herbst, 1793), by monotypy.

Family-group name: PARAMECOSOMINI Reitter, 1874. Stem: *Paramecosomat*-.

***Paramellon* C.O. Waterhouse, 1882**: iv. Current status: valid genus in TENEBRIONIDAE: PIMELIINAE: COSSYPHODINI: PARAMELLONINA.

Type species: *Paramellonsociale* C.O. Waterhouse, 1882, by monotypy.

Family-group name: PARAMELLONINAE Andreae, 1961. Stem: *Paramellon*-.

***Parandra* Latreille, 1802b**: 160. Current status: valid genus in CERAMBYCIDAE: PRIONINAE: PARANDRINI: PARANDRINA.

Type species: *Attelabusglaber* DeGeer, 1774, by monotypy.

Family-group name: PARANDRIDES Blanchard, 1845. Stem: *Parandr*-.

†***Parandrexis* Martynov, 1926**: 19. Current status: valid genus in †PARANDREXIDAE: †PARANDREXINAE. Note: Middle-Upper Jurassic (Kazakhstan; China).

Type species: †*Parandrexisparvula* Martynov, 1926, by original designation.

Family-group name: †PARANDREXIDAE Kirejtshuk, 1994. Stem: *Parandrex*-.

***Paraptochus* Seidlitz, 1868**: 3, 35. Current status: valid genus in CURCULIONIDAE: ENTIMINAE: CYPHICERITAE: PERITELINI: PERITELINA.

Type species: *Paraptochuscalifornicus* Seidlitz, 1868 (= *Peritelussellatus* Boheman, 1859), by monotypy.

Family-group name: PARAPTOCHI Pierce, 1913. Stem: *Paraptoch*-.

***Parastasia* Westwood, 1841d**: 204. Current status: valid genus in SCARABAEIDAE: RUTELINAE: RUTELINI: PARASTASIINA.

Type species: *Parastasiacanaliculata* Westwood, 1841, by monotypy.

Family-group name: PARASTASIIDAE Burmeister, 1844. Stem: *Parastasi*-.

***Parasthetops* Perkins & J. Balfour-Browne, 1994**: 37. Current status: valid genus in HYDRAENIDAE: PROSTHETOPINAE: PARASTHETOPINI.

Type species: *Parasthetopsnigritus* Perkins & J. Balfour-Browne, 1994, by original designation.

Family-group name: PARASTHETOPINI Perkins, 1994. Stem: *Parasthetop*-.

***Parasynaptopsis* Legalov, 2003**: 377. Current status: valid genus in ATTELABIDAE: ATTELABINAE: EUOPINI.

Type species: *Euopschinensis* Voss, 1922, by original designation.

Family-group name: PARASYNAPTOPSISINA Legalov, 2007. Stem: *Parasynaptopsis*- (ICZN 1999a, Article 29.4).

***Paratassa* Marseul, 1883**: 168. Current status: valid genus in BUPRESTIDAE: CHRYSOCHROINAE: PARATASSINI.

Type species: *Sphenopteracaroli* Marseul, 1883 (= *Sphenopteracoraebiformis* Fairmaire, 1875), by monotypy.

Family-group name: PARATASSINI Bílý & Volkovitsh, 1996. Stem: *Paratass*-.

†***Parathyrea* Alexeev, 1994**: 11. Current status: valid genus in BUPRESTIDAE: †PARATHYREINAE. Note: Upper Jurassic (Kazakhstan).

Type species: †*Parathyreajurassica* Alexeev, 1994, by original designation.

Family-group name: †PARATHYREINAE Alexeev, 1994. Stem: *Parathyre*-.

***Paratinus* Abeille de Perrin, 1891**: 220. Current status: junior synonym of *Apalochrus* Erichson, 1840 in MELYRIDAE: MALACHIINAE: MALACHIINI.

Type species: *Apalochrusfemoralis* Erichson, 1840, by subsequent designation (Evers 1987: 13).

Family-group name: PARATINI Portevin, 1931. Stem: *Paratin*-.

***Paratomapoderus* Voss, 1926**: 85. Current status: valid genus in ATTELABIDAE: APODERINAE: HOPLAPODERINI: PARATOMAPODERINA.

Type species: *Apoderusminiatus* Faust, 1882, by original designation.

Family-group name: PARATOMAPODERINA Legalov, 2003. Stem: *Paratomapoder*-.

***Paratrachys* Saunders, 1873**: 523. Current status: valid genus in BUPRESTIDAE: POLYCESTINAE: PARATRACHEINI.

Type species: *Paratrachyshederae* Saunders, 1873, by monotypy.

Family-group name: PARATRACHYSAE Cobos, 1980. Stem: *Paratrache*-.

***Parauletanus* Legalov, 2007**: 31. Current status: valid genus in ATTELABIDAE: RHYNCHITINAE: RHINOCARTITAE: PARAULETANINI.

Type species: *Auletanusdisparatus* Voss, 1922, by original designation.

Family-group name: PARAULETANINA Legalov, 2007. Stem: *Parauletan*-.

***Parecatus* Fairmaire, 1900**: 245. Current status: junior synonym of *Scotinesthes* Fairmaire, 1895 in TENEBRIONIDAE: PIMELIINAE: ASIDINI.

Type species: *Parecatusplicatulus* Fairmaire, 1900, by subsequent designation (Gebien 1937: 741).

Family-group name: PARECATINI Chatanay, 1914. Stem: *Parecat*-.

***Parhydraena* Orchymont, 1937**: 457. Current status: valid genus in HYDRAENIDAE: HYDRAENINAE: PARHYDRAENINI.

Type species: *Hydraenabrevipalpis* Régimbart, 1906, by original designation.

Family-group name: PARHYDRAENINI Perkins, 1997. Stem: *Parhydraen*-.

***Paristemia* Westwood, 1841c**: 124. Current status: valid genus in CERAMBYCIDAE: CERAMBYCINAE: TRACHYDERINI: TRACHYDERINA.

Type species: *Paristemiaplatyptera* Westwood, 1841, by monotypy.

Family-group name: PARISTÉMIIDES Lacordaire, 1868. Stem: *Paristemi*-.

***Parmena* Dejean, 1821**: 108. Current status: valid genus in CERAMBYCIDAE: LAMIINAE: PARMENINI.

Type species: *Cerambyxfasciatus* Villers, 1789 (= *Cerambyxbalteus* Linnaeus, 1767), by subsequent designation (Guérin-Méneville 1826: 187).

Family-group name: PARMÉNAIRES Mulsant, 1839. Stem: *Parmen*-.

***Parmulus* Gundlach in Poey, 1854**: 323, 336. Current status: junior synonym of *Clypastraea* Haldeman, 1842 in CORYLOPHIDAE: CORYLOPHINAE: PARMULINI. Note: new replacement name for *Clypeaster* Dejean, 1821.

Type species: *Cossyphuspusillus* Gyllenhal, 1810, by monotypy (type fixation for *Clypeaster* Dejean).

Family-group name: PARMULINI Poey, 1854. Stem: *Parmul*-.

***Parnus* Fabricius, 1792a**: 245. Current status: junior synonym of *Dryops* G.-A. Olivier, 1791 in DRYOPIDAE.

Type species: *Parnusprolifericornis* Fabricius, 1792 (= *Dermestesauriculatus* Geoffroy, 1785), by subsequent designation (Curtis 1825: pl. 80).

Family-group name: PARNIDEA Leach, 1817. Stem: *Parn*-.

***Paromalus* Erichson, 1834**: 167. Current status: valid genus in HISTERIDAE: DENDROPHILINAE: PAROMALINI.

Type species: *Histerflavicornis* Herbst, 1791, by subsequent designation (Westwood 1838c: 22).

Family-group name: PAROMALINI Reitter, 1909. Stem: *Paromal*-.

***Paropsis* G.-A. Olivier, 1807**: 597. Current status: valid genus in CHRYSOMELIDAE: CHRYSOMELINAE: CHRYSOMELINI.

Type species: *Chrysomelaaustralasiae* Fabricius, 1801, by subsequent designation (Latreille 1810: 432).

Family-group name: PAROPSINES Motschulsky, 1860. Stem: *Paropse*-.

***Parosorius* Bernhauer, 1904**: 222. Current status: valid genus in STAPHYLINIDAE: OSORIINAE: OSORIINI: PAROSORIINA.

Type species: *Ancaeusfoersteri* Bernhauer, 1903, by monotypy.

Family-group name: PAROSORII Bernhauer & Schubert, 1911. Stem: *Parosori*-.

***Pasimachus* Bonelli, 1813**: 476. Current status: valid genus in CARABIDAE: SCARITINAE: PASIMACHINI.

Type species: *Scaritesdepressus* Fabricius, 1787, by subsequent designation (Hope 1838a: 94).

Family-group name: PASIMACHIDES Putzeys, 1867. Stem: *Pasimach*-.

***Passalidius* Chaudoir, 1863b**: 116. Current status: valid genus in CARABIDAE: SCARITINAE: SCARITINI: SCAPTERINA.

Type species: *Passalidiusafer* Chaudoir, 1863 (= *Scaritesfortipes* Boheman, 1860), by subsequent designation (Lorenz 1998: 139).

Family-group name: PASSALIDIINA Csiki, 1927. Stem: *Passalidi*-.

†***Passalopalpus* Boucher & Bai in Boucher et al., 2016**: 71. Current status: valid genus in †PASSALOPALPIDAE. Note: mid-Cretaceous (Myanmar).

Type species: †*Passalopalpuscheni* Boucher, Bai & Zhang, 2016, by original designation.

Family-group name: †PASSALOPALPIDAE Boucher & Bai in Boucher et al., 2016: 68. Stem: *Passalopalp*-.

***Passalus* Fabricius, 1792b**: 240. Current status: valid genus and subgenus in PASSALIDAE: PASSALINAE: PASSALINI.

Type species: *Scarabaeusinterruptus* Linnaeus, 1758, by subsequent designation (Latreille 1810: 429).

Family-group name: PASSALIDA Leach, 1815. Stem: *Passal*-.

***Passandra* Dalman in Schönherr, 1817**: 146. Current status: valid genus in PASSANDRIDAE: PASSANDRINAE.

Type species: *Passandrasexstriata* Dalman, 1817, by monotypy.

Family-group name: PASSANDRITES Blanchard, 1845. Stem: *Passandr*-.

***Passandrella* Grouvelle, 1916a**: 6. Current status: valid genus in PASSANDRIDAE: PASSANDRINAE.

Type species: *Passandrellavisenda* Grouvelle, 1916, by monotypy.

Family-group name: PASSANDRELLINI Grouvelle, 1916. Stem: *Passandrell*-.

***Patrizia* Alluaud, 1931**: 9. Current status: junior synonym of *Agastus* Schmidt-Göbel, 1846 in CARABIDAE: HARPALINAE: ZUPHIINI: PATRIZIINA.

Type species: *Patriziazuphioides* Alluaud, 1931, by monotypy.

Family-group name: PATRIZIINI Basilewsky, 1953. Stem: *Patrizi*-.

***Patrobus* Dejean, 1821**: 10. Current status: valid genus in CARABIDAE: PATROBINAE: PATROBINI: PATROBINA.

Type species: **here fixed** (under ICZN 1999a, Article 70.3.2) as *Carabusatrorufus* Strøm, 1768, misidentified as *Carabusrufipes* Fabricius, 1792 (sensu Duftschmid, 1812), in the subsequent designation by Curtis (1827: pl. 192).

Family-group name: PATROBIDAE W. Kirby, 1837. Stem: *Patrob*-.

***Paulusiella* Löbl, 2007**: 32. Current status: valid genus in ELATERIDAE: HEMIOPINAE: PAULISIELLINI.

Type species: *Paulusiellafossulatipennis* Mandl, 1974, by original designation.

Family-group name: PAULUSIELLINI Kusy, Motyka & Bocák, 2023: 8. Stem: *Paulisiell*-.

***Paussobrenthus* Gestro, 1919**: 271. Current status: valid genus in BRENTIDAE: BRENTINAE: BRENTINI: PAUSSOBRENTHINA.

Type species: *Paussobrenthusbakeri* Gestro, 1919, by monotypy.

Family-group name: PAUSSOBRENTHINI Gestro, 1919. Stem: *Paussobrenth*-.

***Paussus* Linnaeus, 1775**: 6. Current status: valid genus in CARABIDAE: PAUSSINAE: PAUSSINI: PAUSSINA.

Type species: *Paussusmicrocephalus* Linnaeus, 1775, by monotypy.

Family-group name: PAUSSILI Latreille, 1806. Stem: *Pauss*-.

***Paxillus* W.S. MacLeay, 1819**: 96, 105. Current status: valid genus in PASSALIDAE: PASSALINAE: PASSALINI.

Type species: *Paxillusleachii* W.S. MacLeay, 1819, by subsequent designation (Gravely 1918: 48).

Family-group name: PAXILLINAE Kuwert, 1891. Stem: *Paxill*-.

***Pectocera* Hope, 1842c**: 79. Current status: valid genus in ELATERIDAE: DENDROMETRINAE: OXYNOPTERINI.

Type species: *Pectoceracantori* Hope, 1842, by subsequent designation (Hyslop 1921: 663).

Family-group name: PECTOCERINI Gurjeva, 1974. Stem: *Pectocer*-.

***Pediacus* Shuckard, 1839**: 150, 185. Current status: valid genus in CUCUJIDAE: PEDIACINAE.

Type species: *Cucujusdermestoides* Fabricius, 1792, by monotypy.

Family-group name: PEDIACINAE Jin, Zwick, Ślipiński, Marris, Thomas & H. Pang, 2020a: 260. Stem: *Pediac*-.

***Pediculota* Ádám, 1987**: 156. Current status: valid genus in STAPHYLINIDAE: ALEOCHARINAE: PEDICULOTINI.

Type species: *Pediculotahamoriae* Ádám, 1987 (= *Microdotamontandoni* Roubal, 1909), by original designation.

Family-group name: PEDICULOTINI Ádám, 1987. Stem: *Pediculot*-.

***Pedilophorus* Steffahny, 1842**: 35. Current status: valid genus in BYRRHIDAE: BYRRHINAE: PEDILOPHORINI.

Type species: *Byrrhusauratus* Duftschmid, 1825, by monotypy.

Family-group name: PEDILOPHORINI Casey, 1912. Stem: *Pedilophor*-.

***Pedilus* Fischer, 1820**: pl. 5. Current status: valid genus in PYROCHROIDAE: PEDILINAE.

Type species: *Pedilusfuscus* Fischer, 1820, by monotypy.

Family-group name: PÉDILIDES Lacordaire, 1859. Stem: *Pedil*-.

***Pedinus* Latreille, 1797**: 20. Current status: valid genus in TENEBRIONIDAE: BLAPTINAE: PEDININI: PEDININA.

Type species: *Tenebriofemoralis* Linnaeus, 1767, by subsequent designation (Stephens 1835b: 159). Note: no species were originally included in this genus by Latreille (1797); the first ones were the two species listed by Latreille (1802b: 175).

Family-group name: PEDINIDEN Eschscholtz, 1829. Stem: *Pedin*-.

***Pegylis* Erichson, 1847b**: 657. Current status: valid genus in SCARABAEIDAE: MELOLONTHINAE: MELOLONTHINI: PEGYLINA.

Type species: *Pegylismorio* Erichson, 1847, by monotypy.

Family-group name: PEGYLINI M. Lacroix, 1989. Stem: *Pegyl*-.

***Pelecium* W. Kirby, 1819**: 377. Current status: valid genus in CARABIDAE: HARPALINAE: PELECIINI.

Type species: *Peleciumcyanipes* W. Kirby, 1819, by monotypy.

Family-group name: PÉLÉCIDES Chaudoir, 1880. Stem: *Peleci*-.

***Pelecophora* Dejean, 1821**: 115. Current status: valid genus in RHADALIDAE: RHADALINAE.

Type species: *Notoxusilligeri* Gyllenhal, 1808, by monotypy.

Family-group name: PELECOPHORINI Majer, 1987. Stem: *Pelecophor*-.

***Pelecotoma* Fischer, 1809**: 293. Current status: valid genus in RIPIPHORIDAE: PELECOTOMINAE.

Type species: *Pelecotomamosquense* Fischer, 1809 (= *Ripiphorusfennicus* Paykull, 1798), by monotypy.

Family-group name: PELECOTOMINI Guérin-Méneville, 1857. Stem: *Pelecotom*-.

***Pelidnota* W.S. MacLeay, 1819**: 157. Current status: valid genus in SCARABAEIDAE: RUTELINAE: RUTELINI: RUTELINA. Note: name placed on the Official List of Generic Names in Zoology (ICZN 2003c).

Type species: *Scarabaeuspunctatus* Linnaeus, 1758, by monotypy (see ICZN 2003c). Note: name placed on the Official List of Specific Names in Zoology (ICZN 2003c).

Family-group name: PELIDNOTIDAE Burmeister, 1844. Stem: *Pelidnot*-.

***Peliocypas* Schmidt-Göbel, 1846**: 33. Current status: valid genus in CARABIDAE: HARPALINAE: LEBIINI: DEMETRIADINA.

Type species: *Peliocypassuturalis* Schmidt-Göbel, 1846, by subsequent designation (Jeannel 1949b: 991).

Family-group name: PELIOCYPINI Basilewsky, 1984. Stem: *Peliocypad*-.

***Pelmatellus* H.W. Bates, 1882**: 68. Current status: valid genus in CARABIDAE: HARPALINAE: HARPALINI: PELMATELLINA.

Type species: *Pelmatellusnitescens* H.W. Bates, 1882, by subsequent designation (Goulet 1974: 84).

Family-group name: PELMATELLINAE H.W. Bates, 1882. Stem: *Pelmatell*-.

***Pelonium* Spinola, 1845a**: 347. Current status: valid genus in CLERIDAE: KORYNETINAE.

Type species: *Enopliumviridipenne* W. Kirby, 1818, by subsequent designation (Audinet- Serville 1845: 429).

Family-group name: PELONIINAE Opitz, 2010. Stem: *Peloni*-.

***Pelonomus* Erichson, 1847a**: 510. Current status: valid genus in DRYOPIDAE.

Type species: *Dryopspicipes* G.-A. Olivier, 1791, by subsequent designation (Lacordaire 1854: 503).

Family-group name: PELONOMINAE Böving & Craighead, 1931. Stem: *Pelonom*-.

***Pelophila* Dejean, 1821**: 7. Current status: valid genus in CARABIDAE: NEBRIINAE: PELOPHILINI.

Type species: *Carabusborealis* Paykull, 1790, by monotypy.

Family-group name: PELOPHILINI Kavanaugh, 1996. Stem: *Pelophil*-.

***Pelops* Kaup, 1871**: 37. Current status: invalid senior synonym of *Protomocoelus* Zang, 1905 in PASSALIDAE: PASSALINAE: MACROLININI. Note: junior homonym of *Pelops* Gistel, 1834 [Coleoptera: TENEBRIONIDAE] and *Pelops* C.L. Koch, 1835 [Acari]; the name *Pelops* Gistel, 1834 was recently treated as a nomen oblitum by Bousquet and Bouchard (2017: 132).

Type species: *Passalusaustralis* Boisduval, 1835, by subsequent designation (Gravely 1918: 107).

Family-group name: PELOPINAE Kuwert, 1896. Stem: *Pelop*-. Note: senior homonym of PELOPINAE Ewing, 1917 (type genus *Pelops* C.L. Koch, 1835) in Acari; family-group name permanently invalid (ICZN 1999a, Article 39).

***Peloropus* Schönherr, 1835**: 456. Current status: valid genus in CURCULIONIDAE: CONODERINAE: PELOROPODINI.

Type species: *Peloropusulula* Gyllenhal, 1835, by original designation.

Family-group name: PELEROPINI Hustache, 1932. Stem: *Peloropod*-.

***Pelossus* J. Thomson, 1864**: 222. Current status: valid genus in CERAMBYCIDAE: CERAMBYCINAE: LYGRINI.

Type species: *Corethrogasterruber* J. Thomson, 1858, by original designation.

Family-group name: PELOSSINI Tavakilian, 2013: 111, **syn. nov.** Stem: *Peloss-.* Note: this family-group name is unnecessary since it was proposed as a new replacement name for LYGRINI Sama, 2008 because of the synonymy of *Lygrus* Fåhraeus, 1872 with *Pelossus* J. Thomson, 1864; however, according to Article 40.1 (ICZN 1999a) when a name of a type genus is considered to be a junior synonym, the family-group name is not to be replaced on that account alone.

***Peltastica* Mannerheim, 1852**: 334. Current status: valid genus in DERODONTIDAE: PELTASTICINAE.

Type species: *Peltasticatuberculata* Mannerheim, 1852, by monotypy.

Family-group name: PELTASTICIDAE J.L. LeConte, 1861. Stem: *Peltastic*-.

***Peltinodes* Paulian, 1950**: 19. Current status: junior synonym of *Holopsis* Broun, 1883 in CORYLOPHIDAE: CORYLOPHINAE: PELTINODINI.

Type species: *Peltinusgigas* Abeille de Perrin, 1894, by monotypy.

Family-group name: PELTINODITAE Paulian, 1950. Stem: *Peltinod*-.

***Peltinus* Mulsant & Rey, 1861**: 137. Current status: invalid senior synonym of *Teplinus* Pakaluk, Ślipiński & Lawrence, 1994 in CORYLOPHIDAE: CORYLOPHINAE: TEPLININI. Note: junior homonym of *Peltinus* Rafinesque, 1815 (unjustified emendation of *Peltis* Kugelann, 1792) [Coleoptera: PELTIDAE].

Type species: *Peltinusvelatus* Mulsant & Rey, 1861, by monotypy.

Family-group name: PELTININI Paulian, 1950. Stem: *Peltin*-. Note: family-group name permanently invalid (ICZN 1999a, Article 39).

***Peltis* Kugelann, 1792**: 508. Current status: junior synonym of *Ostoma* Laicharting, 1781 in PELTIDAE. Note: this name has been credited to O.F. Müller (1764: 13) in the literature (e.g., Kolibáč 2007: 366, Kolibáč et al. 2016: 683) but in Opinion 1754 (ICZN 1994a) the Commission ruled that *Peltis* Geoffroy, 1762 and all other uses of the name prior to the publication of *Peltis* Kugelann, 1792 are suppressed for the purposes of both the Principle of Priority and the Principle of Homonymy, while *Peltis* Kugelann, 1792 was placed on the Official List of Generic Names in Zoology (ICZN 1994a); the change of authorship for *Peltis* from “O.F. Müller, 1764” to “Kugelann, 1792” by the Commission (ICZN 1994a) resulted in the fact that *Ostoma* Laicharting, 1781 is now the oldest name for this genus and should be used as valid.

Type species: *Silphagrossa* Linnaeus, 1758, by subsequent designation (Hope 1841a: 150; see ICZN 1994a). Note: name placed on the Official List of Specific Names in Zoology (ICZN 1994a).

Family-group name: PELTIDAE W. Kirby, 1837. Stem: *Pelt*-.

***Peltodytes* Régimbart, 1879**: 450, 457. Current status: valid genus in HALIPLIDAE.

Type species: *Dytiscuscaesus* Duftschmid, 1805, by subsequent designation (F. Balfour- Browne 1936: 68).

Family-group name: PELTODYTINAE Böving & Craighead, 1931. Stem: *Peltodyt*-.

***Peltonotus* Burmeister, 1847**: 75. Current status: valid genus in SCARABAEIDAE: DYNASTINAE: CYCLOCEPHALINI.

Type species: *Peltonotusmorio* Burmeister, 1847, by monotypy. Note: Burmeister (1847: 75) mentioned a second species, *Melolonthascarabaeina* Gyllenhal, 1817 [attributed to Schönherr], but in the Anhang [supplement] in the same publication (p. 521) he placed it in *Cyclocephala* Dejean, 1821, which is where it is placed currently; therefore, we do not consider *Melolonthascarabaeina* Gyllenhal, 1817 to be an originally included species.

Family-group name: PELTONOTINI Arrow, 1917. Stem: *Peltonot*-.

†***Peltosyne* Ponomarenko, 1977**: 105. Current status: valid genus in †PELTOSYNIDAE. Note: Lower Triassic (Kyrgyzstan).

Type species: †*Peltosynetriassica* Ponomarenko, 1977, by original designation.

Family-group name: †PELTOSYNIDAE Yan, Beutel & Ponomarenko, 2017b: 516. Stem: *Peltosyn*-.

***Peneta* Lacordaire, 1859a**: 319. Current status: valid genus in TENEBRIONIDAE: PHRENAPATINAE: PENETINI.

Type species: *Penetalebasii* Lacordaire, 1859, by subsequent designation (R. Lucas 1920: 492).

Family-group name: PÉNÉTIDES Lacordaire, 1859. Stem: *Penet*-.

***Penia* Laporte, 1838**: 11. Current status: valid genus in ELATERIDAE: DENDROMETRINAE: DIMINI.

Type species: *Elatereschscholtzii* Hope, 1831, by monotypy.

Family-group name: PENIINI Dollin, 1990. Stem: *Peni*-.

***Penichrolucanus* Deyrolle, 1863**: 485. Current status: valid genus in LUCANIDAE: LUCANINAE: FIGULINI.

Type species: *Penichrolucanuscopricephalus* Deyrolle, 1863, by monotypy.

Family-group name: PENICHROLUCANINAE Arrow, 1950. Stem: *Penichrolucan*-.

***Penicillophorus* Paulus, 1975**: 69. Current status: valid genus in PHENGODIDAE: PENICILLOPHORINAE.

Type species: *Penicillophorusctenotarsus* Paulus, 1975, by original designation.

Family-group name: PENICILLOPHORINI Paulus, 1975. Stem: *Penicillophor*-.

***Pentagonica* Schmidt-Göbel, 1846**: 47. Current status: valid genus in CARABIDAE: HARPALINAE: ODACANTHINI: PENTAGONICINA.

Type species: *Pentagonicaruficollis* Schmidt-Göbel, 1846, by subsequent designation (Andrewes 1938: 137).

Family-group name: PENTAGONICINAE H.W. Bates, 1873. Stem: *Pentagonic*-.

***Pentaphyllus* Dejean, 1821**: 68. Current status: valid genus in TENEBRIONIDAE: DIAPERINAE: DIAPERINI: DIAPERINA.

Type species: *Mycetophagustestaceus* Hellwig, 1792, by monotypy.

Family-group name: PENTAPHYLLAIRES Mulsant, 1854. Stem: *Pentaphyll*-. Note: senior homonym of PENTAPHYLLINAE Schindewolf, 1942 (type genus *Pentaphyllum* de Koninck, 1872) in Anthozoa.

***Pentaplatarthrus* Westwood, 1833**: 616. Current status: valid genus in CARABIDAE: PAUSSINAE: PAUSSINI: PENTAPLATARTHRINA.

Type species: *Pentaplatarthruspaussoides* Westwood, 1833, by original designation.

Family-group name: PENTAPLATARTHRINI Jeannel, 1946. Stem: *Pentaplatarthr*-.

***Pentaria* Mulsant, 1856c**: 135. Current status: valid genus in SCRAPTIIDAE: ANASPIDINAE: PENTARIINI.

Type species: *Pentariasericaria* Mulsant, 1856 (= *Anaspisbadia* Rosenhauer, 1847), by monotypy.

Family-group name: PENTARIINI Franciscolo, 1954. Stem: *Pentari*-.

***Pentarthrum* Wollaston, 1854**: 129. Current status: valid genus in CURCULIONIDAE: COSSONINAE: PENTARTHRINI.

Type species: *Pentarthrumhuttoni* Wollaston, 1854, by monotypy.

Family-group name: PENTARTHRIDES Lacordaire, 1865. Stem: *Pentarthr*-.

***Penthe* Newman, 1838**: 373. Current status: valid genus in TETRATOMIDAE: PENTHINAE.

Type species: *Helopsobliquatus* Fabricius, 1798, by subsequent designation (Hope 1841a: 133).

Family-group name: PENTHIDES Lacordaire, 1859. Stem: *Penth*-.

***Pentilia* Mulsant, 1850**: 502. Current status: valid genus in COCCINELLIDAE: COCCINELLINAE: CRYPTOGNATHINI.

Type species: *Pentiliaegena* Mulsant, 1850, by subsequent designation (Crotch 1874a: 199).

Family-group name: PENTILIINI Casey, 1899. Stem: *Pentili*-.

***Pentodon* Hope, 1837**: 92. Current status: valid genus in SCARABAEIDAE: DYNASTINAE: PENTODONTINI: PENTODONTINA. Note: name placed on the Official List of Generic Names in Zoology (ICZN 2003c).

Type species: *Scarabaeuspunctatus* Villers, 1789, by original designation (see ICZN 2003c). Note: name ruled not to be invalid by reason of being a junior primary homonym of *Scarabaeuspunctatus* Linnaeus, 1758 and placed on the Official List of Specific Names in Zoology (ICZN 2003c).

Family-group name: PENTODONAIRES Mulsant, 1842. Stem: *Pentodont*-.

***Pericalus* W.S. MacLeay, 1825**: 15. Current status: valid genus in CARABIDAE: HARPALINAE: LEBIINI: PERICALINA.

Type species: *Catascopuscicindeloides* W.S. MacLeay, 1825, by monotypy.

Family-group name: PERICALLIDAE Hope, 1838. Stem: *Perical*-.

***Peridinetus* Schönherr, 1837**: 467. Current status: valid genus in CURCULIONIDAE: BARIDINAE: PERIDINETINI.

Type species: *Curculioirroratus* Fabricius, 1787, by original designation.

Family-group name: PÉRIDINÉTIDES Lacordaire, 1865. Stem: *Peridinet*-.

***Perieges* Schönherr, 1842a**: 419. Current status: valid genus in CURCULIONIDAE: BRACHYCERINAE: CRYPTOLARYNGINI.

Type species: *Periegesbardus* Boheman, 1842, by original designation.

Family-group name: PERIEGINI Legalov, 2003. Stem: *Perieg*-.

***Periglossium* Liebke, 1929**: 246. Current status: junior synonym of *Inna* Putzeys, 1861 in CARABIDAE: HARPALINAE: LEBIINI: PERICALINA.

Type species: *Periglossiumnevermanni* Liebke, 1929, by original designation.

Family-group name: PERIGLOSSIINAE Liebke, 1929. Stem: *Periglossi*-.

***Perigona* Laporte, 1834c**: 151. Current status: valid genus in CARABIDAE: HARPALINAE: PERIGONINI.

Type species: *Perigonapallida* Laporte, 1834, by monotypy.

Family-group name: PERIGONAE G.H. Horn, 1881. Stem: *Perigon*-. Note: nomen protectum (see Bouchard et al. 2011: 140).

***Perileptus* Schaum, 1860**: 663. Current status: valid genus in CARABIDAE: TRECHINAE: TRECHINI: TRECHODINA.

Type species: *Carabusareolatus* Creutzer, 1799, by original designation.

Family-group name: PERILEPTIDES Sloane, 1903. Stem: *Perilept*-.

***Perimylops* C. Müller, 1884**: 419. Current status: valid genus in PROMECHEILIDAE.

Type species: *Perimylopsantarcticus* C. Müller, 1884, by monotypy. Note: the original spelling *antracticus* was emended to *antarcticus* by Gebien (1905: 255); the emended name is in prevailing usage, attributed to the publication of the original spelling, and therefore deemed to be a justified emendation and the correct original spelling (ICZN 1999a, Article 33.2.3.1).

Family-group name: PERIMYLOPIDAE St. George, 1939. Stem: *Perimylop*-.

***Perinthus* Casey, 1890a**: 192. Current status: valid genus in STAPHYLINIDAE: ALEOCHARINAE: TERMITONANNINI: PERINTHINA.

Type species: *Perinthusdudleyanus* Casey, 1890, by monotypy.

Family-group name: PERINTHI Bernhauer & Scheerpeltz, 1926. Stem: *Perinth*-.

***Periommatus* Chapuis, 1865**: 316. Current status: valid genus in CURCULIONIDAE: PLATYPODINAE: TESSEROCERINI: TESSEROCERINA.

Type species: *Periommatuslongicollis* Chapuis, 1865, by monotypy.

Family-group name: PERIOMATINI Schedl, 1939. Stem: *Periommat*-.

***Periptyctus* Blackburn, 1895**: 234. Current status: valid genus in CORYLOPHIDAE: PERIPTYCTINAE.

Type species: *Periptyctusrussulus* Blackburn, 1895, by monotypy.

Family-group name: PERIPTYCTINAE Ślipiński, Lawrence & Tomaszewska, 2001. Stem: *Periptyct*-.

***Peritelus* Germar, 1823**: 407. Current status: valid genus in CURCULIONIDAE: ENTIMINAE: CYPHICERITAE: PERITELINI: PERITELINA.

Type species: *Peritelussphaeroides* Germar, 1823, by subsequent designation (Schönherr 1826: 193).

Family-group name: PÉRITÉLIDES Lacordaire, 1863. Stem: *Peritel*-.

†***Permocupes* Martynov, 1933**: 85. Current status: valid genus in †PERMOCUPEDIDAE: †PERMOCUPEDINAE. Note: Permian (Russia).

Type species: †*Permocupessemenovi* Martynov, 1933, by original designation.

Family-group name: †PERMOCUPIDAE Martynov, 1933. Stem: *Permocuped*-.

†***Permosyne* Tillyard, 1924**: 431. Current status: valid genus in †PERMOSYNIDAE. Note: Permian (Australia, South Africa, Russia).

Type species: †*Permosynebelmontensis* Tillyard, 1924, by original designation.

Family-group name: †PERMOSYNIDAE Tillyard, 1924. Stem: *Permosyn*-.

***Perochnoristhus* Basilewsky, 1973**: 225. Current status: valid genus in CARABIDAE: HARPALINAE: IDIOMORPHINI.

Type species: *Perochnoristhuspenrithae* Basilewsky, 1973, by original designation.

Family-group name: PEROCHNORISTHINAE Basilewsky, 1973. Stem: *Perochnoristh*-.

***Perothops* Eschscholtz in Laporte, 1838**: table. Current status: valid genus in EUCNEMIDAE: PEROTHOPINAE.

Type species: *Perothopscervinus* Germar, 1839, by subsequent monotypy (Germar 1839: 197).

Family-group name: PÉROTHOPIDES Lacordaire, 1857. Stem: *Perothop*-.

***Perrhynchites* Voss, 1953**: 45. Current status: valid genus in ATTELABIDAE: RHYNCHITINAE: RHYNCHITITAE: RHYNCHITINI: PERRHYNCHITINA.

Type species: *Rhynchitesaereipennis* Desbrochers des Loges, 1895, by monotypy.

Family-group name: PERRHYNCHITINA Legalov, 2003. Stem: *Perrhynchit*-.

***Perrotius* Fleutiaux, 1938**: 134. Current status: valid genus in EUCNEMIDAE: EUCNEMINAE: PERROTIINI.

Type species: *Perrotiustenuipes* Fleutiaux, 1938, by monotypy.

Family-group name: PERROTINI Muona, 1993. Stem: *Perroti*-.

***Pertinax* Harold in Gemminger and Harold, 1868c**: 976. Current status: valid subgenus of *Passalus* Fabricius, 1792 in PASSALIDAE: PASSALINAE: PASSALINI. Note: this name is attributed to Kaup (1869: 20) in the literature; however, it was first made available by Harold in 1868 when is listed several nominal species under this genus name.

Type species: *Passalusconvexus* Dalman, 1817, by subsequent designation (Hincks and Dibb 1935: 38).

Family-group name: PERTINACEAE Kaup, 1871. Stem: *Pertinac*-.

***Perucola* Théry, 1925**: 122. Current status: valid genus in BUPRESTIDAE: POLYCESTINAE: PERUCOLINI.

Type species: *Perucolapicturata* Théry, 1925, by monotypy.

Family-group name: PERUCOLINI Cobos, 1980. Stem: *Perucol*-.

***Peryphus* Dejean, 1821**: 17. Current status: valid subgenus of *Bembidion* Latreille, 1802 in CARABIDAE: TRECHINAE: BEMBIDIINI.

Type species: *Carabuslittoralis* G.-A. Olivier, 1795 (= *Bembidiontetracolum* Say, 1823), by subsequent designation (Stephens 1835b: 286).

Family-group name: PERYPHIDAE W. Kirby, 1837. Stem: *Peryph*-.

***Petalochilus* Schönherr, 1836**: 591. Current status: valid genus in CURCULIONIDAE: MOLYTINAE: PETALOCHILINI.

Type species: *Petalochilusgemellus* Gyllenhal, 1836, by original designation.

Family-group name: PÉTALOCHILIDES Lacordaire, 1863. Stem: *Petalochil*-.

***Petanops* Jeannel, 1954c**: 99. Current status: junior synonym of *Daveyia* Lea, 1912 in STAPHYLINIDAE: PSELAPHINAE: PSELAPHITAE: TYRINI: CENTROPHTHALMINA.

Type species: *Petanopsarmstrongi* Jeannel, 1954 (= *Daveyiamira* Lea, 1912), by monotypy.

Family-group name: PETANOPINI Jeannel, 1954. Stem: *Petanop*-.

***Petrejus* Harold in Gemminger and Harold, 1868c**: 978. Current status: valid genus in PASSALIDAE: PASSALINAE: PASSALINI. Note: this name is attributed to Kaup (1869: 36) in the literature; however, it was first made available by Harold in 1868 when he listed four nominal species under this genus name.

Type species: *Passalusmucronatus* Burmeister, 1847, by subsequent designation (Brauer 1870: 127).

Family-group name: PETREJINAE Kuwert, 1891. Stem: *Petrej*-.

***Petria* Semenov, 1894**: 363. Current status: valid genus in TENEBRIONIDAE: ALLECULINAE: CTENIOPODINI.

Type species: *Petriatachyptera* Semenov, 1894, by subsequent designation (R. Lucas 1920: 496).

Family-group name: PETRIIDAE Semenov, 1894. Stem: *Petri*-.

***Petrognatha* Leach, 1819**: 495. Current status: valid genus in CERAMBYCIDAE: LAMIINAE: PETROGNATHINI.

Type species: *Lamiagigas* Fabricius, 1792, by monotypy.

Family-group name: PÉTROGNATHITES Blanchard, 1845. Stem: *Petrognath*-.

***Phacellus* Dejean, 1835**: 335. Current status: valid genus in CERAMBYCIDAE: LAMIINAE: PHACELLINI.

Type species: *Acanthocinusboryi* Gory, 1832, by monotypy.

Family-group name: PHACELLIDES Lacordaire, 1872. Stem: *Phacell*-.

***Phaedimus* G.R. Waterhouse in Westwood, 1841a**: 5. Current status: valid genus in SCARABAEIDAE: CETONIINAE: PHAEDIMINI.

Type species: *Mycteristescumingii* G.R. Waterhouse, 1841, by monotypy.

Family-group name: PHAEDIMI Schoch, 1894. Stem: *Phaedim*-. Note: junior homonym of PHAEDIMIDAE Robineau-Desvoidy, 1863 (type genus *Phaedima* Robineau-Desvoidy, 1863) in Diptera and PHAEDIMIDAE Thorell, 1890 (type genus *Phaedima* Thorell, 1881) in Arachnida; Thorell’s name is permanently invalid (based on a preoccupied type genus).

***Phaedon* Latreille, 1829b**: 151. Current status: valid genus in CHRYSOMELIDAE: CHRYSOMELINAE: CHRYSOMELINI.

Type species: *Chrysomelaarmoraciae* Linnaeus, 1758, by subsequent designation (Stephens 1835b: 296).

Family-group name: PHAEDONINI Weise, 1915. Stem: *Phaedon*-.

***Phaenithon* Schönherr, 1826**: 37. Current status: valid genus in ANTHRIBIDAE: ANTHRIBINAE: CORRHECERINI.

Type species: *Anthribuscostatus* Schönherr, 1826 (= *Anthribuscurvipes* Germar, 1823), by original designation.

Family-group name: PHAENITHONINI Pierce, 1930. Stem: *Phaenithon*-.

***Phaenocephalus* Wollaston, 1873c**: 167. Current status: valid genus in PHALACRIDAE: PHAENOCEPHALINAE.

Type species: *Phaenocephaluscastaneus* Wollaston, 1873, by monotypy.

Family-group name: PHAENOCEPHALIDAE A. Matthews, 1899. Stem: *Phaenocephal*-.

***Phaenocerus* Bonvouloir, 1871**: 68, 285. Current status: valid genus in EUCNEMIDAE: EUCNEMINAE: PHAENOCERINI. Note: this name was first used by Gemminger in Gemminger and von Harold (1869b: 1469) but it is not available from that date since it was not accompanied by a description or indication (ICZN 1999a: Article 12.1).

Type species: *Phaenocerussubclavatus* Bonvouloir, 1871, by monotypy.

Family-group name: PHAENOCERINI Muona, 1993. Stem: *Phaenocer*-.

***Phaenognatha* Hope, 1842a**: 425. Current status: valid genus in SCARABAEIDAE: ACLOPINAE: PHAENOGNATHINI. Note: two original spellings were used, including *Phaenognathus* (p. 425); subsequently, Hope (1845a: 113) used only *Phaenognatha* and therefore acted as First Reviser (ICZN 1999a, Article 24.2.4).

Type species: *Phaenognathaerichsoni* Hope, 1842, by monotypy.

Family-group name: PHAENOGNATHINI Iablokoff-Khnzorian, 1977. Stem: *Phaenognath*-.

***Phaenomeris* Hope, 1833**: 62. Current status: valid genus in SCARABAEIDAE: PHAENOMERIDINAE. Note: name placed on the Official List of Generic Names in Zoology (ICZN 1962).

Type species: *Phaenomerismagnifica* Hope, 1833, by monotypy (see ICZN 1962). Note: name placed on the Official List of Specific Names in Zoology (ICZN 1962).

Family-group name: PHAENOMERINI Erichson, 1847. Stem: *Phaenomerid*- (ICZN 1962).

***Phaenomerus* Schönherr, 1836**: 632. Current status: valid genus in CURCULIONIDAE: CONODERINAE: CAMPYLOSCELINI: PHAENOMERINA. Note: name placed on the Official List of Generic Names in Zoology (ICZN 1962).

Type species: *Phaenomerussundewalli* Boheman, 1836, by original designation (see ICZN 1962). Note: name placed on the Official List of Specific Names in Zoology (ICZN 1962).

Family-group name: PHAENOMERINA Faust, 1898. Stem: *Phaenomer*-.

***Phaeochrous* Laporte, 1840b**: 108. Current status: valid genus in HYBOSORIDAE: HYBOSORINAE.

Type species: *Phaeochrousemarginatus* Laporte, 1840, by subsequent designation (Paulian 1937: 139).

Family-group name: PHAEOCHROINEN Kolbe, 1912. Stem: *Phaeochro*-.

***Phaeopholus* Roelofs, 1873**: 180. Current status: valid genus in CURCULIONIDAE: HYPERINAE: HYPERINI: PHAEOPHOLINA. Note: two original spellings were used, including *Phoeophilus* (p. 193); Alonso-Zarazaga and Lyal (1999: 189) acted as First Revisers (ICZN 1999a, Article 24.2.3) and rejected the spelling *Phoeophilus*; the alternative original spelling *Phaaeopholus* was corrected to *Phaeopholus* in an unnumbered page entitled ‘Errata pour Curculionides recueillis au Japon par M. G. Lewis’ inserted toward the end of the journal volume.

Type species: *Phaeopholusornatus* Roelofs, 1874, by monotypy.

Family-group name: PHAEOPHOLINA Legalov, 2011d: 153. Stem: *Phaeophol*-.

***Phalacrus* Paykull, 1800**: 438. Current status: valid genus in PHALACRIDAE: PHALACRINAE.

Type species: *Anisotomacorrusca* Panzer, 1796, by subsequent designation (Westwood 1838c: 10).

Family-group name: PHALACRURIDA Leach, 1815. Stem: *Phalacr*-.

***Phalangogonia* Burmeister, 1844**: 451. Current status: valid genus in SCARABAEIDAE: RUTELINAE: ANOPLOGNATHINI: PHALANGOGONIINA.

Type species: *Phalangogoniaobesa* Burmeister, 1844, by monotypy.

Family-group name: PHALANGOGONIINA Ohaus, 1918. Stem: *Phalangogoni*-.

***Phalepsus* Westwood, 1870**: 131. Current status: valid genus in STAPHYLINIDAE: PSELAPHINAE: PSELAPHITAE: PHALEPSINI.

Type species: *Phalepsussubglobosus* Westwood, 1870, by monotypy.

Family-group name: PHALEPSINI Jeannel, 1949. Stem: *Phaleps*-.

***Phaleria* Latreille, 1802b**: 162. Current status: valid genus in TENEBRIONIDAE: DIAPERINAE: PHALERIINI. Note: name placed on the Official List of Generic Names in Zoology (ICZN 1975).

Type species: *Tenebriocadaverinus* Fabricius, 1792, by plenary power (see ICZN 1975). Note: name placed on the Official List of Specific Names in Zoology (ICZN 1975).

Family-group name: PHALÉRIIDES Blanchard, 1845. Stem: *Phaleri*-.

***Phalidura* Fischer, 1823b**: 265. Current status: junior synonym of *Amycterus* Schönherr, 1823 in CURCULIONIDAE: CYCLOMINAE: AMYCTERINI.

Type species: *Phaliduramirabilis* Fischer, 1823 (= *Amycterustalpa* Schönherr, 1823), by monotypy.

Family-group name: PSALIDURIDAE Pierce, 1914. Stem: *Phalidur*-.

***Phalota* Pascoe, 1863b**: 559. Current status: valid genus in CERAMBYCIDAE: CERAMBYCINAE: PHALOTINI.

Type species: *Phalotatenella* Pascoe, 1863, by monotypy.

Family-group name: PHALOTIDES Lacordaire, 1868. Stem: *Phalot*-.

***Phanaeus* W.S. MacLeay, 1819**: 124. Current status: valid genus in SCARABAEIDAE: SCARABAEINAE: PHANAEINI: PHANAEINA.

Type species: **here fixed** (under ICZN 1999a, Article 70.3.2) as *Phanaeusvindex* W.S. MacLeay, 1819, misidentified as *Scarabaeuscarnifex* Linnaeus, 1758 (sensu Linnaeus, 1767), in the subsequent designation by d’Olsoufieff (1924: 23). Note: see Edmonds (1994: 90) for remarks about the misidentification of the type species.

Family-group name: PHANAEIDAE Hope, 1838. Stem: *Phanae*-.

***Phanerochila* Fleutiaux, 1896**: 314. Current status: valid genus in PTINIDAE: PTILININAE: PHANEROCHILINI.

Type species: *Phanerochilaboliviensis* Fleutiaux, 1896, by monotypy.

Family-group name: PHANEROCHILINI Zahradník in Zahradník and Háva, 2014: 307. Stem: *Phanerochil*-.

***Phanerotoma* Solier, 1843**: 82, 126. Current status: invalid senior synonym of *Ocnodes* Fåhraeus, 1870 in TENEBRIONIDAE: PIMELIINAE: SEPIDIINI: MOLURINA. Note: junior homonym of *Phanerotoma* Wesmael, 1838 [Hymenoptera].

Type species: *Phanerotomaelongata* Solier, 1843 (= *Pimelialaevigata* G.-A. Olivier, 1795), by original designation.

Family-group name: PHANEROTOMOIDE C. Koch, 1955. Stem: *Phanerotom*-. Note: family- group name permanently invalid (ICZN 1999a, Article 39).

***Phanerotomea* C. Koch, 1958**: 58. Current status: junior synonym of *Ocnodes* Fåhraeus, 1870 in TENEBRIONIDAE: PIMELIINAE: SEPIDIINI: MOLURINA. Note: new replacement name for *Phanerotoma* Solier, 1843.

Type species: *Phanerotomaelongata* Solier, 1843 (= *Pimelialaevigata* G.-A. Olivier, 1795), by original designation (type fixation for *Phanerotoma* Solier).

Family-group name: PHANEROTOMEINA C. Koch, 1958. Stem: *Phanerotome*-.

***Phantasis* J. Thomson, 1860a**: 25. Current status: valid genus in CERAMBYCIDAE: LAMIINAE: PHANTASINI.

Type species: *Phantasisterribilis* J. Thomson, 1860 (= *Phrissomagiganteum* Guérin- Méneville, 1844), by subsequent designation (J. Thomson 1864: 42).

Family-group name: PHANTASINAE Kolbe, 1897. Stem: *Phantas*-.

***Pharangispa* Maulik, 1929**: 233. Current status: valid genus in CHRYSOMELIDAE: CASSIDINAE: COELAENOMENODERINI.

Type species: *Pharangispapurpureipennis* Maulik, 1929, by monotypy.

Family-group name: PHARANGISPINI Uhmann, 1940. Stem: *Pharangisp*-.

***Pharaxonotha* Reitter, 1875b**: 39, 44. Current status: valid genus in EROTYLIDAE: PHARAXONOTHINAE.

Type species: *Pharaxonothakirschii* Reitter, 1875, by monotypy.

Family-group name: PHARAXONOTHINAE Crowson, 1952. Stem: *Pharaxonoth*-.

***Pharochilus* Kaup, 1868a**: 20. Current status: valid genus in PASSALIDAE: PASSALINAE: MACROLININI.

Type species: *Passalusdilatatus* Dalman, 1817, by subsequent designation (Gravely 1918: 97).

Family-group name: PHAROCHILINAE Kuwert, 1891. Stem: *Pharochil*-.

***Pharus* Mulsant, 1850**: 948, 949. Current status: invalid senior synonym of *Pharoscymnus* Bedel, 1906 in COCCINELLIDAE: COCCINELLINAE: STICHOLOTIDINI. Note: junior homonym of *Pharus* Konig, 1841 [Mollusca].

Type species: *Coccinellasexguttata* Gyllenhal, 1808, by monotypy.

Family-group name: PHARINI Casey, 1899. Stem: *Phar*-. Note: family-group name permanently invalid (ICZN 1999a, Article 39).

***Phellopsis* J.L. LeConte, 1862**: 216. Current status: valid genus in ZOPHERIDAE: ZOPHERINAE: PHELLOPSINI.

Type species: *Bolitophagusobcordatus* W. Kirby, 1837, by subsequent designation (Casey 1907: 470).

Family-group name: PHELLOPSINI Ślipiński & Lawrence, 1999. Stem: *Phellops*-.

***Phengodes* Illiger, 1807b**: 341. Current status: valid genus in PHENGODIDAE: PHENGODINAE.

Type species: *Lampyrisplumosa* G.-A. Olivier, 1790, by monotypy.

Family-group name: PHENGODINI J.L. LeConte, 1861. Stem: *Phengod*-.

***Pheropsophus* Solier, 1834a**: 461. Current status: valid genus in CARABIDAE: BRACHININAE: BRACHININI: PHEROPSOPHINA.

Type species [suggested]: *Brachinusmadagascariensis* Dejean, 1831, by subsequent designation (E. Desmarest 1851: 85). Note: the valid type species fixation is that of Hope (1838a: 99), who selected *Carabuscomplanatus* Linnaeus, 1767 (sensu Fabricius, 1801) (= *Cicindelaaequinoctialis* Linnaeus, 1763); however, neither the nominal species nor the taxonomic species involved in the type species fixation by Hope (1838a: 99) is currently recognized as a member of the genus *Pheropsophus* Solier; an application to the Commission is necessary to fix *Brachinusmadagascariensis* Dejean, 1831 as the type species of *Pheropsophus* Solier, 1834.

Family-group name: PHEROPSOPHINI Jeannel, 1949. Stem: *Pheropsoph*-.

***Phialodes* Roelofs, 1874**: 137. Current status: valid genus in ATTELABIDAE: ATTELABINAE: ATTELABINI: PHIALODINA.

Type species: *Phialodesrufipennis* Roelofs, 1874, by subsequent designation (Voss 1925: 264).

Family-group name: PHIALODINA Legalov, 2003. Stem: *Phialod*-.

***Phibalus* Gistel, 1856**: 384. Current status: valid genus in TENEBRIONIDAE: ALLECULINAE: CTENIOPODINI.

Type species: *Chrysomelapubescens* Linnaeus, 1767, by monotypy.

Family-group name: PHIBALIDAE Gistel, 1856. Stem: *Phibal*-.

***Philacamatus* Bruch, 1933b**: 206. Current status: valid genus in STAPHYLINIDAE: ALEOCHARINAE: CREMATOXENINI.

Type species: *Philacamatusbosqi* Bruch, 1933, by monotypy.

Family-group name: PHILACAMATINI Bruch, 1933. Stem: *Philacamat*-.

***Philanthaxia* Deyrolle, 1865**: 72. Current status: valid genus in BUPRESTIDAE: BUPRESTINAE: THOMASSETIINI: PHILANTHAXIINA.

Type species: *Philanthaxiacurta* Deyrolle, 1865, by monotypy.

Family-group name: PHILANTHAXIINA Holyński, 1988. Stem: *Philanthaxi*-.

***Phileurus* Latreille, 1806**: 103. Current status: valid genus in SCARABAEIDAE: DYNASTINAE: PHILEURINI: PHILEURINA.

Type species: *Scarabaeusdidymus* Linnaeus, 1758, by subsequent designation (Latreille 1810: 428).

Family-group name: PHILEURIDAE Burmeister, 1847. Stem: *Phileur*-.

***Philippinauletes* Legalov, 2023c**: 24. Current status: valid genus in ATTELABIDAE: RHYNCHITINAE: RHINOCARTITAE: PHILIPPINAULETINI. Note: *Philippinauletes*Legalov (2018c: 795) was published in a work that is nomenclaturally unavailable (ICZN 2012f, Article 8.5.3).

Type species: *Philippinauletesrubrauletiformis* Legalov, 2023, by original designation. Note: *Philippinauletesrubrauletiformis* Legalov, 2018 was published in a work that is nomenclaturally unavailable (ICZN 2012f, Article 8.5.3).

Family-group name: PHILIPPINAULETINI Legalov, 2023: 24. Stem: *Philippinaulet*-. Note: PHILIPPINAULETINILegalov (2018c: 794) was published in a work that is nomenclaturally unavailable (ICZN 201f2, Article 8.5.3).

***Philonthus* Stephens, 1829b**: 23. Current status: valid genus in STAPHYLINIDAE: STAPHYLININAE: STAPHYLININI: PHILONTHINA.

Type species: *Staphylinuspolitus* Linnaeus, 1758, by subsequent designation (Stephens 1835b: 312). Note: the type species usually listed for *Philonthus* Stephens is *Staphylinussplendens* Fabricius, 1792 (e.g., Schülke and Smetana 2015: 1034) by subsequent designation of Curtis (1836: pl. 610) but Stephens’ designation is older; both species are currently included in the nominotypical subgenus of *Philonthus* Stephens, 1829.

Family-group name: PHILONTHIDAE W. Kirby, 1837. Stem: *Philonth*-.

***Philopedon* Schönherr, 1826**: 97. Current status: valid genus in CURCULIONIDAE: ENTIMINAE: POLYDRUSITAE: CNEORHININI.

Type species: *Curculiogeminatus* Fabricius, 1787 (= *Curculioplagiatus* Schaller, 1783), by original designation.

Family-group name: PHILOPEDINI Bedel, 1883. Stem: *Philoped*-.

***Philotermes* Kraatz, 1857a**: 13. Current status: valid genus in STAPHYLINIDAE: ALEOCHARINAE: PHILOTERMITINI.

Type species: *Philotermespilosus* Kraatz, 1857, by subsequent designation (Fenyes 1919: 24).

Family-group name: PHILOTERMITINI Seevers, 1957. Stem: *Philotermit*-.

***Philothalpus* Kraatz, 1857b**: 540. Current status: valid genus in STAPHYLINIDAE: STAPHYLININAE: STAPHYLININI: PHILOTHALPINA.

Type species: *Philonthusfervidus* Erichson, 1840, by subsequent designation (Herman 2001: 10).

Family-group name: PHILOTHALPINA Chatzimanolis & Brunke in Chani-Posse, Brunke, Chatzimanolis, Schillhammer and Solodovnikov, 2018: 34. Stem: *Philothalp*-.

***Philus* Saunders, 1853**: 110. Current status: valid genus in VESPERIDAE: PHILINAE.

Type species: *Philusinconspicuus* Saunders, 1853 (= *Stenochorusantennatus* Gyllenhal, 1817), by monotypy.

Family-group name: PHILITAE J. Thomson, 1861. Stem: *Phil*-.

***Phlegon* Laporte, 1840a**: 254. Current status: valid genus in EUCNEMIDAE: PHLEGONINAE.

Type species: *Phlegonbuqueti* Laporte, 1840, by monotypy.

Family-group name: PHLEGONINAE Muona, 1993. Stem: *Phlegon*-.

***Phloeoborus* Erichson, 1836**: 54. Current status: valid genus in CURCULIONIDAE: SCOLYTINAE: HYLESININI.

Type species: *Phloeoborusrudis* Erichson, 1836, by subsequent designation (Hopkins 1914: 126).

Family-group name: PHLOEOBORI W.F.H. Blandford, 1893. Stem: *Phloeobor*-.

***Phloeocharis* Mannerheim, 1830**: 50. Current status: valid genus in STAPHYLINIDAE: PHLOEOCHARINAE.

Type species: *Phloeocharissubtilissima* Mannerheim, 1830, by monotypy.

Family-group name: PHLOEOCHARINI Erichson, 1839. Stem: *Phloeochar*-.

***Phloeonomus* Heer, 1840**: 184. Current status: valid genus in STAPHYLINIDAE: OMALIINAE: OMALIINI.

Type species [suggested]: *Omaliumpusillum* Gravenhorst, 1806, by subsequent designation (C.G. Thomson 1859: 51). Note: the valid type species fixation is that of E. Desmarest (1852: 115), who selected *Omaliummonilicorne* Gyllenhal, 1810, but that species is the type species of *Xylostiba* Ganglbauer, 1895; an application to the Commission is necessary to fix *Omaliumpusillum* Gravenhorst, 1806 as the type species of *Phloeonomus* Heer, 1840.

Family-group name: PHLOEONOMINI Ádám, 2001. Stem: *Phloeonom*-.

***Phloeophagus* Schönherr, 1838**: 1047. Current status: valid genus in CURCULIONIDAE: COSSONINAE: RHYNCOLINI: PHLOEOPHAGINA.

Type species: **here fixed** (under ICZN 1999a, Article 70.3.2) as *Phloeophagusturbatus* Schönherr, 1845, misidentified as *Curculiolignarius* Marsham, 1802 (sensu Boheman, 1838), by original designation by Schönherr (1838: 1047).

Family-group name: PHLOEOPHAGINA Voss, 1955. Stem: *Phloeophag*-.

***Phloeophilus* Schönherr, 1833**: 156. Current status: valid genus in ANTHRIBIDAE: ANTHRIBINAE: PHLOEOPHILINI.

Type species: *Phloeophilusagrestis* Boheman, 1833, by original designation.

Family-group name: PHLOEOPHILIDES Lacordaire, 1865. Stem: *Phloeophil*-.

***Phloeopora* Erichson, 1837**: 311. Current status: valid genus in STAPHYLINIDAE: ALEOCHARINAE: OXYPODINI: PHLOEOPORINA.

Type species: *Aleocharacorticalis* Gravenhorst, 1802, by subsequent designation (Westwood 1838c: 19).

Family-group name: PHLOEOPORIDES C.G. Thomson, 1859. Stem: *Phloeopor*-.

***Phloeosinus* Chapuis, 1869**: 37. Current status: valid genus in CURCULIONIDAE: SCOLYTINAE: PHLOEOSININI. Note: name placed on the Official List of Generic Names in Zoology (ICZN 1981a).

Type species: *Hylesinusthujae* Perris, 1855 [as *thuyae*], by subsequent designation (Hopkins 1914: 126; see ICZN 1981a). Note: name placed on the Official List of Specific Names in Zoology (ICZN 1981a).

Family-group name: PHLOEOSINIDES Nüsslin, 1912. Stem: *Phloeosin*-.

***Phloeostichus* W. Redtenbacher, 1842**: 15. Current status: valid genus in PHLOEOSTICHIDAE.

Type species: *Phloeostichusdenticollis* W. Redtenbacher, 1842, by monotypy.

Family-group name: PHLOEOSTICHINI Reitter, 1911. Stem: *Phloeostich*-.

***Phloeotragus* Schönherr, 1823**: 1135. Current status: valid genus in ANTHRIBIDAE: ANTHRIBINAE: PHLOEOTRAGINI.

Type species: *Anthribusheros* Fabricius, 1801, by original designation.

Family-group name: PHLOEOTRAGINAE Kolbe, 1897. Stem: *Phloeotrag*-.

***Phloeotribus* Latreille, 1797**: 50. Current status: valid genus in CURCULIONIDAE: SCOLYTINAE: PHLOEOTRIBINI. Note: the original spelling was *Phloiotribus* but *Phloeotribus*, introduced by Illiger (1804: 108), was ruled to be a justified emendation and *Phloeotribus* Latreille, 1797 was placed on the Official List of Generic Names in Zoology (ICZN 1979a, as *Phloeotribus* Latreille, 1796).

Type species: *Bostrichusoleae* Fabricius, 1792 (= *Scolytusscarabaeoides* Bernard, 1788), by subsequent monotypy (Latreille 1802b: 204; see ICZN 1979a). Note: *Scolytusscarabaeoides* Bernard, 1788 was placed on the Official List of Specific Names in Zoology (ICZN 1979a).

Family-group name: PHLOEOTRIBIDAE Chapuis, 1869. Stem: *Phloeotrib*-.

***Phloeotrupes* Erichson, 1836**: 53. Current status: junior synonym of *Phloeoborus* Erichson, 1836 in CURCULIONIDAE: SCOLYTINAE: HYLESININI.

Type species: *Phloeotrupesgrandis* Erichson, 1836, by subsequent designation (Hopkins 1914: 127).

Family-group name: PHLOEOTRUPIDES Lacordaire, 1865. Stem: *Phloeotrup*-.

***Phloiophilus* Stephens, 1830**: 81. Current status: valid genus in PHLOIOPHILIDAE.

Type species: *Phloiophilusedwardsii* Stephens,1830, by subsequent designation (Stephens 1835b: 314).

Family-group name: PHLOEOPHILINI Kiesenwetter, 1863. Stem: *Phloiophil*-.

***Phlyctaenodes* Newman, 1840a**: 20. Current status: valid genus in CERAMBYCIDAE: CERAMBYCINAE: PHLYCTAENODINI.

Type species: *Phlyctaenodespustulosa* Newman, 1840, by monotypy.

Family-group name: PHLYCTÉNODIDES Lacordaire, 1868. Stem: *Phlyctaenod*-.

***Phobelius* Blanchard, 1842c**: pl. 14. Current status: valid genus in TENEBRIONIDAE: LAGRIINAE: LAGRIINI: PHOBELIINA. Note: this name is often credited to Blanchard (1845b: 39) in the literature but it was first made available by Blanchard (1842c: pl. 14, fig. 9) when he illustrated the species *Phobeliuscrenatus* (see Bouchard et al. 2021: 297).

Type species: *Phobeliuscrenatus* Blanchard, 1842, by monotypy.

Family-group name: PHOBELIIDES F. Bates, 1890. Stem: *Phobeli*-.

***Phoberus* W.S. MacLeay, 1819**: 137. Current status: valid genus in TROGIDAE: TROGINAE.

Type species: *Troxhorridus* Fabricius, 1775, by monotypy.

Family-group name: PHOBERIDAE Gistel, 1848. Stem: *Phober*-.

***Phobetus* J.L. LeConte, 1856b**: 227. Current status: valid genus in SCARABAEIDAE: MELOLONTHINAE: PHOBETUSINI.

Type species: *Phobetuscomatus* J.L. LeConte, 1856, by monotypy.

Family-group name: PHOBETUSINI Evans & A.B.T. Smith, 2020: 66. Stem: *Phobetus*- (ICZN 1999a, Article 29.4).

***Phodaga* J.L. LeConte, 1858a**: 76. Current status: valid genus in MELOIDAE: MELOINAE: EUPOMPHINI.

Type species: *Phodagaalticeps* J.L. LeConte, 1858, by monotypy.

Family-group name: PHODAGAE J.L. LeConte, 1862. Stem: *Phodag*-.

***Phoenicobates* Champion, 1914b**: 417. Current status: valid genus in CURCULIONIDAE: MOLYTINAE: PHOENICOBATINI.

Type species: *Phoenicobatesvittatus* Champion, 1914, by original designation.

Family-group name: PHOENICOBATINA Champion, 1914. Stem: *Phoenicobat*-.

***Pholeuon* Hampe, 1856**: 463. Current status: valid genus in LEIODIDAE: CHOLEVINAE: LEPTODIRINI: PHOLEUINA. Note: the original spelling was *Poleuon* but it was changed to *Pholeuon* on page [724] of the same journal.

Type species: *Pholeuonangusticolle* Hampe, 1856, by monotypy.

Family-group name: PHOLEUONES Reitter, 1886. Stem: *Pholeu*-.

***Pholidochlamys* Lacordaire, 1865**: 473. Current status: valid genus in BRENTIDAE: BRENTINAE: ULOCERINI: PHOLIDOCHLAMYDINA.

Type species: *Pholidochlamysmadagascariensis* Lacordaire, 1865, by monotypy.

Family-group name: PHOLIDOCHLAMYDINAE Damoiseau, 1962. Stem: *Pholidochlamyd*-.

***Pholidotus* W.S. MacLeay, 1819**: 96, 97. Current status: synonym of *Casignetus* W.S. MacLeay, 1819 in LUCANIDAE: LUCANINAE: LUCANINI. Note: junior homonym of *Pholidotus* Brisson, 1762 [Mammalia]; First Reviser (ICZN 1999a, Article 24.2.2) for *Pholidotus* W.S. MacLeay, 1819 versus *Casignetus* W.S. MacLeay, 1819 is Westwood (1834: 119).

Type species: *Pholidotuslepidosus* W.S. MacLeay, 1819 (= *Lamprimahumboldti* Gyllenhal, 1817), by monotypy.

Family-group name: PHOLIDOTINI Kikuta, 1986. Stem: *Pholidot*-. Note: family-group name permanently invalid (ICZN 1999a, Article 39).

***Pholidus* Mulsant & Rey, 1856**: 7. Current status: junior synonym of *Euphanias* Fairmaire & Laboulbène, 1856 in STAPHYLINIDAE: OXYTELINAE: EUPHANIINI. Note: junior homonym of *Pholidus* F. Moore, 1854 (unjustified emendation of *Phodilus* Geoffroy, 1830) [Aves].

Type species: *Pholidusinsignis* Mulsant & Rey, 1856, by monotypy.

Family-group name: PHOLIDINI Acloque, 1895. Stem: *Pholid*-. Note: family-group name permanently invalid (ICZN 1999a, Article 39).

***Phoracantha* Newman, 1840a**: 19. Current status: valid genus in CERAMBYCIDAE: CERAMBYCINAE: PHORACANTHINI.

Type species: *Stenocorussemipunctatus* Fabricius, 1775, by original designation.

Family-group name: PHORACANTHIDAE Newman, 1840. Stem: *Phoracanth*-.

***Phoroneus* Kaup, 1868b**: 19. Current status: junior synonym of *Passalus* Fabricius, 1792 in PASSALIDAE: PASSALINAE: PASSALINI. Note: junior homonym of *Phoroneus* Stål, 1865 [Hemiptera].

Type species: *Passalusquadricollis* Eschscholtz, 1829, by subsequent designation (Hincks and Dibb 1935: 38).

Family-group name: PHORONEAE Kaup, 1871. Stem: *Phorone*-. Note: family-group name permanently invalid (ICZN 1999a, Article 39).

†***Phoroschizus* Bouchard & Bousquet, 2020a**: 70. Current status: valid genus in †PHOROSCHIZIDAE. Note: new replacement name for *Schizophorus* Ponomarenko, 1968; Upper Jurassic (Kazakhstan).

Type species: †*Schizophoruscrassus* Ponomarenko, 1968, by original designation (type fixation for *Schizophorus* Ponomarenko).

Family-group name: †PHOROSCHIZIDAE Bouchard & Bousquet, 2020a: 70. Stem: *Phoroschiz*-.

***Phosphaenus* Laporte, 1833c**: 138. Current status: valid genus in LAMPYRIDAE: LAMPYRINAE: LUCIDOTINI: PHOTININA.

Type species: *Lampyrishemiptera* Geoffroy, 1762, by monotypy.

Family-group name: PHOSPHAENIDES E. Olivier, 1884. Stem: *Phosphaen*-.

***Photinus* Laporte, 1833c**: 140. Current status: valid genus in LAMPYRIDAE: LAMPYRINAE: LUCIDOTINI: PHOTININA. Note: name placed on the Official List of Generic Names in Zoology (ICZN 2017b).

Type species: *Lampyrispallens* Fabricius, 1798 [as *pullens*], by subsequent designation (Paulian 1947: 160; see ICZN 2017b). Note: the spelling of the type species was ruled to be a justified emendation of the original spelling *L.pullens* by the Commission and *Lampyrispallens* Fabricius, 1798 was placed on the Official List of Specific Names in Zoology (ICZN 2017b).

Family-group name: PHOTINI J.L. LeConte, 1881. Stem: *Photin*-. Note: the spelling of the family-group name PHOTININAE Giglio-Tos, 1915 [Mantodea] was ruled to be PHOTINAINAE by the Commission (see ICZN 2017b) to remove the homonymy with J.L. LeConte’s name.

***Photuris* Dejean, 1833b**: 103. Current status: valid genus in LAMPYRIDAE: PHOTURINAE.

Type species: *Lampyrisversicolor* Fabricius, 1798, by subsequent designation (Barber 1951: 11).

Family-group name: PHOTURIDES Lacordaire, 1857. Stem: *Photur*-.

***Phratora* Chevrolat in Dejean, 1836a**: 405. Current status: valid genus in CHRYSOMELIDAE: CHRYSOMELINAE: CHRYSOMELINI.

Type species: *Chrysomelavulgatissima* Linnaeus, 1758, by subsequent designation (Motschulsky 1860b: 219). Note: the type species for this genus should be *Chrysomelavitellinae* Linnaeus, 1758 (ICZN 1999a, Article 68.3) as it was the only taxonomic species cited by Chevrolat in Dejean (1836a: 405), but that species is currently included in the subgenus Phyllodecta W. Kirby, 1837 of the genus *Phratora* (e.g., Kippenberg 2010: 395); a request should be submitted to the Commission to fix *Chrysomelavulgatissima* Linnaeus, 1758 as the type species of the genus *Phratora* Chevrolat, 1836.

Family-group name: PHRATHORINES Motschulsky, 1860. Stem: *Phrator*-.

***Phreatodytes* Uéno, 1957**: 252. Current status: valid genus in NOTERIDAE: NOTOMICRINAE: PHREATODYTINI.

Type species: *Phreatodytesrelictus* Uéno, 1957, by original designation.

Family-group name: PHREATODYTIDAE Uéno, 1957. Stem: *Phreatodyt*-.

***Phrenapates* G.R. Gray in Griffith and Pidgeon, 1831**: pls 50, 69. Current status: valid genus in TENEBRIONIDAE: PHRENAPATINAE: PHRENAPATINI.

Type species: *Phrenapatesbennettii* G.R. Gray, 1831, by monotypy.

Family-group name: PHRÉPATIDES Solier, 1834. Stem: *Phrenapat*-.

***Phrissoma* Dejean, 1835**: 345. Current status: valid genus in CERAMBYCIDAE: LAMIINAE: LAMIINI.

Type species: *Lamiacrispa* Fabricius, 1777, by subsequent designation (E. Desmarest 1860: 324).

Family-group name: PHRISSOMITAE J. Thomson, 1860. Stem: *Phrissomat*-.

***Phrixia* Deyrolle, 1865**: 66. Current status: valid genus in BUPRESTIDAE: BUPRESTINAE: PHRIXIINI.

Type species: *Phrixiafiliformis* Deyrolle, 1865, by subsequent designation (Cobos 1975: 104).

Family-group name: PHRIXIINI Cobos, 1975. Stem: *Phrixi*-.

***Phrixosoma* W.F.H. Blandford, 1896**: 143. Current status: valid genus in CURCULIONIDAE: SCOLYTINAE: PHRIXOSOMATINI.

Type species: *Phrixosomarude* W.F.H. Blandford, 1897, by subsequent monotypy (W.F.H. Blandford 1897: 148).

Family-group name: PHRIXOSOMINI Wood, 1978. Stem: *Phrixosomat*-.

***Phrydiuchus* Gozis, 1885**: 129. Current status: valid genus in CURCULIONIDAE: CEUTORHYNCHINAE: CEUTORHYNCHINI.

Type species: *Ceutorhynchustopiarius* Germar, 1823, by monotypy.

Family-group name: PHRYDIUCHINA Wagner, 1938. Stem: *Phrydiuch*-.

***Phryneta* Dejean, 1835**: 341. Current status: valid genus in CERAMBYCIDAE: LAMIINAE: PHRYNETINI.

Type species: *Lamiamarmorea* G.-A. Olivier, 1792, by subsequent designation (J. Thomson 1864: 71).

Family-group name: PHRYNETITAE J. Thomson, 1864. Stem: *Phrynet*-.

***Phrynixus* Pascoe, 1875**: 221. Current status: valid genus in CURCULIONIDAE: MOLYTINAE: PHRYNIXINI: PHRYNIXINA.

Type species: *Phrynixusterreus* Pascoe, 1875, by monotypy.

Family-group name: PHRYNIXINAE Kuschel, 1964. Stem: *Phrynix*-.

***Phrynocarenum* Gebien, 1928**: 106. Current status: valid genus in TENEBRIONIDAE: PIMELIINAE: PHRYNOCARENINI.

Type species: *Phrynocarenumbruchianum* Gebien, 1928 (= *Emmalloderastrangulate* Fairmaire, 1905), by monotypy.

Family-group name: PHRYNOCARENINAE Gebien, 1928. Stem: *Phrynocaren*-.

***Phtegnomus* Raffray, 1890a**: 104, 107. Current status: valid genus in STAPHYLINIDAE: PSELAPHINAE: EUPLECTITAE: TROGASTRINI: PHTEGNOMINA.

Type species: *Phtegnomusoberthuri* Raffray, 1890, by monotypy.

Family-group name: PHTEGNOMINI Park, 1951. Stem: *Phtegnom*-.

***Phthorophloeus* Rey in Eichhoff, 1883**: 128. Current status: junior synonym of *Phloeotribus* Latreille, 1797 in CURCULIONIDAE: SCOLYTINAE: PHLOEOTRIBINI.

Type species: *Phloeophthorusspinulosus* Rey, 1883, by monotypy.

Family-group name: PHTHOROPHLOEIDES Nüsslin, 1912. Stem: *Phthorophloe*-.

***Phtora* Mulsant, 1854**: 228. Current status: invalid senior synonym of *Clamoris* des Gozis, 1886 in TENEBRIONIDAE: PHRENAPATINAE: PENETINI. Note: junior homonym of *Phtora* Germar, 1836 [Coleoptera: TENEBRIONIDAE].

Type species: *Phtoracrenata* Mulsant, 1854 (= *Clamorisinsurgens* Gozis, 1886), by monotypy.

Family-group name: PHTHORINI Boddy, 1965. Stem: *Phtor*-. Note: family-group name permanently invalid (ICZN 1999a, Article 39).

***Phycocoetes* J.L. LeConte in J.L. LeConte and G.H. Horn, 1876**: 189. Current status: invalid senior synonym of *Thalasselephas* Egorov & Korotyaev, 1976 in CURCULIONIDAE: MOLYTINAE: EMPHYASTINI. Note: junior homonym of *Phycocoetes* Agassiz, 1846 (unjustified emendation of *Phucocoetes* Jenyns, 1842) [Pisces].

Type species: *Phycocoetestestaceus* J.L. LeConte, 1876, by monotypy.

Family-group name: PHYCOCOETES J.L. LeConte, 1876. Stem: *Phycocoet*-. Note: family-group name permanently invalid (ICZN 1999a, Article 39).

***Phycocus* Broun, 1883**: 299. Current status: valid genus in SCARABAEIDAE: APHODIINAE: PSAMMODIINI: PHYCOCINA.

Type species: *Phycocusgraniceps* Broun, 1883, by monotypy.

Family-group name: PHYCOCHI Landin, 1960. Stem: *Phycoc*-.

***Phycosecis* Pascoe, 1875**: 213. Current status: valid genus in PHYCOSECIDAE.

Type species: *Phycosecislitoralis* Pascoe, 1875, by **present designation**. Note: Crowson (1964: 313) selected *Dermesteslimbatus* Fabricius, 1781 as the type species for this genus- group name but it is not an originally included nominal species.

Family-group name: PHYCOSECIDAE Crowson, 1952. Stem: *Phycosec*-.

***Phylax* Brullé, 1832**: 209. Current status: junior synonym of *Phylan* Sturm, 1826 in TENEBRIONIDAE: BLAPTINAE: DENDARINI: DENDARINA. Note: unnecessary new replacement name for *Phylan* Sturm, 1826 (see Bouchard and Bousquet 2020a: 100, 101).

Type species: *Opatrumgibbum* Fabricius, 1775, by subsequent monotypy (Steven 1828: 99) (type fixation for *Phylan* Sturm).

Family-group name: PHYLACIDES Lacordaire, 1859. Stem: *Phylac*-.

***Phyllarthrius* Hope, 1843b**: 366. Current status: valid genus in CERAMBYCIDAE: CERAMBYCINAE: PHYLLARTHRIINI.

Type species: *Phyllarthriusafricanus* Hope, 1843, by subsequent designation (J. Thomson 1864: 269).

Family-group name: PHYLLARTHRIINI Lepesme & Breuning, 1956. Stem: *Phyllarthri*-.

***Phyllastolus* Gistel, 1856**: 372. Current status: junior synonym of *Trachyphloeus* Germar, 1817 in CURCULIONIDAE: ENTIMINAE: CYPHICERITAE: TRACHYPHLOEINI: TRACHYPHLOEINA.

Type species: *Curculioscabriculus* Linnaeus, 1771 [as *scabricul*], by subsequent designation (Alonso-Zarazaga and Lyal 1999: 22, 183).

Family-group name: PHYLLASTOLIDAE Gistel, 1856. Stem: *Phyllastol*-.

***Phyllecthris* Dejean, 1836a**: 382. Current status: valid genus in CHRYSOMELIDAE: GALERUCINAE: LUPERINI.

Type species: *Galerucadorsalis* G.-A. Olivier, 1808, by monotypy.

Family-group name: PHYLLECTHRITES G.H. Horn, 1893. Stem: *Phyllecthr*-.

***Phyllobaenus* Dejean, 1833b**: 113. Current status: valid genus in CLERIDAE: CLERINAE: CLERITAE: HYDNOCERINI: HYDNOCERINA.

Type species: *Clerushumeralis* Germar, 1823, by monotypy.

Family-group name: PHYLLOBÉNIDES Lacordaire, 1857. Stem: *Phyllobaen*-.

***Phyllobius* Germar, 1823**: 447. Current status: valid genus in CURCULIONIDAE: ENTIMINAE: OTIORHYNCHITAE: PHYLLOBIINI. Note: name placed on the Official List of Generic Names in Zoology (ICZN 1981c, as *Phyllobius* Germar, 1824).

Type species: *Curculiopyri* Linnaeus, 1758, by subsequent designation (Schönherr 1826: 15; see ICZN 1981c). Note: name placed on the Official List of Specific Names in Zoology (ICZN 1981c).

Family-group name: PHYLLOBIDES Schönherr, 1826. Stem: *Phyllobi*-.

***Phyllobrotica* Chevrolat in Dejean, 1836a**: 381. Current status: valid genus in CHRYSOMELIDAE: GALERUCINAE: LUPERINI.

Type species: *Chrysomelaquadrimaculata* Linnaeus, 1758, by subsequent designation (C.G. Thomson 1859: 156).

Family-group name: PHYLLOBROTICITES Chapuis, 1875. Stem: *Phyllobrotic*-.

***Phyllocerus* Lepeletier & Audinet-Serville, 1825**: 116. Current status: valid genus in EUCNEMIDAE: PHYLLOCERINAE: PHYLLOCERINI.

Type species: *Phyllocerusflavipennis* Lepeletier & Audinet-Serville, 1825, by monotypy.

Family-group name: PHYLLOCERIDAE Reitter, 1905. Stem: *Phyllocer*-.

***Phyllocharis* Dalman, 1824**: 21. Current status: valid genus in CHRYSOMELIDAE: CHRYSOMELINAE: CHRYSOMELINI.

Type species: *Chrysomelacyanipes* Fabricius, 1775, by subsequent designation (Hope 1841a: 163).

Family-group name: PHYLLOCHARITES Chapuis, 1874. Stem: *Phyllocharit*-.

***Phyllodecta* W. Kirby, 1837**: 216. Current status: valid subgenus of *Phratora* Chevrolat, 1836 in CHRYSOMELIDAE: CHRYSOMELINAE: CHRYSOMELINI.

Type species: *Chrysomelavitellinae* Linnaeus, 1758, by monotypy. Note: see remark about the type species of *Phratora* Chevrolat, 1836.

Family-group name: PHYLLODECTAE J.L. LeConte & G.H. Horn, 1883. Stem: *Phyllodect*-.

***Phyllodinarda* Wasmann, 1916b**: 105. Current status: valid genus in STAPHYLINIDAE: ALEOCHARINAE: PHYLLODINARDINI.

Type species: *Phyllodinardaxenocephala* Wasmann, 1916, by subsequent designation (Blackwelder 1952: 307).

Family-group name: PHYLLODINARDINI Wasmann, 1916. Stem: *Phyllodinard*-.

***Phyllomanes* Gistel, 1848b**: xi. Current status: junior synonym of *Polydrusus* Germar, 1817 in CURCULIONIDAE: ENTIMINAE: POLYDRUSITAE: POLYDRUSINI. Note: unnecessary new replacement name for *Polydrosus* Schönherr, 1823 (an unjustified emendation of *Polydrusus* Germar, 1817); junior homonym of *Phyllomanes* Cabanis, 1847 [Aves].

Type species: *Curculioundatus* Fabricius, 1781 (= *Curculiotereticollis* DeGeer, 1775), by subsequent designation (Schönherr 1826: 12; see ICZN 1981c) (type fixation for *Polydrusus* Germar).

Family-group name: PHYLLOMANISIDAE Gistel, 1848. Stem: *Phylloman*-. Note: family-group name permanently invalid (ICZN 1999a, Article 39).

***Phyllonomeus* Gistel, 1856**: 372. Current status: valid genus in CURCULIONIDAE: LIXINAE: LIXINI.

Type species: *Curculioiaceae* Fabricius, 1775, by subsequent designation (Alonso-Zarazaga and Lyal 1999: 22, 190).

Family-group name: PHYLLONOMEIDAE Gistel, 1856. Stem: *Phyllonome*-.

***Phyllophorus* Hope, 1842c**: 73. Current status: junior synonym of *Tetralobus* Lepeletier & Audinet- Serville, 1828 in ELATERIDAE: TETRALOBINAE: TETRALOBINI. Note: junior homonym of *Phyllophorus* Grube, 1840 [Echinodermata].

Type species: *Elatergigas* Fabricius, 1801, by monotypy.

Family-group name: PHYLLOPHORIDAE Hope, 1842. Stem: *Phyllophor*-. Note: family-group name permanently invalid (ICZN 1999a, Article 39).

***Phylloplatypus* Kato, 1998**: 72. Current status: valid genus in CURCULIONIDAE: COSSONINAE: COSSONINI.

Type species: *Phylloplatypuspandani* Kato, 1998, by original designation.

Family-group name: PHYLLOPLATYPODINI Kato, 1998. Stem: *Phylloplatypod*-.

***Phyllotocidium* Blackburn, 1898**: 21, 24. Current status: valid genus in SCARABAEIDAE: MELOLONTHINAE: PHYLLOTOCIDIINI.

Type species: *Cheiragramacleayi* Blackburn, 1892, by original designation.

Family-group name: PHYLLOTOCIDIINI Britton, 1957. Stem: *Phyllotocidi*-.

***Phyllotocus* Fischer, 1823b**: 255. Current status: valid genus in SCARABAEIDAE: SERICOIDINAE: PHYLLOTOCINI.

Type species: *Phyllotocusmacleayi* Fischer, 1823, by monotypy.

Family-group name: PHYLLOTOCIDAE Burmeister, 1855. Stem: *Phyllotoc*-.

***Phyllotrox* Schönherr, 1843**: 189. Current status: valid genus in CURCULIONIDAE: CURCULIONINAE: DERELOMINI: PHYLLOTROGINA.

Type species: *Phyllotroxsemirufus* Boheman, 1843, by original designation (as *rufus*, lapsus).

Family-group name: PHYLLOTROGINA N.M. Franz, 2006. Stem: *Phyllotrog*-.

***Phyllurga* Gistel, 1848b**: xi, xiii. Current status: junior synonym of *Euchlora* W.S. MacLeay, 1819 in SCARABAEIDAE: RUTELINAE: ANOMALINI: ANOMALINA. Note: unnecessary new replacement name for *Euchlora* W.S. MacLeay, 1819.

Type species: *Melolonthaviridis* Fabricius, 1775, by subsequent designation (Hope 1837: 71) (type fixation for *Euchlora* W.S. MacLeay).

Family-group name: PHYLLURGAEIDAE Gistel, 1848. Stem: *Phyllurg*-.

***Phymaphora* Newman, 1838**: 389. Current status: valid genus in ENDOMYCHIDAE: LEIESTINAE.

Type species: *Phymaphorapulchella* Newman, 1838, by monotypy.

Family-group name: PHYMAPHORINA Jakobson, 1915. Stem: *Phymaphor*-.

***Phymasterna* Laporte, 1840b**: 473. Current status: valid genus in CERAMBYCIDAE: LAMIINAE: PHYMASTERNINI. Note: the senior homonym *Phymasterna* Dejean, 1835 is available but in a different sense (see Bousquet and Bouchard 2013a: 88); we recommend that an application be submitted to the Commission to suppress Dejean’s name for the Principles of Priority and Homonymy.

Type species: *Phymasternalacteoguttata* Laporte, 1840, by monotypy.

Family-group name: PHYMASTERNINI Téocchi, 1989. Stem: *Phymastern*-.

***Phymatolabus* Jekel, 1860**: 197. Current status: valid genus in ATTELABIDAE: ATTELABINAE: ATTELABINI: PHYMATOLABINA.

Type species: *Attelabusdentipennis* Gyllenhal, 1839, by original designation.

Family-group name: PHYMATOLABINA Voss, 1925. Stem: *Phymatolab*-.

***Phymatopsinus* Voss, 1925**: 205. Current status: valid genus in ATTELABIDAE: ATTELABINAE: ATTELABINI: PHYMATOPSININA.

Type species: *Attelabuspustula* Ancey, 1881, by original designation.

Family-group name: PHYMATOPSININA Legalov, 2003. Stem: *Phymatopsin*-.

***Physea* Brullé, 1834**: 473. Current status: valid genus in CARABIDAE: PAUSSINAE: OZAENINI. Note: new replacement name for *Trachelizus* Solier, 1834.

Type species: *Trachelizusrufus* Solier, 1834 (= *Ozaenatestudinea* Klug, 1834), by monotypy (type fixation for *Trachelizus* Solier).

Family-group name: PHYSEITAE Jeannel, 1946. Stem: *Physe*-.

***Physocrotaphus* Parry, 1849**: 180. Current status: valid genus in CARABIDAE: HARPALINAE: PHYSOCROTAPHINI.

Type species: *Physocrotaphusceylonicus* Parry, 1849, by monotypy.

Family-group name: PHYSOCROTAPHIDES Chaudoir, 1863. Stem: *Physocrotaph*-.

***Physodactylus* Fischer, 1823c**: 302. Current status: valid genus in ELATERIDAE: PHYSODACTYLINAE.

Type species: *Physodactylushenningii* Fischer, 1823, by monotypy.

Family-group name: PHYSODACTYLIDES Lacordaire, 1857. Stem: *Physodactyl*-.

***Physodera* Eschscholtz, 1829c**: 8. Current status: valid genus in CARABIDAE: HARPALINAE: LEBIINI: PHYSODERINA.

Type species: *Physoderadejeanii* Eschscholtz, 1829, by monotypy.

Family-group name: PHYSODÉRIDES Chaudoir, 1877. Stem: *Physoder*-. Note: senior homonym of PHYSODERINAE N.C.E. Miller, 1954 (type genus *Physoderes* Westwood, 1846) in Hemiptera.

***Physogaster* Lacordaire, 1830a**: 276. Current status: valid genus in TENEBRIONIDAE: PIMELIINAE: PHYSOGASTERINI.

Type species: *Physogastermendocina* Lacordaire, 1830, by monotypy.

Family-group name: PHYSOGASTÉRIDES Lacordaire, 1859. Stem: *Physogaster*-.

***Physognathus* Solier, 1849**: 303. Current status: invalid senior synonym of *Solierius* Bernhauer, 1921 in STAPHYLINIDAE: SOLIERIINAE. Note: junior homonym of *Physognathus* Agassiz, 1846 (unjustified emendation of *Physignathus* Cuvier, 1829) [Reptilia].

Type species: *Physognathusobscurus* Solier, 1849, by monotypy.

Family-group name: FISOGNATITOS Solier, 1849. Stem: *Physognath*-. Note: family-group name permanently invalid (ICZN 1999a, Article 39).

***Physonota* Boheman, 1854**: 190. Current status: valid genus in CHRYSOMELIDAE: CASSIDINAE: ISCHYROSONYCHINI. Note: this genus was first made available by Chevrolat in Dejean (1836a: 374) in another sense, with *Cassidafuscata* Klug, 1829 as type species by monotypy; to promote nomenclatural stability a request should be submitted to the Commission to reject the name *Physonota* Chevrolat, 1836 for the Principles of Priority and Homonymy (see Bousquet and Bouchard 2013a: 129).

Type species: *Physonotaalutacea* Boheman, 1854, by subsequent designation (Hincks 1952: 336).

Family-group name: PHYSONOTITAE Spaeth, 1942. Stem: *Physonot*-.

***Physorhinus* Eschscholtz in Laporte, 1838**: table. Current status: valid genus in ELATERIDAE: ELATERINAE: PHYSORHININI.

Type species: *Elatererythrocephalus* Fabricius, 1801, by subsequent designation (Lacordaire 1857: 176). Note: no species were originally included in this genus by Eschscholtz in Laporte (1838); the first ones were the three species described or mentioned by Germar (1840a: 245; 1840b: 439, 440).

Family-group name: PHYSORHINITES Candèze, 1859. Stem: *Physorhin*-.

***Phytobaenus* R.F. Sahlberg, 1834**: 9. Current status: valid genus in ADERIDAE.

Type species: *Phytobaenusamabilis* R.F. Sahlberg, 1834, by monotypy.

Family-group name: PHYTOBAENINI Báguena Corella, 1948. Stem: *Phytobaen*-.

***Phytobius* Schönherr, 1833**: 20. Current status: valid genus in CURCULIONIDAE: CEUTORHYNCHINAE: PHYTOBIINI. Note: new replacement name for *Hydaticus* Schönherr, 1825, which was placed on the Official Index of Rejected and Invalid Generic Names in Zoology and *Phytobius* Schönherr, 1833 was placed on the Official List of Generic Names in Zoology (ICZN 2001); the name *Phytobius* Dejean, 1835 had previously been placed on the Official List of Generic Names in Zoology (ICZN 1989b) but this entry was deleted from the Official List in the more recent ruling (ICZN 2001).

Type species: *Rhynchaenusmyriophylli* Gyllenhal, 1813 (= *Curculioleucogaster* Marsham, 1802), by original designation (see ICZN 2001) (type fixation for *Hydaticus* Schönherr). Note: *Curculioleucogaster* Marsham, 1802 was placed on the Official List of Specific Names in Zoology (ICZN 2001).

Family-group name: PHYTOBIIDAE Gistel, 1848. Stem: *Phytobi*-.

***Phytoecia* Dejean, 1835**: 351. Current status: valid genus in CERAMBYCIDAE: LAMIINAE: SAPERDINI.

Type species: *Cerambyxcylindricus* Linnaeus, 1758, by subsequent designation (C.G. Thomson 1859: 153).

Family-group name: PHYTOECIAIRES Mulsant, 1839. Stem: *Phytoeci*-.

***Phytonomus* Schönherr, 1823**: 1143. Current status: junior synonym of *Hypera* Germar, 1817 in CURCULIONIDAE: HYPERINAE: HYPERINI: CEPURINA. Note: unnecessary new replacement name for *Hypera* Germar, 1817.

Type species: *Curculionigrirostris* Fabricius, 1775, by subsequent designation (Samouelle 1819: 205) (type fixation for *Hypera* Germar).

Family-group name: PHYTONOMIDAE Gistel, 1848. Stem: *Phytonom*-. Note: a number of authors (see Casalini and Colonnelli 2023 and references therein) have treated PHYTONOMINAE and PHYTONOMINI as valid instead of HYPERINAE and HYPERINI; we prefer to conserve usage of HYPERINAE and HYPERINI as valid (ICZN 1999a, Article 40.2, see Bouchard et al. 2011: 619).

***Phytoscaphus* Schönherr, 1826**: 210. Current status: valid genus in CURCULIONIDAE: ENTIMINAE: CYPHICERITAE: CYPHICERINI: PHYTOSCAPHINA.

Type species: *Phytoscaphuslixabundus* Schönherr, 1826 (= *Curculiolanatus* Fabricius, 1801), by original designation.

Family-group name: PHYTOSCAPHIDES Lacordaire, 1863. Stem: *Phytoscaph*-.

***Phytosus* Curtis, 1838a**: pl. 718. Current status: valid genus in STAPHYLINIDAE: ALEOCHARINAE: PHYTOSINI.

Type species: *Phytosusspinifer* Curtis, 1838, by original designation.

Family-group name: PHYTOSIDES C.G. Thomson, 1867. Stem: *Phytos*-.

***Phytotera* Gistel, 1856**: 366. Current status: junior synonym of *Trachys* Fabricius, 1801 in BUPRESTIDAE: AGRILINAE: TRACHEINI: TRACHEINA.

Type species: *Buprestisminuta* Linnaeus, 1758, by subsequent designation (Bellamy 2003: 11). Note: name placed on the Official List of Specific Names in Zoology (ICZN 2009a).

Family-group name: PHYTOTERADAE Gistel, 1856. Stem: *Phytoter*-.

***Phyxelis* Schönherr, 1842b**: 122. Current status: valid genus in CURCULIONIDAE: ENTIMINAE: ENTIMITAE: BYRSOPAGINI: PHYXELIDINA.

Type species: *Barynotusrigidus* Say, 1832, by original designation.

Family-group name: PHYXELES G.H. Horn, 1876. Stem: *Phyxelid*-.

***Piazomias* Schönherr, 1840a**: 936. Current status: valid genus in CURCULIONIDAE: ENTIMINAE: POLYDRUSITAE: TANYMECINI: PIAZOMIINA.

Type species: *Piazomiasvirescens* Boheman, 1840, by original designation.

Family-group name: PIAZOMIINI Reitter, 1913. Stem: *Piazomi*-.

***Piazorhinus* Schönherr, 1835**: 471. Current status: valid genus in CURCULIONIDAE: CURCULIONINAE: PIAZORHININI.

Type species: *Attelabusscutellaris* Say, 1826, by original designation.

Family-group name: PIAZORHINIDES Lacordaire, 1863. Stem: *Piazorhin*-.

***Piazurus* Schönherr, 1825**: 586. Current status: valid genus in CURCULIONIDAE: CONODERINAE: PIAZURINI.

Type species: *Poecilmastipitosum* Germar, 1823, by original designation.

Family-group name: PIAZURIDES Lacordaire, 1865. Stem: *Piazur*-.

***Picrotus* Sharp, 1886**: 394. Current status: valid genus in CRYPTOPHAGIDAE: CRYPTOPHAGINAE: PICROTINI.

Type species: *Picrotusthoracicus* Sharp, 1886, by monotypy.

Family-group name: PICROTINAE Sen Gupta & Crowson, 1971. Stem: *Picrot*-.

***Pidonia* Mulsant, 1863**: 570. Current status: valid genus in CERAMBYCIDAE: LEPTURINAE: PIDONIINI.

Type species: *Lepturalurida* Fabricius, 1793, by subsequent designation (Swaine and Hopping 1928: 12).

Family-group name: PIDONIINI Zamoroka, 2022: 308. Stem: *Pidoni*-.

***Piesarthrius* Hope, 1834a**: 107. Current status: valid genus in CERAMBYCIDAE: CERAMBYCINAE: PIESARTHRIINI.

Type species: *Piesarthriusmarginellus* Hope, 1834, by original designation.

Family-group name: PIESARTHRINI McKeown, 1947. Stem: *Piesarthri*-.

***Piesocorynus* Dejean, 1834**: 235. Current status: valid genus in ANTHRIBIDAE: ANTHRIBINAE: PIESOCORYNINI.

Type species: *Eupariusdispar* Gyllenhal, 1833, by monotypy.

Family-group name: PIESOCORYNINI B.D. Valentine, 1960. Stem: *Piesocoryn*-.

***Piestus* Gravenhorst, 1806**: 223. Current status: valid genus in STAPHYLINIDAE: PIESTINAE.

Type species: *Piestussulcatus* Gravenhorst, 1806, by monotypy.

Family-group name: PIESTINI Erichson, 1839. Stem: *Piest*-.

***Piezocera* Audinet-Serville, 1834b**: 92. Current status: valid genus in CERAMBYCIDAE: CERAMBYCINAE: PIEZOCERINI: PIEZOCERINA.

Type species: *Piezocerabivittata* Audinet-Serville, 1834, by monotypy.

Family-group name: PIÉZOCÉRIDES Lacordaire, 1868. Stem: *Piezocer*-.

***Piezophyllus* Hope, 1842c**: 76. Current status: valid genus in ELATERIDAE: TETRALOBINAE: PIEZOPHYLLINI.

Type species: *Tetralobusrobustus* Hope, 1842 (= *Tetralobusmacrocerus* Laporte, 1838), by original designation.

Family-group name: PIEZOPHYLLINI Laurent, 1967. Stem: *Piezophyll*-.

***Piezotrachelus* Schönherr, 1839**: 365. Current status: valid genus in BRENTIDAE: APIONINAE: APIONITAE: PIEZOTRACHELINI.

Type species: *Piezotrachelusgermarii* Gyllenhal, 1839, by original designation.

Family-group name: PIEZOTRACHELINI Voss, 1959. Stem: *Piezotrachel*-.

***Pilinurgus* Burmeister, 1842**: 658. Current status: valid genus in SCARABAEIDAE: CETONIINAE: CREMASTOCHEILINI: PILINURGINA.

Type species: *Cremastocheilushirtus* Gory & Percheron, 1833, by monotypy.

Family-group name: PILINURGINA Krikken, 1984. Stem: *Pilinurg*-.

***Pilipalpus* Fairmaire, 1876**: 384. Current status: valid genus in PYROCHROIDAE: PILIPALPINAE.

Type species: *Pilipalpusdasytoides* Fairmaire, 1876, by monotypy.

Family-group name: PILIPALPINI M. Abdullah, 1964. Stem: *Pilipalp*-.

***Pilolabus* Jekel, 1860**: 196. Current status: valid genus in ATTELABIDAE: ATTELABINAE: PILOLABINI.

Type species: *Attelabusklugii* Gyllenhal, 1839, by original designation.

Family-group name: PILOLABINI Voss, 1925. Stem: *Pilolab*-.

***Pimelia* Fabricius, 1775**: 251. Current status: valid genus in TENEBRIONIDAE: PIMELIINAE: PIMELIINI: PIMELIINA.

Type species: *Pimeliaangulata* Fabricius, 1775, by subsequent designation (Hope 1841a: 118).

Family-group name: PIMELIARIAE Latreille, 1802. Stem: *Pimeli*-.

***Pimelopus* Erichson, 1842**: 159. Current status: valid genus in SCARABAEIDAE: DYNASTINAE: PENTODONTINI: PENTODONTINA.

Type species: *Pimelopusporcellus* Erichson, 1842, by monotypy.

Family-group name: PIMELOPODEA Burmeister, 1847. Stem: *Pimelopod*-.

***Pimidia* Rafinesque, 1815**: 113. Current status: junior synonym of *Pimelia* Fabricius, 1775 in TENEBRIONIDAE: PIMELIINAE: PIMELIINI: PIMELIINA. Note: unnecessary new replacement name for *Pimelia* Fabricius, 1775.

Type species: *Pimeliaangulata* Fabricius, 1775, by subsequent designation (Hope 1841a: 118) (type fixation for *Pimelia* Fabricius).

Family-group name: PIMIDINIA Rafinesque, 1815. Stem: *Pimidi*-.

***Pinodytes* G.H. Horn, 1881**: 248. Current status: junior synonym of *Catopocerus* Motschulsky, 1869 in LEIODIDAE: CATOPOCERINAE: CATOPOCERINI.

Type species: *Catopscryptophagoides* Mannerheim, 1852, by monotypy.

Family-group name: PINODYTINI G.H. Horn, 1881. Stem: *Pinodyt*-.

***Pinophilus* Gravenhorst, 1802**: 201. Current status: valid genus in STAPHYLINIDAE: PAEDERINAE: PINOPHILINI: PINOPHILINA.

Type species: *Pinophiluslatipes* Gravenhorst, 1802, by monotypy.

Family-group name: PINOPHILINIFORMES Nordmann, 1837. Stem: *Pinophil*-.

***Pinotus* Erichson, 1847c**: 108. Current status: junior synonym of *Dichotomius* Hope, 1838 in SCARABAEIDAE: SCARABAEINAE: DICHOTOMIINI.

Type species: *Pinotustalaus* Erichson, 1847, by **present designation**.

Family-group name: PINOTINAE Kolbe, 1905. Stem: *Pinot*-.

***Piochardia* Heyden, 1870**: 75. Current status: valid genus in STAPHYLINIDAE: ALEOCHARINAE: ALEOCHARINI: ALEOCHARINA.

Type species: *Piochardialepismiformis* Heyden, 1870, by monotypy.

Family-group name: PIOCHARDIAE Fenyes, 1919. Stem: *Piochardi*-.

***Pisenus* Casey, 1900**: 167, 171. Current status: valid genus in TETRATOMIDAE: PISENINAE.

Type species: *Cryptophagushumeralis* W. Kirby, 1837, by subsequent designation (Nikitsky 1989: 27).

Family-group name: PISENINI Miyatake, 1960. Stem: *Pisen*-.

***Pissodes* Germar, 1817b**: 340. Current status: valid genus in CURCULIONIDAE: MOLYTINAE: PISSODINI: PISSODINA.

Type species: *Curculiopini* Linnaeus, 1758, by subsequent designation (Schönherr 1825: 582).

Family-group name: PISSODISIDAE Gistel, 1848. Stem: *Pissod*-.

***Pithocles* J. Thomson, 1864**: 291. Current status: junior synonym of *Derobrachus* Audinet-Serville, 1832 in CERAMBYCIDAE: PRIONINAE: PRIONINI.

Type species: *Derobrachusprocerus* J. Thomson, 1860, by original designation.

Family-group name: PITHOCLITAE J. Thomson, 1864. Stem: *Pithocl*-.

***Pityobius* J.L. LeConte, 1853f**: 428. Current status: valid genus in ELATERIDAE: PITYOBIINAE: PITYOBIINI.

Type species: *Pityobiusanguinus* J.L. LeConte, 1853, by monotypy.

Family-group name: PITYOBINI Hyslop, 1917. Stem: *Pityobi*-.

***Pityogenes* Bedel, 1888**: 397. Current status: valid genus in CURCULIONIDAE: SCOLYTINAE: IPINI: IPINA.

Type species: *Dermesteschalcographus* Linnaeus, 1761 [as *calcographus*], by original designation. Note: the original spelling of the type species is *calcographus* but the incorrect subsequent spelling *chalcographus* is in prevailing usage, attributed to the publication of the original spelling, and therefore deemed to be the correct original spelling (ICZN 1999a, Article 33.3.1).

Family-group name: PITYOGENINA Balachowsky, 1949. Stem: *Pityogen*-.

***Pityophagus* Shuckard, 1839**: 171. Current status: valid genus in NITIDULIDAE: CRYPTARCHINAE: CRYPTARCHINI.

Type species: *Dermestesferrugineus* Linnaeus, 1761, by monotypy.

Family-group name: PITYOPHAGINI Schilsky, 1888. Stem: *Pityophag*-.

***Pityophthorus* Eichhoff, 1864**: 39, 45. Current status: valid genus in CURCULIONIDAE: SCOLYTINAE: CORTHYLINI: PITYOPHTHORINA.

Type species: *Bostrichuslichtensteinii* Ratzeburg, 1837, by subsequent designation (Hopkins 1914: 127).

Family-group name: PITYOPHTHORIDAE Eichhoff, 1878. Stem: *Pityophthor*-.

***Placonycha* G.H. Horn, 1880a**: 111. Current status: junior synonym of *Eubrianax* Kiesenwetter, 1874 in PSEPHENIDAE: EUBRIANACINAE.

Type species: *Dicranopselaphusedwardsii* J.L. LeConte, 1874, by monotypy.

Family-group name: PLACONYCHINI G.H. Horn, 1880. Stem: *Placonych*-.

***Placusa* Erichson, 1837**: 370. Current status: valid genus in STAPHYLINIDAE: ALEOCHARINAE: PLACUSINI.

Type species: *Aleocharapumilio* Gravenhorst, 1802, by monotypy.

Family-group name: PLACUSATES Mulsant & Rey, 1871. Stem: *Placus*-.

***Plagiarthrina* Keys, 1920**: 131. Current status: junior synonym of *Philhygra* Mulsant & Rey, 1873 in STAPHYLINIDAE: ALEOCHARINAE: ATHETINI: ATHETINA.

Type species: *Metaxyafordhamiana* Keys, 1920 (= *Aleocharaterminalis* Gravenhorst, 1806), by monotypy.

Family-group name: PLAGIARTHRINI Cameron, 1926. Stem: *Plagiarthrin*-.

***Planetes* W.S. MacLeay, 1825**: 28. Current status: valid genus in CARABIDAE: HARPALINAE: GALERITINI: PLANETINA.

Type species: *Planetesbimaculatus* W.S. MacLeay, 1825, by monotypy.

Family-group name: PLANETINI Jedlička, 1941. Stem: *Planet*-.

***Planeustomus* Jacquelin du Val, 1857b**: 58. Current status: valid genus in STAPHYLINIDAE: OXYTELINAE: PLANEUSTOMINI.

Type species: *Acrognathuspalpalis* Erichson, 1839, by monotypy.

Family-group name: PLANEUSTOMITES Jacquelin du Val, 1857. Stem: *Planeustom*-.

***Plastocerus* Schaum, 1852**: 49. Current status: valid genus in ELATERIDAE: DENDROMETRINAE: PLASTOCERINI.

Type species: *Callirhipisangulosus* Germar, 1844, by monotypy.

Family-group name: PLASTOCERIDAE Crowson, 1972. Stem: *Plastocer*-.

***Platamodes* Ménétriés, 1849b**: 233. Current status: valid genus in TENEBRIONIDAE: PIMELIINAE: STENOSINI: PLATAMODINA.

Type species: *Platamodesdentipes* Ménétriés, 1849, by monotypy.

Family-group name: PLATAMODINA Reitter, 1900. Stem: *Platamod*-.

***Platandria* Casey, 1893**: 345. Current status: valid genus in STAPHYLINIDAE: ALEOCHARINAE: HOPLANDRIINI: PLATANDRIINA.

Type species: *Platandriamormonica* Casey, 1893, by monotypy.

Family-group name: PLATANDRIINA Hanley, 2002. Stem: *Platandri*-.

***Platerodrilus* Pic, 1921**: 13. Current status: valid genus in LYCIDAE: PLATERODRILINAE.

Type species: *Platerodrilussinuatus* Pic, 1921, by subsequent designation (N.D. Riley 1923: 124).

Family-group name: PLATERODRILINI Kazantsev, 2004. Stem: *Platerodril*-.

***Plateros* Bourgeois, 1879**: xix. Current status: valid genus in LYCIDAE: LYCINAE: PLATERODINI.

Type species: *Erosbrasiliensis* P.H. Lucas, 1857, by subsequent designation (Zaragoza Caballero 1999: 2).

Family-group name: PLATERODINAE Kleine, 1929. Stem: *Platerod*-.

***Plateumaris* C.G. Thomson, 1859**: 154. Current status: valid genus in CHRYSOMELIDAE: DONACIINAE: PLATEUMARINI.

Type species: *Donacianigra* Fabricius, 1792 (= *Prionusbracatus* Scopoli, 1772), by original designation.

Family-group name: PLATEUMARINI Böving, 1922. Stem: *Plateumar*-.

***Platidiolus* Chaudoir, 1878b**: 77. Current status: valid genus in CARABIDAE: PATROBINAE: PATROBINI: PLATIDIOLINA.

Type species: *Platidiolusrufus* Chaudoir, 1878, by monotypy.

Family-group name: PLATIDIOLINI Zamotajlov & Lafer, 2001. Stem: *Platidiol*-.

***Platisus* Erichson, 1842**: 216. Current status: valid genus in CUCUJIDAE: PLATISINAE.

Type species: *Platisusobscurus* Erichson, 1842, by monotypy.

Family-group name: PLATISINAE Jin, Zwick, Ślipiński, Marris, Thomas & H. Pang, 2020a: 260. Stem: *Platis*-.

***Platyarcha* Kirejtshuk, 1987**: 78. Current status: valid genus in NITIDULIDAE: CRYPTARCHINAE: PLATYARCHINI.

Type species: *Platyarchapacta* Kirejtshuk, 1987, by original designation.

Family-group name: PLATYARCHINI Kirejtshuk, 1998. Stem: *Platyarch*-.

***Platyarthron* Guérin-Méneville, 1844a**: 230. Current status: valid genus in CERAMBYCIDAE: CERAMBYCINAE: PLATYARTHRINI.

Type species: *Platyarthronbilineatum* Guérin-Méneville, 1844, by monotypy.

Family-group name: PLATYARTHRINAE H.W. Bates, 1870. Stem: *Platyarthr*-.

***Platyauchenia* Sturm, 1843**: 358. Current status: valid genus in CHRYSOMELIDAE: CASSIDINAE: ALURNINI.

Type species: *Platyauchenialimbata* Sturm, 1843 (= *Cassidalatreillii* Laporte, 1840), by monotypy.

Family-group name: PLATYAUCHENIITAE Spaeth, 1929. Stem: *Platyaucheni*-.

***Platycephala* Montrouzier, 1861b**: 268. Current status: invalid senior synonym of *Aralius* Kuschel, 1990 in BELIDAE: OXYCORYNINAE: AGLYCYDERITAE: AGLYCYDERINI. Note: junior homonym of *Platycephala* Fallén, 1820 [Diptera].

Type species: *Platycephalaolivieri* Montrouzier, 1861, by monotypy.

Family-group name: PLATYCEPHALITAE Paulian, 1944. Stem: *Platycephal*-. Note: family-group name permanently invalid (ICZN 1999a, Article 39); junior homonym of PLATYCEPHALIDAE Swainson, 1839 (type genus *Platycephalus* Bloch, 1795) in Pisces.

***Platyceroides* Benesh, 1946**: 175. Current status: valid genus in LUCANIDAE: LUCANINAE: PLATYCEROIDINI.

Type species: *Platycerusagassii* J.L. LeConte, 1861, by original designation.

Family-group name: PLATYCEROIDINI Paulsen & Hawks, 2008. Stem: *Platyceroid*-.

***Platycerus* Geoffroy, 1762**: 59. Current status: valid genus in LUCANIDAE: LUCANINAE: PLATYCERINI. Note: name placed on the Official List of Generic Names in Zoology (ICZN 1994a).

Type species: *Scarabaeuscaraboides* Linnaeus, 1758, by subsequent designation (Latreille 1810: 429; see ICZN 1994a). Note: name placed on the Official List of Specific Names in Zoology (ICZN 1994a).

Family-group name: PLATYCÉRAIRES Mulsant, 1842. Stem: *Platycer*-.

***Platychile* W.S. MacLeay, 1825**: 9. Current status: valid genus in CICINDELIDAE: MANTICORINI.

Type species: *Manticorapallida* Fabricius, 1801, by monotypy.

Family-group name: PLATYCHILIDAE W. Horn, 1893. Stem: *Platychil*-.

***Platycholeus* G.H. Horn, 1881**: 254. Current status: valid genus in LEIODIDAE: CHOLEVINAE: LEPTODIRINI: PLATYCHOLEINA.

Type species: *Ptomaphagusleptinoides* Crotch, 1874, by monotypy.

Family-group name: PLATYCHOLEI G.H. Horn, 1881. Stem: *Platychole*-.

***Platycnemus* Nordmann, 1837**: 6, 135. Current status: junior synonym of *Haematodes* Laporte, 1835 in STAPHYLINIDAE: STAPHYLININAE: STAPHYLININI: XANTHOPYGINA. Note: name placed on the Official List of Generic Names in Zoology (ICZN 1996c).

Type species: *Platycnemuslateritius* Nordmann, 1837, by monotypy (see ICZN 1996c). Note: name placed on the Official List of Specific Names in Zoology (ICZN 1996c).

Family-group name: PLATYCNEMIDIFORMES Nordmann, 1837. Stem: *Platycnem*-.

***Platycoelia* Dejean, 1833b**: 154. Current status: valid genus in SCARABAEIDAE: RUTELINAE: ANOPLOGNATHINI: PLATYCOELIINA.

Type species: *Melolonthaflavostriata* Latreille, 1813, by monotypy.

Family-group name: PLATYCOELIIDAE Burmeister, 1844. Stem: *Platycoeli*-.

***Platycorynus* Chevrolat in Dejean, 1836a**: 413. Current status: valid genus in CHRYSOMELIDAE: EUMOLPINAE: EUMOLPINI.

Type species: *Eumolpuscompressicornis* Fabricius, 1801, by subsequent designation (Chapuis 1874: 339).

Family-group name: PLATYCORYNI L.N. Medvedev & Rybakova, 1985: 118. Stem: *Platycoryn*-. Note: this name was overlooked in Bouchard et al. (2011) and Bouchard and Bousquet (2020a); this family-group name was proposed as a new replacement name for CORYNODINI T.A. Marshall, 1865 because of the synonymy and invalidity of its type genus; however, a family-group name is not to be replaced on that account alone (ICZN 1999a, Article 40.1) unless the substitute name was proposed before 1961 and is in prevailing usage (ICZN 1999a, Article 40.2), which is not the case here.

***Platycrepidius* Candèze, 1859**: 159. Current status: valid genus in ELATERIDAE: AGRYPNINAE: PLATYCREPIDIINI. Note: name first proposed as a synonym of *Eudactylus* Sallé, 1855 and subsequently treated as valid (e.g., Fleutiaux 1934: 181) before 1961 (see ICZN 1999a, Article 11.6).

Type species: *Eudactyluswapleri* Sallé, 1855, by subsequent designation (Hyslop 1921: 665).

Family-group name: PLATYCREPIDIINI C. Costa & Casari-Chen, 1993. Stem: *Platycrepidi*-.

***Platydascillus* Everts, 1909**: 6. Current status: valid genus in BYTURIDAE: PLATYDASCILLINAE.

Type species: *Platydascillussumatranus* Everts, 1909, by monotypy.

Family-group name: PLATYDASCILLIDAE Pic, 1914. Stem: *Platydascill*-.

***Platydema* Laporte & Brullé, 1831**: 332, 350. Current status: valid genus in TENEBRIONIDAE: DIAPERINAE: DIAPERINI: DIAPERINA.

Type species: *Diaperisviolacea* Fabricius, 1790, by subsequent designation (Westwood 1838c: 32).

Family-group name: PLATYDEMINAE Reitter, 1917. Stem: *Platydem*-.

***Platygenia* W.S. MacLeay, 1819**: 151. Current status: valid genus in SCARABAEIDAE: CETONIINAE: PLATYGENIINI.

Type species: *Platygeniazairica* W.S. MacLeay, 1819 (= *Trichiusbarbatus* Afzelius, 1817), by monotypy.

Family-group name: PLATYGENIINI Krikken, 1984. Stem: *Platygeni*-.

***Platygnathus* Audinet-Serville, 1832**: 150. Current status: valid genus in CERAMBYCIDAE: PRIONINAE: MACROTOMINI: PLATYGNATHINA.

Type species: *Prionusoctangularis* G.-A. Olivier, 1795, by subsequent designation (Laporte 1840b: 404).

Family-group name: PLATYGNATHINA Gilmour, 1954. Stem: *Platygnath*-.

***Platynaspis* L. Redtenbacher, 1843**: 11, 15. Current status: valid genus in COCCINELLIDAE: COCCINELLINAE: PLATYNASPINI.

Type species: *Coccinellabisbipustulata* Fabricius, 1792 (= *Coccinellaluteorubra* Goeze, 1777), by monotypy. Note: Goeze’s (1777) work is not consistently binominal (see ICZN 2021a) and therefore an application to the Commission is necessary to retain *Coccinellaluteorubra* Goeze, 1777 as the valid name for the type species.

Family-group name: PLATYNASPIAIRES Mulsant, 1846. Stem: *Platynasp*-.

***Platynectes* Régimbart, 1879**: 462. Current status: valid genus in DYTISCIDAE: AGABINAE: PLATYNECTINI. Note: name placed on the Official List of Generic Names in Zoology (ICZN 1995a).

Type species: *Agabusdecemnotatus* Aubé, 1838, by subsequent designation (Guignot 1946: 117; see ICZN 1995a). Note: name placed on the Official List of Specific Names in Zoology (ICZN 1995a).

Family-group name: PLATYNECTINI Toussaint & Balke in Toussaint et al., 2017: 504. Stem: *Platynect*-. Note: family-group name used as valid in recent publications but it is not an available name since it is not accompanied by a description or definition that states in words the characters that are purported to differentiate the taxon (ICZN 1999a, Article 13.1); in order to promote stability, we believe that a request should be submitted to the Commission to accept this family-group name as available from its first date of publication.

***Platynoptera* Chevrolat, 1834**: [18] 1. Current status: valid genus in CLERIDAE: KORYNETINAE.

Type species: *Platynopteralyciformis* Chevrolat, 1834, by monotypy.

Family-group name: PLATYNOPTÉROÏDES Spinola, 1844. Stem: *Platynopter*-.

***Platynotus* Fabricius, 1801a**: 138. Current status: valid genus in TENEBRIONIDAE: BLAPTINAE: PLATYNOTINI: PLATYNOTINA.

Type species: *Blaps excavata* Fabricius, 1775, by subsequent designation (Hope 1841a: 110).

Family-group name: PLATYNOTAIRES Mulsant & Rey, 1853. Stem: *Platynot*-.

***Platynus* Bonelli, 1810**: Tab. Syn. Current status: valid genus in CARABIDAE: HARPALINAE: PLATYNINI.

Type species: *Carabusangusticollis* Fabricius, 1801 (= *Carabusassimilis* Paykull, 1790), by subsequent monotypy (Germar 1817a: 303). Note: *Platynuscomplanatus* Dejean, 1831 is sometimes listed as the type species of this genus (e.g., J. Schmidt 2017: 666) but is not an originally included nominal species in Germar (1817a) and thus cannot be the type species of *Platynus* Bonelli, 1810 (ICZN 1999a, Article 67.2.2).

Family-group name: PLATYNII Bonelli, 1810. Stem: *Platyn*-.

***Platyomus* C.R. Sahlberg, 1823a**: 29. Current status: valid genus in CURCULIONIDAE: ENTIMINAE: POLYDRUSITAE: NAUPACTINI: PLATYOMINA. Note: we consider *Platyomus* used by Schönherr (1823: 1140) a subsequent usage of *Platyomus* C.R. Sahlberg, 1823.

Type species: *Platyomusnodipennis* C.R. Sahlberg, 1823, by monotypy.

Family-group name: PLATYOMINA Champion, 1911. Stem: *Platyom*-.

***Platyope* Fischer, 1820**: pl. 15. Current status: valid genus in TENEBRIONIDAE: PIMELIINAE: PIMELIINI: PIMELIINA.

Type species: *Tenebrioleucographus* Pallas, 1773, by subsequent designation (Jacquelin du Val 1860b: 261). Note: Pallas (1773) used two different spellings for this species, *Tenebrioleucographus* (p. 474) and *Tenebrioleucogrammus* (p. 719); subsequently, Pallas (1781: 54) used *T.leucographus* as the valid name of the species and therefore acted as First Reviser (ICZN 1999a, Article 24.2.4).

Family-group name: PLATYOPIDAE Semenov, 1893. Stem: *Platyop*-.

***Platypicerus* Nunberg, 1953**: 46. Current status: junior synonym of *Mitosoma* Chapuis, 1865 in CURCULIONIDAE: PLATYPODINAE: TESSEROCERINI: TESSEROCERINA.

Type species: *Platypicerushamatus* Nunberg, 1953, by monotypy.

Family-group name: PLATYPICERINAE Nunberg, 1953. Stem: *Platypicer*-.

***Platyprosopus* Mannerheim, 1830**: 36. Current status: valid genus in STAPHYLINIDAE: STAPHYLININAE: PLATYPROSOPINI.

Type species: *Platyprosopuselongatus* Mannerheim, 1830, by monotypy.

Family-group name: PLATYPROSOPARIA Lynch Arribálzaga, 1884. Stem: *Platyprosop*-.

***Platypsyllus* Ritsema, 1869**: [23]. Current status: valid genus in LEIODIDAE: PLATYPSYLLINAE. Note: this taxon was also made proposed subsequently the same year by Westwood (1869b: 118).

Type species: *Platypsylluscastoris* Ritsema, 1869, by monotypy.

Family-group name: PLATYPSYLLIDAE Ritsema, 1869. Stem: *Platypsyll*-.

***Platypus* Herbst, 1793**: 128. Current status: valid genus in CURCULIONIDAE: PLATYPODINAE: PLATYPODINI.

Type species: *Bostrichuscylindrus* Fabricius, 1792, by monotypy.

Family-group name: PLATYPODIDAE Shuckard, 1839. Stem: *Platypod*-.

***Platyrhinus* Clairville, 1798**: 56, 112. Current status: valid genus in ANTHRIBIDAE: ANTHRIBINAE: ZYGAENODINI.

Type species: *Platyrhinuscostirostris* Clairville, 1798 (= *Curculioresinosus* Scopoli, 1763), by subsequent designation (Pierce 1930: 12).

Family-group name: PLATYRRHINIDAE Bedel, 1882. Stem: *Platyrhin*-. Note: senior homonym of PLATYRHINIDAE Jordan, 1923 (type genus *Platyrhina* J. Müller & Henle, 1838) in Pisces.

***Platyrhopalus* Westwood, 1833**: 615, 654. Current status: valid genus in CARABIDAE: PAUSSINAE: PAUSSINI: PAUSSINA.

Type species: *Paussusdenticornis* Donovan, 1804, by original designation.

Family-group name: PLATYRHOPALINI Jeannel, 1946. Stem: *Platyrhopal*-.

***Platyscelis* Latreille, 1818**: 23. Current status: valid genus in TENEBRIONIDAE: BLAPTINAE: PLATYSCELIDINI. Note: name placed on the Official List of Generic Names in Zoology (ICZN 1993f).

Type species: *Tenebriohypolithus* Pallas, 1781, by plenary power (see ICZN 1993f). Note: name placed on the Official List of Specific Names in Zoology (ICZN 1993f).

Family-group name: PLATYSCÉLIDES Lacordaire, 1859. Stem: *Platyscelid*-.

***Platysma* Bonelli, 1810**: Tab. Syn. Current status: valid genus in CARABIDAE: HARPALINAE: PTEROSTICHINI: PTEROSTICHINA.

Type species: *Carabusniger* Schaller, 1783, by subsequent designation (Stephens 1835b: 414). Note: No species were originally included in this genus by Bonelli (1810); the first ones were the two species listed by Panzer (1813: 55).

Family-group name: PLATYSMATINI Tschitschérine, 1899. Stem: *Platysmat*-.

***Platysoma* Leach, 1817**: 77. Current status: valid genus in HISTERIDAE: HISTERINAE: PLATYSOMATINI.

Type species: *Histerdepressus* Fabricius, 1787 (= *Histercompressus* Herbst, 1783), by subsequent designation (Stephens 1835b: 415).

Family-group name: PLATYSOMINI Bickhardt, 1914. Stem: *Platysomat*-.

***Platyspartus* Faust, 1898b**: 235, 247. Current status: valid genus in CURCULIONIDAE: ENTIMINAE: OTIORHYNCHITAE: CELEUTHETINI: CELEUTHETINA.

Type species: *Piezonotuslatiscapus* Heller, 1896, by original designation.

Family-group name: PLATYSPARTINA Voss, 1940: 279. Stem: *Platyspart*-. Note: this name was overlooked in Bouchard et al. (2011) and Bouchard and Bousquet (2020a).

***Platysternus* Dejean, 1835**: 336. Current status: valid genus in CERAMBYCIDAE: LAMIINAE: ANISOCERINI.

Type species: *Cerambyxhebraeus* Fabricius, 1781, by monotypy.

Family-group name: PLATYSTERNIDES Lacordaire, 1872. Stem: *Platystern*-. Note: junior homonym of PLATYSTERNIDAE J.E. Gray, 1869 (type genus *Platysternon* J.E. Gray, 1831) in Reptilia.

***Platystomos* D.H. Schneider in Fabricius, 1791**: 21. Current status: valid genus in ANTHRIBIDAE: ANTHRIBINAE: PLATYSTOMINI.

Type species: *Curculioalbinus* Linnaeus, 1758, by subsequent designation (Pierce 1916: 463).

Family-group name: PLATYSTOMOIDEA Pierce, 1916. Stem: *Platystom*-.

***Platytarsulus* Schedl, 1935**: 632. Current status: valid genus in CURCULIONIDAE: PLATYPODINAE: TESSEROCERINI: TESSEROCERINA.

Type species: *Platytarsulusmarshalli* Schedl, 1935, by original designation.

Family-group name: PLATYTARSILIDAE Schedl, 1939. Stem: *Platytarsul*-.

***Platyxantha* Baly, 1864a**: 233. Current status: junior synonym of *Taumacera* Thunberg, 1814 in CHRYSOMELIDAE: GALERUCINAE: LUPERINI.

Type species: *Platyxanthaapicalis* Baly, 1864, by original designation.

Family-group name: PLATYXANTHITES Chapuis, 1875. Stem: *Platyxanth*-.

***Plaumanniola* Costa Lima, 1962**: 415. Current status: valid genus in STAPHYLINIDAE: SCYDMAENINAE: SCYDMAENITAE: PLAUMANNIOLINI.

Type species: *Plaumanniolasanctaecatharinae* Costa Lima, 1962, by monotypy.

Family-group name: PLAUMANNIOLINAE Costa Lima, 1962. Stem: *Plaumanniol*-.

***Plectogaster* C.O. Waterhouse, 1881**: 429. Current status: valid genus in CERAMBYCIDAE: CERAMBYCINAE: PLECTOGASTERINI.

Type species: *Megacoeluspectinicornis* H.W. Bates, 1881, by subsequent designation (Quentin and Villiers 1969: 615).

Family-group name: PLECTOGASTERINI Quentin & Villiers, 1969. Stem: *Plectogaster*-.

***Plectris* Lepeletier & Audinet-Serville, 1828**: 369. Current status: valid genus in SCARABAEIDAE: MELOLONTHINAE: MACRODACTYLINI.

Type species: *Plectristomentosa* Lepeletier & Audinet-Serville, 1828, by original designation.

Family-group name: PLECTRIDAE Burmeister, 1855. Stem: *Plectr*-.

***Plectromerus* Haldeman, 1847**: 43. Current status: valid genus in CERAMBYCIDAE: CERAMBYCINAE: PLECTROMERINI.

Type species [suggested]: *Obriumdentatum* J.E. LeConte, 1824 (= *Callidiumdentipes* G.-A. Olivier, 1790), by subsequent designation (Linsley 1963: 135). Note: the valid type species fixation is that of J.L. LeConte (1873: 189), who selected *Plectromerusconcinnatus* Haldeman, 1847 (= *Curiusdentatus* Newman, 1840), but that nominal species is the type species of *Curius* Newman, 1840; although J.L. LeConte (1873: 189) listed *Curiusdentatus*, which is not an originally included nominal species, as type species of *Plectromerus*, the fact that he listed *Plectromerusconcinnatus* Haldeman, 1847, one of the two originally included species in *Plectromerus*, at the same time in synonymy with *Curiusdentatus* Newman, he is deemed to have designated *Plectromerusconcinnatus* Haldeman as type species of *Plectromerus* (Article 69.2.2) (see Bousquet et al. 2009: 53); an application to the Commission is necessary to fix *Obriumdentatum* J.E. LeConte, 1824 as the type species of *Plectromerus* Haldeman, 1847.

Family-group name: PLECTROMERINI Nearns & Braham, 2008. Stem: *Plectromer*-.

***Plectrophorus* Schönherr, 1826**: 106. Current status: invalid senior synonym of *Plectrophoroides* Wibmer & O’Brien, 1986 in CURCULIONIDAE: ENTIMINAE: POLYDRUSITAE: NAUPACTINI: NAUPACTINA. Note: junior homonym of *Plectrophorus* Férussac, 1819 [Mollusca].

Type species: *Leptoceruslutra* Schönherr, 1826, by original designation.

Family-group name: PLECTROPHORINA Voss, 1954. Stem: *Plectrophor*-. Note: family-group name permanently invalid (ICZN 1999a, Article 39).

***Plectroscelis* Chevrolat in Dejean, 1836a**: 393. Current status: junior synonym of *Chaetocnema* Stephens, 1831 in CHRYSOMELIDAE: GALERUCINAE: ALTICINI.

Type species: *Alticaaridula* Gyllenhal, 1827, by subsequent designation (Monrós and Bechyné 1956: 1134).

Family-group name: PLECTROSCELIDES C.G. Thomson, 1866. Stem: *Plectroscelid*-.

***Plegaderus* Erichson, 1834**: 203. Current status: valid genus in HISTERIDAE: ABRAEINAE: PLEGADERINI.

Type species: *Histercaesus* Herbst, 1791, by subsequent designation (Hope 1841a: 105).

Family-group name: PLEGADERINI Portevin, 1929. Stem: *Plegader*-.

***Pleganophorus* Hampe, 1855**: 97. Current status: valid genus in ENDOMYCHIDAE: PLEGANOPHORINAE.

Type species: *Pleganophorusbispinosus* Hampe, 1855, by monotypy.

Family-group name: PLÉGANOPHORIDES Jacquelin du Val, 1858. Stem: *Pleganophor*-.

***Pleiarthrocerus* Bruch, 1915**: 340. Current status: valid genus in CERAMBYCIDAE: CERAMBYCINAE: PLEIARTHROCERINI.

Type species: *Pleiarthrocerusopacus* Bruch, 1915, by original designation.

Family-group name: PLEIARTHROCERINAE Lane, 1950. Stem: *Pleiarthrocer*-.

***Pleocoma* J.L. LeConte, 1856a**: 24. Current status: valid genus in PLEOCOMIDAE: PLEOCOMINAE.

Type species: *Pleocomafimbriata* J.L. LeConte, 1856, by monotypy.

Family-group name: PLEOCOMINI J.L. LeConte, 1861. Stem: *Pleocom*-.

***Pleonomus* Ménétriés, 1849a**: 48. Current status: valid genus in ELATERIDAE: DENDROMETRINAE: PLEONOMINI.

Type species: *Pleonomustereticollis* Ménétriés, 1849, by subsequent designation (Lacordaire 1857: 222).

Family-group name: PLEONOMINI Semenov-Tjan-Shansky & Pjatakova, 1936. Stem: *Pleonom*-.

***Pleotomus* J.L. LeConte, 1861**: 184. Current status: valid genus in LAMPYRIDAE: LAMPYRINAE: PLEOTOMINI.

Type species: *Pleotomuspallens* J.L. LeConte, 1865, by subsequent monotypy (J.L. LeConte 1865: 88).

Family-group name: PLEOTOMINI Summers, 1874. Stem: *Pleotom*-.

***Pleroticus* Péringuey, 1896**: 521. Current status: valid genus in CARABIDAE: HARPALINAE: CHLAENIINI: CHLAENIINA.

Type species: *Vertaguslucidulus* Boheman, 1848, by monotypy.

Family-group name: PLEROTICINI Basilewsky, 1950. Stem: *Plerotic*-.

***Plesioclytus* Giesbert, 1993**: 129. Current status: valid genus in CERAMBYCIDAE: CERAMBYCINAE: PLESIOCLYTINI.

Type species: *Plesioclytusrelictus* Giesbert, 1993, by original designation.

Family-group name: PLESIOCLYTINI Wappes & Skelley, 2015: 2. Stem: *Plesioclyt*-.

***Pleurarius* Kaup, 1868b**: 1. Current status: valid genus in PASSALIDAE: PASSALINAE: MACROLININI.

Type species: *Pleurariuspilipes* Kaup, 1868, by monotypy.

Family-group name: PLEURARIINAE Kuwert, 1896. Stem: *Pleurari*-.

***Pleurolabus* Jekel, 1860**: 197. Current status: valid genus in ATTELABIDAE: ATTELABINAE: ATTELABINI: PLEUROLABINA.

Type species: *Rhynchitesexaratus* Boheman, 1828, by original designation.

Family-group name: PLEUROLABINA Legalov, 2003. Stem: *Pleurolab*-.

***Pleurophorus* Mulsant, 1842a**: 312. Current status: valid genus in SCARABAEIDAE: APHODIINAE: PSAMMODIINI: PSAMMODIINA.

Type species: *Scarabaeuscaesus* Panzer, 1796, by monotypy. Note: see Branco (2007: 19) for comments about the authorship of this species-group name.

Family-group name: PLEUROPHORATES Mulsant, 1842. Stem: *Pleurophor*-. Note: senior homonym of PLEUROPHORIDAE Weller, 1886 (type genus *Pleurophorus* King, 1844) in Mollusca; Weller’s name is permanently invalid (based on a preoccupied type genus).

***Pleuropterus* Westwood, 1841a**: 11. Current status: invalid senior synonym of *Heteropaussus* J. Thomson, 1860 in CARABIDAE: PAUSSINAE: PAUSSINI: HETEROPAUSSINA. Note: junior homonym of *Pleuropterus* Burnett, 1829 [Mammalia].

Type species: *Cerapteruswestermanni* Westwood, 1841, by monotypy.

Family-group name: PLEUROPTERINI Wasmann, 1920. Stem: *Pleuropter*-. Note: family-group name permanently invalid (ICZN 1999a, Article 39).

***Plinthus* Germar, 1817b**: 340. Current status: valid genus in CURCULIONIDAE: MOLYTINAE: MOLYTINI: PLINTHINA. Note: name placed on the Official List of Generic Names in Zoology (ICZN 2012d).

Type species: *Curculiomegerlei* Panzer, 1803, by plenary power (see ICZN 2012d). Note: name placed on the Official List of Specific Names in Zoology (ICZN 2012d).

Family-group name: PLINTHIDES Lacordaire, 1863. Stem: *Plinth*-.

***Plocamotrechus* Jeannel, 1926**: 480, 525. Current status: junior synonym of *Pachydesus* Motschulsky, 1864 in CARABIDAE: TRECHINAE: TRECHINI: TRECHINA.

Type species: *Trechuspallipes* Boheman, 1848 (= *Plocamotrechusbohemani* Jeannel, 1926), by original designation.

Family-group name: PLOCAMOTRECHINA Jeannel, 1960. Stem: *Plocamotrech*-.

***Plocastes* Gistel, 1856**: 365. Current status: junior synonym of *Aesalus* Fabricius, 1801 in LUCANIDAE: AESALINAE: AESALINI.

Type species: *Lucanusscarabaeoides* Panzer, 1795, by monotypy.

Family-group name: PLOCASTEIDAE Gistel, 1856. Stem: *Plocast*-.

***Plochionus* Dejean, 1821**: 5. Current status: valid genus in CARABIDAE: HARPALINAE: LEBIINI: CALLEIDINA.

Type species: *Lebiabonfilsi* Audinet-Serville, 1821 (= *Carabuspallens* Fabricius, 1775), by monotypy.

Family-group name: PLOCHIONIDAE Gozis, 1875. Stem: *Plochion*-.

***Ploeosoma* Wollaston, 1854**: 147. Current status: valid genus in CERYLONIDAE: CERYLONINAE.

Type species: *Ploeosomaellipticum* Wollaston, 1854, by monotypy.

Family-group name: PLEOSOMIDES Fauvel, 1891. Stem: *Ploeosomat*-.

***Plotina* Lewis, 1896**: 35. Current status: valid genus in COCCINELLIDAE: COCCINELLINAE: PLOTININI.

Type species: *Plotinaversicolor* Lewis, 1896, by original designation.

Family-group name: PLOTININI Miyatake, 1994. Stem: *Plotin*-.

***Plusiotis* Burmeister, 1844**: 417. Current status: valid genus in SCARABAEIDAE: RUTELINAE: RUTELINI: RUTELINA.

Type species: *Pelidnotavictorina* Hope, 1841, by subsequent designation (Ohaus 1934: 16).

Family-group name: PLUSIOTINA H.W. Bates, 1888. Stem: *Plusiotid*-.

***Pocadius* Erichson, 1843a**: 318. Current status: valid genus in NITIDULIDAE: NITIDULINAE: NITIDULINI.

Type species: *Nitidulaferruginea* Fabricius, 1775, by subsequent designation (E. Desmarest 1851: 281).

Family-group name: POCADIINI Seidlitz, 1872 [Gtt.]. Stem: *Pocadi*-.

***Podabrocephalus* Pic, 1913**: 118. Current status: valid genus in PTILODACTYLIDAE: PTILODACTYLINAE.

Type species: *Podabrocephalussinuaticollis* Pic, 1913, by monotypy.

Family-group name: PODABROCEPHALIDAE Pic, 1930. Stem: *Podabrocephal*-.

***Podabrus* Dejean, 1833b**: 105. Current status: valid genus in CANTHARIDAE: CANTHARINAE: PODABRINI.

Type species: *Cantharisalpinus* Paykull, 1798, by subsequent designation (Stephens 1835a: 416).

Family-group name: PODABRIDAE Gistel, 1856. Stem: *Podabr*-.

***Podagrica* Chevrolat in Dejean, 1836a**: 394. Current status: valid genus in CHRYSOMELIDAE: GALERUCINAE: ALTICINI.

Type species: *Alticafuscipes* Fabricius, 1775, by subsequent designation (Maulik 1926: 273).

Family-group name: PODAGRICINI Bechyné, 1968. Stem: *Podagric*-.

***Podalgus* Burmeister, 1847**: 90, 117. Current status: valid genus in SCARABAEIDAE: DYNASTINAE: PENTODONTINI: PENTODONTINA. Note: name placed on the Official List of Generic Names in Zoology (ICZN 2004a).

Type species: *Podalguscuniculus* Burmeister, 1847, by subsequent designation (Arrow 1908: 341; see ICZN 2004a). Note: name placed on the Official List of Specific Names in Zoology (ICZN 2004a).

Family-group name: PODALGIDAE Burmeister, 1847. Stem: *Podalg*-.

***Podapion* C.V. Riley, 1883**: 62. Current status: valid genus in BRENTIDAE: APIONINAE: PODAPIITAE.

Type species: *Podapiongallicola* C.V. Riley, 1883, by monotypy.

Family-group name: PODAPIITAE Wanat, 2001. Stem: *Podapi*-.

***Podolasia* Harold, 1869b**: 122. Current status: valid genus in SCARABAEIDAE: PODOLASIINAE. Note: new replacement name for *Lasiopus* J.L. LeConte, 1856.

Type species: *Lasiopusferrugineus* J.L. LeConte, 1856, by monotypy (type fixation for *Lasiopus* J.L. LeConte).

Family-group name: PODOLASIINI Howden, 1997. Stem: *Podolasi*-.

***Podonta* Solier, 1835b**: 247. Current status: valid genus in TENEBRIONIDAE: ALLECULINAE: CTENIOPODINI.

Type species: *Cistelanigrita* Fabricius, 1794, by subsequent designation (Cazurro Ruiz 1894: 883).

Family-group name: PODONTINI Portevin, 1934. Stem: *Podont*-.

***Poecilonota* Eschscholtz, 1829b**: 9. Current status: valid genus in BUPRESTIDAE: CHRYSOCHROINAE: POECILONOTINI: POECILONOTINA. Note: name placed on the Official List of Generic Names in Zoology (ICZN 1996a).

Type species: *Buprestisvariolosa* Paykull, 1799, by plenary power (see ICZN 1996a). Note: name placed on the Official List of Specific Names in Zoology (ICZN 1996a).

Family-group name: POECILONOTINA Jakobson, 1913. Stem: *Poecilonot*-.

***Poecilopeplus* Dejean, 1835**: 319. Current status: valid genus in CERAMBYCIDAE: CERAMBYCINAE: TRACHYDERINI: TRACHYDERINA.

Type species: *Prionuscorallifer* Sturm, 1826, by monotypy.

Family-group name: POECILOPÉPLIDES Lacordaire, 1868. Stem: *Poecilopepl*-.

***Poecilus* Bonelli, 1810**: Tab. Syn. Current status: valid genus in CARABIDAE: HARPALINAE: PTEROSTICHINI: PTEROSTICHINA.

Type species: *Carabuscupreus* Linnaeus, 1758, by subsequent designation (Curtis 1827: pl. 187). Note: no species were originally included in this genus by Bonelli (1810); the first ones were the six species listed by Panzer (1813: 60, 61).

Family-group name: POECILII Bonelli, 1810. Stem: *Poecil*-.

***Poekilosoma* Audinet-Serville, 1832**: 184. Current status: valid genus in CERAMBYCIDAE: PRIONINAE: ANACOLINI.

Type species: *Prionusornatus* Dalman, 1823, by subsequent designation (J. Thomson 1864: 276).

Family-group name: POECILOSOMI H.W. Bates, 1869. Stem: *Poekilosomat*-.

***Pogonocerus* Fischer, 1812**: 281. Current status: valid genus in PYROCHROIDAE: POGONOCERINAE.

Type species: *Pogonocerusthoracicus* Fischer, 1812, by monotypy.

Family-group name: POGONOCERINAE Iablokoff-Khnzorian, 1985. Stem: *Pogonocer*-.

***Pogonocherus* Dejean, 1821**: 107. Current status: valid genus in CERAMBYCIDAE: LAMIINAE: POGONOCHERINI.

Type species: *Cerambyxhispidus* Linnaeus, 1758, by subsequent designation (Guérin- Méneville 1826: 186).

Family-group name: POGONOCHÉRAIRES Mulsant, 1839. Stem: *Pogonocher*-.

***Pogonopsis* Bedel, 1898**: 241. Current status: valid genus in CARABIDAE: TRECHINAE: POGONINI.

Type species: *Pogonopsispallida* Bedel, 1898, by monotypy.

Family-group name: POGONOPSINI Bedel, 1900. Stem: *Pogonopse*-.

***Pogonostoma* Klug, 1835**: 382. Current status: valid genus in CICINDELIDAE: CTENOSTOMATINI.

Type species: *Pogonostomachalybaeum* Klug, 1835, by subsequent designation (W. Horn 1926: 12).

Family-group name: POGONOSTOMINI Mandl, 1954. Stem: *Pogonostomat*-.

***Pogonus* Dejean, 1821**: 9. Current status: valid genus in CARABIDAE: TRECHINAE: POGONINI.

Type species: *Carabuslittoralis* Duftschmid, 1812, by monotypy.

Family-group name: POGONIDAE Laporte, 1834. Stem: *Pogon*-.

***Polistichus* Bonelli, 1810**: Tab. Syn. Current status: valid genus in CARABIDAE: HARPALINAE: ZUPHIINI: ZUPHIINA.

Type species: **here fixed** (under ICZN 1999a, Article 70.3.2) as *Buprestisconnexus* Geoffroy, 1785, misidentified as *Carabusfasciolatus* Rossi, 1790 (sensu Fabricius, 1801) in the designation by subsequent monotypy in Latreille (1816: 184).

Family-group name: POLYSTICHINAE H.W. Bates, 1886. Stem: *Polistich*-.

†***Pollostelater* Alexeev, 2011**: 65. Current status: valid genus in ELATERIDAE: †PROTAGRYPNINAE: †POLLOSTELATERINI. Note: Lower Cretaceous (Russia).

Type species: †*Pollostelaterbaissensis* Alexeev, 2011, by original designation.

Family-group name: †POLLOSTELATERINI Alexeev, 2011: 65. Stem: *Pollostelater*-. Note: this name was overlooked in Bouchard et al. (2011), Bouchard and Bousquet (2020a).

***Polpochila* Solier, 1849**: 217. Current status: valid genus in CARABIDAE: HARPALINAE: HARPALINI: STENOLOPHINA.

Type species: *Polpochilaparallela* Solier, 1849 (= *Melanotuschilensis* Chaudoir, 1837), by original designation.

Family-group name: POLPOCHILINAE H.W. Bates, 1891. Stem: *Polpochil*-.

***Polyarthron* Audinet-Serville, 1832**: 189. Current status: valid genus in CERAMBYCIDAE: PRIONINAE: PRIONINI.

Type species: *Prionuspectinicornis* Fabricius, 1792, by monotypy.

Family-group name: POLYARTHRINI Gounelle, 1911. Stem: *Polyarthr*-. Note: junior homonym of POLYARTHRIDAE Daday, 1893 (type genus *Polyarthra* Ehrenberg, 1834) in Rotifera.

***Polybothris* Dupont, 1833**: pl. 77. Current status: valid genus in BUPRESTIDAE: CHRYSOCHROINAE: DICERCINI: DICERCINA. Note: the original spelling was *Polybotris* but the Commission ruled that *Polybothris* was the correct original spelling and placed *Polybothris* Dupont, 1833 on the Official List of Generic Names in Zoology (ICZN 2015a).

Type species: *Polybothriscroesus* Dupont, 1833 (= *Buprestissumptuosa* Klug, 1833), by monotypy (see ICZN 2015a). Note: *Polybothriscroesus* Dupont, 1833 was placed on the Official List of Specific Names in Zoology (ICZN 2015a).

Family-group name: POLYBOTHRISIDAE Gistel, 1848. Stem: *Polybothrid*-.

***Polycaon* Laporte, 1838**: 30. Current status: valid genus in BOSTRICHIDAE: POLYCAONINAE.

Type species: *Polycaonchiliensis* Laporte, 1838, by monotypy.

Family-group name: POLYCAONINAE Lesne, 1896. Stem: *Polycaon*-.

***Polycatus* Heller, 1913**: 379. Current status: valid genus in CURCULIONIDAE: ENTIMINAE: OTIORHYNCHITAE: POLYCATINI.

Type species: *Polycatusaurofasciatus* Heller, 1913, by original designation.

Family-group name: POLYCATINI G.A.K. Marshall, 1956. Stem: *Polycat*-.

***Polycesta* Dejean, 1833a**: 78. Current status: valid genus in BUPRESTIDAE: POLYCESTINAE: POLYCESTINI: POLYCESTINA.

Type species: *Buprestisporcata* Fabricius, 1775, by monotypy.

Family-group name: POLYCESTIDES Lacordaire, 1857. Stem: *Polycest*-.

***Polyctesis* Marseul, 1865**: 264. Current status: valid genus in BUPRESTIDAE: POLYCESTINAE: POLYCTESINI.

Type species: *Polyctesisrhois* Marseul, 1865, by monotypy.

Family-group name: POLYCTESINI Cobos, 1955. Stem: *Polyctes*-.

***Polycystophorus* Gistel, 1856**: 385. Current status: junior synonym of *Malachius* Fabricius, 1775 in MELYRIDAE: MALACHIINAE: MALACHIINI.

Type species: *Cantharisaenea* Linnaeus, 1758, by subsequent designation (Bouchard et al. 2011: 352).

Family-group name: POLYCYSTOPHORIDAE Gistel, 1856. Stem: *Polycystophor*-.

***Polydrusus* Germar, 1817b**: 341. Current status: valid genus in CURCULIONIDAE: ENTIMINAE: POLYDRUSITAE: POLYDRUSINI. Note: name placed on the Official List of Generic Names in Zoology (ICZN 1981c).

Type species: *Curculioundatus* Fabricius, 1781 (= *Curculiotereticollis* DeGeer, 1775), by subsequent designation (Schönherr 1826: 12; see ICZN 1981c). Note: *Curculioundatus* Fabricius, 1781 was placed on the Official List of Specific Names in Zoology (ICZN 1981c).

Family-group name: POLYDROSIDES Schönherr, 1823. Stem: *Polydrus*-.

***Polygraphus* Erichson, 1836**: 57. Current status: valid genus in CURCULIONIDAE: SCOLYTINAE: POLYGRAPHINI.

Type species: *Bostrichuspubescens* Fabricius, 1792 (= *Dermestespoligraphus* Linnaeus, 1758), by monotypy.

Family-group name: POLYGRAPHIDAE Chapuis, 1869. Stem: *Polygraph*-. Note: senior homonym of POLYGRAPHINI Rydon, 1971 (type genus *Polygrapha* Staudinger, 1887) in Lepidoptera.

***Polyhirma* Chaudoir, 1850**: 44. Current status: senior synonym of *Cypholoba* Chaudoir, 1850 in CARABIDAE: HARPALINAE: ANTHIINI. Note: First Reviser (ICZN 1999a, Article 24.2.2) for *Polyhirma* Chaudoir, 1850 versus *Cypholoba* Chaudoir, 1850 is Péringuey (1896: 339) who selected *Polyhirma* as the valid name (the accepted synonymy between the two genus-group names found in the literature is given to G. Strohmeyer (1928: 298) who selected *Cypholoba*); an application to the Commission is necessary to retain *Cypholoba* Chaudoir, 1850 as the valid name.

Type species: *Carabusmacilentus* G.-A. Olivier, 1795, by subsequent designation (Lorenz 1998: 148).

Family-group name: POLYHIRMI Rousseau, 1905. Stem: *Polyhirm*-.

***Polyphylla* Harris, 1841**: 30. Current status: valid genus in SCARABAEIDAE: MELOLONTHINAE: MELOLONTHINI: MELOLONTHINA.

Type species: *Melolonthavariolosa* Hentz, 1830, by subsequent designation (R.M. Young 1988: 21).

Family-group name: POLYPHYLLIDAE Burmeister, 1855. Stem: *Polyphyll*-.

***Polypleurus* Eschscholtz, 1831**: 10, 11. Current status: valid genus in TENEBRIONIDAE: STENOCHIINAE: CNODALONINI.

Type species: *Polypleurusgeminatus* Eschscholtz, 1831, by monotypy.

Family-group name: POLYPLEURI J.L. LeConte, 1862. Stem: *Polypleur*-.

***Polypria* Chevrolat, 1874**: 330. Current status: valid genus in OEDEMERIDAE: POLYPRIINAE.

Type species: *Polypriacruxrufa* Chevrolat, 1874, by monotypy.

Family-group name: POLYPRIINAE Lawrence, 2005. Stem: *Polypri*-.

***Polyrhaphis* Audinet-Serville, 1835a**: 26. Current status: valid genus in CERAMBYCIDAE: LAMIINAE: POLYRHAPHIDINI.

Type species: *Lamiahorrida* Fabricius, 1792 (= *Cerambyxspinosus* Drury, 1773), by subsequent designation (E. Desmarest 1860: 321).

Family-group name: POLYRHAPHITAE J. Thomson, 1860. Stem: *Polyrhaphid*-.

***Polytus* Faust, 1894a**: 353. Current status: valid genus in CURCULIONIDAE: DRYOPHTHORINAE: LITOSOMINI: POLYTINA.

Type species: *Sitophilusmellerborgii* Boheman, 1838, by original designation.

Family-group name: POLYTINI Zimmerman, 1993. Stem: *Polyt*-.

***Pomachilius* Eschscholtz, 1829a**: 31. Current status: valid genus in ELATERIDAE: ELATERINAE: POMACHILIINI.

Type species: *Elatersubfasciatus* Germar, 1823, by monotypy.

Family-group name: POMACHILIITES Candèze, 1859. Stem: *Pomachili*-.

***Pondonatus* John, 1954**: 309. Current status: valid genus in DISCOLOMATIDAE: PONDONATINAE.

Type species: *Pondonatusturneri* John, 1954, by original designation.

Family-group name: PONDONATINAE John, 1954. Stem: *Pondonat*-.

†***Ponomarenkia* Yan, Lawrence, Beattie & Beutel, 2017c**: 613. Current status: invalid senior synonym of †*Ponomarenkium* Yan, Lawrence, Beattie & Beutel, 2018 in †PONOMARENKIUMIDAE. Note: junior homonym of *Ponomarenkia* Perkovsky, 2001 in Coleoptera: AGYRTIDAE. Note: upper Permian (Australia).

Type species: †*Ponomarenkiabelmonthensis* Yan, Lawrence, Beattie & Beutel, 2017, by original designation.

Family-group name: †PONOMARENKIIDAE Yan, Lawrence, Beattie & Beutel, 2017c: 612. Stem: *Ponomarenki*-. Note: family-group name permanently invalid (ICZN 1999a, Article 39).

†***Ponomarenkium* Yan, Lawrence, Beattie & Beutel, 2018**: 110. Current status: valid genus in †PONOMARENKIUMIDAE. Note: upper Permian (Australia).

Type species: †*Ponomarenkiabelmonthensis* Yan, Lawrence, Beattie & Beutel, 2017, by original designation.

Family-group name: †PONOMARENKIUMIDAE Lawrence & Bouchard in Cai et al., 2022: 12, 13 (Supp. Inf.). Stem: *Ponomarenkium*-.

***Poophagus* Schönherr, 1837**: 590. Current status: valid genus in CURCULIONIDAE: CEUTORHYNCHINAE: CEUTORHYNCHINI.

Type species: *Curculiosisymbrii* Fabricius, 1777, by original designation.

Family-group name: POOPHAGIDAE Schultze, 1902. Stem: *Poophag*-.

***Popilius* Kaup, 1871**: 75. Current status: valid genus in PASSALIDAE: PASSALINAE: PROCULINI.

Type species: *Passalusmarginatus* Percheron, 1834, by subsequent designation (Gravely 1918: 26).

Family-group name: POPILIINAE Kuwert, 1896. Stem: *Popili*-.

***Popillia* Dejean, 1821**: 60. Current status: valid genus in SCARABAEIDAE: RUTELINAE: ANOMALINI: POPILLIINA.

Type species: *Trichiusbipunctatus* Fabricius, 1787, by monotypy.

Family-group name: POPILLIIDAE Ohaus, 1902. Stem: *Popilli*-.

***Poria* Mulsant, 1850**: 885. Current status: valid genus in COCCINELLIDAE: COCCINELLINAE: COCCIDULINI.

Type species: *Poriacyanea* Mulsant, 1850, by subsequent designation (Crotch 1874a: 288).

Family-group name: PORIENS Mulsant, 1850. Stem: *Pori*-.

***Porphyraspis* Hope, 1841a**: 154. Current status: junior synonym of *Hemisphaerota* Chevrolat, 1836 in CHRYSOMELIDAE: CASSIDINAE: HEMISPHAEROTINI.

Type species: *Cassidaerythrocera* Germar, 1823, by original designation. Note: this species is generally listed as a junior synonym of *Imatidiumcyaneum* Say, 1824, but Germar’s name is older and should be used as the valid name for the species; an application to the Commission is necessary to conserve usage of the commonly used name *Hemisphaerotacyanea* (Say, 1824).

Family-group name: PORPHYRASPITAE Spaeth, 1929. Stem: *Porphyraspid*-.

***Porphyronota* Burmeister, 1842**: 622. Current status: valid genus in SCARABAEIDAE: CETONIINAE: DIPLOGNATHINI.

Type species: *Cetoniacarnifex* Fabricius, 1781, by subsequent designation (Holm 1990: 113).

Family-group name: PORPHYRONOTII Péringuey, 1907. Stem: *Porphyronot*-.

***Potamophilus* Germar, 1811**: 41. Current status: valid genus in ELMIDAE: LARAINAE: POTAMOPHILINI.

Type species: *Parnusacuminatus* Fabricius, 1792, by monotypy.

Family-group name: POTAMOPHILIDAE Fournel & Géhin, 1845. Stem: *Potamophil*-. Note: this family-group name was attributed to “Mulsant & Rey, 1872” in Bouchard et al. (2011: 292); however, it was first made available by Fournel and Géhin (1845: 119); the tribe POTAMOPHILINI is currently included in the subfamily LARAINAE J.L. LeConte, 1861 and since POTAMOPHILINI is older than LARAINAE, it should be the valid name for the subfamily; however, usage of LARAINAE J.L. LeConte, 1861 is conserved following Article 35.5 (ICZN 1999a).

***Potemnemus* J. Thomson, 1864**: 81. Current status: valid genus in CERAMBYCIDAE: LAMIINAE: LAMIINI.

Type species: *Cerambyxscabrosus* G.-A. Olivier, 1790, by original designation.

Family-group name: POTEMNEMINI Aurivillius, 1922. Stem: *Potemnem*-.

***Potergus* Bonvouloir, 1871**: 64, 110. Current status: valid genus in THROSCIDAE.

Type species: *Potergusfiliformis* Bonvouloir, 1871, by monotypy.

Family-group name: POTERGINI Cobos, 1961. Stem: *Poterg*-.

†***Praelaterium* Dolin, 1973**: 75. Current status: valid genus in CEROPHYTIDAE. Note: Jurassic (Kyrgyzstan).

Type species: †*Praelateriumproblematicum* Dolin, 1973, by original designation.

Family-group name: †PRAELATERIIDAE Dolin, 1973. Stem: *Praelateri*-.

†***Praemordella* Ščegoleva-Barovskaya, 1929**: 27. Current status: valid genus in MORDELLIDAE: †PRAEMORDELLINAE. Note: Upper Jurassic (Kazakhstan).

Type species: †*Praemordellamartynovi* Ščegoleva-Barovskaya, 1929, by monotypy.

Family-group name: †PRAEMORDELLINAE Ščegoleva-Barovskaya, 1929. Stem: *Praemordell*-.

***Praeugena* Laporte, 1840b**: 241. Current status: valid genus in TENEBRIONIDAE: TENEBRIONINAE: PRAEUGENINI. Note: nomen protectum (see Bouchard and Bousquet 2020b: 6).

Type species: *Helopsmarginatus* Fabricius, 1792, by subsequent designation (Hope 1841a: 133).

Family-group name: PRAEUGENINA De Moor, 1970. Stem: *Praeugen*-.

***Praocis* Eschscholtz, 1829d**: 6. Current status: valid genus in TENEBRIONIDAE: PIMELIINAE: PRAOCIINI.

Type species: *Praocisrufipes* Eschscholtz, 1829, by subsequent designation (Guérin-Méneville 1834b: 8–9).

Family-group name: PRAOCIDAE Eschscholtz, 1829. Stem: *Praoci*-.

***Prasocuris* Latreille, 1802b**: 224. Current status: valid genus in CHRYSOMELIDAE: CHRYSOMELINAE: CHRYSOMELINI.

Type species: *Chrysomelaphellandrii* Linnaeus, 1758, by monotypy.

Family-group name: PRASOCURISIDAE Gistel, 1848. Stem: *Prasocur*-.

***Prasoidea* Weise, 1907**: 133. Current status: junior synonym of *Odontionopa* Chevrolat, 1836 in CHRYSOMELIDAE: EUMOLPINAE: EURYOPINI.

Type species: *Colaspissericea* Gyllenhal, 1808, by monotypy.

Family-group name: PRASOIDEINI Clavareau, 1914. Stem: *Prasoide*-.

***Prateus* J.L. LeConte, 1862**: 238. Current status: valid genus in TENEBRIONIDAE: LAGRIINAE: PRATEINI.

Type species: *Prateusfusculus* J.L. LeConte, 1862, by original designation.

Family-group name: PRATEINI Aalbu, Kanda, Merkl, Ivie & Johnston, 2023: 161. Stem: *Prate*-.

***Premnobius* Eichhoff, 1878**: 404. Current status: valid genus in CURCULIONIDAE: SCOLYTINAE: IPINI: PREMNOBIINA.

Type species: *Premnobiuscavipennis* Eichhoff, 1878, by monotypy.

Family-group name: PREMNOBIINI Browne, 1962. Stem: *Premnobi*-.

***Premnotrypes* Pierce, 1914**: 348. Current status: valid genus in CURCULIONIDAE: ENTIMINAE: ENTIMITAE: PREMNOTRYPINI.

Type species: *Premnotrypessolani* Pierce, 1914, by original designation.

Family-group name: PREMNOTRYPINI Kuschel, 1956. Stem: *Premnotryp*-.

***Prepusa* Chaudoir, 1850**: 15. Current status: junior synonym of *Eulampra* Chaudoir, 1848 in CICINDELIDAE: CICINDELINI: DROMICINA. Note: unnecessary new replacement name for *Eulampra* Chaudoir, 1848.

Type species: *Cicindelamiranda* Chaudoir, 1843, by monotypy (type fixation for *Eulampra* Chaudoir).

Family-group name: PREPUSINI W. Horn, 1899. Stem: *Prepus*-.

***Pretilia* H.W. Bates, 1866**: 302. Current status: valid genus in CERAMBYCIDAE: LAMIINAE: PRETILIINI.

Type species: *Pretiliatelephoroides* H.W. Bates, 1866 (= *Saperdatuberculata* Fabricius, 1801), by monotypy.

Family-group name: PRETILIINI Martins & Galileo, 1990. Stem: *Pretili*-.

***Priacma* J.L. LeConte, 1874c**: 87. Current status: valid genus in CUPEDIDAE: PRIACMINAE.

Type species: *Cupesserrata* J.L. LeConte, 1861, by monotypy.

Family-group name: PRIACMINI Crowson, 1962. Stem: *Priacm*-.

***Priasilpha* Broun, 1893b**: 1077. Current status: valid genus in PRIASILPHIDAE.

Type species: *Priasilphaobscura* Broun, 1893, by monotypy.

Family-group name: PRIASILPHINAE Crowson, 1973. Stem: *Priasilph*-.

***Prinobius* Mulsant, 1842b**: 207. Current status: valid genus in CERAMBYCIDAE: PRIONINAE: MACROTOMINI: MACROTOMINA.

Type species: *Prinobiusmyardi* Mulsant, 1842, by monotypy.

Family-group name: PRINOBIINI Vives, 2000. Stem: *Prinobi*-.

***Priocera* W. Kirby, 1819**: 389. Current status: valid genus in CLERIDAE: CLERINAE: PRIOCERINI.

Type species: *Prioceravariegata* W. Kirby, 1819, by monotypy.

Family-group name: PRIOCERIDAE Laporte, 1838. Stem: *Priocer*-.

***Priolomus* Erichson, 1845a**: 256. Current status: valid genus in ZOPHERIDAE: COLYDIINAE: SYNCHITINI.

Type species: *Priolomusspinicollis* Fairmaire, 1869, by subsequent monotypy (Fairmaire 1869: 204).

Family-group name: PRIOLOMINI Dajoz, 1980. Stem: *Priolom*-.

***Prionobrachium* Faust, 1894b**: 341. Current status: valid genus in CURCULIONIDAE: CURCULIONINAE: PRIONOBRACHIINI.

Type species: *Prionobrachiumschoenherri* Faust, 1894, by monotypy.

Family-group name: PRIONOBRACHIINA Hustache, 1938. Stem: *Prionobrachi*-.

***Prionocerus* Perty, 1831**: xxxiii. Current status: valid genus in PRIONOCERIDAE.

Type species: *Prionoceruscoeruleipennis* Perty, 1831, by monotypy.

Family-group name: PRIONOCÉRIDES Lacordaire, 1857. Stem: *Prionocer*-. Note: senior homonym of PRIONOCERINI Savchenko, 1966 (type genus *Prionocera* Loew, 1844) in Diptera.

***Prionomerus* Schönherr, 1835**: 359. Current status: junior synonym of *Odontopus* Say, 1832 in CURCULIONIDAE: CURCULIONINAE: CAMAROTINI: PRIONOMERINA.

Type species: *Prionomeruscarbonarius* Gyllenhal, 1835 (= *Anthonomuscalceatus* Say, 1832), by original designation.

Family-group name: PRIONOMÉRIDES Lacordaire, 1863. Stem: *Prionomer*-.

***Prionomma* A. White, 1853**: 19. Current status: valid genus in CERAMBYCIDAE: PRIONINAE: PRIONINI.

Type species: *Prionusorientalis* G.-A. Olivier, 1795 (= *Prionusatratus* Gmelin, 1790 [as *attratus*]), by monotypy.

Family-group name: PRIONOMMITAE J. Thomson, 1861. Stem: *Prionommat*-.

***Prionus* Geoffroy, 1762**: 198. Current status: valid genus in CERAMBYCIDAE: PRIONINAE: PRIONINI. Note: name placed on the Official List of Generic Names in Zoology (ICZN 1994a).

Type species: *Cerambyxcoriarius* Linnaeus, 1758, by plenary power (see ICZN 1994a). Note: name placed on the Official List of Specific Names in Zoology (ICZN 1994a).

Family-group name: PRIONII Latreille, 1802. Stem: *Prion*-.

***Prioptera* Hope, 1841a**: 153, 176. Current status: junior synonym of *Basiprionota* Chevrolat, 1836 in CHRYSOMELIDAE: CASSIDINAE: BASIPRIONOTINI.

Type species: *Cassidaoctopunctata* Fabricius, 1787, by original designation.

Family-group name: PRIOPTERITAE Spaeth, 1914. Stem: *Priopter*-.

***Prioscelis* Hope, 1841a**: 128. Current status: valid genus in TENEBRIONIDAE: LAGRIINAE: PYCNOCERINI.

Type species: *Prioscelisfabricii* Hope, 1841, by original designation.

Family-group name: PRIOSCELINA Skopin, 1964. Stem: *Prioscelid*-.

***Prisahypnus* Stibick, 1979**: 166. Current status: valid genus in ELATERIDAE: HYPNOIDINAE.

Type species: *Cryptohypnusfrontalis* Sharp, 1877, by original designation.

Family-group name: PRISAHYPINI Stibick, 1979. Stem: *Prisahypn*-.

***Pristiptera* Dejean, 1833a**: 77. Current status: senior synonym of *Pelecopselaphus* Solier, 1833 in BUPRESTIDAE: CHRYSOCHROINAE: PARALEPTODEMINI: PRISTIPTERINA. Note: this name is a senior subjective synonym of *Pelecopselaphus* Solier, 1833 and should be used as valid (see Bousquet and Bouchard 2013a: 22); however, the junior name is currently used as valid and an application to the Commission is necessary to conserve the usage of *Pelecopselaphus* Solier, 1833.

Type species: *Buprestisblanda* Fabricius, 1781, by monotypy.

Family-group name: PRISTIPTERINA Holyński, 1993. Stem: *Pristipter*-.

***Pristolycus* Gorham, 1883b**: 407. Current status: valid genus in LAMPYRIDAE: LAMPYRINAE: LUCIDOTINI: LUCIDOTINA.

Type species: *Pristolycussagulatus* Gorham, 1883, by monotypy.

Family-group name: PRISTOLYCINI J.R. Winkler, 1953. Stem: *Pristolyc*-.

***Pristonychus* Dejean, 1828**: 3, 43. Current status: valid subgenus of *Laemostenus* Bonelli, 1810 in CARABIDAE: HARPALINAE: SPHODRINI: SPHODRINA.

Type species: *Carabusterricola* Herbst, 1784, by subsequent designation (Westwood 1838c: 2).

Family-group name: PRISTONYCHIDAE Gistel, 1848. Stem: *Pristonych*-.

†***Pristorhynchus* Heer, 1847**: 190. Current status: valid genus in CURCULIONIDAE: ENTIMINAE incertae sedis. Note: Upper Miocene (Germany).

Type species: †*Pristorhynchusellipticus* Heer, 1847, by monotypy.

Family-group name: †PRISTORHYNCHIDEN Heer, 1847. Stem: *Pristorhynch*-.

***Pristosia* Motschulsky, 1865**: 311. Current status: valid genus in CARABIDAE: HARPALINAE: SPHODRINI: PRISTOSIINA.

Type species: *Pristosiapicea* Motschulsky, 1865, by monotypy.

Family-group name: PRISTOSIAE Lindroth, 1956. Stem: *Pristosi*-.

†***Probelus* Arnoldi, 1977**: 151. Current status: valid genus in NEMONYCHIDAE: †EOBELINAE: †PROBELINI. Note: Upper Jurassic (Kazakhstan).

Type species: †*Probeluscurvispinus* Arnoldi, 1977, by original designation.

Family-group name: †PROBELINI Legalov, 2009. Stem: *Probel*-.

***Problechilus* Eichhoff, 1878**: 34, 167. Current status: junior synonym of *Gymnochilus* Eichhoff, 1868 in CURCULIONIDAE: SCOLYTINAE: HEXACOLINI. Note: unnecessary new replacement name for *Gymnochilus* Eichhoff, 1868.

Type species: *Gymnochiluszonatus* Eichhoff, 1868, by monotypy (type fixation for *Gymnochilus* Eichhoff).

Family-group name: PROBLECHILIDAE Eichhoff, 1878. Stem: *Problechil*-.

***Procerus* Dejean, 1821**: 5. Current status: valid subgenus of *Carabus* Linnaeaus, 1758 in CARABIDAE: CARABINAE: CARABINI: CARABINA.

Type species: **here fixed** (under ICZN 1999a, Article 70.3.2) as *Carabusgigas* Creutzer, 1799, misidentified as *Carabusscabrosus* G.-A. Olivier, 1790 (sensu Fabricius, 1801) in the subsequent designation by Hope (1838a: 47).

Family-group name: PROCERIDAE Laporte, 1834. Stem: *Procer*-.

***Procirrus* Latreille, 1829a**: 436. Current status: valid genus in STAPHYLINIDAE: PAEDERINAE: PINOPHILINI: PROCIRRINA.

Type species: *Procirruslefebvrei* Latreille, 1829 [as *lefeburi*], by monotypy.

Family-group name: PROCIRRI Bernhauer & Schubert, 1912. Stem: *Procirr*-.

***Procletus* Péringuey, 1896**: 568, 570. Current status: valid genus in CARABIDAE: HARPALINAE: CHLAENIINI: CHLAENIINA.

Type species: *Procletussingularis* Péringuey,1896, by monotypy.

Family-group name: PROCLETINI Basilewsky, 1950. Stem: *Proclet*-.

***Procrustes* Bonelli, 1810**: 39, Tab. Syn. Current status: valid genus in CARABIDAE: CARABINAE: CARABINI: CARABINA.

Type species: *Carabuscoriaceus* Linnaeus, 1758, by monotypy.

Family-group name: PROCRUSTOGENICI Roeschke, 1898. Stem: *Procrust*-.

***Proctocera* Chevrolat, 1855**: 285. Current status: valid genus in CERAMBYCIDAE: LAMIINAE: PROCTOCERINI.

Type species: *Proctocerascalaris* Chevrolat, 1855, by monotypy.

Family-group name: PROCTOCERINI Aurivillius, 1922. Stem: *Proctocer*-.

***Proctophanes* Harold, 1861**: 110, 111. Current status: valid genus in SCARABAEIDAE: APHODIINAE: APHODIINI: PROCTOPHANINA.

Type species: *Aphodiussculptus* Hope, 1848, by monotypy.

Family-group name: PROCTOPHANINI Stebnicka & Howden, 1995. Stem: *Proctophan*-.

***Proculejus* Kaup, 1868b**: 13. Current status: valid genus in PASSALIDAE: PASSALINAE: PROCULINI.

Type species: *Proculejustruquii* Kaup, 1868, by subsequent designation (Gravely 1918: 31).

Family-group name: PROCULEJINAE Kuwert, 1896. Stem: *Proculej*-.

***Proculus* Kaup, 1868b**: 8. Current status: valid genus in PASSALIDAE: PASSALINAE: PROCULINI.

Type species: *Passalusgoryi* Melly, 1833, by subsequent designation (Gravely 1918: 42).

Family-group name: PROCULINAE Kaup, 1868. Stem: *Procul*-.

†***Procurculio* Arnoldi, 1977**: 157. Current status: valid genus in NEMONYCHIDAE: †ECCOPTARTHRINAE. Note: Upper Jurassic (Kazakhstan).

Type species: †*Procurculiofortipes* Arnoldi, 1977, by original designation.

Family-group name: †PROCURCULIONINI Arnoldi, 1977. Stem: *Procurculion*-.

***Prodoretus* Brenske, 1893**: 1. Current status: valid genus in SCARABAEIDAE: RUTELINAE: ADORETINI: PRODORETINA.

Type species: *Prodoretusvittatus* Brenske, 1893, by subsequent monotypy (Brenske 1893: 10). Note: *Prodoretus* Brenske, 1893 and *Prodoretusvittatus* Brenske, 1893 were published in two separate parts (published on different dates) of the same article, hence the use of subsequent monotypy for the type species designation.

Family-group name: PRODORETINA Ohaus, 1912. Stem: *Prodoret*-.

***Proeces* Schönherr, 1838**: 1080. Current status: valid genus in CURCULIONIDAE: COSSONINAE: COSSONINI. Note: two original spellings were used, including *Oeces* (p. 1140); Neave (1940: 910) acted as First Reviser (ICZN 1999a, Article 24.2.3) and selected *Proeces*, the spelling currently in prevailing usage.

Type species: *Proecesmacer* Boheman, 1838, by original designation.

Family-group name: PROECINA Voss, 1956. Stem: *Proec*-.

***Prognathus* Blondel, 1827**: 413. Current status: junior synonym of *Siagonium* W. Kirby & Spence, 1815 in STAPHYLINIDAE: PIESTINAE.

Type species: *Prognathusrufipennis* Blondel, 1827 (= *Siagoniumquadricorne* W. Kirby & Spence, 1815), by monotypy.

Family-group name: PROGNATHITES Blanchard, 1845. Stem: *Prognath*-.

***Proholopterus* Monné, 2012**: 44. Current status: junior synonym of *Holopterus* Blanchard, 1851, **stat. nov.** in CERAMBYCIDAE: CERAMBYCINAE: HOLOPTERINI. Note: unnecessary new replacement name for *Holopterus* Blanchard, 1851; *Holopterus* Brehm, 1845 [Aves] is an incorrect subsequent spelling of *Hoplopterus* Bonaparte, 1831 not in prevailing usage and therefore *Holopterus* Blanchard, 1851, **stat. nov.** can be used as the valid name.

Type species: *Holopteruschilensis* Blanchard, 1851, by monotypy (type fixation for *Holopterus* Blanchard).

Family-group name: PROHOLOPTERINI Monné, 2012: 44. Stem: *Proholopter*-.

***Prolytta* Kaszab, 1959**: 110. Current status: valid genus in MELOIDAE: MELOINAE: LYTTINI.

Type species: *Lyttalucida* Haag-Rutenberg, 1880, by original designation.

Family-group name: PROLYTTINI Selander, 1960. Stem: *Prolytt*-.

***Promecheilus* Solier, 1851**: 251. Current status: valid genus in PROMECHEILIDAE.

Type species: *Promecheilusvariegatus* Solier, 1851, by monotypy.

Family-group name: PROMÉCHILIDES Lacordaire, 1859. Stem: *Promecheil*-.

***Promecognathus* Chaudoir, 1847**: 515, 524. Current status: valid genus in CARABIDAE: SCARITINAE: PROMECOGNATHINI.

Type species: *Eripuslaevissimus* Dejean, 1829, by monotypy.

Family-group name: PROMECOGNATHI J.L. LeConte, 1853. Stem: *Promecognath*-.

***Promecops* C.R. Sahlberg, 1823a**: 30. Current status: valid genus in CURCULIONIDAE: ENTIMINAE: ENTIMITAE: EUDIAGOGINI. Note: we consider *Promecops* used by Schönherr (1823: 1141) a subsequent use of *Promecops* C.R. Sahlberg, 1823, not a distinct taxon.

Type species: *Promecopsnubifer* C.R. Sahlberg, 1823, by monotypy. Note: combined description by indication of a new genus and a single new species (ICZN 1999a, Article 12.2.6).

Family-group name: PROMÉCOPIDES Lacordaire, 1863. Stem: *Promecop*-. Note: this family- group name has priority over EUDIAGOGINI J.L. LeConte, 1874, which is currently use as valid; a request to the Commission is necessary to conserve usage of J.L. LeConte’s name.

***Promecotheca* Guérin-Méneville, 1840b**: 334. Current status: valid genus in CHRYSOMELIDAE: CASSIDINAE: PROMECOTHECINI. Note: as pointed out by Bouchard and Bousquet (2020a: 109), this genus is credited to Blanchard (1853: 312) by several authors including by Gressitt (1957: 279) and Staines (2015: 1), but it was first made available by Guérin-Méneville in 1840 by indication, with the inclusion of at least one available species included under a new genus-group name (ICZN 1999a, Article 12.2.5).

Type species: *Promecothecapetelii* Guérin-Méneville, 1840, by **present designation**.

Family-group name: PROMECOTHÉCITES Chapuis, 1875. Stem: *Promecothec*-.

***Prometopia* Erichson, 1843a**: 279. Current status: valid genus in NITIDULIDAE: PROMETOPIINAE.

Type species: *Nitidulasexmaculata* Say, 1825, by subsequent designation (R. Lucas 1920: 539).

Family-group name: PROMETOPINAE Böving & Craighead, 1931. Stem: *Prometopi*-.

***Pronomaea* Erichson, 1837**: 378. Current status: valid genus in STAPHYLINIDAE: ALEOCHARINAE: PRONOMAEINI.

Type species: *Pronomaearostrata* Erichson, 1837, by monotypy.

Family-group name: PRONOMÉATES Mulsant & Rey, 1873. Stem: *Pronomae*-.

***Pronoterus* Sharp, 1882b**: 263. Current status: valid genus in NOTERIDAE: NOTERINAE: NOTERINI.

Type species: *Pronoteruspunctipennis* Sharp, 1882, by monotypy.

Family-group name: PRONOTERINI Nilsson, 2005. Stem: *Pronoter*-.

***Propalticus* Sharp, 1879**: 88. Current status: valid genus in LAEMOPHLOEIDAE.

Type species: *Propalticusoculatus* Sharp, 1879, by monotypy.

Family-group name: PROPALTICIDAE Crowson, 1952. Stem: *Propaltic*-.

***Proscarabaeus* Schrank, 1781**: 225. Current status: junior synonym of *Meloe* Linnaeus, 1758 in MELOIDAE: MELOINAE: MELOINI.

Type species: *Meloeproscarabaeus* Linnaeus, 1758, by absolute tautonymy.

Family-group name: PROSCARABAEIDAE Gistel, 1848. Stem: *Proscarabae*-.

***Proscoporhinus* Montrouzier, 1861a**: 868. Current status: valid genus in ANTHRIBIDAE: ANTHRIBINAE: PROSCOPORHININI.

Type species: *Proscoporhinusamyoti* Montrouzier, 1861, by monotypy.

Family-group name: PROSCOPORHINIDES Lacordaire, 1865. Stem: *Proscoporhin*-.

***Prosodes* Eschscholtz, 1829d**: 9. Current status: valid genus in TENEBRIONIDAE: BLAPTINAE: BLAPTINI: PROSODINA.

Type species: *Blapsattenuata* Fischer, 1820 (= *Blaps obtusa* Fabricius, 1798), by subsequent designation (Gebien 1937: 846).

Family-group name: PROSODINA Skopin, 1960. Stem: *Prosod*-.

***Prosopocera* Blanchard, 1845a**: 160, 176. Current status: valid genus in CERAMBYCIDAE: LAMIINAE: PROSOPOCERINI.

Type species: *Lamiafronticornis* Fabricius, 1781 (= *Cerambyxbipunctatus* Drury, 1773), by monotypy.

Family-group name: PROSOPOCERITAE J. Thomson, 1864. Stem: *Prosopocer*-.

***Prosopocoilus* Hope, 1845b**: 30. Current status: valid genus in LUCANIDAE: LUCANINAE: DORCINI.

Type species: *Lucanuscavifrons* Hope, 1845, by subsequent designation (Arrow 1950: 77).

Family-group name: PROSOPOCOILINI Benesh, 1960. Stem: *Prosopocoil*-.

***Prosopodonta* Baly, 1858**: 68. Current status: valid genus in CHRYSOMELIDAE: CASSIDINAE: PROSOPODONTINI.

Type species: *Prosopodontalimbata* Baly, 1858, by original designation.

Family-group name: PROSOPODONTINI Weise, 1910. Stem: *Prosopodont*-.

***Prospheres* Saunders, 1868**: 7. Current status: valid genus in BUPRESTIDAE: POLYCESTINAE: PROSPHERINI.

Type species: *Buprestisaurantiopicta* Laporte & Gory, 1838, by monotypy.

Family-group name: PROSPHERESINI Cobos, 1980. Stem: *Prospher*-.

***Prosphodrus* Britton, 1959**: 106. Current status: valid genus in CARABIDAE: HARPALINAE: PLATYNINI.

Type species: *Prosphodruswaltoni* Britton, 1959, by monotypy.

Family-group name: PROSPHODRINI J.M. Valentine, 1987. Stem: *Prosphodr*-.

***Prosternon* Latreille, 1834**: 151. Current status: valid genus in ELATERIDAE: DENDROMETRINAE: PROSTERNINI.

Type species: *Elaterholosericeus* G.-A. Olivier, 1790 (= *Elatertessellatus* Linnaeus, 1758), by subsequent designation (Westwood 1838c: 26).

Family-group name: PROSTERNIDAE Gistel, 1856. Stem: *Prostern*-. Note: nomen protectum (see Bouchard et al. 2011: 315).

***Prosthetops* C.O. Waterhouse, 1879b**: 533. Current status: valid genus in HYDRAENIDAE: PROSTHETOPINAE: PROSTHETOPINI.

Type species: *Prosthetopscapensis* C.O. Waterhouse, 1879 (= *Ochthebiusmegacephalus* Boheman, 1851), by monotypy.

Family-group name: PROSTHETOPINAE Perkins, 1994. Stem: *Prosthetop*-.

***Prostominia* Reitter, 1889f**: 315. Current status: valid genus in SALPINGIDAE: PROSTOMINIINAE.

Type species: *Prostominialewisi* Reitter, 1889, by monotypy.

Family-group name: PROSTOMININI Grouvelle, 1914. Stem: *Prostomini*-.

***Prostomis* Latreille, 1819a**: 171. Current status: valid genus in PROSTOMIDAE.

Type species: *Trogositamandibularis* Fabricius, 1801, by subsequent monotypy (Lepeletier and Audinet-Serville 1825: 215). Note: Latreille (1819a: 171) listed one species under his genus, “trogosite mandibulaire de Fabricius”; this name being French vernacular is not an available scientific name and cannot be used as the type species.

Family-group name: PROSTOMIDAE C.G. Thomson, 1859. Stem: *Prostom*-.

†***Protactus* Heer, 1847**: 28. Current status: valid genus in STAPHYLINIDAE: †PROTACTINAE. Note: Upper Miocene (Germany).

Type species: †*Protactuserichsonii* Heer, 1847, by monotypy.

Family-group name: †PROTACTIDEN Heer, 1847. Stem: *Protact*-.

†***Protagrypnus* Dolin, 1973**: 75. Current status: valid genus in ELATERIDAE: †PROTAGRYPNINAE: †PROTAGRYPNINI. Note: Jurassic (China; Kyrgkzstan).

Type species: †*Protagrypnusexoletus* Dolin, 1973, by original designation.

Family-group name: †PROTAGRYPNINI Dolin, 1973. Stem: *Protagrypn*-.

***Protaxis* Gahan, 1906**: xiv, 93. Current status: valid genus in CERAMBYCIDAE: CERAMBYCINAE: PROTAXINI.

Type species: *Protaxisfulvescens* Gahan, 1906, by original designation.

Family-group name: PROTAXINI Gahan, 1906. Stem: *Protax*-.

***Proteinus* Latreille, 1797**: 9. Current status: valid genus in STAPHYLINIDAE: PROTEININAE: PROTEININI. Note: name placed on the Official List of Generic Names in Zoology (ICZN 1969a).

Type species: *Dermestesbrachypterus* Fabricius, 1792, by plenary power (see ICZN 1969a). Note: name placed on the Official List of Specific Names in Zoology (ICZN 1969a).

Family-group name: PROTEININI Erichson, 1839. Stem: *Protein*-.

***Protelater* Sharp, 1877c**: 482. Current status: valid genus in ELATERIDAE: LISSOMINAE: PROTELATERINI.

Type species: *Protelaterelongatus* Sharp, 1877, by subsequent designation (Hyslop 1921: 667).

Family-group name: PROTELATERIDAE O. Schwarz, 1902. Stem: *Protelater*-.

***Protelmis* Grouvelle, 1911**: 265. Current status: valid genus in PROTELMIDAE.

Type species: *Protelmislimnioides* Grouvelle, 1911, by monotypy.

Family-group name: PROTELMINI Jeannel, 1950. Stem: *Protelm*-.

***Proterhinus* Sharp, 1878a**: 16, 20. Current status: valid genus in BELIDAE: OXYCORYNINAE: AGLYCYDERITAE: AGLYCYDERINI.

Type species: *Proterhinusvestitus* Sharp, 1878, by subsequent designation (R. Lucas 1920: 541).

Family-group name: PROTERHINIDES Fauvel, 1891. Stem: *Proterhin*-.

***Proterotaphes* Kazantsev, 2006**: 218. Current status: junior synonym of *Plateros* Kazantsev, 2004 in LYCIDAE: EROTINAE: PROTEROTAPHINI. Note: unnecessary new replacement name for *Proteros* Kazantsev, 2004; the name *Proteros* Holmberg, 1917 [Hymenoptera] was suppressed for both the Principles of Priority and Homonymy and placed on the Index of Rejected and Invalid Generic Names in Zoology (ICZN 2004b).

Type species: *Proterossempiternus* Kazantsev, 2004, by original designation (type fixation for *Proteros* Kazantsev).

Family-group name: PROTEROTAPHINI Kazantsev, 2012b: 399. Stem: *Proterotaph*-.

***Proterus* Raffray, 1897a**: 231. Current status: valid genus in STAPHYLINIDAE: PSELAPHINAE: GONIACERITAE: PROTERINI.

Type species: *Proteruspunctatus* Raffray, 1897, by monotypy.

Family-group name: PROTERINI Jeannel, 1949. Stem: *Proter*-.

***Proteugnamptus* Voss, 1939**: 446. Current status: valid genus in ATTELABIDAE: RHYNCHITINAE: RHINOCARTITAE: RHINOCARTINI.

Type species: *Proteugnamptusmadagassus* Voss, 1939, by monotypy.

Family-group name: PROTEUGNAMPTINI Legalov, 2003. Stem: *Proteugnampt*-.

***Prothema* Pascoe, 1856**: 43. Current status: valid genus in CERAMBYCIDAE: CERAMBYCINAE: PROTHEMINI.

Type species: *Prothemasignata* Pascoe, 1856, by subsequent designation (J. Thomson 1864: 182).

Family-group name: PROTHÉMIDES Lacordaire, 1868. Stem: *Prothem*-.

***Prothydrus* Guignot, 1954**: 45. Current status: junior synonym of *Enhydrus* Laporte, 1835 in GYRINIDAE: GYRININAE: DINEUTINI: ENHYDRUSINA. Note: new replacement name for *Enhydrus* Laporte, 1835; name suppressed for both the Principles of Priority and Homonymy and placed on the Official Index of Rejected and Invalid Generic Names in Zoology (ICZN 1964).

Type species: *Gyrinussulcatus* Wiedemann, 1821, by monotypy (type fixation for *Enhydrus* Laporte).

Family-group name: PROTHYDRINAE Guignot, 1954. Stem: *Prothydr*-. Note: family-group name permanently invalid (ICZN 1999a, Article 39); name placed on the Official Index of Rejected and Invalid Family-group Names in Zoology (ICZN 1964).

***Prothyma* Hope, 1838a**: 12, 27. Current status: valid genus in CICINDELIDAE: CICINDELINI: DROMICINA.

Type species: *Cicindelaquadripunctata* Fabricius, 1801, by monotypy.

Family-group name: PROTHYMINI W. Horn, 1906. Stem: *Prothym*-.

***Protochthebius* Perkins, 1997**: 154. Current status: valid genus in HYDRAENIDAE: OCHTHEBIINAE: OCHTHEBIINI.

Type species: *Protochthebiussatoi* Perkins, 1997, by original designation.

Family-group name: PROTOCHTHEBIINA Perkins, 1997. Stem: *Protochthebi*-.

†***Protoclaviger* J. Parker & Grimaldi, 2014**: 2428. Current status: valid genus in STAPHYLINIDAE: PSELAPHINAE: CLAVIGERITAE: †PROTOCLAVIGERINI. Note: lower Eocene (India).

Type species: †*Protoclavigertrichodens* J. Parker & Grimaldi, 2014, by monotypy.

Family-group name: †PROTOCLAVIGERINI J. Parker & Grimaldi, 2014: 2428. Stem: *Protoclaviger*-.

***Protocucujus* Crowson, 1954**: 60. Current status: junior synonym of *Ericmodes* Reitter, 1878 in PROTOCUCUJIDAE.

Type species: *Protocucujuschilensis* Crowson, 1954 (= *Ericmodesfuscitarsis* Reitter, 1878), by original designation.

Family-group name: PROTOCUCUJIDAE Crowson, 1954. Stem: *Protocucuj*-.

†***Protolucanus* Nikolajev, 2007b**: 18, 213. Current status: valid genus in LUCANIDAE: †PROTOLUCANINAE. Note: Lower Cretaceous (Mongolia).

Type species: †*Protolucanusjurassicus* Nikolajev, 2007, by original designation.

Family-group name: †PROTOLUCANINAE Nikolajev, 2007. Stem: *Protolucan*-.

***Protomantis* Schönherr, 1840a**: 721. Current status: valid genus in CURCULIONIDAE: BRACHYCERINAE: BRACHYCERINI.

Type species: *Protomantisdregei* Gyllenhal, 1840, by original designation.

Family-group name: PROTOMANTIINAE Aurivillius, 1886. Stem: *Protomanti*-.

***Protomeloe* M. Abdullah, 1965**: 248. Current status: valid genus in MELOIDAE: ELETICINAE: SPASTICINI: PROTOMELOINA.

Type species: *Protomeloeargentinensis* M. Abdullah, 1965 (= *Spasticawagneri* Pic, 1915), by original designation.

Family-group name: PROTOMELOINI M. Abdullah, 1965. Stem: *Protomelo*-.

***Protonarthron* J. Thomson, 1858b**: 180. Current status: valid genus in CERAMBYCIDAE: LAMIINAE: DORCASCHEMATINI.

Type species: *Protonarthrondiabolicum* J. Thomson, 1858, by monotypy.

Family-group name: PROTONARTHRONITAE J. Thomson, 1864. Stem: *Protonarthr*-.

†***Protopassalus* Aquino dos Santos, Mattos, Mermudes, Scheffler & Reyes-Castillo, 2021**: 4. Current status: valid genus in PASSALIDAE: PASSALINAE: †PROTOPASSALINI. Note: Lower Cretaceous (Brazil).

Type species: †*Protopassalusararipensis* Aquino dos Santos, Mattos, Mermudes, Scheffler & Reyes-Castillo, 2021, by original designation.

Family-group name: †PROTOPASSALINI Aquino dos Santos, Mattos, Mermudes, Scheffler & Reyes-Castillo, 2021: 4. Stem: *Protopassal*-.

***Protopaussus* Gestro, 1892**: 706. Current status: valid genus in CARABIDAE: PAUSSINAE: PROTOPAUSSINI.

Type species: *Protopaussusfeae* Gestro, 1892, by monotypy.

Family-group name: PROTOPAUSSINI Gestro, 1892. Stem: *Protopauss*-.

***Protopeltis* Crowson, 1964**: 287. Current status: valid genus in PROTOPELTIDAE.

Type species: *Grynomaviridescens* Broun, 1886, by original designation.

Family-group name: PROTOPELTINI Crowson, 1966. Stem: *Protopelt*-.

***Protopselaphus* Newton & Thayer, 1995**: 227. Current status: valid genus in STAPHYLINIDAE: PROTOPSELAPHINAE.

Type species: *Protopselaphuswatrousi* Newton & Thayer, 1995, by original designation.

Family-group name: PROTOPSELAPHINAE Newton & Thayer, 1995. Stem: *Protopselaph*-.

†***Protorabus* Ponomarenko, 1977**: 72. Current status: valid genus in CARABIDAE: †PROTORABINAE. Note: Upper Jurassic (Kazakhstan).

Type species: †*Protorabusplanus* Ponomarenko, 1977, by original designation.

Family-group name: †PROTORABINAE Ponomarenko, 1977. Stem: *Protorab*-.

***Protorhopala* J. Thomson, 1860a**: 91. Current status: valid genus in CERAMBYCIDAE: LAMIINAE: PTEROPLIINI.

Type species: *Lamiasexnotata* Klug, 1833, by monotypy.

Family-group name: PROTORHOPALITAE J. Thomson, 1864. Stem: *Protorhopal*-.

†***Protoscelis* L.N. Medvedev, 1968**: 156. Current status: valid genus in CHRYSOMELIDAE: †PROTOSCELIDINAE. Note: Cretaceous, Upper Jurassic (China, Kazakhstan).

Type species: †*Protoscelisjurassica* L.N. Medvedev, 1968, by original designation.

Family-group name: †PROTOSCELINAE L.N. Medvedev, 1968. Stem: *Protoscelid*-.

***Protosphindus* Sen Gupta & Crowson, 1979**: 181. Current status: valid genus in SPHINDIDAE: PROTOSPHINDINAE.

Type species: *Protosphinduschilensis* Sen Gupta & Crowson, 1979, by original designation.

Family-group name: PROTOSPHINDINAE Sen Gupta & Crowson, 1979. Stem: *Protosphind*-.

***Protosternum* Sharp, 1890a**: 356. Current status: valid genus in HYDROPHILIDAE: SPHAERIDIINAE: PROTOSTERNINI.

Type species: *Protosternumatomarium* Sharp, 1890, by monotypy.

Family-group name: PROTOSTERNINI Hansen, 1991. Stem: *Protostern*-.

***Protosthetops* Perkins in Perkins and J. Balfour-Browne, 1994**: 8, in key. Current status: valid genus in HYDRAENIDAE: PROSTHETOPINAE: PROTOSTHETOPINI.

Type species: *Prosthetopskenyensis* Orchymont, 1948, by original designation.

Family-group name: PROTOSTHETOPINI Perkins, 1994. Stem: *Protosthetop*-.

***Prototrichapion* Voss, 1959**: 316. Current status: valid genus in BRENTIDAE: APIONINAE: ASPIDAPIITAE: PROTOTRICHAPIINI.

Type species: *Apionpenicillatum* G.A.K. Marshall, 1932, by original designation.

Family-group name: PROTOTRICHAPIINI Wanat, 1995. Stem: *Prototrichapi*-.

†***Prototrox* Nikolajev, 2000a**: 65. Current status: valid genus in SCARABAEIDAE: †PROTOTROGINAE. Note: Lower Cretaceous (Mongolia; Russia).

Type species: †*Prototroxtransbaikalicus* Nikolajev, 2000, by original designation.

Family-group name: †PROTOTROGINAE Nikolajev, 2000. Stem: *Prototrog*-.

***Proutianus* Muona, 1993**: 24. Current status: valid genus in EUCNEMIDAE: EUCNEMINAE: PROUTIANINI.

Type species: *Phaenocerusamericanus* G.H. Horn, 1872, by original designation.

Family-group name: PROUTIANINI Muona, 1993. Stem: *Proutian*-.

***Prypnus* Schönherr, 1823**: 1139. Current status: valid genus in CURCULIONIDAE: ENTIMINAE: POLYDRUSITAE: PRYPNINI.

Type species: *Prypnusquinquenodosus* Schönherr, 1823, by original designation.

Family-group name: PRYPNIDES Lacordaire, 1863. Stem: *Prypn*-.

***Psalidognathus* G.R. Gray in Griffith and Pidgeon, 1831**: pls 6, 14. Current status: valid genus in CERAMBYCIDAE: PRIONINAE: PRIONINI.

Type species: *Psalidognathusfriendii* G.R. Gray, 1831, by monotypy.

Family-group name: PSALIDOGNATHITAE J. Thomson, 1861. Stem: *Psalidognath*-.

***Psallidium* Herbst, 1795**: 342. Current status: valid genus in CURCULIONIDAE: ENTIMINAE: POLYDRUSITAE: PSALLIDIINI.

Type species: *Curculiomaxillosus* Fabricius, 1792, by monotypy.

Family-group name: PSALIDIIDES Lacordaire, 1863. Stem: *Psallidi*-.

†***Psammaegialia* Nikolajev, B. Wang & Zhang, 2014**: 221. Current status: valid genus in SCARABAEIDAE: APHODIINAE: †PSAMMAEGIALIINI. Note: Lower Cretaceous (China).

Type species: †*Psammaegialiaabdita* Nikolajev, B. Wang & Zhang, 2014, by original designation.

Family-group name: †PSAMMAEGIALIINI Nikolajev, B. Wang & Zhang, 2014: 220. Stem: *Psammaegiali*-.

***Psammobius* Heer, 1841**: 531. Current status: junior synonym of *Psammodius* Fallén, 1807 in SCARABAEIDAE: APHODIINAE: PSAMMODIINI: PSAMMODIINA.

Type species: *Aphodiussulcicollis* Illiger, 1802 (= *Scarabaeusasper* Fabricius, 1775), by subsequent designation (Tesař 1957: 168).

Family-group name: PSAMMOBIINA Reitter, 1909. Stem: *Psammobi*-. Note: junior homonym of PSAMMOBIIDAE Fleming, 1828 (type genus *Psammobia* Lamarck, 1818) in Mollusca.

***Psammodius* Fallén, 1807**: 37. Current status: valid genus in SCARABAEIDAE: APHODIINAE: PSAMMODIINI: PSAMMODIINA.

Type species: *Aphodiussulcicollis* Illiger, 1802 (= *Scarabaeusasper* Fabricius, 1775), by subsequent designation (Curtis 1829: pl. 258).

Family-group name: PSAMMODIAIRES Mulsant, 1842. Stem: *Psammodi*-.

***Psammoecus* Latreille, 1829b**: 135. Current status: valid genus in SILVANIDAE: BRONTINAE: TELEPHANINI.

Type species: *Notoxusbipunctatus* Fabricius, 1792, by monotypy.

Family-group name: PSAMMOECINI Reitter, 1879. Stem: *Psammoec*-.

***Psathyrus* J. Thomson, 1857**: 192. Current status: valid genus in CERAMBYCIDAE: CERAMBYCINAE: AUXESINI.

Type species: *Psathyrusaeolis* J. Thomson, 1857, by monotypy.

Family-group name: PSATHYRINI Quentin, 1954. Stem: *Psathyr*-.

***Psebium* Pascoe, 1864b**: 289. Current status: valid genus in CERAMBYCIDAE: CERAMBYCINAE: PSEBIINI.

Type species: *Psebiumbrevipenne* Pascoe, 1864, by monotypy.

Family-group name: PSÉBIIDES Lacordaire, 1868. Stem: *Psebi*-.

***Pselaphus* Herbst, 1791**: 106. Current status: valid genus in STAPHYLINIDAE: PSELAPHINAE: PSELAPHITAE: PSELAPHINI.

Type species: *Pselaphusheisei* Herbst, 1791, by subsequent designation (Guérin-Méneville 1828: 316).

Family-group name: PSELAPHII Latreille, 1802. Stem: *Pselaph*-.

***Pselaptus* J.L. LeConte, 1881a**: 184. Current status: valid genus in STAPHYLINIDAE: PSELAPHINAE: GONIACERITAE: BRACHYGLUTINI: BRACHYGLUTINA.

Type species: *Pselaptusbelfragei* J.L. LeConte, 1881, by monotypy.

Family-group name: PSELAPTINA Park, 1976. Stem: *Pselapt*-.

***Psenocerus* J.L. LeConte, 1852a**: 158. Current status: valid genus in CERAMBYCIDAE: LAMIINAE: DESMIPHORINI.

Type species: **here fixed** (under ICZN 1999a, Article 70.3.2) as *Clytussupernotatus* Say, 1824, misidentified as *Callidiumpini* G.-A. Olivier, 1795 (sensu J.L. LeConte, 1852) in the original designation by monotypy in J.L. LeConte (1852a: 158).

Family-group name: PSENOCERINI J.L. LeConte, 1873. Stem: *Psenocer*-.

***Psephenoides* Gahan, 1914**: 189. Current status: valid genus in PSEPHENIDAE: PSEPHENOIDINAE.

Type species: *Psephenoidesimmsi* Gahan, 1914, by original designation.

Family-group name: PSEPHENOIDINI Bollow, 1938. Stem: *Psephenoid*-.

***Psephenus* Haldeman in Melsheimer, 1853**: 34. Current status: valid genus in PSEPHENIDAE: PSEPHENINAE. Note: new replacement name for *Eurypalpus* J.L. LeConte, 1850.

Type species: *Eurypalpuslecontei* J.L. LeConte, 1850 (= *Fluvicolaherricki* DeKay, 1844), by monotypy (type fixation for *Eurypalpus* J.L. LeConte).

Family-group name: PSÉPHÉNIDES Lacordaire, 1854. Stem: *Psephen*-.

***Psepholax* A. White in A. White and Doubleday, 1843**: 275. Current status: valid genus in CURCULIONIDAE: CRYPTORHYNCHINAE: PSEPHOLACINI.

Type species: *Psepholaxsulcatus* A. White, 1843, by monotypy.

Family-group name: PSÉPHOLACIDES Lacordaire, 1865. Stem: *Psepholac*-.

***Pseudacanthus* Kaup, 1868b**: 19. Current status: valid genus in PASSALIDAE: PASSALINAE: PROCULINI.

Type species: *Passalusmexicanus* Truqui, 1857, by subsequent designation (Gravely 1918: 30).

Family-group name: PSEUDACANTHEAE Kaup, 1871. Stem: *Pseudacanth*-.

***Pseudacherusia* Kerremans, 1905**: 382. Current status: valid genus in BUPRESTIDAE: POLYCESTINAE: TYNDARIDINI: PSEUDACHERUSIINA.

Type species: *Pseudacherusiatheryi* Kerremans, 1905, by monotypy.

Family-group name: PSEUDOACHERUSINI Cobos, 1980. Stem: *Pseudacherusi*-.

***Pseudadoretus* Semenov, 1889b**: 202. Current status: valid genus in SCARABAEIDAE: RUTELINAE: ADORETINI: ADORETINA.

Type species: *Pseudadoretusdilutellus* Semenov, 1889, by original designation. Note: *Adoretusphthisicus* Dohrn, 1882 is sometimes cited as the type species of this genus (e.g., Zorn and A. Bezděk 2016: 320) but Semenov (1889b: 202) clearly designated *P.dilutellus* [as *ditutellus*] as type species; two spellings were originally used for the type species, including *ditutellus* (p. 202); as far as we know no one has acted as First Reviser (ICZN 1999a, Article 24.2.3) and we **here select***dilutellus* as the correct spelling.

Family-group name: PSEUDADORETINA Ohaus, 1912. Stem: *Pseudadoret*-.

***Pseudaplatopterus* Kleine, 1940**: 179. Current status: valid genus in LYCIDAE: EROTINAE: EROTINI.

Type species: *Aplatopterusscheelei* Kleine, 1940, by monotypy. Note: just after Kleine’s (1940: 179) description of *Pseudaplatopterus*, he described a new species under the name “A. Scheeli” in honor of Major Scheele; Kazantsev (2013c: 97) used the spelling “*ascheelei*” for the type species but we believe the “A.” is either an error for “P.” or stands as an abbreviation for *Aplatopterus* Reitter, 1911; we think the second option is more likely the correct one.

Family-group name: PSEUDAPLATOPTERINA Kazantsev, 2012b: 399. Stem: *Pseudaplatopteri*-.

***Pseudapotrepus* Champion, 1909**: 21. Current status: valid genus in CURCULIONIDAE: COSSONINAE: PSEUDAPOTREPINI.

Type species: *Pseudapotrepusmacrophthalmus* Champion, 1909, by original designation.

Family-group name: PSEUDAPOTREPIDES Champion, 1909. Stem: *Pseudapotrep*-.

***Pseudauletes* Voss, 1922a**: 27, 95. Current status: valid genus in ATTELABIDAE: RHYNCHITINAE: RHYNCHITITAE: AULETINI: PSEUDAULETINA.

Type species: *Rhynchitesrufiventris* Jekel, 1860, by subsequent designation (Legalov 2011e: 96).

Family-group name: PSEUDAULETINA Voss, 1933. Stem: *Pseudaulet*-.

***Pseudeucinetus* Heller, 1921**: 155. Current status: valid genus in LIMNICHIDAE: THAUMASTODINAE.

Type species: *Pseudeucinetuszygops* Heller, 1921, by original designation.

Family-group name: PSEUDEUCINETINI Csiki, 1924. Stem: *Pseudeucinet*-.

***Pseudobagous* Sharp, 1917**: 27. Current status: valid genus in CURCULIONIDAE: BAGOINAE.

Type species: *Bagouslongulus* Gyllenhal, 1836, by original designation.

Family-group name: PSEUDOBAGOINI Sharp, 1917. Stem: *Pseudobago*-.

***Pseudobalaninus* Faust, 1889**: 97. Current status: valid genus in CURCULIONIDAE: CURCULIONINAE: CURCULIONINI: PSEUDOBALANININA.

Type species: *Pseudobalaninussemifasciatus* Faust, 1889, by subsequent designation (Heller 1925b: 93).

Family-group name: PSEUDOBALANININA Heller, 1925. Stem: *Pseudobalanin*-.

†***Pseudobrienia* Legalov, 2012**: 72. Current status: valid genus in †OBRIENIIDAE: †PSEUDOBRIENIINAE. Note: Upper Jurassic (Kazakhstan).

Type species: †*Pseudobrieniarasnitsyni* Legalov, 2012, by original designation.

Family-group name: †PSEUDOBRIENIINAE Legalov, 2012: 73. Stem: *Pseudobrieni*-.

***Pseudoceocephalus* Kleine, 1920**: 18. Current status: valid genus in BRENTIDAE: BRENTINAE: TRACHELIZINI: PSEUDOCEOCEPHALINA.

Type species: *Brentusdepressus* Lund, 1802, by original designation.

Family-group name: PSEUDOCEOCEPHALIDAE Kleine, 1922. Stem: *Pseudoceocephal*-.

***Pseudocephalus* Newman, 1842b**: 353. Current status: valid genus in CERAMBYCIDAE: CERAMBYCINAE: PSEUDOCEPHALINI.

Type species: *Pseudocephalusformicides* Newman, 1842, by monotypy.

Family-group name: PSEUDOCEPHALINI Aurivillius, 1912. Stem: *Pseudocephal*-.

***Pseudochodaeus* Carlson & Ritcher, 1974**: 99. Current status: valid genus in OCHODAEIDAE: CHAETOCANTHINAE: PSEUDOCHODAEINI.

Type species: *Ochodaeusestriatus* Schaeffer, 1906, by original designation.

Family-group name: PSEUDOCHODAEINI Scholtz, 1988. Stem: *Pseudochodae*-.

***Pseudocistela* Crotch, 1874b**: 108. Current status: valid genus in TENEBRIONIDAE: ALLECULINAE: ALLECULINI: GONODERINA.

Type species: *Cistelabrevis* Say, 1824, by subsequent designation (Novák and Pettersson 2008: 327).

Family-group name: PSEUDOCISTELINI Portevin, 1934. Stem: *Pseudocistel*-.

***Pseudocneorhinus* Roelofs, 1873**: 177. Current status: valid genus in CURCULIONIDAE: ENTIMINAE: CYPHICERITAE: TRACHYPHLOEINI: PSEUDOCNEORHININA.

Type species: *Pseudocneorhinusobesus* Roelofs, 1873, by monotypy.

Family-group name: PSEUDOCNEORRHININI Kôno, 1930. Stem: *Pseudocneorhin*-.

***Pseudocolaspis* Laporte, 1833a**: 19, 23. Current status: valid genus in CHRYSOMELIDAE: EUMOLPINAE: BROMIINI.

Type species: *Pseudocolaspiscoerulea* Laporte, 1833, by subsequent designation (Baly 1878a: 248).

Family-group name: PSEUDOCOLASPITES Chapuis, 1874. Stem: *Pseudocolaspid*-.

***Pseudocrioceris* Pic, 1916b**: 3. Current status: valid genus in CHRYSOMELIDAE: CRIOCERINAE: PSEUDOCRIOCERINI.

Type species: *Brachydactylaannulipes* Pic, 1913, by **present designation**. Note: Heinze (1962: 201) listed *Criocerisdiscoidea* Guérin-Méneville, 1844 as the type species of *Pseudocrioceris* Pic, 1916 but this is not an originally included nominal species.

Family-group name: PSEUDOCRIOCERINI Heinze, 1962. Stem: *Pseudocriocer*-.

***Pseudocycnotrachelus* Legalov, 2003**: 525. Current status: valid genus in ATTELABIDAE: APODERINAE: APODERINI.

Type species: *Apoderusledyardi* Heller, 1915, by original designation.

Family-group name: PSEUDOCYCNOTRACHELINA Legalov, 2003. Stem: *Pseudocycnotrachel*-.

***Pseudocyphus* Schaeffer, 1905**: 179. Current status: junior synonym of *Platyomus* C.R. Sahlberg, 1823 in CURCULIONIDAE: ENTIMINAE: POLYDRUSITAE: NAUPACTINI: NAUPACTINA.

Type species: *Pseudocyphusflexicaulis* Schaeffer, 1905, by monotypy.

Family-group name: PSEUDOCYPHI Pierce, 1913. Stem: *Pseudocyph*-.

***Pseudodorcus* Parry, 1870**: 94. Current status: valid genus in LUCANIDAE: LUCANINAE: LUCANINI.

Type species: *Lucanushydrophiloides* Hope, 1845, by monotypy.

Family-group name: PSEUDODORCINI Benesh, 1960. Stem: *Pseudodorc*-.

***Pseudolampsis* G.H. Horn, 1889**: 174. Current status: valid genus in CHRYSOMELIDAE: GALERUCINAE: ALTICINI.

Type species: *Hypolampsisguttatus* J.L. LeConte, 1884, by monotypy.

Family-group name: PSEUDOLAMPSES G.H. Horn, 1889. Stem: *Pseudolampse*-.

***Pseudoleptura* J. Thomson, 1861**: 142, 148. Current status: junior synonym of *Erythrus* A. White, 1853 in CERAMBYCIDAE: CERAMBYCINAE: PSEUDOLEPTURINI. Note: unnecessary new replacement name for *Erythrus* A. White, 1853.

Type species: *Erythruschampioni* A. White, 1853, by original designation.

Family-group name: PSEUDOLEPTURITAE J. Thomson, 1861. Stem: *Pseudoleptur*-.

***Pseudoliodes* Portevin, 1926**: 78. Current status: junior synonym of *Pseudcolenis* Reitter, 1885 in LEIODIDAE: LEIODINAE: PSEUDOLIODINI.

Type species: *Pseudcolenisgrandis* Portevin, 1905, by original designation.

Family-group name: PSEUDOLIODINI Portevin, 1926. Stem: *Pseudoliod*-.

***Pseudolotelus* Pic, 1901**: 67. Current status: valid genus in ADERIDAE.

Type species: *Euglenespunctatissimus* Reitter, 1885, by subsequent designation (Nardi 2007: 29).

Family-group name: PSEUDOLOTELINA Báguena Corella, 1948. Stem: *Pseudolotel*-.

***Pseudomasoreus* Desbrochers des Loges, 1904**: 140, 163. Current status: valid genus in CARABIDAE: HARPALINAE: LEBIINI: CYMINDIDINA.

Type species: *Cymindiscanigoulensis* Fairmaire & Laboulbène, 1854, by monotypy.

Family-group name: PSEUDOMASOREINI Jeannel, 1942. Stem: *Pseudomasore*-.

***Pseudomelanactes* Mathieu, 1961**: 476. Current status: junior synonym of *Anthracalaus* Fairmaire, 1889 in ELATERIDAE: AGRYPNINAE: PSEUDOMELANACTINI.

Type species: *Melanactesagrypnoides* Van Dyke, 1932, by original designation.

Family-group name: PSEUDOMELANACTINI Arnett, 1967. Stem: *Pseudomelanact*-.

***Pseudomenes* Fleutiaux, 1902**: 663. Current status: valid genus in EUCNEMIDAE: PSEUDOMENINAE. Note: new replacement name for *Eumenes* Bonvouloir, 1871 [cited by Fleutiaux as 1875].

Type species: *Eumenesbakewelli* Bonvouloir, 1872, by subsequent monotypy (type fixation for *Eumenes* Bonvouloir).

Family-group name: PSEUDOMENINAE Muona, 1993. Stem: *Pseudomen*-.

***Pseudomesauletes* Legalov, 2001b**: 53. Current status: valid genus in ATTELABIDAE: RHYNCHITINAE: RHYNCHITITAE: AULETINI: PSEUDOMESAULETINA.

Type species: *Auletesuniformis* Roelofs, 1875, by original designation.

Family-group name: PSEUDOMESAULETINA Legalov, 2003. Stem: *Pseudomesaulet*-.

***Pseudometrioxena* Legalov, 2023b**: 39. Current status: valid genus in BELIDAE: OXYCORYNINAE: OXYCORYNITAE: METRIOXENINI: PSEUDOMETRIOXENINA.

Type species: *Metrioxenadecisa* Pascoe, 1885, by original designation.

Family-group name: PSEUDOMETRIOXENINA Legalov, 2023: 39. Stem: *Pseudometrioxen*-.

***Pseudomimus* Hartmann, 1904**: 416. Current status: valid genus in CURCULIONIDAE: COSSONINAE: RHYNCOLINI: PSEUDOMIMINA.

Type species: *Pseudomimuscorpulentus* Hartmann, 1904, by monotypy.

Family-group name: PSEUDOMIMINI Voss, 1939. Stem: *Pseudomim*-.

***Pseudomorpha* W. Kirby, 1823a**: 98. Current status: valid genus in CARABIDAE: HARPALINAE: PSEUDOMORPHINI. Note: W. Kirby originally used two different spellings for this taxon, including *Heteromorpha* (p. 109); subsequently, W. Kirby (1823b: 420) used only *Pseudomorpha* and therefore acted as First Reviser (ICZN 1999a, Article 24.2.4).

Type species: *Pseudomorphaexcrucians* W. Kirby, 1823, by monotypy.

Family-group name: HETEROMORPHIDAE Hope, 1838. Stem: *Pseudomorph*-.

***Pseudoperinthus* Wasmann, 1916a**: 194. Current status: valid genus in STAPHYLINIDAE: ALEOCHARINAE: PSEUDOPERINTHINI.

Type species: *Pseudoperinthusmalayanus* Wasmann, 1916, by monotypy.

Family-group name: PSEUDOPERINTHINAE Cameron, 1939. Stem: *Pseudoperinth*-.

***Pseudoperotis* Obenberger, 1936**: 115. Current status: valid genus in BUPRESTIDAE: CHRYSOCHROINAE: DICERCINI: PSEUDOPEROTINA.

Type species: *Oedisternascabrosula* Obenberger, 1936, by original designation.

Family-group name: PSEUDOPEROTINA Tôyama, 1987. Stem: *Pseudoperot*-.

***Pseudophanus* J.L. LeConte, 1859a**: 84. Current status: junior synonym of *Cryptomorpha* Wollaston, 1854 in SILVANIDAE: BRONTINAE: TELEPHANINI.

Type species: *Pseudophanussignatus* J.L. LeConte, 1859 (= *Psammoecusdesjardinsii* Guérin- Méneville, 1844), by monotypy.

Family-group name: PSEUDOPHANINI J.L. LeConte, 1861. Stem: *Pseudophan*-. Note: junior homonym of PSEUDOPHANINAE Amyot & Audinet-Serville, 1843 (type genus *Pseudophana* Burmeister, 1835) in Hemiptera.

***Pseudophengodes* Pic, 1930**: 319. Current status: valid genus in PHENGODIDAE: PHENGODINAE.

Type species: *Phengodespulchella* Guérin-Méneville, 1843, by subsequent designation (Wittmer 1976b: 425).

Family-group name: PSEUDOPHENGODIDAE Pic, 1930. Stem: *Pseudophengod*-.

***Pseudophrysus* Legalov, 2003**: 479. Current status: valid genus in ATTELABIDAE: APODERINAE: CLITOSTYLINI: PSEUDOPHRYSINA.

Type species: *Cycnotrachelusparvulus* Voss, 1928, by original designation.

Family-group name: PSEUDOPHRYSINA Legalov, 2003. Stem: *Pseudophrys*-.

***Pseudoplandria* Fenyes, 1921**: 30. Current status: valid genus in STAPHYLINIDAE: ALEOCHARINAE: HOPLANDRIINI: PSEUDOPLANDRIINA.

Type species: *Pseudoplandrialaeta* Fenyes, 1921, by original designation.

Family-group name: PSEUDOPLANDRIINA Hanley, 2002. Stem: *Pseudoplandri*-.

***Pseudopsis* Newman, 1834**: 313. Current status: valid genus in STAPHYLINIDAE: PSEUDOPSINAE.

Type species: *Pseudopsissulcatus* Newman, 1834, by monotypy.

Family-group name: PSEUDOPSINI Ganglbauer, 1895. Stem: *Pseudops*-.

***Pseudoryctes* Sharp, 1873**: 267. Current status: valid genus in SCARABAEIDAE: DYNASTINAE: PENTODONTINI: PSEUDORYCTINA.

Type species: *Oryctesmullerianus* A. White, 1859, by monotypy.

Family-group name: PSEUDORYCTINA Carne, 1957. Stem: *Pseudoryct*-.

***Pseudotrechus* Rosenhauer, 1856**: 22. Current status: valid genus in CARABIDAE: HARPALINAE: LEBIINI: PSEUDOTRECHINA.

Type species: *Pseudotrechusmutilatus* Rosenhauer, 1856, by monotypy.

Family-group name: PSEUDOTRECHINI Basilewsky, 1984. Stem: *Pseudotrech*-.

***Pseudozaena* Laporte, 1834b**: 55. Current status: valid genus in CARABIDAE: PAUSSINAE: OZAENINI.

Type species: *Ozaenamegacephala* Laporte, 1834 (= *Ozaenaorientalis* Klug, 1834), by monotypy.

Family-group name: PSEUDOZAENINI Sloane, 1905. Stem: *Pseudozaen*-.

***Psilapha* Clark, 1865**: 377, 389. Current status: valid genus in CHRYSOMELIDAE: GALERUCINAE: ALTICINI.

Type species: *Psilaphaflava* Clark, 1865, by monotypy.

Family-group name: PSILAPHINA Bechyné & Špringlová de Bechyné, 1973. Stem: *Psilaph*-.

***Psilocladus* Blanchard, 1845b**: 121. Current status: valid genus in LAMPYRIDAE: PSILOCLADINAE.

Type species: *Psilocladusmiltoderus* Blanchard, 1845, by monotypy.

Family-group name: PSILOCLADINA McDermott, 1964. Stem: *Psiloclad*-.

***Psilomorpha* Saunders, 1850**: 80. Current status: valid genus in CERAMBYCIDAE: CERAMBYCINAE: PSILOMORPHINI.

Type species: *Psilomorphatenuipes* Saunders, 1850, by monotypy.

Family-group name: PSILOMORPHIDES Lacordaire, 1868. Stem: *Psilomorph*-.

***Psilonychus* Burmeister, 1855**: 288. Current status: valid genus in SCARABAEIDAE: MELOLONTHINAE: SCHIZONYCHINI.

Type species: *Psilonychusecklonii* Burmeister, 1855, by **present designation**.

Family-group name: PSILONYCHIDES Péringuey, 1904. Stem: *Psilonych*-.

***Psiloptera* Dejean, 1833a**: 76. Current status: valid genus in BUPRESTIDAE: CHRYSOCHROINAE: DICERCINI: DICERCINA.

Type species: *Buprestisattenuata* Fabricius, 1792, by subsequent designation (J. Thomson 1878: 29).

Family-group name: PSILOPTÉRIDES Lacordaire, 1857. Stem: *Psilopter*-. Note: senior homonym of PSILOPTERINAE Dolgopol de Saez, 1927 (type genus *Psilopterus* Moreno & Mercerat, 1891) in Aves.

***Psoa* Herbst, 1797**: 214. Current status: valid genus in BOSTRICHIDAE: PSOINAE: PSOINI.

Type species: *Psoaviennensis* Herbst, 1797, by monotypy.

Family-group name: PSOITAS Blanchard, 1851. Stem: *Pso*-.

***Psydrus* J.L. LeConte, 1846**: 153. Current status: valid genus in CARABIDAE: PSYDRINAE: PSYDRINI.

Type species: *Psydruspiceus* J.L. LeConte, 1846, by monotypy.

Family-group name: PSYDRI J.L. LeConte, 1853. Stem: *Psydr*-.

***Psylliodes* Latreille, 1829b**: 154. Current status: valid genus in CHRYSOMELIDAE: GALERUCINAE: ALTICINI.

Type species: *Galerucaaffinis* Paykull, 1799, by subsequent designation (Blanchard in Audouin et al. 1844: pl. 73). Note: *Chrysomelachrysocephala* Linnaeus, 1758 is the type species usually listed for this genus (e.g., Konstantinov and Vanderberg 1996: 373; Döberl 2010: 552) by subsequent designation of Maulik (1926: 144) but Blanchard’s designation is earlier; both species are currently included in the nominotypical subgenus of *Psylliodes* Latreille.

Family-group name: PSYLLIODITES Chapuis, 1875. Stem: *Psylliod*-.

***Psyllobora* Chevrolat in Dejean, 1836a**: 434. Current status: valid genus in COCCINELLIDAE: COCCINELLINAE: COCCINELLINI.

Type species: *Coccinellalineola* Fabricius, 1792 (= *Psylloborafabricii* Crotch, 1871), by subsequent designation (Timberlake 1943: 41).

Family-group name: PSYLLOBORINI Casey, 1899. Stem: *Psyllobor*-.

***Ptenidium* Erichson, 1845a**: 34. Current status: valid genus in PTILIIDAE: PTILIINAE: PTENIDIINI. Note: name given precedence over *Anisarthria* Stephens, 1830 whenever the two are considered synonyms and placed on the Official List of Generic Names in Zoology (ICZN 1984b).

Type species: *Scaphidiumpusillum* Gyllenhal, 1808, by subsequent designation (C.G. Thomson 1859: 63; see ICZN 1984b). Note: given precedence over *Dermestesmelas* Marsham, 1802 whenever the two are considered synonyms and placed on the Official List of Specific Names in Zoology (ICZN 1984b).

Family-group name: PTENIDIINI Flach, 1889. Stem: *Ptenidi*-.

***Pteracantha* Newman, 1838**: 392. Current status: valid genus in CERAMBYCIDAE: CERAMBYCINAE: TRACHYDERINI: TRACHYDERINA.

Type species: *Pteracanthafasciata* Newman, 1838, by monotypy.

Family-group name: PTERACANTHITAE J. Thomson, 1864. Stem: *Pteracanth*-.

***Pteracmes* Raffray, 1890a**: 95, 102. Current status: valid genus in STAPHYLINIDAE: PSELAPHINAE: EUPLECTITAE: TRICHONYCHINI: TRICHONYCHINA.

Type species: *Pteracmesschaufussi* Raffray, 1890, by monotypy.

Family-group name: PTERACMINI Jeannel, 1962. Stem: *Pteracmet*-.

***Ptericoptus* Lacordaire, 1830b**: 185. Current status: valid genus in CERAMBYCIDAE: LAMIINAE: APOMECYNINI.

Type species: *Ptericoptusdorsalis* Audinet-Serville, 1835, by subsequent monotypy (Audinet- Serville 1835a: 61).

Family-group name: PTÉRICOPTIDES Lacordaire, 1872. Stem: *Ptericopt*-.

***Pterobothris* Fairmaire & Germain, 1859**: 714. Current status: valid genus in BUPRESTIDAE: BUPRESTINAE: PTEROBOTHRINI.

Type species: *Pterobothriscorrosus* Fairmaire & Germain, 1859, by monotypy.

Family-group name: PTEROBOTHRINI Volkovitsh, 2001. Stem: *Pterobothr*-.

***Pterocolus* Schönherr in Say, 1831**: 5. Current status: valid genus in ATTELABIDAE: PTEROCOLINAE. Note: see Prena (2018: 331) for the authorship of this genus-group name.

Type species: *Attelabusovatus* Fabricius, 1801, by monotypy.

Family-group name: PTÉROCOLIDES Lacordaire, 1865. Stem: *Pterocol*-.

***Pterogenius* Candèze, 1861**: 363. Current status: valid genus in PTEROGENIIDAE.

Type species: *Pterogeniusnietneri* Candèze, 1861, by monotypy.

Family-group name: PTEROGENIIDAE Crowson, 1953. Stem: *Pterogeni*-.

***Pteroloma* Gyllenhal, 1827**: 418. Current status: valid genus in AGYRTIDAE: PTEROLOMATINAE.

Type species: *Harpalusforsstromii* Gyllenhal, 1810, by monotypy.

Family-group name: PTEROLOMINA C.G. Thomson, 1862. Stem: *Pterolomat*-.

***Pteronius* Blackwelder, 1952**: 331. Current status: junior synonym of *Proteinus* Latreille, 1797 in STAPHYLINIDAE: PROTEININAE: PROTEININI.

Type species: *Dermestesbrachypterus* Fabricius, 1792, by original designation.

Family-group name: PTERONINI Arnett, 1961. Stem: *Pteroni*-.

***Pteroplatus* Buquet, 1840b**: 287. Current status: valid genus in CERAMBYCIDAE: CERAMBYCINAE: PTEROPLATINI.

Type species: *Pteroplatuspulcher* Buquet, 1840, by subsequent designation (J. Thomson 1864: 258).

Family-group name: PTEROPLATITAE J. Thomson, 1861. Stem: *Pteroplat*-.

***Pteroplius* Lacordaire, 1830b**: 183. Current status: valid genus in CERAMBYCIDAE: LAMIINAE: PTEROPLIINI. Note: the original spelling was *Pterhoplius*, but *Pteroplius*, an incorrect subsequent spelling, is in prevailing usage, attributed to the publication of the original spelling, and therefore deemed to be the original spelling (ICZN 1999a, Article 33.3.1).

Type species: *Pteropliusacuminatus* Audinet-Serville, 1835, by subsequent monotypy. Note: Audinet-Serville (1835a: 66) included two species in the genus *Pteroplius*, one in the section ‘propriè dictus’ and one in the section Rhaphiptera, a new genus-group name; the type species of the genus is the species cited in the first section.

Family-group name: PTEROPLIITAE J. Thomson, 1860. Stem: *Pteropli*-.

***Pterosthetops* Perkins in Perkins and J. Balfour-Browne, 1994**: 17. Current status: valid genus in HYDRAENIDAE: PROSTHETOPINAE: PROSTHETOPINI.

Type species: *Pterosthetopsimpressus* Perkins & J. Balfour-Browne, 1994, by original designation.

Family-group name: PTEROSTHETOPINI Perkins, 1994. Stem: *Pterosthetop*-.

***Pterostichus* Bonelli, 1810**: Tab. Syn. Current status: valid genus in CARABIDAE: HARPALINAE: PTEROSTICHINI: PTEROSTICHINA.

Type species: *Carabusfasciatopunctatus* Creutzer, 1799, by subsequent designation (Curtis 1828: pl. 196). Note: no species were originally included in this genus by Bonelli (1810); the first ones were the four species listed by Panzer (1813: 70).

Family-group name: PTEROSTICHII Bonelli, 1810. Stem: *Pterostich*-.

***Pterotarsus* Guérin-Méneville, 1829a**: pl. 12. Current status: valid genus in ELATERIDAE: THYLACOSTERNINAE.

Type species: *Pterotarsushistrio* Guérin-Méneville, 1829, by monotypy.

Family-group name: PTEROTARSINI Fleutiaux, 1902. Stem: *Pterotars*-.

***Pterotus* J.L. LeConte, 1859a**: 86. Current status: valid genus in LAMPYRIDAE: PTEROTINAE.

Type species: *Pterotusobscuripennis* J.L. LeConte, 1859, by monotypy.

Family-group name: PTEROTINI J.L. LeConte, 1861. Stem: *Pterot*-.

***Ptichopus* Kaup, 1868b**: 20. Current status: valid genus in PASSALIDAE: PASSALINAE: PASSALINI.

Type species: *Passalusangulatus* Percheron, 1834, by monotypy.

Family-group name: PTICHOPINAE Kuwert, 1896. Stem: *Ptichopod*-.

***Ptilineurus* Reitter, 1902**: 24. Current status: valid genus in PTINIDAE: DRYOPHILINAE: PTILINEURINI.

Type species: *Ptilinusmarmoratus* Reitter, 1877, by monotypy.

Family-group name: PTILINEURINI Böving, 1927. Stem: *Ptilineur*-.

***Ptilinus* Geoffroy, 1762**: 64. Current status: valid genus in PTINIDAE: PTILININAE: PTILININI. Note: name placed on the Official List of Generic Names in Zoology (ICZN 1994a).

Type species: *Ptilinusfuscus* Geoffroy, 1785, by subsequent monotypy (O.F. Müller 1776: 81), as *Ptilinuscylindricus* O.F. Müller, 1776, the name of the senior subjective synonym of *P.fuscus* Geoffroy that has been suppressed for the purposes of both Principles of Priority and Homonymy; see ICZN 1994a. Note: *Ptilinusfuscus* Geoffroy, 1785 was placed on the Official List of Specific Names in Zoology (ICZN 1994a).

Family-group name: PTILINIDAE Shuckard, 1839. Stem: *Ptilin*-.

***Ptilium* Gyllenhal, 1827**: 292. Current status: valid genus in PTILIIDAE: PTILIINAE: PTILIINI. Note: name placed on the Official List of Generic Names in Zoology (ICZN 1984b).

Type species: *Ptiliumcaesum* Erichson, 1845, by plenary power (see ICZN 1984b). Note: name placed on the Official List of Specific Names in Zoology (ICZN 1984b).

Family-group name: PTILINA Erichson, 1845. Stem: *Ptili*-.

***Ptilodactyla* Illiger, 1807b**: 342. Current status: valid genus in PTILODACTYLIDAE: PTILODACTYLINAE.

Type species: *Ptilodactylaelaterina* Illiger, 1807 (= *Pyrochroanitida* DeGeer, 1775), by monotypy.

Family-group name: PTILODACTYLIDAE Laporte, 1838. Stem: *Ptilodactyl*-.

***Ptilophorus* Dejean, 1834**: 218. Current status: valid genus in RIPIPHORIDAE: PTILOPHORINAE.

Type species: *Pelecotomadufourii* Latreille, 1818, by monotypy.

Family-group name: PTILOPHORINI Gerstaecker, 1855. Stem: *Ptilophor*-.

***Ptilopterium* Gistel, 1848b**: x. Current status: junior synonym of *Ptilium* Gyllenhal, 1827 in PTILIIDAE: PTILIINAE: PTILIINI. Note: unnecessary new replacement name for *Ptilium* Gyllenhal, 1827.

Type species: *Ptiliumcaesum* Erichson, 1845, by plenary power (see ICZN 1984b) (type fixation for *Ptilium* Gyllenhal).

Family-group name: PTILOPTERIIDAE Gistel, 1848. Stem: *Ptilopteri*-.

***Ptinella* Motschulsky, 1845a**: 819. Current status: valid genus in PTILIIDAE: PTILIINAE: PTINELLINI. Note: this genus is usually credited to Motschulsky (“1844”/1845a: 819) in the literature, referring to volume 17 (part 4) of the ‘Bulletin de la Société Impériale des Naturalistes de Moscou’, which was issued by 27 January 1845 (Bousquet and Davies 2021: 15); however, it is possible that *Ptinella* was first made available in the December issue of the ‘Revue Zoologique’ for the year 1844, which was received by the Académie des Sciences of France, also on 27 January 1845 (see Motschulsky, 1844b: 446); *Ptinella* Motschulsky, 1845 was placed on the Official List of Generic Names in Zoology (ICZN 1985b); we prefer to use the data as it appears on the Official List until the date priority of the two publications can be ascertained.

Type species: *Ptiliumapterum* Guérin-Méneville, 1839, by plenary power (see ICZN 1985b). Note: name placed on the Official List of Specific Names in Zoology (ICZN 1985b).

Family-group name: PTINELLINI Reitter, 1906. Stem: *Ptinell*-.

***Ptinus* Linnaeus, 1767**: 537, 565. Current status: valid genus in PTINIDAE: PTININAE: PTININI. Note: name placed on the Official List of Generic Names in Zoology (ICZN 1995b).

Type species: *Cerambyxfur* Linnaeus, 1758, by subsequent designation (Latreille 1810: 427; see ICZN 1995b). Note: name placed on the Official List of Specific Names in Zoology (ICZN 1995b).

Family-group name: PTINIORES Latreille, 1802. Stem: *Ptin*-.

†***Ptisma* Kirejtshuk & Azar in Kirejtshuk et al., 2016a**: 204. Current status: valid genus in CLAMBIDAE. Note: Lower Cretaceous (Lebanon).

Type species: †*Ptismazasukhae* Kirejtshuk & Azar, 2016, by original designation. Note: two original spellings were used in Kirejtshuk et al. (2016a): *P.zhasukhai* (p. 204) and *P.zasukhae* (pp. 201–209); Kirejtshuk et al. (2016b: 275) acted as First Revisers (ICZN 1999a, Article 24.2.4) and selected *P.zasukhae*.

Family-group name: †PTISMIDAE Kirejtshuk & Azar in Kirejtshuk et al., 2016a: 202. Stem: *Ptism*-.

***Ptochus* Schönherr, 1826**: 187. Current status: valid genus in CURCULIONIDAE: ENTIMINAE: CYPHICERITAE: CYPHICERINI: MYLLOCERINA. Note: name placed on the Official List of Generic Names in Zoology (ICZN 1990d).

Type species: *Ptochusporcellus* Boheman, 1834, by subsequent designation (Marshall 1916: 259; see ICZN 1990d). Note: name placed on the Official List of Specific Names in Zoology (ICZN 1990d).

Family-group name: PTOCHINI Reitter, 1913. Stem: *Ptoch*-.

***Ptomaphaginus* Portevin, 1914**: 194. Current status: valid genus in LEIODIDAE: CHOLEVINAE: PTOMAPHAGINI: PTOMAPHAGININA.

Type species: *Ptomaphaginuslongitarsis* Portevin, 1914, by monotypy.

Family-group name: PTOMAPHAGININI Szymczakowski, 1964. Stem: *Ptomaphagin*-.

***Ptomaphagus* Hellwig, 1795**: 358. Current status: valid genus in LEIODIDAE: CHOLEVINAE: PTOMAPHAGINI: PTOMAPHAGINA.

Type species: *Tritomasericea* Fabricius, 1787 (= *Silphasubvillosa* Goeze, 1777), by monotypy. Note: Goeze’s (1777) work is not consistently binominal (see ICZN 2021a) and therefore an application to the Commission is necessary to retain *Silphasubvillosa* Goeze, 1777 as the valid name for the type species.

Family-group name: PTOMAPHAGINI Jeannel, 1911. Stem: *Ptomaphag*-.

***Ptosima* Dejean, 1833a**: 79. Current status: valid genus in BUPRESTIDAE: POLYCESTINAE: PTOSIMINI.

Type species: **here fixed** (under ICZN 1999a, Article 70.3.2) as *Buprestisundecimmaculata* Herbst, 1784, misidentified as *Buprestisnovemmaculata* Linnaeus, 1767 (sensu Fabricius, 1775) in the original designation by monotypy in Dejean (1833a: 79).

Family-group name: PTOSIMITES Kerremans, 1903. Stem: *Ptosim*-.

***Ptychoderes* Schönherr, 1823**: 1135. Current status: valid genus in ANTHRIBIDAE: ANTHRIBINAE: PTYCHODERINI.

Type species: *Macrocephalusnebulosus* G.-A. Olivier, 1800, by original designation.

Family-group name: PTYCHODERIDAE Jekel, 1855. Stem: *Ptychoder*-. Note: senior homonym of PTYCHODERIDAE Spengel, 1893 (type genus *Ptychodera* Eschscholtz, 1825) in Enteropneusta (acorn worms).

***Ptychodes* Audinet-Serville, 1835a**: 74. Current status: valid genus in CERAMBYCIDAE: LAMIINAE: LAMIINI.

Type species: *Ptychodespolitus* Audinet-Serville, 1835, by monotypy.

Family-group name: PTYCHODES J.L. LeConte, 1873. Stem: *Ptychod*-.

***Ptyopteryx* Reichardt & C. Costa, 1967**: 13. Current status: invalid senior synonym of *Iapir* Py-Daniel, Fonseca & Barbosa, 1993 in TORRIDINCOLIDAE: TORRIDINCOLINAE. Note: junior homonym of *Ptyopteryx* Kolenati, 1848 [Trichoptera].

Type species: *Ptyopteryxbritskii* Reichardt & C. Costa, 1967, by original designation.

Family-group name: PTYOPTERINAE M. Abdullah, 1974. Stem: *Ptyopteryg*-. Note: family-group name permanently invalid (ICZN 1999a, Article 39).

***Pulicomorpha* Mann, 1924**: 87. Current status: valid genus in STAPHYLINIDAE: ALEOCHARINAE: CREMATOXENINI.

Type species: *Pulicomorphacoecum* Mann, 1924, by original designation.

Family-group name: PULICOMORPHINI Mann, 1924. Stem: *Pulicomorph*-.

***Purpuricenus* Dejean, 1821**: 105. Current status: valid genus in CERAMBYCIDAE: CERAMBYCINAE: TRACHYDERINI: TRACHYDERINA.

Type species: *Cerambyxkaehleri* Linnaeus, 1758, by subsequent designation (Blanchard in Audouin et al. 1843: pl. 66).

Family-group name: PURPURICENITAE J. Thomson, 1861. Stem: *Purpuricen*-.

***Pycnocerus* Westwood, 1842c**: 67. Current status: valid genus in TENEBRIONIDAE: LAGRIINAE: PYCNOCERINI. Note: new replacement name for *Pachylocerus* Hope, 1841.

Type species: *Pachyloceruswestermanni* Hope, 1841, by monotypy (type fixation for *Pachylocerus* Hope).

Family-group name: PYCNOCÉRIDES Lacordaire, 1859. Stem: *Pycnocer*-.

***Pycnomerus* Erichson, 1842**: 214. Current status: valid genus in ZOPHERIDAE: ZOPHERINAE: PYCNOMERINI.

Type species: *Ipsterebrans* G.-A. Olivier, 1790, by subsequent designation (Chevrolat 1847b: 644).

Family-group name: PYCNOMERINI Erichson, 1845. Stem: *Pycnomer*-.

***Pygomolpus* Bechyné, 1949**: 533. Current status: valid genus in CHRYSOMELIDAE: EUMOLPINAE: PYGOMOLPINI.

Type species: *Pygomolpusopacus* Bechyné, 1949, by monotypy.

Family-group name: PYGOMOLPINI Bechyné, 1949. Stem: *Pygomolp*-.

***Pygostenus* Kraatz, 1858b**: 361. Current status: valid genus in STAPHYLINIDAE: ALEOCHARINAE: PYGOSTENINI.

Type species: *Pygostenusmicrocerus* Kraatz, 1858, by monotypy.

Family-group name: PYGOSTENINI Fauvel, 1899. Stem: *Pygosten*-.

***Pygoxyon* Reitter in Leder, 1881**: 508. Current status: valid genus in STAPHYLINIDAE: PSELAPHINAE: GONIACERITAE: PYGOXYINI.

Type species: *Pygoxyonscydmaeniforme* Reitter, 1881, by monotypy.

Family-group name: PYGOXYINI Reitter, 1909. Stem: *Pygoxy*-.

***Pyrestes* Pascoe, 1857**: 96. Current status: valid genus in CERAMBYCIDAE: CERAMBYCINAE: PYRESTINI.

Type species: *Pyresteshaematicus* Pascoe, 1857, by subsequent designation (J. Thomson 1864: 159).

Family-group name: PYRESTHIDES Lacordaire, 1868. Stem: *Pyrest*-.

***Pyrochroa* Geoffroy, 1762**: 338. Current status: valid genus in PYROCHROIDAE: PYROCHROINAE. Note: name placed on the Official List of Generic Names in Zoology (ICZN 1994a).

Type species: *Canthariscoccinea* Linnaeus, 1761, by subsequent designation (Westwood 1838c: 30; see ICZN 1994a). Note: name placed on the Official List of Specific Names in Zoology (ICZN 1994a).

Family-group name: PYROCHROIDES Latreille, 1806. Stem: *Pyrochro*-.

***Pyrodes* Audinet-Serville, 1832**: 186. Current status: valid genus in CERAMBYCIDAE: PRIONINAE: MALLASPINI.

Type species: *Prionusspeciosus* G.-A. Olivier, 1795 (= *Prionusnitidus* Fabricius, 1787), by subsequent designation (E. Desmarest 1860: 308).

Family-group name: PYRODINI Harold, 1879. Stem: *Pyrod*-.

***Pyrophorus* Billberg, 1820**: 20. Current status: valid genus in ELATERIDAE: AGRYPNINAE: PYROPHORINI: PYROPHORINA.

Type species: *Elaternoctilucus* Linnaeus, 1758, by subsequent designation (J. Meyer 1850: 120).

Family-group name: PYROPHORITES Candèze, 1863. Stem: *Pyrophor*-.

***Pyropus* Schönherr, 1836**: 641. Current status: valid genus in CURCULIONIDAE: CURCULIONINAE: PYROPINI.

Type species: *Pyropussapphirinus* Gyllenhal, 1836, by original designation.

Family-group name: PYROPIDES Lacordaire, 1865. Stem: *Pyrop*-.

***Pyrota* Dejean, 1834**: 224. Current status: valid genus in MELOIDAE: MELOINAE: PYROTINI.

Type species: *Lyttadispar* Germar, 1823, by subsequent designation (Aksentjev 1988: 578). Note: the generic assignment of the type species was given as *Tetraonyx*, which was changed in the corrigenda (Germar, 1823: 623) to *Lytta*.

Family-group name: PYROTINI MacSwain, 1956. Stem: *Pyrot*-.

***Pytheus* Newman, 1840c**: 14. Current status: junior synonym of *Brachytria* Newman, 1840 in CERAMBYCIDAE: CERAMBYCINAE: CERTALLINI.

Type species: *Pytheusjugosus* Newman, 1840, by monotypy.

Family-group name: PYTHEITAE J. Thomson, 1864. Stem: *Pythe*-.

***Pythiopus* C. Koch, 1953**: 245. Current status: valid genus in TENEBRIONIDAE: BLAPTINAE: DENDARINI: DENDARINA.

Type species: *Pythiopuscornutipectus* C. Koch, 1953, by original designation.

Family-group name: PYTHIOPINI C. Koch, 1953. Stem: *Pythiop*-.

***Pytho* Latreille, 1797**: 23. Current status: valid genus in PYTHIDAE.

Type species: *Cucujuscoeruleus* Fabricius, 1787 (= *Tenebriodepressus* Linnaeus, 1767), by subsequent designation (Latreille 1810: 429). Note: no species were originally included in this genus by Latreille (1797); the first ones were the three species listed by Fabricius (1801b: 95, 96).

Family-group name: PYTHITES Solier, 1834. Stem: *Pyth*-.

***Pyxidicerus* Motschulsky, 1863**: 422. Current status: valid genus in STAPHYLINIDAE: PSELAPHINAE: EUPLECTITAE: BYTHINOPLECTINI: PYXIDICERINA.

Type species: *Pyxidiceruscastaneus* Motschulsky, 1863, by monotypy.

Family-group name: PYXIDICERINI Raffray, 1904. Stem: *Pyxidicer*-.

***Quasimus* Gozis, 1886**: 22. Current status: valid genus in ELATERIDAE: NEGASTRIINAE: QUASIMUSINI: QUASIMUSINA.

Type species: *Elaterminutissimus* Germar, 1822, by monotypy.

Family-group name: QUASIMUSINI Schimmel & Tarnawski, 2009. Stem: *Quasimus*- (ICZN 1999a, Article 29.4).

***Quedius* Stephens, 1829b**: 22. Current status: valid genus in STAPHYLINIDAE: STAPHYLININAE: STAPHYLININI: QUEDIINA. Note: name placed on the Official List of Generic Names in Zoology (ICZN 1996c).

Type species: *Staphylinuslevicollis* Brullé, 1832, by plenary power (see ICZN 1996c). Note: name placed on the Official List of Specific Names in Zoology (ICZN 1996c).

Family-group name: QUEDIIFORMES Kraatz, 1857. Stem: *Quedi*-.

***Quelaestrygon* Smetana, 1999**: 241. Current status: valid genus in STAPHYLINIDAE: STAPHYLININAE: STAPHYLININI: QUELAESTRYGONINA.

Type species: *Quelaestrygonpuetzi* Smetana, 1999, by original designation.

Family-group name: QUELAESTRYGONINI Brunke in Brunke et al., 2021: 415. Stem: *Quelaestrygon*-.

***Radama* Raffray, 1883**: 230. Current status: valid genus in STAPHYLINIDAE: PSELAPHINAE: CLAVIGERITAE: CLAVIGERINI.

Type species: *Radamainflatus* Raffray, 1883, by subsequent designation (R. Lucas 1920: 564).

Family-group name: RADAMINI Jeannel, 1954. Stem: *Radam*-.

***Raffrayia* Reitter, 1882a**: 198, 211. Current status: valid genus in STAPHYLINIDAE: PSELAPHINAE: EUPLECTITAE: TRICHONYCHINI: TRICHONYCHINA.

Type species: *Trichonyxantennatus* Raffray, 1877, by original designation.

Family-group name: RAFFRAYINA Jeannel, 1949. Stem: *Raffrayi*-.

***Ranavala* Raffray, 1898a**: 206. Current status: valid genus in STAPHYLINIDAE: PSELAPHINAE: EUPLECTITAE: TRICHONYCHINI: TRICHONYCHINA.

Type species: *Autoplectusintegricollis* Raffray, 1887, by subsequent monotypy (Raffray 1898a: 224).

Family-group name: RANAVALINI Jeannel, 1954. Stem: *Ranaval*-.

***Ranolus* Blair, 1929**: 382. Current status: valid genus in DERMESTIDAE: ORPHILINAE.

Type species: *Attagenuscavernicola* Blair, 1929, by monotypy. Note: combined description of a new genus-group taxon and a single new species (ICZN 1999a, Article 12.2.6).

Family-group name: RANOLINI Háva in Zahradník and Háva, 2014: 306. Stem: *Ranol*-.

***Raphidopalpa* Chevrolat in Dejean, 1836a**: 378. Current status: junior synonym of *Aulacophora* Chevrolat, 1836 in CHRYSOMELIDAE: GALERUCINAE: LUPERINI. Note: First Reviser (ICZN 1999a, Article 24.2.2) for *Raphidopalpa* Chevrolat, 1836 versus *Aulacophora* Chevrolat, 1836 is Gestro (1889: 72).

Type species: *Criocerisabdominalis* Fabricius, 1781, by subsequent designation (Chevrolat 1845: 6).

Family-group name: RHAPHIDOPALPINI Weise, 1902. Stem: *Raphidopalp*-.

***Rathymus* Dejean, 1831**: 695, 783. Current status: valid genus in CARABIDAE: HARPALINAE: PTEROSTICHINI: PTEROSTICHINA.

Type species: *Rathymuscarbonarius* Dejean, 1831, by monotypy.

Family-group name: RHATHYMINAE H.W. Bates, 1891. Stem: *Rathym*-.

***Raymondionymus* Wollaston, 1873b**: 531. Current status: valid genus in CURCULIONIDAE: BRACHYCERINAE: RAYMONDIONYMINI. Note: new replacement name for *Raymondia* Aubé, 1861.

Type species: *Raymondiafossor* Aubé, 1861, by monotypy (type fixation for *Raymondia* Aubé).

Family-group name: RAYMONDIONYMINI Reitter, 1913. Stem: *Raymondionym*-.

***Reicheia* Saulcy, 1862**: 285. Current status: valid genus in CARABIDAE: SCARITINAE: CLIVININI: CLIVININA.

Type species: *Reicheialucifuga* Saulcy, 1862, by monotypy.

Family-group name: REICHEINA Jeannel, 1957. Stem: *Reichei*-.

***Reichenbachia* Leach, 1826**: 451. Current status: valid genus in STAPHYLINIDAE: PSELAPHINAE: GONIACERITAE: BRACHYGLUTINI: BRACHYGLUTINA.

Type species: *Bryaxisjuncorum* Leach, 1817, by subsequent designation (E. Desmarest 1852: 136).

Family-group name: REICHENBACHIINA Jakobson, 1910. Stem: *Reichenbachi*-.

***Rembus* W.S. MacLeay, 1825**: 16. Current status: invalid senior synonym of *Diplocheila* Brullé, 1834 in CARABIDAE: HARPALINAE: LICININI: DICAELINA. Note: junior homonym of *Rembus* Germar, 1823 [Coleoptera: CURCULIONIDAE].

Type species: *Carabuspolitus* Fabricius, 1792, by monotypy.

Family-group name: REMBIDAE Gistel, 1848. Stem: *Remb*-. Note: family-group name permanently invalid (ICZN 1999a, Article 39).

***Remipedella* Semenov-Tjan-Shansky, 1907**: 257. Current status: valid genus in TENEBRIONIDAE: BLAPTINAE: BLAPTINI: REMIPEDELLINA.

Type species: *Remipedelladeserti* Semenov-Tjan-Shansky, 1907, by monotypy.

Family-group name: REMIPEDELLINI Semenov-Tjan-Shansky, 1907. Stem: *Remipedell*-.

***Remphan* G.R. Waterhouse, 1835**: 67. Current status: valid genus in CERAMBYCIDAE: PRIONINAE: MACROTOMINI: REMPHANINA.

Type species: *Remphanhopei* G.R. Waterhouse, 1835, by monotypy.

Family-group name: REMPHANIDES Lacordaire, 1868. Stem: *Remphan*-.

***Renania* Lewis, 1887**: 60. Current status: valid genus in EROTYLIDAE: EROTYLINAE: TRITOMINI.

Type species: *Renaniaatrocyanea* Lewis, 1887, by monotypy.

Family-group name: RENANIINAE Chûjô, 1941. Stem: *Renani*-.

***Rentonium* Crowson, 1966**: 121. Current status: valid genus in RENTONIIDAE.

Type species: *Rentoniumdaldiniae* Crowson, 1966, by original designation.

Family-group name: RENTONIINAE Crowson, 1966. Stem: *Rentoni*-.

***Repsimus* W.S. MacLeay, 1819**: 144. Current status: valid genus in SCARABAEIDAE: RUTELINAE: ANOPLOGNATHINI: ANOPLOGNATHINA.

Type species: *Anoplognathusdytiscoides* W.S. MacLeay, 1819 (= *Rutelamanicata* Swartz, 1817), by subsequent designation (Crotch 1870b: 234).

Family-group name: RHEPSIMIDAE Streubel, 1846. Stem: *Repsim*-.

†***Retromalisus* Kazantsev, 2020**: 1074. Current status: valid genus in †BERENDTIMIRIDAE: RETROMALISINAE. Note: [geological time not mentioned] (Baltic amber).

Type species: †*Retromalisusdamzeni* Kazantsev, 2020, by original designation.

Family-group name: †RETROMALISINAE Fanti, Vitali & Pankowski, 2023: 135. Stem: *Retromalis*-.

***Reynoldsiella* Ray, 1930**: 164. Current status: valid genus in MORDELLIDAE: MORDELLINAE: REYNOLDSIELLINI.

Type species: *Reynoldsiellaparallela* Ray, 1930, by original designation.

Family-group name: REYNOLDSIELLINI Franciscolo, 1957. Stem: *Reynoldsiell*-.

***Rhadalus* J.L. LeConte, 1852d**: 212. Current status: valid genus in RHADALIDAE: RHADALINAE.

Type species: *Rhadalustestaceus* J.L. LeConte, 1852, by monotypy.

Family-group name: RHADALINI J.L. LeConte, 1861. Stem: *Rhadal*-.

***Rhadinocyba* Faust, 1889**: 78, 79. Current status: valid genus in BRENTIDAE: APIONINAE: RHADINOCYBITAE: RHADINOCYBINI.

Type species: *Rhadinocybanitidipennis* Faust, 1889, by monotypy.

Family-group name: RHADINOCYBINI Alonso-Zarazaga, 1992. Stem: *Rhadinocyb*-.

***Rhadinosomus* Schönherr, 1840b**: 473. Current status: valid genus in CURCULIONIDAE: CYCLOMINAE: ATERPINI: RHADINOSOMINA. Note: new replacement name for *Leptosomus* Schönherr, 1823.

Type species: *Curculioacuminatus* Fabricius, 1775, by original designation (type fixation for *Leptosomus* Schönherr).

Family-group name: RHADINOSOMIDES Lacordaire, 1863. Stem: *Rhadinosom*-.

***Rhaeboscelis* Chevrolat, 1838**: 103. Current status: valid genus in BUPRESTIDAE: AGRILINAE: AGRILINI: RHAEBOSCELIDINA. Note: the original spelling was *Raeboscelis* but the incorrect subsequent spelling *Rhaeboscelis* is in prevailing usage, attributed to the publication of the original spelling, and therefore deemed to be the correct original spelling (ICZN 1999a, Article 33.3.1).

Type species: *Rhaeboscelispurpureus* Chevrolat, 1838, by monotypy. Note: Chevrolat (1838: 104) added that “*carbonculus* de Gory parait devoir rentrer dans cette coupe [it appears that *carbonculus* of Gory should belong in this genus]” and we consider, from that statement, that *carbonculus* is doubtfully included in the genus and therefore not an originally included species (ICZN 1999a, Article 67.2.5).

Family-group name: RHAEBOSCELIDI Cobos, 1976. Stem: *Rhaeboscelid*-.

***Rhaebus* Fischer, 1823a**: pl. 47. Current status: valid genus in CHRYSOMELIDAE: BRUCHINAE: RHAEBINI.

Type species: *Rhaebusgebleri* Fischer, 1823, by monotypy.

Family-group name: RHAEBITES Blanchard, 1845. Stem: *Rhaeb*-.

***Rhaetulus* Westwood, 1871**: 353. Current status: valid genus in LUCANIDAE: LUCANINAE: DORCINI.

Type species: *Rhaetuluscrenatus* Westwood, 1871, by monotypy.

Family-group name: RHAETULINAE Miwa, 1931. Stem: *Rhaetul*-.

***Rhagiomorpha* Newman, 1840a**: 21. Current status: junior synonym of *Stenoderus* Dejean, 1821 in CERAMBYCIDAE: CERAMBYCINAE: RHAGIOMORPHINI.

Type species: *Stenoderusconcolor* W.S. MacLeay, 1826, by subsequent designation (J. Thomson 1864: 136).

Family-group name: RHAGIOMORPHIDAE Newman, 1841. Stem: *Rhagiomorph*-.

***Rhagium* Fabricius, 1775**: 182. Current status: valid genus in CERAMBYCIDAE: LEPTURINAE: RHAGIINI.

Type species: *Cerambyxinquisitor* Linnaeus, 1758, by subsequent designation (Westwood 1838c: 41).

Family-group name: RHAGIADAE W. Kirby, 1837. Stem: *Rhagi*-.

***Rhagocrepis* Eschscholtz, 1829c**: 5. Current status: junior synonym of *Leptotrachelus* Latreille, 1829 in CARABIDAE: HARPALINAE: CTENODACTYLINI.

Type species: *Rhagocrepisriedelii* Eschscholtz, 1829, by monotypy.

Family-group name: RHAGOCREPIDES Chaudoir, 1848. Stem: *Rhagocrepid*-.

***Rhagodera* Mannerheim, 1843**: 300. Current status: valid genus in ZOPHERIDAE: COLYDIINAE: RHAGODERINI.

Type species: *Rhagoderatuberculata* Mannerheim, 1843, by monotypy.

Family-group name: RHAGODERINI G.H. Horn, 1878. Stem: *Rhagoder*-.

***Rhagophthalmus* Motschulsky, 1853a**: 45. Current status: valid genus in RHAGOPHTHALMIDAE.

Type species: *Rhagophthalmusscutellatus* Motschulsky, 1853, by monotypy.

Family-group name: RHAGOPHTHALMIDAE E. Olivier, 1907. Stem: *Rhagophthalm*-.

***Rhamnusium* Latreille, 1829b**: 130. Current status: valid genus in CERAMBYCIDAE: LEPTURINAE: RHAMNUSIINI.

Type species: *Callidiumsalicis* Fabricius, 1787 (= *Cerambyxbicolor* Schrank, 1781), by monotypy.

Family-group name: RHAMNUSIINI Sama, 2009. Stem: *Rhamnusi*-.

***Rhamphophorus* Poinar & A.E. Brown, 2021**: 2. Current status: valid genus in POLYPHAGA incertae sedis.

Type species: *Rhamphophoruslegalovii* Poinar & A.E. Brown, 2021, by original designation.

Family-group name: RHAMPHOPHORINI Poinar & A.E. Brown, 2021: 2. Stem: *Rhamphophor*-. Note: combined description of a new family-group taxon and a single new nominal genus (ICZN 1999a, Article 13.5).

***Rhamphus* Clairville, 1798**: 57, 104. Current status: valid genus in CURCULIONIDAE: CURCULIONINAE: RHAMPHINI: RHAMPHINA. Note: two original spellings were used, including *Ramphus* (p. 104); Lepeletier and Audinet-Serville (1825: 284) acted as First Revisers (ICZN 1999a, Article 24.2.3) and selected *Rhamphus*.

Type species: *Rhamphusflavicornis* Clairville, 1798 (= *Curculiopulicarius* Herbst, 1795), by monotypy.

Family-group name: RAMPHORIA Rafinesque, 1815. Stem: *Rhamph*-.

***Rhanis* J.L. LeConte, 1853d**: 357, 360. Current status: invalid senior synonym of *Rhanidea* Strohecker, 1953 in ENDOMYCHIDAE: LEIESTINAE. Note: junior homonym of *Rhanis* C.L. Koch, 1846 [Arachnida].

Type species: *Lycoperdinaunicolor* Ziegler, 1845, by monotypy.

Family-group name: RHANES J.L. LeConte & G.H. Horn, 1883. Stem: *Rhanid*-. Note: family- group name permanently invalid (ICZN 1999a, Article 39).

***Rhaphipodus* Audinet-Serville, 1832**: 168. Current status: valid genus in CERAMBYCIDAE: PRIONINAE: REMPHANINI.

Type species: *Rhaphipodussuturalis* Audinet-Serville, 1832, by monotypy.

Family-group name: RHAPHIPODI Lameere, 1912. Stem: *Rhaphipod*-.

***Rhexius* J.L. LeConte, 1849**: 102. Current status: valid genus in STAPHYLINIDAE: PSELAPHINAE: EUPLECTITAE: TROGASTRINI: RHEXIINA.

Type species: *Rhexiusinsculptus* J.L. LeConte, 1849, by monotypy.

Family-group name: RHEXINI Park, 1951. Stem: *Rhexi*-.

***Rhigopsis* J.L. LeConte, 1874a**: 459. Current status: valid genus in CURCULIONIDAE: ENTIMINAE: ENTIMITAE: BYRSOPAGINI: STRANGALIODINA.

Type species: *Rhigopsiseffracta* J.L. LeConte, 1874, by monotypy.

Family-group name: RHIGOPSINI J.L. LeConte, 1874. Stem: *Rhigopse*-.

***Rhigus* Germar, 1823**: 438. Current status: valid genus in CURCULIONIDAE: ENTIMINAE: ENTIMITAE: ENTIMINI: RHIGIINA.

Type species: *Curculiotribuloides* Pallas, 1781, by original designation.

Family-group name: RHIGIINA Legalov, 2020b: 339. Stem: *Rhigi*- (ICZN 1999a, Article 29.4).

***Rhina* Latreille, 1802b**: 198. Current status: invalid senior synonym of *Rhinostomus* Rafinesque, 1815 in CURCULIONIDAE: DRYOPHTHORINAE: RHINOSTOMINI. Note: junior homonym of *Rhina* J.G. Schneider, 1801 [Pisces]; name placed on the Official Index of Rejected and Invalid Generic Names in Zoology (ICZN 1955a).

Type species: *Curculiobarbicornis* Fabricius, 1775, by monotypy. Note: name placed on the Official List of Specific Names in Zoology (ICZN 1955a).

Family-group name: RHINIDAE J.L. LeConte, 1874. Stem: *Rhin*-. Note: family-group name permanently invalid (ICZN 1999a, Article 39).

***Rhinaria* W. Kirby, 1819**: 430. Current status: valid genus in CURCULIONIDAE: CYCLOMINAE: ATERPINI: ATERPINA.

Type species: *Rhinariacristata* W. Kirby, 1819, by original designation.

Family-group name: RHINARIDES Jekel, 1865. Stem: *Rhinari*-.

***Rhinastus* Schönherr, 1825**: 584. Current status: valid genus in CURCULIONIDAE: MOLYTINAE: CHOLINI: RHINASTINA.

Type species: *Cholussternicornis* Germar, 1823, by monotypy.

Family-group name: RHINASTINI Vaurie, 1973. Stem: *Rhinast*-.

***Rhinocartus* Voss, 1922a**: 17, 18. Current status: valid genus in ATTELABIDAE: RHYNCHITINAE: RHINOCARTITAE: RHINOCARTINI.

Type species: *Rhinocartustessmanni* Voss, 1922, by monotypy.

Family-group name: RHINOCARTINI Voss, 1931. Stem: *Rhinocart*-.

***Rhinocyllus* Germar, 1817b**: 341. Current status: valid genus in CURCULIONIDAE: LIXINAE: RHINOCYLLINI.

Type species: *Curculiothaumaturgus* Rossi, 1794 (= *Curculioconicus* Frölich, 1792), by monotypy.

Family-group name: RHINOCYLLIDES Lacordaire, 1863. Stem: *Rhinocyll*-.

***Rhinomacer* Fabricius, 1781**: 199. Current status: name in MYCTERIDAE: MYCTERINAE. Note: name placed on the Official Index of Rejected and Invalid Generic Names in Zoology (see ICZN 2005c).

Type species: *Rhinomacercurculioides* Fabricius, 1781, by monotypy.

Family-group name: RHINOMACERIDAE Fleming, 1821. Stem: *Rhinomacr*-. Note: family-group name permanently invalid (ICZN 1999a, Article 39).

***Rhinoncus* Schönherr, 1825**: 586. Current status: valid genus in CURCULIONIDAE: CEUTORHYNCHINAE: PHYTOBIINI. Note: name placed on the Official List of Generic Names in Zoology (ICZN 1989b).

Type species: *Curculiopericarpius* Linnaeus, 1758, by plenary power (see ICZN 1989b). Note: name placed on the Official List of Specific Names in Zoology (ICZN 1989b).

Family-group name: RHINONCIDES C.G. Thomson, 1865. Stem: *Rhinonc*-.

***Rhinophthalmus* J. Thomson, 1861**: 152. Current status: valid genus in CERAMBYCIDAE: CERAMBYCINAE: URACANTHINI. Note: new replacement name for *Stephanops* Shuckard, 1838.

Type species: *Stephanopsnasutus* Newman, 1838, by monotypy (type fixation for *Stephanops* Shuckard).

Family-group name: RHINOPHTHALMITAE J. Thomson, 1861. Stem: *Rhinophthalm*-.

***Rhinorhipus* Lawrence, 1988**: 4. Current status: valid genus in RHINORHIPIDAE.

Type species: *Rhinorhipustamborinensis* Lawrence, 1988, by original designation.

Family-group name: RHINORHIPIDAE Lawrence, 1988. Stem: *Rhinorhip*-.

***Rhinorhynchidius* Voss, 1922a**: 107. Current status: valid genus in BRENTIDAE: RHINORHYNCHIDIITAE.

Type species: *Rhinorhynchidiuscylindrirostris* Voss, 1922 (= *Apionmeridionale* Lea, 1926), by monotypy.

Family-group name: RHINORHYNCHIDIINAE Zimmerman, 1994. Stem: *Rhinorhynchidi*-.

***Rhinorhynchus* Sharp, 1882a**: 88. Current status: valid genus in NEMONYCHIDAE: RHINORHYNCHINAE: RHINORHYNCHINI.

Type species: *Rhinorhynchuszealandicus* Sharp, 1882 (= *Rhinomacerrufulus* Broun, 1880), by monotypy.

Family-group name: RHINORHYNCHINI Voss, 1922. Stem: *Rhinorhynch*-.

***Rhinoscepsis* J.L. LeConte, 1878a**: 382. Current status: valid genus in STAPHYLINIDAE: PSELAPHINAE: EUPLECTITAE: METOPIASINI: RHINOSCEPSINA.

Type species: *Rhinoscepsisbistriatus* J.L. LeConte, 1878, by monotypy.

Family-group name: RHINOSCEPSII Bowman, 1934. Stem: *Rhinosceps*-.

***Rhinosimus* Latreille, 1802b**: 192. Current status: junior synonym of *Salpingus* Illiger, 1802 in SALPINGIDAE: SALPINGINAE.

Type species: *Curculioplanirostris* Fabricius, 1787, by monotypy.

Family-group name: RHINOSIMIDAE Hope, 1841. Stem: *Rhinosim*-.

***Rhinostomus* Rafinesque, 1815**: 115. Current status: valid genus in CURCULIONIDAE: DRYOPHTHORINAE: RHINOSTOMINI. Note: name placed on the Official List of Generic Names in Zoology (ICZN 1955a).

Type species: *Curculiobarbirostris* Fabricius, 1775, by plenary power (see ICZN 1955a). Note: name placed on the Official List of Specific Names in Zoology (ICZN 1955a).

Family-group name: RHINOSTOMINI Kuschel, 1995. Stem: *Rhinostom*-.

***Rhinotragus* Germar, 1823**: 513. Current status: valid genus in CERAMBYCIDAE: CERAMBYCINAE: RHINOTRAGINI.

Type species: *Rhinotragusdorsiger* Germar, 1823, by monotypy.

Family-group name: RHINOTRAGITAE J. Thomson, 1861. Stem: *Rhinotrag*-.

***Rhipicera* Latreille, 1816**: 235. Current status: valid genus in RHIPICERIDAE: RHIPICERINAE.

Type species: *Hispamystacina* Fabricius, 1775, by monotypy.

Family-group name: RHIPICERIDES Latreille, 1834. Stem: *Rhipicer*-.

***Rhipidandrus* J.L. LeConte, 1862**: 236. Current status: valid genus in TENEBRIONIDAE: TENEBRIONINAE: BOLITOPHAGINI.

Type species: *Xyletinusflabellicornis* Sturm, 1826 (= *Melolonthaparadoxa* Palisot de Beauvois, 1818), by monotypy.

Family-group name: RHIPIDANDRI J.L. LeConte, 1862. Stem: *Rhipidandr*-.

***Rhipidocerus* Westwood, 1842d**: 70. Current status: valid genus in CERAMBYCIDAE: PRIONINAE: RHIPIDOCERINI.

Type species: *Rhipidocerusaustralasiae* Westwood, 1842, by monotypy.

Family-group name: RHIPIDOCERINI Jin, De Keyzer, Ashman, Zwick & Ślipiński, 2023: 507. Stem: *Rhipidocer*-.

***Rhizophagus* Herbst, 1793**: 18. Current status: valid genus in MONOTOMIDAE: RHIZOPHAGINAE. Note: the original spelling *Ryzophagus* was changed to *Rhizophagus* and *Rhizophagus* Herbst, 1793 placed on the Official List of Generic Names in Zoology (ICZN 1995c).

Type species: *Lyctusbipustulatus* Fabricius, 1792, by subsequent designation (Westwood 1838c: 13; see ICZN 1995c). Note: name placed on the Official List of Specific Names in Zoology (ICZN 1995c).

Family-group name: RHIZOPHAGI L. Redtenbacher, 1845. Stem: *Rhizophag*-.

†***Rhizophtoma* Kirejtshuk & Azar in Kirejtshuk et al., 2009a**: 123. Current status: valid genus in MONOTOMIDAE: MONOTOMINAE: †RHIZOPHTOMINI. Note: Lower Cretaceous (Lebanon; Spain).

Type species: †*Rhizophtomaelateroides* Kirejtshuk & Azar, 2009, by original designation.

Family-group name: †RHIZOPHTOMINAE Kirejtshuk & Azar, 2009. Stem: *Rhizophtom*-.

***Rhizotrogus* Berthold, 1827**: 362. Current status: valid genus in SCARABAEIDAE: MELOLONTHINAE: RHIZOTROGINI. Note: this name is sometimes credited to Latreille (1825: 371) (e.g., Branco 2007: 20; J. Bezděk 2016: 275) but it was proposed in that work under the French vernacular name Rhizotrogue, not under a scientific name.

Type species: *Melolonthaaestiva* G.-A. Olivier, 1789, by monotypy.

Family-group name: RHIZOTROGIDAE Burmeister, 1855. Stem: *Rhizotrog*-.

***Rhodocanthopus* Kaup, 1871**: 90. Current status: valid genus in PASSALIDAE: PASSALINAE: PASSALINI.

Type species: *Passaluscaelatus* Erichson, by subsequent designation (Hincks and Dibb 1935: 39)

Family-group name: RHODACANTHOPINAE Kuwert, 1896. Stem: *Rhodocanthopod*-.

***Rhodopina* Gressitt, 1951**: 439. Current status: valid genus in CERAMBYCIDAE: LAMIINAE: LAMIINI. Note: new replacement name for *Rhodopis* J. Thomson, 1857.

Type species: *Rhodopispubera* J. Thomson, 1857, by monotypy (type fixation for *Rhodopis* J. Thomson).

Family-group name: RHODOPININI Gressitt, 1951. Stem: *Rhodopin*-.

***Rhodopis* J. Thomson, 1857**: 174. Current status: invalid senior synonym of *Rhodopina* Gressit, 1951 in CERAMBYCIDAE: LAMIINAE: LAMIINI. Note: junior homonym of *Rhodopis* L. Reichenbach, 1854 [Aves].

Type species: *Rhodopispubera* J. Thomson, 1857, by monotypy.

Family-group name: RHODOPIDES Lacordaire, 1872. Stem: *Rhodopid*-. Note: family-group name permanently invalid (ICZN 1999a, Article 39).

†***Rhombocoleus* Rohdendorf, 1961**: 432. Current status: valid genus in †RHOMBOCOLEIDAE. Note: Permian (Siberia, Russia).

Type species: †*Rhombocoleusandreae* Rohdendorf, 1961, by original designation.

Family-group name: †RHOMBOCOLEIDAE Rohdendorf, 1961. Stem: *Rhombocole*-.

***Rhomborhina* Hope, 1837**: 120. Current status: valid genus in SCARABAEIDAE: CETONIINAE: GOLIATHINI: RHOMBORHININA.

Type species: *Goliathusheros* Gory & Percheron, 1833, by original designation.

Family-group name: RHOMBORRHINAE Westwood, 1842. Stem: *Rhomborh*-. Note: this family- group name, issued 1 January 1842, has precedence over CORYPHOCERINA Burmeister, 1842, issued by 28 December 1842.

***Rhopalocerus* W. Redtenbacher, 1842**: 21. Current status: valid genus in ZOPHERIDAE: COLYDIINAE: RHOPALOCERINI. Note: name placed on the Official List of Generic Names in Zoology (ICZN 1986b).

Type species: *Rhopalocerussetosus* W. Redtenbacher, 1842 (= *Monotomarondanii* A. Villa & G.B. Villa, 1833), by monotypy (see ICZN 1986b). Note: *Monotomarondanii* A. Villa & G.B. Villa, 1833 was placed on the Official List of Specific Names in Zoology (ICZN 1986b).

Family-group name: RHOPALOCERINI Reitter, 1911. Stem: *Rhopalocer*-.

***Rhopalogastrum* Bernhauer, 1912**: 68. Current status: valid genus in STAPHYLINIDAE: ALEOCHARINAE: AUTALIINI.

Type species: *Rhopalogastrumclaviventre* Bernhauer, 1912, by monotypy.

Family-group name: RHOPALOGASTRA Fenyes, 1919. Stem: *Rhopalogastr*-.

***Rhopalomelus* Boheman, 1848**: 165. Current status: valid genus in CARABIDAE: HARPALINAE: CHLAENIINI: CHLAENIINA.

Type species: *Rhopalomelusangusticollis* Boheman, 1848, by monotypy.

Family-group name: RHOPALOMELINI Alluaud, 1930. Stem: *Rhopalomel*-.

***Rhopalophora* Audinet-Serville, 1834b**: 100. Current status: valid genus in CERAMBYCIDAE: CERAMBYCINAE: RHOPALOPHORINI.

Type species: *Rhopalophorasanguinicollis* Audinet-Serville, 1834 (= *Callichromacollare* Germar, 1823), by monotypy.

Family-group name: RHOPALOPHORITES Blanchard, 1845. Stem: *Rhopalophor*-.

***Rhopalosilpha* Arrow, 1929b**: 97. Current status: valid genus in DERMESTIDAE: DERMESTINAE: MARIOUTINI.

Type species: *Rhopalosilphawasmanni* Arrow, 1929, by monotypy.

Family-group name: RHOPALOSILPHINAE Arrow, 1929. Stem: *Rhopalosilph*-.

***Rhynchaenus* Clairville, 1798**: 71, 142. Current status: valid genus in CURCULIONIDAE: CURCULIONINAE: RHAMPHINI: RHAMPHINA. Note: two original spellings were used, including *Rynchaenus* (pp. 56, 57, 71–73); Buchanan (1939: 81) acted as First Reviser (ICZN 1999a, Article 24.2.3) and selected the spelling *Rhynchaenus*.

Type species: *Rhynchaenusxylostei* Clairville, 1798 (= *Curculiolonicerae* Herbst, 1795), by subsequent designation (Crotch 1870a: 50).

Family-group name: RHYNCHENIDAE Blanchard, 1853. Stem: *Rhynchaen*-.

***Rhynchitallus* Voss, 1960**: 415. Current status: valid genus in ATTELABIDAE: RHYNCHITINAE: RHYNCHITITAE: RHYNCHITINI: RHYNCHITINA.

Type species: *Rhynchitalluscyclops* Voss, 1960, by original designation.

Family-group name: RHYNCHITALLINA Legalov, 2003. Stem: *Rhynchitall*-.

***Rhynchitapion* Wanat, 2021**: 59. Current status: valid genus in BRENTIDAE: APIONINAE incertae sedis.

Type species: *Rhynchitapionvariiforme* Wanat, 2021, by original designation.

Family-group name: RHYNCHITAPIINI Wanat, 2021: 56. Stem: *Rhynchitapi*-.

***Rhynchites* D.H. Schneider, 1791**: 83. Current status: valid genus in ATTELABIDAE: RHYNCHITINAE: RHYNCHITITAE: RHYNCHITINI: RHYNCHITINA.

Type species: *Curculiobacchus* Linnaeus, 1758, by subsequent designation (Latreille 1810: 430).

Family-group name: RHYNCHITISIDAE Gistel, 1848. Stem: *Rhynchit*-.

***Rhynchitomacer* Voss, 1937b**: 201. Current status: valid genus in NEMONYCHIDAE: RHINORHYNCHINAE: RHYNCHITOMACRINI.

Type species: *Rhynchitomacerflavus* Voss, 1937, by original designation.

Family-group name: RHYNCHITOMACERINI May, 1993. Stem: *Rhynchitomacr*-.

***Rhynchitoplesius* Voss, 1952**: 177. Current status: valid genus in NEMONYCHIDAE: RHINORHYNCHINAE: MECOMACERINI: RHYNCHITOPLESIINA.

Type species: *Rhynchitomacereximius* Voss, 1937, by monotypy.

Family-group name: RHYNCHITOPLESIINA Legalov, 2011a: 163. Stem: *Rhynchitoplesi*-

***Rhynchophorus* Herbst, 1795**: 3, pl. lx. Current status: valid genus in CURCULIONIDAE: DRYOPHTHORINAE: RHYNCHOPHORINI. Note: two original spellings were used, including *Rynchophorus* (p. 3); Alonso-Zarazaga and Lyal (1999: 64) acted as First Reviser (ICZN 1999a, Article 24.2.3) and selected the spelling *Rhynchophorus*.

Type species: *Curculiopalmarum* Linnaeus, 1758, by subsequent designation (Schönherr 1826: 23).

Family-group name: RHYNCHOPHORIDES Schönherr, 1833. Stem: *Rhynchophor*-.

***Rhyncogonus* Sharp in Blackburn and Sharp, 1885**: 176. Current status: valid genus in CURCULIONIDAE: ENTIMINAE: OTIORHYNCHITAE: RHYNCOGONINI.

Type species: *Rhyncogonusblackburni* Sharp, 1885, by subsequent designation (Zimmerman 1964: 308).

Family-group name: RHYNCOGONIDES Sharp, 1919. Stem: *Rhyncogon*-.

***Rhyncolus* Germar, 1817b**: 340. Current status: valid genus in CURCULIONIDAE: COSSONINAE: RHYNCOLINI: RHYNCOLINA. Note: name placed on the Official List of Generic Names in Zoology (ICZN 1991b).

Type species: *Curculioater* Linnaeus, 1758, by plenary power (see ICZN 1991b). Note: name placed on the Official List of Specific Names in Zoology (ICZN 1991b).

Family-group name: RHYNCHALIDAE Gistel, 1848. Stem: *Rhyncol*-.

***Rhyparosomus* Schönherr, 1842a**: 200. Current status: valid genus in CURCULIONIDAE: CYCLOMINAE: RHYTHIRRININI.

Type species: *Rhyparosomushorridus* Boheman, 1842, by original designation.

Family-group name: RHYPAROSOMIDES Lacordaire, 1863. Stem: *Rhyparosom*-.

***Rhyparus* Westwood, 1845c**: 93. Current status: valid genus in SCARABAEIDAE: APHODIINAE: RHYPARINI. Note: the original spelling was *Ryparus* but the unjustified emendation *Rhyparus*, proposed by Agassiz (1846: 328), is in prevailing usage, attributed to the publication of the original spelling, and therefore deemed to be the correct original spelling (ICZN 1999a, Article 33.2.3.1); accepting Agassiz’s emendation as a justified emendation avoids homonymy with *Ryparus* Spinola, 1845 [Coleoptera: CLERIDAE] (see A.B.T. Smith 2006: 159).

Type species: *Rhyparusdesjardinsii* Westwood, 1845, by monotypy (type fixation for *Ryparus* Westwood).

Family-group name: RHYPARINA A. Schmidt, 1910. Stem: *Rhypar*-.

***Rhysodes* Germar, 1822a**: no 1. Current status: valid genus in CARABIDAE: RHYSODINAE: RHYSODINI.

Type species: *Rhysodeseuropaeus* Germar, 1822 (= *Cucujussulcatus* Fabricius, 1787), by monotypy.

Family-group name: RHYSODITES Laporte, 1840. Stem: *Rhysod*-.

***Rhysopaussus* Wasmann, 1896**: 616. Current status: valid genus in TENEBRIONIDAE: TENEBRIONINAE: RHYSOPAUSSINI.

Type species: *Rhysopaussusdohertyi* Wasmann, 1896, by monotypy.

Family-group name: RHYSOPAUSSIDAE Wasmann, 1896. Stem: *Rhysopauss*-.

***Rhyssemus* Mulsant, 1842a**: 305, 314. Current status: valid genus in SCARABAEIDAE: APHODIINAE: PSAMMODIINI: RHYSSEMINA.

Type species: **here fixed** (under ICZN 1999a, Article 70.3.2) as *Ptinusgermanus* Linnaeus, 1767, misidentified as *Scarabaeusasper* Fabricius, 1775 (sensu Mulsant, 1842), in the subsequent designation of C.G. Thomson (1859: 81). Note: see Branco (2007: 20) for additional information regarding the misidentified type species.

Family-group name: RHYSSEMINA Pittino & Mariani, 1986. Stem: *Rhyssem*-.

***Rhythirrinus* Schönherr, 1823**: 1142. Current status: valid genus in CURCULIONIDAE: CYCLOMINAE: RHYTHIRRININI.

Type species: *Curculioinaequalis* Fabricius, 1781, by original designation.

Family-group name: RHYTIRHINIDES Lacordaire, 1863. Stem: *Rhythirrin*-.

***Rhyticephalus* Chevrolat, 1839**: 174. Current status: junior synonym of *Ischyromerus* Labram & Imhoff, 1838 in BRENTIDAE: BRENTINAE: TRACHELIZINI: RHYTICEPHALINA.

Type species: *Rhyticephalusbrevicornis* Chevrolat, 1839 (= *Brenthusmadagascariensis* Labram & Imhoff, 1838), by subsequent designation (R. Lucas 1920: 574; typification for *Rhytidocephalus*, an unjustified emendation by Agassiz (1846: 327) of *Rhyticephalus* Chevrolat).

Family-group name: RHYTICEPHALINI Kleine, 1922. Stem: *Rhyticephal*-.

***Rhytidophloeus* Schönherr, 1842a**: 290. Current status: valid genus in CURCULIONIDAE: MOLYTINAE: LITHININI: RHYTIDOPHLOEINA.

Type species: *Curculioalbipes* G.-A. Olivier, 1791, by original designation.

Family-group name: RHYTIDOPHLOEINI Voss, 1963. Stem: *Rhytidophloe*-.

***Rhyzobius* Stephens, 1829b**: 19. Current status: valid genus in COCCINELLIDAE: COCCINELLINAE: COCCIDULINI.

Type species: *Nitidulalitura* Fabricius, 1787, by monotypy.

Family-group name: RHIZOBIINA C.G. Thomson, 1866. Stem: *Rhyzobi*-.

***Riedelinius* Legalov, 2003**: 362. Current status: valid genus in ATTELABIDAE: ATTELABINAE: EUOPINI.

Type species: *Euopsarmatus* Riedel, 1999, by original designation.

Family-group name: RIEDELININA Legalov, 2007. Stem: *Riedelin*- (ICZN 1999a, Article 29.4).

***Ripidius* Thunberg, 1806**: 4. Current status: valid genus in RIPIPHORIDAE: RIPIDIINAE: RIPIDIINI.

Type species: *Ripidiuspectinicornis* Thunberg, 1806, by monotypy.

Family-group name: RHIPIDIINI Gerstaecker, 1855. Stem: *Ripidi*-.

***Ripiphorus* Bosc, 1791**: 327. Current status: valid genus in RIPIPHORIDAE: RIPIPHORINAE: RIPIPHORINI. Note: name placed on the Official List of Generic Names in Zoology (ICZN 2020b).

Type species: *Ripiphorussubdipterus* Fabricius, 1792, by plenary power (ICZN 2020b). Note: no species were originally included in this genus by Bosc (1791); the first ones are the twelve species listed by Fabricius (1792b: 109–112) (see Bousquet and Bouchard 2018: 36–37); name placed on the Official List of Specific Names in Zoology (ICZN 2020b).

Family-group name: RHIPIPHORITES Laporte, 1840. Stem: *Ripiphor*-.

***Rondoniella* Kaszab, 1970**: 112. Current status: valid genus in TENEBRIONIDAE: PIMELIINAE: CNEMEPLATIINI: RONDONIELLINA.

Type species: *Rondoniellacostata* Kaszab, 1970, by original designation.

Family-group name: RONDONIELLINA Ferrer & Moragues, 2000. Stem: *Rondoniell*-.

***Ropalodontus* Mellié, 1847**: 109. Current status: valid genus in CIIDAE: CIINAE: OROPHIINI.

Type species: *Cisperforatus* Gyllenhal, 1813, by monotypy.

Family-group name: RHOPALODONTINI Everts, 1898. Stem: *Ropalodont*-.

***Rosalia* Audinet-Serville, 1834a**: 561. Current status: valid genus in CERAMBYCIDAE: CERAMBYCINAE: ROSALIINI.

Type species: *Cerambyxalpinus* Linnaeus, 1758, by monotypy.

Family-group name: ROSALIITES Fairmaire, 1864. Stem: *Rosali*-.

***Rosiroia* Bechyné, 1950**: 148. Current status: valid genus in CHRYSOMELIDAE: EUMOLPINAE: ROSIROIINI.

Type species: *Rosiroiaanisotomoides* Bechyné, 1950, by monotypy.

Family-group name: ROSIROIINI Bechyné, 1950. Stem: *Rosiroi*-.

***Rostricephalus* Fleutiaux, 1918b**: 252. Current status: valid genus in ELATERIDAE: LISSOMINAE: PROTELATERINI.

Type species: *Rostricephalusvitalisi* Fleutiaux, 1918, by monotypy.

Family-group name: ROSTRICEPHALINAE Fleutiaux, 1947. Stem: *Rostricephal*-.

***Rugilus* Leach in Samouelle, 1819**: 173. Current status: valid genus in STAPHYLINIDAE: PAEDERINAE: LATHROBIINI: STILICINA.

Type species: *Staphylinusorbiculatus* Paykull, 1789, by original designation.

Family-group name: RUGILINA Hatch, 1957. Stem: *Rugil*-.

***Rupilia* Clark, 1864**: 260. Current status: valid genus in CHRYSOMELIDAE: GALERUCINAE: GALERUCINI.

Type species: *Rupiliaruficollis* Clark, 1864, by original designation.

Family-group name: RUPILIITES Chapuis, 1875. Stem: *Rupili*-.

***Rutela* Latreille, 1802b**: 151. Current status: valid genus in SCARABAEIDAE: RUTELINAE: RUTELINI: RUTELINA.

Type species: *Scarabaeuslineola* Linnaeus, 1758, by subsequent designation (MacLeay 1819: 72).

Family-group name: RUTELIDAE W.S. MacLeay, 1819. Stem: *Rutel*-.

***Rygmodus* A. White, 1846**: 11. Current status: valid genus in HYDROPHILIDAE: CYLOMINAE.

Type species: *Rygmodusmodestus* A. White, 1846, by subsequent designation (Knisch 1924: 107).

Family-group name: RYGMODINI Orchymont, 1916. Stem: *Rygmod*-.

***Rypobius* J.L. LeConte, 1852e**: 141, 142. Current status: valid genus in CORYLOPHIDAE: CORYLOPHINAE: RYPOBIINI.

Type species: *Rypobiusmarinus* J.L. LeConte, 1852, by monotypy.

Family-group name: RHYPOBIINI Paulian, 1950. Stem: *Rypobi*-.

***Ryssonotus* W.S. MacLeay, 1819**: 96, 98. Current status: valid genus in LUCANIDAE: LUCANINAE: RHYSSONOTINI. Note: name also referred to as *Rhyssonotus*, an unjustified emendation by Agassiz (1846: 329), in recent literature.

Type species: *Lucanusnebulosus* W. Kirby, 1819, by monotypy. Note: as noted by Reid and Beatson (2016: 3), *Lucanusfoveolatus* Thunberg, 1806, often listed as a senior synonym of *Lucanusnebulosus* W. Kirby, 1819, is a junior synonym of the North American *Lucanuscapreolus* (Linnaeus, 1763).

Family-group name: RHYSSONOTINI Benesh, 1960. Stem: *Ryssonot*-.

***Sachalinobia* Jakobson, 1899**: 39. Current status: valid genus in CERAMBYCIDAE: LEPTURINAE: SACHALINOBIINI.

Type species: *Sachalinobiaretata* Jakobson, 1899 (= *Brachytakoltzei* Heyden, 1887), by original designation.

Family-group name: SACHALINOBIINI Danilevsky, 2010. Stem: *Sachalinobi*-.

***Sacium* J.L. LeConte, 1852e**: 144. Current status: junior synonym of *Clypastraea* Haldeman, 1842 in CORYLOPHIDAE: CORYLOPHINAE: PARMULINI. Note: new replacement name for *Clypeaster* Dejean, 1821.

Type species: *Cossyphuspusillus* Gyllenhal, 1810, by monotypy (type fixation for *Clypeaster* Dejean).

Family-group name: SACIINA A. Matthews, 1888. Stem: *Saci*-.

***Sagra* Fabricius, 1792b**: 51. Current status: valid genus in CHRYSOMELIDAE: SAGRINAE: SAGRINI.

Type species: *Tenebriofemoratus* Drury, 1773, by subsequent designation (Latreille 1810: 431).

Family-group name: SAGRIDA Leach, 1815. Stem: *Sagr*-.

***Sahlbergius* Bernhauer, 1927**: 378. Current status: valid genus in STAPHYLINIDAE: ALEOCHARINAE: SAHLBERGIINI.

Type species: *Sahlbergiusmirabilis* Bernhauer, 1927, by monotypy.

Family-group name: SAHLBERGIINI Kistner, 1993. Stem: *Sahlbergi*-.

***Salax* Guérin-Méneville, 1834b**: 11. Current status: valid genus in TENEBRIONIDAE: PIMELIINAE: TRILOBOCARINI.

Type species: *Salaxlacordairii* Guérin-Méneville, 1834, by monotypy.

Family-group name: SALAXINI Casey, 1907. Stem: *Salac*-.

***Salcedia* Fairmaire, 1899**: 512. Current status: valid genus in CARABIDAE: SCARITINAE: SALCEDIINI.

Type species: *Salcediaperrieri* Fairmaire, 1899, by monotypy.

Family-group name: SALCEDIINI Alluaud, 1930. Stem: *Salcedi*-.

***Salpingus* Illiger, 1802**: 301. Current status: valid genus in SALPINGIDAE: SALPINGINAE.

Type species: *Curculioroboris* Fabricius, 1787 (= *Curculioruficollis* Linnaeus, 1761), by subsequent designation (Crotch 1870b: 215).

Family-group name: SALPINGIDES Leach, 1815. Stem: *Salping*-.

***Sandalus* Knoch, 1801**: 131. Current status: valid genus in RHIPICERIDAE: SANDALINAE.

Type species: *Sandalusniger* Knoch, 1801, by subsequent designation (Stephens 1840: 305). Note: the type species usually listed is *Sandaluspetrophya* Knoch, 1801 (e.g., Hájek 2016: 431) by subsequent designation of Guérin-Méneville (1843c [No 2]: 2) but Stephens’ designation is earlier; both species are currently included in *Sandalus* Knoch, 1801.

Family-group name: SANDALIDAE Jakobson, 1913. Stem: *Sandal*-.

***Sapriniana* Strand, 1917**: 85. Current status: valid genus in SCARABAEIDAE: AEGIALIINAE: SAPRINIANINI. Note: new replacement name for *Saprus* Blackburn, 1904.

Type species: *Saprusgriffithi* Blackburn, 1904, by monotypy (type fixation for *Saprus* Blackburn).

Family-group name: SAPRINIANINI Nikolajev, 2011: 328. Stem: *Saprinian*-.

***Saperda* Fabricius, 1775**: 184. Current status: valid genus in CERAMBYCIDAE: LAMIINAE: SAPERDINI. Note: name placed on the Official List of Generic Names in Zoology (ICZN 1980).

Type species: *Cerambyxcarcharias* Linnaeus, 1758, by subsequent designation (Guérin- Méneville 1829b: 151). Note: *Cerambyxscalaris* Linnaeus, 1758 is listed as the type species by subsequent designation in Curtis (1829: pl. 275) on the Official List of Specific Names in Zoology (ICZN 1980); however, as pointed out by Bousquet (2008a: 622), the type species *Cerambyxcarcharias* Linnaeus, 1758 was fixed earlier by Guérin- Méneville (1829b: 151), as recognized by recent authors (e.g., Danilevsky 2020: 473); the Official List of Generic Names in Zoology needs to be updated to reflect the changes for the type species mentioned above.

Family-group name: SAPERDAIRES Mulsant, 1839. Stem: *Saperd*-.

***Saphanus* Audinet-Serville, 1834b**: 81. Current status: valid genus in CERAMBYCIDAE: SPONDYLIDINAE: SAPHANINI.

Type species: *Callidiumspinosum* Fabricius, 1792 (= *Callidiumpiceum* Laicharting, 1784), by monotypy.

Family-group name: SAPHANIDAE Gistel, 1848. Stem: *Saphan*-.

***Saphoglossa* Sharp, 1883a**: 291. Current status: valid genus in STAPHYLINIDAE: ALEOCHARINAE: HYGRONOMINI: SAPHOGLOSSINA.

Type species: *Saphoglossapictipennis* Sharp, 1883, by monotypy.

Family-group name: SAPHOGLOSSAE Bernhauer & Scheerpeltz, 1926. Stem: *Saphogloss*-.

***Saprinus* Erichson, 1834**: 172. Current status: valid genus in HISTERIDAE: SAPRININAE: SAPRININI.

Type species: *Histernitidulus* Fabricius, 1801 (= *Histersemistriatus* Scriba, 1790), by subsequent designation (Westwood 1838c: 22).

Family-group name: SAPRINITES Blanchard, 1845. Stem: *Saprin*-.

***Saprus* Blackburn, 1904**: 150, 178. Current status: invalid senior synonym of *Sapriniana* Strand, 1917 in SCARABAEIDAE: AEGIALIINAE: SAPRINIANINI. Note: junior homonym of *Saprus* Gistel, 1856 [Coleoptera: SPHAERIUSIDAE].

Type species: *Saprusgriffithi* Blackburn, 1904, by monotypy.

Family-group name: SAPRINI Nikolajev, 2008. Stem: *Sapr*-. Note: family-group name permanently invalid (ICZN 1999a, Article 39).

***Sarothrias* Grouvelle, 1918**: 7. Current status: valid genus in JACOBSONIIDAE.

Type species: *Sarothriaseximius* Grouvelle, 1918, by monotypy.

Family-group name: SAROTHRIIDAE Crowson, 1955. Stem: *Sarothri*-.

***Sarothrocrepis* Chaudoir, 1850**: 76. Current status: valid genus in CARABIDAE: HARPALINAE: CYCLOSOMINI.

Type species: *Carabuscorticalis* Fabricius, 1801, by subsequent designation (Lacordaire 1854: 129).

Family-group name: SAROTHROCREPIDAE Chaudoir, 1876. Stem: *Sarothrocrepid*-.

***Sarrotrium* Illiger, 1798**: 339. Current status: junior synonym of *Orthocerus* Latreille, 1797 in ZOPHERIDAE: COLYDIINAE: ORTHOCERINI.

Type species: *Hispamutica* Linnaeus, 1767 (= *Dermestesclavicornis* Linnaeus, 1758), by monotypy.

Family-group name: SARROTRIIDES Billberg, 1820. Stem: *Sarrotri*-. Note: name placed on the Official Index of Rejected and Invalid Family-Group Names in Zoology (ICZN 1995d).

***Sawadaeuops* Legalov, 2003**: 398. Current status: junior synonym of *Riedeliops* Alonso-Zarazaga & Lyal, 2002 in ATTELABIDAE: ATTELABINAE: EUOPINI.

Type species: *Attelabuspunctatostriatus* Motschulsky, 1860, by original designation.

Family-group name: SAWADAEUOPSINA Legalov, 2007. Stem: *Sawadaeuops*- (ICZN 1999a, Article 29.4).

†***Sayrevilleus* Gratshev & Zherikhin, 2000b**: 244. Current status: valid genus in ATTELABIDAE: SAYREVILLEINAE: †SAYREVILLEINI. Note: Upper Cretaceous (New Jersey, USA).

Type species: †*Sayrevilleusgrimaldii* Gratshev & Zherikhin, 2000, by original designation.

Family-group name: †SANYREVILLEINA Legalov, 2003. Stem: *Sayreville*-.

***Scalidia* Erichson, 1845a**: 305. Current status: junior synonym of *Catogenus* Westwood, 1830 in PASSANDRIDAE: PASSANDRINAE.

Type species: *Passandracylindricollis* Lacordaire, 1854, by subsequent monotypy (Lacordaire 1854: 397).

Family-group name: SCALIDIINI Grouvelle, 1916. Stem: *Scalidi*-.

***Scaphicoma* Motschulsky, 1863**: 435. Current status: valid genus in STAPHYLINIDAE: SCAPHIDIINAE: SCAPHISOMATINI.

Type species: *Scaphicomaflavovittata* Motschulsky, 1863, by monotypy.

Family-group name: SCAPHICOMITAE Achard, 1924. Stem: *Scaphicom*-.

***Scaphidema* L. Redtenbacher, 1848**: 591. Current status: valid genus in TENEBRIONIDAE: DIAPERINAE: SCAPHIDEMINI.

Type species: *Scaphidiumbicolor* Fabricius, 1798, by monotypy.

Family-group name: SCAPHIDEMINI Reitter, 1922. Stem: *Scaphidem*-.

***Scaphidium* G.-A. Olivier, 1790a**: [20] 3. Current status: valid genus in STAPHYLINIDAE: SCAPHIDIINAE: SCAPHIDIINI.

Type species: *Scaphidiumquadrimaculatum* G.-A. Olivier, 1790, by subsequent designation (Latreille 1810: 427).

Family-group name: SCAPHIDILIA Latreille, 1806. Stem: *Scaphidi*-.

***Scaphisoma* Leach, 1815a**: 89. Current status: valid genus in STAPHYLINIDAE: SCAPHIDIINAE: SCAPHISOMATINI.

Type species: *Silphaagaricina* Linnaeus, 1758, by monotypy.

Family-group name: SCAPHISOMINI Casey, 1893. Stem: *Scaphisomat*-.

***Scaphium* W. Kirby, 1837**: 108. Current status: valid genus in STAPHYLINIDAE: SCAPHIDIINAE: SCAPHIINI.

Type species: *Scaphiumcastanipes* W. Kirby, 1837, by monotypy.

Family-group name: SCAPHIITAE Achard, 1924. Stem: *Scaphi*-.

***Scaphorhinadoretus* Ohaus, 1912a**: 426. Current status: valid genus in SCARABAEIDAE: RUTELINAE: ADORETINI: ADORETINA.

Type species: *Scaphorhinadoretusbimaculatus* Ohaus, 1912, by subsequent monotypy (Ohaus 1912a: 509).

Family-group name: SCAPHORHINADORETINA Ohaus, 1912. Stem: *Scaphorhinadoret*-.

***Scapterus* Dejean, 1826**: 471. Current status: valid genus in CARABIDAE: SCARITINAE: SCARITINI: SCAPTERINA.

Type species: *Scapterusguerini* Dejean, 1826, by monotypy.

Family-group name: SCAPTÉRIDES Putzeys, 1867. Stem: *Scapter*-.

***Scarabaeus* Linnaeus, 1758**: 342, 345. Current status: valid genus in SCARABAEIDAE: SCARABAEINAE: SCARABAEINI. Note: name placed on the Official List of Generic Names in Zoology (ICZN 2014a).

Type species: *Scarabaeussacer* Linnaeus, 1758, by subsequent designation (Hope 1837: 22; see ICZN 2014a). Note: name placed on the Official List of Specific Names in Zoology (ICZN 2014a).

Family-group name: SCARABAEÏDES Latreille, 1802. Stem: *Scarabae*-.

***Scarabatermes* Howden, 1973**: 29. Current status: valid genus in HYBOSORIDAE: CERATOCANTHINAE: SCARABATERMITINI.

Type species: *Scarabatermesamazonensis* Howden, 1973, by original designation.

Family-group name: SCARABATERMITINI Nikolajev, 1999. Stem: *Scarabatermit*-.

***Scardamyctes* Gistel, 1848b**: xi. Current status: junior synonym of *Magdalis* Germar, 1817 in CURCULIONIDAE: MESOPTILIINAE: MAGDALIDINI. Note: unnecessary new replacement name for *Thamnophilus* Schönherr, 1823 (an unnecessary new replacement name for *Magdalis* Germar, 1817).

Type species: *Curculioviolaceus* Linnaeus, 1758, by plenary power (see ICZN 1955a) (type fixation for *Magdalis* Germar).

Family-group name: SCARDAMYCTISIDAE Gistel, 1848. Stem: *Scardamyct*-. Note: nomen oblitum (see Bouchard et al. 2011: 621).

***Scarites* Fabricius, 1775**: 249. Current status: valid genus in CARABIDAE: SCARITINAE: SCARITINI: SCARITINA.

Type species: *Scaritessubterraneus* Fabricius, 1775, by subsequent designation (Stephens 1840: 352).

Family-group name: SCARITIDES Bonelli, 1810. Stem: *Scarit*-.

***Scatimus* Erichson, 1847c**: 110. Current status: valid genus in SCARABAEIDAE: SCARABAEINAE: ATEUCHINI: SCATIMINA.

Type species: *Scatimuscucullatus* Erichson, 1847, by monotypy.

Family-group name: SCATIMINA Vaz-de-Mello, 2008. Stem: *Scatim*-.

***Scatonomus* Erichson, 1835**: 256. Current status: valid genus in SCARABAEIDAE: SCARABAEINAE: DELTOCHILINI.

Type species: *Scatonomusviridis* Erichson, 1835, by subsequent designation (Westwood 1847a: 230)

Family-group name: SCATONOMIDES Lacordaire, 1855. Stem: *Scatonom*-.

***Scaurus* Fabricius, 1775**: 253. Current status: valid genus in TENEBRIONIDAE: TENEBRIONINAE: SCAURINI.

Type species: *Scaurusatratus* Fabricius, 1775, by monotypy.

Family-group name: SCAURIDES Billberg, 1820. Stem: *Scaur*-.

***Sceleocantha* Newman, 1840a**: 14. Current status: valid genus in CERAMBYCIDAE: PRIONINAE: SCELEOCANTHINI.

Type species: *Prionuspilosicollis* Hope, 1834, by subsequent designation (Chevrolat 1848: 411).

Family-group name: SCÉLÉOCANTHIDES Lacordaire, 1868. Stem: *Sceleocanth*-. Note: this name was treated as unavailable by Bouchard et al. (2011: 456) but was accepted as valid and attributed to Lacordaire, 1868 by Jin et al. (2023: 507).

***Scelida* Chapuis, 1875**: 184. Current status: valid genus in CHRYSOMELIDAE: GALERUCINAE: LUPERINI.

Type species: *Scelidaelegans* Chapuis, 1875, by monotypy.

Family-group name: SCELIDITES Chapuis, 1875. Stem: *Scelid*-.

***Scelodonta* Westwood, 1838b**: 129. Current status: junior synonym of *Heteraspis* Chevrolat, 1836 in CHRYSOMELIDAE: EUMOLPINAE: BROMIINI.

Type species: *Scelodontacurculionoides* Westwood, 1838, by monotypy.

Family-group name: SCELODONTITES Chapuis, 1874. Stem: *Scelodont*-.

***Sceloenopla* Chevrolat in Dejean, 1836a**: 364. Current status: valid genus in CHRYSOMELIDAE: CASSIDINAE: SCELOENOPLINI.

Type species: *Hispaspinipes* Fabricius, 1794 (= *Hispamaculata* G.-A. Olivier, 1792), by monotypy.

Family-group name: SCELOENOPLINI Uhmann, 1930. Stem: *Sceloenopl*-.

***Scelophysa* Burmeister, 1844**: 168. Current status: valid genus in SCARABAEIDAE: MELOLONTHINAE: HOPLIINI: HOPLIINA.

Type species: *Scelophysapruinosa* Burmeister, 1844, by subsequent designation (Dombrow 1999: 179).

Family-group name: SCELOPHYSIDES Péringuey, 1902. Stem: *Scelophys*-.

***Sceptobius* Sharp, 1883a**: 211. Current status: valid genus in STAPHYLINIDAE: ALEOCHARINAE: SCEPTOBIINI.

Type species: *Sceptobiusdispar* Sharp, 1883, by monotypy.

Family-group name: SCEPTOBIINI Seevers, 1978. Stem: *Sceptobi*-.

***Schedarosus* Reitter, 1876b**: 42. Current status: junior synonym of *Adelina* Dejean, 1835 in TENEBRIONIDAE: DIAPERINAE: DIAPERINI: ADELININA.

Type species: *Schedarosuscucujiformis* Reitter, 1876 (= *Pythopallida* Say, 1823), by subsequent designation (Löbl et al. 2008: 42).

Family-group name: SCHEDAROSINI Reitter, 1876. Stem: *Schedaros*-.

***Schedlarius* Wood, 1958**: 103. Current status: valid genus in CURCULIONIDAE: PLATYPODINAE: SCHEDLARIINI. Note: new replacement name for *Chapuisia* Dugès, 1886.

Type species: *Chapuisiamexicana* Dugès, 1886, by monotypy (type fixation for *Chapuisia* Dugès).

Family-group name: SCHEDLARINI Wood & Bright, 1992. Stem: *Schedlari*-.

***Schematiza* Chevrolat in Dejean, 1836a**: 377. Current status: valid genus in CHRYSOMELIDAE: GALERUCINAE: GALERUCINI.

Type species: *Lycuslaevigatus* Fabricius, 1801, by subsequent designation (Barber 1947b: 242).

Family-group name: SCHEMATIZITES Chapuis, 1875. Stem: *Schematiz*-.

***Schistodactylus* Raffray, 1883**: 243. Current status: valid genus in STAPHYLINIDAE: PSELAPHINAE: PSELAPHITAE: SCHISTODACTYLINI.

Type species: *Schistodactylusphantasma* Raffray, 1883, by monotypy.

Family-group name: SCHISTODACTYLINI Raffray, 1890. Stem: *Schistodactyl*-.

***Schistogenia* Kraatz, 1857a**: 39. Current status: valid genus in STAPHYLINIDAE: ALEOCHARINAE: ATHETINI: SCHISTOGENIINA.

Type species: *Schistogeniacrenicollis* Kraatz, 1857, by monotypy.

Family-group name: SCHISTOGENIAE Fenyes, 1919. Stem: *Schistogeni*-.

†***Schistomerus* Palmer, 1957**: 259. Current status: valid genus in DYTISCIDAE: HYDROPORINAE: †SCHISTOMERINI. Note: Miocene (USA).

Type species: †*Schistomeruscalifornense* Palmer, 1957, by original designation.

Family-group name: †SCHISTOMERINI Palmer, 1957. Stem: *Schistomer*-.

***Schizelythron* Kemner, 1925**: 110. Current status: valid genus in STAPHYLINIDAE: ALEOCHARINAE: TRICHOPSENIINI.

Type species: *Schizelythronjavanicum* Kemner, 1925, by original designation.

Family-group name: SCHIZELYTHRINAE Kemner, 1925. Stem: *Schizelythr*-.

†***Schizocoleus* Rohdendorf, 1961**: 438. Current status: valid genus in †SCHIZOCOLEIDAE. Note: Permian/Early Triassic (Russia)

Type species: †*Schizocoleuskuznetskiensis* Rohdendorf, 1961, by original designation.

Family-group name: †SCHIZOCOLEIDAE Rohdendorf, 1961. Stem: *Schizocole*-.

***Schizogenius* Putzeys, 1846**: 131. Current status: valid genus in CARABIDAE: SCARITINAE: CLIVININI: SCHIZOGENIINA.

Type species: *Schizogeniusstrigicollis* Putzeys, 1846, by subsequent designation (E. Desmarest 1851: 102).

Family-group name: SCHIZOGEINA Dostal, 2017: 115. Stem: *Schizogeni*-.

***Schizognathus* Fischer, 1823b**: 263. Current status: valid genus in SCARABAEIDAE: RUTELINAE: ANOPLOGNATHINI: SCHIZOGNATHINA.

Type species: *Schizognathusmacleayi* Fischer, 1823, by monotypy.

Family-group name: SCHIZOGNATHINA Ohaus, 1918. Stem: *Schizognath*-.

***Schizonycha* Dejean, 1833b**: 161. Current status: valid genus in SCARABAEIDAE: MELOLONTHINAE: SCHIZONYCHINI.

Type species: *Scarabaeusglobator* Fabricius, 1781, by subsequent designation (Pope 1960: 68).

Family-group name: SCHIZONYCHIDAE Burmeister, 1855. Stem: *Schizonych*-.

***Schizophilus* Bonvouloir, 1871**: 73. Current status: valid genus in EUCNEMIDAE: SCHIZOPHILINAE. Note: this name was first used by Gemminger in Gemminger and von Harold (1869b: 1480) but is unavailable from that date since it was not accompanied by a description or indication (ICZN 1999a, Article 12.1).

Type species: *Elatersubrufa* Randall, 1838, by subsequent monotypy (Bonvouloir 1872b: pl. 34, fig. 5). Note: the name on pl. 34 of Bonvouloir’s work is “*Schizophilustrilobatus*” but it was changed on p. 710 and in the “Corrections” (p. 885) to *S.subrufus*; the change was proposed at a later date (i.e., 1875) than the plate (1872b) but in one of the parts of the same volume and therefore the spelling must be corrected (ICZN 1999a, Article 32.5.1.1).

Family-group name: SCHIZOPHILINI Muona, 1993. Stem: *Schizophil*-.

†***Schizophorus* Ponomarenko, 1968**: 130. Current status: invalid senior synonym of †*Phoroschizus* Bouchard & Bousquet, 2020 in †PHOROSCHIZIDAE. Note: junior homonym of †*Schizophorus* Balashova, 1953 [Trilobita]; Upper Jurassic (Kazakhstan).

Type species: †*Schizophoruscrassus* Ponomarenko, 1968, by original designation.

Family-group name: †SCHIZOPHORIDAE Ponomarenko, 1968. Stem: *Schizophor*-. Note: family-group name permanently invalid (ICZN 1999a, Article 39).

***Schizopus* J.L. LeConte, 1858a**: 70. Current status: valid genus in SCHIZOPODIDAE: SCHIZOPODINI. Note: name placed on the Official List of Generic names in Zoology (ICZN 1993e).

Type species: *Schizopuslaetus* J.L. LeConte, 1858, by monotypy (see ICZN 1993e). Note: name placed on the Official List of Specific Names in Zoology (ICZN 1993e).

Family-group name: SCHIZOPODIDAE J.L. LeConte, 1859. Stem: *Schizopod*-.

***Schizorhina* W. Kirby, 1825**: 570. Current status: valid genus in SCARABAEIDAE: CETONIINAE: SCHIZORHININI: SCHIZORHININA.

Type species: *Cetoniaatropunctata* W. Kirby, 1819, by original designation.

Family-group name: SCHIZORRHINIDAE Burmeister, 1842. Stem: *Schizorhin*-.

***Schoenherriella* Viana, 1952**: 232. Current status: junior synonym of *Epipedophyes* G.A.K. Marshall, 1946 in CURCULIONIDAE: MOLYTINAE: PETALOCHILINI. Note: new replacement name for *Epipedus* Schönherr, 1842.

Type species: *Epipedussquamifer* Boheman, 1842, by original designation (type fixation for *Epipedus* Schönherr).

Family-group name: SCHOENHERRIELLINAE Viana, 1952. Stem: *Schoenherriell*-.

***Schyzoschelus* C. Koch, 1954**: 72. Current status: valid genus in TENEBRIONIDAE: BLAPTINAE: PLATYNOTINI: EURYNOTINA.

Type species: *Schyzoscheluskaszabi* C. Koch, 1954, by original designation.

Family-group name: SCHYZOSCHELINA C. Koch, 1956. Stem: *Schyzoschel*-.

†***Sciabregma* Scudder, 1893**: 146. Current status: valid genus in CURCULIONIDAE: MOLYTINAE: †SCIABREGMINI. Note: Tertiary (USA).

Type species: †*Sciabregmarugosa* Scudder, 1893, by monotypy.

Family-group name: †SCIABREGMINI Legalov, Kirejtshuk & Nel, 2020b: 736. Stem: *Sciabregm*-.

***Sciacharis* Broun, 1893b**: 1062. Current status: valid genus in STAPHYLINIDAE: SCYDMAENINAE: SCYDMAENITAE: STENICHNINI.

Type species: *Sciacharisfulva* Broun, 1893, by monotypy.

Family-group name: SCIACHARINI Csiki, 1919. Stem: *Sciacharit*-.

***Sciaphilus* Schönherr, 1823**: 1139. Current status: valid genus in CURCULIONIDAE: ENTIMINAE: CYPHICERITAE: SCIAPHILINI.

Type species: *Curculiomuricatus* Fabricius, 1792 (= *Curculioasperatus* Bonsdorff, 1785), by original designation.

Family-group name: SCIAPHILINA Sharp, 1891. Stem: *Sciaphil*-.

***Sciaphyes* Jeannel, 1910**: 7. Current status: valid genus in LEIODIDAE: CHOLEVINAE: SCIAPHYINI.

Type species: *Bathysciasibirica* Reitter, 1887, by original designation.

Family-group name: SCIAPHYINI Perreau, 2000. Stem: *Sciaphy*-.

***Sciatrophes* Blackburn, 1903**: 100. Current status: junior synonym of *Baeocera* Erichson, 1845 in STAPHYLINIDAE: SCAPHIDIINAE: SCAPHISOMATINI.

Type species: *Sciatropheslatens* Blackburn, 1903, by monotypy.

Family-group name: SCIATROPHITAE Achard, 1924. Stem: *Sciatroph*-.

***Scirtes* Illiger, 1807a**: 301. Current status: valid genus in SCIRTIDAE: SCIRTINAE.

Type species: *Chrysomelahemisphaerica* Linnaeus, 1758, by subsequent monotypy (Leach 1815a: 86).

Family-group name: SCIRTESIDAE Fleming, 1821. Stem: *Scirt*-.

***Scitala* Erichson, 1842**: 166. Current status: valid genus in SCARABAEIDAE: SERICOIDINAE: SCITALINI.

Type species: *Scitalasericans* Erichson, 1842, by subsequent designation (Blackburn 1898: 37).

Family-group name: SCITALINI Britton, 1957. Stem: *Scital*-.

***Sclerastes* Gistel, 1856**: 368. Current status: junior synonym of *Ptilinus* Geoffroy, 1762 in PTINIDAE: PTILININAE.

Type species: *Ptilinuscostatus* Gyllenhal, 1827 (= *Bostrichusfuscus* Geoffroy, 1785), by subsequent designation (Bouchard et al. 2011: 340).

Family-group name: SCLERASTEIDAE Gistel, 1856. Stem: *Sclerast*-.

***Sclerocardius* Schönherr, 1847**: 82. Current status: valid genus in CURCULIONIDAE: MOLYTINAE: ITHYPORINI: SCLEROCARDIINA.

Type species: *Sclerocardiusbohemani* Schönherr, 1847 (= *Heteropusafricanus* Boheman, 1845), by original designation.

Family-group name: SCLÉROCARDIIDES Lacordaire, 1865. Stem: *Sclerocardi*-.

***Scleron* Hope, 1841a**: 111. Current status: junior synonym of *Sclerum* Dejean, 1834 in TENEBRIONIDAE: BLAPTINAE: OPATRINI: SCLERINA.

Type species: *Opatrumorientale* Fabricius, 1775, by original designation.

Family-group name: SCLÉRIDES Lacordaire, 1859. Stem: *Scler*-.

***Scleropterus* Schönherr, 1825**: 585. Current status: valid genus in CURCULIONIDAE: CEUTORHYNCHINAE: SCLEROPTERINI.

Type species: *Cryptorhynchusserratus* Germar, 1823, by original designation.

Family-group name: SCLEROPTERIDAE Schultze, 1902. Stem: *Scleropter*-. Note: junior homonym of SCLEROPTERINAE Saussure, 1877 (type genus *Scleropterus* de Haan, 1842) in Orthoptera; Saussure’s name is permanently invalid (based on a preoccupied type genus).

***Sclerostomus* Burmeister, 1847**: 410, 423. Current status: valid genus in LUCANIDAE: LUCANINAE: SCLEROSTOMINI. Note: new replacement name for *Sclerognathus* Hope, 1845.

Type species: *Sclerognathuscostatus* Hope, 1845, by monotypy (type fixation for *Sclerognathus* Hope).

Family-group name: SCLEROSTOMINI Benesh, 1955. Stem: *Sclerostom*-.

***Scolopterus* A. White, 1846**: 14. Current status: valid genus in CURCULIONIDAE: CURCULIONINAE: EUGNOMINI: EUGNOMINA.

Type species: *Scolopterustetracanthus* A. White, 1846, by subsequent designation (Broun 1880: 473).

Family-group name: SCOLOPTÉRIDES Lacordaire, 1863. Stem: *Scolopter*-.

***Scolytoplatypus* C. Schaufuss, 1891b**: 31. Current status: valid genus in CURCULIONIDAE: SCOLYTINAE: SCOLYTOPLATYPODINI.

Type species: *Scolytoplatypuspermirus* C. Schaufuss, 1891, by monotypy.

Family-group name: SCOLYTOPLATYPINI W.F.H. Blandford, 1893. Stem: *Scolytoplatypod*-.

***Scolytus* Fabricius, 1790**: 221. Current status: invalid senior synonym of *Omophron* Latreille, 1802 in CARABIDAE: OMOPHRONINAE. Note: junior homonym of *Scolytus* Geoffroy, 1762 [Coleoptera: CURCULIONIDAE].

Type species: *Carabuslimbatus* Fabricius, 1777, by subsequent designation (Latreille 1810: 426) (type fixation for *Omophron* Latreille).

Family-group name: SCOLYTI Motschulsky, 1850. Stem: *Scolyt*-. Note: family-group name permanently invalid (ICZN 1999a, Article 39).

***Scolytus* Geoffroy, 1762**: 309. Current status: valid genus in CURCULIONIDAE: SCOLYTINAE: SCOLYTINI. Note: name placed on the Official List of Generic Names in Zoology (ICZN 1963c).

Type species: *Bostrichusscolytus* Fabricius, 1775, by plenary power (see ICZN 1963c). Note: name placed on the Official List of Specific Names in Zoology (ICZN 1963c).

Family-group name: SCOLITARII Latreille, 1804. Stem: *Scolyt*-.

***Scopadus* Pascoe, 1857**: 100. Current status: valid genus in CERAMBYCIDAE: LAMIINAE: CYRTININI.

Type species: *Scopadusciliatus* Pascoe, 1857, by monotypy.

Family-group name: SCOPADINI Villiers, 1980. Stem: *Scopad*-.

***Scopaeus* Erichson, 1839b**: 29. Current status: valid genus in STAPHYLINIDAE: PAEDERINAE: LATHROBIINI: SCOPAEINA.

Type species: *Paederuslaevigatus* Gyllenhal, 1827, by subsequent designation (Duponchel 1841b: 57). Note: no species were originally included in this genus by Erichson (1839b); the first ones were the nine species listed by Erichson (1840a: 605–609).

Family-group name: SCOPÉATES Mulsant & Rey, 1878. Stem: *Scopae*-.

***Scopodes* Erichson, 1842**: 123. Current status: valid genus in CARABIDAE: HARPALINAE: ODACANTHINI: PENTAGONICINA.

Type species: *Scopodesboops* Erichson, 1842, by monotypy.

Family-group name: SCOPODINAE H.W. Bates, 1874. Stem: *Scopod*-.

***Scortizus* Westwood, 1834**: 119. Current status: valid genus in LUCANIDAE: LUCANINAE: SCLEROSTOMINI.

Type species: *Pholidotusirroratus* Hope, 1833 (= *Lucanusmaculatus* Klug, 1825), by monotypy.

Family-group name: SCORTIZINI Benesh, 1955. Stem: *Scortiz*-.

***Scotobius* Germar, 1823**: 135. Current status: valid genus in TENEBRIONIDAE: TENEBRIONINAE: SCOTOBIINI.

Type species: *Scotobiuspilularius* Germar, 1823, by subsequent designation (Lacordaire 1830a: 282).

Family-group name: SCOTOBITES Solier, 1838. Stem: *Scotobi*-.

***Scotocryptus* M. Girard, 1874a**: 123. Current status: valid genus in LEIODIDAE: LEIODINAE: SCOTOCRYPTINI.

Type species: *Scotocryptusmeliponae* M. Girard, 1874, by monotypy. Note: combined description of a new genus and a single new species (ICZN 1999a, Article 12.2.6); the genus was redescribed and the species subsequently formally described by M. Girard (1875: 574).

Family-group name: SCOTOCRYPTINI Reitter, 1884. Stem: *Scotocrypt*-.

***Scotodipnus* Schaum, 1860**: 667. Current status: valid genus in CARABIDAE: TRECHINAE: ANILLINI.

Type species: *Anillusglaber* Baudi di Selve, 1859, by monotypy.

Family-group name: SCOTODIPNINA Jeannel, 1937. Stem: *Scotodipn*-.

***Scotodytes* Saulcy, 1865**: 18. Current status: valid subgenus of *Phloeocharis* Mannerheim, 1830 in STAPHYLINIDAE: PHLOEOCHARINAE.

Type species: *Scotodytesparadoxus* Saulcy, 1865, by monotypy.

Family-group name: SCOTODYTIDAE Saulcy, 1870. Stem: *Scotodyt*-.

***Scotoscymnus* Weise, 1901**: 458. Current status: junior synonym of *Scymnomorphus* Weise, 1897 in COCCINELLIDAE: MICROWEISEINAE: MICROWEISEINI. Note: unnecessary new replacement name for *Scymnomorphus* Weise, 1897.

Type species: *Scymnomorphusrotundatus* Weise, 1897, by subsequent designation (Pope 1962: 628).

Family-group name: SCOTOSCYMNINAE Duverger, 2003. Stem: *Scotoscymn*-.

***Scraptia* Latreille, 1806**: 199. Current status: valid genus in SCRAPTIIDAE: SCRAPTIINAE: SCRAPTIINI.

Type species: *Melandryafusca* Latreille, 1804 (= *Melyrisdubia* G.-A. Olivier, 1790), by monotypy.

Family-group name: SCRAPTIAEIDAE Gistel, 1848. Stem: *Scrapti*-.

***Scydmaenus* Latreille, 1802b**: 116. Current status: valid genus in STAPHYLINIDAE: SCYDMAENINAE: SCYDMAENITAE: SCYDMAENINI.

Type species: **here fixed** (under ICZN 1999a, Article 70.3.2) as *Scydmaenustarsatus* P.W.J. Müller & Kunze, 1822, misidentified as *Pselaphushellwigii* Herbst, 1791 (sensu Paykull, 1800) in the original designation by monotypy in Latreille (1802b: 116).

Family-group name: SCYDMAENIDES Leach, 1815. Stem: *Scydmaen*-.

***Scymnillus* G.H. Horn, 1895a**: 110. Current status: junior synonym of *Zilus* Mulsant, 1850 in COCCINELLIDAE: COCCINELLINAE: COCCIDULINI.

Type species: *Scymnillusaterrimus* G.H. Horn, 1895, by monotypy.

Family-group name: SCYMNILLINI Casey, 1899. Stem: *Scymnill*-.

***Scymnus* Kugelann, 1794**: 545. Current status: valid genus in COCCINELLIDAE: COCCINELLINAE: COCCIDULINI.

Type species: *Scymnusnigrinus* Kugelann, 1794, by subsequent designation (Westwood 1838c: 43). Note: this name is listed as nomen protectum (over *Coccinellaminima* O.F. Müller, 1776) by Kovář (2007: 590) but we were unable to verify that the conditions of Article23.9.1.2 (ICZN 1999a) were met.

Family-group name: SCYMNIAIRES Mulsant, 1846. Stem: *Scymn*-.

***Scythropus* Schönherr, 1826**: 12, 140. Current status: junior synonym of *Pachyrhinus* Schönherr, 1823 in CURCULIONIDAE: ENTIMINAE: CYPHICERITAE: SCYTHROPINI. Note: two original spellings were used, including *Scytropus* (p. 338); subsequently, Schönherr (1840b: 301) used *Scythropus* and therefore acted as First Reviser (ICZN 1999a, Article 24.2.4).

Type species: *Curculiomustela* Herbst, 1797 (= *Curculiosquamulosus* Herbst, 1795), by original designation.

Family-group name: SCYTHROPIDES Lacordaire, 1863. Stem: *Scythrop*-.

***Selatosomus* Stephens, 1830**: 268. Current status: valid genus in ELATERIDAE: DENDROMETRINAE: SELATOSOMINI: SELATOSOMINA.

Type species: *Elateraeneus* Linnaeus, 1758, by subsequent designation (Curtis 1838a: pl. 694).

Family-group name: SELATOSOMINI Schimmel, Tarnawski, Han & Platia, 2015: 30. Stem: *Selatosom*-.

†***Selengarhynchus* Gratshev & Legalov, 2009**: 415. Current status: valid genus in NEMONYCHIDAE: †SELENGARHYNCHINAE. Note: Lower Cretaceous (Mongolia).

Type species: †*Selengarhynchusovalis* Gratshev & Legalov, 2009, by original designation.

Family-group name: †SELENGARHYNCHINAE Gratshev & Legalov, 2009. Stem: *Selengarhynch*-.

***Selenophorus* Dejean, 1829**: 4, 80. Current status: valid genus in CARABIDAE: HARPALINAE: HARPALINI: HARPALINA.

Type species: *Carabuspalliatus* Fabricius, 1798, by subsequent designation (Hope 1838a: 84).

Family-group name: SELENOPHORINI Casey, 1914. Stem: *Selenophor*-.

***Selina* Motschulsky, 1857c**: 110. Current status: valid genus in CARABIDAE: HARPALINAE: LACHNOPHORINI: SELININA.

Type species: *Selinawestermanni* Motschulsky, 1857, by monotypy.

Family-group name: SELININI Jeannel, 1948. Stem: *Selin*-.

***Selvadius* Casey, 1899**: 134, 137. Current status: valid genus in COCCINELLIDAE: COCCINELLINAE: HYPERASPIDINI: SELVADIINA.

Type species: *Selvadiusrectus* Casey, 1899, by original designation.

Family-group name: SELVADIINI Gordon, 1985. Stem: *Selvadi*-.

***Semicyclus* Kaup, 1871**: 28. Current status: valid genus in PASSALIDAE: PASSALINAE: SOLENOCYCLINI.

Type species: *Semicyclusgrayi* Kaup, 1871, by monotypy.

Family-group name: SEMICYCLINAE Kuwert, 1891. Stem: *Semicycl*-.

***Semiotus* Eschscholtz, 1829a**: 31. Current status: valid genus in ELATERIDAE: DENDROMETRINAE: SEMIOTINI.

Type species: *Elaterfurcatus* Fabricius, 1792, by subsequent designation (Hyslop 1921: 668).

Family-group name: SEMIOTINA Jakobson, 1913. Stem: *Semiot*-.

***Senodonia* Laporte, 1838**: 12. Current status: valid genus in ELATERIDAE: LISSOMINAE: PROTELATERINI.

Type species: *Semiotusquadricollis* Laporte, 1838, by monotypy.

Family-group name: SENODONIINAE Schenkling, 1927. Stem: *Senodoni*-.

***Sepedonastes* Gistel, 1856**: 382. Current status: junior synonym of *Phaleria* Latreille, 1802 in TENEBRIONIDAE: DIAPERINAE: PHALERIINI.

Type species: *Tenebriobimaculatus* Herbst, 1799 (= *Dytiscusbimaculatus* Linnaeus, 1767), by subsequent designation (Bouchard et al. 2005: 501).

Family-group name: SEPEDONASTIDAE Gistel, 1856. Stem: *Sepedonast*-.

***Sepedophilus* Gistel, 1856**: 267, 386. Current status: valid genus in STAPHYLINIDAE: TACHYPORINAE: TACHYPORINI: EUCONOSOMATINA.

Type species: *Staphylinuspubescens* Paykull, 1790 (= *Staphylinuslittoreus* Linnaeus, 1758), by subsequent designation (Blackwelder 1952: 350). Note: *Staphylinuslittoreus* Linnaeus, 1758 was placed on the Official List of Specific Names in Zoology (ICZN 2003b).

Family-group name: SEPEDOPHILINI Ádám, 2001. Stem: *Sepedophil*-.

***Sepidium* Fabricius, 1775**: 250. Current status: valid genus in TENEBRIONIDAE: PIMELIINAE: SEPIDIINI: SEPIDIINA.

Type species: *Sepidium tricuspidatum* Fabricius, 1775, by subsequent designation (Latreille 1810: 429).

Family-group name: SEPIDIAE Eschscholtz, 1829. Stem: *Sepidi*-.

†***Septiventer* Bai, Ren, Shih & X.-K. Yang in Bai et al., 2012b**: 364. Current status: valid genus in †SEPTIVENTERIDAE. Note: Lower Cretaceous (China).

Type species: †*Septiventerquadridentatus* Bai, Ren, Shih & X.-K. Yang, 2012, by original designation.

Family-group name: †SEPTIVENTERIDAE Bai, Ren, Shih & X.-K. Yang in Bai et al., 2012b: 364. Stem: *Septiventer*- (ICZN 1999a, Article 29.4).

***Serangium* Blackburn, 1889**: 187, 209. Current status: valid genus in COCCINELLIDAE: MICROWEISEINAE: SERANGIINI.

Type species: *Serangiummysticum* Blackburn, 1889, by monotypy.

Family-group name: SERANGIINI Blackwelder, 1945. Stem: *Serangi*-.

***Serica* W.S. MacLeay, 1819**: 146. Current status: valid genus in SCARABAEIDAE: SERICINAE: SERICINI: SERICINA.

Type species: *Scarabaeusbrunneus* Linnaeus, 1758 [as *brunnus*], by monotypy.

Family-group name: SERICIDAE W. Kirby, 1837. Stem: *Seric*-.

***Sericoda* W. Kirby, 1837**: 14. Current status: valid genus in CARABIDAE: HARPALINAE: PLATYNINI.

Type species: *Sericodabembidioides* W. Kirby, 1837, by monotypy.

Family-group name: SERICODIADAE W. Kirby, 1837. Stem: *Sericod*-.

***Sericoderus* Stephens, 1829a**: 158. Current status: valid genus in CORYLOPHIDAE: CORYLOPHINAE: SERICODERINI.

Type species: *Sericoderusthoracicus* Stephens, 1829 (= *Cossyphuslateralis* Gyllenhal, 1827), by subsequent monotypy (Stephens 1829a: 188).

Family-group name: SERICODERINA A. Matthews, 1886. Stem: *Sericoder*-.

***Sericoides* Guérin-Méneville, 1839b**: 301. Current status: valid genus in SCARABAEIDAE: SERICOIDINAE: SERICOIDINI.

Type species: *Melolonthaglacialis* Fabricius, 1775, by subsequent designation (E. Desmarest 1860: 68).

Family-group name: SERICOIDEAE Erichson, 1847. Stem: *Sericoid*-.

***Sericosomus* Dejean, 1833a**: 96. Current status: junior synonym of *Sericus* Eschscholtz, 1829 in ELATERIDAE: ELATERINAE: ELATERINI. Note: unnecessary new replacement name for *Sericus* Eschscholtz, 1829.

Type species: *Elaterbrunneus* Linnaeus, 1758, by subsequent designation (Curtis 1838a: pl. 694) (type fixation for *Sericus* Eschscholtz).

Family-group name: SERICOSOMINA Hyslop, 1917. Stem: *Sericosom*-.

***Sermyla* Chapuis, 1875**: 224. Current status: invalid senior synonym of *Sermylassa* Reitter, 1912 in CHRYSOMELIDAE: GALERUCINAE: HYLASPINI. Note: junior homonym of *Sermyla* Adams, 1854 [Mollusca] and *Sermyla* Walker, 1854 [Lepidoptera].

Type species: *Chrysomelahalensis* Linnaeus, 1767, by monotypy.

Family-group name: SERMYLITES Chapuis, 1875. Stem: *Sermyl*-. Note: family-group name permanently invalid (ICZN 1999a, Article 39).

***Sermylassa* Reitter, 1913c**: 138. Current status: valid genus in CHRYSOMELIDAE: GALERUCINAE: HYLASPINI. Note: new replacement name for *Sermyla* Chapuis, 1875.

Type species: *Chrysomelahalensis* Linnaeus, 1767, by monotypy (type fixation for *Sermyla* Chapuis).

Family-group name: SERMYLASSINI Mroczkowski, 1991. Stem: *Sermylass*-.

***Serraticollis* B.E. White, 1942**: 17. Current status: junior synonym of *Aulacothorax* Boheman, 1858 in CHRYSOMELIDAE: GALERUCINAE: SERRATICOLLINI.

Type species: *Serraticollisrhois* B.E. White, 1942 (= *Halticarecticollis* J.L. LeConte, 1861), by original designation.

Family-group name: SERRATICOLLINI B.E. White, 1942. Stem: *Serraticoll*-.

***Serropalpus* Hellenius, 1786**: 310. Current status: valid genus in MELANDRYIDAE: MELANDRYINAE: SERROPALPINI.

Type species: *Serropalpusstriatus* Hellenius, 1786 (= *Mordellabarbata* Schaller, 1783), by subsequent designation (Blanchard in Audouin et al. 1844: pl. 53).

Family-group name: SERROPALPIDES Latreille, 1829. Stem: *Serropalp*-.

***Sertorius* Kaup, 1871**: 114. Current status: invalid senior synonym of *Arrox* Zang, 1905 in PASSALIDAE: PASSALINAE: PROCULINI. Note: junior homonym of *Sertorius* Stål, 1866 [Hemiptera].

Type species: *Sertoriusagassizi* Kaup, 1871, by monotypy.

Family-group name: SERTORINAE Kuwert, 1891. Stem: *Sertori*-. Note: family-group name permanently invalid (ICZN 1999a, Article 39).

***Sestyra* Pascoe, 1867a**: 513. Current status: valid genus in CERAMBYCIDAE: CERAMBYCINAE: SESTYRINI.

Type species: *Sestyracephalotes* Pascoe, 1867, by original designation.

Family-group name: SESTYRIDES Lacordaire, 1868. Stem: *Sestyr*-.

***Setapion* F. Balfour-Browne, 1944**: 71. Current status: valid genus in BRENTIDAE: APIONINAE: SETAPIITAE.

Type species: *Setapionquantillum* F. Balfour-Browne, 1944, by original designation.

Family-group name: SETAPIITAE Legalov, 2023c: 30. Stem: *Setapi*-. Note: SETAPIITAELegalov (2018c: 811) was published in a work that is nomenclaturally unavailable (ICZN 2012f, Article 8.5.3).

***Setaria* Mulsant & Rey, 1864a**: 1. Current status: invalid senior synonym of *Setariola* Jakobson, 1915 in EROTYLIDAE: PHARAXONOTHINAE. Note: junior homonym of *Setaria* Viborg, 1795 [Vermes] and *Setaria* Blyth, 1844 [Aves].

Type species: *Setariasericea* Mulsant & Rey, 1864, by monotypy.

Family-group name: SETARIINI Casey, 1900. Stem: *Setari*-. Note: family-group name permanently invalid (ICZN 1999a, Article 39).

***Setariola* Jakobson, 1915**: 941. Current status: valid genus in EROTYLIDAE: PHARAXONOTHINAE. Note: new replacement name for *Setaria* Mulsant & Rey, 1864.

Type species: *Setariasericea* Mulsant & Rey, 1864, by monotypy (type fixation for *Setaria* Mulsant & Rey).

Family-group name: SETARIOLINAE Crowson, 1952. Stem: *Setariol*-.

***Shirozuella* Sasaji, 1967**: 24. Current status: valid genus in COCCINELLIDAE: COCCINELLINAE: SHIROZUELLINI.

Type species: *Shirozuellamirabilis* Sasaji, 1967, by original designation.

Family-group name: SHIROZUELLINI Sasaji, 1967. Stem: *Shirozuell*-.

***Siagona* Latreille, 1804a**: 141. Current status: valid genus in CARABIDAE: SIAGONINAE: SIAGONINI.

Type species: *Cucujusrufipes* Fabricius, 1792, by monotypy.

Family-group name: SIAGONES Bonelli, 1813. Stem: *Siagon*-.

***Siamites* H. Franz, 1989**: 44. Current status: valid genus in STAPHYLINIDAE: SCYDMAENINAE: SCYDMAENITAE: STENICHNINI.

Type species: *Siamitesloebli* H. Franz, 1989, by original designation.

Family-group name: SIAMITINI H. Franz, 1989. Stem: *Siamit*-.

***Sibinia* Germar, 1817b**: 340. Current status: valid genus in CURCULIONIDAE: CURCULIONINAE: TYCHIINI: TYCHIINA.

Type species: *Curculioviscariae* Linnaeus, 1761, by subsequent designation (Schönherr 1825: 583).

Family-group name: SIBYNIDAE Marseul, 1863. Stem: *Sibini*-.

***Sibylla* J. Thomson, 1858a**: 406. Current status: invalid senior synonym of *Zehra* Özdikmen, 2008 in CERAMBYCIDAE: CERAMBYCINAE: BIMIINI. Note: junior homonym of *Sibylla* Stål, 1856 [Orthoptera].

Type species: *Phoedinuscoemeterii* J. Thomson, 1856, by monotypy.

Family-group name: SIBYLLINI Cerda, 1973. Stem: *Sibyll*-. Note: family-group name permanently invalid (ICZN 1999a, Article 39).

***Siderodactylus* Schönherr, 1834a**: 125. Current status: junior synonym of *Hadromerus* Schönherr, 1823 in CURCULIONIDAE: ENTIMINAE: POLYDRUSITAE: TANYMECINI: TANYMECINA.

Type species: *Curculiosagittarius* G.-A. Olivier, 1807, by original designation.

Family-group name: SIDERODACTYLIDES Jekel, 1865. Stem: *Siderodactyl*-.

***Siettitia* Abeille de Perrin, 1904**: 226. Current status: valid genus in DYTISCIDAE: HYDROPORINAE: HYDROPORINI: SIETTITIINA.

Type species: *Siettitiabalsetensis* Abeille de Perrin, 1904, by monotypy.

Family-group name: SIETTITIINI Smrž, 1982. Stem: *Siettiti*-.

***Sikhotealinia* Lafer, 1996**: 392. Current status: valid genus in JURODIDAE.

Type species: *Sikhotealiniazhiltzovae* Lafer, 1996, by original designation.

Family-group name: SIKHOTEALINIIDAE Lafer, 1996. Stem: *Sikhotealini*-.

***Silis* Charpentier, 1825**: 194. Current status: valid genus in CANTHARIDAE: SILINAE: SILINI.

Type species: *Silisrubricollis* Charpentier, 1825 (= *Cantharisruficollis* Fabricius, 1775), by subsequent designation (Hope 1841a: 141). Note: Kazantsev (2011: 28) mentioned that the type species was designated by Westwood (1838c) but Westwood cited “*Cantharisruficollis* Fab.” as type species, which is not an originally included species, and he did not mention that the species was a synonym of *Silisrubricollis* Charpentier, one of the two originally included species.

Family-group name: SILIAIRES Mulsant, 1862. Stem: *Sil*-.

***Silluvia* Landin, 1950**: 5. Current status: valid genus in SCARABAEIDAE: AEGIALIINAE: AEGIALIINI.

Type species: *Silluviaelongata* Landin, 1950, by original designation.

Family-group name: SILLUVIINAE Landin, 1950. Stem: *Silluvi*-.

***Silpha* Linnaeus, 1758**: 342, 359. Current status: valid genus in STAPHYLINIDAE: SILPHINAE: SILPHINI.

Type species [suggested]: *Silphaobscura* Linnaeus, 1758, by subsequent designation (Curtis 1839: pl. 742). Note: the valid type species fixation is that of Latreille (1810: 427) who selected *Silphalittoralis* Linnaeus, 1758, but that species is the type species of the genus *Necrodes* Leach, 1815; a subsequent designation based on an originally included nominal species is that of Westwood (1838c: 11), who selected *Silphaquadripunctata* Linnaeus, 1758, but that species is the type species of the genus *Glischrochilus* Reitter, 1873; an application to the Commission is necessary to fix *Silphaobscura* Linnaeus, 1758 as the type species of *Silpha* Linnaeus, 1758.

Family-group name: SILPHALES Latreille, 1806. Stem: *Silph*-.

***Silphotelus* Broun, 1895**: 83. Current status: valid genus in STAPHYLINIDAE: PROTEININAE: SILPHOTELINI.

Type species: *Silphotelusnitidus* Broun, 1895, by monotypy.

Family-group name: SILPHOTELINI Newton & Thayer, 1995. Stem: *Silphotel*-.

***Silusa* Erichson, 1837**: 377. Current status: valid genus in STAPHYLINIDAE: ALEOCHARINAE: HOMALOTINI: SILUSINA.

Type species: *Silusarubiginosa* Erichson, 1837, by monotypy.

Family-group name: SILUSAE Fenyes, 1919. Stem: *Silus*-.

***Silvanus* Latreille, 1804a**: 158. Current status: valid genus in SILVANIDAE: SILVANINAE.

Type species: *Ipsunidentata* G.-A. Olivier, 1790, by monotypy.

Family-group name: SYLVANIDAE W. Kirby, 1837. Stem: *Silvan*-.

***Simo* Dejean, 1821**: 92. Current status: valid genus in CURCULIONIDAE: ENTIMINAE: CYPHICERITAE: PERITELINI: SIMONINA.

Type species: *Curculiohirticornis* Herbst, 1795, by monotypy.

Family-group name: SIMOINI Pierce, 1913. Stem: *Simon*-.

***Simous* Chaudoir, 1882**: 373. Current status: valid genus in CARABIDAE: HARPALINAE: OODINI.

Type species: *Oodesnigriceps* Wiedemann, 1821, by subsequent designation (Andrewes 1938: 138).

Family-group name: SIMOINI Basilewsky, 1953. Stem: *Simo*-.

***Simplocaria* Stephens, 1829b**: 9. Current status: valid genus in BYRRHIDAE: BYRRHINAE: SIMPLOCARIINI. Note: name placed on the Official List of Generic Names in Zoology (ICZN 1985c).

Type species: *Byrrhussemistriatus* Fabricius, 1794, by subsequent designation (Curtis 1830: pl. 335; see ICZN 1985c where the same type species was recorded on the Official List as subsequently designated by Jacquelin du Val (1859b: 267)). Note: name placed on the Official List of Specific Names in Zoology (ICZN 1985c).

Family-group name: SIMPLOCARIATES Mulsant & Rey, 1869. Stem: *Simplocari*-.

***Simus* Raffray, 1882**: 6. Current status: invalid senior synonym of *Ipsimus* Reitter, 1885 in STAPHYLINIDAE: PSELAPHINAE: GONIACERITAE: GONIACERINI. Note: junior homonym of *Simus* Bonaparte, 1838 [Reptilia].

Type species: *Simusfracticornis* Raffray, 1882, by monotypy.

Family-group name: SIMINI L.W. Schaufuss, 1890. Stem: *Sim*-. Note: family-group name permanently invalid (ICZN 1999a, Article 39).

***Sinanarchusa* Pace, 2013b**: 23. Current status: valid genus in STAPHYLINIDAE: ALEOCHARINAE: HIMALUSINI.

Type species: *Sinanarchusadaxuensis* Pace, 2013, by original designation.

Family-group name: SINANARCHUSINI Pace, 2013b: 22. Stem: *Sinanarchus*-.

***Singhikalia* Kapur, 1963**: 16. Current status: valid genus in COCCINELLIDAE: COCCINELLINAE: COCCINELLINI.

Type species: *Singhikaliaornata* Kapur, 1963, by original designation.

Family-group name: SINGHIKALIINI Miyatake, 1972. Stem: *Singhikali*-.

***Singiliomimus* Péringuey, 1896**: 243. Current status: valid genus in CARABIDAE: HARPALINAE: LEBIINI: DROMIUSINA.

Type species: *Singiliomimusposticalis* Péringuey, 1896, by subsequent designation (Lorenz 1998: 161).

Family-group name: SINGILIOMIMINA Basilewsky, 1984. Stem: *Singiliomim*-.

***Singilis* Rambur, 1837**: 25. Current status: valid genus in CARABIDAE: HARPALINAE: LEBIINI: DROMIUSINA.

Type species: *Singilisbicolor* Rambur, 1837, by subsequent designation (Antoine 1963: 551).

Family-group name: SINGILINI Jeannel, 1949. Stem: *Singil*-.

†***Sinisilvana* Hong, 2002**: 134. Current status: valid genus in †SINISILVANIDAE. Note: Eocene (China).

Type species: †*Sinisilvanafushunensis* Hong, 2002, by original designation.

Family-group name: †SINISILVANIDAE Hong, 2002. Stem: *Sinisilvan*-.

***Sinodendron* Hellwig in Fabricius, 1791**: 18. Current status: valid genus in LUCANIDAE: SYNDESINAE: SINODENDRINI. Note: this name was first proposed as a synonym of *Ligniperda* Fabricius, 1791 and, since it was used as valid before 1961 (e.g., Latreille 1804a: 149), it therefore dates from its proposal in synonymy (ICZN 1999a, Article 11.6.1), as opposed to being credited to Hellwig (1792: 391) as in recent literature (e.g., Bartolozzi et al. 2016: 60).

Type species: *Scarabaeuscylindricus* Linnaeus, 1758, by monotypy.

Family-group name: SINODENDRIENS Mulsant, 1842. Stem: *Sinodendr*-.

†***Sinodryopites* Hong, 2002**: 110. Current status: valid genus in SCIRTIDAE incertae sedis. Note: Eocene (China).

Type species: †*Sinodryopitesovalifemorales* Hong, 2002, by original designation.

Family-group name: †SINODRYOPITIDAE Hong, 2002. Stem: *Sinodryopit*-.

***Sinopyrophorus* Bi & Li in Bi et al., 2019**: 83. Current status: valid genus in SINOPYROPHORIDAE.

Type species: *Sinopyrophorusschimmeli* Bi & X.-Y. Li, 2019, by original designation.

Family-group name: SINOPYROPHORINAE Bi & X.-Y. Li in Bi et al., 2019: 89. Stem: *Sinopyrophor*-.

***Sinoxylon* Duftschmid, 1825**: 85. Current status: valid genus in BOSTRICHIDAE: BOSTRICHINAE: SINOXYLINI.

Type species: *Dermestesmuricatus* Linnaeus, 1767 nomen oblitum (= *Bostrichussexdentatus* G.-A. Olivier, 1790 nomen protectum), by monotypy. Note: see Ivie (2010: 40) regarding the identity of the type species.

Family-group name: SINOXYLIDAE Marseul, 1857. Stem: *Sinoxyl*-.

***Sinozolus* Deuve, 1997**: 32. Current status: valid genus in CARABIDAE: TRECHINAE: SINOZOLINI: SINOZOLINA.

Type species: *Sinozolusyuae* Deuve, 1997, by monotypy. Note: combined description of a new genus and a single new species (ICZN 1999a, Article 13.4).

Family-group name: SINOZOLINI Deuve, 1997. Stem: *Sinozol*-.

***Sintor* Schönherr, 1839**: 148. Current status: valid genus in ANTHRIBIDAE: ANTHRIBINAE: SINTORINI.

Type species: *Sintorquadrilineatus* Fåhraeus, 1839, by original designation.

Family-group name: SINTORIDES Lacordaire, 1865. Stem: *Sintor*-.

***Sipalia* Mulsant & Rey, 1853b**: 32. Current status: invalid senior synonym of *Cyllopisalia* Pace, 1982 in STAPHYLINIDAE: ALEOCHARINAE: HOMALOTINI: BOLITOCHARINA. Note: name suppressed for the purposes of the Principle of Priority and placed on the Official Index of Rejected and Invalid Generic Names in Zoology (ICZN 2005d).

Type species: *Homalotadifformis* Mulsant & Rey, 1853, by subsequent designation (Fauvel 1902: 40). Note: name placed on the Official List of Specific Names in Zoology (ICZN 2005d).

Family-group name: SIPALIAE Casey, 1910. Stem: *Sipali*-. Note: family-group name permanently invalid (ICZN 1999a, Article 39).

***Sipalinus* G.A.K. Marshall, 1943**: 119. Current status: valid genus in CURCULIONIDAE: DRYOPHTHORINAE: ORTHOGNATHINI. Note: new replacement name for *Sipalus* Schönherr, 1825.

Type species: *Calandragranulata* Fabricius, 1801 (= *Curculiogigas* Fabricius, 1775), by original designation (type fixation for *Sipalus* Schönherr).

Family-group name: SIPALININAE Zimmerman, 1993. Stem: *Sipalin*-.

***Sipalomimus* Voss, 1958**: 125. Current status: valid genus in CURCULIONIDAE: DRYOPHTHORINAE: SIPALOMIMINI.

Type species: *Sipalomimussingularis* Voss, 1958, by original designation.

Family-group name: SIPALOMIMINI Legalov, 2020a: 325. Stem: *Sipalomim*-.

***Sipalus* Schönherr, 1825**: 587. Current status: invalid senior synonym of *Sipalinus* G.A.K. Marshall, 1943 in CURCULIONIDAE: DRYOPHTHORINAE: ORTHOGNATHINI. Note: junior homonym of *Sipalus* Fischer, 1813 [Mammalia].

Type species: *Calandragranulata* Fabricius, 1801 (= *Curculiogigas* Fabricius, 1775), by original designation.

Family-group name: SIPALIDES Lacordaire, 1865. Stem: *Sipal*-. Note: family-group name permanently invalid (ICZN 1999a, Article 39).

***Sisyphus* Latreille, 1806**: 79. Current status: valid genus in SCARABAEIDAE: SCARABAEINAE: SISYPHINI. Note: the original spelling was *Sisyphe* but *Sisyphus*, an incorrect subsequent spelling, is in prevailing usage, attributed to the publication of the original spelling, and therefore deemed to be the correct original spelling (ICZN 1999a, Article 33.3.1).

Type species: *Scarabaeusschaefferi* Linnaeus, 1758, by monotypy.

Family-group name: SISYPHAIRES Mulsant, 1842. Stem: *Sisyph*-.

***Sitaris* Latreille, 1802b**: 187. Current status: valid genus in MELOIDAE: NEMOGNATHINAE: NEMOGNATHINI: SITARINA.

Type species: *Necydalishumeralis* Fabricius, 1775 (= *Necydalismuralis* Forster, 1771), by monotypy. Note: Fabricius’s specific name was spelled *humuralis* but the spelling *humeralis*, an incorrect subsequent spelling, is in prevailing usage, attributed to the publication of the original spelling, and therefore deemed to be the correct original spelling (ICZN 1999a, Article 33.3.1).

Family-group name: SITARATES Mulsant, 1857. Stem: *Sitar*-.

***Sitona* Germar, 1817b**: 341. Current status: valid genus in CURCULIONIDAE: ENTIMINAE: POLYDRUSITAE: SITONINI.

Type species: *Curculiolineatus* Linnaeus, 1758, by subsequent designation (Schönherr 1823: 1141).

Family-group name: SITONISIDAE Gistel, 1848. Stem: *Siton*-.

***Sitophilus* Schönherr, 1838**: 967. Current status: valid genus in CURCULIONIDAE: DRYOPHTHORINAE: LITOSOMINI: SITOPHILINA. Note: name placed on the Official List of Generic Names in Zoology (ICZN 1959b).

Type species: *Curculiooryzae* Linnaeus, 1763, by original designation (see ICZN 1959b). Note: although the species was originally spelled *oryza*, the emended spelling *oryzae* was validated by the Commission and the name placed on the Official List of Specific Names in Zoology (ICZN 1959b).

Family-group name: SCASITOPHILIPHIDILIA Csiki, 1936. Stem: *Sitophil*-.

***Skatitoxenus* Kistner & Pasteels, 1969**: 1190. Current status: valid genus in STAPHYLINIDAE: ALEOCHARINAE: SKATITOXENINI.

Type species: *Skatitoxenuscoatoni* Kistner & Pasteels, 1969, by original designation.

Family-group name: SKATITOXENINI Kistner & Pasteels, 1969. Stem: *Skatitoxen*-.

***Slipinskia* Bocák & Bocáková, 1992**: 257. Current status: junior synonym of *Flagrax* Kasantsev, 1992 in LYCIDAE: EROTINAE: SLIPINSKIINI.

Type species: *Stadenusaethiops* Kleine, 1933, by original designation.

Family-group name: SLIPINSKIININA Bocák & Bocáková, 1992. Stem: *Slipinski*-.

***Sloanoglymmius* R.T. Bell & J.R. Bell, 1991**: 185. Current status: valid genus in CARABIDAE: RHYSODINAE: SLOANOGLYMMIINI.

Type species: *Rhysodesplanatus* Lea, 1904, by original designation.

Family-group name: SLOANOGLYMMIINA R.T. Bell & J.R. Bell, 1991. Stem: *Sloanoglymmi*-.

†***Slonik* Zherikhin, 1977b**: 180. Current status: valid genus in NEMONYCHIDAE: †SLONIKINAE. Note: Lower Cretaceous (Kazakhstan).

Type species: †*Sloniksibiricus* Zherikhin, 1977, by original designation.

Family-group name: †SLONIKINAE Zherikhin, 1977. Stem: *Slonik*-.

***Smicrips* J.L. LeConte, 1878a**: 399. Current status: valid genus in SMICRIPIDAE.

Type species: *Smicripspalmicola* J.L. LeConte, 1878 (= *Tisiphonehypocoproides* Reitter, 1876), by monotypy.

Family-group name: SMICRIPINI G.H. Horn, 1880. Stem: *Smicrip*-.

***Smicronyx* Schönherr, 1843**: 313. Current status: valid genus in CURCULIONIDAE: CURCULIONINAE: SMICRONYCHINI. Note: new replacement name for *Micronyx* Schönherr, 1835.

Type species: *Micronyxreichii* Gyllenhal, 1835, by original designation (type fixation for *Micronyx* Schönherr).

Family-group name: SMICRONYCINA Seidlitz, 1891. Stem: *Smicronych*-. Note: nomen protectum (see Bouchard et al. 2011: 584).

***Smodicum* Haldeman, 1847**: 38. Current status: valid genus in CERAMBYCIDAE: CERAMBYCINAE: SMODICINI.

Type species: *Callidiumcucujiforme* Say, 1826, by monotypy.

Family-group name: SMODICIDES Lacordaire, 1868. Stem: *Smodic*-.

***Sobarus* Harold, 1879**: 164. Current status: valid genus in CERAMBYCIDAE: PRIONINAE: ANACOLINI. Note: junior homonym of *Sobarus* Loew, 1855 [Diptera]; since no new replacement name has been proposed for *Sobarus* Harold, we here propose *Basorus* Bouchard & Bousquet, **nomen novum**.

Type species: *Sobaruspoggei* Harold, 1879, by monotypy.

Family-group name: SOBARINI Lameere, 1913. Stem: *Sobar*-. Note: family-group name permanently invalid (ICZN 1999a, Article 39).

***Sogda* Lopatin, 1961**: 121. Current status: valid genus in LEIODIDAE: LEIODINAE: SOGDINI.

Type species: *Sogdapavlovskii* Lopatin, 1961, by original designation.

Family-group name: SOGDIIDAE Lopatin, 1961. Stem: *Sogd*-.

***Soleniscus* Bonvouloir, 1871**: 76 [in key]. Current status: invalid senior synonym of *Cussolenis* Fleutiaux, 1918 in ELATERIDAE: THYLACOSTERNINAE. Note: junior homonym of *Soleniscus* Meek & Worthen, 1860 [Mollusca]; this name was first used by Gemminger in Gemminger and von Harold (1869b: 1486) but is unavailable from that date as the species associated with the name is a nomen nudum; the plates of Bonvouloir’s work on EUCNEMIDAE referred to by Gemminger were not engraved until 1870 as listed at the top of the plates, and the part including *Soleniscus* was not published until 1871.

Type species: *Soleniscusmutabilis* Bonvouloir, 1875, by subsequent monotypy (de Bonvouloir 1875: 828).

Family-group name: SOLENISCINAE Lameere, 1900. Stem: *Solenisc*-. Note: family-group name permanently invalid (ICZN 1999a, Article 39).

***Solenocyclus* Kaup, 1868a**: 10. Current status: valid genus in PASSALIDAE: PASSALINAE: SOLENOCYCLINI.

Type species: *Passalusexaratus* Klug, 1833, by monotypy.

Family-group name: SOLENOCYCLEAE Kaup, 1871. Stem: *Solenocycl*-.

***Solenogenys* Westwood, 1860**: 170. Current status: valid genus in CARABIDAE: SCARITINAE: SALCEDIINI: SOLENOGENYINA. Note: this taxon is a junior synonym of *Aulacinia* J. Thomson, 1857 and an application to the Commission is necessary to preserve *Solenogenys* as a valid taxon.

Type species: *Solenogenysfoeda* Westwood, 1860 (= *Aulaciniarhysodoides* J. Thompson, 1857), by monotypy.

Family-group name: SOLENOGENYINA R.T. Bell, 1998. Stem: *Solenogeny*-.

***Solenoptera* Audinet-Serville, 1832**: 183. Current status: valid genus in CERAMBYCIDAE: PRIONINAE: SOLENOPTERINI.

Type species: *Cerambyxthomae* Linnaeus, 1767, by subsequent designation (Cazurro Ruiz 1896: 430).

Family-group name: SOLÉNOPTÉRIDES Lacordaire, 1868. Stem: *Solenopter*-.

***Solierius* Bernhauer, 1921**: 68. Current status: valid genus in STAPHYLINIDAE: SOLIERIINAE. Note: new replacement name for *Physognathus*Solier 1849 (junior homonym of *Physognathus*Agassiz 1846, an emendation of *Physignathus* Cuvier, 1829).

Type species: *Physognathusobscurus* Solier, 1849, by monotypy (type fixation for *Physognathus* Solier).

Family-group name: SOLIERIINAE Newton & Thayer, 1992. Stem: *Solieri*-.

***Somatipion* L.W. Schaufuss, 1877b**: 457. Current status: valid genus in STAPHYLINIDAE: PSELAPHINAE: PSELAPHITAE: TYRINI: SOMATIPIONINA.

Type species: *Somatipionglobulifer* L.W. Schaufuss, 1877 (= *Faronuspunctatus* King, 1866), by monotypy.

Family-group name: SOMATIPIONINI Jeannel, 1949. Stem: *Somatipion*-.

***Somatodes* Schönherr, 1823**: 1139. Current status: junior synonym of *Pachyrhynchus* Germar, 1823 in CURCULIONIDAE: ENTIMINAE: POLYDRUSITAE: PACHYRHYNCHINI. Note: name suppressed for the Principles of Priority and Homonymy and placed on the Official Index of Rejected and Invalid Generic Names in Zoology (see ICZN 1994c).

Type species: *Somatodessanctus* Schönherr, 1823, by original designation. Note: name suppressed for the Principle of Priority and placed on the Official Index of Rejected and Invalid Specific Names in Zoology (see ICZN 1994c).

Family-group name: SOMATODIDES Schönherr, 1823. Stem: *Somatod*-. Note: family-group name permanently invalid (ICZN 1999a, Article 39); name placed on the Official Index of Rejected and Invalid Family-group Names in Zoology (ICZN 1994c).

***Somatodes* Schönherr, 1840a**: 800. Current status: valid genus in CURCULIONIDAE: CYCLOMINAE: CYCLOMINI. Note: the senior homonym *Somatodes* Schönherr, 1823, and all uses of *Somatodes* prior to the publication of *Somatodes* Schönherr, 1840, was suppressed for the purposes of both the Principle of Priority and Homonymy and placed on the Official Index of Rejected and Invalid Generic Names in Zoology while *Somatodes* Schönherr, 1840 was placed on the Official List of Generic Names in Zoology (ICZN 1994c).

Type species: *Somatodesmisumenus* Gyllenhal, 1840, by original designation (see ICZN 1994c). Note: name placed on the Official List of Specific Names in Zoology (ICZN 1994c, as type species fixed by monotypy).

Family-group name: SOMATODIDES Lacordaire, 1863. Stem: *Somatod*-. Note: junior homonym of SOMATODINI Schönherr, 1823 (type genus *Somatodes* Schönherr, 1823) in Coleoptera: CURCULIONIDAE; Schönherr’s name is permanently invalid (based on a suppressed type genus).

***Somotrichus* Seidlitz, 1887**: 7 [Gatt.]. Current status: valid genus in CARABIDAE: HARPALINAE: LEBIINI: PERICALINA.

Type species: *Carabuselevatus* Fabricius, 1792 [p. 162] (= *Lebiaunifasciata* Dejean, 1831), by monotypy.

Family-group name: SOMOTRICHINI Mateu, 1963. Stem: *Somotrich*-.

***Sonnetius* Casey, 1922**: 321. Current status: junior synonym of *Barymerus* Lacordaire, 1865 in CURCULIONIDAE: BARIDINAE: MADARINI: BARYMERINA.

Type species: *Sonnetiusbinarius* Casey, 1922, by **present designation**.

Family-group name: SONNETIINI Casey, 1922. Stem: *Sonneti*-.

***Sophrorhinus* Rouzet, 1855**: 80. Current status: valid genus in CURCULIONIDAE: CRYPTORHYNCHINAE: SOPHRORHININI.

Type species: *Sophrorhinusduvernoyi* Rouzet, 1855, by monotypy.

Family-group name: SOPHRORHINIDES Lacordaire, 1865. Stem: *Sophrorhin*-.

***Sosylopsis* Grouvelle, 1910a**: 269. Current status: valid genus in TEREDIDAE: TEREDINAE: SOSYLOPSINI.

Type species: *Sosylopsisgeayi* Grouvelle, 1910, by monotypy.

Family-group name: SOSYLOPSINI Dajoz, 1980. Stem: *Sosylops*-.

***Spalacopsis* Newman, 1842a**: 305. Current status: valid genus in CERAMBYCIDAE: LAMIINAE: AGAPANTHIINI.

Type species: *Spalacopsisstellio* Newman, 1842 (= *Hippopsisfilum* Klug, 1829), by subsequent designation (J. Thomson 1864: 95).

Family-group name: SPALACOPSIDES Lacordaire, 1872. Stem: *Spalacopse*-.

***Spanglerogyrus* Folkerts, 1979**: 2. Current status: valid genus in GYRINIDAE: SPANGLEROGYRINAE.

Type species: *Spanglerogyrusalbiventris* Folkerts, 1979, by original designation.

Family-group name: SPANGLEROGYRINAE Folkerts, 1979. Stem: *Spanglerogyr*-.

***Spaniophaenus* Reitter, 1875b**: 4, 8. Current status: valid genus in CRYPTOPHAGIDAE: CRYPTOPHAGINAE: CRYPTOPHAGINI.

Type species: *Cryptophagusamplicollis* Brisout de Barneville, 1866 (= *Cryptophaguslapidarius* Fairmaire, 1862), by subsequent designation (Otero and Diaz Pazos 1995: 183). Note: Otero and Diaz Pazos (1995: 183) designated *Cryptophaguslapidarius* as type species, which is not an originally included species; however, as they listed it in synonymy with *Cryptophagusamplicollis*, one of the two originally included species, they are deemed to have designated the latter species as type species (ICZN 1999a, Article 69.2.2); *Cryptophaguslaticollis* L. Miller, 1858 (= *Catopochrotidestermitophilus* Kieseritzky & Reikhardt, 1936) is often listed as type species of *Spaniophaenus*, by subsequent designation of Leschen (1996: 601) but Otero and Diaz Pazos’ designation is earlier; both species are currently included in the genus *Spaniophaenus* Reitter.

Family-group name: SPANIOPHAENI Casey, 1900. Stem: *Spaniophaen*-.

***Sparedrus* Dejean, 1821**: 72. Current status: valid genus in OEDEMERIDAE: CALOPODINAE.

Type species: *Calopustestaceus* Andersch, 1797, by monotypy.

Family-group name: SPAREDRIIDAE Gistel, 1848. Stem: *Sparedr*-. Note: nomen oblitum (see Bouchard et al. 2011: 435).

***Sparostes* Putzeys, 1867**: 27. Current status: valid genus in CARABIDAE: SCARITINAE: CLIVININI: SPAROSTESINA.

Type species: *Sparostesbrevicollis* Putzeys, 1867, by subsequent designation (Andrewes 1929: 344).

Family-group name: SPAROSTESINA Dostal, 2017: 117. Stem: *Sparostes*-.

***Sparrmannia* Laporte, 1840b**: 132. Current status: valid genus in SCARABAEIDAE: MELOLONTHINAE: TANYPROCTINI: TANYPROCTINA. Note: nomen protectum (see Bouchard et al. 2011: 255); the original spelling was *Sparmannia* but the unjustified emendation *Sparrmannia*, introduced by von Dalla Torre (1913: 291), is in prevailing usage, attributed to Laporte (1840b), and therefore is a justified emendation and the correct original spelling (ICZN 1999a, Article 33.2.3.1).

Type species: *Melolonthaalopex* Fabricius, 1787, by subsequent designation (Evans 1989: 14).

Family-group name: SPARRMANNINI Péringuey, 1904. Stem: *Sparrmanni*-.

***Spastica* Guérin-Méneville, 1844a**: 137. Current status: valid genus in MELOIDAE: ELETICINAE: SPASTICINI: SPASTICINA. Note: this name was first proposed as a synonym of *Gnathium* W. Kirby, 1819 and treated as an available name before 1961 (e.g., Lacordaire 1859b: 679), *Spastica* was therefore made available from its first publication as a synonym (ICZN 1999a, Article 11.6.1).

Type species: *Gnathiumflavicolle* Guérin-Méneville, 1834, by monotypy.

Family-group name: SPASTICINA Kaszab, 1959. Stem: *Spastic*-.

***Spelaeobates* J. Müller, 1901**: 16, 28. Current status: valid genus in LEIODIDAE: CHOLEVINAE: LEPTODIRINI: SPELAEOBATINA.

Type species: *Spelaeobatesnovaki* J. Müller, 1901, by subsequent designation (Jeannel 1910: 25).

Family-group name: SPELAEOBATINA Guéorguiev, 1974. Stem: *Spelaeobat*-.

***Speleobama* Park, 1951**: 52. Current status: valid genus in STAPHYLINIDAE: PSELAPHINAE: GONIACERITAE: SPELEOBAMINI.

Type species: *Speleobamavana* Park, 1951, by original designation.

Family-group name: SPELEOBAMINI Park, 1951. Stem: *Speleobam*-.

***Spercheus* Kugelann in Illiger, 1798**: 241. Current status: valid genus in SPERCHEIDAE.

Type species: *Dytiscusemarginatus* Schaller, 1783, by subsequent designation (Latreille 1810: 428).

Family-group name: SPERCHEINI Erichson, 1837. Stem: *Sperche*-.

***Sperchopsis* J.L. LeConte, 1861**: 47. Current status: valid genus in HYDROPHILIDAE: HYDROPHILINAE: HYDROBIUSINI.

Type species: *Spercheustesselatus* Ziegler, 1844, by original designation.

Family-group name: SPERCHOPSINI Hansen, 1991. Stem: *Sperchops*-.

***Spermophagus* Schönherr, 1833**: 102. Current status: valid genus in CHRYSOMELIDAE: BRUCHINAE: AMBLYCERINI: SPERMOPHAGINA.

Type species: *Spermophagustitivilitius* Boheman, 1833, by original designation.

Family-group name: SPERMOPHAGINI Borowiec, 1987. Stem: *Spermophag*-.

***Sphadasmus* Schönherr, 1844**: 290. Current status: valid genus in CURCULIONIDAE: CONODERINAE: SPHADASMINI. Note: new replacement name for *Cyrtomon* Schönherr, 1836.

Type species: *Cyrtomoncamelus* Gyllenhal, 1836, by original designation (type fixation for *Cyrtomon* Schönherr).

Family-group name: SPHADASMIDES Lacordaire, 1865. Stem: *Sphadasm*-.

***Sphaenelater* O. Schwarz, 1902**: 365. Current status: valid genus in ELATERIDAE: LISSOMINAE: PROTELATERINI.

Type species: *Sphaenelaternigricornis* O. Schwarz, 1902, by monotypy.

Family-group name: SPHAENELATERINI Stibick, 1979. Stem: *Sphaenelater*-.

***Sphaenothecus* Dupont, 1836**: pls 141–164 [p. 2]. Current status: valid genus in CERAMBYCIDAE: CERAMBYCINAE: TRACHYDERINI: TRACHYDERINA.

Type species: *Sphaenothecustomentosus* Dupont, 1838 (= *Sphaenothecustrilineatus* Dupont, 1838), by subsequent designation (J. Thomson 1864: 205). Note: no species were originally included in this genus by Dupont (1836); the first ones were the four species listed by Dupont (1838: 56–59).

Family-group name: SPHAENOTHECITAE J. Thomson, 1861. Stem: *Sphaenothec*-.

***Sphaeratrix* Gistel, 1848b**: 190. Current status: valid genus in CHRYSOMELIDAE: CHRYSOMELINAE: CHRYSOMELINI.

Type species: *Sphaeratrixlatifrons* Gistel, 1848, by monotypy.

Family-group name: SPHAERATRIXINI Daccordi, 1981. Stem: *Sphaeratrix*-.

***Sphaericus* Wollaston, 1854**: 263. Current status: valid genus in PTINIDAE: PTININAE: SPHAERICINI.

Type species: *Ptinusdawsoni* Wollaston, 1854, by subsequent designation (Bellés 1991: 24).

Family-group name: SPHAERICINI Portevin, 1931. Stem: *Sphaeric*-.

***Sphaeridium* Fabricius, 1775**: 66. Current status: valid genus in HYDROPHILIDAE: SPHAERIDIINAE: SPHAERIDIINI.

Type species: *Dermestesscarabaeoides* Linnaeus, 1758, by subsequent designation (Latreille 1810: 428).

Family-group name: SPHAERIDIOTA Latreille, 1802. Stem: *Sphaeridi*-. Note: senior homonym of SPHAERIDIINI Richards, 1968 (type genus *Sphaeridia* Linnaniemi, 1912) in Collembola.

***Sphaerion* Audinet-Serville, 1834b**: 68. Current status: valid genus in CERAMBYCIDAE: CERAMBYCINAE: ELAPHIDIINI.

Type species: *Elaphidioncyanipennis* Audinet-Serville, 1834, by original designation.

Family-group name: SPHÉRIONIDES Lacordaire, 1868. Stem: *Sphaeri*-. Note: junior homonym of SPHAERIIDAE Deshayes, 1855 (type genus *Sphaerium* Scopoli, 1877) in Mollusca.

***Sphaeriopoeus* Kuschel, 2003a**: 59. Current status: valid genus in CURCULIONIDAE: CURCULIONINAE: SPHAERIOPOEINI.

Type species: *Sphaeriopoeusfaber* Kuschel, 2003, by original designation.

Family-group name: SPHAERIOPOEINI Kuschel, 2003. Stem: *Sphaeriopoe*-.

***Sphaerites* Duftschmid, 1805**: 205. Current status: valid genus in SPHAERITIDAE.

Type species: *Histerglabratus* Fabricius, 1792, by monotypy.

Family-group name: SPHAERITIDAE Shuckard, 1839. Stem: *Sphaerit*-.

***Sphaerius* Waltl, 1838**: 272. Current status: valid genus in SPHAERIUSIDAE. Note: name suppressed for the purposes of both the Principle of Priority and Homonymy and placed on the Official Index of Rejected and Invalid Generic Names in Zoology (ICZN 1985d) but subsequently placed on the Official List of Generic Names in Zoology (ICZN 2000).

Type species: *Sphaeriusacaroides* Waltl, 1838, by monotypy (see ICZN 2000). Note: name placed on the Official List of Specific Names in Zoology (ICZN 2000).

Family-group name: SPHAERINA Erichson, 1845. Stem: *Sphaerius*- (ICZN 2000).

***Sphaerocharis* Lacordaire, 1848**: 634. Current status: valid genus in CHRYSOMELIDAE: LAMPROSOMATINAE: SPHAEROCHARINI.

Type species: *Lamprosomamarginicolle* Guérin-Méneville, 1844, by subsequent designation (Achard 1914b: 21).

Family-group name: SPHOEROCHARIDES Chapuis, 1874. Stem: *Sphaerochar*-.

***Sphaeronum* Sharp, 1876b**: 224. Current status: valid genus in STAPHYLINIDAE: PAEDERINAE: LATHROBIINI: SPHAERONINA.

Type species: *Sphaeronumpallidum* Sharp, 1876, by subsequent designation (Casey 1905: 55).

Family-group name: SPHAERONIA Casey, 1905. Stem: *Sphaeron*-.

***Sphaeronychus* Dejean, 1836a**: 383. Current status: valid genus in CHRYSOMELIDAE: GALERUCINAE: ALTICINI. Note: the original spelling was *Sphraeronychus* but *Sphaeronychus*, an incorrect subsequent spelling, is prevailing usage, attributed to the publication of the original spelling, and therefore deemed to be the correct original spelling (ICZN 1999a, Article 33.3.1).

Type species: *Alticamelanura* G.-A. Olivier, 1808, by monotypy.

Family-group name: SPHAERONYCHINI Bechyné & Špringlová de Bechyné, 1960. Stem: *Sphaeronych*-.

***Sphaerosoma* Stephens, 1832a**: 391. Current status: valid genus in ALEXIIDAE. Note: this genus-group name is often credited to “Samouelle, 1819” in the literature; however, we use the corrected name and author proposed by Löbl and Smetana (2011: 31).

Type species: *Sphaerosomaquercus* Stephens, 1832 (= *Tritomapilosum* Panzer, 1793), by monotypy. Note: we use the data published by Löbl and Smetana (2011: 31) regarding the author and year of the type species as well as its valid name.

Family-group name: SPHAEROSOMINAE Ganglbauer, 1899. Stem: *Sphaerosomat*-.

***Sphaerotrypes* W.F.H. Blandford, 1894**: 61. Current status: valid genus in CURCULIONIDAE: SCOLYTINAE: DIAMERINI.

Type species: *Sphaerotrypespila* W.F.H. Blandford, 1894, by subsequent designation (Hopkins 1914: 129).

Family-group name: SPHAEROTRYPINI Murayama, 1963. Stem: *Sphaerotryp*-.

***Sphallotrichus* Fragoso, 1982**: 145. Current status: valid genus in CERAMBYCIDAE: CERAMBYCINAE: CERAMBYCINI: SPHALLOTRICHINA.

Type species: *Cerambyxsetosus* Germar, 1823, by original designation.

Family-group name: SPHALLOTRICHINA Martins & Monné, 2005. Stem: *Sphallotrich*-.

***Sphenocorynes* Schönherr, 1838**: 866. Current status: valid genus in CURCULIONIDAE: DRYOPHTHORINAE: SPHENOPHORINI: SPHENOCORYNINA.

Type species: *Curculioquadripunctatus* Weber, 1801 (= *Rhynchophoruscinereus* Illiger, 1800), by original designation.

Family-group name: SPHÉNOCORYNIDES Lacordaire, 1865. Stem: *Sphenocoryn*-.

***Sphenophorus* Schönherr, 1838**: 874. Current status: valid genus in CURCULIONIDAE: DRYOPHTHORINAE: SPHENOPHORINI: SPHENOPHORINA. Note: name placed on the Official List of Generic Names in Zoology (ICZN 1959b).

Type species: *Curculioabbreviatus* Fabricius, 1787, by original designation (see ICZN 1959b). Note: name validated by the Commission (i.e., not a primary homonym of *Curculioabbreviates* Linnaeus, 1758) and placed on the Official List of Specific Names in Zoology (ICZN 1959b).

Family-group name: SPHÉNOPHORIDES Lacordaire, 1865. Stem: *Sphenophor*-.

***Sphenoptera* Dejean, 1833a**: 81. Current status: valid genus in BUPRESTIDAE: CHRYSOCHROINAE: SPHENOPTERINI.

Type species: *Buprestisantiqua* Illiger, 1803, by subsequent designation (Volkovitsh and Kalashian 2002: 166).

Family-group name: SPHÉNOPTÉRIDES Lacordaire, 1857. Stem: *Sphenopter*-.

***Sphinctovalgus* Kolbe, 1904**: 51. Current status: valid genus in SCARABAEIDAE: CETONIINAE: VALGINI: VALGINA.

Type species: *Sphinctovalgusconradti* Kolbe, 1904, by monotypy.

Family-group name: SPHINCTOVALGINAE Kolbe, 1904. Stem: *Sphinctovalg*-.

***Sphindiphorus* Sen Gupta & Crowson, 1979**: 184. Current status: valid genus in SPHINDIDAE: SPHINDIPHORINAE.

Type species: *Sphindiphorusnatalensis* Sen Gupta & Crowson, 1979, by original designation.

Family-group name: SPHINDIPHORINI Sen Gupta & Crowson, 1979. Stem: *Sphindiphor*-.

***Sphindocis* Fall, 1917**: 170. Current status: valid genus in TETRATOMIDAE: SPHINDOCIINAE.

Type species: *Sphindocisdenticollis* Fall, 1917, by monotypy.

Family-group name: SPHINDOCIINAE Lawrence, 1974. Stem: *Sphindoci*-.

***Sphindus* Dejean, 1821**: 102. Current status: valid genus in SPHINDIDAE: SPHINDINAE. name placed (as *Sphindus* Megerle in Dejean, 1821) on the Official List of Generic Names in Zoology (ICZN 1997).

Type species: *Nitiduladubia* Gyllenhal, 1808, by monotypy (see ICZN 1997). Note: name placed on the Official List of Specific Names in Zoology (ICZN 1997).

Family-group name: SPHINDIDES Jacquelin du Val, 1860. Stem: *Sphind*-.

***Sphingnotus* Perroud, 1855b**: 410. Current status: valid genus in CERAMBYCIDAE: LAMIINAE: TMESISTERNINI.

Type species: *Tmesisternusmirabilis* Boisduval, 1835, by subsequent designation (J. Thomson 1864: 31).

Family-group name: SPINGNOTHITAE J. Thomson, 1864. Stem: *Sphingnot*-.

***Sphodrosomus* Perroud & Montrouzier, 1865**: 58. Current status: valid genus in CARABIDAE: HARPALINAE: PTEROSTICHINI: ABACOMORPHINA.

Type species: *Sphodrosomusseisseti* Perroud & Montrouzier, 1865, by monotypy.

Family-group name: SPHODROSOMINI Tschitschérine, 1902. Stem: *Sphodrosom*-.

***Sphodrus* Clairville, 1806**: 84. Current status: valid genus in CARABIDAE: HARPALINAE: SPHODRINI: SPHODRINA.

Type species: *Carabusplanus* Fabricius, 1792 (= *Carabusleucophthalmus* Linnaeus, 1758), by monotypy.

Family-group name: SPHODRIDAE Laporte, 1834. Stem: *Sphodr*-.

***Sphoerodes* Chaudoir, 1883**: 519. Current status: valid genus in CARABIDAE: HARPALINAE: OODINI.

Type species: *Oodesstriatus* Dejean, 1831, by monotypy.

Family-group name: SPHAERODINI Jeannel, 1949. Stem: *Sphoerod*-.

***Sphondylia* Weise, 1902**: 120. Current status: valid genus in MEGALOPODIDAE: MEGALOPODINAE: SPHONDYLIINI.

Type species: *Sphondyliamagnicollis* Weise, 1902, by subsequent designation (Jacoby and Clavareau 1905: 7).

Family-group name: SPHONDYLIINI Rodríguez-Mirón in Rodríguez-Mirón et al., 2021: 694. Stem: *Sphondyli*-.

***Sphuridaethes* Pace, 1988**: 980. Current status: valid genus in STAPHYLINIDAE: ALEOCHARINAE: COROTOCINI: SPHURIDAETHINA.

Type species: *Sphuridaethesloebli* Pace, 1988, by original designation.

Family-group name: SPHURIDAETHINA Pace, 1988. Stem: *Sphuridaeth*-.

***Spilophora* Boheman, 1850**: 107. Current status: valid genus in CHRYSOMELIDAE: CASSIDINAE: SPILOPHORINI.

Type species: *Calyptocephalatrigemina* Guérin-Méneville, 1844, by subsequent designation (Monrós and Viana 1951: 389).

Family-group name: SPILOPHORITES Chapuis, 1875. Stem: *Spilophor*-. Note: senior homonym of SPILOPHORINA Krikken, 1984 (type genus *Spilophorus* Westwood, 1848) in Coleoptera: SCARABAEIDAE.

***Spilophorus* Schaum, 1848**: 61. Current status: valid genus in SCARABAEIDAE: CETONIINAE: CREMASTOCHEILINI: SPILOPHORINA.

Type species: *Cremastocheilusmaculatus* Gory & Percheron, 1833, by subsequent designation (Kraatz 1899: 63).

Family-group name: SPILOPHORINA Krikken, 1984. Stem: *Spilophor*-. Note: junior homonym of SPILOPHORINI Chapuis, 1875 (type genus *Spilophora* Boheman, 1850) in Coleoptera: CHRYSOMELIDAE.

***Spilopyra* Baly, 1860**: 24. Current status: valid genus in CHRYSOMELIDAE: SPILOPYRINAE.

Type species: *Spilopyrasumptuosa* Baly, 1860, by original designation.

Family-group name: SPILOPYRITES Chapuis, 1874. Stem: *Spilopyr*-.

***Spintheria* J. Thomson, 1861**: 357. Current status: valid genus in CERAMBYCIDAE: CERAMBYCINAE: SPINTHERIINI.

Type species: *Tmesisternusgratiosus* Pascoe, 1856, by monotypy.

Family-group name: SPINTHÉRIIDES Lacordaire, 1869. Stem: *Spintheri*-.

***Spodochlamys* Burmeister, 1855**: 528. Current status: valid genus in SCARABAEIDAE: RUTELINAE: ANATISTINI.

Type species: *Spodochlamyscaesarea* Burmeister, 1855, by monotypy.

Family-group name: SPODOCHLAMYDINI Ohaus, 1918. Stem: *Spodochlamyd*-.

***Spondylis* Fabricius, 1775**: 159. Current status: valid genus in CERAMBYCIDAE: SPONDYLIDINAE: SPONDYLIDINI.

Type species: *Attelabusbuprestoides* Linnaeus, 1758, by subsequent designation (Latreille 1810: 431).

Family-group name: SPONDYLII Audinet-Serville, 1832. Stem: *Spondylid*-.

***Spongocerus* W.F.H. Blandford, 1893**: 431. Current status: junior synonym of *Scolytoplatypus* C. Schaufuss, 1891 in CURCULIONIDAE: SCOLYTINAE: SCOLYTOPLATYPODINI.

Type species: *Scolytoplatypustycon* W.F.H. Blandford, 1893, by subsequent designation (Hopkins 1914: 129).

Family-group name: SPONGOCERINAE Hagedorn, 1909. Stem: *Spongocer*-.

***Spurius* Kaup, 1871**: 75. Current status: valid genus in PASSALIDAE: PASSALINAE: PROCULINI.

Type species: *Passalusbicornis* Truqui, 1857, by monotypy.

Family-group name: SPURIINAE Kuwert, 1896. Stem: *Spuri*-.

***Stagobius* Schiødte, 1848**: 78. Current status: junior synonym of *Leptodirus* F.J. Schmidt, 1832 in LEIODIDAE: CHOLEVINAE: LEPTODIRINI: LEPTODIRINA.

Type species: *Stagobiustroglodytes* Schiødte, 1848 (= *Leptodirushochenwartii* F.J. Schmidt, 1832), by monotypy.

Family-group name: STAGOBIINAE Schiødte, 1849. Stem: *Stagobi*-.

***Staminodeus* N.M. Franz, 2002**: 413. Current status: valid genus in CURCULIONIDAE: CURCULIONINAE: DERELOMINI: STAMINODEINA.

Type species: *Staminodeusvectoris* N.M. Franz, 2002, by original designation.

Family-group name: STAMINODEINA N.M. Franz, 2006. Stem: *Staminode*-.

***Stanus* Ślipiński, Tomaszewska & Lawrence, 2009**: 423. Current status: valid genus in CORYLOPHIDAE: CORYLOPHINAE: STANINI.

Type species: *Stanusbowesteadi* Ślipiński, Tomaszewska & Lawrence, 2009, by original designation.

Family-group name: STANINI Robertson, Ślipiński & McHugh in Robertson et al., 2012: 229. Stem: *Stan*-.

***Staphylinus* Linnaeus, 1758**: 342, 421. Current status: valid genus in STAPHYLINIDAE: STAPHYLININAE: STAPHYLININI: STAPHYLININA. Note: name placed on the Official List of Generic Names in Zoology (ICZN 1959a).

Type species: *Staphylinuserythropterus* Linnaeus, 1758, by plenary power (see ICZN 1959a). Note: the species name was originally spelled *erytropterus* but it was modified to *erythropterus* by the Commission and placed on the Official List of Specific Names in Zoology (ICZN 1959a).

Family-group name: STAPHYLINIAE Latreille, 1802. Stem: *Staphylin*-.

***Statira* Lepeletier & Audinet-Serville, 1828**: 479. Current status: valid genus in TENEBRIONIDAE: LAGRIINAE: LAGRIINI: STATIRINA.

Type species: *Statiraagroides* Lepeletier & Audinet-Serville, 1828, by subsequent designation (Blanchard in Audouin et al. 1844: pl. 53bis).

Family-group name: STATYRITES Blanchard, 1845. Stem: *Statir*-.

***Steatoderus* Dejean, 1833a**: 94. Current status: junior synonym of *Elater* Linnaeus, 1758 in ELATERIDAE: ELATERINAE: ELATERINI.

Type species: *Elaterferrugineus* Linnaeus, 1758, by monotypy.

Family-group name: STEATODERIDAE Gistel, 1848. Stem: *Steatoder*-.

***Stenaesthetus* Sharp, 1874a**: 79. Current status: valid genus in STAPHYLINIDAE: EUAESTHETINAE: STENAESTHETINI.

Type species: *Stenaesthetussunioides* Sharp, 1874, by monotypy.

Family-group name: STENAESTHETINI Bernhauer & Schubert, 1911. Stem: *Stenaesthet*-.

***Stenalia* Mulsant, 1856c**: 83. Current status: valid genus in MORDELLIDAE: MORDELLINAE: STENALIINI.

Type species: *Mordellatestacea* Fabricius, 1787, by subsequent designation (J.L. LeConte 1859b: 18).

Family-group name: STENALIINI Franciscolo, 1955. Stem: *Stenali*-.

***Stenapion* Wagner, 1912b**: 20. Current status: valid genus in BRENTIDAE: APIONINAE: APIONITAE: APIONINI: STENAPIINA.

Type species: *Apionconstricticolle* Sharp, 1890, by subsequent designation (Kissinger 1968: 29).

Family-group name: STENAPIINA Poinar & Legalov, 2015: 154. Stem: *Stenapi*-.

***Stenaspidius* Westwood, 1848b**: 366. Current status: valid genus in BOLBOCERATIDAE: BOLBOCERATINAE: STENASPIDIINI.

Type species: *Bolbocerasnigricornis* Westwood, 1848, by monotypy.

Family-group name: STENASPIDIINI Nikolajev, 2003. Stem: *Stenaspidi*-.

***Stenaspis* Audinet-Serville, 1834b**: 51. Current status: valid genus in CERAMBYCIDAE: CERAMBYCINAE: TRACHYDERINI: TRACHYDERINA.

Type species: *Stenaspisverticalis* Audinet-Serville, 1834, by monotypy.

Family-group name: STÉNASPIDES Lacordaire, 1868. Stem: *Stenaspid*-.

***Stenelmis* Dufour, 1835**: 158. Current status: valid genus in ELMIDAE: ELMINAE: ELMINI: STENELMINA.

Type species: *Limniuscanaliculatus* Gyllenhal, 1808, by subsequent designation (C.G. Thomson 1859: 21).

Family-group name: STENELMISATES Mulsant & Rey, 1872. Stem: *Stenelm*-.

***Stenhomalus* A. White, 1855**: 243. Current status: valid genus in CERAMBYCIDAE: CERAMBYCINAE: STENHOMALINI.

Type species: *Stenhomalusfenestratus* A. White, 1855, by monotypy.

Family-group name: STENHOMALINI Miroshnikov, 1989. Stem: *Stenhomal*-.

***Stenichnus* C.G. Thomson, 1859**: 61. Current status: valid genus in STAPHYLINIDAE: SCYDMAENINAE: SCYDMAENITAE: STENICHNINI.

Type species: *Scydmaenusexilis* Erichson, 1837 (= *Scydmaenusbicolor* Denny, 1825), by original designation.

Family-group name: STENICHNINI Fauvel, 1885. Stem: *Stenichn*-.

***Stenobia* Lacordaire, 1872**: 442. Current status: valid genus in CERAMBYCIDAE: LAMIINAE: STENOBIINI.

Type species: *Stenobiapradieri* Lacordaire, 1872, by monotypy.

Family-group name: STENOBIINI Breuning, 1950. Stem: *Stenobi*-.

***Stenocerus* Schönherr, 1826**: 39. Current status: valid genus in ANTHRIBIDAE: ANTHRIBINAE: STENOCERINI.

Type species: *Anthribusfulvitarsis* Germar, 1823, by original designation.

Family-group name: STENOCERINARUM Kolbe, 1895. Stem: *Stenocer*-.

***Stenochia* W. Kirby, 1819**: 423. Current status: junior synonym of *Strongylium* W. Kirby, 1819 in TENEBRIONIDAE: STENOCHIINAE: STENOCHIINI.

Type species: *Stenochiarufipes* W. Kirby, 1819, by subsequent designation (Hope 1841a: 133).

Family-group name: STENOCHIADAE W. Kirby, 1837. Stem: *Stenochi*-.

***Stenocorus* Geoffroy, 1762**: 221. Current status: valid genus in CERAMBYCIDAE: LEPTURINAE: STENOCORINI. Note: name placed on the Official List of Generic Names in Zoology (ICZN 1994a).

Type species: *Lepturameridiana* Linnaeus, 1758, by plenary power (see ICZN 1994a). Note: name placed on the Official List of Specific Names in Zoology (ICZN 1994a).

Family-group name: STENOCORITAE J. Thomson, 1861. Stem: *Stenocor*-.

***Stenocorynus* Schönherr, 1823**: 1142. Current status: valid genus in CURCULIONIDAE: ENTIMINAE: ENTIMITAE: BYRSOPAGINI: STRANGALIODINA.

Type species: *Curculiocrenulatus* Fabricius, 1775, by original designation.

Family-group name: STENOCORYNINI McKeown, 1939. Stem: *Stenocoryn*-.

***Stenocyphon* Lawrence, 2001**: 357. Current status: valid genus in SCIRTIDAE: STENOCYPHONINAE.

Type species: *Stenocyphonsasajii* Lawrence, 2001, by original designation.

Family-group name: STENOCYPHONINAE Lawrence & Yoshitomi, 2007. Stem: *Stenocyphon*-.

***Stenodera* Eschscholtz, 1818**: 469. Current status: valid genus in MELOIDAE: NEMOGNATHINAE: STENODERINI. Note: *Stenoderasexpunctata* Eschscholtz, 1818 was placed on the Official List of Generic Names in Zoology (ICZN 2020a).

Type species: *Stenoderasexpunctata* Eschscholtz, 1818 (= *Meloecaucasica* Pallas, 1782), by monotypy (see ICZN 2020a). Note: name placed on the Official List of Specific Names in Zoology (ICZN 2020a).

Family-group name: STENODERINI Selander, 1991. Stem: *Stenoder*-.

***Stenoderus* Dejean, 1821**: 112. Current status: valid genus in CERAMBYCIDAE: CERAMBYCINAE: RHAGIOMORPHINI. Note: name placed on the Official List of Generic Names in Zoology (ICZN 2020a).

Type species: *Cerambyxabbreviatus* Fabricius, 1801 (= *Stenocorussuturalis* G.-A. Olivier, 1800), by monotypy (see ICZN 2020a). Note: *Cerambyxabbreviatus* Fabricius, 1801 placed on the Official List of Specific Names in Zoology (ICZN 2020a).

Family-group name: STENODERINAE Pascoe, 1867. Stem: *Stenoderus*- (see ICZN 2020a).

***Stenodontes* Audinet-Serville, 1832**: 173. Current status: valid genus in CERAMBYCIDAE: PRIONINAE: MACROTOMINI: MALLODONINA.

Type species: *Prionusexsertus* G.-A. Olivier, 1795, by subsequent designation (E. Desmarest 1860: 307).

Family-group name: STENODONTINI Lameere, 1903. Stem: *Stenodont*-. Note: homonym of STENODONTINA Schmiedeknecht, 1903 (type genus *Stenodontus* Berthoumieu, 1897) in Hymenoptera.

***Stenolamus* Gebien, 1920**: 107. Current status: valid genus in TENEBRIONIDAE: BLAPTINAE: OPATRINI: STENOLAMINA.

Type species: *Stenolamussulciceps* Gebien, 1920, by subsequent designation (Gebien 1938b: 397).

Family-group name: STENOLAMINA C. Koch, 1956. Stem: *Stenolam*-.

***Stenolophus* Dejean, 1821**: 15. Current status: valid genus in CARABIDAE: HARPALINAE: HARPALINI: STENOLOPHINA.

Type species: **here fixed** (under ICZN 1999a, Article 70.3.2) as *Carabusteutonus* Schrank, 1781, misidentified as *Carabusvaporariorum* Linnaeus, 1758 (sensu Fabricius, 1787) in the subsequent designation by Westwood (1838c: 5).

Family-group name: STENOLOPHIDAE W. Kirby, 1837. Stem: *Stenoloph*-.

***Stenomela* Erichson, 1847c**: 159. Current status: valid genus in CHRYSOMELIDAE: SPILOPYRINAE.

Type species: *Stenomelapallida* Erichson, 1847, by monotypy.

Family-group name: STENOMÈLITES Chapuis, 1874. Stem: *Stenomel*-.

***Stenomorphus* Dejean, 1831**: 696. Current status: valid genus in CARABIDAE: HARPALINAE: HARPALINI: HARPALINA.

Type species: *Stenomorphusangustatus* Dejean, 1831, by monotypy.

Family-group name: STENOMORPHIDAE Laporte, 1834. Stem: *Stenomorph*-.

***Stenopelmus* Schönherr, 1835**: 468. Current status: valid genus in CURCULIONIDAE: BRACHYCERINAE: TANYSPHYRINI.

Type species: *Stenopelmusrufinasus* Gyllenhal, 1835, by original designation.

Family-group name: STENOPELMI J.L. LeConte, 1876. Stem: *Stenopelm*-.

***Stenophanes* Solsky, 1876**: 294. Current status: valid genus in TENEBRIONIDAE: STENOCHIINAE: CNODALONINI.

Type species: *Hedyphanesmesostenus* Solsky, 1871, by original designation.

Family-group name: STENOPHANINI Reitter, 1922. Stem: *Stenophan*-.

***Stenopodius* G.H. Horn, 1884**: 301. Current status: valid genus in CHRYSOMELIDAE: CASSIDINAE: CHALEPINI.

Type species: *Stenopodiusflavidus* G.H. Horn, 1884, by monotypy.

Family-group name: STENOPODIIDES G.H. Horn, 1884. Stem: *Stenopodi*-. Note: nomen oblitum (see Bouchard and Bousquet 2020a: 108).

***Stenopterus* Illiger, 1804**: 120. Current status: valid genus in CERAMBYCIDAE: CERAMBYCINAE: STENOPTERINI.

Type species: *Necydalisrufa* Linnaeus, 1767, by monotypy.

Family-group name: STENOPTERIDAE Gistel, 1848. Stem: *Stenopter*-.

***Stenorhis* Jordan, 1928**: 167. Current status: valid genus in ANTHRIBIDAE: CHORAGINAE: STENORHININI.

Type species: *Stenorhisampedus* Jordan, 1928, by original designation.

Family-group name: STENORHININI B.D. Valentine in Trýzna and B.D. Valentine, 2011: 65. Stem: *Stenorhin*-.

***Stenoria* Mulsant, 1857a**: 186. Current status: valid genus in MELOIDAE: NEMOGNATHINAE: NEMOGNATHINI: SITARINA.

Type species: *Sitarisapicalis* Latreille, 1804, by monotypy.

Family-group name: STENORIIDES J.B. Parker & Böving, 1924. Stem: *Stenori*-.

***Stenoscelis* Wollaston, 1861b**: 141. Current status: valid genus in CURCULIONIDAE: COSSONINAE: RHYNCOLINI: STENOSCELIDINA.

Type species: *Stenoscelishylastoides* Wollaston, 1861, by monotypy.

Family-group name: STENOSCELIDES Wollaston, 1877. Stem: *Stenoscelid*-.

***Stenosis* Herbst, 1799**: 160. Current status: valid genus in TENEBRIONIDAE: PIMELIINAE: STENOSINI: STENOSINA.

Type species: fixed by Bouchard et al. (2021: 351) according to Article 70.3.2 (ICZN 1999a) as *Tageniaintermedia* Solier, 1838, the taxonomic species involved in the misidentification of *Pimeliaangustata* Fabricius, 1775 (sensu Herbst, 1799), as designated subsequently by Lacordaire (1859a: 102).

Family-group name: STENOSIDAE Schaum, 1859. Stem: *Stenos*-.

***Stenosphenus* Haldeman, 1847**: 39. Current status: valid genus in CERAMBYCIDAE: CERAMBYCINAE: ELAPHIDIINI.

Type species: *Callidiumnotatum* G.-A. Olivier, 1800, by monotypy.

Family-group name: STENOSPHENINI J.L. LeConte, 1873. Stem: *Stenosphen*-.

***Stenostoma* Latreille, 1810**: 217. Current status: valid genus in OEDEMERIDAE: OEDEMERINAE: STENOSTOMATINI.

Type species: *Lepturarostrata* Fabricius, 1787, by monotypy.

Family-group name: STÉNOSTOMATES Mulsant, 1858. Stem: *Stenostomat*-.

***Stenotarsia* Burmeister, 1842**: 590. Current status: valid genus in SCARABAEIDAE: CETONIINAE: STENOTARSIINI: STENOTARSIINA.

Type species: *Cetoniavelutina* Gory & Percheron, 1835, by subsequent designation (Sakai and Nagai 1998: 179). Note: this is a junior primary homonym of *Cetoniavelutina* G.-A. Olivier, 1789 [South Africa], which was also treated as a member of *Cetonia* by Gory and Percheron (1833: 61); Burmeister (1842: 368) placed *Cetoniavelutina* G.-A. Olivier, 1789 in his new genus *Gametis*, and it was subsequently treated by Boheman (1857: 19) as a synonym of *Gametissubfasciatus* (Swederus, 1787) [now *Gametoidessubfasciata* (Swederus, 1787)]; the primary homonym *Cetoniavelutina* Gory & Percheron, 1835 [Madagascar] should be considered permanently invalid unless conditions outlined in Article 57.2 (ICZN 1999a) are met.

Family-group name: STENOTARSIDEN Kraatz, 1880. Stem: *Stenotarsi*-.

***Stenotarsus* Perty, 1832**: 112. Current status: valid genus in ENDOMYCHIDAE: ENDOMYCHINAE.

Type species: *Stenotarsusbrevicollis* Perty, 1832, by monotypy.

Family-group name: STÉNOTARSITES Chapuis, 1876. Stem: *Stenotars*-.

***Stenotrachelus* Berthold, 1827**: 371. Current status: valid genus in STENOTRACHELIDAE: STENOTRACHELINAE.

Type species: *Dryopsaeneus* Paykull, 1799, by subsequent monotypy (Lepeletier and Audinet- Serville 1828: 488). Note: although *Stenotrachelusaeneus* (Paykull, 1799) is used as a valid name in recent literature (e.g., Löbl 2020: 499), Paykull (1799: 152) clearly refers to *Dryopsaenea* (Fabricius 1792b: 75), who in turn refers to his *Lagriaaenea* (Fabricius 1787: 94); more research is needed to confirm if *Dryopsaeneus* Paykull, 1799 should indeed be used as the correct type species for this genus-group name or if a misidentification was involved.

Family-group name: STENOTRACHELIDAE C.G. Thomson, 1859. Stem: *Stenotrachel*-.

***Stenus* Latreille, 1797**: 77. Current status: valid genus in STAPHYLINIDAE: STENINAE.

Type species: *Staphylinusjuno* Paykull, 1789, by subsequent monotypy (Paykull 1800: 433).

Family-group name: STENIDAE W.S. MacLeay, 1825. Stem: *Sten*-.

***Stephanocephalus* Kaup, 1868b**: 19. Current status: valid genus in PASSALIDAE: PASSALINAE: SOLENOCYCLINI.

Type species: *Passalushostilis* Percheron, 1841, by monotypy.

Family-group name: STEPHANOCEPHALEAE Kaup, 1871. Stem: *Stephanocephal*-.

***Stephanorhynchus* A. White, 1846**: 17. Current status: valid genus in CURCULIONIDAE: CURCULIONINAE: EUGNOMINI: EUGNOMINA.

Type species: *Stephanorhynchuscurvipes* A. White, 1846, by monotypy.

Family-group name: STEPHANORHYNCHINA Voss, 1936. Stem: *Stephanorhynch*-.

***Stephanorrhina* Burmeister, 1842**: 208. Current status: valid genus in SCARABAEIDAE: CETONIINAE: GOLIATHINI: RHOMBORHININA.

Type species: *Cetoniaguttata* G.-A. Olivier, 1789, by monotypy.

Family-group name: STEPHANORRHININA Schenkling, 1921. Stem: *Stephanorrhin*-.

***Stereocorynes* Wollaston, 1873b**: 500, 588. Current status: valid genus in CURCULIONIDAE: COSSONINAE: RHYNCOLINI: STENOSCELIDINA.

Type species: *Cossonustruncorum* Germar, 1823, by monotypy.

Family-group name: STEREOCORYNINA Voss, 1955. Stem: *Stereocoryn*-.

***Stereodermus* Lacordaire, 1865**: 419. Current status: valid genus in BRENTIDAE: BRENTINAE: TRACHELIZINI: STEREODERMINA.

Type species: *Arrenodespygmaeus* Schönherr, 1833, by original designation.

Family-group name: STEREODERMINA Sharp, 1895. Stem: *Stereoderm*-.

***Stereolus* Rafinesque, 1815**: 112. Current status: junior synonym of *Trixagus* Kugelann, 1794 in THROSCIDAE. Note: unnecessary new replacement name for *Throscus* Latreille, 1797.

Type species: *Elaterdermestoides* Linnaeus, 1767, by subsequent designation (Curtis 1827: pl. 163) (type fixation for *Throscus* Latreille).

Family-group name: STEREOLIA Rafinesque, 1815. Stem: *Stereol*-. Note: nomen oblitum (see Bouchard et al. 2011: 306).

***Stereomera* Arrow, 1905**: 534. Current status: valid genus in SCARABAEIDAE: APHODIINAE: STEREOMERINI.

Type species: *Stereomerapusilla* Arrow, 1905, by monotypy.

Family-group name: STEREOMERINI Howden & Storey, 1992. Stem: *Stereomer*-.

***Sternacanthus* Audinet-Serville, 1832**: 172. Current status: valid genus in CERAMBYCIDAE: CERAMBYCINAE: TRACHYDERINI: TRACHYDERINA.

Type species: *Prionusundatus* G.-A. Olivier, 1795, by monotypy.

Family-group name: STERNACANTHITAE J. Thomson, 1864. Stem: *Sternacanth*-.

***Sternechosomus* Voss, 1958**: 46. Current status: valid genus in CURCULIONIDAE: MOLYTINAE: METATYGINI.

Type species: *Sternechosomusoctotuberculatus* Voss, 1958, by original designation.

Family-group name: STERNECHOSOMINI Voss, 1958. Stem: *Sternechosom*-.

***Sternechus* Schönherr, 1826**: 251. Current status: valid genus in CURCULIONIDAE: MOLYTINAE: STERNECHINI.

Type species: *Orobitistrachyptomus* Germar, 1823, by original designation.

Family-group name: STERNÉCHIDES Lacordaire, 1863. Stem: *Sternech*-.

***Sternocera* Eschscholtz, 1829b**: 8. Current status: valid genus in BUPRESTIDAE: JULODINAE. Note: this name is a nomen protectum and its senior synonym *Sternoxus* Billberg, 1820 is a nomen oblitum (Bílý 2001: 148).

Type species: *Buprestischrysis* Fabricius, 1775, by subsequent designation (E. Desmarest 1860: 39).

Family-group name: STERNOCERINI Csiki, 1904. Stem: *Sternocer*-.

***Sternodea* Reitter, 1875b**: 78. Current status: valid genus in CRYPTOPHAGIDAE: CRYPTOPHAGINAE: CAENOSCELINI.

Type species: *Sternodeabaudii* Reitter, 1875, by monotypy.

Family-group name: STERNODEINI Casey, 1900. Stem: *Sternode*-.

***Sternopriscus* Sharp, 1880**: cxlviii. Current status: valid genus in DYTISCIDAE: HYDROPORINAE: HYDROPORINI: STERNOPRISCINA.

Type species: *Hydroporusmultimaculatus* Clark, 1862, by monotypy.

Family-group name: STERNOPRISCINI Branden, 1884. Stem: *Sternoprisc*-.

***Sternotomis* Percheron in Guérin-Méneville and Percheron, 1836**: pl. 16. Current status: valid genus in CERAMBYCIDAE: LAMIINAE: STERNOTOMINI.

Type species: *Sternotomisaper* Percheron, 1836 (= *Lamiaducalis* Klug, 1835), by subsequent designation (J. Thomson 1868: 176). Note: J. Thomson (1868: 176) selected *Lamiaducalis* Klug, 1835 as type species, which was not an originally included nominal species, but since he listed it in synonymy with *Sternotomisaper* Percheron, 1836, an originally included nominal species, he is deemed to have designated the latter species as type species (ICZN 1999a, Article 69.2.2).

Family-group name: STERNOTOMITAE J. Thomson, 1860. Stem: *Sternotom*-.

***Steropes* Steven, 1806**: 166. Current status: valid genus in ANTHICIDAE: STEROPINAE.

Type species: *Steropescaspius* Stevens, 1806, by monotypy.

Family-group name: STÉROPITES Jacquelin du Val, 1863. Stem: *Sterop*-. Note: junior homonym of STEROPINAE Dana, 1854 (type genus *Sterope* Goodsir, 1845) in Crustacea.

***Stethaspis* Hope, 1837**: 71, 104. Current status: valid genus in SCARABAEIDAE: MELOLONTHINAE: COLYMBOMORPHINI. Note: name placed on the Official List of Generic Names in Zoology (ICZN 1983b).

Type species: *Melolonthasuturalis* Fabricius, 1775, by plenary power (see ICZN 1983b). Note: name placed on the Official List of Specific Names in Zoology (ICZN 1983b).

Family-group name: STETHASPIDIDAE Burmeister, 1855. Stem: *Stethaspid*-.

***Stethodesma* Hope in Bainbridge, 1841**: 482. Current status: valid genus in SCARABAEIDAE: CETONIINAE: GYMNETINI: GYMNETINA.

Type species: *Stethodesmastrachani* Hope, 1841, by monotypy.

Family-group name: STETHODESMAE Schoch, 1894. Stem: *Stethodesmat*-.

***Stethorus* Weise, 1885a**: 65. Current status: valid genus in COCCINELLIDAE: COCCINELLINAE: COCCIDULINI.

Type species: *Coccinellaminima* Rossi, 1794 (= *Scymnuspusillus* Herbst, 1797), by subsequent designation (Korschefsky 1931: 111). Note: Korschefsky (1931: 111) selected *Stethoruspunctillum* Weise, 1891 as type species, not an originally included species, but since he listed it in synonymy with *Coccinellaminima* Rossi, 1794, an originally included species, he is deemed to have designated the latter species as type species (ICZN 1999a, Article 69.2.2).

Family-group name: STETHORINI Dobzhansky, 1924. Stem: *Stethor*-.

***Sthereus* Motschulsky, 1845c**: 373. Current status: valid genus in CURCULIONIDAE: MOLYTINAE: STHEREINI.

Type species: *Sthereusquadrituberculatus* Motschulsky, 1845, by subsequent designation (Buchanan 1936: 178).

Family-group name: STHEREINI Hatch, 1971. Stem: *Sthere*-.

***Sticholotis* Crotch, 1874a**: 200. Current status: valid genus in COCCINELLIDAE: COCCINELLINAE: STICHOLOTIDINI.

Type species: *Sticholotissubstriatus* Crotch, 1874, by original designation.

Family-group name: STICHOLOTINI Weise, 1901. Stem: *Sticholotid*-.

***Stictocema* Jacoby, 1906**: 33. Current status: valid genus in CHRYSOMELIDAE: GALERUCINAE: METACYCLINI.

Type species: *Stictocemafasciata* Jacoby, 1906, by subsequent designation (Wilcox 1971: 169).

Family-group name: STICTOCEMITES Laboissière, 1922. Stem: *Stictocem*-.

***Stictocranius* J.L. LeConte, 1867**: 374. Current status: valid genus in STAPHYLINIDAE: EUAESTHETINAE: STICTOCRANIINI.

Type species: *Stictocraniuspuncticeps* J.L. LeConte, 1867, by monotypy.

Family-group name: STICTOCRANIINI Jakobson, 1914. Stem: *Stictocrani*-.

***Stigmatrachelus* Schönherr, 1840b**: 123. Current status: valid genus in CURCULIONIDAE: ENTIMINAE: POLYDRUSITAE: DERMATODINI.

Type species: *Curculiocinctus* G.-A. Olivier, 1807, by original designation.

Family-group name: STIGMATRACHELINI Richard, 1983. Stem: *Stigmatrachel*-.

***Stigmodera* Eschscholtz, 1829b**: 9. Current status: valid genus in BUPRESTIDAE: BUPRESTINAE: STIGMODERINI.

Type species: *Buprestismacularia* Donovan, 1805, by subsequent designation (Solier 1833: 293).

Family-group name: STIGMODÉRIDES Lacordaire, 1857. Stem: *Stigmoder*-.

***Stilbus* Seidlitz, 1872**: 35 [Gatt.]. Current status: valid genus in PHALACRIDAE: PHALACRINAE. Note: new replacement name for *Olistherus* Seidlitz, 1872.

Type species: *Anisotomatestacea* Panzer, 1797, by subsequent designation (Guillebeau 1894: 283) (type fixation for *Eustilbus* Sharp, 1888, an unnecessary new replacement name for *Stilbus* Seidlitz).

Family-group name: STILBINI Jakobson, 1915. Stem: *Stilb*-.

***Stilicopsis* Sachse, 1852**: 144. Current status: valid genus in STAPHYLINIDAE: PAEDERINAE: LATHROBIINI: STILICOPSINA.

Type species: *Stilicopsisparadoxa* Sachse, 1852, by monotypy.

Family-group name: STILICOPSES Casey, 1905. Stem: *Stilicops*-.

***Stilicus* Berthold, 1827**: 331. Current status: junior synonym of *Rugilus* Leach, 1819 in STAPHYLINIDAE: PAEDERINAE: LATHROBIINI: STILICINA.

Type species: *Staphylinusorbiculatus* Paykull, 1789, by subsequent designation (Blanchard in Audouin et al. 1842: pl. 27).

Family-group name: STILICI Casey, 1905. Stem: *Stilic*-.

***Stilipalpus* Jeannel, 1951b**: 73. Current status: valid genus in STAPHYLINIDAE: PSELAPHINAE: BATRISITAE: BATRISINI: STILIPALPINA.

Type species: *Stilipalpusclavipes* Jeannel, 1951, by original designation.

Family-group name: STILIPALPINA Jeannel, 1954. Stem: *Stilipalp*-.

***Stilpnonotus* G.R. Gray in Griffith and Pidgeon, 1831**: pls 48, 61. Current status: valid genus in MYCTERIDAE: EURYPINAE.

Type species: *Stilpnonotuseurypiformis* G.R. Gray, 1831, by monotypy.

Family-group name: STILPNONOTINAE Borchmann, 1936. Stem: *Stilpnonot*-.

***Stizopus* Erichson, 1843b**: 245. Current status: valid genus in TENEBRIONIDAE: BLAPTINAE: OPATRINI: STIZOPODINA.

Type species: *Stizopuslaticollis* Erichson, 1843, by monotypy.

Family-group name: STIZOPIDES Lacordaire, 1859. Stem: *Stizopod*-.

***Stolas* Billberg, 1820**: 58. Current status: valid genus in CHRYSOMELIDAE: CASSIDINAE: MESOMPHALIINI.

Type species: *Cassidadiscors* Fabricius, 1801 (= *Cassidadiscoides* Linnaeus, 1758), by subsequent designation (Barber and Bridwell 1940: 9).

Family-group name: STOLAINI Hincks, 1952. Stem: *Stolad*-.

***Stolius* Lewis, 1895a**: 433. Current status: valid genus in STENOTRACHELIDAE: STOLIINAE.

Type species: *Stoliusvagepictus* Lewis, 1895, by monotypy.

Family-group name: STOLIINAE Nikitsky, 1985. Stem: *Stoli*-.

***Stomis* Clairville, 1806**: 46. Current status: valid genus in CARABIDAE: HARPALINAE: PTEROSTICHINI: PTEROSTICHINA.

Type species: *Carabuspumicatus* Panzer, 1795, by monotypy.

Family-group name: STOMIDES Gené, 1839. Stem: *Stomid*-.

***Storeus* Schönherr, 1843**: 293. Current status: valid genus in CURCULIONIDAE: CURCULIONINAE: STOREINI.

Type species: *Storeusvariegatus* Boheman, 1843, by original designation.

Family-group name: STORÉIDES Lacordaire, 1863. Stem: *Store*-.

***Storthodontus* Chaudoir, 1855**: 23. Current status: valid genus in CARABIDAE: SCARITINAE: SCARITINI: STORTHODONTINA. Note: Chaudoir originally used two different spellings, including *Horthodontus* (p. 4); Chaudoir (1879: 128, 176) used *Storthodontus* and therefore acted as First Reviser (ICZN 1999a, Article 24.2.4).

Type species: *Storthodontusnimrod* Chaudoir, 1855, by subsequent designation (Motschulsky 1857b: 94).

Family-group name: STORTHODONTINI Jeannel, 1946. Stem: *Storthodont*-.

***Strangaliodes* Schönherr, 1842a**: 219. Current status: valid genus in CURCULIONIDAE: ENTIMINAE: ENTIMITAE: BYRSOPAGINI: STRANGALIODINA.

Type species: *Strangaliodesalbosquamosus* Boheman, 1842, by original designation.

Family-group name: STRANGALIODIDES Lacordaire, 1863. Stem: *Strangaliod*-.

***Strategus* W. Kirby in W. Kirby and Spence, 1828b**: 349, 644. Current status: valid genus in SCARABAEIDAE: DYNASTINAE: ORYCTINI.

Type species: *Scarabaeusaloeus* Linnaeus, 1758, by subsequent designation (Hope 1837: 87).

Family-group name: STRATEGIDAE Burmeister, 1847. Stem: *Strateg*-.

***Streptocerus* Fairmaire, 1850**: 53. Current status: valid genus in LUCANIDAE: LAMPRIMINAE: STREPTOCERINI.

Type species: *Streptocerusspeciosus* Fairmaire, 1850, by monotypy.

Family-group name: STREPTOCERINI Kikuta, 1986. Stem: *Streptocer*-.

***Striatoquasimus* Schimmel & Tarnawski, 2009**: 28, 105. Current status: valid genus in ELATERIDAE: NEGASTRIINAE: QUASIMUSINI: STRIATOQUASINA.

Type species: *Striatoquasimusdolini* Schimmel & Tarnawski, 2009, by original designation.

Family-group name: STRIATOQUASINA Schimmel & Tarnawski, 2009. Stem: *Striatoquas*- (ICZN 1999a, Article 29.4).

***Strigota* Casey, 1910**: 176. Current status: valid genus in STAPHYLINIDAE: ALEOCHARINAE: ATHETINI: ATHETINA.

Type species: *Strigotaoppidana* Casey, 1910 (= *Homalotaambigua* Erichson, 1839), by original designation (according to Casey’s first species rule, stipulated on page 90).

Family-group name: STRIGOTAE Casey, 1910. Stem: *Strigot*-.

***Strombophorus* Hagedorn, 1909**: 740. Current status: valid genus in CURCULIONIDAE: SCOLYTINAE: DIAMERINI.

Type species: *Strombophoruscrenatus* Hagedorn, 1909, by subsequent designation (Hopkins 1914: 130).

Family-group name: STROMBOPHORINI Schedl, 1959. Stem: *Strombophor*-.

***Stromboscerus* Schönherr, 1838**: 814. Current status: valid genus in CURCULIONIDAE: DRYOPHTHORINAE: STROMBOSCERINI.

Type species: *Stromboscerusschuppeli* Gyllenhal, 1838, by original designation.

Family-group name: STROMBOSCÉRIDES Lacordaire, 1865. Stem: *Stromboscer*-.

***Strongylium* W. Kirby, 1819**: 417. Current status: valid genus in TENEBRIONIDAE: STENOCHIINAE: STENOCHIINI. Note: nomen protectum (Bouchard et al. 2005: 501).

Type species: *Strongyliumchalconatum* W. Kirby, 1819, by monotypy.

Family-group name: STRONGYLIIDES Lacordaire, 1859. Stem: *Strongyli*-.

***Strongylopterus* Schönherr, 1837**: 473. Current status: valid genus in CURCULIONIDAE: CRYPTORHYNCHINAE: PSEPHOLACINI.

Type species: *Strongylopterusovatus* Boheman, 1837, by original designation.

Family-group name: STRONGYLOPTÉRIDES Lacordaire, 1865. Stem: *Strongylopter*-.

***Strongylurus* Hope, 1834a**: 107. Current status: valid genus in CERAMBYCIDAE: CERAMBYCINAE: STRONGYLURINI.

Type species: *Strongylurusscutellatus* Hope, 1834, by monotypy.

Family-group name: STRONGYLURIDES Lacordaire, 1868. Stem: *Strongylur*-.

***Strongylus* Herbst, 1792**: 179. Current status: invalid senior synonym of *Cyllodes* Erichson, 1843 in NITIDULIDAE: NITIDULINAE: CYLLODINI. Note: junior homonym of *Strongylus* O.F. Müller, 1780 [Nematoda].

Type species [suggested]: *Strongylusater* Herbst, 1792, by subsequent designation (Jelínek and Audisio 2003: 166). Note: the valid type species fixation is that of Curtis (1831: pl. 339), who selected *Nitidulastrigata* Fabricius, 1787, but that is the type species of *Cryptarcha* Shuckard, 1840; an application to the Commission is needed to retain *Strongylusater* Herbst, 1792 as the type species of *Strongylus* Herbst, 1792.

Family-group name: STRONGYLINAE Erichson, 1843. Stem: *Strongyl*-. Note: family-group name permanently invalid (ICZN 1999a, Article 39).

***Strophosoma* Billberg, 1820**: 44. Current status: valid genus in CURCULIONIDAE: ENTIMINAE: POLYDRUSITAE: BRACHYDERINI. Note: we consider the name *Strophosomum* Gistel, 1856 as an incorrect subsequent spelling of *Strophosoma* Billberg, notwithstanding the status given by Alonso-Zarazaga and Lyal (1999: 147), who consider the name an unnecessary new replacement name for Billberg’s name.

Type species: *Curculiocoryli* Fabricius, 1775 (= *Curculiomelanogrammus* Forster, 1771), by subsequent designation (Pierce 1913: 411).

Family-group name: STROPHOSOMIDAE Gistel, 1848. Stem: *Strophosomat*-.

***Styanax* Pascoe, 1871b**: 164. Current status: valid genus in CURCULIONIDAE: MOLYTINAE: STYANACINI.

Type species: *Styanaxcarbonarius* Pascoe, 1871, by monotypy.

Family-group name: STYANACINAE Chûjô & Voss, 1960. Stem: *Styanac*-.

***Stylopodus* Benick, 1917**: 190. Current status: junior synonym of *Megalopinus* Eichelbaum, 1915 in STAPHYLINIDAE: MEGALOPSIDIINAE.

Type species: *Megalopscephalotes* Erichson, 1840, by original designation.

Family-group name: STYLOPODINAE Blackwelder, 1943. Stem: *Stylopod*-.

***Stylosomus* Suffrian, 1847**: 8. Current status: valid genus in CHRYSOMELIDAE: CRYPTOCEPHALINAE: CRYPTOCEPHALINI: STYLOSOMINA.

Type species [suggested]: *Cryptocephalustamaricis* Herrich-Schäffer, 1838, by subsequent designation (Gressitt and Kimoto 1961: 109). Note: Suffrian (1847: 8) originally included a single nominal species (p. 10) in his new genus, *Cryptocephalusminutissimus* Germar, 1823, but that species is currently placed in the subgenus Stylomicrus Petitpierre & Alonso-Zarazaga, 2000, not the nominotypical subgenus; subsequently, Suffrian (1848: 146) described the genus in length and included three species, including *C.tamaricis*; an application to the Commission is necessary to fix *Cryptocephalustamaricis* Herrich- Schäffer, 1838 as the type species of *Stylosomus* Suffrian, 1847.

Family-group name: STYLOSOMITES Chapuis, 1874. Stem: *Stylosom*-.

***Styphlomerus* Chaudoir in Putzeys, 1875**: iv. Current status: valid genus in CARABIDAE: BRACHININAE: BRACHININI: APTININA.

Type species: *Brachinusquadrimaculatus* Dejean, 1831, by subsequent designation (Andrewes 1938: 138).

Family-group name: STYPHLOMERINI Habu, 1984. Stem: *Styphlomer*-.

***Styphlus* Schönherr, 1826**: 258. Current status: valid genus in CURCULIONIDAE: CURCULIONINAE: STYPHLINI.

Type species: *Styphluspenicillus* Schönherr, 1826, by original designation.

Family-group name: STYPHLIDAE Jekel, 1861. Stem: *Styphl*-.

***Subcoccinella* Agassiz, 1846**: 156. Current status: valid genus in COCCINELLIDAE: COCCINELLINAE: EPILACHNINI. Note: this name was first introduced under the vernacular name “Subcoccinelle” by Huber (1842: 376) and therefore it is not available from that publication; this genus-group name has been treated as a junior objective synonym of *Lasia* Hope, 1841 (e.g., Tomaszewska and Szawaryn 2016: 68), which is a junior homonym of *Lasia* Wiedemann, 1824 [Diptera]; however, we could not find evidence that Agassiz (1846: 156) proposed *Subcoccinella* as a new replacement name for *Lasia* Hope.

Type species: *Coccinellaglobosa* D.H. Schneider, 1792 (= *Coccinellavigintiquatuorpunctata* Linnaeus, 1758), by original designation (type fixation for *Lasia* Hope).

Family-group name: SUBCOCCINELLINI Jakobson, 1915. Stem: *Subcoccinell*-.

***Subprotelater* Fleutiaux, 1916**: 387. Current status: valid genus in ELATERIDAE: SUBPROTELATERINAE.

Type species: *Subprotelaterbakeri* Fleutiaux, 1916, by monotypy.

Family-group name: SUBPROTELATERINAE Fleutiaux, 1920. Stem: *Subprotelater*-.

***Sueus* Murayama, 1951**: 1. Current status: valid genus in CURCULIONIDAE: SCOLYTINAE: HYORRHYNCHINI.

Type species: *Sueussphaerotrypoides* Murayama, 1951 (= *Hyorrhynchusniisimai* Eggers, 1926), by original designation.

Family-group name: SUEINAE Murayama, 1959. Stem: *Sue*-.

***Sugimotoa* Habu, 1975**: 77. Current status: valid genus in CARABIDAE: HARPALINAE: LEBIINI: SUGIMOTOINA.

Type species: *Sugimotoaparallela* Habu, 1975 (= *Pediomorphusanthracoides* Straneo, 1967), by original designation.

Family-group name: SUGIMOTOINA Habu, 1975. Stem: *Sugimoto*-.

***Sukunahikona* Kamiya, 1960**: 22. Current status: junior synonym of *Scymnomorphus* Weise, 1897 in COCCINELLIDAE: MICROWEISEINAE: SUKUNAHIKONINI.

Type species: *Sukunahikonajaponica* Kamiya, 1960 (= *Alexiajaponica* Reitter, 1889), by original designation.

Family-group name: SUKUNAHIKONINI Kamiya, 1960. Stem: *Sukunahikon*-.

***Sumnius* Weise, 1892**: 29. Current status: valid genus in COCCINELLIDAE: COCCINELLINAE: COCCIDULINI.

Type species: *Sumniuscardoni* Weise, 1892, by subsequent designation (Korschefsky 1931: 94).

Family-group name: SUMNIINI Hoang, 1982. Stem: *Sumni*-.

***Suniops* Voss, 1929a**: 362. Current status: valid genus in ATTELABIDAE: ATTELABINAE: EUOPINI. Note: new replacement name for *Synechops* Voss, 1924.

Type species: *Euopssemicuprea* Voss, 1924, by subsequent designation (Legalov 2003: 365) (type fixation for *Synechops* Voss).

Family-group name: SUNIOPSINA Legalov, 2003. Stem: *Suniops*- (ICZN 1999a, Article 29.4).

***Suphis* Aubé, 1836**: 42. Current status: valid genus in NOTERIDAE: NOTERINAE: NOTERINI.

Type species: *Suphiscimicoides* Aubé, 1837, by subsequent monotypy (Aubé 1837: 207).

Family-group name: SUPHISINI Sharp, 1880. Stem: *Suphid*-.

***Svetlanaebyctiscus* Legalov, 2001a**: 341. Current status: valid genus in ATTELABIDAE: RHYNCHITINAE: RHYNCHITITAE: BYCTISCINI: SVETLANAEBYCTISCINA.

Type species: *Coenorhinusvitis* Ter-Minassian, 1959, by original designation.

Family-group name: SVETLANAEBYCTISCINA Legalov, 2003. Stem: *Svetlanaebyctisc*-.

***Sybaris* Stephens, 1832b**: 70. Current status: valid genus in MELOIDAE: MELOINAE: LYTTINI.

Type species: *Sybarisimmunis* Stephens, 1832, by monotypy.

Family-group name: SYBARIDES Wellman, 1910. Stem: *Sybare*-.

***Sydax* Lacordaire, 1868**: 335. Current status: valid genus in CERAMBYCIDAE: CERAMBYCINAE: SYDACINI.

Type species: *Sydaxstramineus* Lacordaire, 1868, by original designation.

Family-group name: SYDACINI Martins in Martins et al., 2014: 381. Stem: *Sydac*-.

***Syllitus* Pascoe, 1859a**: 24. Current status: valid genus in CERAMBYCIDAE: CERAMBYCINAE: RHAGIOMORPHINI. Note: name placed on the Official List of Generic Names in Zoology (ICZN 2020a).

Type species: *Stenoderusgrammicus* Newman, 1840, by subsequent designation (McKeown 1947: 73; see ICZN 2020a). Note: name placed on the Official List of Specific Names in Zoology (ICZN 2020a).

Family-group name: SYLLITAE J. Thomson, 1864. Stem: *Syllit*-.

***Symbiotes* L. Redtenbacher, 1847b**: 198. Current status: valid genus in ANAMORPHIDAE.

Type species: *Symbioteslatus* L. Redtenbacher, 1847, by monotypy.

Family-group name: SYMBIOTINAE Joy, 1932. Stem: *Symbiot*-.

***Symmathetes* Schönherr, 1847**: 31. Current status: junior synonym of *Pantomorus* Schönherr, 1840 in CURCULIONIDAE: ENTIMINAE: POLYDRUSITAE: NAUPACTINI: NAUPACTINA.

Type species: *Symmatheteskollari* Schönherr, 1847, by original designation.

Family-group name: SYMMATHETES J.L. LeConte, 1874. Stem: *Symmathet*-.

***Symmerus* Chapuis, 1865**: 42, 319. Current status: invalid senior synonym of *Chaetastus* Nunberg, 1953 in CURCULIONIDAE: PLATYPODINAE: TESSEROCERINI: TESSEROCERINA. Note: junior homonym of *Symmerus* Walker, 1848 [Diptera].

Type species: *Symmerustuberculatus* Chapuis, 1865, by monotypy.

Family-group name: SYMMERARIAE H. Strohmeyer, 1914. Stem: *Symmer*-. Note: family-group name permanently invalid (ICZN 1999a, Article 39).

***Symmixus* Bernhauer, 1915b**: 56. Current status: valid genus in STAPHYLINIDAE: TACHYPORINAE: TACHYPORINI: TACHYPORINA.

Type species: *Symmixussikkimensis* Bernhauer, 1915, by monotypy.

Family-group name: SYMMIXINI Bernhauer, 1915. Stem: *Symmix*-.

***Sympiezopus* Schönherr, 1837**: 707. Current status: valid genus in CURCULIONIDAE: CONODERINAE: CORYSSOPODINI.

Type species: *Sympiezopusaciculatus* Schönherr, 1837, by original designation.

Family-group name: SYMPIEZOPINORUM Faust, 1886. Stem: *Sympiezopod*-.

***Sympiezoscelus* G.R. Waterhouse, 1853**: 203. Current status: valid genus in CURCULIONIDAE: CRYPTORHYNCHINAE: PSEPHOLACINI.

Type species: *Sympiezoscelusspencii* G.R. Waterhouse, 1853, by monotypy.

Family-group name: SYMPIÉZOSCÉLIDES Lacordaire, 1865. Stem: *Sympiezoscel*-.

***Sympolemon* Wasmann, 1900b**: 262. Current status: valid genus in STAPHYLINIDAE: ALEOCHARINAE: PYGOSTENINI.

Type species: *Sympolemonanommatis* Wasmann, 1900, by monotypy.

Family-group name: SYMPOLEMONINI Fenyes, 1919. Stem: *Sympolemont*-.

***Synapion* Schilsky, 1902**: no 13. Current status: valid genus in BRENTIDAE: APIONINAE: APIONITAE: APIONINI: SYNAPIINA.

Type species: *Apionebeninum* W. Kirby, 1808, by original designation.

Family-group name: SYNAPIINA Alonso-Zarazaga, 1990. Stem: *Synapi*-.

***Synaptonyx* G.R. Waterhouse, 1853**: 187. Current status: valid genus in CURCULIONIDAE: ENTIMINAE: ENTIMITAE: BYRSOPAGINI: STRANGALIODINA.

Type species: *Synaptonyxovatus* G.R. Waterhouse, 1853, by monotypy.

Family-group name: SYNAPTONYCIDES Lacordaire, 1863. Stem: *Synaptonych*-.

***Synaptops* Jekel, 1860**: 221. Current status: valid genus in ATTELABIDAE: ATTELABINAE: EUOPINI.

Type species: *Euopsnietneri* Jekel, 1860 (= *Rhynchitessuffundens* Walker, 1859), by original designation.

Family-group name: SYNAPTOPSINA Legalov, 2003. Stem: *Synaptops*- (ICZN 1999a, Article 29.4).

***Synaptus* Eschscholtz, 1829a**: 32. Current status: valid genus in ELATERIDAE: ELATERINAE: SYNAPTINI.

Type species: *Elaterfiliformis* Fabricius, 1781, by subsequent designation (Lacordaire 1857: 215).

Family-group name: SYNAPTIDAE Gistel, 1856. Stem: *Synapt*-.

***Syncalypta* Dillwyn, 1829**: 25. Current status: junior synonym of *Chaetophora* W. Kirby, 1823 in BYRRHIDAE: SYNCALYPTINAE: SYNCALYPTINI.

Type species: *Byrrhusarenarius* Sturm, 1807 (= *Byrrhusspinosus* Rossi, 1794), by subsequent designation (Westwood 1838c: 21).

Family-group name: SYNCALYPTAIRES Mulsant & Rey, 1869. Stem: *Syncalypt*-.

***Synchita* Hellwig, 1792**: 401. Current status: valid genus in ZOPHERIDAE: COLYDIINAE: SYNCHITINI.

Type species: *Synchitajuglandis* Hellwig, 1792 (= *Elophorushumeralis* Fabricius, 1792), by subsequent designation (Blanchard in Audouin et al. 1843: pl. 62).

Family-group name: SYNCHITAE L. Redtenbacher, 1845. Stem: *Synchit*-.

***Synchroa* Newman, 1838**: 378. Current status: valid genus in SYNCHROIDAE.

Type species: *Synchroapunctata* Newman, 1838, by monotypy.

Family-group name: SYNCHROÏDES Lacordaire, 1859. Stem: *Synchro*-.

***Syncosmetus* Sharp, 1891**: 6. Current status: valid genus in CIIDAE: XYLOGRAPHELLINAE: SYNCOSMETINI.

Type species: *Syncosmetusjaponicus* Sharp, 1891, by monotypy.

Family-group name: SYNCOSMETINA Lopes-Andrade, 2008. Stem: *Syncosmet*-.

***Syndesus* W.S. MacLeay, 1819**: 96, 104. Current status: valid genus in LUCANIDAE: SYNDESINAE: SYNDESINI.

Type species: *Sinodendroncornutum* Fabricius, 1801, by monotypy.

Family-group name: SYNDESIDAE W.S. MacLeay, 1819. Stem: *Syndes*-.

***Syndicus* Motschulsky, 1852**: 502. Current status: valid genus in STAPHYLINIDAE: SCYDMAENINAE: SCYDMAENITAE: STENICHNINI.

Type species: *Syndicuspilicornis* Motschulsky, 1852, by monotypy.

Family-group name: SYNDICINI Csiki, 1919. Stem: *Syndic*-.

***Synechocera* Deyrolle, 1865**: 115. Current status: valid genus in BUPRESTIDAE: AGRILINAE: CORAEBINI: SYNECHOCERINA.

Type species: *Buprestisdeplana* Fabricius, 1801, by monotypy.

Family-group name: SYNECHOCERINA Majer, 2000. Stem: *Synechocer*-.

***Synercticus* Newman, 1842c**: 403. Current status: valid genus in BORIDAE: SYNERCTICINAE.

Type species: *Synercticusheteromerus* Newman, 1842, by monotypy.

Family-group name: SYNERCTICINAE Lawrence & Pollock, 1994. Stem: *Synerctic*-.

***Syneta* Dejean, 1835**: 359. Current status: valid genus in CHRYSOMELIDAE: SYNETINAE.

Type species: *Criocerisbetulae* Fabricius, 1792, by monotypy.

Family-group name: SYNETAE J.L. LeConte & G.H. Horn, 1883. Stem: *Synet*-.

***Synirmus* Bedel, 1883**: 61. Current status: junior synonym of *Tropiphorus* Schönherr, 1842 in CURCULIONIDAE: ENTIMINAE: ENTIMITAE: BYRSOPAGINI: TROPIPHORINA. Note: unnecessary new replacement name for *Tropiphorus* Schönherr, 1842.

Type species: *Curculiomercurialis* Fabricius, 1801 (= *Curculioelevatus* Herbst, 1795), by original designation (see ICZN 1988c) (type fixation for *Tropiphorus* Schönherr).

Family-group name: SYNIRMINI Bedel, 1883. Stem: *Synirm*-.

***Synochodaeus* Kolbe, 1907**: 27. Current status: valid genus in OCHODAEIDAE: CHAETOCANTHINAE: SYNOCHODAEINI.

Type species: *Synochodaeusmodestus* Kolbe, 1907, by monotypy.

Family-group name: SYNOCHODAEINI Scholtz, 1988. Stem: *Synochodae*-.

***Synoditulus* Reichensperger, 1924**: 212. Current status: valid genus in HISTERIDAE: HAETERIINAE: SYNODITULINI.

Type species: *Synoditesseparatus* Reichensperger, 1924, by monotypy.

Family-group name: SYNODITULINI Tishechkin, 2007. Stem: *Synoditul*-.

***Synonycha* Chevrolat in Dejean, 1836a**: 436. Current status: valid genus in COCCINELLIDAE: COCCINELLINAE: COCCINELLINI.

Type species: *Coccinellaversicolor* Fabricius, 1792 (= *Coccinellagrandis* Thunberg, 1781), by monotypy.

Family-group name: SYNONYCHINI Weise, 1885. Stem: *Synonych*-.

***Synophthalmus* Lacordaire, 1863**: 516. Current status: junior synonym of *Phytophilus* Schönherr, 1835 **stat. nov.** in CURCULIONIDAE: CONODERINAE: CORYSSOMERINI. Note: unnecessary new replacement name for *Phytophilus* Schönherr, 1835 since *Phytophilus*, as used by Guérin-Méneville (1838: 99), is an incorrect subsequent spelling for *Phitophilus* Guérin- Méneville, 1831 in TENEBRIONIDAE (see Bouchard et al. 2021: 538).

Type species: *Phytophiluscruciferus* Gyllenhal, 1835, by original designation (type fixation for *Phytophilus* Schönherr).

Family-group name: SYNOPHTHALMIDES Lacordaire, 1863. Stem: *Synophthalm*-.

***Syntelia* Westwood, 1864c**: 11. Current status: valid genus in SYNTELIIDAE.

Type species: *Synteliaindica* Westwood, 1864, by subsequent designation (Yuasa 1930: 256).

Family-group name: SYNTELIIDAE Lewis, 1882. Stem: *Synteli*-.

***Syntomium* Curtis, 1828**: pl. 228. Current status: valid genus in STAPHYLINIDAE: OXYTELINAE: SYNTOMIINI.

Type species: *Syntomiumnigroaeneum* Curtis, 1828 (= *Omaliumaeneum* P.W.J. Müller, 1821), by original designation.

Family-group name: SYNTOMIINAE Böving & Craighead, 1931. Stem: *Syntomi*-.

***Synuchus* Gyllenhal, 1810**: 77. Current status: valid genus in CARABIDAE: HARPALINAE: SPHODRINI: SYNUCHINA.

Type species: *Carabusvivalis* Illiger, 1798, by monotypy.

Family-group name: SYNUCHI Lindroth, 1956. Stem: *Synuch*-.

***Syphorbus* Pascoe, 1881**: 92. Current status: valid genus in CURCULIONIDAE: MOLYTINAE: HYLOBIINI: HYLOBIINA.

Type species: *Syphorbusturgidus* Pascoe, 1881, by monotypy.

Family-group name: SYPHORBINA G.A.K. Marshall, 1932. Stem: *Syphorb*-.

***Sysolus* Grouvelle, 1908**: 429. Current status: valid genus in TEREDIDAE: TEREDINAE: SYSOLINI.

Type species: *Sysolusantennatus* Grouvelle, 1908, by monotypy.

Family-group name: SYSOLINI Ślipiński & Pal, 1985. Stem: *Sysol*-.

***Systellopus* Sharp, 1877a**: 315. Current status: valid genus in SCARABAEIDAE: MELOLONTHINAE: SYSTELLOPINI.

Type species: *Systellopusobtusus* Sharp, 1877, by subsequent designation (Allsopp 1990: 204).

Family-group name: SYSTELLOPIDES Sharp, 1877. Stem: *Systellop*-.

***Systena* Chevrolat in Dejean, 1836a**: 390. Current status: valid genus in CHRYSOMELIDAE: GALERUCINAE: ALTICINI.

Type species: *Gallerucafrontalis* Fabricius, 1801, by subsequent designation (Monrós and Bechyné 1956: 1133).

Family-group name: SYSTENAE G.H. Horn, 1889. Stem: *Systen*-.

***Systenocerus* Weise in Heyden et al., 1883**: 93. Current status: junior synonym of *Platycerus* Geoffroy, 1762 in LUCANIDAE: LUCANINAE: PLATYCERINI.

Type species: *Scarabaeuscaraboides* Linnaeus, 1758, by subsequent designation (Didier and Séguy 1953: 169).

Family-group name: SYSTENOCERINI Portevin, 1931. Stem: *Systenocer*-.

***Systolosoma* Solier, 1849**: 241. Current status: valid genus in TRACHYPACHIDAE: TRACHYPACHINAE.

Type species: *Systolosomabreve* Solier, 1849, by original designation.

Family-group name: SYSTOLOSOMINI Erwin, 1991. Stem: *Systolosomat*-.

***Syzetoninus* Blackburn, 1891**: 339. Current status: valid genus in ADERIDAE.

Type species: *Syzetoninusmundus* Blackburn, 1891, by subsequent designation (Nardi 2007: 30).

Family-group name: SYZETONININI Báguena Corella, 1948. Stem: *Syzetonin*-.

***Tachinus* Gravenhorst, 1802**: 134. Current status: valid genus in STAPHYLINIDAE: TACHYPORINAE: TACHINUSINI. Note: name placed on the Official List of Generic Names in Zoology (ICZN 1993g).

Type species: *Staphylinusrufipes* Linnaeus, 1758, by subsequent designation (Latreille 1810: 427; see ICZN 1993g). Note: name placed on the Official List of Specific Names in Zoology (ICZN 1993g).

Family-group name: TACHINIDAE Fleming, 1821. Stem: *Tachinus*- (ICZN 1993g).

***Tachygonus* Guérin-Méneville, 1833a**: pl. 38. Current status: valid genus in CURCULIONIDAE: CURCULIONINAE: RHAMPHINI: TACHYGONINA. Note: the name *Tachygonus* was proposed simultaneously by both Schönherr (1833: 6, 311) and Guérin-Méneville (1833a: pl. 38) in June 1833 and recorded on 6 July 1833 in the ‘Bibliographie de la France’ (see Bousquet 2016: 234, 484); Alonso-Zarazaga and Lyal (1999: 82) acted as First Revisers (ICZN 1999a, Article 24.2.2) and selected Guérin-Méneville’s name to have precedence; we consider *Tachygonus* used by Schönherr, 1833 a subsequent usage of *Tachygonus* Guérin-Méneville, 1833.

Type species: *Tachygonushorridus* Guérin-Méneville, 1833, by monotypy.

Family-group name: TACHYGONIDES Lacordaire, 1865. Stem: *Tachygon*-.

***Tachyoryctidium* Jeannel & Paulian, 1945**: 58. Current status: junior synonym of *Euxestoxenus* Arrow, 1925 in EUXESTIDAE.

Type species: *Tachyoryctidiumchappuisi* Jeannel & Paulian, 1945, by original designation.

Family-group name: TACHYORYCTIDIINI Jeannel & Paulian, 1945. Stem: *Tachyoryctidi*-.

***Tachyporus* Gravenhorst, 1802**: 124. Current status: valid genus in STAPHYLINIDAE: TACHYPORINAE: TACHYPORINI: TACHYPORINA. Note: name placed on the Official List of Generic Names in Zoology (ICZN 1993g).

Type species: *Staphylinuschrysomelinus* Linnaeus, 1758, by subsequent designation (Latreille 1810: 427; see ICZN 1993g). Note: name placed on the Official List of Specific Names in Zoology (ICZN 1993g).

Family-group name: TACHYPORIDAE W.S. MacLeay, 1825. Stem: *Tachypor*-.

***Tachypterellus* Fall & Cockerell, 1907**: 214. Current status: valid subgenus of *Anthonomus* Germar, 1817 in CURCULIONIDAE: CURCULIONINAE: ANTHONOMINI. Note: new replacement name for *Tachypterus* Dietz, 1891.

Type species: *Anthonomusquadrigibbus* Say, 1832, by original designation (type fixation for *Tachypterus* Dietz).

Family-group name: TACHYPTERELLINA Voss, 1944. Stem: *Tachypterell*-.

***Tachys* Dejean, 1821**: 16. Current status: valid genus in CARABIDAE: TRECHINAE: TACHYINI: TACHYINA. Note: name placed on the Official List of Generic Names in Zoology (ICZN 1990c).

Type species: *Tachysscutellaris* Stephens, 1828, by plenary power (see ICZN 1990c). Note: name placed on the Official List of Specific Names in Zoology (ICZN 1990c).

Family-group name: TACHYAIRES Motschulsky, 1862. Stem: *Tachy*-.

***Tachyusa* Erichson, 1837**: 307. Current status: valid genus in STAPHYLINIDAE: ALEOCHARINAE: TACHYUSINI. Note: name placed on the Official List of Generic Names in Zoology (ICZN 1961b).

Type species: *Tachyusaconstricta* Erichson, 1837, by plenary power (see ICZN 1961b). Note: name placed on the Official List of Specific Names in Zoology (ICZN 1961b).

Family-group name: TACHYUSIDES C.G. Thomson, 1859. Stem: *Tachyus*-.

***Tadius* Pascoe, 1885**: 253. Current status: valid genus in CURCULIONIDAE: BRACHYCERINAE: ERIRHININI.

Type species: *Tadiuserirhinoides* Pascoe, 1885, by monotypy.

Family-group name: TADIINI Zimmerman, 1993. Stem: *Tadi*-.

***Taeniocerus* W.F.H. Blandford, 1893**: 437. Current status: junior synonym of *Scolytoplatypus* C. Schaufuss, 1891 in CURCULIONIDAE: SCOLYTINAE: SCOLYTOPLATYPODINI. Note: junior homonym of *Taeniocerus* Kaup, 1871 [Coleoptera: PASSALIDAE].

Type species: *Scolytoplatypusmikado* W.F.H. Blandford, 1893, by subsequent designation (Hopkins 1914: 130).

Family-group name: TAENIOCERINI W.F.H. Blandford, 1893. Stem: *Taeniocer*-. Note: family-group name permanently invalid (ICZN 1999a, Article 39).

***Taeniodera* Burmeister, 1842**: 325. Current status: valid genus in SCARABAEIDAE: CETONIINAE: TAENIODERINI: TAENIODERINA.

Type species: *Macronotamonacha* Gory & Percheron, 1834, by subsequent designation (Cazurro Ruiz 1897a: 585).

Family-group name: TAENIODERINA Mikšić, 1976. Stem: *Taenioder*-.

***Taenioncus* Kirejtshuk, 1984**: 192. Current status: valid genus in NITIDULIDAE: EPURAEINAE: TAENIONCINI.

Type species: *Carpophiluscylindricus* Murray, 1864, by original designation.

Family-group name: TAENIONCINI Kirejtshuk, 1998. Stem: *Taenionc*-.

***Taeniotes* Audinet-Serville, 1835a**: 90. Current status: valid genus in CERAMBYCIDAE: LAMIINAE: LAMIINI.

Type species: *Cerambyxpulverulentus* G.-A. Olivier, 1790, by subsequent designation (E. Desmarest 1860: 324).

Family-group name: TAENIOTITAE J. Thomson, 1864. Stem: *Taeniot*-.

***Tagenia* Latreille, 1802b**: 170. Current status: junior synonym of *Stenosis* Herbst, 1799 in TENEBRIONIDAE: PIMELIINAE: STENOSINI: STENOSINA.

Type species: *Tenebriofiliformis* Fabricius, 1792, by monotypy.

Family-group name: TAGÉNITES Solier, 1834. Stem: *Tageni*-.

†***Taimyraltica* Nadein in Nadein and Perkovsky, 2018**: 98. Current status: valid genus in CHRYSOMELIDAE: GALERUCINAE: †TAIMYRALTICINI. Note: Upper Cretaceous (Russia).

Type species: †*Taimyralticacalcarata* Nadein, 2018, by original designation.

Family-group name: †TAIMYRALTICINI Nadein in Nadein and Perkovsky 2017: 98. Stem: *Taimyraltic*-.

***Tainophthalmus* Desbrochers des Loges, 1873b**: 426. Current status: valid genus in CURCULIONIDAE: ENTIMINAE: POLYDRUSITAE: TANYMECINI: TAINOPHTHALMINA.

Type species: *Tainophthalmuscrotchi* Desbrochers des Loges, 1873, by monotypy.

Family-group name: TAINOPHTHALMIDAE Desbrochers des Loges, 1873. Stem: *Tainophthalm*-.

***Talanus* Jacquelin du Val, 1857a**: 156. Current status: valid genus in TENEBRIONIDAE: STENOCHIINAE: TALANINI.

Type species: *Talanuscribrarius* Jacquelin du Val, 1857, by monotypy.

Family-group name: TALANIDES Champion, 1887. Stem: *Talan*-.

†***Taldycupes* Rohdendorf, 1961**: 421. Current status: valid genus in †PERMOCUPEDIDAE: †TALDYCUPEDINAE. Note: Permian (Russia).

Type species: †*Taldycupeskhalfini* Rohdendorf, 1961, by original designation.

Family-group name: †TALDYCUPIDAE Rohdendorf, 1961. Stem: *Taldycuped*-.

***Tamotus* L.W. Schaufuss, 1872**: 248. Current status: valid genus in STAPHYLINIDAE: EUAESTHETINAE: EUAESTHETINI.

Type species: *Tamotusfemoratus* L.W. Schaufuss, 1874, by subsequent monotypy (Schaufuss 1874: 289).

Family-group name: TAMOTINI Coiffait, 1984. Stem: *Tamot*-.

***Tamulus* Gistel, 1848b**: ix, 127. Current status: junior synonym of *Malachius* Fabricius, 1775 in MELYRIDAE: MALACHIINAE: MALACHIINI. Note: unnecessary new replacement name for *Malachius* Fabricius, 1775.

Type species: *Cantharisaenea* Linnaeus, 1758, by subsequent designation (Latreille 1810: 426) (type fixation for *Malachius* Fabricius).

Family-group name: TAMULIDAE Gistel, 1848. Stem: *Tamul*-.

***Tanaos* Schönherr, 1826**: 63. Current status: valid genus in BRENTIDAE: APIONINAE: TANAITAE: TANAINI.

Type species: *Apionsanguineum* Thunberg, 1815, by original designation.

Family-group name: TANAONIDES Schönherr, 1839. Stem: *Tana*-.

***Tanygnathinus* Reitter, 1909**: 164. Current status: junior synonym of *Atanygnathus* Jakobson, 1909 in STAPHYLINIDAE: STAPHYLININAE: STAPHYLININI: TANYGNATHININA. Note: replacement name for *Tanygnathus* Erichson, 1839.

Type species: *Tanygnathusterminalis* Erichson, 1839, by monotypy (type fixation for *Tanygnathus* Erichson).

Family-group name: TANYGNATHININI Reitter, 1909. Stem: *Tanygnathin*-.

***Tanygnathus* Erichson, 1839a**: 417. Current status: invalid senior synonym of *Atanygnathus* Jakobson, 1909 in STAPHYLINIDAE: STAPHYLININAE: STAPHYLININI: TANYGNATHININA. Note: junior homonym of *Tanygnathus* Wagler, 1832 [Aves].

Type species: *Tanygnathusterminalis* Erichson, 1839, by monotypy.

Family-group name: TANYGNATHINI Casey, 1915. Stem: *Tanygnath*-. Note: family-group name permanently invalid (ICZN 1999a, Article 39).

***Tanymecus* Germar, 1817b**: 341. Current status: valid genus in CURCULIONIDAE: ENTIMINAE: POLYDRUSITAE: TANYMECINI: TANYMECINA.

Type species: *Curculiopalliatus* Fabricius, 1787, by subsequent designation (Schönherr 1823: 1144).

Family-group name: TANYMÉCIDES Lacordaire, 1863. Stem: *Tanymec*-.

***Tanypleurus* Raffray, 1890a**: 128, 130. Current status: invalid senior synonym of *Natypleurus* Newton & Thayer, 1992 in STAPHYLINIDAE: PSELAPHINAE: GONIACERITAE: INIOCYPHINI: NATYPLEURINA. Note: junior homonym of *Tanypleurus* Steenstrup & Lütken, 1861 [Crustacea].

Type species: *Tanypleurusmalaianus* Raffray, 1890, by subsequent monotypy (Raffray 1890b: 206).

Family-group name: TANYPLEURINI Jeannel, 1949. Stem: *Tanypleur*-. Note: family-group name permanently invalid (ICZN 1999a, Article 39).

***Tanyproctus* Ménétriés, 1832**: 185. Current status: valid genus in SCARABAEIDAE: MELOLONTHINAE: TANYPROCTINI: TANYPROCTINA.

Type species: *Tanyproctuspersicus* Ménétriés, 1832, by monotypy.

Family-group name: TANYPROCTINI Erichson, 1847. Stem: *Tanyproct*-.

***Tanyrhynchus* Schönherr, 1826**: 212. Current status: valid genus in CURCULIONIDAE: ENTIMINAE: ENTIMITAE: TANYRHYNCHINI.

Type species: *Tanyrhynchusterranus* Schönherr, 1826 (= *Curculiostrigirostris* Sparrman, 1785), by original designation.

Family-group name: TANYRHYNCHIDES Schönherr, 1826. Stem: *Tanyrhynch*-.

***Tanysphyrus* Germar, 1817b**: 340. Current status: valid genus in CURCULIONIDAE: BRACHYCERINAE: TANYSPHYRINI.

Type species: *Curculiolemnae* Fabricius, 1792, by monotypy.

Family-group name: TANYSPHYRIDAE Gistel, 1848. Stem: *Tanysphyr*-.

***Tapeina* Lepeletier & Audinet-Serville, 1828**: 545. Current status: valid genus in CERAMBYCIDAE: LAMIINAE: TAPEININI.

Type species: *Tapeinacoronata* Lepeletier & Audinet-Serville, 1828, by subsequent designation (Chevrolat 1849a: 344).

Family-group name: TAPEINITES J. Thomson, 1857. Stem: *Tapein*-.

***Taphes* C.O. Waterhouse, 1878**: 102. Current status: valid genus in LYCIDAE: EROTINAE: TAPHINI.

Type species: *Taphesbrevicollis* C.O. Waterhouse, 1878, by original designation.

Family-group name: TAPHININA Bocák & Bocáková, 1990. Stem: *Taph*-.

***Taphroderes* Schönherr, 1823**: 1137. Current status: valid genus in BRENTIDAE: BRENTINAE: TAPHRODERINI.

Type species: *Brentusfoveatus* Lund, 1802, by original designation.

Family-group name: TAPHRODÉRIDES Lacordaire, 1865. Stem: *Taphroder*-.

***Taphrorychus* Eichhoff, 1878**: 204. Current status: valid genus in CURCULIONIDAE: SCOLYTINAE: DRYOCOETINI.

Type species: *Bostrichusbicolor* Herbst, 1793, by subsequent designation (Hopkins 1914: 130).

Family-group name: TAPHRORYCHINI Reitter, 1913. Stem: *Taphrorych*-.

***Tapinotus* Schönherr, 1826**: 21, 292. Current status: valid genus in CURCULIONIDAE: CEUTORHYNCHINAE: CEUTORHYNCHINI. Note: the spelling *Tapeinotus* (p. 21) was corrected to *Tapinotus* in the *corrigenda* [unpaginated page].

Type species: *Tapinotusephippiger* Schönherr, 1826 (= *Attelabussellatus* Fabricius, 1794), by original designation.

Family-group name: TAPINOTINAE Joy, 1932. Stem: *Tapinot*-.

***Tapiromimus* Wollaston, 1877**: 149. Current status: valid genus in CURCULIONIDAE: COSSONINAE: TAPIROMIMINI.

Type species: *Tapiromimusgibbirostris* Wollaston, 1877, by monotypy.

Family-group name: TAPIROMIMINI Voss, 1972. Stem: *Tapiromim*-.

***Tarphius* Erichson, 1845a**: 256. Current status: valid genus in ZOPHERIDAE: COLYDIINAE: SYNCHITINI.

Type species: *Tarphiusgibbulus* Erichson, 1845, by monotypy.

Family-group name: TARPHIINAE Sharp, 1894. Stem: *Tarphi*-.

***Tarquinius* Kuwert, 1891**: 164. Current status: valid genus in PASSALIDAE: PASSALINAE: MACROLININI.

Type species: *Tarquiniusparadoxus* Kuwert, 1891, by monotypy.

Family-group name: TARQUININAE Kuwert, 1891. Stem: *Tarquini*-.

***Tarsostenus* Spinola, 1845a**: 287. Current status: valid genus in CLERIDAE: KORYNETINAE.

Type species: *Clerusunivittatus* Rossi, 1792, by monotypy.

Family-group name: TARSOSTÉNITES Jacquelin du Val, 1860. Stem: *Tarsosten*-.

***Tarus* Clairville, 1806**: 94. Current status: junior synonym of *Cymindis* Latreille, 1805 in CARABIDAE: HARPALINAE: LEBIINI: CYMINDIDINA.

Type species: *Buprestishumeralis* Geoffroy, 1785, by subsequent designation (Curtis 1828: pl. 235).

Family-group name: TARIDAE Gistel, 1848. Stem: *Tar*-.

***Tasmosalpingus* Lea, 1919a**: 743. Current status: valid genus in TASMOSALPINGIDAE.

Type species: *Tasmosalpingusquadrispilotus* Lea, 1919, by original designation.

Family-group name: TASMOSALPINGINAE Lawrence & Britton, 1991. Stem: *Tasmosalping*-.

***Taurocerastes* R.A. Philippi, 1865**: 115. Current status: valid genus in GEOTRUPIDAE: TAUROCERASTINAE.

Type species: *Taurocerastespatagonicus* R.A. Philippi, 1865, by monotypy.

Family-group name: TAUROCERASTIDAE Germain, 1897. Stem: *Taurocerast*-.

***Tauroma* Hope, 1839**: 97. Current status: junior synonym of *Omocerus* Chevrolat, 1835 in CHRYSOMELIDAE: CASSIDINAE: OMOCERINI.

Type species: *Cassidataurus* Fabricius, 1787, by original designation.

Family-group name: TAUROMITAE Spaeth, 1923. Stem: *Taurom*-.

***Taxicera* Mulsant & Rey, 1873**: 188. Current status: valid genus in STAPHYLINIDAE: ALEOCHARINAE: TAXICERINI.

Type species: *Taxiceraperfoliata* Mulsant & Rey, 1873, by subsequent designation (Blackwelder 1952: 374).

Family-group name: TAXICERINA Lohse, 1989. Stem: *Taxicer*-.

***Techmessa* F. Bates, 1874**: 113. Current status: valid genus in PYROCHROIDAE: PILIPALPINAE.

Type species: *Techmessaconcolor* F. Bates, 1874, by subsequent designation (Watt 1987: 124).

Family-group name: TECHMESSINAE Paulus, 1972. Stem: *Techmess*-.

***Tefflus* Leach, 1819**: 494. Current status: valid genus in CARABIDAE: HARPALINAE: PANAGAEINI: TEFFLINA.

Type species: *Carabusmeyerlei* Fabricius, 1801, by monotypy.

Family-group name: TEFFLINI Basilewsky, 1946. Stem: *Teffl*-.

***Tegrodera* J.L. LeConte, 1851a**: 159. Current status: valid genus in MELOIDAE: MELOINAE: EUPOMPHINI.

Type species: *Tegroderaerosa* J.L. LeConte, 1851, by monotypy.

Family-group name: TEGRODERINI L.S. Dillon, 1952. Stem: *Tegroder*-.

***Telacis* Poey, 1854**: 322. Current status: junior synonym of *Cteniopus* Solier, 1835 in TENEBRIONIDAE: ALLECULINAE: CTENIOPODINI. Note: new replacement name for *Cistela* Fabricius, 1775.

Type species: *Chrysomelasulphurea* Linnaeus, 1758, by subsequent designation (Latreille 1810: 429) (type fixation for *Cistela* Fabricius).

Family-group name: TELACIANAE Poey, 1854. Stem: *Telac*-.

***Teledapus* Pascoe, 1871c**: 268. Current status: valid genus in CERAMBYCIDAE: LEPTURINAE: TELEDAPINI.

Type species: *Teledapusdorcadioides* Pascoe, 1871, by monotypy.

Family-group name: TELEDAPINAE Pascoe, 1871. Stem: *Teledap*-.

***Telegeusis* G.H. Horn, 1895b**: 242. Current status: valid genus in OMETHIDAE: TELEGEUSINAE.

Type species: *Telegeusisdebilis* G.H. Horn, 1895, by monotypy.

Family-group name: TELEGEUSIDAE Leng, 1920. Stem: *Telegeus*-.

***Telephanus* Erichson, 1846**: 329. Current status: valid genus in SILVANIDAE: BRONTINAE: TELEPHANINI.

Type species: *Telephanusatricapillus* Erichson, 1846, by monotypy.

Family-group name: TELEPHANIDAE J.L. LeConte, 1861. Stem: *Telephan*-.

***Telephorus* Schäffer, 1766**: pl. cxxiii. Current status: junior synonym of *Cantharis* Linnaeus, 1758 in CANTHARIDAE: CANTHARINAE: CANTHARINI. Note: this name was first made available by Schäffer (1764) under the spelling *Thelophorus* (see Bousquet 2016: 469); the spelling *Telephorus*, which is in prevailing usage, is an incorrect subsequent spelling of *Thelephorus* but since *Telephorus* is not attributed to the publication of the original spelling, Article 33.3.1 (ICZN 1999a) cannot be used; an application to the Commission is necessary to conserve the currently-used spelling *Telephorus*.

Type species: *Cantharisfusca* Linnaeus, 1758, by subsequent designation (Latreille 1810: 426).

Family-group name: TELEPHORIDES Leach, 1815. Stem: *Telephor*-.

***Telmatophilus* Heer, 1841**: 417. Current status: valid genus in CRYPTOPHAGIDAE: CRYPTOPHAGINAE: CRYPTOPHAGINI.

Type species: *Ipscaricis* G.-A. Olivier, 1790, by subsequent designation (Chevrolat 1849a: 445). Note: *Cryptophagustyphae* Fallén, 1802 is often listed as type species of *Telmatophilus*, by subsequent designation of Leschen (1996: 603) but Chevrolat’s designation is earlier; both species are currently included in the genus *Telmatophilus* Heer.

Family-group name: TELMATOPHILIDES Jacquelin du Val, 1859. Stem: *Telmatophil*-.

***Telochilus* Krikken, 1975**: 21. Current status: valid genus in SCARABAEIDAE: CETONIINAE: CREMASTOCHEILINI: TELOCHILINA.

Type species: *Telochilusfreyi* Krikken, 1975, by original designation.

Family-group name: TELOCHILINA Krikken, 1984. Stem: *Telochil*-.

***Telsimia* Casey, 1899**: 165. Current status: valid genus in COCCINELLIDAE: COCCINELLINAE: TELSIMIINI.

Type species: *Telsimiatetrasticta* Casey, 1899, by subsequent designation (Chapin 1926: 130).

Family-group name: TELSIMIINI Casey, 1899. Stem: *Telsimi*-.

***Temnaspis* Lacordaire, 1845**: 716. Current status: valid genus in MEGALOPODIDAE: MEGALOPODINAE: MEGALOPODINI: TEMNASPIDINA.

Type species: *Megalopusjavanus* Guérin-Méneville, 1844, by subsequent designation (Jacoby and Clavareau 1905: 12).

Family-group name: TEMNASPIDINA Rodríguez-Mirón in Rodríguez-Mirón et al., 2021: 695. Stem: *Temnaspid*-.

***Temnocerus* Thunberg, 1815**: 110. Current status: valid genus in ATTELABIDAE: RHYNCHITINAE: RHYNCHITITAE: RHYNCHITINI: TEMNOCERINA.

Type species: *Attelabusplanirostris* Fabricius, 1801 (= *Curculionanus* Paykull, 1792), by subsequent designation (Crotch 1870b: 227).

Family-group name: TEMNOCERINA Legalov, 2003. Stem: *Temnocer*-.

***Temnoscheila* Westwood, 1830b**: 231. Current status: valid genus in TROGOSSITIDAE: TROGOSSITINAE: TROGOSSITINI.

Type species: *Trogossitacaerulea* G.-A. Olivier, 1790, by subsequent designation (E. Desmarest 1851: 275).

Family-group name: TEMNOCHILINI Léveillé, 1888. Stem: *Temnoscheil*-.

***Temnostega* Enderlein, 1905**: 719. Current status: valid genus in CARABIDAE: TRECHINAE: TRECHINI: TRECHINA.

Type species: *Temnostegaantarctica* Enderlein, 1905, by monotypy.

Family-group name: TEMNOSTEGINI Enderlein, 1909. Stem: *Temnosteg*-.

***Tenebrio* Linnaeus, 1758**: 417. Current status: valid genus in TENEBRIONIDAE: TENEBRIONINAE: TENEBRIONINI.

Type species: *Tenebriomolitor* Linnaeus, 1758, by subsequent designation (Latreille 1810: 429).

Family-group name: TENEBRIONITES Latreille, 1802. Stem: *Tenebrion*-.

***Tenebroides* Piller & Mitterpacher, 1783**: 87. Current status: valid genus in TROGOSSITIDAE: TROGOSSITINAE: TROGOSSITINI.

Type species: *Tenebroidescomplanatus* Piller & Mitterpacher, 1783 (= *Tenebriomauritanicus* Linnaeus, 1758), by monotypy.

Family-group name: TENEBRIOIDINI Ganglbauer, 1899. Stem: *Tenebroid*-.

***Tentyria* Latreille, 1802b**: 170. Current status: valid genus in TENEBRIONIDAE: PIMELIINAE: TENTYRIINI. Note: name placed on the Official List of Generic Names in Zoology (ICZN 2010b).

Type species: *Tentyrialigurica* Solier, 1835, by plenary power (see ICZN 2010b). Note: name placed on the Official List of Specific Names in Zoology (ICZN 2010b).

Family-group name: TENTYRIDAE Eschscholtz, 1831. Stem: *Tentyri*-.

***Tephraea* Burmeister, 1842**: 419. Current status: valid genus in SCARABAEIDAE: CETONIINAE: CETONIINI: CETONIINA.

Type species: *Cetoniapulverulenta* Gory & Percheron, 1834, by subsequent designation (Kraatz 1882a: 67).

Family-group name: TEPHRAEIDES Schenkling, 1921. Stem: *Tephrae*-.

***Teplinus* Pakaluk, Ślipiński & Lawrence, 1994**: 228. Current status: valid genus in CORYLOPHIDAE: CORYLOPHINAE: TEPLININI. Note: new replacement name for *Peltinus* Mulsant & Rey, 1861.

Type species: *Peltinusvelatus* Mulsant & Rey, 1861, by monotypy (type fixation for *Peltinus* Mulsant & Rey).

Family-group name: TEPLININI Pakaluk, Ślipiński & Lawrence, 1994. Stem: *Teplin*-.

***Terambus* Gistel, 1848b**: 130. Current status: junior synonym of *Aromia* Audinet-Serville, 1834 in CERAMBYCIDAE: CERAMBYCINAE: CALLICHROMATINI.

Type species: *Cerambyxmoschatus* Linnaeus, 1758, by subsequent designation (Bousquet et al. 2009: 42).

Family-group name: TERAMBIDAE Gistel, 1848. Stem: *Teramb*-.

***Teredus* Dejean, 1835**: 313. Current status: valid genus in TEREDIDAE: TEREDINAE: TEREDINI.

Type species: *Lyctusnitidus* Fabricius, 1792 (= *Ipscylindrica* G.-A. Olivier, 1790), by monotypy.

Family-group name: TEREDINI Seidlitz, 1888. Stem: *Tered*-.

***Tereticus* C.O. Waterhouse, 1879c**: 534. Current status: valid genus in CERAMBYCIDAE: PRIONINAE: TERETICINI.

Type species: *Tereticuspectinicornis* C.O. Waterhouse, 1879, by monotypy.

Family-group name: TERETICI Lameere, 1913. Stem: *Teretic*-.

***Teretrius* Erichson, 1834**: 201. Current status: valid genus in HISTERIDAE: ABRAEINAE: TERETRIINI.

Type species: *Histerpicipes* Fabricius, 1792 (= *Teretriusfabricii* Mazur, 1972), by monotypy.

Family-group name: TERETRIINAE Bickhardt, 1914. Stem: *Teretri*-.

***Termitella* Wasmann, 1911**: 170. Current status: valid genus in STAPHYLINIDAE: ALEOCHARINAE: COROTOCINI: TERMITOGASTRINA.

Type species: *Termitellalujae* Wasmann, 1911, by monotypy.

Family-group name: TERMITELLICI Jacobson, Kistner & Pasteels, 1986. Stem: *Termitell*-.

***Termitochara* Wasmann, 1893b**: 247. Current status: valid genus in STAPHYLINIDAE: ALEOCHARINAE: COROTOCINI: TERMITOCHARINA.

Type species: *Termitocharakraatzi* Wasmann, 1893, by monotypy.

Family-group name: TERMITOCHARINA Seevers, 1957. Stem: *Termitochar*-.

***Termitocupidus* Jacobson, Kistner & Pasteels, 1986**: 36. Current status: valid genus in STAPHYLINIDAE: ALEOCHARINAE: COROTOCINI: TERMITOCUPIDINA.

Type species: *Termitocupidusjudithae* Jacobson, Kistner & Pasteels, 1986, by original designation.

Family-group name: TERMITOCUPIDINA Jacobson, Kistner & Pasteels, 1986. Stem: *Termitocupid*-.

***Termitoderus* Mateu, 1966**: 718. Current status: valid genus in SCARABAEIDAE: APHODIINAE: TERMITODERINI.

Type species: *Termitoderusgrassei* Mateu, 1966, by monotypy.

Family-group name: TERMITODERINI Tangelder & Krikken, 1982. Stem: *Termitoder*-.

***Termitodiscus* Wasmann, 1899**: 147. Current status: valid genus in STAPHYLINIDAE: ALEOCHARINAE: TERMITODISCINI.

Type species: *Termitodiscusheimi* Wasmann, 1899, by monotypy.

Family-group name: TERMITODISCINI Wasmann, 1904. Stem: *Termitodisc*-.

***Termitogaster* Casey, 1889**: 384. Current status: valid genus in STAPHYLINIDAE: ALEOCHARINAE: COROTOCINI: TERMITOGASTRINA.

Type species: *Termitogasterinsolens* Casey, 1889, by monotypy.

Family-group name: TERMITOGASTRI Bernhauer & Scheerpeltz, 1926. Stem: *Termitogastr*-.

***Termitohospes* Seevers, 1941**: 333. Current status: valid genus in STAPHYLINIDAE: ALEOCHARINAE: TERMITOHOSPITINI: TERMITOHOSPITINA.

Type species: *Termitohospesmiricorniger* Seevers, 1941, by original designation.

Family-group name: TERMITOHOSPINI Seevers, 1941. Stem: *Termitohospit*-.

***Termitoiceus* Silvestri, 1901**: 5. Current status: valid genus in STAPHYLINIDAE: ALEOCHARINAE: COROTOCINI: TERMITOICEINA.

Type species: *Termitoiceusanastrephoproctus* Silvestri, 1901, by original designation.

Family-group name: TERMITOICEINA Jacobson, Kistner & Pasteels, 1986. Stem: *Termitoice*-.

***Termitomimus* Trägårdh, 1907**: 173. Current status: valid genus in STAPHYLINIDAE: ALEOCHARINAE: COROTOCINI: COROTOCINA.

Type species: *Termitomimusentendveniensis* Trägårdh, 1907, by monotypy.

Family-group name: TERMITOMIMINI Fenyes, 1921. Stem: *Termitomim*-.

***Termitonannus* Wasmann, 1902**: 2. Current status: valid genus in STAPHYLINIDAE: ALEOCHARINAE: TERMITONANNINI: TERMITONANNINA.

Type species: *Termitonannusschmalzi* Wasmann, 1902, by subsequent designation (Fenyes 1919: 25).

Family-group name: TERMITONANNINI Fenyes, 1919. Stem: *Termitonann*-.

***Termitonda* Seevers, 1957**: 238. Current status: valid genus in STAPHYLINIDAE: ALEOCHARINAE: TERMITOPAEDIINI.

Type species: *Termitondaliberiae* Seevers, 1957, by original designation.

Family-group name: TERMITONDINA Seevers, 1957. Stem: *Termitond*-.

***Termitopaedia* Wasmann, 1911**: 114. Current status: valid genus in STAPHYLINIDAE: ALEOCHARINAE: TERMITOPAEDIINI.

Type species: *Termitopaediakohli* Wasmann, 1911, by monotypy.

Family-group name: TERMITOPAEDIINI Seevers, 1957. Stem: *Termitopaedi*-.

***Termitopithus* Seevers, 1957**: 80. Current status: valid genus in STAPHYLINIDAE: ALEOCHARINAE: COROTOCINI: TERMITOPITHINA.

Type species: *Termitopithuscrassiusculus* Seevers, 1957, by original designation.

Family-group name: TERMITOPITHINA Jacobson, Kistner & Pasteels, 1986. Stem: *Termitopith*-.

***Termitopsenius* Wasmann, 1902**: 4. Current status: valid genus in STAPHYLINIDAE: ALEOCHARINAE: TRICHOPSENIINI.

Type species: *Termitopseniuslimulus* Wasmann, 1902, by monotypy.

Family-group name: TERMITOPSENINI Wasmann, 1916. Stem: *Termitopseni*-.

***Termitoptochus* Silvestri, 1910**: 37. Current status: valid genus in STAPHYLINIDAE: ALEOCHARINAE: COROTOCINI: TERMITOPTOCHINA.

Type species: *Termitoptochusindicus* Silvestri, 1910, by monotypy.

Family-group name: TERMITOPTOCHINI Fenyes, 1921. Stem: *Termitoptoch*-.

***Termitospectrum* Mann, 1926**: 154. Current status: valid genus in STAPHYLINIDAE: ALEOCHARINAE: TERMITUSINI: TERMITOSPECTRINA.

Type species: *Termitospectrumthoracicum* Mann, 1926, by original designation.

Family-group name: TERMITOSPECTRINA Seevers, 1957. Stem: *Termitospectr*-.

***Termitotelus* Wasmann, 1908**: 444. Current status: valid genus in STAPHYLINIDAE: ALEOCHARINAE: ATHETINI: TERMITOTELINA.

Type species: *Termitotelusschultzei* Wasmann, 1908, by monotypy.

Family-group name: TERMITOTELINA Kistner, 1970. Stem: *Termitotel*-.

***Termitotrox* Reichensperger, 1915**: 16. Current status: valid genus in SCARABAEIDAE: TERMITOTROGINAE.

Type species: *Termitotroxconsobrinus* Reichenbacher, 1915, by monotypy.

Family-group name: TERMITOTROGINI Wasmann, 1918. Stem: *Termitotrog*-.

***Termitozyras* Seevers, 1957**: 242. Current status: valid genus in STAPHYLINIDAE: ALEOCHARINAE: LOMECHUSINI: TERMITOZYRINA.

Type species: *Termitozyrasadamsoni* Seevers, 1957, by original designation.

Family-group name: TERMITOZYRINA Seevers, 1957. Stem: *Termitozyr*-.

***Termitusa* Wasmann, 1905**: 199. Current status: valid genus in STAPHYLINIDAE: ALEOCHARINAE: TERMITUSINI: TERMITUSINA.

Type species: *Termitusasjoestedti* Wasmann, 1905, by monotypy.

Family-group name: TERMITUSAE Fenyes, 1919. Stem: *Termitus*-.

***Tessaromma* Newman, 1840a**: 20. Current status: valid genus in CERAMBYCIDAE: CERAMBYCINAE: TESSAROMMATINI.

Type species: *Tessarommaundatum* Newman, 1840, by monotypy.

Family-group name: TESSAROMMIDES Lacordaire, 1868. Stem: *Tessarommat*-.

***Tesserocerus* Saunders, 1837**: 155, 157. Current status: valid genus in CURCULIONIDAE: PLATYPODINAE: TESSEROCERINI: TESSEROCERINA.

Type species: *Platypusinsignis* Saunders, 1837, by monotypy.

Family-group name: TESSEROCERINAE H. Strohmeyer, 1914. Stem: *Tesserocer*-.

***Tetrabrachys* Kapur, 1948**: 319. Current status: valid genus in COCCINELLIDAE: COCCINELLINAE: COCCIDULINI. Note: new replacement name for *Lithophilus* Frölich, 1799.

Type species: *Lithophilusruficollis* Frölich, 1799 (= *Tritomaconnata* Panzer, 1796), by monotypy (type fixation for *Lithophilus* Frölich).

Family-group name: TETRABRACHINAE Kapur, 1948. Stem: *Tetrabrach*-.

***Tetracha* Hope, 1838a**: 6, 7. Current status: valid genus in CICINDELIDAE: MEGACEPHALINI. Note: nomen protectum (see Bousquet 2002: 23).

Type species: *Cicindelacarolina* Linnaeus, 1763, by original designation.

Family-group name: TETRACHAE Leng & Mutchler, 1916. Stem: *Tetrach*-.

***Tetradelus* Fauvel, 1904**: 90. Current status: valid genus in STAPHYLINIDAE: OMALIINAE: OMALIINI.

Type species: *Tetradelustrigonuroides* Fauvel, 1904, by monotypy.

Family-group name: TETRADELI Fauvel, 1904. Stem: *Tetradel*-.

***Tetragonoderus* Dejean, 1829**: 5, 485. Current status: valid genus in CARABIDAE: HARPALINAE: CYCLOSOMINI. Note: nomen protectum (see Bousquet 2002: 50).

Type species: *Carabusquadrum* Fabricius, 1792, by subsequent designation (Hope 1838a: 89).

Family-group name: TÉTRAGONODÉRIDES Chaudoir, 1871. Stem: *Tetragonoder*-.

***Tetralobus* Lepeletier & Audinet-Serville, 1828**: 594. Current status: valid genus in ELATERIDAE: TETRALOBINAE: TETRALOBINI.

Type species: *Elaterflabellicornis* Linnaeus, 1767, by subsequent designation (Lacordaire 1857: 164).

Family-group name: TETRALOBITES Laporte, 1840. Stem: *Tetralob*-.

†***Tetrameropsis* Kirejtshuk & Azar, 2008**: 37. Current status: valid genus in †TETRAMEROPSIDAE. Note: Lower Cretaceous (Lebanon).

Type species: †*Tetrameropsismesozoica* Kirejtshuk & Azar, 2008, by original designation.

Family-group name: †TETRAMEROPSINAE Kirejtshuk & Azar, 2008. Stem: *Tetramerops*- (ICZN 1999a, Article 29.4).

***Tetraonyx* Latreille, 1809b**: 204, 237. Current status: valid genus in MELOIDAE: TETRAONYCINAE.

Type species: *Tetraonyxoctomaculatum* Latreille, 1809, by monotypy.

Family-group name: TETRAONYCIDAE Böving & Craighead, 1931. Stem: *Tetraonyc*-.

***Tetraopes* Schönherr, 1817**: 401. Current status: valid genus in CERAMBYCIDAE: LAMIINAE: TETRAOPINI.

Type species: *Lamiatornator* Fabricius, 1775 (= *Cerambyxtetrophthalmus* Forster, 1771), by subsequent designation (Drapiez 1845: 374).

Family-group name: TETRAOPESITAE J. Thomson, 1860. Stem: *Tetraop*-.

***Tetraphalerus* C.O. Waterhouse, 1901**: 520. Current status: valid genus in OMMATIDAE: TETRAPHALERINAE.

Type species: *Tetraphaleruswagneri* C.O. Waterhouse, 1901, by monotypy.

Family-group name: TETRAPHALERINI Crowson, 1962. Stem: *Tetraphaler*-.

***Tetratoma* Fabricius, 1790**: 217. Current status: valid genus in TETRATOMIDAE: TETRATOMINAE: TETRATOMINI.

Type species: *Tetratomafungorum* Fabricius, 1790, by subsequent designation (Latreille 1810: 429).

Family-group name: TETRATOMAEDES Billberg, 1820. Stem: *Tetratom*-.

***Tetraulax* Jordan, 1903**: 182. Current status: valid genus in CERAMBYCIDAE: LAMIINAE: TETRAULAXINI.

Type species: *Tetraulaxlateralis* Jordan, 1903, by original designation.

Family-group name: TETRAULAXINI Breuning & Téocchi, 1977. Stem: *Tetraulax*-.

***Tetrigus* Candèze, 1857**: 254. Current status: valid genus in ELATERIDAE: AGRYPNINAE: HEMIRHIPINI: LUDIOCTENINA.

Type species: *Tetrigusparallelus* Candèze, 1857, by subsequent designation (Hyslop 1921: 671).

Family-group name: TETRIGUSINA Schimmel & Tarnawski, 2012: 116. Stem: *Tetrigus*- (ICZN 1999a, Article 29.4).

***Tetropium* W. Kirby, 1837**: 174. Current status: valid genus in CERAMBYCIDAE: SPONDYLIDINAE: TETROPIINI. Note: name placed on the Official List of Generic Names in Zoology (ICZN 1988b).

Type species: *Callidiumcinnamopterum* W. Kirby, 1837, by subsequent designation (J. Thomson 1864: 266; see ICZN 1988b). Note: name placed on the Official List of Specific Names in Zoology (ICZN 1988b).

Family-group name: TETROPIINA Seidlitz, 1891. Stem: *Tetropi*-.

***Tetrops* Stephens, 1829b**: 16. Current status: valid genus in CERAMBYCIDAE: LAMIINAE: TETROPINI. Note: this name was first made available by W. Kirby in W. Kirby and Spence (1826: 498) citing only *Lamiatornator* Fabricius, 1775 (= *Cerambyxtetrophthalmus* Forster, 1771) in it, but currently that species is placed in the genus *Tetraopes* Dalman, 1817; as advocated by Vives and Alonso- Zarazaga (2000: 660–661) and Bousquet et al. (2009: 38–39), a request should be submitted to the Commission to suppress the name *Tetrops* W. Kirby, 1826 for the purpose of the Principles of Priority and Homonymy.

Type species: *Lepturapraeusta* Linnaeus, 1758, by monotypy.

Family-group name: TETROPINI Portevin, 1927. Stem: *Tetrop*-.

***Thalasselephas* Egorov & Korotyaev, 1976**: 47. Current status: valid genus in CURCULIONIDAE: MOLYTINAE: EMPHYASTINI.

Type species: *Thalasselephasmajor* Egorov & Korotyaev, 1976, by original designation.

Family-group name: THALASSELEPHANTINI Alonso-Zarazaga & Lyal, 1999. Stem: *Thalasselephant*-.

***Thalassophilus* Wollaston, 1854**: xx, 71. Current status: valid genus in CARABIDAE: TRECHINAE: TRECHINI: TRECHODINA.

Type species: *Thalassophiluswhitei* Wollaston, 1854, by monotypy.

Family-group name: THALASSOPHILI Csiki, 1928. Stem: *Thalassophil*-.

***Thalia* Hope, 1838a**: 70. Current status: junior synonym of *Poecilus* Bonelli, 1810 in CARABIDAE: HARPALINAE: PTEROSTICHINI: PTEROSTICHINA. Note: unnecessary new replacement name for *Feronia* Latreille, 1816; junior homonym of *Thalia* Bruguière, 1791 [Mollusca] and *Thalia* Blumenbach, 1827 [Tunicata].

Type species: *Carabuscupreus* Linnaeus, 1758, by subsequent designation (Blanchard in Audouin et al. 1842: pl. 22) (type fixation for *Feronia* Latreille).

Family-group name: THALIADAE Hope, 1838. Stem: *Thali*-. Note: family-group name permanently invalid (ICZN 1999a, Article 39).

***Thaliabaris* Bondar, 1943**: 131. Current status: valid genus in CURCULIONIDAE: BARIDINAE: MADARINI: MADARINA.

Type species: *Thaliabarisgica* Bondar, 1943, by original designation.

Family-group name: THALIABARINI Bondar, 1943. Stem: *Thaliabarid*-.

***Thallisella* Crotch, 1876**: 402. Current status: valid genus in EROTYLIDAE: LANGURIINAE: THALLISELLINI.

Type species: *Thallisellaperuviana* Crotch, 1876, by original designation.

Family-group name: THALLISELLINI Sen Gupta, 1968. Stem: *Thallisell*-.

***Thalycra* Erichson, 1843a**: 305. Current status: valid genus in NITIDULIDAE: NITIDULINAE: NITIDULINI.

Type species: *Strongylussericeus* Sturm, 1839 (= *Nitidulafervida* G.-A. Olivier, 1790), by monotypy.

Family-group name: THALYCRINA C.G. Thomson, 1859. Stem: *Thalycr*-.

***Thamiaraea* C.G. Thomson, 1858**: 35. Current status: valid genus in STAPHYLINIDAE: ALEOCHARINAE: ATHETINI: THAMIARAEINA.

Type species: *Aleocharacinnamomea* Gravenhorst, 1802, by monotypy.

Family-group name: THAMIARAEINI Fenyes, 1921. Stem: *Thamiarae*-.

***Thamnophilus* Schönherr, 1823**: 1136. Current status: junior synonym of *Magdalis* Germar, 1817 in CURCULIONIDAE: MESOPTILIINAE: MAGDALIDINI. Note: unnecessary new replacement name for *Magdalis* Germar, 1817; junior homonym of *Thamnophilus* Vieillot, 1816 [Aves]; name placed on the Official Index of Rejected and Invalid Names in Zoology (see ICZN 1955a).

Type species: *Curculioviolaceus* Linnaeus, 1758, by plenary power (see ICZN 1955a) (type fixation for *Magdalis* Germar).

Family-group name: THAMNOPHILIDES Schönherr, 1823. Stem: *Thamnophil*-. Note: family-group name permanently invalid (ICZN 1999a, Article 39).

***Thamnurgus* Eichhoff, 1864**: 40, 45. Current status: valid genus in CURCULIONIDAE: SCOLYTINAE: DRYOCOETINI.

Type species: *Bostrichuseuphorbiae* Küster, 1845, by subsequent designation (Cazurro Ruiz 1897a: 186).

Family-group name: THAMNURGINAE Nüsslin, 1911. Stem: *Thamnurg*-.

***Thanasimus* Latreille, 1805**: 270. Current status: valid genus in CLERIDAE: CLERINAE: CLERITAE: CLERINI.

Type species: *Attelabusformicarius* Linnaeus, 1758, by subsequent designation (Latreille 1810: 427).

Family-group name: THANASIMIIDAE Gistel, 1848. Stem: *Thanasim*-.

***Thaneroclerus* Lefebvre, 1838b**: xiii. Current status: valid genus in THANEROCLERIDAE: THANEROCLERINAE: THANEROCLERINI.

Type species: *Clerusbuqueti* Lefebvre, 1835 [as *buquet*], by monotypy.

Family-group name: THANEROCLERINAE Chapin, 1924. Stem: *Thanerocler*-.

***Thaumaphrastus* Blaisdell, 1927**: 123. Current status: junior synonym of *Thorictodes* Reitter, 1875 in DERMESTIDAE: DERMESTINAE: THORICTINI.

Type species: *Thaumaphrastuskaranisensis* Blaisdell, 1927 (= *Thorictodesheydeni* Reitter, 1875), by monotypy.

Family-group name: THAUMAPHRASTINAE W.H. Anderson, 1949. Stem: *Thaumaphrast*-.

***Thaumastocephalus* Poggi, Nonveiller, Colla, Pavićević & Rada, 2001**: 3. Current status: valid genus in STAPHYLINIDAE: PSELAPHINAE: BATRISITAE: THAUMASTOCEPHALINI.

Type species: *Thaumastocephalusfolliculipalpus* Poggi, Nonveiller, Colla, Pavićević & Rada, 2001, by original designation.

Family-group name: THAUMASTOCEPHALINI Poggi, Nonveiller, Colla, Pavićević & Rada, 2001. Stem: *Thaumastocephal*-.

***Thaumastodus* Champion, 1924**: 25. Current status: junior synonym of *Pseudeucinetus* Heller, 1921 in LIMNICHIDAE: THAUMASTODINAE.

Type species: *Thaumastodusfusiformis* Champion, 1924 (= *Pseudeucinetuszygops* Heller, 1921), by original designation.

Family-group name: THAUMASTODINAE Champion, 1924. Stem: *Thaumastod*-.

***Thaumasus* Reiche, 1853**: xxiii. Current status: valid genus in CERAMBYCIDAE: CERAMBYCINAE: TORNEUTINI.

Type species: *Ipsgigas* G.-A. Olivier, 1792, by monotypy.

Family-group name: THAUMASIDAE J. Thomson, 1864. Stem: *Thaumas*-.

†***Thayeralinus* Solodovnikov & Yue in Solodovnikov et al., 2013**: 383. Current status: valid genus in STAPHYLINIDAE: STAPHYLININAE: †THAYERALININI. Note: Lower Cretaceous (China).

Type species: †*Thayeralinusfieldi* Solodovnikov & Yue, 2013, by original designation.

Family-group name: †THAYERALININI Solodovnikov & Yue in Solodovnikov et al., 2013: 383. Stem: *Thayeralin*-.

***Thecesternus* Say, 1831**: 8. Current status: valid genus in CURCULIONIDAE: CURCULIONINAE: THECESTERNINI.

Type species: *Brachycerushumeralis* Say, 1827, by monotypy.

Family-group name: THÉCESTERNIDES Lacordaire, 1863. Stem: *Thecestern*-.

***Thecturota* Casey, 1893**: 357. Current status: valid genus in STAPHYLINIDAE: ALEOCHARINAE: HOMALOTINI: HOMALOTINA.

Type species: *Thecturotatenuissima* Casey, 1893, by subsequent designation (Fenyes 1919: 25).

Family-group name: THECTUROTAE Fenyes, 1919. Stem: *Thecturot*-.

***Theocerus* Raffray, 1897a**: 280. Current status: valid genus in STAPHYLINIDAE: PSELAPHINAE: CLAVIGERITAE: CLAVIGERINI.

Type species: *Theoceruscrenulatus* Raffray, 1897, by monotypy.

Family-group name: THEOCERINI Jeannel, 1954. Stem: *Theocer*-.

***Theocris* J. Thomson, 1858b**: 193. Current status: valid genus in CERAMBYCIDAE: LAMIINAE: THEOCRINI.

Type species: *Theocrissaga* J. Thomson, 1858, by monotypy.

Family-group name: THÉOCRIDES Lacordaire, 1872. Stem: *Theocr*-.

***Theone* Gistel, 1857**: 585. Current status: valid genus in CHRYSOMELIDAE: GALERUCINAE: GALERUCINI.

Type species: *Gallerucasilphoides* Dalman, 1823, by original designation.

Family-group name: THEONINA Laboissière, 1934. Stem: *Theon*-.

***Therates* Latreille, 1816**: 179. Current status: valid genus in CICINDELIDAE: CICINDELINI: THERATINA.

Type species: *Cicindelalabiata* Weber, 1801, by monotypy. Note: this species was described in 1801 by both Weber (p. 44) and Fabricius (p. 232); bibliographic research suggests that Weber’s book (issued by March 1801, see Evenhuis 1997b: 809) was issued before Fabricius first volume of his ‘Systema Eleutheratorum’ (issued by 26 April 1801, see Bousquet 2017a: 108; see also Chapin 1946: 79).

Family-group name: THERATIDAE W. Horn, 1893. Stem: *Therat*-.

***Thermonectus* Dejean, 1833a**: 53. Current status: valid genus in DYTISCIDAE: DYTISCINAE: ACILIINI. Note: the original spelling was *Thermonetus* but *Thermonectus*, an incorrect subsequent spelling, is in prevailing usage, attributed to the publication of the original spelling, and therefore deemed to be the correct original spelling (ICZN 1999a, Article 33.3.1).

Type species: *Dytiscuscircumscriptus* Latreille, 1809, by monotypy.

Family-group name: THERMONECTINI Sharp, 1880. Stem: *Thermonect*-.

***Thilmanus* Baudi di Selve, 1872**: 97. Current status: valid genus in ELATERIDAE: OMALISINAE. Note: this name is credited to Gemminger in Gemminger and Harold (1869c: 1683) by the vast majority of authors in the literature but there is no description nor indication that would make the name available from that work.

Type species: *Thilmanusobscurus* Baudi di Selve, 1872, by monotypy. Note: Baudi di Selve (1874: clxxxvii) noted that the word “*obscurus*” was erroneously left out (at the time of the impression of the work) before the description, which started with the word “*fuscus*” and that subsequent authors have incorrectly interpreted that the name of the species was *Thilmanusfuscus*.

Family-group name: THILMANINAE Kazantsev, 2004. Stem: *Thilman*-.

***Thinobatis* Eschscholtz, 1831**: 5, 8. Current status: valid genus in TENEBRIONIDAE: PIMELIINAE: THINOBATINI.

Type species: *Thinobatisferruginea* Eschscholtz, 1831, by monotypy.

Family-group name: THINOBATIDES Lacordaire, 1859. Stem: *Thinobat*-.

***Thinobius* Kiesenwetter, 1844**: 355. Current status: valid genus in STAPHYLINIDAE: OXYTELINAE: THINOBIINI.

Type species: *Thinobiusciliatus* Kiesenwetter, 1844, by monotypy.

Family-group name: THINOBIIDES J.R. Sahlberg, 1876. Stem: *Thinobi*-.

***Thinopinus* J.L. LeConte, 1852d**: 215. Current status: valid genus in STAPHYLINIDAE: STAPHYLININAE: STAPHYLININI: STAPHYLININA.

Type species: *Thinopinuspictus* J.L. LeConte, 1852, by monotypy.

Family-group name: THINOPININAE Böving & Craighead, 1931. Stem: *Thinopin*-.

***Thinorycter* Semenov-Tjan-Shansky & Reikhardt, 1925**: 83. Current status: valid genus in SCARABAEIDAE: DYNAMOPODINAE: THINORYCTERINI.

Type species: *Thinorycterchlamydatus* Semenov-Tjan-Shansky & Reikhardt, 1925, by original designation.

Family-group name: THINORYCTERINA Semenov-Tjan-Shansky & Reikhardt, 1925. Stem: *Thinorycter*-.

***Thione* Sharp, 1899**: 544. Current status: valid genus in MONOTOMIDAE: MONOTOMINAE: THIONINI.

Type species: *Thionecephalotes* Sharp, 1899, by subsequent designation (Sen Gupta 1988: 51).

Family-group name: THIONINAE Crowson, 1952. Stem: *Thion*-.

***Thisias* Champion, 1889**: 102. Current status: valid genus in MYCTERIDAE: EURYPINAE.

Type species: *Thisiasmarmoratus* Champion, 1889, by monotypy.

Family-group name: THISIINA Seidlitz, 1917. Stem: *Thisi*-.

***Thliboclivina* Kult, 1959**: 175 [in key]. Current status: valid genus in CARABIDAE: SCARITINAE: CLIVININI: ARDISTOMINA.

Type species: *Clivinamicrophthalma* Burgeon, 1937, by original designation.

Family-group name: THLIBOCLIVININA Balkenohl, 2022: 107. Stem: *Thliboclivin*-.

***Thomassetia* Théry, 1929**: 183. Current status: valid genus in BUPRESTIDAE: BUPRESTINAE: THOMASSETIINI: THOMASSETIINA.

Type species: *Philanthaxianatalensis* Théry, 1929, by monotypy.

Family-group name: THOMASSETIINI Bellamy, 1987. Stem: *Thomasseti*-.

***Thonalmus* Bourgeois, 1884**: 376. Current status: valid genus in LYCIDAE: LYCINAE: THONALMINI.

Type species: *Cantharisbicolor* Linnaeus, 1763, by subsequent designation (Bourgeois 1891: 343).

Family-group name: THONALMINI Kleine, 1933. Stem: *Thonalm*-.

***Thoracophorus* Motschulsky, 1837**: 98. Current status: valid genus in STAPHYLINIDAE: OSORIINAE: THORACOPHORINI: THORACOPHORINA. Note: the original spelling was *Thoraxophorus* but *Thoracophorus*, an unjustified emendation by Gemminger and von Harold (1868b: 677), is in prevailing usage, attributed to the original author and date, and therefore is deemed to be a justified emendation (ICZN 1999a, Article 33.2.3.1).

Type species: *Thoracophoruscorticinus* Motschulsky, 1837, by monotypy.

Family-group name: THORACOPHORINAE Reitter, 1909. Stem: *Thoracophor*-.

***Thorictosoma* Lea, 1919b**: 257. Current status: valid genus in TENEBRIONIDAE: PIMELIINAE: CNEMEPLATIINI: THORICTOSOMATINA.

Type species: *Thorictosomaectatommae* Lea, 1919, by original designation.

Family-group name: THORICTOSOMATINA Watt, 1992. Stem: *Thorictosomat*-.

***Thorictus* Germar, 1834**: No 15. Current status: valid genus in DERMESTIDAE: DERMESTINAE: THORICTINI.

Type species: *Thorictuscastaneus* Germar, 1834, by monotypy.

Family-group name: THORICTIDES Erichson, 1846. Stem: *Thorict*-. Note: the authorship of this family-group name was credited to “Agassiz” in Bouchard et al. (2011: 334) but here we follow Bouchard et al. (2021: 9) in crediting new nomenclatural acts in the fascicle on Coleoptera of Agassiz’ ‘Nomenclator Zoologicus’ to the beetle specialist W.F. Erichson, whose contribution to the publication was acknowledged on the fascicle’s title page.

***Thranius* Pascoe, 1859a**: 22. Current status: valid genus in CERAMBYCIDAE: CERAMBYCINAE: THRANIINI.

Type species: *Thraniusbimaculatus* Pascoe, 1859, by subsequent designation (J. Thomson 1864: 162).

Family-group name: THRANIINI Gahan, 1906. Stem: *Thrani*-.

***Thrincopyge* J.L. LeConte, 1858b**: 17. Current status: valid genus in BUPRESTIDAE: POLYCESTINAE: THRINCOPYGINI.

Type species: *Buprestisambiens* J.L. LeConte, 1854, by original designation.

Family-group name: THRINCOPYGINI J.L. LeConte, 1861. Stem: *Thrincopyg*-.

***Thrombosternus* G.A.K. Marshall, 1955b**: 270. Current status: valid genus in CURCULIONIDAE: MOLYTINAE: CYCLOTERINI: THROMBOSTERNINA.

Type species: *Thrombosternuscucullatus* G.A.K. Marshall, 1955, by original designation.

Family-group name: THROMBOSTERNINA Voss, 1965. Stem: *Thrombostern*-.

†***Throscogenius* Iablokoff-Khnzorian, 1962**: 81. Current status: valid genus in EUCNEMIDAE: MACRAULACINAE: †THROSCOGENIINI. Note: Eocene (Lithuania).

Type species: †*Throscogeniustakhtajani* Iablokoff-Khnzorian, 1962, by original designation.

Family-group name: †THROSCOGENIINAE Iablokoff-Khnzorian, 1962. Stem: *Throscogeni*-.

***Throscus* Latreille, 1797**: 42. Current status: junior synonym of *Trixagus* Kugelann, 1794 in THROSCIDAE.

Type species: *Elaterdermestoides* Linnaeus, 1767, by subsequent designation (Curtis 1827: pl. 163). Note: no species were originally included in this genus by Latreille (1797); the first ones were the two species listed by Latreille (1802b: 100).

Family-group name: THROSCITES Laporte, 1840. Stem: *Throsc*-. Note: nomen protectum (see Bouchard et al. 2011: 306).

***Thryptocerus* Chaudoir, 1878b**: 74. Current status: valid genus in CARABIDAE: HARPALINAE: OODINI.

Type species: *Thryptoceruspolitus* Chaudoir, 1878, by monotypy.

Family-group name: THRYPTOCERINI Jeannel, 1949. Stem: *Thryptocer*-.

***Thylacites* Germar, 1817b**: 341. Current status: invalid senior synonym of *Brachyderes* Schönherr, 1823 in CURCULIONIDAE: ENTIMINAE: POLYDRUSITAE: BRACHYDERINI. Note: name suppressed for the Principle of Priority and placed on the Official Index of Rejected and Invalid Names in Zoology (ICZN 1987a).

Type species: *Curculioincanus* Linnaeus, 1758, by subsequent designation (Samouelle 1819: 205). Note: name placed on the Official List of Specific Names in Zoology (ICZN 1987a).

Family-group name: THYLACITIDAE W. Kirby, 1837. Stem: *Thylacit*-. Note: family-group name permanently invalid (ICZN 1999a, Article 39); name placed on the Official List of Rejected and Invalid Family-Group Names in Zoology (ICZN 1987a).

***Thylacosternus* Gemminger in Gemminger and Harold, 1869b**: 1486. Current status: valid genus in ELATERIDAE: THYLACOSTERNINAE. Note: this name is credited to Bonvouloir (1871: 76) in the literature but it was made available by Gemminger in 1869 when he associated an available species with it; the other ten species listed by Gemminger and von Harold (1869b: 1486, 1487) are nomina nuda.

Type species: *Pterotarsuswalckenaerii* Guérin-Méneville, 1843, by monotypy.

Family-group name: THYLACOSTERNINAE Fleutiaux, 1920. Stem: *Thylacostern*-.

***Thylodrias* Motschulsky, 1839**: 75. Current status: valid genus in DERMESTIDAE: TRINODINAE: THYLODRIINI.

Type species: *Thylodriascontractus* Motschulsky, 1839, by monotypy.

Family-group name: THELYDRIINI Semenov-Tjan-Shansky, 1909. Stem: *Thylodri*-.

***Thymalus* Latreille, 1802b**: 133. Current status: valid genus in THYMALIDAE: THYMALINAE.

Type species: *Cassidabrunnea* Thunberg, 1787 (= *Cassidalimbata* Fabricius, 1787), by monotypy.

Family-group name: THYMALINI Léveillé, 1888. Stem: *Thymal*-.

***Thyreopterus* Dejean, 1831**: 279, 445. Current status: valid genus in CARABIDAE: HARPALINAE: LEBIINI: PERICALINA.

Type species: *Thyreopterusflavosignatus* Dejean, 1831, by subsequent designation (Hope 1838a: 105).

Family-group name: THYRÉOPTÉRIDES Chaudoir, 1869. Stem: *Thyreopter*-.

***Thyrsia* Dalman, 1819**: 117. Current status: valid genus in CERAMBYCIDAE: CERAMBYCINAE: THYRSIINI.

Type species: *Thyrsialateralis* Dalman, 1819, by monotypy.

Family-group name: THYRSIINI Marinoni & Napp, 1984. Stem: *Thyrsi*-.

***Thysanotus* Chaudoir, 1848**: 123. Current status: valid genus in CARABIDAE: HARPALINAE: LEBIINI: PERICALINA.

Type species: *Euryderaanchomenoides* Laporte & Gory, 1835, by monotypy.

Family-group name: THYSANOTINI Jeannel, 1949. Stem: *Thysanot*-.

***Thysdarius* Fairmaire, 1904b**: 117. Current status: valid genus in STAPHYLINIDAE: PSELAPHINAE: CLAVIGERITAE: CLAVIGERINI. Note: new replacement name for *Thysdrus* Fairmaire, 1899.

Type species: *Thysdrusperrieri* Fairmaire, 1899, by monotypy (type fixation for *Thysdrus* Fairmaire).

Family-group name: THYSDARIINI Jeannel, 1954. Stem: *Thysdari*-.

***Tibionema* Solier, 1851**: 30. Current status: valid genus in ELATERIDAE: PITYOBIINAE: *TIBIONEMINI.

Type species: *Tibionemarufiventre* Solier, 1851, by original designation.

Family-group name: *TIBIONEMINI Motyka, Kusy, Arias-Bohart, Bybee & Bocák, 2023a: 7. Stem: *Tibionem*-.

***Tichonia* Semenov, 1904**: 201. Current status: junior synonym of *Microcephalus* Dejean, 1828 in CARABIDAE: HARPALINAE: PTEROSTICHINI: MICROCEPHALINA. Note: new replacement name for *Caletor* Tschitschérine 1903, an unnecessary new replacement name for *Microcephalus* Dejean, 1828; junior homonym of *Tichonia* Hübner, 1825 [Lepidoptera].

Type species: *Microcephalusdepressicollis* Dejean, 1828, by monotypy (type fixation for *Microcephalus* Dejean).

Family-group name: TICHONIINI Semenov, 1904. Stem: *Tichoni*-. Note: family-group name permanently invalid (ICZN 1999a, Article 39).

***Tichonilla* Strand, 1942**: 393. Current status: junior synonym of *Microcephalus* Dejean, 1828 in CARABIDAE: HARPALINAE: PTEROSTICHINI: MICROCEPHALINA. Note: unnecessary new replacement name for *Tichonia* Semenov, 1904 (an unnecessary new replacement name for *Microcephalus* Dejean, 1828).

Type species: *Microcephalusdepressicollis* Dejean, 1828, by monotypy (type fixation for *Microcephalus* Dejean).

Family-group name: TICHONILLINA Emden, 1958. Stem: *Tichonill*-.

***Tillomorpha* Blanchard, 1851**: 482. Current status: valid genus in CERAMBYCIDAE: CERAMBYCINAE: TILLOMORPHINI.

Type species: *Tillomorphalineoligera* Blanchard, 1851, by monotypy.

Family-group name: TILLOMORPHIDES Lacordaire, 1868. Stem: *Tillomorph*-.

***Tillus* G.-A. Olivier, 1790a**: [22] 3. Current status: valid genus in CLERIDAE: TILLINAE.

Type species: *Chrysomelaelongata* Linnaeus, 1758, by subsequent designation (Latreille 1810: 427). Note: name placed on the Official List of Specific Names in Zoology (ICZN 2011c).

Family-group name: TILLII Fischer, 1813. Stem: *Till*-.

***Timarcha* Samouelle, 1819**: 213. Current status: valid genus in CHRYSOMELIDAE: CHRYSOMELINAE: TIMARCHINI. Note: this genus is often credited to Latreille (1829b: 150) (e.g., Gómez- Zurita and Kippenberg 2010: 437) but it was made available earlier by Samouelle in 1819 when he associated two available nominal species, *Chrysomelatenebricosa* Fabricius, 1775 (on page 213) and *Chrysomelacoriaria* Laicharting, 1781 (on page 445), with the name.

Type species: *Chrysomelatenebricosa* Fabricius, 1775, by subsequent designation (Chevrolat 1843a: 655). Note: the type species often listed for the genus *Timarcha*, *Tenebriolaevigatus* Linnaeus, 1767 (e.g., Gómez-Zurita and Kippenberg 2010: 437), is not a species originally included species in *Timarcha*; *Chrysomelatenebricosa* Fabricius, 1775 was given precedence over *Tenebriolaevigatus* Linnaeus, 1767 and placed on the Official List of Specific Names in Zoology (ICZN 2021b).

Family-group name: TIMARCHAEINES Motschulsky, 1860. Stem: *Timarch*-.

†***Timarchopsis* Brauer, J. Redtenbacher & Ganglbauer, 1889**: 17. Current status: valid genus in †COPTOCLAVIDAE: †TIMARCHOPSINAE. Note: Jurassic (Russia).

Type species: †*Timarchopsisczekanowskii* Brauer, J. Redtenbacher & Ganglbauer, 1889, by monotypy.

Family-group name: †TIMARCHOPSINAE B. Wang, Ponomarenko & Zhang, 2010. Stem: *Timarchops*- (ICZN 1999a, Article 29.4).

***Timeparthenus* Silvestri, 1901**: 10. Current status: valid genus in STAPHYLINIDAE: ALEOCHARINAE: COROTOCINI: TIMEPARTHENINA.

Type species: *Timeparthenusregius* Silvestri, 1901, by original designation.

Family-group name: TIMEPARTHENINI Fenyes, 1921. Stem: *Timeparthen*-.

***Timola* Pascoe, 1886**: 331. Current status: valid genus in CURCULIONIDAE: CURCULIONINAE: CURCULIONINI: TIMOLINA.

Type species: *Timolasuturalis* Pascoe, 1886 (= *Balaninussuturalis* Gyllenhal, 1835), by monotypy.

Family-group name: TIMOLINA Heller, 1925. Stem: *Timol*-.

***Tiracerus* Besuchet, 1986**: 262. Current status: valid genus in STAPHYLINIDAE: PSELAPHINAE: CLAVIGERITAE: TIRACERINI.

Type species: *Articeruscurvicornis* Westwood, 1856, by original designation.

Family-group name: TIRACERINI Besuchet, 1986. Stem: *Tiracer*-.

***Tisiphone* Reitter, 1876a**: 297. Current status: invalid senior synonym of *Smicrips* J.L. LeConte, 1878 in SMICRIPIDAE. Note: junior homonym of *Tisiphone* Hübner, 1819 [Lepidoptera].

Type species: *Tisiphonehypocoproides* Reitter, 1876, by subsequent designation (Casey 1916: 104)

Family-group name: TISIPHONINAE Sharp, 1900. Stem: *Tisiphon*-. Note: family-group name permanently invalid (ICZN 1999a, Article 39).

***Titaena* Erichson, 1842**: 178. Current status: valid genus in TENEBRIONIDAE: TENEBRIONINAE: TITAENINI.

Type species: *Titaenacolumbina* Erichson, 1842, by subsequent designation (Chevrolat 1849a: 594).

Family-group name: TITAENINI Fauvel, 1905. Stem: *Titaen*-.

***Titanus* Audinet-Serville, 1832**: 133. Current status: valid genus in CERAMBYCIDAE: PRIONINAE: PRIONINI.

Type species: *Cerambyxgiganteus* Linnaeus, 1771, by monotypy.

Family-group name: TITANITAE J. Thomson, 1864. Stem: *Titan*-.

***Tmesiphorus* J.L. LeConte, 1849**: 75. Current status: valid genus in STAPHYLINIDAE: PSELAPHINAE: PSELAPHITAE: TMESIPHORINI.

Type species: *Pselaphuscarinatus* Say, 1824, by subsequent designation (Bowman 1934: 144).

Family-group name: TMESIPHORINI Jeannel, 1949. Stem: *Tmesiphor*-.

***Tmesisternus* Latreille, 1829b**: 121. Current status: valid genus in CERAMBYCIDAE: LAMIINAE: TMESISTERNINI.

Type species: *Tmesisternusbizonulatus* Guérin-Méneville, 1831, by subsequent monotypy (Guérin-Méneville 1831b: pl. 45). Note: this species is incorrectly listed in the literature as a junior synonym of *Tmesisternustrivittatus* Guérin-Méneville, 1831, which is available from plate 7 (issued 13 October 1831, see Bousquet 2016: 235) of the ‘Voyage autour du monde...La Coquille’; *T.bizonulatus* is available from plate 45 (issue by March 1831, see Bousquet 2016: 234) of the ‘Iconographie du règne animal’.

Family-group name: TMESISTERNITAE Blanchard, 1853. Stem: *Tmesistern*-.

***Tmesorrhina* Westwood, 1842b**: 71. Current status: valid genus in SCARABAEIDAE: CETONIINAE: GOLIATHINI: RHOMBORHININA.

Type species: *Gnathoceraamabilis* Bainbridge, 1840, by subsequent designation (Sakai and Nagai 1998: 230).

Family-group name: TMESORRHINAE Schoch, 1894. Stem: *Tmesorrhin*-.

***Tolyphus* Erichson, 1845a**: 108. Current status: valid genus in PHALACRIDAE: PHALACRINAE.

Type species: *Phalacrusgranulatus* Guérin-Méneville, 1834, by monotypy.

Family-group name: TOLYPHINI Guillebeau, 1892. Stem: *Tolyph*-.

***Tomicus* Latreille, 1802b**: 203. Current status: valid genus in CURCULIONIDAE: SCOLYTINAE: HYLURGINI. Note: name placed on the Official List of Generic Names in Zoology (ICZN 1963a).

Type species: *Dermestespiniperda* Linnaeus, 1758, by monotypy (see ICZN 1963a). Note: name placed on the Official List of Specific Names in Zoology (ICZN 1963a).

Family-group name: TOMICINI Wood, 1978. Stem: *Tomic*-.

***Tomoderus* LaFerté-Sénectère in Guérin-Méneville, 1849**: [no 26] 1. Current status: valid genus in ANTHICIDAE: TOMODERINAE.

Type species: *Tomoderuscruciatus* LaFerté-Sénectère, 1849, by subsequent designation (Bonadona 1958: 28).

Family-group name: TOMODERINI Bonadona, 1961. Stem: *Tomoder*-.

***Tomorhinus* Jekel, 1856**: 8. Current status: valid genus in CURCULIONIDAE: ENTIMINAE: ENTIMITAE: LORDOPINI.

Type species: *Hypsonotusdesignatus* Fåhraeus, 1842, by monotypy.

Family-group name: TOMORHINI Jekel, 1856. Stem: *Tomorhin*-.

***Tomyris* Chapuis, 1874**: 265. Current status: invalid senior synonym of *Eboo* Reid, 1993 in CHRYSOMELIDAE: EUMOLPINAE: BROMIINI. Note: junior homonym of *Tomyris* Eichwald, 1831 [Reptilia].

Type species: *Tomyrispulchella* Chapuis, 1874, by monotypy.

Family-group name: TOMYRITES Chapuis, 1874. Stem: *Tomyri*-. Note: family-group name permanently invalid (ICZN 1999a, Article 39).

***Tonerus* K.B. Miller, 2009**: 201. Current status: valid genus in NOTERIDAE: NOTERINAE: NOTERINI.

Type species: *Toneruswheeleri* K.B. Miller, 2009, by original designation.

Family-group name: TONERINI K.B. Miller, 2009. Stem: *Toner*-.

***Tonesia* Casey, 1922**: 337. Current status: valid genus in CURCULIONIDAE: BARIDINAE: EUTOXINI: TONESIINA.

Type species: *Tonesiaglabra* Casey, 1922, by original designation.

Family-group name: TONESIINA Alonso-Zarazaga & Lyal, 1999. Stem: *Tonesi*-.

***Tophoderes* Dejean, 1834**: 236. Current status: valid genus in ANTHRIBIDAE: ANTHRIBINAE: TOPHODERINI.

Type species: *Anthribusfrenatus* Klug, 1833, by monotypy.

Family-group name: TOPHODÉRIDES Lacordaire, 1865. Stem: *Tophoder*-.

***Toramus* Grouvelle, 1916b**: 26. Current status: valid genus in EROTYLIDAE: CRYPTOPHILINAE: TORAMINI. Note: new replacement name for *Tomarus* J.L. LeConte, 1861.

Type species: *Tomaruspulchellus* J.L. LeConte, 1863, by subsequent monotypy (J.L. LeConte 1863b: 71) (type fixation for *Tomarus* J.L. LeConte).

Family-group name: TORAMINAE Sen Gupta, 1967. Stem: *Toram*-.

***Torcus* Casey, 1922**: 315. Current status: valid genus in CURCULIONIDAE: BARIDINAE: APOSTASIMERINI: TORCINA.

Type species: *Torcusfasciatus* Casey, 1922, by original designation.

Family-group name: TORCOCINA Bondar, 1943. Stem: *Torc*-.

***Tormissus* Broun, 1893b**: 1021. Current status: valid genus in HYDROPHILIDAE: CYLOMINAE.

Type species: *Tormissusmagnulus* Broun, 1893 (= *Hydrostygnuslinsi* Sharp, 1884), by subsequent designation (Knisch 1924: 107).

Family-group name: TORMISSINI Hansen, 1991. Stem: *Tormiss*-.

***Torneuma* Wollaston, 1860**: 453. Current status: valid genus in CURCULIONIDAE: CRYPTORHYNCHINAE: TORNEUMATINI.

Type species: *Torneumacaecum* Wollaston, 1860, by monotypy.

Family-group name: TORNEUMATINI Bedel, 1884. Stem: *Torneumat*-.

***Torneutes* Reich, 1838**: 9. Current status: valid genus in CERAMBYCIDAE: CERAMBYCINAE: TORNEUTINI.

Type species: *Torneutespallidipennis* Reich, 1838, by monotypy.

Family-group name: TORNEUTITAE J. Thomson, 1861. Stem: *Torneut*-.

***Torrentomus* Bierig, 1934**: 213. Current status: junior synonym of *Thinobius* Kiesenwetter, 1844 in STAPHYLINIDAE: OXYTELINAE: THINOBIINI.

Type species: *Torrentomustorrei* Bierig, 1934, by original designation.

Family-group name: TORRENTOMI Bierig, 1934. Stem: *Torrentom*-.

***Torridincola* Steffan, 1964**: 193. Current status: valid genus in TORRIDINCOLIDAE: TORRIDINCOLINAE.

Type species: *Torridincolarhodesica* Steffan, 1964, by original designation.

Family-group name: TORRIDINCOLIDAE Steffan, 1964. Stem: *Torridincol*-.

***Tostegoptera* Blanchard in H. Milne-Edwards et al., 1851**: 149. Current status: valid genus in SCARABAEIDAE: MELOLONTHINAE: RHIZOTROGINI.

Type species: *Melolonthalanceolata* Say, 1824, by monotypy.

Family-group name: TOSTEGOPTERAE J.L. LeConte, 1861. Stem: *Tostegopter*-.

***Toxicum* Latreille, 1802b**: 174. Current status: valid genus in TENEBRIONIDAE: TENEBRIONINAE: TOXICINI: TOXICINA.

Type species: *Toxicumrichesianum* Latreille, 1802, by monotypy. Note: combined description of a new genus and a single new species (ICZN 1999a, Article 12.2.6).

Family-group name: TOXICIDEN Oken, 1843. Stem: *Toxic*-.

***Toxidium* J.L. LeConte, 1860b**: 322, 324. Current status: valid genus in STAPHYLINIDAE: SCAPHIDIINAE: SCAPHISOMATINI.

Type species: *Toxidiumgammaroides* J.L. LeConte, 1860, by monotypy.

Family-group name: TOXIDIINI Achard, 1924. Stem: *Toxidi*-.

***Toxoderus* Fauvel, 1900b**: 189. Current status: junior synonym of *Homalotrichus* Solier, 1849 in STAPHYLINIDAE: OXYTELINAE: COPROPHILINI. Note: new replacement name for *Sharpia* Fauvel, 1878.

Type species: *Sharpiabanksi* Fauvel, 1878, by monotypy (type fixation for *Sharpia* Fauvel).

Family-group name: TOXODERI Bernhauer & Schubert, 1911. Stem: *Toxoder*-. Note: junior homonym of TOXODERINI Saussure, 1869 (type genus *Toxodera* Audinet-Serville, 1837) in Mantodea.

***Toxognathus* Fairmaire, 1878**: 271. Current status: valid genus in ELATERIDAE: PHYSODACTYLINAE.

Type species: *Toxognathuscostulatus* Fairmaire, 1878, by monotypy.

Family-group name: TOXOGNATHINAE Fleutiaux, 1941. Stem: *Toxognath*-.

†***Toxorhynchus* Scudder, 1893**: 26. Current status: valid genus in BRENTIDAE: APIONINAE: APIONITAE: APIONINI: TOXORHYNCHINA. Note: Oligocene (Colorado, USA).

Type species: †*Toxorhynchusminusculus* Scudder, 1893, by original designation.

Family-group name: †TOXORHYNCHINI Scudder, 1893. Stem: *Toxorhynch*-.

***Toxoscelus* Deyrolle, 1865**: 127. Current status: valid genus in BUPRESTIDAE: AGRILINAE: CORAEBINI: TOXOSCELINA.

Type species: *Toxoscelusundatus* Deyrolle, 1865, by subsequent designation (Descarpentries and Villiers 1967: 490).

Family-group name: TOXOSCELINA Majer, 2000. Stem: *Toxoscel*-.

***Toxotus* Dejean, 1821**: 112. Current status: junior synonym of *Stenocorus* Geoffroy, 1762 in CERAMBYCIDAE: LEPTURINAE: STENOCORINI.

Type species: *Lepturameridiana* Linnaeus, 1758, by subsequent designation (Westwood 1838c: 41).

Family-group name: TOXOTI J.L. LeConte & G.H. Horn, 1883. Stem: *Toxot*-. Note: junior homonym of TOXOTIDAE Günther, 1860 (type genus *Toxotes* Cloquet, 1816) in Pisces.

***Trabisus* Raffray, 1890a**: 110. Current status: junior synonym of *Atheropterus* Raffray, 1882 in STAPHYLINIDAE: PSELAPHINAE: BATRISITAE: BATRISINI: BATRISINA.

Type species: *Batrisuszanzibaricus* Raffray, 1877, by subsequent designation (Jeannel 1949a: 117).

Family-group name: TRABISINA Jeannel, 1949. Stem: *Trabis*-.

***Trachelizus* Dejean, 1834**: 243. Current status: valid genus in BRENTIDAE: BRENTINAE: TRACHELIZINI: TRACHELIZINA. Note: this name was first made available by Gyllenhal in Schönherr (1833: 334) but in a different sense; an application to the Commission in needed to suppress *Trachelizus* Gyllenhal, 1833 (see Alonso-Zarazaga and Lyal 1999: 56).

Type species: *Brentusbisulcatus* Lund, 1802, by subsequent designation (Schönherr 1840a: 490).

Family-group name: TRACHÉLIZIDES Lacordaire, 1865. Stem: *Tracheliz*-.

***Trachelonia* Gistel, 1850**: 75. Current status: junior synonym of *Collyris* Fabricius, 1801 in CICINDELIDAE: COLLYRIDINI: COLLYRIDINA. Note: unnecessary new replacement name for *Collyris* Fabricius, 1801.

Type species: *Cicindelalongicollis* Fabricius, 1787, by subsequent designation (Latreille 1810: 425) (type fixation for *Collyris* Fabricius).

Family-group name: TRACHELONIADAE Gistel, 1850. Stem: *Tracheloni*-.

***Trachelophorus* Jekel, 1860**: 158. Current status: valid genus in ATTELABIDAE: APODERINAE: TRACHELOPHORINI.

Type species: *Apoderusgiraffa* Jekel, 1860, by subsequent designation (Voss 1925: 5).

Family-group name: TRACHELOPHORINI Voss, 1926. Stem: *Trachelophor*-.

***Trachelostenus* Solier, 1851**: 255. Current status: valid genus in TENEBRIONIDAE: TENEBRIONINAE: TRACHELOSTENINI.

Type species: *Trachelostenusinaequalis* Solier, 1851, by monotypy.

Family-group name: TRACHÉLOSTÉNIDES Lacordaire, 1859. Stem: *Trachelosten*-.

***Trachodes* Germar, 1823**: 325. Current status: valid genus in CURCULIONIDAE: MOLYTINAE: TRACHODINI.

Type species: *Curculiosquamifer* Paykull, 1800 (= *Curculiohispidus* Linnaeus, 1758), by subsequent designation (Schönherr 1825: 584).

Family-group name: TRACHODISIDAE Gistel, 1848. Stem: *Trachod*-.

***Trachyderes* Dalman in Schönherr, 1817**: 364. Current status: valid genus in CERAMBYCIDAE: CERAMBYCINAE: TRACHYDERINI: TRACHYDERINA.

Type species: *Cerambyxsuccinctus* Linnaeus, 1758, by subsequent designation (Blanchard in Audouin et al. 1843: pl. 65).

Family-group name: TRACHYDÉRIDES Dupont, 1836. Stem: *Trachyder*-.

***Trachykele* Marseul, 1865**: 149. Current status: valid genus in BUPRESTIDAE: BUPRESTINAE: BUPRESTINI: TRACHYKELINA.

Type species: *Trachykeleblondeli* Marseul, 1865, by monotypy.

Family-group name: TRACHYKELINA Holyński, 1988. Stem: *Trachykel*-.

***Trachynotus* Latreille, 1828a**: 579. Current status: valid genus in TENEBRIONIDAE: PIMELIINAE: SEPIDIINI: TRACHYNOTINA.

Type species: *Sepidium vittatum* Fabricius, 1781, by subsequent designation (Kamiński et al. 2019: 93).

Family-group name: TRACHYNOTINA C. Koch, 1955. Stem: *Trachynot*-. Note: junior homonym of TRACHYNOTIDAE Schrammen, 1924 (type genus *Trachynotus* Schrammen, 1924) in Porifera and TRACHYNOTIDAE Förster, 1869 (type genus *Trachynotus* Gravenhorst, 1829) in Hymenoptera; both Schrammen and Förster names are permanently invalid (based on a preoccupied type genera).

***Trachypachus* Motschulsky, 1844a**: 86. Current status: valid genus in TRACHYPACHIDAE: TRACHYPACHINAE.

Type species: *Blethisazetterstedtii* Gyllenhal, 1827, by subsequent designation (C.G. Thomson 1859: 3).

Family-group name: TRACHYPACHINI C.G. Thomson, 1857. Stem: *Trachypach*-.

***Trachyphilus* Faust, 1887**: 164. Current status: valid genus in CURCULIONIDAE: ENTIMINAE: CYPHICERITAE: TRACHYPHLOEINI: TRACHYPHILINA.

Type species: *Trachyphilussaluber* Faust, 1887, by monotypy.

Family-group name: TRACHYPHILINA Voss, 1948. Stem: *Trachyphil*-.

***Trachyphloeus* Germar, 1817b**: 341. Current status: valid genus in CURCULIONIDAE: ENTIMINAE: CYPHICERITAE: TRACHYPHLOEINI: TRACHYPHLOEINA.

Type species: *Curculioscabriculus* Linnaeus, 1771 [as *scabricul*], by monotypy.

Family-group name: TRACHYPHLOEIDAE Gistel, 1848. Stem: *Trachyphloe*-.

***Trachypholis* Erichson, 1845a**: 257. Current status: valid genus in ZOPHERIDAE: COLYDIINAE: SYNCHITINI.

Type species: *Opatrumhispidum* Weber, 1801, by monotypy.

Family-group name: TRACHYPHOLINI Grouvelle, 1911. Stem: *Trachypholid*-.

***Trachys* Fabricius, 1801a**: xviii. Current status: valid genus in BUPRESTIDAE: AGRILINAE: TRACHEINI: TRACHEINA. Note: name placed on the Official List of Generic Names in Zoology (ICZN 2009a).

Type species: *Buprestisminuta* Linnaeus, 1758, by subsequent designation (Westwood 1838c: 25; see ICZN 2009a). Note: no species were originally included in this genus by Fabricius (1801a); the first ones were the 11 species listed by Fabricius (1801b: 218– 220); name placed on the Official List of Specific Names in Zoology (ICZN 2009a).

Family-group name: TRACHISIDAE Laporte, 1835. Stem: *Trache*-.

***Trachyscelis* Latreille, 1809a**: 379. Current status: valid genus in TENEBRIONIDAE: DIAPERINAE: TRACHYSCELINI.

Type species: *Trachyscelisaphodioides* Latreille, 1809, by monotypy.

Family-group name: TRACHYSCÉLIDES Blanchard, 1845. Stem: *Trachyscel*-.

***Tragocephala* Dejean, 1835**: 342. Current status: valid genus in CERAMBYCIDAE: LAMIINAE: TRAGOCEPHALINI.

Type species: *Lamiaformosa* G.-A. Olivier, 1792, by subsequent designation (Chevrolat 1849a: 632).

Family-group name: TRAGOCEPHALITES J. Thomson, 1857. Stem: *Tragocephal*-.

***Tragocerus* Latreille, 1829b**: 121. Current status: valid genus in CERAMBYCIDAE: CERAMBYCINAE: TRAGOCERINI.

Type species: *Prionusbidentatus* Donovan, 1805, by subsequent monotypy (Guérin-Méneville 1831b: pl. 45).

Family-group name: TRAGOCERINAE Pascoe, 1867. Stem: *Tragocer*-.

***Tragosoma* Audinet-Serville, 1832**: 159. Current status: valid genus in CERAMBYCIDAE: PRIONINAE: MEROSCELISINI.

Type species: *Cerambyxdepsarius* Linnaeus, 1767, by monotypy.

Family-group name: TRAGOSOMITAE J. Thomson, 1864. Stem: *Tragosomat*-.

***Tranes* Schönherr, 1843**: 129. Current status: valid genus in CURCULIONIDAE: MOLYTINAE: TRANESINI.

Type species: *Tranesvigorsii* Boheman, 1843, by original designation.

Family-group name: TRANESINI Legalov, 2018d: 345. Stem: *Tranes*-.

***Trechicus* J.L. LeConte, 1853e**: 384, 386. Current status: valid subgenus of *Perigona* Laporte, 1834 in CARABIDAE: HARPALINAE: PERIGONINI.

Type species: *Trechicusumbripennis* J.L. LeConte, 1853 (= *Bembidiumnigriceps* Dejean, 1831), by subsequent designation (Jeannel 1926: 247).

Family-group name: TRECHICHINAE H.W. Bates, 1873. Stem: *Trechic*-. Note: nomen oblitum (see Bouchard et al. 2011: 140).

***Trechodes* Blackburn, 1901**: 119. Current status: valid genus in CARABIDAE: TRECHINAE: TRECHINI: TRECHODINA.

Type species: *Bembidiumsecalioides* Blackburn, 1891, by subsequent designation (Jeannel 1926: 484).

Family-group name: TRECHODINI Jeannel, 1926. Stem: *Trechod*-. Note: the recent placement of *Perileptus* Schaum, 1860 within the valid subtribe TRECHODINA by Maddison et al. (2019: 173) creates a priority problem as PERILEPTINA Sloane, 1903 is the oldest available family-group name for this subtribe.

***Trechus* Clairville, 1806**: 22. Current status: valid genus in CARABIDAE: TRECHINAE: TRECHINI: TRECHINA.

Type species [suggested]: *Carabusquadristriatus* Schrank, 1781, misidentified as *Carabusrubens* Fabricius, 1792 (sensu Clairville, 1806) in the subsequent designation by Blanchard in Audouin et al. (1841: pl. 25). Note: the valid type species fixation is that of Latreille (1810: 426), who selected *Carabusmeridianus* Linnaeus, 1761, but this is currently the type species of the genus *Acupalpus* Latreille, 1829; a new application to the Commission is necessary to fix *Carabusquadristriatus* Schrank, 1781 as the type species of *Trechus* Clairville, 1806 since a previous request was postponed (ICZN 1950: 447).

Family-group name: TRECHII Bonelli, 1810. Stem: *Trech*-.

***Tretothorax* Lea, 1910**: 211. Current status: valid genus in SALPINGIDAE: DACODERINAE.

Type species: *Tretothoraxcleistostoma* Lea, 1910, by monotypy.

Family-group name: TRETOTHORACIDAE Lea, 1910. Stem: *Tretothorac*-.

***Triacrus* Nordmann, 1837**: 5, 19. Current status: valid genus in STAPHYLINIDAE: STAPHYLININAE: STAPHYLININI: XANTHOPYGINA.

Type species: *Triacrusdilatus* Nordmann, 1837, by monotypy.

Family-group name: TRIACRI Bernhauer, 1931. Stem: *Triacr*-.

†***Triadocupes* Ponomarenko, 1966**: 51. Current status: valid genus in †TRIADOCUPEDIDAE. Note: Upper Triassic (Kyrgyzstan).

Type species: †*Triadocupesferghanensis* Ponomarenko, 1966, by original designation.

Family-group name: †TRIADOCUPEDINAE Ponomarenko, 1966. Stem: *Triadocuped*-.

†***Triamyxa* Qvarnström, Fikáček, Wernström, Huld, Beutel, Arriaga-Varela, Ahlberg & Niedźwiedzki, 2021**: 3378. Current status: valid genus in HYDROSCAPHIDAE: †TRIAMYXINAE. Note: Triassic (Poland).

Type species: †*Triamyxacoprolithica*Qvarnström et al., 2021, by original designation.

Family-group name: †TRIAMYXIDAE Qvarnström, Fikáček, Wernström, Huld, Beutel, Arriaga-Varela, Ahlberg & Niedźwiedzki, 2021: 3376. Stem: *Triamyx*-.

†***Triaplus* Ponomarenko, 1977**: 17. Current status: valid genus in †TRIAPLIDAE. Note: Permian/Triassic (Kyrgyzstan; Russia).

Type species: †*Triaplusmacroplatus* Ponomarenko, 1977, by original designation.

Family-group name: †TRIAPLIDAE Ponomarenko, 1977. Stem: *Triapl*-.

***Triarthron* Märkel, 1840**: 141. Current status: valid genus in LEIODIDAE: LEIODINAE: SOGDINI.

Type species: *Triarthronmaerkelii* W.L.E. Schmidt, 1840, by monotypy.

Family-group name: TRIARTHRINI Jeannel, 1962. Stem: *Triarthr*-. Note: junior homonym of TRIARTHRIDAE Ulrich, 1930 (type genus *Triarthrus* Green, 1832) in Trilobita.

***Tribalus* Erichson, 1834**: 164. Current status: valid genus in HISTERIDAE: TRIBALINAE.

Type species: *Histercapensis* Paykull, 1811, by subsequent designation (Hope 1841a: 105).

Family-group name: TRIBALINI Bickhardt, 1914. Stem: *Tribal*-.

***Tribax* Fischer, 1817**: 463. Current status: valid subgenus of *Carabus* Linnaeus, 1758 in CARABIDAE: CARABINAE: CARABINI: CARABINA. Note: this name was originally proposed as *Tribacis*, which is the genitive of *Tribax*; according to Article 11.8.1 (ICZN 1999a) the name, if it meets the other requirements of availability, is available but must corrected to the nominative singular.

Type species: *Carabuspuschkini* Adams, 1817, by subsequent designation (Breuning 1934: 1033).

Family-group name: TRIBACOGENICI Csiki, 1927. Stem: *Tribac*-.

***Tribolium* W.S. MacLeay, 1825**: 47. Current status: valid genus in TENEBRIONIDAE: TENEBRIONINAE: TRIBOLIINI. Note: name placed on the Official List of Generic Names in Zoology (ICZN 1988e).

Type species: *Colydiumcastaneum* Herbst, 1797, by monotypy (see ICZN 1988e). Note: name placed on the Official List of Specific Names in Zoology (ICZN 1988e).

Family-group name: TRIBOLIIDAE Gistel, 1848. Stem: *Triboli*-.

***Trichalus* C.O. Waterhouse, 1877**: 82. Current status: valid genus in LYCIDAE: METRIORRHYNCHINAE: METRIORRHYNCHINI: METRIORRHYNCHINA.

Type species: *Trichalusflavopictus* C.O. Waterhouse, 1877, by subsequent designation (C.O. Waterhouse 1878: 103).

Family-group name: TRICHALINAE Kleine, 1929. Stem: *Trichal*-.

***Trichapion* Wagner, 1912a**: 99, 116. Current status: valid genus in BRENTIDAE: APIONINAE: APIONITAE: APIONINI: SYNAPIINA.

Type species: *Apionaurichalceum* Wagner, 1912, by subsequent designation (Kissinger 1959: 248).

Family-group name: TRICHAPIINA Alonso-Zarazaga, 1990. Stem: *Trichapi*-.

***Trichelodes* Carter, 1935**: 189. Current status: valid genus in DERMESTIDAE: TRINODINAE: TRICHELODINI.

Type species: *Trichelodesdelicatula* Carter, 1935, by monotypy.

Family-group name: TRICHELODINI Peacock, 1978. Stem: *Trichelod*-.

***Trichinorhipis* Barr, 1948**: 70. Current status: valid genus in BUPRESTIDAE: BUPRESTINAE: XENORHIPIDINI: TRICHINORHIPIDINA.

Type species: *Trichinorhipisknulli* Barr, 1948, by original designation.

Family-group name: TRICHINORHIPIDINA Bellamy, 2006. Stem: *Trichinorhipid*-.

***Trichis* Klug, 1832**: pl. xxi. Current status: valid genus in CARABIDAE: HARPALINAE: LEBIINI: TRICHINA.

Type species: *Trichispallida* Klug, 1832, by subsequent designation (Andrewes 1938: 138).

Family-group name: TRICHINI Basilewsky, 1984. Stem: *Trich*-.

***Trichius* Fabricius, 1775**: 40. Current status: valid genus in SCARABAEIDAE: CETONIINAE: TRICHIINI. Note: name placed on the Official List of Generic Names in Zoology (ICZN 2004d).

Type species: *Scarabaeusfasciatus* Linnaeus, 1758, by subsequent designation (Latreille 1810: 428; see ICZN 2004d). Note: name placed on the Official List of Specific Names in Zoology (ICZN 2004d).

Family-group name: TRICHIADAE Fleming, 1821. Stem: *Trichi*-.

***Trichochrysea* Baly, 1861**: 195. Current status: valid genus in CHRYSOMELIDAE: EUMOLPINAE: BROMIINI.

Type species: *Trichochryseamouhoti* Baly, 1861, by original designation.

Family-group name: TRICHOCHRYSEINI Clavareau, 1914. Stem: *Trichochryse*-.

***Trichodes* Herbst, 1792**: 154. Current status: valid genus in CLERIDAE: CLERINAE: OPILONITAE: DIEROPSEINI. Note: name placed on the Official List of Generic Names in Zoology (ICZN 1984a).

Type species: *Attelabusapiarius* Linnaeus, 1758, by subsequent designation (Hope 1841a: 137; see ICZN 1984a). Note: name placed on the Official List of Specific Names in Zoology (ICZN 1984a).

Family-group name: TRICHODIDAE Streubel, 1839. Stem: *Trichod*-. Note: as pointed out by Bouchard and Bousquet (2020a: 92), TRICHODIDAE Streubel, 1839 is an available family- group name and has priority over DIEROPSEINI J.R. Winkler, 1964 based on the recent classification of Bartlett (2021), an application to the Commission is needed to conserve DIEROPSEINI J.R. Winkler, 1964 as the valid name for this tribe; TRICHODINI Streubel, 1839 is a senior homonym of TRICHODINA van der Hoeven, 1849 (type genus *Trichoda* O.F. Müller, 1773) in Protozoa: Ciliophora.

***Trichodocerus* Chevrolat, 1879**: 123. Current status: valid genus in CURCULIONIDAE: CONODERINAE: TRICHODOCERINI.

Type species: *Trichodocerusspinolae* Chevrolat, 1879, by subsequent designation (Champion 1906: 713).

Family-group name: TRICHODOCERIDES Champion, 1906. Stem: *Trichodocer*-.

***Trichomesia* Pascoe, 1859a**: 18. Current status: valid genus in CERAMBYCIDAE: CERAMBYCINAE: TRICHOMESIINI.

Type species: *Trichomesianewmani* Pascoe, 1859, by monotypy.

Family-group name: TRICHOMESIINI Aurivillius, 1912. Stem: *Trichomesi*-.

***Trichonyx* Chaudoir, 1845**: 164. Current status: valid genus in STAPHYLINIDAE: PSELAPHINAE: EUPLECTITAE: TRICHONYCHINI: TRICHONYCHINA.

Type species: *Pselaphussulcicollis* H.G.L. Reichenbach, 1816, by monotypy.

Family-group name: TRICHONYIDES Reitter, 1882. Stem: *Trichonych*-.

***Trichophya* Mannerheim, 1830**: 73. Current status: valid genus in STAPHYLINIDAE: TRICHOPHYINAE.

Type species: *Aleocharapilicornis* Gyllenhal, 1810, by monotypy.

Family-group name: TRICHOPHYINI C.G. Thomson, 1858. Stem: *Trichophy*-.

***Trichoplus* Burmeister, 1842**: 660. Current status: valid genus in SCARABAEIDAE: CETONIINAE: CREMASTOCHEILINI: TRICHOPLINA.

Type species: *Cremastocheiluslaevis* Gory & Percheron, 1833, by monotypy.

Family-group name: TRICHOPLINA Krikken, 1984. Stem: *Trichopl*-.

***Trichopselaphus* Chaudoir, 1843**: 399. Current status: valid genus in CARABIDAE: HARPALINAE: HARPALINI: HARPALINA.

Type species: *Trichopselaphussubiridescens* Chaudoir, 1843, by monotypy.

Family-group name: TRICHOPSELAPHINI Tschitschérine, 1900. Stem: *Trichopselaph*-.

***Trichopsenius* G.H. Horn, 1877**: 88. Current status: valid genus in STAPHYLINIDAE: ALEOCHARINAE: TRICHOPSENIINI.

Type species: *Hypocyptusdepressus* J.L. LeConte, 1863, by monotypy.

Family-group name: TRICHOPSENII J.L. LeConte & G.H. Horn, 1883. Stem: *Trichopseni*-.

***Trichopteryx* W. Kirby in W. Kirby and Spence, 1826**: 41. Current status: invalid senior synonym of *Acrotrichis* Motschulsky, 1848 in PTILIIDAE: PTILIINAE: ACROTRICHINI. Note: junior homonym of *Trichopteryx* Hübner, 1825 [Lepidoptera].

Type species: *Dermestesatomarius* DeGeer, 1774, by monotypy.

Family-group name: TRICHOPTERYGIA Erichson, 1845. Stem: *Trichopteryg*-. Note: family-group name permanently invalid (ICZN 1999a, Article 39).

***Trichotichnus* Morawitz, 1863**: 63. Current status: valid genus in CARABIDAE: HARPALINAE: HARPALINI: HARPALINA. Note: nomen protectum (see Bousquet 2008b: 328).

Type species: *Trichoptichnuslongitarsis* Morawitz, 1863, by monotypy.

Family-group name: TRICHOTICHNINI Jeannel, 1942. Stem: *Trichotichn*-.

†***Tricoleus* Ponomarenko, 1969a**: 139. Current status: valid genus in †TRICOLEIDAE. Note: Jurassic (Kazakhstan).

Type species: †*Tricoleuspunctatus* Ponomarenko, 1969, by original designation.

Family-group name: †TRICOLEIDAE Ponomarenko, 1969. Stem: *Tricole*-.

***Tricondyla* Latreille in Latreille and Dejean, 1822**: 65. Current status: valid genus in CICINDELIDAE: COLLYRIDINI: TRICONDYLINA.

Type species: *Cicindelaaptera* G.-A. Olivier, 1791, by monotypy.

Family-group name: TRICONDYLINA Naviaux, 1991. Stem: *Tricondyl*-.

***Tricorynus* G.R. Waterhouse, 1849**: lxviii. Current status: valid genus in PTINIDAE: MESOCOELOPODINAE: TRICORYNINI.

Type species: *Tricorynuszeae* G.R. Waterhouse, 1849, by monotypy.

Family-group name: TRICORYNINAE R.E. White, 1971. Stem: *Tricoryn*-.

***Tricrania* J.L. LeConte, 1860a**: 320. Current status: valid genus in MELOIDAE: NEMOGNATHINAE: NEMOGNATHINI: NEMOGNATHINA.

Type species: *Horiasanguinipennis* Say, 1824, by subsequent designation (Wellman 1910b: 219).

Family-group name: TRICRANIIDES Wellman, 1910. Stem: *Tricrani*-.

***Trictenotoma* G.R. Gray in Griffith and Pidgeon, 1831**: 534, pl. 5. Current status: valid genus in TRICTENOTOMIDAE.

Type species: *Trictenotomachildreni* G.R. Gray, 1831, by monotypy.

Family-group name: TRICTÉNOTOMIDES Blanchard, 1845. Stem: *Trictenotom*-.

***Trientoma* Solier, 1835c**: 253, 256. Current status: valid genus in TENEBRIONIDAE: PIMELIINAE: EDROTINI.

Type species: *Trientomavarvasi* Solier, 1835, by monotypy.

Family-group name: TRIENTOMINI Casey, 1907. Stem: *Trientom*-.

***Trigonarthron* Boppe, 1913**: 403. Current status: valid genus in CERAMBYCIDAE: CERAMBYCINAE: TRIGONARTHRINI.

Type species: *Trigonarthroncinnabarinum* Boppe, 1913, by monotypy.

Family-group name: TRIGONARTHRINI Villiers, 1984. Stem: *Trigonarthr*-.

***Trigonobregma* Scheerpeltz, 1944**: 170. Current status: valid genus in STAPHYLINIDAE: OXYTELINAE incertae sedis.

Type species: *Thinobiusocularis* Fauvel, 1902, by monotypy.

Family-group name: TRIGONOBREGMINI Scheerpeltz, 1944. Stem: *Trigonobregmat*-.

***Trigonocolus* Lacordaire, 1863**: 593. Current status: valid genus in CURCULIONIDAE: MOLYTINAE: TRIGONOCOLINI. Note: new replacement name for *Megarhinus* Schönherr, 1835.

Type species: *Megarhinusfirmus* Gyllenhal, 1835, by original designation (type fixation for *Megarhinus* Schönherr).

Family-group name: TRIGONOCOLIDES Lacordaire, 1863. Stem: *Trigonocol*-.

***Trigonodactyla* Dejean, 1831**: 278, 288. Current status: junior synonym of *Hexagonia* W. Kirby, 1825 in CARABIDAE: HARPALINAE: HEXAGONIINI.

Type species: *Trigonodactylaterminata* Dejean, 1831 (= *Hexagoniaterminalis* Gemminger & Harold, 1868), by subsequent designation (Hope 1838a: 104).

Family-group name: TRIGONODACTYLIENS Brullé, 1834. Stem: *Trigonodactyl*-.

***Trigonogenium* Harold, 1869b**: 123. Current status: valid genus in BUPRESTIDAE: BUPRESTINAE: TRIGONOGENIINI. Note: new replacement name for *Trigonophorus* Solier, 1849.

Type species: *Trigonophorusangulosus* Solier, 1849, by monotypy (type fixation for *Trigonophorus* Solier).

Family-group name: TRIGONOGENIINI Cobos, 1956. Stem: *Trigonogeni*-.

***Trigonognatha* Motschulsky, 1857a**: 25. Current status: valid subgenus of *Myas* Sturm, 1826 in CARABIDAE: HARPALINAE: PTEROSTICHINI: PTEROSTICHINA.

Type species: *Trigonognathacuprescens* Motschulsky, 1857, by monotypy.

Family-group name: TRIGONOGNATHIDES Tschitschérine, 1898. Stem: *Trigonognath*-.

***Trigonoptera* Perroud, 1855a**: 336. Current status: valid genus in CERAMBYCIDAE: LAMIINAE: TMESISTERNINI.

Type species: *Trigonopteramaculata* Perroud, 1855, by monotypy.

Family-group name: TRIGONOPTERINI Aurivillius, 1922. Stem: *Trigonopter*-.

***Trigonopus* Mulsant & Rey, 1853d**: 105. Current status: valid genus in TENEBRIONIDAE: BLAPTINAE: PLATYNOTINI: PLATYNOTINA.

Type species: *Trigonopuscapicola* Mulsant & Rey, 1853, by subsequent designation (Cazurro Ruiz 1897b: 539).

Family-group name: TRIGONOPAIRES Mulsant & Rey, 1853. Stem: *Trigonopod*-.

***Trigonorhinus* Wollaston, 1861a**: 102. Current status: valid genus in ANTHRIBIDAE: ANTHRIBINAE: ANTHRIBINI.

Type species: *Trigonorhinuspardalis* Wollaston, 1861 (= *Brachytarsusareolatus* Boheman, 1845), by monotypy.

Family-group name: TRIGONORHININI B.D. Valentine, 1999. Stem: *Trigonorhin*-.

***Trigonoscuta* Motschulsky, 1853b**: 79. Current status: valid genus in CURCULIONIDAE: ENTIMINAE: POLYDRUSITAE: PSALLIDIINI.

Type species: *Trigonoscutapilosa* Motschulsky, 1853, by monotypy.

Family-group name: TRIGONOSCUTAE J.L. LeConte, 1874. Stem: *Trigonoscut*-.

***Trigonostomus* Brenske, 1893**: 1. Current status: valid genus in SCARABAEIDAE: RUTELINAE: ADORETINI: TRIGONOSTOMUSINA. Note: this name was deemed available from Brenske, 1893, not invalid by reason of being an incorrect subsequent spelling of *Trigonostomum* Burmeister, 1844 and placed on the Official List of Generic Names in Zoology (ICZN 2009b).

Type species: *Trigonostomusmucoreum* Burmeister, 1844, by plenary power (see ICZN 2009b). Note: name placed on the Official List of Specific Names in Zoology (ICZN 2009b).

Family-group name: TRIGONOSTOMINA Ohaus, 1912. Stem: *Trigonostomus*- (ICZN 2009b).

***Trigonotoma* Dejean, 1828**: 4, 182. Current status: valid genus in CARABIDAE: HARPALINAE: PTEROSTICHINI: PTEROSTICHINA.

Type species: **here fixed** (under ICZN 1999a, Article 70.3.2) as *Trigonotomaindica* Brullé, 1834, misidentified as *Omaseusviridicollis* W.S. MacLeay, 1825 (sensu Dejean, 1825) in the subsequent designation by Hope (1838a: 111).

Family-group name: TROGONOTOMIDAE Laporte, 1834. Stem: *Trigonotom*-.

***Trigonurus* Mulsant, 1848**: 515. Current status: valid genus in STAPHYLINIDAE: TRIGONURINAE.

Type species: *Trigonurusmellyi* Mulsant, 1848, by monotypy.

Family-group name: TRIGONURIDES Reiche, 1866. Stem: *Trigonur*-.

***Trilobitideus* Raffray, 1898b**: 351. Current status: valid genus in STAPHYLINIDAE: ALEOCHARINAE: TRILOBITIDEINI.

Type species: *Trilobitideusmirabilis* Raffray, 1898, by monotypy.

Family-group name: TRILOBITIDEIDAE Fauvel, 1899. Stem: *Trilobitide*-.

***Trilobocara* Solier, 1851**: 129. Current status: valid genus in TENEBRIONIDAE: PIMELIINAE: TRILOBOCARINI.

Type species: *Trilobocaraciliatum* Solier, 1851, by original designation.

Family-group name: TRIBOLOCARIDES Lacordaire, 1859. Stem: *Trilobocar*-.

***Trimerus* Chaudoir, 1878b**: 89. Current status: invalid senior synonym of *Amorphomerus* Sloane, 1923 in CARABIDAE: HARPALINAE: AMORPHOMERINI. Note: junior homonym of *Trimerus* Green, 1832 [Trilobita].

Type species: *Trimerusraffrayi* Chaudoir, 1878, by monotypy.

Family-group name: TRIMERINAE Alluaud, 1922. Stem: *Trimer*-. Note: family-group name permanently invalid (ICZN 1999a, Article 39).

***Trimiodytes* Raffray, 1897b**: 52. Current status: valid genus in STAPHYLINIDAE: PSELAPHINAE: EUPLECTITAE: TRICHONYCHINI: TRICHONYCHINA.

Type species: *Trimiodytespalustris* Raffray, 1897, by monotypy.

Family-group name: TRIMIODYTINA Jeannel, 1964. Stem: *Trimiodyt*-.

***Trimium* Aubé, 1833**: 44. Current status: valid genus in STAPHYLINIDAE: PSELAPHINAE: EUPLECTITAE: TRICHONYCHINI: TRIMIINA.

Type species: *Pselaphusbrevicornis* H.G.L. Reichenbach, 1816, by monotypy.

Family-group name: TRIMIINI Brendel & Wickham, 1890. Stem: *Trimi*-.

***Trimytis* J.L. Leconte, 1851a**: 141. Current status: valid genus in TENEBRIONIDAE: PIMELIINAE: EDROTINI.

Type species: *Trimytispruinosa* J.L. LeConte, 1851, by monotypy.

Family-group name: TRIMYTINI Casey, 1907. Stem: *Trimytid*-.

***Trinodes* Dejean, 1821**: 47. Current status: valid genus in DERMESTIDAE: TRINODINAE: TRINODINI.

Type species: *Nitidulahirta* Fabricius, 1781, by monotypy.

Family-group name: TRINODINI Casey, 1900. Stem: *Trinod*-.

***Trinoparvus* Háva, 2004**: 152, 155. Current status: valid genus in DERMESTIDAE: TRINODINAE: TRINODINI.

Type species: *Trinoparvusvillosus* Háva, 2004, by original designation.

Family-group name: TRINOPARVINI Háva, 2010. Stem: *Trinoparv*-.

***Triodonyx* Saylor, 1942**: 158. Current status: valid genus in SCARABAEIDAE: MELOLONTHINAE: RHIZOTROGINI.

Type species: *Phyllophagagigantissima* Saylor, 1935, by original designation.

Family-group name: TRIODONINA Rivera-Gasperín & Morón, 2013: 816. Stem: *Triodon*-.

***Triorophus* J.L. LeConte, 1851a**: 141. Current status: valid genus in TENEBRIONIDAE: PIMELIINAE: EDROTINI.

Type species: *Triorophuslaevis* J.L. LeConte, 1851, by subsequent designation (Casey 1907: 432).

Family-group name: TRIOROPHI J.L. LeConte & G.H. Horn, 1883. Stem: *Trioroph*-.

***Triphyllus* Dejean, 1821**: 102. Current status: valid genus in MYCETOPHAGIDAE: MYCETOPHAGINAE: MYCETOPHAGINI.

Type species: *Mycetophaguspunctatus* Fabricius, 1792 (= *Nitidulabicolor* Fabricius, 1777), by subsequent designation (Westwood 1838c: 14).

Family-group name: TRIPHYLLIDAE Crotch, 1873. Stem: *Triphyll*-.

***Triplax* Herbst, 1793**: 146. Current status: valid genus in EROTYLIDAE: EROTYLINAE: TRITOMINI.

Type species: *Silpharussica* Linnaeus, 1758, by subsequent designation (Curtis 1838a: pl. 706).

Family-group name: TRIPLACINAE Streubel, 1839. Stem: *Triplac*-.

***Trisignis* Park & Schuster, 1955**: 1. Current status: valid genus in STAPHYLINIDAE: PSELAPHINAE: EUPLECTITAE: TRICHONYCHINI: PANAPHANTINA.

Type species: *Trisignismarshi* Park & Schuster, 1955, by original designation.

Family-group name: TRISIGNINA Park & Schuster, 1955. Stem: *Trisign*-.

***Tristaria* Reitter, 1878a**: 320. Current status: valid genus in BOSTRICHIDAE: LYCTINAE: TROGOXYLINI.

Type species: *Tristariagrouvellei* Reitter, 1878, by subsequent designation (Lesne 1911: 206).

Family-group name: TRISTARIINI Lesne, 1921. Stem: *Tristari*-.

†***Tritarsus* Hong, 2002**: 105. Current status: valid genus in †TRITARSUSIDAE. Note: Eocene (China).

Type species: †*Tritarsuslatus* Hong, 2002, by original designation.

Family-group name: †TRITARSUSIDAE Hong, 2002. Stem: *Tritarsus*- (ICZN 1999a, Article 29.4).

***Tritoma* Fabricius, 1775**: 68. Current status: valid genus in EROTYLIDAE: EROTYLINAE: TRITOMINI. Note: the senior homonym *Tritoma* Geoffroy, 1762 (and all other uses of the name prior to the publication of *Tritoma* Fabricius, 1775) was suppressed for both Principles of Priority and Homonymy and placed on the Official Index of Rejected and Invalid Generic Names in Zoology while *Tritoma* Fabricius, 1775 was placed on the Official List of Generic Names in Zoology (ICZN 1994a).

Type species: *Tritomabipustulata* Fabricius, 1775, by subsequent designation (Latreille 1810: 432; see ICZN 1994a). Note: name placed on the Official List of Specific Names in Zoology (ICZN 1994a).

Family-group name: TRITOMIDAE Curtis, 1834. Stem: *Tritom*-.

***Tritoma* Geoffroy, 1762**: 335. Current status: invalid senior synonym of *Mycetophagus* Fabricius, 1792 in MYCETOPHAGIDAE: MYCETOPHAGINAE: MYCETOPHAGINI. Note: name suppressed for both Principles of Priority and Homonymy and placed on the Official Index of Rejected and Invalid Names in Zoology (see ICZN 1994a).

Type species: to be determined.

Family-group name: TRITOMIDAE Crotch, 1873. Stem: *Tritom*-. Note: family-group name permanently invalid (ICZN 1999a, Article 39).

***Trixagus* Kugelann, 1794**: 534. Current status: valid genus in THROSCIDAE.

Type species: *Dermestesadstrictor* Herbst, 1792 (= *Elaterdermestoides* Linnaeus, 1767), by subsequent designation (Barber 1942: 8, 32).

Family-group name: TRIXAGIDAE Gistel, 1848. Stem: *Trixag*-.

***Trochalus* Laporte, 1832c**: pl. 44. Current status: valid genus in SCARABAEIDAE: SERICINAE: SERICINI: TROCHALINA.

Type species: *Trochalusrotundatus* Laporte, 1832, by monotypy.

Family-group name: TROCHALINAE Brenske, 1898. Stem: *Trochal*-.

***Trochoideus* Westwood, 1833**: 673. Current status: valid genus in ENDOMYCHIDAE: PLEGANOPHORINAE.

Type species: *Paussuscruciatus* Dalman, 1826, by monotypy.

Family-group name: TROCHOIDÉITES Chapuis, 1876. Stem: *Trochoide*-.

***Trogaster* Sharp, 1874b**: 83. Current status: valid genus in STAPHYLINIDAE: PSELAPHINAE: EUPLECTITAE: TROGASTRINI: TROGASTRINA.

Type species: *Trogasteraberrans* Sharp, 1874, by monotypy.

Family-group name: TROGASTERINI Brendel & Wickham, 1890. Stem: *Trogastr*-.

***Trogloderus* J.L. LeConte, 1879**: 2. Current status: valid genus in TENEBRIONIDAE: BLAPTINAE: AMPHIDORINI.

Type species: *Trogloderuscostatus* J.L. LeConte, 1879, by monotypy.

Family-group name: TROGLODERINA La Rivers, 1948. Stem: *Trogloder*-.

***Troglops* Erichson, 1840b**: 125. Current status: valid genus in MELYRIDAE: MALACHIINAE: MALACHIINI.

Type species: *Cantharisalbicans* Linnaeus, 1767, by subsequent designation (Chevrolat 1849a: 700).

Family-group name: TROGLOPATES Mulsant & Rey, 1867. Stem: *Troglop*-.

***Trogocryptus* Sharp, 1900**: 602. Current status: valid genus in SALPINGIDAE: PROSTOMINIINAE.

Type species: *Trogocryptusnigripectus* Sharp, 1900, by monotypy.

Family-group name: TROGOCRYPTINAE Lawrence, 1991. Stem: *Trogocrypt*-.

***Trogoderma* Dejean, 1821**: 46. Current status: valid genus in DERMESTIDAE: MEGATOMINAE: MEGATOMINI: TROGODERMINA.

Type species: *Anthrenuselongatulus* Fabricius, 1801 (= *Anthrenusglaber* Herbst, 1783), by subsequent designation (Hope 1841a: 143).

Family-group name: TROGODERMATES Mulsant & Rey, 1867. Stem: *Trogoderm*-. Note: this name was treated as unavailable by Bouchard et al. (2011: 335); however, it has been used in latinized form and generally accepted since (e.g., Háva 2015: xxii, Zhou et al. 2022: 18).

***Trogodes* Boheman, 1857**: 54. Current status: valid genus in SCARABAEIDAE: CETONIINAE: CREMASTOCHEILINI: TROGODINA. Note: several authors credit the authorship of this name to Westwood since the name *Trogodes* in the original description is followed by “(Westw.)”; however, the signature at the end of the combined description of the genus and species is “BHN” [Boheman] and we believe that Boheman should be credited with both the genus-group and species-group names (see Bouchard and Bousquet 2020a: 83, incorrectly credited to “Boheman, 1851” instead of “Boheman, 1857”).

Type species: *Trogodesrotundicollis* Boheman, 1857, by monotypy.

Family-group name: TROGODINA Krikken, 1984. Stem: *Trogod*-.

***Trogoparvus* Háva, 2002**: 27. Current status: valid genus in DERMESTIDAE: TROGOPARVINAE.

Type species: *Trogoparvussumatrensis* Háva, 2002, by original designation.

Family-group name: TROGOPARVINAE Zhou, Nicholls, Liu, Hartley, Szito, Ślipiński & Zwick, 2022: 14. Stem: *Trogoparv*-.

***Trogophloeus* Mannerheim, 1830**: 49. Current status: valid subgenus of *Carpelimus* Leach, 1819 in STAPHYLINIDAE: OXYTELINAE: OXYTELINI.

Type species: *Oxyteluscorticinus* Gravenhorst, 1806, by monotypy.

Family-group name: TROGOPHLÉAIRES Mulsant & Rey, 1878. Stem: *Trogophloe*-.

***Trogossita* G.-A. Olivier, 1790a**: [19] 5. Current status: junior synonym of *Tenebroides* Piller & Mitterpacher, 1783 in TROGOSSITIDAE: TROGOSSITINAE: TROGOSSITINI.

Type species: *Tenebriomauritanicus* Linnaeus, 1758, by subsequent designation (Partington 1837: 816).

Family-group name: TROGOSSITARII Latreille, 1802. Stem: *Trogossit*-.

***Trogoxylon* J.L. LeConte, 1862**: 209. Current status: valid genus in BOSTRICHIDAE: LYCTINAE: TROGOXYLINI.

Type species: *Xylotrogusparallelipipedus* Melsheimer, 1844, by original designation.

Family-group name: TROGOXYLINI Lesne, 1921. Stem: *Trogoxyl*-.

***Tropicus* M.F. Pacheco, 1964**: 102. Current status: valid genus in HETEROCERIDAE: HETEROCERINAE: TROPICINI.

Type species: *Heteroceruspusillus* Say, 1823, by original designation.

Family-group name: TROPICINI M.F. Pacheco, 1964. Stem: *Tropic*-.

***Tropideres* Schönherr, 1823**: 1135. Current status: valid genus in ANTHRIBIDAE: ANTHRIBINAE: TROPIDERINI.

Type species: *Curculioalbirostris* Schaller, 1783, by original designation.

Family-group name: TROPIDÉRIDES Lacordaire, 1865. Stem: *Tropider*-.

***Tropidion* J. Thomson, 1867**: 138. Current status: valid genus in CERAMBYCIDAE: CERAMBYCINAE: TROPIDINI: TROPIDINA.

Type species: *Ibidionflavipes* J. Thomson, 1867, by subsequent designation (Martins 1968: 355).

Family-group name: TROPIDINA Martins & Galileo, 2007. Stem: *Tropid*-.

***Tropidosoma* Perty, 1830**: 15. Current status: junior synonym of *Allocerus* Lacordaire, 1830 in CERAMBYCIDAE: CERAMBYCINAE: TRACHYDERINI: TRACHYDERINA.

Type species: *Prionusspencei* W. Kirby, 1818, by monotypy.

Family-group name: TROPIDOSOMITAE J. Thomson, 1864. Stem: *Tropidosomat*-.

***Tropiphorus* Schönherr, 1842a**: 257. Current status: valid genus in CURCULIONIDAE: ENTIMINAE: ENTIMITAE: BYRSOPAGINI: TROPIPHORINA. Note: name placed on the Official List of Generic Names in Zoology (ICZN 1988c).

Type species: *Curculiomercurialis* Fabricius, 1801 (= *Curculioelevatus* Herbst, 1795), by original designation (see ICZN 1988c). Note: *Curculiomercurialis* Fabricius, 1801 was placed on the Official List of Specific Names in Zoology (ICZN 1988c).

Family-group name: TROPIPHORIDAE Marseul, 1863. Stem: *Tropiphor*-.

***Tropocalymma* J. Thomson, 1864**: 138. Current status: valid genus in CERAMBYCIDAE: CERAMBYCINAE: TROPOCALYMMATINI.

Type species: *Tropisdimidiata* Newman, 1841, by original designation.

Family-group name: TROPOCALYMMIDES Lacordaire, 1868. Stem: *Tropocalymmat*-.

***Tropopterus* Solier, 1849**: 211. Current status: valid genus in CARABIDAE: PSYDRINAE: MORIOMORPHINI: TROPOPTERINA.

Type species: *Tropopterusgiraudyi* Solier, 1849, by subsequent designation (Cazurro Ruiz 1897b: 641).

Family-group name: TROPOPTERIDES Sloane, 1898. Stem: *Tropopter*-.

***Trotommidea* Reitter, 1883b**: 307. Current status: valid genus in SCRAPTIIDAE: SCRAPTIINAE: SCRAPTIINI.

Type species: *Trotommideasalonae* Reitter, 1883, by monotypy.

Family-group name: TROTOMMIDEINI Pic, 1903. Stem: *Trotommide*-.

***Trox* Fabricius, 1775**: 31. Current status: valid genus in TROGIDAE: TROGINAE.

Type species: *Scarabaeussabulosus* Linnaeus, 1758, by subsequent designation (Latreille 1810: 428).

Family-group name: TROGIDAE W.S. MacLeay, 1819. Stem: *Trog*-.

***Trypanaeus* Eschscholtz, 1829b**: 11. Current status: valid genus in HISTERIDAE: ABRAEINAE: TRYPANAEINI.

Type species [suggested]: *Bostrichusthoracicus* Fabricius, 1801, by subsequent designation (Bickhardt 1916: 43). Note: the valid type species fixation is that of Hope (1841a: 105), who selected *Bostrichusproboscideus* Fabricius, 1801, but that species is the type species of the genus *Coptotrophis* Lewis, 1902; an application to the Commission is necessary to fix *Bostrichusthoracicus* Fabricius, 1801 as the type species of *Trypanaeus* Eschscholtz, 1829.

Family-group name: TRYPANÉENS Marseul, 1857. Stem: *Trypanae*-.

***Trypanidius* Blanchard, 1842c**: pl. 22. Current status: valid genus in CERAMBYCIDAE: LAMIINAE: ACANTHOCININI.

Type species: *Trypanidiusandicola* Blanchard, 1842, by monotypy.

Family-group name: TRYPANIDIITAE J. Thomson, 1860. Stem: *Trypanidi*-.

***Trypetes* Schönherr, 1836**: 595. Current status: valid genus in CURCULIONIDAE: MOLYTINAE: TRYPETIDINI. Note: name placed on the Official List of Generic Names in Zoology (ICZN 1974a).

Type species: *Trypetesrhinoides* Gyllenhal, 1836, by original designation (see ICZN 1974a). Note: name placed on the Official List of Specific Names in Zoology (ICZN 1974a).

Family-group name: TRYPÉTIDES Lacordaire, 1865. Stem: *Trypetid*- (ICZN 1974a).

***Trypeticus* Marseul, 1864**: 281. Current status: valid genus in HISTERIDAE: ABRAEINAE: TRYPETICINI.

Type species: *Trypanaeusgilolous* Marseul, 1864, by subsequent designation (Lewis 1897: 194).

Family-group name: TRYPETICINAE Bickhardt, 1913. Stem: *Trypetic*-.

***Trypodendron* Stephens, 1830**: 353. Current status: valid genus in CURCULIONIDAE: SCOLYTINAE: XYLOTERINI.

Type species: *Dermestesdomesticus* Linnaeus, 1758, by subsequent designation (Westwood 1838c: 39).

Family-group name: TRYPODENDRINA Nunberg, 1954. Stem: *Trypodendr*-.

***Trypophloeus* Fairmaire, 1864**: 105. Current status: valid genus in CURCULIONIDAE: SCOLYTINAE: TRYPOPHLOEINI.

Type species: *Bostrichusbinodulus* Ratzeburg, 1837, by monotypy.

Family-group name: TRYPOPHLOEINAE Nüsslin, 1911. Stem: *Trypophloe*-.

†***Tshekardocoleus* Rohdendorf, 1944**: 252. Current status: valid genus in †TSHEKARDOCOLEIDAE. Note: Permian (Russia).

Type species: †*Tshekardocoleusmagnus* Rohdendorf, 1944, by original designation.

Family-group name: †TSHEKARDOCOLEIDAE Rohdendorf, 1944. Stem: *Tshekardocole*-.

***Tychaeus* Fischer, 1823b**: 266. Current status: junior synonym of *Nemorhinus* Schönherr, 1823 in BRENTIDAE: BRENTINAE: BRENTINI: TYCHAEINA.

Type species: *Brentuscurvidens* Lund, 1802 (= *Brentusmyrmecophaga* Herbst, 1797), by subsequent designation (R. Lucas 1920: 661).

Family-group name: TYCHAEIDAE Schoenfeldt, 1908. Stem: *Tychae*-.

***Tychius* Germar, 1817b**: 340. Current status: valid genus in CURCULIONIDAE: CURCULIONINAE: TYCHIINI: TYCHIINA.

Type species: *Curculioquinquepunctatus* Linnaeus, 1758, by subsequent designation (Schönherr 1825: 583).

Family-group name: TYCHIIDAE Gistel, 1848. Stem: *Tychi*-.

***Tychus* Leach, 1817**: 81, 84. Current status: valid genus in STAPHYLINIDAE: PSELAPHINAE: GONIACERITAE: TYCHINI.

Type species: *Pselaphusniger* Paykull, 1800, by monotypy.

Family-group name: TYCHINI Raffray, 1904. Stem: *Tych*-. Note: junior homonym of TYCHINAE Dana, 1851 (type genus *Tyche* T. Bell, 1835) in Crustacea.

***Tydessa* Peacock, 1982**: 362. Current status: valid genus in PYROCHROIDAE: TYDESSINAE.

Type species: *Dasytesconstrictus* Lewis, 1895 (= *Dasyteslewisi* Pic, 1937), by original designation.

Family-group name: TYDESSINI Nikitsky, 1986. Stem: *Tydess*-.

***Tylauchenia* Burmeister, 1872**: 377. Current status: valid genus in BUPRESTIDAE: POLYCESTINAE: TYNDARIDINI: TYLAUCHENIINA. Note: this name is considered an invalid junior synonym of *Ocypetes* Saunders, 1871 by some authors but Saunders’ name is a junior homonym of *Ocypetes* Risso, 1826 [Arachnida], *Ocypetes* Wagler, 1829 [Aves] and *Ocypetes* Lesson, 1842 [Mammalia].

Type species: *Buprestiscrassicollis* Laporte & Gory, 1838, by monotypy.

Family-group name: TYLACHENIAE Cobos, 1959. Stem: *Tylaucheni*-.

***Tylodes* C.R. Sahlberg, 1823c**: 49. Current status: valid genus in CURCULIONIDAE: CRYPTORHYNCHINAE: CRYPTORHYNCHINI: TYLODINA.

Type species: *Tylodesarmadillo* C.R. Sahlberg, 1823, by monotypy.

Family-group name: TYLODIDES Lacordaire, 1865. Stem: *Tylod*-.

***Tylonotus* Schaum, 1863a**: 74. Current status: invalid senior synonym of *Nototylus* Gemminger & Harold, 1868 in CARABIDAE: NOTOTYLINAE. Note: junior homonym of *Tylonotus* Haldeman, 1847 [Coleoptera: CERAMBYCIDAE].

Type species: *Tylonotusfryi* Schaum, 1863, by monotypy.

Family-group name: TYLONOTINI Schaum, 1863. Stem: *Tylonot*-. Note: family-group name permanently invalid (ICZN 1999a, Article 39).

***Tylosis* J.L. LeConte, 1850c**: 9. Current status: valid genus in CERAMBYCIDAE: CERAMBYCINAE: TRACHYDERINI: TRACHYDERINA.

Type species: *Tylosismaculatus* J.L. LeConte, 1850, by subsequent designation (J. Thomson 1864: 200).

Family-group name: TYLOSITAE J. Thomson, 1861. Stem: *Tylose*-.

***Tyndaris* J. Thomson, 1857**: 168. Current status: valid genus in BUPRESTIDAE: POLYCESTINAE: TYNDARIDINI: TYNDARIDINA.

Type species: *Ptosimaplanata* Laporte & Gory, 1838, by monotypy.

Family-group name: TYNDARINI Cobos, 1955. Stem: *Tyndarid*-.

***Typhaea* Stephens, 1829b**: 8. Current status: valid genus in MYCETOPHAGIDAE: MYCETOPHAGINAE: TYPHAEINI.

Type species: *Mycetophagustestaceus* Fabricius, 1792 (= *Dermestesstercoreus* Linnaeus, 1758), by subsequent designation (Westwood 1838c: 14).

Family-group name: TYPHAEINA C.G. Thomson, 1863. Stem: *Typhae*-.

***Typhlocharis* Dieck, 1869**: 6. Current status: valid genus in CARABIDAE: TRECHINAE: ANILLINI.

Type species: *Typhlocharissilvanoides* Dieck, 1869, by monotypy.

Family-group name: TYPHLOCHARINA Jeanne, 1973. Stem: *Typhlocharit*-.

***Typhlorhinus* Kuschel, 1954b**: 287. Current status: junior synonym of *Hapactorrhynchus* Richard, 1953 in CURCULIONIDAE: ENTIMINAE: OTIORHYNCHITAE: TYPHLORHININI.

Type species: *Typhlorhinusjeanneli* Kuschel, 1954 (= *Hapactorrhynchusvadoni* Richard, 1953), by original designation.

Family-group name: TYPHLORHININI Kuschel, 1954. Stem: *Typhlorhin*-.

***Typhlusechus* Linell, 1897**: 154. Current status: valid genus in TENEBRIONIDAE: PIMELIINAE: STENOSINI: TYPHLUSECHIINA.

Type species: *Typhlusechussingularis* Linell, 1897, by original designation.

Family-group name: TYPHLUSECHINI Casey, 1907. Stem: *Typhlusech*-.

***Typhocesis* Pascoe, 1863b**: 561. Current status: valid genus in CERAMBYCIDAE: CERAMBYCINAE: TYPHOCESINI.

Type species: *Typhocesismacleayi* Pascoe, 1863, by monotypy.

Family-group name: TYPHOCÉSIDES Lacordaire, 1868. Stem: *Typhoces*-.

***Typoderus* G.A.K. Marshall, 1953**: 104. Current status: valid genus in CURCULIONIDAE: MOLYTINAE: MOLYTINI: TYPODERINA.

Type species: *Typoderusmachadoi* G.A.K. Marshall, 1953, by original designation.

Family-group name: TYPODERINA Voss, 1965. Stem: *Typoder*-.

***Typophorus* Chevrolat in Dejean, 1836a**: 412. Current status: valid genus in CHRYSOMELIDAE: EUMOLPINAE: TYPOPHORINI.

Type species: *Eumolpusnigritus* Fabricius, 1801, by subsequent designation (Monrós and Bechyné 1956: 1127).

Family-group name: TYPOPHORINAE Baly, 1865. Stem: *Typophor*-.

***Tyrus* Aubé, 1833**: 15. Current status: valid genus in STAPHYLINIDAE: PSELAPHINAE: PSELAPHITAE: TYRINI: TYRINA.

Type species: *Pselaphusmucronatus* Panzer, 1802, by monotypy.

Family-group name: TYRIDES Reitter, 1882. Stem: *Tyr*-.

***Tytthaspis* Crotch, 1874a**: 181. Current status: valid genus in COCCINELLIDAE: COCCINELLINAE: COCCINELLINI.

Type species: *Coccinellasedecimpunctata* Linnaeus, 1761, by original designation.

Family-group name: TYTTHASPIDES Crotch, 1874. Stem: *Tytthaspid*-.

***Tytthonyx* J.L. LeConte, 1851b**: 347. Current status: valid genus in CANTHARIDAE: MALTHININAE: TYTTHONYXINI.

Type species: *Lampyriserythrocephala* Fabricius, 1801, by monotypy.

Family-group name: TYTTHONYINI Arnett, 1962. Stem: *Tytthonyx*-.

***Uleiota* Latreille, 1797**: 46. Current status: valid genus in SILVANIDAE: BRONTINAE: BRONTINI.

Type species: *Cucujusflavipes* Fabricius, 1779 (= *Cerambyxplanatus* Linnaeus, 1761), by subsequent monotypy (Latreille 1802b: 210).

Family-group name: ULEIOTAEIDAE Gistel, 1848. Stem: *Uleiot*-.

***Ulocerus* Schönherr, 1823**: 1137. Current status: valid genus in BRENTIDAE: BRENTINAE: ULOCERINI: ULOCERINA.

Type species: *Uloceruslacerosus* Schönherr, 1823, by original designation.

Family-group name: ULOCERIDES Schönherr, 1823. Stem: *Ulocer*-.

***Ulodes* Erichson, 1842**: 180. Current status: valid genus in ULODIDAE.

Type species: *Ulodesverrucosus* Erichson, 1842, by monotypy.

Family-group name: ULODINAE Pascoe, 1869. Stem: *Ulod*-.

***Uloma* Dejean, 1821**: 67. Current status: valid genus in TENEBRIONIDAE: TENEBRIONINAE: ULOMINI. Note: name placed on the Official List of Generic Names in Zoology (ICZN 1975).

Type species: *Tenebrioculinaris* Linnaeus, 1758, by plenary power (see ICZN 1975). Note: name placed on the Official List of Specific Names in Zoology (ICZN 1975).

Family-group name: ULOMITES Blanchard, 1845. Stem: *Ulom*-.

***Ulomascus* Fairmaire, 1848**: 173. Current status: valid genus in CURCULIONIDAE: CURCULIONINAE: ULOMASCINI.

Type species: *Ulomascuscaviventris* Fairmaire, 1848, by monotypy.

Family-group name: ULOMASCIDES Lacordaire, 1865. Stem: *Ulomasc*-.

†***Ulyana* Zherikhin, 1993**: 27. Current status: valid genus in ANTHRIBIDAE: †ULYANINAE. Note: Cretaceous (Mongolia; Russia).

Type species: †*Ulyananobilis* Zherikhin, 1993, by original designation.

Family-group name: †ULYANIDAE Zherikhin, 1993. Stem: *Ulyan*-.

†***Ulyanisca* Gratshev, 1998**: 45. Current status: valid genus in BRENTIDAE: ITHYCERINAE: †ULYANISCINI. Note: Lower Cretaceous (Mongolia).

Type species: †*Ulyaniscadentipes* Gratschev, 1998, by original designation.

Family-group name: †ULYANISCINI Legalov, 2009. Stem: *Ulyanisc*-.

***Undulifer* Gemminger in Gemminger and Harold, 1868c**: 974. Current status: valid genus in PASSALIDAE: PASSALINAE: PROCULINI. Note: this name was previously attributed to Kaup (1869: 6) in the literature; however, it was first made available by Gemminger in 1868 when he associated one nominal species under this name.

Type species: *Passalusincisus* Truqui, 1857, by monotypy.

Family-group name: UNDULIFERINAE Kuwert, 1891. Stem: *Undulifer*-.

***Unxia* J. Thomson, 1861**: 234, 252. Current status: valid genus in CERAMBYCIDAE: CERAMBYCINAE: UNXIINI.

Type species: *Unxiainsignis* J. Thomson, 1861 (= *Cosmisomainsigne* Guérin-Méneville, 1844), by monotypy.

Family-group name: UNXIINI Napp, 2007. Stem: *Unxi*-.

***Upinella* Mulsant, 1857b**: 17. Current status: valid genus in TENEBRIONIDAE: ALLECULINAE: ALLECULINI: ALLECULINA.

Type species: *Alleculaaterrima* Rosenhauer, 1847, by monotypy.

Family-group name: UPINELLAE J.L. LeConte, 1866. Stem: *Upinell*-.

***Upis* Fabricius, 1792b**: 515. Current status: valid genus in TENEBRIONIDAE: STENOCHIINAE: CNODALONINI.

Type species: *Attelabusceramboides* Linnaeus, 1758, by monotypy.

Family-group name: UPIDAE C.G. Thomson, 1859. Stem: *Upid*-.

***Uptona* G.S. Medvedev & Lawrence, 1986**: 582. Current status: valid genus in TENEBRIONIDAE: DIAPERINAE: HYOCIINI: UPTONINA.

Type species: *Uptonapallida* G.S. Medvedev & Lawrence, 1986, by original designation.

Family-group name: UPTONINA G.S. Medvedev & Lawrence, 1986. Stem: *Upton*-.

***Uracanthus* Hope, 1833**: 64. Current status: valid genus in CERAMBYCIDAE: CERAMBYCINAE: URACANTHINI.

Type species: *Uracanthustriangularis* Hope, 1833, by monotypy.

Family-group name: URACANTHITAE Blanchard, 1853. Stem: *Uracanth*-.

†***Uralocoleus* Zalesskiy, 1947**: 858. Current status: valid genus in †TSHEKARDOCOLEIDAE. Note: Permian (Russia).

Type species: †*Uralocoleussplendens* Zalesskiy, 1947, by original designation.

Family-group name: †URALOCOLEIDAE Zalesskiy, 1947. Stem: *Uralocole*-.

***Urodon* Schönherr, 1823**: 1134. Current status: junior synonym of *Bruchela* Dejean, 1821 in ANTHRIBIDAE: URODONTINAE. Note: unnecessary new replacement name for *Bruchela* Dejean, 1821.

Type species: *Anthribussericeus* Fabricius, 1801 (= *Bruchusrufipes* G.-A. Olivier, 1790), by original designation.

Family-group name: URODONTIDES C.G. Thomson, 1859. Stem: *Urodont*-.

***Uroplata* Chevrolat in Dejean, 1836a**: 365. Current status: valid genus in CHRYSOMELIDAE: CASSIDINAE: UROPLATINI. Note: name placed on the Official List of Generic Names in Zoology (ICZN 1985f).

Type species: *Hispamucronata* G.-A. Olivier, 1808, by subsequent designation (R.E. White 1981: 714; see ICZN 1985f). Note: name placed on the Official List of Specific Names in Zoology (ICZN 1985f).

Family-group name: UROPLATINI Weise, 1910. Stem: *Uroplat*-.

***Uroptera* Berthold, 1827**: 383. Current status: valid genus in BRENTIDAE: BRENTINAE: TRACHELIZINI: PSEUDOCEOCEPHALINA.

Type species: **here fixed** (under ICZN 1999a, Article 70.3.2) as *Ceocephalusappendiculatus* Boheman, 1833, misidentified as *Brentuscaudatus* Herbst, 1797 (sensu Latreille, 1828) in the original designation by subsequent monotypy in Latreille (1828b: 595, as *Uropterus*).

Family-group name: UROPTERINI Jakobson, 1911. Stem: *Uropter*-.

***Usechus* Motschulsky, 1845b**: 79. Current status: valid genus in ZOPHERIDAE: ZOPHERINAE: USECHINI.

Type species: *Usechuslacerta* Motschulsky, 1845, by monotypy.

Family-group name: USECHINI G.H. Horn, 1867. Stem: *Usech*-.

***Vacronus* Casey, 1907**: 501, 508. Current status: junior synonym of *Alaephus* G.H. Horn, 1870 in TENEBRIONIDAE: PIMELIINAE: VACRONINI.

Type species: *Vacronustenuicornis* Casey, 1907, by original designation.

Family-group name: VACRONINAE Gebien, 1910. Stem: *Vacron*-.

***Vadonaxia* Descarpentries, 1970**: 189. Current status: valid genus in BUPRESTIDAE: CHRYSOCHROINAE: VADONAXIINI.

Type species: *Vadonaxiapeyrierasi* Descarpentries, 1970, by original designation.

Family-group name: VADONAXIINI Descarpentries, 1970. Stem: *Vadonaxi*-.

***Valda* Casey, 1893**: 493. Current status: valid genus in STAPHYLINIDAE: PSELAPHINAE: GONIACERITAE: VALDINI.

Type species: *Valdafrontalis* Casey, 1893, by monotypy.

Family-group name: VALDIINI Park, 1953. Stem: *Vald*-.

***Valenfriesia* Alonso-Zarazaga & Lyal, 1999**: 38. Current status: valid genus in ANTHRIBIDAE: CHORAGINAE: VALENFRIESIINI. Note: new replacement name for *Notioxenus* Wollaston, 1861.

Type species: *Notioxenusbewickii* Wollaston, 1861, by subsequent designation (Jordan 1907: 380) (type fixation for *Notioxenus* Wollaston).

Family-group name: VALENFRIESIINI Alonso-Zarazaga & Lyal, 1999. Stem: *Valenfriesi*-.

***Valgus* Scriba, 1790**: 66. Current status: valid genus in SCARABAEIDAE: CETONIINAE: VALGINI.

Type species: *Scarabaeushemipterus* Linnaeaus, 1758, by monotypy.

Family-group name: VALGUAIRES Mulsant, 1842. Stem: *Valg*-.

***Vansonium* C. Koch, 1950b**: 354. Current status: valid genus in TENEBRIONIDAE: PIMELIINAE: CRYPTOCHILINI: VANSONIINA.

Type species: *Vansoniumbushmanicum* C. Koch, 1950, by original designation.

Family-group name: VANSONINI C. Koch, 1955. Stem: *Vansoni*-.

***Vatellus* Aubé, 1837**: 218, 221. Current status: valid genus in DYTISCIDAE: HYDROPORINAE: VATELLINI. Note: the older name *Leucorea* Laporte, 1835, which was later replaced with *Vatellus* by Aubé (1837), was suppressed for the Principle of Priority but not the Principle of Homonymy and placed on the Official Index of Rejected and Invalid Names in Zoology while *Vatellus* Aubé, 1837 was placed on the Official List of Generic Names in Zoology (ICZN 1992).

Type species: *Hydroporustarsatus* Laporte, 1835, by monotypy (see ICZN 1992). Note: name placed on the Official List of Specific Names in Zoology (ICZN 1992).

Family-group name: VATELLINI Sharp, 1880. Stem: *Vatell*-.

***Vatesus* Sharp, 1876a**: 201. Current status: valid genus in STAPHYLINIDAE: TACHYPORINAE: VATESINI.

Type species: *Vatesuslatitans* Sharp, 1876, by monotypy.

Family-group name: VATESINI Seevers, 1958. Stem: *Vates*-.

***Vatinius* Harold in Gemminger and Harold, 1868c**: 978. Current status: junior synonym of *Passalus* Fabricius, 1792 in PASSALIDAE: PASSALINAE: PASSALINI. Note: junior homonym of *Vatinius* Stål, 1865 [Hemiptera]; this name is attributed to Kaup (1869: 35) in the literature; however, it was first made available by Harold in 1868 when he associated several nominal species with this name.

Type species: *Passalustoriferus* Eschscholtz, 1828 [as *torifer*], by subsequent designation (Hincks and Dibb 1935: 38).

Family-group name: VATINIINAE Kuwert, 1896. Stem: *Vatini*-. Note: family-group name permanently invalid (ICZN 1999a, Article 39).

***Vellejus* Kaup, 1871**: 35. Current status: junior synonym of *Labienus* Kaup, 1871 in PASSALIDAE: PASSALINAE: MACROLININI. Note: junior homonym of *Vellejus* Stål, 1865 [Hemiptera].

Type species: *Passaluscompergus* Boisduval, 1835, by subsequent designation (Hincks and Dibb 1935: 93).

Family-group name: VELLEJINAE Kuwert, 1891. Stem: *Vellej*-. Note: family-group name permanently invalid (ICZN 1999a, Article 39).

***Velora* J. Thomson, 1864**: 56. Current status: valid genus in CERAMBYCIDAE: LAMIINAE: DESMIPHORINI.

Type species: *Veloraaustralis* J. Thomson, 1864, by original designation.

Family-group name: VELORINI Aurivillius, 1917. Stem: *Velor*-.

***Verania* Mulsant, 1850**: 358. Current status: junior synonym of *Micraspis* Chevrolat, 1836 in COCCINELLIDAE: COCCINELLINAE: COCCINELLINI. Note: junior homonym of *Verania* Krohn, 1846 [Mollusca].

Type species: *Coccinellacomma* Thunberg, 1781, by subsequent designation (Crotch 1874a: 175).

Family-group name: VERANIINI F.A. Schilder & M. Schilder, 1928. Stem: *Verani*-. Note: family-group name permanently invalid (ICZN 1999a, Article 39).

***Vesperella* Dayrem, 1933**: 89. Current status: valid genus in CERAMBYCIDAE: CERAMBYCINAE: VESPERELLINI.

Type species: *Vesperellapallida* Dayrem, 1933, by monotypy.

Family-group name: VESPERELLINI Sama, 2008. Stem: *Vesperell*-.

***Vesperoctenus* H.W. Bates, 1891**: 159. Current status: valid genus in CERAMBYCIDAE: PRIONINAE: VESPEROCTENINI.

Type species: *Vesperoctenusflorhi* H.W. Bates, 1891, by monotypy.

Family-group name: VESPEROCTENINI Vives, 2005. Stem: *Vesperocten*-.

***Vesperus* Dejean, 1821**: 111. Current status: valid genus in VESPERIDAE: VESPERINAE.

Type species: *Stenocorusstrepens* Fabricius, 1792, by subsequent designation (Chevrolat 1849b: 216).

Family-group name: VESPÉRAIRES Mulsant, 1839. Stem: *Vesper*-.

***Vesta* Laporte, 1833c**: 126, 132. Current status: valid genus in LAMPYRIDAE incertae sedis.

Type species: *Vestachevrolatii* Laporte, 1833, by monotypy.

Family-group name: VESTINA McDermott, 1964. Stem: *Vest*-.

†***Vetuproteinus* Cai, Newton & Thayer in Cai et al., 2016**: 2. Current status: valid genus in STAPHYLINIDAE: PROTEININAE: †VETUPROTEININI. Note: mid-Cretaceous (Myanmar).

Type species: †*Vetuproteinuscretaceus* Cai, Newton & Thayer, 2016, by original designation.

Family-group name: †VETUPROTEININI Cai, Newton & Thayer in Cai et al., 2016: 2. Stem: *Vetuprotein*-.

***Veturius* Kaup, 1871**: 110. Current status: valid genus in PASSALIDAE: PASSALINAE: PROCULINI.

Type species: *Passalusheydenii* Kaup, 1868, by subsequent designation (Gravely 1918: 35).

Family-group name: VETURINAE Kuwert, 1891. Stem: *Veturi*-.

***Vikhrevia* Kazantsev, 2013d**: 248. Current status: valid genus in LYCIDAE incertae sedis.

Type species: *Vikhrevialudificator* Kazantsev, 2013, by original designation.

Family-group name: VIKHREVINI Kazantsev, 2013d: 247. Stem: *Vikhrev*- (ICZN 1999a, Article 29.4).

***Vindex* Kaup, 1871**: 78. Current status: valid genus in PASSALIDAE: PASSALINAE: PROCULINI.

Type species: *Passalusagnoscendus* Percheron, 1841, by monotypy.

Family-group name: VINDICINAE Kuwert, 1896. Stem: *Vindic*-.

***Viticis* Lea, 1930**: 463. Current status: valid genus in CURCULIONIDAE: CURCULIONINAE: VITICIINI.

Type species: *Viticisbidentatus* Lea, 1930, by original designation.

Family-group name: VITICIINAE Morimoto, 1983. Stem: *Vitici*-.

***Viticlerus* Miyatake, 1977**: 105. Current status: valid genus in THANEROCLERIDAE: THANEROCLERINAE: THANEROCLERINI.

Type species: *Viticlerusformicinus* Miyatake, 1977, by original designation.

Family-group name: VITICLERINI J.R. Winkler, 1982. Stem: *Viticler*-.

***Vossicartus* Legalov, 2003**: 79. Current status: valid genus in ATTELABIDAE: SAYREVILLEINAE: VOSSICARTINI.

Type species: *Rhinocartusbruncki* Voss, 1974, by original designation.

Family-group name: VOSSICARTINI Legalov, 2003. Stem: *Vossicart*-.

***Vrilletta* J.L. LeConte, 1874b**: 64. Current status: valid genus in PTINIDAE: XYLETININAE: XYLETININI.

Type species: *Vrillettamurrayi* J.L. LeConte, 1874, by subsequent designation (R.E. White 1974: 453). Note: R.E. White (1974: 453) listed R. Lucas (1920: 668) as the author who selected the type species; however, R. Lucas listed two species and this is considered a selection made in an ambiguous manner and cannot be considered as a designation under the Code (ICZN 1999a, Article 67.5.3).

Family-group name: VRILETTINI Böving, 1927. Stem: *Vrillett*-.

†***Wabbel* Alekseev, 2017**: 30. Current status: valid genus in †WABBELIDAE. Note: mid-Eocene (Kaliningrad region, Russia).

Type species: †*Wabbelcerebricavus* Alekseev, 2017, by original designation.

Family-group name: †WABBELIDAE Alekseev, 2017: 34. Stem: *Wabbel*-.

†***Waidelotus* Bukejs, Alekseev & Pollock, 2019**: 264. Current status: valid genus in PYROCHROIDAE: †WAIDELOTINAE. Note: mid-Eocene (Kaliningrad region, Russia).

Type species: †*Waidelotushoffeinsorum* Bukejs, Alekseev & Pollock, 2019, by original designation.

Family-group name: †WAIDELOTINAE Bukejs, Alekseev & Pollock, 2019: 262. Stem: *Waidelot*-.

***Warwickia* A.B.T. Smith & Evans, 2005**: 42. Current status: valid genus in SCARABAEIDAE: MELOLONTHINAE: WARWICKIINI. Note: new replacement name for *Benedictia* Sanderson, 1939.

Type species: *Benedictiapilosa* Sanderson, 1939, by original designation (type fixation for *Benedictia* Sanderson).

Family-group name: WARWICKIINI Evans & A.B.T. Smith, 2020: 68. Stem: *Warwicki*-.

***Webbia* Hopkins, 1915**: 222. Current status: valid genus in CURCULIONIDAE: SCOLYTINAE: XYLEBORINI.

Type species: *Webbiadipterocarpi* Hopkins, 1915, by original designation.

Family-group name: WEBBINAE Hopkins, 1915. Stem: *Webbi*-.

***Wittmeraltica* Bechyné, 1956**: 1011. Current status: valid genus in CHRYSOMELIDAE: GALERUCINAE: ALTICINI.

Type species: *Wittmeralticamuriensis* Bechyné, 1956, by monotypy.

Family-group name: WITTMERALTICINA Bechyné, 1968. Stem: *Wittmeraltic*-.

***Wittmeroquasimus* Dolin, 1993**: 195. Current status: valid genus in ELATERIDAE: NEGASTRIINAE: QUASIMUSINI: WITTMEROQUASINA.

Type species: *Quasimusocellatus* Dolin, 1993, by original designation.

Family-group name: WITTMEROQUASINA Schimmel & Tarnawski, 2009. Stem: *Wittmeroquas*- (ICZN 1999a, Article 29.4).

***Wooldridgeus* Spangler, 1999**: 181. Current status: junior synonym of *Platypelochares* Champion, 1923 in LIMNICHIDAE: LIMNICHINAE: WOOLDRIDGEINI.

Type species: *Wooldridgeusperforatus* Spangler, 1999, by original designation.

Family-group name: WOOLDRIDGEINI Spangler, 1999. Stem: *Wooldridge*-.

***Wow* Jałoszyński, Maruyama & Klimaszewski, 2023**: 575. Current status: valid genus in STAPHYLINIDAE: ALEOCHARINAE: WOWINI.

Type species: *Wowassingi* Jałoszyński, Maruyama & Klimaszewski, 2023, by original designation.

Family-group name: WOWINI Jałoszyński, Maruyama & Klimaszewski, 2023: 574. Stem: *Wow*-.

***Xantholinus* Dejean, 1821**: 23. Current status: valid genus in STAPHYLINIDAE: STAPHYLININAE: XANTHOLININI. Note: name placed on the Official List of Generic Names in Zoology (ICZN 1983c).

Type species: *Staphylinuslinearis* G.-A. Olivier, 1795, by plenary power (see ICZN 1983c). Note: name placed on the Official List of Specific Names in Zoology (ICZN 1983c, as *Staphylinuslinearis* G.-A. Olivier, 1794).

Family-group name: XANTHOLININI Erichson, 1839. Stem: *Xantholin*-. Note: family-group name given precedence over AGRODINI Nordmann, 1837 and GYROHYPNINI Kirby, 1837 and placed on the Official List of Family-Group Names in Zoology (ICZN 1996c).

***Xanthopygus* Kraatz, 1857b**: 539. Current status: valid genus in STAPHYLINIDAE: STAPHYLININAE: STAPHYLININI: XANTHOPYGINA.

Type species: *Staphylinusxanthopygus* Nordmann, 1837, by absolute tautonymy.

Family-group name: XANTHOPYGINA Sharp, 1884. Stem: *Xanthopyg*-.

***Xenaroswelliana* Erwin, 2007**: 563. Current status: valid genus in CARABIDAE: HARPALINAE: XENAROSWELLIANINI.

Type species: *Xenaroswellianadeltaquadrant* Erwin, 2007, by original designation.

Family-group name: XENAROSWELLANINI Erwin, 2007. Stem: *Xenaroswellian*-.

***Xenaster* Bierig, 1939**: 179. Current status: invalid senior synonym of *Xenasterides* Newton, 2017 in STAPHYLINIDAE: PAEDERINAE: LATHROBIINI: STILICOPSINA. Note: junior homonym of *Xenaster* Simonwitsch, 1871 [Echinodermata].

Type species: *Xenasterplaumanni* Bierig, 1939, by original designation.

Family-group name: XENASTERES Bierig, 1939. Stem: *Xenaster*-. Note: family-group name permanently invalid (ICZN 1999a, Article 39).

***Xenicotela* H.W. Bates, 1884**: 242. Current status: valid genus in CERAMBYCIDAE: LAMIINAE: XENICOTELINI.

Type species: *Xenicotelafuscula* H.W. Bates, 1884 (= *Monohammuspardalinus* H.W. Bates, 1884), by monotypy.

Family-group name: XENICOTELINI Matsushita, 1933. Stem: *Xenicotel*-.

***Xenobythus* Peyerimhoff, 1901**: 203. Current status: valid genus in STAPHYLINIDAE: PSELAPHINAE: GONIACERITAE: BYTHININI: XENOBYTHINA.

Type species: *Xenobythusserullazi* Peyerimhoff, 1901, by monotypy.

Family-group name: XENOBYTHINA Jeannel, 1950. Stem: *Xenobyth*-.

***Xenocephalus* Wasmann, 1887**: 411. Current status: junior synonym of *Vatesus* Sharp, 1876 in STAPHYLINIDAE: TACHYPORINAE: VATESINI. Note: junior homonym of *Xenocephalus* Kaup, 1858 [Pisces].

Type species: *Xenocephalusclypeatus* Wasmann, 1887, by monotypy.

Family-group name: XENOCEPHALINI Wasmann, 1887. Stem: *Xenocephal*-. Note: family-group name permanently invalid (ICZN 1999a, Article 39).

***Xenocerus* Schönherr, 1833**: 3, 117. Current status: valid genus in ANTHRIBIDAE: ANTHRIBINAE: XENOCERINI.

Type species: *Xenocerussaperdoides* Gyllenhal, 1833, by original designation.

Family-group name: XÉNOCERIDES Lacordaire, 1865. Stem: *Xenocer*-.

***Xenodusa* Wasmann, 1894**: 205. Current status: valid genus in STAPHYLINIDAE: ALEOCHARINAE: LOMECHUSINI: LOMECHUSINA.

Type species: *Atemelescava* J.L. LeConte, 1863, by subsequent designation (Fenyes 1919: 26).

Family-group name: XENODUSAE Seevers, 1978. Stem: *Xenodus*-.

***Xenofrea* H.W. Bates, 1885**: 373. Current status: valid genus in CERAMBYCIDAE: LAMIINAE: XENOFREINI.

Type species: *Xenofreaareolata* H.W. Bates, 1885, by subsequent designation (Marinoni 1977: 50).

Family-group name: XENOFREINI Aurivillius, 1923. Stem: *Xenofre*-.

***Xenolea* J. Thomson, 1864**: 91. Current status: valid genus in CERAMBYCIDAE: LAMIINAE: XENOLEINI.

Type species: *Xenoleacollaris* J. Thomson, 1864, by original designation.

Family-group name: XÉNOLÉIDES Lacordaire, 1872. Stem: *Xenole*-.

***Xenomycetes* G.H. Horn, 1880b**: 141. Current status: valid genus in ENDOMYCHIDAE: XENOMYCETINAE.

Type species: *Xenomycetesmorrisoni* G.H. Horn, 1880, by monotypy.

Family-group name: XENOMYCETINAE Strohecker, 1962. Stem: *Xenomycet*-.

***Xenopsis* Saunders, 1867**: 514. Current status: valid genus in BUPRESTIDAE: POLYCESTINAE: POLYCESTINI: XENOPSINA.

Type species: *Xenopsislaevis* Saunders, 1867, by monotypy.

Family-group name: XENOPSINA Volkovitsh, 2008. Stem: *Xenops*- (ICZN 1999a, Article 29.4).

***Xenorchestes* Wollaston, 1854**: 417. Current status: valid genus in ANTHRIBIDAE: CHORAGINAE: XENORCHESTINI.

Type species: *Xenorchestessaltitans* Wollaston, 1854, by monotypy.

Family-group name: XÉNORCHESTIDES Lacordaire, 1865. Stem: *Xenorchest*-.

***Xenorhipis* J.L. LeConte, 1867**: 384. Current status: valid genus in BUPRESTIDAE: BUPRESTINAE: XENORHIPIDINI: XENORHIPIDINA.

Type species: *Xenorhipisbrendeli* J.L. LeConte, 1867, by monotypy.

Family-group name: XENORHIPINI Cobos, 1986. Stem: *Xenorhipid*-.

***Xenoscelinus* Grouvelle, 1910b**: 143. Current status: junior synonym of *Cathartocryptus* Sharp, 1886 in EROTYLIDAE: PHARAXONOTHINAE.

Type species: *Xenoscelinusmalaicus* Grouvelle, 1910, by monotypy.

Family-group name: XENOSCELININI Sen Gupta & Crowson, 1971. Stem: *Xenoscelin*-.

***Xenoscelis* Wollaston, 1864**: 132. Current status: valid genus in EROTYLIDAE: XENOSCELINAE. Note: new replacement name for *Pristoscelis* Wollaston, 1862.

Type species: *Pristoscelisdeplanatus* Wollaston, 1862, by monotypy (type fixation for *Pristoscelis* Wollaston).

Family-group name: XENOSCELINI Ganglbauer, 1899. Stem: *Xenoscel*-.

***Xenospasta* Selander, 1966**: 467. Current status: valid genus in MELOIDAE: ELETICINAE: SPASTICINI: XENOSPASTINA.

Type species: *Xenospastaflava* Selander, 1966, by original designation.

Family-group name: XENOSPASTINA Selander, 1966. Stem: *Xenospast*-.

***Xenostanus* Y.-D. Li, Szawaryn & Cai in Y.-D. Li et al., 2022**: 413. Current status: valid genus in CORYLOPHIDAE: CORYLOPHINAE: XENOSTANINI.

Type species: *Xenostanusjiangkuni* Y.-D. Li, Szawaryn & Cai, 2022, by original designation.

Family-group name: XENOSTANINI Y.-D. Li, Szawaryn & Cai in Y.-D. Li et al., 2022: 413. Stem: *Xenostan*-.

***Xerasiborus* Jordal, 2021**: 102. Current status: valid genus in CURCULIONIDAE: SCOLYTINAE: HYPOBORINI: XERASIBORINA.

Type species: *Xerasiboruseuphorbiae* Jordal, 2021, by original designation.

Family-group name: XERASIBORINA Jordal, 2021: 102. Stem: *Xerasibor*-.

***Xerobia* Gistel, 1856**: 373. Current status: junior synonym of *Cleonis* Dejean, 1821 in CURCULIONIDAE: LIXINAE: CLEONINI.

Type species: *Curculiosulcirostris* Linnaeus, 1758, by subsequent designation (Alonso- Zarazaga and Lyal 1999: 22, 191).

Family-group name: XEROBIADAE Gistel, 1856. Stem: *Xerobi*-.

***Xestobium* Motschulsky, 1845b**: 35. Current status: valid genus in PTINIDAE: ERNOBIINAE: XESTOBIINI.

Type species: *Dermestestessellatus* Fabricius, 1775 (= *Ptinusrufovillosus* DeGeer, 1774), by original designation.

Family-group name: XESTOBIINI Böving, 1927. Stem: *Xestobi*-.

***Xiphaspis* G.A.K. Marshall, 1920**: 393. Current status: valid genus in CURCULIONIDAE: XIPHASPIDINAE.

Type species: *Xiphaspislongiclavis* G.A.K. Marshall, 1920, by original designation.

Family-group name: XIPHASPIDINAE G.A.K. Marshall, 1920. Stem: *Xiphaspid*-.

***Xiphoscelis* Burmeister, 1842**: 613. Current status: valid genus in SCARABAEIDAE: CETONIINAE: XIPHOSCELIDINI.

Type species: *Xiphoscelisschuckardi* Burmeister, 1842, by subsequent designation (Lacordaire 1855: 495). Note: Lacordaire (1855: 495) designated *Cetoniagariepena* Gory & Percheron, 1833 as type species of *Xiphoscelis*, a nominal species not originally included, but since he placed that species in synonymy with *Xiphoscelisschuckardi* Burmeister, a species originally included, he is deemed to have designated the latter species as type species (ICZN 1999a, Article 69.2.2).

Family-group name: XIPHOSCELIDEAE Burmeister, 1842. Stem: *Xiphoscelid*-.

***Xixuthrus* J. Thomson, 1864**: 296. Current status: valid genus in CERAMBYCIDAE: PRIONINAE: MACROTOMINI: XIXUTHRINA.

Type species: *Macrotomamicrocerus* A. White, 1853, by original designation.

Family-group name: XIXUTHRI Lameere, 1912. Stem: *Xixuthr*-.

***Xylariophilus* Pal & Lawrence, 1986**: 185. Current status: valid genus in TEREDIDAE: XYLARIOPHILINAE.

Type species: *Xylariophilushonoratus* Pal & Lawrence, 1986, by original designation. Note: *Xylariophiluscomatus* Pal & Lawrence, 1986 is sometimes mentioned as the type species (e.g., Ślipiński 2007: 551).

Family-group name: XYLARIOPHILINAE Pal & Lawrence, 1986. Stem: *Xylariophil*-.

***Xyleborips* Reitter, 1913b**: 111. Current status: junior synonym of *Monarthrum* Kirsch, 1866 in CURCULIONIDAE: SCOLYTINAE: CORTHYLINI: CORTHYLINA.

Type species: *Xyloborusmeuseli* Reitter, 1905, by monotypy.

Family-group name: XYLEBORIPINA Reitter, 1913. Stem: *Xyleborip*-.

***Xyleborus* Eichhoff, 1864**: 37, 45. Current status: valid genus in CURCULIONIDAE: SCOLYTINAE: XYLEBORINI. Note: the senior homonym *Xyleborus* Bowdich, 1825 [Coleoptera: BUPRESTIDAE], and all other uses of the name *Xyleborus* prior to *Xyleborus* Eichhoff, 1864, were suppressed for the purposes of the Principle of Priority and the Principle of Homonymy by the Commission and *Xyleborus* Eichhoff, 1864 was placed on the Official List of Generic Names in Zoology (ICZN 1968a).

Type species: *Bostrichusmonographus* Fabricius, 1792, by subsequent designation (Lacordaire 1865: 381; see ICZN 1968a). Note: name placed on the Official List of Specific Names in Zoology (ICZN 1968a).

Family-group name: XYLEBORI J.L. LeConte, 1876. Stem: *Xylebor*-.

***Xylechinus* Chapuis, 1869**: 36. Current status: valid genus in CURCULIONIDAE: SCOLYTINAE: HYLURGINI.

Type species: *Hylesinuspilosus* Ratzeburg, 1837, by monotypy.

Family-group name: XYLECHINIDES Nüsslin, 1912. Stem: *Xylechin*-.

***Xyletinus* Latreille, 1809a**: 376. Current status: valid genus in PTINIDAE: XYLETININAE: XYLETININI. Note: name placed on the Official List of Generic Names in Zoology (ICZN 1971).

Type species: *Ptilinusater* Panzer, 1796, by plenary power (ICZN 1971, as *Ptilinusater* Creutzer, 1796). Note: Panzer (1796) listed Creutzer after the species’ name on the plate; however, Panzer was responsible for the work and he is the one that satisfied the criteria of availability (ICZN 1999a, Article 50.1).

Family-group name: XYLETINIDAE Gistel, 1848. Stem: *Xyletin*-.

***Xylinades* Latreille, 1828b**: 590. Current status: junior synonym of *Xylinada* Berthold, 1827 in ANTHRIBIDAE: ANTHRIBINAE: XYLINADINI.

Type species: *Xylinadeswestermanni* Schönherr, 1833, by subsequent designation (Schönherr 1833: 177). Note: no species were originally included in this genus by Latreille (1828b); the first ones were the six species listed by Schönherr (1833: 178–181).

Family-group name: XYLINADIDES Lacordaire, 1865. Stem: *Xylinad*-.

***Xylita* Paykull, 1798**: 249. Current status: valid genus in MELANDRYIDAE: MELANDRYINAE: XYLITINI.

Type species: *Elaterbuprestoides* Fabricius, 1792 (= *Serropalpuslaevigatus* Hellenius, 1786), by subsequent designation (Mulsant 1856b: 86). Note: Mulsant (1856b: 86) designated *Serropalpuslaevigatus* Hellenius, 1786 as type species, a species not originally included in *Xylita*, but since he listed the species in synonymy with *Elaterbuprestoides* Fabricius, 1792 at the same time, a species originally included in *Xylita*, he is deemed to have selected the latter species (ICZN 1999a, Article 69.2.2).

Family-group name: XYLITINA C.G. Thomson, 1864. Stem: *Xylit*-.

***Xylobius* Latreille, 1834**: 124. Current status: junior synonym of *Xylophilus* Mannerheim, 1823 in EUCNEMIDAE: MELASINAE: XYLOBIINI. Note: unnecessary new replacement name for *Xylophilus* Mannerheim, 1823.

Type species: *Elateralni* Fabricius, 1801 (= *Elatercorticalis* Paykull, 1800), by subsequent designation (Guérin-Méneville 1843b: 175).

Family-group name: XYLOBIINI Reitter, 1911. Stem: *Xylobi*-.

***Xyloctonus* Eichhoff, 1872**: 134. Current status: valid genus in CURCULIONIDAE: SCOLYTINAE: XYLOCTONINI: XYLOCTONINA.

Type species: *Xyloctonusscolytoides* Eichhoff, 1872, by monotypy.

Family-group name: XYLOCTONIDAE Eichhoff, 1878. Stem: *Xylocton*-.

***Xylographella* Miyatake, 1985**: 279. Current status: valid genus in CIIDAE: CIINAE: XYLOGRAPHELLINAE: XYLOGRAPHELLINI.

Type species: *Xylographellapunctata* Miyatake, 1985, by monotypy.

Family-group name: XYLOGRAPHELLINI Kawanabe & Miyatake, 1996. Stem: *Xylographell*-.

***Xylonichus* Boisduval, 1835**: 186. Current status: valid genus in SCARABAEIDAE: MELOLONTHINAE: COLYMBOMORPHINI.

Type species: *Xylonichuseucalypti* Boisduval, 1835, by monotypy.

Family-group name: XYLONYCHINI Britton, 1957. Stem: *Xylonich*-.

***Xylopertha* Guérin-Méneville, 1845**: xvii. Current status: valid genus in BOSTRICHIDAE: BOSTRICHINAE: XYLOPERTHINI.

Type species: *Apatesinuata* Fabricius, 1792 (= *Bostrichusretusus* G.-A. Olivier, 1790), by subsequent designation (E. Desmarest 1860: 258).

Family-group name: XYLOPERTHINI Lesne, 1921. Stem: *Xyloperth*-.

***Xylophilus* Latreille, 1828b**: 589. Current status: invalid senior synonym of *Euglenes* Westwood, 1830 in ADERIDAE. Note: Latreille (1825: 383) used the vernacular name “Xylophile” for this genus, the name is therefore unavailable from that work; junior homonym of *Xylophilus* Mannerheim, 1823 [Coleoptera: EUCNEMIDAE].

Type species: *Anthicusoculatus* Paykull, 1798, by subsequent designation (Curtis 1830: pl. 299). Note: the type species often given in the literature is *Notoxuspopulneus* Creutzer, 1796 (e.g., Nardi 2007: 23) by subsequent designation of Westwood (1830a: 57, 61); however, we failed to find any indication in Westwood’s work that he designated a type species for *Xylophilus* Latreille, 1828. The valid type species fixation is that of Curtis (1830: pl. 299) and therefore *Xylophilus* Latreille, 1828 is a senior synonym of *Euglenes* Westwood, 1830, not of *Aderus* Stephens, 1829, as is sometimes recognized (e.g., Nardi 2020: 626).

Family-group name: XYLOPHILIDAE Shuckard, 1839. Stem: *Xylophil*-. Note: family-group name permanently invalid (ICZN 1999a, Article 39).

***Xylorhiza* Dejean, 1835**: 344. Current status: valid genus in CERAMBYCIDAE: LAMIINAE: XYLORHIZINI.

Type species: *Lamiaadusta* Wiedemann, 1819, by monotypy.

Family-group name: XYLORHIZIDES Lacordaire, 1872. Stem: *Xylorhiz*-.

***Xylosteus* Frivaldszky von Frivald, 1837**: 180. Current status: valid genus in CERAMBYCIDAE: LEPTURINAE: XYLOSTEINI.

Type species: *Xylosteusspinolae* Frivaldszky von Frivald, 1837, by monotypy.

Family-group name: XYLOSTEINA Reitter, 1913. Stem: *Xyloste*-.

***Xyloterus* Erichson, 1836**: 60. Current status: junior synonym of *Trypodendron* Stephens, 1830 in CURCULIONIDAE: SCOLYTINAE: XYLOTERINI.

Type species: *Bostrichuslineatus* G.-A. Olivier, 1795, by subsequent designation (C.G. Thomson 1859: 146).

Family-group name: XYLOTERI J.L. LeConte, 1876. Stem: *Xyloter*-.

***Xylotrogus* Stephens, 1830**: 116. Current status: valid genus in BOSTRICHIDAE: LYCTINAE: LYCTINI.

Type species: *Xylotrogusbrunneus* Stephens, 1830, by monotypy (see ICZN 1994d).

Family-group name: XYLOTROGIDAE Schönfeldt, 1887. Stem: *Xylotrog*-.

***Xylotrupes* Hope, 1837**: 15, 19, 29, 30. Current status: valid genus in SCARABAEIDAE: DYNASTINAE: DYNASTINI: XYLOTRUPINA.

Type species [suggested]: *Scarabaeusgideon* Linnaeus, 1767, by subsequent designation (E. Desmarest 1860: 92). Note: the valid type species fixation is that of Shuckard in Swainson and Shuckard (1840: 216), who selected *Scarabaeuscentaurus* Fabricius, 1775, but that species is currently included in the genus *Augosoma* Burmeister, 1841; an application to the Commission is necessary to fix *Scarabaeusgideon* Linnaeus, 1767 as the type species of *Xylotrupes* Hope, 1837.

Family-group name: XYLOTRUPIDAE Hope, 1838. Stem: *Xylotrup*-.

***Xyroscelis* Saunders, 1868**: 53. Current status: valid genus in BUPRESTIDAE: POLYCESTINAE: XYROSCELIDINI.

Type species: *Amorphosomacrocatum* Gory & Laporte, 1839, by monotypy.

Family-group name: XIROSCELINI Cobos, 1955. Stem: *Xyroscelid*-.

***Xystosomus* Schaum, 1863b**: 89. Current status: valid genus in CARABIDAE: TRECHINAE: TACHYINI: XYSTOSOMINA.

Type species: *Tachysinflatus* Schaum, 1859, by subsequent designation (Erwin 1973: 4).

Family-group name: XYSTOSOMINA Erwin, 1994. Stem: *Xystosom*-.

***Xystrocera* Audinet-Serville, 1834b**: 69. Current status: valid genus in CERAMBYCIDAE: CERAMBYCINAE: XYSTROCERINI.

Type species: *Cerambyxglobosus* G.-A. Olivier, 1795, by subsequent designation (J. Thomson 1864: 247).

Family-group name: XYSTROCÉRITES Blanchard, 1845. Stem: *Xystrocer*-.

***Xystropus* Solier, 1835b**: 241. Current status: valid genus in TENEBRIONIDAE: ALLECULINAE: ALLECULINI: XYSTROPODINA.

Type species: *Helopspilosus* Solier, 1835, by monotypy. Note: see Bousquet et al. (2015: 139) for comments regarding the species originally included in this genus.

Family-group name: XYSTROPIDES Solier, 1835. Stem: *Xystropod*-.

***Yanga* Muona, 1993**: 25. Current status: invalid senior synonym of *Muonaja* Özdikmen, 2008 in EUCNEMIDAE: EUCNEMINAE: MUONAJINI. Note: junior homonym of *Yanga* Distant, 1904 [Hemiptera].

Type species: *Yangayonde* Muona, 1993, by original designation.

Family-group name: YANGINI Muona, 1993. Stem: *Yang*-. Note: family-group name permanently invalid (ICZN 1999a, Article 39).

†***Yixianscarabaeus* Nikolajev, 2015b**: 299. Current status: valid genus in SCARABAEIDAE: EREMAZINAE: †YIXIANSCARABAEINI. Note: Lower Cretaceous (China).

Type species: †*Yixianscarabaeussulcatus* Nikolajev, 2015, by original designation.

Family-group name: †YIXIANSCARABAEINAE Nikolajev, 2015b: 298. Stem: *Yixianscarabae*-.

***Zabrus* Clairville, 1806**: 80, 81. Current status: valid genus in CARABIDAE: HARPALINAE: ZABRINI: ZABRINA.

Type species: *Carabusgibbus* Fabricius, 1794 (= *Carabustenebrioides* Goeze, 1777), by monotypy. Note: Goeze’s (1777) work is not consistently binominal (see ICZN 2021a) and therefore an application to the Commission is necessary to retain *Carabustenebrioides* Goeze, 1777 as the valid name for the type species.

Family-group name: ZABRIDES Bonelli, 1810. Stem: *Zabr*-.

***Zacotus* J.L. LeConte, 1869a**: 373. Current status: valid genus in CARABIDAE: BROSCINAE: BROSCINI: BROSCINA.

Type species: *Zacotusmatthewsii* J.L. LeConte, 1869, by monotypy.

Family-group name: ZACOTINI G.H. Horn, 1881. Stem: *Zacot*-.

***Zadenos* Laporte, 1840b**: 210. Current status: valid subgenus of *Selenepistoma* Dejean, 1834 in TENEBRIONIDAE: BLAPTINAE: DENDARINI: MELAMBIINA.

Type species: *Opatrumlongipalpe* Wiedemann, 1823, by monotypy.

Family-group name: ZADENINA C. Koch, 1956. Stem: *Zaden*-.

***Zaplous* J.L. LeConte, 1878a**: 415. Current status: valid genus in CERAMBYCIDAE: LAMIINAE: POGONOCHERINI.

Type species: *Zaploushubbardi* J.L. LeConte, 1878 (= *Ecyrusannulatus* Chevrolat, 1862), by monotypy.

Family-group name: ZAPLOI J.L. LeConte & G.H. Horn, 1883. Stem: *Zaplo*-.

***Zarax* Pascoe, 1867b**: 410. Current status: junior synonym of *Eurypoda* Saunders, 1853 in CERAMBYCIDAE: PRIONINAE: EURYPODINI.

Type species: *Zaraxeurypodioides* Pascoe, 1867 (= *Eurypodanigrita* J. Thomson, 1865), by monotypy.

Family-group name: ZARACIDES Lacordaire, 1868. Stem: *Zarac*-.

***Zarudniola* Semenov-Tjan-Shansky & Martynov in Martynov, 1925**: 74. Current status: junior synonym of *Karumia* Escalera, 1913 in DASCILLIDAE: KARUMIINAE: KARUMIINI.

Type species: *Zarudniolastaphylinus* Semenov-Tjan-Shansky & Martynov, 1925, by monotypy.

Family-group name: ZARUDNIOLIDAE Semenov-Tjan-Shansky & Martynov, 1925. Stem: *Zarudniol*-.

***Zelliboria* Lane, 1951**: 5. Current status: valid genus in CERAMBYCIDAE: CERAMBYCINAE: PIEZOCERINI: PIEZOCERINA.

Type species: *Rhagiumdaedaleum* Perty, 1832, by original designation.

Family-group name: ZELLIBORIINAE Lane, 1951. Stem: *Zellibori*-.

***Zelma* Andrewes, 1920**: 451. Current status: junior synonym of *Salcedia* Fairmaire, 1899 in CARABIDAE: SCARITINAE: SALCEDIINI: SALCEDIINA.

Type species: *Zelmamiranda* Andrewes, 1920, by monotypy.

Family-group name: ZELMIDES Andrewes, 1929. Stem: *Zelm*-.

***Zenoa* Say, 1835**: 153. Current status: valid genus in CALLIRHIPIDAE.

Type species: *Sandalusbrunneus* Say, 1835 (= *Melasispicea* Palisot de Beauvois 1805), by monotypy.

Family-group name: ZENOINI J.L. LeConte, 1866. Stem: *Zeno*-.

***Zenodosus* Wolcott, 1910**: 321. Current status: valid genus in THANEROCLERIDAE: ZENODOSINAE.

Type species: *Clerussanguineus* Say, 1835, by original designation.

Family-group name: ZENODOSINI Kolibáč, 1992. Stem: *Zenodos*-.

***Zethopsus* Reitter, 1880**: 85. Current status: valid genus in STAPHYLINIDAE: PSELAPHINAE: EUPLECTITAE: BYTHINOPLECTINI: BYTHINOPLECTINA. Note: new replacement name for *Zethus* L.W. Schaufuss, 1872.

Type species: *Zethusopacus* L.W. Schaufuss, 1877, by subsequent monotypy (L.W. Schaufuss 1877a: 12) (type fixation for *Zethus* L.W. Schaufuss).

Family-group name: ZETHOPSINA Jeannel, 1952. Stem: *Zethops*-.

***Zethus* L.W. Schaufuss, 1872**: 246. Current status: invalid senior synonym of *Zethopsus* Reitter, 1880 in STAPHYLINIDAE: PSELAPHINAE: EUPLECTITAE: BYTHINOPLECTINI: BYTHINOPLECTINA. Note: junior homonym of *Zethus* Fabricius, 1805 [Hymenoptera].

Type species: *Zethusopacus* L.W. Schaufuss, 1877, by subsequent monotypy (L.W. Schaufuss 1877a: 12).

Family-group name: ZETHINI L.W. Schaufuss, 1890. Stem: *Zeth*-. Note: family-group name permanently invalid (ICZN 1999a, Article 39).

***Zeugophora* Kunze, 1818**: 71. Current status: valid genus in MEGALOPODIDAE: ZEUGOPHORINAE. Note: name placed on the Official List of Generic Names in Zoology (ICZN 1986a).

Type species: *Criocerissubspinosa* Fabricius, 1781, by subsequent designation (Westwood 1838c: 42; see ICZN 1986a). Note: name placed on the Official List of Specific Names in Zoology (ICZN 1986a).

Family-group name: ZEUGOPHORINAE Böving & Craighead, 1931. Stem: *Zeugophor*-.

***Zherichinixena* Legalov, 2009b**: 309, 310. Current status: valid genus in BELIDAE: OXYCORYNINAE: OXYCORYNITAE: METRIOXENINI: ZHERICHINIXENINA.

Type species: *Zherichinixenanigra* Legalov, 2009, by original designation.

Family-group name: ZHERICHINIXENINA Legalov, 2009. Stem: *Zherichinixen*-.

***Zilora* Mulsant, 1856b**: 84. Current status: valid genus in MELANDRYIDAE: MELANDRYINAE: ZILORINI.

Type species: **here fixed** (under ICZN 1999a, Article 70.3.2) as *Parnusobscurus* Fabricius, 1794, misidentified as *Xylitaferruginea* Paykull, 1798 (sensu Mulsant, 1856) in the original designation by monotypy in Mulsant (1856b: 84).

Family-group name: ZILORINI Desbrochers des Loges, 1900. Stem: *Zilor*-.

***Zilus* Mulsant, 1850**: 958. Current status: valid genus in COCCINELLIDAE: COCCINELLINAE: COCCIDULINI.

Type species: *Scymnusfulvipes* Mulsant, 1850, by monotypy.

Family-group name: ZILINI Gordon, 1985. Stem: *Zil*-.

***Zolinopatrobus* Deuve & Tian, 2001**: 418. Current status: junior synonym of *Lissopogonus* Andrewes, 1923 in CARABIDAE: PATROBINAE: LISSOPOGONINI.

Type species: *Zolinopatrobusnanlingensis* Deuve & Tian, 2001, by original designation.

Family-group name: ZOLINOPATROBINA Deuve & Tian, 2001. Stem: *Zolinopatrob*-.

***Zimmiellus* Kuschel in Kuschel & Leschen, 2011**: 598. Current status: valid genus in NEMONYCHIDAE: RHINORHYNCHINAE: ZIMMIELLINI.

Type species: *Zimmiellusfronto* Kuschel, 2011, by original designation.

Family-group name: ZIMMIELLINI Legalov, 2023c: 21. Stem: *Zimmiell*-. Note: ZIMMIELLINI (Legalov, 2017a: 71) was published in a work that is nomenclaturally unavailable (ICZN 2012f, Article 8.5.3).

***Zolodinus* Blanchard, 1853**: 159. Current status: valid genus in TENEBRIONIDAE: ZOLODININAE.

Type species: *Zolodinuszelandicus* Blanchard, 1853, by monotypy.

Family-group name: ZOLODININAE Watt, 1975. Stem: *Zolodin*-.

***Zoltanzonitis* Bologna & Pinto, 2018**: 438. Current status: valid genus in MELOIDAE: NEMOGNATHINAE: ZOLTANZONITINI.

Type species: *Zonitisnatala* Beauregard, 1890, by original designation.

Family-group name: ZOLTANZONITINI Riccieri, Capogna, Pinto & Bologna, 2023: 109. Stem: *Zoltanzonit*- (ICZN 1999a, Article 29.4).

***Zolus* Sharp, 1886**: 371. Current status: junior synonym of *Oopterus* Guérin-Méneville, 1841 in CARABIDAE: TRECHINAE: ZOLINI.

Type species: *Zolushelmsi* Sharp, 1886, by monotypy.

Family-group name: ZOLINI Sharp, 1886. Stem: *Zol*-.

***Zonabris* Harold, 1879**: 134. Current status: junior synonym of *Mylabris* Fabricius, 1775 in MELOIDAE: MELOINAE: MYLABRINI. Note: new replacement name for *Mylabris* Fabricius, 1775.

Type species: *Meloecichorii* Linnaeus, 1758, by subsequent designation (Latreille 1810: 430) (see nomenclatural problem with typification of *Mylabris* Fabricius).

Family-group name: ZONABRINI Wellman, 1908. Stem: *Zonabr*-.

***Zonitis* Fabricius, 1775**: 126. Current status: valid genus in MELOIDAE: NEMOGNATHINAE: NEMOGNATHINI: ZONITIDINA. Note: name placed on the Official List of Generic Names in Zoology (ICZN 1999c).

Type species: *Zonitisflava* Fabriciuis, 1775, by subsequent designation (Selander 1987: 341; see ICZN 1999c). name placed on the Official List of Specific Names in Zoology (ICZN 1999c).

Family-group name: ZONITAIRES Mulsant, 1857. Stem: *Zonitid*- (ICZN 1999c).

***Zopheromimus* Alexeev & Nabozhenko, 2023**: 438. Current status: valid genus in ZOPHERIDAE: ZOPHERINAE: ZOPHEROMIMINI.

Type species: *Zopheromimusauriborussiensis* Alexeev & Nabozhenko, 2023, by original designation. Note: combined description of a new genus and a single new species (ICZN 1999a, Article 13.4).

Family-group name: ZOPHEROMIMINI Alexeev & Nabozhenko, 2023: 437. Stem: *Zopheromim*-.

***Zopherosis* A. White, 1859**: 121. Current status: valid genus in ZOPHERIDAE: ZOPHERINAE: ZOPHERINI.

Type species: *Zopherosisgeorgii* A. White, 1859, by monotypy.

Family-group name: ZOPHEROSINI Casey, 1907. Stem: *Zopherose*-.

***Zopherus* G.R. Gray in Griffith and Pidgeon, 1831**: pls 50, 69. Current status: valid genus in ZOPHERIDAE: ZOPHERINAE: ZOPHERINI.

Type species: *Zopherusmexicanus* G.R. Gray, 1831, by monotypy. Note: a second species in this genus, *Zopheruschilensis* G.R. Gray, 1832, was made available in the second volume of this work published one year later (Griffith and Pidgeon 1832: 796, pl. 124).

Family-group name: ZOPHÉRITES Solier, 1834. Stem: *Zopher*-.

***Zophosis* Latreille, 1802b**: 167. Current status: valid genus in TENEBRIONIDAE: PIMELIINAE: ZOPHOSINI.

Type species: *Erodiustestudinarius* Fabricius, 1781, by monotypy.

Family-group name: ZOPHOSITES Solier, 1834. Stem: *Zophos*-.

***Zuphium* Latreille, 1805**: 198, 227. Current status: valid genus in CARABIDAE: HARPALINAE: ZUPHIINI.

Type species: *Carabusolens* Rossi, 1790, by subsequent designation (Latreille 1810: 426).

Family-group name: ZUPHIETAE Bonelli, 1810. Stem: *Zuphi*-.

***Zygaenodes* Pascoe, 1859b**: 328. Current status: junior synonym of *Exechesops* Schönherr, 1847 in ANTHRIBIDAE: ANTHRIBINAE: ZYGAENODINI.

Type species: *Zygaenodeswollastoni* Pascoe, 1859, by monotypy.

Family-group name: ZYGÉNODIDES Lacordaire, 1865. Stem: *Zygaenod*-.

***Zygia* Fabricius, 1775**: 126. Current status: junior synonym of *Melyris* Fabricius, 1775 in MELYRIDAE: MELYRINAE: MELYRINI. Note: the First Reviser (ICZN 1999a, Article 24.2.2) for *Melyris* Fabricius, 1775 versus *Zygia* Fabricius, 1775 is Latreille (1816: 243).

Type species: *Zygiaoblonga* Fabricius, 1775, by monotypy.

Family-group name: ZYGIIDAE Jakobson, 1911. Stem: *Zygi*-.

***Zygobaris* J.L. LeConte in J.L. LeConte and G.H. Horn, 1876**: 301, 317. Current status: valid genus in CURCULIONIDAE: BARIDINAE: APOSTASIMERINI: DIORYMERINA.

Type species: *Zygobarisnitens* J.L. LeConte, 1876, by subsequent designation (Champion 1908: 330).

Family-group name: ZYGOBARI Pierce, 1907. Stem: *Zygobarid*-.

***Zygocera* Erichson, 1842**: 223. Current status: valid genus in CERAMBYCIDAE: LAMIINAE: ZYGOCERINI. Note: this name was first used and made available by Dejean (1835: 344) but in a different taxonomic sense; we believe the best avenue is to apply to the Commission to suppress Dejean’s name for the purpose of the Principles of Priority and Homonymy (see Bousquet and Bouchard 2013a: 95).

Type species: *Zygoceracanosa* Erichson, 1842, by monotypy.

Family-group name: ZYGOCERITAE J. Thomson, 1864. Stem: *Zygocer*-.

***Zygogramma* Chevrolat in Dejean, 1836a**: 398. Current status: valid genus in CHRYSOMELIDAE: CHRYSOMELINAE: CHRYSOMELINI.

Type species: *Chrysomelapulcra* Fabricius, 1792 (= *Chrysomelasuturalis* Fabricius, 1775), by subsequent designation (Motschulsky 1860b: 181).

Family-group name: ZYGOGRAMMINI Weise, 1915. Stem: *Zygogrammat*-.

***Zygops* Schönherr, 1825**: 586. Current status: valid genus in CURCULIONIDAE: CONODERINAE: ZYGOPINI. Note: name placed on the Official List of Generic Names in Zoology (ICZN 1987e).

Type species: *Poecilmawiedii* Germar, 1823, by original designation (see ICZN 1987e). Note: placed on the Official List of Specific Names in Zoology (ICZN 1987e, as *Poecilmawiedii* Germar, 1824).

Family-group name: ZYGOPIDES Lacordaire, 1865. Stem: *Zygop*-.

***Zyras* Stephens, 1835a**: 430. Current status: valid genus in STAPHYLINIDAE: ALEOCHARINAE: LOMECHUSINI: MYRMEDONIINA. Note: name placed on the Official List of Generic Names in Zoology (ICZN 1961a).

Type species: *Aleocharahaworthi* Stephens, 1832, by monotypy (see ICZN 1961a). Note: name placed on the Official List of Specific Names in Zoology (ICZN 1961a).

Family-group name: ZYRINI Bradley, 1930. Stem: *Zyr*-.

## Footnotes

Heer (1864) used two spellings for this name, Byrrhidium (p. 64, 65, pl.viii) and Byrrhydium (p. 89); Neave (1939: 507) acted as First Reviser (ICZN 1999a, Article 24.2) and selected Byrrhidium.

Original vernacular name available: first used in latinized form and generally accepted (e.g., Anonymous 1842: 141).

## ﻿Additional information

### Author ORCIDs

Patrice Bouchard https://orcid.org/0000-0001-7443-4547

Anthony Davies https://orcid.org/0009-0007-9506-9017

Chenyang Cai https://orcid.org/0000-0002-9283-8323

### Data availability

The data that support the findings of this study are available from the authors upon request.

## ﻿Appendix 1

Annotated classification of the order Coleoptera. All known nomenclaturally available family-group names are included, except for the names based on misidentified type genera, which are listed in Table 1. The classification, down to the rank of subfamily, follows Cai et al. (2022) unless indicated otherwise. Alternative placement of taxa in other classifications is sometimes indicated in footnotes. Valid tribes and subtribes are listed in alphabetical order while their synonyms, which are preceded by “=”, are listed in chronological order. Unresolved nomenclatural problems based on the Principle of Priority and the Principle of Homonymy (ICZN 1999a) are indicated with the symbol ^P^ and ^H^ respectively (for additional details see Bouchard et al. 2011, Bouchard and Bousquet 2020 and this work). Nomenclatural problems associated with a misidentification or altered concept of its type genus are indicated with the symbol ^M^. Family-group names that have been suppressed by the Commission are followed by the symbol ^S^. ^I^ = ichnotaxon, [NO] = nomen oblitum, [NP] = nomen protectum, [PI] = permanently invalid, * = nomenclaturally unavailable (see comments in section Availability and date of publication of electronic works published after 2011).

**ORDER COLEOPTERA** ^3^

**SUBORDER ALPHACOLEOPTera**

**Superfamily Coleopsoidea Kirejtshuk & Nel, 2016**†

**Family Coleopsidae Kirejtshuk & Nel, 2016**†

**Superfamily Permocupedoidea Martynov, 1933**†

**Family Tshekardocoleidae Rohdendorf, 1944**†

= Uralocoleidae Zalesskiy, 1947†

= Moravocoleidae Kukalova-Peck & Beutel, 2012†^4^

**Family Labradorocoleidae Ponomarenko, 1969**†

**Family Oborocoleidae Kukalová, 1969**†

**Family Permocupedidae Martynov, 1933**†

Subfamily Permocupedinae Martynov, 1933†

= Kaltanocoleinae Rohdendorf, 1961†

Subfamily Taldycupedinae Rohdendorf, 1961†

**Superfamily Permosynoidea Tillyard, 1924**†

**Family Ademosynidae Ponomarenko, 1968**† ^5^

**Family Permosynidae Tillyard, 1924**†

**CAPAXORDER ZACOLEOPTera**

**SUBORDER METAXYCOLEOPTera**

**Family Ponomarenkiumidae Lawrence & Bouchard, 2022**† ^6^

= Ponomarenkiidae Yan et al., 2017† [PI]

**HYPORDER EUCOLEOPTera**

**SUBORDER ADEPHAGA**

**Family Tritarsusidae Hong, 2002**†

**Family Triaplidae Ponomarenko, 1977**† ^7^

**Family Haliplidae Aubé, 1836**

= Peltodytidae Böving & Craighead, 1931

= Brychiidae Ádám, 1996

**Family Gyrinidae Latreille, 1810** ^8^

Subfamily Spanglerogyrinae Folkerts, 1979

Subfamily Gyrininae Latreille, 1810

Tribe Dineutini Desmarest, 1851

Subtribe Dineutina Desmarest, 1851

Subtribe Enhydrusina Branden, 1882

= Aulonogyrina Ochs, 1953

= Prothydrina Guignot, 1954 [PI] ^S^

Tribe Gyrinini Latreille, 1810

Tribe Orectochilini Kolbe, 1880

Subfamily Heterogyrinae Brinck, 1956

**Family Coptoclavidae Ponomarenko, 1961**†

Subfamily Timarchopsinae B. Wang et al., 2010†

= Necronectinae Ponomarenko, 1977† [PI]

Subfamily Charonoscaphinae Ponomarenko, 1977†

Subfamily Coptoclavinae Ponomarenko, 1961†

Subfamily CoptoclaviscinaeSoriano et al., 2007†

Subfamily HispanoclavinaeSoriano et al., 2007†

**Family Noteridae C.G. Thomson, 1860** ^9^

Subfamily Notomicrinae Branden, 1884

Tribe Notomicrini Branden, 1884

Tribe Phreatodytini Uéno, 1957

Subfamily Noterinae C.G. Thomson, 1860

Tribe Noterini C.G. Thomson, 1860

= Hydrocanthini Sharp, 1880

= Suphidini Sharp, 1880

= Neohydrocoptini Zalat et al., 2000

= Pronoterini Nilsson, 2005

= Tonerini K.B. Miller, 2009

**Family Meruidae Spangler & Steiner, 2005**

**Family AspidytidaeRibera et al., 2002**

**Family Amphizoidae J.L. LeConte, 1853**

**Family Hygrobiidae Régimbart, 1879 (1837)**

= Paelobiidae Erichson, 1837

**Family Dytiscidae Leach, 1815** ^10^

Subfamily Matinae Branden, 1884

Subfamily Lancetinae Branden, 1884

Subfamily Agabinae C.G. Thomson, 1867

Tribe Agabini C.G. Thomson, 1867

= Agabinini Crotch, 1873

= Ilybiini Acloque, 1895

= Hydronebriini Guignot, 1948

Tribe Hydrotrupini Roughley, 2000

Tribe *Platynectini Toussaint & Balke, 2017 ^11^

Subfamily Colymbetinae Erichson, 1837

= Cymatopterinae Portevin, 1929

= Anisomeriinae Brinck, 1948

= Carabdytinae Pederzani, 1995

Subfamily Copelatinae Branden, 1884

= Aglymbinae Branden, 1884

= Lacconectinae Branden, 1885

Subfamily Laccophilinae Gistel, 1848

Tribe Agabetini Branden, 1884

Tribe Laccophilini Gistel, 1848

Subfamily Cybisterinae Sharp, 1880

Subfamily Dytiscinae Leach, 1815

Tribe Aciliini C.G. Thomson, 1867

= Thermonectini Sharp, 1880

= Graphoderini Houlbert, 1934

Tribe AmbarticiniYang et al., 2019†

Tribe Aubehydrini Guignot, 1942

Tribe Dytiscini Leach, 1815

= Hyderodini K.B. Miller, 2000

Tribe Eretini Crotch, 1873

= Eunectini Portevin, 1929 [PI]

Tribe Hydaticini Sharp, 1880

Subfamily Coptotominae Branden, 1885

Subfamily Hydrodytinae K.B. Miller, 2001

Subfamily Hydroporinae Aubé, 1836

Tribe Bidessini Sharp, 1880

Tribe Hydroporini Aubé, 1836

Subtribe Deronectina Galewski, 1994

Subtribe Hydroporina Aubé, 1836

= Hydrocoptina Kolbe, 1883

Subtribe Siettitiina Smrž, 1982

Subtribe Sternopriscina Branden, 1884

= Carabhydrina Watts, 1978

Tribe Hydrovatini Sharp, 1880

Tribe Hygrotini Portevin, 1929

Tribe Hyphydrini Gistel, 1848

= Actobaenini Gistel, 1856

Tribe Laccornellini K.B. Miller & Bergsten, 2014

Tribe Laccornini Wolfe & Roughley, 1990

Tribe Methlini Branden, 1884

= Celinini Branden, 1884

Tribe Pachydrini Young, 1980

Tribe Schistomerini Palmer, 1957†

Tribe Vatellini Sharp, 1880

Subfamily Palaeogyrininae Schlechtendal, 1894†

Subfamily Liadytiscinae Prokin & Ren, 2010†

Tribe Liadytiscini Prokin & Ren, 2010†

Tribe MesoderiniProkin et al., 2013†

**Family Colymbotethidae Ponomarenko, 1994**†

**Family Parahygrobiidae Ponomarenko, 1977**†

**Family Liadytidae Ponomarenko, 1977**†

**Family Trachypachidae C.G. Thomson, 1857**

Subfamily Eodromeinae Ponomarenko, 1977†

= Leptopodocoleinae Hong, 1982†

Subfamily Trachypachinae C.G. Thomson, 1857

= Systolosomatinae Erwin, 1991

**Family Cicindelidae Latreille, 1802** ^12^

Tribe Cicindelini Latreille, 1802

Subtribe Apteroessina Rivalier, 1971

Subtribe Cicindelina Latreille, 1802

= Euryodina W. Horn, 1899

Subtribe Dromicina J. Thomson, 1859 ^H^

= Caledonicina W. Horn, 1893

= Odontocheilina W. Horn, 1899

= Prepusina W. Horn, 1899

= Prothymina W. Horn, 1906

Subtribe Iresiina Rivalier, 1971 ^P^

= Distipsiderina W. Horn, 1893

= Eucalliina W. Horn, 1893

= Euprosopina W. Horn, 1893

Subtribe Theratina W. Horn, 1893

Tribe Collyridini Brullé, 1834

Subtribe Collyridina Brullé, 1834

= Tracheloniina Gistel, 1850

Subtribe Tricondylina Naviaux, 1991

Tribe Ctenostomatini Laporte, 1834

= Pogonostomatini Mandl, 1954

Tribe Manticorini Laporte, 1834

= Platychilini W. Horn, 1893

= Amblycheilini Csiki, 1903

= Omini W. Horn, 1907

Tribe Megacephalini Laporte, 1834

= Tetrachini Leng & Mutchler, 1916

Tribe Oxycheilini J. Thomson, 1857

**Family Carabidae Latreille, 1802**

Subfamily Protorabinae Ponomarenko, 1977†

Subfamily Conjunctiinae Ponomarenko, 1977†

Subfamily Rhysodinae Laporte, 1840

Tribe Clinidiini R.T. Bell & J.R. Bell, 1978

Tribe Dhysorini R.T. Bell & J.R. Bell, 1978

Tribe Leoglymmiini R.T. Bell & J.R. Bell, 1978

Tribe Medisorini R.T. Bell & J.R. Bell, 1987

Tribe Omoglymmiini R.T. Bell & J.R. Bell, 1978

Tribe Rhysodini Laporte, 1840

Tribe Sloanoglymmiini R.T. Bell & J.R. Bell, 1991

Subfamily Nebriinae Laporte, 1834

Tribe Nebriini Laporte, 1834

Tribe Notiokasiini Kavanaugh & Nègre, 1983

Tribe Notiophilini Motschulsky, 1850

Tribe Opisthiini Dupuis, 1912

Tribe Pelophilini Kavanaugh, 1996

Subfamily Carabinae Latreille, 1802

Tribe Carabini Latreille, 1802

Subtribe Calosomatina Bonelli, 1810

= Callisthenina Gistel, 1848

= Aplothoracina Lapouge, 1927

Subtribe Carabina Latreille, 1802

= Procerina Laporte, 1834

= Procrustina Roeschke, 1898

= Cechenina Csiki, 1927 [PI]

= Megodontina Csiki, 1927

= Tribacina Csiki, 1927

Tribe Ceroglossini Lapouge, 1927

Tribe Cychrini Perty, 1830

Tribe Pamborini Hope, 1838

Subfamily Loricerinae Bonelli, 1810

Subfamily Omophroninae Bonelli, 1810

= Scolytinae Motschulsky, 1850 [PI]

= Epactiinae Fauvel, 1888

Subfamily Elaphrinae Latreille, 1802

Subfamily Migadopinae Chaudoir, 1861

Tribe Amarotypini Erwin, 1985

Tribe Migadopini Chaudoir, 1861

Subtribe Aquilicina Moret, 2005

Subtribe Migadopina Chaudoir, 1861

= Monolobina Jeannel, 1938

= Loxomerina Erwin, 1985

Subfamily Hiletinae Schiødte, 1847

= Camaragnathinae Csiki, 1927

Subfamily Scaritinae Bonelli, 1810

Tribe Carenini W.J. MacLeay, 1887

Tribe Clivinini Rafinesque, 1815

Subtribe Ardistomina Putzeys, 1867

Subtribe Clivinina Rafinesque, 1815

= Italodytina Jeannel, 1957

= Reicheiina Jeannel, 1957

Subtribe Schizogeniina Dostal, 2017

Subtribe Sparostesina Dostal, 2017

Subtribe Thliboclivinina Balkenohl, 2022

Tribe Dalyatini Mateu, 2002

Tribe Dyschiriini Kolbe, 1880

Tribe Forcipatorini Bänninger, 1938

= Oxystomini Csiki, 1927 [PI]

Tribe Palaeoaxinidiini McKay, 1991†

Tribe Pasimachini Putzeys, 1867

Tribe Promecognathini J.L. LeConte, 1853

= Axinidiini Basilewsky, 1963

Tribe Salcediini Alluaud, 1930 (1929)

Subtribe Androzelmina R.T. Bell, 1998

Subtribe Salcediina Alluaud, 1930 (1929)

= Zelmina Andrewes, 1929

Subtribe Solenogenyina R.T. Bell, 1998

Tribe Scaritini Bonelli, 1810

Subtribe Acanthoscelina Csiki, 1927

Subtribe Corintascarina Basilewsky, 1973

Subtribe Dyscherina Basilewsky, 1973

Subtribe Ochyropina Basilewsky, 1973

Subtribe Oxylobina Andrewes, 1929

Subtribe Scapterina Putzeys, 1867

= Passalidiina Csiki, 1927

Subtribe Scaritina Bonelli, 1810

Subtribe Storthodontina Jeannel, 1946

Subfamily Broscinae Hope, 1838

Tribe Broscini Hope, 1838

Subtribe Axonyina Roig-Juñent, 2000

Subtribe Baripodina Jeannel, 1941

Subtribe Broscina Hope, 1838

= Cephalotina Heer, 1838 [PI]

= Zacotina G.H. Horn, 1881

Subtribe Creobiina Jeannel, 1941

Subtribe Nothobroscina Roig-Juñent, 2000

Subfamily Apotominae J.L. LeConte, 1853

Subfamily Siagoninae Bonelli, 1813

Tribe Enceladini G.H. Horn, 1881

Tribe Lupercini Lecordier, 1977

Tribe Siagonini Bonelli, 1813

Subfamily Melaeninae Csiki, 1933

= Cosciniinae Chaudoir, 1876 [PI]

= Cymbionotinae Andrewes, 1933

Subfamily Gehringiinae Darlington, 1933

Tribe Gehringiini Darlington, 1933

Subtribe Gehringiina Darlington, 1933

Subtribe Helenaeina Deuve, 2007

Subfamily Trechinae Bonelli, 1810 ^13^

Tribe Anillini Jeannel, 1937

= Scotodipnini Jeannel, 1937

= Typhlocharitini Jeanne, 1973

Tribe Bembidarenini Maddison & Erwin, 2019

Tribe Bembidiini Stephens, 1827

= Peryphini W. Kirby, 1837

Tribe Horologionini Jeannel, 1949

Tribe LovriciiniGiachino et al., 2011

Tribe Pogonini Laporte, 1834

= Pogonopseini Bedel, 1900

Tribe Sinozolini Deuve, 1997

Subtribe Chalteniina Roig-Juñent & Cicchino, 2001

Subtribe Sinozolina Deuve, 1997

Tribe Tachyini Motschulsky, 1862

Subtribe Tachyina Motschulsky, 1862

= Micratopina Casey, 1914

= Lymnastina Jeannel, 1937

Subtribe Xystosomina Erwin, 1994

Tribe Trechini Bonelli, 1810

Subtribe Trechina Bonelli, 1810

= Aepina Fowler, 1887

= Temnostegina Enderlein, 1909

= Omaloderina Jeannel, 1926

= Plocamotrechina Jeannel, 1960

Subtribe Trechodina Jeannel, 1926 ^P^

= Perileptina Sloane, 1903

= Ochthephilina Jeannel, 1922 [PI]

= Thalassophilina Csiki, 1928

= Cnidina Jeannel, 1958

Tribe Zolini Sharp, 1886

= Merizodontini Sloane, 1920

= Oopterini Jeannel, 1940

Subfamily Patrobinae W. Kirby, 1837

Tribe Lissopogonini Zamotajlov, 2000

= Zolinopatrobini Deuve & Tian, 2001

Tribe Patrobini W. Kirby, 1837

Subtribe Deltomerina Chaudoir, 1871

Subtribe Deltomerodina Zamotajlov, 2002

Subtribe Patrobina W. Kirby, 1837

Subtribe Platidiolina Zamotajlov & Lafer, 2001

Subfamily Psydrinae J.L. LeConte, 1853

Tribe Moriomorphini Sloane, 1890 ^14^

Subtribe Amblytelina Blackburn, 1892 ^H^

= Meonina Sloane, 1898

= Mecyclothoracina Jeannel, 1940

Subtribe Moriomorphina Sloane, 1890

= Melisoderina Sloane, 1898

Subtribe Tropopterina Sloane, 1898

Tribe Psydrini J.L. LeConte, 1853

= Nomiusini Gozis, 1875

Subfamily Nototylinae Bänninger, 1927

= Tylonotinae Schaum, 1863 [PI]

Subfamily Paussinae Latreille, 1806

Tribe KRYZHANOVSKIANINI Deuve, 2020† **new status**^15^

Tribe Metriini J.L. LeConte, 1853

Tribe Microzaenini Deuve, 2020

Tribe Mystropomini G.H. Horn, 1881

Tribe Ozaenini Hope, 1838

= Pseudozaenini Sloane, 1905

= Eustrini Jeannel, 1946

= Pachytelini Jeannel, 1946

= Physeini Jeannel, 1946

Tribe Paussini Latreille, 1806

Subtribe Arthropteritina Luna de Carvalho, 1961†

Subtribe Carabidomemnina Wasmann, 1928

= Eohomopterina Wasmann, 1929

Subtribe Cerapterina Billberg, 1820

= Megalopaussina Wasmann, 1920

= Arthropterina Wasmann, 1928

Subtribe Eopaussina Luna de Carvalho, 1951†

Subtribe Heteropaussina E. Janssens, 1950

= Pleuropterina Wasmann, 1920 [PI]

= Jansseniina Luna de Carvalho, 1951

Subtribe Homopterina Wasmann, 1920 ^H^

Subtribe Paussina Latreille, 1806

= Merismoderina Wasmann, 1929

= Enneapaussina Jeannel, 1946

= Platyrhopalina Jeannel, 1946 ^16^

= Ceratoderina Darlington, 1950 ^17^

= Hylotorina Luna de Carvalho, 1951

= Leleupaussina Luna de Carvalho, 1989

Subtribe Pentaplatarthrina Jeannel, 1946

= Hexaplatarthrina Luna de Carvalho, 1961

Tribe Protopaussini Gestro, 1892

Subfamily Cicindinae Csiki, 1927

Subfamily Brachininae Bonelli, 1810

Tribe Brachinini Bonelli, 1810

Subtribe Aptinina Gistel, 1848

= Styphlomerina Habu, 1984

Subtribe Brachinina Bonelli, 1810

Subtribe Mastacina Erwin, 1970

Subtribe Pheropsophina Jeannel, 1949

Tribe Crepidogastrini Jeannel, 1949

Subfamily Harpalinae Bonelli, 1810

Tribe Abacetini Chaudoir, 1873

Subtribe Abacetina Chaudoir, 1873

= Celioscheseina Jeannel, 1948

Subtribe Loxandrina Erwin & Sims, 1984

Tribe Amorphomerini Sloane, 1923

= Trimerini Alluaud, 1922 [PI]

Tribe Anthiini Bonelli, 1813

= Polyhirmini Rousseau, 1905

= Cypholobini G. Strohmeyer, 1928

Tribe Atranini G.H. Horn, 1881 ^18^

Tribe Bascanini Basilewsky, 1953

Tribe Calophaenini Jeannel, 1948

Tribe Catapieseini H.W. Bates, 1882

Tribe Chaetodactylini Tschitschérine, 1903 ^H^

Tribe Chaetogenyini Emden, 1958

Tribe Chlaeniini Brullé, 1834

Subtribe Callistina Laporte, 1834

= Eusynetina Gistel, 1856

Subtribe Chlaeniina Brullé, 1834

= Lissaucheniina Gistel, 1848

= Rhopalomelina Alluaud, 1930

= Chlaeniodina Jeannel, 1949

= Chlaenionina Jeannel, 1949

= Eccoptomenina Jeannel, 1949

= Callistoidina Basilewsky, 1950

= Harpaglossina Basilewsky, 1950

= Pleroticina Basilewsky, 1950

= Procletina Basilewsky, 1950

= Brachylobina Basilewsky & Grundmann, 1955

= Chlaenioctenina Basilewsky & Grundmann, 1955

= Leptodinodina Basilewsky & Grundmann, 1955

Tribe Cnemalobini Germain, 1911

Tribe Corsyrini Ganglbauer, 1891

= Discopterini Jedlička, 1941

Tribe Cratocerini Lacordaire, 1854

Tribe Ctenodactylini Laporte, 1834 ^H^

= Rhagocrepidini Chaudoir, 1848

Tribe Cuneipectini Sloane, 1907

Tribe Cyclosomini Laporte, 1834

= Tetragonoderini Chaudoir, 1871

= Sarothrocrepidini Chaudoir, 1876

Tribe Dercylini Sloane, 1923

Tribe Drimostomatini Chaudoir, 1872

= Caelostomini Burgeon, 1935

= Cyrtolaini Whitehead & Ball, 1975

Tribe Dryptini Bonelli, 1810

Tribe Enoicini Basilewsky, 1985

Tribe Galeritini W. Kirby, 1825

Subtribe Galeritina W. Kirby, 1825

= Galeritinina Jeannel, 1949 ^S^

= Galeritulina Jedlička, 1963 ^S^

Subtribe Planetina Jedlička, 1941

Tribe Geobaenini Péringuey, 1896

Tribe Ginemini Ball & Shpeley, 2002

Tribe Glyptini G.H. Horn, 1881 ^H^

Tribe Graphipterini Latreille, 1802

Tribe Harpalini Bonelli, 1810

Subtribe Amblystomina Fauvel, 1889

Subtribe Anisodactylina Lacordaire, 1854 [NP]

= Eurytrichina J.L. LeConte, 1847 [NO]

= Anisotarsina Csiki, 1932

Subtribe Ditomina Bonelli, 1810

= Pachycarina Stichel, 1923

= Machozetina Schauberger, 1934

= Eriotomina Antoine, 1959

Subtribe Harpalina Bonelli, 1810

= Acinopodina Laporte, 1834

= Ophonina Laporte, 1834

= Cratognathina Laporte, 1834

= Stenomorphina Laporte, 1834

= Daptina J.L. LeConte, 1847

= Trichopselaphina Tschitschérine, 1900

= Selenophorina Casey, 1914

= Bradybaenina Csiki, 1932 ^H^

= Diorychina Csiki, 1932

= Trichotichnina Jeannel, 1942

= Bleuseina Antoine, 1959

= Granigerina Antoine, 1959

= Cratacanthina Lindroth, 1968

Subtribe Pelmatellina H.W. Bates, 1882

Subtribe Stenolophina W. Kirby, 1837

= Polpochilina H.W. Bates, 1891

= Acupalpina Tschitschérine, 1900

= Cratocarina Casey, 1914

= Pachytrachelina Csiki, 1932

= Anoplogeniina Schauberger, 1937

= Bradycellina Jeannel, 1942

= Hippoloetina Basilewsky, 1951

= Anthracina Schuler, 1970

Tribe Helluonini Hope, 1838

Subtribe Helluonina Hope, 1838

Subtribe Omphrina Jedlička, 1941

= Helluomorphina H. Reichardt, 1974

Tribe Hexagoniini G.H. Horn, 1881 (1834)

= Trigonodactylini Brullé, 1834

Tribe Idiomorphini H.W. Bates, 1891

= Perochnoristhini Basilewsky, 1973

Tribe Lachnophorini J.L. LeConte, 1853

Subtribe Lachnophorina J.L. LeConte, 1853

= Anchonoderina Lacordaire, 1854

= Eucaerina J.L. LeConte, 1861

= Egina G.H. Horn, 1881

Subtribe Selinina Jeannel, 1948

Tribe Lebiini Bonelli, 1810

Subtribe Agrina W. Kirby, 1837

Subtribe Apenina Ball, 1983

Subtribe Calleidina Chaudoir, 1873

= Plochionina Gozis, 1875

= Anomotarina Habu, 1967

Subtribe Celaenephina Habu, 1982

Subtribe Cymindidina Laporte, 1834

= Tarina Gistel, 1848

= Pseudomasoreina Jeannel, 1942

Subtribe Demetriadina H.W. Bates, 1886

= Peliocypadina Basilewsky, 1984

Subtribe Dromiusina Bonelli, 1810

= Lichnasthenina J. Thomson, 1858

= Lionychina Jeannel, 1948

= Singilina Jeannel, 1949

= Metadromiina Basilewsky, 1984

= Metaxymorphina Basilewsky, 1984

= Singiliomimina Basilewsky, 1984

Subtribe Gallerucidiina Chaudoir, 1872

= Lebidiina Jakobson, 1907

Subtribe Lebiina Bonelli, 1810

= Encratina Gistel, 1856

= Lampriina Chaudoir, 1871

Subtribe Metallicina Basilewsky, 1984

Subtribe Nemotarsina H.W. Bates, 1883

Subtribe Pericalina Hope, 1838

= Coptoderina Chaudoir, 1848

= Mormolycina Desmarest, 1851

= Thyreopterina Chaudoir, 1869

= Eucheilina H.W. Bates, 1883

= Miscelina Sloane, 1907

= Periglossiina Liebke, 1929

= Catascopina Csiki, 1932

= Lobodontina Jeannel, 1949

= Thysanotina Jeannel, 1949

= Somotrichina Mateu, 1963

Subtribe Physoderina Chaudoir, 1877 ^H^

Subtribe Pseudotrechina Basilewsky, 1984

Subtribe Sugimotoina Habu, 1975

Subtribe Trichina Basilewsky, 1984

Tribe Licinini Bonelli, 1810 ^H^

Subtribe Dicaelina Laporte, 1834

= Rembina Gistel, 1848 [PI]

Subtribe Dicrochilina Ball, 1992

Subtribe Lestignathina Ball, 1992

Subtribe Licinina Bonelli, 1810 ^H^

= Badisterina Gistel, 1856

Tribe Masoreini Chaudoir, 1871

= Aephnidiini Jakobson, 1907

= Anaulacini Csiki, 1932

Tribe Melanchitonini Jeannel, 1948

= Melanodini Alluaud, 1916 [PI]

Tribe Microcheilini Jeannel, 1948

Tribe Morionini Brullé, 1835

Tribe Odacanthini Laporte, 1834 ^19^

Subtribe Actenonycina H.W. Bates, 1871

Subtribe Homethina Liebherr, 2016

Subtribe Odacanthina Laporte, 1834

= Cosnaniina J.L. LeConte, 1861

= Colliurina Bedel, 1910

= Lasiocerina Jeannel, 1948

Subtribe Pentagonicina H.W. Bates, 1873

= Scopodini H.W. Bates, 1874

Tribe Omphreini Ganglbauer, 1891

Tribe Oodini LaFerté-Sénectère, 1851

= Thryptocerini Jeannel, 1949

= Sphoerodini Jeannel, 1949

= Simoini Basilewsky, 1953

Tribe Orthogoniini Schaum, 1857

Tribe Panagaeini Bonelli, 1810

Subtribe Brachygnathina Basilewsky, 1946

Subtribe Panagaeina Bonelli, 1810

Subtribe Tefflina Basilewsky, 1946

Tribe Peleciini Chaudoir, 1880

Subtribe Agonicina Sloane, 1920

Subtribe Peleciina Chaudoir, 1880

= Disphericina Sloane, 1923

Tribe Perigonini G.H. Horn, 1881 [NP]

= Trechicini H.W. Bates, 1873 [NO]

Tribe Physocrotaphini Chaudoir, 1863

= Helluodini Csiki, 1932

Tribe Platynini Bonelli, 1810

= Anchomenini Bonelli, 1810

= Sericodini W. Kirby, 1837

= Agonumini W. Kirby, 1837

= Colpodini Chaudoir, 1872 ^H^

= Agelaeini Jakobson, 1907

= Prosphodrini J.M. Valentine, 1987

= Meleagrosini Morvan, 2004

Tribe Pseudomorphini Hope, 1838

Tribe Pterostichini Bonelli, 1810

Subtribe Abacomorphina Tschitschérine, 1902

= Sphodrosomina Tschitschérine, 1902

Subtribe Euchroina Chaudoir, 1874

Subtribe Metiina Straneo, 1951

= Antarctiina Lacordaire, 1854 [PI]

= Kuschelinina Jeannel, 1967

Subtribe Microcephalina Tschitschérine, 1898

= Tichoniina Semenov, 1904 [PI]

= Tichonillina Emden, 1958

Subtribe Pterostichina Bonelli, 1810

= Molopina Bonelli, 1810

= Poecilina Bonelli, 1810

= Feroniina Dejean, 1825

= Trigonotomina Laporte, 1834

= Catadromina Brullé, 1834

= Thaliina Hope, 1838 [PI]

= Stomidina Gené, 1839

= Rathymina H.W. Bates, 1891

= Trigonognathina Tschitschérine, 1898

= Platysmatina Tschitschérine, 1899

= Cyphosomatina Tschitschérine, 1902 [PI]

= Cyrtoderina Tschitschérine, 1902

= Darodiliina Tschitschérine, 1902

= Deliniina Tschitschérine, 1902

= Myadina Jakobson, 1907

= Cratogastrina Csiki, 1929

= Abacina Schuler, 1970

= Aristochroodina Sciaky, 1996

Tribe Sphodrini Laporte, 1834

Subtribe Atranopsina Baehr, 1982

Subtribe Calathina Laporte, 1834

Subtribe Dolichina Brullé, 1834

Subtribe Pristosiina Lindroth, 1956

Subtribe Sphodrina Laporte, 1834

= Pristonychina Gistel, 1848

Subtribe Synuchina Lindroth, 1956

Tribe Xenaroswellianini Erwin, 2007

Tribe Zabrini Bonelli, 1810

Subtribe Amarina C. Zimmermann, 1832

= Isopleurina W. Kirby, 1837

= Agronomina Gistel, 1848 [PI]

= Pangetina Gistel, 1856

Subtribe Zabrina Bonelli, 1810

Tribe Zuphiini Bonelli, 1810

Subtribe Dicrodontina Machado, 1992

Subtribe Leleupidiina Basilewsky, 1951

Subtribe Metazuphiina Mateu, 1992

Subtribe Mischocephalina Mateu, 1992

Subtribe Patriziina Basilewsky, 1953

Subtribe Zuphiina Bonelli, 1810

= Polistichina H.W. Bates, 1886

**SUBORDER ARCHOSTEMATA**

**Superfamily Cupedoidea Laporte, 1838**

**Family Crowsoniellidae Iablokoff-Khnzorian, 1983**

**Family Cupedidae Laporte, 1838**

Subfamily Priacminae Crowson, 1962

Subfamily Mesocupedinae Ponomarenko, 1969†

Subfamily Cupedinae Laporte, 1838

**Family Micromalthidae Barber, 1913**

**Family Ommatidae Sharp & Muir, 1912**

Subfamily Brochocoleinae Hong, 1982†

Subfamily Ommatinae Sharp & Muir, 1912

Subfamily Tetraphalerinae Crowson, 1962

**Ommatidae incertae sedis**

ClessidrommatiniJarzembowski et al., 2018† ^20^

**Family Triadocupedidae Ponomarenko, 1966**†

**Cupedoidea incertae sedis**

**Magnocoleidae Hong, 1998**†

**Superfamily Asiocoleoidea Rohdendorf, 1961**†

**Family Asiocoleidae Rohdendorf, 1961**†

**Family Tricoleidae Ponomarenko, 1969**†

**Superfamily Rhombocoleoidea Rohdendorf, 1961**†

**Family Rhombocoleidae Rohdendorf, 1961**†

**Superfamily Schizocoleoidea Rohdendorf, 1961**†

**Family Schizocoleidae Rohdendorf, 1961**†

= Curculiopseidae Martynov, 1937† [PI]

**Family Phoroschizidae Bouchard & Bousquet, 2020**†

= Schizophoridae Ponomarenko, 1968† [PI]

**Family Catiniidae Ponomarenko, 1968**† ^H^

**ARCHOSTEMATA incertae sedis ^21^**

Lithocupedini Ponomarenko, 1969†

Notocupedini Ponomarenko, 1966†

**Obrieniidae Zherikhin & Gratshev, 1994**†

Kararhynchinae Zherikhin & Gratshev, 1994†

Kararhynchini Zherikhin & Gratshev, 1994†

Kenderlykanini Legalov, 2009†

Obrieniinae Zherikhin & Gratshev, 1994†

Pseudobrieniinae Legalov, 2012†

**SUBORDER MYXOPHAGA**

**Superfamily Lepiceroidea Hinton, 1936 (1882)**

**Family Lepiceridae Hinton, 1936 (1882)**

= Cyathoceridae Sharp, 1882

= Haplochelidae Kirejtshuk & Poinar, 2006†

**Superfamily Sphaeriusoidea Erichson, 1845**

**Family Torridincolidae Steffan, 1964**

Subfamily Torridincolinae Steffan, 1964

= Ptyopteryginae M. Abdullah, 1974 [PI]

Subfamily Deleveinae Endrödy-Younga, 1997

**Family Hydroscaphidae J.L. LeConte, 1874**

Subfamily Hydroscaphinae J.L. LeConte, 1874

Subfamily Leehermaniinae Kirejtshuk et al. 2023†

Subfamily TriamyxinaeQvarnström et al., 2021† ^22^

**Family Sphaeriusidae Erichson, 1845**

= Microsporidae Crotch, 1873

**SUBORDER POLYPHAGA**

**SERIES SCIRTIFORMIA**

**Superfamily Scirtoidea Fleming, 1821**

**Family DecliniidaeNikitsky et al., 1994**

**Family Scirtidae Fleming, 1821**

= Sinodryopitidae Hong, 2002†

Subfamily Mesernobiinae Engel, 2010†

Subfamily Nipponocyphoninae Lawrence & Yoshitomi, 2007

Subfamily Stenocyphoninae Lawrence & Yoshitomi, 2007

Subfamily Scirtinae Fleming, 1821

= Cyphoninae Stephens, 1829

= Elodinae Shuckard, 1839

= Atopidinae Pic, 1914

**Family Elodophthalmidae Kirejtshuk & Azar, 2008**†

**Family Boleopsidae Kirejtshuk & Nel, 2013**†

**SERIES CLAMBIFORMIA**

**Superfamily Clamboidea Fischer von Waldheim, 1821**

**Family Derodontidae J.L. LeConte, 1861**

Subfamily Peltasticinae J.L. LeConte, 1861

Subfamily Derodontinae J.L. LeConte, 1861

Subfamily Laricobiinae Mulsant & Rey, 1864

**Family Clambidae Fischer von Waldheim, 1821**

= Ptismidae Kirejtshuk & Azar, 2016† ^23^

Subfamily Calyptomerinae Crowson, 1955

Subfamily Acalyptomerinae Crowson, 1979

Subfamily Clambinae Fischer von Waldheim, 1821

**Family Eucinetidae Lacordaire, 1857**

= Cryptomeridae Broun, 1893 [PI]

**Family Mesocinetidae Kirejtshuk & Ponomarenko, 2010**†

**SERIES RHINORHIPIFORMIA**

**Superfamily Rhinorhipoidea Lawrence, 1988**

**Family Rhinorhipidae Lawrence, 1988**

**SERIES ELATERIFORMIA**

**Superfamily Dascilloidea Guérin-Méneville, 1843 (1834)**

**Family Dascillidae Guérin-Méneville, 1843 (1834)**

Subfamily Dascillinae Guérin-Méneville, 1843 (1834)

Tribe Cinnabariini Pic, 1914

Tribe Dascillini Guérin-Méneville, 1843 (1834)

= Atopini Laporte, 1834

Subfamily Karumiinae Escalera, 1913

Tribe Emmitini Escalera, 1914

= Emmini Escalera, 1913 [PI]

Tribe Escalerinini Paulus, 1972

Tribe Genecerini Pic, 1914

Tribe Karumiini Escalera, 1913

= Zarudniolini Semenov-Tjan-Shansky & Martynov, 1925

**Family Rhipiceridae Latreille, 1834**

Subfamily Rhipicerinae Latreille, 1834

Subfamily Sandalinae Jakobson, 1913

**Superfamily Byrrhoidea Latreille, 1804**

**Family Byrrhidae Latreille, 1804**

Subfamily Byrrhinae Latreille, 1804

Tribe Byrrhini Latreille, 1804

Tribe Exomellini Casey, 1914

Tribe Morychini El Moursy, 1961

Tribe Pedilophorini Casey, 1912

Tribe Simplocariini Mulsant & Rey, 1869

= Lioonini Casey, 1912

Subfamily Syncalyptinae Mulsant & Rey, 1869

Tribe Microchaetini Paulus, 1973 ^H^

Tribe Syncalyptini Mulsant & Rey, 1869

Subfamily Amphicyrtinae J.L. LeConte, 1861

Subfamily Lidryopinae Kirejtshuk & Nel, 2016†

**Superfamily Buprestoidea Leach, 1815**

**Family Schizopodidae J.L. LeConte, 1859**

Tribe Dystaxiini Théry, 1929

= Glyptoscelimorphini Cobos, 1963

Tribe Electrapatini Iablokoff-Khnzorian, 1962†

Tribe Schizopodini J.L. LeConte, 1859

**Family Buprestidae Leach, 1815**

Subfamily Julodinae Lacordaire, 1857

= Sternocerinae Csiki, 1904

= Amblysterninae Cobos, 1955

Subfamily Polycestinae Lacordaire, 1857

Tribe Acmaeoderini Kerremans, 1893

Subtribe Acmaeoderina Kerremans, 1893

Subtribe Acmaeoderoidina Cobos, 1955

Subtribe Nothomorphina Cobos, 1955

Tribe Astraeini Cobos, 1980

Tribe Bulini Bellamy, 1995

Tribe Haplostethini J.L. LeConte, 1861

= Mastogeniini J.L. LeConte & G.H. Horn, 1883

Tribe Paratracheini Cobos, 1980

Tribe Perucolini Cobos, 1980

Tribe Polycestini Lacordaire, 1857

Subtribe Polycestina Lacordaire, 1857

Subtribe Xenopsina Volkovitsh, 2008

Tribe Polyctesini Cobos, 1955

Tribe Prospherini Cobos, 1980

Tribe Ptosimini Kerremans, 1903

Tribe Thrincopygini J.L. LeConte, 1861

Tribe Tyndaridini Cobos, 1955

Subtribe Mimicoclytrinina Bellamy, 2003

= Acherusiina Cobos, 1955 [PI]

Subtribe Pseudacherusiina Cobos, 1980

Subtribe Tylaucheniina Cobos, 1959

Subtribe Tyndaridina Cobos, 1955

Tribe Xyroscelidini Cobos, 1955

Subfamily Galbellinae Reitter, 1911

Subfamily Chrysochroinae Laporte, 1835

Tribe Chrysochroini Laporte, 1835

Subtribe Chalcophorina Lacordaire, 1857 (1848)

= Anaglyptina Gistel, 1848 [NO]

= Chrysodemina Kerremans, 1892

= Chalcophorellina Tôyama, 1986

= Iridotaeniina Tôyama, 1987

= Lampropeplina Holyński, 1993

Subtribe Chrysochroina Laporte, 1835

= Catoxanthina Jakobson, 1913 [PI]

Subtribe Eucallopistina Bellamy, 2003

= Callopistina Kurosawa, 1990 [PI]

Tribe Dicercini Gistel, 1848

Subtribe Dicercina Gistel, 1848

= Polybothridina Gistel, 1848

= Psilopterina Lacordaire, 1857 ^H^

= Capnodina Csiki, 1909

Subtribe Haplotrinchina Holyński, 1993

Subtribe Hippomelanina Holyński, 1993

Subtribe Pseudoperotina Tôyama, 1987

Tribe Evidini Tôyama, 1987

Tribe Paraleptodemini Cobos, 1975

Subtribe Euchromatina Holyński, 1993

Subtribe Euplectaleciina Holyński, 1993

Subtribe Hypoprasina Holyński, 1993

Subtribe Paraleptodemina Cobos, 1975

= Cinyrina Cobos, 1979

Subtribe Pristipterina Holyński, 1993

Tribe Paratassini Bílý & Volkovitsh, 1996

Tribe Poecilonotini Jakobson, 1913

Subtribe Nesotrinchina Bílý et al., 2009

Subtribe Poecilonotina Jakobson, 1913

Tribe Sphenopterini Lacordaire, 1857

Tribe Vadonaxiini Descarpentries, 1970

Subfamily Buprestinae Leach, 1815

Tribe Actenodini Gistel, 1848

= Belionotini Jakobson, 1913

Tribe Anthaxiini Gory & Laporte, 1839

Tribe Bubastini Obenberger, 1920

Tribe Buprestini Leach, 1815

Subtribe Agaeocerina Nelson, 1982

Subtribe Buprestina Leach, 1815

= Ancylocheirina Jakobson, 1913

Subtribe Lamprocheilina Holyński, 1993

Subtribe Trachykelina Holyński, 1988

Tribe Chrysobothrini Gory & Laporte, 1836

Tribe Coomaniellini Bílý, 1974

Tribe Curidini Holyński, 1988

Subtribe Anilarina Bílý, 2000

Subtribe Curidina Holyński, 1988

Subtribe Neocuridina Holyński, 1988

Tribe Epistomentini Levey, 1978

Tribe Exagistini Tôyama, 1987

Tribe Glaphyropterini Pongrácz, 1935†

Tribe Julodimorphini Kerremans, 1903

Tribe Kisanthobiini Richter, 1949

Tribe Maoraxiini Holyński, 1984

Tribe Melanophilini Bedel, 1921

Tribe Melobasini Bílý, 2000

Tribe Mendizabaliini Cobos, 1968

Tribe Nascionini Holyński, 1988

Tribe Phrixiini Cobos, 1975

Tribe Pterobothrini Volkovitsh, 2001

Tribe Stigmoderini Lacordaire, 1857

Tribe Thomassetiini Bellamy, 1987

Subtribe Philanthaxiina Holyński, 1988

Subtribe Thomassetiina Bellamy, 1987

Tribe Trigonogeniini Cobos, 1956

Tribe Xenorhipidini Cobos, 1986

Subtribe Trichinorhipidina Bellamy, 2006

Subtribe Xenorhipidina Cobos, 1986

Subfamily Agrilinae Laporte, 1835

Tribe Agrilini Laporte, 1835

Subtribe Agrilina Laporte, 1835

Subtribe Amorphosternina Cobos, 1974

Subtribe Amyiina Holyński, 1993

Subtribe Rhaeboscelidina Cobos, 1976

Tribe Aphanisticini Jacquelin du Val, 1859

Subtribe Anthaxomorphina Holyński, 1993

Subtribe Aphanisticina Jacquelin du Val, 1859

Subtribe Cylindromorphina Portevin, 1931

Subtribe Cylindromorphoidina Cobos, 1979

Subtribe Germaricina Cobos, 1979

Tribe Coraebini Bedel, 1921

Subtribe Amorphosomina Majer, 2000

Subtribe Cisseina Majer, 2000

Subtribe Clemina Majer, 2000

Subtribe Coraebina Bedel, 1921

Subtribe Dismorphina Cobos, 1990

Subtribe Ethoniina Majer, 2001

Subtribe Geraliina Cobos, 1988

Subtribe Meliboeina Majer, 2000

Subtribe Synechocerina Majer, 2000

Subtribe Toxoscelina Majer, 2000

Tribe Tracheini Laporte, 1835

Subtribe Brachina J.L. LeConte, 1861

Subtribe Leiopleurina Holyński, 1993

Subtribe Pachyschelina Böving & Craighead, 1931

Subtribe Tracheina Laporte, 1835

= Phytoterina Gistel, 1856

Subfamily Parathyreinae Alexeev, 1994†

**Superfamily Dryopoidea Billberg, 1820 (1817)**

**Family MastigocoleidaeTihelka et al., 2022**†

**Family Lutrochidae Kasap & Crowson, 1975**

**Family Dryopidae Billberg, 1820 (1817)**

= Parnidae Leach, 1817

= Pelonomidae Böving & Craighead, 1931

= Chiloeidae Dajoz, 1973

**Family Eulichadidae Crowson, 1973**

= Lichadidae Csiki, 1902 [PI]

**Family Callirhipidae Emden, 1924**

= Zenoidae J.L. LeConte, 1866 [PI]

**Family Ptilodactylidae Laporte, 1838**

Subfamily Cladotominae Pic, 1914

Subfamily Anchytarsinae Champion, 1897 ^P^

= Coloboderinae Erichson, 1847

Subfamily Aploglossinae Champion, 1897

Subfamily Araeopidiinae Lawrence, 1991

Subfamily Ptilodactylinae Laporte, 1838

= Podabrocephalinae Pic, 1930 ^24^

**Family Cneoglossidae Champion, 1897**

**Family Chelonariidae Blanchard, 1845**

**Family Psephenidae Lacordaire, 1854**

Subfamily Afroeubriinae Lee et al., 2007

Subfamily Eubriinae Lacordaire, 1857

Subfamily Psephenoidinae Bollow, 1938

Subfamily Eubrianacinae Jakobson, 1913 (1880)

= Placonychinae G.H. Horn, 1880

Subfamily Psepheninae Lacordaire, 1854

= Eurypalpinae J.L. LeConte, 1852 [PI]

**Family Protelmidae Jeannel, 1950 ^25^**

**Family Elmidae Curtis, 1830**

Subfamily Larainae J.L. LeConte, 1861

Tribe Laraini J.L. LeConte, 1861

Tribe Potamophilini Fournel & Géhin, 1845

Subfamily Elminae Curtis, 1830

Tribe Ancyronychini Ganglbauer, 1904

Tribe Elmini Curtis, 1830

Subtribe Elmina Curtis, 1830

= Limniina C.G. Thomson, 1859

Subtribe Stenelmina Mulsant & Rey, 1872

Tribe Macronychini Gistel, 1848

**Family Limnichidae Erichson, 1846**

Subfamily Hyphalinae Britton, 1971

Subfamily Limnichinae Erichson, 1846

Tribe Limnichini Erichson, 1846

= Bothriophorini Mulsant & Rey, 1869

Tribe Wooldridgeini Spangler, 1999

Subfamily Cephalobyrrhinae Champion, 1925

Subfamily Thaumastodinae Champion, 1924 ^P^

= Pseudeucinetinae Csiki, 1924

**Family Heteroceridae W.S. MacLeay, 1825**

Subfamily Elythomerinae Pacheco, 1964

Subfamily Heterocerinae W.S. MacLeay, 1825

Tribe Augylini Pacheco, 1964

Tribe Haraiaini García & Jiménez-Ramos, 2020

Tribe Heterocerini W.S. MacLeay, 1825

Tribe Micilini Pacheco, 1964

Tribe Tropicini Pacheco, 1964

**Superfamily Elateroidea Leach, 1815**

**Family Artematopodidae Lacordaire, 1857**

Subfamily Electribiinae Crowson, 1975

Subfamily Allopogoniinae Crowson, 1973

Subfamily Artematopodinae Lacordaire, 1857

Tribe Artematopodini Lacordaire, 1857

Tribe Ctesibiini Crowson, 1973

Tribe Macropogonini J.L. LeConte, 1861

= Eurypogonini Böving & Craighead, 1931

**Family Omethidae J.L. LeConte, 1861**

Subfamily Driloniinae Crowson, 1972

Subfamily Matheteinae J.L. LeConte, 1881

Subfamily Omethinae J.L. LeConte, 1861

Subfamily Telegeusinae Leng, 1920

**Family Brachypsectridae G.H. Horn, 1881**

**Family Throscidae Laporte, 1840** [NP]

= Stereolidae Rafinesque, 1815 [NO]

= Trixagidae Gistel, 1848

= Potergidae Cobos, 1961

= Palaeothroscidae Iablokoff-Khnzorian, 1962†

**Family Eucnemidae Eschscholtz, 1829**

Subfamily Perothopinae Lacordaire, 1857

Subfamily Phyllocerinae Reitter, 1905

Tribe Anelastini Reitter, 1911

Tribe Phyllocerini Reitter, 1905

Subfamily Pseudomeninae Muona, 1993

Subfamily Schizophilinae Muona, 1993 ^26^

Subfamily Palaeoxeninae Muona, 1993

Subfamily Phlegoninae Muona, 1993

Subfamily Anischiinae Fleutiaux, 1936

Subfamily Melasinae Fleming, 1821

Tribe Calyptocerini Muona, 1993

Tribe Ceballosmelasini Muona, 1993

Tribe Dirhagini Reitter, 1911

= Microrhagini Fleutiaux, 1919

= Arrhipidini Cobos, 1965 [PI]

Tribe Epiphanini Muona, 1993 ^H^

Tribe Hylocharini Jacquelin du Val, 1859

Tribe Melasini Fleming, 1821

Subtribe Compsocnemina Muona, 1993

Subtribe Melasina Fleming, 1821

Tribe Neocharini Muona, 1993

Tribe Xylobiini Reitter, 1911

Subfamily Eucneminae Eschscholtz, 1829

Tribe Dendrocharini Fleutiaux, 1920

Tribe Dyscharachthini Muona, 1993

Tribe Entomosatopini Muona, 1993

Tribe Eucnemini Eschscholtz, 1829

= Gastraulacini Fleutiaux, 1902

Tribe Galbitini Muona, 1991

= Galbini Beaulieu, 1919 [PI]

Tribe Mesogenini Muona, 1993

Tribe Muonajini Özdikmen, 2008

= Yangini Muona, 1993 [PI]

Tribe Perrotiini Muona, 1993

Tribe Phaenocerini Muona, 1993

Tribe Proutianini Muona, 1993

Subfamily Macraulacinae Fleutiaux, 1923 ^P^

Tribe Anelastidini Muona, 1993

Tribe Echthrogasterini Cobos, 1965

Tribe Euryptychini Mamaev, 1976

Tribe Macraulacini Fleutiaux, 1923 ^P^

= Dromaeolini Beaulieu, 1919

= Fornacini Beaulieu, 1919

Tribe Jenibuntorini Muona, 1993

Tribe Nematodini Leiler, 1976

Tribe Oisocerini Muona, 1993

Tribe Orodotini Muona, 1993

= Cryptostomatini Laporte, 1835 [PI]

Tribe Throscogeniini Iablokoff-Khnzorian, 1962†

**Family Cerophytidae Latreille, 1834 ^27^**

= Praelateriidae Dolin, 1973†

= Aphytocerinae Kirejtshuk, 2013†

**Family Jurasaidae Rosa et al., 2020**

**Family Elateridae Leach, 1815 ^28^**

Subfamily Elaterinae Leach, 1815

Tribe Agriotini Laporte, 1840

Subtribe Agriotina Laporte, 1840

Subtribe Cardiorhinina Candèze, 1863

Tribe Ampedini Gistel, 1848

Tribe Anisomerini Girard, 2019 ^H^

Tribe Cebrionini Latreille, 1802 ^29^

Subtribe Aplastina Stibick, 1979

Subtribe Cebrionina Latreille, 1802

Tribe Dicrepidiini J. Thomson, 1858

Tribe Elaterini Leach, 1815

= Arneini Gistel, 1848

= Steatoderini Gistel, 1848

= Amphilabrini Gistel, 1856

= Ludiini Lacordaire, 1857 ^H^

= Hypodeseini Candèze, 1863

= Sericosomini Hyslop, 1917

Tribe Eudicronychini Girard, 1971 ^30^

Tribe Megapenthini Gurjeva, 1973

Tribe Melanotini Candèze, 1859 (1848)

= Cratonychini Gistel, 1848

Tribe Odontonychini Girard, 1973

Tribe Physorhinini Candèze, 1859

Tribe Pomachiliini Candèze, 1859

Tribe Synaptini Gistel, 1856

= Dairini Gistel, 1848 [PI]

= Adrastini Candèze, 1863

Subfamily Tetralobinae Laporte, 1840

Tribe Piezophyllini Laurent, 1967

Tribe Tetralobini Laporte, 1840

= Phyllophorini Hope, 1842 [PI]

Subfamily Omalisinae Lacordaire, 1857 ^31^

= Thilmaninae Kazantsev, 2004

= Euanominae Kazantsev, 2010

= ParadrilinaeKundrata et al., 2015

Subfamily Agrypninae Candèze, 1857 [NP] ^32^

Tribe Agrypnini Candèze, 1857 [NP]

= Adelocerini Gistel, 1848 [NO]

= Pangaurini Gistel, 1856 [NO]

= Octocryptini Schwarz, 1906

= Cavicoxini Pic, 1928

= Laconini Dajoz, 1964

Tribe Anaissini Golbach, 1984

= Alampini C. Costa, 1975 [PI]

Tribe Cryptocardiini Dolin, 1980†

Tribe Drilini Blanchard, 1845

Tribe Euplinthini C. Costa, 1975

Subtribe Cleidecostina P.J. Johnson, 2002

= Heligmina C. Costa, 1975 [PI]

Subtribe Compsoplinthina C. Costa, 1975

Subtribe Euplinthina C. Costa, 1975

Tribe Hemirhipini Candèze, 1857

Subtribe Hemirhipina Candèze, 1857

= Chalcolepidiina Candèze, 1857

= Alaina Candèze, 1874

Subtribe Ludioctenina Jakobson, 1913

= Tetrigusina Schimmel & Tarnawski, 2012

Tribe Oophorini Gistel, 1848

= Monocrepidiini Candèze, 1859

= Drasteriini Houlbert, 1912 ^H^

= Conoderini Fleutiaux, 1919 ^H^

= Pachyderini Fleutiaux, 1919

Tribe Platycrepidiini C. Costa & Casari-Chen, 1993

= Eudactylini Candèze, 1859 [PI]

Tribe Pseudomelanactini Arnett, 1967

Tribe Pyrophorini Candèze, 1863

Subtribe Hapsodrilina C. Costa, 1975

Subtribe Nyctophyxina C. Costa, 1975

Subtribe Pyrophorina Candèze, 1863

Subfamily HapatesinaeKusy et al., 2021

Subfamily Pityobiinae Hyslop, 1917 ^33^

Tribe Campyloxenini C. Costa, 1975

Tribe ParablaciniKundrata et al., 2015

Tribe Pityobiini Hyslop, 1917

Tribe *Tibionemini Motyka et al. 2023

Subfamily Lissominae Laporte, 1835

Tribe Lissomini Laporte, 1835

= Drapetini J.L. LeConte, 1863

Tribe Protelaterini Schwarz, 1902

= Allotriini Candèze, 1863 [PI]

= Senodoniini Schenkling, 1927 ^34^

= Rostricephalini Fleutiaux, 1947

= Sphaenelaterini Stibick, 1979

Subfamily Thylacosterninae Fleutiaux, 1920

= Soleniscinae Lameere, 1900 [PI]

= Pterotarsinae Fleutiaux, 1902

= Balginae Fleutiaux, 1926

= Cussoleninae Cobos, 1961

Subfamily Hypnoidinae Schwarz, 1906 (1860) ^35^

= Cryptohypninae Candèze, 1860

= Hypolithinae Fleutiaux, 1928

= Prisahypninae Stibick, 1979

Subfamily Dendrometrinae Gistel, 1848

Tribe Crepidomenini Candèze, 1863

Tribe Dendrometrini Gistel, 1848

Subtribe Dendrometrina Gistel, 1848

= Athoina Candèze, 1859

= Limoniina Jakobson, 1913 ^H^

Subtribe Denticollina Stein & Weise, 1877 (1848)

= Campylina Gistel, 1848

= Lepturoidina Schwarz, 1906

Subtribe Hemicrepidiina Champion, 1896

= Asaphina Candèze, 1863 [PI] ^36^

Tribe Dimini Candèze, 1863

= Peniini Dolin, 1990

Tribe Oxynopterini Candèze, 1857

= Melanactini Candèze, 1857

= Campsosternini Fleutiaux, 1927

= Pectocerini Gurjeva, 1974

Tribe Plastocerini Crowson, 1972 ^37^

Tribe Pleonomini Semenov-Tjan-Shansky & Pjatakova, 1936

Tribe Prosternini Gistel, 1856 [NP]

= Diacanthini Gistel, 1848 [NO]

= Corymbitini J.L. LeConte, 1861

= Ctenicerini Jakobson, 1913

Tribe SelatosominiSchimmel et al., 2015

Subtribe MosotalesinaSchimmel et al., 2015

Subtribe SelatosominaSchimmel et al., 2015

Tribe Semiotini Jakobson, 1913

Subfamily Negastriinae Nakane & Kishii, 1956

Tribe Negastriini Nakane & Kishii, 1956

Tribe Quasimusini Schimmel & Tarnawski, 2009

Subtribe Loebliquasina Schimmel & Tarnawski, 2009

Subtribe Quasimusina Schimmel & Tarnawski, 2009

Subtribe Striatoquasina Schimmel & Tarnawski, 2009

Subtribe Wittmeroquasina Schimmel & Tarnawski, 2009

Subfamily Cardiophorinae Candèze, 1859

= Aphricinae J.L. LeConte, 1861

= Aptopodinae Jakobson, 1913

= Esthesopodinae Fleutiaux, 1919

= Nyctorinae Semenov-Tjan-Shansky & Pjatakova, 1936

= Cebriognathinae Paulus, 1981 ^38^

Subfamily Oestodinae Hyslop, 1917

Subfamily Hemiopinae Fleutiaux, 1941

Tribe Hemiopini Fleutiaux, 1941

Tribe PaulusielliniKusy et al., 2023

Subfamily Physodactylinae Lacordaire, 1857

= Toxognathinae Fleutiaux, 1941

Subfamily Subprotelaterinae Fleutiaux, 1920

Subfamily Morostominae Dolin, 2000

Subfamily Protagrypninae Dolin, 1973†

Tribe Desmatini Dolin, 1975†

Tribe Hypnomorphini Dolin, 1975†

Tribe Pollostelaterini Alexeev, 2011†

Tribe Protagrypnini Dolin, 1973†

**Elateridae incertae sedis** ^39^

Adzusini Kishii, 1989

Athoomorphinae Laurent, 1966

Beliophorina Jakobson, 1913

**Family Sinopyrophoridae Bi & X.-Y. Li, 2019 ^40^**

**Family Lycidae Laporte, 1838** ^41^

Subfamily Dexorinae Bocák & Bocáková, 1989

Tribe Cretolycini Tihelka et al., 2019†

Tribe Dexorini Bocák & Bocáková, 1989

Subtribe BurmolycinaBocák et al., 2019†

Subtribe Dexorina Bocák & Bocáková, 1989

Tribe *Mimolibnetini Kazantsev, 2013 ^42^

Subtribe Lampyrolycina Kazantsev, 2018

Subtribe *Mimolibnetina Kazantsev, 2013

Subfamily Calochrominae Lacordaire, 1857

= Lygistopterinae J.L. LeConte, 1881

Subfamily Erotinae J.L. LeConte, 1881

Tribe Dictyopterini Houlbert, 1922

Tribe Erotini J.L. LeConte, 1881

= Lopherotini Kazantsev, 2012

= Pseudaplatopterini Kazantsev, 2012

Tribe Proterotaphini Kazantsev, 2012

Tribe Slipinskiini Bocák & Bocáková, 1992

= Aferotini Kazantsev, 2004

= Flagraxini Kazantsev, 2004

Tribe Taphini Bocák & Bocáková, 1990

Subfamily Ateliinae Kleine, 1929

Tribe Ateliini Kleine, 1929

Tribe Lyponiini Bocák & Bocáková, 1990

Tribe Macrolycini Kleine, 1929

Subfamily Lycinae Laporte, 1838

Tribe Calopterini Green, 1949

Subtribe Acroleptina Bocáková, 2005

Subtribe Calopterina Green, 1949

Tribe Conderini Bocák & Bocáková, 1990

Tribe Eurrhacini Bocáková, 2005

Tribe Leptolycini Leng & Mutchler, 1922

Subtribe Dominopterina Kazantsev, 2013

Subtribe Electropterina Kazantsev, 2013†

Subtribe Leptolycina Leng & Mutchler, 1922

Tribe Lycini Laporte, 1838

Tribe Platerodini Kleine, 1929

Tribe Thonalmini Kleine, 1933

Subfamily Lyropaeinae Bocák & Bocáková, 1989

Tribe Alyculini Bocák & Bocáková, 2008

Tribe Antennolycini Bocák & Bocáková, 2008

Tribe Lyropaeini Bocák & Bocáková, 1989

= Paralycini L.N. Medvedev & Kazantsev, 1992 [PI]

Tribe Miniduliticolini Kazantsev, 2003

Subfamily Platerodrilinae

Subfamily Metriorrhynchinae Kleine, 1926

Tribe Dihammatini Bocák & Bocáková, 2008

Tribe Dilophotini Kleine, 1929

Tribe Libnetini Bocák & Bocáková, 1990

Tribe Lycoprogenthini Bocák & Bocáková, 2008

Tribe Metriorrhynchini Kleine, 1926

Subtribe Cautirina Sklenarova et al., 2014

Subtribe MetanoeinaSklenarova et al., 2014

Subtribe Metriorrhynchina Kleine, 1926

= Dilolycina Kleine, 1926

= Cladophorina Kleine, 1929

= Trichalina Kleine, 1929

= Hemiconderinina Bocák & Bocáková, 1990

**Lycidae incertae sedis**

Macrolycinellini Kazantsev, 2012

Melanerotini Kazantsev, 2010

Vikhrevini Kazantsev, 2013

**Family IberobaeniidaeBocák et al., 2016**

**Family Berendtimiridae Winkler, 1987**†

Subfamily Berendtimirinae Winkler, 1987†

Subfamily RetromalisinaeFanti et al., 2023†

**Family Cretophengodidae Y.-D. Li et al., 2021**†

**Family Phengodidae J.L. LeConte, 1861**

Subfamily Cydistinae Paulus, 1972 ^43^

Subfamily Phengodinae J.L. LeConte, 1861

= Pseudophengodinae Pic, 1930

Subfamily Mastinocerinae J.L. LeConte, 1881

Subfamily Penicillophorinae Paulus, 1975

**Family Rhagophthalmidae E. Olivier, 1907**

**Family Lampyridae Rafinesque, 1815** ^44^

Subfamily Luciolinae Lacordaire, 1857

Tribe Curtosini McDermott, 1964

Tribe Luciolini Lacordaire, 1857

Subfamily Pterotinae J.L. LeConte, 1861

Subfamily Ototretinae McDermott, 1964

= Ototretadrilinae Crowson, 1972

Subfamily Lamprohizinae Kazantsev, 2010

Subfamily ChespiritoinaeFerreira et al., 2020

Subfamily Cyphonocerinae Crowson, 1972

Subfamily Psilocladinae McDermott, 1964

Subfamily Amydetinae E. Olivier, 1907

= Megalophthalminae E. Olivier, 1907 [PI]

Subfamily Cheguevariinae Kazantsev, 2007

Subfamily Photurinae Lacordaire, 1857

Subfamily Cladodinae Bocákova et al., 2022 ^H?^

Subfamily Lampyrinae Rafinesque, 1815

Tribe Cratomorphini E. Olivier, 1911

Tribe Lamprocerini E. Olivier, 1907

Tribe Lampyrini Rafinesque, 1815

Tribe Lucidotini Lacordaire, 1857

Subtribe Dadophorina E. Olivier, 1907

Subtribe Lucidotina Lacordaire, 1857

= Pristolycina J. R. Winkler, 1953

Subtribe Photinina J.L. LeConte, 1881

= Phosphaenina E. Olivier, 1884

Tribe Pleotomini Summers, 1874

= Calyptocephalini Jakobson, 1911

**Lampyridae incertae sedis**

Lamprigerina McDermott, 1964

Vestini McDermott, 1964

**Family Mysteriomorphidae Alekseev & Ellenberger, 2019**†

**Family Cantharidae Imhoff, 1856 (1815)** ^45^

Subfamily Chauliognathinae J.L. LeConte, 1861

Tribe Chauliognathini J.L. LeConte, 1861

Tribe Ichthyurini Champion, 1915 ^H^

Subfamily Malthininae Kiesenwetter, 1852

Tribe Malchinini Brancucci, 1980

Tribe Malthinini Kiesenwetter, 1852

Tribe Malthodini Böving & Craighead, 1931

Tribe Mimoplatycini Kazantsev, 2013†

Tribe Nothotytthonychini Fanti, 2022†

Tribe Tytthonyxini Arnett, 1962

Subfamily Dysmorphocerinae Brancucci, 1980

Subfamily Heteromastiginae Motyka et al., 2023

Subfamily Silinae Mulsant, 1862

Tribe Silini Mulsant, 1862

Subfamily Cantharinae Imhoff, 1856 (1815)

Tribe Cacomorphocerini Fanti & Kupryjanowicz, 2018†

Tribe Cantharini Imhoff, 1856 (1815)

= Telephorini Leach, 1815

Tribe Podabrini Gistel, 1856

**SERIES NOSODENDRIFORMIA**

**Superfamily Nosodendroidea Erichson, 1846**

**Family Nosodendridae Erichson, 1846**

SERIES STAPHYLINIFORMIA

**Superfamily Histeroidea Gyllenhal, 1808**

**Family Synteliidae Lewis, 1882**

**Family Sphaeritidae Shuckard, 1839**

**Family CretohisteridaeZhou et al., 2018**†

**Family Histeridae Gyllenhal, 1808**

Subfamily AntigracilinaeZhou et al., 2020†

Subfamily Niponiinae Fowler, 1912

Subfamily Abraeinae W.S. MacLeay, 1819

Tribe Abraeini W.S. MacLeay, 1819

Tribe Acritini Wenzel, 1944

Tribe Acritomorphini Wenzel, 1944

Tribe PantostictiniZhou et al., 2020†

Tribe Plegaderini Portevin, 1929

Tribe Teretriini Bickhardt, 1914

Tribe Trypanaeini Marseul, 1857 ^46^

Tribe Trypeticini Bickhardt, 1913 ^47^

Subfamily Saprininae Blanchard, 1845 ^48^

Tribe Eremosaprinini Lackner, 2024

Tribe Euspilotini Lackner, 2024

Tribe Hypocaccini Lackner, 2024

Tribe Myrmetini Portevin, 1929

Tribe Saprinini Blanchard, 1845

Subfamily Dendrophilinae Reitter, 1909

Tribe Anapleini Olexa, 1982

Tribe Bacaniini Kryzhanovskij, 1976

Tribe Dendrophilini Reitter, 1909

Tribe Paromalini Reitter, 1909

Subfamily Onthophilinae W.S. MacLeay, 1819

Subfamily Tribalinae Bickhardt, 1914

Subfamily Histerinae Gyllenhal, 1808

Tribe Exosternini Bickhardt, 1914

Tribe Histerini Gyllenhal, 1808

Tribe Hololeptini Hope, 1841

Tribe Omalodini Kryzhanovskij, 1972

Tribe Platysomatini Bickhardt, 1914

= Althanini Cooman, 1939

Subfamily Haeteriinae Marseul, 1857

Tribe Haeteriini Marseul, 1857

Tribe Nymphisterini Tishechkin, 2007

Tribe Synoditulini Tishechkin, 2007

Subfamily Chlamydopsinae Bickhardt, 1914

**Superfamily Hydrophiloidea Latreille, 1802**

**Family Hydrophilidae Latreille, 1802** ^49^

Subfamily Hydrophilinae Latreille, 1802

Tribe Amphiopini Kuwert, 1890

Tribe Berosini Mulsant, 1844

= Derallini Bertrand, 1967

Tribe Hydrobiusini Mulsant, 1844

= Sperchopsini Hansen, 1991

Tribe Hydrophilini Latreille, 1802

= Hydatophilini Gistel, 1856

Tribe Laccobiini Houlbert, 1922

= Oocyclini Hansen, 1991

Subfamily Chaetarthriinae Bedel, 1881

Tribe Anacaenini Hansen, 1991

= Horelophini Hansen, 1991

Tribe Chaetarthriini Bedel, 1881

Subfamily Enochrinae Short & Fikáček, 2013

Subfamily Acidocerinae Zaitzev, 1908

= Helopeltinae G.H. Horn, 1873 [PI]

= Helocharinae Orchymont, 1919

= Horelophopsinae Hansen, 1997

= Globuloseinae García, 2001

Subfamily Cylominae Zaitzev, 1908

= Rygmodinae Orchymont, 1916 ^50^

= Andotypinae Hansen, 1991

= Borborophorinae Hansen, 1991

= Tormissinae Hansen, 1991

Subfamily Sphaeridiinae Latreille, 1802 ^H^

Tribe Coelostomatini Heyden, 1891 (1890)

= Cyclonotini G.H. Horn, 1890

Tribe Megasternini Mulsant, 1844

Subtribe Megasternina Mulsant, 1844

= Cercyonina G.H. Horn, 1890

Subtribe Oosternina Arriaga-Varela & Fikáček, 2021

Tribe Omicrini Smetana, 1975

Tribe Protosternini Hansen, 1991

Tribe Sphaeridiini Latreille, 1802 ^H^

**Hydrophilidae incertae sedis**

Escheriini Kolbe, 1880†

**Family Helophoridae Leach, 1815**

**Family Epimetopidae Zaitzev, 1908**

**Family Georissidae Laporte, 1840** ^H^

**Family Hydrochidae C.G. Thomson, 1859**

**Family Spercheidae Erichson, 1837**

**Superfamily Scarabaeoidea Latreille, 1802**

**Family Alloioscarabaeidae Bai et al., 2012**†

**Family Septiventeridae Bai et al., 2012**†

**Family Lucanidae Latreille, 1804**

Subfamily Protolucaninae Nikolajev, 2007†

Subfamily Aesalinae W.S. MacLeay, 1819

Tribe Aesalini W.S. MacLeay, 1819

= Plocastini Gistel, 1856

Tribe Ceratognathini Sharp, 1899

Tribe Nicagini J.L. LeConte, 1861

Subfamily Ceruchitinae Nikolajev, 2006†

Subfamily Syndesinae W.S. MacLeay, 1819

Tribe Ceruchini Jacquelin du Val, 1859

Tribe Sinodendrini Mulsant, 1842

Tribe Syndesini W.S. MacLeay, 1819

Subfamily Lampriminae W.S. MacLeay, 1819

Tribe Lamprimini W.S. MacLeay, 1819

Tribe Streptocerini Kikuta, 1986

Subfamily Lucaninae Latreille, 1804 ^51^

Tribe Aegini H. Huang & C.H. Chen, 2013

Tribe Capreolucanini H. Huang & C.H. Chen, 2013

Tribe Casignetini Reid, 1999

Tribe Chiasognathini Burmeister, 1847

Tribe Cyclommatini H. Huang & C.H. Chen, 2013

Tribe Dorcini Parry, 1864

= Cladognathini Parry, 1870

= Rhaetulini Miwa, 1931

= Prosopocoilini Benesh, 1960

Tribe Figulini Burmeister, 1847

= Nigidiini Jakobson, 1911

= Penichrolucanini Arrow, 1950

Tribe Lucanini Latreille, 1804

= Corypticini Gistel, 1848

= Leptinopterini Jakobson, 1911

= Calcodini Didier & Séguy, 1953

= Dendeziini Benesh, 1955

= Pseudodorcini Benesh, 1960

= Pholidotini Kikuta, 1986 [PI]

= Brasilucanini Nikolajev, 1999

Tribe Noseolucanini H. Huang & C.H. Chen, 2013

Tribe Odontolabidini Parry, 1870

Tribe Platycerini Mulsant, 1842

= Systenocerini Portevin, 1931

Tribe Platyceroidini Paulsen & Hawks, 2008

Tribe Rhyssonotini Benesh, 1960

Tribe Sclerostomini Benesh, 1955

= Lissotetini Benesh, 1955

= Scortizini Benesh, 1955

Subfamily Paralucaninae Nikolajev, 2000†

Subfamily Litholampriminae Nikolajev, 2015†

**Family Trogidae W.S. MacLeay, 1819**

Subfamily Kresnikinae Tihelka et al., 2019†

Subfamily Troginae W.S. MacLeay, 1819

= Phoberinae Gistel, 1848

Subfamily Omorginae Nikolajev, 2005

**Family Glaresidae Prudhomme de Borre, 1886**

**Family Pleocomidae J.L. LeConte, 1861**

Subfamily Cretocominae Nikolajev, 2002†

Subfamily Archescarabaeinae Nikolajev, 2010†

Subfamily Pleocominae J.L. LeConte, 1861

**Family Bolboceratidae Mulsant, 1842 ^52^**

Subfamily Athyreinae Lynch Arribálzaga, 1878

Subfamily Bolboceratinae Mulsant, 1842

Tribe Bolbelasmini Iablokoff-Khnzorian, 1977

Tribe Bolboceratini Mulsant, 1842

= Australobolbini Nikolajev, 1996

Tribe Bolbochromini Nikolajev, 1970

Tribe Eubolbitini Nikolajev, 1970

Tribe Eucanthini Nikolajev, 2003

Tribe Gilletinini Nikolajev, 1990

Tribe Odonteini Streubel, 1846

Tribe Stenaspidiini Nikolajev, 2003

**Family Diphyllostomatidae Holloway, 1972**

**Family Geotrupidae Latreille, 1802**

Subfamily Taurocerastinae Germain, 1897

Subfamily Geotrupinae Latreille, 1802

Tribe Ceratotrupini Zunino, 1984

Tribe Cretogeotrupini Nikolajev, 1996†

Tribe Enoplotrupini Paulian, 1945

= Chromogeotrupini Zunino, 1984

Tribe Geotrupini Latreille, 1802

Tribe Lethrini Oken, 1843 ^53^

**Family Passalopalpidae Boucher & Bai, 2016**†

**Family Passalidae Leach, 1815**

Subfamily Aulacocyclinae Kaup, 1868

Tribe Aulacocyclini Kaup, 1868

Tribe Ceracupedini Boucher, 2006

Tribe Cylindrocaulini Zang, 1905

= Ceracyclini Boucher & Bai, 2017†^54^

Subfamily Passalinae Leach, 1815

Tribe Leptaulacini Kaup, 1871

Tribe Macrolinini Kaup, 1871

= Aceraiini Kaup, 1871

= Eriocnemidini Kaup, 1871 [PI]

= Gonatini Kuwert, 1891 ^H^

= Mastachilini Kuwert, 1891

= Pharochilini Kuwert, 1891

= Tarquiniini Kuwert, 1891

= Vellejini Kuwert, 1891 [PI]

= Aureliini Kuwert, 1896

= Lachini Kuwert, 1896 [PI]

= Pelopini Kuwert, 1896 [PI]

= Pleurariini Kuwert, 1896

= Gnaphalocnemidini Heller, 1900

= Kaupiolini Zang, 1905

= Austropassalini Mjöberg, 1917

Tribe Passalini Leach, 1815

= Neleini Kaup, 1869 [PI]

= Pertinacini Kaup, 1871

= Phoroneini Kaup, 1871 [PI]

= Mitrorhinini Kuwert, 1891

= Paxillini Kuwert, 1891

= Petrejini Kuwert, 1891

= Neleidini Kuwert, 1896

= Ptichopodini Kuwert, 1896

= Rhodocanthopodini Kuwert, 1896

= Vatiniini Kuwert, 1896 [PI]

Tribe Proculini Kaup, 1868

= Pseudacanthini Kaup, 1871

= Oileini Kuwert, 1891

= Sertoriini Kuwert, 1891 [PI]

= Unduliferini Kuwert, 1891

= Veturiini Kuwert, 1891

= Popiliini Kuwert, 1896

= Proculejini Kuwert, 1896

= Spuriini Kuwert, 1896

= Vindicini Kuwert, 1896

Tribe ProtopassaliniAquino dos Santos et al., 2021†

Tribe Solenocyclini Kaup, 1871

= Stephanocephalini Kaup, 1871

= Ciceroniini Kuwert, 1891

= Erionomini Kuwert, 1891

= Flaminiini Kuwert, 1891

= Semicyclini Kuwert, 1891

**Family Belohinidae Paulian, 1959**

**Family Ochodaeidae Streubel, 1846**

Subfamily Cretochodaeinae Nikolajev, 1995†

Subfamily Ochodaeinae Streubel, 1846

Tribe Enodognathini Scholtz, 1988

Tribe Nothochodaeini Nikolajev, 2015

Tribe Ochodaeini Streubel, 1846

Subfamily Chaetocanthinae Scholtz, 1988

Tribe Chaetocanthini Scholtz, 1988

Tribe Pseudochodaeini Scholtz, 1988

Tribe Synochodaeini Scholtz, 1988

**Family Glaphyridae W.S. MacLeay, 1819**

Subfamily Cretoglaphyrinae Nikolajev, 2005†

Subfamily Glaphyrinae W.S. MacLeay, 1819

Subfamily Amphicominae Blanchard, 1845

= Anthypninae Chalande, 1884

**Family Hybosoridae Erichson, 1847 ^55^**

Subfamily Mimaphodiinae Nikolajev, 2007†

Subfamily Anaidinae Nikolajev, 1996

= Cryptogeniinae Howden, 2001

Subfamily Ceratocanthinae Martínez, 1968

Tribe Ceratocanthini Martínez, 1968

= Acanthocerini Streubel, 1846 [PI]

Tribe Scarabatermitini Nikolajev, 1999

= Ivieolini Howden & Gill, 2000 ^56^

Subfamily Hybosorinae Erichson, 1847

= Phaeochroinae Kolbe, 1912

Subfamily Liparochrinae Ocampo, 2006

Subfamily Pachyplectrinae Ocampo, 2006

**Family Scarabaeidae Latreille, 1802**

Subfamily Lithoscarabaeinae Nikolajev, 1992†

Subfamily Chironinae Blanchard, 1845

Subfamily Aegialiinae Laporte, 1840

Tribe Aegialiini Laporte, 1840

= Silluviini Landin, 1950

Tribe Saprinianini Nikolajev, 2011

= Saprini Nikolajev, 2008 [PI]

Subfamily Eremazinae Stebnicka, 1977

Tribe Eremazini Stebnicka, 1977

Tribe Yixianscarabaeini Nikolajev, 2015†

Subfamily Aphodiinae Leach, 1815

Tribe Aphodiini Leach, 1815

Subtribe Aphodiina Leach, 1815

Subtribe Didactyliina Pittino, 1985

Subtribe Proctophanina Stebnicka & Howden, 1995

Tribe Corythoderini Schmidt, 1910

Tribe Eupariini A. Schmidt, 1910 [NP] ^H^

= Ataeniini Harold, 1868 [NO]

= Lomanoxiini Stebnicka, 1999

Tribe HornietiiniMinkina, 2020

Tribe Odochilini Rakovič, 1987

Tribe Odontolochini Stebnicka & Howden, 1996

Tribe PsammaegialiiniNikolajev et al., 2014†

Tribe Psammodiini Mulsant, 1842

Subtribe Phycocina Landin, 1960

Subtribe Psammodiina Mulsant, 1842

= Psammobiina Reitter, 1909 ^H^

Subtribe Rhyssemina Pittino & Mariani, 1986 ^P^

= Pleurophorina Mulsant, 1842 ^57^

Tribe Rhyparini A. Schmidt, 1910

Tribe Stereomerini Howden & Storey, 1992

Tribe Termitoderini Tangelder & Krikken, 1982

Subfamily Aulonocneminae A. Janssens, 1946

Subfamily Termitotroginae Wasmann, 1918

Subfamily Scarabaeinae Latreille, 1802 ^58^

Tribe Ateuchini Perty, 1830

Subtribe Ateuchina Perty, 1830

= Choeridiina Harold, 1867

Subtribe Scatimina Vaz-de-Mello, 2008

Ateuchini incertae sedis

Demarziellini Balthasar, 1961

Tribe ByrrhidiiniDavis et al., 2019 [PI]

Tribe Coprini Leach, 1815

= Coptodactylini Paulian, 1933

Tribe Deltochilini Lacordaire, 1855

= Mentophilini Lacordaire, 1855

= Scatonomini Lacordaire, 1855

= Coprobiini Burmeister, 1873

= Canthonini van Lansberge, 1875 ^59^

= Panelini Arrow, 1931

Tribe Dichotomiini Pereira, 1954 ^P^

= Pinotini Kolbe, 1905

Tribe EndroedyoliniDavis et al., 2019

Tribe EpactoidiniRossini et al., 2022

Tribe Epilissini van Lansberge, 1875

Tribe Epirinini van Lansberge, 1875

Tribe Eucraniini Burmeister, 1873

= Ennearabdini Pereira & Martínez, 1956

Tribe Eurysternini Vulcano et al., 1961 ^H^

Tribe Gymnopleurini Streubel, 1846

Tribe OdontolominiDavis et al., 2019

Tribe Oniticellini Kolbe, 1905 ^60^

Subtribe Attavicina Philips, 2016

Subtribe Drepanocerina van Lansberge, 1875

Subtribe Helictopleurina A. Janssens, 1946

Subtribe Liatongina Philips, 2016

Subtribe Oniticellina Kolbe, 1905

Tribe Onitini Laporte, 1840

Tribe Onthophagini Streubel, 1846

= Alloscelini A. Janssens, 1946

Tribe Parachoriini Tarasov, 2017

Tribe Phanaeini Hope, 1838

Subtribe Gromphadina Zunino, 1985

Subtribe Phanaeina Hope, 1838

Tribe Scarabaeini Latreille, 1802

= Pachysomatini Ferreira, 1953

= Actinophorini Ádám, 2003

Tribe Sisyphini Mulsant, 1842

Subfamily Prototroginae Nikolajev, 2000†

Subfamily Cretoscarabaeinae Nikolajev, 1995†

Subfamily Dynamopodinae Arrow, 1911 ^61^

Tribe Dynamopodini Arrow, 1911

Tribe Thinorycterini Semenov-Tjan-Shansky & Reichardt, 1925

Subfamily Electrorubesopsinae Bai & B. Wang, 2018†

Subfamily Phaenomeridinae Erichson, 1847

Subfamily Orphninae Erichson, 1847

Tribe Aegidiini Paulian, 1984

Subtribe Aegidiina Paulian, 1984

Subtribe AegidininaFrolov et al., 2023

Tribe Orphnini Erichson, 1847

= Hybalini Marseul, 1857

Subfamily Allidiostomatinae Arrow, 1940

= Idiostomatinae Arrow, 1904 [PI]

Subfamily Aclopinae Blanchard, 1850

Tribe Aclopini Blanchard, 1850

Tribe Holcorobeini Nikolajev, 1992†

Tribe Phaenognathini Iablokoff-Khnzorian, 1977

Subfamily Melolonthinae Leach, 1819 ^62^

Tribe Acomini Evans & Smith, 2020

Tribe Ardellini Paulsen, 2021

Tribe Athliini Smith & Evans, 2018

Tribe Chasmatopterini Lacordaire, 1855

Tribe Chnaunanthini Evans & Smith, 2020

Tribe Colymbomorphini Blanchard, 1850

= Stethaspidini Burmeister, 1855

= Xylonichini Britton, 1957

Tribe Comophini Britton, 1978

= Comophorini Britton, 1957 [PI]

= Comophorinini Britton, 1987

Tribe Cretomelolonthini Nikolajev, 1998†

Tribe Dichelonychini Burmeister, 1855

Tribe Diphycerini S.I. Medvedev, 1952

Tribe Diplotaxini W. Kirby, 1837

= Apogoniini Blanchard, 1851

= Liogenyini Blanchard, 1851

Tribe Euchirini Hope, 1841

Tribe Heptophyllini S.I. Medvedev, 1951

Tribe Hopliini Latreille, 1829

Subtribe Hopliina Latreille, 1829

= Dichelina Oken, 1843

= Gymnolomatina Burmeister, 1844

= Heterochelina Burmeister, 1844

= Lepisiina Burmeister, 1844

= Chasmina Streubel, 1846

= Scelophysina Péringuey, 1902

Subtribe Lepithrichina Perty, 1840

= Pachycnemina Laporte, 1840

= Anisonychina Burmeister, 1844 ^H^

Tribe Langbianellini Prokofiev, 2015

Tribe Leucopholini Burmeister, 1855

Tribe Lichniini Burmeister, 1844

Tribe Macrodactylini W. Kirby, 1837

= Ceraspidini Burmeister, 1855

= Dicraniini Burmeister, 1855

= Isonychini Burmeister, 1855

= Microcraniini Burmeister, 1855

= Plectrini Burmeister, 1855

= Clavipalpini Lacordaire, 1855

Tribe Melolonthini Leach, 1819

Subtribe Enariina Dewailly, 1950

Subtribe Melolonthina Leach, 1819

= Polyphyllina Burmeister, 1855

Subtribe Pegylina Lacroix, 1989

Tribe Pachypodini Erichson, 1840

Tribe Pachytrichini Burmeister, 1855

Tribe Phobetusini Evans & Smith, 2020

Tribe Phyllotocidiini Britton, 1957

Tribe Rhizotrogini Burmeister, 1855

= Tostegopterini J.L. LeConte, 1861

= Triodonini Rivera-Gasperín & Morón, 2013

Tribe Schizonychini Burmeister, 1855

= Psilonychini Péringuey, 1904

Tribe Systellopini Sharp, 1877

Tribe Tanyproctini Erichson, 1847

Subtribe Macrophyllina Burmeister, 1855 ^H^

= Aegosthetina Lacroix, 2007

Subtribe Tanyproctina Erichson, 1847

= Elaphocerina Blanchard, 1851

= Achloina Burmeister, 1855

= Cephalotrichiina Burmeister, 1855

= Pachydemina Burmeister, 1855 ^63^

= Sparrmanniini Péringuey, 1904

Tribe Warwickiini Evans & Smith, 2020

Subfamily Sericinae W. Kirby, 1837 ^64^

Tribe Ablaberini Blanchard, 1850

= Camentini Machatschke, 1959

Tribe Diphucephalini Laporte, 1840

Tribe Sericini W. Kirby, 1837

Subtribe Astaenina Burmeister, 1855 ^65^

Subtribe Sericina W. Kirby, 1837

= Omalopliina Blanchard, 1845

Subtribe Trochalina Brenske, 1898

Subfamily Sericoidinae Erichson, 1847 ^66^

Tribe Automoliini Britton, 1978

= Caulobiini Burmeister, 1855 [PI]

= Automolini Britton, 1957 [PI]

Tribe Heteronychini Lacordaire, 1855

Tribe Liparetrini Burmeister, 1855

= Aplonychini Burmeister, 1855 ^H^

= Allarini Britton, 1955

= Colpochilini Britton, 1957

Tribe Maechidiini Burmeister, 1855

Tribe Phyllotocini Burmeister, 1855

Tribe Scitalini Britton, 1957

Tribe Sericoidini Erichson, 1847

Subfamily Oncerinae J.L. LeConte, 1861 ^67^

Subfamily Podolasiinae Howden, 1997 ^68^

= Lasiopodinae J.L. LeConte, 1856 [PI]

Subfamily Rutelinae W.S. MacLeay, 1819

Tribe Adoretini Burmeister, 1844

Subtribe Adoretina Burmeister, 1844

= Adorodociina Ohaus, 1912

= Adoroleptina Ohaus, 1912

= Pseudadoretina Ohaus, 1912

= Scaphorhinadoretina Ohaus, 1912

Subtribe Adorrhinyptiina Arrow, 1917

Subtribe Pachyrhinadoretina Ohaus, 1912

Subtribe Prodoretina Ohaus, 1912

Subtribe Trigonostomusina Ohaus, 1912

Tribe Alvarengiini Frey, 1975

Tribe Anatistini Lacordaire, 1855

= Spodochlamydini Ohaus, 1918

Tribe Anomalini Streubel, 1839 [NP]

Subtribe Anisopliina Burmeister, 1844

Subtribe Anomalina Streubel, 1839 [NP]

= Euchlorina Hope, 1839 [NO]

= Phyllurgina Gistel, 1848

= Mimelina Theobald, 1882

= Dilophochilina Ohaus, 1918

Subtribe Isopliina Péringuey, 1902

Subtribe Leptohopliina Potts, 1974

Subtribe Popilliina Ohaus, 1902

Tribe Anoplognathini W.S. MacLeay, 1819

Subtribe Anoplognathina W.S. MacLeay, 1819

= Repsimina Streubel, 1846

Subtribe Brachysternina Burmeister, 1844

Subtribe Phalangogoniina Ohaus, 1918

Subtribe Platycoeliina Burmeister, 1844

Subtribe Schizognathina Ohaus, 1918

Tribe Geniatini Burmeister, 1844

= Leucothyreini Burmeister, 1844

Tribe Rutelini W.S. MacLeay, 1819

Subtribe Areodina Burmeister, 1844

Subtribe Desmonychina Arrow, 1917

Subtribe Didrepanephorina Ohaus, 1918

Subtribe Heterosternina H.W. Bates, 1888 [NP]

= Macropnina G.H. Horn, 1867 [NO]

Subtribe Lasiocalina Ohaus, 1918

Subtribe Oryctomorphina Burmeister, 1847

Subtribe Parastasiina Burmeister, 1844

Subtribe Rutelina W.S. MacLeay, 1819

= Macraspidina Perty, 1840

= Chasmodiina Burmeister, 1844

= Chrysophorina Burmeister, 1844

= Pelidnotina Burmeister, 1844

= Ometina Streubel, 1846

= Anticheirina Lacordaire, 1855

= Plusiotidina H.W. Bates, 1888

= Fruhstorferiina Ohaus, 1918

Subfamily Dynastinae W.S. MacLeay, 1819

Tribe Agaocephalini Burmeister, 1847

Tribe Cyclocephalini Laporte, 1840

= Chalepini Streubel, 1846 [PI]

= Peltonotini Arrow, 1917

= Acrobolbiini Ohaus, 1918

Tribe Dynastini W.S. MacLeay, 1819 ^69^

Subtribe Chalcosomina Rowland & K.B. Miller, 2012

Subtribe Dynastina W.S. MacLeay, 1819

= Megasomatina Swainson, 1840

Subtribe Xylotrupina Hope, 1838

Tribe Hexodontini Lacordaire, 1855

Tribe Oryctini Mulsant, 1842

= Megaceratini Burmeister, 1847

= Strategini Burmeister, 1847

Tribe Oryctoderini Endrödi, 1966

Tribe Pentodontini Mulsant, 1842

Subtribe Cheiroplatina Carne, 1957

Subtribe Dipelicina Carne, 1957

Subtribe Pentodontina Mulsant, 1842

= Calicnemidina Mulsant, 1842

= Bothynina Burmeister, 1847

= Pimelopodina Burmeister, 1847

= Podalgina Burmeister, 1847

= Metanastina Carne, 1957

Subtribe Pseudoryctina Carne, 1957

Tribe Phileurini Burmeister, 1847

Subtribe Cryptodina Burmeister & Schaum, 1840

Subtribe Phileurina Burmeister, 1847

Subfamily Cetoniinae Leach, 1815 ^70^

Tribe Cetoniini Leach, 1815

Subtribe Cetoniina Leach, 1815

= Elaphinina Schoch, 1894

= Glycyphanina Schoch, 1894

= Pachnodina Péringuey, 1907 ^H^

= Tephraeina Schenkling, 1921

Subtribe Euphoriina G.H. Horn, 1880

Subtribe Leucocelina Kraatz, 1882

Tribe Cremastocheilini Burmeister & Schaum, 1841

Subtribe Aspilina Krikken, 1984

Subtribe Coenochilina Burmeister, 1842

Subtribe Cremastocheilina Burmeister & Schaum, 1841

Subtribe Cymophorina Krikken, 1984

Subtribe Genuchina Krikken, 1984

Subtribe Goliathopsidina Krikken, 1984

Subtribe Heterogeniina Krikken, 1984

Subtribe Lissogeniina Krikken, 1984

Subtribe Macromina Burmeister & Schaum, 1840

Subtribe Nyassinina Krikken, 1984

Subtribe Oplostomina Krikken, 1984

Subtribe Pilinurgina Krikken, 1984

Subtribe Spilophorina Krikken, 1984 ^H^

Subtribe Telochilina Krikken, 1984

Subtribe Trichoplina Krikken, 1984

Subtribe Trogodina Krikken, 1984

Tribe Cryptodontini Lacordaire, 1855 ^H^

Tribe Diplognathini Burmeister, 1842

= Porphyronotini Péringuey, 1907

Tribe Goliathini Latreille, 1829

Subtribe Dicronocephalina Krikken, 1984

Subtribe Goliathina Latreille, 1829

= Hypselogeniina Schoch, 1894

Subtribe Ichnestomatina Burmeister, 1842

Subtribe Rhomborhinina Westwood, 1842

= Coryphocerina Burmeister, 1842

= Jumnina Burmeister, 1842

= Ceratorhinina Kraatz, 1880

= Heterorhinina Kraatz, 1880

= Bothrorrhinina Schoch, 1894

= Gnathocerina Schoch, 1894

= Ischnoscelidina Schoch, 1894

= Tmesorrhinina Schoch, 1894

= Coelorrhinina Schoch, 1895

= Mecynorhinina Schenkling, 1921

= Stephanorrhinina Schenkling, 1921

Tribe Gymnetini W. Kirby, 1827

Subtribe Blaesiina Schoch, 1895

Subtribe Gymnetina W. Kirby, 1827

= Clinteriina Kraatz, 1882

= Stethodesmatina Schoch, 1894

Tribe Incini Burmeister, 1842

Tribe Osmodermatini Schenkling, 1922

Tribe Phaedimini Schoch, 1894 ^H^

Tribe Platygeniini Krikken, 1984

Tribe Schizorhinini Burmeister, 1842

Subtribe Lomapterina Burmeister, 1842

= Macronotina Burmeister, 1842

Subtribe Schizorhinina Burmeister, 1842

= Diaphoniina Kraatz, 1880

= Eupoecilina Kraatz, 1880

= Hemipharina Kraatz, 1880

Tribe Stenotarsiini Kraatz, 1880

Subtribe Anochiliina Krikken, 1984

Subtribe Chromoptiliina Krikken, 1984

Subtribe Coptomiina Schenkling, 1921

Subtribe Doryscelina Schenkling, 1921

Subtribe Euchroeina Paulian & Descarpentries, 1982 ^H^

Subtribe Heterophanina Schoch, 1894

Subtribe Heterosomatina Krikken, 1984

Subtribe Pantoliina Krikken, 1984

Subtribe Parachiliina Krikken, 1984

Subtribe Stenotarsiina Kraatz, 1880

Tribe Taenioderini Mikšić, 1976

Subtribe Chalcotheina Mikšić, 1976

Subtribe Taenioderina Mikšić, 1976

Tribe Trichiini Fleming, 1821

= Paniscini Gistel, 1848 [PI]

= Evambatini Gistel, 1856

= Elpidini Péringuey, 1907

= Myodermatini Péringuey, 1907

Tribe Valgini Mulsant, 1842

Subtribe Microvalgina Kolbe, 1904

Subtribe Valgina Mulsant, 1842

= Acanthovalgina Kolbe, 1904

= Cosmovalgina Kolbe, 1904

= Dasyvalgina Kolbe, 1904

= Ischnovalgina Kolbe, 1904

= Sphinctovalgina Kolbe, 1904

Tribe Xiphoscelidini Burmeister, 1842

**Family Coprinisphaeridae Genise, 2004† ^I^**

**Family Pallichnidae Genise, 2004† ^I^**

**Superfamily Staphylinoidea Latreille, 1802**

**Family Jacobsoniidae Heller, 1926**

= Sarothriidae Crowson, 1955

= Derolathrinae Sen Gupta, 1979

**Family Ptiliidae Erichson, 1845** ^71^

Subfamily Nossidiinae Sörensson & Delgado, 2019

Subfamily Ptiliinae Erichson, 1845

Tribe Acrotrichini Reitter, 1909 (1856)

= Trichopterygini Erichson, 1845 [PI]

= Cleopteriini Gistel, 1856

= Nephanini Portevin, 1929

Tribe Cephaloplectini Sharp, 1883

= Limulodini Ganglbauer, 1898

Tribe Discheramocephalini Grebennikov, 2009

Tribe Nanosellini Barber, 1924

Tribe Ptenidiini Flach, 1889

Tribe Ptiliini Erichson, 1845

= Ptilopteriini Gistel, 1848

= Actidiini Portevin, 1929

Tribe Ptinellini Reitter, 1906 (1891)

= Neuglenini Reitter, 1891

**Family Hydraenidae Mulsant, 1844**

Subfamily Orchymontiinae Perkins, 1997

Subfamily Prosthetopinae Perkins, 1994

Tribe Coelometoponini Perkins, 2005

Tribe Nucleotopini Perkins, 1994

Tribe Parasthetopini Perkins, 1994

Tribe Prosthetopini Perkins, 1994

= Pterosthetopini Perkins, 1994 ^72^

Tribe Protosthetopini Perkins, 1994

Subfamily Hydraeninae Mulsant, 1844

Tribe Hydraenidini Perkins, 1980

Tribe Hydraenini Mulsant, 1844

Tribe Limnebiini Mulsant, 1844

Tribe Madagastrini Perkins, 1997

Tribe Parhydraenini Perkins, 1997

Subfamily Ochthebiinae C.G. Thomson, 1859 ^73^

Tribe Ochthebiini C.G. Thomson, 1859

= Enicocerini Perkins, 1997

= Meropathini Perkins, 1997

= Neochthebiini Perkins, 1997

= Protochthebiini Perkins, 1997

Tribe Ochtheosini Perkins, 1997

**Family Colonidae G.H. Horn, 1880 (1859)**

= Myloechidae C.G. Thomson, 1859

**Family Agyrtidae C.G. Thomson, 1859**

Subfamily Agyrtinae C.G. Thomson, 1859

= Lyrosomatinae G.H. Horn, 1880

= Lendominae Bradley, 1930

Subfamily Necrophilinae Newton, 1997

Subfamily Pterolomatinae C.G. Thomson, 1862

**Family Leiodidae Fleming, 1821**

Subfamily Camiarinae Jeannel, 1911

Tribe Agyrtodini Jeannel, 1936

Tribe Camiarini Jeannel, 1911

Tribe Neopelatopini Jeannel, 1962

Subfamily Catopocerinae Hatch, 1927 (1880)

Tribe Catopocerini Hatch, 1927 (1880)

= Pinodytini G.H. Horn, 1880

Tribe Glacicavicolini Westcott, 1968

Subfamily Leiodinae Fleming, 1821

Tribe Anisotomini Horaninow, 1834

= Agathidiini Westwood, 1838

Tribe Estadiini Portevin, 1914

Tribe Leiodini Fleming, 1821

Tribe Pseudoliodini Portevin, 1926

= Dermatohomoeini Hlisnikovský, 1963

Tribe Scotocryptini Reitter, 1884

Tribe Sogdini Lopatin, 1961

= Hydnobiini Jeannel, 1962

= Triarthrini Jeannel, 1962 ^H^

Subfamily Cholevinae W. Kirby, 1837

Tribe Anemadini Hatch, 1927

Subtribe Anemadina Hatch, 1927

Subtribe Eocatopina Jeannel, 1936

Subtribe Eunemadina Newton, 1998

Subtribe Nemadina Jeannel, 1936

Subtribe Paracatopina Jeannel, 1936

Tribe Cholevini W. Kirby, 1837

Subtribe Catopina Chaudoir, 1845

Subtribe Cholevina W. Kirby, 1837

Tribe Eucatopini Jeannel, 1921

Tribe Leptodirini Lacordaire, 1854 (1849)

Subtribe Anthroherponina Jeannel, 1910

= Hadesiina Absolon, 1913

Subtribe Bathysciina G.H. Horn, 1880

= Oryotina Reitter, 1889

= Neotropospeonellina Perkovsky, 1997

Subtribe Bathysciotina Guéorguiev, 1974

Subtribe Leptodirina Lacordaire, 1854 (1849)

= Stagobiina Schiødte, 1849

Subtribe Pholeuina Reitter, 1886

= Ghidiniina Guéorguiev, 1974

Subtribe Platycholeina G.H. Horn, 1880

Subtribe Spelaeobatina Guéorguiev, 1974

Tribe Oritocatopini Jeannel, 1936

Tribe Ptomaphagini Jeannel, 1911

Subtribe Baryodirina Perreau, 2000

Subtribe Ptomaphagina Jeannel, 1911

Subtribe Ptomaphaginina Szymczakowski, 1964

Tribe Sciaphyini Perreau, 2000

Subfamily Platypsyllinae Ritsema, 1869

= Leptininae J.L. LeConte, 1872

**Family Staphylinidae Latreille, 1802**

Subfamily Silphinae Latreille, 1806

Tribe Silphini Latreille, 1806

= Necrodini Gistel, 1848

Tribe Nicrophorini W. Kirby, 1837

Subfamily Apateticinae Fauvel, 1895

Subfamily Trigonurinae Reiche, 1866

Subfamily Scaphidiinae Latreille, 1806

Tribe Cypariini Achard, 1924

Tribe Scaphidiini Latreille, 1806

= Cerambyciscaphini Pic, 1915

= Diateliini Achard, 1924

Tribe Scaphiini Achard, 1924

Tribe Scaphisomatini Casey, 1893

= Heteroscaphini Achard, 1914

= Baeoceridiini Achard, 1924

= Baeocerini Achard, 1924

= Cyparellini Achard, 1924

= Scaphicomini Achard, 1924

= Sciatrophini Achard, 1924

= Toxidiini Achard, 1924

Subfamily Piestinae Erichson, 1839

= Prognathinae Blanchard, 1845

Subfamily Osoriinae Erichson, 1839

Tribe Eleusinini Sharp, 1887

Tribe Leptochirini Sharp, 1887

Tribe Osoriini Erichson, 1839

Subtribe Osoriina Erichson, 1839

Subtribe Parosoriina Bernhauer & Schubert, 1911

Tribe Thoracophorini Reitter, 1909

Subtribe Clavilispinina Newton & Tayer, 1992

= Paralispinina Blackwelder, 1942 [PI]

Subtribe Glyptomina Newton & Tayer, 1992

= Calocerina Blackwelder, 1942 [PI]

Subtribe Lispinina Bernhauer & Schubert, 1910

Subtribe Thoracophorina Reitter, 1909

Subfamily Oxytelinae Fleming, 1821

Tribe Blediini Ádám, 2001

Tribe Coprophilini Heer, 1839

= Toxoderini Bernhauer & Schubert, 1911 ^H^

Tribe Deleasterini Reitter, 1909 ^74^

Tribe Euphaniini Reitter, 1909

= Pholidini Acloque, 1895 [PI]

Tribe Oxytelini Fleming, 1821

= Trogophloeini Mulsant & Rey, 1878

= Apocellini Lynch Arribálzaga, 1884

= Ecitoclimacini Borgmeier, 1934

Tribe Planeustomini Jacquelin du Val, 1857

= Acrognathini Reitter, 1909 [PI]

= Mandini Gildenkov, 2003

Tribe Syntomiini Böving & Craighead, 1931 ^75^

Tribe Thinobiini J.R. Sahlberg, 1876 ^76^

= Torrentomini Bierig, 1934

= Carpelimini Hatch, 1957

= Aploderini Ádám, 2001

Oxytelinae incertae sedis

Trigonobregmatini Scheerpeltz, 1944 ^77^

Subfamily Protactinae Heer, 1847†

Subfamily Omaliinae W.S. MacLeay, 1825

Tribe Anthophagini C.G. Thomson, 1859

= Brathinini J.L. LeConte, 1861

= Lestevini Jakobson, 1908

Tribe Aphaenostemmini Peyerimhoff, 1914

Tribe Corneolabiini Steel, 1950

Tribe Coryphiini Jakobson, 1908

Subtribe Boreaphilina Zerche, 1990

Subtribe Coryphiina Jakobson, 1908

Tribe Eusphalerini Hatch, 1957

Tribe Hadrognathini Portevin, 1929

Tribe Omaliini W.S. MacLeay, 1825

= Micralymmatini Mulsant & Rey, 1880 ^78^

= Tetradelini Fauvel, 1904

= Arpediomimini Cameron, 1917

= Arpediopseini Cameron, 1917 [PI]

= Phloeonomini Ádám, 2001

Subfamily Microsilphinae Crowson, 1950

= Micragyrtinae Jeannel, 1962

Subfamily Glypholomatinae Jeannel, 1962

Subfamily Empelinae Newton & Tayer, 1992

Subfamily Proteininae Erichson, 1839

Tribe Anepiini Steel, 1966

Tribe Austrorhysini Newton & Thayer, 1995

Tribe Nesoneini Steel, 1966

Tribe Proteinini Erichson, 1839

= Megarthrini Joy, 1932

= Metopsiini Tottenham, 1954

= Pteroniini Arnett, 1961

Tribe Silphotelini Newton & Tayer, 1995

Tribe VetuproteininiCai et al., 2016†

Subfamily Micropeplinae Leach, 1815

Subfamily Neophoninae Fauvel, 1905

Subfamily Dasycerinae Reitter, 1887

Subfamily Protopselaphinae Newton & Tayer, 1995

Subfamily Pselaphinae Latreille, 1802

Supertribe Batrisitae Reitter, 1882

Tribe Amauropini Jeannel, 1948

Tribe Batrisini Reitter, 1882

Subtribe Ambicocerina Leleup, 1970

Subtribe Batrisina Reitter, 1882

= Orropygiina Jeannel, 1949

= Trabisina Jeannel, 1949

Subtribe Leupeliina Jeannel, 1954

Subtribe Stilipalpina Jeannel, 1954

Tribe ThaumastocephaliniPoggi et al., 2001

Supertribe Clavigeritae Leach, 1815 ^H, 79^

Tribe Clavigerini Leach, 1815 ^H^

= Adranini Desmarest, 1852 ^H^

= Articerini Desmarest, 1852

= Clavigerodini L.W. Schaufuss, 1882

= Commatocerini L.W. Schaufuss, 1882

= Clavigeropseini L.W. Schaufuss, 1890

= Fustigerini Jeannel, 1949

= Miroclavigerini Jeannel, 1949

= Apoderigerini Jeannel, 1954

= Neocerini Jeannel, 1954

= Radamini Jeannel, 1954

= Theocerini Jeannel, 1954

= Thysdariini Jeannel, 1954

= Hoplitoxenini Célis, 1969

= Dimerometopini Célis, 1970

= Neoceratopseini Célis, 1970

Tribe Disarthricerini Jeannel, 1949

Tribe Lunillini Célis, 1969

Tribe Mastigerini Jeannel, 1954

Tribe Protoclavigerini J. Parker & Grimaldi, 2014†

Tribe Tiracerini Besuchet, 1986

Supertribe Euplectitae Streubel, 1839

Tribe Bythinoplectini L.W. Schaufuss, 1890

Subtribe Bythinoplectina L.W. Schaufuss, 1890

= Zethina L.W. Schaufuss, 1890 [PI]

= Zethopsina Jeannel, 1952

Subtribe Pyxidicerina Raffray, 1904

Tribe Dimerini Raffray, 1908

= Octomicrini Jeannel, 1952

Tribe Euplectini Streubel, 1839

= Mitracephalini Park, 1951 [PI]

= Mitrametopini Park, 1952

Tribe Jubini Raffray, 1898

= Auxenocerini Jeannel, 1962

Tribe Mayetiini Scheerpeltz, 1925

Tribe Metopiasini Raffray, 1904

Subtribe Metopiasina Raffray, 1904

Subtribe Rhinoscepsina Bowman, 1934

Tribe Trichonychini Reitter, 1882

Subtribe Bibloporina Park, 1951

= Bibloporellina Jeannel, 1952

Subtribe Panaphantina Jeannel, 1950

= Trisignina Park & Schuster, 1955

= Acetaliina Jeannel, 1958

= Bibloplectina Jeannel, 1959

Subtribe Trichonychina Reitter, 1882

= Raffrayiina Jeannel, 1949

= Ranavalina Jeannel, 1954

= Chrestomerina Jeannel, 1962

= Pteracmetina Jeannel, 1962

= Trimiodytina Jeannel, 1964

Subtribe Trimiina Brendel & Wickham, 1890

Tribe Trogastrini Brendel & Wickham, 1890

Subtribe Phtegnomina Park, 1951

Subtribe Rhexiina Park, 1951

Subtribe Trogastrina Brendel & Wickham, 1890

Supertribe Faronitae Reitter, 1882

Supertribe Goniaceritae Reitter, 1882 (1872)

Tribe Arnylliini Jeannel, 1952

Tribe Barrosellini Leleup, 1973

Tribe Brachyglutini Raffray, 1904

Subtribe Baradina Park, 1951

Subtribe Brachyglutina Raffray, 1904

= Bryaxeina J.L. LeConte, 1861 [PI]

= Reichenbachiina Jakobson, 1910

= Globina Jeannel, 1959

= Halorabyxina Leleup, 1969

= Pselaptina Park, 1976

Subtribe Decarthrina Park, 1951

Subtribe Eupseniina Park, 1951

Tribe Bythinini Raffray, 1890

Subtribe Bythinina Raffray, 1890

= Bryaxeina Jakobson, 1910

Subtribe Machaeritina Jeannel, 1950

Subtribe Xenobythina Jeannel, 1950

Tribe Cyathigerini L.W. Schaufuss, 1872

Tribe Goniacerini Reitter, 1882 (1872)

= Goniastini L.W. Schaufuss, 1872

= Listriophorini L.W. Schaufuss, 1890

= Simini L.W. Schaufuss, 1890 [PI]

Tribe Imirini Jeannel, 1949

= Mirini Raffray, 1917 [PI]

Tribe Iniocyphini Park, 1951

Subtribe Iniocyphina Park, 1951

= Dalmodina Park, 1951

Subtribe Natypleurina Newton & Tayer, 1992

= Tanypleurina Jeannel, 1949 [PI]

Tribe Machadoini Jeannel, 1951

Tribe Proterini Jeannel, 1949

Tribe Pygoxyini Reitter, 1909

Tribe Speleobamini Park, 1951

Tribe Tychini Raffray, 1904 ^H^

Tribe Valdini Park, 1953

Supertribe Pselaphitae Latreille, 1802

Tribe Arhytodini Raffray, 1890

= Holozodini Raffray, 1900

Tribe Attapseniini Bruch, 1933

Tribe Colilodionini Besuchet, 1991 ^80^

Tribe Ctenistini Blanchard, 1845

= Chenniini Reitter, 1882

Tribe Hybocephalini Raffray, 1890

= Mestogastrini Jakobson, 1910

Tribe Odontalgini Jeannel, 1949

Tribe Pachygastrodini Leleup, 1969

Tribe Phalepsini Jeannel, 1949

Tribe Pselaphini Latreille, 1802

Tribe Schistodactylini Raffray, 1890

Tribe Tmesiphorini Jeannel, 1949

Tribe Tyrini Reitter, 1882

Subtribe Centrophthalmina Jeannel, 1949

= Petanopina Jeannel, 1954

Subtribe Janusculina Cerruti, 1970

Subtribe Somatipionina Jeannel, 1949

= Hamotina Park, 1951

Subtribe Tyrina Reitter, 1882

= Chalcoplectina Oke, 1925

= Ceophyllina Park, 1951

Subfamily Mycetoporinae C.G. Thomson, 1859

= Bolitobiinae G.H. Horn, 1877

Subfamily Tachyporinae W.S. MacLeay, 1825 ^81^

Tribe Deropini Smetana, 1983

Tribe Tachinusini Fleming, 1821

= Megarthropsini Cameron, 1919

Tribe Tachyporini W.S. MacLeay, 1825

Subtribe Euconosomatina Cameron, 1918

= Conosomatina Jeannel & Jarrige, 1949 [PI]

= Sepedophilina Ádám, 2001

Subtribe Tachyporina W.S. MacLeay, 1825

= Symmixina Bernhauer, 1915

Tribe Vatesini Seevers, 1958

= Xenocephalini Wasmann, 1887 [PI]

Subfamily Phloeocharinae Erichson, 1839

= Scotodytinae Saulcy, 1870

Subfamily Trichophyinae C.G. Thomson, 1858

Subfamily Habrocerinae Mulsant & Rey, 1876

Subfamily Aleocharinae Fleming, 1821 ^82^

Tribe Actocharini Bernhauer & Schubert, 1911 ^83^

Tribe Aenictoteratini Kistner, 1993

= Aenictobiini Kistner, 1997

Tribe Akatastopsisini Pace, 2000

Tribe Aleocharini Fleming, 1821

Subtribe Aleocharina Fleming, 1821

= Piochardiina Fenyes, 1918

Subtribe Compactopediina Kistner, 1970

Subtribe Hodoxenina Kistner, 1970

Tribe Antillusini Pace, 2012

Tribe Athetini Casey, 1910

Subtribe Athetina Casey, 1910

= Strigotina Casey, 1910

= Plagiarthrinina Cameron, 1926

= Acrotonina Seevers, 1978

= Dimetrotina Seevers, 1978

Subtribe Coptotermoeciina Kistner & Pasteels, 1970

Subtribe Ecitocharina Seevers, 1965 ^84^

Subtribe Microceroxenina Kistner, 1970

Subtribe Nasutiphilina Kistner, 1970

Subtribe Schistogeniina Fenyes, 1918

Subtribe Termitotelina Kistner, 1970

Subtribe Thamiaraeina Fenyes, 1921

Tribe Australestesini Pace, 2015

Tribe Autaliini C.G. Thomson, 1859

= Ophioglossini Fenyes, 1918

= Rhopalogastrini Fenyes, 1918

Tribe Boreocyphini Klimaszewski & Langor, 2011

Tribe Cordobanini Bernhauer, 1910

Tribe Corotocini Fenyes, 1918

Subtribe Abrotelina Seevers, 1957

Subtribe Corotocina Fenyes, 1918

= Termitomimina Fenyes, 1921

Subtribe EburniogastrinaJacobson et al., 1986

Subtribe NasutitellinaJacobson et al., 1986

Subtribe Sphuridaethina Pace, 1988

Subtribe Termitocharina Seevers, 1957

Subtribe TermitocupidinaJacobson et al., 1986

Subtribe Termitogastrina Bernhauer & Scheerpeltz, 1926

= TermitellinaJacobson et al., 1986

= TermitogastrinaJacobson et al., 1986

Subtribe TermitoiceinaJacobson et al., 1986

Subtribe TermitopithinaJacobson et al., 1986

Subtribe Termitoptochina Fenyes, 1921

Subtribe Timeparthenina Fenyes, 1921

Tribe Crematoxenini Mann, 1921

= Pulicomorphini Mann, 1924

= Philacamatini Bruch, 1933

Tribe Cryptonotopsisini Pace, 2003

Tribe Diestotini Mulsant & Rey, 1871

= Elachistarthrini Notman, 1920

Tribe Diglottini Jakobson, 1909

= Diglossini Mulsant & Rey, 1873 [PI]

Tribe Digrammini Fauvel, 1900

Tribe Dorylogastrini Wasmann, 1916

Tribe Dorylomimini Wasmann, 1916

Tribe Drepanoxenini Kistner & Watson, 1972

Tribe Ecitogastrini Fenyes, 1918

Tribe Eusteniamorphini Bernhauer & Scheerpeltz, 1926

Tribe Falagriini Mulsant & Rey, 1873

Tribe Feldini Kistner, 1972

Tribe Geostibini Seevers, 1978 ^85^

= Callicerini Jakobson, 1908 ^S^

Tribe Gymnusini Heer, 1839

= Deinopsini Sharp, 1883

= Adinopseini Cameron, 1919

Tribe Himalusini Klimaszewski et al., 2010

= Sinanarchusini Pace, 2013 ^86^

Tribe Homalotini Heer, 1839

Subtribe Bolitocharina C.G. Thomson, 1859

= Euryusina C.G. Thomson, 1859

= Sipaliina Casey, 1910 [PI]

= Heterotina Fenyes, 1918

= Leptusina Fenyes, 1918

= Nanoglossina Fenyes, 1918

= Ditropaliina Hatch, 1957

Subtribe Dinardopsina Bernhauer & Scheerpeltz, 1926

Subtribe Gyrophaenina Kraatz, 1856

Subtribe Homalotina Heer, 1839

= Thecturotina Fenyes, 1918

Subtribe Silusina Fenyes, 1918

Tribe Hoplandriini Casey, 1910

Subtribe Hoplandriina Casey, 1910

Subtribe Platandriina Hanley, 2002

Subtribe Pseudoplandriina Hanley, 2002

Tribe Hygronomini C.G. Thomson, 1859

Subtribe Hygronomina C.G. Thomson, 1859

Subtribe Saphoglossina Bernhauer & Scheerpeltz, 1926

Tribe Hypocyphtini Laporte, 1835

= Oligotini C.G. Thomson, 1859

= Nematoscelidini Fenyes, 1921

Tribe Leucocraspedini Fenyes, 1921

Tribe Liparocephalini Fenyes, 1918

Tribe Lomechusini Fleming, 1821

Subtribe Lomechusina Fleming, 1821

= Xenodusina Seevers, 1978

Subtribe Myrmedoniina C.G. Thomson, 1867

= Zyrina Bradley, 1930

= Athexeniina Pace, 2000

Subtribe Termitozyrina Seevers, 1957

Tribe Masuriini Cameron, 1939

Tribe Mesoporini Cameron, 1959

Tribe Mimanommatini Wasmann, 1912

Subtribe Dorylophilina Fenyes, 1921

= Deremina Seevers, 1965

Subtribe Mimanommatina Wasmann, 1912

Tribe Mimecitini Wasmann, 1909

Subtribe Labidopullina Jacobson & Kistner, 1991

Subtribe Leptanillophilina Fenyes, 1918

Subtribe Mimecitina Wasmann, 1909

Subtribe Mimonillina Bernhauer & Scheerpeltz, 1926

Tribe Myllaenini Ganglbauer, 1895

= Dimonomerini Cameron, 1933

Tribe Oxypodini C.G. Thomson, 1859 ^87^

Subtribe Aphytopodina Bernhauer & Scheerpeltz, 1926

Subtribe Dinardina Mulsant & Rey, 1873

= Decusina Fenyes, 1918

= Blepharhymenina Klimaszewski & Peck, 1986

Subtribe Meoticina Seevers, 1978

Subtribe Microglottina Fenyes, 1918

Subtribe Oxypodina C.G. Thomson, 1859

= Ocaleina C.G. Thomson, 1859

= Ocyusina Mulsant & Rey, 1874

= Caloderina Mulsant & Rey, 1874

Subtribe Phloeoporina C.G. Thomson, 1859

Tribe Oxypodinini Fenyes, 1918

= Heterotaxini Fenyes, 1921

Tribe Paglini Newton & Tayer, 1992

= Pachyglossini Fenyes, 1918 [PI]

Tribe Paradoxenusini Bruch, 1937

Tribe Pediculotini Ádám, 1987

Tribe Philotermitini Seevers, 1957

Tribe Phyllodinardini Wasmann, 1916

Tribe Phytosini C.G. Thomson, 1867

Tribe Placusini Mulsant & Rey, 1871

Tribe Pronomaeini Mulsant & Rey, 1873

Tribe Pseudoperinthini Cameron, 1939

Tribe Pygostenini Fauvel, 1899

= Sympolemontini Fenyes, 1918

Tribe Sahlbergiini Kistner, 1993

Tribe Sceptobiini Seevers, 1978

Tribe Skatitoxenini Kistner & Pasteels, 1969

Tribe Tachyusini C.G. Thomson, 1859

Tribe Taxicerini Lohse, 1989

Tribe Termitodiscini Wasmann, 1904

Tribe Termitohospitini Seevers, 1941

Subtribe Hetairotermitina Seevers, 1957

Subtribe Termitohospitina Seevers, 1941

Tribe Termitonannini Fenyes, 1918

Subtribe Perinthina Bernhauer & Scheerpeltz, 1926

Subtribe Termitonannina Fenyes, 1918

Tribe Termitopaediini Seevers, 1957

= Termitondini Seevers, 1957

Tribe Termitusini Fenyes, 1918

Subtribe Termitospectrina Seevers, 1957

Subtribe Termitusina Fenyes, 1918

Tribe Trichopseniini J.L. LeConte & G.H. Horn, 1883

= Termitopseniini Wasmann, 1916

= Schizelythrini Kemner, 1925

Tribe Trilobitideini Fauvel, 1899

Tribe WowiniJałoszyński et al., 2023

Subfamily Leptotyphlinae Fauvel, 1874

Tribe Australiotyphlini Pace, 2013

Tribe Cephalotyphlini Coiffait, 1963

Tribe Entomoculiini Coiffait, 1957

Tribe Leptotyphlini Fauvel, 1874

Tribe Metrotyphlini Coiffait, 1963

Tribe Neotyphlini Coiffait, 1963

Subfamily Oxyporinae Fleming, 1821

Subfamily Megalopsidiinae Leng, 1920

= Megalopinae Erichson, 1839 [PI]

= Aulacotrachelinae L. Benick, 1920

= Stylopodinae Blackwelder, 1943

= Megalopininae Naomi, 1986

Subfamily Steninae W.S. MacLeay, 1825

Subfamily Euaesthetinae C.G. Thomson, 1859

Tribe Alzadaesthetini Scheerpeltz, 1974

Tribe Austroesthetini Cameron, 1944

Tribe Euaesthetini C.G. Thomson, 1859

= Tamotini Coiffait, 1984

Tribe Nordenskioldiini Bernhauer & Schubert, 1911

Tribe Stenaesthetini Bernhauer & Schubert, 1911

Tribe Stictocraniini Jakobson, 1914

= Fenderiini Scheerpeltz, 1974

Subfamily Solieriinae Newton & Tayer, 1992

= Physognathinae Solier, 1849 [PI]

Subfamily Scydmaeninae Leach, 1815

Supertribe Hapsomelitae Poinar & Brown, 2004†

Supertribe Mastigitae Fleming, 1821 ^88^

Tribe Baltostigini Jałoszyński & Brunke, 2018†

Tribe Clidicini Casey, 1897

Tribe Leptochromini Jałoszyński & Brunke, 2018

Tribe Leptomastacini Casey, 1897

Tribe Mastigini Fleming, 1821

Tribe Papusini Jałoszyński & Brunke, 2018

Supertribe Cephenniitae Reitter, 1882 ^89^

Tribe Cephenniini Reitter, 1882

= Anisosphaerini Tömösváry, 1883

Tribe Eutheiini Casey, 1897

= Ascydmini Casey, 1897

= Euthiodini Casey, 1897

Tribe Marcepaniini Jałoszyński, 2013

Supertribe Scydmaenitae Leach, 1815

Tribe Chevrolatiini Reitter, 1882

Tribe Leptoscydmini Casey, 1897

Tribe Plaumanniolini Costa Lima, 1962

Tribe Scydmaenini Leach, 1815

= Eumicrini Reitter, 1882

Tribe Stenichnini Fauvel, 1885

= Cyrtoscydmini L.W. Schaufuss, 1889

= Glandulariini L.W. Schaufuss, 1889

= Euconnini Casey, 1897

= Opresini Casey, 1897

= Lophioderini Casey, 1897

= Neuraphini Csiki, 1909

= Syndicini Csiki, 1919

= Sciacharitini Csiki, 1919

= Siamitini H. Franz, 1989

Subfamily Pseudopsinae Ganglbauer, 1895

Subfamily Olisthaerinae C.G. Thomson, 1858

Subfamily Paederinae Fleming, 1821 ^90^

Tribe Lathrobiini Laporte, 1835

Subtribe Astenina Hatch, 1957

Subtribe Cylindroxystina Bierig, 1943 ^91^

Subtribe Echiasterina Casey, 1905

Subtribe Lathrobiina Laporte, 1835

Subtribe Medonina Casey, 1905

= Lithocharina Casey, 1905

= Acanthoglossina Coiffait, 1982

Subtribe Scopaeina Mulsant & Rey, 1878

Subtribe Sphaeronina Casey, 1905 ^92^

Subtribe Stilicina Casey, 1905

= Rugilina Hatch, 1957

Subtribe Stilicopsina Casey, 1905

= Xenasterina Bierig, 1939 [PI]

Tribe Paederini Fleming, 1821

Subtribe Cryptobiina Casey, 1905 ^H^

Subtribe Dicaxina Schomann & Solodovnikov, 2017

Subtribe Dolicaonina Casey, 1905

= Gnathymenina Solier, 1849

= Leptobiina Bordoni, 1980

Subtribe Paederina Fleming, 1821

= Geopaederina Gistel, 1848

Tribe Pinophilini Nordmann, 1837

Subtribe Pinophilina Nordmann, 1837

Subtribe Procirrina Bernhauer & Schubert, 1912

Subfamily Staphylininae Latreille, 1802 ^93^

Tribe Arrowinini Solodovnikov & Newton, 2005

Tribe Coomaniini Żyła & Solodovnikov, 2019

Tribe Diochini Casey, 1906

Tribe Maorothiini Assing, 2000

Tribe Othiini C.G. Thomson, 1859

= Atrecini Hatch, 1957

Tribe Platyprosopini Lynch Arribálzaga, 1884

Tribe Staphylinini Latreille, 1802

Subtribe Acylophorina Outerelo & Gamarra, 1985

Subtribe AfroquediinaBrunke et al., 2019

Subtribe Algonina Schillhammer & Brunke, 2018

Subtribe Amblyopinina Seevers, 1944

= Heterothopina Coiffait, 1978

Subtribe Anisolinina Hayashi, 1993

Subtribe AntimerinaBrunke et al., 2019

Subtribe BaltognathinaBrunke et al., 2019†

Subtribe Cyrtoquediina Brunke & Solodovnikov, 2016

Subtribe Erichsoniina Brunke & Solodovnikov, 2016 ^H^

Subtribe Hyptiomina Casey, 1906

= Holisina Newton, 1988

Subtribe Indoquediina Brunke & Solodovnikov, 2016

Subtribe Philonthina W. Kirby, 1837

= Craspedomerina Bernhauer, 1911

Subtribe Philothalpina Chatzimanolis & Brunke, 2018

Subtribe Quediina Kraatz, 1857

Subtribe Quelaestrygonini Brunke, 2021

Subtribe Staphylinina Latreille, 1802

= Creophilina W. Kirby, 1837

= Eucibdelina Sharp, 1889

= Thinopinina Böving & Craighead, 1931

= Ocypodina Hatch, 1957 ^H^

Subtribe Tanygnathinina Reitter, 1909

= Tanygnathina Casey, 1915 [PI]

= Atanygnathina Lohse, 1964

Subtribe Xanthopygina Sharp, 1884

= Platycnemina Nordmann, 1837

= Triacrina Bernhauer, 1931

Tribe Thayeralinini Solodovnikov & Yue, 2013†

Tribe Xantholinini Erichson, 1839

= Agrodini Nordmann, 1837

= Gyrohypnini W. Kirby, 1837

= Araeocnemini Casey, 1906

= Metoponcini Casey, 1906

**Staphylinoidea incertae sedis**

Hameediini M. Abdullah & Quadri, 1968

**SERIES BOSTRICHIFORMIA**

**Superfamily Bostrichoidea Latreille, 1802**

**Family Dermestidae Latreille, 1804 ^94^**

Subfamily Orphilinae J.L. LeConte, 1861

= Ranolinae Háva, 2014

Subfamily Trinodinae Casey, 1900

Tribe Cretonodini Kirejtshuk & Azar, 2009†

Tribe Thylodriini Semenov-Tjan-Shansky, 1909

Tribe Trichelodini Peacock, 1978

Tribe Trinodini Casey, 1900

= Trinoparvini Háva, 2010

Subfamily Dermestinae Latreille, 1804

Tribe Dermestini Latreille, 1804

Tribe Marioutini Jakobson, 1913

= Rhopalosilphini Arrow, 1929

Tribe Paradermestini Deng et al., 2017†

Tribe Thorictini Erichson, 1846

= Thaumaphrastini W.H. Anderson, 1949

Subfamily TrogoparvinaeZhou et al., 2022

Subfamily Attageninae Laporte, 1840

Tribe Adelaidiini Háva, 2015

Tribe Attagenini Laporte, 1840

Tribe Cretodermestini Deng et al., 2017†

Tribe Eckfeldattagenini Háva, 2015†

Attageninae incertae sedis

Apphianini Háva, 2014

Egidyellini Semenov-Tjan-Shansky, 1914

Subfamily Megatominae Leach, 1815

Tribe Anthrenini Gistel, 1848

= Dermeanthrenini Háva, 2011

Tribe Ctesiini Rees, 1943

Tribe Megatomini Leach, 1815

Subtribe Megatomina Leach, 1815

Subtribe OrphininaZhou et al., 2022

Subtribe Trogodermina Mulsant & Rey, 1867 ^95^

= Anthrenocerina Háva, 2015

**Family Bostrichidae Latreille, 1802** ^96^

Subfamily Endecatominae J.L. LeConte, 1861 ^97^

Subfamily Dysidinae Lesne, 1894

= Apoleontinae Gardner, 1933

Subfamily Polycaoninae Lesne, 1896

Subfamily Bostrichinae Latreille, 1802

Tribe Apatini Billberg, 1820

= Ligniperdini Jacobi, 1906

= Dinapatini Lesne, 1910

Tribe Bostrichini Latreille, 1802

= Bostrychopseini Lesne, 1921

= Apatidini Bradley, 1930

= Lichenophanini Portevin, 1931

Tribe Sinoxylini Marseul, 1857

Tribe Xyloperthini Lesne, 1921

Subfamily Psoinae Blanchard, 1851

Tribe Chileniini Lesne, 1921

Tribe Psoini Blanchard, 1851

Subfamily Dinoderinae C.G. Thomson, 1863

Subfamily Lyctinae Billberg, 1820

Tribe Cephalotomini L.Y. Liu, 2011

Tribe Lyctini Billberg, 1820

= Xylotrogini Schönfeldt, 1887

Tribe Trogoxylini Lesne, 1921 ^P^

= Tristariini Lesne, 1921

Subfamily Euderiinae Lesne, 1934

Subfamily AlitrepaninaePeng et al., 2022†

**Family Ptinidae Latreille, 1802**

Subfamily Eucradinae J.L. LeConte, 1861

Tribe Eucradini J.L. LeConte, 1861

Tribe Hedobiini Mulsant & Rey, 1868

Subfamily Ptininae Latreille, 1802

Tribe Ectrephini Wasmann, 1894

Tribe Gibbiini Jacquelin du Val, 1860

Tribe Gnostini King, 1866

Tribe Meziini Bellés, 1985

Tribe Ptinini Latreille, 1802

Tribe Sphaericini Portevin, 1931

Subfamily Dryophilinae Gistel, 1848

Tribe Dryophilini Gistel, 1848

= Dryobiini Gistel, 1856 ^H^

Tribe Ptilineurini Böving, 1927

Subfamily Ernobiinae Pic, 1912

Tribe Ernobiini Pic, 1912

= Cerocosmini Pic, 1912

Tribe Ochinini Zahradník, 2014

Tribe Xestobiini Böving, 1927

Subfamily Anobiinae Fleming, 1821

Subfamily Ptilininae Shuckard, 1839

Tribe Phanerochilini Zahradník, 2014

Tribe Ptilinini Shuckard, 1839

= Sclerastinae Gistel, 1856

Subfamily Alvarenganiellinae Viana & Martínez, 1971

Subfamily Xyletininae Gistel, 1848

Tribe Lasiodermini Böving, 1927

Tribe Metholcini Zahradník, 2009

Tribe Xyletinini Gistel, 1848

= Vrillettini Böving, 1927

Subfamily Dorcatominae C.G. Thomson, 1859

= Caenocarinae Böving, 1927

Subfamily Mesocoelopodinae Mulsant & Rey, 1864

Tribe Mesocoelopodini Mulsant & Rey, 1864

= Mesothetini Portevin, 1931

Tribe Tricorynini R.E. White, 1971

**Ptinidae incertae sedis**

Fabrasiini Lawrence & Reichardt, 1966

= Fabiini Martínez & Viana, 1964 [PI]

**SERIES CUCUJIFORMIA**

**Superfamily Cleroidea Latreille, 1802 ^98^**

**Family Rentoniidae Crowson, 1966**

**Family Byturidae Gistel, 1848**

Subfamily Byturinae Gistel, 1848

= Homoeoplastinae Gistel, 1856

Subfamily Platydascillinae Pic, 1914

**Family Biphyllidae J.L. LeConte, 1861**

**Family Acanthocnemidae Crowson, 1964**

**Family Protopeltidae Crowson, 1966**

**Family Peltidae W. Kirby, 1837**

= Ostomatidae Harold, 1876

**Family Lophocateridae Crowson, 1964** [NP]

= Lycoptidae Casey, 1890 [NO]

= Ancyronidae Kolibáč, 2006

= Colydiopeltidae Kolibáč, 2006

**Family Trogossitidae Latreille, 1802**

Subfamily Calityinae Houlbert, 1922

Subfamily Egoliinae Lacordaire, 1854

Subfamily Larinotinae Ślipiński, 1992

Subfamily Trogossitinae Latreille, 1802

Tribe Lithostomini Kolibáč & D.-Y. Huang, 2008†

Tribe Trogossitini Latreille, 1802

= Nemozomatini Leach, 1815

= Gymnochilini Lacordaire, 1854

= Leperinini Reitter, 1876

= Temnoscheilini Léveillé, 1888

= Tenebroidini Ganglbauer, 1899

**Family Thymalidae Léveillé, 1888**

Subfamily Thymalinae Léveillé, 1888

Subfamily Decamerinae Crowson, 1964 ^H^

**Family Phycosecidae Crowson, 1952**

**Family Prionoceridae Lacordaire, 1857** ^H^

= Lobonychidae Majer, 1987

**Family Mauroniscidae Majer, 1995**

**Family Rhadalidae J.L. LeConte, 1861**

Subfamily Gietellinae Constantin & Menier, 1987

Subfamily Rhadalinae J.L. LeConte, 1861

= Aplocneminae Mulsant & Rey, 1868

= Microjulistinae Majer, 1987

= Pelecophorinae Majer, 1987

**Family Melyridae Leach, 1815**

Subfamily Malachiinae Fleming, 1821

Tribe Amalthocini Majer, 2002

Tribe Apalochrini Mulsant & Rey, 1867

Tribe Carphurini Champion, 1923

Tribe Lemphini Wittmer, 1976

= Attalomimini Majer, 1995

Tribe Malachiini Fleming, 1821

= Tamulini Gistel, 1848

= Polycystophorini Gistel, 1856

= Troglopini Mulsant & Rey, 1867

= Attalini Abeille de Perrin, 1890

= Colotini Abeille de Perrin, 1890

= Illopini Jakobson, 1911

= Laiini Jakobson, 1911

= Ebaeini Portevin, 1931

= Paratinini Portevin, 1931

= Collopini Hatch, 1962

Tribe Pagurodactylini Constantin, 2001

Tribe Palpattalini Tshernyshev, 2020†

Malachiinae incertae sedis

Elosomatinae Jakobson, 1915

Subfamily Melyrinae Leach, 1815

Tribe Arthrobrachini Majer, 1987

Tribe Astylini Pic, 1929

Tribe Cerallini Pic, 1929

Tribe Melyrini Leach, 1815

= Zygiini Jakobson, 1911

Subfamily Dasytinae Laporte, 1840

Tribe Chaetomalachiini Majer, 1987

Tribe Danaceini C.G. Thomson, 1859

= Amauronioidini Majer, 1987

= Danacaeomimini Majer, 1987

Tribe Dasytini Laporte, 1840

= Colpothini Gistel, 1848

= Eparchiini Bernhauer, 1934

Tribe Enicopini Escalera, 1927 ^99^

Tribe Listrini Majer, 1990

**Family Phloiophilidae Kiesenwetter, 1863**

**Family Chaetosomatidae Crowson, 1952**

= Metaxinidae Kolibáč, 2004

**Family Thanerocleridae Chapin, 1924**

Subfamily Zenodosinae Kolibáč, 1992

Subfamily Thaneroclerinae Chapin, 1924

Tribe Isoclerini Kolibáč, 1992

Tribe Thaneroclerini Chapin, 1924

= Viticlerini Winkler, 1982

**Family Cleridae Latreille, 1802**

Subfamily Tillinae Fischer von Waldheim, 1813

= Cylidrinae Reitter, 1894

Subfamily Korynetinae Laporte, 1838

= Ichneinae Spinola, 1841

= Platynopterinae Spinola, 1844

= Enopliinae Gistel, 1848

= Necrobiinae Gistel, 1848

= Tarsosteninae Jacquelin du Val, 1860

= Epiphloeinae Kuwert, 1893

= Dermestoidinae Jakobson, 1911

= Orthopleurinae Böving & Craighead, 1931

= Neorthopleurinae Opitz, 2009

= Peloniinae Opitz, 2010

Subfamily EpiclininaeGunter et al., 2013

Subfamily Clerinae Latreille, 1802 ^100^

Tribe Priocerini Laporte, 1838

Tribe Nataliini Bartlett, 2021

Subtribe Cormodina Bartlett, 2021

Subtribe Nataliina Bartlett, 2021

Supertribe Opilonitae Gistel, 1848

Tribe Dieropseini J.R. Winkler, 1964 ^P^

= Trichodini Streubel, 1839 ^H^

Tribe Opilonini Gistel, 1848

Subtribe Cleropiestina J.R. Winkler, 1980

Subtribe Opilonina Gistel, 1848

= Dendroplanetina Gistel, 1856

Supertribe Cleritae Latreille, 1802

Tribe Clerini Latreille, 1802

= Thanasimini Gistel, 1848

Tribe Hydnocerini Spinola, 1844

Subtribe Anthicoclerina Opitz, 2010

Subtribe Callimerina Kolibáč, 1998

Subtribe Hydnocerina Spinola, 1844

= Phyllobaenina Lacordaire, 1857

Subtribe Lemidiina Kolibáč, 1998

**Superfamily Lymexyloidea Fleming, 1821**

**Family Lymexylidae Fleming, 1821**

Subfamily Hylecoetinae Germar, 1818

Subfamily Lymexylinae Fleming, 1821

Subfamily Atractocerinae Laporte, 1840

Subfamily Melittommatinae Wheeler, 1986

**Superfamily Tenebrionoidea Latreille, 1802**

**Family Ripiphoridae Laporte, 1840**

Subfamily Pelecotominae Guérin-Méneville, 1857

= Micholaeminae Viana, 1971

Subfamily Ptilophorinae Gerstaecker, 1855

= Evaniocerinae Lacordaire, 1859

Subfamily Hemirhipidiinae Heller, 1920

= Nephritinae Selander, 1957

Subfamily Ripidiinae Gerstaecker, 1855

Tribe Eorhipidiini Iablokoff-Khnzorian, 1986

Tribe Ripidiini Gerstaecker, 1855

Subfamily Ripiphorinae Laporte, 1840

Tribe Macrosiagonini Heyden, 1908

Tribe Ripiphorini Laporte, 1840

= Myoditini Gerstaecker, 1855

**Family Mordellidae Latreille, 1802**

Subfamily Praemordellinae Ščegoleva-Barovskaya, 1929†

Subfamily Ctenidiinae Franciscolo, 1951

Subfamily Mordellinae Latreille, 1802

Tribe Conaliini Ermisch, 1956

Tribe Curtimordini Odnosum, 2010

Tribe Mediumiugini Peris & Ruzzier, 2013†

Tribe Mordellini Latreille, 1802

Tribe Mordellistenini Ermisch, 1941

Tribe Mordellochroini Odnosum, 2010

Tribe Reynoldsiellini Franciscolo, 1957

Tribe Stenaliini Franciscolo, 1955

**Family ApotomouridaeBao et al., 2018**†

**Family Aderidae Csiki, 1909** ^101^

= Xylophilidae Shuckard, 1839 [PI]

= Hylophilidae Streubel, 1846 [PI]

= Euglenesidae Seidlitz, 1875

= Aderidae Csiki, 1909

= Cnopidae Báguena Corella, 1948

= Emelinidae Báguena Corella, 1948

= Olotelidae Báguena Corella, 1948 [PI]

= Phytobaenidae Báguena Corella, 1948

= Pseudolotelidae Báguena Corella, 1948

= Syzetoninidae Báguena Corella, 1948

= Circaeidae Iablokoff-Khnzorian, 1961†

= Gompeliidae Bouchard, 2011

**Family Ischaliidae Blair, 1920**

**Family Trictenotomidae Blanchard, 1845**

**Family Scraptiidae Gistel, 1848**

Subfamily Scraptiinae Gistel, 1848

Tribe Allopodini Franciscolo, 1964

Tribe Scraptiini Gistel, 1848

= Trotommideini Pic, 1903

Subfamily Anaspidinae Mulsant, 1856

Tribe Anaspidini Mulsant, 1856

Tribe Anaspimordini Franciscolo, 1954

Tribe Menuthianaspidini Franciscolo, 1972

Tribe Pentariini Franciscolo, 1954

**Family Mycteridae Perty, 1840**

Subfamily Mycterinae Perty, 1840

= Rhinomacrinae Fleming, 1821 [PI]

= Artaxinae Gistel, 1848

= Eutryptinae Gistel, 1856

Subfamily Eurypinae J. Thomson, 1860

= Lacconotinae J.L. LeConte, 1862

= Batobiinae Seidlitz, 1917

= Thisiinae Seidlitz, 1917

= Stilpnonotinae Borchmann, 1936

Subfamily Hemipeplinae Lacordaire, 1854

**Family Oedemeridae Latreille, 1810**

Subfamily Polypriinae Lawrence, 2005

Subfamily Calopodinae A. Costa, 1852 [NP]

= Sparedrinae Gistel, 1848 [NO]

= Catachirotetinae Gistel, 1856

Subfamily Oedemerinae Latreille, 1810

Tribe Asclerini Gistel, 1848

= Ganglbaueriini Semenov, 1894

= Hypasclerini Arnett, 1984

= Oxacidini Arnett, 1984

= Oxycopidini Arnett, 1984

Tribe Ditylini Mulsant, 1858

Tribe Nacerdini Mulsant, 1858

Tribe Oedemerini Latreille, 1810

= Oncomerini Gistel, 1856

Tribe Stenostomatini Mulsant, 1858

**Family Boridae C.G. Thomson, 1859**

Subfamily Borinae C.G. Thomson, 1859

Subfamily Synercticinae Lawrence & Pollock, 1994

**Family Pythidae Solier, 1834**

= Enoptidae Gistel, 1848

= Ischyomiidae Champion, 1886

= Osphyoplesiidae A. Winkler, 1915

= Anaplopodidae M. Abdullah, 1966

**Family Salpingidae Leach, 1815**

Subfamily Othniinae J.L. LeConte, 1861

= Elacatinae Reitter, 1879 ^H^

Subfamily Prostominiinae Grouvelle, 1914

= Trogocryptinae Lawrence, 1991

Subfamily Agleninae G.H. Horn, 1878

Subfamily Inopeplinae Grouvelle, 1908

Subfamily Dacoderinae J.L. LeConte, 1862

= Tretothoracinae Lea, 1910

Subfamily Aegialitinae J.L. LeConte, 1862

= Eurystethinae Seidlitz, 1916

Subfamily Salpinginae Leach, 1815

= Rhinosiminae Hope, 1841

= Lissodeminae Seidlitz, 1916

= Istrisiinae Nikitsky, 1992

**Family Pyrochroidae Latreille, 1806**

Subfamily Tydessinae Nikitsky, 1986

Subfamily Pilipalpinae M. Abdullah, 1964

= Techmessinae Paulus, 1972

Subfamily WaidelotinaeBukejs et al., 2019†

Subfamily Pedilinae Lacordaire, 1859

= Neopedilinae M. Abdullah, 1969

Subfamily Pyrochroinae Latreille, 1806

= Anthomanidae Gistel, 1848

Subfamily Pogonocerinae Iablokoff-Khnzorian, 1985

Subfamily Agnathinae Lacordaire, 1859

= Cononotinae J.L. LeConte, 1862

**Family Anthicidae Latreille, 1819**

Subfamily Eurygeniinae J.L. LeConte, 1862

Tribe Eurygeniini J.L. LeConte, 1862

Tribe Ictistygnini Borchmann, 1936

Tribe Mitraelabrini M. Abdullah & A. Abdullah, 1968

Subfamily Macratriinae J.L. LeConte, 1862

Tribe Camelomorphini Kirejtshuk et al., 2008†

Tribe Macratriini J.L. LeConte, 1862

Subfamily Steropinae Jacquelin du Val, 1863 ^H^

Subfamily Copobaeninae M. Abdullah, 1969

Subfamily Lemodinae E.G. Matthews, 1987

Subfamily Tomoderinae Bonadona, 1961

Subfamily Anthicinae Latreille, 1819

Tribe Anthicini Latreille, 1819

= Amblyderini Desbrochers des Loges, 1899

Tribe Endomiini Kaszab, 1956

Tribe Formicomini Bonadona, 1974

Tribe Microhoriini Bonadona, 1974

= Liparoderini Bonadona, 1990

Subfamily Notoxinae Stephens, 1829

**Family Meloidae Gyllenhal, 1810**

Subfamily Eleticinae Wellman, 1910

Tribe Derideini Wellman, 1910

Tribe Eleticini Wellman, 1910

Subtribe Eleticina Wellman, 1910

Subtribe Eospastina Selander, 1966

Tribe Ertlianini Selander, 1966

= Ertliini Kaszab, 1959 [PI]

= Morphozonitidini Kaszab, 1969

Tribe Spasticini Kaszab, 1959

Subtribe Anthicoxenina Selander, 1966

Subtribe Protomeloina M. Abdullah, 1965

Subtribe Spasticina Kaszab, 1959

Subtribe Xenospastina Selander, 1966

Subfamily Meloinae Gyllenhal, 1810

Tribe Cerocomini Leach, 1815

Tribe Epicautini J.B. Parker & Böving, 1924 ^P^

= Macrobaseini J.L. LeConte, 1862

= Apterospastini Wellman, 1910

Tribe Eupomphini J.L. LeConte, 1862

= Phodagini J.L. LeConte, 1862

= Calospastini Wellman, 1910

= Cordylospastini Wellman, 1910

= Cysteodemini Wellman, 1910

= Gynaecomeloini Wellman, 1910

= Tegroderini L.S. Dillon, 1952

= Megetrini Kaszab, 1959

Tribe Lyttini Streubel, 1846

= Sybareini Wellman, 1910

= Lydini Kaszab, 1959 ^H^

= Prolyttini Selander, 1960

Tribe Meloini Gyllenhal, 1810

= Proscarabaeini Gistel, 1848

Tribe Mylabrini Rafinesque, 1815

= Zonabrini Wellman, 1908

= Calydini Kaszab, 1960

Tribe Pyrotini MacSwain, 1956

Subfamily Tetraonycinae Böving & Craighead, 1931

= Meloetyphlinae Borgmeier, 1937

Subfamily Nemognathinae Laporte, 1840

Tribe Horiini Latreille, 1802

= Cissitini Kaszab, 1966

Tribe Nemognathini Laporte, 1840

Subtribe Nemognathina Laporte, 1840

= Horniina Dugès, 1889

= Tricraniina Wellman, 1910

Subtribe Sitarina Mulsant, 1857

= Apalina J.B. Parker & Böving, 1924

= Stenoriina J.B. Parker & Böving, 1924

Subtribe Zonitidina Mulsant, 1857

= Leptopalpina Kaszab, 1963

Tribe PalaestriniBologna et al., 2013

Tribe Stenoderini Selander, 1991

Tribe ZoltanzonitiniRiccieri et al., 2023

**Family Stenotrachelidae C.G. Thomson, 1859**

Subfamily Stenotrachelinae C.G. Thomson, 1859

Subfamily Cephaloinae J.L. LeConte, 1862

Subfamily Nematoplinae J.L. LeConte, 1862

Subfamily Stoliinae Nikitsky, 1985

**Family Tetratomidae Billberg, 1820**

Subfamily Tetratominae Billberg, 1820

Tribe ArchaeolupropiniNabozhenko et al., 2023† ^102^

Tribe Tetratomini Billberg, 1820

Subfamily Piseninae Miyatake, 1960

Subfamily Penthinae Lacordaire, 1859

Subfamily Hallomeninae Gistel, 1848

= Mycetomatinae Mulsant, 1856

Subfamily Eustrophinae Gistel, 1848

Tribe Eustrophini Gistel, 1848

Tribe Holostrophini Nikitsky, 1998

Subfamily Sphindociinae Lawrence, 1974 ^103^

**Family Melandryidae Leach, 1815**

Subfamily Melandryinae Leach, 1815

Tribe Anisoxiellini Nikitsky, 2007

Tribe Dircaeini W. Kirby, 1837

Tribe Hypulini Gistel, 1848

= Maroliini Portevin, 1934

Tribe Melandryini Leach, 1815

= Hylepnigalionini Gistel, 1856

Tribe Orchesiini Mulsant, 1856

Tribe Serropalpini Latreille, 1829

Tribe Xylitini C.G. Thomson, 1864

Tribe Zilorini Desbrochers des Loges, 1900

Subfamily Osphyinae Mulsant, 1856 (1839)

= Nothinae Shuckard, 1839

= Conopalpinae Mulsant, 1856

**Family Synchroidae Lacordaire, 1859**

= Mallodryidae G.H. Horn, 1888

**Family Prostomidae C.G. Thomson, 1859**

**Family Ciidae Leach, 1819**

Subfamily Ciinae Leach, 1819

Tribe Ciini Leach, 1819

= Ennearthrini Chûjô, 1939

Tribe Orophiini C.G. Thomson, 1863 ^H^

= Octotemnini Reitter, 1878

= Ropalodontini Everts, 1898

Subfamily Xylographellinae Kawanabe & Miyatake, 1996 ^104^

Tribe Syncosmetini Lopes-Andrade, 2008

Tribe Xylographellini Kawanabe & Miyatake, 1996

**Family Ulodidae Pascoe, 1869**

= Merycidae Crowson, 1953

**Family Archeocrypticidae Kaszab, 1964**

**Family Pterogeniidae Crowson, 1953**

**Family Mycetophagidae Leach, 1815**

Subfamily Esarcinae Reitter, 1882

Subfamily Mycetophaginae Leach, 1815

Tribe Mycetophagini Leach, 1815

= Triphyllini Crotch, 1873

= Tritomini Crotch, 1873 [PI]

Tribe Typhaeini C.G. Thomson, 1863

Subfamily Bergininae Leng, 1920

**Family Tenebrionidae Latreille, 1802** ^105^

Subfamily Lagriinae Latreille, 1825 (1820)

Tribe Adeliini W. Kirby, 1828 ^H^

Tribe Belopini Reitter, 1917

= Calcarini Mulsant, 1854 [PI]

Tribe Chaerodini Doyen et al., 1990

Tribe Cossyphini Latreille, 1802 ^H^

Tribe Eschatoporiini Blaisdell, 1906

Tribe Goniaderini Lacordaire, 1859

Tribe GonialaeniniNabozhenko et al., 2019†

Tribe Laenini Seidlitz, 1895

Tribe Lagriini Latreille, 1825 (1820)

Subtribe Lagriina Latreille, 1825 (1820)

= Lachnina Billberg, 1820 [NO]

= Loubacantina Bonadona, 1959

Subtribe Phobeliina F. Bates, 1890 ^106^

Subtribe Statirina Blanchard, 1845

Tribe Lupropini Lesne, 1926

Tribe PrateiniAalbu et al., 2023

Tribe Pycnocerini Lacordaire, 1859 [NP]

= Chiroscelidini Hope, 1841 [NO]

= Prioscelidini Skopin, 1964

Subfamily Nilioninae Oken, 1843

Subfamily Phrenapatinae Solier, 1834

Tribe Archaeoglenini Watt, 1975

Tribe Penetini Lacordaire, 1859

= Phtorini Boddy, 1965 [PI]

Tribe Phrenapatini Solier, 1834

Subfamily Zolodininae Watt, 1975

Subfamily Pimeliinae Latreille, 1802

Tribe Adelostomini Solier, 1834

= Eurychorini Hope, 1841

Tribe Adesmiini Lacordaire, 1859 [NP]

= Macropodini Erichson, 1846 [NO]

= Megageniini Solier, 1851 [NO]

= Epiphysini Lacordaire, 1859

Tribe Akidini Billberg, 1820

Tribe Anepsiini J.L. LeConte, 1862

= Batuliini G.H. Horn, 1870

Tribe Asidini Fleming, 1821

= Astrotini G.H. Horn, 1870

= Craniotini J.L. LeConte & G.H. Horn, 1883

= Machlini Chatanay, 1914

= Parecatini Chatanay, 1914

Tribe Boromorphini Skopin, 1978

Tribe Branchini J.L. LeConte, 1862

Tribe Caenocrypticini C. Koch, 1958

Tribe Ceratanisini Gebien, 1937

= Apolitini Seidlitz, 1895 [PI]

= Anisocerini Reitter, 1906 [PI]

Tribe Cnemeplatiini Jacquelin du Val, 1861

Subtribe Actizetina Watt, 1992

Subtribe AlaudinaAalbu et al., 2018^H^

Subtribe Cnemeplatiina Jacquelin du Val, 1861

Subtribe Rondoniellina Ferrer & Moragues, 2000

Subtribe Thorictosomatina Watt, 1992

Tribe Cnemodinini Gebien, 1910

= Cnemodini G.H. Horn, 1870 [PI]

Tribe Coniontini Waterhouse, 1858

= Coelini Casey, 1907

= Eusattini Casey, 1908

Tribe Cossyphodini Wasmann, 1899

Subtribe Cossyphodina Wasmann, 1899

Subtribe Cossyphoditina Basilewsky, 1950

Subtribe Esemephina Steiner, 1980

Subtribe Paramellonina Andreae, 1961

Tribe Cryptochilini Solier, 1841 ^H^

Subtribe Calognathina Lacordaire, 1859

Subtribe Cryptochilina Solier, 1841

= Horatomina C. Koch, 1955

Subtribe Homebiina Endrödy-Younga, 1989

Subtribe Vansoniina C. Koch, 1955

Tribe Cryptoglossini J.L. LeConte, 1862 [NP]

= Centriopterini Lacordaire, 1859 [NO]

Tribe Edrotini Lacordaire, 1859

= Auchmobiini J.L. LeConte & G.H. Horn, 1883

= Triorophini J.L. LeConte & G.H. Horn, 1883

= Eurymetopini Casey, 1907

= Trientomini Casey, 1907

= Trimytidini Casey, 1907

Tribe Elenophorini Solier, 1837

Tribe Epitragini Blanchard, 1845 [NP]

= Lygophilini Rafinesque, 1815 [NO]

Tribe Erodiini Billberg, 1820 [NP]

= Cephacerini Rafinesque, 1815 [NO]

Tribe Evaniosomini Lacordaire, 1859

Tribe Falsomycterini Gebien, 1910

Tribe Idisiini G.S. Medvedev, 1973

Tribe Klewariini Gebien, 1910

Tribe Lachnogyini Seidlitz, 1894

Subtribe Lachnodactylina Reitter, 1904

Subtribe Lachnogyina Seidlitz, 1894

Subtribe Netuschiliina Ferrer & Yvinec, 2004

Tribe Leptodini Lacordaire, 1859 ^H^

Tribe Megelenophorini Ferrer, 2015 ^107^

Tribe Nycteliini Solier, 1834

Tribe Nyctoporini Lacordaire, 1859 ^H^

Tribe Phrynocarenini Gebien, 1928

Tribe Physogasterini Lacordaire, 1859

Tribe Pimeliini Latreille, 1802

Subtribe Habrobatina Nabozhenko & Chigray, 2022

Subtribe Pimeliina Latreille, 1802

= Pimidiina Rafinesque, 1815

= Platyopina Semenov, 1893

= Leucolaephina Pierre, 1961

Tribe Praociini Eschscholtz, 1829

Tribe Sepidiini Eschscholtz, 1829

Subtribe Hypomelina C. Koch, 1955

Subtribe Molurina Solier, 1834

= Phanerotomina C. Koch, 1955 [PI]

= Phanerotomeina C. Koch, 1958

Subtribe Palpomodina Kamiński & Gearner, 2022

Subtribe Oxurina C. Koch, 1955

Subtribe Sepidiina Eschscholtz, 1829

Subtribe Trachynotina C. Koch, 1955

Tribe Stenosini Schaum, 1859 (1834)

Subtribe Araeoschizina Casey, 1907

Subtribe Dichillina Reitter, 1916

= Anchommatina G.H. Horn, 1878

Subtribe Harvengiina Ferrer, 2004

Subtribe Platamodina Reitter, 1900

Subtribe Stenosina Schaum, 1859 (1834)

= Tageniina Solier, 1834

Subtribe Typhlusechina Casey, 1907

Tribe Tentyriini Eschscholtz, 1831

= Gnathosiini Lacordaire, 1859

= Capnisini Casey, 1907

= Himatismini Skopin, 1979

= Pachycerini Skopin, 1979 [PI]

Tribe Thinobatini Lacordaire, 1859

Tribe Trilobocarini Lacordaire, 1859

= Salacini Casey, 1907

Tribe Vacronini Gebien, 1910

Tribe Zophosini Solier, 1834

= Dactylocalcarini Gebien, 1938

Subfamily Kuhitangiinae G.S. Medvedev, 1962

Tribe Foranotini Nabozhenko & Sadeghi, 2017

Tribe Kuhitangiini G.S. Medvedev, 1962

Subfamily Blaptinae Leach, 1815

Tribe Amphidorini J.L. LeConte, 1862

= Eleodini Blaisdell, 1909

= Eleodopseini Blaisdell, 1939

= Lariversiini La Rivers, 1948

= Trogloderini La Rivers, 1948

Tribe Blaptini Leach, 1815

Subtribe Blaptina Leach, 1815

= Dilina Ren, 2016

Subtribe Gnaptorina G.S. Medvedev, 2001

Subtribe Gnaptorinina G.S. Medvedev, 2001

Subtribe Prosodina Skopin, 1960

Subtribe Remipedellina Semenov-Tjan-Shansky, 1907

Tribe Dendarini Mulsant & Rey, 1854

Subtribe Dendarina Mulsant & Rey, 1854

= Heliopatina Mulsant & Rey, 1854

= Micrositina Mulsant & Rey, 1854

= Phylacina Lacordaire, 1859

= Olocratina Baudi di Selve, 1875

= Bioplanetina A.N. Reichardt, 1936

= Pythiopina C. Koch, 1953

Subtribe Melambiina Mulsant & Rey, 1854

= Litoborina Antoine, 1941

= Zadenina C. Koch, 1956

Tribe Opatrini Brullé, 1832

Subtribe Ammobiina Desbrochers des Loges, 1902

= Emmalina C. Koch, 1956

Subtribe Blapstinina Mulsant & Rey, 1853

Subtribe Heterotarsina Blanchard, 1845

= Heterocheirina C. Koch, 1956

Subtribe NeopachypterinaBouchard et al., 2007

= Pachypterina Mulsant & Rey, 1859 [PI]

Subtribe Opatrina Brullé, 1832

= Gonocephalina Mulsant & Revelière, 1861

Subtribe Sclerina Lacordaire, 1859

Subtribe Stenolamina C. Koch, 1956 ^108^

Subtribe Stizopodina Lacordaire, 1859

Tribe Pedinini Eschscholtz, 1830

Subtribe Helopinina Lacordaire, 1859

= Micrantereina Reitter, 1917

= Aptilina C. Koch, 1958

= Drosochrina C. Koch, 1958

= Oncosomina C. Koch, 1958

Subtribe Leichenina Mulsant, 1854

Subtribe Pedinina Eschscholtz, 1830

= Loensina C. Koch, 1956

Tribe Platynotini Mulsant & Rey, 1853

Subtribe Eurynotina Mulsant & Rey, 1854

= Oncotina C. Koch, 1953

= Schyzoschelina C. Koch, 1956

Subtribe Platynotina Mulsant & Rey, 1853

= Opatrinina Mulsant & Rey, 1853

= Trigonopodina Mulsant & Rey, 1853

= Gonopodina Lacordaire, 1859

= Anomalipodina C. Koch, 1954

Tribe Platyscelidini Lacordaire, 1859

Subfamily Tenebrioninae latreille, 1802

Tribe Acropteronini Doyen, 1989

Tribe Alphitobiini Reitter, 1917

Tribe Amarygmini Gistel, 1848

= Megacanthini Lacordaire, 1859

= Meracanthini Lacordaire, 1859

Tribe Apocryphini Lacordaire, 1859

= Diplocyrtini Escalera, 1914

Tribe Bolitophagini W. Kirby, 1837 [NP]

= Eledonini Billberg, 1820 [NO]

= Rhipidandrini J.L. LeConte, 1862

= Eutomini Lacordaire, 1865

= Heptaphyllini Prudhomme de Borre, 1886

Tribe Centronopini Doyen, 1989

Tribe Cerenopini G.H. Horn, 1870

Tribe Dissonomini G.S. Medvedev, 1968

Tribe Eulabini G.H. Horn, 1870

Tribe Falsocossyphini Ferrer, 2006

Tribe Heleini Fleming, 1821

Subtribe Asphalina E.G. Matthews & Lawrence, 2005

Subtribe Cyphaleina Lacordaire, 1859

= Nyctozoilina Lacordaire, 1859

Subtribe Heleina Fleming, 1821

= Brisina Carter, 1924

Tribe Helopini Latreille, 1802

Subtribe Cylindrinotina Español, 1956

Subtribe Enoplopodina Reitter, 1917

Subtribe Helopina Latreille, 1802

= Hypulina Rafinesque, 1815 [PI]

= Nephodina Reitter, 1917 [PI]

= Hedyphanina Reitter, 1922

Tribe Melanimonini Seidlitz, 1894 (1854)

= Microzoini Mulsant, 1854

Tribe Metaclisini Steiner, 2016

Tribe Palorini E.G. Matthews, 2003

Tribe Paoligenini Ferrer, 2013

Tribe Praeugenini De Moor, 1970

Tribe Rhysopaussini Wasmann, 1896

Tribe Scaurini Billberg, 1820

Tribe Scotobiini Solier, 1838

Tribe Tenebrionini Latreille, 1802

= Biini Skopin, 1978

Tribe Titaenini Fauvel, 1905

Tribe Toxicini Oken, 1843

Subtribe Dysantina Gebien, 1922

= EudysantinaBouchard et al., 2005

Subtribe Nycteropina Lacordaire, 1859

Subtribe Toxicina Oken, 1843

= Anthraciina Houlbert, 1922

Tribe Trachelostenini Lacordaire, 1859 ^109^

Tribe Triboliini Gistel, 1848

Tribe Ulomini Blanchard, 1845

= Alegoriini Lacordaire, 1859

= Neopsectropodini Kaszab, 1941

Subfamily Alleculinae Laporte, 1840

Tribe Alleculini Laporte, 1840

Subtribe Alleculina Laporte, 1840

= Upinellina J.L. LeConte, 1866

Subtribe Gonoderina Seidlitz, 1896

= Pseudocistelina Portevin, 1934

Subtribe Mycetocharina Gistel, 1848

Subtribe Xystropodina Solier, 1835

= Lystronychina Lacrdaire, 1859

Tribe Cteniopodini Solier, 1835

= Cistelini Latreille, 1802 [PI]

= Telacini Poey, 1854

= Phibalini Gistel, 1856

= Omophlini Mulsant, 1856

= Petriini Semenov, 1894

= Podontini Portevin, 1934

Subfamily Diaperinae Latreille, 1802

Tribe Crypticini Brullé, 1832

= Oochrotini Desbrochers des Loges, 1901

Tribe Diaperini Latreille, 1802

Subtribe Adelinina J.L. LeConte, 1862 ^P^

= Alphitophagina Gistel, 1856

= Schedarosina Reitter, 1876

= Doliemina Reitter, 1917

= Gnatocerina Skopin, 1978

Subtribe Diaperina Latreille, 1802

= Pentaphyllina Mulsant, 1854 ^H^

= Platydemina Reitter, 1917

Tribe Ectychini Doyen et al., 1990

Tribe Gnathidiini Gebien, 1921

Subtribe Anopidiina Jeannel & Paulian, 1945

Subtribe Betschiina Dajoz, 1980

Subtribe Gnathidiina Gebien, 1921

Tribe Hyociini G.S. Medvedev & Lawrence, 1982

Subtribe Brittonina G.S. Medvedev & Lawrence, 1986

Subtribe Hyociina G.S. Medvedev & Lawrence, 1982

Subtribe Uptonina G.S. Medvedev & Lawrence, 1986

Tribe Hypophlaeini Billberg, 1820

= Corticeini Boddy, 1965

Tribe Leiochrinini Lewis, 1894

Tribe Myrmechixenini Jacquelin du Val, 1858

Tribe Phaleriini Blanchard, 1845

= Sepedonastini Gistel, 1856

= Cataphronetini Reitter, 1917

Tribe Scaphidemini Reitter, 1922

Tribe Trachyscelini Blanchard, 1845

Subfamily Stenochiinae W. Kirby, 1837

Tribe Cnodalonini Oken, 1843

= Coelometopini Schaum, 1859

= Upidini C.G. Thomson, 1859

= Catapiestini Lacordaire, 1859

= Eutelini Lacordaire, 1859 [PI]

= Misolampini Lacordaire, 1859

= Polypleurini J.L. LeConte, 1862

= Misolampidiini Reitter, 1917

= Menephilini Reitter, 1920

= Hegemonini Reitter, 1922

= Stenophanini Reitter, 1922

= Camariini Anonymous, 1924

= Nodotelini C. Koch, 1950

Tribe Stenochiini W. Kirby, 1837

= Strongyliini Lacordaire, 1859

Tribe Talanini Champion, 1887 (1883)

= Dignamptini J.L. LeConte & G.H. Horn, 1883

**Tenebrionidae incertae sedis**

Ancylopomatinae Pascoe, 1871

**Family Zopheridae Solier, 1834**

Subfamily Colydiinae Billberg, 1820

Tribe Acropini Sharp, 1894

Tribe Adimerini Sharp, 1894

= Monoedini Schaeffer, 1911

Tribe Colydiini Billberg, 1820

Tribe Gempylodini Sharp, 1893

Tribe Nematidiini G.H. Horn, 1878

Tribe Orthocerini Blanchard, 1845

= Sarrotriini Billberg, 1820 ^S^

= Euglochinini Gistel, 1856

Tribe Rhagoderini G.H. Horn, 1878

Tribe Rhopalocerini Reitter, 1911

= Apistini Ganglbauer, 1899 [PI] ^S^

Tribe Synchitini L. Redtenbacher, 1845

= Bitomini Imhoff, 1856

= Endophloeini Wollaston, 1868

= Coxelini Seidlitz, 1872

= Corticini Reitter, 1882

= Langelandiini Fowler, 1889

= Megataphrini Casey, 1890

= Tarphiini Sharp, 1894

= Diodesmatini Reitter, 1911

= Trachypholidini Grouvelle, 1911

= Priolomini Dajoz, 1980

Subfamily Zopherinae Solier, 1834

Tribe Latometini Ślipiński & Lawrence, 1999

Tribe Monommatini Blanchard, 1845

Tribe Phellopsini Ślipiński & Lawrence, 1999

Tribe Pycnomerini Erichson, 1845

Tribe Usechini G.H. Horn, 1867

Tribe Zopherini Solier, 1834

= Zopheroseini Casey, 1907

Tribe ZOPHEROMIMINI Alexeev & Nabozhenko, 2023

**Family Promecheilidae Lacordaire, 1859**

= Perimylopidae St. George, 1939

= Chanopteridae Borchmann, 1915

**Family Chalcodryidae Watt, 1974**

**Family Lagrioididae M. Abdullah & A. Abdullah, 1968 ^110^**

**Tenebrionoidea incertae sedis**

Afreminae Levey, 1985

**Superfamily Coccinelloidea Latreille, 1807 ^111^**

**Family Bothrideridae Erichson, 1845**

= Deretaphridae G.H. Horn, 1878

= Dastarcidae Reitter, 1922

**Family Cerylonidae Billberg, 1820**

Subfamily Ostomopsinae Sen Gupta & Crowson, 1973

Subfamily Loeblioryloninae Ślipiński, 1990

Subfamily Ceryloninae Billberg, 1820

= Ploeosomatinae Fauvel, 1891

= Lapethinae Sharp, 1894

= Aculagnathinae Oke, 1932

= Dolosinae Dajoz, 1963

**Family Murmidiidae Jacquelin du Val, 1858** [NP]

= Ceutoceridae Mannerheim, 1852 [NO]

**Family Discolomatidae G.H. Horn, 1878**

Subfamily Notiophyginae Jakobson, 1915

Tribe Dystheamonini John, 1954

Tribe Notiophygini Jakobson, 1915

Tribe Pachyplacini John, 1954

Subfamily Discolomatinae G.H. Horn, 1878

Subfamily Aphanocephalinae Jakobson, 1904

Subfamily Cephalophaninae John, 1954

Subfamily Pondonatinae John, 1954

**Family Euxestidae Grouvelle, 1908** ^H^

= Pachyochthidae Reitter, 1911

= Metacerylonidae Heinze, 1944

= Cycloxenidae Jeannel & Paulian, 1945

= Tachyoryctidiidae Jeannel & Paulian, 1945

= Hypodacnidae Dajoz, 1976

**Family Teredidae Seidlitz, 1888**

Subfamily Teredinae Seidlitz, 1888

Tribe Sosylopsini Dajoz, 1980

Tribe Sysolini Ślipiński & Pal, 1985

Tribe Teredini Seidlitz, 1888

Subfamily Anommatinae Ganglbauer, 1899

Subfamily Xylariophilinae Pal & Lawrence, 1986

**Family Alexiidae Imhoff, 1856**

= Sphaerosomatidae Ganglbauer, 1899

**Family Akalyptoischiidae Lord et al., 2010**

**Family Latridiidae Erichson, 1842**

Subfamily Latridiinae Erichson, 1842

Subfamily Corticariinae Curtis, 1829

= Melanophthalminae Stickney, 1923

**Family Anamorphidae Strohecker, 1953** ^P^

= Symbiotidae Joy, 1932

= Mychothenidae Sasaji, 1978

= Acritosomatidae Pakaluk & Ślipiński, 1995

**Family Corylophidae J.L. LeConte, 1852**

Subfamily Periptyctinae Ślipiński et al., 2001

Subfamily Corylophinae J.L. LeConte, 1852

Tribe Aenigmaticini Casey, 1900

Tribe Cleidostethini Bowestead et al., 2001

Tribe Corylophini J.L. LeConte, 1852

Tribe FoadiiniŚlipiński et al., 2009

Tribe Orthoperini Jacquelin du Val, 1857

Tribe Parmulini Poey, 1854

= Clypeasterini Perty, 1840 [PI]

= Saciini A. Matthews, 1888

= Arthrolipini Böving & Craighead, 1931

Tribe Peltinodini Paulian, 1950

= Corylophodini Paulian, 1950

Tribe Rypobiini Paulian, 1950

= Gloeosomatini Bowestead, 1999

Tribe Sericoderini A. Matthews, 1886

Tribe StaniniRobertson et al., 2012

Tribe TeplininiPakaluk et al., 1994

= Peltinini Paulian, 1950 [PI]

Tribe Xenostanini Y.-D. Li et al., 2022

**Family Endomychidae Leach, 1815**

Subfamily Merophysiinae Seidlitz, 1872

= Holoparamecinae Seidlitz, 1888

Subfamily Pleganophorinae Jacquelin du Val, 1858

= Trochoideinae Chapuis, 1876

Subfamily Leiestinae C.G. Thomson, 1863

= Rhanidinae J.L. LeConte & G.H. Horn, 1883 [PI]

= Phymaphorinae Jakobson, 1915

Subfamily Xenomycetinae Strohecker, 1962

Subfamily Danascelinae Tomaszewska, 2000

Subfamily Cyclotominae Imhoff, 1856

Subfamily Endomychinae Leach, 1815

= Stenotarsinae Chapuis, 1876

Subfamily Epipocinae Gorham, 1873

Subfamily Lycoperdininae Bromhead, 1838

= Eumorphinae Gistel, 1848 ^H^

= Dapsinae Gerstaecker, 1858

= Corynomalinae Gorham, 1873

= Amphicinae Csiki, 1910

= Beccariinae Arrow, 1925 [PI]

= Amphisterninae Strohecker, 1964

= Mycetininae Lafer, 1992

**Family Tetrameropsidae Kirejtshuk & Azar, 2008**†

**Family Mycetaeidae Jacquelin du Val, 1857**

**Family Cerasommatidiidae Brèthes, 1925 ^112^**

**Family Eupsilobiidae Casey, 1895**

= Eidoreidae Sasaji, 1986

**Family Coccinellidae Latreille, 1807** ^113^

Subfamily Microweiseinae Leng, 1920

Tribe CarinoduliniGordon et al., 1989

Tribe *MadeiroduliniSzawaryn et al., 2020

Tribe Microweiseini Leng, 1920

Tribe Serangiini Blackwelder, 1845

Tribe Sukunahikonini Kamiya, 1960

= Scotoscymnini Duverger, 2003

Subfamily Monocoryninae Miyatake, 1988 ^H^

Subfamily Coccinellinae Latreille, 1807

Tribe Argentipilosini Gordon & de Almeida, 1991

Tribe Aspidimerini Mulsant, 1850

Tribe Cephaloscymnini Gordon, 1985

Tribe Chilocorini Mulsant, 1846

Tribe Coccidulini Mulsant, 1846

= Scymnini Mulsant, 1846

= Azyini Mulsant, 1850

= Chnoodini Mulsant, 1850

= Cranophorini Mulsant, 1850

= Poriini Mulsant, 1850

= Lithophilini Imhoff, 1856 [PI]

= Rhyzobiini C.G. Thomson, 1866

= Exoplectrini Crotch, 1874

= Scymnillini Casey, 1899

= Stethorini Dobzhansky, 1924

= Tetrabrachini Kapur, 1948

= Oryssomini Gordon, 1974

= Sumniini Hoang, 1982

= Zilini Gordon, 1985

Tribe Coccinellini Latreille, 1807

= Halyziini Mulsant, 1846

= Alesiini Mulsant, 1850

= Coelophorini Mulsant, 1850

= Cydoniini Mulsant, 1850

= Discotomini Mulsant, 1850

= Tytthaspidini Crotch, 1874

= Hippodamiini Weise, 1885

= Synonychini Weise, 1885

= Psylloborini Casey, 1899

= Anisostictini Jakobson, 1915

= Cheilomenini F.A. Schilder & M. Schilder, 1928

= Veraniini F.A. Schilder & M. Schilder, 1928 [PI]

= Anisolemniini Mader, 1954

= Adaliini Hatch, 1962

= Myziini Hatch, 1962

= Bulaeini Savoyskaya, 1969

= Singhikaliini Miyatake, 1972

Tribe Cryptognathini Mulsant, 1850 ^H^

= Oeneini Casey, 1899 [PI]

= Pentiliini Casey, 1899

Tribe Diomini Gordon, 1999

Tribe Epilachnini Mulsant, 1846

= Cynegetini C.G. Thomson, 1866

= Subcoccinellini Jakobson, 1915

= Madini Gordon, 1975

= Epivertini X.-F. Pang & Mao, 1979

= Eremochilini Gordon & Vanderberg, 1987

Tribe Hyperaspidini Mulsant, 1846 ^114^

Subtribe Brachiacanthina Mulsant, 1850

Subtribe Hyperaspidina Mulsant, 1846

Subtribe Mimoscymnina Vandenberg & Hanson, 2019

Subtribe Selvadiina Gordon, 1985

Tribe Limnichopharini Miyatake, 1994

Tribe Noviini Mulsant, 1846

Tribe Ortaliini Mulsant, 1850

Tribe Platynaspini Mulsant, 1846

Tribe Plotinini Miyatake, 1994

Tribe Shirozuellini Sasaji, 1967

= Ghaniini Ahmad, 1973

Tribe Sticholotidini Weise, 1901

= Pharini Casey, 1899 [PI]

= Clanini Weise, 1901 [PI]

= Coelopterini Weise, 1906

Tribe Telsimiini Casey, 1899

**Superfamily Erotyloidea Latreille, 1802**

**Family Boganiidae Sen Gupta & Crowson, 1966**

Subfamily Paracucujinae Endrödy-Younga & Crowson, 1986

= Athertoniinae Crowson, 1990

Subfamily Boganiinae Sen Gupta & Crowson, 1966

**Family Erotylidae Latreille, 1802**

Subfamily Cryptophilinae Casey, 1900

Tribe Cryptophilini Casey, 1900

Tribe Empocryptini Leschen, 2003

Tribe Toramini Sen Gupta, 1967

Subfamily Languriinae Hope, 1841

Tribe Hapalipini Leschen, 2003

Tribe Languriini Hope, 1841

= Cladoxenini Arrow, 1925

Tribe Thallisellini Sen Gupta, 1968

Subfamily Xenoscelinae Ganglbauer, 1899

= Eicolyctinae Vogt, 1967

= Loberonothinae Sen Gupta & Crowson, 1969

Subfamily Pharaxonothinae Crowson, 1952

= Setariinae Casey, 1900 [PI]

= Setariolinae Crowson, 1952

= Xenoscelininae Sen Gupta & Crowson, 1971

Subfamily Loberinae Bruce, 1951

Subfamily Erotylinae Latreille, 1802

Tribe Dacnini Gistel, 1848

= Cryptodacnini Sen Gupta, 1970

Tribe Encaustini Crotch, 1876

= Engini W.S. MacLeay, 1825 [PI]

Tribe Erotylini Latreille, 1802

Tribe Megalodacnini Sen Gupta, 1970

Tribe Tritomini Curtis, 1834

= Triplacini Streubel, 1839

= Renaniini Chûjô, 1941

= Cyrtotriplacini Chûjô, 1969

**Superfamily Nitiduloidea Latreille, 1802**

**Family Helotidae Chapuis, 1876** ^H^

**Family Sphindidae Jacquelin du Val, 1860**

Subfamily Libanopsinae Kirejtshuk, 2015†

Subfamily Protosphindinae Sen Gupta & Crowson, 1979

Subfamily Odontosphindinae Sen Gupta & Crowson, 1979

Subfamily Sphindiphorinae Sen Gupta & Crowson, 1979

Subfamily Sphindinae Jacquelin du Val, 1860

= Coniporinae C.G. Thomson, 1859 [PI]

= Aspidiphorinae Kiesenwetter, 1877

= Eurysphindinae Sen Gupta & Crowson, 1979

**Family Protocucujidae Crowson, 1954**

**Family Monotomidae Laporte, 1840**

Subfamily Rhizophaginae L. Redtenbacher, 1845

Subfamily Monotominae Laporte, 1840

Tribe Cretakarenniini Peris & Delclòs, 2015†

Tribe Europini Sen Gupta, 1988

Tribe Lenacini Crowson, 1952

Tribe Monotomini Laporte, 1840

Tribe Rhizophtomini Kirejtshuk & Azar, 2009†

Tribe Thionini Crowson, 1952

**Family Apophisandridae Molino-Olmedo, 2017**† ^115^

**Family Kateretidae W. Kirby, 1837**

Subfamily Kateretinae W. Kirby, 1837

= Brachypterinae Erichson, 1845

= Cercinae Desmarest, 1851

Subfamily Antirhelinae Kirejtshuk, 2023†

**Family Nitidulidae Latreille, 1802**

Subfamily Cryptarchinae C.G. Thomson, 1859

Tribe Arhinopini Kirejtshuk & Bouchard, 2018

= Arhinini Kirejtshuk, 1987 [PI]

Tribe Cryptarchini C.G. Thomson, 1859

= Pityophagini Schilsky, 1888

= Glischrochilini Chagnon, 1934

Tribe Eucalosphaerini Kirejtshuk, 1987

Tribe Platyarchini Kirejtshuk, 1998

Subfamily Prometopiinae Böving & Craighead, 1931

Subfamily Amphicrossinae Kirejtshuk, 1986

Subfamily Carpophilinae Erichson, 1842

Subfamily Cybocephalinae Jacquelin du Val, 1858

Subfamily Calonecrinae Kirejtshuk, 1982

Subfamily Epuraeinae Kirejtshuk, 1986

Tribe Epuraeini Kirejtshuk, 1986

Tribe Taenioncini Kirejtshuk, 1998

Subfamily Meligethinae C.G. Thomson, 1859

Subfamily Nitidulinae Latreille, 1802

Tribe Cychramini Gistel, 1848

Tribe Cychramptodini Kirejtshuk & Lawrence, 1992

Tribe Cyllodini Everts, 1898

= Strongylini Erichson, 1843 [PI]

= Amborotubini Leschen & Carlton, 2004

Tribe Lawrencerosini Kirejtshuk, 1991

Tribe Mystropini Murray, 1864

Tribe Nitidulini Latreille, 1802

= Thalycrini C.G. Thomson, 1859

= Pocadiini Seidlitz, 1872

= Pallodini Reitter, 1873

= Orvoeniini Dajoz, 1980

Subfamily Maynipeplinae Kirejtshuk, 1998

Subfamily Cillaeinae Kirejtshuk & Audisio, 1986

Tribe Cillaeini Kirejtshuk & Audisio, 1986

Tribe Conotelini Kirejtshuk & Kovalev, 2022

**Family Smicripidae G.H. Horn, 1880**

= Tisiphonidae Sharp, 1900 [PI]

**Nitiduloidea incertae sedis**

Meligethiellinae Kirejtshuk & Ponomarenko, 1990†

**Superfamily Cucujoidea Latreille, 1802 ^116^**

**Family Hobartiidae Sen Gupta & Crowson, 1966**

**Family Cryptophagidae W. Kirby, 1826**

= Lebanophytidae Kirejtshuk, 2013† ^117^

Subfamily Cryptophaginae W. Kirby, 1826

Tribe Caenoscelini Casey, 1900

= Sternodeini Casey, 1900

Tribe Cryptophagini W. Kirby, 1826

= Telmatophilini Jacquelin du Val, 1859

= Antherophagini J.L. LeConte, 1861

= Paramecosomatini Reitter, 1874

= Catopochrotini Reitter, 1889

= Emphylini Casey, 1900

= Spaniophaenini Casey, 1900

Tribe Picrotini Sen Gupta & Crowson, 1971

= Cryptosomatulini Leschen, 1996

Subfamily Atomariinae J.L. LeConte, 1861

Tribe Atomariini J.L. LeConte, 1861

= Ephistemini Casey, 1900

Tribe Cryptafricini Leschen, 1996

Tribe Hypocoprini Reitter, 1879

**Family Silvanidae W. Kirby, 1837**

Subfamily Brontinae Blanchard, 1845

Tribe Brontini Blanchard, 1845

= Dendrophagini Gistel, 1848

= Uleiotini Gistel, 1848

Tribe Telephanini J.L. LeConte, 1861

= Pseudophanini J.L. LeConte, 1861 ^H^

= Psammoecini Reitter, 1879

= Cryptamorphini Casey, 1884

Subfamily Silvaninae W. Kirby, 1837

**Family Cucujidae Latreille, 1802**

Subfamily Cucujinae Latreille, 1802

= Earophilinae Gistel, 1856

Subfamily Pediacinae Jin et al., 2020

Subfamily Platisinae Jin et al., 2020

**Family Phloeostichidae Reitter, 1911**

= Hymaeidae Sen Gupta & Crowson, 1966

**Family Agapythidae Sen Gupta & Crowson, 1969**

**Family Priasilphidae Crowson, 1973**

**Family Cavognathidae Sen Gupta & Crowson, 1966**

**Family Lamingtoniidae Sen Gupta & Crowson, 1969**

**Family Tasmosalpingidae Lawrence & Britton, 1991**

**Family Cyclaxyridae Gimmel et al., 2009**

**Family Passandridae Blanchard, 1845**

Subfamily MesopassandrinaeJin et al., 2019†

Subfamily Passandrinae Blanchard, 1845

= Ancistriinae Sharp, 1899

= Catogeninae Grouvelle, 1916

= Passandrellinae Grouvelle, 1916

= Scalidiinae Grouvelle, 1916

= Laemotmetinae Kessel, 1921

**Family Myraboliidae Lawrence & Britton, 1991**

**Family Phalacridae Leach, 1815**

Subfamily Phaenocephalinae A. Matthews, 1899

Subfamily Phalacrinae Leach, 1815

= Idiobiinae Gistel, 1856

= Eustilbinae Guillebeau, 1892

= Olibrinae Guillebeau, 1892

= Tolyphinae Guillebeau, 1892

= Biophytinae Guillebeau, 1894

= Megapalpinae Guillebeau, 1894 [PI]

= Ochrolitinae Guillebeau, 1894

= Stilbinae Jakobson, 1915

**Family Laemophloeidae Ganglbauer, 1899** ^P^

= Nartheciidae Casey, 1890

= Lathropidae Casey, 1916

= Propalticidae Crowson, 1952

**Cucujoidea incertae sedis**

**Sinisilvanidae Hong, 2002**†

**Superfamily Curculionoidea Latreille, 1802 ^118^**

**Family Cimberididae Gozis, 1882 ^119^**

Tribe Cimberidini Gozis, 1882

Tribe Doydirhynchini Pierce, 1916

Tribe Kuschelomacerini Riedel, 2010†

**Family Nemonychidae Bedel, 1882**

Subfamily Rhinorhynchinae Voss, 1922

Tribe Argentinomacerini Legalov, 2023

Tribe Burmomaceratini Legalov, 2020†

Tribe Idiomacerini Legalov, 2011 ^120^

Tribe Mecomacerini Kuschel, 1994

Subtribe Brarina Legalov, 2009

Subtribe Bunyaeina Legalov, 2023

Subtribe Mecomacerina Kuschel, 1994

Subtribe Rhynchitoplesiina Legalov, 2011

Tribe Rhinorhynchini Voss, 1922

Tribe Rhynchitomacrini May, 1993

Tribe Zimmiellini Legalov, 2023

Subfamily Brenthorrhininae Arnoldi, 1977†

Tribe Brenthorrhinini Arnoldi, 1977†

Subtribe Brenthorrhinina Arnoldi, 1977†

Subtribe Brenthorrhinoidina Legalov, 2003†

Tribe Distenorrhinini Arnoldi, 1977†

Tribe Juranthribini Legalov, 2011† ^121^

Subfamily Eobelinae Arnoldi, 1977†

Tribe Distenorrhinoidini Legalov, 2009†

Tribe Eobelini Arnoldi, 1977†

Tribe Karataucarini Legalov, 2009†

= Medmetrioxenoidesini Legalov, 2010†

Tribe Oxycorynoidini Arnoldi, 1977†

Tribe Probelini Legalov, 2009†

Subfamily Paleocartinae Legalov, 2003†

Tribe Oropsini Legalov & Kirejtshuk, 2017†

Tribe Paleocartini Legalov, 2003†

Subtribe Nebrenthorrhinina Legalov, 2007†

Subtribe Paleocartina Legalov, 2003†

Subfamily Metrioxenoidinae Legalov, 2009†

= Megametrioxenoidesinae Legalov, 2010†

Subfamily Cretonemonychinae Gratshev & Legalov, 2009†

Tribe Cretonemonychini Gratshev & Legalov, 2009†

Tribe Eocaenonemonychini Legalov, 2013†

Subfamily Selengarhynchinae Gratshev & Legalov, 2009†

Subfamily Slonikinae Zherikhin, 1977†

Subfamily Eccoptarthrinae Arnoldi, 1977†

= Procurculioninae Arnoldi, 1977†

= Eccoptothoracinae Arnoldi, 1977†

Subfamily Nemonychinae Bedel, 1882

**Family Anthribidae Billberg, 1820**

Subfamily Ulyaninae Zherikhin, 1993† ^122^

Subfamily Urodontinae C.G. Thomson, 1859

= Bruchelinae Pierce, 1916

Subfamily Choraginae W. Kirby, 1819

Tribe Araecerini Lacordaire, 1865

Tribe Choragini W. Kirby, 1819

Tribe Cisanthribini Zimmerman, 1994

Tribe Cretochoragini Legalov, 2016†

Tribe Homoeoderini Wollaston, 1870

Tribe Stenorhinini B.D. Valentine, 2011

Tribe Valenfriesiini Alonso-Zarazaga & Lyal, 1999

= Notioxenini Lacordaire, 1865 [PI]

Tribe Xenorchestini Lacordaire, 1865

Subfamily Anthribinae Billberg, 1820

Tribe Acanthopygini Legalov, 2023

Tribe Allandrini Pierce, 1930

Tribe Anthribini Billberg, 1820

= Brachytarsini C.G. Thomson, 1859

= Trigonorhinini B.D. Valentine, 1999 ^123^

Tribe Apolectini Lacordaire, 1865 ^H^

Tribe Basitropini Lacordaire, 1865

Tribe Corrhecerini Lacordaire, 1865

= Phaenithonini Pierce, 1930

Tribe Cratoparini J.L. LeConte, 1876

= Eupariini B.D. Valentine, 1960 ^H^

Tribe Cretanthribini Legalov, 2009†

Tribe Decataphanini Lacordaire, 1865

Tribe Discotenini Lacordaire, 1865

Tribe Ecelonerini Lacordaire, 1865

Tribe Eugonini Lacordaire, 1865

Tribe Gymnognathini B.D. Valentine, 1960

Tribe Mauiini B.D. Valentine, 1990

Tribe Mecocerini Lacordaire, 1865

= Ischnocerini Lacordaire, 1865

= Meconemini Pierce, 1930 ^H^

Tribe Mycteini Morimoto, 1972

Tribe Oiserhinini Legalov et al., 2020†

Tribe Ozotomerini Morimoto, 1972

Tribe Phloeophilini Lacordaire, 1865 ^124^

= Cappadocini Alonso-Zarazaga & Lyal, 1999

Tribe Phloeotragini Kolbe, 1897

Tribe Piesocorynini B.D. Valentine, 1960

Tribe Platystomini Pierce, 1916

= Anthotribini Gistel, 1856 [PI]

Tribe Proscoporhinini Lacordaire, 1865

= Jordanthribini Morimoto, 1980 ^125^

Tribe Ptychoderini Jekel, 1855 ^H^

Tribe Sintorini Lacordaire, 1865

Tribe Stenocerini Kolbe, 1895

Tribe Tophoderini Lacordaire, 1865

Tribe Tropiderini Lacordaire, 1865

= Acorynini Lacordaire, 1865

= Eurymycterini Pierce, 1930

Tribe Xenocerini Lacordaire, 1865

Tribe Xylinadini Lacordaire, 1865

Tribe Zygaenodini Lacordaire, 1865

= Ormiscini J.L. LeConte, 1876

= Platyrhinini Bedel, 1882 ^H, 126^

= Nessiarini Morimoto, 1972

**Family Belidae Schönherr, 1826**

Subfamily Montsecbelinae Legalov, 2016†

Tribe Gratshevbelini Legalov, 2016†

Tribe Montsecbelini Legalov, 2016†

Subfamily Oxycoryninae Schönherr, 1840

Supertribe Oxycorynitae Schönherr, 1840

Tribe Afrocorynini Voss, 1957

Tribe Allocorynini Sharp, 1890

Tribe Archicorynini R.S. Anderson & Marvaldi, 2013

Tribe Hispodini Voss, 1957

Tribe Metrioxenini Voss, 1953

Subtribe Metrioxenina Voss, 1953

Subtribe Pseudometrioxenina Legalov, 2023

Subtribe Zherichinixenina Legalov, 2009

Tribe Oxycorynini Schönherr, 1840

Tribe Oxycraspedini Marvaldi & Oberprieler, 2006

Tribe Palaeorhopalotriini Legalov, 2013†

Supertribe Aglycyderitae Wollaston, 1864

Tribe Aglycyderini Wollaston, 1864

= Proterhinini Fauvel, 1891

= Platycephalini Paulian, 1944 [PI] ^H^

Tribe Alloxycorynini Legalov, 2009

Subfamily Belinae Schönherr, 1826

Supertribe Pachyuritae Kuschel, 1959

Tribe Agnesiotidini Zimmerman, 1994

Tribe Pachyurini Kuschel, 1959

Supertribe Belitae Schönherr, 1826

Tribe Belini Schönherr, 1826

Subtribe Belina Schönherr, 1826

Subtribe Homalocerina Legalov, 2009

Tribe Davidibelini Legalov, 2016†

**Family Mesophyletidae Poinar, 2006**† ^127^

Subfamily Aepyceratinae Poinar et al., 2017†

Subfamily Mesophyletinae Poinar, 2006†

= Anchineinae Poinar & Legalov, 2014†

= Mekorhamphinae Poinar et al., 2016†

= Burmocoryninae Legalov, 2018†

**Family Attelabidae Billberg, 1820**

Subfamily Attelabinae Billberg, 1820

Tribe Attelabini Billberg, 1820

Subtribe Attelabina Billberg, 1820

Subtribe Euscelina Voss, 1925

= Alleuscelina Legalov, 2003

= Clinolabina Legalov, 2003

Subtribe Euscelophilina Voss, 1925

Subtribe Henicolabina Legalov, 2007

Subtribe Himatolabina Legalov, 2003

Subtribe Hybolabina Voss, 1925

Subtribe Isolabina Legalov, 2007

Subtribe Lagenoderina Voss, 1925

Subtribe Lamprolabina Voss, 1925

Subtribe Metocalolabina Legalov, 2003

Subtribe Omolabina Legalov, 2003

Subtribe Paramecolabina Legalov, 2003

Subtribe Phialodina Legalov, 2003

Subtribe Phymatolabina Voss, 1925

Subtribe Phymatopsinina Legalov, 2003

Subtribe Pleurolabina Legalov, 2003

Tribe Euopini Voss, 1925

= Archeuopsini Legalov, 2003

= Suniopsini Legalov, 2003

= Synaptopsini Legalov, 2003

= Ljudmilinini Legalov, 2007

= Parasynaptopsisini Legalov, 2007

= Riedelinini Legalov, 2007

= Sawadaeuopsini Legalov, 2007

Tribe Pilolabini Voss, 1925

Subfamily Apoderinae Jekel, 1860

Tribe Apoderini Jekel, 1860

= Anisonychini Legalov, 2003 ^H^

= Centrocorynini Legalov, 2003

= Cycnotrachelini Legalov, 2003

= Opanassenkoviini Legalov, 2003

= Pseudocycnotrachelini Legalov, 2003

Tribe Clitostylini Voss, 1929

Subtribe Allapoderina Legalov, 2003

Subtribe Clitostylina Voss, 1929

Subtribe Pseudophrysina Legalov, 2003

Tribe Hoplapoderini Voss, 1926

Subtribe Afroapoderina Legalov, 2003

Subtribe Hoplapoderina Voss, 1926

Subtribe Paratomapoderina Legalov, 2003

Tribe Trachelophorini Voss, 1926

Subfamily Sayrevilleinae Legalov, 2003 ^128^

Tribe Minurini Legalov, 2003

Tribe Sayrevilleini Legalov, 2003†

Tribe Vossicartini Legalov, 2003

Subfamily Rhynchitinae Gistel, 1848

Supertribe Rhinocartitae Voss, 1931

Tribe Parauletanini Legalov, 2007

Tribe Philippinauletini Legalov, 2023

Tribe Rhinocartini Voss, 1931

= Proteugnamptini Legalov, 2003

Supertribe Rhynchititae Gistel, 1848

Tribe Auletini Desbrochers des Loges, 1908

Subtribe Auletina Desbrochers des Loges, 1908

Subtribe Auletobiina Voss, 1930

= Auletorhinina Voss, 1935

= Auletanini Legalov, 2003

= Guineauletina Legalov, 2003 ^129^

Subtribe Mandelschtamiina Legalov, 2003

= Eosalacina Legalov, 2007 ^130^

Subtribe Pseudauletina Voss, 1933

Subtribe Pseudomesauletina Legalov, 2003

Tribe Byctiscini Voss, 1923

Subtribe Byctiscina Voss, 1923

Subtribe Listrobyctiscina Legalov, 2003

Subtribe Svetlanaebyctiscina Legalov, 2003

Tribe Cesauletini Legalov, 2003

Tribe Deporaini Voss, 1929

= Chonostropheini Morimoto, 1962 ^131^

= Depasophilini Legalov, 2003

Tribe Eugnamptini Voss, 1930

= Acritorrhynchitini Legalov, 2007 ^132^

Tribe Rhynchitini Gistel, 1848

Subtribe Lasiorhynchitina Legalov, 2003

Subtribe Perrhynchitina Legalov, 2003

Subtribe Rhynchitina Gistel, 1848

= Isotheina Scudder, 1893†

= Rhynchitallina Legalov, 2003 ^133^

Subtribe Temnocerina Legalov, 2003

= Anisomerinina Legalov, 2003 ^134^

Subfamily Pterocolinae Lacordaire, 1865

**Family Caridae Thompson, 1992**

Subfamily Baissorhynchinae Zherikhin, 1993† ^P^

Tribe Abrocarini Legalov, 2009†

Tribe Baissorhynchini Zherikhin, 1993†

Tribe Gobicarini Legalov, 2009†

Tribe Mongolobrenthorrhinini Gratshev & Legalov, 2011†

Tribe Nanophydini Arnoldi, 1977†

Subfamily Chilecarinae Legalov, 2009

Tribe Carodesini Legalov, 2009

Tribe Chilecarini Legalov, 2009

Tribe Cretuliini Legalov, 2009†

Subfamily Carinae Thompson, 1992

**Family Brentidae Billberg, 1820**

Subfamily Ithycerinae Schönherr, 1823

Tribe Ithycerini Schönherr, 1823

= Pachyrhynchini W. Kirby, 1837 [PI]

Tribe Mongolocarini Gratshev & Legalov, 2011†

Tribe Montsecanomalini Legalov, 2016†

Tribe Ulyaniscini Legalov, 2009†

Subfamily Brentinae Billberg, 1820

Tribe Brentini Billberg, 1820

Subtribe Arrenodina Lacordaire, 1865 ^M^

= Belopherina Lacordaire, 1865

= Belorhynchina Lacordaire, 1865

= Eutrachelina Lacordaire, 1865

= Orychodina Senna, 1895

Subtribe Brentina Billberg, 1820

Subtribe Eremoxenina Semenov, 1892

= Amorphocephalina Power, 1879 [PI]

Subtribe Ithystenina Lacordaire, 1865

= Ischnomerina Kolbe, 1883

= Leptorhynchina Schoenfeldt, 1908 [PI]

= Ozodecerina Jakobson, 1911

= Acratina Alonso-Zarazaga et al., 1999 ^135^

Subtribe Paussobrenthina Gestro, 1919

Subtribe Tychaeina Schoenfeldt, 1908

Tribe Cyladini Schönherr, 1823

Subtribe Cyladina Schönherr, 1823

Subtribe Miocenocyladina Legalov, 2018†

Tribe Cyphagogini Kolbe, 1892

= Calodromini Kleine, 1922

Tribe Taphroderini Lacordaire, 1865

= Ischnomerini Lacordaire, 1865 [PI]

= Dominibrentini Poinar, 2009†

Tribe Trachelizini Lacordaire, 1865

Subtribe Atopobrentina Damoiseau, 1965

Subtribe Hephebocerina Lacordaire, 1865

= Anchisteina Schedl, 1961

Subtribe Hoplopisthiina Senna & Calabresi, 1919

Subtribe Microtrachelizina Zimmerman, 1994

Subtribe Pseudoceocephalina Kleine, 1922 ^P^

= Uropterina Jakobson, 1911

Subtribe Rhyticephalina Kleine, 1922

Subtribe Stereodermina Sharp, 1895

Subtribe Trachelizina Lacordaire, 1865

Tribe Ulocerini Schönherr, 1823

Subtribe Pholidochlamydina Damoiseau, 1962

Subtribe Ulocerina Schönherr, 1823

Subfamily Microcerinae Lacordaire, 1863

Subfamily Eurhynchinae Lacordaire, 1863

Tribe Axelrodiellini Legalov, 2009†

Tribe Eurhynchini Lacordaire, 1863

= Eurhinini Kissinger, 1968 [PI]

Subfamily Apioninae Schönherr, 1823

Supertribe Tanaitae Schönherr, 1839

Tribe Apiomorphini Legalov, 2023

Tribe Palaeotanaini Legalov et al., 2020†

Tribe Tanaini Schönherr, 1839

Supertribe Mecolenitae Wanat, 2001

Supertribe Chilapiitae Wanat, 2001

Supertribe Cybebitae Lacordaire, 1863

Supertribe Myrmacicelitae Zimmerman, 1994

Tribe Lispotheriini Wanat, 2001

Tribe Myrmacicelini Zimmerman, 1994

Supertribe Rhadinocybitae Alonso-Zarazaga, 1992

Tribe Notapionini Zimmerman, 1994

Tribe Rhadinocybini Alonso-Zarazaga, 1992

Supertribe Noterapionitae Kissinger, 2004

Supertribe Rhinorhynchidiitae Zimmerman, 1994

Supertribe Podapiitae Wanat, 2001

Supertribe Setapiitae Legalov, 2023

Supertribe Aspidapiitae Alonso-Zarazaga, 1990

Tribe Aspidapiini Alonso-Zarazaga, 1990

Tribe Ceratapiini Alonso-Zarazaga, 1990

Tribe Kalcapiini Alonso-Zarazaga, 1990

Tribe Malvapiini Alonso-Zarazaga, 1990

Tribe Metapiini Alonso-Zarazaga, 1990

Tribe Prototrichapiini Wanat, 1995

Supertribe Apionitae Schönherr, 1823

Tribe Apionini Schönherr, 1823

Subtribe Apionina Schönherr, 1823

= Oxeostomina Gistel, 1856

Subtribe Stenapiina Poinar & Legalov, 2014

Subtribe Synapiina Alonso-Zarazaga, 1990

= Trichapiina Alonso-Zarazaga, 1990 ^136^

Subtribe Toxorhynchina Scudder, 1893

= Oxystomatina Alonso-Zarazaga, 1990 ^137^

Tribe Aplemonini Kissinger, 1968

Tribe Catapiini Alonso-Zarazaga, 1990

Tribe Exapiini Alonso-Zarazaga, 1990

Tribe Ixapiini Alonso-Zarazaga, 1990

Tribe Piezotrachelini Voss, 1959

Supertribe Antliarhinitae Schönherr, 1823

Apioninae incertae sedis ^138^

Apodytapiini Wanat, 2021

Lepanomini Wanat, 2021

Rhynchitapiini Wanat, 2021

Subfamily Nanophyinae Gistel, 1848

Tribe Corimaliini Alonso-Zarazaga, 1989

Tribe Nanophyini Gistel, 1848

**Family Curculionidae Latreille, 1802 ^139^**

Subfamily Brachycerinae Billberg, 1820

Tribe Brachycerini Billberg, 1820

= Cryptopini Schönherr, 1826 [PI]

= Byrsopini Germar, 1829

= Protomantiini Aurivillius, 1886

= Brotheini G.A.K. Marshall, 1907 ^H^

Tribe Cryptolaryngini Schalkwyk, 1966

= Cryptopharyngini G.A.K. Marshall, 1957 [PI]

= Periegini Legalov, 2003

Tribe Erirhinini Schönherr, 1825

= Ocladiini Lacordaire, 1865

= Arthrostenini Reitter, 1913

= Notodermini Voss, 1952

= Desmidophorini Morimoto, 1962

= Aonychini Zimmerman, 1993 ^H^

= Tadiini Zimmerman, 1993

= Echinocnemini Legalov, 2020

Tribe Himasthlophallini Zherikhin, 1991

Tribe Myrtonymini Kuschel, 1990

Tribe Raymondionymini Reitter, 1913

Tribe Tanysphyrini Gistel, 1848

= Brachypodini J.L. LeConte, 1876 [PI]

= Stenopelmini J.L. LeConte, 1876

= Lissorhoptrini Böving & Craighead, 1931

Subfamily Dryophthorinae Schönherr, 1825 ^140^

Tribe Cryptodermatini Bovie, 1908

= Oxyrhynchini Schönherr, 1823 [PI]

Tribe Dryophthorini Schönherr, 1825

Tribe Litosomini Lacordaire, 1865 ^H^

Subtribe Crepidotina Legalov, 2020

Subtribe Diocalandrina Zimmerman, 1993

Subtribe Laogenina Legalov, 2020

Subtribe Litosomina Lacordaire, 1865 ^H^

Subtribe Polytina Zimmerman, 1993

Subtribe Sitophilina Csiki, 1936

= Calandrina C.G. Thomson, 1859 [PI]

Tribe Ommatolampini Lacordaire, 1865

Tribe Orthognathini Lacordaire, 1865

= Sipalini Lacordaire, 1865 [PI]

= Sipalinini Zimmerman, 1993

Tribe Rhinostomini Kuschel, 1995

= Rhinini J.L. LeConte, 1874 [PI]

Tribe Rhynchophorini Schönherr, 1833

Tribe Sipalomimini Legalov, 2020

Tribe Sphenophorini Lacordaire, 1865

Subtribe Sphenocorynina Lacordaire, 1865

Subtribe Sphenophorina Lacordaire, 1865

= Calandrina Billberg, 1820 [PI]

Tribe Stromboscerini Lacordaire, 1865

Subfamily Platypodinae Shuckard, 1839

Tribe Mecopelmini Tompson, 1992

Tribe Platypodini Shuckard, 1839

= Crossotarsini H. Strohmeyer, 1914

Tribe Schedlariini Wood & Bright, 1992

= Chapuisiini Blandford, 1895 [PI]

Tribe Tesserocerini H. Strohmeyer, 1914

Subtribe Diapodina H. Strohmeyer, 1914

= Genyocerina Hopkins, 1915

Subtribe Tesserocerina H. Strohmeyer, 1914

= Cenocephalina H. Strohmeyer, 1914

= Symmerina H. Strohmeyer, 1914 [PI]

= Periommatina Schedl, 1939

= Platytarsulina Schedl, 1939

= Platypicerina Nunberg, 1953

Subfamily Bagoinae C.G. Thomson, 1859 [NP]

= Lyprinae Gistel, 1848 [NO]

= Hydronominae Lacordaire, 1863

= Pseudobagoinae Sharp, 1917

Subfamily Hyperinae Lacordaire, 1863 (1848)

Tribe Gonipterini Lacordaire, 1863

Tribe Hyperini Lacordaire, 1863 (1848)

Subtribe Cepurina Capiomont, 1867

Subtribe Coniatina Legalov, 2007

Subtribe Hyperina Lacordaire, 1863 (1848)

= Phytonomina Gistel, 1848

Subtribe Macrotarrhusina Legalov, 2007

Subtribe Phaeopholina Legalov, 2011

Subfamily Entiminae Schönherr, 1823 ^141^

Supertribe Cylydrorhinitae Lacordaire, 1863

Tribe Cylydrorhinini Lacordaire, 1863

Supertribe Entimitae Schönherr, 1823

Tribe Anomophthalmini Morrone, 1998

Tribe Byrsopagini Lacordaire, 1863 ^142^

Subtribe Byrsopagina Lacordaire, 1863

= Dirotognathina G.H. Horn, 1876

Subtribe Phyxelidina G.H. Horn, 1876

Subtribe Strangaliodina Lacordaire, 1863

= Pantopoeina Lacordaire, 1863

= Synaptonychina Lacordaire, 1863

= Alophina J.L. LeConte, 1874

= Dyslobina J.L. LeConte, 1874

= Rhigopseina J.L. LeConte, 1874

= Stenocorynina McKeown, 1939

= Leptosina G.A.K. Marshall, 1952

Subtribe Tropiphorina Marseul, 1863

= Synirmina Bedel, 1883

Tribe Entimini Schönherr, 1823

Subtribe Entimina Schönherr, 1823

Subtribe Rhigiina Legalov, 2020

Tribe Eudiagogini J.L. LeConte, 1874 ^P^

= Promecopini Lacordaire, 1863

= Leptopini Lacordaire, 1863 [PI]

= Bathyrinini J.L. LeConte, 1874

= Colecerini Jekel, 1875

= Leptopiini Oke, 1951 ^143^

Tribe Eurylobini Jekel, 1856

Tribe Leptostethini Lacordaire, 1863

Tribe Lordopini Schönherr, 1823

= Hypsonotini Jekel, 1853

= Alocorhinini Jekel, 1856

= Elytroxeini Jekel, 1856

= Merodontini Jekel, 1856

= Tomorhinini Jekel, 1856

Tribe Ophryastini Lacordaire, 1863

Subtribe Deracanthina Legalov, 2020 ^H^

Subtribe Ophryastina Lacordaire, 1863

Tribe Premnotrypini Kuschel, 1956

Tribe Tanyrhynchini Schönherr, 1826

= Eremnini Lacordaire, 1863

Supertribe Otiorhynchitae Schönherr, 1826

Tribe Agraphini G.H. Horn, 1876

Tribe Celeuthetini Lacordaire, 1863

Subtribe Celeuthetina Lacordaire, 1863

= Platyspartina Voss, 1940

Subtribe Isopterina Morimoto & Kojima, 2001

Tribe Ectemnorhinini Lacordaire, 1863 ^144^

= Canonopseini Dreux & Voisin, 1989

= DiskeriniVoisin et al., 2017

Tribe Elytrurini G.A.K. Marshall, 1956

Tribe Episomini Lacordaire, 1863

Tribe Holcorhinini Desbrochers des Loges, 1898

= Cyclopterini Reitter, 1913 [PI]

Tribe Hormorini G.H. Horn, 1876

Tribe Isanirini Legalov, 2020

Tribe Laparocerini Lacordaire, 1863

Tribe Mesostyloidini Bouchard & Bousquet, 2020

= Mesostylini Reitter, 1913 [PI]

Tribe Myorhinini Marseul, 1863 ^H^

Tribe Nastini Reitter, 1913

Tribe Nothognathini G.A.K. Marshall, 1916

Tribe Otiorhynchini Schönherr, 1826

= Loborhynchini Schönherr, 1823 [PI] ^S^

= Brachyrhinini Bedel, 1883 [PI] ^S^

Tribe Phyllobiini Schönherr, 1826

= Aphrastini J.L. LeConte, 1874

Tribe Polycatini G.A.K. Marshall, 1956

Tribe Rhyncogonini Sharp, 1919

Tribe Typhlorhinini Kuschel, 1954

Supertribe Cyphiceritae Lacordaire, 1863

Tribe Cyphicerini Lacordaire, 1863

Subtribe Acanthotrachelina G.A.K. Marshall, 1944

Subtribe Cyphicerina Lacordaire, 1863

= Corigetina Faust, 1885

Subtribe Mylacorrhinina Reitter, 1913 ^145^

Subtribe Myllocerina Pierce, 1913

= Ptochina Reitter, 1913

Subtribe Phytoscaphina Lacordaire, 1863

Tribe Embrithini G.A.K. Marshall, 1942

Tribe Metacinopini Reitter, 1913

= Auchmeresthetini Reitter, 1913

Tribe Omiini Shuckard, 1839 ^H^

= Barypeithini Pic, 1897

= Mylacini Reitter, 1913

Tribe Oosomini Lacordaire, 1863

Tribe Peritelini Lacordaire, 1863

Subtribe Peritelina Lacordaire, 1863

= Paraptochina Pierce, 1913

Subtribe Simonina Pierce, 1913

= Homorhythmina Hoffmann, 1950

Tribe Sciaphilini Sharp, 1891

Tribe Scythropini Lacordaire, 1863

Tribe Trachyphloeini Gistel, 1848

Subtribe Pseudocneorhinina Kôno, 1930

Subtribe Trachyphilina Voss, 1948

Subtribe Trachyphloeina Gistel, 1848

= Phyllastolina Gistel, 1856

= Cathormiocerina Reitter, 1913

Supertribe Polydrusitae Schönherr, 1823

Tribe Anypotactini Champion, 1911

Tribe Blosyrini Lacordaire, 1863

Tribe Brachyderini Schönherr, 1826

= Thylacitini W. Kirby, 1837 [PI] ^S^

= Strophosomatini Gistel, 1848

Tribe Cneorhinini Lacordaire, 1863

= Philopedini Bedel, 1883

= Callirhopalini Voss, 1956 ^146^

Tribe Cratopodini Hustache, 1919

Tribe Dermatodini Emden, 1936

= Ophtalmorrhynchini Hoffmann, 1965

= Stigmatrachelini Richard, 1983

Tribe Eupholini Günther, 1943

Tribe Eustylini Lacordaire, 1863

Subtribe Brachycamacina Poinar et al., 2013†

Subtribe Eustylina Lacordaire, 1863

= Exophthalmina G.H. Horn, 1876

= Omileina G.H. Horn, 1876

= Compsina Pierce, 1913 ^H^

= Menoetiina Pierce, 1913 [PI]

= Exophthalmodina Voss, 1954

Tribe Evotini J.L. LeConte, 1874

Tribe Geonemini Gistel, 1848

= Barynotini Lacordaire, 1863

= Epicaerini G.H. Horn, 1876

Tribe Naupactini Gistel, 1848 [NP]

Subtribe Mesagroicina Legalov, 2020

Subtribe Naupactina Gistel, 1848 [NP]

= Iphiina Schönherr, 1823 [NO]

= Cyphina Lacordaire, 1863

= Macrostylina J.L. LeConte, 1874

= Symmathetina J.L. LeConte, 1874

= Artipodina G.H. Horn, 1876

= Alceentina Pierce, 1913

= Glaphyrometopina Pierce, 1913

= Pseudocyphina Pierce, 1913

= Neocyphina Hustache, 1919

= Canephorotomina Voss, 1954

= Pantomorina Voss, 1954

= Plectrophorina Voss, 1954 [PI]

Subtribe Platyomina Champion, 1911

Tribe Ottistirini Heller, 1925

Tribe Pachyrhynchini Schönherr, 1826

= Somatodini Schönherr, 1823 [PI] ^S^

Tribe Polydrusini Schönherr, 1823

= Liophloeini Gistel, 1848

= Phyllomanini Gistel, 1848 [PI]

= Oligoceini Gistel, 1856

Tribe Prypnini Lacordaire, 1863

Tribe Psallidiini Lacordaire, 1863

= Trigonoscutini J.L. LeConte, 1874

= Calyptillini G.H. Horn, 1876

Tribe Sitonini Gistel, 1848

Tribe Tanymecini Lacordaire, 1863

Subtribe Pandeleteiina Pierce, 1913

Subtribe Piazomiina Reitter, 1913

Subtribe Tainophthalmina Desbrochers des Loges, 1873

= Amomphina J.L. LeConte, 1874

Subtribe Tanymecina Lacordaire, 1863

= Hadromerina Jekel, 1865 [PI]

= Siderodactylina Jekel, 1865

= Pachnaeina J.L. LeConte, 1874

= Minyomerina G.H. Horn, 1876

= Cycloderina Hoffmann, 1950

Entiminae incertae sedis

Namaini Borovec & Meregalli, 2021

Pristorhynchini Heer, 1847†

Subfamily Cyclominae Schönherr, 1826

Tribe Amycterini G.R. Waterhouse, 1854

= Euomini Lacordaire, 1863

= Phalidurini Pierce, 1914

Tribe Aterpini Lacordaire, 1863

Subtribe Aterpina Lacordaire, 1863 [NP]

= Heliomenina Gistel, 1848 [NO]

= Lophotina Jekel, 1865 [PI]

= Rhinariina Jekel, 1865

Subtribe Rhadinosomina Lacordaire, 1863

Tribe Cyclomini Schönherr, 1826

= Somatodini Lacordaire, 1863

Tribe Dichotrachelini Hoffmann, 1957

Tribe Hipporhinini Lacordaire, 1863

= Gronopini Bedel, 1884

Tribe Listroderini J.L. LeConte, 1876 ^147^

Subtribe Falklandiina Morrone, 2013

Subtribe Listroderina J.L. LeConte, 1876

Subtribe Macrostyphlina Morrone, 2013

Subtribe Palaechthina C. Brinck, 1948

Tribe Notiomimetini Wollaston, 1873 ^148^

= Aphelini Csiki, 1936

Tribe Rhythirrinini Lacordaire, 1863

= Eupagini Lacordaire, 1863

= Rhyparosomini Lacordaire, 1863

= Rhytidorhinini Wollaston, 1865 [PI]

Subfamily Curculioninae Latreille, 1802

Tribe Acalyptini C.G. Thomson, 1859 ^149^

Tribe Acentrini Seidlitz, 1890

Tribe Ancylocnemidini Voss, 1962

Tribe Anthonomini C.G. Thomson, 1859

= Loncophorini Pierce, 1916

= Bradybatini Pierce, 1919 ^H^

= Brachonychini Joy, 1932

= Tachypterellini Voss, 1944

Tribe Camarotini Schönherr, 1833

Subtribe Camarotina Schönherr, 1833

Subtribe Prionomerina Lacordaire, 1863

Tribe Ceratopodini Lacordaire, 1863

Tribe Cionini Schönherr, 1825

Tribe Cranopoeini Kuschel, 2009

Tribe Cryptoplini Lacordaire, 1863

= Haplonychini Lacordaire, 1865

Tribe Curculionini Latreille, 1802

Subtribe Balanobiina Prena, 2021

= Archariina Pelsue & O’Brien, 2011 [PI]

Subtribe Curculionina Latreille, 1802

= Balaninina Gistel, 1848

Subtribe Erganiina Pelsue & O’Brien, 2011

Subtribe Labaninina Pelsue & O’Brien, 2011

Subtribe Pseudobalaninina Heller, 1925

Subtribe Timolina Heller, 1925

Tribe DerelominI Lacordaire, 1865

Subtribe Derelomina Lacordaire, 1865

= Hoplorhinina Champion, 1903

= Celetina Voss, 1954

Subtribe Notolomina N.M. Franz, 2006

Subtribe Phyllotrogina N.M. Franz, 2006

Subtribe Staminodeina N.M. Franz, 2006

Tribe Ellescini C.G. Thomson, 1859

= Dorytomini Bedel, 1886

Tribe Erodiscini Lacordaire, 1863 ^M^

Tribe Eugnomini Lacordaire, 1863

Subtribe Eugnomina Lacordaire, 1863

= Scolopterina Lacordaire, 1863

= Pactolina Voss, 1936

= Stephanorhynchina Voss, 1936

Subtribe Meriphina G.A.K. Marshall, 1937

Tribe Mecinini Gistel, 1848

= Gymnetrini C.G. Thomson, 1859 ^H^

= Miarini Tournier, 1874

Tribe Microstylini Lacordaire, 1865

= Archolabini Voss, 1929

= Galloisiini Morimoto, 1962 ^150^

Tribe Nerthopini Lacordaire, 1865

= Acallopistini Lacordaire, 1865

Tribe Ochyromerini Voss, 1935 ^151^

= Endaeini Voss, 1958

Tribe Otidocephalini Lacordaire, 1863

= Myrmecini Tanner, 1966

Tribe Palaeoanoplini Legalov, 2021†

Tribe Piazorhinini Lacordaire, 1863

Tribe Prionobrachiini Hustache, 1938

Tribe Pyropini Lacordaire, 1865

Tribe Rhamphini Rafinesque, 1815

Subtribe Dinorhopalina Voss, 1936

Subtribe Ixalmina Voss, 1936

Subtribe Palaeorhamphina Legalov, 2017†

Subtribe Rhamphina Rafinesque, 1815

= Orchestina Latreille, 1828

= Rhynchaenina Blanchard, 1853

Subtribe Tachygonina Lacordaire, 1865

Tribe Smicronychini Seidlitz, 1891 [NP]

= Desmorinini J.L. LeConte, 1876 [NO]

Tribe Sphaeriopoeini Kuschel, 2003

Tribe Storeini Lacordaire, 1863

= Hybomorphini Lacordaire, 1865

Tribe Styphlini Jekel, 1861

= Orthochaetini Joy, 1932

Tribe Thecesternini Lacordaire, 1863 ^152^

Tribe Tychiini Gistel, 1848

Subtribe Demimaeina Voss, 1937

Subtribe Eocenesibiniina Legalov, 2017†

Subtribe Lignyodina Bedel, 1883

Subtribe Tychiina Gistel, 1848

= Miccotrogina Gistel, 1856

= Sibiniina Marseul, 1863

Tribe Ulomascini Lacordaire, 1865

Tribe Viticiini Morimoto, 1983

Subfamily Baridinae Schönherr, 1836

Tribe Ambatini Lacordaire, 1863

Tribe Apostasimerini Schönherr, 1844

Subtribe Apostasimerina Schönherr, 1844

Subtribe Coelonertina Casey, 1922

Subtribe Diorymerina Jekel, 1865

= Centrinina Jekel, 1865 ^H^

= Zygobaridina Pierce, 1907

= Limnobaridina Casey, 1922

Subtribe Nertinina Voss, 1954

= Nertina Bondar, 1949 [PI]

Subtribe Torcina Bondar, 1943

Tribe Baridini Schönherr, 1836

Subtribe Baridina Schönherr, 1836

= Anopsilina Bondar, 1942

Subtribe Coleomerina Casey, 1922

Subtribe Eurhinina Lacordaire, 1865

Subtribe Madopterina Lacordaire, 1865

Subtribe Parallelodemina Legalov, 2018

Tribe Eutoxini Champion, 1908

Subtribe Eutoxina Champion, 1908

Subtribe Tonesiina Alonso-Zarazaga & Lyal, 1999

Tribe Madarini Jekel, 1865

Subtribe Barymerina Lacordaire, 1865

= Sonnetiina Casey, 1922

Subtribe Madarina Jekel, 1865

= Leptoschoinina Lacordaire, 1865

= Thaliabaridina Bondar, 1943

Subtribe Neosharpiina Hoffmann, 1956

Tribe Optatini Champion, 1907

= Cyrionychini Casey, 1922

Tribe Pantotelini Lacordaire, 1865

Tribe Peridinetini Lacordaire, 1865

Subfamily Ceutorhynchinae Gistel, 1848

Tribe Amalini Wagner, 1936

Tribe Ceutorhynchini Gistel, 1848

= Isorhynchini Lacordaire, 1865

= Coeliodini J.L. LeConte, 1876

= Poophagini Schultze, 1902

= Tapinotini Joy, 1932

= Phrydiuchini Wagner, 1938

= Oxyonychini Hoffmann, 1957

Tribe Cnemogonini Colonnelli, 1979

Tribe Egriini Pajni & Kohli, 1982

Tribe Hypohypurini Colonnelli, 2004

Tribe Hypurini Schultze, 1902

Tribe Lioxyonychini Colonnelli, 1984

Tribe Mecysmoderini Wagner, 1938

Tribe Mononychini J.L. LeConte, 1876 ^H^

Tribe Phytobiini Gistel, 1848

= Rhinoncini C.G. Thomson, 1865

Tribe Scleropterini Schultze, 1902

Subfamily Conoderinae Schönherr, 1833 ^H^

Tribe Campyloscelini Schönherr, 1845

Subtribe Campyloscelina Schönherr, 1845

Subtribe Phaenomerina Faust, 1898

Tribe Conoderini Schönherr, 1833 ^H^

= Conophorini Schönherr, 1837 [PI]

Tribe Corynemerini Hustache, 1929

Tribe Coryssomerini C.G. Thomson, 1859

= Synophthalmini Lacordaire, 1863

= Metialmini Hustache, 1932

Tribe Coryssopodini Lacordaire, 1865

= Sympiezopodini Faust, 1886

Tribe Electrocoryssopini Legalov, 2023†

Tribe Lechriopini Lacordaire, 1865

= Copturini Desbrochers des Loges, 1892

Tribe Lobotrachelini Lacordaire, 1865

Tribe Mecopini Lacordaire, 1865

Tribe Menemachini Lacordaire, 1865

Tribe Othippiini Morimoto, 1962

Tribe Palaeomallerini Legalov, 2018†

Tribe Peloropodini Hustache, 1932

Tribe Piazurini Lacordaire, 1865

Tribe Sphadasmini Lacordaire, 1865

Tribe Trichodocerini Champion, 1906

Tribe Zygopini Lacordaire, 1865

= Eccoptini Pierce, 1919 [PI]

= Cylindrocopturini Böving, 1927

Subfamily Cossoninae Schönherr, 1825 ^153^

Tribe Acamptini J.L. LeConte, 1876

Tribe Acanthinomerini Voss, 1972

Tribe Allomorphini Folwaczny, 1973

Tribe Araucariini Kuschel, 1966

Tribe Cossonini Schönherr, 1825

= Proecini Voss, 1956

= Phylloplatypodini Kato, 1998

Tribe Cryptommatini Voss, 1972

Tribe Dryotribini J.L. LeConte, 1876

= Cotasterini Faust, 1886

Tribe Himatinini Konishi, 1962

Tribe Microxylobiini Voss, 1972

Tribe Nesiobiini Alonso-Zarazaga & Lyal, 1999

= Nesiotini Voss, 1972 [PI]

Tribe Neumatorini Folwaczny, 1973

Tribe Onychiini Chapuis, 1869 ^H^

Tribe Onycholipini Wollaston, 1873

Tribe Pentarthrini Lacordaire, 1865

Tribe Pseudapotrepini Champion, 1909

Tribe Rhyncolini Gistel, 1848

Subtribe Choerorhinina Folwaczny, 1973

Subtribe Phloeophagina Voss, 1955

Subtribe Pseudomimina Voss, 1939

= Aphyllurina Voss, 1955

Subtribe Rhyncolina Gistel, 1848

= Eremotina Voss, 1939

= Cotasterosomatina Konishi, 1962

Subtribe Stenoscelidina Wollaston, 1877

= Stereocorynina Voss, 1955

Tribe Tapiromimini Voss, 1972

Subfamily Molytinae Schönherr, 1823

Tribe Amalactini Lacordaire, 1863

Tribe Aminyopini Voss, 1956

= Niphadini Voss, 1963

= Niphadonothini Voss, 1965

Tribe Amorphocerini Voss, 1939

Tribe Anchonini Imhoff, 1856

Tribe Anoplini Bedel, 1883

Tribe Arachnopodini Lacordaire, 1865 ^154^

Tribe Brachyceropseini Aurivillius, 1926

Tribe Cholini Schönherr, 1825

Subtribe Cholina Schönherr, 1825

= Amerinina Pierce, 1919

Subtribe Cholomina Vaurie, 1974

Subtribe Rhinastina Vaurie, 1973

Tribe Cleogonini Gistel, 1848

= Conotrachelini Jekel, 1865

= Echinaspidini Blatchley, 1922 [PI]

Tribe Cycloterini Lacordaire, 1863

Subtribe Cycloterina Lacordaire, 1863

Subtribe Thrombosternina Voss, 1965

Tribe Dinomorphini Lacordaire, 1863

Tribe Emphyastini Lacordaire, 1863

= Phycocoetini J.L. LeConte, 1876 [PI]

= Thalasselephantini Alonso-Zarazaga & Lyal, 1999

Tribe Euderini Lacordaire, 1865 ^H^

Tribe Eudyasmini Legalov, 2018

Tribe Guioperini Lacordaire, 1865

Tribe Hylobiini W. Kirby, 1837

Subtribe Epistrophina G.A.K. Marshall, 1932

Subtribe Hylobiina W. Kirby, 1837

= Heilipodina Faust, 1892

= Syphorbina G.A.K. Marshall, 1932

Tribe Ithyporini Lacordaire, 1865

Subtribe Colobodina Voss, 1958

Subtribe Ithyporina Lacordaire, 1865

Subtribe Sclerocardiina Lacordaire, 1865

Tribe Itini Reitter, 1913 ^H^

Tribe Juanorhinini Aurivillius, 1931

Tribe Lepyrini W. Kirby, 1837

Tribe Lithinini Lacordaire, 1863 ^H^

Subtribe Lithinina Lacordaire, 1863 ^H^

= Diabathrariina Lacordaire, 1863 ^155^

Subtribe Rhytidophloeina Voss, 1963

Tribe Lymantini Lacordaire, 1865 ^156^

Subtribe Caecossonina Osella, 1980

Subtribe Lymantina Lacordaire, 1865

= Ithaurina Kuschel, 1959

Tribe Mecysolobini Reitter, 1913

= Alcidini Jekel, 1865 [PI]

= Alcidodini G.A.K. Marshall, 1939

Tribe Metatygini Pascoe, 1888

= Omophorini G.A.K. Marshall, 1917

= Sternechosomini Voss, 1958

Tribe Molytini Schönherr, 1823

Subtribe Leiosomatina Reitter, 1913

Subtribe Molytina Schönherr, 1823

= Liparina Latreille, 1828 ^H^

Subtribe Plinthina Lacordaire, 1863

= Minyopina Marseul, 1863

Subtribe Typoderina Voss, 1965

Tribe Nettarhinini Lacordaire, 1865

Tribe Pacholenini Lacordaire, 1863

Tribe Paipalesomini G.A.K. Marshall, 1932

Tribe Palaeocryptorhynchini Poinar & Legalov, 2016†

Tribe Petalochilini Lacordaire, 1863

= Epipedini Lacordaire, 1865 [PI]

= Hormopini J.L. LeConte, 1876

= Schoenherriellini Viana, 1952

= Epipedophyini Kuschel, 1955

Tribe Phoenicobatini Champion, 1914

Tribe Phrynixini Kuschel, 1964

Subtribe Geochina Legalov, 2020

Subtribe Phrynixina Kuschel, 1964

Tribe Pissodini Gistel, 1848

Subtribe Cotasteromimina Morimoto, 1962

Subtribe Orthorhinina Jekel, 1865

= Angianina Sharp, 1919

Subtribe Pissodina Gistel, 1848

Tribe Sciabregmini Legalov et al., 2020†

Tribe Sternechini Lacordaire, 1863

Tribe Sthereini Hatch, 1971

Tribe Styanacini Chûjô & Voss, 1960

Tribe Trachodini Gistel, 1848

= Blastophilini Gistel, 1856

= Acicnemidini Lacordaire, 1865

Tribe Tranesini Legalov, 2018

Tribe Trigonocolini Lacordaire, 1863

= Megarhinini Faust, 1888 [PI]

Tribe Trypetidini Lacordaire, 1865 ^H^

Subfamily Cryptorhynchinae Schönherr, 1825

Tribe Aedemonini Faust, 1898

= Mechistocerini Morimoto, 1978

= Derbyiellini Zimmerman, 1994

Tribe Camptorhinini Lacordaire, 1865

Tribe Cryptorhynchini Schönherr, 1825

Subtribe Cryptorhynchina Schönherr, 1825

= Cryptorhynchidiina Bradley, 1930

Subtribe Mecistostylina Lacordaire, 1865

= Cylindrocorynina Pascoe, 1875

Subtribe Tylodina Lacordaire, 1865

= Acallina Joy, 1932

Tribe Gasterocercini Zherikhin, 1991

Tribe Psepholacini Lacordaire, 1865

= Strongylopterini Lacordaire, 1865

= Sympiezoscelini Lacordaire, 1865

Tribe Sophrorhinini Lacordaire, 1865

Tribe Torneumatini Bedel, 1884

Subfamily Lixinae Schönherr, 1823

Tribe Cleonini Schönherr, 1826 ^H^ [NP]

= Geomorini Schönherr, 1823 [NO]

= Cleonini W. Kirby, 1837 ^H^

= Xerobiini Gistel, 1856

= Epirrhynchini Petri, 1914

Tribe Lixini Schönherr, 1823

= Larinini Gistel, 1848

= Phyllonomeini Gistel, 1856

Tribe Rhinocyllini Lacordaire, 1863

Subfamily Mesoptiliinae Lacordaire, 1863

Tribe Carciliini Pierce, 1916

Tribe Laemosaccini Lacordaire, 1865

Tribe Magdalidini Pascoe, 1870 [NP]

= Thamnophilini Schönherr, 1823 [PI]

= Scardamyctini Gistel, 1848 [NO]

= Magdalinini Gistel, 1856 [PI]

Tribe Mesoptiliini Lacordaire, 1863

= Cnemidophorini Hustache, 1937 [PI]

= Cnemidontini Kuschel, 1955

Subfamily Scolytinae Latreille, 1804

Tribe Amphiscolytini Mandelshtam & Beaver, 2003

Tribe Bothrosternini Blandford, 1896

Tribe Cactopinini Chamberlin, 1939

Tribe Carphodicticini Wood, 1971

Tribe Chaetophloeini Jordal, 2021

Tribe Coptonotini Chapuis, 1869

Tribe Coriacephilini A.J. Johnson, 2020

Tribe Corthylini J.L. LeConte, 1876

Subtribe Corthylina J.L. LeConte, 1876

= Amphicranina Eichhoff, 1878

= Xyleboripina Reitter, 1913

= Gnathotrichina Balachowsky, 1949

Subtribe Pityophthorina Eichhoff, 1878

= Araptina Eichhoff, 1878

Tribe Cryphalini Lindemann, 1876 ^157^

Tribe Crypturgini J.L. LeConte, 1876

Tribe Cylindrobrotini Kirejtshuk et al., 2009†

Tribe Diamerini Hagedorn, 1909

= Strombophorini Schedl, 1959

= Sphaerotrypini Murayama, 1963

Tribe Dryocoetini Lindemann, 1877

= Thamnurgini Nüsslin, 1911

= Taphrorychini Reitter, 1913

Tribe Ernoporini Nüsslin, 1911

Tribe Hexacolini Eichhoff, 1878

= Ctenophorini Chapuis, 1869 [PI]

= Problechilini Eichhoff, 1878

= Erineophilini Hopkins, 1902

Tribe Hylastini J.L. LeConte, 1876

= Hylurgopini Balachowsky, 1949

Tribe Hylesinini Erichson, 1836

= Phloeotrupini Lacordaire, 1865

= Dactylipalpini Blandford, 1893

= Phloeoborini Blandford, 1893

= Alniphagini Murayama, 1963

Tribe Hylurgini Gistel, 1848

= Dendroctonini Nüsslin, 1912

= Xylechinini Nüsslin, 1912

= Tomicini Wood, 1978

Tribe Hyorrhynchini Hopkins, 1915

= Sueini Murayama, 1959

Tribe Hypoborini Nüsslin, 1911

Subtribe Hypoborina Nüsslin, 1911

Subtribe Xerasiborina Jordal, 2021

Tribe Ipini Bedel, 1888

Subtribe Ipina Bedel, 1888

= Pityogenina Balachowsky, 1949

Subtribe Premnobiina Browne, 1962 ^158^

Tribe Micracidini J.L. LeConte, 1876

= Hylocurini Eichhoff, 1878

Tribe Phloeosinini Nüsslin, 1912

Tribe Phloeotribini Chapuis, 1869

= Phthorophloeini Nüsslin, 1912

Tribe Phrixosomatini Wood, 1978

Tribe Polygraphini Chapuis, 1869 ^H^

= Carphoborini Nüsslin, 1911

Tribe Scolytini Latreille, 1804

= Ekkoptogastrini Gistel, 1848 [PI]

= Camptocerini Lacordaire, 1865 ^H^

= Minulini Reitter, 1913

Tribe Scolytoplatypodini Blandford, 1893

= Taeniocerini Blandford, 1893 [PI]

= Spongocerini Hagedorn, 1909

Tribe Trypophloeini Nüsslin, 1911

Tribe Xyleborini J.L. LeConte, 1876

= Webbiini Hopkins, 1915

= Eccoptopterini Kalshoven, 1959

Tribe Xyloctonini Eichhoff, 1878

Subtribe Glostatina Jordal, 2023

Subtribe Xyloctonina Eichhoff, 1878

Tribe Xyloterini J.L. LeConte, 1876

= Trypodendrini Nunberg, 1954

Subfamily Orobitidinae C.G. Thomson, 1859

Subfamily Xiphaspidinae G.A.K. Marshall, 1920

**Superfamily Chrysomeloidea Latreille, 1802**

**Family Oxypeltidae Lacordaire, 1868**

**Family Vesperidae Mulsant, 1839**

Subfamily Philinae J. Thomson, 1861

Subfamily Vesperinae Mulsant, 1839

Subfamily Anoplodermatinae Guérin-Méneville, 1840

Tribe Anoplodermatini Guérin-Méneville, 1840

= Cherrocriini Prosen, 1960

Tribe Hypocephalini Blanchard, 1845

Tribe Mysteriini Prosen, 1960

**Family Disteniidae J. Thomson, 1861**

Tribe Cyrtonopini Gressitt, 1940

Tribe Disteniini J. Thomson, 1861

Tribe Dynamostini Lacordaire, 1868

Tribe Heteropalpini Villiers, 1961

**Family Cerambycidae Latreille, 1802 ^159^**

Subfamily Prioninae Latreille, 1802

Tribe Acanthophorini J. Thomson, 1864

Tribe Aegosomatini J. Thomson, 1861

= Jamwonini Kolbe, 1897

= Megopidini Lameere, 1912

Tribe Anacolini J. Thomson, 1857

= Poekilosomatini H.W. Bates, 1869

= Erythraenini H.W. Bates, 1875

= Delocheilini Lameere, 1913

= Sobarini Lameere, 1913 [PI]

Tribe Cacoscelini J. Thomson, 1861

= Colpoderini Lacordaire, 1868

= Notophyseini Lameere, 1903

Tribe Callipogonini J. Thomson, 1861

= Anacanthini J. Thomson, 1864 [PI]

= Ctenoscelidini J. Thomson, 1864

= Enoplocerini J. Thomson, 1864

= Orthomegalini J. Thomson, 1864

Tribe Calocomini Galileo & Martins, 1993

Tribe Cantharocnemini J. Thomson, 1861

Tribe Catypnini Lacordaire, 1868 ^160^

Tribe Ergatini Fairmaire, 1864

Tribe Eurypodini Gahan, 1906 (1868)

= Zaracini Lacordaire, 1868

Tribe Hopliderini J. Thomson, 1864

Tribe Macrodontiini J. Thomson, 1861

= Acanthinoderini J. Thomson, 1864

= Ancistrotini Lacordaire, 1868

Tribe Macrotomini J. Thomson, 1861

Subtribe Archetypina Lameere, 1912

Subtribe Basitoxina Lameere, 1912

= Mecosarthrina Melzer, 1919

Subtribe Macrotomina J. Thomson, 1861

= Aulacopodina Kolbe, 1897

= Cnemoplitina Schröder, 1905

= Prinobiina Vives, 2000

Subtribe Mallodonina J. Thomson, 1861 ^161^

= Stenodontina Lameere, 1903 ^H^

Subtribe Platygnathina Gilmour, 1954

Subtribe Remphanina Lacordaire, 1868 ^162^

= Rhaphipodina Lameere, 1912

Subtribe Xixuthrina Lameere, 1912

Tribe Mallaspini J. Thomson, 1861

= Pyrodini Harold, 1879

Tribe Meroscelisini J. Thomson, 1861

= Tragosomatini J. Thomson, 1864

= Closterini Lacordaire, 1868

= Monodesmini Gahan, 1890

= Luluini Gilmour, 1956

Tribe OsphryoniniJin et al., 2023

Tribe Parandrini Blanchard, 1845 ^163^

Subtribe Erichsoniina J. Thomson, 1861 ^H^

Subtribe Parandrina Blanchard, 1845

Tribe Prionini Latreille, 1802

= Cyrtognathini J. Thomson, 1861

= Prionommatini J. Thomson, 1861

= Psalidognathini J. Thomson, 1861

= Aulacocerini J. Thomson, 1864

= Derobrachini J. Thomson, 1864

= Orthosomatini J. Thomson, 1864

= Pithoclini J. Thomson, 1864

= Titanini J. Thomson, 1864

= Micropsalidini Gounelle, 1911 [PI]

= Polyarthrini Gounelle, 1911 ^H^

Tribe RhipidoceriniJin et al., 2023

Tribe Sceleocanthini Lacordaire, 1868 ^164^

Tribe Solenopterini Lacordaire, 1868

= Derancistrini Lameere, 1912

Tribe Tereticini Lameere, 1913

Tribe Vesperoctenini Vives, 2005

Subfamily Cerambycinae Latreille, 1802

Tribe Acangassuini Galileo & Martins, 2001

Tribe Achrysonini Lacordaire, 1868

Tribe Agallissini J.L. LeConte, 1873

Tribe Alanizini Di Iorio, 2003

Tribe Anaglyptini Lacordaire, 1868 [NP]

Tribe Aphanasiini Lacordaire, 1868

Tribe Aphneopini Lacordaire, 1868

Tribe Auxesini Lacordaire, 1872

= Psathyrini Quentin, 1954

Tribe Basipterini Fragoso et al., 1987

Tribe Bimiini Lacordaire, 1868

= Sibyllini Cerda, 1973 [PI]

Tribe Bothriospilini Lane, 1950

Tribe Brachypteromini Sama, 2008

= Dolocerini Özdikmen, 2016 **syn. nov.**

Tribe Callichromatini Swainson, 1840

= Terambini Gistel, 1848

Tribe Callidiini W. Kirby, 1837

Tribe Callidiopini Lacordaire, 1868

Tribe Cerambycini Latreille, 1802

Subtribe Cerambycina Latreille, 1802

Subtribe Sphallotrichina Martins & Monné, 2005

Tribe Certallini Fairmaire, 1864

= Pytheini J. Thomson, 1864

Tribe Chlidonini C.O. Waterhouse, 1879

Tribe Cleomenini Lacordaire, 1868

Tribe Clytini Mulsant, 1839

= Neoclytini J. Thomson, 1861

= Cyllenini J. Thomson, 1864 [PI]

Tribe Compsocerini J. Thomson, 1864

Tribe Coptommatini Lacordaire, 1869

= Navomorphini Lacordaire, 1869

Tribe Curiini J.L. LeConte, 1873

Tribe Deilini Fairmaire, 1864

Tribe Dejanirini Lacordaire, 1868

Tribe Dichophyiini Gistel, 1848 ^165^

Tribe Diorini Lane, 1950

Tribe Distichocerini Pascoe, 1867

Tribe Dodecosini Aurivillius, 1912

= Olexandrellini Zajciw, 1960

Tribe Dorcasomini Lacordaire, 1868 ^166^

= Apatophyseini Lacordaire, 1869

Tribe Dryobiini Arnett, 1962 ^H^

Tribe Eburiini Blanchard, 1845

= Heteropsini Lacordaire, 1868 ^167^

Tribe Ectenessini Martins, 1998

Tribe Elaphidiini J. Thomson, 1864

= Sphaeriini Lacordaire, 1868 ^H^

= Stenosphenini J.L. LeConte, 1873

Tribe Eligmodermini Lacordaire, 1868

Tribe Erlandiini Aurivillius, 1912

Tribe Eroschemini Lacordaire, 1868

Tribe Eumichthini Linsley, 1940

Tribe Gahaniini Quentin & Villiers, 1969

Tribe Glaucytini Lacordaire, 1868

Tribe Graciliini Mulsant, 1839

Tribe Hesperophanini Mulsant, 1839

Subtribe Daramina Sama, 2008

Subtribe Hesperophanina Mulsant, 1839

= Cerasphorina J. Thomson, 1861

Tribe Hesthesini Pascoe, 1867

Tribe Hexoplonini Martins, 2006

Tribe Holopleurini Chemsak & Linsley, 1974

Tribe Holopterini Lacordaire, 1868

= Proholopterini Monné, 2012 **syn. nov.**

Tribe Hyboderini Linsley, 1940

Tribe Hylotrupini Rose, 1983

Tribe Ideratini Martins & Napp, 2009

Tribe Lissonotini Swainson, 1840 ^H^

Tribe Luscosmodicini Martins, 2003

Tribe Lygrini Sama, 2008

= Pelossini Tavakilian, 2013 **syn. nov.**

Tribe Macronini Lacordaire, 1868 ^P^

= Enchopterini J. Thomson, 1861

Tribe Megacoelini Quentin & Villiers, 1969

Tribe Methiini J. Thomson, 1860

Tribe Molorchini Gistel, 1848

Tribe Mythodini Lacordaire, 1868

Tribe Necydalopsini Lacordaire, 1868

Tribe Neocorini Martins, 2005

Tribe Obriini Mulsant, 1839

Tribe Ochyrini Pascoe, 1871

Tribe Oedenoderini Aurivillius, 1912

Tribe Oemini Lacordaire, 1868

Subtribe Methioidina Martins, 1997

Subtribe Oemina Lacordaire, 1868

Tribe Opsimini J.L. LeConte, 1873

Tribe Oxycoleini Martins & Galileo, 2003

Tribe Paraholopterini Martins, 1997

Tribe Phalotini Lacordaire, 1868

Tribe Phlyctaenodini Lacordaire, 1868

Tribe Phoracanthini Newman, 1840

Tribe Phyllarthriini Lepesme & Breuning, 1956

Tribe Piesarthriini McKeown, 1947

Tribe Piezocerini Lacordaire, 1868

Subtribe Haruspicina Martins, 1976

Subtribe Piezocerina Lacordaire, 1868

= Zelliboriina Lane, 1951

Tribe Platyarthrini H.W. Bates, 1870

Tribe Plectogasterini Quentin & Villiers, 1969

Tribe Plectromerini Nearns & Braham, 2008

Tribe Pleiarthrocerini Lane, 1950

Tribe Plesioclytini Wappes & Skelley, 2015

Tribe Protaxini Gahan, 1906

Tribe Prothemini Lacordaire, 1868

Tribe Psebiini Lacordaire, 1868

= Leptideini Perrier, 1893 [PI]

= Cambaiini Lane, 1951

= Nathriini Arnett, 1962

Tribe Pseudocephalini Aurivillius, 1912 (1861)

= Ametrocephalini J. Thomson, 1861

Tribe Pseudolepturini J. Thomson, 1861

= Erythrini Pascoe, 1866

Tribe Psilomorphini Lacordaire, 1868

Tribe Pteroplatini J. Thomson, 1861

Tribe Pyrestini Lacordaire, 1868

Tribe Rhagiomorphini Newman, 1841

= Syllitini J. Thomson, 1864

= Stenoderusini Pascoe, 1867

= Calliprasini McKeown, 1947

Tribe Rhinotragini J. Thomson, 1861

Tribe Rhopalophorini Blanchard, 1845

Tribe Rosaliini Fairmaire, 1864

Tribe Sestyrini Lacordaire, 1868

Tribe Smodicini Lacordaire, 1868

Tribe Spintheriini Lacordaire, 1869

Tribe Stenhomalini Miroshnikov, 1989

Tribe Stenopterini Gistel, 1848

Tribe Strongylurini Lacordaire, 1868

Tribe Sydacini Martins, 2014

Tribe Tessarommatini Lacordaire, 1868

Tribe Thraniini Gahan, 1906

Tribe Thyrsiini Marinoni & Napp, 1984

Tribe Tillomorphini Lacordaire, 1868

= Epipedocerini Gahan, 1906

Tribe Torneutini J. Thomson, 1861

= Thaumasini J. Thomson, 1864

Tribe Trachyderini Dupont, 1836

Subtribe Ancylocerina J. Thomson, 1864

Subtribe Trachyderina Dupont, 1836

= Megaderina J. Thomson, 1861 ^H^

= Purpuricenina J. Thomson, 1861

= Sphaenothecina J. Thomson, 1861

= Tyloseina J. Thomson, 1861

= Eriphina J. Thomson, 1864

= Metopocoilina J. Thomson, 1864

= Pteracanthina J. Thomson, 1864

= Sternacanthina J. Thomson, 1864

= Tropidosomatina J. Thomson, 1864

= Paristemiina Lacordaire, 1868

= Poecilopeplina Lacordaire, 1868

= Stenaspidina Lacordaire, 1868

= Dorcacerina H.W. Bates, 1870

Tribe Tragocerini Pascoe, 1867

Tribe Trichomesiini Aurivillius, 1912

Tribe Trigonarthrini Villiers, 1984

Tribe Tropidini Martins & Galileo, 2007

Subtribe Compsina Martins & Galileo, 2007 ^H^

Subtribe Neoibidionina Monné, 2012

= Ibidionina J. Thomson, 1861 [PI]

Subtribe Tropidina Martins & Galileo, 2007

Tribe Tropocalymmatini Lacordaire, 1868

Tribe Typhocesini Lacordaire, 1868

Tribe Unxiini Napp, 2007

Tribe Uracanthini Blanchard, 1853

= Rhinophthalmini J. Thomson, 1861

= Neostenini Lacordaire, 1868 ^168^

Tribe Vesperellini Sama, 2008

Tribe Xystrocerini Blanchard, 1845

Subfamily Lepturinae Latreille, 1802 ^H, 169^

Tribe Acmaeopini Della Beffa, 1915

= Cariliini Zamoroka, 2022 **syn. nov.**

Tribe CaraphiiniOhbayashi et al., 2016^170^

Tribe Desmocerini Blanchard, 1845 ^171^

Tribe Encyclopini J.L. LeConte, 1873 ^172^

Tribe Evodinini Zamoroka, 2022

Tribe Lepturini Latreille, 1802 ^H^

= Grammopterini Della Beffa, 1915

Tribe Necydalini Latreille, 1825 ^173^

Tribe Oxymirini Danilevsky, 1997

Tribe Pidoniini Zamoroka, 2022

Tribe Rhagiini W. Kirby, 1837

= Pachytini Portevin, 1934

Tribe Stenocorini J. Thomson, 1861

= Toxotini J.L. LeConte & G.H. Horn, 1883 ^H^

Tribe Rhamnusiini Sama, 2009

Tribe Sachalinobiini Danilevsky, 2010

Tribe Teledapini Pascoe, 1871 ^174^

Tribe Xylosteini Reitter, 1913

Subfamily Spondylidinae Audinet-Serville, 1832

Tribe Anisarthrini Mamaev & Danilevsky, 1973

Tribe Asemini J. Thomson, 1861

= Criocephalini Perrier, 1893

Tribe Atimiini J.L. LeConte, 1873

Tribe Nothorhinini Zagajkevich, 1991

Tribe Saphanini Gistel, 1848

= Michthisomatini J.L. LeConte, 1873

Tribe Spondylidini Audinet-Serville, 1832

Tribe Tetropiini Seidlitz, 1891

= Criomorphini Portevin, 1927 [PI]

Subfamily Lamiinae Latreille, 1825

Tribe Acanthocinini Blanchard, 1845

= Dectini J. Thomson, 1860

= Trypanidiini J. Thomson, 1860

= Lagocheirini H.W. Bates, 1863

= Moneilemini J. Thomson, 1864

= Leiopodini J.L. LeConte, 1873

= Aedilini Perrier, 1893

= Graphisurini Leng, 1920

= Astynomini Portevin, 1927

Tribe Acanthoderini J. Thomson, 1860

= Dryoctenini J. Thomson, 1860

= Oreoderini J. Thomson, 1860

= Oplosiini J.L. LeConte & G.H. Horn, 1883

Tribe Acmocerini J. Thomson, 1864

Tribe Acridocephalini E.S. Dillon & L.S. Dillon, 1959

Tribe Acrocinini Swainson, 1840

Tribe Aderpasini Breuning & Téocchi, 1978

Tribe Aerenicini Lacordaire, 1872

Tribe Agapanthiini Mulsant, 1839

= Hippopseini J. Thomson, 1860

= Anauxeseini J. Thomson, 1864

= Aprosopini J. Thomson, 1864

= Nemotragini J. Thomson, 1864

= Aegoprepini Pascoe, 1871

= Pachypezini Lacordaire, 1872

= Spalacopseini Lacordaire, 1872

= Didymonychini Aurivillius, 1922

= Hippopsiconini L.S. Dillon & E.S. Dillon, 1945

Tribe Amphoecini Breuning, 1951

Tribe Ancitini Aurivillius, 1917 ^P^

= Hebesecidini Pascoe, 1871

Tribe Ancylonotini Lacordaire, 1869

Tribe Anisocerini J. Thomson, 1860

= Onychocerini J. Thomson, 1864

= Platysternini Lacordaire, 1872 ^H^

Tribe Apomecynini J. Thomson, 1860

= Adetini Lacordaire, 1872

= Ectatosiini Lacordaire, 1872

= Ischiolonchini Lacordaire, 1872

= Ptericoptini Lacordaire, 1872

Tribe Astathini J. Thomson, 1864

Tribe Batocerini J. Thomson, 1864

Tribe Calliini J. Thomson, 1864

= Gryllicini Lacordaire, 1872

Tribe Ceroplesini J. Thomson, 1860

Subtribe Ceroplesina J. Thomson, 1860

Subtribe Crossotina J. Thomson, 1864

= Ecyroschematina Lacordaire, 1872

= Hecyridina Lacordaire, 1872

= Corynofreina Aurivillius, 1911

Tribe Cloniocerini Lacordaire, 1872

Tribe Colobotheini J. Thomson, 1860

Tribe Compsosomatini J. Thomson, 1857

= Aereneini J. Thomson, 1868

Tribe Cyrtinini J. Thomson, 1864 ^H^

= Acanthomerosternoplini Tippmann, 1956

= Scopadini Villiers, 1980

Tribe Desmiphorini J. Thomson, 1860

= Metontini Lacordaire, 1869

= Amymomini Lacordaire, 1872

= Apodasyini Lacordaire, 1872

= Crinotarsini Lacordaire, 1872

= Estolini Lacordaire, 1872

= Nedinini Lacordaire, 1872

= Epicastini Lacordaire, 1872

= Eupogoniini J.L. LeConte, 1873

= Psenocerini J.L. LeConte, 1873

= Anaesthetini Perrier, 1893

= Essisini Aurivillius, 1917

= Velorini Aurivillius, 1917

Tribe Dorcadionini Swainson, 1840

= Dorcadodiini Gistel, 1856

Tribe Dorcaschematini J. Thomson, 1860

= Protonarthrini J. Thomson, 1864

Tribe Elytracanthinini Bousquet, 2009

= Elytracanthini Lane, 1955 [PI]

Tribe Enicodini J. Thomson, 1864

= Leptonotini J. Thomson, 1864

= Nemaschematini J. Thomson, 1864

Tribe Eunidiini Téocchi et al., 2010

Tribe Eupromerini Galileo & Martins, 1995

Tribe Exocentrini Pascoe, 1864

Tribe Forsteriini Tippmann, 1960

= Hebestolini J. Thomson, 1864 [PI]

= Falsamblesthini Gilmour, 1961

Tribe Gyaritini Breuning, 1950

Tribe Heliolini Breuning, 1951

Tribe Hemilophini J. Thomson, 1868 [NP]

= Amphionychini J. Thomson, 1860 [NO]

= Itini Lepesme, 1943 ^H^

Tribe Homonoeini J. Thomson, 1864

= Bumetopini Lacordaire, 1872

Tribe Hyborhabdini Aurivillius, 1911

Tribe Lamiini Latreille, 1825

= Monochamini Gistel, 1848

= Pachystolini Gistel, 1848

= Gnomini J. Thomson, 1860

= Phrissomatini J. Thomson, 1860

= Agniini J. Thomson, 1864

= Geraniini J. Thomson, 1864

= Morimini J. Thomson, 1864

= Taeniotini J. Thomson, 1864

= Rhodopidini Lacordaire, 1872 [PI]

= Goetini J.L. LeConte, 1873

= Ptychodini J.L. LeConte, 1873

= Potemnemini Aurivillius, 1922

= Rhodopinini Gressitt, 1951

= Docohammini E.S. Dillon & L.S. Dillon, 1959

Tribe Laticraniini Lane, 1959

Tribe Mauesiini Lane, 1956

Tribe Megabasini J. Thomson, 1860

Tribe Mesosini Mulsant, 1839

Tribe Microcymaturini Breuning & Téocchi, 1985

Tribe Morimonellini Lobanov et al., 1981

Tribe Morimopsini Lacordaire, 1869

Tribe Nyctimeniini Gressitt, 1951

= Nyctimenini J. Thomson, 1864 [PI]

Tribe Oculariini Breuning, 1950

Tribe Onciderini J. Thomson, 1860

= Hypsiomatini J. Thomson, 1860

= Onocephalini J. Thomson, 1860

= Hypselomini Pascoe, 1864

Tribe Oncideropsidini Aurivillius, 1922

Tribe Onychogleneini Aurivillius, 1923

Tribe Parmenini Mulsant, 1839

= Hexatrichini J. Thomson, 1864

Tribe Petrognathini Blanchard, 1845

Tribe Phacellini Lacordaire, 1872

Tribe Phantasini Kolbe, 1897

Tribe Phrynetini J. Thomson, 1864

Tribe Phymasternini Téocchi, 1989

Tribe Pogonocherini Mulsant, 1839

= Zaploini J.L. LeConte & G.H. Horn, 1883

Tribe Polyrhaphidini J. Thomson, 1860

Tribe Pretiliini Martins & Galileo, 1990

Tribe Proctocerini Aurivillius, 1922

Tribe Prosopocerini J. Thomson, 1864

Tribe Pteropliini J. Thomson, 1860

= Abrynini J. Thomson, 1864

= Protorhopalini J. Thomson, 1864

= Niphonini Pascoe, 1864

= Ataxiini Lacordaire, 1872

= Atossini Lacordaire, 1872

= Baraeini Lacordaire, 1872

= Emphytoeciini Lacordaire, 1872

= Metagnomini Aurivillius, 1925

Tribe Saperdini Mulsant, 1839

= Phytoeciini Mulsant, 1839

= Gleneini J. Thomson, 1864

= Obereini J. Thomson, 1864

Tribe Stenobiini Breuning, 1950

Tribe Sternotomini J. Thomson, 1860

Tribe Tapeinini J. Thomson, 1857

Tribe Tetraopini J. Thomson, 1860

Tribe Tetraulaxini Breuning & Téocchi, 1977

Tribe Tetropini Portevin, 1927

Tribe Theocrini Lacordaire, 1872

Tribe Tmesisternini Blanchard, 1853

= Ichthyosomatini J. Thomson, 1864

= Sphingnotini J. Thomson, 1864

= Trigonopterini Aurivillius, 1922

Tribe Tragocephalini J. Thomson, 1857

Tribe Xenicotelini Matsushita, 1933

Tribe Xenofreini Aurivillius, 1923

Tribe Xenoleini Lacordaire, 1872

Tribe Xylorhizini Lacordaire, 1872

Tribe Zygocerini J. Thomson, 1864

= Disternini Pascoe, 1871

**Family Megalopodidae Latreille, 1802**

Subfamily Megalopodinae Latreille, 1802

Tribe Leucasteini Rodríguez-Mirón, 2021

Tribe Megalopodini Latreille, 1802

Subtribe Macrolophina Rodríguez-Mirón, 2021

Subtribe Megalopodina Latreille, 1802

Subtribe Temnaspidina Rodríguez-Mirón, 2021

Tribe Sphondyliini Rodríguez-Mirón, 2021

Subfamily Palophaginae Kuschel & May, 1990

Tribe Lobanoviellini Kirejtshuk & Reid, 2021

Tribe Palophagini Kuschel & May, 1990

Subfamily Zeugophorinae Böving & Craighead, 1931

Subfamily Atelederinae Rodríguez-Mirón, 2021

**Family Orsodacnidae C.G. Thomson, 1859**

Subfamily Orsodacninae C.G. Thomson, 1859

Subfamily Aulacoscelidinae Chapuis, 1874

**Family Chrysomelidae Latreille, 1802**

Subfamily Chrysomelinae Latreille, 1802

Tribe Chrysomelini Latreille, 1802

= Eleiini Gistel, 1848

= Prasocurini Gistel, 1848

= Chloemelini Gistel, 1856

= Doryphorini Motschulsky, 1860 ^H^

= Gonioctenini Motschulsky, 1860

= Linini Motschulsky, 1860

= Ovosomatini Motschulsky, 1860

= Paropseini Motschulsky, 1860

= Phratorini Motschulsky, 1860

= Colaphini Siegel, 1866

= Entomoscelidini Chapuis, 1874

= Phyllocharitini Chapuis, 1874

= Phyllodectini J.L. LeConte & G.H. Horn, 1883

= Dicranosternini Weise, 1915

= Phaedonini Weise, 1915

= Zygogrammatini Weise, 1915

= Chrysolinini S.H. Chen, 1936

= Barymelini Bechyné, 1948

= Doryphorini Bechyné, 1950

= Monarditini Bechyné, 1953

= Oreinini Bechyné, 1958 ^H^

= Hispostomatini Daccordi, 1981

= Sphaeratrixini Daccordi, 1981

= Gastrophysini Kippenberg, 2010 ^H^

Tribe MesolpininiKirejtshuk et al., 2016†

Tribe Timarchini Motschulsky, 1860

Subfamily Galerucinae Latreille, 1802

Tribe Alticini Newman, 1834 ^175^

= Longitarsini Maehler, 1850

= Plectroscelidini C.G. Thomson, 1866

= Aphthonini Chapuis, 1875

= Arsipodini Chapuis, 1875

= Aspicelini Chapuis, 1875

= Blepharidini Chapuis, 1875

= Crepidoderini Chapuis, 1875

= Diboliini Chapuis, 1875

= Lacticini Chapuis, 1875

= Mniophilini Chapuis, 1875

= Monoplatini Chapuis, 1875

= Oedionychini Chapuis, 1875

= Oxygonini Chapuis, 1875

= Psylliodini Chapuis, 1875

= Chaetocnemini J.L. LeConte & G.H. Horn, 1883

= Disonychini J.L. LeConte & G.H. Horn, 1883

= Euplectroscelidini G.H. Horn, 1889

= Pseudolampseini G.H. Horn, 1889

= Systenini G.H. Horn, 1889

= Luperalticini Leng, 1920

= Octogonotini Weise, 1921

= Sphaeronychini Bechyné & Špringlová de Bechyné, 1960

= Cacoscelidini Bechyné, 1968

= Lypneini Bechyné, 1968

= Podagricini Bechyné, 1968

= Wittmeralticini Bechyné, 1968

= Psilaphini Bechyné & Špringlová de Bechyné, 1973

= Diphaulacini Bechyné & Špringlová de Bechyné, 1975

= Manobiini Bechyné & Špringlová de Bechyné, 1975

= Marcapatiini Bechyné & Špringlová de Bechyné, 1975

= Monomacrini Bechyné & Špringlová de Bechyné, 1975

= Hermaeophagini Bechyné, 1997

= Ocnoscelidini Bechyné, 1997

= Febraini Nadein, 2013

Tribe Decarthrocerini Laboissière, 1937

Tribe Galerucini Latreille, 1802

= Apophyliini Chapuis, 1875

= Atysini Chapuis, 1875

= Coelomerini Chapuis, 1875

= Rupiliini Chapuis, 1875

= Schematizini Chapuis, 1875

= Mombasicini Laboissière, 1922

= Chorinini Weise, 1923 ^H^

= Leptosonychini Weise, 1924

= Theonini Laboissière, 1934

Tribe Hylaspini Chapuis, 1875

= Agelasticini Chapuis, 1875

= Antiphini Chapuis, 1875 [PI]

= Sermylini Chapuis, 1875 [PI]

= Bonesiini Laboissière, 1926

= Capulini Ogloblin, 1936 ^H^

= Gallerucidini Gressitt & Kimoto, 1963

= Sermylassini Mroczkowski, 1991

Tribe Luperini Gistel, 1848

= Aulacophorini Chapuis, 1875

= Cerophysini Chapuis, 1875

= Cerotomini Chapuis, 1875

= Diabroticini Chapuis, 1875

= Mimastrini Chapuis, 1875

= Monoleptini Chapuis, 1875

= Ornithognathini Chapuis, 1875

= Phyllobroticini Chapuis, 1875

= Platyxanthini Chapuis, 1875

= Scelidini Chapuis, 1875

= Androlyperini G.H. Horn, 1893

= Phyllecthrini G.H. Horn, 1893

= Diacanthini Weise, 1902

= Raphidopalpini Weise, 1902

= Idacanthini Laboissière, 1921

= Hyperacanthini Laboissière, 1924

= Megalognathini Laboissière, 1926

Tribe Metacyclini Chapuis, 1875

= Stictocemini Laboissière, 1922

Tribe Oidini Laboissière, 1921 (1875)

= Adoriini Chapuis, 1875

Tribe Serraticollini B.E. White, 1942 ^176^

Tribe Taimyralticini Nadein, 2018†

Subfamily Synetinae J.L. LeConte & G.H. Horn, 1883

Subfamily Sagrinae Leach, 1815

Tribe Carpophagini Chapuis, 1874 ^H^

Tribe Diaphanopsidini Monrós, 1958

Tribe Megamerini Chapuis, 1874

= Ametallini Chapuis, 1874

= Mecynoderini Chapuis, 1874

Tribe Sagrini Leach, 1815

Subfamily Bruchinae Latreille, 1802

Tribe Amblycerini Bridwell, 1932

Subtribe Amblycerina Bridwell, 1932

Subtribe Spermophagina Borowiec, 1987

Tribe Bruchini Latreille, 1802

Subtribe Acanthoscelidina Bridwell, 1946

= Bruchidiina Bridwell, 1946

Subtribe Bruchina Latreille, 1802

= Lariina Bedel, 1901 [PI]

Subtribe Megacerina Bridwell, 1946 ^H^

Tribe Eubaptini Bridwell, 1932

Tribe Kytorhinini Bridwell, 1932

Tribe Myanmaropini Legalov & Kirejtshuk, 2020†

Tribe Pachymerini Bridwell, 1929

Subtribe Caryedontina Bridwell, 1929

Subtribe Caryopemina Bridwell, 1929

Subtribe Pachymerina Bridwell, 1929

Tribe Rhaebini Blanchard, 1845

Subfamily Donaciinae W. Kirby, 1837

Tribe Donaciini W. Kirby, 1837

Tribe Haemoniini S.H. Chen, 1941

= Macropleini Lopatin, 1977

Tribe Plateumarini Böving, 1922

Subfamily Criocerinae Latreille, 1804

Tribe Criocerini Latreille, 1804

Tribe Lemini Gyllenhal, 1813

Tribe Pseudocriocerini Heinze, 1962

Subfamily Cassidinae Gyllenhal, 1813

Tribe Alurnini Chapuis, 1875

= Platyaucheniini Spaeth, 1929

Tribe Anisoderini Chapuis, 1875

Tribe Aproidini Weise, 1911

Tribe Arescini Chapuis, 1875

Tribe Aspidimorphini Chapuis, 1875

Tribe Basiprionotini Gressitt, 1952 (1929)

= Priopterini Spaeth, 1914

= Epistictinini Hincks, 1952

Tribe Botryonopini Chapuis, 1875

Tribe Callispini Chapuis, 1875

Tribe Callohispini Uhmann, 1960

Tribe Cassidini Gyllenhal, 1813

= Evaspistini Gistel, 1856

= Basiptini Chapuis, 1875

= Coptocyclini Spaeth & Reitter, 1926

= Charidotidini Spaeth, 1942

Tribe Cephaloleiini Chapuis, 1875

Tribe Chalepini Weise, 1910 [NP]

= Microrhopalini G.H. Horn, 1883 [NO]

= Stenopodiini G.H. Horn, 1883 [NO]

Tribe Coelaenomenoderini Weise, 1911

= Pharangispini Uhmann, 1940

Tribe Cryptonychini Chapuis, 1875

Tribe Delocraniini Spaeth, 1929

Tribe Dorynotini Monrós & Viana, 1949 (1923)

= Batonotini Spaeth, 1923

Tribe Eurispini Chapuis, 1875

Tribe Exothispini Weise, 1911

Tribe Goniocheniini Spaeth, 1942

Tribe Gonophorini Chapuis, 1875

Tribe Hemisphaerotini Monrós & Viana, 1951 (1929)

= Porphyraspidini Spaeth, 1929

Tribe Hispini Gyllenhal, 1813

= Monochirini Weise, 1905 [PI]

Tribe Hispoleptini Chapuis, 1875

Tribe Hybosispini Weise, 1910

Tribe Imatidiini Hope, 1841

Tribe Ischyrosonychini Chapuis, 1875

= Physonotini Spaeth, 1942

= Asterizini Hincks, 1952

Tribe Leptispini Fairmaire, 1868

Tribe Mesomphaliini Hope, 1841

= Eugenysini Hincks, 1952 ^177^

= Stoladini Hincks, 1952

Tribe Notosacanthini Gressitt, 1952 (1929)

= Hoplionotini Spaeth, 1929

Tribe Oediopalpini Monrós & Viana, 1947 (1910)

= Amplipalpini Weise, 1910

Tribe Omocerini Hincks, 1952 (1923)

= Tauromini Spaeth, 1923

Tribe Oncocephalini Chapuis, 1875

= Chaeridionini Weise, 1911

Tribe Promecothecini Chapuis, 1875

Tribe Prosopodontini Weise, 1910

Tribe Sceloenoplini Uhmann, 1930 (1906)

= Cephalodontini Gestro, 1906

Tribe Spilophorini Chapuis, 1875 ^H^

Tribe Uroplatini Weise, 1910 ^P^

= Octotomini G.H. Horn, 1883

Subfamily Eumolpinae Hope, 1841 ^178^

Tribe Bromiini Baly, 1865 (1863) ^P^

= Adoxini Baly, 1863

= Heteraspidini Baly, 1863

= Myochroini Baly, 1865

= Leprotetini Chapuis, 1874

= Pseudocolaspidini Chapuis, 1874

= Scelodontini Chapuis, 1874

= Tomyriini Chapuis, 1874 [PI]

= Nerissini Kuntzen, 1912

= Cynoiini Clavareau, 1914

= Odontionopini Clavareau, 1914 [PI]

= Trichochryseini Clavareau, 1914

= Lypesthetini Chûjô, 1956

= Ebooini Reid, 1993

Tribe Caryonodini Bechyné, 1951

Tribe Cubispini Monrós, 1954

Tribe Eumolpini Hope, 1841

= Colaspidini Hope, 1841

= Corynodini T.A. Marshall, 1865

= Chalcophanini Chapuis, 1874 ^H^

= Edusini Chapuis, 1874

= Endocephalini Chapuis, 1874

= Iphimeini Chapuis, 1874

= Chrysodinini Lefèvre, 1885

= Edusellini Clavareau, 1914

= Colaspoidini S.H. Chen, 1940

= Platycorynini L.N. Medvedev & Rybakova, 1985

Tribe Euryopini Chapuis, 1874

= Odontionopini Lefèvre, 1876

= Prasoideini Clavareau, 1914

= Colasposomatini Špringlová de Bechyné, 1960

Tribe Habrophorini Bechyné & Špringlová de Bechyné, 1969

Tribe Hemydacnini Bechyné, 1951

Tribe Megascelidini Chapuis, 1874

Tribe Merodini Chapuis, 1874

Tribe Pygomolpini Bechyné, 1949

Tribe Rosiroiini Bechyné, 1950

Tribe Typophorini Baly, 1865

= Callisinini Chapuis, 1874

= Metachromatini Chapuis, 1874

= Nodostomatini Chapuis, 1874

= Pagriini Lefèvre, 1884

= Cheirideini Lefèvre, 1885

= Nodinini S.H. Chen, 1940

= Basileptini Chûjô, 1956

= Nodini Selman, 1965 [PI]

Subfamily Lamprosomatinae Lacordaire, 1848

Tribe Cachiporrini Chamorro & Konstantinov, 2011

Tribe Lamprosomatini Lacordaire, 1848

Tribe Neochlamysini Monrós, 1959

Tribe Sphaerocharini Chapuis, 1874

Subfamily Cryptocephalinae Gyllenhal, 1813 ^179^

Tribe Clytrini W. Kirby, 1837

Subtribe Arateina Moldenke, 1981

Subtribe Babiina Lacordaire, 1848

= Ischiopacheina Chapuis, 1874

Subtribe Clytrina W. Kirby, 1837

= Melolonthina Bedel, 1891 [PI]

Subtribe Eoclytrina Monrós, 1958

Subtribe Megalostomidina Lacordaire, 1848

Tribe Cryptocephalini Gyllenhal, 1813

Subtribe Achaenopina Chapuis, 1874

Subtribe Cryptocephalina Gyllenhal, 1813

Subtribe Monachulina Leng, 1920

= Monachina Chapuis, 1874 [PI]

= Lexiphanina Wilcox, 1954

Subtribe Stylosomina Chapuis, 1874

Tribe Fulcidacini Jakobson, 1924

= Chlamydini Lacordaire, 1848 [PI]

= Chlamisini Gressitt, 1946

Tribe Mylassini Gómez-Zurita & Cardoso, 2021

Tribe Pachybrachini Chapuis, 1874

Subfamily Spilopyrinae Chapuis, 1874

= Stenomelinae Chapuis, 1874

= Hornibiinae Crowson, 1946

Subfamily Protoscelidinae L.N. Medvedev, 1968†

**CUCUJIFORMIA incertae sedis**

**Parandrexidae Kirejtshuk, 1994**†

Parandrexinae Kirejtshuk, 1994†

Cretoparacucujinae Kirejtshuk, 2023†

**Wabbelidae Alekseev, 2017**†

**POLYPHAGA incertae sedis**

Rhamphophorini Poinar & A.E. Brown, 2021 ^180^

Palaeotylinae Poinar et al., 2018† ^181^

**Lasiosynidae Kirejtshuk et al., 2010**†

**Peltosynidae Yan et al., 2017**†

**Jurodidae Ponomarenko, 1985** ^182^

= Sikhotealiniidae Lafer, 1996

“**CROWN COLEOPTERA” incertae sedis**

Avitortorinae Nikolajev, 2007†

**COLEOPTERA incertae sedis**

**Liaoximordellidae W.I. Wang, 1993**† ^183^

## Appendix 2

Supporting references for the conservation of *Aplastus* J.L. LeConte, 1859 [Coleoptera: ELATERIDAE] over *Aplastus* Gistel, 1848, an unnecessary new replacement name for *Morion* Latreille, 1810 [Coleoptera: CARABIDAE] (Article 23.9.2). The name *Aplastus* Gistel, 1848 has not been used as valid after 1899 to our knowledge.

Arnett Jr RH (2000) American insects. A handbook of the insects of America north of Mexico. Second edition. CRC Press, Boca Raton, xvii + 1003 pp.

Bocák L, Motyka M, Bocek M, Bocáková M (2018) Incomplete sclerotization and phylogeny: the phylogenetic classification of *Plastocerus* (Coleoptera: Elateroidea). PLOS ONE 13(3): e0194026. https://doi.org/10.1371/journal.pone.0194026

Bouchard P, Bousquet Y, Davies AE, Alonso-Zarazaga MA, Lawrence JF, Lyal CHC, Newton AF, Reid CAM, Schmitt M, Ślipiński SA, Smith ABT (2011) Family-group names in Coleoptera (Insecta). ZooKeys 88: 1–972. https://doi.org/10.3897/zookeys.88.807

Branham MA (2010) 4.8 Plastoceridae Crowson, 1972. In: Leschen RAB, Beutel RG, Lawrence JF (Eds) Handbook of Zoology. Arthropoda: Insecta. Coleoptera, beetles. Volume 2: Morphology and systematics (Elateroidea, Bostrichiformia, Cucujiformia partim). Walter de Gruyter GmbH & Co., Berlin/New York, 103–104. https://doi.org/10.1515/9783110911213.103

Calder AA, Lawrence JF, Trueman JWH (1993) *Austrelater*, gen. nov. (Coleoptera: Elateridae), with a description of the larva and comments on elaterid relationships. Invertebrate Taxonomy 7: 1349–1394. https://doi.org/10.1071/IT9931349

Costa C, Lawrence JF, Rosa SP (2010) 4.7 Elateridae Leach, 1815. In: Leschen RAB, Beutel RG, Lawrence JF (Eds) Handbook of Zoology. Arthropoda: Insecta. Coleoptera, beetles. Volume 2: Morphology and systematics (Elateroidea, Bostrichiformia, Cucujiformia partim). Walter de Gruyter GmbH & Co., Berlin/New York, 75–103. https://doi.org/10.1515/9783110911213.75

Etzler FE, Johnson PJ (2018) *Athoplastus* Johnson and Etzler (Coleoptera: Elateridae: Dendrometrinae), a new genus of click beetle from the northwestern continental USA. The Coleopterists Bulletin 72(3): 503–521. https://doi.org/10.1649/0010-065X-72.3.503

Evans AV (2021) Beetles of western North America. Princeton University Press, Princeton, 624 pp. Evans AV, Hogue JN (2004) Introduction to California beetles. University of California Press, Berkeley, xvii + 299 pp.

Evans AV, Hogue JN (2006) Field guide to beetles of California. University of California Press, Berkeley, xiv + 334 pp.

Hlavac TF 1975. The prothorax of Coleoptera: (except bostrichiformia - cucujiformia). Bulletin of the Museum of Comparative Zoology 147(4): 137–183.

Johnson PJ (2002) 58. Elateridae Leach 1815. In: Arnett Jr RH, Thomas MC, Skelley PE, Frank JH (Eds) American beetles. Volume 2. Polyphaga: Scarabaeoidea through Curculionoidea. CRC Press, Boca Raton, 160–173.

Johnson PJ (2014) *Barrelateraudreyae*, new genus and species, from the United States Intermountain West (Coleoptera: Elateridae). Giornale Italiano di Entomologia, 13: 407–411.

Jonson PJ (2017) A new species of *Dodecacius* Schwarz (Coleoptera: Elateridae) from Madre de Dios, Peru. Revista Peruana de Biología 24(3): 243–248. https://doi.org/10.15381/rpb.v24i3.13903

Kukalová-Peck, Lawrence JF (2004) Relationships among Coleoptera suborders and major endoneopteran lineages: evidence from hind wing characters. European Journal of Entomology 101: 95–144. https://doi.org/10.14411/eje.2004.018

Kundrata R, Bocak L (2011) The phylogeny and limits of Elateridae (Insecta, Coleoptera): is there a common tendency of click beetles to soft-bodiedness and neoteny? Zoologica Scripta 40: 364–378. https://doi.org/10.1111/j.1463-6409.2011.00476.x

Lawrence JF (1988) Rhinorhipidae, a new beetle family from Australia, with comments on the phylogeny of the Elateriformia. Invertebrate Taxonomy 2: 1–53. https://doi.org/10.1071/IT9880001

Lawrence JF (1991) Plastoceridae (Cantharoidea). In: Stehr FW (Ed) Immature Insects. Volume 2. Kendall/Hunt, Dubuque, Iowa, xvi + 975 pp.

Lawrence JF, Newton Jr AF (1995) Families and subfamilies of Coleoptera (with selected genera, notes, references and data on family-group names). In: Pakaluk J, Ślipiński SA (Eds) Biology, phylogeny, and classification of Coleoptera: Papers celebrating the 80th birthday of Roy A. Crowson. Muzeum i Instytut Zoologii PAN, Warsaw, 779–1006.

Lawrence JF, Zhou Y-L, Lemann C, Sinclair B, Ślipiński A (2021) The hind wing of Coleoptera (Insecta): morphology, nomenclature and phylogenetic significance. Part 1. General discussion and Archostemata–Elateroidea. Annales Zoologici 71: 421–606. https://doi.org/10.3161/00034541ANZ2021.71.3.001

Mani MS (1974) Modern Classification of Insects. Satish Book Enterprise, Agra, vi + 331 pp.

Poole RW, Gentili P (1996) Nomina Insecta Nearctica. A Check List of the Insects of North America. Volume 1: Coleoptera, Stresiptera. Entomological Information Services, Rockville, Maryland, 827 pp.

Schimmel R, Platia G, Tarnawski D (2008) A new genus *Sinoaplastinus*, with a new species *S.kadeji* from China, the first elaterid-beetle with bi-lamellate antennomeres from the Palaearctic Region (Insecta: Coleoptera: Elateridae). Genus 19(4): 669–674.

Stibick JNL (1979) Classification of the Elateridae (Coleoptera): relationships and classification of the subfamilies and tribes. Pacific Insects 20(2/3): 145–186.

United States Department of Agriculture (1987) List of intercepted plant pests. Fiscal year 1986. Plant Protection and Quarantine. APHIS 82-13, V + 216 + [4] pp.
